# Integrated transcriptomic and metabolomic analysis provides insight into the regulation of leaf senescence in rice

**DOI:** 10.1038/s41598-021-93532-x

**Published:** 2021-07-08

**Authors:** Jiao Xue, Dongbai Lu, Shiguang Wang, Zhanhua Lu, Wei Liu, Xiaofei Wang, Zhiqiang Fang, Xiuying He

**Affiliations:** Guangdong Key Laboratory of New Technology in Rice Breeding, Rice Research Institute, Guangdong Academy of Agricultural Sciences, Guangzhou, 510640 People’s Republic of China

**Keywords:** Transcriptomics, Molecular biology, Plant sciences

## Abstract

Leaf senescence is one of the most precisely modulated developmental process and affects various agronomic traits of rice. Anti-senescence rice varieties are important for breeding application. However, little is known about the mechanisms underlying the metabolic regulatory process of leaf senescence in rice. In this study, we performed transcriptomic and metabolomic analyses of the flag leaves in Yuenong Simiao (YN) and YB, two *indica* rice cultivars that differ in terms of their leaf senescence. We found 8524 genes/204 metabolites were differentially expressed/accumulated in YN at 30 days after flowering (DAF) compared to 0 DAF, and 8799 genes/205 metabolites were differentially expressed in YB at 30 DAF compared to 0 DAF. Integrative analyses showed that a set of genes and metabolites involved in flavonoid pathway were significantly enriched. We identified that relative accumulation of *PHENYLALANINE AMMONIA-LYASE* (*PAL*), *CINNAMATE 4-HYDROXYLASE* (*C4H*), *4-COUMAROYL-COA LIGASE* (*4CL*), *CHALCONE SYNTHASE* (*CHS*) and *CHALCONE ISOMERASE* (*CHI*) in YN30/0 was higher than that in YB30/0. Three flavonoid derivatives, including phloretin, luteolin and eriodictyol, showed lower abundances in YB than in YN at 30 DAF. We further revealed a MYB transcription factor, which is encoded by *OsR498G0101613100* gene, could suppress the expression of *CHI* and *CHS*. Our results suggested a comprehensive analysis of leaf senescence in a view of transcriptome and metabolome and would contribute to exploring the molecular mechanism of leaf senescence in rice.

## Introduction

Leaf senescence, an orderly process of degradation and transformation, is the last stage of leaf development and is controlled by complex internal factors such as hormones, genetics, and external gene network regulatory factors, including light, water, temperature, mineral elements, and microorganisms^1–3^. Leaf senescence plays important roles in regulating nutrient distribution and improving the ability of plants to adapt to the environment. Through the process of senescence, leaves transfer nutrients to seeds or fruits at the end of development, providing necessary energy for offspring, seed germination and early growth of seedlings^4^. During senescence, leaf cells undergo dramatic changes in cell metabolism, structure, and gene expression. The most striking feature of these changes is that the decomposition of chlorophyll during chloroplast denaturation results in the yellowing of leaves, followed by hydrolysis of macromolecules such as lipids, proteins and nucleic acids, which in turn leads to separation of mitochondria and nuclei and ultimately cell death^5^. Together, the identification and characterization of hundreds of senescence-associated genes (SAGs) in plants and senescence-related mutants constitute one of the best approaches for understanding leaf senescence at the molecular level^5–7^. The leaf senescence database (LSD) also provides comprehensive information concerning SAGs and their corresponding mutants^8^.

Transcriptome sequencing is an important method for obtaining gene expression data in organisms^9^. High-throughput profiling of transcripts is also an efficient method for investigating the process of plant leaf senescence, as has been demonstrated for *Arabidopsis thaliana*, rice, tobacco, soybean, wheat, cotton, and maize^10–15^. However, metabolites are the basis and direct manifestation of an organism’s phenotype. Thus, metabolomics provides a global physiochemical view of cellular status during leaf senescence. By detecting the major carbohydrate and nitrogen metabolite markers of tobacco leaves at different developmental stages, researchers identified a sink-to-source transition at a particular stage in which an accumulation of carbohydrates and a depletion of both organic and inorganic nitrogen stores were observed^16^. By determining changes in chemical composition during leaf senescence in *Arabidopsis thaliana*, researchers also found that the C, Cr, Cu, Fe, K, Mo, N, P, S and Zn mobilized from senescing leaves^17^. In recent years, studies combining transcriptomics and metabolomics have been successfully used to research the biosynthesis pathway of medicinally important active ingredients in plants^18,19^, to study the fruit ripening and disease resistance of tomato^20,21^, to elucidate molecular mechanism underlying rice drought tolerance^22^, and so on. New genes related to senescence could still be discovered, and the metabolic pathways in which they are involved concerning the regulation of senescence should be explored by multi-omic approaches.

Rice is one of the most important crop species in the world and is the staple food for more than half of the global population^23^. Rice yield and other agronomic traits are of concern to scientists. In the process of breeding, whether the process of functional leaves turning from green to yellow is stable and smooth in the late-mature stage of rice is also a concern of breeders. This stage is accompanied by the degradation of chlorophyll, pigment deposition and rapid changes in metabolite content. Leaves, especially flag leaves, which are the main functional organs of photosynthesis in rice, are the primary contributor to the accumulation of dry matter in grains. Therefore, premature leaf senescence is one of the main factors affecting rice yield stability. The leaf senescence database currently contains more than 180 SAGs experimentally identified in rice^8^; these SAGs are involved in chloroplast degradation, hormones and transcription factors associated with photosynthesis, the energy metabolism pathway, nitrogen mobilization and so on. However, the biochemical basis and molecular regulatory mechanisms underlying these processes in rice are not clear.

Yuenong Simiao (YN) is a major rice cultivar in South China and exhibits a strong anti-senescence phenotype at the late-mature stage. To explore the underlying molecular mechanisms of the anti-senescence of YN, we performed transcriptomic and metabonomic analyses of the flag leaves of YN as well as YB, another rice variety that exhibits an early-senescence phenotype but is similar to YN in terms of flowering time, resistance, and yield. We identified 8524 differentially expressed genes (DEGs) in YN and 8799 DEGs in YB. By integrating transcriptome and metabolomic data, we found that a set of genes and metabolites involved in flavonoid pathway may exhibit different expression patterns among YN and YB. Interestingly, we found that one MYB TF, *OsR498G0101613100*, could inhibit the expression of *CHALCONE ISOMERASE* (*CHI*) and *CHALCONE SYNTHASE* (*CHS*), which were indispensable for the flavonoid pathway in rice. The findings can help to understand the regulatory mechanisms of flavonoid biosynthesis and may be useful for the further research on anti-senescence in rice.

## Results

### High-quality Yuenong Simiao rice exhibits a significant anti-senescence phenotype

Yuenong Simiao is a major rice variety with good quality in South China, with a total planting area of more than 200,000 ha. This variety has high yields, strong disease resistance and other excellent traits. In addition, the leaf colour changes slowly from green to yellow in the late stage of development, which is an important characteristic selected by breeders. To study the characteristics of leaf colour transformation, we chose YB as a control variety. The flowering period of YB was the same as that of YN, but the leaf colour changed from green to yellow and withered rapidly. Given that the leaf colour change from green to yellow is a notable indicator of senescence of plants, we performed detailed physiological, biochemical and molecular assays to determine whether YN has anti-senescence traits.

**Figure 1:**
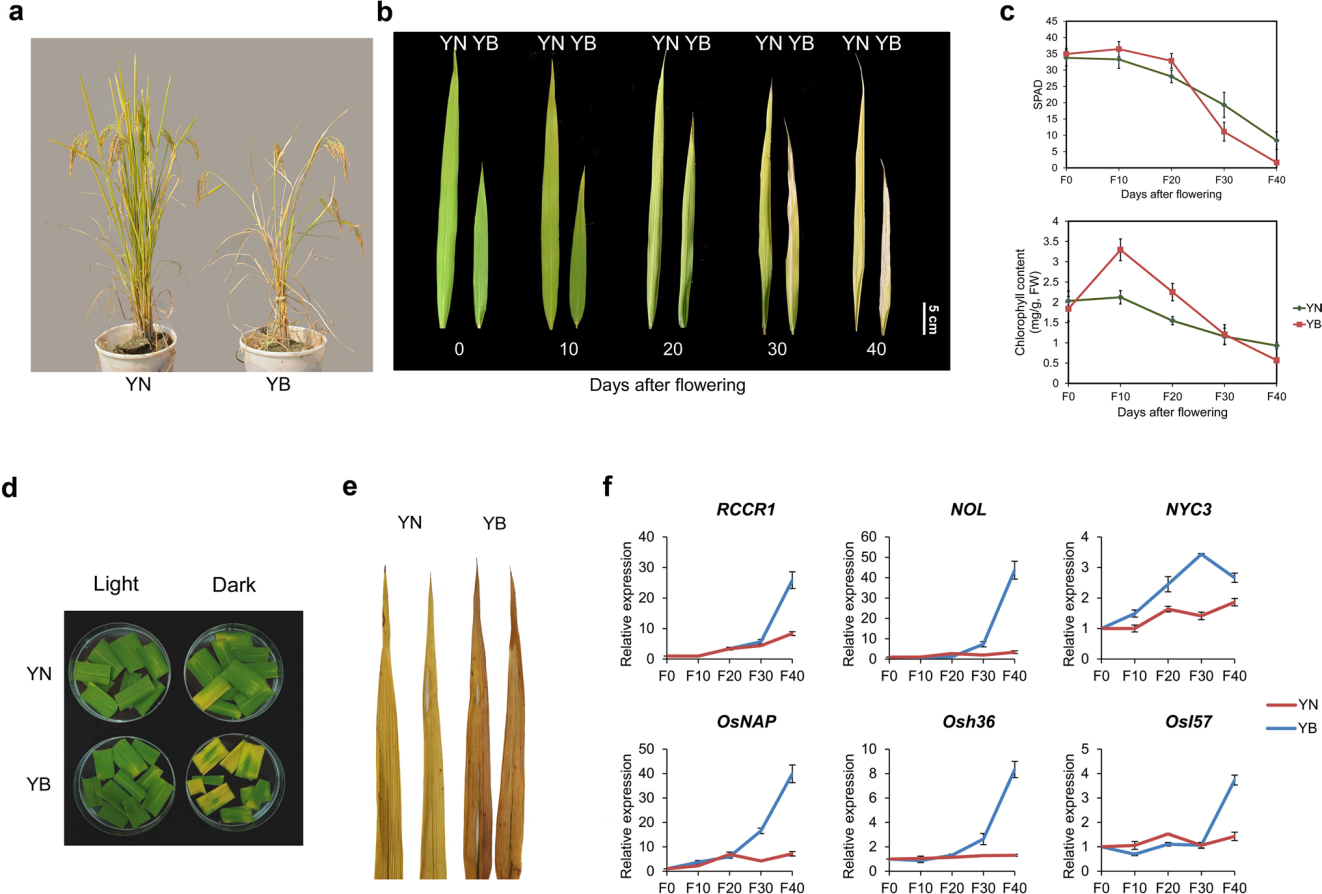
Phenotypes of two rice cultivars. (**a**) Phenotypes of YN and YB at 30 DAF. YN is shown on the left, and YB in shown on the right. (**b**) Phenotypes of flag leaves of YN and YB plants at different stages after flowering. Bars = 5 cm. (**c**) SPAD values and chlorophyll contents of flag leaves of YN and YB plants after flowering. The values are the means ± SDs of 3 measurements. *FW* fresh weight. (**d**) Detached flag leaves from YN and YB plants at the flowering stage were incubated in water for 5 days in darkness. (**e**) DAB staining of flag leaves of YN and YB at 30 DAF. (**f**) Relative expression of *RCCR1*, *NOL*, *NYC3*, *OsNAP*, *Osh36* and *OsI57* in YN and YB flag leaves at different stages after flowering. The values are the means ± SDs of 3 repeats.

After flowering, YN plants exhibited a delayed green-to-yellow phenotype, becoming senescent approximately 10 days later than the YB plants did (Fig. 1a, b). In addition, compared with the YB plants, the YN plants underwent slower chlorophyll degradation. The most pronounced difference between the YN and YB plants was that, at 30 days after flowering (DAF), the chlorophyll content of YB decreased dramatically, while that of YN showed a slower decrease (Fig. 1c). Darkness is one of the most powerful external stimuli of leaf senescence and is used frequently as an effective method of simulating synchronous senescence^24^. Leaf segments of YN and YB were subjected to darkness for 5 days. We found that, compared with YB, YN showed significantly delayed dark-induced leaf senescence (Fig. 1d). Reactive oxygen species (ROS) constitute one of the factors that promote senescence^25^. We used 3,3′-diaminobenzidine (DAB) staining to examine H_2_O_2_ levels in the two rice cultivars, and the results showed that the YN leaves displayed less H_2_O_2_ accumulation than the YB leaves did (Fig. 1e). Similarly, chlorophyll degradation-related genes (Red Chlorophyll Catabolite Reductase 1 (*RCCR1*), *NYC1* Like (*NOL*) and Non Yellow Colouring 3 (*NYC3*)) and three other senescence-associated genes (SAGs) (*Oryza sativa* NAC-like, activated by apetala 3/pistillata (*OsNAP*), *Osh36* and *OsI57*) were expressed at higher levels in the fully expanded leaves of YB than in those of YN (according to the qPCR results) (Fig. 1f). These results clearly demonstrated that YN is an elite rice cultivar with a strong anti-senescence phenotype at the filling stage.

### Transcriptional analysis reveals a set of differentially expressed genes involved in leaf senescence

**Figure 2:**
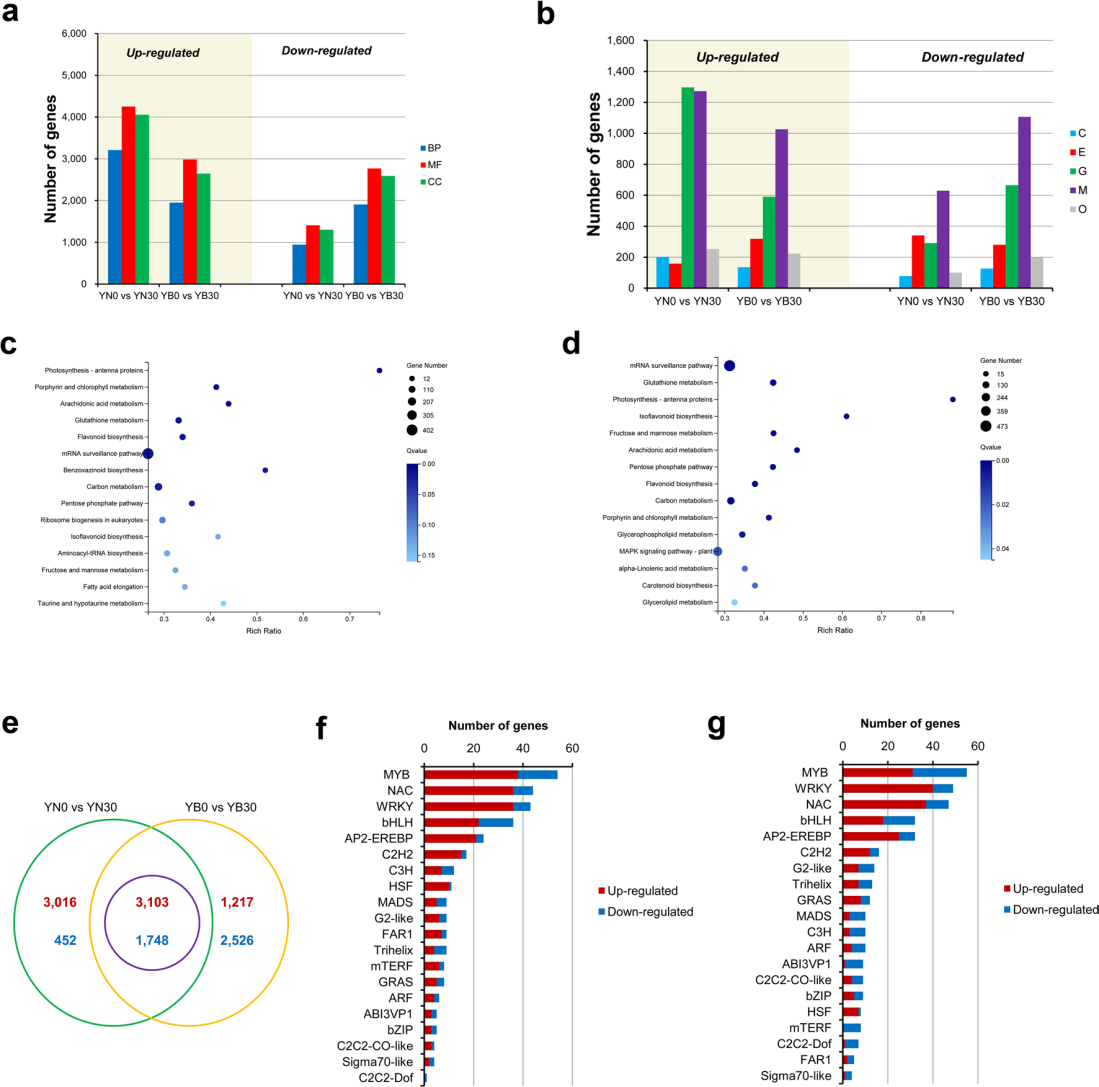
Analysis of differentially expressed genes in YN and YB. (**a**) GO functions of DEGs. *BP* biological process, *MF* molecular function, *CC* cellular component. (**b**) KEGG analysis of DEGs. *C* cellular processes, *E* environmental information processing, *G* genetic information processing, *M* metabolism, *O* organismal systems. (**c**) KEGG enrichment analysis of DEGs in the YN0 versus YN30 comparison. The modules show only the top 15 pathways with the most significant enrichment. (**d**) KEGG enrichment analysis of the DEGs in the YB0 versus YB30 comparison. The modules show only the top 15 pathways with the most significant enrichment. (**e**) Venn diagrams of the number of DEGs in 30 DAF plants compared with 0 DAF plants. The red characters represent numbers of genes whose expression is upregulated, and the blue characters represent numbers of genes whose expression is downregulated. (**f**) Analysis of differentially expressed putative transcription factors associated with the DEGs in the YN0 versus YN30 comparison. (**g**) Analysis of differentially expressed putative transcription factors associated with the DEGs in the YB0 versus YB30 comparison.

To understand the transcriptional landscape in leaves during senescence, we performed an RNA-seq analysis of the flag leaves of YN and YB. We evaluated the flag leaves at 0 DAF and 30 DAF. Pearson’s correlation coefficients between biological replicates indicated high reproducibility of the biological replicates and the array platform (Supplemental Table S1). Nearly 80% of the transcripts were covered more than 60% of the reads, indicating substantial coverage of the transcriptome (Supplemental Table S1). A snapshot of the transcriptome was generated by clustering all the samples using principal component analysis (PCA). Consistent with their distinct developmental programmes and cultivars, the samples of different cultivars at different developmental stages were clustered (Supplemental Figure S1). Compared to those in the 0 DAF samples, the transcripts in 30 DAF samples had 8524 DEGs in YN and 8799 DEGs in YB, with their expression levels differing by twofold or more. Compared to YB, the transcripts in YN had 6311 DEGs at 0 DAF and 3230 DEGs at 30 DAF (Supplemental Table S2). GO and KEGG analyses were performed for the DEGs, revealing information on the annotations of genes involved in biological processes, molecular functions and cellular components (GO analysis) and those involved in cellular processing, environmental information processing, genetic information processing, metabolism and organismal systems (KEGG analysis) (Fig. 2a, b, Supplemental Figure S2, Supplemental Table S3). We analysed the top 15 metabolic pathways that were significantly enriched and found that genes differentially expressed in YN at 30 DAF compared to 0 DAF were significantly enriched in the following: photosynthesis—antenna proteins, porphyrin and chlorophyll metabolism, arachidonic acid metabolism, glutathione metabolism, flavonoid biosynthesis, the mRNA surveillance pathway, benzoxazinoid biosynthesis, carbon metabolism, the pentose phosphate pathway, ribosome biogenesis in eukaryotes, isoflavonoid biosynthesis, aminoacyl-tRNA biosynthesis, fructose and mannose metabolism, fatty acid elongation, taurine and hypotaurine metabolism. Similarly, and found that genes differentially expressed in YB at 30 DAF compared to 0 DAF were significantly enriched in the following: the mRNA surveillance pathway, glutathione metabolism, photosynthesis-antenna proteins, isoflavonoid biosynthesis, fructose and mannose metabolism, arachidonic acid metabolism, the pentose phosphate pathway, flavonoid biosynthesis, carbon metabolism, porphyrin and chlorophyll metabolism, glycerophospholipid metabolism, MAPK signalling pathway-plant, alpha-linolenic acid metabolism, carotenoid biosynthesis, and glycerolipid metabolism (Fig. 2c, d, Supplemental Table S3). Interestingly, YN exhibited more upregulated genes at 30 DAF than at 0 DAF, and YB exhibited more downregulated genes. We identified 3016 genes whose expression was upregulated and 452 genes whose expression was downregulated only in YN0 versus YN30, and we identified 1217 genes whose expression was upregulated and 2526 genes whose expression was downregulated only in the YB0 versus YB30 comparison. A common set of 4851 (3103 upregulated and 1748 downregulated) genes was detected between the YN0 versus YN30 and YB0 versus YB30 comparisons (Fig. 2e). Taken together, these results suggest that at 30 DAF, when YN is still in a period of strong gene expression and metabolism, the gene expression in YB is attenuated (Fig. 2a, b), which is consistent with YB showing an increased-senescence phenotype.

Transcription factors play important regulatory roles in plant growth and development. In total, 385 and 448 transcription factors (TFs) were differentially expressed in YN and YB, respectively (Fig. 2f, g, Supplemental Table S4), 112 TFs of which were downregulated and 273 TFs of which were upregulated in YN plants at 30 DAF compared to 0 DAF, and 185 TFs and 263 TFs were downregulated and upregulated, respectively, in YB plants at 30 DAF compared 0 DAF.

### Metabolome profiling analysis reveals a set of changes in metabolites involved leaf senescence

**Figure 3:**
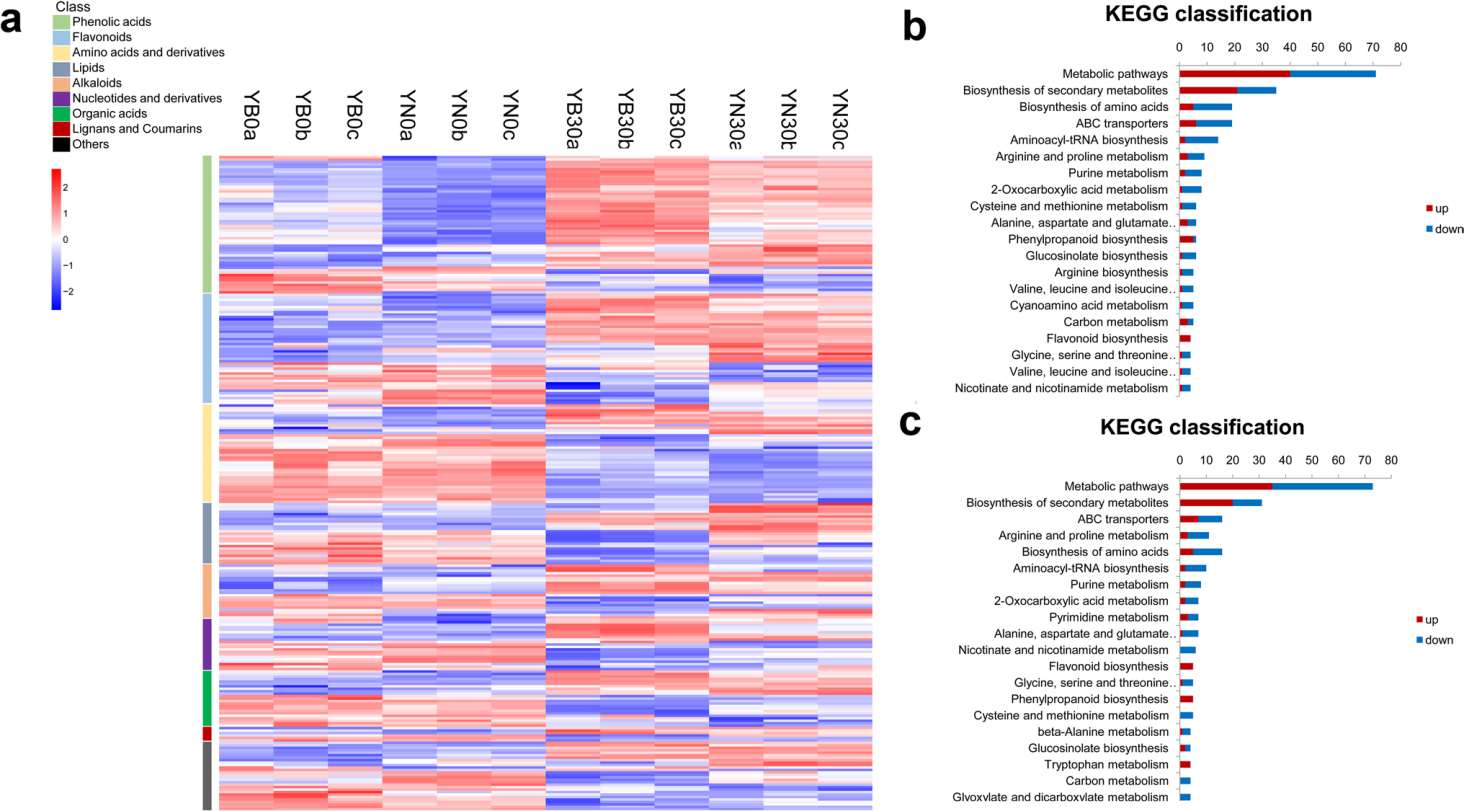
Analysis of differentially accumulated metabolites. (**a**) Heat map analysis of differentially accumulated metabolites. The data are homogenized by row. (**b**) KEGG classification of differentially accumulated metabolites in the YN0 versus YN30 comparison. The abscissa represents the number of metabolites. (**c**) KEGG classification of differentially accumulated metabolites in the YB0 versus YB30 comparison. The abscissa represents the number of metabolites.

To investigate the main metabolic changes reflecting the variation during leaf senescence and to evaluate their putative role in metabolic signalling, a widely targeted metabolome method was used to quantify primary and secondary metabolites identified at different leaf stages. Flag leaves of YN and YB sampled at 0 DAF and 30 DAF were subjected to UPLC–ESI–MS/MS analysis. In this work, 512 and 510 metabolites were identified and quantified in YB and YN, respectively (Supplemental Table S5). PCA showed that YB and YN were clearly separated in the PC1 × PC2 score plots (Supplemental Figure S3). Of the metabolites, 204 markedly changed in YN at 30 DAF compared to 0 DAF, in which the accumulation of 121 metabolites was upregulated and that of 83 metabolites was downregulated. A total of 205 metabolites markedly changed in YB at 30 DAF compared to 0 DAF, with the accumulation of 111 and 94 metabolites was upregulated and downregulated (Supplemental Table S5). Cluster analysis was carried out for different metabolic components, including phenolic acids, amino acids and their derivatives, alkaloids, flavonoids, lipids, nucleotides and their derivatives, organic acids, lignans and coumarins, tannins, etc., which revealed different degrees of expression in the two rice cultivars at 30 DAF compared to 0 DAF (Fig. 3a). Among the content of these metabolites, the content of organic acids and amino acids decreased at the later stage of ageing, and flavonoids, alkaloids and lipids accumulated more in YN at 30 DAF than in YB at 30 DAF. We then classified the different KEGG pathways (Supplemental Table S6). We analysed the top 20 metabolic pathways with the largest number of differentially accumulated metabolites (Fig. 3b, c) and found that, in the two rice cultivars, metabolites involved in the following accumulated more at 30 DAF than at 0 DAF: metabolic pathways; biosynthesis of secondary metabolites; ABC transporters; aminoacyl-tRNA biosynthesis; arginine and proline metabolism; 2-oxocarboxylic acid metabolism; alanine, aspartate and glutamate metabolism; flavonoid biosynthesis; etc. In recent years, we have noticed that secondary metabolism, such as phenylpropanoid metabolism and flavonoid metabolism, has been reported to be involved in reducing oxidative damage^26^. Our results are consistent with those in the literature, further suggesting that the substances involved in these metabolic processes may be involved in the regulation of plant senescence.

### Integrated analysis reveals substantial changes in the flavonoid biosynthesis pathway during leaf senescence

To explore the differences in regulatory mechanisms underlying leaf senescence between the two rice cultivars, differentially accumulated metabolites and DEGs in KEGG pathways were selected for integrated analysis. We selected the metabolic pathways of differentially accumulated metabolites and their corresponding genes (Table 1). It is worth noting that the flavonoid synthesis pathway in both rice cultivars was significantly enriched in differentially accumulated metabolites and DEGs. We rearranged 10 major compounds of flavonoid biosynthesis and 5 DEGs to corresponding positions of the flavonoid biosynthesis pathway (Fig. 4a, b). In the flavonoid biosynthesis pathway, the abundance of 6 compounds increased at 30 DAF compared with 0 DAF between the two cultivars (Fig. 4b, d). Our metabolic analysis revealed that flavonoids and flavonoid derivatives, including phloretin, luteolin and eriodictyol, showed lower abundances in YB than in YN at 30 DAF (Fig. 4c, d). Secondary metabolites, including flavonoids, play an important role in maintaining redox homeostasis in cells by quenching reactive oxygen species^27,28^. The transcriptomic data showed that the relative expression of 5 DEGs (*PAL*, *C4H*, *4CL*, *CHS*, *CHI*) in YN30/YN0 were generally higher than in YB30/YB0. To confirm this, we detected the expression of these five genes by qRT-PCR. The results were consistent with those obtained from RNA sequencing, the relative expression of *C4H*, *4CL*, *CHS* and *CHI* were significantly increased, relative expression of *PAL* increased slightly, but not significantly, indicating that the sequencing data sets are reliable. (Supplemental Figure S4). Taken together, these results suggest that the increased accumulation of flavonoids may be due to alterations in the expression of these DEGs in YN.

**Table 1:** Integrated analysis of differentially expressed genes and differentially accumulated metabolites associated with KEGG pathways.

#Kegg_pathway	ko_id	Gene count	Meta count	Meta ID
**YN**				
Carbon metabolism	ko01200	156	5	C00979,C00025,C00049,C00234,C00122
Glutathione metabolism	ko00480	72	1	C00025
Flavonoid biosynthesis	ko00941	64	4	C01514,C00774,C05631,C09826
Aminoacyl-tRNA biosynthesis	ko00970	58	14	C00152,C00123,C00082,C00407,C00188,C00064,C00025,C00062,C00183,C00049,C00148,C00135,C00073,C00234
Fructose and mannose metabolism	ko00051	39	1	C00794
Porphyrin and chlorophyll metabolism	ko00860	38	2	C00188,C00025
Taurine and hypotaurine metabolism	ko00430	12	1	C00025
**YB**				
Carbon metabolism	ko01200	171	4	C00025,C00049,C00042,C00158
Glutathione metabolism	ko00480	92	2	C00025, C00315
Flavonoid biosynthesis	ko00941	71	5	C01514,C09826,C00774,C12136,C01477
Fructose and mannose metabolism	ko00051	51	1	C00794
Porphyrin and chlorophyll metabolism	ko00860	38	2	C00188,C00025

**Figure 4:**
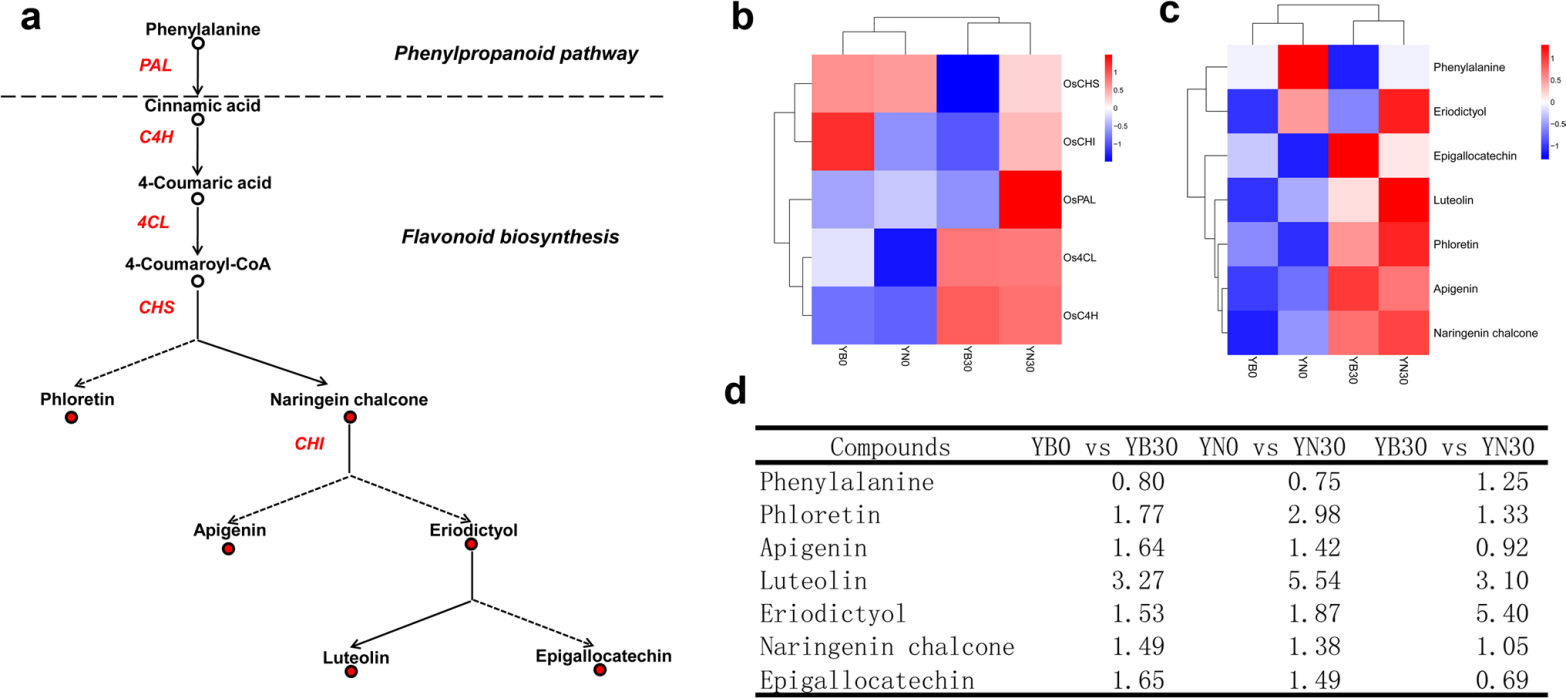
Differentially expressed genes and metabolites in the flavonoid biosynthesis pathway. (**a**) Representative genes and metabolites detected downstream of flavonoid biosynthesis. The red dots represent metabolites detected via metabolomics. *PAL* phenylalanine ammonia-lyase, *C4H* cinnamate 4-hydroxylase, *4CL* 4-coumaroyl-CoA:ligase, *CHS* chalcone synthase, *CHI* chalcone isomerase. (**b**) Heat map analysis of the expression of 5 genes in the flavonoid biosynthesis pathway. The data are homogenized by row. (**c**) Heat map analysis of 7 metabolites in the flavonoid biosynthesis pathway. The data are homogenized by row. (**d**) Metabolites detected in the flavonoid biosynthesis pathway.

### A MYB transcription factor negatively regulates the expression of *OsCHS *and *OsCHI*

**Figure 5:**
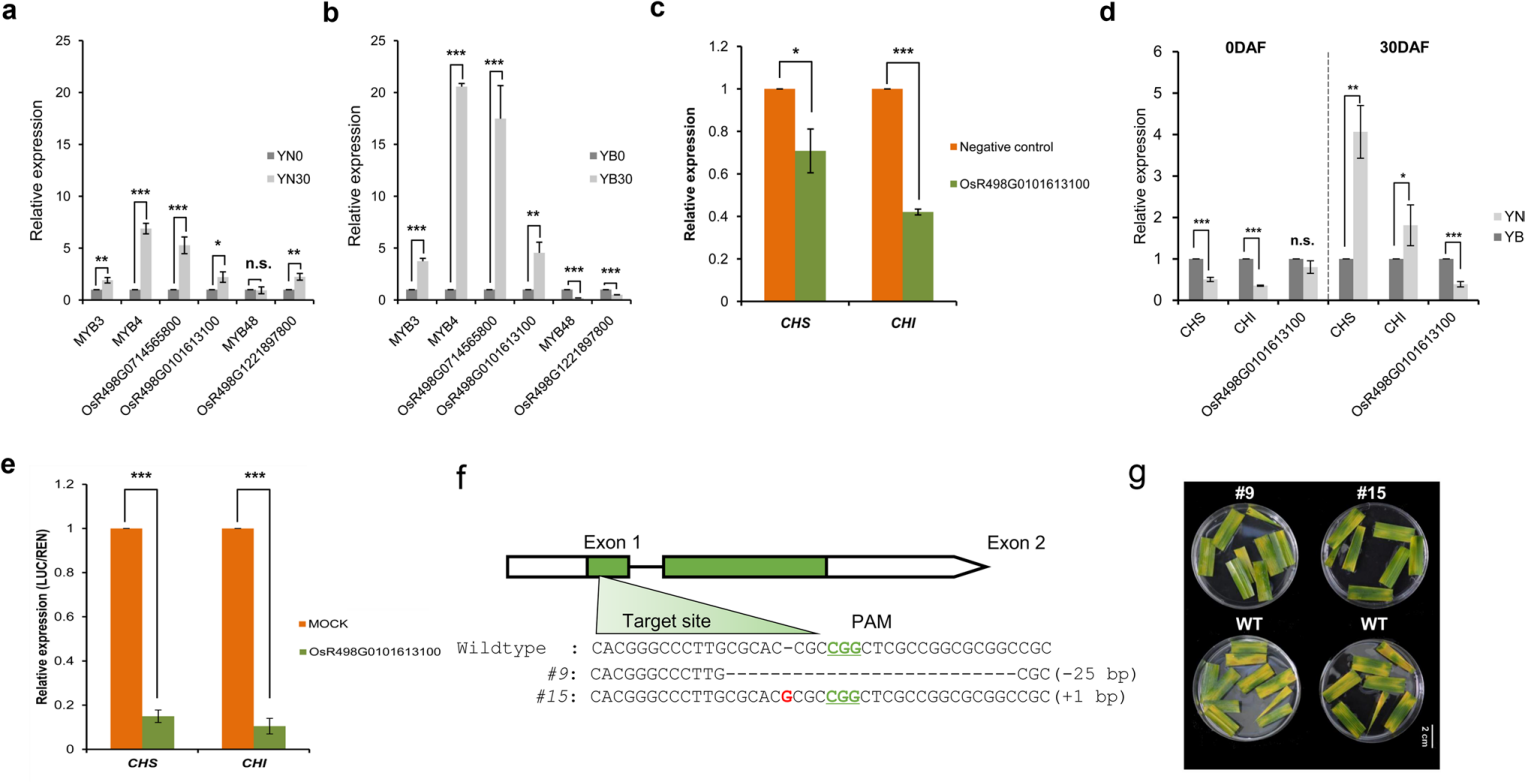
Expression regulation and anti-senescence analyses of MYB TFs. (**a**) qRT-PCR validation of MYB TFs in the YN0 versus YN30 comparison. (**b**) qRT-PCR validation of MYB TFs in the YB0 versus YB30 comparison. (**c**) qRT-PCR results of endogenous *CHS* and *CHI* genes in protoplasts transfected with *OsR498G0101613100*. Statistical analysis was performed using Student’s t-test: *, *P* < 0.05; **, *P* < 0.01; ***, *P* < 0.001; *n.s.* not significant. (**d**) qRT-PCR validation of *OsR498G0101613100*, *OsCHS* and *OsCHI* between YN and YB. (**e**) Promoter activity analysis shows that OsR498G0101613100 significantly suppresses the transcriptional activity of *CHS* and *CHI*. ***, *P* < 0.001*, *t*-test. (**f**) CRISPR-Cas9-mediated knockout of *OsR498G0101613100*. The green box represents the exon. Sequences of target in the CRISPR-Cas9 transgenic lines of rice variety Zhonghua11 (ZH11) were shown. Wild type refers to the sequence of ZH11. #9, #15 refer to the different types of knockout mutants. (**g**) Leaves phenotype after darkness treatment on the ko9 and ko15 mutants and the wild-type control ZH11. Photographs were taken 5 days after darkness treatment. Bar, 2 cm.

Transcription factors play important roles in plant growth, development, reproduction and senescence. As shown above, a large number of MYB transcription factors were differentially expressed at the later filling stage between the two rice cultivars and thus may be involved in important biological functions. Previous studies have reported that MYB TFs are involved in regulating the expression of genes involved in the flavonoid synthesis pathway in plants^29–31^. According to our results, the expression of *OsCHS* and *OsCHI*, which encode chalcone synthase and chalcone isomerase, respectively, was significantly downregulated in YB at 30 DAF, which did not significantly differ from the results of YN (Supplemental Figure S4). We speculate that MYB TFs may be involved in the differential expression of *CHS* and *CHI* between the two rice varieties. To examine the relationship between MYBs and flavonoid synthesis, we first analysed and screened potential MYBs that positively or negatively regulated the expression of *OsCHS* and *OsCHI*. For example, the transcripts of *OsR498G0102785500* (*LOC_Os01g74410*, *MYB48*) and *OsR498G1221897800*(*LOC_Os12g37970*), whose abundance decreased more significantly in YB at 30 DAF, may be involved in the positive regulation of flavonoid synthesis (Fig. 5a, b). By contrast, the transcripts of *OsR498G0102429100* (*LOC_Os01g65370*, *MYB3*), *OsR498G0408899800* (*LOC_Os04g43680*, *MYB4*), *OsR498G0714565800*(*LOC_Os07g37210*) and *OsR498G0101613100*(*LOC_Os01g45090*), whose abundance increased more significantly in YB at 30 DAF, may be involved in the negative regulation of flavonoid synthesis (Fig. 5a, b). We subsequently used protoplast transient assays to determine the activity of different MYB TFs in the activation of *OsCHS* and *OsCHI* transcription, with blank GFP vectors used as negative controls. The expression of endogenous Os*CHS* and *OsCHI* was significantly activated when upon transfection with the 35S:*OsR498G0101613100* plasmid compared with other plasmids (Fig. 5c, Supplemental Figure S5). Comparison of *OsR498G0101613100*, *OsCHS* and *OsCHI* expression between YN and YB showed that expression of *OsCHS* and *OsCHI* were upregulated at 30 DAF in the YB 30 versus YN 30 comparison, while expression of *OsR498G0101613100* was downregulated at 30 DAF in this comparison (Fig. 5d). In addition, the promoter activity analysis also showed that the presence of OsR498G0101613100 could significantly suppress the LUC/REN ratio (Fig. 5e), suggesting that OsR498G0101613100 could essentially inhibit the transcriptional activity of *OsCHS* and *OsCHI*. In addition, we also generated knockout lines of *OsR498G0101613100* in rice by using CRISPR-Cas9-mediated gene editing (Fig. 5f). Functional analysis of two homologous lines showed anti-senescence phenotype after darkness treatment (Fig. 5g). These results indicated that *OsR498G0101613100* plays a negative role in regulating the expression of *OsCHS* and *OsCHI*, which may regulate the accumulation of metabolites in flavonoid biosynthesis pathway.

## Discussion

High-quality rice with high yield, resistance, good quality and other good characteristics is the goal of rice breeding. Leaf colour transformation is one of important traits that been considered by breeders in the late stage of crop development, because it is actually a process of leaf senescence, in which the leaf colour changes from green to yellow, the photosynthetic capacity of leaves gradually decreases and the chlorophyll constantly degrades. It is estimated that the rice yield can be increased by 2% in theory if the life of functional leaves is prolonged one day at maturity stage^32^. Therefore, it is of great importance to cultivate anti-senescence rice varieties for stable and increased yield of rice. Yuenong Simiao is a conventional rice variety with high yield, disease resistance and senescence resistance, and therefore, is of practical significance for theoretical research and application of rice breeding to analyze its anti-senescence mechanism and explore the regulatory factors in the process of senescence.

Leaf senescence is the last stage of plant leaf development, but it is not the least important stage. During this period, a large number of functional genes and metabolites change rapidly, which makes the plant leaves turn from green to yellow, accompanied by the gradual transfer of nutrients to the seeds. Our study combined metabolomics with the transcriptomics to analyse metabolism-related genes and metabolites involved in the process of rice leaf senescence. We found that, at the late stage of ageing, the contents of organic acids and amino acids in the plants decreased, which was consistent with findings associated with plant ageing and development^33^. At the late stage of development, nitrogen is used for the sink/source transition, and the supply of nitrogen from leaves is weakened; the levels of the majority of amino acids and organic acids during this time have been studied^16^. On the other hand, at 30 DAF, the anti-senescence variety YN accumulated more flavonoids, alkaloids and lipids than did YB, which may be due to the specificity of the YN variety. In the senescence process of Arabidopsis rosette leaves, contents decreased for chloroplast-localized lipid species; for example, the monogalactosyl diacylglycerols (MGDGs) and digalactosyl diacylglycerols (DGDGs) in the chloroplasts were degraded continuously during this process. Other lipid species, such as diacylglycerols, triacylglycerides (TAGs), ceramides, and glucosylceramides, and lysolipids such as lysophosphatidylethanolamines and lysophosphatidylcholines continuously increased during leaf senescence, which showed much higher accumulation in siliques than in senescent leaves^34^. In our study, the content of several free fatty acids increased at 30 DAF, including the following: lauric acid and 9,10-dihydroxy-12-octadecenoic acid; some sphingolipids, such as D-erythro-dihydrosphingosine and 4-hydroxysphinganine; and lysophosphatidylcholines, such as LysoPC 17:0 and LysoPC 18:0. However, lysophosphatidylcholines such as LysoPC 16:0, LysoPC 18:4, and LysoPC 18:3 increased the most at 30 DAF in YN (Supplemental Table S5). These differences in lipid metabolite content patterns may be related to the difference in senescence patterns between monocotyledons and dicotyledons.

Through integrated analysis of transcriptional and metabolic data, we focused on changes in the flavonoid biosynthesis pathway. Flavonoids have been reported to be involved in reducing oxidative damage in plants^35,36^. The levels of flavonol glycosides and anthocyanins can be increased by overexpressing flavonoid structural genes such as *CHS* and DIHYDROFLAVONOL-4-REDUCTASE (*DFR*), which resulted in a reduction in ROS accumulation and enhanced tolerance to salt stress in rice^37^. In this pathway, phloretin, luteolin and eriodictyol, which were previously reported to be involved in attenuating oxidative stress, were detected to accumulate the most in YN 30 DAF (Fig. 4c, d)^38–41^. These compounds are antioxidant regulators in human and mouse cells, but their activity in plants has not been reported. Nevertheless, we speculate that these compounds may be associated with the anti-senescence phenotype of YN. However, apigenin, naringenin chalcone, and epigallocatechin also accumulated at 30 DAF. Due to their small differences between the two varieties, we did not focus on these compounds.

MYB TF family genes have been identified in different monocot and dicot species and have a wide range of important biological functions. MYB TFs are also involved in the regulation of plant flavonoid synthesis pathways in different species concerning the regulation of plant secondary metabolic responses. For example, *AtMYB111*, *AtMYB12*, and *AtMYB11* are all independently capable of activating the expression of flavonol synthase (*FLS*), flavanone 3-hydroxylase (*F3H*), chalcone isomerase (*CHI*), and chalcone synthase (*CHS*)^42–44^. *GbMYBFL* has been reported to enhance the accumulation of flavonoids^45^, *DmMYB1* and *CsMYB4a* negatively regulate the synthesis of flavonoids^46^, *GtMYBP3* and *GtMYBP4* activate the expression of flavonol synthesis genes^47^, and so on. However, research on the relationship between MYBs and leaf senescence is limited. *OsMYB102* inhibits ABA accumulation and downregulates ABA signalling responses to delay rice leaf senescence^48^. We found that the MYB TF *OsR498G0101613100* may negatively regulate flavonoid biosynthesis to affect the antioxidant content. Furthermore, relationships between MYB TFs and the flavonoid pathway have been reported in citrus species. *CsMYBF1* functions in controlling flavonol and hydroxycinnamic acid biosynthesis; interestingly, *CsMYBF1* could activate the *CHS* gene promoter in *Citrus* but not in tomato^49^. We found that the MYB TF *OsR498G0101613100* may inhibit the expression of *CHS* and *CHI*, although whether this regulation occurs directly requires further experimental verification. *CHI* was previously reported to be involved in the regulation of hull and internode colour^50^. However, we detected the expression of *CHI* in flag leaves. Further functional experiments are needed to confirm whether *OsR498G0101613100* regulates *CHI* directly during leaf senescence.

## Methods

### Plant materials and growth conditions

Leaf senescence assays were conducted under field conditions. For RNA-seq and gene expression analysis, various flag leaves were collected starting from the heading stage. To determine the precise expression patterns in YN and YB, flag leaves were harvested every 10 days after flowering. To generate the knockout lines of *OsR498G0101613100*, the gRNA was designed to target the Exon 1 of this gene. The target sequence (+ PAM) is 5′-CACGGGCCCTTGCGCACCGCCGG-3′. The constructs were then introduced into pYLCRISPR/Cas9Pubi-H vector and then transformed into the *japonica* rice Zhonghua11 cultivar by the *Agrobacterium*-mediated method^51^.

### Chlorophyll and leaf SPAD measurements

Chlorophyll was extracted from 100 mg of leaf tissue (fresh weight), after which its content was determined by measuring the absorbance at 470 nm, 645 nm and 663 nm using a Varioskan Flash spectrophotometer (Thermo Scientific) as described previously^52^. The SPAD values of the flag leaves were determined by a SPAD-502 m.

### Darkness-induced leaf senescence

Fully expanded flag leaves were excised carefully. The detached leaves were cut into 3 cm pieces and floated on 25 ml of water in Petri dishes, with the adaxial leaf side up. The samples were then incubated at 28 °C in darkness for 5 days.

### Expression analysis

Total RNA was extracted from leaf tissues or protoplasts using an RNAiso Plus Kit (Takara) according to the manufacturer’s instructions. The RNA was reverse-transcribed using a Prime Script RT Reagent Kit together with gDNA Eraser (Takara). Quantitative real-time PCR was then performed using a TB Green Premix Ex Taq Kit (Takara) in conjunction with a CFX96 Real-Time PCR Detection System (Bio-Rad) according to the manufacturer’s instructions. Rice *OsActin1* was used as an internal control. The primers used for quantitative RT-PCR (qRT-PCR) are listed in Supplemental Table S7. The values reported are the means ± SDs of three biological repeats. Student’s *t*-test was used for statistical analysis.

### RNA-seq analysis

Total RNA was extracted from the flag leaves of rice plants at 0 DAF and 30 DAF. Three replicate RNA-seq libraries were prepared from YB0, YB30, YN0 and YN30. A total of 12 libraries were sequenced separately using a BGISEQ-500 sequencer (BGI-Shenzhen, China). Raw sequencing reads were cleaned by removing adaptor sequences, reads containing poly-N sequences, and low-quality reads. Approximately 40 M clean reads were mapped to the R498 reference genome (http://mbkbase.org/R498/) using HISAT2/Bowtie2 tools^53,54^. After the data were mapped, normalization was performed, and then FPKM (fragments per kilobase per million mapped reads) values were calculated using RESM software^55^. As previously described^56^, an adjusted *P* value ≤ 0.001 and a fold-change ≥ 2 were used to identify differentially expressed genes (DEGs) in the YB0 versus YB30, YN0 versus YN30, YN0 versus YB0 and YN30 versus YB30 sample comparisons. GO (www.geneontology.org) and KEGG (www.kegg.jp) enrichment analyses of annotated differentially expressed genes were performed by phyper (https://en.wikipedia.org/wiki/Hypergeometric_distribution) based on the hypergeometric test. The significance levels of terms and pathways were corrected by Q values with a rigorous threshold (Q value ≤ 0.05) via Bonferroni corrections. TF annotation analysis was performed by getorf (http://emboss.sourceforge.net/apps/cvs/emboss/apps/getorf.html) and HMMsearch (http://hmmer.org).

### Sample extraction and analysis of metabolites by ultra-performance liquid chromatography and tandem mass spectrometry

The flag leaves of rice plants at 0 DAF and 30 DAF were first freeze dried. Sample extract analysis, metabolite identification and quantification were performed at Wuhan MetWare Biotechnology Co., Ltd. (www.metware.cn), using a widely targeted metabolome method and in accordance with their standard procedures, as described in full previously^57,58^. Metabolite data analysis was conducted with Analyst 1.6.1 software (AB SCIEX, Ontario, Canada). The supervised multivariate method of partial least squares-discriminant analysis (PLS-DA) was used to maximize the metabolome differences between the pairs of samples. The relative importance of each metabolite to the PLS-DA model was checked using the variable importance in projection (VIP). Metabolites with a VIP ≥ 1 and a fold-change ≥ 1.5 or a fold-change ≤ 0.67 were considered differentially accumulated metabolites for group discrimination in the YB0 versus YB30 and YN0 versus YN30 sample comparisons.

### Integrated metabolome and transcriptome analysis

Differentially accumulated metabolites and DEGs in KEGG pathways were selected for integrated analysis. Differentially accumulated metabolites used for the integrated analysis were selected according to the information in Supplemental Table S6, and the DEGs used for the integrated analysis were selected from the top 15 metabolic pathways that were significantly enriched. We then analysed the overlapping metabolites and differentially expressed genes within these KEGG pathways.

### Protoplast isolation and transfection

Ten-day-old rice plants were used for protoplast isolation according to a previously described procedure^59,60^. Briefly, the rice seedlings were cut into 0.5 mm strips and digested in an enzyme solution consisting of 1.5% Cellulase R10, 0.4% macerozyme R10, 0.4 M mannitol, 20 mM MES (pH 5.7), 20 mM KCl, 10 mM CaCl_2_ and 0.1% BSA for 3 h. After mixing with 10 ml of W5 solution consisting of 154 mM NaCl, 125 mM CaCl_2_, 5 mM KCl and 2 mM MES (pH 5.7), the digestion mixture was filtered through a Falcon cell strainer. Cells were collected by centrifugation for 2 min at 150 × g, resuspended in 10 ml of W5 solution and then incubated on ice for 30 min. After centrifugation for 1 min, the cell pellet was resuspended in MMg solution consisting of 0.4 M mannitol, 15 mM MgCl_2_ and 4 mM MES (pH 5.7) to a concentration of 2 × 10^5^ cells/ml. DNA transfection was carried out in a 2 ml round-bottom microcentrifuge tube, where 200 μl of protoplasts, 20 μl of DNA (2 μg/μl), and 220 μl of PEG solution (40% PEG 4000 (v/v), 0.2 M mannitol and 0.1 M CaCl_2_) were mixed together. The mixture was subsequently incubated at room temperature for 15 min before the transfection was quenched by adding 800 μl of W5 solution. The transfected cells were harvested by centrifugation for 3 min at 150 × g and then incubated in a 1 ml WI solution consisting of 0.5 M mannitol, 20 mM KCl and 4 mM MES (pH 5.7) in the dark for 12 h for RNA extraction.

### Staining for hydrogen peroxide DAB-based analysis

Hydrogen peroxide was visually detected in leaves using DAB staining following the method described before^61^. The leaf samples were soaked in 1 mg/ml 3,3′-diaminobenzidine (DAB) for 8 h in the dark at 25 °C before being heated in a boiling 95% ethanol bath for 10 min for destaining. Finally, the samples were equilibrated for 4 h in ethanol at 25 °C.

### Promoter activity analysis

The 1384 bp and 2000 bp DNA fragments upstream of the starting codons of *CHS* and *CHI* genes, respectively, were amplified by polymerase chain reaction. The resulting promoter fragments of *OsCHS* and *OsCHI* were inserted into the pGreenII 0800-LUC vector to generate the reporter construct. The MYB transcriptional factor was fused with GFP in its C terminus, results in the effector construct of Ubi::OsR498G0101613100-GFP. The Ubi::GFP vector was used as the negative control. Different combinations of these above constructs were used for co-transfection in rice protoplasts. After co-transfection, the protoplasts were incubated at 28 °C for 12 h, and then total RNA were extracted. The reporter gene expression was calculated as relative ratio of *LUC* to *REN* used qRT-PCR. Student’s *t*-test was used for statistical analysis.

### Statement on the use of plant

The authors declare that the use of plants parts in the present study complies with international, national and institutional guidelines.

### Permissions statement

The authors declare that the collection of plant specimens comply with the IUCN Policy Statement on Research Involving Species at Risk of Extinction and the Convention on the Trade in Endangered Species of Wild Fauna and Flora.

## Supplementary information

Supplementary Information.
