# Supplementary material for: Integrated transcriptomic and metabolomic analysis provides insight into the regulation of leaf senescence in rice
Source: Sci Rep. 2021 Jul 8;11:14083. doi: 10.1038/s41598-021-93532-x (PMC8266841; doi:10.1038/s41598-021-93532-x)
Supplement: Supplementary file 1 — Supplementary Information. [file 41598_2021_93532_MOESM1_ESM.pdf]

Integrated transcriptomic and metabolomic analysis provides insight into the regulation of leaf senescence in rice

Authors: Jiao Xue[1,#], Dongbai Lu[1,#], Shiguang Wang[1], Zhanhua Lu[1], Wei Liu[1], Xiaofei Wang[1], Zhiqiang Fang[1], Xiuying He[1,*]

Affiliation:

[1] Rice Research Institute, Guangdong Academy of Agricultural Sciences, Guangdong Key Laboratory of New Technology in Rice Breeding, Guangzhou 510640, China.

[#] These authors contributed equally to this work.

***Corresponding author:**

Rice Research Institute, Guangdong Academy of Agricultural Sciences, Guangzhou 510640, P. R. China

Phone: 86-20-85266185

Fax: 86-20-87596559

Email: hexiuying@gdaas.cn to X.Y. He

Fig. S1. Principle component analysis of samples seuquenced in RNA-seq.

Fig. S2. GO functions and KEGG analysis of differentially expressed genes in YB0 vs YN0 and YB30 vs YN30.

Fig. S3. Principle component analysis of samples tested in metabolome profiling analysis.

Fig. S4. qRT-PCR verification of 5 DEGs in flavonoids biosynthesis pathway.

Fig. S5. qRT-PCR results of endogenous *CHS* and *CHI* in protoplast transfection for MYB plasmids.

Table S1. Pearson’s correlation and transcripts coverage percentage in RNA-seq.

Table S2. Differentially expressed genes.

Table S3. GO and KEGG pathways analysis of differentially expressed genes.

Table S4. Differentially expressed TFs in YN and YB.

Table S5. Metabolites identified in YN and YB and markedly changed metabolites.

Table S6. Classification of differential KEGG pathways.

Table S7. qRT-PCR primers used in this study.

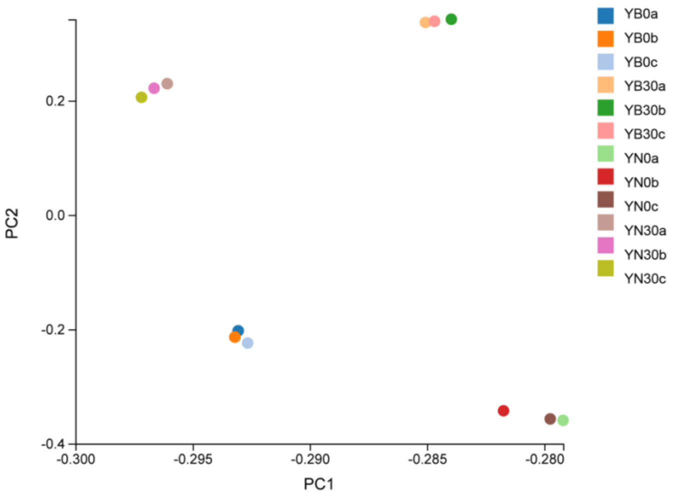

Supplemental Fig. S1. Principle component analysis of samples seuquenced in RNA-seq.

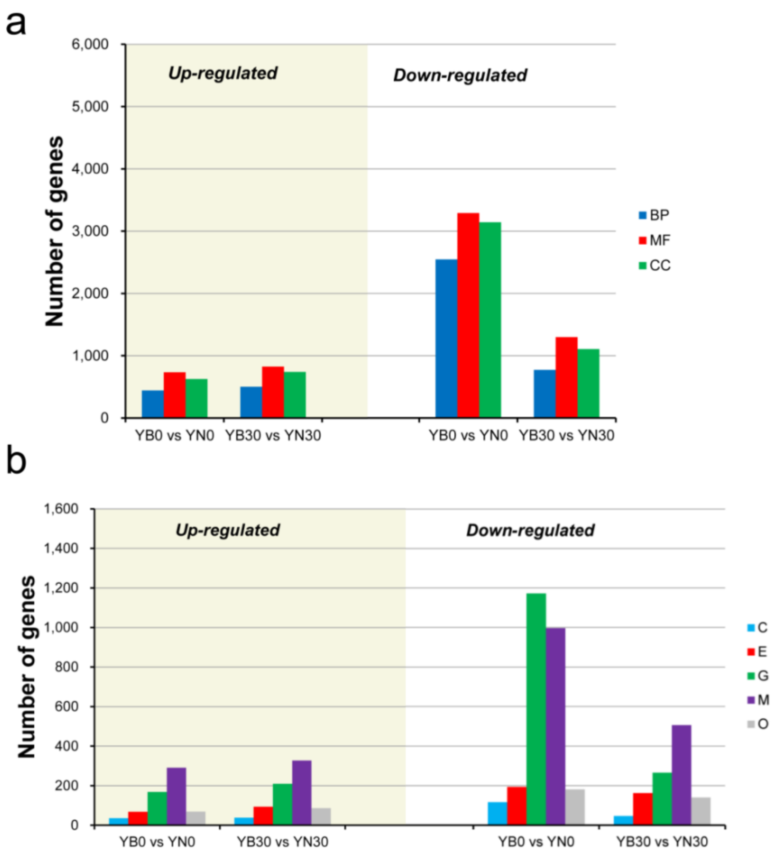

Supplemental Fig. S2. GO functions and KEGG analysis of differentially expressed genes in YB0 vs YN0 and YB30 vs YN30.

(a) GO functions of DEGs. BP, biological process; MF, molecular function; CC, cellular component. (b) KEGG analysis of DEGs. C, Cellular processes; E, Environmental information processing; G, Genetic information processing; M, Metabolism; O, Organismal systems.

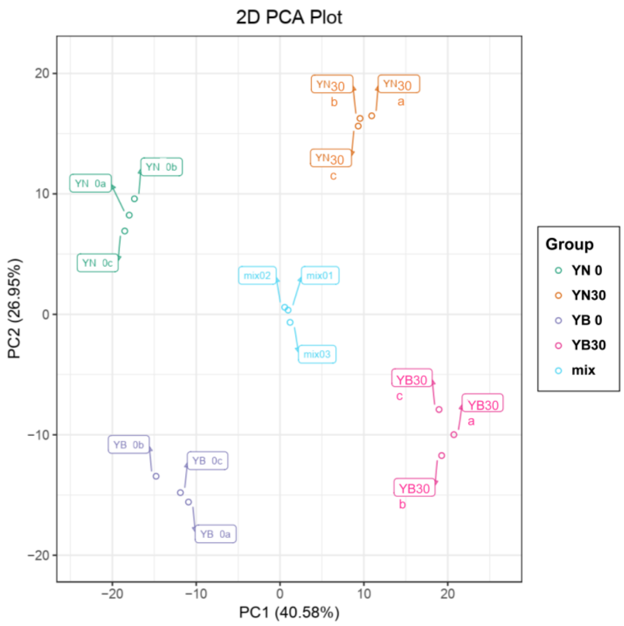

Supplemental Figure S3. Principle component analysis of samples tested in metabolome profiling analysis.

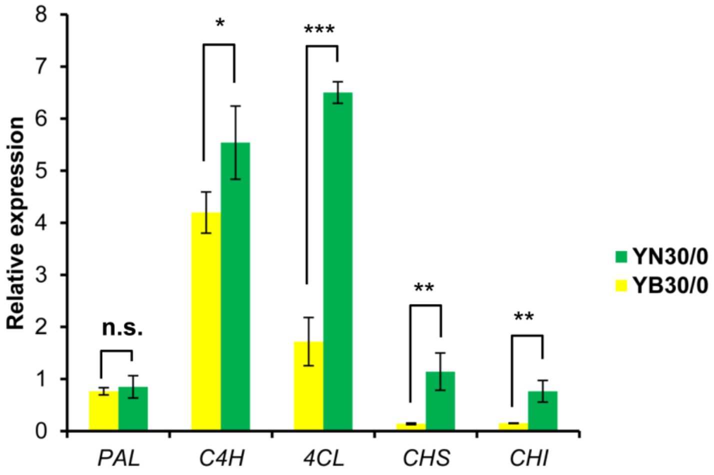

Supplemental Fig. S4. qRT-PCR verification of 5 DEGs in flavonoids biosynthesis pathway.

Statistical analysis was performed using Student's t-test: *, $P < 0.05$; **, $P < 0.01$; ***, $P < 0.001$; n.s., not significant.

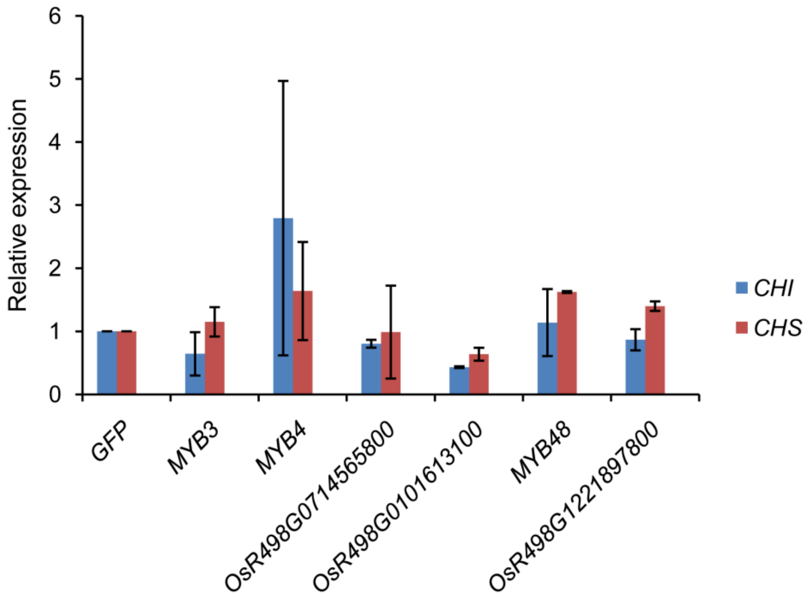

Supplemental Fig. S5. qRT-PCR results of endogenous *CHS* and *CHI* in protoplast transfection for MYB plasmids.

Supplemental Table S1 Pearson's correlation and transcripts coverage percentage in RNA-seq

Pearson's correlation in RNA-seq

Sample	YB0a	YB0b	YB0c	YB30a	YB30b	YB30c	YN0a	YN0b	YN0c	YN30a	YN30b	YN30c
YB0a	1	0.9989	0.9988	0.8038	0.799	0.8024	0.9222	0.9275	0.924	0.8687	0.8718	0.8771
YB0b	0.9989	1	0.9993	0.7998	0.7945	0.7982	0.9293	0.9343	0.9311	0.8672	0.8707	0.8769
YB0c	0.9988	0.9993	1	0.7954	0.7907	0.7939	0.9333	0.9376	0.9346	0.8616	0.8648	0.8705
YB30a	0.8038	0.7998	0.7954	1	0.9993	0.9994	0.7162	0.7297	0.7186	0.979	0.9774	0.9722
YB30b	0.799	0.7945	0.7907	0.9993	1	0.9998	0.7113	0.7247	0.7134	0.9759	0.9743	0.9677
YB30c	0.8024	0.7982	0.7939	0.9994	0.9998	1	0.7142	0.7279	0.7165	0.9779	0.9765	0.9701
YN0a	0.9222	0.9293	0.9333	0.7162	0.7113	0.7142	1	0.9995	0.9998	0.7844	0.7895	0.797
YN0b	0.9275	0.9343	0.9376	0.7297	0.7247	0.7279	0.9995	1	0.9997	0.7973	0.8024	0.8094
YN0c	0.924	0.9311	0.9346	0.7186	0.7134	0.7165	0.9998	0.9997	1	0.7871	0.7922	0.8
YN30a	0.8687	0.8672	0.8616	0.979	0.9759	0.9779	0.7844	0.7973	0.7871	1	0.9998	0.9984
YN30b	0.8718	0.8707	0.8648	0.9774	0.9743	0.9765	0.7895	0.8024	0.7922	0.9998	1	0.9987
YN30c	0.8771	0.8769	0.8705	0.9722	0.9677	0.9701	0.797	0.8094	0.8	0.9984	0.9987	1

Transcripts coverage percentage in RNA-seq

Percent covered(%)	Percentage of Transcripts(%)											
	YB0a	YB0b	YB0c	YB30a	YB30b	YB30c	YN0a	YN0b	YN0c	YN30a	YN30b	YN30c
0-10	0.9	0.87	0.81	0.86	0.92	0.89	1.13	1.06	1.14	0.86	0.88	0.97
10-20	3.01	2.99	2.91	3.23	3.31	3.18	4.05	3.94	4.05	3.12	3.19	3.43
20-30	3.64	3.6	3.51	3.92	3.87	3.92	4.83	4.87	4.74	3.85	3.85	3.99
30-40	4.35	4.18	4	4.49	4.64	4.51	5.25	5.36	5.5	4.46	4.34	4.55
40-50	5.33	5.36	5.01	5.43	5.58	5.45	6.26	6.3	6.53	5.48	5.46	5.51
50-60	6.91	6.82	6.61	6.94	6.99	7.07	7.77	7.84	7.76	6.79	6.88	7.17
60-70	9.62	9.39	9.27	9.43	9.41	9.45	9.94	10.1	10.18	9.42	9.36	9.44
70-80	13.92	13.59	13.33	13.04	13.13	13.07	13.56	13.55	13.41	13.18	12.99	12.83
80-90	20.35	20.18	20.38	19.07	19.42	19.43	18.91	18.99	18.78	19.32	19.47	19.19
90-100	31.97	33.02	34.18	33.58	32.74	33.02	28.3	27.99	27.92	33.52	33.59	32.93

Supplemental Table S2 Differentially expressed genes.

Differentially expressed genes in YB0 vs YB30

Gene ID	YB0 FPKM	YB30 FPKM	log2(YB30/YB0)	Qvalue(YB0 vs YB30)	Pvalue(YB0 vs YB30)	YB0a FPKM	YB0b FPKM	YB0c FPKM	YB30a FPKM	YB30b FPKM	YB30c FPKM
OsR498G0022198100.01	0	0.063	4.275487	0.0008443	0.0020202	0	0	0	0.07	0.07	0.05
OsR498G0022198700.01	0.093	0.263	1.563755	6.53E-05	0.0001442	0.07	0.11	0.1	0.24	0.27	0.28
OsR498G0022202400.01	0.053	0.18	1.791235	1.63E-07	3.13E-07	0.12	0.04	0	0.21	0.17	0.16
OsR498G0022202500.01	0.233	0.823	1.836857	1.00E-17	1.42E-17	0.21	0.35	0.14	1.04	0.42	1.01
OsR498G0022209200.01	0.296	0.72	1.369676	2.62E-07	5.10E-07	0.35	0.3	0.24	0.76	0.61	0.79
OsR498G0022210100.01	0.196	0.453	1.210646	5.14E-06	1.07E-05	0.22	0.22	0.15	0.63	0.34	0.39
OsR498G0022212500.01	0.893	0.22	-2.00063	2.05E-13	3.22E-13	1.05	0.57	1.06	0.14	0.43	0.09
OsR498G0022215800.01	0.076	0.156	1.044273	0.0006362	0.0015088	0.09	0.1	0.04	0.13	0.15	0.19
OsR498G0022220000.01	0.14	0.29	1.040412	0.0002082	0.0004755	0.17	0.09	0.16	0.43	0.18	0.26
OsR498G0022222200.01	0.193	0	-4.5025	0.0002635	0.0006068	0	0.58	0	0	0	0
OsR498G0022223500.01	0.48	1.056	1.137371	9.33E-07	1.86E-06	0.63	0.45	0.36	0.96	1.41	0.8
OsR498G0022225600.01	0.093	0.233	1.300225	0.0001647	0.0003733	0.1	0.17	0.01	0.2	0.24	0.26
OsR498G0022229900.01	0.25	0.61	1.321074	7.15E-13	1.14E-12	0	0.75	0	0	1.15	0.68
OsR498G0022230200.01	0.193	0	-4.5025	0.0002635	0.0006068	0	0.58	0	0	0	0
OsR498G0022235600.01	34.853	12.006	-1.56574	7.34E-51	6.52E-51	29.99	32.82	41.75	12.1	11.82	12.1
OsR498G0022236200.01	12.32	2.676	-2.22927	1.27E-21	1.65E-21	18.21	11.36	7.39	2.93	2.18	2.92
OsR498G0022236400.01	110.45	19.476	-2.50757	0	0	124.99	99.69	106.7	21.29	15.72	21.42
OsR498G0022237000.01	43.823	19.13	-1.19735	5.52E-225	2.09E-225	51.56	41.94	37.97	20.59	17.93	18.87
OsR498G0022237600.01	73.103	32.416	-1.20141	0	0	76.97	71.17	71.17	33.96	32.52	30.77
OsR498G0022237700.01	80.223	16.526	-2.27538	0	0	78.62	87.68	74.37	18.54	13.11	17.93
OsR498G0022238000.01	5.666	2.48	-1.19392	2.00E-60	1.63E-60	6.04	5.82	5.14	2.39	2.42	2.63
OsR498G0022238200.01	0.72	3.68	2.318177	1.39E-135	7.20E-136	0.27	0.58	1.31	2.11	4.55	4.38
OsR498G0022238900.01	43.823	19.13	-1.19735	5.52E-225	2.09E-225	51.56	41.94	37.97	20.59	17.93	18.87
OsR498G0022240800.01	113.47	32.133	-1.81648	0	0	123.05	108.78	108.6	33.05	32.28	31.07
OsR498G0022240900.01	0.793	5.686	2.214747	1.50E-87	9.97E-88	1.03	0.64	0.71	7.17	4.76	5.13
OsR498G0100001500.01	0.51	0.113	-2.17656	6.24E-11	1.06E-10	0.65	0.56	0.32	0.16	0.12	0.06
OsR498G0100006700.01	101.44	43.286	-1.23727	1.05E-269	3.50E-270	103.8	99.13	101.4	44.39	42.8	42.67
OsR498G0100011500.01	7.64	17.426	1.17801	1.48E-58	1.22E-58	7.99	7.79	7.14	17.64	18.32	16.32
OsR498G0100011700.01	210.98	43.596	-2.29353	0	0	213.06	211.55	208.3	42.2	45.04	43.55
OsR498G0100012400.01	2.813	0.51	-2.4838	5.15E-08	9.70E-08	2.54	3.27	2.63	0.19	0.96	0.38
OsR498G0100012800.01	230.67	16.95	-3.76883	0	0	230.99	231.07	230	18.73	16.11	16.01
OsR498G0100013300.01	2.243	1.033	-1.11938	3.84E-12	6.29E-12	2.03	2.35	2.35	1.28	0.87	0.95
OsR498G0100014200.01	17.043	35.243	1.046506	1.59E-88	1.05E-88	17.86	16.91	16.36	35.68	35.08	34.97
OsR498G0100014300.01	0.62	3.36	2.401845	7.19E-50	6.46E-50	0.61	0.78	0.47	4.1	3.1	2.88
OsR498G0100015900.01	0.41	2.96	2.847115	6.07E-21	8.04E-21	0.36	0.45	0.42	2.79	3.55	2.54
OsR498G0100016300.01	134.72	36.53	-1.88757	0	0	132.59	135.83	135.8	37.87	34.45	37.27
OsR498G0100023400.01	9.243	130.47	3.812742	0	0	8.23	9.21	10.29	136.61	128.21	126.59
OsR498G0100023900.01	0.73	0.376	-1.02164	2.95E-07	5.75E-07	0.5	0.83	0.86	0.33	0.43	0.37
OsR498G0100024500.01	5.643	2.623	-1.11131	4.26E-61	3.44E-61	5.27	6.03	5.63	2.32	2.84	2.71
OsR498G0100024800.01	403.83	159.03	-1.35949	0	0	402.7	402.97	405.8	158.04	156.12	162.92
OsR498G0100026400.01	0.386	0.09	-1.69721	0.000322	0.0007458	0.34	0.34	0.48	0.06	0.12	0.09
OsR498G0100027300.01	0.72	0.146	-2.26402	2.19E-11	3.67E-11	0.65	0.78	0.73	0.11	0.11	0.22
OsR498G0100030100.01	0.703	0	-7.28217	2.04E-19	2.80E-19	0.47	0.84	0.8	0	0	0
OsR498G0100033300.01	4.323	1.486	-1.64317	1.93E-53	1.67E-53	4.1	4.42	4.45	1.57	1.31	1.58
OsR498G0100033900.01	11.096	26.033	1.240613	2.67E-119	1.49E-119	11.32	10.64	11.33	28.05	25.76	24.29
OsR498G0100034600.01	0.92	0.166	-2.47365	1.41E-25	1.71E-25	0.92	0.86	0.98	0.16	0.15	0.19
OsR498G0100035600.01	0.793	0.016	-5.6041	1.77E-13	2.79E-13	0.52	1.09	0.77	0	0.05	0
OsR498G0100035900.01	1.12	0.443	-1.36463	0.0001911	0.0004353	1.21	1.27	0.88	0.54	0.24	0.55
OsR498G0100038300.01	3.456	1.076	-1.75542	3.47E-17	4.99E-17	4.09	3.05	3.23	1.42	0.97	0.84
OsR498G0100043700.01	0.04	0.173	2.03976	0.0006606	0.0015689	0.07	0.03	0.02	0.15	0.24	0.13
OsR498G0100044700.01	0.873	0.233	-1.91444	7.35E-08	1.39E-07	0.8	0.8	1.02	0.26	0.18	0.26
OsR498G0100045000.01	2.916	14.13	2.150755	2.30E-138	1.18E-138	3.07	2.77	2.91	14.53	14.13	13.73
OsR498G0100045100.01	0.27	2.286	3.075322	1.02E-49	9.13E-50	0.38	0.18	0.25	2.06	2.08	2.72
OsR498G0100045700.01	0.206	0.903	2.101578	1.29E-14	1.97E-14	0.21	0.17	0.24	0.66	0.98	1.07
OsR498G0100047600.01	2.55	1.15	-1.15163	2.80E-13	4.43E-13	2.45	2.72	2.48	1.07	1.18	1.2
OsR498G0100048500.01	0.523	1.126	1.102044	6.91E-05	0.0001529	0.41	0.67	0.49	1.17	1.4	0.81
OsR498G0100052000.01	0.216	0.45	1.068004	1.73E-05	3.69E-05	0.28	0.22	0.15	0.39	0.44	0.52
OsR498G0100053600.01	17.77	2.826	-2.65254	0	0	19.12	16.85	17.34	2.97	2.51	3
OsR498G0100053700.01	36.483	5.003	-2.83387	5.58E-291	1.76E-291	38.05	38.74	32.66	4.89	5.36	4.76
OsR498G0100056000.01	4.6	10.843	1.200848	2.90E-95	1.83E-95	4.3	4.54	4.96	10.26	11.62	10.65
OsR498G0100056100.01	4.183	10.49	1.293019	3.51E-49	3.18E-49	3.88	4.3	4.37	10.85	10.49	10.13
OsR498G0100056600.01	7.23	0.463	-3.9701	3.30E-177	1.46E-177	7.2	7.3	7.19	0.54	0.4	0.45
OsR498G0100056700.01	3.476	1.203	-1.5279	3.27E-20	4.40E-20	3.15	3.48	3.8	1.17	1.08	1.36
OsR498G0100060600.01	1.706	12.793	3.034477	8.00E-234	2.95E-234	1.15	1.83	2.14	13.03	12.52	12.83
OsR498G0100064800.01	28.44	12.746	-1.09481	6.80E-239	2.46E-239	28.52	29.05	27.75	12.18	12.63	13.43

OsR498G0100065000.01	8.716	52.52	2.585387	0	0	10.51	8.25	7.39	52.13	55.78	49.65
OsR498G0100065900.01	6.866	18.806	1.446266	4.15E-96	2.61E-96	6.27	7.39	6.94	20.04	18.8	17.58
OsR498G0100070300.01	0.323	0	-4.28217	0.0007127	0.0016975	0.49	0.1	0.38	0	0	0
OsR498G0100070800.01	0.096	1.076	3.476055	3.66E-10	6.37E-10	0	0.15	0.14	0.96	1.17	1.1
OsR498G0100072700.01	0.32	0.056	-2.4838	7.36E-05	0.0001629	0.29	0.31	0.36	0.04	0.09	0.04
OsR498G0100074200.01	0	1.503	7.710487	4.59E-24	5.72E-24	0	0	0	0	4.51	0
OsR498G0100078900.01	0	0.343	5.127223	8.48E-06	1.78E-05	0	0	0	0.24	0.06	0.73
OsR498G0100080200.01	22.616	0.253	-6.53616	1.34E-219	5.15E-220	21.18	22.89	23.78	0.5	0.09	0.17
OsR498G0100081000.01	1.03	8.246	2.989387	4.15E-134	2.16E-134	0.97	0.89	1.23	9.28	7.92	7.54
OsR498G0100081600.01	0.016	0.533	5.03976	2.36E-09	4.22E-09	0.05	0	0	0.54	0.76	0.3
OsR498G0100082800.01	0.533	9.306	4.361688	1.37E-99	8.44E-100	0.43	0.34	0.83	8.92	9.9	9.1
OsR498G0100084700.01	0.553	0.126	-2.3337	5.73E-09	1.04E-08	0.67	0.49	0.5	0.12	0.17	0.09
OsR498G0100085500.01	13.53	28.17	1.061899	1.27E-229	4.76E-230	13.24	14.26	13.09	27.62	28.87	28.02
OsR498G0100087200.01	0.136	0.046	-1.68096	0.0003678	0.0008555	0.1	0.13	0.18	0.06	0.06	0.02
OsR498G0100091500.01	2.296	54.82	4.648489	0	0	1.7	3.01	2.18	53.04	55.3	56.12
OsR498G0100091900.01	0.38	5.973	3.959947	1.57E-116	8.92E-117	0.3	0.34	0.5	5.78	6.67	5.47
OsR498G0100093200.01	0.05	3.663	6.244331	7.98E-72	5.90E-72	0.07	0.08	0	3.91	3.41	3.67
OsR498G0100094400.01	0.31	2.973	4.262353	1.64E-112	9.46E-113	0.54	0.25	0.14	2.93	2.81	3.18
OsR498G0100094500.01	1.163	198.49	7.495764	0	0	1.2	1.22	1.07	195.07	197.59	202.81
OsR498G0100094800.01	2.48	7.193	1.527951	2.46E-32	2.69E-32	2.75	2.27	2.42	7.16	7.23	7.19
OsR498G0100099600.01	0.293	0.12	-1.66105	6.89E-05	0.0001524	0.27	0.52	0.09	0.25	0.11	0
OsR498G0100102600.01	17.733	39.06	1.098429	5.31E-165	2.44E-165	17.96	18.12	17.12	40.55	39.31	37.32
OsR498G0100103600.01	31.466	7.516	-2.06917	6.84E-144	3.42E-144	32.45	32.53	29.42	8.94	6.35	7.26
OsR498G0100104900.01	0.056	0	-4.41967	0.0003895	0.0009082	0.03	0.05	0.09	0	0	0
OsR498G0100106000.01	1.17	0.13	-3.15289	7.27E-13	1.16E-12	0.99	0.98	1.54	0.11	0.17	0.11
OsR498G0100111600.01	1.996	35.486	4.139296	7.36E-287	2.34E-287	1.78	1.96	2.25	37.03	35.92	33.51
OsR498G0100119100.01	5.943	2.933	-1.02274	4.72E-17	6.80E-17	6.63	6.12	5.08	2.88	2.84	3.08
OsR498G0100119500.01	1.006	68.733	6.09554	0	0	1.1	1.04	0.88	70.61	67.09	68.5
OsR498G0100120800.01	5.25	29.176	2.460229	2.00E-171	8.97E-172	5.56	4.49	5.7	28.57	29.76	29.2
OsR498G0100122800.01	3.353	10.96	1.690263	1.40E-43	1.34E-43	3.23	2.56	4.27	10.14	8.15	14.59
OsR498G0100122900.01	125.47	270.51	1.098116	0	0	131.02	121.69	123.7	287.91	264.11	259.52
OsR498G0100124800.01	3.026	0.283	-3.41967	3.69E-22	4.77E-22	3.6	3.19	2.29	0.08	0.34	0.43
OsR498G0100125200.01	40.346	77.666	1.028693	0	0	39.66	41.62	39.76	75.18	78.24	79.58
OsR498G0100125600.01	0	0.523	5.794648	2.79E-08	5.20E-08	0	0	0	0.57	0.24	0.76
OsR498G0100126200.01	0.063	3.97	6.008542	9.48E-32	1.04E-31	0	0.19	0	4.43	3.45	4.03
OsR498G0100126800.01	1.566	6.563	1.99798	1.24E-121	6.86E-122	1.39	1.64	1.67	6.39	6.87	6.43
OsR498G0100127100.01	0.086	0.41	2.177264	0.0002386	0.000548	0.05	0.11	0.1	0.5	0.34	0.39
OsR498G0100127300.01	1.22	0.166	-2.36736	5.39E-08	1.02E-07	0.74	1.84	1.08	0.5	0	0
OsR498G0100131100.01	4.34	9.266	1.116813	3.69E-43	3.56E-43	3.92	4.18	4.92	8.91	9.7	9.19
OsR498G0100132900.01	2.26	34.3	3.985385	0	0	2.27	2.43	2.08	33.2	35.61	34.09
OsR498G0100133900.01	1.253	0.596	-1.07728	3.57E-08	6.68E-08	1.51	1.22	1.03	0.57	0.56	0.66
OsR498G0100134300.01	0.17	0.44	1.375363	4.52E-05	9.87E-05	0.07	0.3	0.14	0.51	0.48	0.33
OsR498G0100135100.01	10.573	35.28	1.723614	1.93E-209	7.68E-210	10.88	10.44	10.4	36.15	35.04	34.65
OsR498G0100135400.01	2.093	0.91	-1.18263	3.39E-08	6.34E-08	1.95	1.99	2.34	0.94	1.15	0.64
OsR498G0100136000.01	13.46	1.133	-3.56882	2.72E-236	9.96E-237	12.86	13.91	13.61	1.06	1.19	1.15
OsR498G0100138600.01	4.75	1.103	-2.11894	3.28E-23	4.16E-23	5.34	3.97	4.94	1.14	1.4	0.77
OsR498G0100141500.01	0.233	0.573	1.280768	3.78E-06	7.77E-06	0.29	0.19	0.22	0.64	0.45	0.63
OsR498G0100149000.01	71.916	187.44	1.377355	0	0	72.52	72.79	70.44	192.58	183.51	186.22
OsR498G0100151400.01	115.46	19.183	-2.5017	0	0	114.76	116.42	115.2	19.77	18.34	19.44
OsR498G0100151700.01	37.696	255.39	2.752029	0	0	36.85	38.7	37.54	253.55	256.43	256.19
OsR498G0100152200.01	23.926	52.696	1.175407	5.33E-236	1.95E-236	23.58	24.84	23.36	52.56	54.1	51.43
OsR498G0100152900.01	7.646	27.623	1.746831	0	0	6.51	8.82	7.61	28.78	26.97	27.12
OsR498G0100159600.01	4.27	1.523	-1.49516	2.17E-19	2.97E-19	4.71	3.99	4.11	1.58	1.11	1.88
OsR498G0100162800.01	15.616	1.303	-3.75868	2.21E-161	1.03E-161	17.42	15.14	14.29	1.31	1.07	1.53
OsR498G0100164500.01	8.03	2.966	-1.44567	2.02E-17	2.89E-17	8.71	7.82	7.56	3.1	2.86	2.94
OsR498G0100167300.01	1.546	0.286	-2.44137	1.09E-10	1.87E-10	1.45	1.53	1.66	0.2	0.44	0.22
OsR498G0100172400.01	1.933	0.47	-2.05139	2.62E-12	4.26E-12	1.94	2.16	1.7	0.36	0.31	0.74
OsR498G0100172600.01	0.84	0.173	-2.27432	1.22E-11	2.02E-11	1	0.96	0.56	0.24	0.18	0.1
OsR498G0100172800.01	8.14	1.923	-2.08767	1.86E-124	1.01E-124	8.14	8.2	8.08	1.89	2.08	1.8
OsR498G0100177500.01	3.94	8.656	1.219737	4.35E-22	5.64E-22	5.04	2.46	4.32	8.51	8.74	8.72
OsR498G0100178200.01	8.356	28.606	1.766156	0	0	8.55	8.67	7.85	29.49	28.26	28.07
OsR498G0100178800.01	513.11	232.75	-1.15173	0	0	520.47	504.88	514	244.64	236.11	217.5
OsR498G0100180500.01	0.953	0.426	-1.23777	6.56E-06	1.37E-05	0.88	1.07	0.91	0.49	0.22	0.57
OsR498G0100181300.01	0.493	0.18	-1.59767	0.0007574	0.0018079	0.37	0.75	0.36	0.28	0.1	0.16
OsR498G0100183500.01	2.806	8.8	1.644373	8.15E-92	5.25E-92	2.51	2.86	3.05	9.25	9.3	7.85
OsR498G0100183700.01	0.453	0.126	-1.86698	2.20E-06	4.48E-06	0.56	0.29	0.51	0.23	0.06	0.09
OsR498G0100184500.01	46.086	7.066	-2.71549	0	0	49.21	45.71	43.34	7.93	6.62	6.65
OsR498G0100185300.01	1.15	0.396	-1.5452	1.37E-11	2.28E-11	1.57	1.09	0.79	0.29	0.45	0.45
OsR498G0100193000.01	3.58	0.706	-2.23377	1.79E-57	1.49E-57	3.14	3.59	4.01	0.6	0.73	0.79

OsR498G0100194300.01	0.91	0.14	-2.66068	2.16E-09	3.87E-09	1.04	1.14	0.55	0.26	0.05	0.11
OsR498G0100194700.01	4.853	0.12	-5.64206	2.44E-73	1.78E-73	6.17	3.9	4.49	0.18	0	0.18
OsR498G0100195800.01	0.073	0.536	2.927285	6.41E-08	1.21E-07	0	0.18	0.04	0.82	0.31	0.48
OsR498G0100196800.01	1.04	7.883	2.901563	9.94E-33	1.08E-32	1.32	0.88	0.92	8.17	7.32	8.16
OsR498G0100197000.01	0.716	6.586	3.182718	1.22E-39	1.21E-39	0.45	0.75	0.95	7.92	5.1	6.74
OsR498G0100198300.01	1.586	23.116	3.847115	3.69E-152	1.79E-152	1.19	1.54	2.03	24.54	20.52	24.29
OsR498G0100203300.01	0.226	0.05	-2.20817	0.0005899	0.0013949	0.32	0.22	0.14	0.04	0	0.11
OsR498G0100203700.01	19.38	51.75	1.407804	2.02E-203	8.16E-204	20.17	18.25	19.72	51.08	54.22	49.95
OsR498G0100204500.01	19.093	74.06	1.972743	0	0	19.85	19.27	18.16	75.09	74.68	72.41
OsR498G0100209200.01	26.95	160.54	2.581885	0	0	26.14	27.49	27.22	157.85	160.66	163.11
OsR498G0100209900.01	4.27	0	-7.31779	8.28E-20	1.12E-19	3.11	4.6	5.1	0	0	0
OsR498G0100210300.01	0.903	2.79	1.60646	3.53E-14	5.46E-14	0.81	1.02	0.88	2.75	2.82	2.8
OsR498G0100211200.01	5.56	13.256	1.245026	1.12E-86	7.49E-87	5.63	4.96	6.09	12.29	14.22	13.26
OsR498G0100212300.01	0.576	0.063	-3.22327	1.04E-18	1.44E-18	0.45	0.53	0.75	0.09	0.06	0.04
OsR498G0100213400.01	7.393	3.66	-1.01517	2.41E-65	1.88E-65	7.55	7.09	7.54	3.47	3.55	3.96
OsR498G0100213800.01	0.443	15.843	5.162343	0	0	0.54	0.34	0.45	15.26	15.8	16.47
OsR498G0100215600.01	210.84	94.753	-1.17235	0	0	217.15	211.03	204.3	92.13	97.4	94.73
OsR498G0100216000.01	0.43	0.136	-1.80351	8.11E-13	1.30E-12	0.4	0.43	0.46	0.21	0.08	0.12
OsR498G0100216100.01	1.686	11.943	2.64642	6.41E-277	2.09E-277	2.26	1.47	1.33	11.29	12.59	11.95
OsR498G0100216400.01	0	1.063	7.56755	3.22E-22	4.16E-22	0	0	0	0.85	0.93	1.41
OsR498G0100220900.01	50.636	21.536	-1.24715	1.13E-200	4.63E-201	49.79	52.25	49.87	21.48	21.35	21.78
OsR498G0100221800.01	3.203	0.886	-1.86645	2.08E-36	2.16E-36	2.75	2.73	4.13	0.79	1.04	0.83
OsR498G0100222500.01	1.03	0.313	-1.66068	5.17E-07	1.02E-06	0.84	1.08	1.17	0.14	0.47	0.33
OsR498G0100223300.01	0.496	0.023	-4.28217	4.39E-06	9.05E-06	0.72	0.56	0.21	0.07	0	0
OsR498G0100224600.01	2.39	1.066	-1.17483	1.61E-13	2.52E-13	2.04	2.46	2.67	0.91	1.47	0.82
OsR498G0100225200.01	53.136	108.03	1.03083	1.46E-242	5.24E-243	49.84	57.89	51.68	108.7	105.14	110.25
OsR498G0100226300.01	3.4	7.036	1.035736	2.61E-30	2.93E-30	3.48	3.24	3.48	6.51	6.59	8.01
OsR498G0100226500.01	12.42	26.023	1.062395	2.11E-113	1.22E-113	12.25	12.16	12.85	27.16	24.74	26.17
OsR498G0100227000.01	0.36	0.043	-2.99165	4.74E-06	9.79E-06	0.54	0.22	0.32	0.04	0	0.09
OsR498G0100237500.01	0.586	0.206	-1.50621	7.05E-07	1.40E-06	0.67	0.53	0.56	0.22	0.2	0.2
OsR498G0100238400.01	2.313	0.356	-2.69105	1.06E-47	9.74E-48	1.89	2.61	2.44	0.4	0.28	0.39
OsR498G0100238600.01	0.526	1.236	1.293517	4.40E-07	8.64E-07	0.47	0.6	0.51	1.18	1.44	1.09
OsR498G0100242100.01	39.06	118.85	1.594722	0	0	40.03	38.35	38.8	120.12	119.37	117.07
OsR498G0100242900.01	19.873	6.99	-1.42817	1.76E-87	1.17E-87	18.83	20.16	20.63	7.34	7.28	6.35
OsR498G0100243100.01	7.926	3.29	-1.27283	1.42E-22	1.83E-22	9.09	7.93	6.76	4.08	3.04	2.75
OsR498G0100243500.01	0.313	1.273	2.009386	7.98E-07	1.59E-06	0.31	0.33	0.3	1.04	1.64	1.14
OsR498G0100243800.01	28.043	80.683	1.514655	9.83E-253	3.42E-253	29.02	27.36	27.75	76.97	83.68	81.4
OsR498G0100246500.01	7.666	46.776	2.606047	0	0	7.27	7.75	7.98	45.18	48.46	46.69
OsR498G0100246800.01	1.256	5.373	2.084154	4.42E-35	4.67E-35	1.31	1.15	1.31	5.01	6.32	4.79
OsR498G0100247800.01	12.503	5.72	-1.14701	1.23E-14	1.88E-14	13.29	13.05	11.17	4.69	7.16	5.31
OsR498G0100251200.01	2.496	5.75	1.196333	3.68E-14	5.70E-14	2.91	2.56	2.02	4.51	5.48	7.26
OsR498G0100252000.01	20.073	0.196	-6.69845	0	0	19.37	18.42	22.43	0.23	0.22	0.14
OsR498G0100253000.01	246.2	783.28	1.665985	0	0	249.81	242.53	246.3	759.16	791.69	799
OsR498G0100253200.01	0.376	3.49	3.188152	2.55E-49	2.30E-49	0.42	0.2	0.51	3.87	3.32	3.28
OsR498G0100253300.01	3.463	18.07	2.392512	8.40E-230	3.13E-230	3.75	3.41	3.23	17.91	19.02	17.28
OsR498G0100258100.01	0.12	0.056	-2.03063	0.0006092	0.0014421	0.07	0.11	0.18	0.06	0	0.11
OsR498G0100258600.01	0.586	5.28	3.166872	1.02E-60	8.25E-61	0.65	0.53	0.58	6.29	4.8	4.75
OsR498G0100263100.01	26.146	10.38	-1.31544	3.15E-123	1.72E-123	26.03	26.21	26.2	10.68	9.99	10.47
OsR498G0100263800.01	13.883	0.863	-4.01368	1.21E-152	5.83E-153	13.7	13.95	14	0.89	0.86	0.84
OsR498G0100264200.01	4.686	0.22	-4.42252	3.98E-112	2.31E-112	4.69	4.89	4.48	0.17	0.2	0.29
OsR498G0100266100.01	2.17	4.77	1.188152	3.00E-28	3.47E-28	2.45	2.02	2.04	4.95	4.41	4.95
OsR498G0100267100.01	2.126	6.403	1.573629	1.70E-131	8.97E-132	1.77	2.41	2.2	6.19	6.38	6.64
OsR498G0100267300.01	0.793	3.493	2.954442	3.06E-20	4.12E-20	0.62	1.37	0.39	3.11	3.56	3.81
OsR498G0100268500.01	7.313	16.543	1.235412	9.09E-244	3.25E-244	6.94	7.09	7.91	15.45	16.6	17.58
OsR498G0100269200.01	40.6	99.69	1.294832	0	0	39.74	39.55	42.51	108.96	97.37	92.74
OsR498G0100269800.01	27.433	65.896	1.25729	2.77E-206	1.11E-206	28.29	27.25	26.76	66.16	66.58	64.95
OsR498G0100270600.01	0.293	0.743	1.232405	3.90E-05	8.49E-05	0.36	0.21	0.31	0.43	0.72	1.08
OsR498G0100274000.01	1.796	3.966	1.121796	7.04E-20	9.54E-20	1.72	1.32	2.35	3.37	4.38	4.15
OsR498G0100278000.01	0.646	0.11	-2.5452	4.32E-05	9.42E-05	0.4	0.92	0.62	0.08	0.17	0.08
OsR498G0100282600.01	0.423	1.516	1.831767	1.65E-19	2.25E-19	0.31	0.42	0.54	1.48	1.36	1.71
OsR498G0100286200.01	8.51	3.363	-1.35054	1.00E-59	8.21E-60	8.08	8.82	8.63	2.99	3.79	3.31
OsR498G0100286900.01	0.016	0.47	5.084154	1.31E-09	2.33E-09	0	0.05	0	0.42	0.56	0.43
OsR498G0100287600.01	0.023	0.776	5.051491	2.92E-10	5.07E-10	0	0.07	0	0.45	1.12	0.76
OsR498G0100287700.01	1.98	30.336	3.927997	0	0	1.81	1.86	2.27	32.09	27.98	30.94
OsR498G0100287900.01	11.11	24.143	1.094486	1.47E-140	7.46E-141	10.26	11.18	11.89	24.69	23.61	24.13
OsR498G0100288300.01	3.56	0.97	-1.96024	3.26E-33	3.53E-33	2.92	3.69	4.07	0.8	1.43	0.68
OsR498G0100288900.01	0.05	0.27	2.241394	0.0001422	0.0003211	0.06	0.03	0.06	0.24	0.19	0.38
OsR498G0100289600.01	0.043	1.083	4.65447	4.45E-13	7.07E-13	0.13	0	0	1.24	0.74	1.27
OsR498G0100292200.01	27.613	55.993	1.013092	1.05E-247	3.69E-248	28.04	26.12	28.68	55.09	56.98	55.91

OsR498G0100293900.01	0.563	87.646	7.227787	0	0	0.71	0.48	0.5	91.74	87.12	84.08
OsR498G0100297400.01	0	0.83	7.127223	2.30E-17	3.29E-17	0	0	0	0.75	0.82	0.92
OsR498G0100300100.01	0.12	0.526	2.361688	1.06E-05	2.23E-05	0.14	0.13	0.09	0.47	0.63	0.48
OsR498G0100303400.01	25.56	11.756	-1.04549	4.35E-92	2.80E-92	26.06	24.88	25.74	11.03	11.41	12.83
OsR498G0100304200.01	2.146	0.923	-1.2199	5.10E-13	8.10E-13	1.33	2.67	2.44	0.79	0.88	1.1
OsR498G0100306900.01	4.503	1.456	-1.63562	2.29E-35	2.41E-35	4.75	4.35	4.41	1.44	1.29	1.64
OsR498G0100307200.01	3.566	1.55	-1.21022	2.33E-19	3.19E-19	3.39	3.68	3.63	1.77	1.37	1.51
OsR498G0100310200.01	49.913	1.053	-5.64985	0	0	50.62	51.44	47.68	1.17	1.08	0.91
OsR498G0100312900.01	59.263	261.03	2.127957	0	0	61.18	57	59.61	256.02	263.3	263.77
OsR498G0100313800.01	1.28	0.296	-2.02252	2.85E-09	5.12E-09	1.06	1.52	1.26	0.26	0.37	0.26
OsR498G0100315600.01	3.25	0.32	-3.37031	1.54E-11	2.56E-11	2.53	4.25	2.97	0	0.77	0.19
OsR498G0100326000.01	4.143	10.813	1.365533	1.37E-66	1.06E-66	3.8	3.85	4.78	10.78	11.31	10.35
OsR498G0100328800.01	3.896	27.233	2.80067	0	0	3.78	3.96	3.95	27.26	28.56	25.88
OsR498G0100331100.01	6.48	2.926	-1.15082	1.40E-15	2.10E-15	7.36	6.84	5.24	3.1	2.72	2.96
OsR498G0100335400.01	0.13	1.233	3.262152	4.64E-12	7.61E-12	0.13	0.2	0.06	1.17	1.14	1.39
OsR498G0100336900.01	6.353	2.673	-1.25775	5.16E-59	4.25E-59	6.11	6.09	6.86	2.63	2.57	2.82
OsR498G0100339300.01	0.066	16.453	7.972369	0	0	0.03	0.17	0	16.44	17.5	15.42
OsR498G0100341300.01	3.34	14.05	2.073707	1.79E-154	8.59E-155	3.21	3.42	3.39	14.74	13.75	13.66
OsR498G0100345000.01	0.56	1.403	1.249874	1.55E-16	2.26E-16	0.55	0.47	0.66	1.37	1.28	1.56
OsR498G0100347500.01	41.48	20.523	-1.03043	2.95E-83	2.01E-83	43.49	39.76	41.19	19.85	23.04	18.68
OsR498G0100348800.01	36.66	173.91	2.240964	0	0	38.22	35.16	36.6	171.21	176.81	173.72
OsR498G0100349600.01	0.6	10.183	4.100456	2.77E-59	2.28E-59	0.44	1.04	0.32	12.11	9.36	9.08
OsR498G0100352000.01	0.966	0.3	-1.68816	6.87E-15	1.04E-14	0.89	0.99	1.02	0.35	0.32	0.23
OsR498G0100352400.01	4.65	1.556	-1.61005	9.08E-23	1.16E-22	4.92	5.29	3.74	1.16	1.46	2.05
OsR498G0100352500.01	6.896	2.106	-1.72303	3.55E-46	3.32E-46	7.73	6.17	6.79	1.84	2.48	2
OsR498G0100356900.01	0.28	1.563	2.454798	4.53E-28	5.27E-28	0.4	0.17	0.27	1.7	1.54	1.45
OsR498G0100367000.01	27.933	62.693	1.161162	8.02E-229	3.00E-229	29.02	27.22	27.56	66.65	61.01	60.42
OsR498G0100368300.01	6.336	0.59	-3.43921	3.68E-43	3.55E-43	5.76	6.78	6.47	0.7	0.54	0.53
OsR498G0100369400.01	2.646	6.15	1.24676	4.48E-45	4.23E-45	2.43	2.69	2.82	6.71	5.84	5.9
OsR498G0100370600.01	8.44	20.026	1.237881	2.49E-125	1.35E-125	8.19	8.83	8.3	19.75	20.4	19.93
OsR498G0100371200.01	0.403	0.14	-1.56792	1.62E-06	3.27E-06	0.38	0.32	0.51	0.1	0.16	0.16
OsR498G0100372900.01	1.406	0.63	-1.23196	6.03E-06	1.25E-05	0.89	1.97	1.36	0.43	0.97	0.49
OsR498G0100374100.01	22.343	0.833	-4.74622	8.73E-212	3.44E-212	23.32	21.21	22.5	0.43	1.27	0.8
OsR498G0100377600.01	40.386	14.353	-1.49501	4.70E-294	1.47E-294	39.47	41.7	39.99	14.52	13.56	14.98
OsR498G0100380200.01	0.466	0.136	-1.86257	1.36E-07	2.60E-07	0.45	0.28	0.67	0.03	0.29	0.09
OsR498G0100381300.01	0.2	0.016	-3.5452	0.0005186	0.0012198	0.1	0.11	0.39	0	0	0.05
OsR498G0100384500.01	36.633	14.903	-1.32203	0	0	32.59	38.84	38.47	15.28	14.94	14.49
OsR498G0100385100.01	19.573	8.14	-1.25547	2.35E-121	1.30E-121	19.2	19.89	19.63	8.1	7.77	8.55
OsR498G0100386600.01	17.893	5.68	-1.65969	5.80E-118	3.26E-118	19.41	17.55	16.72	5.87	5.33	5.84
OsR498G0100389000.01	0.043	6.013	7.676385	4.91E-132	2.58E-132	0	0.09	0.04	6.42	5.56	6.06
OsR498G0100389600.01	4.443	15.37	1.785384	9.27E-159	4.36E-159	4.65	4.16	4.52	15.73	15.34	15.04
OsR498G0100390700.01	0.103	1.666	4.062128	8.71E-16	1.29E-15	0	0.31	0	1.59	1.71	1.7
OsR498G0100392200.01	56.133	12.423	-2.18395	0	0	55.3	55.19	57.91	11.84	11.85	13.58
OsR498G0100393600.01	10.116	5.07	-1.15237	9.92E-75	7.18E-75	9.97	10.63	9.75	4.85	6.05	4.31
OsR498G0100399100.01	0.166	1.033	2.624723	9.25E-18	1.31E-17	0.14	0.2	0.16	1.01	1.05	1.04
OsR498G0100400400.01	0.216	0.016	-3.86713	8.60E-05	0.0001913	0.21	0.29	0.15	0	0	0.05
OsR498G0100402300.01	6.21	1.13	-2.45849	6.94E-92	4.47E-92	6.73	6.19	5.71	1.19	1.18	1.02
OsR498G0100405900.01	0.086	1.436	4.065822	1.73E-85	1.16E-85	0.09	0.09	0.08	1.49	1.47	1.35
OsR498G0100407600.01	0.18	0.063	-2.28217	8.27E-05	0.0001838	0.22	0.2	0.12	0.03	0.04	0.12
OsR498G0100410000.01	13.3	34.883	1.386313	3.00E-128	1.60E-128	12.91	14.18	12.81	35.27	32.3	37.08
OsR498G0100410700.01	0.753	15.11	4.322438	1.02E-211	4.03E-212	0.85	0.73	0.68	14.69	14.92	15.72
OsR498G0100411100.01	0.35	1.383	2.113009	2.33E-13	3.67E-13	0.36	0.29	0.4	1.7	0.84	1.61
OsR498G0100411900.01	27.273	111.45	2.02759	0	0	26.66	28.15	27.01	118.31	106.28	109.77
OsR498G0100420900.01	1.023	0.106	-3.26402	7.04E-16	1.04E-15	1.21	0.72	1.14	0.04	0.16	0.12
OsR498G0100422900.01	4.576	1.693	-1.44137	1.03E-36	1.06E-36	4.49	4.65	4.59	1.68	1.8	1.6
OsR498G0100423700.01	20.886	42.22	1.0433	0	0	19.63	21.18	21.85	41.12	41.64	43.9
OsR498G0100424900.01	5.126	21.27	2.170464	1.86E-255	6.44E-256	3.93	5.81	5.64	22.31	21.59	19.91
OsR498G0100426100.01	0.196	3.99	4.327802	7.27E-123	3.99E-123	0.13	0.2	0.26	3.78	4.12	4.07
OsR498G0100439000.01	2.833	1.366	-1.05402	3.09E-13	4.88E-13	2.99	2.7	2.81	1.59	1.34	1.17
OsR498G0100439900.01	76.5	197.27	1.369805	0	0	81.3	74.71	73.49	202.12	189.89	199.8
OsR498G0100440200.01	0.47	1.51	1.536186	1.26E-07	2.42E-07	0.51	0.21	0.69	1.56	1.54	1.43
OsR498G0100443200.01	1.546	0.176	-3.21505	3.01E-20	4.05E-20	1.99	1.29	1.36	0.13	0.11	0.29
OsR498G0100443400.01	11.743	3.243	-2.10314	4.17E-237	1.52E-237	11.28	12	11.95	3.44	2.97	3.32
OsR498G0100443500.01	0.1	0.446	1.831173	4.90E-06	1.01E-05	0.07	0.12	0.11	0.44	0.55	0.35
OsR498G0100444800.01	15.923	2.403	-2.74778	3.73E-186	1.60E-186	16.87	15.13	15.77	2.46	2.57	2.18
OsR498G0100446600.01	2.77	9.283	1.774496	1.45E-79	1.01E-79	2.37	2.79	3.15	8.82	10.47	8.56
OsR498G0100447600.01	11.463	2.163	-2.38018	2.12E-303	6.44E-304	10.61	11.72	12.06	1.93	2.38	2.18
OsR498G0100448100.01	0	0.08	4.499192	0.0003163	0.0007323	0	0	0	0.04	0.07	0.13
OsR498G0100452900.01	1.543	6.78	2.129958	3.21E-32	3.52E-32	1.48	1.79	1.36	6.22	6.86	7.26

OsR498G0100453300.01	3.886	1.51	-1.37528	4.39E-10	7.66E-10	3.66	4.21	3.79	1.57	1.62	1.34
OsR498G0100453700.01	8.87	18.8	1.027832	8.38E-54	7.23E-54	7.8	8.69	10.12	18.59	19.68	18.13
OsR498G0100456800.01	1.06	27.356	4.586888	0	0	1.11	0.81	1.26	29.01	28.08	24.98
OsR498G0100463300.01	7	2.733	-1.35764	5.65E-63	4.49E-63	6.76	7.16	7.08	2.8	2.68	2.72
OsR498G0100466700.01	58.84	231.88	1.967638	0	0	62.58	55.15	58.79	231.53	234.39	229.71
OsR498G0100467900.01	17.02	54.26	1.66994	0	0	17.71	15.48	17.87	56.41	53.58	52.79
OsR498G0100468100.01	60.98	139.08	1.184021	0	0	63.04	59.01	60.89	140.55	136.98	139.72
OsR498G0100468300.01	17.71	8.543	-1.05238	8.88E-77	6.32E-77	18.1	18.11	16.92	9.51	6.91	9.21
OsR498G0100469500.01	8.056	25.26	1.643355	2.83E-178	1.25E-178	7.92	7.88	8.37	26.55	25.24	23.99
OsR498G0100471100.01	13.383	36.176	1.433475	4.31E-292	1.35E-292	13.55	13.44	13.16	38.98	35.61	33.94
OsR498G0100475200.01	4.436	0.066	-6.02633	5.54E-64	4.36E-64	4.02	4.17	5.12	0.2	0	0
OsR498G0100476300.01	13.863	2	-2.53563	4.03E-105	2.41E-105	14.37	13.41	13.81	2.41	1.85	1.74
OsR498G0100477800.01	6.32	20.04	1.639201	2.82E-171	1.27E-171	5.79	6.23	6.94	19.18	21.19	19.75
OsR498G0100478200.01	0.316	0.033	-3.28217	2.42E-05	5.21E-05	0.28	0.35	0.32	0.05	0	0.05
OsR498G0100479700.01	20.536	5.65	-1.86596	2.17E-203	8.77E-204	21.39	21.07	19.15	5.2	5.51	6.24
OsR498G0100481300.01	0.076	0.526	2.7402	8.55E-06	1.79E-05	0.12	0	0.11	0.48	0.49	0.61
OsR498G0100481700.01	10.416	3.64	-1.51001	1.24E-52	1.08E-52	10.56	12.13	8.56	2.76	4.89	3.27
OsR498G0100482300.01	6.3	0.633	-3.37152	1.39E-71	1.03E-71	6.62	6.23	6.05	0.93	0.67	0.3
OsR498G0100482700.01	0.183	0.62	1.776726	2.13E-05	4.58E-05	0.27	0.1	0.18	0.6	0.67	0.59
OsR498G0100484700.01	15.526	6.216	-1.31214	2.52E-152	1.22E-152	16.05	15.39	15.14	5.69	6.48	6.48
OsR498G0100487200.01	29.72	108.3	1.865651	0	0	30.33	29.8	29.03	112.89	102.22	109.8
OsR498G0100488600.01	2.933	1.353	-1.11691	2.77E-16	4.07E-16	3.26	3	2.54	1.48	1.31	1.27
OsR498G0100489900.01	0.6	0.196	-1.56792	1.62E-06	3.27E-06	0.63	0.61	0.56	0.26	0.18	0.15
OsR498G0100490700.01	1.18	0.446	-1.409	3.57E-08	6.67E-08	0.87	1.32	1.35	0.46	0.59	0.29
OsR498G0100502100.01	110.97	52.29	-1.08867	0	0	110.66	111.11	111.2	49.52	53.91	53.44
OsR498G0100503600.01	58.726	7.92	-3.0042	0	0	61.42	54.92	59.84	8.52	7.87	7.37
OsR498G0100505800.01	0.063	0.576	3.287688	8.37E-09	1.53E-08	0.09	0.1	0	0.49	0.37	0.87
OsR498G0100506800.01	51.756	18.26	-1.49075	4.81E-142	2.43E-142	55.5	50.88	48.89	19.16	17.21	18.41
OsR498G0100507700.01	117.66	0.883	-6.95006	0	0	113.53	119.38	120.1	0.87	0.75	1.03
OsR498G0100510600.01	321.16	74.313	-2.12113	0	0	330.96	316.23	316.3	74.19	75.17	73.58
OsR498G0100512300.01	5.143	2.08	-1.31493	3.00E-62	2.40E-62	5.05	4.71	5.67	1.84	2.32	2.08
OsR498G0100519100.01	0.363	1.196	1.704893	2.50E-11	4.18E-11	0.34	0.39	0.36	1.27	1.28	1.04
OsR498G0100522400.01	18.776	8.866	-1.15724	7.29E-48	6.69E-48	19.83	18.25	18.25	8.4	7.85	10.35
OsR498G0100524200.01	0.736	5.77	2.961248	3.06E-76	2.19E-76	0.82	0.74	0.65	5.4	5.7	6.21
OsR498G0100525200.01	13.706	38.22	1.430448	9.95E-272	3.29E-272	13.79	12.47	14.86	38.6	38.54	37.52
OsR498G0100529000.01	0.15	2.046	4.182718	5.29E-71	3.95E-71	0.22	0.14	0.09	1.87	1.91	2.36
OsR498G0100539500.01	316.07	3886.1	3.629719	0	0	311.61	324.71	311.9	4130.7	3797.7	3729.8
OsR498G0100540400.01	0.036	0.476	3.624723	0.0004041	0.0009439	0	0	0.11	0.59	0.24	0.6
OsR498G0100541900.01	6.21	101	4.020067	0	0	6.13	5.55	6.95	108.06	100.22	94.73
OsR498G0100549900.01	23.763	52.473	1.141369	2.70E-297	8.37E-298	24.28	23.31	23.7	50.93	53.21	53.28
OsR498G0100551500.01	0.85	0.296	-1.47481	0.0001919	0.0004372	0.73	1.08	0.74	0.25	0.26	0.38
OsR498G0100552800.01	0.69	0.113	-2.84777	1.37E-07	2.63E-07	0.76	0.45	0.86	0.08	0.17	0.09
OsR498G0100559700.01	129.25	309.19	1.255548	0	0	130.98	129.72	127	309.28	304.75	313.54
OsR498G0100560700.01	18.88	2.993	-2.66265	2.13E-161	9.89E-162	19.61	17.28	19.75	3.7	2.73	2.55
OsR498G0100564900.01	1.826	33.63	4.20902	0	0	1.62	2.01	1.85	34.79	33.56	32.54
OsR498G0100567600.01	52.973	110.18	1.040923	1.10E-150	5.33E-151	54.26	51.7	52.96	111.37	109.16	110.02
OsR498G0100571700.01	65.83	18.953	-1.80036	0	0	69.47	64.64	63.38	18.14	19.09	19.63
OsR498G0100573900.01	0.256	5.853	4.472719	1.86E-50	1.66E-50	0.16	0.16	0.45	5.76	5.68	6.12
OsR498G0100576000.01	153.2	380.44	1.317489	0	0	159.01	158.47	142.1	411.48	375.75	354.09
OsR498G0100578400.01	1.346	0.233	-2.64394	6.38E-39	6.43E-39	1.33	1.24	1.47	0.23	0.28	0.19
OsR498G0100578800.01	0.703	0.15	-2.22327	0.0001374	0.00031	1.23	0.37	0.51	0.18	0	0.27
OsR498G0100583600.01	0.013	0.126	3.209685	5.30E-05	0.0001164	0.02	0	0.02	0.16	0.08	0.14
OsR498G0100585300.01	7.646	2.84	-1.43179	3.40E-19	4.68E-19	7.9	7.91	7.13	2.88	2.7	2.94
OsR498G0100590400.01	0.06	0.776	3.81235	9.79E-19	1.36E-18	0.03	0.12	0.03	0.98	0.78	0.57
OsR498G0100591100.01	0.11	0.816	3.016124	7.49E-10	1.32E-09	0.06	0.13	0.14	0.79	0.64	1.02
OsR498G0100591500.01	0.91	0.31	-1.5554	7.14E-33	7.75E-33	0.75	0.98	1	0.3	0.33	0.3
OsR498G0100592700.01	40.406	11.56	-1.85858	7.48E-167	3.41E-167	42.75	38.1	40.37	12.5	10.56	11.62
OsR498G0100594800.01	20.723	145.09	2.80165	0	0	22.56	18.81	20.8	147.08	143.28	144.9
OsR498G0100602700.01	13.903	0.793	-4.14315	4.90E-166	2.24E-166	14.15	13.84	13.72	0.65	1.12	0.61
OsR498G0100603300.01	0.446	0.07	-2.73283	5.97E-08	1.13E-07	0.4	0.5	0.44	0.13	0.08	0
OsR498G0100607700.01	0.726	0.376	-1.09215	3.89E-06	8.00E-06	0.81	0.73	0.64	0.37	0.3	0.46
OsR498G0100609500.01	43.57	103.27	1.235079	6.58E-213	2.58E-213	44.07	43.35	43.29	103.13	105.18	101.51
OsR498G0100612000.01	0.28	0.05	-2.5452	4.32E-05	9.42E-05	0.35	0.22	0.27	0.04	0.04	0.07
OsR498G0100613400.01	7.403	2.853	-1.30525	1.19E-54	1.02E-54	6.82	7.4	7.99	2.73	2.83	3
OsR498G0100616100.01	0.536	0.173	-1.69721	4.52E-05	9.87E-05	0.51	0.6	0.5	0.17	0.21	0.14
OsR498G0100616300.01	0.193	0.03	-2.8536	1.27E-07	2.42E-07	0.1	0.25	0.23	0.04	0	0.05
OsR498G0100622100.01	68.94	165.22	1.254791	0	0	69.13	69.25	68.44	171.39	158.75	165.52
OsR498G0100622500.01	0.106	3.33	4.939764	1.21E-50	1.08E-50	0.09	0.1	0.13	3.9	2.63	3.46
OsR498G0100623200.01	0.936	2.923	1.630436	3.84E-22	4.96E-22	1.17	0.7	0.94	2.65	3.55	2.57

OsR498G0100624700.01	0.023	0.24	3.287688	2.92E-05	6.31E-05	0	0	0.07	0.3	0.27	0.15
OsR498G0100625600.01	115.41	336.74	1.540633	0	0	116.65	115.95	113.6	332.9	340.88	336.43
OsR498G0100627900.01	0.153	20.326	7.004745	0	0	0.09	0.16	0.21	20.03	20.95	20
OsR498G0100628700.01	21.573	57.31	1.413204	1.34E-303	4.08E-304	22.54	21.27	20.91	57.44	57.49	57
OsR498G0100628900.01	6.4	1.113	-2.53767	1.76E-54	1.51E-54	6.58	6.31	6.31	1.26	0.88	1.2
OsR498G0100632000.01	35.893	84.973	1.236353	2.76E-277	8.99E-278	34.85	36.21	36.62	86.31	84.87	83.74
OsR498G0100638600.01	1.976	11.053	2.467398	6.51E-128	3.49E-128	2.16	1.42	2.35	11.59	11.09	10.48
OsR498G0100639700.01	2.93	0.18	-4.06178	2.53E-23	3.19E-23	3.36	2.54	2.89	0.18	0.28	0.08
OsR498G0100640000.01	1.03	0.433	-1.246	4.06E-07	7.96E-07	0.75	1.44	0.9	0.39	0.57	0.34
OsR498G0100642500.01	0.883	3.4	1.928503	7.93E-26	9.58E-26	0.87	0.7	1.08	3.42	3.06	3.72
OsR498G0100653900.01	35.773	75.343	1.068869	4.24E-265	1.43E-265	35.39	36.24	35.69	73.6	76.73	75.7
OsR498G0100655000.01	1.563	4.35	1.464851	3.63E-28	4.22E-28	2.2	1.1	1.39	3.69	5.27	4.09
OsR498G0100655200.01	8.043	1.726	-2.23708	1.00E-61	8.06E-62	8.6	7.16	8.37	1.34	2.11	1.73
OsR498G0100655300.01	0.506	0.04	-3.73283	6.30E-10	1.11E-09	0.52	0.54	0.46	0.04	0.04	0.04
OsR498G0100656400.01	59.736	15.206	-1.98467	2.41E-220	9.27E-221	60.71	60.18	58.32	16.39	14.98	14.25
OsR498G0100658300.01	1.306	0.3	-2.1032	2.23E-19	3.05E-19	1.33	1.31	1.28	0.47	0.23	0.2
OsR498G0100658600.01	7.196	16.11	1.162908	1.11E-159	5.18E-160	7.18	7.35	7.06	15.77	16.15	16.41
OsR498G0100660500.01	3.133	7.606	1.273479	9.79E-21	1.30E-20	2.9	2.97	3.53	8.05	7.59	7.18
OsR498G0100660800.01	0.966	7.216	2.892656	1.92E-59	1.57E-59	1.06	0.93	0.91	7.82	7.32	6.51
OsR498G0100663200.01	1.816	0.31	-2.57691	6.39E-06	1.33E-05	2	2.81	0.64	0.34	0	0.59
OsR498G0100668500.01	883.68	180.29	-2.2998	0	0	899.17	874.18	877.7	186.78	175.05	179.05
OsR498G0100668900.01	82.436	280.02	1.754958	0	0	85.26	81.03	81.02	284.42	267.39	288.26
OsR498G0100669200.01	28.32	9.853	-1.48317	1.73E-174	7.69E-175	27.36	28.83	28.77	10.21	9.41	9.94
OsR498G0100670400.01	34.15	16.786	-1.03295	5.67E-77	4.03E-77	35.89	32.93	33.63	15.36	18.94	16.06
OsR498G0100670500.01	1.156	0.383	-1.60078	2.23E-08	4.15E-08	1.03	1.16	1.28	0.46	0.43	0.26
OsR498G0100672300.01	2.193	0.71	-1.62479	1.73E-23	2.19E-23	2.26	2.23	2.09	0.79	0.72	0.62
OsR498G0100678600.01	0.5	0.253	-1.08053	0.0008528	0.0020409	0.41	0.67	0.42	0.2	0.25	0.31
OsR498G0100679800.01	0.683	0.276	-1.32637	1.77E-08	3.28E-08	0.93	0.57	0.55	0.27	0.3	0.26
OsR498G0100681000.01	8.756	26.82	1.612111	4.99E-181	2.18E-181	9.07	8.67	8.53	27.28	27.53	25.65
OsR498G0100688400.01	1.193	21.753	4.184246	0	0	1.12	1.38	1.08	21.94	20.76	22.56
OsR498G0100694000.01	40.113	94.49	1.233541	0	0	39.62	41.59	39.13	94.81	94.7	93.96
OsR498G0100701100.01	0.52	2.03	1.960326	8.99E-24	1.13E-23	0.78	0.38	0.4	1.97	1.87	2.25
OsR498G0100702400.01	0.4	2.223	2.467622	2.19E-17	3.13E-17	0.34	0.48	0.38	2.38	1.98	2.31
OsR498G0100708900.01	1.08	26.22	4.595559	0	0	0.94	1.02	1.28	25.98	25.39	27.29
OsR498G0100709200.01	1.416	30.873	4.436654	0	0	1.31	1.48	1.46	35.33	29.13	28.16
OsR498G0100711500.01	0.56	1.406	1.328005	2.47E-09	4.41E-09	0.64	0.54	0.5	1.24	1.48	1.5
OsR498G0100715000.01	0.663	0.156	-1.93223	7.75E-07	1.54E-06	0.48	0.84	0.67	0.08	0.25	0.14
OsR498G0100721100.01	8.193	3.826	-1.15987	3.18E-25	3.88E-25	8.25	8.24	8.09	4.1	4	3.38
OsR498G0100721700.01	21.556	67.196	1.623801	0	0	21.18	21.86	21.63	66.16	66.82	68.61
OsR498G0100723300.01	2.68	1.406	-1.02217	2.26E-15	3.40E-15	2.92	2.98	2.14	1.38	1.65	1.19
OsR498G0100727600.01	17.28	91.176	2.443882	0	0	16.6	18.59	16.65	88.45	92.11	92.97
OsR498G0100728400.01	21.136	8.266	-1.44859	1.30E-124	7.06E-125	21.76	20.85	20.8	7.77	8.71	8.32
OsR498G0100728900.01	7.236	17.12	1.23649	1.72E-68	1.31E-68	8.09	6.79	6.83	18.17	17.02	16.17
OsR498G0100731500.01	0.09	1.193	3.7402	1.30E-25	1.57E-25	0.09	0.12	0.06	1.26	1.37	0.95
OsR498G0100734400.01	0.263	4.55	4.098654	6.94E-45	6.57E-45	0.32	0.2	0.27	5.5	4.26	3.89
OsR498G0100734500.01	0.036	0.453	3.624723	1.45E-06	2.92E-06	0.05	0.06	0	0.56	0.29	0.51
OsR498G0100736700.01	0.166	0.52	1.624723	7.41E-06	1.55E-05	0.09	0.15	0.26	0.48	0.56	0.52
OsR498G0100743700.01	0.146	0.36	1.319868	9.63E-05	0.0002148	0.14	0.22	0.08	0.33	0.28	0.47
OsR498G0100744000.01	3.09	0.983	-1.59496	1.35E-28	1.56E-28	3.41	2.75	3.11	0.86	0.86	1.23
OsR498G0100745200.01	0.083	0.31	1.900213	0.0002357	0.0005411	0	0.11	0.14	0.39	0.22	0.32
OsR498G0100745600.01	1.276	0.36	-1.9499	9.67E-16	1.44E-15	1.23	1.3	1.3	0.28	0.4	0.4
OsR498G0100746600.01	23.596	10.486	-1.17984	3.70E-51	3.28E-51	24.08	22.72	23.99	12.07	10.42	8.97
OsR498G0100748200.01	1.026	0.16	-2.68271	1.22E-06	2.44E-06	0.84	0.98	1.26	0.38	0.1	0
OsR498G0100750800.01	0.06	1.936	5.009386	6.51E-24	8.15E-24	0.06	0	0.12	1.81	1.7	2.3
OsR498G0100751600.01	0.573	9.64	4.042195	3.36E-128	1.80E-128	0.6	0.63	0.49	8.51	10.2	10.21
OsR498G0100752500.01	0.663	0.19	-1.87778	5.39E-05	0.0001184	0.58	0.73	0.68	0.18	0.13	0.26
OsR498G0100753700.01	0.333	4.323	3.7402	1.31E-125	7.08E-126	0.21	0.49	0.3	3.31	4.8	4.86
OsR498G0100754500.01	0	6.026	8.754006	6.46E-43	6.25E-43	0	0	0	5.51	6.44	6.13
OsR498G0100756500.01	27	125.57	2.242979	0	0	26.1	27.73	27.17	129.24	124.98	122.49
OsR498G0100762200.01	62.33	12.126	-2.33229	0	0	63.27	60.54	63.18	11.39	12.25	12.74
OsR498G0100765000.01	0.31	0.036	-3.13016	5.66E-08	1.07E-07	0.28	0.43	0.22	0.03	0.05	0.03
OsR498G0100768200.01	2.233	232.18	6.691621	0	0	2.21	2.21	2.28	243.64	223.02	229.89
OsR498G0100771800.01	0.053	0.396	2.914229	2.18E-05	4.67E-05	0.05	0.06	0.05	0.48	0.33	0.38
OsR498G0100775700.01	47.903	8.253	-2.53917	0	0	45.5	48.45	49.76	9.07	8.14	7.55
OsR498G0100776800.01	5.953	46.07	2.964332	0	0	5.9	6.23	5.73	44.08	46.2	47.93
OsR498G0100777200.01	0.556	0.21	-1.43422	0.0007507	0.0017911	0.36	0.42	0.89	0.24	0.39	0
OsR498G0100778500.01	1.643	9.703	2.556336	5.46E-46	5.11E-46	1.72	1.9	1.31	9.38	10.66	9.07
OsR498G0100778900.01	0.823	2.42	1.539331	7.25E-08	1.37E-07	1.1	0.63	0.74	2.27	2.77	2.22
OsR498G0100780000.01	0	0.11	4.624723	0.0001711	0.0003885	0	0	0	0.24	0.06	0.03

OsR498G0100785100.01	0.24	1.69	2.809147	1.02E-13	1.59E-13	0.13	0.34	0.25	0.86	2.05	2.16
OsR498G0100786600.01	3.43	0.973	-1.6499	9.05E-10	1.60E-09	3.3	4.22	2.77	1.02	0.75	1.15
OsR498G0100786800.01	103.72	43.66	-1.25831	1.13E-295	3.50E-296	106.17	103.35	101.7	41.91	46.91	42.16
OsR498G0100788100.01	2.836	1.043	-1.46613	1.53E-06	3.09E-06	2.52	2.4	3.59	1.24	1.01	0.88
OsR498G0100791600.01	20.73	52.493	1.337195	0	0	21.86	19.53	20.8	51.99	52.29	53.2
OsR498G0100797300.01	5.56	0.42	-3.73107	1.29E-55	1.10E-55	6.44	5.42	4.82	0.37	0.38	0.51
OsR498G0100800300.01	7.046	24.153	1.759216	8.68E-262	2.95E-262	7.24	6.84	7.06	22.73	25.7	24.03
OsR498G0100800900.01	0.013	0.166	4.03976	3.45E-05	7.49E-05	0	0.04	0	0.15	0.19	0.16
OsR498G0100818800.01	1.103	0	-7.67449	3.42E-24	4.25E-24	0.91	1.28	1.12	0	0	0
OsR498G0100819700.01	21.913	0.98	-4.44235	0	0	22.14	20.94	22.66	1.16	0.82	0.96
OsR498G0100833100.01	0.24	3.163	3.732782	3.68E-41	3.62E-41	0.19	0.3	0.23	3.46	3.18	2.85
OsR498G0100834400.01	0.59	0.11	-2.41967	5.12E-09	9.27E-09	0.68	0.44	0.65	0.13	0.07	0.13
OsR498G0100834600.01	7.223	3.056	-1.24832	3.95E-25	4.83E-25	7.46	7.13	7.08	2.95	2.85	3.37
OsR498G0100834800.01	5.613	1.933	-1.64967	1.34E-68	1.02E-68	5.99	5.91	4.94	2.04	1.52	2.24
OsR498G0100836600.01	0.11	0.563	2.337441	1.92E-09	3.42E-09	0.14	0.03	0.16	0.62	0.47	0.6
OsR498G0100840600.01	17.683	62.62	1.828211	0	0	17.32	18.4	17.33	61.93	61.42	64.51
OsR498G0100841100.01	3.693	8.36	1.208118	4.86E-72	3.59E-72	4.22	3.21	3.65	7.77	8.4	8.91
OsR498G0100845700.01	2.256	0.54	-2.06777	2.26E-21	2.97E-21	2.36	1.77	2.64	0.72	0.47	0.43
OsR498G0100850400.01	281.13	4.056	-6.11632	0	0	282.32	282.75	278.3	4.08	3.95	4.14
OsR498G0100850700.01	13.193	2.146	-2.63645	0	0	13.41	12.9	13.27	2.03	2.13	2.28
OsR498G0100859000.01	2.603	0.796	-1.71767	8.82E-14	1.38E-13	2.19	2.76	2.86	0.73	0.92	0.74
OsR498G0100861300.01	0.363	1.03	1.484545	3.97E-12	6.49E-12	0.39	0.23	0.47	0.74	1.19	1.16
OsR498G0100863300.01	26.846	60.476	1.166413	1.16E-299	3.57E-300	26	27.57	26.97	60.68	60.71	60.04
OsR498G0100866200.01	3.84	0.72	-2.41194	2.74E-62	2.19E-62	3.65	4.01	3.86	0.68	0.78	0.7
OsR498G0100874900.01	0.426	0	-5.66068	7.26E-08	1.38E-07	0.39	0.41	0.48	0	0	0
OsR498G0100879300.01	5.84	1.69	-1.72923	2.76E-44	2.63E-44	6.48	5.88	5.16	1.21	2.48	1.38
OsR498G0100880900.01	0.363	0.716	1.333943	6.04E-08	1.14E-07	0.38	0.46	0.25	0.63	0.54	0.98
OsR498G0100885800.01	3.1	0.906	-1.87383	3.36E-17	4.83E-17	2.77	3.2	3.33	0.76	1.15	0.81
OsR498G0100886700.01	1.296	2.676	1.03976	4.61E-08	8.67E-08	1.34	1.4	1.15	2.32	2.87	2.84
OsR498G0100887900.01	2.533	1.156	-1.21251	4.07E-39	4.09E-39	2.02	2.78	2.8	1.45	1.1	0.92
OsR498G0100893500.01	18.736	7.4	-1.11612	1.08E-68	8.19E-69	16.13	21	19.08	7.65	6.76	7.79
OsR498G0100897100.01	0.32	0.033	-2.76759	4.11E-07	8.06E-07	0.19	0.43	0.34	0.06	0.02	0.02
OsR498G0100897400.01	6.663	19.103	1.492619	3.71E-171	1.67E-171	6.96	6.78	6.25	18.62	19.23	19.46
OsR498G0100902200.01	0.156	1.033	2.965759	3.62E-08	6.78E-08	0.21	0.06	0.2	1.28	0.39	1.43
OsR498G0100908700.01	1.133	0.536	-1.12463	1.46E-07	2.80E-07	1.29	0.85	1.26	0.51	0.46	0.64
OsR498G0100909100.01	0.43	0.213	-1.13712	0.0004468	0.0010466	0.32	0.59	0.38	0.22	0.27	0.15
OsR498G0100913700.01	7.446	0.196	-5.22166	2.85E-125	1.55E-125	7.7	7.64	7	0.29	0.17	0.13
OsR498G0100914900.01	0	0.316	4.499192	0.0003163	0.0007323	0	0	0	0.34	0.35	0.26
OsR498G0100916600.01	6.056	2.186	-1.51782	1.28E-44	1.22E-44	6.01	6.04	6.12	2.25	2.46	1.85
OsR498G0100918400.01	9.24	18.513	1.003806	3.30E-130	1.75E-130	8.97	9.67	9.08	18.32	18.26	18.96
OsR498G0100930500.01	276.98	4.07	-6.08876	0	0	267.1	284.21	279.6	4.72	3.91	3.58
OsR498G0100933100.01	25.84	8.49	-1.60404	0	0	24.42	26.85	26.25	7.42	9.31	8.74
OsR498G0100933400.01	6.256	18.57	1.559413	1.01E-53	8.71E-54	6.59	6.19	5.99	20.02	17.8	17.89
OsR498G0100940200.01	1.47	0.323	-2.33478	2.77E-47	2.55E-47	1.32	1.74	1.35	0.32	0.41	0.24
OsR498G0100943000.01	31.186	4.866	-2.66747	0	0	31.59	30.48	31.49	4.53	5	5.07
OsR498G0100944800.01	28.046	13.45	-1.03867	1.64E-60	1.33E-60	28.6	29.33	26.21	13.9	12.71	13.74
OsR498G0100946200.01	0.276	0.44	1.10389	8.36E-05	0.0001858	0.26	0.36	0.21	0.45	0.37	0.5
OsR498G0100948400.01	74.713	176.47	1.225658	0	0	73.08	74.28	76.78	169.22	177.6	182.58
OsR498G0100952300.01	12.983	5.97	-1.1019	1.41E-31	1.56E-31	12.91	13.69	12.35	6.01	5.62	6.28
OsR498G0100961200.01	9.546	3.916	-1.41374	1.92E-55	1.63E-55	9.11	9.41	10.12	3.96	3.89	3.9
OsR498G0100962700.01	26.05	53.856	1.051795	2.09E-251	7.31E-252	25.21	26.14	26.8	52.45	55.26	53.86
OsR498G0100964000.01	31.12	95.723	1.635476	0	0	30.4	32.06	30.9	91.12	95.61	100.44
OsR498G0100964300.01	0.593	0.14	-2.08577	4.85E-07	9.55E-07	0.85	0.39	0.54	0.15	0.12	0.15
OsR498G0100966100.01	2.463	0.43	-2.52318	1.11E-19	1.51E-19	2.94	2.32	2.13	0.64	0.24	0.41
OsR498G0100966900.01	101.8	5.17	-4.3105	0	0	104.53	104.96	95.92	4.78	5.69	5.04
OsR498G0100969300.01	0.64	0	-6.08952	5.87E-10	1.03E-09	0.6	0.58	0.74	0	0	0
OsR498G0100969800.01	25.026	177.12	2.81639	0	0	25.26	26.07	23.75	186.23	176.93	168.2
OsR498G0100971200.01	0.093	1.066	3.482704	1.31E-18	1.82E-18	0.07	0.07	0.14	1.12	0.9	1.18
OsR498G0100974400.01	130.23	304.43	1.206187	0	0	124.85	131.39	134.5	309.22	302.55	301.51
OsR498G0100980000.01	0	7.823	8.676385	4.01E-41	3.95E-41	0	0	0	6.52	9.03	7.92
OsR498G0100980700.01	0.023	0.48	4.361688	3.01E-06	6.15E-06	0	0.07	0	0.64	0.44	0.36
OsR498G0100981200.01	0.14	0.37	1.389344	8.35E-07	1.66E-06	0.11	0.18	0.13	0.4	0.36	0.35
OsR498G0100987100.01	5.39	3.186	-1.02081	3.45E-13	5.47E-13	6.07	4.85	5.25	2.88	3.29	3.39
OsR498G0100992500.01	0.26	0.02	-3.66068	0.0002846	0.0006567	0.36	0.19	0.23	0	0.06	0
OsR498G0100996100.01	1.346	0.346	-1.96024	1.25E-06	2.51E-06	1.67	1.32	1.05	0.52	0.26	0.26
OsR498G0100996400.01	0	0.953	6.44983	6.88E-12	1.13E-11	0	0	0	0.69	1.1	1.07
OsR498G0100997400.01	1.06	0.18	-2.5452	8.68E-07	1.73E-06	1.06	0.93	1.19	0.36	0.09	0.09
OsR498G0101000100.01	23.02	4.97	-2.27553	9.84E-186	4.23E-186	24.8	22.45	21.81	4.23	5.13	5.55
OsR498G0101004100.01	94.856	215.73	1.181773	0	0	91.96	98.6	94.01	207.6	219.74	219.84

OsR498G0101007700.01	5.82	89.996	3.945433	0	0	5.92	6.53	5.01	88.91	86.88	94.2
OsR498G0101010400.01	0.03	0.31	3.454798	6.11E-08	1.15E-07	0.03	0.06	0	0.4	0.27	0.26
OsR498G0101015100.01	107.29	47.526	-1.21049	0	0	111.2	102.68	108	48.16	46.59	47.83
OsR498G0101021000.01	0.31	0	-5.66068	7.26E-08	1.38E-07	0.32	0.34	0.27	0	0	0
OsR498G0101023400.01	1.303	0.156	-3.06876	1.96E-13	3.08E-13	1.18	1.59	1.14	0.23	0.12	0.12
OsR498G0101025500.01	0.236	22.37	6.549992	1.10E-279	3.55E-280	0.16	0.34	0.21	23.16	23.09	20.86
OsR498G0101026700.01	1.223	234.53	7.584823	0	0	0.74	1.74	1.19	246.58	230.91	226.09
OsR498G0101028000.01	4.136	0.233	-4.20931	6.31E-10	1.11E-09	4.9	2.79	4.72	0	0.35	0.35
OsR498G0101029700.01	19.253	46.643	1.305733	1.15E-300	3.53E-301	18.4	19.94	19.42	43.63	50.67	45.63
OsR498G0101034000.01	3.673	1.23	-1.40365	4.66E-20	6.29E-20	3.33	3.75	3.94	0.95	1.86	0.88
OsR498G0101038400.01	0.08	0.746	3.229585	4.73E-15	7.15E-15	0.09	0.06	0.09	0.66	0.75	0.83
OsR498G0101042900.01	23.416	8.833	-1.43919	4.57E-91	2.96E-91	23.27	22.08	24.9	9.78	8.25	8.47
OsR498G0101046700.01	32.796	13.68	-1.2479	3.20E-218	1.24E-218	32.64	33.3	32.45	13.54	13.99	13.51
OsR498G0101052000.01	0.05	15.5	8.382279	0	0	0.03	0.09	0.03	16.36	15.12	15.02
OsR498G0101057900.01	2.4	0.36	-2.91866	3.41E-29	3.90E-29	2.17	2.83	2.2	0.47	0.23	0.38
OsR498G0101060600.01	106.13	22.326	-2.25387	0	0	110.72	103.99	103.7	22.4	22.63	21.95
OsR498G0101060700.01	2046.2	414.87	-2.30213	0	0	2057.2	2039.3	2042	433.02	409.13	402.45
OsR498G0101065300.01	0.573	1.356	1.234505	0.0003571	0.0008298	0.6	0.54	0.58	1.23	1.07	1.77
OsR498G0101068400.01	15.28	1.696	-3.47814	0	0	15.3	15.6	14.94	1.78	1.4	1.91
OsR498G0101070200.01	11.686	0.723	-4.03028	4.28E-172	1.92E-172	11.4	10.84	12.82	0.87	0.72	0.58
OsR498G0101080700.01	7.866	17.556	1.152183	2.57E-72	1.89E-72	7.72	8.09	7.79	16.38	19.29	17
OsR498G0101084100.01	127.44	27.266	-2.22046	0	0	129.22	125.9	127.2	27.44	27.42	26.94
OsR498G0101085300.01	0.566	0.053	-3.84288	9.93E-14	1.55E-13	0.81	0.2	0.69	0.06	0	0.1
OsR498G0101085900.01	0.576	14.613	4.644622	3.21E-85	2.16E-85	1.03	0.48	0.22	15.44	15.75	12.65
OsR498G0101089100.01	2.156	0.3	-2.86713	1.23E-13	1.92E-13	2.58	1.9	1.99	0.18	0.27	0.45
OsR498G0101096000.01	0.46	6.42	3.765585	6.35E-37	6.55E-37	0.21	0.34	0.83	6.19	6.89	6.18
OsR498G0101099600.01	0.103	0.433	2.084154	1.79E-05	3.83E-05	0.04	0.16	0.11	0.58	0.52	0.2
OsR498G0101102400.01	2.166	36.66	4.07381	0	0	1.84	2.48	2.18	38.43	36.43	35.12
OsR498G0101103600.01	8.063	3.613	-1.16428	2.47E-38	2.51E-38	8.2	8	7.99	3.55	3.68	3.61
OsR498G0101108400.01	6.666	15.603	1.212769	1.01E-79	7.06E-80	7.4	6.19	6.41	14.29	15.28	17.24
OsR498G0101115500.01	8.3	2.543	-1.89504	3.18E-153	1.53E-153	7.9	8.4	8.6	2.33	2.77	2.53
OsR498G0101117500.01	0.27	1.01	1.903698	6.84E-11	1.16E-10	0.26	0.27	0.28	1.01	1.18	0.84
OsR498G0101132500.01	2.636	0.616	-2.10067	1.01E-32	1.10E-32	2.31	2.84	2.76	0.65	0.74	0.46
OsR498G0101140500.01	0.006	0.126	4.03976	3.45E-05	7.49E-05	0.02	0	0	0.14	0.12	0.12
OsR498G0101141800.01	5.24	0.846	-2.67353	3.10E-11	5.21E-11	5.46	4.84	5.42	0.74	1.03	0.77
OsR498G0101142900.01	14.9	2.406	-2.63779	5.20E-157	2.46E-157	15.84	13.88	14.98	2.6	2	2.62
OsR498G0101143700.01	14.323	3.106	-2.25366	0	0	14.67	14.77	13.53	3.06	3.17	3.09
OsR498G0101144200.01	0.09	0.333	1.85649	5.09E-06	1.05E-05	0.09	0.09	0.09	0.31	0.28	0.41
OsR498G0101147900.01	6.373	0.373	-4.10999	1.22E-31	1.35E-31	7.5	5.8	5.82	0.56	0.28	0.28
OsR498G0101155200.01	1.466	0.136	-3.45541	1.31E-62	1.05E-62	1.7	1.4	1.3	0.14	0.14	0.13
OsR498G0101164000.01	0.88	4.5	2.350962	5.54E-54	4.78E-54	0.94	0.82	0.88	4.38	4.57	4.55
OsR498G0101164800.01	0.306	0.9	1.534525	0.0005222	0.0012286	0.33	0.35	0.24	1.12	0.79	0.79
OsR498G0101166100.01	31.586	13.303	-1.19782	4.79E-95	3.03E-95	32.82	31.32	30.62	13.37	14.13	12.41
OsR498G0101169200.01	14.573	2.85	-2.36303	8.00E-116	4.55E-116	15.46	13.93	14.33	2.72	2.6	3.23
OsR498G0101172800.01	0.43	1.076	1.354962	2.05E-21	2.69E-21	0.41	0.5	0.38	1.17	1.06	1
OsR498G0101178700.01	4.586	17.183	1.90533	0	0	4.41	4.38	4.97	16.46	18.11	16.98
OsR498G0101179100.01	14.916	47.8	1.678805	0	0	14.7	15.77	14.28	47.68	47.37	48.35
OsR498G0101179400.01	5.973	1.053	-2.51295	2.45E-158	1.16E-158	5.98	6.23	5.71	1	1.09	1.07
OsR498G0101188200.01	0.45	8.25	4.185023	1.11E-228	4.17E-229	0.32	0.5	0.53	7.66	8.5	8.59
OsR498G0101225100.01	0.423	2.343	2.464782	9.36E-22	1.22E-21	0.33	0.3	0.64	2.69	2.13	2.21
OsR498G0101225400.01	27.29	106.35	1.910755	0	0	27.05	27.02	27.8	103.54	110.59	104.92
OsR498G0101226100.01	3.813	1.153	-1.77171	1.28E-32	1.39E-32	4.21	4.04	3.19	1.38	1.17	0.91
OsR498G0101240900.01	0.18	1.83	3.664251	1.14E-16	1.66E-16	0.23	0.07	0.24	3.52	0.85	1.12
OsR498G0101243900.01	0.01	0.193	4.361688	3.01E-06	6.15E-06	0	0	0.03	0.3	0.09	0.19
OsR498G0101246700.01	0.04	0.343	3.182718	2.66E-11	4.47E-11	0.07	0.05	0	0.41	0.45	0.17
OsR498G0101257300.01	0.83	0.243	-1.75379	7.77E-11	1.32E-10	1	0.85	0.64	0.31	0.25	0.17
OsR498G0101271400.01	0.44	5.086	3.496138	8.68E-93	5.56E-93	0.58	0.39	0.35	5.4	5.15	4.71
OsR498G0101278700.01	44.983	89.073	1.121083	0	0	46.48	43.71	44.76	88.02	90.09	89.11
OsR498G0101280600.01	226.05	929.61	1.974317	0	0	207.31	239.85	231	997.76	897.24	893.83
OsR498G0101280700.01	0.293	9.743	5.106451	0	0	0	0.88	0	10.64	10.33	8.26
OsR498G0101298600.01	0.046	0.536	3.499192	4.83E-06	9.99E-06	0.07	0	0.07	0.43	0.52	0.66
OsR498G0101311800.01	3.043	0.376	-2.89313	2.56E-68	1.95E-68	2.31	2.3	4.52	0.34	0.36	0.43
OsR498G0101312200.01	0.633	0.163	-1.96024	5.52E-05	0.0001214	0.59	0.62	0.69	0.18	0.19	0.12
OsR498G0101327900.01	0.493	0.203	-1.28032	9.67E-08	1.84E-07	0.57	0.35	0.56	0.22	0.13	0.26
OsR498G0101330600.01	1.246	51.383	5.361133	0	0	1.2	1.38	1.16	52.78	52.8	48.57
OsR498G0101330900.01	11.016	25.276	1.196384	2.02E-177	8.92E-178	11.09	11.18	10.78	25.16	26.1	24.57
OsR498G0101333000.01	101.88	350.64	1.774387	0	0	106.32	99.08	100.2	356.36	349.84	345.73
OsR498G0101333500.01	0.083	6.99	6.391435	9.95E-60	8.14E-60	0	0.17	0.08	7.93	7.11	5.93
OsR498G0101333800.01	32.073	12.946	-1.31677	3.00E-146	1.49E-146	34.07	31.8	30.35	12.65	13.34	12.85

OsR498G0101337500.01	196.04	413.77	1.07914	0	0	192.84	199.84	195.5	422.85	407.75	410.72
OsR498G0101339400.01	37.84	11.083	-1.78234	2.64E-97	1.65E-97	36.24	41.36	35.92	11.81	9	12.44
OsR498G0101343800.01	0.806	0.066	-3.5452	3.01E-13	4.76E-13	0.97	0.63	0.82	0.08	0.08	0.04
OsR498G0101346000.01	4.573	1.043	-2.66426	1.47E-60	1.19E-60	4.37	5.22	4.13	0.85	1.07	1.21
OsR498G0101357600.01	0.316	0	-6.20817	1.23E-10	2.11E-10	0.35	0.29	0.31	0	0	0
OsR498G0101359400.01	8.41	48.28	2.514019	1.72E-275	5.64E-276	9.33	8.68	7.22	46.64	48.8	49.4
OsR498G0101368300.01	0.706	0.103	-2.83471	3.45E-05	7.49E-05	0.8	0.7	0.62	0.14	0.17	0
OsR498G0101368900.01	13.433	5.263	-1.35585	1.48E-170	6.66E-171	13.39	13.12	13.79	4.91	5.46	5.42
OsR498G0101369200.01	4.286	43.926	3.370334	0	0	3.71	4.26	4.89	43.77	44.21	43.8
OsR498G0101369900.01	2.34	0.606	-1.95476	3.38E-28	3.92E-28	2.01	2.86	2.15	0.43	0.81	0.58
OsR498G0101370900.01	0.35	2.1	2.556818	1.36E-25	1.65E-25	0.51	0.19	0.35	2.16	2.16	1.98
OsR498G0101373900.01	2.236	0.813	-1.46491	5.41E-24	6.75E-24	2.28	2.13	2.3	0.88	0.83	0.73
OsR498G0101379100.01	2.163	0.6	-1.86146	8.98E-14	1.40E-13	2.59	2.13	1.77	0.41	0.75	0.64
OsR498G0101379800.01	2.063	0.216	-3.4087	5.77E-26	6.96E-26	2.25	2.03	1.91	0.15	0.04	0.46
OsR498G0101382500.01	0.493	51.236	6.68665	0	0	0.52	0.41	0.55	53.31	50.98	49.42
OsR498G0101383700.01	0.76	4.193	1.241986	1.29E-12	2.08E-12	0.96	0.58	0.74	2.41	4.08	6.09
OsR498G0101384900.01	2.6	0.073	-5.15006	1.23E-18	1.71E-18	2.78	2.03	2.99	0.22	0	0
OsR498G0101393500.01	1.343	0.296	-2.16591	3.29E-18	4.61E-18	1.34	1.61	1.08	0.25	0.35	0.29
OsR498G0101400000.01	30.12	12.366	-1.29293	1.22E-104	7.31E-105	30.42	29.38	30.56	11.09	14.19	11.82
OsR498G0101400200.01	16.74	5.606	-1.50093	6.44E-75	4.65E-75	18.38	16.31	15.53	6.23	6.18	4.41
OsR498G0101403500.01	22.343	2.776	-3.00896	0	0	22.12	21.66	23.25	2.77	2.73	2.83
OsR498G0101403700.01	0.23	0.06	-1.9196	3.40E-05	7.37E-05	0.29	0.23	0.17	0.1	0.08	0
OsR498G0101407900.01	3.486	33.123	3.291838	0	0	3.31	3.74	3.41	34.48	32.31	32.58
OsR498G0101409500.01	17.936	36.616	1.024849	3.77E-133	1.97E-133	17.95	18.79	17.07	37.01	36.76	36.08
OsR498G0101413800.01	0.386	0.763	1.020131	0.0001385	0.0003125	0.47	0.39	0.3	0.91	0.75	0.63
OsR498G0101415300.01	0.183	1.063	2.54226	0.0003732	0.0008685	0	0.38	0.17	1.12	1.32	0.75
OsR498G0101416500.01	4.633	63.163	3.756213	0	0	5.17	3.89	4.84	63.06	64.03	62.4
OsR498G0101417300.01	12.173	35.59	1.54519	2.85E-196	1.18E-196	13.34	12.38	10.8	35.79	35.23	35.75
OsR498G0101417500.01	0.076	0.463	2.54226	0.0003732	0.0008685	0	0.08	0.15	0.24	0.74	0.41
OsR498G0101418800.01	80.116	39.613	-1.02633	1.04E-131	5.47E-132	82.02	78.82	79.51	41.15	40.12	37.57
OsR498G0101420100.01	18.023	37.263	1.046336	7.03E-259	2.41E-259	18.13	18.5	17.44	36.8	36.44	38.55
OsR498G0101420300.01	40.523	91.396	1.161378	0	0	41.04	40.51	40.02	89	91.98	93.21
OsR498G0101423600.01	0	2.003	6.847115	7.65E-15	1.16E-14	0	0	0	2.33	2.06	1.62
OsR498G0101428600.01	0.16	2.686	4.03154	3.78E-39	3.80E-39	0.22	0.09	0.17	2.99	2.48	2.59
OsR498G0101433200.01	326.96	107.68	-1.63354	0	0	339.92	318.06	322.9	109.8	107.31	105.93
OsR498G0101435800.01	21.886	4.326	-2.33846	2.18E-250	7.67E-251	22.66	21.01	21.99	3.85	4.31	4.82
OsR498G0101439000.01	1.563	3.243	1.069668	3.96E-13	6.28E-13	1.95	2.74	0	0	2.79	6.94
OsR498G0101442100.01	0.57	0.093	-2.56228	1.04E-13	1.63E-13	0.51	0.7	0.5	0.18	0.06	0.04
OsR498G0101443900.01	29.14	87.956	1.591347	0	0	27.03	30.84	29.55	89.87	86.73	87.27
OsR498G0101445100.01	13.596	58.43	2.09464	0	0	12.37	14.41	14.01	57.42	60.25	57.62
OsR498G0101449900.01	0.376	1.386	1.847115	8.52E-06	1.79E-05	0.19	0.3	0.64	1.57	1	1.59
OsR498G0101453300.01	9.316	3.356	-1.56266	1.44E-50	1.28E-50	9.4	9.26	9.29	3.7	2.71	3.66
OsR498G0101455300.01	10.846	27.416	1.335406	1.13E-124	6.12E-125	11.02	11.76	9.76	27.1	27.96	27.19
OsR498G0101459000.01	0.38	0.046	-3.0477	0.0001372	0.0003095	0.4	0.35	0.39	0.14	0	0
OsR498G0101462100.01	21.15	73.463	1.783371	0	0	20	23.26	20.19	78.47	71.98	69.94
OsR498G0101466500.01	1.14	3.396	1.576412	5.67E-19	7.85E-19	0.86	1.36	1.2	3.28	3.13	3.78
OsR498G0101469100.01	0.31	24.446	6.291333	0	0	0.14	0.41	0.38	25.4	24.64	23.3
OsR498G0101469300.01	0.77	40.62	5.722476	0	0	0.55	0.95	0.81	43.7	40.76	37.4
OsR498G0101471100.01	0.183	2.896	3.953504	2.86E-46	2.67E-46	0.16	0.08	0.31	2.78	3.08	2.83
OsR498G0101471400.01	0.643	1.41	1.115424	5.77E-08	1.09E-07	0.77	0.47	0.69	1.24	1.63	1.36
OsR498G0101471900.01	1.323	0.136	-3.28217	4.93E-25	6.04E-25	1.36	1.25	1.36	0	0.19	0.22
OsR498G0101474700.01	12.353	4.453	-1.4712	1.08E-108	6.35E-109	12.1	12.04	12.92	4.19	4.29	4.88
OsR498G0101475400.01	2.53	32.506	3.67044	0	0	2.21	2.18	3.2	34.15	30.01	33.36
OsR498G0101476300.01	1457.8	14.066	-6.48352	0	0	1553.8	1405.2	1414	15.32	15.08	11.8
OsR498G0101476600.01	16.486	2.693	-2.63538	3.85E-66	2.98E-66	16.92	16.03	16.51	3.23	2.5	2.35
OsR498G0101477000.01	3.663	0.326	-3.72208	3.56E-63	2.82E-63	3.58	3.61	3.8	0.34	0.18	0.46
OsR498G0101477700.01	1656.1	8.796	-7.54386	0	0	1606.6	1633.9	1728	10.39	5.97	10.03
OsR498G0101479600.01	0.996	0.056	-4.15664	1.80E-13	2.83E-13	0.81	1.08	1.1	0.06	0.11	0
OsR498G0101480400.01	2.53	9.033	1.835221	2.44E-144	1.22E-144	2.48	2.64	2.47	8.59	9.2	9.31
OsR498G0101482200.01	12.57	125.14	3.331838	0	0	12.87	12.44	12.4	122.16	126.88	126.37
OsR498G0101482400.01	15.856	281.33	4.115001	0	0	16.44	15.26	15.87	283.1	275.81	285.08
OsR498G0101483000.01	21.8	83.97	1.930323	0	0	23.62	19.95	21.83	86.34	81.81	83.76
OsR498G0101483200.01	0.08	0.413	2.361688	4.96E-05	0.0001086	0.1	0	0.14	0.44	0.55	0.25
OsR498G0101485500.01	6.96	2.326	-1.58375	6.01E-84	4.08E-84	7.08	6.41	7.39	2.37	2.48	2.13
OsR498G0101487100.01	0.056	0.326	2.625005	2.32E-09	4.15E-09	0	0.17	0	0.16	0	0.82
OsR498G0101487400.01	2.44	5.083	1.078549	3.97E-16	5.85E-16	2.96	1.83	2.53	5.35	5.29	4.61
OsR498G0101488500.01	1.483	0.303	-2.28508	3.70E-12	6.05E-12	1.48	1.81	1.16	0.46	0.27	0.18
OsR498G0101488800.01	1.22	0.333	-1.89915	0.0005254	0.0012366	2.43	0.33	0.9	0.39	0	0.61
OsR498G0101497700.01	1.46	4.18	1.509347	9.60E-28	1.12E-27	1.55	1.05	1.78	4.51	3.49	4.54

OsR498G0101497900.01	0.013	0.173	3.361688	2.58E-09	4.62E-09	0.01	0.01	0.02	0.17	0.2	0.15
OsR498G0101500000.01	1.396	5.75	2.028789	2.63E-44	2.51E-44	1.36	1.15	1.68	5.6	5.66	5.99
OsR498G0101503700.01	1.713	19.28	3.496831	0	0	1.57	1.57	2	20.19	18.19	19.46
OsR498G0101506700.01	7.203	2.306	-1.65212	7.32E-52	6.44E-52	7.15	6.85	7.61	2.12	2.56	2.24
OsR498G0101507200.01	0.15	0	-4.96024	2.02E-05	4.33E-05	0.08	0.13	0.24	0	0	0
OsR498G0101515200.01	2.57	10.106	1.979469	2.36E-273	7.78E-274	2.31	3.13	2.27	9.81	9.92	10.59
OsR498G0101517800.01	0.373	34.566	6.537327	0	0	0.39	0.4	0.33	32.94	34.95	35.81
OsR498G0101518800.01	7.73	3.616	-1.11482	5.43E-10	9.51E-10	8.13	7.8	7.26	2.94	4.16	3.75
OsR498G0101526300.01	0.223	0.073	-1.71513	0.0005388	0.0012692	0.15	0.13	0.39	0.08	0.11	0.03
OsR498G0101526600.01	1.67	0.73	-1.17783	3.81E-16	5.62E-16	1.61	1.85	1.55	0.67	0.66	0.86
OsR498G0101530100.01	1.053	0.366	-1.63484	2.54E-08	4.72E-08	1.37	0.8	0.99	0.23	0.51	0.36
OsR498G0101530400.01	47.366	102.82	1.123235	0	0	48.54	46.99	46.57	100.92	106.09	101.46
OsR498G0101532700.01	1.45	0.3	-2.29929	1.62E-43	1.56E-43	1.3	1.4	1.65	0.36	0.2	0.34
OsR498G0101533500.01	27.656	63.21	1.193834	5.44E-276	1.78E-276	28.13	27.89	26.95	62.2	63.78	63.65
OsR498G0101537000.01	20.816	10.326	-1.0379	5.06E-75	3.65E-75	22.7	20.04	19.71	9.24	10.98	10.76
OsR498G0101540400.01	11.11	239.99	4.464551	0	0	11.48	11.9	9.95	247.74	226.43	245.8
OsR498G0101540500.01	0.836	32.943	5.280892	1.99E-252	6.93E-253	0.45	0.85	1.21	33.23	30.2	35.4
OsR498G0101550000.01	0.586	0.2	-1.5452	2.42E-06	4.93E-06	0.7	0.41	0.65	0.14	0.26	0.2
OsR498G0101550900.01	37.136	8.206	-2.18087	9.71E-285	3.10E-285	40.64	34.52	36.25	8.3	7.53	8.79
OsR498G0101551600.01	11.98	1.233	-3.29904	1.92E-155	9.14E-156	12.28	11.19	12.47	1.68	1.19	0.83
OsR498G0101551700.01	6.386	2.39	-1.44998	1.23E-32	1.34E-32	6.37	6.42	6.37	2.4	1.98	2.79
OsR498G0101552600.01	0.85	0.093	-3.2031	1.23E-26	1.47E-26	0.82	1.01	0.72	0.07	0.06	0.15
OsR498G0101553100.01	11.413	23.64	1.046024	3.32E-104	2.00E-104	11.13	11.59	11.52	22.69	23.55	24.68
OsR498G0101554700.01	0.893	0.41	-1.07313	1.36E-05	2.88E-05	0.94	0.91	0.83	0.44	0.27	0.52
OsR498G0101557500.01	3.323	0.43	-3.5587	8.08E-101	4.96E-101	3.83	2.91	3.23	0.57	0.5	0.22
OsR498G0101557800.01	40.746	0.236	-7.4618	0	0	40.96	41.56	39.72	0.28	0.36	0.07
OsR498G0101558500.01	1.096	0.26	-2.07113	2.76E-13	4.35E-13	1.08	1.39	0.82	0.23	0.42	0.13
OsR498G0101558800.01	0.396	0.106	-1.91976	0.0001314	0.0002958	0.53	0.12	0.54	0.06	0	0.26
OsR498G0101559000.01	3.826	0.03	-7.09979	2.11E-64	1.66E-64	2.87	4.54	4.07	0	0.09	0
OsR498G0101561600.01	0.163	1.336	3.094208	1.23E-20	1.64E-20	0.12	0.17	0.2	1.3	1.4	1.31
OsR498G0101562000.01	33.23	80.95	1.279415	0	0	31.52	34.04	34.13	79.84	83.3	79.71
OsR498G0101563100.01	18.563	41.676	1.153569	3.96E-97	2.47E-97	19.26	17.78	18.65	39.95	41.24	43.84
OsR498G0101565200.01	8.316	3.65	-1.22046	4.35E-84	2.94E-84	7.68	8.63	8.64	3.25	3.93	3.77
OsR498G0101571300.01	41.1	153.47	1.906356	0	0	41.92	40.18	41.2	148.42	154.47	157.51
OsR498G0101574800.01	0.026	0.273	3.361688	1.61E-05	3.42E-05	0.04	0.04	0	0.32	0.25	0.25
OsR498G0101576700.01	0.796	0.093	-3.10708	2.23E-12	3.62E-12	1.08	0.6	0.71	0.08	0.16	0.04
OsR498G0101577900.01	0.533	2.56	2.353869	8.81E-26	1.07E-25	0.69	0.43	0.48	2.96	2.1	2.62
OsR498G0101580700.01	19.946	68.563	1.775884	0	0	18.94	21.05	19.85	69.72	68.55	67.42
OsR498G0101581000.01	94.913	300.47	1.64698	0	0	96.83	95.8	92.11	294.13	303.36	303.91
OsR498G0101581900.01	0	0.96	7.952649	1.52E-27	1.79E-27	0	0	0	0.99	1.04	0.85
OsR498G0101582200.01	0.09	17.43	7.619286	0	0	0.18	0.06	0.03	17.35	18.34	16.6
OsR498G0101582300.01	0.61	21.63	5.132517	0	0	0.6	0.54	0.69	22.06	21.47	21.36
OsR498G0101582500.01	1.306	335.55	7.998395	0	0	1.46	1.23	1.23	340.61	335.23	330.82
OsR498G0101583500.01	0	0.236	5.499192	4.66E-07	9.16E-07	0	0	0	0.19	0.23	0.29
OsR498G0101585100.01	2.916	44.37	3.923014	0	0	3.16	2.8	2.79	45.48	43.28	44.35
OsR498G0101585300.01	9.356	78.186	3.057414	0	0	8.69	9.48	9.9	78.09	79	77.47
OsR498G0101585700.01	0.05	0.843	4.009386	7.69E-12	1.27E-11	0.1	0.05	0	0.79	1.04	0.7
OsR498G0101586800.01	0.126	1.146	3.17492	9.46E-24	1.19E-23	0.16	0.17	0.05	1.29	1.11	1.04
OsR498G0101590900.01	3.38	1.133	-1.90839	8.75E-73	6.43E-73	3.63	3.21	3.3	1.52	0.67	1.21
OsR498G0101593100.01	90.623	43.343	-1.06917	0	0	95.72	87.39	88.76	40.9	44.99	44.14
OsR498G0101594500.01	0.246	0.11	-1.63831	0.0002956	0.0006829	0.23	0.21	0.3	0.17	0.12	0.04
OsR498G0101594900.01	0.433	141.87	8.357173	0	0	0.48	0.67	0.15	146	133.41	146.19
OsR498G0101597400.01	0.51	0.163	-1.66068	0.0008332	0.0019932	0.41	0.55	0.57	0.18	0.19	0.12
OsR498G0101599100.01	2.44	5.206	1.065145	3.53E-16	5.19E-16	2.17	2.56	2.59	4.55	5.06	6.01
OsR498G0101599900.01	0.27	0	-5.35256	1.16E-06	2.33E-06	0.35	0.16	0.3	0	0	0
OsR498G0101603100.01	108.74	14.85	-2.88468	0	0	106.88	112.65	106.7	17.05	12.43	15.07
OsR498G0101606000.01	0.016	0.61	5.397312	1.30E-11	2.17E-11	0	0.05	0	0.84	0.36	0.63
OsR498G0101609600.01	2.1	21.093	3.3001	1.42E-237	5.14E-238	1.75	2.3	2.25	20.18	22.05	21.05
OsR498G0101613100.01	0.46	4.06	3.136622	1.75E-42	1.70E-42	0.36	0.53	0.49	3.52	4.54	4.12
OsR498G0101614100.01	0.54	115.07	7.7335	0	0	0.44	0.61	0.57	116.48	114.81	113.91
OsR498G0101615700.01	5.483	0.233	-4.57822	1.77E-99	1.10E-99	4.53	5.49	6.43	0.19	0.16	0.35
OsR498G0101616800.01	4.25	8.703	1.027687	3.29E-25	4.02E-25	3.77	4.31	4.67	9.57	8.07	8.47
OsR498G0101620900.01	6.906	15.496	1.155237	1.56E-34	1.66E-34	6.3	6.96	7.46	15.47	15.38	15.64
OsR498G0101621700.01	4482.8	226.17	-4.29165	0	0	4627	4308.7	4513	239.43	211.64	227.44
OsR498G0101622300.01	0.446	1.753	1.978359	1.29E-06	2.60E-06	0	0.92	0.42	1.13	2.07	2.06
OsR498G0101624900.01	9.973	2.196	-2.19005	4.46E-107	2.65E-107	10.17	9.42	10.33	1.9	2.17	2.52
OsR498G0101626900.01	0.3	0.68	1.165291	0.0004816	0.00113	0.25	0.21	0.44	0.54	0.39	1.11
OsR498G0101627300.01	12.566	50.923	1.962599	0	0	13.37	11.8	12.53	50.01	51.66	51.1
OsR498G0101629300.01	0.853	0.223	-1.96024	0.0009885	0.0023786	0.64	0.94	0.98	0	0.4	0.27

OsR498G0101631500.01	0.126	1.146	3.155237	4.76E-11	8.04E-11	0.19	0.07	0.12	1.17	1.21	1.06
OsR498G0101636800.01	4.266	10.063	1.272216	4.57E-60	3.72E-60	4.27	4.09	4.44	10	9.53	10.66
OsR498G0101637100.01	8.7	259.24	4.898055	0	0	7.53	9.46	9.11	264.8	257.95	254.98
OsR498G0101637800.01	0	0.403	4.361688	0.0005877	0.0013892	0	0	0	0.72	0.37	0.12
OsR498G0101640300.01	0.02	0.563	4.683616	1.48E-07	2.85E-07	0	0	0.06	0.4	0.61	0.68
OsR498G0101644900.01	0.29	0.64	1.129397	1.40E-05	2.97E-05	0.26	0.31	0.3	0.62	0.76	0.54
OsR498G0101648200.01	51.846	0.016	-11.5699	0	0	53.36	49.61	52.57	0.05	0	0
OsR498G0101651000.01	4.23	13.246	1.655617	1.08E-194	4.50E-195	3.55	4.62	4.52	12.91	13.05	13.78
OsR498G0101653700.01	16.256	5.853	-1.43542	7.65E-60	6.24E-60	16.01	17	15.76	5.52	6.32	5.72
OsR498G0101654100.01	0.22	0	-4.28217	0.0007127	0.0016975	0.07	0.27	0.32	0	0	0
OsR498G0101655500.01	8.913	55.093	2.62943	0	0	9.65	8.72	8.37	55.05	54.41	55.82
OsR498G0101656100.01	1.34	0.086	-3.98999	4.59E-22	5.95E-22	1.44	1.23	1.35	0	0.18	0.08
OsR498G0101656300.01	2.963	0.066	-5.47331	1.26E-33	1.36E-33	2.59	2.92	3.38	0.2	0	0
OsR498G0101660900.01	0.2	0.423	1.113761	2.35E-05	5.04E-05	0.19	0.17	0.24	0.39	0.37	0.51
OsR498G0101661500.01	0.91	0	-6.66068	1.07E-13	1.68E-13	0.85	0.99	0.89	0	0	0
OsR498G0101665200.01	0.183	0.86	2.361688	2.37E-08	4.40E-08	0.22	0.33	0	0.98	0.66	0.94
OsR498G0101665700.01	48.673	19.496	-1.32041	0	0	49.4	48.41	48.21	19.24	20.27	18.98
OsR498G0101677000.01	79.233	169.52	1.091906	0	0	80.51	78.31	78.88	169.48	170.18	168.91
OsR498G0101680500.01	0.036	0.446	3.629588	4.03E-07	7.90E-07	0.07	0.04	0	0.65	0.53	0.16
OsR498G0101680700.01	11.796	26.616	1.172135	6.53E-231	2.43E-231	11.69	11.9	11.8	25.81	26.54	27.5
OsR498G0101683700.01	0.916	0.383	-1.2628	1.02E-05	2.15E-05	0.89	1.08	0.78	0.26	0.43	0.46
OsR498G0101685000.01	2.736	10.743	1.956237	5.68E-33	6.16E-33	2.25	2.8	3.16	12.66	9.6	9.97
OsR498G0101690800.01	20.986	55.156	1.389075	3.56E-197	1.47E-197	30.4	18.81	13.75	57.58	52.66	55.23
OsR498G0101691600.01	3.613	51.21	3.839297	0	0	3.92	3.59	3.33	50.77	52.26	50.6
OsR498G0101693900.01	0.486	1.086	1.135519	7.75E-07	1.54E-06	0.48	0.54	0.44	0.97	1.29	1
OsR498G0101694200.01	0.06	0.466	2.946651	8.78E-07	1.75E-06	0	0.09	0.09	0.46	0.24	0.7
OsR498G0101695000.01	4.783	1.83	-1.38947	1.76E-37	1.80E-37	4.24	5.18	4.93	1.75	1.81	1.93
OsR498G0101697900.01	19.54	6.356	-1.62713	1.35E-87	8.94E-88	19.15	20.32	19.15	6.78	6.25	6.04
OsR498G0101698100.01	0.91	0.323	-1.49458	1.38E-07	2.65E-07	0.89	1.05	0.79	0.48	0.32	0.17
OsR498G0101698500.01	6.463	0.71	-3.18263	9.37E-70	7.06E-70	6.51	6.16	6.72	0.9	0.67	0.56
OsR498G0101701400.01	568.75	267.07	-1.09839	0	0	588.7	562.89	554.7	276.44	270.2	254.56
OsR498G0101702400.01	26.47	0.266	-6.63831	1.52E-234	5.59E-235	26.85	26.77	25.79	0.24	0.16	0.4
OsR498G0101705300.01	26.57	9.46	-1.51354	7.82E-197	3.23E-197	27.2	25.86	26.65	9.22	9.68	9.48
OsR498G0101706400.01	0.403	0.183	-1.13712	0.0004468	0.0010466	0.35	0.34	0.52	0.14	0.17	0.24
OsR498G0101709700.01	65.326	29.403	-1.14757	0	0	64.77	66.97	64.24	29.05	28.76	30.4
OsR498G0101710000.01	26.73	58.443	1.123663	1.50E-244	5.33E-245	27.1	27.39	25.7	59.89	55.53	59.91
OsR498G0101710100.01	3.106	0.853	-1.87535	7.10E-27	8.43E-27	3.34	3.2	2.78	0.89	0.82	0.85
OsR498G0101711000.01	0.966	7.026	2.853893	1.75E-104	1.05E-104	0.89	0.87	1.14	6.69	7.44	6.95
OsR498G0101713800.01	0.41	1.63	1.972646	3.59E-06	7.37E-06	0.56	0.35	0.32	2.19	1.41	1.29
OsR498G0101714900.01	12.256	5.063	-1.29155	2.47E-27	2.91E-27	11.85	12.64	12.28	4.47	5.42	5.3
OsR498G0101715500.01	325.28	162.05	-1.01444	0	0	332.96	323.18	319.7	165.94	159.7	160.51
OsR498G0101720100.01	7.836	37.813	2.2789	8.42E-247	2.98E-247	7.37	8.65	7.49	39.51	35.09	38.84
OsR498G0101722000.01	14.84	153.51	3.363922	0	0	14.84	15.46	14.22	163.54	148.79	148.19
OsR498G0101724500.01	2.61	0.903	-1.55048	9.97E-22	1.30E-21	2.76	2.45	2.62	1.1	0.59	1.02
OsR498G0101724900.01	0.566	0.203	-1.57691	1.15E-08	2.12E-08	0.38	0.74	0.58	0.21	0.24	0.16
OsR498G0101727800.01	1.073	0.23	-2.23074	3.61E-21	4.76E-21	1.04	1.24	0.94	0.22	0.25	0.22
OsR498G0101730000.01	0.563	2.606	2.213557	3.42E-48	3.13E-48	0.69	0.55	0.45	2.79	2.67	2.36
OsR498G0101730500.01	1.006	2.196	1.125733	2.55E-07	4.95E-07	1.33	0.89	0.8	2.22	2.43	1.94
OsR498G0101731100.01	0.06	0.24	1.978359	0.0003815	0.0008887	0.09	0.03	0.06	0.25	0.25	0.22
OsR498G0101734300.01	2.04	0.98	-1.06419	2.15E-09	3.84E-09	1.89	2.22	2.01	1.06	0.93	0.95
OsR498G0101734600.01	0.093	0.57	2.817368	1.89E-09	3.37E-09	0.09	0.04	0.15	0.52	0.56	0.63
OsR498G0101735400.01	0.226	0.466	1.03976	0.0008356	0.0019992	0.1	0.43	0.15	0.45	0.3	0.65
OsR498G0101736500.01	59.176	145.23	1.294076	0	0	60.5	57.76	59.27	144.76	140.85	150.07
OsR498G0101737000.01	6	14.116	1.238759	6.22E-239	2.25E-239	5.84	6.19	5.97	13.99	14.53	13.83
OsR498G0101745200.01	5.143	17.483	1.759441	1.14E-144	5.68E-145	5.44	4.7	5.29	19.1	17.22	16.13
OsR498G0101746700.01	9.316	108.89	3.54065	0	0	9.85	10.08	8.02	116.7	100.32	109.65
OsR498G0101747500.01	31.08	8.49	-1.85627	0	0	31.38	30.25	31.61	8.54	8.25	8.68
OsR498G0101747700.01	4.663	2.363	-1.00556	7.25E-27	8.62E-27	4.56	4.44	4.99	2.41	2.25	2.43
OsR498G0101748500.01	0.446	0	-6.76759	1.53E-14	2.34E-14	0.5	0.52	0.32	0	0	0
OsR498G0101749600.01	15.363	32.123	1.068588	4.91E-160	2.30E-160	15.22	15.72	15.15	32.32	32.34	31.71
OsR498G0101750900.01	43.613	21.153	-1.04788	1.15E-157	5.45E-158	44.75	42.12	43.97	22.06	20.27	21.13
OsR498G0101751200.01	1.106	7.733	2.800105	1.19E-81	8.18E-82	1.16	0.95	1.21	8.9	7.5	6.8
OsR498G0101752900.01	11.953	0.823	-3.86937	4.08E-132	2.14E-132	12.95	11.15	11.76	0.96	0.76	0.75
OsR498G0101757600.01	0.026	0.413	3.847115	0.0001179	0.0002648	0.08	0	0	0.52	0.54	0.18
OsR498G0101758800.01	13.403	1.613	-3.0622	0	0	11.37	14.37	14.47	1.45	1.78	1.61
OsR498G0101760800.01	0.58	0.18	-1.69721	6.43E-06	1.34E-05	0.59	0.33	0.82	0.14	0.26	0.14
OsR498G0101761100.01	0.35	0.81	1.214275	3.13E-09	5.63E-09	0.28	0.41	0.36	0.95	0.55	0.93
OsR498G0101761400.01	18.513	43.54	1.22255	2.89E-85	1.95E-85	17.7	18.76	19.08	46.1	45.11	39.41
OsR498G0101764500.01	36.9	78.963	1.08033	0	0	34.74	38.32	37.64	77.3	79.72	79.87

OsR498G0101767700.01	6.503	1.996	-1.71918	1.32E-31	1.46E-31	7.74	4.65	7.12	2.02	1.92	2.05
OsR498G0101769600.01	2.193	0.31	-2.84112	3.12E-38	3.17E-38	2.37	1.68	2.53	0.29	0.44	0.2
OsR498G0101770000.01	2.033	4.64	1.302137	5.26E-27	6.24E-27	1.61	2.09	2.4	4.94	3.98	5
OsR498G0101770600.01	3.246	35.736	3.436443	0	0	3.32	3.15	3.27	34	37.65	35.56
OsR498G0101772000.01	4.366	1.773	-1.30982	9.89E-11	1.69E-10	4.37	4.59	4.14	1.94	1.74	1.64
OsR498G0101772400.01	6.12	13.47	1.129658	2.52E-235	9.26E-236	5.11	6.82	6.43	12.89	13.6	13.92
OsR498G0101778900.01	1.17	13.6	3.532122	1.72E-139	8.78E-140	1.27	1.22	1.02	14.03	13.89	12.88
OsR498G0101785700.01	0.443	0.063	-2.77754	2.29E-11	3.84E-11	0.47	0.38	0.48	0.08	0.07	0.04
OsR498G0101787300.01	0.373	0.08	-2.24035	7.69E-06	1.61E-05	0.29	0.24	0.59	0.03	0.07	0.14
OsR498G0101788000.01	7.336	19.973	1.439496	1.21E-97	7.53E-98	7.33	7.25	7.43	18.52	21.3	20.1
OsR498G0101791800.01	3.013	0.81	-1.96024	3.55E-23	4.50E-23	2.97	3.15	2.92	0.7	1.12	0.61
OsR498G0101794400.01	4.833	12.486	1.365299	2.23E-63	1.77E-63	4.49	5.08	4.93	13.16	12.79	11.51
OsR498G0101799100.01	9.623	4.473	-1.09074	2.10E-51	1.86E-51	9.87	9.16	9.84	4.65	4.21	4.56
OsR498G0101800300.01	1.643	17.116	3.345941	4.00E-306	1.21E-306	1.71	1.33	1.89	16.69	16.43	18.23
OsR498G0101801800.01	39.15	19.043	-1.10226	4.00E-152	1.94E-152	39.17	38.86	39.42	18.16	19.28	19.69
OsR498G0101802300.01	31.733	74.186	1.229665	0	0	31.66	31.3	32.24	73.41	74.33	74.82
OsR498G0101803200.01	0.816	16.723	4.338009	3.60E-189	1.53E-189	0.86	0.89	0.7	18.37	15.93	15.87
OsR498G0101803400.01	1.593	17.686	3.463786	1.00E-155	4.77E-156	1.9	1.47	1.41	17.11	18.23	17.72
OsR498G0101804400.01	9.366	23.273	1.300551	3.85E-119	2.15E-119	10.01	9.03	9.06	23.99	23.68	22.15
OsR498G0101806500.01	12.313	169.1	3.765102	0	0	13.35	12.4	11.19	164.82	164.42	178.07
OsR498G0101808700.01	4.07	23.153	2.498744	2.67E-268	8.93E-269	4.19	3.47	4.55	22.82	23.08	23.56
OsR498G0101809000.01	0.213	0.693	1.743132	0.0001094	0.000245	0.29	0.1	0.25	0.47	0.74	0.87
OsR498G0101813200.01	17.406	7.016	-1.31475	1.76E-105	1.05E-105	16.59	17.07	18.56	7.51	7.24	6.3
OsR498G0101813900.01	0.8	0.126	-2.65056	3.97E-12	6.51E-12	0.87	0.78	0.75	0.14	0.14	0.1
OsR498G0101819100.01	60.616	5.976	-3.33216	0	0	59.59	62.69	59.57	5.88	6.79	5.26
OsR498G0101819800.01	10.993	467.48	5.403908	0	0	10.42	11.36	11.2	447.64	478.29	476.5
OsR498G0101820100.01	0.016	0.553	4.946651	7.63E-09	1.39E-08	0.05	0	0	0.6	0.56	0.5
OsR498G0101820800.01	2.423	0.42	-2.73026	3.48E-19	4.79E-19	2.46	2.2	2.61	0.29	0.45	0.52
OsR498G0101822500.01	2.51	10.073	2.007541	2.84E-237	1.03E-237	2.6	2.39	2.54	8.99	10.05	11.18
OsR498G0101823100.01	6.08	40.366	2.775033	0	0	6.75	6.52	4.97	44.78	37.92	38.4
OsR498G0101823600.01	0.123	0.89	2.864188	1.42E-15	2.12E-15	0.18	0.1	0.09	0.8	0.99	0.88
OsR498G0101829500.01	1.846	13.163	2.824202	1.80E-150	8.74E-151	1.67	1.75	2.12	13.66	12.33	13.5
OsR498G0101831600.01	0.186	18.22	6.601415	3.88E-289	1.23E-289	0.13	0.14	0.29	18.09	18.84	17.73
OsR498G0101832600.01	0.026	16.003	9.105849	3.28E-202	1.33E-202	0.04	0	0.04	16.85	15.69	15.47
OsR498G0101835100.01	85.466	170.97	1.004903	0	0	85.41	86.28	84.71	170.89	169.43	172.6
OsR498G0101837200.01	0.5	1.803	1.837267	1.07E-15	1.60E-15	0.36	0.52	0.62	1.49	2.18	1.74
OsR498G0101838400.01	4.623	13.123	1.490255	3.49E-123	1.91E-123	4.64	4.4	4.83	12.77	13.85	12.75
OsR498G0101841800.01	0.283	0.063	-2.16187	0.000227	0.0005204	0.07	0.35	0.43	0.07	0.12	0
OsR498G0101844100.01	354.23	149.67	-1.24858	0	0	366.01	349.53	347.2	142.04	155.45	151.53
OsR498G0101845100.01	13.086	1.1	-3.57717	3.79E-280	1.23E-280	13.35	12.67	13.24	1.14	1.3	0.86
OsR498G0101846200.01	3.413	0.733	-2.22917	1.16E-36	1.20E-36	3.54	3.24	3.46	0.78	0.66	0.76
OsR498G0101847400.01	0.573	3.296	2.531613	3.46E-08	6.48E-08	0.42	1.1	0.2	3.73	3.96	2.2
OsR498G0101849200.01	0.59	6.486	3.441208	3.38E-62	2.71E-62	0.47	0.44	0.86	7.8	6.29	5.37
OsR498G0101853300.01	0.25	4.15	4.036536	4.86E-97	3.04E-97	0.16	0.25	0.34	3.96	4.24	4.25
OsR498G0101856100.01	12.363	62.423	2.332725	0	0	11.38	13.09	12.62	61.5	62.39	63.38
OsR498G0101856600.01	2.933	16.24	2.549754	0	0	3.1	2.74	2.96	15.99	16.01	16.72
OsR498G0101856700.01	0	1.726	7.018185	2.46E-16	3.60E-16	0	0	0	1.74	2.24	1.2
OsR498G0101857000.01	0.233	3.67	3.954794	9.45E-44	9.06E-44	0.28	0.19	0.23	3.38	3.76	3.87
OsR498G0101864100.01	7.326	75.856	3.385451	0	0	6.54	8.27	7.17	73.51	75.07	78.99
OsR498G0101869400.01	26.03	2.156	-3.70418	0	0	25.02	25.75	27.32	2.11	2.59	1.77
OsR498G0101869700.01	32.076	69.11	1.097488	3.01E-307	9.02E-308	32.58	31.21	32.44	66.53	68.07	72.73
OsR498G0101869900.01	18.403	8.49	-1.19006	3.01E-80	2.09E-80	20.26	18.12	16.83	9.23	8.32	7.92
OsR498G0101870300.01	0.13	0.02	-2.96024	0.000243	0.0005585	0.07	0.08	0.24	0.03	0.03	0
OsR498G0101871100.01	19.433	55.603	1.378645	5.09E-258	1.75E-258	18.73	19.4	20.17	55.48	55.65	55.68
OsR498G0101875000.01	11.743	39.166	1.731559	3.44E-248	1.21E-248	11.87	11.39	11.97	41.25	40.57	35.68
OsR498G0101877100.01	4.24	0.353	-3.58906	9.10E-66	7.08E-66	3.9	4.84	3.98	0.19	0.52	0.35
OsR498G0101880600.01	3.85	87.546	4.695352	0	0	3.26	3.72	4.57	90.32	86.57	85.75
OsR498G0101881000.01	0	2.19	7.229585	2.17E-18	3.04E-18	0	0	0	1.77	3.09	1.71
OsR498G0101882900.01	5.97	26.796	2.003575	1.93E-181	8.41E-182	5.73	6.04	6.14	28.94	24.29	27.16
OsR498G0101884300.01	1.14	6.313	2.419903	3.94E-144	1.97E-144	1.32	1.22	0.88	5.74	6.91	6.29
OsR498G0101884600.01	0.183	0.866	2.182718	4.13E-08	7.76E-08	0.29	0.13	0.13	0.58	0.87	1.15
OsR498G0101885900.01	0.026	0.206	3.03976	0.0001737	0.0003945	0	0.04	0.04	0.19	0.16	0.27
OsR498G0101886400.01	0.07	0.24	1.7402	0.0005099	0.0011984	0.03	0.08	0.1	0.24	0.2	0.28
OsR498G0101896900.01	21.256	0.01	-10.9438	4.86E-283	1.56E-283	20.31	21.41	22.05	0.03	0	0
OsR498G0101898100.01	18.26	6.883	-1.41306	1.48E-111	8.59E-112	17.58	18.36	18.84	7.63	6.35	6.67
OsR498G0101902900.01	0.24	6.056	4.636695	1.60E-29	1.82E-29	0.14	0.3	0.28	5.8	6.65	5.72
OsR498G0101903500.01	0.53	9.013	4.101961	9.17E-221	3.52E-221	0.49	0.7	0.4	8.13	9.73	9.18
OsR498G0101904800.01	356.69	153.66	-1.22333	0	0	358.31	347.74	364	146.78	157.69	156.51
OsR498G0101904900.01	9.38	1.916	-2.31508	7.93E-35	8.40E-35	9.24	9.34	9.56	1.86	1.67	2.22

OsR498G0101906500.01	0.603	3.506	2.532295	1.51E-76	1.08E-76	0.56	0.55	0.7	3.21	3.36	3.95
OsR498G0101906700.01	0.69	0.1	-3.10529	2.25E-21	2.95E-21	0.6	0.78	0.69	0.14	0.04	0.12
OsR498G0101907100.01	1.78	0.3	-2.56358	7.09E-13	1.13E-12	2.5	1.68	1.16	0.27	0.49	0.14
OsR498G0101908400.01	85.216	805.02	3.236734	0	0	83.3	84.77	87.58	884.12	774.04	756.91
OsR498G0101908500.01	0.196	2.123	3.398783	3.27E-16	4.81E-16	0.25	0.12	0.22	3.19	1.62	1.56
OsR498G0101912100.01	4.35	1.736	-1.32919	4.47E-26	5.37E-26	4.38	4.59	4.08	1.79	1.69	1.73
OsR498G0101915900.01	1.806	0.63	-1.4979	2.91E-14	4.48E-14	1.92	1.46	2.04	0.57	0.67	0.65
OsR498G0101916100.01	29.373	14.613	-1.0202	9.32E-53	8.13E-53	28.96	27.34	31.82	14.24	15.28	14.32
OsR498G0101918900.01	1.613	6.786	2.065849	1.65E-63	1.30E-63	1.9	1.48	1.46	6.62	7.08	6.66
OsR498G0101919300.01	0.633	0.143	-2.11405	1.02E-11	1.69E-11	0.81	0.58	0.51	0.06	0.11	0.26
OsR498G0101922900.01	0.203	3.133	3.930531	1.51E-20	2.02E-20	0.3	0.11	0.2	3.13	3.09	3.18
OsR498G0101925700.01	0.126	3.953	4.716221	5.02E-43	4.85E-43	0.16	0.05	0.17	4.01	4.29	3.56
OsR498G0101926800.01	14.453	6.62	-1.13247	2.70E-59	2.22E-59	16.59	13.07	13.7	6.75	6.32	6.79
OsR498G0101929300.01	0	0.076	4.361688	0.0005877	0.0013892	0	0	0	0.09	0.07	0.07
OsR498G0101929800.01	0.046	9.81	7.757672	3.39E-260	1.16E-260	0.09	0.05	0	10.48	9.96	8.99
OsR498G0101930100.01	0.053	1.406	4.712185	1.36E-13	2.13E-13	0.08	0.08	0	1.3	1.34	1.58
OsR498G0101931000.01	5.986	1.356	-2.14539	7.62E-104	4.61E-104	5.76	5.85	6.35	1.22	1.33	1.52
OsR498G0101932000.01	0.116	2.876	4.769281	7.60E-106	4.53E-106	0.13	0.11	0.11	3.1	2.89	2.64
OsR498G0101932200.01	14.743	107.16	2.874596	0	0	15.57	14.25	14.41	108.71	107.28	105.49
OsR498G0101934600.01	0.35	0.033	-3.41967	0.0009442	0.002269	0.56	0.49	0	0	0.1	0
OsR498G0101936400.01	1.883	4.13	1.126269	4.39E-19	6.05E-19	1.8	1.89	1.96	3.66	4.36	4.37
OsR498G0101937700.01	299.99	19.046	-3.98244	0	0	297.05	301.48	301.4	18.76	19.66	18.72
OsR498G0101938600.01	0.186	2.48	3.690812	2.99E-42	2.91E-42	0.21	0.11	0.24	2.54	2.52	2.38
OsR498G0101939300.01	72.226	30.81	-1.23505	4.10E-236	1.50E-236	73.64	72.28	70.76	32.86	29.1	30.47
OsR498G0101939600.01	1.126	12.343	3.450434	0	0	0.99	1.1	1.29	11.88	12.55	12.6
OsR498G0101940200.01	1.026	3.393	1.721864	1.74E-20	2.33E-20	0.96	1.15	0.97	4.15	2.74	3.29
OsR498G0101941000.01	0.323	1.59	2.298494	2.61E-10	4.52E-10	0.28	0.29	0.4	2.25	1.09	1.43
OsR498G0101941700.01	11.413	5.346	-1.0995	6.99E-39	7.05E-39	12.77	10.85	10.62	5.46	5.22	5.36
OsR498G0101942000.01	1.66	0.39	-2.1055	2.58E-11	4.33E-11	1.89	1.29	1.8	0.42	0.47	0.28
OsR498G0101942300.01	0.026	0.39	3.794648	2.37E-07	4.61E-07	0.04	0.04	0	0.38	0.44	0.35
OsR498G0101944100.01	0.366	0.106	-1.80635	2.32E-05	4.98E-05	0.37	0.3	0.43	0.1	0.02	0.2
OsR498G0101946900.01	0.953	0.08	-3.61232	8.18E-19	1.14E-18	1.13	1.01	0.72	0.13	0.07	0.04
OsR498G0101947500.01	0.033	13.896	8.648939	3.66E-156	1.74E-156	0.05	0.05	0	14.53	14.57	12.59
OsR498G0101948700.01	8.563	2.786	-1.70482	2.09E-117	1.18E-117	9.03	8.78	7.88	2.56	2.62	3.18
OsR498G0101950700.01	18.006	173.06	3.263659	0	0	14.97	20.72	18.33	179.18	167.37	172.64
OsR498G0101951000.01	2.8	0.256	-3.04255	3.40E-14	5.25E-14	7.12	0	1.28	0	0.3	0.47
OsR498G0101951500.01	1.896	13.666	2.844549	2.19E-189	9.31E-190	1.93	1.7	2.06	13.75	14.54	12.71
OsR498G0101953400.01	3.113	15.563	2.316776	7.97E-163	3.69E-163	2.71	3.25	3.38	16.52	15.53	14.64
OsR498G0101953800.01	1.646	9.546	2.525869	6.67E-121	3.70E-121	1.83	1.37	1.74	10.2	9.19	9.25
OsR498G0101954500.01	0.273	2.483	3.163142	1.68E-23	2.12E-23	0.47	0.12	0.23	2.22	2.35	2.88
OsR498G0101957300.01	1.016	10.616	3.298041	1.12E-168	5.07E-169	1.28	0.73	1.04	11.36	10.14	10.35
OsR498G0101968200.01	3.916	26.843	2.912968	0	0	4.13	4.21	3.41	25.86	27.78	26.89
OsR498G0101968800.01	13.686	28.286	1.09292	1.58E-225	5.95E-226	13.4	13.9	13.76	28.61	27.91	28.34
OsR498G0101969600.01	0.123	0	-4.66068	0.0001179	0.0002647	0.2	0.05	0.12	0	0	0
OsR498G0101971900.01	7.713	31.593	2.025389	6.34E-292	1.99E-292	7.27	8.01	7.86	32.57	30.72	31.49
OsR498G0101973900.01	1.883	116.29	5.947238	0	0	1.31	2.17	2.17	121.51	113.75	113.6
OsR498G0101977900.01	2.636	1.153	-1.2007	9.87E-08	1.88E-07	2.24	3.08	2.59	1.07	1.23	1.16
OsR498G0101978300.01	25.66	6.27	-1.97567	4.66E-216	1.81E-216	28.28	23.92	24.78	6.3	6.22	6.29
OsR498G0101978600.01	1.543	0.393	-1.97654	6.83E-11	1.16E-10	1.34	1.79	1.5	0.47	0.49	0.22
OsR498G0101984100.01	13.093	5.286	-1.31371	9.84E-109	5.78E-109	13.5	12.87	12.91	5.65	5.21	5
OsR498G0101985200.01	0.22	0	-5.41967	6.63E-07	1.31E-06	0.26	0.29	0.11	0	0	0
OsR498G0101986700.01	12.16	25.323	1.134993	4.01E-81	2.78E-81	12.06	12.41	12.01	26.39	25.82	23.76
OsR498G0101987900.01	10.87	5.076	-1.09237	8.88E-117	5.03E-117	10.87	10.75	10.99	4.82	5.03	5.38
OsR498G0101988800.01	9.996	45.896	2.193156	0	0	10.65	9.91	9.43	46.6	47.98	43.11
OsR498G0101989600.01	0.233	0.766	1.717832	1.62E-11	2.69E-11	0.32	0.19	0.19	0.89	0.75	0.66
OsR498G0101992300.01	120.78	14.056	-3.11501	0	0	128.14	119.36	114.8	13.59	13.96	14.62
OsR498G0101993100.01	10.4	51.31	2.317609	0	0	10.72	10.65	9.83	49.51	52.86	51.56
OsR498G0101994300.01	14.353	39.246	1.446266	1.45E-265	4.86E-266	14.52	14.12	14.42	39.71	39.09	38.94
OsR498G0101996500.01	9.15	18.826	1.034606	3.62E-86	2.42E-86	8.73	9.25	9.47	17.26	20.63	18.59
OsR498G0101997600.01	9.956	44.68	2.159073	0	0	9.03	10.06	10.78	43.19	45.38	45.47
OsR498G0101999100.01	0.116	0.32	1.349088	3.19E-05	6.92E-05	0.16	0.03	0.16	0.28	0.31	0.37
OsR498G0102001400.01	2.016	0.473	-2.03063	1.09E-10	1.86E-10	1.58	2.43	2.04	0.52	0.33	0.57
OsR498G0102002900.01	6.486	1.116	-2.56457	3.02E-33	3.26E-33	7.29	6.7	5.47	1.32	1.13	0.9
OsR498G0102009100.01	18.766	92.07	2.293332	0	0	18.71	18.15	19.44	92.03	94.43	89.75
OsR498G0102009300.01	0.423	1.77	2.072182	8.34E-07	1.66E-06	0.34	0.71	0.22	1.75	2.02	1.54
OsR498G0102009600.01	0.663	2.43	1.885589	6.51E-15	9.87E-15	0.79	0.89	0.31	2.8	2.45	2.04
OsR498G0102010200.01	15.576	3.55	-2.13016	4.16E-283	1.33E-283	15.69	15.21	15.83	3.79	3.56	3.3
OsR498G0102012800.01	0.08	0.24	1.580328	0.0003419	0.0007932	0.02	0.07	0.15	0.22	0.34	0.16
OsR498G0102015300.01	0.97	0.136	-2.81822	8.48E-11	1.44E-10	0.7	1	1.21	0.2	0.16	0.05

OsR498G0102021500.01	0.03	11.74	8.488221	1.13E-212	4.43E-213	0.06	0.03	0	12.3	11.55	11.37
OsR498G0102022800.01	36.426	8.63	-2.08895	7.09E-193	2.98E-193	37.04	35.29	36.95	7.89	9.21	8.79
OsR498G0102025000.01	16.71	6.563	-1.35566	7.33E-73	5.38E-73	17.64	16.74	15.75	5.58	7.35	6.76
OsR498G0102026800.01	0.02	1.98	6.726261	6.93E-26	8.37E-26	0	0.06	0	1.87	2.1	1.97
OsR498G0102035800.01	0	0.593	5.287688	2.63E-06	5.36E-06	0	0	0	0.74	0.57	0.47
OsR498G0102036600.01	2.75	1.063	-1.36882	1.08E-28	1.25E-28	2.48	3.12	2.65	1.12	1.02	1.05
OsR498G0102038700.01	0.486	1.933	1.993956	8.54E-25	1.05E-24	0.47	0.49	0.5	2.07	1.77	1.96
OsR498G0102040200.01	38.3	78.47	1.031922	0	0	39.66	36.13	39.11	80	79.38	76.03
OsR498G0102040900.01	827.74	191.37	-2.10832	0	0	854.87	803.13	825.2	194.41	189.64	190.05
OsR498G0102044200.01	2.74	5.55	1.016126	4.20E-53	3.64E-53	2.89	2.6	2.73	5.48	5.56	5.61
OsR498G0102044400.01	233.58	108.44	-1.08299	0	0	257.09	225.79	217.9	113.01	104.34	107.98
OsR498G0102044800.01	2.07	0.85	-1.28217	3.80E-06	7.81E-06	1.62	2.51	2.08	1.34	0.65	0.56
OsR498G0102046000.01	5.873	1.86	-1.67266	8.19E-46	7.67E-46	5.41	6.31	5.9	2	2.04	1.54
OsR498G0102048200.01	123.43	61.21	-1.0177	2.41E-297	7.45E-298	121.8	126.39	122.1	62.45	60.68	60.5
OsR498G0102050400.01	90.7	17.116	-2.45529	0	0	92.63	90.79	88.68	16.62	15.51	19.22
OsR498G0102051900.01	4.92	58.693	3.543795	0	0	4.89	4.79	5.08	60.98	59.27	55.83
OsR498G0102052200.01	62.696	8.913	-2.80898	0	0	64.13	62.04	61.92	8.18	8.79	9.77
OsR498G0102052400.01	0.99	4.72	1.970497	6.45E-40	6.42E-40	1.23	1.17	0.57	4.79	5.12	4.25
OsR498G0102053200.01	0.256	0.696	1.453081	2.23E-05	4.78E-05	0.23	0.28	0.26	0.67	0.53	0.89
OsR498G0102054200.01	11.103	36.2	1.698453	4.09E-216	1.59E-216	10.49	11.6	11.22	35.51	36.71	36.38
OsR498G0102060600.01	0.126	1.816	3.817368	1.25E-21	1.63E-21	0.16	0.11	0.11	2.01	2.3	1.14
OsR498G0102064100.01	0.183	0.046	-1.96024	5.52E-05	0.0001214	0.14	0.21	0.2	0.02	0.07	0.05
OsR498G0102064500.01	3.12	0.813	-1.94436	1.07E-55	9.11E-56	2.77	3.45	3.14	0.7	0.87	0.87
OsR498G0102064700.01	5.63	16.456	1.520732	4.98E-142	2.52E-142	6.07	5.15	5.67	17.37	15.75	16.25
OsR498G0102065000.01	27.676	1.026	-4.7671	0	0	28.45	27.33	27.25	1.01	0.86	1.21
OsR498G0102065600.01	0.793	0	-7.28253	2.02E-19	2.77E-19	0.97	0.78	0.63	0	0	0
OsR498G0102068600.01	2.9	1.01	-1.52985	3.73E-15	5.62E-15	3.1	2.87	2.73	0.94	1.22	0.87
OsR498G0102070800.01	7.26	2.613	-1.50016	2.02E-71	1.50E-71	7.34	7.32	7.12	2.55	2.65	2.64
OsR498G0102072700.01	4.873	33.153	2.75228	8.33E-252	2.91E-252	5.53	4.82	4.27	32.66	33.6	33.2
OsR498G0102074800.01	0	0.523	6.325162	4.31E-11	7.27E-11	0	0	0	0.59	0.63	0.35
OsR498G0102075000.01	0.256	1.013	2.029706	2.48E-17	3.55E-17	0.37	0.08	0.32	1.25	0.73	1.06
OsR498G0102075300.01	4.496	20.103	2.149325	0	0	4.81	4.45	4.23	18.53	20.88	20.9
OsR498G0102077400.01	1754.9	326.81	-2.43649	0	0	1799.5	1685.7	1779	350.03	315.13	315.26
OsR498G0102078100.01	0.02	9.753	8.976398	5.41E-95	3.42E-95	0	0	0.06	10.99	9.15	9.12
OsR498G0102083800.01	0.076	0.573	2.87265	4.55E-11	7.69E-11	0.09	0.06	0.08	0.65	0.58	0.49
OsR498G0102085600.01	18.053	7.733	-1.22748	2.10E-113	1.20E-113	16.96	18.32	18.88	7.61	8.2	7.39
OsR498G0102087900.01	0.433	1.033	1.271086	9.71E-05	0.0002168	0.11	0.76	0.43	1.19	0.99	0.92
OsR498G0102088400.01	3.373	7.576	1.189584	4.43E-31	4.93E-31	3.07	3.76	3.29	7.19	7.84	7.7
OsR498G0102088500.01	0.133	0.443	2.165291	1.88E-07	3.63E-07	0.13	0.1	0.17	0.29	0.46	0.58
OsR498G0102089000.01	6.316	45.656	2.837309	0	0	6.24	6.36	6.35	45.01	46.08	45.88
OsR498G0102091500.01	1.393	6.27	2.167516	5.94E-85	4.00E-85	1.38	1.57	1.23	6.19	6.48	6.14
OsR498G0102091700.01	0.77	2.406	1.631936	4.99E-34	5.34E-34	0.96	0.61	0.74	2.16	2.42	2.64
OsR498G0102093700.01	0.383	0.086	-2.15289	2.08E-05	4.46E-05	0.11	0.56	0.48	0.04	0.04	0.18
OsR498G0102095500.01	22.066	55.096	1.303483	0	0	22.37	22.76	21.07	55.09	55.08	55.12
OsR498G0102095700.01	4.78	90.7	4.239609	0	0	4.61	4.66	5.07	89.57	91.55	90.98
OsR498G0102097100.01	0.676	3.486	2.368231	1.17E-59	9.60E-60	0.82	0.7	0.51	3.64	3.54	3.28
OsR498G0102097400.01	19.646	45.97	1.218351	0	0	19.36	19.66	19.92	44.45	45.99	47.47
OsR498G0102100200.01	2.696	19.483	2.839794	2.20E-292	6.89E-293	2.29	3.33	2.47	19.88	19.67	18.9
OsR498G0102100700.01	0.193	0.76	1.926219	1.50E-07	2.88E-07	0.14	0.07	0.37	0.45	1.03	0.8
OsR498G0102101700.01	13.28	35.346	1.407114	6.13E-113	3.53E-113	13.32	14.17	12.35	37.66	33.03	35.35
OsR498G0102101900.01	0.36	3.466	3.265583	1.60E-78	1.13E-78	0.41	0.37	0.3	3.22	3.34	3.84
OsR498G0102106700.01	2.86	0.9	-1.62591	2.43E-36	2.53E-36	2.68	3.08	2.82	1.03	0.88	0.79
OsR498G0102107800.01	7.366	16.586	1.15543	6.65E-61	5.38E-61	7.98	6.87	7.25	17.77	16.26	15.73
OsR498G0102109500.01	1.023	2.823	1.461916	1.02E-16	1.49E-16	0.8	1.26	1.01	2.75	2.79	2.93
OsR498G0102109700.01	3.113	13.04	2.165248	2.17E-190	9.18E-191	3.21	3.21	2.92	11.82	14.08	13.22
OsR498G0102110200.01	12.093	2.926	-2.077	7.29E-205	2.93E-205	13.04	11.22	12.02	2.97	2.84	2.97
OsR498G0102111100.01	159.82	595.5	1.867646	0	0	158.42	163.84	157.2	604.3	588.03	594.18
OsR498G0102111800.01	3.176	1.483	-1.10226	8.22E-13	1.32E-12	3.04	3.07	3.42	1.81	1.6	1.04
OsR498G0102119200.01	3.196	0.076	-5.39765	5.53E-62	4.43E-62	2.76	3.24	3.59	0.04	0.04	0.15
OsR498G0102119400.01	12.376	1.53	-3.03035	1.08E-172	4.84E-173	12.15	12.29	12.69	1.4	1.64	1.55
OsR498G0102119600.01	26.68	56.296	1.065472	0	0	25.71	26.97	27.36	54.05	58.37	56.47
OsR498G0102120400.01	0.853	20.043	4.525893	0	0	0.66	1.06	0.84	20.73	20.13	19.27
OsR498G0102121700.01	4.746	15.976	1.752478	3.77E-101	2.31E-101	4.26	4.92	5.06	15.38	16.71	15.84
OsR498G0102122700.01	0.823	0.36	-1.20417	0.0005981	0.0014149	1.04	0.69	0.74	0.39	0.52	0.17
OsR498G0102125500.01	0.39	4.793	3.525772	1.85E-97	1.16E-97	0.37	0.25	0.55	4.94	4.55	4.89
OsR498G0102125800.01	1.986	4.683	1.234964	2.48E-44	2.36E-44	1.87	2.35	1.74	4.74	4.67	4.64
OsR498G0102126800.01	0.793	0.35	-1.18731	9.01E-07	1.80E-06	0.81	0.72	0.85	0.33	0.19	0.53
OsR498G0102127700.01	0.41	0	-6.16969	2.07E-10	3.57E-10	0.43	0.45	0.35	0	0	0
OsR498G0102129600.01	25.923	97.84	1.876948	0	0	27.46	23.63	26.68	96.44	99.77	97.31

OsR498G0102129800.01	0.076	18.486	7.883158	2.51E-149	1.23E-149	0.15	0.08	0	19.75	17.44	18.27
OsR498G0102130200.01	5.096	100.02	4.280281	0	0	5.93	4.59	4.77	99.5	103.45	97.1
OsR498G0102130600.01	1.27	21.46	4.072927	1.67E-152	8.06E-153	1.23	1.48	1.1	21.73	21.55	21.1
OsR498G0102131700.01	0.013	0.473	4.993956	4.24E-09	7.66E-09	0.04	0	0	0.27	0.42	0.73
OsR498G0102132300.01	10.543	48.44	2.241258	0	0	9.83	11.06	10.74	48.07	49.71	47.54
OsR498G0102133700.01	0.506	5.513	3.433102	1.78E-135	9.24E-136	0.46	0.54	0.52	5.43	5.54	5.57
OsR498G0102134700.01	3.673	12.18	1.724594	9.02E-129	4.82E-129	3.92	3.75	3.35	12.87	11.45	12.22
OsR498G0102135900.01	1.126	15.163	3.744304	2.98E-73	2.18E-73	1.61	1.17	0.6	16.63	12.64	16.22
OsR498G0102136500.01	0	0.456	5.624723	1.50E-07	2.88E-07	0	0	0	0.45	0.52	0.4
OsR498G0102136800.01	0.66	4.146	2.653577	6.24E-32	6.86E-32	0.77	0.75	0.46	4.55	3.19	4.7
OsR498G0102137100.01	4.48	35.253	2.947642	2.16E-122	1.19E-122	4.43	3.66	5.35	36.85	32.62	36.29
OsR498G0102137300.01	0.24	3.23	3.721584	1.92E-17	2.74E-17	0.12	0.25	0.35	2.74	3.17	3.78
OsR498G0102137700.01	5.356	150.52	4.787108	0	0	5.75	5.68	4.64	154.29	147.76	149.51
OsR498G0102139500.01	38.88	84.336	1.117876	1.90E-256	6.56E-257	42.41	35.4	38.83	80.44	84.67	87.9
OsR498G0102139800.01	23.176	11.793	-1.04815	8.58E-66	6.67E-66	23.06	23.15	23.32	11.54	10.97	12.87
OsR498G0102148800.01	3.603	17.743	2.278136	3.03E-194	1.26E-194	3.45	3.58	3.78	18.15	18.84	16.24
OsR498G0102149100.01	11.64	120.49	3.43322	0	0	11.04	11.71	12.17	120.13	120.65	120.68
OsR498G0102153400.01	1.203	2.79	1.199937	9.86E-17	1.43E-16	1.01	1.31	1.29	2.48	2.95	2.94
OsR498G0102154100.01	48.733	14.043	-1.78799	6.15E-270	2.04E-270	49.64	47.15	49.41	14.94	14.02	13.17
OsR498G0102154900.01	14.446	4.746	-1.53775	1.73E-98	1.07E-98	14.75	14.74	13.85	4.57	5.18	4.49
OsR498G0102157700.01	158.92	46.103	-1.81634	0	0	168	155.39	153.4	45.69	47.83	44.79
OsR498G0102159600.01	2.833	0.033	-6.36963	1.71E-60	1.39E-60	2.49	3.03	2.98	0	0.07	0.03
OsR498G0102165300.01	0.086	0.343	2.009386	7.98E-07	1.59E-06	0.06	0.16	0.04	0.28	0.42	0.33
OsR498G0102165700.01	0.636	0.316	-1.01087	0.0006974	0.0016588	0.69	0.87	0.35	0.29	0.33	0.33
OsR498G0102166000.01	730.79	153.5	-2.25191	0	0	734.96	727.66	729.8	156.61	153.22	150.67
OsR498G0102166100.01	3.06	0.803	-1.9424	3.73E-14	5.77E-14	2.26	3.38	3.54	0.77	0.62	1.02
OsR498G0102166300.01	90.163	8.746	-3.37272	0	0	93.5	88.43	88.56	9.08	9.07	8.09
OsR498G0102166500.01	0.61	3.2	2.387299	6.83E-47	6.32E-47	0.57	0.6	0.66	3.37	3.12	3.11
OsR498G0102166700.01	3.36	0.75	-2.17035	3.96E-33	4.29E-33	3.35	3.43	3.3	0.56	1.04	0.65
OsR498G0102167600.01	21.566	5.8	-1.88248	1.49E-118	8.33E-119	22.22	21.54	20.94	6.5	5.16	5.74
OsR498G0102167900.01	1.27	0.206	-2.79031	2.02E-21	2.65E-21	1	1.43	1.38	0.28	0.19	0.15
OsR498G0102169100.01	1.19	0.56	-1.1091	0.0006186	0.0014653	1.47	1.23	0.87	0.35	0.67	0.66
OsR498G0102177400.01	8.896	4.433	-1.07319	3.53E-47	3.26E-47	9.42	8.01	9.26	4.49	3.88	4.93
OsR498G0102178300.01	0.7	0	-7.24564	5.05E-19	6.99E-19	0.91	0.59	0.6	0	0	0
OsR498G0102180100.01	0.716	0.02	-5.13016	2.16E-18	3.02E-18	0.68	0.9	0.57	0	0.03	0.03
OsR498G0102182300.01	111.43	285.15	1.344483	0	0	112.89	108.27	113.1	287.26	278.17	290.01
OsR498G0102184500.01	0.156	1.216	2.981029	4.14E-19	5.70E-19	0.11	0.26	0.1	0.81	1.47	1.37
OsR498G0102187200.01	16.62	0.526	-4.96786	0	0	17.31	16.18	16.37	0.5	0.46	0.62
OsR498G0102187300.01	2.906	0.473	-2.6571	5.18E-31	5.77E-31	2.72	3.03	2.97	0.5	0.51	0.41
OsR498G0102187500.01	54.913	7.883	-2.80703	0	0	53.18	56.53	55.03	7.84	6.93	8.88
OsR498G0102193100.01	2.356	9.133	1.953048	1.06E-36	1.09E-36	2.18	2.94	1.95	10.04	8.11	9.25
OsR498G0102199200.01	2.89	0.016	-6.76759	3.21E-27	3.79E-27	3.15	2.77	2.75	0	0.05	0
OsR498G0102199400.01	2.09	0.88	-1.23777	4.43E-10	7.74E-10	2.07	2.19	2.01	1.04	1.08	0.52
OsR498G0102200100.01	0.023	1.12	5.87265	1.73E-15	2.59E-15	0	0.07	0	0.95	1	1.41
OsR498G0102201900.01	7.07	3.353	-1.08189	1.18E-32	1.28E-32	7.79	7.21	6.21	3.79	3.29	2.98
OsR498G0102202700.01	0.14	0.44	1.702725	1.16E-06	2.32E-06	0.05	0.28	0.09	0.37	0.4	0.55
OsR498G0102203000.01	18.766	41.24	1.139522	1.05E-254	3.63E-255	19.43	18.55	18.32	41.27	42.74	39.71
OsR498G0102205200.01	6.14	17.626	1.519606	1.32E-113	7.59E-114	6.26	6.29	5.87	18.44	17.89	16.55
OsR498G0102206700.01	26.356	7.683	-1.77736	0	0	26.54	26.93	25.6	8.15	6.56	8.34
OsR498G0102207400.01	0.39	1.28	1.706517	1.02E-12	1.65E-12	0.62	0.22	0.33	1.34	1.25	1.25
OsR498G0102207500.01	0.056	0.493	3.115048	3.58E-12	5.86E-12	0.07	0.05	0.05	0.62	0.46	0.4
OsR498G0102207800.01	0.846	26.16	4.965596	0	0	0.52	1.31	0.71	25.87	26.7	25.91
OsR498G0102210700.01	0.61	0.273	-1.14244	3.68E-07	7.19E-07	0.63	0.65	0.55	0.35	0.22	0.25
OsR498G0102211800.01	16.42	36.083	1.013597	5.47E-96	3.44E-96	16.22	16.39	16.65	36.51	37.13	34.61
OsR498G0102213600.01	0.323	0	-5.13016	6.38E-06	1.33E-05	0.43	0.39	0.15	0	0	0
OsR498G0102214200.01	21.306	9.356	-1.25167	3.31E-80	2.31E-80	22.01	19.9	22.01	9.77	8.59	9.71
OsR498G0102215800.01	0.076	0.486	3.338208	3.51E-18	4.94E-18	0.02	0.09	0.12	0.52	0.55	0.39
OsR498G0102216700.01	3.573	1.51	-1.11469	4.90E-19	6.76E-19	3.09	4.08	3.55	1.73	1.39	1.41
OsR498G0102218300.01	22.473	47.683	1.130446	7.86E-278	2.55E-278	22.29	23.77	21.36	47.95	45.85	49.25
OsR498G0102222100.01	0.11	3.33	4.924624	1.35E-77	9.57E-78	0.12	0.07	0.14	3.21	3.63	3.15
OsR498G0102222400.01	0.153	0.48	1.624723	1.38E-06	2.77E-06	0.18	0.12	0.16	0.4	0.49	0.55
OsR498G0102223200.01	0.243	8.473	5.122973	2.30E-161	1.07E-161	0.18	0.3	0.25	9.47	7.36	8.59
OsR498G0102228300.01	0.42	1.06	1.33225	2.84E-08	5.30E-08	0.39	0.4	0.47	1.09	1.09	1
OsR498G0102228600.01	12.753	6.156	-1.06172	2.94E-46	2.74E-46	14.06	12.16	12.04	5.89	6.19	6.39
OsR498G0102231000.01	14.86	52.573	1.816821	0	0	15.43	14.05	15.1	53.42	52.5	51.8
OsR498G0102233600.01	8.906	1.273	-2.86713	3.00E-98	1.86E-98	8.67	8	10.05	1.06	1.16	1.6
OsR498G0102236300.01	4.196	1.303	-1.80824	8.72E-41	8.61E-41	4.26	4.14	4.19	0.79	1.54	1.58
OsR498G0102236900.01	0.463	1.15	1.330991	2.52E-07	4.90E-07	0.47	0.27	0.65	1.29	1.08	1.08
OsR498G0102239000.01	0.25	1.11	2.163796	6.25E-26	7.53E-26	0.36	0.24	0.15	1.16	0.94	1.23

OsR498G0102239800.01	0.93	3.73	2.001754	1.58E-53	1.36E-53	1.02	0.93	0.84	3.83	3.95	3.41
OsR498G0102242100.01	8.083	26.693	1.732511	0	0	7.59	8.56	8.1	25.88	27.81	26.39
OsR498G0102242700.01	3.663	16.956	2.183038	1.35E-179	5.93E-180	3.51	3.94	3.54	17.64	16.06	17.17
OsR498G0102247400.01	11.153	137.99	3.617986	0	0	9.86	11.23	12.37	145.47	136.78	131.73
OsR498G0102247500.01	3.966	13.34	1.788368	2.21E-145	1.10E-145	3.56	4.21	4.13	13.64	14.1	12.28
OsR498G0102247900.01	56.813	24.95	-1.1945	1.20E-102	7.32E-103	55.69	59.74	55.01	26.4	24.74	23.71
OsR498G0102249300.01	1.47	127.07	6.438002	0	0	1.35	1.81	1.25	129.87	126.37	124.98
OsR498G0102251200.01	1.716	0.093	-4.16969	1.62E-17	2.30E-17	1.72	2.03	1.4	0.14	0	0.14
OsR498G0102254000.01	0.94	2.863	1.557046	3.50E-32	3.83E-32	0.76	1.05	1.01	2.92	2.6	3.07
OsR498G0102260500.01	1.31	0.636	-1.2876	4.07E-09	7.34E-09	1.1	1.53	1.3	0.72	0.52	0.67
OsR498G0102261800.01	1.606	4.98	1.574599	2.48E-52	2.17E-52	1.69	1.52	1.61	4.52	4.71	5.71
OsR498G0102262400.01	2.193	14.24	2.700011	1.43E-162	6.63E-163	2.02	2.61	1.95	14.97	14.67	13.08
OsR498G0102265100.01	1.58	39.233	4.631187	0	0	1.28	1.85	1.61	39.95	39.5	38.25
OsR498G0102268800.01	0.256	4.47	4.092871	5.83E-91	3.79E-91	0.15	0.32	0.3	4.92	4.57	3.92
OsR498G0102270000.01	25.753	51.55	1.000146	4.47E-104	2.70E-104	26.51	26.46	24.29	51.34	49.08	54.23
OsR498G0102271500.01	0.03	25.676	9.673966	0	0	0.03	0	0.06	27.5	24.83	24.7
OsR498G0102273300.01	1.01	9.503	3.227503	3.04E-251	1.06E-251	0.98	1.21	0.84	9.52	9.41	9.58
OsR498G0102273600.01	1.6	0.15	-3.43502	7.77E-37	8.01E-37	1.47	1.54	1.79	0.18	0.16	0.11
OsR498G0102273800.01	16.88	37.973	1.161075	1.08E-139	5.49E-140	18	15.41	17.23	37.35	37.34	39.23
OsR498G0102274300.01	2.533	15.32	2.572905	9.02E-116	5.12E-116	2.38	2.23	2.99	15.99	15.42	14.55
OsR498G0102274800.01	0.116	0.316	1.441858	0.0003225	0.0007473	0.2	0.08	0.07	0.25	0.21	0.49
OsR498G0102275800.01	1.306	0.056	-4.4838	3.73E-37	3.84E-37	1.12	1.18	1.62	0.07	0	0.1
OsR498G0102276700.01	17.376	37.74	1.144073	1.16E-285	3.72E-286	18.22	17.1	16.81	36.4	39.74	37.08
OsR498G0102277200.01	0.626	0.266	-1.23469	0.0002449	0.0005631	0.47	1.17	0.24	0	0.42	0.38
OsR498G0102281000.01	1.78	6.03	1.758859	5.88E-21	7.78E-21	1.71	2.14	1.49	6.5	5.48	6.11
OsR498G0102283700.01	58.273	11.81	-2.27842	0	0	59.01	56.29	59.52	11.78	12.04	11.61
OsR498G0102284000.01	16.123	31.49	1.049293	9.32E-143	4.69E-143	15.24	17.48	15.65	30.44	30.67	33.36
OsR498G0102284100.01	0.15	0.556	1.902257	9.28E-06	1.95E-05	0.16	0.21	0.08	0.53	0.3	0.84
OsR498G0102289000.01	17.2	50.743	1.490505	0	0	15.57	19.86	16.17	57.11	46.45	48.67
OsR498G0102290000.01	2.01	69.796	5.11402	0	0	2.11	2.19	1.73	68.34	71.25	69.8
OsR498G0102290700.01	0.65	1.92	1.55221	4.50E-09	8.14E-09	0.57	0.72	0.66	1.52	2.22	2.02
OsR498G0102291100.01	8.346	23.11	1.447448	2.52E-133	1.32E-133	8.01	8.83	8.2	21.08	23.4	24.85
OsR498G0102298800.01	0.043	20.663	8.925456	1.07E-182	4.64E-183	0.13	0	0	22.64	19.87	19.48
OsR498G0102299000.01	0.37	0.013	-4.91444	7.22E-09	1.32E-08	0.54	0.19	0.38	0	0.04	0
OsR498G0102301300.01	0.063	0.423	2.887757	1.13E-07	2.17E-07	0.07	0.07	0.05	0.32	0.53	0.42
OsR498G0102306400.01	1.013	2.246	1.201765	3.95E-09	7.13E-09	0.82	1.14	1.08	2.05	2.35	2.34
OsR498G0102309500.01	13.853	34.026	1.310966	1.25E-269	4.15E-270	14.38	13.26	13.92	33.23	34.04	34.81
OsR498G0102309900.01	4.76	401.17	6.390676	0	0	4.75	5.22	4.31	408.92	400.79	393.8
OsR498G0102311900.01	53.476	238.59	2.152288	0	0	51.76	54	54.67	236.49	241.38	237.9
OsR498G0102312600.01	0	0.27	5.897741	9.23E-09	1.69E-08	0	0	0	0.22	0.28	0.31
OsR498G0102315900.01	1.31	3.176	1.283272	2.04E-22	2.63E-22	1.31	1.43	1.19	2.97	3.07	3.49
OsR498G0102318300.01	5.453	15.683	1.560894	6.73E-80	4.68E-80	5.18	5.72	5.46	15.32	15.72	16.01
OsR498G0102318600.01	5.316	12.89	1.255193	1.91E-61	1.54E-61	5.86	5.21	4.88	12.41	13.59	12.67
OsR498G0102319600.01	1.196	0.226	-2.41275	2.10E-15	3.15E-15	1.23	1.47	0.89	0.21	0.29	0.18
OsR498G0102321300.01	0.086	0.273	1.468603	0.0003802	0.0008855	0.06	0.06	0.14	0.27	0.32	0.23
OsR498G0102322000.01	0.126	8.413	6.071181	1.70E-147	8.37E-148	0.17	0.13	0.08	9.29	7.16	8.79
OsR498G0102322100.01	0.04	4.873	6.868507	1.86E-97	1.16E-97	0.06	0	0.06	4.98	4.6	5.04
OsR498G0102325500.01	17.576	47.82	1.426505	2.66E-290	8.39E-291	15.19	18.82	18.72	49.76	49.12	44.58
OsR498G0102327700.01	37.453	133.99	1.818782	0	0	37.24	35.52	39.6	128.92	136.24	136.8
OsR498G0102328500.01	144.51	300.58	1.049573	0	0	146.37	142.06	145.1	299.44	301.3	301
OsR498G0102328800.01	3.483	19.14	2.44444	5.14E-121	2.85E-121	3.33	3.17	3.95	19.8	19	18.62
OsR498G0102329500.01	78.516	259.28	1.714952	0	0	80.54	75.83	79.18	257.43	258.97	261.44
OsR498G0102330000.01	7.476	2.433	-1.62959	3.75E-37	3.85E-37	7.33	7.6	7.5	2.08	2.44	2.78
OsR498G0102331100.01	9.703	2.35	-2.05538	2.26E-195	9.38E-196	9.68	10.01	9.42	2.17	2.21	2.67
OsR498G0102331600.01	1.41	0.746	-1.01237	3.20E-11	5.39E-11	1.4	1.21	1.62	0.69	0.89	0.66
OsR498G0102333600.01	1.513	3.676	1.264547	7.41E-13	1.19E-12	1.8	1.05	1.69	3.45	3.89	3.69
OsR498G0102334700.01	11.86	71.88	2.603753	0	0	12.75	11.96	10.87	70.57	71.16	73.91
OsR498G0102335000.01	1.603	0.456	-1.80824	1.13E-06	2.26E-06	1.49	2.22	1.1	0.81	0.28	0.28
OsR498G0102335800.01	0.97	0.35	-1.47049	1.36E-07	2.60E-07	0.71	1.1	1.1	0.54	0.14	0.37
OsR498G0102335900.01	17.923	7.556	-1.26478	7.92E-22	1.03E-21	17.2	19.35	17.22	7.29	7.36	8.02
OsR498G0102338100.01	10.08	26.306	1.350069	1.15E-162	5.31E-163	9.16	11.2	9.88	28.24	25.24	25.44
OsR498G0102338900.01	7.863	59.676	2.915371	0	0	8.51	7.75	7.33	58.23	60.25	60.55
OsR498G0102339600.01	0.176	0.503	1.468603	0.0003802	0.0008855	0	0.17	0.36	0.51	0.61	0.39
OsR498G0102339900.01	1.1	118.03	6.74499	0	0	1.28	0.88	1.14	118.08	119.88	116.12
OsR498G0102340200.01	0.613	18.953	4.91368	3.72E-67	2.86E-67	0	0.73	1.11	19.88	17.96	19.02
OsR498G0102341300.01	81.983	1351.4	4.036273	0	0	77.86	87.94	80.15	1412.3	1332.4	1309.5
OsR498G0102341800.01	27.44	57.616	1.092951	1.13E-257	3.88E-258	27.69	26.87	27.76	56.26	57.28	59.31
OsR498G0102345000.01	92.216	18.833	-2.30225	0	0	91.49	91.31	93.85	16.63	20.15	19.72
OsR498G0102345600.01	4.03	1.883	-1.07113	5.53E-19	7.65E-19	3.71	3.89	4.49	2.09	1.91	1.65

OsR498G0102350700.01	33.22	68.97	1.048679	0	0	33.39	33.51	32.76	69.67	69.64	67.6
OsR498G0102352000.01	1.333	3.07	1.186601	7.83E-05	0.0001739	1.27	1.5	1.23	3.53	3.89	1.79
OsR498G0102354500.01	18.553	3.516	-2.40401	1.97E-268	6.57E-269	17.57	18.69	19.4	3.66	3.51	3.38
OsR498G0102360100.01	18.886	8.89	-1.08549	4.62E-119	2.59E-119	18.59	20.38	17.69	10.08	8.65	7.94
OsR498G0102361800.01	0.403	9.416	4.549992	5.41E-270	1.79E-270	0.37	0.48	0.36	9.96	9.17	9.12
OsR498G0102367000.01	3.343	1.06	-1.665	3.67E-42	3.58E-42	3.56	3.38	3.09	0.93	1.14	1.11
OsR498G0102368400.01	2.946	0.803	-1.88797	1.33E-18	1.86E-18	3.13	2.44	3.27	0.93	0.48	1
OsR498G0102368800.01	12.196	43.926	1.735812	5.98E-260	2.04E-260	11.65	12.44	12.5	46.57	42	43.21
OsR498G0102369300.01	0.84	0.046	-3.99838	1.36E-25	1.64E-25	0.99	0.83	0.7	0	0.06	0.08
OsR498G0102369600.01	0.43	0.943	1.130908	1.44E-08	2.65E-08	0.37	0.51	0.41	1.02	0.96	0.85
OsR498G0102369900.01	15.82	6.49	-1.29265	2.14E-123	1.17E-123	16.79	15.53	15.14	6.22	6.49	6.76
OsR498G0102370400.01	2.56	5.32	1.053057	9.49E-11	1.62E-10	2.61	2.96	2.11	6.06	4.9	5
OsR498G0102370800.01	2.166	10.633	2.252948	1.99E-168	9.02E-169	2.15	1.92	2.43	11.48	10.05	10.37
OsR498G0102373300.01	2.933	5.956	1.019296	1.52E-23	1.92E-23	3.15	2.88	2.77	6.58	6.29	5
OsR498G0102374400.01	0.166	0.503	1.594349	2.00E-05	4.27E-05	0.31	0.16	0.03	0.54	0.36	0.61
OsR498G0102377300.01	0	0.156	4.7402	9.30E-05	0.0002074	0	0	0	0.14	0.11	0.22
OsR498G0102377800.01	2.903	0.993	-1.58973	7.05E-22	9.16E-22	2.65	3.06	3	1.15	0.73	1.1
OsR498G0102380900.01	4.163	9.266	1.145583	4.42E-54	3.80E-54	3.87	4.51	4.11	9.82	8.98	9
OsR498G0102383900.01	111.26	13.86	-3.01414	0	0	104.96	118.05	110.8	13.76	13.11	14.71
OsR498G0102384500.01	1.323	3.976	1.588381	5.40E-17	7.80E-17	1.18	1.48	1.31	4.19	3.59	4.15
OsR498G0102385500.01	0.603	15.756	4.685313	1.01E-196	4.17E-197	0.48	0.45	0.88	15.12	15.71	16.44
OsR498G0102387800.01	9.813	3.49	-1.43728	4.22E-69	3.19E-69	9.8	9.81	9.83	4.05	3.18	3.24
OsR498G0102387900.01	0.95	20.893	4.462292	1.16E-261	3.93E-262	0.78	1.26	0.81	21.65	20.65	20.38
OsR498G0102390500.01	5.58	64.11	3.50972	0	0	5.85	5.32	5.57	64.02	64.01	64.3
OsR498G0102391600.01	0.936	544.04	9.163991	0	0	1.08	0.69	1.04	575	529.24	527.87
OsR498G0102391800.01	5.823	1.473	-1.97306	9.30E-105	5.58E-105	5.32	6.05	6.1	1.6	1.37	1.45
OsR498G0102395000.01	6.363	17.866	1.480625	9.56E-100	5.90E-100	6.34	6.42	6.33	17.38	18.65	17.57
OsR498G0102395800.01	1.26	3.933	1.633768	1.40E-27	1.64E-27	1.11	1.12	1.55	4.42	3.86	3.52
OsR498G0102399300.01	0.02	0.476	4.361688	3.01E-06	6.15E-06	0	0	0.06	0.39	0.42	0.62
OsR498G0102400500.01	10.02	52.453	2.381628	2.06E-260	7.02E-261	9.62	11.08	9.36	57.8	48.54	51.02
OsR498G0102404000.01	3.943	27.07	2.847564	0	0	3.9	4.32	3.61	26.76	27.13	27.32
OsR498G0102404900.01	6.82	13.743	1.008416	1.17E-60	9.53E-61	6.63	7	6.83	13.27	14.06	13.9
OsR498G0102406000.01	1.353	6.263	2.276137	1.14E-40	1.13E-40	1.03	1.44	1.59	5.39	5.33	8.07
OsR498G0102408600.01	0.19	0.796	2.087513	5.72E-26	6.89E-26	0.12	0.25	0.2	0.82	0.86	0.71
OsR498G0102411000.01	0.03	0.116	1.847115	0.000202	0.0004608	0.02	0.05	0.02	0.06	0.12	0.17
OsR498G0102411200.01	0.326	10.563	5.024653	2.88E-224	1.09E-224	0.23	0.47	0.28	10.27	11.11	10.31
OsR498G0102412100.01	26.286	85.756	1.693281	0	0	27.96	26.73	24.17	85.14	88.47	83.66
OsR498G0102414800.01	7.483	3.393	-1.14529	7.89E-50	7.09E-50	6.32	7.8	8.33	3.18	3.81	3.19
OsR498G0102415600.01	7.386	2.39	-1.61608	5.29E-82	3.63E-82	6.61	8	7.55	2.84	2.02	2.31
OsR498G0102416600.01	0.57	2.13	1.865731	1.49E-05	3.16E-05	0.62	0.49	0.6	2.11	1.98	2.3
OsR498G0102416900.01	620.36	230.57	-1.42888	0	0	622.77	624.23	614.1	233.52	227.85	230.34
OsR498G0102418100.01	10.563	0.893	-3.67508	8.38E-237	3.06E-237	10.13	10.58	10.98	1.08	0.86	0.74
OsR498G0102421700.01	0.546	0.213	-1.41967	2.84E-05	6.14E-05	0.61	0.61	0.42	0.28	0.13	0.23
OsR498G0102423600.01	9.773	3.666	-1.41624	6.48E-75	4.68E-75	9.44	9.83	10.05	4.21	3.22	3.57
OsR498G0102428000.01	36.88	17.013	-1.16124	8.86E-203	3.58E-203	38.24	35.91	36.49	18.18	16.33	16.53
OsR498G0102429100.01	4.283	40.733	3.239142	0	0	5.3	3.47	4.08	43.5	38.22	40.48
OsR498G0102431700.01	2.646	13.923	2.420443	1.79E-233	6.60E-234	2.63	2.91	2.4	14.27	14.57	12.93
OsR498G0102435700.01	0.85	0.286	-1.5452	5.16E-05	0.0001132	0.62	1.29	0.64	0.34	0.23	0.29
OsR498G0102436100.01	0.08	0.01	-3.5452	0.0005186	0.0012198	0.1	0.08	0.06	0.03	0	0
OsR498G0102437500.01	1.133	0.116	-3.44853	1.26E-20	1.68E-20	1.42	0.76	1.22	0.09	0.14	0.12
OsR498G0102439000.01	6.036	29.586	2.277078	0	0	6.59	6.02	5.5	28.44	29.84	30.48
OsR498G0102444800.01	0.516	0	-5.91444	4.87E-09	8.81E-09	0.46	0.63	0.46	0	0	0
OsR498G0102446100.01	32.916	7.823	-2.07079	0	0	32.93	33.71	32.11	7.8	7.38	8.29
OsR498G0102446400.01	1.433	14.926	3.36948	2.75E-252	9.61E-253	0.99	1.41	1.9	16.43	14.73	13.62
OsR498G0102447200.01	2.696	6.69	1.308577	1.79E-36	1.86E-36	2.46	2.78	2.85	7.51	6.37	6.19
OsR498G0102447400.01	2.303	21.8	3.242852	3.00E-288	9.52E-289	1.95	2.8	2.16	22.95	21.6	20.85
OsR498G0102448800.01	1.613	3.47	1.093471	6.22E-15	9.42E-15	1.82	1.55	1.47	3.05	3.63	3.73
OsR498G0102459900.01	1.926	0.363	-2.40955	2.52E-20	3.38E-20	1.91	1.87	2	0.33	0.38	0.38
OsR498G0102460200.01	23.416	5.2	-2.26656	1.08E-245	3.84E-246	23.41	23.1	23.74	5.27	4.93	5.4
OsR498G0102460500.01	0	0.746	5.432077	8.26E-07	1.64E-06	0	0	0	0.11	1.4	0.73
OsR498G0102462100.01	8.403	32.973	1.954479	0	0	8.29	7.75	9.17	31.84	33.58	33.5
OsR498G0102462400.01	107.76	40.53	-1.41859	0	0	111.93	109.02	102.3	43.64	38.71	39.24
OsR498G0102467600.01	73.953	731	3.300807	0	0	72.83	74.58	74.45	749.55	743.55	699.89
OsR498G0102469100.01	0.73	4.683	2.651952	1.54E-101	9.40E-102	0.52	0.87	0.8	4.65	4.66	4.74
OsR498G0102470900.01	1.35	0.5	-1.44023	3.22E-05	6.99E-05	1.29	1.13	1.63	0.63	0.32	0.55
OsR498G0102472000.01	0.28	0.13	-1.44567	0.0001697	0.0003852	0.4	0.13	0.31	0.08	0.17	0.14
OsR498G0102473900.01	0.08	1.02	3.673966	1.03E-31	1.13E-31	0.1	0.06	0.08	0.97	1.02	1.07
OsR498G0102478700.01	22.44	6.266	-1.85117	1.24E-163	5.75E-164	22.15	21.82	23.35	6.19	5.94	6.67
OsR498G0102485500.01	0.25	2.42	3.252754	2.07E-20	2.77E-20	0.27	0.22	0.26	2.03	2.52	2.71

OsR498G0102486200.01	0.17	5.61	4.984618	6.49E-38	6.62E-38	0.21	0	0.3	4.74	6.17	5.92
OsR498G0102488600.01	17.43	7.336	-1.34622	7.11E-95	4.50E-95	18.12	17.56	16.61	7.42	7.82	6.77
OsR498G0102488800.01	0.383	0.026	-3.86713	5.54E-14	8.61E-14	0.25	0.5	0.4	0.04	0.02	0.02
OsR498G0102489400.01	3.603	1.53	-1.214	5.15E-08	9.70E-08	3.65	3.45	3.71	2	0.8	1.79
OsR498G0102489700.01	0.166	0.63	1.949505	2.08E-10	3.58E-10	0.11	0.23	0.16	0.65	0.68	0.56
OsR498G0102490000.01	0.083	0.446	2.428628	1.37E-06	2.74E-06	0.12	0	0.13	0.52	0.2	0.62
OsR498G0102492500.01	4.893	20.676	2.080402	3.35E-146	1.66E-146	6.06	3.93	4.69	21.55	20.52	19.96
OsR498G0102492800.01	2.603	10.946	2.050022	5.12E-87	3.40E-87	2.46	2.74	2.61	10.76	11.39	10.69
OsR498G0102495900.01	1.026	0.1	-3.37528	1.17E-07	2.24E-07	1.34	1	0.74	0.1	0	0.2
OsR498G0102496500.01	2.5	0.333	-2.8111	6.05E-33	6.56E-33	2.97	2.2	2.33	0.16	0.41	0.43
OsR498G0102502800.01	1.87	0.13	-3.56475	3.89E-21	5.13E-21	1.24	2.52	1.85	0.04	0.24	0.11
OsR498G0102503400.01	79.92	16.953	-2.27085	0	0	79.55	81.36	78.85	17.72	15.6	17.54
OsR498G0102503900.01	1.086	3.306	1.589957	3.67E-08	6.87E-08	1.39	0.98	0.89	2.99	2.56	4.37
OsR498G0102505400.01	0	0.303	5.746291	4.58E-08	8.61E-08	0	0	0	0.64	0.27	0
OsR498G0102505500.01	0.356	1.03	1.496859	6.06E-06	1.26E-05	0.25	0.24	0.58	0.54	1.44	1.11
OsR498G0102506100.01	0.26	0.623	1.278547	2.14E-08	3.97E-08	0.22	0.26	0.3	0.68	0.63	0.56
OsR498G0102506400.01	193.51	61.123	-1.70148	0	0	194.65	195.11	190.8	61.93	60.44	61
OsR498G0102508000.01	20.53	59.98	1.546045	0	0	21.74	19.93	19.92	61.26	60.52	58.16
OsR498G0102508400.01	3.466	0.993	-1.84288	1.05E-23	1.32E-23	3.23	3.65	3.52	0.83	0.98	1.17
OsR498G0102509100.01	30.51	10.07	-1.61492	1.04E-232	3.84E-233	29.37	30.19	31.97	9.34	10.96	9.91
OsR498G0102513000.01	14.91	42.97	1.520666	0	0	14.38	15.42	14.93	41.16	43.8	43.95
OsR498G0102513500.01	2.563	1.136	-1.18937	9.62E-09	1.76E-08	2.38	2.59	2.72	0.89	1.32	1.2
OsR498G0102513700.01	0.96	0.44	-1.16535	1.14E-05	2.41E-05	0.87	0.91	1.1	0.63	0.4	0.29
OsR498G0102514700.01	135.89	23.17	-2.55743	0	0	137.5	136	134.2	24.8	22.42	22.29
OsR498G0102514900.01	9.3	22.786	1.284718	1.11E-137	5.71E-138	10.3	8.82	8.78	22.37	22.84	23.15
OsR498G0102515200.01	11.47	4.41	-1.3879	3.04E-65	2.37E-65	11.34	11.33	11.74	4.03	4.7	4.5
OsR498G0102515800.01	10.816	2.31	-2.24773	1.67E-115	9.49E-116	11.07	10.65	10.73	1.79	2.86	2.28
OsR498G0102520500.01	2.37	5.653	1.258248	1.43E-48	1.31E-48	2.69	2.36	2.06	4.88	5.81	6.27
OsR498G0102525600.01	28.446	12.013	-1.26145	2.61E-122	1.43E-122	30.2	28.12	27.02	11.22	12.57	12.25
OsR498G0102526000.01	1.736	0.44	-1.99537	8.54E-32	9.40E-32	1.57	1.96	1.68	0.38	0.29	0.65
OsR498G0102527000.01	5.063	1.696	-1.56876	2.89E-38	2.93E-38	4.72	5.62	4.85	1.54	1.72	1.83
OsR498G0102527100.01	0.48	0.19	-1.39634	0.0001312	0.0002954	0.33	0.46	0.65	0.17	0.12	0.28
OsR498G0102527700.01	9.44	20.876	1.140804	2.25E-103	1.36E-103	9.29	9.65	9.38	20.75	20.01	21.87
OsR498G0102527900.01	0.296	0.046	-2.76759	4.11E-07	8.06E-07	0.28	0.37	0.24	0.05	0.03	0.06
OsR498G0102528100.01	3.256	9.186	1.538622	2.25E-84	1.52E-84	3.55	3.2	3.02	8.7	10	8.86
OsR498G0102528400.01	9.21	20.613	1.136585	5.58E-41	5.50E-41	10.88	7.67	9.08	20.28	22.23	19.33
OsR498G0102528600.01	7.206	24.36	1.769192	8.13E-259	2.79E-259	7.29	7.31	7.02	22.96	25.54	24.58
OsR498G0102529000.01	1.666	0.12	-3.91315	1.42E-97	8.86E-98	1.71	1.7	1.59	0.12	0.09	0.15
OsR498G0102529300.01	4.536	1.316	-1.7906	2.02E-52	1.77E-52	4.24	4.4	4.97	1.16	1.49	1.3
OsR498G0102529600.01	2.2	15.213	2.786034	7.25E-237	2.65E-237	2.61	2	1.99	15.7	14.48	15.46
OsR498G0102530100.01	0.683	0.063	-3.46803	7.57E-19	1.05E-18	0.86	0.47	0.72	0.05	0.07	0.07
OsR498G0102533100.01	14.103	4.803	-1.61179	2.75E-61	2.22E-61	15.22	13.43	13.66	4.45	5.19	4.77
OsR498G0102533800.01	1.12	0.5	-1.1674	3.34E-09	6.01E-09	1.25	0.76	1.35	0.52	0.48	0.5
OsR498G0102534400.01	17.83	7.136	-1.29957	3.52E-108	2.07E-108	17.01	18.37	18.11	7.36	7.34	6.71
OsR498G0102535400.01	0.07	0	-4.41967	0.0003895	0.0009082	0.04	0.1	0.07	0	0	0
OsR498G0102535900.01	0.453	3.396	2.896725	1.09E-59	8.96E-60	0.47	0.41	0.48	3.24	3.41	3.54
OsR498G0102536100.01	0.096	1.19	3.376043	8.06E-21	1.07E-20	0.09	0.03	0.17	0.97	1.39	1.21
OsR498G0102537700.01	47.236	19.506	-1.28783	4.05E-197	1.67E-197	45.14	50.32	46.25	19.85	18.33	20.34
OsR498G0102539200.01	3.043	25.91	3.054091	0	0	3.11	3.12	2.9	26.3	24.78	26.65
OsR498G0102547300.01	13.813	36.033	1.394333	6.20E-165	2.85E-165	14.14	13.02	14.28	38.43	34.61	35.06
OsR498G0102548500.01	0.03	0.246	3.098654	3.81E-06	7.84E-06	0.03	0.03	0.03	0.23	0.27	0.24
OsR498G0102548800.01	4.746	109.15	4.522166	0	0	4.82	4.69	4.73	105.76	111.09	110.59
OsR498G0102550900.01	5.933	2.376	-1.32863	2.39E-26	2.86E-26	5.97	6.37	5.46	2.26	2.31	2.56
OsR498G0102552000.01	0.153	0	-4.5452	0.0002138	0.0004889	0.07	0.17	0.22	0	0	0
OsR498G0102553700.01	3.11	7.233	1.203879	2.85E-11	4.78E-11	3	3.18	3.15	6.61	6.92	8.17
OsR498G0102553900.01	47.063	12.513	-1.91247	0	0	42.53	48.48	50.18	12.83	12.41	12.3
OsR498G0102554600.01	0.453	4.41	3.262152	2.81E-22	3.62E-22	0.45	0.36	0.55	4.32	5.24	3.67
OsR498G0102554800.01	18406	7055	-1.40109	0	0	18193	19385	17641	7551.7	6548	7065.3
OsR498G0102560100.01	649.41	120.48	-2.44242	0	0	662.61	647.41	638.2	124.09	115.27	122.09
OsR498G0102561600.01	1.453	0.693	-1.08285	2.55E-15	3.82E-15	1.59	1.09	1.68	0.8	0.52	0.76
OsR498G0102562000.01	1.643	0.616	-1.42419	6.41E-07	1.27E-06	1.71	1.61	1.61	0.88	0.46	0.51
OsR498G0102563800.01	0.1	1.163	3.683616	6.81E-12	1.12E-11	0.07	0.14	0.09	1.1	1.41	0.98
OsR498G0102565000.01	0.053	0.453	3.498754	5.54E-21	7.34E-21	0	0.07	0.09	0.41	0.52	0.43
OsR498G0102565100.01	0.043	0.486	4.127223	1.87E-05	4.00E-05	0	0	0.13	0.52	0.42	0.52
OsR498G0102571400.01	0.29	6.303	4.306547	7.56E-87	5.04E-87	0.25	0.36	0.26	6.54	5.29	7.08
OsR498G0102571600.01	0	0.816	6.993956	4.08E-16	6.02E-16	0	0	0	0.79	0.83	0.83
OsR498G0102572700.01	74.4	199.48	1.416668	0	0	75.98	74.63	72.59	203.56	197.34	197.53
OsR498G0102573500.01	2.416	0.643	-1.9163	2.60E-11	4.36E-11	2.53	2.35	2.37	0.53	0.78	0.62
OsR498G0102575500.01	5.053	0.606	-3.0477	1.16E-103	7.01E-104	5.58	5.3	4.28	0.67	0.57	0.58

OsR498G0102575800.01	1.306	0.216	-2.67296	1.85E-10	3.19E-10	1.46	1.19	1.27	0.27	0.17	0.21
OsR498G0102576200.01	45.936	16.843	-1.48053	0	0	46.74	46.73	44.34	16.77	16.81	16.95
OsR498G0102576500.01	3.613	0.98	-1.88003	8.04E-90	5.26E-90	3.19	3.86	3.79	0.99	0.99	0.96
OsR498G0102584400.01	44.343	91.506	1.043702	0	0	42.81	45.62	44.6	91.3	90.79	92.43
OsR498G0102585300.01	14.806	43.803	1.57375	8.02E-209	3.19E-209	14.97	15.62	13.83	45.58	45.86	39.97
OsR498G0102586900.01	60.13	17.11	-1.75642	0	0	59.68	60.42	60.29	17.36	17.76	16.21
OsR498G0102588000.01	182.96	71.6	-1.35707	0	0	183.42	182.4	183.1	74.14	70.27	70.39
OsR498G0102589900.01	32.226	8.863	-1.88754	0	0	32.55	32.04	32.09	8.85	8.38	9.36
OsR498G0102591900.01	0.05	0.39	2.914229	2.18E-05	4.67E-05	0.05	0.05	0.05	0.31	0.22	0.64
OsR498G0102592100.01	0.6	0.036	-4.1032	5.78E-13	9.22E-13	0.81	0.43	0.56	0	0	0.11
OsR498G0102593100.01	3.993	12	1.582362	2.33E-130	1.24E-130	3.92	3.89	4.17	11.6	12.41	11.99
OsR498G0102593500.01	51.276	23.913	-1.10772	6.02E-133	3.15E-133	50.65	53.86	49.32	24.07	24.27	23.4
OsR498G0102596300.01	2.483	0.896	-1.49458	1.38E-07	2.65E-07	2.4	1.96	3.09	0.73	0.66	1.3
OsR498G0102597800.01	7.58	17.1	1.215012	9.43E-103	5.74E-103	7.57	7.57	7.6	17.99	15.58	17.73
OsR498G0102605100.01	1.176	5.693	2.272421	2.72E-25	3.31E-25	1.14	1.29	1.1	7.18	4.51	5.39
OsR498G0102610500.01	0.033	0.133	2.03976	0.0006606	0.0015689	0.04	0.04	0.02	0.14	0.12	0.14
OsR498G0102613700.01	44.353	15.586	-1.52056	0	0	43.04	44.38	45.64	15.33	15.87	15.56
OsR498G0102617400.01	1.403	5.366	1.910862	3.36E-87	2.23E-87	1.28	1.37	1.56	5.73	5.13	5.24
OsR498G0102619200.01	17.163	209.54	3.606381	0	0	17.86	16.62	17.01	208.91	209.83	209.88
OsR498G0102620800.01	69.603	17.226	-2.01771	0	0	67.05	72.02	69.74	18.07	16.23	17.38
OsR498G0102622000.01	3.506	0.173	-4.36963	2.04E-75	1.47E-75	3.21	3.53	3.78	0.06	0.23	0.23
OsR498G0102622400.01	1.933	5.086	1.328242	1.48E-29	1.69E-29	1.84	1.89	2.07	4.61	5.46	5.19
OsR498G0102622700.01	56.436	241.8	2.091394	0	0	56.1	56.35	56.86	251.31	235.5	238.6
OsR498G0102624400.01	1.47	4.923	1.943084	1.69E-22	2.17E-22	1.3	1.36	1.75	4.83	4.76	5.18
OsR498G0102627600.01	9.003	1.53	-2.5673	1.98E-139	1.01E-139	9.74	8.72	8.55	1.37	1.4	1.82
OsR498G0102627800.01	17.303	5.19	-1.77167	7.06E-126	3.81E-126	16.8	17.65	17.46	5.05	4.96	5.56
OsR498G0102632900.01	40.16	17.323	-1.21658	0	0	40.91	40.96	38.61	16.83	17.45	17.69
OsR498G0102633200.01	2.143	0.433	-2.55725	8.03E-53	6.99E-53	2.26	2.07	2.1	0.19	0.66	0.45
OsR498G0102635300.01	21.673	7.56	-1.56181	2.77E-91	1.79E-91	21.53	22.32	21.17	7.29	6.81	8.58
OsR498G0102635500.01	53.063	178.41	1.754808	0	0	49.23	55.47	54.49	177.9	179.78	177.56
OsR498G0102636800.01	0.176	1.026	2.563322	9.59E-12	1.59E-11	0.16	0.17	0.2	1.01	1.04	1.03
OsR498G0102636900.01	9.186	24.286	1.403165	2.37E-223	8.99E-224	8.99	10.14	8.43	24.44	24.39	24.03
OsR498G0102639100.01	4.81	32.453	2.710955	1.83E-46	1.71E-46	4.67	4.65	5.11	35.38	31.36	30.62
OsR498G0102646700.01	2.183	16.54	2.930531	1.96E-104	1.18E-104	1.67	2.59	2.29	17.01	16.66	15.95
OsR498G0102647600.01	21.433	10.75	-1.00077	2.13E-66	1.65E-66	21.11	21.96	21.23	10.74	11.24	10.27
OsR498G0102648200.01	5.466	12.236	1.15733	1.13E-34	1.20E-34	5.03	5.87	5.5	13.34	12.58	10.79
OsR498G0102648800.01	11.263	33.856	1.581234	1.01E-256	3.47E-257	10.76	11.48	11.55	34.29	34.39	32.89
OsR498G0102649000.01	10.723	106.98	3.318872	0	0	11.33	10.54	10.3	116.84	102.88	101.23
OsR498G0102649700.01	16.69	72.4	2.101212	0	0	14.59	18.93	16.55	71.36	72.6	73.24
OsR498G0102653900.01	3.043	0.83	-1.89987	2.00E-24	2.47E-24	3.1	3.09	2.94	1.13	0.61	0.75
OsR498G0102654200.01	5.576	0.793	-2.81034	5.12E-65	4.01E-65	4.9	6.32	5.51	0.86	0.38	1.14
OsR498G0102655100.01	31.123	90.646	1.5369	0	0	30.68	31.34	31.35	90.91	91.55	89.48
OsR498G0102657500.01	13.626	59.55	2.119691	0	0	14.71	12.79	13.38	56.2	62.05	60.4
OsR498G0102662000.01	0.093	0.03	-1.72577	0.0001248	0.0002809	0.14	0.03	0.11	0.04	0.02	0.03
OsR498G0102663000.01	2.506	1.176	-1.03157	2.59E-31	2.88E-31	2.01	2.56	2.95	0.98	1.36	1.19
OsR498G0102666300.01	1.363	0.53	-1.36832	3.84E-10	6.70E-10	1.47	1.48	1.14	0.54	0.31	0.74
OsR498G0102666800.01	1.823	0.463	-1.9669	8.91E-24	1.12E-23	1.56	1.95	1.96	0.61	0.42	0.36
OsR498G0102667700.01	0.023	0.286	3.55023	2.53E-13	4.00E-13	0.03	0.03	0.01	0.31	0.34	0.21
OsR498G0102668900.01	42.92	15.9	-1.46099	3.66E-211	1.44E-211	45.84	41.34	41.58	14.94	16.05	16.71
OsR498G0102672000.01	1.386	6.066	1.9687	7.04E-73	5.17E-73	1.02	1.23	1.91	6.58	5.07	6.55
OsR498G0102673700.01	21.41	4.426	-2.28711	6.57E-109	3.85E-109	23.17	20.22	20.84	3.88	5.37	4.03
OsR498G0102674100.01	11.183	1.83	-2.67696	7.00E-119	3.92E-119	9.95	11.73	11.87	1.59	1.82	2.08
OsR498G0102674300.01	0.856	2.443	1.499192	8.90E-13	1.43E-12	0.78	0.72	1.07	2.62	2.13	2.58
OsR498G0102679000.01	0	0.313	5.683616	8.53E-08	1.62E-07	0	0	0	0.48	0.23	0.23
OsR498G0102685400.01	0.39	0.103	-1.90777	0.0002294	0.000526	0.3	0.45	0.42	0.04	0.18	0.09
OsR498G0102686000.01	2.223	36.733	4.042131	0	0	2.27	2.29	2.11	38.12	36.47	35.61
OsR498G0102686800.01	0.176	1.85	3.54226	5.34E-35	5.65E-35	0.12	0.26	0.15	2.02	1.44	2.09
OsR498G0102687000.01	11.623	1.283	-3.29288	9.36E-223	3.56E-223	11.23	12.08	11.56	1.09	1.43	1.33
OsR498G0102687600.01	0.526	39.816	6.267329	0	0	0.56	0.84	0.18	42.21	38.95	38.29
OsR498G0102688000.01	0.736	38.56	5.674037	0	0	0.56	0.84	0.81	40.68	37.18	37.82
OsR498G0102692900.01	3.16	10.666	1.77115	1.93E-102	1.17E-102	2.58	3.54	3.36	12.63	9.68	9.69
OsR498G0102694000.01	2.923	12.113	2.044772	2.83E-190	1.20E-190	2.98	2.97	2.82	12.06	12.07	12.21
OsR498G0102700100.01	14.816	164.59	3.464466	0	0	14.64	13.89	15.92	173.54	163.75	156.48
OsR498G0102700400.01	3.83	37.566	3.289232	1.02E-197	4.19E-198	6.8	3.73	0.96	28.43	44.28	39.99
OsR498G0102700800.01	87.44	340.59	1.953592	0	0	91.7	83.79	86.83	372.52	325.17	324.07
OsR498G0102701900.01	4.76	43.426	3.190241	0	0	4.55	5.22	4.51	41.68	45.32	43.28
OsR498G0102702100.01	0.096	1.006	3.361688	2.58E-09	4.62E-09	0.22	0	0.07	1.12	1.07	0.83
OsR498G0102702500.01	0.333	1.963	2.517807	8.11E-13	1.30E-12	0.43	0.15	0.42	1.41	2.59	1.89
OsR498G0102702900.01	1.116	10.623	3.244119	1.26E-78	8.88E-79	0.94	1.29	1.12	11.03	10.81	10.03

OsR498G0102704300.01	6.506	2.82	-1.10506	1.70E-42	1.65E-42	6.59	6.68	6.25	2.54	2.88	3.04
OsR498G0102704900.01	4.736	2.236	-1.08451	1.40E-23	1.76E-23	5	4.68	4.53	2.23	2.37	2.11
OsR498G0102705500.01	3.25	8.1	1.315592	1.24E-46	1.15E-46	2.84	3.55	3.36	8.17	7.86	8.27
OsR498G0102710200.01	8.186	0.72	-2.60933	3.11E-80	2.17E-80	8.53	8.36	7.67	0.74	0.66	0.76
OsR498G0102716500.01	20.653	71.043	1.803681	0	0	20.24	21.07	20.65	71.14	73.34	68.65
OsR498G0102717800.01	1.523	12.913	3.045054	6.27E-235	2.30E-235	1.55	1.48	1.54	13.92	12.47	12.35
OsR498G0102720600.01	1.513	0.286	-2.39688	3.78E-22	4.89E-22	1.4	1.36	1.78	0.32	0.18	0.36
OsR498G0102721000.01	0.943	384.08	8.666233	0	0	0.85	1.03	0.95	386.19	383.73	382.33
OsR498G0102722700.01	0.93	22.936	4.622467	2.34E-198	9.58E-199	0.77	1.13	0.89	24.68	20.99	23.14
OsR498G0102723000.01	0.046	1.953	5.47772	3.43E-12	5.60E-12	0	0.14	0	1.08	2.33	2.45
OsR498G0102723100.01	14.5	85.793	2.556553	0	0	13.6	15.46	14.44	86.36	85.28	85.74
OsR498G0102726000.01	0.2	1.946	3.270058	3.81E-24	4.74E-24	0.18	0.24	0.18	2.16	1.56	2.12
OsR498G0102726800.01	2.306	7.203	1.639518	9.65E-63	7.69E-63	2.35	2.49	2.08	7.9	6.81	6.9
OsR498G0102730100.01	0.046	0.893	4.259871	4.59E-43	4.43E-43	0.03	0.06	0.05	0.91	1.03	0.74
OsR498G0102730700.01	8.45	20.663	1.28638	4.90E-141	2.49E-141	7.44	8.94	8.97	20.91	20.71	20.37
OsR498G0102736100.01	27.26	86.436	1.660604	0	0	27.5	27.13	27.15	86.47	86.09	86.75
OsR498G0102736400.01	13.743	4.853	-1.52655	2.73E-71	2.03E-71	14.23	13.72	13.28	4.68	4.55	5.33
OsR498G0102736500.01	4.58	1.956	-1.20017	6.37E-35	6.74E-35	4.78	4.28	4.68	1.75	2.22	1.9
OsR498G0102737000.01	0.073	82.77	10.11924	0	0	0.11	0	0.11	85.04	77.49	85.78
OsR498G0102737100.01	0.133	30.273	7.837422	4.04E-193	1.69E-193	0	0.31	0.09	34.89	24.77	31.16
OsR498G0102738500.01	0.12	1.026	3.062128	3.08E-24	3.82E-24	0.09	0.14	0.13	1.07	1.01	1
OsR498G0102738900.01	16.996	285.54	4.037117	0	0	15.7	16.44	18.85	276.13	289.62	290.86
OsR498G0102739700.01	140.97	39.306	-1.85301	0	0	148.98	135.92	138	40.21	36.55	41.16
OsR498G0102739900.01	18.116	53.17	1.545833	1.94E-166	8.89E-167	17.74	19	17.61	57	50.3	52.21
OsR498G0102740300.01	0.913	10.94	3.584081	4.11E-249	1.45E-249	1.01	1	0.73	11.29	11.15	10.38
OsR498G0102740800.01	15.52	6.203	-1.35497	3.50E-192	1.47E-192	13.88	15.74	16.94	5.95	6.29	6.37
OsR498G0102742000.01	39.54	97.396	1.297227	0	0	39.54	40.15	38.93	96.16	100.71	95.32
OsR498G0102743100.01	7.453	34.566	2.219221	0	0	7.76	6.93	7.67	33.72	33.45	36.53
OsR498G0102744500.01	0.09	0.936	3.361688	2.58E-09	4.62E-09	0.07	0	0.2	0.9	1.35	0.56
OsR498G0102745600.01	2.15	0.996	-1.11419	1.30E-12	2.10E-12	1.85	2.21	2.39	0.98	0.74	1.27
OsR498G0102746100.01	0.566	3.136	2.465244	1.54E-33	1.65E-33	0.43	0.66	0.61	3.32	3.39	2.7
OsR498G0102747100.01	0.743	1.786	1.27054	3.65E-08	6.83E-08	0.72	0.9	0.61	2.34	1.28	1.74
OsR498G0102749300.01	463.11	150.12	-1.63035	0	0	455.12	473.76	460.5	158.59	147.88	143.88
OsR498G0102750100.01	3.143	1.146	-2.10059	1.55E-57	1.29E-57	3.01	3.01	3.41	1.27	1.35	0.82
OsR498G0102754100.01	101.1	29.896	-1.7659	0	0	103.9	100.19	99.22	30.03	28.13	31.53
OsR498G0102755800.01	83.666	438.05	2.357864	0	0	86.61	81.12	83.27	457.13	427.97	429.04
OsR498G0102756700.01	19.86	50.15	1.336742	0	0	18.08	20.92	20.58	46.89	51.59	51.97
OsR498G0102757000.01	6.32	2.206	-1.53343	2.86E-19	3.92E-19	5.67	5.56	7.73	2.79	2.13	1.7
OsR498G0102771100.01	8.32	21.85	1.382397	1.16E-54	9.89E-55	9.13	7.95	7.88	22.01	19.94	23.6
OsR498G0102772400.01	1.756	0.823	-1.08024	1.15E-10	1.96E-10	1.72	1.6	1.95	0.91	0.66	0.9
OsR498G0102773200.01	60.34	268.86	1.943186	0	0	60.21	60.65	60.16	264.88	266.43	275.28
OsR498G0102773700.01	0.013	0.18	3.7402	0.0002182	0.0004994	0	0	0.04	0.08	0.21	0.25
OsR498G0102774600.01	0.02	0.196	2.946651	0.0003132	0.0007244	0.03	0	0.03	0.09	0.21	0.29
OsR498G0102775200.01	1.396	0.406	-1.80824	3.32E-26	3.98E-26	1.46	1.34	1.39	0.27	0.59	0.36
OsR498G0102775500.01	0.55	8.166	3.878664	1.25E-126	6.73E-127	0.55	0.45	0.65	9.82	7.42	7.26
OsR498G0102776000.01	0.563	3.666	2.690996	7.11E-39	7.18E-39	0.43	0.59	0.67	3.97	3.65	3.38
OsR498G0102778900.01	0.01	0.19	4.287688	5.52E-06	1.15E-05	0.03	0	0	0.21	0.15	0.21
OsR498G0102779100.01	0.46	0.176	-1.41967	0.0007987	0.0019083	0.49	0.4	0.49	0.17	0.24	0.12
OsR498G0102779300.01	46.42	0.08	-9.08866	0	0	47.36	46.22	45.68	0.16	0	0.08
OsR498G0102779600.01	0.686	0.203	-1.69721	6.43E-06	1.34E-05	0.54	0.98	0.54	0.32	0.08	0.21
OsR498G0102780000.01	26.876	63.293	1.229612	4.98E-256	1.72E-256	27.12	26	27.51	63.29	63.18	63.41
OsR498G0102780200.01	0.913	0.39	-1.214	1.92E-06	3.88E-06	0.91	1.04	0.79	0.5	0.37	0.3
OsR498G0102781300.01	234.5	5.996	-5.30795	0	0	249.12	228.67	225.7	6.02	6.31	5.66
OsR498G0102782800.01	1.586	0.443	-1.94006	6.04E-16	8.93E-16	1.65	1.27	1.84	0.36	0.36	0.61
OsR498G0102782900.01	4.576	0.78	-2.40502	6.21E-21	8.23E-21	5.17	4.7	3.86	0.5	0.67	1.17
OsR498G0102783200.01	11.736	38.136	1.608114	2.65E-279	8.56E-280	11.46	12.69	11.06	38.07	36.47	39.87
OsR498G0102784200.01	0.253	16.97	6.100172	3.32E-303	1.01E-303	0.16	0.49	0.11	18.33	17.66	14.92
OsR498G0102785500.01	34.406	14.656	-1.24967	9.68E-112	5.61E-112	31.81	33.44	37.97	14.32	14.09	15.56
OsR498G0102787800.01	361.02	15.523	-4.54314	0	0	375.33	366.2	341.5	16.16	14.77	15.64
OsR498G0102789100.01	0.606	1.493	1.530086	4.09E-19	5.63E-19	0.73	0.43	0.66	1.44	1.3	1.74
OsR498G0102790800.01	3.343	0.633	-2.41358	2.46E-109	1.44E-109	3.36	3.22	3.45	0.59	0.64	0.67
OsR498G0102791900.01	0.05	0.34	2.624723	5.29E-08	9.97E-08	0.02	0.02	0.11	0.38	0.37	0.27
OsR498G0102795800.01	3.99	0.616	-2.68482	8.75E-38	8.94E-38	3.31	4.96	3.7	0.8	0.65	0.4
OsR498G0202798700.01	5.596	1.676	-1.75079	6.13E-24	7.67E-24	5.56	5.29	5.94	1.67	1.65	1.71
OsR498G0202799200.01	1.32	3.383	1.347333	5.85E-17	8.45E-17	1.41	1.07	1.48	3.55	2.97	3.63
OsR498G0202803900.01	23.37	2.73	-3.13611	0	0	20.16	23.8	26.15	3.1	2.62	2.47
OsR498G0202804500.01	1.273	5.65	2.133901	3.40E-28	3.94E-28	1.38	0.92	1.52	5.71	5.19	6.05
OsR498G0202805700.01	3.536	1.546	-1.23284	3.46E-20	4.66E-20	3.09	3.97	3.55	1.42	2.09	1.13
OsR498G0202806100.01	16.29	2.826	-2.53068	6.47E-300	1.99E-300	16.16	15.81	16.9	2.59	2.94	2.95

OsR498G0202807200.01	0.086	0.28	1.683616	0.0008008	0.0019134	0.1	0.1	0.06	0.3	0.17	0.37
OsR498G0202807400.01	7.24	3.533	-1.0438	9.15E-17	1.33E-16	6.13	7.8	7.79	4.01	4.35	2.24
OsR498G0202810200.01	0.313	0.11	-4.0477	2.60E-05	5.61E-05	0.44	0.1	0.4	0	0	0.33
OsR498G0202812300.01	20.38	8.123	-1.35417	2.11E-127	1.13E-127	19.99	20.13	21.02	7.34	8.65	8.38
OsR498G0202817500.01	4.886	24.866	2.341838	0	0	4.8	4.5	5.36	25.93	24.99	23.68
OsR498G0202817800.01	0.07	0.466	2.789782	1.78E-13	2.79E-13	0.09	0.08	0.04	0.56	0.42	0.42
OsR498G0202818900.01	4.006	1.936	-1.01633	1.47E-19	2.01E-19	3.6	4.41	4.01	2.03	1.89	1.89
OsR498G0202819600.01	0.97	0.053	-4.18906	9.04E-18	1.28E-17	0.89	1.01	1.01	0.04	0	0.12
OsR498G0202820900.01	1.043	0.31	-1.8912	1.53E-09	2.73E-09	0.79	1.18	1.16	0.34	0.34	0.25
OsR498G0202827500.01	0.383	0.08	-2.3932	2.95E-05	6.38E-05	0.69	0.12	0.34	0	0.2	0.04
OsR498G0202832500.01	2.866	0.35	-3.053	2.12E-47	1.95E-47	2.79	2.93	2.88	0.32	0.4	0.33
OsR498G0202833400.01	0.776	0.06	-3.78467	8.58E-19	1.19E-18	0.55	1.01	0.77	0.06	0.03	0.09
OsR498G0202835200.01	0.756	2.62	1.811548	5.95E-27	7.06E-27	0.82	0.53	0.92	2.94	2.31	2.61
OsR498G0202842400.01	3.213	14.163	2.139939	7.66E-150	3.74E-150	3.24	3.15	3.25	14.06	14.15	14.28
OsR498G0202842600.01	4.896	10.146	1.043417	1.25E-33	1.34E-33	5.66	4.06	4.97	11.89	9.21	9.34
OsR498G0202844500.01	1.406	9.466	2.721584	2.00E-72	1.47E-72	1.12	0.99	2.11	9.3	11.27	7.83
OsR498G0202849700.01	0.786	0.006	-6.57495	2.96E-24	3.68E-24	0.76	1.05	0.55	0	0.02	0
OsR498G0202852400.01	0.536	0.133	-1.95802	4.93E-07	9.71E-07	0.57	0.64	0.4	0.12	0.03	0.25
OsR498G0202852500.01	40.256	19.436	-1.09034	5.41E-106	3.23E-106	40.72	38.59	41.46	17.8	19.41	21.1
OsR498G0202853000.01	1.626	0.643	-1.32124	2.66E-09	4.77E-09	1.83	1.53	1.52	0.59	0.5	0.84
OsR498G0202854300.01	16.083	122.02	2.918441	0	0	15.12	16.53	16.6	119.65	123.41	123.01
OsR498G0202854500.01	3.093	1.116	-1.40151	2.50E-20	3.36E-20	2.47	3.03	3.78	1.17	0.99	1.19
OsR498G0202856500.01	2.436	0.856	-1.9162	6.06E-62	4.86E-62	1.91	2.87	2.53	0.85	0.79	0.93
OsR498G0202858200.01	10.57	5.266	-1.00867	1.34E-36	1.38E-36	11.81	9.87	10.03	4.9	5.79	5.11
OsR498G0202858300.01	1.19	0.57	-1.05335	6.78E-07	1.34E-06	1.27	1.42	0.88	0.7	0.35	0.66
OsR498G0202859900.01	0.363	17.766	5.610917	0	0	0.34	0.42	0.33	18.61	17.71	16.98
OsR498G0202862500.01	0.193	1.67	3.092871	2.76E-16	4.05E-16	0.12	0.24	0.22	1.73	1.77	1.51
OsR498G0202863200.01	0.266	0.09	-1.5452	0.0004052	0.0009466	0.24	0.18	0.38	0.07	0.15	0.05
OsR498G0202863500.01	501.09	180.8	-1.48339	0	0	515.04	497.22	491	182.34	177.36	182.71
OsR498G0202863800.01	1.69	0.343	-2.30507	1.56E-17	2.23E-17	1.44	1.43	2.2	0.49	0.29	0.25
OsR498G0202866500.01	2301.4	992.03	-1.2173	0	0	2252.2	2343.1	2309	1034.6	969.78	971.68
OsR498G0202867800.01	52.673	3.636	-3.84254	0	0	50.23	55.32	52.47	3.29	4.21	3.41
OsR498G0202870300.01	0.47	0.153	-1.70747	1.07E-05	2.25E-05	0.45	0.19	0.77	0.2	0.16	0.1
OsR498G0202871200.01	1.37	0.593	-1.28217	1.83E-12	2.97E-12	1.36	1.26	1.49	0.23	0.67	0.88
OsR498G0202871400.01	77.61	166.87	1.122085	0	0	82.14	73.65	77.04	177.73	161.71	161.17
OsR498G0202871600.01	26.15	79.393	1.594046	2.86E-232	1.06E-232	26.62	27.01	24.82	89.66	71.07	77.45
OsR498G0202872600.01	59.143	22.24	-1.49589	0	0	57.44	59.94	60.05	23.33	20.85	22.54
OsR498G0202872800.01	23.38	8.44	-1.48468	1.97E-155	9.41E-156	23.15	24.44	22.55	8.43	8.11	8.78
OsR498G0202874400.01	21.006	54.256	1.332542	5.16E-231	1.92E-231	21.4	19.8	21.82	54.46	53.13	55.18
OsR498G0202875100.01	4.683	11.913	1.329267	7.09E-28	8.27E-28	5.26	4.82	3.97	11.83	11.94	11.97
OsR498G0202876700.01	2.663	1.063	-1.32392	1.17E-16	1.71E-16	2.78	2.7	2.51	1.07	1.22	0.9
OsR498G0202877500.01	37.033	12.673	-1.55296	5.64E-137	2.91E-137	37.64	38.7	34.76	13.44	11.98	12.6
OsR498G0202878700.01	12.37	80.633	2.638148	0	0	12.36	11.87	12.88	75.83	80.89	85.18
OsR498G0202879100.01	0.016	0.396	4.7402	8.16E-08	1.55E-07	0	0.05	0	0.22	0.51	0.46
OsR498G0202882200.01	0.103	0.473	2.127223	1.13E-07	2.15E-07	0.13	0.08	0.1	0.49	0.51	0.42
OsR498G0202882700.01	9.506	263.64	4.778582	0	0	8.78	9.64	10.1	252.17	265.61	273.14
OsR498G0202882900.01	0.9	1.906	1.209685	3.04E-20	4.09E-20	0.85	1.09	0.76	1.7	1.81	2.21
OsR498G0202885300.01	1.123	2.29	1.027619	8.58E-16	1.27E-15	0.92	1.16	1.29	2.18	2.34	2.35
OsR498G0202886100.01	6.403	24.836	1.94548	5.09E-159	2.39E-159	7.04	5.97	6.2	26.85	22.49	25.17
OsR498G0202886300.01	9.963	2.45	-2.0427	5.02E-40	4.99E-40	10.94	8.46	10.49	1.87	2.88	2.6
OsR498G0202887600.01	7.23	2.61	-1.45537	1.24E-72	9.08E-73	7.31	7.23	7.15	2.8	2.26	2.77
OsR498G0202888400.01	2.483	1.046	-1.24712	2.20E-11	3.68E-11	2.84	2.22	2.39	0.81	1.17	1.16
OsR498G0202892300.01	3.643	0.443	-3.04688	4.00E-88	2.65E-88	3.66	3.78	3.49	0.45	0.19	0.69
OsR498G0202895000.01	69.64	0.9	-6.05353	0	0	71.77	66.57	70.58	0.85	1.04	0.81
OsR498G0202895300.01	3.656	7.956	1.126003	6.87E-49	6.23E-49	3.52	4.04	3.41	7.58	8.52	7.77
OsR498G0202897600.01	5.053	1.803	-1.50673	2.69E-17	3.86E-17	5.27	3.88	6.01	1.61	1.94	1.86
OsR498G0202901600.01	0.78	0.15	-2.37528	7.02E-06	1.47E-05	0.43	1.07	0.84	0.07	0.15	0.23
OsR498G0202902000.01	25.65	11.726	-1.08922	0	0	18.7	30.55	27.7	10.53	12.1	12.55
OsR498G0202902100.01	8.773	3.53	-1.34839	9.15E-38	9.35E-38	3.44	12.44	10.44	3.45	3.26	3.88
OsR498G0202905400.01	0.306	0.816	1.432077	3.45E-10	6.01E-10	0.33	0.37	0.22	0.84	0.8	0.81
OsR498G0202907000.01	93.266	30	-1.67262	0	0	83.57	96.53	99.7	30.56	29.33	30.11
OsR498G0202912200.01	0.38	0.88	1.193006	2.88E-08	5.37E-08	0.33	0.39	0.42	0.6	0.76	1.28
OsR498G0202912700.01	9.543	41.063	2.085403	0	0	9.53	9.67	9.43	41.1	41.93	40.16
OsR498G0202913200.01	1.03	17.54	4.089708	3.10E-197	1.28E-197	0.67	1.34	1.08	19.43	18.28	14.91
OsR498G0202914700.01	21.433	62.44	1.541597	0	0	19.41	22.13	22.76	61.19	64.57	61.56
OsR498G0202916400.01	13.73	48.303	1.809322	0	0	13.91	13.31	13.97	43.86	49.91	51.14
OsR498G0202917000.01	0.153	0.01	-4.0477	2.60E-05	5.61E-05	0.19	0.17	0.1	0.03	0	0
OsR498G0202917300.01	6.543	43.19	2.723787	0	0	6.3	6.4	6.93	44.11	41.61	43.85
OsR498G0202919500.01	4.933	16.023	1.693272	1.21E-74	8.78E-75	4.46	5.36	4.98	16	17.02	15.05

OsR498G0202920000.01	0.006	0.113	3.946651	6.37E-05	0.0001407	0	0	0.02	0.09	0.18	0.07
OsR498G0202925100.01	18.49	8.1	-1.22775	1.41E-93	8.97E-94	19.61	18.1	17.76	8.37	7.72	8.21
OsR498G0202927100.01	39.526	84.646	1.10355	2.51E-154	1.20E-154	42.86	37.71	38.01	83.85	80.44	89.65
OsR498G0202932700.01	0.74	4.693	2.624557	4.98E-53	4.33E-53	0.53	0.8	0.89	4.35	4.69	5.04
OsR498G0202935500.01	21.386	8.753	-1.29701	2.12E-114	1.21E-114	23.86	21.69	18.61	9.7	7.45	9.11
OsR498G0202937800.01	18.59	9.26	-1.10174	3.38E-88	2.23E-88	19.45	18.22	18.1	9.74	10.33	7.71
OsR498G0202938400.01	0.37	0	-6.0477	9.92E-10	1.75E-09	0.39	0.31	0.41	0	0	0
OsR498G0202938800.01	4.663	0.08	-6.35568	1.34E-214	5.23E-215	4.65	5.01	4.33	0.07	0.08	0.09
OsR498G0202940300.01	0.24	4.26	4.147054	2.84E-65	2.22E-65	0.21	0.31	0.2	4.28	4.43	4.07
OsR498G0202941400.01	3.46	8.516	1.306106	9.69E-106	5.79E-106	3.22	3.91	3.25	8.45	8.25	8.85
OsR498G0202947900.01	0.756	0.29	-1.38908	0.0006992	0.0016633	0.65	0.75	0.87	0.4	0.34	0.13
OsR498G0202957700.01	1.163	0.516	-1.18851	9.55E-06	2.01E-05	1.1	1.16	1.23	0.39	0.85	0.31
OsR498G0202961100.01	5.93	2.94	-1.05183	2.00E-24	2.47E-24	6.03	6.37	5.39	2.78	3.13	2.91
OsR498G0202963300.01	4.076	1.39	-1.58083	2.27E-29	2.59E-29	3.37	4.4	4.46	1.26	2.04	0.87
OsR498G0202966500.01	21.063	48.653	1.185798	8.76E-299	2.70E-299	20.83	21.13	21.23	47.18	50.98	47.8
OsR498G0202967300.01	20.983	47.95	1.190357	0	0	20.43	21.14	21.38	47.58	46.71	49.56
OsR498G0202968000.01	16.45	35.23	1.104004	6.97E-162	3.24E-162	17.69	15.86	15.8	34.4	37.87	33.42
OsR498G0202971900.01	38.943	9.473	-2.02905	0	0	37.42	39.29	40.12	9.9	10.12	8.4
OsR498G0202977600.01	0.133	0.01	-3.5452	2.35E-06	4.79E-06	0.15	0.14	0.11	0.02	0.01	0
OsR498G0202977900.01	101.19	206.87	1.029417	0	0	99.87	97.92	105.8	202.87	208.35	209.4
OsR498G0202980800.01	0.723	0	-6.89098	1.38E-15	2.06E-15	0.71	0.74	0.72	0	0	0
OsR498G0202983400.01	1.773	4.236	1.245495	3.01E-43	2.90E-43	1.64	1.83	1.85	3.88	4.11	4.72
OsR498G0202986300.01	0.133	0.343	1.683616	5.50E-06	1.14E-05	0.1	0.18	0.12	0.43	0.27	0.33
OsR498G0202988500.01	0.363	0.043	-3.09774	2.78E-09	4.99E-09	0.28	0.51	0.3	0.06	0.07	0
OsR498G0202989000.01	1.413	2.91	1.04248	3.40E-23	4.31E-23	1.26	1.58	1.4	2.8	2.83	3.1
OsR498G0202990800.01	1.3	8.566	2.720262	1.80E-237	6.55E-238	1.25	1.45	1.2	7.93	8.95	8.82
OsR498G0202991600.01	1.013	2.526	1.311062	3.22E-30	3.62E-30	0.9	1.03	1.11	2.39	2.63	2.56
OsR498G0202993300.01	1.373	0.513	-1.4252	2.13E-14	3.27E-14	1.46	1.45	1.21	0.49	0.51	0.54
OsR498G0202995100.01	17.15	6.36	-1.51728	7.89E-79	5.54E-79	17.77	16.62	17.06	7.19	5.55	6.34
OsR498G0202995400.01	10.153	1.26	-3.03088	6.90E-45	6.53E-45	11.37	10.46	8.63	0	1.32	2.46
OsR498G0202996700.01	15.64	7.506	-1.08591	5.52E-106	3.29E-106	15.86	15.26	15.8	7.43	7.49	7.6
OsR498G0202998200.01	2.37	21.686	3.188263	0	0	2.53	2.42	2.16	20.52	21.74	22.8
OsR498G0203000700.01	0.99	0.383	-1.32737	4.53E-12	7.43E-12	0.98	1.03	0.96	0.41	0.37	0.37
OsR498G0203000900.01	7.72	3.486	-1.19835	5.34E-63	4.24E-63	6.66	8.51	7.99	3.73	4.15	2.58
OsR498G0203001200.01	3.44	0.93	-1.92721	5.63E-37	5.80E-37	3.33	3.46	3.53	0.25	1.12	1.42
OsR498G0203001700.01	2.016	0.13	-3.77539	2.08E-28	2.40E-28	1.71	1.82	2.52	0	0.07	0.32
OsR498G0203003800.01	0.196	0.013	-4.28217	4.39E-06	9.05E-06	0.34	0.1	0.15	0	0	0.04
OsR498G0203003900.01	0.643	0.066	-3.28217	2.42E-05	5.21E-05	0.76	0.8	0.37	0.1	0.1	0
OsR498G0203004900.01	0.67	0.093	-2.82531	9.90E-10	1.75E-09	0.79	0.54	0.68	0.16	0.08	0.04
OsR498G0203005200.01	24.183	11.97	-1.04186	1.09E-60	8.80E-61	24.34	23.65	24.56	11.26	12.65	12
OsR498G0203007700.01	14.62	6.123	-1.26701	7.44E-113	4.29E-113	14.33	14.08	15.45	5.72	6.07	6.58
OsR498G0203008400.01	1.06	0.443	-1.2628	1.06E-09	1.87E-09	1.16	1.05	0.97	0.37	0.47	0.49
OsR498G0203008600.01	1.823	4.183	1.200017	1.77E-22	2.27E-22	1.69	2	1.78	4.32	4.07	4.16
OsR498G0203011500.01	0.07	0.273	1.946651	7.80E-05	0.000173	0.08	0	0.13	0.3	0.22	0.3
OsR498G0203012800.01	1266.5	340.12	-1.8966	0	0	1350.6	1228.1	1221	365.44	323.17	331.76
OsR498G0203013600.01	1.713	0.393	-2.13025	2.10E-20	2.82E-20	1.85	1.52	1.77	0.44	0.49	0.25
OsR498G0203014500.01	23.78	7.41	-1.69009	3.42E-112	1.98E-112	22.3	23.92	25.12	8	7.74	6.49
OsR498G0203014800.01	6.903	1.776	-1.95751	2.10E-29	2.39E-29	7.95	5.75	7.01	1.51	0.74	3.08
OsR498G0203015000.01	15.133	31.416	1.043559	9.34E-170	4.22E-170	14.78	15.69	14.93	32.04	31.95	30.26
OsR498G0203015300.01	0.036	0.206	2.54226	1.24E-06	2.49E-06	0.07	0.02	0.02	0.16	0.15	0.31
OsR498G0203016200.01	0.02	0.096	2.401658	4.95E-05	0.0001083	0.02	0.04	0	0.08	0.11	0.1
OsR498G0203019400.01	478.67	1927.6	2.009171	0	0	454.32	498.63	483.1	1992.6	1851.3	1938.9
OsR498G0203023000.01	19.186	39.626	1.09702	0	0	18.29	19.79	19.48	36.91	41.04	40.93
OsR498G0203023100.01	6.393	57.593	3.17102	6.78E-298	2.09E-298	7.62	7.8	3.76	68.44	57.53	46.81
OsR498G0203027300.01	23.98	8.716	-1.45414	9.82E-92	6.34E-92	26.29	23.94	21.71	8.31	9.08	8.76
OsR498G0203029300.01	24.02	8.25	-1.53782	0	0	22.97	24.94	24.15	7.64	8.59	8.52
OsR498G0203029600.01	23.316	8.283	-1.53175	7.89E-184	3.42E-184	22.53	24.59	22.83	8.15	8.2	8.5
OsR498G0203030300.01	3.003	57.94	4.194786	0	0	3.63	2.89	2.49	57.89	59.84	56.09
OsR498G0203032000.01	0.043	6.313	7.121909	1.49E-63	1.17E-63	0	0.07	0.06	5.87	6.11	6.96
OsR498G0203040500.01	61.75	27.413	-1.20023	1.03E-215	4.02E-216	63.06	60.9	61.29	27.24	26.14	28.86
OsR498G0203044600.01	0	1.803	7.499192	2.20E-21	2.88E-21	0	0	0	1.63	1.56	2.22
OsR498G0203047200.01	8.793	4.226	-1.05837	1.56E-19	2.13E-19	9.43	9.78	7.17	4.54	4.12	4.02
OsR498G0203053100.01	5	2.056	-1.12066	5.78E-62	4.64E-62	4.44	5.35	5.21	1.95	2.14	2.08
OsR498G0203053600.01	49.296	10.67	-2.22057	0	0	50.76	48.5	48.63	10.86	9.96	11.19
OsR498G0203053700.01	27.083	1.063	-4.66068	0	0	24.79	27.98	28.48	1.3	1.11	0.78
OsR498G0203054900.01	9.07	2.74	-1.72736	3.45E-48	3.16E-48	9.36	8.42	9.43	3.11	2.8	2.31
OsR498G0203057100.01	14.486	6.336	-1.20236	9.49E-66	7.38E-66	15.53	14.27	13.66	6.32	5.86	6.83
OsR498G0203064500.01	3.796	136.89	5.162629	0	0	4.67	3.16	3.56	139.04	136.69	134.93
OsR498G0203065800.01	17.46	91.066	2.374439	0	0	17.18	17.4	17.8	90.18	88.22	94.8

OsR498G0203068700.01	0.946	0.016	-5.93752	1.66E-16	2.42E-16	0.94	1.17	0.73	0	0	0.05
OsR498G0203069700.01	13.286	4.763	-1.48151	3.51E-74	2.55E-74	13.5	12.59	13.77	4.61	4.91	4.77
OsR498G0203071300.01	1.1	0.383	-1.52584	2.47E-12	4.02E-12	1.06	1.32	0.92	0.31	0.4	0.44
OsR498G0203071500.01	15.166	31.386	1.041541	1.34E-131	7.08E-132	14.84	14.84	15.82	31.33	31.1	31.73
OsR498G0203071700.01	0.576	1.373	1.263185	0.0001491	0.0003371	0.72	0.77	0.24	0.99	1.57	1.56
OsR498G0203072200.01	1	0.22	-2.1935	5.86E-15	8.87E-15	0.97	0.85	1.18	0.28	0.18	0.2
OsR498G0203073400.01	7.08	32.923	2.228579	4.93E-263	1.67E-263	6.1	8.01	7.13	35.64	32.26	30.87
OsR498G0203078000.01	9.066	22.593	1.360175	1.13E-234	4.16E-235	8.92	8.92	9.36	22.08	21.9	23.8
OsR498G0203081900.01	11.273	22.86	1.014256	2.98E-94	1.89E-94	10.75	11.05	12.02	23.84	21.74	23
OsR498G0203082200.01	8.516	0.5	-4.01134	8.29E-91	5.39E-91	9.12	8.12	8.31	0.53	0.68	0.29
OsR498G0203083200.01	39.93	19.786	-1.0255	2.17E-144	1.08E-144	40.46	39.49	39.84	19.47	20.04	19.85
OsR498G0203084300.01	109.74	49.313	-1.15957	0	0	120.65	103.41	105.2	51.33	47.63	48.98
OsR498G0203084800.01	0.123	0.64	2.361688	0.0002336	0.0005362	0.09	0.19	0.09	0.95	0.29	0.68
OsR498G0203090900.01	8.133	1.59	-2.3163	7.07E-92	4.56E-92	7.57	7.56	9.27	1.58	1.6	1.59
OsR498G0203092800.01	8.313	2.32	-1.89658	5.56E-53	4.84E-53	7.95	8.75	8.24	2.56	1.93	2.47
OsR498G0203093300.01	5.09	12.296	1.225591	2.94E-55	2.51E-55	5.11	4.94	5.22	13.82	11.49	11.58
OsR498G0203096300.01	3.64	175.18	5.58056	0	0	3.04	3.53	4.35	181.89	174.44	169.2
OsR498G0203105400.01	0.706	0.293	-1.22943	1.37E-06	2.75E-06	0.7	0.73	0.69	0.36	0.18	0.34
OsR498G0203106600.01	165.27	23.446	-2.82249	0	0	163.24	165.81	166.8	22.89	23.41	24.04
OsR498G0203109500.01	3.156	11.613	1.884177	2.36E-96	1.48E-96	3.63	3.16	2.68	11.85	12.5	10.49
OsR498G0203109900.01	0.506	0.136	-1.85836	1.27E-05	2.70E-05	0.48	0.54	0.5	0.07	0.23	0.11
OsR498G0203112700.01	8.216	112.95	3.774804	0	0	8.6	7.8	8.25	115.09	114.26	109.49
OsR498G0203116200.01	8.136	19.623	1.301207	3.23E-95	2.04E-95	8.81	7.11	8.49	19.87	18.94	20.06
OsR498G0203116900.01	0.77	0.27	-1.52212	3.61E-06	7.41E-06	0.82	0.59	0.9	0.46	0.08	0.27
OsR498G0203118400.01	14.373	75.583	2.298245	0	0	14.14	15.25	13.73	82.56	71.43	72.76
OsR498G0203118600.01	0.143	0	-5.20817	3.61E-06	7.40E-06	0.09	0.21	0.13	0	0	0
OsR498G0203119400.01	105.73	32.22	-1.72681	0	0	108.27	106.13	102.8	32.62	32.5	31.54
OsR498G0203119500.01	6.18	1.063	-2.54166	5.10E-58	4.24E-58	6.08	6.28	6.18	1.2	0.86	1.13
OsR498G0203119900.01	0.313	1.233	1.973872	5.15E-13	8.19E-13	0.23	0.32	0.39	1.22	1.58	0.9
OsR498G0203120200.01	7.556	71.356	3.228353	0	0	7.9	7.45	7.32	73.85	70.25	69.97
OsR498G0203122000.01	11.75	57.203	2.273749	0	0	12.42	9.89	12.94	57.81	59.99	53.81
OsR498G0203126600.01	30.303	14.823	-1.03856	7.62E-60	6.22E-60	31.42	30.3	29.19	16.15	13.85	14.47
OsR498G0203127800.01	3.123	10.303	1.774881	8.47E-96	5.33E-96	2.84	3.5	3.03	11.02	10.44	9.45
OsR498G0203128900.01	0.106	4.3	5.370677	1.68E-116	9.51E-117	0.16	0.11	0.05	4.48	3.97	4.45
OsR498G0203132100.01	0.06	0.243	2.316469	4.04E-06	8.31E-06	0.06	0.07	0.05	0.3	0.23	0.2
OsR498G0203132400.01	12.633	4.976	-1.32587	6.98E-62	5.61E-62	12.09	12.09	13.72	6.02	4.58	4.33
OsR498G0203133400.01	7.303	3.513	-1.06054	3.67E-34	3.92E-34	7.42	7.06	7.43	3.53	3.88	3.13
OsR498G0203134100.01	0.063	0.276	2.209685	3.12E-07	6.09E-07	0.07	0.08	0.04	0.23	0.31	0.29
OsR498G0203134400.01	1.896	9.553	2.301855	3.52E-118	1.98E-118	1.67	1.91	2.11	10.42	9.82	8.42
OsR498G0203134700.01	0.426	4.126	3.270714	1.01E-32	1.10E-32	0.64	0.3	0.34	4.62	3.5	4.26
OsR498G0203139800.01	63.696	1.843	-5.12143	0	0	65.77	59.32	66	1.77	1.95	1.81
OsR498G0203139900.01	1.576	11.656	2.885752	6.82E-235	2.50E-235	1.79	1.55	1.39	12.76	11.56	10.65
OsR498G0203141200.01	2.613	39.063	3.890335	0	0	1.9	2.75	3.19	44.25	37	35.94
OsR498G0203141800.01	1.583	46.45	4.909658	0	0	1.89	1.47	1.39	48.87	48.72	41.76
OsR498G0203143200.01	5.113	19.973	1.946651	0	0	4.79	5.39	5.16	19.05	20.46	20.41
OsR498G0203143300.01	13.026	26.236	1.011054	6.52E-57	5.48E-57	13.35	13.58	12.15	25.14	27.94	25.63
OsR498G0203144000.01	20.446	44.973	1.129725	1.01E-202	4.07E-203	21.8	19.86	19.68	44.39	44.8	45.73
OsR498G0203146000.01	22.573	0.296	-6.50251	0	0	21.2	22.25	24.27	0.24	0.45	0.2
OsR498G0203147400.01	4.066	1.02	-1.99401	7.98E-42	7.80E-42	4.14	4.63	3.43	1.11	0.66	1.29
OsR498G0203156600.01	2830.6	872.81	-1.69886	0	0	2852.9	2832.3	2807	938.68	843.05	836.69
OsR498G0203156800.01	4.726	0.35	-3.7528	2.18E-59	1.79E-59	5.28	4.32	4.58	0.51	0.35	0.19
OsR498G0203158500.01	4.223	9.846	1.200952	3.65E-37	3.75E-37	4.77	4.17	3.73	9.33	10.17	10.04
OsR498G0203159400.01	0.186	0.493	1.438309	1.29E-07	2.46E-07	0.14	0.15	0.27	0.52	0.46	0.5
OsR498G0203159600.01	4.086	1.873	-1.14927	5.00E-07	9.85E-07	3.23	3.63	5.4	1.89	1.59	2.14
OsR498G0203160100.01	14.63	32.243	1.12548	2.95E-133	1.54E-133	14.65	14.92	14.32	30	33.46	33.27
OsR498G0203160400.01	0.27	1.033	1.917504	1.28E-16	1.87E-16	0.29	0.24	0.28	1.06	0.98	1.06
OsR498G0203163200.01	6.193	0.763	-2.87383	8.30E-53	7.23E-53	6.73	5.97	5.88	0.8	0.77	0.72
OsR498G0203163700.01	3.953	1.806	-1.13769	1.80E-36	1.86E-36	4.39	3.45	4.02	1.97	1.56	1.89
OsR498G0203165000.01	0.15	0.046	-1.63831	0.0002956	0.0006829	0.08	0.21	0.16	0.06	0.04	0.04
OsR498G0203167900.01	2.12	0.84	-1.32822	7.87E-13	1.26E-12	1.96	2.44	1.96	0.93	1.07	0.52
OsR498G0203169800.01	1.676	32.096	4.236797	0	0	1.29	1.67	2.07	33.92	32.81	29.56
OsR498G0203173700.01	13.276	410.68	4.942084	0	0	12.84	12.99	14	410.16	415.69	406.19
OsR498G0203175400.01	0.363	1.083	1.559134	5.35E-05	0.0001175	0.29	0.38	0.42	0.82	0.99	1.44
OsR498G0203176200.01	62.85	171.51	1.438496	0	0	66.53	59.66	62.36	187.15	180.36	147.02
OsR498G0203179000.01	5.62	12.933	1.203365	3.61E-111	2.09E-111	5.43	6.25	5.18	12.73	13.35	12.72
OsR498G0203181500.01	6.6	38.133	2.485162	0	0	7.05	5.78	6.97	39.94	37.54	36.92
OsR498G0203183800.01	0.063	1.126	4.139296	7.69E-28	8.98E-28	0.08	0.06	0.05	1.1	1.43	0.85
OsR498G0203187500.01	2.96	1.276	-1.20679	8.97E-15	1.36E-14	2.94	3.33	2.61	1.96	0.85	1.02
OsR498G0203188000.01	2.14	120.62	5.910627	0	0	1.74	2.03	2.65	126.95	118.31	116.61

OsR498G0203190000.01	8.863	2.633	-1.74193	5.84E-90	3.82E-90	8.88	8.38	9.33	2.74	2.42	2.74
OsR498G0203191000.01	53.536	24.46	-1.13486	1.71E-222	6.50E-223	54.91	52.45	53.25	26.93	23.6	22.85
OsR498G0203193100.01	9.183	1.016	-3.19903	5.85E-43	5.66E-43	9.43	8.22	9.9	0.72	0.86	1.47
OsR498G0203195500.01	2.176	8.873	2.164195	5.87E-254	2.04E-254	2.1	2.62	1.81	8.7	8.79	9.13
OsR498G0203196000.01	3.976	0	-8.17941	4.58E-32	5.03E-32	4.72	2.7	4.51	0	0	0
OsR498G0203200600.01	2.033	132.54	5.976005	0	0	1.64	1.3	3.16	129.85	140.84	126.94
OsR498G0203200800.01	0.656	9.936	4.072182	6.16E-79	4.32E-79	0.56	0.68	0.73	10.3	10	9.51
OsR498G0203202700.01	0.056	0.16	1.417902	0.0002739	0.0006314	0.09	0.01	0.07	0.21	0.11	0.16
OsR498G0203203000.01	0	0.136	4.36313	0.0005846	0.0013802	0	0	0	0.15	0.18	0.08
OsR498G0203204600.01	0	0.233	5.127223	8.48E-06	1.78E-05	0	0	0	0.24	0.17	0.29
OsR498G0203205200.01	0.066	0.156	1.270976	0.0006228	0.0014756	0.07	0.05	0.08	0.2	0.13	0.14
OsR498G0203209400.01	0.006	0.323	5.287688	7.27E-11	1.24E-10	0.02	0	0	0.35	0.29	0.33
OsR498G0203218700.01	0.253	7.503	5.073183	4.61E-217	1.79E-217	0.33	0.3	0.13	7.33	7.43	7.75
OsR498G0203220300.01	1.556	0.543	-1.53021	4.35E-29	4.98E-29	1.57	1.45	1.65	0.47	0.62	0.54
OsR498G0203220900.01	103.13	191.29	1.281734	0	0	121.92	95.81	91.66	186.99	184.73	202.15
OsR498G0203222800.01	0.073	10.253	7.120133	3.03E-94	1.92E-94	0	0.08	0.14	10.67	9.29	10.8
OsR498G0203225400.01	24.226	52.776	1.119781	7.07E-188	3.01E-188	24.32	24.28	24.08	51.89	52.08	54.36
OsR498G0203227300.01	0.11	0.663	2.432077	3.33E-13	5.28E-13	0.15	0.04	0.14	0.64	0.56	0.79
OsR498G0203227900.01	0	0.61	6.897741	2.86E-15	4.30E-15	0	0	0	0.59	0.54	0.7
OsR498G0203231200.01	74.906	30.563	-1.22436	6.07E-189	2.58E-189	73.21	75.86	75.65	31.54	30.83	29.32
OsR498G0203233000.01	37.11	4.366	-2.88331	0	0	37.84	36.8	36.69	4.42	4.01	4.67
OsR498G0203236900.01	8.033	3.42	-1.2739	1.74E-06	3.51E-06	10.19	6.74	7.17	3.52	2.92	3.82
OsR498G0203237300.01	24.3	106.66	2.094516	0	0	24.39	24.5	24.01	107.35	103.83	108.8
OsR498G0203238000.01	6.433	17.436	1.415475	4.25E-149	2.09E-149	5.63	6.35	7.32	18.23	17.29	16.79
OsR498G0203240400.01	0.97	2.416	1.308626	1.68E-12	2.72E-12	1	0.9	1.01	2.71	2.54	2
OsR498G0203240500.01	3.976	11.193	1.206723	8.45E-74	6.17E-74	3.79	4.32	3.82	10.45	12.34	10.79
OsR498G0203243500.01	111.22	23.773	-2.18484	0	0	113.13	109.54	111	25.5	24.67	21.15
OsR498G0203254500.01	6.683	27.78	2.036024	1.92E-252	6.68E-253	6.25	7.09	6.71	28.26	27.74	27.34
OsR498G0203254800.01	8.893	3.443	-1.7607	3.56E-117	2.01E-117	8.41	9.93	8.34	3.32	3.43	3.58
OsR498G0203255400.01	14.52	2.38	-2.61561	4.03E-132	2.12E-132	15.88	13.7	13.98	2.51	2.79	1.84
OsR498G0203255500.01	0.29	0.05	-2.57978	0.0004761	0.001117	0	0.26	0.61	0	0	0.15
OsR498G0203258100.01	0.13	0.393	1.580328	0.0003419	0.0007932	0.11	0.11	0.17	0.58	0.19	0.41
OsR498G0203259700.01	5.27	11.983	1.177675	3.49E-36	3.63E-36	5.04	5.13	5.64	13.25	10.97	11.73
OsR498G0203259900.01	15.216	83.576	2.443165	0	0	14.74	15.66	15.25	81.9	83.69	85.14
OsR498G0203260200.01	11.823	1.773	-2.74759	7.80E-88	5.16E-88	13.3	11.7	10.47	1.32	1.75	2.25
OsR498G0203262700.01	38.69	4.956	-3.02419	0	0	39.71	38.41	37.95	5.35	5.18	4.34
OsR498G0203262900.01	8.176	3.233	-1.23309	1.26E-65	9.84E-66	7.88	8.6	8.05	3.41	3.18	3.11
OsR498G0203263200.01	0.016	0.143	3.508757	0.0002195	0.0005025	0	0.05	0	0	0.08	0.35
OsR498G0203263400.01	42.81	128.42	1.572501	0	0	46.07	41.47	40.89	132.98	125.82	126.47
OsR498G0203265800.01	3.913	18.55	2.241026	0	0	3.67	3.98	4.09	19.01	17.98	18.66
OsR498G0203282600.01	0.046	3.146	6.046382	1.43E-139	7.29E-140	0.06	0.05	0.03	2.94	2.77	3.73
OsR498G0203284300.01	0.656	5.08	2.99085	2.38E-60	1.94E-60	0.56	0.57	0.84	4.92	5.32	5
OsR498G0203284600.01	1.226	2.61	1.129258	5.00E-14	7.76E-14	0.76	1.64	1.28	2.82	2.03	2.98
OsR498G0203286800.01	0.436	0.116	-1.90777	0.0002294	0.000526	0.49	0.41	0.41	0.15	0.05	0.15
OsR498G0203292500.01	11.603	41.743	1.842483	0	0	11.5	10.9	12.41	43.56	38.26	43.41
OsR498G0203292900.01	3.826	1.106	-1.78925	4.56E-30	5.14E-30	3.64	3.97	3.87	1.54	1.07	0.71
OsR498G0203293400.01	1.223	0.153	-3.01087	2.08E-11	3.48E-11	1.07	1.26	1.34	0.19	0.2	0.07
OsR498G0203294900.01	53.416	109.08	1.020575	7.60E-213	2.99E-213	51.84	53.75	54.66	107.03	110.56	109.64
OsR498G0203300400.01	41.55	104.21	1.321299	0	0	41.09	41.47	42.09	104.37	102.56	105.71
OsR498G0203302700.01	1.713	0.063	-4.92602	3.04E-30	3.42E-30	1.67	1.26	2.21	0.09	0.05	0.05
OsR498G0203308700.01	0.326	0	-5.35256	1.16E-06	2.33E-06	0.38	0.15	0.45	0	0	0
OsR498G0203310700.01	1.76	0.606	-1.53689	3.06E-14	4.72E-14	1.61	2.03	1.64	0.68	0.57	0.57
OsR498G0203314800.01	0.04	2.486	6.024653	3.69E-47	3.41E-47	0.08	0	0.04	2.62	2.75	2.09
OsR498G0203315000.01	3.09	0.293	-3.44211	7.24E-62	5.82E-62	2.61	3.09	3.57	0.23	0.18	0.47
OsR498G0203315300.01	0.453	1.483	1.702725	1.16E-06	2.32E-06	0.6	0.47	0.29	1.54	1.5	1.41
OsR498G0203318800.01	24.286	54.763	1.169189	0	0	23.84	23.85	25.17	54.19	54.73	55.37
OsR498G0203322200.01	12.53	28.666	1.1659	4.15E-184	1.80E-184	11.87	12.68	13.04	27.38	29.5	29.12
OsR498G0203322500.01	0.076	0.416	2.399006	2.57E-05	5.54E-05	0.05	0.09	0.09	0.26	0.31	0.68
OsR498G0203324500.01	0.79	0	-5.81822	1.42E-08	2.62E-08	0.73	1.02	0.62	0	0	0
OsR498G0203327100.01	4.29	2.156	-1.0427	0.0001436	0.0003244	6.13	1.34	5.4	2.08	2.29	2.1
OsR498G0203328000.01	0.07	0.503	2.794648	4.87E-06	1.01E-05	0.05	0.06	0.1	0.72	0.34	0.45
OsR498G0203331500.01	1.533	0.626	-1.30009	2.67E-06	5.44E-06	1.19	1.72	1.69	0.52	0.59	0.77
OsR498G0203336700.01	22.41	156.34	2.772998	0	0	21.73	22.75	22.75	155.18	157.69	156.16
OsR498G0203340000.01	0.803	26.89	5.03976	8.85E-227	3.33E-227	0.81	0.59	1.01	28.07	27.38	25.22
OsR498G0203343200.01	17.04	46.93	1.452981	1.28E-162	5.94E-163	17.43	16.8	16.89	46.38	47.95	46.46
OsR498G0203343400.01	9.026	23.006	1.334574	2.04E-50	1.82E-50	10.87	7.59	8.62	22.3	23.5	23.22
OsR498G0203347200.01	0.533	4.996	3.12121	1.84E-119	1.02E-119	0.45	0.62	0.53	5.26	5.04	4.69
OsR498G0203351800.01	53.346	11.953	-2.1728	0	0	48.96	56.15	54.93	11.52	12.03	12.31
OsR498G0203352400.01	0.28	3.816	3.770702	1.33E-50	1.18E-50	0.42	0.29	0.13	4.43	3.65	3.37

OsR498G0203353700.01	77.373	6.61	-3.54848	0	0	77.98	76.64	77.5	7.09	6.6	6.14
OsR498G0203357500.01	8.623	3.1	-1.48334	3.32E-28	3.85E-28	8.35	9.39	8.13	2.98	2.93	3.39
OsR498G0203361000.01	13.14	6.056	-1.1406	1.75E-64	1.37E-64	13.77	12.24	13.41	5.97	5.73	6.47
OsR498G0203365400.01	19.183	6.733	-1.3619	8.94E-61	7.24E-61	18.09	21.66	17.8	6.49	6.67	7.04
OsR498G0203365500.01	4.39	2.17	-1.01801	5.67E-22	7.36E-22	4.97	4.29	3.91	1.96	2.01	2.54
OsR498G0203377400.01	6.586	2.11	-1.63093	1.76E-50	1.57E-50	6.67	6.43	6.66	2.07	2.21	2.05
OsR498G0203378100.01	3.08	1.446	-1.10775	7.72E-22	1.00E-21	3.16	2.96	3.12	1.41	1.45	1.48
OsR498G0203378400.01	18.243	49.77	1.439754	1.31E-213	5.13E-214	17.52	18.05	19.16	47.55	50.79	50.97
OsR498G0203379500.01	0.033	0.413	3.624723	1.45E-06	2.92E-06	0	0	0.1	0.35	0.68	0.21
OsR498G0203385800.01	0	0.193	5.563322	2.64E-07	5.13E-07	0	0	0	0.15	0.23	0.2
OsR498G0203390500.01	19.803	5.083	-1.97735	1.01E-199	4.11E-200	20.45	19.51	19.45	4.99	5.25	5.01
OsR498G0203392500.01	0.136	0	-5.13016	6.38E-06	1.33E-05	0.07	0.19	0.15	0	0	0
OsR498G0203395500.01	4.516	0.996	-2.20486	9.56E-53	8.33E-53	4.89	4.82	3.84	0.83	1.22	0.94
OsR498G0203396500.01	5.496	2.583	-1.11169	4.14E-27	4.89E-27	5.42	5.03	6.04	2.71	2.53	2.51
OsR498G0203396700.01	5.596	1.946	-1.5179	1.12E-65	8.73E-66	5.35	5.57	5.87	1.98	1.95	1.91
OsR498G0203399200.01	6.6	2.146	-1.65912	1.97E-75	1.42E-75	6.21	6.97	6.62	1.96	2.42	2.06
OsR498G0203399400.01	0.33	0	-4.66068	0.0001179	0.0002647	0.23	0.32	0.44	0	0	0
OsR498G0203400100.01	31.726	114.28	1.843661	0	0	31.29	31.36	32.53	108.04	116.47	118.33
OsR498G0203401800.01	0.506	1.753	1.776726	2.13E-05	4.58E-05	0.38	0.66	0.48	1.17	2.12	1.97
OsR498G0203402100.01	3.403	6.966	1.036825	2.05E-21	2.69E-21	3.32	3.6	3.29	6.8	6.93	7.17
OsR498G0203402400.01	0.63	7.626	3.594514	1.84E-160	8.57E-161	0.74	0.63	0.52	8.62	7.39	6.87
OsR498G0203408600.01	3.95	0.566	-2.80192	1.54E-99	9.51E-100	4.03	4.16	3.66	0.54	0.74	0.42
OsR498G0203410200.01	2.913	0.386	-2.69204	1.60E-56	1.35E-56	2.95	3.34	2.45	0.4	0.39	0.37
OsR498G0203410500.01	3.843	0.316	-3.6064	5.67E-62	4.55E-62	3.56	3.96	4.01	0.22	0.31	0.42
OsR498G0203410800.01	0.473	0	-7.15006	5.00E-18	7.04E-18	0.43	0.37	0.62	0	0	0
OsR498G0203413500.01	31.973	14.693	-1.13117	1.17E-65	9.13E-66	32.5	30.99	32.43	16.11	13.42	14.55
OsR498G0203417000.01	188	31.43	-2.59689	0	0	198.33	180.23	185.5	32.63	30.63	31.03
OsR498G0203417300.01	12.77	30.116	1.237343	9.70E-138	4.99E-138	13.07	12.58	12.66	30.1	29.54	30.71
OsR498G0203420100.01	4.236	17.926	2.076102	0	0	4.83	3.94	3.94	16.54	19.01	18.23
OsR498G0203420700.01	0.746	13.736	4.208014	0	0	0.6	0.99	0.65	13.12	14.34	13.75
OsR498G0203421300.01	1.496	19.436	2.76768	4.05E-298	1.25E-298	1.55	1.45	1.49	19.57	17.87	20.87
OsR498G0203431100.01	342.44	964.93	1.396343	0	0	336.05	346.01	345.3	918.74	993.75	982.3
OsR498G0203435200.01	3.22	0.923	-1.79518	1.21E-13	1.90E-13	3.06	3.44	3.16	0.63	0.92	1.22
OsR498G0203435500.01	1.846	0.066	-5.18263	1.28E-27	1.50E-27	2.14	2.04	1.36	0.15	0	0.05
OsR498G0203438600.01	1.593	6.74	2.076286	3.00E-55	2.56E-55	1.5	1.75	1.53	6.95	7.31	5.96
OsR498G0203438800.01	2.05	6.653	1.688211	7.31E-45	6.92E-45	1.77	2.09	2.29	7.12	5.83	7.01
OsR498G0203450200.01	1.94	0.583	-1.74224	5.19E-26	6.24E-26	1.6	2.34	1.88	0.5	0.63	0.62
OsR498G0203451100.01	0.26	0.856	1.76768	1.46E-06	2.93E-06	0.41	0.1	0.27	0.74	0.79	1.04
OsR498G0203451400.01	0.273	0.033	-3.0477	0.0001372	0.0003095	0.19	0.4	0.23	0.05	0.05	0
OsR498G0203456000.01	12.9	6.36	-1.08859	2.27E-62	1.81E-62	13.63	12.46	12.61	6.24	6.04	6.8
OsR498G0203462400.01	19.54	41.503	1.079617	6.45E-209	2.56E-209	19.35	19.46	19.81	41.8	41.32	41.39
OsR498G0203476400.01	16.52	33.79	1.034148	3.23E-93	2.07E-93	18.29	14.66	16.61	34.15	33.76	33.46
OsR498G0203476700.01	0.106	5.363	5.663276	2.02E-119	1.13E-119	0.06	0.17	0.09	6.06	4.88	5.15
OsR498G0203478900.01	38.396	79.306	1.035357	0	0	36.79	40.85	37.55	78.86	79.96	79.1
OsR498G0203479900.01	5.566	77.063	3.784916	0	0	6.02	5.05	5.63	77.7	77.21	76.28
OsR498G0203482500.01	4.71	2.193	-1.10715	2.15E-26	2.58E-26	5.01	4.37	4.75	2.42	2.18	1.98
OsR498G0203492500.01	61.49	30.59	-1.01689	4.12E-98	2.56E-98	61.74	63.01	59.72	29.82	30.44	31.51
OsR498G0203495200.01	21.313	5.953	-1.85115	2.69E-160	1.26E-160	21.37	22.71	19.86	6.4	6.13	5.33
OsR498G0203498400.01	0.41	1.703	2.046679	1.03E-24	1.27E-24	0.45	0.19	0.59	1.29	1.91	1.91
OsR498G0203501200.01	5.96	1.22	-2.29508	1.01E-166	4.59E-167	5.54	6.1	6.24	1.12	1.18	1.36
OsR498G0203502600.01	10.723	5.043	-1.07989	2.98E-57	2.50E-57	9.57	11.71	10.89	4.86	4.97	5.3
OsR498G0203504400.01	6.133	0.953	-2.7087	9.72E-27	1.16E-26	6.22	5.75	6.43	0.45	1.27	1.14
OsR498G0203509200.01	3.353	1.51	-1.26509	5.27E-22	6.83E-22	3.69	2.53	3.84	1.35	1.75	1.43
OsR498G0203512500.01	32.163	70	1.231268	1.54E-163	7.12E-164	26.5	31.42	38.57	83.37	60.58	66.05
OsR498G0203514900.01	2.056	8.576	2.037202	3.94E-58	3.27E-58	2.14	1.38	2.65	8.94	9.06	7.73
OsR498G0203517300.01	21.653	44.786	1.08011	8.62E-76	6.19E-76	29.99	21.17	13.8	33.34	48.92	52.1
OsR498G0203525300.01	21.533	9.87	-1.13242	3.99E-66	3.09E-66	22.67	20.72	21.21	9.83	8.8	10.98
OsR498G0203538600.01	1.75	0.463	-2.03824	6.40E-14	9.96E-14	2.03	1.26	1.96	0.56	0.49	0.34
OsR498G0203542600.01	7.376	79.783	3.448131	0	0	7.18	7.18	7.77	77.33	79.72	82.3
OsR498G0203543900.01	118.16	46.373	-1.37877	0	0	115.05	120.14	119.3	43.96	46.91	48.25
OsR498G0203544900.01	0.453	23.066	5.659868	0	0	0.39	0.38	0.59	23.11	24.19	21.9
OsR498G0203550700.01	0.443	0.67	1.03976	4.94E-09	8.94E-09	0.36	0.46	0.51	0.65	0.77	0.59
OsR498G0203559000.01	12.29	0.076	-7.37903	0	0	11.73	12.45	12.69	0.08	0.06	0.09
OsR498G0203562400.01	3.726	0.96	-2.13016	4.02E-42	3.91E-42	3.68	3.59	3.91	0.82	0.82	1.24
OsR498G0203563400.01	32.043	9.376	-1.68241	3.17E-120	1.76E-120	33.88	32.53	29.72	9.6	9.87	8.66
OsR498G0203563500.01	4.373	1.156	-1.99149	2.92E-59	2.40E-59	4.42	4.03	4.67	1.14	0.96	1.37
OsR498G0203566300.01	0.706	0.116	-2.57495	1.07E-08	1.96E-08	0.82	0.59	0.71	0.22	0	0.13
OsR498G0203567200.01	0.55	1.176	1.126175	1.13E-09	2.01E-09	0.65	0.55	0.45	1.15	1.18	1.2
OsR498G0203569900.01	0.216	1.1	2.310614	5.58E-16	8.25E-16	0.14	0.09	0.42	1.28	1.15	0.87

OsR498G0203573900.01	3.063	0	-9.24101	1.66E-57	1.39E-57	3.21	2.96	3.02	0	0	0
OsR498G0203585900.01	0.54	7.7	3.969371	1.07E-136	5.50E-137	0.39	0.65	0.58	8.27	7.88	6.95
OsR498G0203591100.01	3.703	1.783	-1.11883	9.23E-16	1.37E-15	3.69	3.87	3.55	1.65	1.72	1.98
OsR498G0203592300.01	325.72	55.633	-2.57212	0	0	318.77	329.57	328.8	51.28	57.51	58.11
OsR498G0203592500.01	2.443	0.103	-4.6041	6.01E-13	9.58E-13	2.21	1.71	3.41	0	0.31	0
OsR498G0203594100.01	14.42	75.906	2.365483	0	0	14.97	14.99	13.3	76.97	72.96	77.79
OsR498G0203595100.01	0.36	61.413	7.420057	0	0	0.36	0.34	0.38	66.09	58.03	60.12
OsR498G0203609300.01	2.33	7.74	1.732169	3.99E-111	2.31E-111	2.1	2.52	2.37	8.24	7.71	7.27
OsR498G0203621100.01	0	1.386	6.348645	3.08E-11	5.18E-11	0	0	0	1.03	1.72	1.41
OsR498G0203623700.01	4.683	1.363	-1.91793	2.21E-67	1.69E-67	5.43	4.76	3.86	1.53	1.51	1.05
OsR498G0203635500.01	4.903	2.346	-1.0576	1.51E-24	1.86E-24	4.86	4.7	5.15	2.29	2.46	2.29
OsR498G0203643200.01	32.233	280.84	3.09968	0	0	32.33	32.86	31.51	283.73	279.42	279.36
OsR498G0203644600.01	0.423	0.113	-1.94253	2.94E-08	5.49E-08	0.41	0.37	0.49	0.09	0.11	0.14
OsR498G0203644800.01	0.996	0.086	-3.5452	1.15E-08	2.12E-08	0.99	0.96	1.04	0.09	0.17	0
OsR498G0203647200.01	7.813	2.196	-1.77515	8.06E-86	5.40E-86	7.41	8.05	7.98	2.01	2	2.58
OsR498G0203648900.01	1.696	0.086	-4.32364	4.78E-55	4.08E-55	1.59	1.55	1.95	0.15	0	0.11
OsR498G0203651600.01	0.436	0.136	-2.48831	1.12E-05	2.37E-05	0.41	0.49	0.41	0.29	0.12	0
OsR498G0203657700.01	0.303	0	-6.13016	3.48E-10	6.05E-10	0.25	0.22	0.44	0	0	0
OsR498G0203660600.01	0.226	1.713	2.922403	2.78E-21	3.65E-21	0.25	0.27	0.16	2.36	1.24	1.54
OsR498G0203665400.01	7.736	3.876	-1.11844	7.76E-61	6.28E-61	7.8	6.99	8.42	4.02	4.09	3.52
OsR498G0203677700.01	5.293	32.713	2.629607	2.29E-306	6.88E-307	5.27	5.37	5.24	33.31	31.34	33.49
OsR498G0203700800.01	7.756	2.65	-1.55588	8.87E-39	8.96E-39	7.89	8.04	7.34	2.45	3.17	2.33
OsR498G0203709300.01	2.323	1.063	-1.53282	4.12E-10	7.20E-10	1.93	3.02	2.02	1.01	1.1	1.08
OsR498G0203719700.01	0.366	1.52	2.03976	1.49E-10	2.56E-10	0.66	0.17	0.27	1.23	1.27	2.06
OsR498G0203727200.01	0.263	0.103	-1.37528	0.0003652	0.0008492	0.32	0.12	0.35	0.08	0.13	0.1
OsR498G0203738600.01	308.01	31.936	-3.27578	0	0	325.59	297.97	300.5	35.59	29	31.22
OsR498G0203738700.01	8.49	1.703	-2.33616	1.29E-33	1.39E-33	9.62	7.12	8.73	2.37	1.22	1.52
OsR498G0203743900.01	1.983	0.856	-1.21313	1.40E-14	2.14E-14	1.74	2.12	2.09	0.86	0.92	0.79
OsR498G0203746600.01	12.576	5.806	-1.22563	1.33E-57	1.11E-57	12.02	12.56	13.15	5.9	6.1	5.42
OsR498G0203746900.01	7.163	29.666	2.037937	1.08E-87	7.16E-88	8.38	7.73	5.38	29.53	29.1	30.37
OsR498G0203747100.01	0.04	13.176	8.403895	1.07E-135	5.54E-136	0.06	0	0.06	14.64	12.48	12.41
OsR498G0203752600.01	0.206	19.41	6.582792	0	0	0.15	0.32	0.15	19.46	18.67	20.1
OsR498G0203757400.01	0.21	2.43	3.550722	2.96E-35	3.12E-35	0.12	0.39	0.12	2.3	2.29	2.7
OsR498G0203758400.01	73.78	250.13	1.765629	0	0	75.22	72.08	74.04	253.14	249.6	247.65
OsR498G0203760300.01	0	0.276	5.499192	4.66E-07	9.16E-07	0	0	0	0.41	0.23	0.19
OsR498G0203761600.01	6.61	3.243	-1.00065	4.36E-15	6.57E-15	6.25	5.81	7.77	3.75	2.83	3.15
OsR498G0203761700.01	0.26	0.023	-3.41967	0.0009442	0.002269	0.28	0.3	0.2	0.07	0	0
OsR498G0203761900.01	0.44	0.123	-1.85332	0.0003648	0.0008482	0.71	0.27	0.34	0.21	0.05	0.11
OsR498G0203764700.01	0.01	0.54	5.82112	5.19E-15	7.84E-15	0.03	0	0	0.52	0.6	0.5
OsR498G0203767300.01	69.16	21.076	-1.71043	0	0	71.11	66.02	70.35	21.77	21.34	20.12
OsR498G0203773700.01	1.14	4.26	1.912729	7.09E-66	5.50E-66	1.15	1.28	0.99	4.34	4.55	3.89
OsR498G0203777200.01	4.07	1.813	-1.16163	2.26E-29	2.58E-29	4.33	3.79	4.09	2.09	1.43	1.92
OsR498G0203781300.01	0.71	0.073	-3.36623	1.68E-11	2.79E-11	0.55	0.99	0.59	0.09	0.13	0
OsR498G0203785700.01	0.573	3.883	2.756917	4.65E-69	3.52E-69	0.57	0.63	0.52	4.51	3.5	3.64
OsR498G0203787200.01	7.063	18.286	1.356204	1.07E-113	6.14E-114	7.36	7.21	6.62	19.27	17.86	17.73
OsR498G0203794900.01	18.043	55.296	1.652628	0	0	17.16	17.98	18.99	54.31	56.64	54.94
OsR498G0203795300.01	79.576	11.703	-2.76901	0	0	83.47	79.51	75.75	11.39	11.78	11.94
OsR498G0203808800.01	2.11	0.82	-1.30848	1.32E-28	1.52E-28	1.91	1.8	2.62	1.05	0.97	0.44
OsR498G0203816400.01	8.906	30.366	1.781392	0	0	8.54	9.1	9.08	28.93	30.58	31.59
OsR498G0203816900.01	7.823	0.683	-3.58007	1.92E-257	6.61E-258	6.82	8.19	8.46	0.73	0.71	0.61
OsR498G0203819600.01	0.31	4.65	3.889138	8.00E-89	5.27E-89	0.16	0.27	0.5	5.27	5.26	3.42
OsR498G0203829100.01	1.21	0.163	-2.89098	3.46E-21	4.56E-21	1.13	1.27	1.23	0.15	0.12	0.22
OsR498G0203835200.01	37.283	103.03	1.478825	0	0	38.7	36.95	36.2	102.74	106.22	100.14
OsR498G0203841100.01	0.02	0.19	3.432077	8.80E-06	1.85E-05	0.03	0.03	0	0.21	0.22	0.14
OsR498G0203847400.01	70.373	9.05	-2.97252	0	0	70.82	70.44	69.86	9.29	8.21	9.65
OsR498G0203859700.01	1.846	0.26	-2.85332	8.89E-67	6.84E-67	1.97	1.85	1.72	0.2	0.34	0.24
OsR498G0203864300.01	26.866	73.696	1.447658	0	0	27.2	26.93	26.47	72.4	73.37	75.32
OsR498G0203871600.01	3.38	0.453	-2.91548	7.85E-22	1.02E-21	2.43	4.2	3.51	0.42	0.67	0.27
OsR498G0203882300.01	0.706	19.886	4.809414	8.56E-229	3.20E-229	0.7	0.8	0.62	18.87	20.5	20.29
OsR498G0203882700.01	0.463	0.053	-3.41967	5.86E-10	1.03E-09	0.38	0.3	0.71	0.06	0	0.1
OsR498G0203883100.01	2.17	50.7	4.548189	0	0	2.32	2.47	1.72	52.01	53.37	46.72
OsR498G0203883500.01	21.303	90.086	1.990838	0	0	21.98	20.54	21.39	93.61	88.85	87.8
OsR498G0203884300.01	1.966	5.356	1.244558	1.11E-62	8.87E-63	1.74	1.99	2.17	4.93	6	5.14
OsR498G0203894800.01	200.92	462.14	1.192201	0	0	227.31	182.88	192.6	496.1	457	433.31
OsR498G0203899300.01	25.696	10.103	-1.36489	1.07E-117	6.01E-118	27.29	25.58	24.22	11	9.12	10.19
OsR498G0203900100.01	1.81	0.65	-1.47481	6.40E-10	1.12E-09	2.02	1.89	1.52	0.73	0.52	0.7
OsR498G0203902500.01	0.3	3.316	3.42749	1.11E-31	1.23E-31	0.25	0.31	0.34	2.64	3.85	3.46
OsR498G0203903500.01	33.723	12.496	-1.49121	7.64E-243	2.74E-243	36	32.58	32.59	11.76	12.93	12.8
OsR498G0203905300.01	0.073	0.386	2.361688	4.96E-05	0.0001086	0.04	0.05	0.13	0.14	0.23	0.79

OsR498G0203907000.01	0	0.226	4.946651	2.78E-05	6.01E-05	0	0	0	0.13	0.28	0.27
OsR498G0203912300.01	5.603	25.423	2.176329	0	0	5.64	5.72	5.45	25.63	26.55	24.09
OsR498G0203912800.01	0	0.7	6.847115	7.65E-15	1.16E-14	0	0	0	0.7	0.84	0.56
OsR498G0203915600.01	0.82	0.13	-2.67173	5.52E-21	7.31E-21	0.85	0.7	0.91	0.11	0.19	0.09
OsR498G0203917700.01	3.41	302.98	6.464346	0	0	3.37	3.24	3.62	303.12	305.72	300.1
OsR498G0203919800.01	0.106	0.45	2.075384	2.31E-06	4.71E-06	0.1	0.1	0.12	0.52	0.5	0.33
OsR498G0203920700.01	62.786	17.826	-1.82431	0	0	63.7	60.75	63.91	16.55	17.95	18.98
OsR498G0203921400.01	0.403	1.8	2.079288	6.43E-06	1.34E-05	0.54	0	0.67	1.57	2.37	1.46
OsR498G0203927400.01	1.956	0.916	-1.08665	7.67E-08	1.46E-07	2.11	2.08	1.68	1.23	0.72	0.8
OsR498G0203939000.01	4.51	54.56	3.609238	0	0	4.73	4.38	4.42	52.59	54.91	56.18
OsR498G0203939200.01	6.863	93.8	3.767297	0	0	6.54	6.89	7.16	97.55	90.6	93.25
OsR498G0203939400.01	26.443	107.85	2.02639	0	0	25.71	27.36	26.26	108.06	110.74	104.76
OsR498G0203948700.01	2.39	1.1	-1.06333	0.0004454	0.0010429	2.77	2.39	2.01	0.6	1.59	1.11
OsR498G0203951900.01	0.063	0.24	1.914229	0.000621	0.0014712	0.1	0.03	0.06	0.16	0.3	0.26
OsR498G0203954400.01	0.196	2.053	3.352643	1.63E-31	1.80E-31	0.11	0.08	0.4	1.82	1.89	2.45
OsR498G0203954800.01	39.546	100.55	1.335892	0	0	39.96	39.09	39.59	98.12	101.32	102.21
OsR498G0203959100.01	14.116	6.126	-1.16168	8.97E-72	6.64E-72	14.07	13.9	14.38	6.34	5.69	6.35
OsR498G0203959300.01	7.06	2.913	-1.20147	7.59E-60	6.20E-60	7.02	7.37	6.79	2.82	2.77	3.15
OsR498G0203962500.01	2.18	30.906	3.8252	0	0	2.24	1.83	2.47	32.26	31.24	29.22
OsR498G0203968800.01	2.45	0.45	-2.44567	5.77E-13	9.20E-13	2.08	2.44	2.83	0.26	0.66	0.43
OsR498G0203969700.01	10.756	4.273	-1.35592	6.14E-56	5.19E-56	10.72	11.45	10.1	3.99	4.14	4.69
OsR498G0203971900.01	1.173	2.726	1.213092	1.33E-23	1.67E-23	0.84	1.4	1.28	2.86	2.87	2.45
OsR498G0203973700.01	6.583	1047.9	7.311379	0	0	6.45	6.67	6.63	1020.2	1071.3	1052.2
OsR498G0203976100.01	0.93	25.363	4.76138	0	0	1.33	0.66	0.8	25.59	24.88	25.62
OsR498G0203976400.01	5.876	80.383	3.938325	0	0	4.88	6.37	6.38	85.04	81.5	74.61
OsR498G0203981000.01	2.006	9.39	1.812912	1.99E-160	9.29E-161	1.96	2.06	2	9.02	8.9	10.25
OsR498G0203983400.01	0.156	0.693	2.159953	2.52E-19	3.45E-19	0.11	0.21	0.15	0.78	0.7	0.6
OsR498G0203986800.01	5.233	2.15	-1.28217	7.16E-49	6.50E-49	4.7	5.62	5.38	2.1	2.2	2.15
OsR498G0203987400.01	67.106	20.813	-1.70323	1.11E-246	3.95E-247	67.96	66.94	66.42	23.59	17.54	21.31
OsR498G0203987700.01	1.786	5.186	1.515493	1.09E-14	1.66E-14	2.26	1.32	1.78	4.91	6.09	4.56
OsR498G0203993000.01	1.493	0.093	-4.02633	9.45E-16	1.41E-15	1.33	1.61	1.54	0.07	0.14	0.07
OsR498G0203995700.01	0.48	1.863	1.946651	3.17E-17	4.56E-17	0.47	0.38	0.59	1.9	1.89	1.8
OsR498G0203996200.01	1.06	5.853	2.454798	1.64E-45	1.54E-45	1.27	0.89	1.02	6.86	4.85	5.85
OsR498G0203998100.01	0.033	0.393	3.543533	1.82E-08	3.37E-08	0.03	0.07	0	0.41	0.42	0.35
OsR498G0203999500.01	0.743	0.146	-2.35256	4.05E-07	7.94E-07	0.58	0.78	0.87	0.22	0.11	0.11
OsR498G0204010400.01	0.236	17.55	6.218563	1.82E-156	8.60E-157	0.23	0.33	0.15	19.18	17.58	15.89
OsR498G0204016000.01	3.606	1.423	-1.42321	6.70E-28	7.81E-28	3.88	3.42	3.52	1.27	1.43	1.57
OsR498G0204017200.01	3.643	7.516	1.054296	1.69E-34	1.79E-34	3.75	3.79	3.39	7.52	8.39	6.64
OsR498G0204022900.01	48.15	140.55	1.526965	0	0	46.68	48.58	49.19	143.29	140.5	137.87
OsR498G0204023600.01	0.01	2.396	7.726261	1.19E-46	1.11E-46	0	0	0.03	3.37	1.92	1.9
OsR498G0204025500.01	0.773	9.03	3.544995	6.61E-139	3.38E-139	0.67	0.82	0.83	9.18	9.34	8.57
OsR498G0204026100.01	17.473	5.97	-1.50272	5.93E-196	2.46E-196	17.45	16.87	18.1	5.86	6.04	6.01
OsR498G0204031200.01	0.013	1.173	6.432077	1.05E-21	1.37E-21	0	0	0.04	1.4	1.32	0.8
OsR498G0204033800.01	0.343	10.716	4.937313	3.79E-217	1.47E-217	0.27	0.28	0.48	11.67	9.78	10.7
OsR498G0204036000.01	0.98	0.19	-2.37528	4.43E-12	7.26E-12	0.84	1.11	0.99	0.19	0.23	0.15
OsR498G0204047500.01	0.706	0.133	-2.34536	4.23E-07	8.31E-07	0.61	0.43	1.08	0.12	0.19	0.09
OsR498G0204065700.01	2.256	13.823	2.608947	0	0	2.13	2.24	2.4	13.31	14.04	14.12
OsR498G0204068700.01	0.74	0.206	-1.82918	4.10E-05	8.94E-05	0.54	0.98	0.7	0.06	0.25	0.31
OsR498G0204080800.01	0.063	0.613	3.272421	1.96E-10	3.38E-10	0.04	0.04	0.11	0.68	0.54	0.62
OsR498G0204084000.01	0.05	0.883	4.084154	3.90E-23	4.94E-23	0	0.08	0.07	0.76	0.77	1.12
OsR498G0204086500.01	0.376	0.14	-1.43829	7.26E-07	1.44E-06	0.4	0.38	0.35	0.16	0.17	0.09
OsR498G0204091800.01	0.433	1.153	1.408026	2.10E-08	3.90E-08	0.66	0.31	0.33	1.24	1.24	0.98
OsR498G0204092600.01	8.116	42.61	2.389933	0	0	7.42	8.47	8.46	42.35	41.74	43.74
OsR498G0204095000.01	1.186	4.91	2.002113	4.59E-70	3.45E-70	1.01	1.22	1.33	5.27	4.68	4.78
OsR498G0204096700.01	9.07	20.033	1.158952	7.31E-113	4.21E-113	9.04	9.05	9.12	19.96	19.47	20.67
OsR498G0204100400.01	0.31	0.133	-1.22327	8.56E-05	0.0001904	0.26	0.37	0.3	0.08	0.16	0.16
OsR498G0204100600.01	246.68	52.136	-2.27024	0	0	243.59	246.36	250.1	50.95	52.9	52.56
OsR498G0204103100.01	9.593	20.953	1.12122	0	0	8.69	10.1	9.99	20	21.59	21.27
OsR498G0204103500.01	25.13	0.483	-5.75877	0	0	26.03	23.98	25.38	0.33	0.65	0.47
OsR498G0204104000.01	15.04	37.51	1.292834	1.80E-277	5.86E-278	15.81	13.72	15.59	36.86	37.53	38.14
OsR498G0204106300.01	5.176	1.07	-2.28217	5.74E-36	5.99E-36	5.23	5.72	4.58	0.73	0.93	1.55
OsR498G0204107200.01	1.35	0.696	-1.0372	8.68E-08	1.65E-07	1.2	1.65	1.2	0.69	0.69	0.71
OsR498G0204107300.01	0.076	3.07	5.302794	2.10E-47	1.93E-47	0.14	0	0.09	2.75	3.25	3.21
OsR498G0204107700.01	7.26	2.363	-1.64895	5.08E-60	4.15E-60	6.85	6.85	8.08	2.61	2.23	2.25
OsR498G0204108000.01	2.236	0.213	-3.38474	3.72E-39	3.74E-39	2.43	2.07	2.21	0.27	0.17	0.2
OsR498G0204108600.01	0.036	0.16	2.110149	0.0003986	0.0009301	0.02	0.05	0.04	0.09	0.23	0.16
OsR498G0204111200.01	5.686	32.96	2.54226	0	0	5.5	5.8	5.76	32.14	33.73	33.01
OsR498G0204113500.01	226.86	60.673	-1.91039	0	0	238.07	230.86	211.7	61.69	60.59	59.74
OsR498G0204113800.01	131.23	30.636	-2.10763	0	0	141.39	125.57	126.7	31.48	29.98	30.45

OsR498G0204117500.01	1.09	11.226	3.506659	0	0	1.17	1.36	0.74	11.08	10.97	11.63
OsR498G0204117800.01	12.43	25.996	1.061752	4.60E-81	3.18E-81	13.55	12.81	10.93	26	25.12	26.87
OsR498G0204121100.01	12.44	36.76	1.672351	6.41E-150	3.13E-150	12.17	12.43	12.72	39.27	36.44	34.57
OsR498G0204121600.01	0.16	31.243	7.587426	0	0	0.15	0.1	0.23	34.02	31.84	27.87
OsR498G0204123300.01	0.473	0.106	-2.01913	4.91E-07	9.67E-07	0.59	0.44	0.39	0.07	0.06	0.19
OsR498G0204124000.01	0.286	1.836	2.608603	7.09E-28	8.27E-28	0.38	0.31	0.17	1.86	1.7	1.95
OsR498G0204130500.01	121.01	53.153	-1.13733	0	0	124.3	118.82	119.9	51.01	53.33	55.12
OsR498G0204131700.01	6.123	1.503	-1.99108	8.28E-36	8.66E-36	5.1	6.75	6.52	1.3	1.58	1.63
OsR498G0204134300.01	0.026	1.16	5.361688	6.81E-21	9.03E-21	0.04	0	0.04	1.02	1.41	1.05
OsR498G0204135900.01	9.436	109.77	3.539112	0	0	10.16	9.66	8.49	118.47	106.23	104.61
OsR498G0204137000.01	4.306	20.09	2.213316	3.64E-265	1.22E-265	4.33	4.2	4.39	18.76	20.98	20.53
OsR498G0204139100.01	0.813	2.39	1.553021	1.38E-13	2.17E-13	0.62	0.93	0.89	2.83	2	2.34
OsR498G0204143300.01	0.32	0.056	-2.49629	1.03E-05	2.18E-05	0.3	0.31	0.35	0	0.07	0.1
OsR498G0204145300.01	0.073	0.54	2.876261	6.13E-10	1.08E-09	0.06	0.07	0.09	0.48	0.39	0.75
OsR498G0204149200.01	1.72	7.686	2.155838	1.11E-88	7.31E-89	1.8	1.81	1.55	7.12	8.11	7.83
OsR498G0204150700.01	13.016	2.65	-2.29916	1.16E-263	3.91E-264	11.32	13.94	13.79	2.6	2.7	2.65
OsR498G0204151800.01	0.756	0	-7.84984	1.10E-26	1.31E-26	0.76	0.7	0.81	0	0	0
OsR498G0204152600.01	11.63	0.03	-8.82631	0	0	11.61	12.07	11.21	0.03	0.06	0
OsR498G0204154400.01	0.04	2.013	5.563322	9.34E-46	8.76E-46	0.06	0.03	0.03	1.64	2.23	2.17
OsR498G0204155600.01	0.916	7.85	3.094473	4.72E-65	3.69E-65	0.92	0.87	0.96	9.37	6.94	7.24
OsR498G0204155800.01	19.486	157.14	3.030686	0	0	20.62	19.97	17.87	153.28	160.76	157.39
OsR498G0204157800.01	2.34	5.68	1.275209	1.42E-30	1.58E-30	2.48	2.17	2.37	6.04	5.65	5.35
OsR498G0204160200.01	23.876	70.97	1.579597	0	0	24.92	23.38	23.33	69.66	70.27	72.98
OsR498G0204163400.01	16.266	40.883	1.323627	2.61E-201	1.06E-201	16.78	15.63	16.39	39.52	41.04	42.09
OsR498G0204164000.01	0.12	0.446	1.987293	0.000135	0.0003042	0.1	0.17	0.09	0.44	0.6	0.3
OsR498G0204164900.01	14.576	43.353	1.540503	0	0	14.7	13.54	15.49	42.49	43.68	43.89
OsR498G0204165200.01	8.883	587.28	6.031836	0	0	8.42	8.87	9.36	622.97	571.25	567.62
OsR498G0204166900.01	12.87	66.203	2.390304	0	0	12.02	12.99	13.6	69.31	64.77	64.53
OsR498G0204167700.01	0.396	3.276	3.064422	5.28E-83	3.60E-83	0.49	0.49	0.21	3.33	3.22	3.28
OsR498G0204171700.01	5.413	3.146	-1.15929	9.51E-64	7.51E-64	5.33	4.93	5.98	3.08	3.32	3.04
OsR498G0204172200.01	3.623	0.703	-2.36996	7.71E-30	8.73E-30	4	3.1	3.77	0.85	0.72	0.54
OsR498G0204172500.01	0.11	1.643	3.847115	1.01E-33	1.09E-33	0.06	0.06	0.21	1.57	1.72	1.64
OsR498G0204173100.01	0.083	1.84	4.445752	1.99E-49	1.79E-49	0.13	0.05	0.07	1.91	1.83	1.78
OsR498G0204173800.01	1.913	0.133	-3.86713	4.74E-20	6.40E-20	1.72	1.87	2.15	0	0.07	0.33
OsR498G0204175500.01	0.793	19.293	4.506078	9.05E-286	2.89E-286	0.61	0.9	0.87	19.28	18.69	19.91
OsR498G0204183100.01	5.113	2.043	-1.3285	2.66E-37	2.73E-37	5.12	4.55	5.67	1.96	1.98	2.19
OsR498G0204183600.01	2.583	15.213	2.551689	1.24E-256	4.27E-257	2.55	2.4	2.8	16.7	14.96	13.98
OsR498G0204184100.01	231.02	15.276	-3.92998	0	0	236.01	231.37	225.7	15.59	15.31	14.93
OsR498G0204185600.01	24.996	51.623	1.02861	1.11E-178	4.88E-179	24.5	24.62	25.87	51.96	52.35	50.56
OsR498G0204186700.01	1.726	0.283	-2.6041	1.69E-12	2.73E-12	1.79	1.59	1.8	0.35	0.14	0.36
OsR498G0204188600.01	0.16	2.586	4.351346	9.43E-33	1.03E-32	0.1	0.2	0.18	2.75	2.24	2.77
OsR498G0204189200.01	0.093	7.873	6.411319	1.65E-99	1.02E-99	0	0.17	0.11	7.91	7.41	8.3
OsR498G0204190000.01	3.923	0.596	-2.73424	1.96E-27	2.30E-27	4.6	3.46	3.71	0.35	0.87	0.57
OsR498G0204192200.01	6.39	57.74	3.17513	0	0	5.85	7.43	5.89	60.39	57.09	55.74
OsR498G0204192400.01	17.82	405.98	4.505407	0	0	16.24	18.53	18.69	416.35	408.93	392.66
OsR498G0204193000.01	0.39	4.36	3.467997	6.11E-37	6.30E-37	0.34	0.43	0.4	4.71	4.71	3.66
OsR498G0204193200.01	3.303	32.263	3.276799	1.04E-242	3.73E-243	3.53	3.11	3.27	32.41	32.52	31.86
OsR498G0204196300.01	37.483	90.85	1.273922	0	0	36.17	39.46	36.82	93.76	89.73	89.06
OsR498G0204197700.01	3.006	7.796	1.327562	3.94E-35	4.16E-35	3.46	2.61	2.95	7.27	8.84	7.28
OsR498G0204202000.01	2.366	0.94	-1.31835	3.26E-20	4.38E-20	1.89	2.73	2.48	0.91	0.84	1.07
OsR498G0204203300.01	0.856	0.39	-1.12199	1.25E-07	2.39E-07	0.9	0.98	0.69	0.35	0.22	0.6
OsR498G0204203500.01	41.543	9.036	-2.20024	1.57E-244	5.61E-245	42.68	42.52	39.43	9.88	7.93	9.3
OsR498G0204203700.01	1.446	0.133	-3.4838	1.85E-10	3.19E-10	0.93	2.06	1.35	0.1	0.2	0.1
OsR498G0204204000.01	18.24	8.366	-1.18527	2.15E-54	1.84E-54	18.71	17.47	18.54	8.34	8.37	8.39
OsR498G0204207000.01	17.373	1.203	-3.85652	2.38E-274	7.82E-275	18.28	16.88	16.96	1.36	1.21	1.04
OsR498G0204209400.01	5.44	0.456	-3.56839	1.86E-85	1.25E-85	5.86	4.59	5.87	0.37	0.49	0.51
OsR498G0204210500.01	0.606	0.283	-1.09149	1.60E-07	3.08E-07	0.61	0.64	0.57	0.24	0.34	0.27
OsR498G0204211300.01	19.973	6.993	-1.81801	1.57E-157	7.43E-158	20.13	20.8	18.99	7.39	6	7.59
OsR498G0204218000.01	0	0.406	5.209685	4.72E-06	9.74E-06	0	0	0	0.2	0.48	0.54
OsR498G0204218200.01	3.083	9.906	1.684906	6.25E-84	4.24E-84	3.3	3.11	2.84	9.47	10.49	9.76
OsR498G0204218400.01	3.743	1.843	-1.01057	7.53E-28	8.80E-28	3.78	3.66	3.79	1.78	1.7	2.05
OsR498G0204218900.01	46.436	200.14	2.089143	0	0	49.27	45.18	44.86	198.82	203.12	198.47
OsR498G0204223200.01	0.98	3.013	1.540161	3.69E-12	6.04E-12	1.01	0.65	1.28	2.51	3.04	3.49
OsR498G0204223300.01	225.29	64.866	-1.75412	0	0	221.51	224.17	230.2	66.77	62.79	65.04
OsR498G0204224400.01	70.606	222.52	1.684643	0	0	70.37	69.43	72.02	232.9	221.9	212.75
OsR498G0204225600.01	5.843	0.11	-5.79772	1.65E-81	1.14E-81	4.94	7.09	5.5	0	0.22	0.11
OsR498G0204228500.01	1.98	0.623	-1.68214	1.43E-22	1.84E-22	1.87	1.84	2.23	0.69	0.67	0.51
OsR498G0204231800.01	29.233	11.82	-1.30932	1.09E-135	5.63E-136	31.25	26.8	29.65	12.27	11.7	11.49
OsR498G0204236200.01	55.586	11.613	-2.36467	0	0	57.95	55.87	52.94	12.52	10.57	11.75

OsR498G0204243900.01	6.75	2.2	-1.6041	1.70E-66	1.31E-66	6.81	6.62	6.82	1.89	2.37	2.34
OsR498G0204252100.01	0.143	0.02	-2.83471	3.45E-05	7.49E-05	0.1	0.17	0.16	0.04	0	0.02
OsR498G0204252400.01	37.93	107.73	1.508315	0	0	36.72	38.69	38.38	105.86	108.87	108.47
OsR498G0204253300.01	23.956	52.9	1.247882	0	0	23.03	24.53	24.31	49.96	54.32	54.42
OsR498G0204253500.01	35.206	8.603	-1.95073	0	0	35.81	34.33	35.48	7.71	8.65	9.45
OsR498G0204255200.01	3.943	0.57	-2.78549	1.10E-67	8.42E-68	4.05	4.3	3.48	0.66	0.51	0.54
OsR498G0204255800.01	0.653	0.086	-2.89884	3.87E-09	6.98E-09	0.68	0.71	0.57	0.09	0.04	0.13
OsR498G0204256200.01	1.913	0.263	-2.84777	3.13E-42	3.05E-42	1.79	2.01	1.94	0.22	0.3	0.27
OsR498G0204267400.01	4.356	10.956	1.324011	4.69E-34	5.02E-34	4.75	4.13	4.19	13.77	9.99	9.11
OsR498G0204272400.01	1.103	0.44	-1.33875	1.46E-05	3.10E-05	1.09	0.78	1.44	0.57	0.42	0.33
OsR498G0204274200.01	1.42	0.183	-2.96024	5.04E-20	6.82E-20	1.48	1.39	1.39	0.31	0.08	0.16
OsR498G0204275600.01	18.463	5.973	-1.64352	1.02E-154	4.90E-155	20.21	17.45	17.73	6.4	6.12	5.4
OsR498G0204275900.01	56.323	1.643	-5.0934	0	0	58.18	54.63	56.16	1.6	1.74	1.59
OsR498G0204278200.01	0.32	0.02	-4.46232	9.14E-07	1.82E-06	0.27	0.43	0.26	0	0	0.06
OsR498G0204280300.01	0.633	3.89	2.616685	4.13E-28	4.80E-28	0.5	0.79	0.61	4.3	3.77	3.6
OsR498G0204285300.01	531.52	1872.8	1.813222	0	0	517.55	541.68	535.3	1873.7	1890.1	1854.5
OsR498G0204286200.01	6.9	3.143	-1.16733	5.51E-59	4.54E-59	7.03	6.92	6.75	3.15	3.06	3.22
OsR498G0204287700.01	7.04	17.343	1.299456	2.05E-90	1.34E-90	6.82	7.96	6.34	16.91	17.18	17.94
OsR498G0204288800.01	0.96	6.363	2.721584	2.01E-25	2.45E-25	0.83	1.25	0.8	6.74	6.88	5.47
OsR498G0204291800.01	0.29	1.623	2.480333	1.29E-17	1.83E-17	0.41	0.3	0.16	2.02	1.26	1.59
OsR498G0204292600.01	36.193	710.32	4.291145	0	0	35.91	36.87	35.8	747.67	704.8	678.49
OsR498G0204292900.01	1.146	3.016	1.369196	9.48E-20	1.29E-19	1.19	1.21	1.04	2.82	2.98	3.25
OsR498G0204296100.01	0.543	1.146	1.087066	0.0002045	0.0004667	0.43	0.73	0.47	1.65	1.01	0.78
OsR498G0204298800.01	29.3	130.32	2.153634	0	0	29.44	29.29	29.17	128.89	130.87	131.2
OsR498G0204299400.01	0.936	2.79	1.569581	2.16E-19	2.97E-19	0.95	0.81	1.05	2.95	2.65	2.77
OsR498G0204300700.01	0.873	5.646	2.855756	1.49E-174	6.61E-175	0.63	0.85	1.14	5.5	5.34	6.1
OsR498G0204309100.01	0.08	0.51	2.702725	0.0001213	0.0002726	0.08	0.16	0	0.48	0.73	0.32
OsR498G0204310300.01	0.083	1.273	3.960499	7.47E-47	6.92E-47	0.21	0.02	0.02	1.71	1.49	0.62
OsR498G0204310500.01	0.436	0.196	-1.14012	5.07E-05	0.0001111	0.59	0.39	0.33	0.11	0.24	0.24
OsR498G0204322200.01	0.063	0.31	2.03976	2.94E-05	6.36E-05	0.02	0.1	0.07	0.29	0.39	0.25
OsR498G0204322800.01	0.856	0.09	-3.2031	1.23E-26	1.47E-26	0.61	1.07	0.89	0.11	0.09	0.07
OsR498G0204324000.01	1.463	0.646	-1.17895	1.66E-20	2.22E-20	1.46	1.56	1.37	0.68	0.61	0.65
OsR498G0204325700.01	0.68	1.816	1.407386	9.34E-20	1.27E-19	0.62	0.72	0.7	1.81	1.78	1.86
OsR498G0204331600.01	0.933	3.386	1.807394	2.49E-49	2.25E-49	0.76	1.02	1.02	3.18	3.44	3.54
OsR498G0204333300.01	13.663	40.61	1.563007	6.16E-131	3.26E-131	15.16	13.49	12.34	41.58	39.99	40.26
OsR498G0204336600.01	10.826	74.123	2.720356	0	0	11.31	10.11	11.06	74.82	73.71	73.84
OsR498G0204338000.01	0.886	0.143	-2.64658	1.62E-19	2.22E-19	0.93	0.98	0.75	0.12	0.11	0.2
OsR498G0204339500.01	60.826	27.086	-1.16099	6.98E-177	3.09E-177	65.24	57.78	59.46	26.67	26.33	28.26
OsR498G0204346000.01	0.116	20.163	7.446319	5.56E-228	2.09E-228	0.06	0.18	0.11	21.1	19.17	20.22
OsR498G0204347900.01	5.226	22.86	2.165997	1.12E-306	3.35E-307	5.54	5.14	5	22.49	23.6	22.49
OsR498G0204349900.01	13.73	39.623	1.529854	0	0	13.08	14.47	13.64	36.76	43.14	38.97
OsR498G0204350100.01	0.08	0.41	2.361688	1.06E-05	2.23E-05	0.08	0.08	0.08	0.4	0.33	0.5
OsR498G0204350900.01	1.71	34.583	4.331846	0	0	1.21	1.94	1.98	36.98	34.61	32.16
OsR498G0204354400.01	16.886	126.64	2.904483	0	0	16.94	16.91	16.81	135.27	125.44	119.21
OsR498G0204354900.01	0.086	2.786	5.009923	6.45E-90	4.22E-90	0.11	0.11	0.04	2.9	2.53	2.93
OsR498G0204355400.01	2.256	6.633	1.552605	1.35E-67	1.03E-67	1.9	2.29	2.58	7.19	6.34	6.37
OsR498G0204358400.01	2.113	0.883	-1.26055	1.18E-17	1.68E-17	2.1	2.28	1.96	0.79	0.96	0.9
OsR498G0204358700.01	0.823	0.286	-1.37528	0.0003652	0.0008492	1.07	0.36	1.04	0.08	0.37	0.41
OsR498G0204360900.01	2.286	0.366	-2.63635	4.06E-44	3.88E-44	2.23	2.36	2.27	0.38	0.29	0.43
OsR498G0204362700.01	0.163	1.626	3.361688	3.37E-11	5.68E-11	0.19	0.2	0.1	1.93	1.57	1.38
OsR498G0204363000.01	0.06	1.676	4.881062	6.88E-22	8.94E-22	0.06	0.12	0	1.67	1.48	1.88
OsR498G0204364800.01	1.136	11.196	3.282525	0	0	1.22	0.97	1.22	11.22	10.75	11.62
OsR498G0204369900.01	20.363	41.493	1.02594	7.50E-148	3.69E-148	19.8	20.6	20.69	40.98	40.33	43.17
OsR498G0204373400.01	0.06	0	-4.21725	0.0009328	0.0022397	0.03	0.05	0.1	0	0	0
OsR498G0204376600.01	1.19	0.22	-2.43005	7.21E-27	8.56E-27	1.01	1.5	1.06	0.17	0.29	0.2
OsR498G0204377400.01	49.546	8.023	-2.69308	0	0	48.56	49.83	50.25	7.48	8.35	8.24
OsR498G0204378000.01	6.006	0.766	-2.97244	1.93E-167	8.77E-168	6.07	6.15	5.8	0.76	0.76	0.78
OsR498G0204378400.01	12.95	4.45	-1.48522	8.34E-114	4.78E-114	14.44	11.28	13.13	5.73	3.84	3.78
OsR498G0204380300.01	24.266	8.49	-1.50948	4.40E-218	1.70E-218	24.4	23.63	24.77	8.36	8.86	8.25
OsR498G0204380700.01	2.073	18.246	3.121737	1.15E-93	7.30E-94	2	1.9	2.32	19.88	14.86	20
OsR498G0204381000.01	0.063	0.586	3.209685	1.18E-06	2.36E-06	0	0.13	0.06	0.64	0.53	0.59
OsR498G0204382100.01	133.06	298.51	1.191505	0	0	140.18	129.7	129.3	320.35	285.59	289.6
OsR498G0204383500.01	32.486	5.71	-2.51669	0	0	31.85	30.59	35.02	6.17	5.96	5
OsR498G0204386200.01	5.45	1.45	-1.91893	6.46E-49	5.86E-49	5.44	5.56	5.35	1.41	1.85	1.09
OsR498G0204387200.01	5.833	12.733	1.121177	3.31E-42	3.22E-42	5.9	6.65	4.95	13.47	12.28	12.45
OsR498G0204388300.01	0.47	1.73	1.838126	2.03E-09	3.62E-09	0.36	0.62	0.43	2.05	1.12	2.02
OsR498G0204391000.01	9.72	19.81	1.017442	4.55E-71	3.39E-71	8.95	9.95	10.26	19.52	19.68	20.23
OsR498G0204391200.01	9.113	19.436	1.088434	8.20E-120	4.56E-120	8.76	8.67	9.91	19.75	18.38	20.18
OsR498G0204392200.01	295.49	115.24	-1.36988	0	0	304.77	288.31	293.4	122.5	117.12	106.1

OsR498G0204392800.01	0.84	14.14	4.076102	0	0	0.89	0.99	0.64	15.69	13.37	13.36
OsR498G0204399600.01	0	0.116	4.7402	9.30E-05	0.0002074	0	0	0	0.16	0.11	0.08
OsR498G0204402500.01	0.06	0.793	3.7402	2.07E-12	3.35E-12	0	0.12	0.06	0.96	0.7	0.72
OsR498G0204404000.01	4.19	0.5	-3.07572	8.19E-37	8.45E-37	4.19	4.07	4.31	0.72	0.31	0.47
OsR498G0204407200.01	68.67	149.54	1.086981	0	0	71.54	67.74	66.73	149.78	147.53	151.32
OsR498G0204410500.01	3.683	16.443	2.151268	2.89E-86	1.93E-86	3.53	3.98	3.54	18.06	17.08	14.19
OsR498G0204410700.01	0.286	0.033	-3.13016	7.72E-05	0.0001712	0.28	0.49	0.09	0	0.05	0.05
OsR498G0204416700.01	3.33	7.913	1.242208	5.73E-49	5.19E-49	3.32	3.04	3.63	8.7	8.15	6.89
OsR498G0204418800.01	0.623	0	-6.86713	2.23E-15	3.34E-15	0.61	0.67	0.59	0	0	0
OsR498G0204420900.01	5.646	0.146	-5.20817	4.61E-124	2.51E-124	5.95	5.62	5.37	0.15	0.03	0.26
OsR498G0204424300.01	3.16	1.54	-1.05646	0.0003222	0.0007464	2.58	3.53	3.37	1.74	1.76	1.12
OsR498G0204424900.01	0.16	1.31	3.023272	1.16E-16	1.69E-16	0.31	0.09	0.08	1.42	1.24	1.27
OsR498G0204425200.01	25.946	55.606	1.080249	1.85E-207	7.40E-208	25.17	25.81	26.86	57.58	55.23	54.01
OsR498G0204430300.01	0.066	1.593	4.616109	5.91E-40	5.89E-40	0	0.12	0.08	1.46	1.71	1.61
OsR498G0204432800.01	10.37	2.496	-2.05314	1.84E-51	1.62E-51	10.88	8.95	11.28	3.03	2.19	2.27
OsR498G0204433400.01	0.526	0	-5.91444	4.87E-09	8.81E-09	0.41	0.48	0.69	0	0	0
OsR498G0204435000.01	0.28	0.06	-2.13016	0.0009788	0.0023546	0.18	0.25	0.41	0.18	0	0
OsR498G0204439300.01	44.916	132.9	1.559597	0	0	44.2	45.91	44.64	135.29	132.89	130.52
OsR498G0204440700.01	4.5	17.5	1.955951	9.48E-210	3.76E-210	4.42	4.57	4.51	18.68	18.16	15.66
OsR498G0204441700.01	0.136	0.013	-3.35256	1.36E-05	2.87E-05	0.1	0.16	0.15	0	0.02	0.02
OsR498G0204443200.01	0.706	50.863	6.163331	0	0	0.85	0.75	0.52	53.29	50.63	48.67
OsR498G0204445300.01	1.746	0.5	-1.8027	3.46E-11	5.83E-11	1.35	2.25	1.64	0.71	0.37	0.42
OsR498G0204447000.01	0.79	0.07	-3.45667	1.44E-16	2.10E-16	0.66	0.84	0.87	0.12	0.03	0.06
OsR498G0204458200.01	5.593	12.563	1.212227	1.04E-52	9.08E-53	4.78	6.3	5.7	12.42	12.9	12.37
OsR498G0204462100.01	2.793	0.356	-2.98014	9.04E-14	1.41E-13	2.28	3.24	2.86	0.47	0.36	0.24
OsR498G0204464600.01	3.373	0.073	-5.53226	4.91E-79	3.44E-79	3.08	4.27	2.77	0.07	0.09	0.06
OsR498G0204466200.01	0.593	0.08	-2.96024	0.000243	0.0005585	0.78	0.35	0.65	0	0.12	0.12
OsR498G0204467100.01	0.293	3.02	3.354802	7.59E-41	7.49E-41	0.18	0.22	0.48	2.98	3.12	2.96
OsR498G0204470900.01	25.316	53.66	1.036967	2.47E-227	9.30E-228	26.12	24.08	25.75	55.41	53	52.57
OsR498G0204472600.01	0.54	2.833	2.40676	6.31E-188	2.69E-188	0.48	0.6	0.54	2.7	2.88	2.92
OsR498G0204474000.01	0.04	0.626	4.073183	5.90E-30	6.66E-30	0.09	0.02	0.01	0.68	0.72	0.48
OsR498G0204474300.01	33.576	115.04	1.773424	0	0	32.98	33.96	33.79	117	111.94	116.18
OsR498G0204477800.01	5.793	0.153	-5.2708	1.30E-31	1.44E-31	6.57	6.5	4.31	0	0	0.46
OsR498G0204482000.01	4.8	2.056	-1.10982	2.79E-13	4.41E-13	5.81	4.73	3.86	2	1.91	2.26
OsR498G0204487100.01	1.646	0.696	-1.18851	9.55E-06	2.01E-05	1.63	1.37	1.94	0.82	0.5	0.77
OsR498G0204488200.01	0.883	0.093	-3.28217	2.44E-14	3.76E-14	1.02	0.87	0.76	0	0.12	0.16
OsR498G0204488400.01	32.253	77.033	1.248629	3.49E-299	1.07E-299	28.96	33.51	34.29	79.35	76.59	75.16
OsR498G0204490700.01	5.023	41.88	3.060324	0	0	5.03	5.75	4.29	45.42	40.89	39.33
OsR498G0204492600.01	6.093	12.32	1.045895	6.84E-59	5.65E-59	5.4	6.64	6.24	12.16	12.21	12.59
OsR498G0204493300.01	16.42	7.05	-1.22421	1.01E-48	9.20E-49	18.56	16.09	14.61	8.14	6.62	6.39
OsR498G0204494300.01	0.093	0.37	1.987293	1.69E-07	3.26E-07	0.16	0.08	0.04	0.44	0.38	0.29
OsR498G0204495600.01	1.566	0.68	-1.19471	1.25E-09	2.22E-09	1.63	1.69	1.38	0.59	0.76	0.69
OsR498G0204500300.01	0.43	0.083	-2.37528	0.0009088	0.0021805	0.16	0.67	0.46	0.08	0.17	0
OsR498G0204501000.01	0.02	0.35	4.249213	5.73E-10	1.00E-09	0	0.03	0.03	0.28	0.4	0.37
OsR498G0204505500.01	9.86	21.253	1.102834	1.51E-143	7.57E-144	9.9	9.92	9.76	19.9	21.34	22.52
OsR498G0204505700.01	19.26	61.51	1.659912	1.76E-260	5.99E-261	19.92	19.23	18.63	58.17	62.71	63.65
OsR498G0204512100.01	0.76	0.133	-2.4838	7.36E-05	0.0001629	0.69	0.63	0.96	0.2	0.1	0.1
OsR498G0204516700.01	0	0.433	6.325162	4.31E-11	7.27E-11	0	0	0	0.49	0.34	0.47
OsR498G0204522800.01	20.846	5.13	-2.03417	5.61E-122	3.09E-122	22.53	18.77	21.24	4.8	5.35	5.24
OsR498G0204524700.01	102.18	362.62	1.842618	0	0	100.79	104.4	101.4	359.84	365.19	362.82
OsR498G0204531300.01	52.4	15.34	-1.73079	0	0	48.77	55.1	53.33	15.63	15.84	14.55
OsR498G0204531800.01	5.153	30.08	2.542514	4.71E-270	1.56E-270	5.02	5.46	4.98	34.64	29.06	26.54
OsR498G0204535200.01	1.956	13.893	2.829119	1.38E-267	4.63E-268	1.57	2.3	2	14.98	14.14	12.56
OsR498G0204536600.01	0.126	9.33	6.234517	8.96E-141	4.55E-141	0	0.29	0.09	10.28	8.88	8.83
OsR498G0204537800.01	2.566	7.053	1.451988	4.57E-48	4.18E-48	3.23	2.16	2.31	7.72	6.23	7.21
OsR498G0204545400.01	15.57	6.06	-1.36904	2.19E-54	1.88E-54	16.35	14.71	15.65	6.92	6.04	5.22
OsR498G0204545500.01	4.13	1.523	-1.44474	6.47E-16	9.58E-16	4.69	4.61	3.09	1.26	1.88	1.43
OsR498G0204546200.01	1.14	3.43	1.58133	6.73E-12	1.11E-11	0.98	1.27	1.17	3.87	2.79	3.63
OsR498G0204547300.01	0.113	0.296	1.340985	4.35E-05	9.50E-05	0.06	0.1	0.18	0.22	0.38	0.29
OsR498G0204548800.01	2.51	19.726	2.956664	0	0	2.65	2.12	2.76	19.79	18.88	20.51
OsR498G0204551100.01	2.8	6.726	1.262511	1.66E-46	1.55E-46	2.95	2.81	2.64	5.88	7	7.3
OsR498G0204555400.01	0.09	0.89	3.249213	1.51E-08	2.78E-08	0.07	0.07	0.13	1.14	0.66	0.87
OsR498G0204558400.01	24.396	55.736	1.186317	1.78E-216	6.93E-217	21.75	26.06	25.38	58.79	54.85	53.57
OsR498G0204564700.01	2.346	8.796	1.900076	1.99E-55	1.70E-55	2.78	2.17	2.09	9.44	9.26	7.69
OsR498G0204565100.01	0.676	0.073	-3.16969	3.21E-08	6.00E-08	0.72	0.46	0.85	0.11	0	0.11
OsR498G0204565900.01	26.266	69.99	1.410704	0	0	27.33	27.36	24.11	70.82	69.19	69.96
OsR498G0204567600.01	8.56	30.95	1.840609	2.70E-253	9.39E-254	9	7.73	8.95	33.01	29.58	30.26
OsR498G0204568600.01	11.803	4.716	-1.35031	1.72E-25	2.08E-25	11.26	12.45	11.7	5.24	4.73	4.18
OsR498G0204568700.01	1.353	0.673	-1.23587	0.000423	0.0009888	1.15	1.71	1.2	0.57	0.58	0.87

OsR498G0204570000.01	1.043	3.073	1.548774	2.55E-07	4.96E-07	0.95	1.38	0.8	2.96	2.76	3.5
OsR498G0204575600.01	1.656	0.786	-1.65276	2.43E-19	3.33E-19	1.87	1.64	1.46	0.68	0.82	0.86
OsR498G0204579800.01	3.613	22.933	2.655917	1.12E-168	5.09E-169	3.43	3.53	3.88	24.13	22.2	22.47
OsR498G0204580300.01	1.456	22.576	3.942359	0	0	1.43	1.31	1.63	22	22.97	22.76
OsR498G0204582800.01	17.806	51.013	1.448115	8.87E-195	3.69E-195	17.72	18.12	17.58	53.54	47.53	51.97
OsR498G0204584000.01	25.656	7.54	-1.79261	6.51E-201	2.66E-201	27.48	23.71	25.78	7.22	7.5	7.9
OsR498G0204589700.01	5.716	14.08	1.305302	3.36E-147	1.66E-147	5.76	5.55	5.84	13.81	13.78	14.65
OsR498G0204590300.01	8.746	2.95	-1.58025	8.42E-30	9.53E-30	9.07	8.26	8.91	3.17	2.93	2.75
OsR498G0204592300.01	85.56	260.05	1.603034	0	0	83.91	86.42	86.35	258.43	258.24	263.47
OsR498G0204592600.01	5.51	28.37	2.353767	0	0	5.86	4.7	5.97	30.1	27.79	27.22
OsR498G0204593700.01	4295.7	1612.3	-1.41811	0	0	4389.1	4298.2	4200	1621.5	1584.2	1631.3
OsR498G0204597500.01	48.746	126.32	1.397557	0	0	49.44	51	45.8	122.11	130.16	126.7
OsR498G0204598100.01	0.316	2.91	3.20221	8.83E-71	6.60E-71	0.4	0.23	0.32	2.71	2.7	3.32
OsR498G0204599700.01	0.36	1.2	1.776726	1.96E-15	2.93E-15	0.33	0.38	0.37	1.5	1.12	0.98
OsR498G0204601200.01	0.746	0.166	-2.23033	1.44E-11	2.39E-11	0.85	0.57	0.82	0.19	0.17	0.14
OsR498G0204604100.01	62.686	24.956	-1.33486	1.55E-275	5.08E-276	64.59	59.84	63.63	24.56	25.78	24.53
OsR498G0204605900.01	0.106	2.156	4.287688	1.93E-14	2.97E-14	0.11	0	0.21	2.13	2.52	1.82
OsR498G0204607500.01	1.28	0.166	-3.02633	4.72E-24	5.89E-24	1.23	1.25	1.36	0.17	0.09	0.24
OsR498G0204608000.01	18.716	39.45	1.071003	0	0	17.35	19.22	19.58	38.64	39.46	40.25
OsR498G0204609700.01	4.726	16.086	1.767909	3.31E-143	1.66E-143	4.56	5.42	4.2	16.42	15.71	16.13
OsR498G0204611500.01	1.953	6.393	1.7119	5.42E-56	4.58E-56	1.74	2.3	1.82	6.44	6.39	6.35
OsR498G0204612300.01	284.83	6.03	-5.56699	0	0	283.62	283.4	287.5	5.47	6.33	6.29
OsR498G0204612600.01	39.513	0.42	-6.57936	0	0	35.15	38.68	44.71	0.24	0.43	0.59
OsR498G0204615400.01	106.78	336.49	1.69499	0	0	106.99	102.87	110.5	347.12	339.82	322.54
OsR498G0204615900.01	4.553	0.856	-2.3998	5.06E-81	3.51E-81	4.93	4.28	4.45	0.7	1	0.87
OsR498G0204616300.01	10.186	58.973	2.518895	0	0	9.73	9.2	11.63	57.49	59.69	59.74
OsR498G0204616700.01	0.096	1.043	3.443991	2.76E-25	3.36E-25	0.05	0.09	0.15	1.06	0.89	1.18
OsR498G0204618400.01	24.803	62.506	1.321684	0	0	24.44	25.25	24.72	59.78	64.3	63.44
OsR498G0204619500.01	157.58	20.573	-2.29671	0	0	160.13	156.9	155.7	20.8	21.93	18.99
OsR498G0204621800.01	0.026	0.193	2.847115	0.0005624	0.0013266	0.04	0	0.04	0.16	0.17	0.25
OsR498G0204622400.01	254.22	97.373	-1.3822	0	0	257.97	253.27	251.4	96.26	96.55	99.31
OsR498G0204625500.01	30.01	6.493	-2.21301	0	0	30.75	29.57	29.71	6.09	6.95	6.44
OsR498G0204625700.01	41.56	17.17	-1.2862	0	0	41.94	40.27	42.47	16.68	16.71	18.12
OsR498G0204626200.01	3.996	1.026	-1.97697	1.78E-63	1.41E-63	4.03	4.14	3.82	0.66	1.18	1.24
OsR498G0204628700.01	4.776	0.093	-5.6926	1.32E-79	9.25E-80	5.58	3.97	4.78	0.09	0.1	0.09
OsR498G0204629400.01	38.543	16.863	-1.19683	2.70E-169	1.22E-169	40.53	38.85	36.25	16.58	17.31	16.7
OsR498G0204635700.01	3.076	11.043	1.852179	4.25E-105	2.54E-105	2.8	3.22	3.21	10.95	11.89	10.29
OsR498G0204636000.01	0.13	0.456	1.808305	0.0002036	0.0004644	0.19	0.14	0.06	0.22	0.49	0.66
OsR498G0204637500.01	1.983	0.88	-1.24035	2.84E-13	4.49E-13	1.62	2.38	1.95	0.63	1.07	0.94
OsR498G0204639600.01	2.933	8.32	1.500072	5.94E-31	6.61E-31	1.7	3.7	3.4	10.32	7.6	7.04
OsR498G0204640800.01	0.103	5.66	5.778112	2.30E-167	1.05E-167	0.05	0.12	0.14	6.56	5.08	5.34
OsR498G0204641800.01	0.013	1.01	6.155237	2.52E-51	2.23E-51	0.01	0.03	0	1	0.96	1.07
OsR498G0204645000.01	0.023	0.233	3.361688	1.61E-05	3.42E-05	0	0.07	0	0.21	0.32	0.17
OsR498G0204646800.01	0.2	0.946	2.217298	2.39E-13	3.77E-13	0.13	0.13	0.34	1.01	0.91	0.92
OsR498G0204647600.01	0.54	0.11	-2.30419	5.60E-10	9.81E-10	0.73	0.49	0.4	0.22	0.08	0.03
OsR498G0204653400.01	74.416	14.283	-2.40526	0	0	74.64	73.27	75.34	15.62	14.15	13.08
OsR498G0204653800.01	0.05	0.41	2.54226	1.24E-06	2.49E-06	0.05	0.05	0.05	0.5	0.22	0.51
OsR498G0204654900.01	1.97	0.556	-2.07066	4.13E-12	6.77E-12	2.04	1.39	2.48	0.26	0.85	0.56
OsR498G0204656700.01	3.713	46.273	3.367632	0	0	3.69	4.04	3.41	44.97	46.89	46.96
OsR498G0204657000.01	1.653	18.753	3.507754	0	0	1.43	1.71	1.82	19.47	17.47	19.32
OsR498G0204658200.01	2.376	117.8	5.608695	0	0	2.7	1.82	2.61	126.74	111.86	114.8
OsR498G0204658500.01	3.9	308.33	6.295302	0	0	4.15	4.64	2.91	340.85	313.28	270.85
OsR498G0204659500.01	105.72	236.57	1.156159	0	0	105.45	102.22	109.5	234.11	234.84	240.77
OsR498G0204660100.01	14.426	30.273	1.049373	9.70E-179	4.27E-179	14.8	14.06	14.42	29.7	30.08	31.04
OsR498G0204661900.01	0.573	0.07	-3.07572	3.63E-06	7.46E-06	0.2	0.56	0.96	0.14	0.07	0
OsR498G0204663200.01	0.51	2.246	2.121554	2.11E-16	3.09E-16	0.57	0.44	0.52	2.28	2.24	2.22
OsR498G0204663700.01	0.11	7.41	5.993956	3.16E-46	2.95E-46	0	0	0.33	5.78	8.19	8.26
OsR498G0204664300.01	0.696	3.633	2.361688	5.29E-54	4.55E-54	0.74	0.58	0.77	3.7	4.1	3.1
OsR498G0204664600.01	0.21	0.933	2.180241	2.40E-13	3.78E-13	0.34	0.15	0.14	0.96	1.09	0.75
OsR498G0204665200.01	3.42	7.223	1.055163	4.08E-48	3.73E-48	3.57	3.21	3.48	6.77	7.18	7.72
OsR498G0204672700.01	0.97	0.183	-2.37528	4.43E-12	7.26E-12	0.93	0.96	1.02	0.17	0.18	0.2
OsR498G0204673000.01	9.586	22.263	1.247868	1.81E-91	1.17E-91	9.94	9.36	9.46	21.57	21.32	23.9
OsR498G0204674100.01	61.8	142.97	1.234481	0	0	62.89	60.94	61.57	141.05	141.79	146.08
OsR498G0204677500.01	0.283	0.026	-4.0477	2.60E-05	5.61E-05	0.39	0.17	0.29	0	0	0.08
OsR498G0204681300.01	3.653	1.77	-1.06917	8.69E-20	1.18E-19	3.29	3.66	4.01	1.68	1.4	2.23
OsR498G0204682900.01	10.63	1.2	-3.339	5.19E-212	2.04E-212	10.28	10.87	10.74	1.12	1.16	1.32
OsR498G0204687800.01	38.446	13.1	-1.56438	1.77E-190	7.51E-191	38.53	38.21	38.6	13.97	12.59	12.74
OsR498G0204689800.01	0.396	10.896	4.762662	5.46E-34	5.85E-34	0.46	0.51	0.22	12.52	10.82	9.35
OsR498G0204690000.01	3.143	0.713	-2.12512	2.83E-81	1.96E-81	2.47	3.46	3.5	0.75	0.69	0.7

OsR498G0204693400.01	22.74	55.966	1.226529	0	0	21.63	23.5	23.09	54.04	57.53	56.33
OsR498G0204696500.01	1.696	9.78	2.495989	1.36E-95	8.58E-96	1.63	1.79	1.67	9.64	9.93	9.77
OsR498G0204697000.01	0.2	0.773	1.972646	3.59E-06	7.37E-06	0.11	0.28	0.21	1.03	0.79	0.5
OsR498G0204698800.01	185.8	67.033	-1.41983	0	0	193.95	178.27	185.2	67.43	68.3	65.37
OsR498G0204702700.01	1.02	10.786	3.390982	1.10E-76	7.84E-77	1.41	0.82	0.83	12.43	9.97	9.96
OsR498G0204704700.01	7.803	54.533	2.808741	0	0	7.62	7.56	8.23	53.04	55.31	55.25
OsR498G0204706700.01	15.613	3.67	-2.26147	3.01E-193	1.26E-193	15.93	15.43	15.48	3.47	3.56	3.98
OsR498G0204708400.01	0.07	2.146	4.594349	2.79E-23	3.53E-23	0.07	0.05	0.09	2.27	2.08	2.09
OsR498G0204708800.01	0.976	0.256	-1.93556	5.39E-12	8.86E-12	1.04	0.75	1.14	0.38	0.12	0.27
OsR498G0204712100.01	0.24	0.02	-3.66068	0.0002846	0.0006567	0.33	0.23	0.16	0.06	0	0
OsR498G0204714500.01	67.623	11.446	-2.56684	0	0	68.59	67.05	67.23	11.08	10.73	12.53
OsR498G0204714800.01	1.756	0.24	-2.89098	1.64E-11	2.74E-11	1.81	1.63	1.83	0.18	0.18	0.36
OsR498G0204715700.01	5.523	2.6	-1.08223	3.71E-14	5.74E-14	5.36	5.77	5.44	2.28	2.93	2.59
OsR498G0204717000.01	0.35	1.566	2.136622	9.72E-11	1.66E-10	0.34	0.3	0.41	1.38	1.8	1.52
OsR498G0204717700.01	12.973	4.9	-1.43173	1.91E-69	1.45E-69	13.81	12.84	12.27	5.02	4.53	5.15
OsR498G0204717900.01	0.033	1.936	5.847115	2.99E-15	4.50E-15	0.1	0	0	1.95	1.99	1.87
OsR498G0204720200.01	1.88	6.963	1.87975	4.89E-25	5.99E-25	1.49	2.26	1.89	6.29	7.52	7.08
OsR498G0204722300.01	20.75	67.04	1.655377	1.31E-180	5.71E-181	20.14	20.74	21.37	71.96	66.1	63.06
OsR498G0204722500.01	0.34	0.99	1.525187	6.72E-07	1.33E-06	0.37	0.17	0.48	1.21	0.86	0.9
OsR498G0204725600.01	5.73	2.39	-1.26305	1.19E-43	1.14E-43	5.79	5.83	5.57	2.31	2.22	2.64
OsR498G0204727300.01	7.063	19.65	1.482132	6.91E-265	2.32E-265	6.78	7.44	6.97	19.06	20.24	19.65
OsR498G0204727600.01	0.25	4.16	3.974871	5.51E-89	3.62E-89	0.37	0.18	0.2	4.19	4.55	3.74
OsR498G0204727800.01	18.056	138.78	2.930775	0	0	18.84	18.4	16.93	143.96	134.53	137.85
OsR498G0204728100.01	6.786	16.143	1.674205	0	0	6.64	6.56	7.16	15.68	16.75	16
OsR498G0204728400.01	0	14.316	10.01526	5.79E-85	3.90E-85	0	0	0	12.02	15.31	15.62
OsR498G0204728500.01	0.023	2.663	6.906571	1.16E-19	1.57E-19	0	0.07	0	2.39	1.21	4.39
OsR498G0204729000.01	0.186	9.513	5.658146	1.36E-95	8.59E-96	0	0.22	0.34	9.51	9.12	9.91
OsR498G0204729800.01	0.993	4.063	2.011746	9.23E-13	1.48E-12	0.69	0.96	1.33	4.49	3.02	4.68
OsR498G0204732200.01	0.323	9.33	4.815488	3.28E-179	1.44E-179	0.42	0.18	0.37	10.1	9.83	8.06
OsR498G0204732500.01	0.746	14.02	4.215266	2.31E-171	1.04E-171	0.78	0.55	0.91	12.86	14.87	14.33
OsR498G0204733700.01	8.696	18.906	1.117233	4.08E-151	1.98E-151	8.18	8.89	9.02	17.65	19.94	19.13
OsR498G0204735300.01	1.203	15.826	3.711071	0	0	0.99	1.25	1.37	16.59	16.28	14.61
OsR498G0204736200.01	10.33	23.063	1.159965	3.20E-203	1.30E-203	10.37	10.97	9.65	22.21	23.45	23.53
OsR498G0204737000.01	0.993	10.526	3.398327	0	0	1.13	0.89	0.96	10.84	10.51	10.23
OsR498G0204738600.01	0.466	43.133	6.530867	0	0	0.39	0.57	0.44	45.26	41.91	42.23
OsR498G0204741800.01	0.106	0.006	-3.86713	8.60E-05	0.0001913	0.15	0.07	0.1	0	0.02	0
OsR498G0204743200.01	0.033	1.713	5.604545	9.13E-36	9.57E-36	0	0.07	0.03	1.67	2.24	1.23
OsR498G0204743700.01	2.186	4.99	1.178736	1.88E-11	3.14E-11	2.42	2.01	2.13	4.28	4.87	5.82
OsR498G0204744000.01	6.743	62.933	3.200861	4.55E-264	1.53E-264	8.24	5.58	6.41	65.44	64.74	58.62
OsR498G0204744400.01	0.576	22.09	5.253793	0	0	0.34	0.57	0.82	22.29	22.37	21.61
OsR498G0204745100.01	9.553	20.053	1.093156	4.00E-84	2.70E-84	9.93	8.65	10.08	20.71	19.5	19.95
OsR498G0204746400.01	0.44	0.06	-2.96024	1.12E-05	2.37E-05	0.49	0.46	0.37	0.06	0.06	0.06
OsR498G0204747800.01	40.903	15.603	-1.41199	3.70E-202	1.50E-202	42.2	40.3	40.21	15.41	15.69	15.71
OsR498G0204748300.01	30.753	89.96	1.54775	0	0	27.89	32.55	31.82	88.69	89.79	91.4
OsR498G0204753500.01	3.863	7.746	1.000766	9.53E-35	1.01E-34	3.62	3.95	4.02	7.94	7.31	7.99
OsR498G0204753800.01	0.38	3.676	3.274225	4.17E-31	4.63E-31	0.27	0.42	0.45	4.35	2.94	3.74
OsR498G0204755400.01	2.623	24.72	3.232341	0	0	2.64	2.62	2.61	24.57	25.47	24.12
OsR498G0204757700.01	8.02	2.456	-1.917	6.36E-50	5.70E-50	8.37	7.21	8.48	1.95	2.81	2.61
OsR498G0204761200.01	0.806	0.193	-2.18263	3.64E-13	5.78E-13	0.74	0.63	1.05	0.26	0.15	0.17
OsR498G0204761600.01	51.986	18.09	-1.52728	0	0	46.39	55.13	54.44	17.52	18	18.75
OsR498G0204762300.01	366.03	74.656	-2.2973	0	0	347.38	374.75	376	78.69	73.47	71.81
OsR498G0204764100.01	13.256	61.1	2.192808	0	0	12.74	13.1	13.93	63.71	62.67	56.92
OsR498G0204765300.01	0.806	3.636	2.170048	4.21E-44	4.02E-44	0.85	0.8	0.77	3.57	3.77	3.57
OsR498G0204766300.01	2.07	0.983	-1.12436	4.63E-10	8.09E-10	2.14	1.73	2.34	0.68	1.19	1.08
OsR498G0204771300.01	0.106	0.006	-3.66068	0.0002846	0.0006567	0.05	0.2	0.07	0	0	0.02
OsR498G0204772500.01	0.29	1.446	2.256078	1.30E-11	2.17E-11	0.27	0.17	0.43	1.56	0.94	1.84
OsR498G0204776600.01	5.153	115.82	4.487449	0	0	5.15	4.92	5.39	113.9	115.66	117.89
OsR498G0204778100.01	0.02	0.276	3.624723	1.45E-06	2.92E-06	0.03	0	0.03	0.24	0.28	0.31
OsR498G0204781600.01	6.433	15.856	1.284396	2.13E-127	1.14E-127	5.89	6.73	6.68	14.38	16.88	16.31
OsR498G0204782700.01	262.64	77.383	-1.76255	0	0	285.72	245.64	256.6	83.87	75.35	72.93
OsR498G0204783300.01	10.11	4.63	-1.13156	4.58E-48	4.19E-48	9.78	10	10.55	4.65	4.57	4.67
OsR498G0204787500.01	55.163	14.343	-1.97955	0	0	54.42	55.8	55.27	15.45	14.2	13.38
OsR498G0204792700.01	0.753	0.056	-3.80154	1.97E-10	3.39E-10	0.58	0.61	1.07	0.11	0.06	0
OsR498G0204794900.01	0.136	9.483	6.117763	2.29E-146	1.13E-146	0.18	0.14	0.09	10.25	9.55	8.65
OsR498G0204795300.01	0.673	0.206	-1.91717	2.88E-08	5.37E-08	0.76	0.54	0.72	0.17	0.26	0.19
OsR498G0204799000.01	0.81	1.9	1.235951	3.12E-17	4.48E-17	0.86	0.67	0.9	1.77	2.09	1.84
OsR498G0204800300.01	1.69	9.213	2.402642	3.83E-150	1.87E-150	2.18	1.42	1.47	8.32	9.7	9.62
OsR498G0204801700.01	0.283	7.93	4.806926	1.78E-165	8.15E-166	0.33	0.24	0.28	8.68	7.55	7.56
OsR498G0204802400.01	0.43	0.06	-2.89884	1.97E-05	4.22E-05	0.61	0.41	0.27	0.06	0.06	0.06

OsR498G0204802800.01	0.04	0.24	2.454798	0.0006489	0.0015399	0.08	0.04	0	0.29	0.21	0.22
OsR498G0204803600.01	1.626	51.836	4.977183	0	0	1.67	1.24	1.97	53.7	50	51.81
OsR498G0204808100.01	0.073	22.55	8.227937	7.09E-183	3.08E-183	0	0.08	0.14	22.15	22.54	22.96
OsR498G0204809800.01	0.35	0.94	1.418272	3.63E-05	7.89E-05	0.16	0.38	0.51	1.39	0.88	0.55
OsR498G0204811000.01	1.403	0.573	-1.27602	3.85E-08	7.21E-08	1.49	1.72	1	0.61	0.67	0.44
OsR498G0204812100.01	3.416	20.97	2.63334	7.24E-274	2.38E-274	3.12	3.63	3.5	22.81	20.37	19.73
OsR498G0204814300.01	351.42	49.003	-2.85097	0	0	377.83	337.2	339.2	48.87	49.49	48.65
OsR498G0204815300.01	0.353	0.1	-1.92547	6.85E-10	1.20E-09	0.43	0.32	0.31	0.15	0.07	0.08
OsR498G0204815600.01	469.11	138.15	-1.78692	0	0	491.66	460.57	455.1	147.38	131.47	135.6
OsR498G0204816000.01	1.63	0.566	-1.5452	8.62E-05	0.0001918	1.4	1.47	2.02	0.72	0.74	0.24
OsR498G0204817700.01	1.653	0.653	-1.32412	7.98E-11	1.36E-10	1.71	1.54	1.71	0.66	0.49	0.81
OsR498G0204819800.01	7.183	0.736	-3.25792	4.82E-54	4.15E-54	7.94	6.17	7.44	0.94	0.31	0.96
OsR498G0204822400.01	0.026	0.113	2.776726	6.87E-05	0.0001519	0	0	0.08	0.12	0.07	0.15
OsR498G0204823000.01	0.073	2.03	4.7402	7.53E-14	1.17E-13	0.11	0	0.11	1.62	2.01	2.46
OsR498G0204823700.01	5.883	79.726	3.791774	0	0	5.63	6.07	5.95	83.76	78.14	77.28
OsR498G0204824700.01	7.993	18.313	1.193516	1.43E-91	9.24E-92	8.11	8.1	7.77	18.1	18.25	18.59
OsR498G0204825100.01	1.363	0.38	-1.85836	1.27E-05	2.70E-05	1.52	1.24	1.33	0.52	0.21	0.41
OsR498G0204825700.01	0.646	2.663	2.03976	1.02E-29	1.15E-29	0.57	0.76	0.61	2.29	2.85	2.85
OsR498G0204826000.01	211.03	43.593	-2.2851	0	0	220.63	206.08	206.4	43.46	46	41.32
OsR498G0204826400.01	0.07	0.503	2.914229	2.18E-05	4.67E-05	0	0.21	0	0.81	0.42	0.28
OsR498G0204827600.01	39.203	2.54	-3.94841	0	0	36.67	41.3	39.64	3.12	2.14	2.36
OsR498G0204829100.01	53.966	23.223	-1.22249	2.44E-150	1.19E-150	57.53	54.7	49.67	24.08	22.6	22.99
OsR498G0204831400.01	0.01	0.246	4.499192	8.97E-07	1.79E-06	0	0.03	0	0.2	0.37	0.17
OsR498G0204832600.01	107.27	222.07	1.042824	0	0	109.51	105.68	106.6	226.81	224.12	215.28
OsR498G0204832700.01	0.026	0.286	3.361688	1.61E-05	3.42E-05	0.04	0.04	0	0.34	0.22	0.3
OsR498G0204833100.01	0.026	0.38	3.847115	1.30E-07	2.49E-07	0	0	0.08	0.44	0.29	0.41
OsR498G0204835000.01	2.1	22.736	3.431275	9.98E-268	3.34E-268	2.27	2.02	2.01	23.79	21.2	23.22
OsR498G0204835400.01	1.87	0.023	-6.41145	4.54E-42	4.43E-42	1.56	2.11	1.94	0	0	0.07
OsR498G0204835900.01	78.726	24.426	-1.65299	0	0	76.93	77.33	81.92	25.42	23.72	24.14
OsR498G0204838800.01	22.416	10.676	-1.08114	1.58E-40	1.57E-40	23.72	20.62	22.91	12.14	9.54	10.35
OsR498G0204839600.01	0.46	0.126	-1.96024	2.13E-05	4.57E-05	0.65	0.36	0.37	0.12	0.06	0.2
OsR498G0204839800.01	0.113	0	-5.01248	1.44E-05	3.05E-05	0.13	0.02	0.19	0	0	0
OsR498G0204841300.01	0.023	0.406	4.209685	1.01E-05	2.14E-05	0.07	0	0	0.53	0.42	0.27
OsR498G0204841500.01	2.306	10.026	2.057997	2.85E-228	1.07E-228	2.32	1.99	2.61	9.5	10.44	10.14
OsR498G0204843700.01	0	1.663	8.01704	1.51E-28	1.74E-28	0	0	0	1.71	1.77	1.51
OsR498G0204846400.01	0.17	1.53	3.105849	1.52E-13	2.39E-13	0.07	0.07	0.37	1.7	1.85	1.04
OsR498G0204849000.01	0.953	0.093	-3.71513	4.31E-23	5.47E-23	0.92	1.13	0.81	0.07	0.13	0.08
OsR498G0204849400.01	22.653	62.146	1.458611	0	0	22.89	23.26	21.81	61.23	62.63	62.58
OsR498G0204850100.01	19.126	2.716	-2.82151	7.40E-214	2.90E-214	20.05	18.78	18.55	2.75	2.63	2.77
OsR498G0204854200.01	2.766	0.326	-3.08952	1.35E-19	1.84E-19	3.47	2.16	2.67	0.32	0.25	0.41
OsR498G0204854800.01	0.036	0.226	2.54226	1.24E-06	2.49E-06	0.02	0.02	0.07	0.16	0.22	0.3
OsR498G0204855900.01	1.73	6.286	2.361688	8.22E-62	6.61E-62	1.29	1.85	2.05	6.21	6	6.65
OsR498G0204857500.01	34.15	9.623	-1.83027	0	0	33.99	35.59	32.87	9.28	9.61	9.98
OsR498G0204860400.01	5.31	0.63	-3.08423	4.26E-59	3.50E-59	5.7	4.9	5.33	0.67	0.64	0.58
OsR498G0204862800.01	1.183	0.29	-2.05978	5.52E-05	0.0001214	1.42	0.87	1.26	0.37	0.13	0.37
OsR498G0204869500.01	0.063	0	-4.5452	0.0002138	0.0004889	0.05	0.1	0.04	0	0	0
OsR498G0204870100.01	16.126	33.19	1.041353	0	0	15.03	16.15	17.2	32.38	33.81	33.38
OsR498G0204872500.01	22.14	83.3	1.790782	0	0	22.84	21.98	21.6	82.61	83.91	83.38
OsR498G0204873100.01	1.903	14.093	2.932845	4.57E-233	1.69E-233	1.7	1.93	2.08	14.15	14.76	13.37
OsR498G0204873700.01	1.77	0.683	-1.38405	1.47E-08	2.71E-08	1.82	1.96	1.53	0.59	0.71	0.75
OsR498G0204882100.01	0.453	0.06	-3.33875	2.97E-11	4.98E-11	0.42	0.39	0.55	0.03	0.06	0.09
OsR498G0204888400.01	3.82	1.603	-1.26047	1.38E-10	2.37E-10	4.43	3.42	3.61	1.7	1.38	1.73
OsR498G0204889600.01	34.533	13.993	-1.36144	1.91E-149	9.33E-150	34.38	33.87	35.35	14.99	13.2	13.79
OsR498G0204892600.01	1.276	0.54	-1.23597	5.34E-09	9.68E-09	1.46	1.34	1.03	0.59	0.6	0.43
OsR498G0204893200.01	4.54	22.13	2.337758	0	0	4.67	4.58	4.37	21.33	22.31	22.75
OsR498G0204894500.01	0	2.35	7.547555	5.69E-22	7.39E-22	0	0	0	2.9	2.59	1.56
OsR498G0204901800.01	0	0.13	4.361688	0.0005877	0.0013892	0	0	0	0.04	0.2	0.15
OsR498G0204905300.01	0.23	0.036	-3.96024	4.73E-05	0.0001035	0.18	0.32	0.19	0	0.11	0
OsR498G0204906600.01	3.336	16.486	2.304438	1.00E-201	4.07E-202	3.48	3.58	2.95	17.41	15.52	16.53
OsR498G0204908700.01	0.85	3.69	2.119994	1.02E-75	7.31E-76	0.85	0.87	0.83	3.64	3.65	3.78
OsR498G0204911000.01	13.45	3.61	-1.90471	3.05E-73	2.23E-73	13.86	13.63	12.86	3.88	3.72	3.23
OsR498G0204911800.01	0.013	0.353	4.624723	8.04E-13	1.29E-12	0.02	0.02	0	0.3	0.29	0.47
OsR498G0204912400.01	76.083	36.136	-1.07249	0	0	81.03	74.87	72.35	37.21	37.89	33.31
OsR498G0204913200.01	14.04	29.533	1.061059	3.20E-193	1.34E-193	13.76	14.34	14.02	29.59	29.91	29.1
OsR498G0204913700.01	3.206	10.853	1.750454	2.47E-117	1.40E-117	3.23	2.85	3.54	10.43	10.24	11.89
OsR498G0204915700.01	4.99	15.376	1.59278	2.76E-188	1.18E-188	5	4.63	5.34	14.49	16.18	15.46
OsR498G0204919300.01	0.783	10.716	3.775856	2.91E-227	1.09E-227	0.9	0.8	0.65	11.67	10.41	10.07
OsR498G0204923400.01	19.823	40.363	1.021451	8.39E-175	3.73E-175	19.24	19.36	20.87	43.44	39.91	37.74
OsR498G0204925200.01	0.876	0.043	-4.41967	4.84E-21	6.39E-21	0.83	0.98	0.82	0.07	0.03	0.03

OsR498G0204925300.01	409.22	19.666	-4.38967	0	0	429.31	399.91	398.4	23.39	17.32	18.29
OsR498G0204927000.01	163.6	56.253	-1.5534	0	0	167.44	164.39	159	55.41	54.75	58.6
OsR498G0204929900.01	3.983	1.943	-1.03805	1.25E-33	1.34E-33	3.59	4.14	4.22	1.8	2.07	1.96
OsR498G0204932100.01	0.59	0.133	-2.0477	3.06E-07	5.97E-07	0.69	0.54	0.54	0.2	0.07	0.13
OsR498G0204932400.01	3.84	8.716	1.10262	3.08E-47	2.84E-47	3.99	3.66	3.87	8.83	8.73	8.59
OsR498G0204936400.01	13.083	35.1	1.419213	0	0	12.52	13.31	13.42	34.63	34.92	35.75
OsR498G0204939700.01	75.986	169.11	1.181119	0	0	79.19	77.77	71	168.35	172.11	166.86
OsR498G0204940000.01	13.62	28.903	1.174043	4.63E-193	1.94E-193	14.32	13.58	12.96	30.19	27.93	28.59
OsR498G0204940800.01	4.756	15.35	1.675797	2.76E-145	1.37E-145	4.8	4.67	4.8	14.75	15.48	15.82
OsR498G0204941300.01	0.116	0.56	2.219271	0.0006343	0.0015039	0.35	0	0	0.31	1.04	0.33
OsR498G0204942200.01	1.25	4.76	1.905673	2.86E-19	3.93E-19	1.01	0.96	1.78	4.71	4.87	4.7
OsR498G0204944000.01	7.17	2.243	-1.69681	1.47E-71	1.09E-71	6.03	6.91	8.57	2.17	2.3	2.26
OsR498G0204944700.01	12.756	41.35	1.701634	3.11E-301	9.49E-302	13.32	13.12	11.83	42.81	40.23	41.01
OsR498G0204946100.01	1.51	0.303	-2.36963	1.56E-23	1.96E-23	1.54	1.39	1.6	0.49	0.21	0.21
OsR498G0204947800.01	4.11	1.586	-1.39158	7.73E-13	1.24E-12	4.65	2.99	4.69	1.63	1.75	1.38
OsR498G0204948900.01	112.83	22.28	-2.36282	0	0	112.73	114.15	111.6	23.06	22.64	21.14
OsR498G0204950400.01	1.44	0.493	-1.56009	1.81E-11	3.02E-11	1.5	1.33	1.49	0.37	0.41	0.7
OsR498G0204953000.01	1.606	0.826	-1.02461	2.08E-08	3.87E-08	1.17	1.58	2.07	0.7	0.69	1.09
OsR498G0204954200.01	2.356	1.02	-1.32535	1.93E-18	2.70E-18	2.18	3.28	1.61	1.02	1.11	0.93
OsR498G0204958300.01	1.903	0.453	-2.07572	2.18E-24	2.70E-24	1.56	2.01	2.14	0.47	0.52	0.37
OsR498G0204959200.01	45.1	115.11	1.346649	0	0	46.02	42.14	47.14	113.62	113.32	118.4
OsR498G0204959400.01	0.473	0.113	-2.0626	1.11E-09	1.97E-09	0.43	0.45	0.54	0.08	0.04	0.22
OsR498G0204960500.01	7.22	62.46	3.149617	0	0	7.52	7.26	6.88	58.45	63.57	65.36
OsR498G0204960900.01	2.333	11.02	2.228933	1.11E-102	6.78E-103	3.27	1.61	2.12	11.89	11.21	9.96
OsR498G0204961100.01	2.376	7.706	1.620046	2.55E-26	3.06E-26	2.94	2.01	2.18	7.33	7.63	8.16
OsR498G0204962300.01	3.76	17.453	2.21067	9.96E-253	3.47E-253	3.62	3.55	4.11	17.41	17.02	17.93
OsR498G0204962700.01	1.736	0.74	-1.23033	5.67E-06	1.18E-05	1.82	2.18	1.21	0.77	0.93	0.52
OsR498G0204964900.01	19.88	6.43	-1.6391	3.91E-59	3.22E-59	18.76	21.18	19.7	6.5	6.42	6.37
OsR498G0204965900.01	0.026	0.293	3.332542	6.08E-11	1.03E-10	0.03	0.05	0	0.32	0.27	0.29
OsR498G0204967700.01	5.353	2.206	-1.29503	1.53E-20	2.04E-20	4.78	5.61	5.67	1.88	1.94	2.8
OsR498G0204968000.01	0.77	3.473	2.074888	3.17E-34	3.38E-34	0.69	0.95	0.67	3.26	3.47	3.69
OsR498G0204969400.01	45.323	16.85	-1.44228	0	0	44.53	45.59	45.85	15.81	17.24	17.5
OsR498G0204969600.01	55.74	27.266	-1.04275	3.33E-149	1.63E-149	59.96	53.01	54.25	27.8	26.09	27.91
OsR498G0204969700.01	19.69	4.953	-2.02932	1.92E-163	8.90E-164	20.32	18.97	19.78	5	4.79	5.07
OsR498G0204972300.01	4.7	38.563	3.037642	0	0	4.82	4.41	4.87	39.41	39.41	36.87
OsR498G0204973800.01	25.293	63.016	1.311683	0	0	22.92	26.87	26.09	60.12	64.11	64.82
OsR498G0204976100.01	6.706	16.026	1.247838	1.73E-89	1.14E-89	6.68	6.78	6.66	16.26	15.85	15.97
OsR498G0204976400.01	1.936	21.366	3.449934	4.41E-265	1.48E-265	1.97	1.55	2.29	21.5	20.95	21.65
OsR498G0204977600.01	1.066	5.37	2.324904	5.71E-91	3.71E-91	1.27	0.91	1.02	5.07	5.38	5.66
OsR498G0204978400.01	0.06	1.366	4.50853	7.12E-37	7.34E-37	0.04	0.03	0.11	1.22	1.51	1.37
OsR498G0204980200.01	0.986	6.956	2.748711	7.32E-120	4.08E-120	0.91	1.13	0.92	7.74	6.08	7.05
OsR498G0204983800.01	10.706	27.4	1.352689	5.20E-210	2.06E-210	10.92	10.92	10.28	26.46	28.18	27.56
OsR498G0204985000.01	0.6	0.146	-2.17239	0.0004839	0.0011356	0.83	0.67	0.3	0.22	0.22	0
OsR498G0204985100.01	28.413	74.046	1.371226	0	0	29.81	28.3	27.13	73.87	73.24	75.03
OsR498G0204987500.01	0.25	0.74	1.551659	1.46E-07	2.81E-07	0.17	0.26	0.32	0.79	0.82	0.61
OsR498G0204995100.01	0.106	1.333	3.613012	9.99E-40	9.97E-40	0.05	0.11	0.16	1.28	1.45	1.27
OsR498G0204995300.01	0.463	3.106	2.735206	1.00E-18	1.40E-18	0.35	0.65	0.39	2.81	3.42	3.09
OsR498G0204995500.01	21.363	2.523	-3.10178	1.58E-231	5.86E-232	22.18	20.53	21.38	3.19	1.86	2.52
OsR498G0204995900.01	73.803	302.44	2.033026	0	0	71.09	73.77	76.55	296.62	304.08	306.61
OsR498G0204996300.01	0.01	0.65	6.127223	4.58E-18	6.45E-18	0.03	0	0	0.62	0.61	0.72
OsR498G0204998600.01	48.16	477.36	3.304086	0	0	42.14	53.26	49.08	492.42	469.55	470.11
OsR498G0205000100.01	13.073	3.356	-2.01507	2.88E-198	1.18E-198	14.53	12.52	12.17	4.08	2.95	3.04
OsR498G0205000900.01	4.586	1.496	-1.61898	3.56E-28	4.13E-28	4.41	5.13	4.22	1.55	1.77	1.17
OsR498G0205001200.01	14.763	6.47	-1.21028	1.34E-42	1.30E-42	14.27	16.38	13.64	6.35	5.52	7.54
OsR498G0205001700.01	27.736	11.066	-1.3302	6.44E-116	3.66E-116	29.1	27.53	26.58	11.72	10.67	10.81
OsR498G0205001900.01	57.476	12.566	-2.20453	1.58E-290	4.98E-291	56.2	57.31	58.92	11.06	11.02	15.62
OsR498G0205002100.01	142.08	7.216	-4.31186	0	0	139.45	143.84	143	6.72	7.59	7.34
OsR498G0205002700.01	9.096	34.696	1.9301	0	0	9.04	9.35	8.9	33.41	36.66	34.02
OsR498G0205005900.01	1.606	0.346	-2.17747	1.27E-44	1.21E-44	1.5	1.63	1.69	0.33	0.44	0.27
OsR498G0205006500.01	21.463	10.826	-1.00361	4.45E-34	4.76E-34	21.81	20.15	22.43	12.46	8.67	11.35
OsR498G0205006800.01	347.83	1076.1	1.700917	0	0	347.66	347.16	348.7	1044.1	1099.9	1084.3
OsR498G0205009300.01	9.226	40.683	2.137265	0	0	9.8	9.47	8.41	41.35	42.01	38.69
OsR498G0205010000.01	74.86	22.483	-1.75466	0	0	77.79	72.13	74.66	23.51	20.87	23.07
OsR498G0205010400.01	19.616	8.76	-1.19911	2.71E-145	1.35E-145	19.14	19.47	20.24	8.9	9	8.38
OsR498G0205010700.01	1150.6	59.756	-4.3667	0	0	1168.7	1151.8	1131	60.48	58.75	60.04
OsR498G0205011600.01	1.31	0.306	-2.13662	1.51E-33	1.63E-33	1.17	1.54	1.22	0.24	0.26	0.42
OsR498G0205012900.01	0.193	0.71	1.847115	4.13E-05	9.00E-05	0.29	0.12	0.17	1.14	0.62	0.37
OsR498G0205014000.01	1.286	0.126	-3.31779	3.28E-09	5.90E-09	1.42	0.84	1.6	0.1	0.17	0.11
OsR498G0205016600.01	0.74	2.173	1.541467	3.00E-24	3.73E-24	0.7	0.82	0.7	1.97	2.04	2.51

OsR498G0205018400.01	10.56	47.95	2.067989	0	0	9.22	11.45	11.01	45.31	48.27	50.27
OsR498G0205020400.01	1.296	0.626	-1.02817	0.0002259	0.0005177	1.15	1.18	1.56	0.68	0.55	0.65
OsR498G0205021000.01	1.72	0.28	-2.6232	4.88E-29	5.59E-29	1.46	2.03	1.67	0.22	0.31	0.31
OsR498G0205024600.01	0.293	1.796	2.615789	1.82E-25	2.21E-25	0.39	0.24	0.25	1.64	2.04	1.71
OsR498G0205028700.01	0.073	0.366	2.262152	9.01E-09	1.65E-08	0.05	0.04	0.13	0.43	0.3	0.37
OsR498G0205029500.01	0.193	0	-4.66068	0.0001179	0.0002647	0.04	0.31	0.23	0	0	0
OsR498G0205031900.01	7.993	3.06	-1.39205	5.20E-25	6.37E-25	7.97	7.7	8.31	3.84	2.68	2.66
OsR498G0205032200.01	2.086	0.62	-1.81079	5.05E-63	4.01E-63	2.08	2.2	1.98	0.7	0.63	0.53
OsR498G0205035300.01	1.05	0.47	-1.19631	5.57E-07	1.10E-06	1.1	0.87	1.18	0.48	0.29	0.64
OsR498G0205036800.01	2.55	0.4	-2.68869	1.82E-51	1.60E-51	2.14	3	2.51	0.18	0.52	0.5
OsR498G0205037100.01	7.833	0.41	-4.12668	1.56E-88	1.03E-88	8.38	7.71	7.41	0.53	0.44	0.26
OsR498G0205037700.01	0.596	0.06	-3.35256	1.36E-05	2.87E-05	0.51	0.54	0.74	0	0	0.18
OsR498G0205041900.01	7.82	19.18	1.286472	6.07E-135	3.16E-135	7.76	8.1	7.6	19.52	18.72	19.3
OsR498G0205043200.01	7.21	2.353	-1.6675	2.91E-57	2.44E-57	6.54	7.63	7.46	2.09	2.33	2.64
OsR498G0205044600.01	5.056	1.95	-1.35852	1.06E-30	1.19E-30	5.04	4.64	5.49	2.12	2.2	1.53
OsR498G0205045700.01	1.086	0.07	-4.15902	1.73E-44	1.65E-44	1.11	1.01	1.14	0.06	0.07	0.08
OsR498G0205046200.01	1.153	0.216	-2.1929	9.13E-13	1.47E-12	1.33	1.15	0.98	0.16	0.26	0.23
OsR498G0205047800.01	122.33	276.96	1.171867	0	0	124.04	120.4	122.6	279.11	271.38	280.38
OsR498G0205048000.01	1.05	0.436	-1.28217	1.06E-06	2.12E-06	1.19	0.81	1.15	0.43	0.52	0.36
OsR498G0205052100.01	0.216	0.103	-1.74451	0.0002079	0.0004747	0.26	0.23	0.16	0	0.07	0.24
OsR498G0205053100.01	0.32	0.08	-2.03076	6.13E-09	1.11E-08	0.23	0.48	0.25	0.11	0.02	0.11
OsR498G0205053300.01	0.476	0.103	-2.20125	6.96E-08	1.32E-07	0.33	0.6	0.5	0.08	0.17	0.06
OsR498G0205056400.01	5.893	16.606	1.525187	1.54E-107	9.13E-108	5.09	6.82	5.77	17.43	16.15	16.24
OsR498G0205059100.01	48.823	22.186	-1.1449	6.73E-127	3.62E-127	51.11	46.03	49.33	22.79	21.96	21.81
OsR498G0205060400.01	6.84	1.31	-2.40081	1.53E-21	2.01E-21	6.55	7.9	6.07	1.25	1.13	1.55
OsR498G0205063900.01	49.923	22.033	-1.18553	4.67E-177	2.07E-177	48.23	49.64	51.9	21.92	23.19	20.99
OsR498G0305064700.01	0.01	0.156	4.03976	3.45E-05	7.49E-05	0.03	0	0	0.17	0.12	0.18
OsR498G0305065200.01	0.416	0.083	-2.33875	4.96E-05	0.0001085	0.29	0.45	0.51	0.2	0	0.05
OsR498G0305066500.01	388.35	102.13	-1.93061	0	0	391.26	388.56	385.2	113.32	93.55	99.51
OsR498G0305067500.01	1.393	0.493	-1.5452	5.66E-09	1.03E-08	1.65	1.38	1.15	0.55	0.52	0.41
OsR498G0305069500.01	0.51	0	-4.29223	0.0006833	0.0016245	1.53	0	0	0	0	0
OsR498G0305070200.01	17.39	1.023	-4.13317	0	0	16.38	17.02	18.77	0.96	1.08	1.03
OsR498G0305070900.01	32.823	12.373	-1.24846	7.39E-122	4.07E-122	34.86	29.41	34.2	12.65	11.15	13.32
OsR498G0305077600.01	1.996	3.793	1.023054	1.14E-07	2.18E-07	2.5	2.13	1.36	3.43	3.64	4.31
OsR498G0305080300.01	0	0.833	5.287688	2.63E-06	5.36E-06	0	0	0	0.52	0.53	1.45
OsR498G0305081700.01	10.003	4.306	-1.2057	2.01E-58	1.66E-58	11.15	9.06	9.8	3.86	4.97	4.09
OsR498G0305082200.01	3.216	7.673	1.164387	1.02E-29	1.16E-29	3.34	3.15	3.16	7.41	8.3	7.31
OsR498G0305085200.01	0.983	0.153	-2.71161	8.50E-33	9.24E-33	0.92	0.97	1.06	0.09	0.18	0.19
OsR498G0305093300.01	0.66	0.21	-1.67379	8.08E-07	1.61E-06	0.81	0.44	0.73	0.23	0.18	0.22
OsR498G0305093800.01	3.413	110.8	5.01533	0	0	3.79	3.59	2.86	119.35	102.14	110.92
OsR498G0305094500.01	2.763	6.19	1.211031	2.91E-29	3.32E-29	2.32	2.96	3.01	5.31	6.51	6.75
OsR498G0305096800.01	0.813	0.166	-2.3173	7.25E-06	1.52E-05	0.55	0.66	1.23	0.16	0.09	0.25
OsR498G0305096900.01	1.08	0.406	-1.52776	1.19E-07	2.28E-07	1.22	1.35	0.67	0.56	0.21	0.45
OsR498G0305099300.01	38.61	12.486	-1.61302	1.27E-203	5.14E-204	40.92	37.33	37.58	11.87	12.13	13.46
OsR498G0305101300.01	8.28	3.596	-1.18462	1.91E-71	1.42E-71	8.17	7.94	8.73	3.67	3.6	3.52
OsR498G0305102600.01	49.743	19.483	-1.35718	6.05E-210	2.40E-210	49.27	50.78	49.18	18.8	20.21	19.44
OsR498G0305103200.01	30.363	13.063	-1.16667	4.48E-303	1.36E-303	29.54	31.87	29.68	12.79	12.81	13.59
OsR498G0305106100.01	347.63	13.99	-4.49129	0	0	349.08	337.94	355.9	12.64	14.63	14.7
OsR498G0305107600.01	11.92	61.283	2.323111	2.41E-237	8.79E-238	13.66	12.05	10.05	62.23	59.41	62.21
OsR498G0305108900.01	20.496	71.736	1.820211	0	0	19.01	21.61	20.87	68.87	71.97	74.37
OsR498G0305115400.01	2.866	9.82	1.770702	8.95E-88	5.93E-88	3.19	2.59	2.82	10.58	10.81	8.07
OsR498G0305118700.01	6.043	18.716	1.63718	4.40E-301	1.34E-301	6.23	6.33	5.57	18.62	18.8	18.73
OsR498G0305119300.01	14.216	41.65	1.548419	2.15E-164	9.90E-165	15.03	13.27	14.35	42.32	42.87	39.76
OsR498G0305119600.01	0.246	9.81	5.285788	1.65E-181	7.19E-182	0.23	0.2	0.31	10.37	8.59	10.47
OsR498G0305121500.01	19.066	130.34	2.81622	0	0	18.43	20.85	17.92	131.84	127.9	131.28
OsR498G0305122300.01	0.836	2.15	1.338605	4.10E-11	6.92E-11	0.83	0.58	1.1	2.29	1.94	2.22
OsR498G0305123500.01	29.65	64.733	1.131219	1.19E-241	4.29E-242	29.42	28.87	30.66	67.9	62.97	63.33
OsR498G0305123700.01	1.026	0.343	-1.48862	6.29E-07	1.24E-06	1.06	1.06	0.96	0.24	0.38	0.41
OsR498G0305126100.01	6.423	0.74	-3.08041	4.55E-88	3.01E-88	5.58	6.72	6.97	0.72	0.86	0.64
OsR498G0305127300.01	9.226	1.956	-2.24909	1.89E-89	1.24E-89	8.3	9.73	9.65	1.71	2.38	1.78
OsR498G0305127600.01	1.23	0.513	-1.28217	7.19E-06	1.50E-05	1.33	0.88	1.48	0.35	0.73	0.46
OsR498G0305128500.01	5.253	1.513	-1.81856	3.02E-50	2.70E-50	5.66	5.18	4.92	1.85	1.47	1.22
OsR498G0305130200.01	2.726	0.08	-5.04269	1.35E-91	8.74E-92	2.1	2.94	3.14	0.03	0.17	0.04
OsR498G0305130300.01	3.92	0.09	-5.3023	2.66E-52	2.33E-52	4.86	3.73	3.17	0.18	0	0.09
OsR498G0305132800.01	2.443	0.933	-1.21674	1.99E-08	3.70E-08	2.87	2.36	2.1	1.06	0.79	0.95
OsR498G0305135000.01	3.513	0.323	-3.50456	4.84E-61	3.91E-61	3.28	4.17	3.09	0.22	0.45	0.3
OsR498G0305136000.01	1.44	0.22	-2.70747	8.86E-16	1.32E-15	1.6	1.3	1.42	0.23	0.19	0.24
OsR498G0305138200.01	2.266	0.673	-1.76759	1.07E-06	2.14E-06	1.69	2.67	2.44	0.75	0.51	0.76
OsR498G0305140000.01	2.01	0.8	-1.33528	1.82E-11	3.04E-11	1.96	1.9	2.17	0.92	0.84	0.64

OsR498G0305143200.01	0.636	6.33	3.346258	2.64E-88	1.74E-88	0.5	0.83	0.58	6.56	6.52	5.91
OsR498G0305149500.01	0	0.153	4.499192	0.0003163	0.0007323	0	0	0	0.16	0.16	0.14
OsR498G0305150500.01	5.966	66.286	3.467892	0	0	6.48	5.85	5.57	70.7	64.33	63.83
OsR498G0305151300.01	5.276	11.343	1.099718	5.16E-76	3.70E-76	4.84	5.17	5.82	11.96	11.95	10.12
OsR498G0305151700.01	1.633	4.686	1.525547	4.60E-60	3.75E-60	1.75	1.85	1.3	5.2	4.61	4.25
OsR498G0305151900.01	17.846	40.56	1.184497	5.35E-185	2.31E-185	17.74	17.19	18.61	41.21	38.71	41.76
OsR498G0305152300.01	10.29	1.183	-3.13016	4.39E-100	2.70E-100	10.78	10.19	9.9	0.82	1.29	1.44
OsR498G0305153800.01	3.003	11.743	1.950977	1.93E-87	1.28E-87	3.41	2.63	2.97	11.49	11.95	11.79
OsR498G0305155500.01	0.153	0.056	-1.51754	0.0003021	0.0006981	0.19	0.12	0.15	0.07	0.04	0.06
OsR498G0305155800.01	12.84	3.483	-1.91782	5.09E-45	4.81E-45	13.42	13.7	11.4	3.69	2.35	4.41
OsR498G0305156900.01	7.8	18.763	1.258922	4.15E-68	3.17E-68	7.57	7.56	8.27	20.55	18.73	17.01
OsR498G0305158900.01	0.596	2.973	2.311062	1.13E-19	1.54E-19	0.8	0.52	0.47	3.33	2.52	3.07
OsR498G0305161400.01	0.886	0.223	-1.92899	2.40E-15	3.61E-15	0.7	0.8	1.16	0.22	0.24	0.21
OsR498G0305165700.01	7.69	12.18	1.050265	5.76E-61	4.65E-61	7.37	8.23	7.47	12.64	12.53	11.37
OsR498G0305166000.01	3.953	112.8	4.833039	0	0	3.62	4.08	4.16	111.7	112.95	113.76
OsR498G0305166900.01	1732.4	260.69	-2.7374	0	0	1701.6	1722.8	1773	268.64	255.65	257.79
OsR498G0305167500.01	100.38	7.543	-3.75235	0	0	110.18	92.41	98.56	6.88	7.58	8.17
OsR498G0305170500.01	21.953	55.173	1.327648	7.73E-235	2.84E-235	22.85	23.39	19.62	55.93	54.68	54.91
OsR498G0305173100.01	1.15	9.023	3.015913	3.84E-83	2.61E-83	1.3	1.1	1.05	9.74	9.39	7.94
OsR498G0305175900.01	4.056	1.246	-1.70171	1.34E-19	1.83E-19	4.51	4.67	2.99	1.26	1.13	1.35
OsR498G0305176100.01	4.79	0.06	-6.34369	1.12E-78	7.88E-79	4.39	4.39	5.59	0.09	0.09	0
OsR498G0305176400.01	1.4	0	-5.35667	1.12E-06	2.25E-06	0.99	1.41	1.8	0	0	0
OsR498G0305178200.01	1.33	0.603	-1.15616	1.22E-07	2.33E-07	0.87	1.67	1.45	0.53	0.33	0.95
OsR498G0305180300.01	54.6	8.18	-2.77139	0	0	52.61	53.72	57.47	8.62	7.47	8.45
OsR498G0305180600.01	4.696	29.266	2.634739	9.78E-279	3.17E-279	4.28	4.97	4.84	30.34	28.23	29.23
OsR498G0305181500.01	6.676	19.853	1.570467	2.37E-128	1.27E-128	6.79	7.08	6.16	20.8	18.02	20.74
OsR498G0305182200.01	0.44	0.993	1.176935	8.02E-08	1.52E-07	0.53	0.39	0.4	1.14	0.96	0.88
OsR498G0305183700.01	61.75	19.78	-1.63841	0	0	63.46	58.71	63.08	19.7	20.17	19.47
OsR498G0305186000.01	0.326	0.033	-3.20817	4.33E-05	9.45E-05	0.46	0.27	0.25	0.05	0	0.05
OsR498G0305186200.01	14.863	6.11	-1.28909	6.20E-42	6.06E-42	15.26	14.3	15.03	7.88	5.57	4.88
OsR498G0305190100.01	2.08	26.29	3.635417	3.49E-136	1.80E-136	2.12	1.64	2.48	26.71	26.1	26.06
OsR498G0305191400.01	14.703	40.87	1.474891	0	0	14.49	13.92	15.7	40.16	40.91	41.54
OsR498G0305193500.01	22.563	71.723	1.668658	0	0	24.24	22.29	21.16	70.96	72.61	71.6
OsR498G0305194700.01	33.596	111.09	1.721375	0	0	35.13	32.89	32.77	115.61	109.2	108.47
OsR498G0305197900.01	10.506	46.15	2.136427	0	0	10.26	11	10.26	45.32	44.4	48.73
OsR498G0305201100.01	5.596	0.93	-2.6007	7.24E-62	5.82E-62	5.73	5.18	5.88	1.21	0.92	0.66
OsR498G0305205100.01	5.293	1.466	-1.85762	2.72E-51	2.41E-51	4.95	4.78	6.15	1.67	1.42	1.31
OsR498G0305205900.01	2.48	0.153	-4.01744	2.36E-60	1.92E-60	2.34	2.39	2.71	0.16	0.22	0.08
OsR498G0305207300.01	4.846	0.423	-3.52414	1.95E-104	1.17E-104	4.32	4.97	5.25	0.46	0.39	0.42
OsR498G0305207800.01	1.76	0.586	-2.22011	2.60E-23	3.29E-23	2.05	1.37	1.86	0.53	0.45	0.78
OsR498G0305210700.01	31.656	173.91	2.452844	0	0	31.21	30.62	33.14	179.76	174.78	167.19
OsR498G0305211900.01	0.14	0.013	-3.76759	0.0001564	0.0003541	0.17	0.12	0.13	0.04	0	0
OsR498G0305213100.01	0	0.493	5.7402	4.87E-08	9.17E-08	0	0	0	0.96	0.29	0.23
OsR498G0305214100.01	0.116	5.493	5.584081	6.03E-202	2.45E-202	0.1	0.16	0.09	5.69	5.13	5.66
OsR498G0305215500.01	7.076	18.713	1.443535	4.82E-306	1.45E-306	7.05	6.88	7.3	18.45	18.5	19.19
OsR498G0305215800.01	30.676	135.72	2.131728	0	0	31.32	30.61	30.1	135.89	136.67	134.59
OsR498G0305220900.01	0.783	2.013	1.293944	1.30E-16	1.89E-16	0.75	0.92	0.68	2.09	1.99	1.96
OsR498G0305223800.01	0.73	2.016	1.139296	5.75E-06	1.19E-05	1.16	0.27	0.76	1.84	1.72	2.49
OsR498G0305224700.01	34.213	218.07	2.665327	0	0	36.86	33.32	32.46	222.35	217.6	214.25
OsR498G0305225000.01	175.96	500.63	1.54599	0	0	171.78	173	183.1	533.23	488.83	479.83
OsR498G0305225100.01	0.246	0.743	1.601314	0.0007082	0.0016851	0.26	0.36	0.12	0.6	1.08	0.55
OsR498G0305226500.01	43.263	207.21	2.254565	0	0	43.21	43.17	43.41	208.18	208.18	205.27
OsR498G0305226700.01	79	161.7	1.017809	0	0	82.31	76.52	78.17	171.63	162.23	151.25
OsR498G0305233000.01	0.52	1.123	1.12129	6.62E-06	1.38E-05	0.5	0.48	0.58	1.16	1.15	1.06
OsR498G0305233300.01	5.756	20.706	1.80232	2.24E-162	1.04E-162	5.88	5.66	5.73	19.83	22.6	19.69
OsR498G0305236200.01	25.276	65.59	1.393515	0	0	22.86	27.94	25.03	67.03	65.12	64.62
OsR498G0305239500.01	9.136	63.03	2.741146	0	0	8.32	9.77	9.32	67.89	59.2	62
OsR498G0305239700.01	1.616	3.59	1.412495	4.99E-16	7.37E-16	1.95	1.27	1.63	2.49	4.8	3.48
OsR498G0305240000.01	0.483	1.373	1.499859	3.15E-05	6.83E-05	0	0.69	0.76	2.05	1.41	0.66
OsR498G0305240600.01.'	0.1	0.243	1.178584	0.0009338	0.0022424	0	0	0.3	0	0.26	0.47
OsR498G0305241400.01	1.443	0.673	-1.1016	5.66E-05	0.0001246	1.48	1.68	1.17	0.76	0.9	0.36
OsR498G0305243800.01	0.733	9.736	3.668349	1.15E-142	5.81E-143	0.46	0.85	0.89	9.68	9.44	10.09
OsR498G0305247700.01	0.076	4.543	6.087793	2.63E-96	1.65E-96	0.15	0.04	0.04	4.9	4.24	4.49
OsR498G0305248100.01	1.413	8.383	2.560926	2.50E-109	1.46E-109	1.58	1.29	1.37	8.5	7.18	9.47
OsR498G0305252200.01	14.733	138.1	3.207477	0	0	17.31	12.59	14.3	136.41	141.67	136.23
OsR498G0305254100.01	2.193	7.346	1.683616	4.71E-73	3.45E-73	2.26	1.75	2.57	6.78	7.67	7.59
OsR498G0305254600.01	99.58	239.07	1.290173	0	0	100.06	100.45	98.23	238.17	232.68	246.37
OsR498G0305255700.01	15.686	2.346	-2.74118	0	0	15.79	15.11	16.16	2.39	2.32	2.33
OsR498G0305256800.01	15.266	4.223	-1.88169	9.85E-101	6.06E-101	15.28	16.12	14.4	5.27	3.39	4.01

OsR498G0305270500.01	180.9	39.53	-2.23096	0	0	185.67	174.93	182.1	40.3	37.46	40.83
OsR498G0305271700.01	0.263	2	3.325162	2.20E-52	1.93E-52	0.23	0.38	0.18	1.88	1.81	2.31
OsR498G0305274100.01	1.256	0.043	-4.96024	1.04E-23	1.30E-23	1.33	1.19	1.25	0.09	0	0.04
OsR498G0305275900.01	1.836	0.816	-1.14489	2.63E-21	3.46E-21	1.81	2.2	1.5	0.82	0.89	0.74
OsR498G0305276100.01	7.05	3.343	-1.08508	1.78E-59	1.46E-59	6.55	7.34	7.26	3.14	3.22	3.67
OsR498G0305276300.01	1.95	0.47	-2.05986	7.49E-24	9.38E-24	2.15	1.88	1.82	0	0.38	1.03
OsR498G0305280600.01	0.103	0.423	2.018386	5.00E-09	9.07E-09	0.05	0.19	0.07	0.35	0.48	0.44
OsR498G0305280800.01	0.073	0.353	2.307704	1.48E-08	2.74E-08	0.07	0.09	0.06	0.21	0.61	0.24
OsR498G0305281400.01	0.876	0.236	-1.76134	7.48E-12	1.24E-11	0.8	0.95	0.88	0.24	0.19	0.28
OsR498G0305281500.01	0.296	0.086	-1.78673	4.56E-06	9.40E-06	0.22	0.33	0.34	0.13	0.11	0.02
OsR498G0305284600.01	0.423	33.613	6.314983	0	0	0.41	0.51	0.35	34.77	32.59	33.48
OsR498G0305285500.01	0.426	0.123	-1.73785	0.0009028	0.0021644	0.71	0.22	0.35	0.21	0	0.16
OsR498G0305289500.01	11.906	5.423	-1.14924	1.90E-71	1.41E-71	11.94	12.53	11.25	5.4	5.94	4.93
OsR498G0305289900.01	0.026	0.116	2.847115	0.0005624	0.0013266	0	0.04	0.04	0.05	0.18	0.12
OsR498G0305291100.01	0.03	0.17	2.54226	0.0003732	0.0008685	0.03	0.03	0.03	0.21	0.12	0.18
OsR498G0305291800.01	0.53	18.81	4.884203	0	0	0.54	0.51	0.54	19.93	17.99	18.51
OsR498G0305293000.01	0.02	0.243	3.499192	3.37E-08	6.30E-08	0	0.02	0.04	0.24	0.27	0.22
OsR498G0305295600.01	2.353	0.853	-1.47961	1.23E-07	2.36E-07	2.62	2.15	2.29	0.92	0.78	0.86
OsR498G0305295700.01	0.276	0.563	1.127223	0.0004621	0.001083	0.24	0.36	0.23	0.56	0.5	0.63
OsR498G0305296300.01	214.7	60.88	-1.81167	0	0	213	211.69	219.4	60.72	61.39	60.53
OsR498G0305296600.01	66.72	20.18	-1.6257	0	0	68.27	66.4	65.49	21.76	19.4	19.38
OsR498G0305297400.01	1.326	5.11	1.935924	1.75E-15	2.61E-15	1.21	1.5	1.27	6.56	5.09	3.68
OsR498G0305297900.01	8.856	3.523	-1.33715	9.67E-44	9.28E-44	9.89	7.88	8.8	2.96	3.65	3.96
OsR498G0305299000.01	66.846	28.61	-1.23558	3.61E-132	1.89E-132	68.26	63.84	68.44	33.75	27.14	24.94
OsR498G0305300200.01	0.316	0	-5.86713	8.31E-09	1.52E-08	0.25	0.46	0.24	0	0	0
OsR498G0305302600.01	22.053	86.07	1.965221	0	0	20.68	21.43	24.05	84.77	87.61	85.83
OsR498G0305304300.01	27.273	211.61	2.923347	0	0	27.34	26.99	27.49	211.94	212.02	210.86
OsR498G0305306100.01	6.59	3.126	-1.05636	7.10E-62	5.70E-62	7.34	5.74	6.69	3.09	3.21	3.08
OsR498G0305306500.01	0.7	1.866	1.402712	0.0003626	0.0008428	1.15	0.72	0.23	2.05	2.24	1.31
OsR498G0305310300.01	3.68	7.523	1.013342	3.51E-39	3.52E-39	3.99	3.65	3.4	7.11	7.96	7.5
OsR498G0305311100.01	13.95	3.406	-2.09849	4.43E-172	1.99E-172	13.76	13.81	14.28	3.55	3.35	3.32
OsR498G0305311800.01	52.916	8.26	-2.70935	0	0	54.13	53.98	50.64	8.6	7.21	8.97
OsR498G0305313900.01	15.903	1.613	-3.10592	1.20E-151	5.83E-152	15.79	15.36	16.56	1.76	1.29	1.79
OsR498G0305314600.01	0.356	0.033	-3.4838	4.22E-06	8.70E-06	0.37	0.39	0.31	0	0.05	0.05
OsR498G0305315100.01	30.676	11.486	-1.4414	1.39E-137	7.18E-138	31.71	28.93	31.39	11.38	12.63	10.45
OsR498G0305316300.01	0.35	0	-5.41967	6.63E-07	1.31E-06	0.48	0.2	0.37	0	0	0
OsR498G0305319400.01	17.696	5.886	-1.60254	5.61E-37	5.78E-37	18.72	18.05	16.32	6.37	5.42	5.87
OsR498G0305322300.01	25.073	8.18	-1.62286	4.84E-108	2.85E-108	25.49	23.89	25.84	9.46	7.24	7.84
OsR498G0305322600.01	0.123	0.64	2.361688	2.29E-06	4.65E-06	0.27	0	0.1	0.76	0.5	0.66
OsR498G0305326300.01	1.96	9.27	2.299451	1.35E-110	7.84E-111	2	2.44	1.44	8.66	8.23	10.92
OsR498G0305326600.01	0.043	2.676	6.01704	6.37E-32	7.00E-32	0	0.13	0	2.38	2	3.65
OsR498G0305327800.01	6.096	14.463	1.242893	6.84E-89	4.50E-89	6.07	6.38	5.84	13.29	16.04	14.06
OsR498G0305328400.01	4.046	1.34	-1.60116	7.68E-16	1.14E-15	4.8	3.66	3.68	1.41	1.38	1.23
OsR498G0305330200.01	0.076	1.746	4.482704	1.76E-21	2.31E-21	0.17	0.06	0	1.42	1.77	2.05
OsR498G0305330900.01	0.796	65.186	6.334764	0	0	1.15	0.42	0.82	68.18	63.72	63.66
OsR498G0305331700.01	0.84	75.71	6.485859	0	0	0.72	0.82	0.98	78.58	75.21	73.34
OsR498G0305333100.01	0.02	0.36	4.287688	3.14E-10	5.46E-10	0.03	0.03	0	0.25	0.46	0.37
OsR498G0305333900.01	2.516	0.8	-1.64161	1.43E-22	1.83E-22	2.29	2.52	2.74	0.63	0.86	0.91
OsR498G0305334700.01	4.643	2.326	-1.033	2.23E-32	2.44E-32	4.63	4.35	4.95	2.26	2.53	2.19
OsR498G0305337400.01	0.263	4.93	4.196974	1.07E-75	7.66E-76	0.41	0.26	0.12	5.05	4.87	4.87
OsR498G0305339100.01	13.236	6.61	-1.0716	1.82E-30	2.04E-30	12.74	14.11	12.86	7.5	5.88	6.45
OsR498G0305340600.01	1.426	18.36	3.666764	8.67E-155	4.14E-155	1.66	0.95	1.67	20.11	17.49	17.48
OsR498G0305343300.01	0.023	0.5	4.209685	1.05E-09	1.85E-09	0	0	0.07	0.55	0.54	0.41
OsR498G0305348700.01	1.533	4.523	1.555237	5.18E-70	3.89E-70	1.57	1.63	1.4	4.36	4.53	4.68
OsR498G0305354100.01	5.03	13	1.404087	1.42E-39	1.42E-39	4.74	6.34	4.01	12.38	12.91	13.71
OsR498G0305356500.01	28.476	362.03	3.631196	0	0	29.14	29.13	27.16	380.14	352.11	353.85
OsR498G0305356700.01	19.263	113.81	2.564805	0	0	20.65	18.54	18.6	115.21	110.35	115.87
OsR498G0305358500.01	5.243	72.64	3.807851	0	0	5.3	4.99	5.44	73.81	72.96	71.15
OsR498G0305361600.01	0.886	83.64	6.554514	0	0	0.74	1.11	0.81	97	78.75	75.17
OsR498G0305362700.01	1.373	10.883	2.984779	7.83E-153	3.78E-153	1.23	1.43	1.46	11.7	11.38	9.57
OsR498G0305363900.01	20.556	124.61	2.593684	0	0	19.18	21.08	21.41	131.22	122.36	120.25
OsR498G0305364400.01	5.553	22.8	2.139296	3.22E-192	1.35E-192	5.56	5.1	6	24.53	21.04	22.83
OsR498G0305366400.01	6.396	2.49	-1.36888	6.06E-38	6.19E-38	5.92	7.05	6.22	2.25	2.67	2.55
OsR498G0305366800.01	3.776	21.336	2.499983	0	0	3.41	4.1	3.82	21.95	22.02	20.04
OsR498G0305367600.01	8.736	38.123	2.139353	0	0	8.49	9.04	8.68	36.91	37.91	39.55
OsR498G0305367800.01	32.86	71.866	1.124119	0	0	30.61	34.26	33.71	68.48	73.46	73.66
OsR498G0305369000.01	7.263	1.39	-2.3893	2.67E-68	2.03E-68	7.29	7.82	6.68	1.15	1.63	1.39
OsR498G0305369900.01	8.546	35.84	2.072182	2.92E-206	1.17E-206	8.42	8.02	9.2	37.32	33	37.2
OsR498G0305371500.01	0.28	11.226	5.327108	1.04E-177	4.61E-178	0.39	0.32	0.13	12.63	10.06	10.99

OsR498G0305373900.01	305.88	145.09	-1.05422	0	0	309.22	304.78	303.6	140.58	147.51	147.17
OsR498G0305374400.01	60.353	287.71	2.251041	0	0	57.67	61.3	62.09	290.67	285.33	287.13
OsR498G0305376000.01	7.516	4.173	-1.18756	2.35E-58	1.95E-58	7.67	7.6	7.28	4.1	3.79	4.63
OsR498G0305377800.01	0.4	0.186	-1.4109	0.0002493	0.0005735	0.42	0.46	0.32	0.18	0.13	0.25
OsR498G0305378500.01	6.936	1.123	-2.6483	2.21E-35	2.33E-35	6.4	7.79	6.62	0.93	1.12	1.32
OsR498G0305379200.01	1.153	3.41	1.560997	3.43E-25	4.20E-25	1.34	1.07	1.05	3.3	3.45	3.48
OsR498G0305379700.01	2.346	26.83	3.483809	1.46E-252	5.10E-253	1.88	1.98	3.18	26.78	28.25	25.46
OsR498G0305380500.01	3.5	118.26	5.076326	0	0	2.98	4.1	3.42	122.83	114.61	117.34
OsR498G0305381100.01	0.176	1.2	2.748711	5.28E-15	7.98E-15	0.2	0.09	0.24	1.37	1.08	1.15
OsR498G0305381800.01	2.5	1.146	-1.05488	5.84E-27	6.93E-27	2.33	2.6	2.57	1.08	1.29	1.07
OsR498G0305382000.01	15.513	53.993	1.798002	0	0	15.09	15.56	15.89	54.14	51.92	55.92
OsR498G0305382100.01	2.13	16.063	2.897741	3.03E-30	3.41E-30	1.83	3.05	1.51	14.14	18.5	15.55
OsR498G0305383900.01	0.033	0.67	4.245043	1.01E-22	1.30E-22	0	0.04	0.06	0.57	0.77	0.67
OsR498G0305387400.01	1.396	0.066	-4.41967	1.94E-11	3.24E-11	1.19	1.25	1.75	0	0.1	0.1
OsR498G0305389700.01	33.086	100.7	1.595767	0	0	31.79	32.34	35.13	106.01	98.33	97.75
OsR498G0305395700.01	245.86	57.376	-2.10262	0	0	248.61	243.39	245.6	58.52	56.79	56.82
OsR498G0305397100.01	5.03	146.22	4.865338	0	0	4.03	6.28	4.78	157.52	149.65	131.48
OsR498G0305397500.01	5.603	61.246	3.533975	0	0	4.95	5.52	6.34	58.96	62.36	62.42
OsR498G0305399100.01	2.703	10.686	1.968734	1.47E-102	8.92E-103	2.38	2.63	3.1	11.01	10.6	10.45
OsR498G0305405200.01	6.293	2.56	-1.27158	9.61E-64	7.59E-64	5.9	6.53	6.45	2.51	2.37	2.8
OsR498G0305405700.01	0	0.49	7.148284	1.43E-17	2.04E-17	0	0	0	0.5	0.52	0.45
OsR498G0305408900.01	0.14	1.436	3.393397	3.32E-19	4.57E-19	0.05	0.24	0.13	1.61	1.24	1.46
OsR498G0305409500.01	0.396	4.846	3.594349	3.70E-48	3.38E-48	0.54	0.25	0.4	4.44	4.83	5.27
OsR498G0305411000.01	3.053	4.09	1.075218	1.37E-24	1.69E-24	3.1	2.88	3.18	4.37	4.67	3.23
OsR498G0305413100.01	4.3	2.05	-1.08903	4.24E-17	6.10E-17	4.88	4.18	3.84	1.74	2.28	2.13
OsR498G0305413600.01	0.49	1.03	1.054115	6.38E-06	1.33E-05	0.47	0.4	0.6	1.05	1.09	0.95
OsR498G0305414400.01	8.506	1.856	-2.21634	8.43E-80	5.88E-80	8.96	7.4	9.16	1.69	1.86	2.02
OsR498G0305416300.01	1.053	0.466	-1.12374	0.0003253	0.0007538	1.08	0.85	1.23	0.52	0.48	0.4
OsR498G0305418900.01	0.16	0.873	2.452143	1.53E-26	1.83E-26	0.11	0.18	0.19	0.95	0.84	0.83
OsR498G0305424000.01	7.96	17.266	1.111145	8.56E-131	4.53E-131	7.94	7.93	8.01	16.85	17.52	17.43
OsR498G0305425700.01	3.023	7.9	1.406203	3.62E-140	1.84E-140	2.83	3.31	2.93	7.58	7.83	8.29
OsR498G0305428000.01	0.136	0.02	-3.0477	0.0001372	0.0003095	0.12	0.13	0.16	0	0.03	0.03
OsR498G0305430600.01	23.07	10.473	-1.14043	1.33E-138	6.84E-139	21.99	23.08	24.14	10.87	10.72	9.83
OsR498G0305431600.01	0.246	0.58	1.203259	0.000137	0.000309	0.12	0.34	0.28	0.43	0.66	0.65
OsR498G0305432300.01	0.783	10.143	3.695426	1.50E-186	6.40E-187	0.93	0.8	0.62	10.58	10.39	9.46
OsR498G0305432700.01	0.403	0.086	-2.20817	3.03E-06	6.20E-06	0.3	0.36	0.55	0.04	0.17	0.05
OsR498G0305434000.01	20.193	44.416	1.134309	7.03E-220	2.70E-220	21.18	20.33	19.07	43.28	43.07	46.9
OsR498G0305434900.01	0.063	0.24	1.932845	0.0002193	0.0005019	0.11	0	0.08	0.27	0.2	0.25
OsR498G0305435900.01	6.323	2.533	-1.31544	3.02E-19	4.16E-19	6.11	7.64	5.22	3.31	2.25	2.04
OsR498G0305436400.01	1.043	0.126	-3.0477	2.40E-10	4.16E-10	1.16	1.15	0.82	0.06	0.13	0.19
OsR498G0305436600.01	2.266	1.926	-1.17483	1.20E-07	2.29E-07	2.08	2.4	2.32	2.39	0.85	2.54
OsR498G0305438000.01	3.723	0.166	-4.51483	2.37E-115	1.35E-115	4.09	3.49	3.59	0.14	0.17	0.19
OsR498G0305443800.01	9.72	3.713	-1.38525	3.85E-81	2.66E-81	9.65	9.51	10	3.38	4.15	3.61
OsR498G0305445400.01	1.17	2.873	1.266036	1.84E-15	2.75E-15	1.53	0.99	0.99	2.94	2.98	2.7
OsR498G0305446300.01	6.583	28.526	2.094852	9.82E-215	3.83E-215	7.13	6.46	6.16	28.19	29.24	28.15
OsR498G0305448700.01	22.143	7.543	-1.55693	1.36E-172	6.11E-173	22.09	21.72	22.62	7.26	8.03	7.34
OsR498G0305449900.01	0.023	0.136	2.432764	0.0001356	0.0003058	0.02	0.05	0	0.11	0.15	0.15
OsR498G0305451600.01	1.09	3.206	1.57928	5.30E-21	7.02E-21	0.84	1.2	1.23	3.2	3.6	2.82
OsR498G0305452800.01	8.666	30.2	1.791301	2.43E-286	7.75E-287	8.75	8.88	8.37	30.2	29.83	30.57
OsR498G0305456700.01	0.913	2.383	1.587248	3.17E-28	3.68E-28	0.81	1.12	0.81	2.36	2.49	2.3
OsR498G0305457400.01	1.8	0.703	-1.36832	2.61E-14	4.02E-14	1.54	1.77	2.09	0.58	0.66	0.87
OsR498G0305457800.01	0.05	0.226	2.177264	0.0002386	0.000548	0.06	0.06	0.03	0.18	0.25	0.25
OsR498G0305462300.01	29.263	9.06	-1.66878	1.46E-66	1.13E-66	29.3	30.02	28.47	11.8	5.61	9.77
OsR498G0305462500.01	31.7	15.346	-1.06562	1.05E-144	5.22E-145	31.92	33.17	30.01	15.52	15.58	14.94
OsR498G0305463200.01	1.083	0.48	-1.16784	2.36E-06	4.80E-06	1.1	1.18	0.97	0.59	0.37	0.48
OsR498G0305463800.01	154.16	788.29	2.345267	0	0	150.84	155.69	156	811.72	781.31	771.84
OsR498G0305464200.01	7.906	1.733	-2.1915	2.21E-121	1.22E-121	7.11	8.57	8.04	1.81	1.59	1.8
OsR498G0305466600.01	149.18	55.103	-1.45002	0	0	158.18	146.21	143.2	53.89	57.85	53.57
OsR498G0305467400.01	41.556	16.49	-1.33661	2.83E-282	9.10E-283	41.69	42.13	40.85	16.36	16.27	16.84
OsR498G0305467700.01	16.063	7.276	-1.18736	5.82E-100	3.59E-100	15.38	16.69	16.12	6.91	7.55	7.37
OsR498G0305468600.01	5.166	1.846	-1.48734	3.76E-40	3.73E-40	5	5.41	5.09	1.73	1.76	2.05
OsR498G0305469900.01	2.163	0.366	-2.57322	1.96E-16	2.87E-16	1.44	1.97	3.08	0.32	0.39	0.39
OsR498G0305472100.01	0.896	4.016	2.175275	4.27E-36	4.45E-36	0.73	1.02	0.94	4.18	3.91	3.96
OsR498G0305472400.01	13.426	2.303	-2.54901	0	0	12.98	13.08	14.22	2.08	2.34	2.49
OsR498G0305476500.01	0.71	0.286	-1.3865	0.0002186	0.0005004	0.47	0.85	0.81	0.24	0.47	0.15
OsR498G0305476700.01	2.706	0.83	-1.73048	6.68E-28	7.79E-28	2.51	2.62	2.99	0.76	0.75	0.98
OsR498G0305479200.01	25.043	56.24	1.246088	7.95E-291	2.50E-291	22.3	28.64	24.19	57.42	55.65	55.65
OsR498G0305483100.01	14.713	40.773	1.467029	0	0	14.62	14.95	14.57	38.65	41.08	42.59
OsR498G0305483400.01	4.933	13.153	1.423771	1.48E-170	6.67E-171	3.93	5.47	5.4	13.24	13.59	12.63

OsR498G0305484900.01	15.796	142.59	3.160959	0	0	16.1	15.67	15.62	140.82	142.32	144.63
OsR498G0305485800.01	3.96	17.13	2.108135	1.45E-131	7.66E-132	4.24	4.04	3.6	17.27	17.36	16.76
OsR498G0305487000.01	0.253	0.586	1.48556	2.43E-07	4.71E-07	0.35	0.17	0.24	0.8	0.56	0.4
OsR498G0305488100.01	0.793	4.196	2.401139	8.16E-71	6.10E-71	0.73	0.74	0.91	4.76	4.1	3.73
OsR498G0305488900.01	0.02	3.95	7.7402	4.26E-92	2.74E-92	0	0.03	0.03	4.28	3.91	3.66
OsR498G0305489600.01	0.29	1.563	2.438309	1.07E-09	1.90E-09	0.16	0.41	0.3	2.08	1.39	1.22
OsR498G0305489900.01	1.206	0.04	-4.86713	5.19E-29	5.95E-29	1.2	1.2	1.22	0.09	0.03	0
OsR498G0305490500.01	284.14	38.103	-2.91383	0	0	286.77	279.45	286.2	37.22	39.19	37.9
OsR498G0305491900.01	0.08	0	-4.28217	0.0007127	0.0016975	0.05	0.12	0.07	0	0	0
OsR498G0305507900.01	1.69	22.353	3.715325	0	0	1.78	1.4	1.89	24.07	20.76	22.23
OsR498G0305512300.01	1.96	3.973	1.008282	3.38E-12	5.52E-12	2.04	1.73	2.11	3.94	4.01	3.97
OsR498G0305514300.01	9.703	78.486	3.056472	0	0	9.72	10.54	8.85	78.75	77.69	79.02
OsR498G0305514800.01	52.256	11.46	-2.15314	0	0	55.2	49.32	52.25	11.04	11.6	11.74
OsR498G0305520600.01	13.036	4.25	-1.62174	8.57E-141	4.35E-141	11.81	13.25	14.05	4.3	4.42	4.03
OsR498G0305522200.01	2.31	0.416	-2.45849	6.41E-32	7.05E-32	2.1	2.45	2.38	0.59	0.3	0.36
OsR498G0305522400.01	5.303	15.683	1.589193	3.16E-116	1.79E-116	5.34	5.05	5.52	15.87	15.59	15.59
OsR498G0305524400.01	0.493	0.043	-3.47481	9.71E-07	1.94E-06	0.52	0.5	0.46	0	0	0.13
OsR498G0305528900.01	0.01	0.186	4.209685	1.01E-05	2.14E-05	0	0	0.03	0.12	0.22	0.22
OsR498G0305529800.01	15.606	5.873	-1.44491	7.72E-126	4.17E-126	15.04	16.06	15.72	5.23	6.05	6.34
OsR498G0305530900.01	1.06	56.75	5.701476	0	0	0.98	0.99	1.21	58.48	56.39	55.38
OsR498G0305541500.01	33.45	374	3.478521	0	0	34.7	32.73	32.92	376.4	368.12	377.47
OsR498G0305542900.01	12.016	56.796	2.237055	0	0	12	11.92	12.13	56.22	58.25	55.92
OsR498G0305543200.01	1.62	0.81	-1.00331	8.51E-07	1.70E-06	1.6	1.78	1.48	0.83	0.62	0.98
OsR498G0305544000.01	0.236	0	-5.28217	2.04E-06	4.14E-06	0.25	0.22	0.24	0	0	0
OsR498G0305546400.01	0.056	1.593	4.783921	7.62E-33	8.28E-33	0.03	0.11	0.03	1.93	1.63	1.22
OsR498G0305548200.01	4.073	10.663	1.404506	1.36E-54	1.16E-54	1.1	7.72	3.4	10.1	12.89	9
OsR498G0305548400.01	23.803	70.226	1.586122	0	0	23.57	23.7	24.14	70.08	68.86	71.74
OsR498G0305549800.01	6.646	2.24	-1.57213	8.43E-54	7.28E-54	6.8	7.07	6.07	2.12	2.18	2.42
OsR498G0305550100.01	2.14	0.933	-1.14869	2.61E-15	3.93E-15	2.03	2	2.39	0.9	0.97	0.93
OsR498G0305550700.01	3.463	1.81	-1.01202	1.61E-05	3.43E-05	0	0	10.39	1.91	2.8	0.72
OsR498G0305554600.01	2.936	1.063	-1.57217	2.41E-26	2.88E-26	2.7	2.72	3.39	0.63	1.33	1.23
OsR498G0305554600.01.[illegible]	2.706	1.22	-1.17867	5.05E-19	6.98E-19	2.99	2.28	2.85	1.36	0.91	1.39
OsR498G0305555700.01	7.226	23.113	1.672718	2.31E-227	8.70E-228	7.82	7	6.86	22.85	23.29	23.2
OsR498G0305556500.01	120.77	29.373	-2.08016	0	0	119.68	124.16	118.5	30.06	27.3	30.76
OsR498G0305557100.01	1.12	3.3	1.584081	4.46E-26	5.36E-26	1.17	1.16	1.03	3.09	3.14	3.67
OsR498G0305559500.01	28.22	12.633	-1.19745	3.68E-121	2.04E-121	29.67	25.81	29.18	12.24	13.01	12.65
OsR498G0305570700.01	7.423	22.556	1.703392	7.78E-163	3.60E-163	4.63	9.69	7.95	22.96	26.18	18.53
OsR498G0305572700.01	3.363	0.68	-2.55498	5.78E-43	5.59E-43	3.03	3.77	3.29	0.64	0.57	0.83
OsR498G0305573700.01	0.893	24.41	4.746285	0	0	0.94	0.92	0.82	25.58	24.04	23.61
OsR498G0305574600.01	14.01	52.286	1.902331	0	0	13.55	14.55	13.93	51.18	51.87	53.81
OsR498G0305575000.01	6.313	19.013	1.586863	6.94E-135	3.62E-135	6.68	6.57	5.69	19.77	19.43	17.84
OsR498G0305576600.01	38	79.013	1.05186	0	0	37.79	39.54	36.67	76.56	79.58	80.9
OsR498G0305578700.01	2.383	5.713	1.297148	1.09E-36	1.13E-36	2.4	2.52	2.23	6.38	5.36	5.4
OsR498G0305579400.01	0.126	0	-4.28217	0.0007127	0.0016975	0.15	0.16	0.07	0	0	0
OsR498G0305581600.01	0.293	0.073	-2.03063	0.0006092	0.0014421	0.19	0.25	0.44	0.12	0.04	0.06
OsR498G0305582100.01	1.68	0.353	-2.28361	0.00035	0.0008129	1.25	1.86	1.93	0.53	0.26	0.27
OsR498G0305586800.01	85.533	1.106	-6.4047	0	0	81.34	87.2	88.06	0.94	1.03	1.35
OsR498G0305587100.01	28.9	123.77	2.104557	0	0	29.04	28.62	29.04	120.82	125.69	124.79
OsR498G0305587400.01	0.263	0	-5.28217	2.04E-06	4.14E-06	0.19	0.45	0.15	0	0	0
OsR498G0305589000.01	11.683	3.126	-1.92645	1.36E-22	1.74E-22	11.72	11.53	11.8	2.76	2.79	3.83
OsR498G0305589200.01	19.023	7.44	-1.35822	4.55E-70	3.42E-70	17.61	19.62	19.84	9.12	6.14	7.06
OsR498G0305593900.01	0	0.46	6.432077	9.00E-12	1.49E-11	0	0	0	0.35	0.47	0.56
OsR498G0305597100.01	1.5	3.363	1.158517	2.34E-19	3.21E-19	1.62	1.34	1.54	3.37	3.44	3.28
OsR498G0305598600.01	28.573	58.946	1.050752	9.46E-216	3.68E-216	28.3	28.41	29.01	57.66	59.38	59.8
OsR498G0305599700.01	0.293	0.86	1.590913	1.17E-07	2.24E-07	0.43	0.26	0.19	0.59	1.31	0.68
OsR498G0305600000.01	5.223	55.476	3.423628	0	0	5.53	4.9	5.24	52.61	56.07	57.75
OsR498G0305602400.01	1.543	24.33	3.973671	1.11E-217	4.30E-218	1.71	1.66	1.26	25.19	24.33	23.47
OsR498G0305606200.01	0.386	1.586	2.022482	9.73E-20	1.32E-19	0.47	0.26	0.43	1.89	1.4	1.47
OsR498G0305607600.01	0.646	1.416	1.136333	3.01E-08	5.63E-08	0.5	0.93	0.51	1.71	1.25	1.29
OsR498G0305608500.01	0.156	0.68	2.115048	1.48E-08	2.73E-08	0.1	0.18	0.19	0.71	0.77	0.56
OsR498G0305612100.01	1.24	0.283	-2.15006	4.14E-10	7.23E-10	1.57	1.22	0.93	0.26	0.27	0.32
OsR498G0305612600.01	6.65	13.64	1.034617	2.07E-68	1.58E-68	6.93	6.61	6.41	14.43	13.48	13.01
OsR498G0305615900.01	17.426	0.983	-4.17436	9.76E-198	4.01E-198	18.54	16.28	17.46	1.09	0.96	0.9
OsR498G0305621000.01	9.523	23.823	1.314054	3.06E-182	1.33E-182	10.05	8.65	9.87	24.51	23.18	23.78
OsR498G0305622000.01	6.55	26.23	2.015452	0	0	5.85	7.32	6.48	24.65	26.86	27.18
OsR498G0305623100.01	13.156	39.933	1.59075	0	0	13.52	13.27	12.68	39.73	39.65	40.42
OsR498G0305625900.01	10.823	3.983	-1.61186	2.99E-105	1.79E-105	10.52	11.28	10.67	3.73	4.28	3.94
OsR498G0305626500.01	69.053	266.06	1.923345	0	0	69.7	68.25	69.21	270.58	263.62	263.97
OsR498G0305627100.01	15.003	5.216	-1.56099	5.05E-183	2.19E-183	15.03	15.04	14.94	5.16	5.44	5.05

OsR498G0305627400.01	5.313	11.743	1.129059	9.06E-47	8.40E-47	4.88	5.8	5.26	11.64	12.48	11.11
OsR498G0305629300.01	6.34	50.563	2.959351	0	0	5.75	6.76	6.51	49.1	51.69	50.9
OsR498G0305629600.01	11.07	48.583	2.129911	0	0	11.63	10.42	11.16	48.49	48.55	48.71
OsR498G0305631400.01	0.3	0.94	1.653292	2.27E-14	3.49E-14	0.21	0.43	0.26	0.72	1.03	1.07
OsR498G0305632600.01	1.35	0.126	-3.40555	2.00E-58	1.66E-58	1.31	1.4	1.34	0.13	0.12	0.13
OsR498G0305632700.01	66.673	17.063	-1.90944	0	0	67.49	65.49	67.04	16.14	16.94	18.11
OsR498G0305635800.01	5.703	0.95	-2.62152	9.94E-77	7.08E-77	6.2	5.74	5.17	1.13	0.99	0.73
OsR498G0305636100.01	1.3	0.303	-2.10708	4.04E-12	6.61E-12	1.13	1.23	1.54	0.43	0.26	0.22
OsR498G0305636700.01	3.143	0.633	-2.25573	7.40E-12	1.22E-11	3.77	1.99	3.67	0.7	0.73	0.47
OsR498G0305638100.01	4.35	0.82	-2.40995	4.19E-79	2.94E-79	4.32	4.4	4.33	0.75	0.88	0.83
OsR498G0305638300.01	1.803	3.95	1.123772	2.04E-09	3.63E-09	1.14	2.14	2.13	3.7	3.38	4.77
OsR498G0305639000.01	2.16	0.73	-1.5896	2.82E-09	5.05E-09	2.35	1.92	2.21	0.54	0.9	0.75
OsR498G0305641600.01	9.226	3.713	-1.31733	8.82E-102	5.39E-102	8.69	9.79	9.2	3.48	4.3	3.36
OsR498G0305644600.01	0.356	0.883	1.397312	4.82E-07	9.49E-07	0.38	0.29	0.4	0.91	0.9	0.84
OsR498G0305645600.01	1.03	13.876	3.742584	3.94E-247	1.39E-247	0.83	1.11	1.15	13.95	14.25	13.43
OsR498G0305650300.01	8.686	34.866	2.003682	0	0	9.03	8.78	8.25	37.56	34.21	32.83
OsR498G0305650800.01	0.35	0.97	1.475556	0.0004608	0.0010797	0.3	0.44	0.31	1.39	0.68	0.84
OsR498G0305651500.01	0.096	2	4.34093	1.71E-32	1.86E-32	0.08	0.13	0.08	2.18	1.99	1.83
OsR498G0305652000.01	1.186	4.793	1.989134	1.85E-25	2.25E-25	1.17	0.78	1.61	5.05	5.01	4.32
OsR498G0305652300.01	4.653	17.146	1.882776	7.09E-194	2.96E-194	4.67	4.78	4.51	17.45	16.95	17.04
OsR498G0305653500.01	10.516	4.956	-1.08813	1.03E-50	9.14E-51	10.48	10.32	10.75	4.86	4.98	5.03
OsR498G0305653700.01	7.046	2.833	-1.31455	2.87E-69	2.17E-69	6.91	7.4	6.83	2.82	2.96	2.72
OsR498G0305653900.01	0.03	0.16	2.413884	0.0007762	0.001853	0.03	0	0.06	0.26	0.16	0.06
OsR498G0305654200.01	9.093	2.883	-1.67589	3.45E-86	2.31E-86	9.64	9.03	8.61	2.2	3.29	3.16
OsR498G0305654300.01	28.276	9.596	-1.53743	5.53E-68	4.23E-68	28.58	28.59	27.66	10.17	9.62	9
OsR498G0305655100.01	4.616	16.783	1.833074	1.80E-117	1.01E-117	4.82	4.97	4.06	17.47	17.04	15.84
OsR498G0305659300.01	25.36	8.166	-1.63784	1.31E-146	6.48E-147	24.99	25.68	25.41	8.24	8.05	8.21
OsR498G0305660600.01	26.276	4.713	-2.48798	1.76E-48	1.60E-48	25.36	29.06	24.41	4.31	5.63	4.2
OsR498G0305665500.01	52.906	152.96	1.526319	0	0	53.9	53.65	51.17	150.94	153.22	154.72
OsR498G0305665700.01	0.513	1.483	1.548774	0.0001666	0.0003778	0.47	0.5	0.57	1.58	1.46	1.41
OsR498G0305667700.01	28.966	65.23	1.158694	0	0	28.49	30.16	28.25	62.48	66.62	66.59
OsR498G0305669400.01	6.983	32.21	2.084759	0	0	6.96	6.66	7.33	32.23	31.99	32.41
OsR498G0305672500.01	9.39	32.173	1.733503	0	0	9.62	9.3	9.25	31.42	32.23	32.87
OsR498G0305677200.01	0.753	0.363	-1.02841	0.0003163	0.0007321	0.65	1.08	0.53	0.45	0.36	0.28
OsR498G0305678500.01	130.01	679.64	2.402053	0	0	119.88	134.91	135.3	705.98	666.1	666.83
OsR498G0305678800.01	0.136	1.736	3.676735	2.76E-15	4.15E-15	0.21	0.2	0	1.93	1.4	1.88
OsR498G0305679000.01	9.893	4.846	-1.04461	1.56E-31	1.73E-31	10.16	9.59	9.93	5.47	4.74	4.33
OsR498G0305680000.01	0.02	0.16	2.946651	0.0003132	0.0007244	0.03	0	0.03	0.25	0.1	0.13
OsR498G0305682600.01	0	0.883	6.7402	5.56E-14	8.64E-14	0	0	0	1	0.52	1.13
OsR498G0305682800.01	0.016	0.786	5.499192	2.38E-12	3.86E-12	0.05	0	0	0.9	0.6	0.86
OsR498G0305684300.01	17.196	137.99	3.005687	0	0	17.76	16.64	17.19	137.11	139.82	137.03
OsR498G0305687200.01	884.95	348.09	-1.35575	0	0	900.2	862.19	892.5	373.26	341.93	329.08
OsR498G0305689200.01	2.716	0.503	-2.43356	3.66E-36	3.81E-36	2.8	2.7	2.65	0.53	0.46	0.52
OsR498G0305690400.01	11.916	1.25	-3.2628	8.92E-133	4.67E-133	12.22	12.15	11.38	1.23	1.47	1.05
OsR498G0305692900.01	0.21	0.52	1.30724	1.58E-05	3.37E-05	0.07	0.3	0.26	0.51	0.58	0.47
OsR498G0305693600.01	19.62	71.603	1.866097	0	0	18.71	20.33	19.82	68.73	72.44	73.64
OsR498G0305694300.01	17.363	0.336	-5.70261	4.08E-188	1.74E-188	17.46	16.9	17.73	0.33	0.48	0.2
OsR498G0305706800.01	0.493	234.83	8.960191	0	0	0.26	0.33	0.89	221.82	238.14	244.52
OsR498G0305707200.01	205.91	879.61	2.082005	0	0	208.85	201.17	207.7	896.52	860.26	882.05
OsR498G0305707600.01	2.396	13.826	2.527743	2.91E-113	1.67E-113	2.53	2.71	1.95	14.59	12.99	13.9
OsR498G0305708400.01	56.066	28.04	-1.01878	2.92E-180	1.28E-180	57.23	54.49	56.48	26.74	29.87	27.51
OsR498G0305709700.01	2.083	6.27	1.587964	8.88E-26	1.07E-25	2.39	2.22	1.64	6.14	6.52	6.15
OsR498G0305710000.01	339.37	88.513	-1.92234	0	0	335.59	338.66	343.9	84.89	88.67	91.98
OsR498G0305711600.01	16.88	61.783	1.883141	0	0	18.06	16.02	16.56	60.07	61.82	63.46
OsR498G0305712500.01	1.17	0.17	-2.78387	4.15E-07	8.14E-07	1.04	1.64	0.83	0.09	0.31	0.11
OsR498G0305712700.01	0.443	0.936	1.072477	1.30E-05	2.76E-05	0.29	0.49	0.55	0.89	0.95	0.97
OsR498G0305714300.01	0.74	18.686	4.663836	9.69E-267	3.24E-267	0.84	0.88	0.5	19.48	19.34	17.24
OsR498G0305715700.01	125.32	265.24	1.065749	0	0	125.93	122.86	127.2	269.48	267.81	258.43
OsR498G0305716200.01	2.253	110.53	5.620355	0	0	2.78	2.28	1.7	115.72	108.02	107.85
OsR498G0305718500.01	27.763	98.763	1.832077	0	0	26.82	29.54	26.93	101.14	96.28	98.87
OsR498G0305720700.01	4.156	1.4	-1.57822	1.00E-34	1.06E-34	4.31	4.11	4.05	1.37	1.27	1.56
OsR498G0305721200.01	0.096	10.77	6.781227	2.15E-76	1.54E-76	0.1	0	0.19	11.02	11.09	10.2
OsR498G0305721600.01	26.19	76.446	1.533502	0	0	25.88	26.3	26.39	75.56	77.21	76.57
OsR498G0305722400.01	0.033	0.356	3.499192	4.83E-06	9.99E-06	0	0.05	0.05	0.38	0.35	0.34
OsR498G0305723500.01	1.043	0.17	-2.75165	5.87E-29	6.74E-29	0.95	1.08	1.1	0.16	0.23	0.12
OsR498G0305724100.01	0.336	0.083	-1.89884	0.0006013	0.0014228	0.36	0.13	0.52	0.08	0.13	0.04
OsR498G0305724500.01	2.423	332.5	7.08528	0	0	2.84	2.12	2.31	350.15	324.39	322.96
OsR498G0305726700.01	5.686	2.573	-1.3708	1.48E-71	1.09E-71	5.32	5.74	6	2.9	1.98	2.84
OsR498G0305727200.01	6.34	24.176	1.927469	5.19E-160	2.43E-160	6.87	6.46	5.69	26.03	22.96	23.54

OsR498G0305728500.01	43.756	132.34	1.595012	0	0	42.9	43.84	44.53	127.45	134.53	135.03
OsR498G0305728900.01	10.72	21.406	1.004427	3.24E-61	2.62E-61	11.64	9.93	10.59	22.58	19.93	21.71
OsR498G0305729500.01	0.143	0.39	1.432077	0.0001554	0.0003517	0.05	0.25	0.13	0.32	0.39	0.46
OsR498G0305731000.01	0	1.593	8.440639	4.14E-36	4.31E-36	0	0	0	1.27	1.83	1.68
OsR498G0305736400.01	0.043	1.313	4.925892	3.88E-50	3.47E-50	0.07	0	0.06	1.2	1.54	1.2
OsR498G0305739300.01	7.576	98.85	3.702053	0	0	8.78	7.24	6.71	98.61	100.37	97.57
OsR498G0305744600.01	18.33	6.99	-1.33821	0	0	17.61	18.75	18.63	6.59	7.09	7.29
OsR498G0305744700.01	2.176	0.383	-2.51459	3.92E-20	5.28E-20	1.93	2.14	2.46	0.28	0.3	0.57
OsR498G0305746000.01	0.42	0.02	-3.51544	3.14E-06	6.42E-06	0.24	0.29	0.73	0.03	0	0.03
OsR498G0305746100.01	1.426	0.023	-6.04228	2.71E-33	2.92E-33	1.6	1.45	1.23	0	0.07	0
OsR498G0305746700.01	1.316	0.556	-1.2557	0.0001341	0.0003022	1.24	1.15	1.56	0.75	0.31	0.61
OsR498G0305747600.01	11.146	4.72	-1.2279	4.01E-62	3.21E-62	9.86	11.68	11.9	4.76	4.79	4.61
OsR498G0305749600.01	0.78	0.116	-2.71827	5.81E-19	8.04E-19	0.83	0.87	0.64	0.1	0.08	0.17
OsR498G0305752300.01	0	0.453	5.7402	4.87E-08	9.17E-08	0	0	0	0.62	0.32	0.42
OsR498G0305753300.01	0.07	0.51	2.946651	0.0003132	0.0007244	0	0.21	0	0.71	0.1	0.72
OsR498G0305756400.01	11.733	0.94	-3.64707	5.13E-151	2.49E-151	12.23	11.5	11.47	0.87	0.91	1.04
OsR498G0305760200.01	2.52	0.79	-1.67234	1.66E-54	1.42E-54	2.15	2.72	2.69	0.58	0.7	1.09
OsR498G0305761900.01	0.276	18.603	6.06912	5.88E-297	1.82E-297	0.35	0.27	0.21	20.51	17.09	18.21
OsR498G0305764800.01	0.473	1.36	1.512696	1.99E-13	3.13E-13	0.34	0.49	0.59	1.39	1.57	1.12
OsR498G0305765300.01	27.38	12.12	-1.16938	5.67E-124	3.09E-124	28.8	26	27.34	12.67	12.28	11.41
OsR498G0305767400.01	0.52	0.046	-3.5452	0.0005186	0.0012198	0.51	0.68	0.37	0	0.14	0
OsR498G0305767800.01	15.22	36.686	1.260501	1.53E-112	8.82E-113	13.85	15.52	16.29	36.71	34.6	38.75
OsR498G0305769300.01	0.35	1.163	1.734347	1.29E-12	2.08E-12	0.52	0.26	0.27	1.28	1.21	1
OsR498G0305770700.01	0.263	2.943	3.468898	4.64E-74	3.38E-74	0.36	0.19	0.24	3.19	2.89	2.75
OsR498G0305773700.01	18.883	118.33	2.743314	0	0	19.29	18.1	19.26	118.33	118.11	118.54
OsR498G0305774500.01	5.71	2.05	-1.51606	1.74E-22	2.24E-22	5.92	5.49	5.72	1.42	2.85	1.88
OsR498G0305774900.01	0.033	0.57	4.03976	1.16E-08	2.14E-08	0	0.05	0.05	0.58	0.43	0.7
OsR498G0305776300.01	1.323	0.413	-1.69721	6.29E-11	1.07E-10	1.26	1.25	1.46	0.48	0.61	0.15
OsR498G0305782200.01	6.77	0.286	-4.52318	4.94E-59	4.07E-59	8	5.97	6.34	0.39	0.08	0.39
OsR498G0305784900.01	0.313	3.493	3.460092	1.10E-36	1.14E-36	0.39	0.12	0.43	3.45	3.62	3.41
OsR498G0305786300.01	1.426	3.596	1.298475	8.00E-11	1.36E-10	1.39	1.78	1.11	3.31	2.73	4.75
OsR498G0305790900.01	18.296	75.553	2.036746	0	0	18.53	18.33	18.03	74.95	77.3	74.41
OsR498G0305793500.01	0.513	0.156	-1.78621	3.12E-05	6.76E-05	0.67	0.52	0.35	0.12	0.24	0.11
OsR498G0305796200.01	12.326	47.97	1.959638	0	0	12.19	12.05	12.74	46.58	48.49	48.84
OsR498G0305797300.01	11.94	5.066	-1.24472	9.87E-144	4.95E-144	11.37	11.81	12.64	5.3	5.36	4.54
OsR498G0305801300.01	0.366	5.22	3.824395	1.38E-92	8.85E-93	0.24	0.43	0.43	5.51	4.73	5.42
OsR498G0305802300.01	63.65	140.29	1.129646	0	0	78.85	53.73	58.37	140.42	147.48	132.96
OsR498G0305804100.01	199.11	77.513	-1.40893	0	0	198.89	198.33	200.1	71.69	79.55	81.3
OsR498G0305804600.01	2.386	0.42	-2.50572	1.01E-71	7.48E-72	2.22	2.39	2.55	0.51	0.32	0.43
OsR498G0305805500.01	0.06	0.456	3.03976	6.82E-06	1.42E-05	0.06	0.12	0	0.56	0.64	0.17
OsR498G0305812400.01	0.51	0.17	-1.5452	1.11E-05	2.35E-05	0.57	0.35	0.61	0.12	0.23	0.16
OsR498G0305813300.01	22.623	6.723	-1.76532	1.47E-222	5.60E-223	22.57	22.54	22.76	6.57	6.69	6.91
OsR498G0305815000.01	15.863	34.72	1.124179	8.85E-179	3.89E-179	14.84	15.81	16.94	35.2	34.34	34.62
OsR498G0305816700.01	92.456	27.283	-1.7476	0	0	97.67	89.71	89.99	30.98	25.47	25.4
OsR498G0305817600.01	12.71	57.19	2.171721	0	0	12.95	13.04	12.14	55.8	56.92	58.85
OsR498G0305819800.01	0.226	0.613	1.428802	2.08E-05	4.45E-05	0.32	0.24	0.12	0.66	0.58	0.6
OsR498G0305823100.01	6.843	27.423	1.986973	1.48E-204	5.94E-205	7.5	6.19	6.84	27.89	26.87	27.51
OsR498G0305824900.01	10.146	48.84	2.285702	0	0	9.77	9.76	10.91	48.49	49.59	48.44
OsR498G0305826500.01	0.216	0.77	1.83494	2.15E-07	4.16E-07	0.15	0.32	0.18	0.96	0.48	0.87
OsR498G0305829400.01	4.706	14.91	1.679081	1.98E-157	9.36E-158	4.52	5.03	4.57	14.65	15.04	15.04
OsR498G0305829600.01	37.093	108.4	1.525439	0	0	37.14	36.83	37.31	104.5	113.81	106.9
OsR498G0305830700.01	11.666	281.16	4.583048	0	0	12.49	11.66	10.85	304.66	270.42	268.39
OsR498G0305836200.01	0.753	1.776	1.228205	6.23E-07	1.23E-06	0.89	0.71	0.66	1.82	2.04	1.47
OsR498G0305839500.01	7.023	0.676	-3.42562	1.81E-91	1.17E-91	7.39	6.18	7.5	0.62	1.05	0.36
OsR498G0305839700.01	11.376	24.753	1.115086	1.93E-63	1.53E-63	11.45	11.57	11.11	26.15	25.68	22.43
OsR498G0305840000.01	10.15	2.693	-1.93461	2.04E-29	2.32E-29	10.24	8.95	11.26	2.92	2.53	2.63
OsR498G0305840900.01	1.82	0.21	-3.05827	8.95E-25	1.10E-24	2.1	1.56	1.8	0.27	0.16	0.2
OsR498G0305842800.01	19.266	52.316	1.421352	0	0	18.71	19.88	19.21	51.06	52.5	53.39
OsR498G0305844700.01	5.283	130.83	4.626802	0	0	4.89	6.07	4.89	131.86	127.4	133.24
OsR498G0305845000.01	1.163	6.966	2.761225	5.05E-117	2.85E-117	0.78	1.34	1.37	7.6	6.68	6.62
OsR498G0305846000.01	0.65	6.766	3.38429	3.96E-38	4.03E-38	0.5	0.96	0.49	8.42	5.43	6.45
OsR498G0305846700.01	90.66	8.84	-3.39364	0	0	92.6	85.65	93.73	8.36	10.05	8.11
OsR498G0305847200.01	1.53	0.103	-3.86999	3.53E-17	5.08E-17	1.91	1.49	1.19	0	0.25	0.06
OsR498G0305847900.01	13.183	51.643	1.972305	0	0	13.51	13.11	12.93	50.33	51.4	53.2
OsR498G0305849300.01	16.306	4.463	-1.87641	1.67E-101	1.02E-101	16.62	16.02	16.28	4.57	4.63	4.19
OsR498G0305854800.01	0.4	11.08	4.7849	0	0	0.3	0.36	0.54	10.81	11.32	11.11
OsR498G0305855900.01	1.42	17.453	3.623729	6.17E-289	1.95E-289	1.41	1.44	1.41	18.54	17.62	16.2
OsR498G0305857300.01	61.576	28.226	-1.12856	3.54E-299	1.09E-299	62.56	61.09	61.08	27.71	28.48	28.49
OsR498G0305859800.01	2.006	18.98	3.247868	0	0	1.82	2.16	2.04	18.72	18.81	19.41

OsR498G0305860200.01	0.143	1.77	3.607445	4.80E-51	4.26E-51	0.12	0.15	0.16	1.97	1.56	1.78
OsR498G0305862400.01	300.1	127.42	-1.25667	0	0	311.57	301.03	287.7	132.57	126.41	123.29
OsR498G0305863600.01	2.05	62.606	4.927535	0	0	1.79	2.17	2.19	65.38	61.64	60.8
OsR498G0305865600.01	3.426	1.57	-1.12076	6.45E-16	9.55E-16	3.18	3.78	3.32	1.8	1.44	1.47
OsR498G0305867600.01	6.356	127.92	4.330319	0	0	6.25	6.96	5.86	130.47	124.98	128.3
OsR498G0305867700.01	0	1.406	4.770943	7.85E-05	0.0001741	0	0	0	2.1	1.75	0.37
OsR498G0305870500.01	5.836	15.663	1.419963	0	0	5.62	6.1	5.79	15.15	15.17	16.67
OsR498G0305874500.01	0.07	0	-4.28217	0.0007127	0.0016975	0.08	0.07	0.06	0	0	0
OsR498G0305876600.01	3.28	0.29	-3.41774	8.46E-79	5.95E-79	2.96	3.82	3.06	0.32	0.3	0.25
OsR498G0305877700.01	9.083	3.71	-1.28495	8.81E-101	5.41E-101	8.01	9.07	10.17	4.03	3.63	3.47
OsR498G0305879300.01	1.37	0.27	-2.3449	1.28E-19	1.74E-19	1.51	1.31	1.29	0.24	0.24	0.33
OsR498G0305882800.01	20.81	71.526	1.772957	0	0	20.89	20.88	20.66	74.36	71.75	68.47
OsR498G0305884200.01	11.18	1.186	-3.33978	5.49E-154	2.63E-154	10.69	11.68	11.17	1.3	1.04	1.22
OsR498G0305884500.01	1.713	0.236	-2.86713	1.23E-13	1.92E-13	1.77	1.79	1.58	0	0.21	0.5
OsR498G0305886000.01	61.763	14.933	-2.05908	0	0	60.4	62.58	62.31	15.19	13.94	15.67
OsR498G0305887500.01	0.623	0.063	-3.36234	2.51E-15	3.77E-15	0.5	0.61	0.76	0.03	0.08	0.08
OsR498G0305888900.01	244.07	16.106	-3.93066	0	0	244.06	236.71	251.4	17.65	15.44	15.23
OsR498G0305890800.01	8.12	49.123	2.598701	0	0	7.52	8.4	8.44	46.64	49.65	51.08
OsR498G0305891500.01	1.22	19.026	3.946119	0	0	1.08	1.21	1.37	18.99	19.56	18.53
OsR498G0305894900.01	0.143	9.923	6.024653	9.32E-183	4.05E-183	0.25	0.04	0.14	11.32	9.03	9.42
OsR498G0305899100.01	19.55	6.326	-1.62111	1.14E-81	7.86E-82	20.81	17.99	19.85	6.8	5.86	6.32
OsR498G0305903100.01	19.13	43.566	1.158295	4.28E-271	1.42E-271	16.32	20.98	20.09	43.2	44.12	43.38
OsR498G0305904000.01	18.293	38.923	1.092889	1.32E-176	5.86E-177	19.11	17.41	18.36	39.83	37.38	39.56
OsR498G0305907000.01	2.883	7.3	1.306303	1.76E-44	1.68E-44	3.04	2.68	2.93	6.73	7.72	7.45
OsR498G0305910100.01	1.036	0	-7.72842	6.20E-25	7.62E-25	0.96	1.25	0.9	0	0	0
OsR498G0305910600.01	58.386	2.9	-4.29588	0	0	57.12	60.77	57.27	2.53	3.12	3.05
OsR498G0305912400.01	4.84	1.153	-2.14395	2.37E-43	2.28E-43	4.63	5.05	4.84	1.54	0.82	1.1
OsR498G0305915600.01	7.926	18.393	1.218647	4.73E-137	2.44E-137	7.77	8.25	7.76	18.17	18.38	18.63
OsR498G0305916700.01	6.386	15.276	1.294574	3.33E-79	2.33E-79	6.97	6.41	5.78	15.23	15.07	15.53
OsR498G0305920400.01	1.826	0.393	-2.22011	1.40E-12	2.27E-12	1.56	2.07	1.85	0.24	0.57	0.37
OsR498G0305921800.01	314.1	29.986	-3.42058	0	0	326.02	310.56	305.7	31.41	27.38	31.17
OsR498G0305923500.01	46.073	8.163	-2.50204	0	0	48.57	43.58	46.07	9.01	7.88	7.6
OsR498G0305924900.01	429.04	16.513	-4.62195	0	0	453.43	424.69	409	19.04	16.51	13.99
OsR498G0305931400.01	34.063	16.4	-1.12367	1.15E-111	6.64E-112	26.42	37.03	38.74	16.34	16.28	16.58
OsR498G0305944200.01	0.296	2.57	3.060822	1.33E-25	1.61E-25	0.15	0.29	0.45	2.59	2.21	2.91
OsR498G0305945000.01	1.056	0.283	-1.90086	2.43E-13	3.84E-13	1.01	1.11	1.05	0.44	0.13	0.28
OsR498G0305948900.01	6.546	15.023	1.290204	1.77E-58	1.46E-58	7.08	5.05	7.51	16.82	13.33	14.92
OsR498G0305952600.01	0.12	0.746	2.624723	0.0002134	0.0004876	0.24	0.12	0	0.86	0.88	0.5
OsR498G0305955900.01	1.216	0.26	-2.24975	4.42E-07	8.69E-07	1.24	0.88	1.53	0.43	0.26	0.09
OsR498G0305957500.01	179.27	398.9	1.147198	0	0	176.21	179.9	181.7	435.88	372.04	388.77
OsR498G0305969100.01	4.47	1.983	-1.18263	6.36E-13	1.02E-12	4.04	4.79	4.58	2.08	2.19	1.68
OsR498G0305969400.01	1.52	9.203	2.594349	4.49E-104	2.71E-104	1.46	1.78	1.32	9.17	9.81	8.63
OsR498G0305972400.01	0.39	4.373	3.474388	4.47E-35	4.73E-35	0.51	0.38	0.28	4.48	4.53	4.11
OsR498G0305973900.01	0.793	0.22	-1.84658	3.54E-13	5.61E-13	0.7	0.95	0.73	0.24	0.32	0.1
OsR498G0305979000.01	11.69	4.04	-1.55669	2.52E-130	1.34E-130	11.74	11.59	11.74	3.86	4.29	3.97
OsR498G0305981700.01	1.583	0.273	-2.5452	1.25E-07	2.39E-07	1.35	1.77	1.63	0.35	0	0.47
OsR498G0305983800.01	0.013	0.19	3.847115	0.0001179	0.0002648	0	0	0.04	0.24	0.25	0.08
OsR498G0305986500.01	0	0.603	6.624723	4.17E-13	6.62E-13	0	0	0	0.52	0.69	0.6
OsR498G0305988200.01	13.056	6.096	-1.106	4.26E-59	3.50E-59	13.41	12.57	13.19	6.5	5.89	5.9
OsR498G0305990700.01	25.96	10.27	-1.12479	1.96E-78	1.38E-78	26.09	25.28	26.51	11.52	10.43	8.86
OsR498G0305991100.01	95.543	208.15	1.062925	0	0	93.22	101.84	91.57	215.15	206.64	202.66
OsR498G0305997700.01	1.056	2.493	1.222732	1.31E-20	1.75E-20	0.96	0.9	1.31	2.19	3.07	2.22
OsR498G0305998700.01	10.743	5.156	-1.07271	2.47E-31	2.74E-31	10.29	11.41	10.53	5.13	5.03	5.31
OsR498G0305999000.01	14.103	5.61	-1.33407	1.48E-83	1.00E-83	14.06	13.42	14.83	5.67	5.22	5.94
OsR498G0306000200.01	0.53	1.576	1.572634	3.36E-12	5.49E-12	0.58	0.46	0.55	1.75	1.6	1.38
OsR498G0306001300.01	15.063	80.553	2.413671	0	0	14.09	14.89	16.21	85.58	80.21	75.87
OsR498G0306007000.01	12.62	3.75	-1.77071	2.18E-42	2.12E-42	13.24	13.24	11.38	3.83	4.45	2.97
OsR498G0306009000.01	5.296	0.543	-3.27751	1.23E-111	7.12E-112	4.61	5.79	5.49	0.75	0.51	0.37
OsR498G0306019600.01	29.133	14.403	-1.01305	1.32E-112	7.64E-113	30.21	28.12	29.07	13.42	15.49	14.3
OsR498G0306020500.01	4.166	1.616	-1.55361	2.72E-64	2.14E-64	4.17	3.98	4.35	1.99	1.57	1.29
OsR498G0306021700.01	0.17	0.03	-2.41967	0.0001248	0.0002807	0.21	0.19	0.11	0.07	0	0.02
OsR498G0306022000.01	1.78	0.36	-2.30601	2.30E-09	4.12E-09	1.82	2.18	1.34	0.36	0.36	0.36
OsR498G0306023100.01	0.333	1.596	2.256078	1.30E-11	2.17E-11	0.29	0.37	0.34	1.31	1.72	1.76
OsR498G0306023800.01	2.47	0.873	-1.50277	1.94E-06	3.93E-06	1.86	3.23	2.32	0.9	0.69	1.03
OsR498G0306024100.01	6.513	3.236	-1.00984	9.44E-42	9.23E-42	6.44	6.71	6.39	3.6	3.23	2.88
OsR498G0306024700.01	0.233	0.073	-2.96024	0.000243	0.0005585	0.19	0.32	0.19	0.11	0.11	0
OsR498G0306025900.01	112.6	8.476	-3.75121	0	0	104.43	115.77	117.6	7.86	8.47	9.1
OsR498G0306032700.01	2.766	28.883	3.431054	0	0	2.79	3.13	2.38	29.52	30.32	26.81
OsR498G0306037400.01	1.043	0.406	-1.29275	1.96E-07	3.79E-07	1.29	0.9	0.94	0.54	0.37	0.31

OsR498G0306043500.01	4.953	2.316	-1.10235	4.84E-26	5.82E-26	4.68	5.17	5.01	2.19	2.5	2.26
OsR498G0306044400.01	11.416	1.856	-2.53106	6.29E-169	2.85E-169	10.73	11.95	11.57	1.7	2.02	1.85
OsR498G0306047600.01	0.97	0.073	-3.76759	2.26E-07	4.38E-07	0.72	1.09	1.1	0.11	0.11	0
OsR498G0306048500.01	50.486	12.96	-1.97118	0	0	53.78	51.69	45.99	13.51	12.93	12.44
OsR498G0306058200.01	0.58	2.816	1.959792	1.05E-41	1.02E-41	0.44	0.66	0.64	2.92	3.05	2.48
OsR498G0306060700.01	14.67	2.743	-2.42655	1.76E-87	1.17E-87	14.82	14.74	14.45	2.99	2.47	2.77
OsR498G0306061200.01	0.703	0.106	-2.73159	0.0002387	0.0005481	0.55	0.68	0.88	0.16	0	0.16
OsR498G0306062300.01	4.933	11.296	1.193187	1.18E-63	9.36E-64	5.06	4.77	4.97	11.21	11.4	11.28
OsR498G0306065700.01	0.696	3.093	2.138494	1.45E-31	1.60E-31	0.77	0.67	0.65	3.07	2.91	3.3
OsR498G0306067400.01	0.446	0.193	-1.3062	0.0001331	0.0002999	0.47	0.44	0.43	0.24	0.15	0.19
OsR498G0306067900.01	0.976	0.193	-2.29904	1.98E-12	3.21E-12	0.64	1.08	1.21	0.18	0.18	0.22
OsR498G0306070300.01	0.206	0.886	2.087066	8.81E-09	1.61E-08	0.17	0.17	0.28	1.1	0.83	0.73
OsR498G0306072100.01	2.41	1.176	-1.06064	2.04E-11	3.41E-11	2.52	2.25	2.46	1.08	1.21	1.24
OsR498G0306076100.01	47.703	324.79	2.764517	0	0	45.37	48.88	48.86	306.34	337.63	330.4
OsR498G0306078100.01	0.473	0	-5.96024	2.86E-09	5.13E-09	0.57	0.55	0.3	0	0	0
OsR498G0306080600.01	0.2	0	-4.66068	0.0001179	0.0002647	0.14	0.19	0.27	0	0	0
OsR498G0306085400.01	45.546	124.24	1.456348	0	0	45.22	44.53	46.89	122.85	122.62	127.25
OsR498G0306098600.01	3.55	11.133	1.630558	3.80E-81	2.63E-81	3.9	3.44	3.31	10.85	11.03	11.52
OsR498G0306105000.01	15.166	5.43	-1.47868	3.36E-106	2.00E-106	15.92	15.45	14.13	4.96	5.44	5.89
OsR498G0306105600.01	7.21	20.03	1.47353	9.94E-223	3.78E-223	6.55	7.85	7.23	19.9	19.67	20.52
OsR498G0306108700.01	51.76	25.613	-1.10477	4.79E-275	1.57E-275	50.14	53.57	51.57	25.71	25.41	25.72
OsR498G0306113700.01	3.796	16.84	2.13033	1.41E-188	6.00E-189	3.88	3.57	3.94	16.8	16.64	17.08
OsR498G0306118900.01	1.136	0.16	-2.51824	4.43E-09	8.00E-09	1.48	0.71	1.22	0.09	0.29	0.1
OsR498G0306125100.01	5.413	21.263	1.934051	3.53E-71	2.63E-71	6.6	4.94	4.7	22.55	19.91	21.33
OsR498G0306128200.01	0.996	0.326	-1.62016	6.33E-08	1.20E-07	1.14	0.79	1.06	0.23	0.32	0.43
OsR498G0306129200.01	2.01	0.083	-4.63267	1.52E-24	1.88E-24	2.01	1.62	2.4	0	0.06	0.19
OsR498G0306131300.01	120.51	44.786	-1.41108	0	0	113.96	124.66	122.9	42.37	46.77	45.22
OsR498G0306132000.01	0.703	0.343	-1.06986	8.03E-07	1.60E-06	0.58	0.59	0.94	0.32	0.31	0.4
OsR498G0306140800.01	0.1	0.523	2.361688	2.29E-06	4.65E-06	0.13	0	0.17	0.71	0.36	0.5
OsR498G0306141500.01	0.203	2.56	3.750253	3.86E-31	4.29E-31	0.2	0.13	0.28	2.57	2.49	2.62
OsR498G0306144200.01	39.64	264.37	2.723537	0	0	43.16	38.29	37.47	273.61	257.94	261.57
OsR498G0306152500.01	7.706	17.62	1.183159	3.41E-62	2.73E-62	8.73	7.04	7.35	17.61	17.6	17.65
OsR498G0306154900.01	1.48	0.176	-3.11161	8.81E-05	0.0001962	1	1.07	2.37	0	0.53	0
OsR498G0306157700.01	8.793	23.323	1.403911	1.83E-177	8.09E-178	8.67	8.95	8.76	22.5	24.35	23.12
OsR498G0306158900.01	0	2.55	8.219669	5.96E-32	6.56E-32	0	0	0	3.17	2.36	2.12
OsR498G0306162200.01	22.426	9.403	-1.3529	3.18E-215	1.24E-215	22.26	22.57	22.45	9.19	9.84	9.18
OsR498G0306167000.01	0.35	4.266	3.575813	1.83E-81	1.26E-81	0.42	0.25	0.38	4.3	4.3	4.2
OsR498G0306174200.01	2.56	0.106	-4.5643	2.00E-57	1.67E-57	2.26	2.78	2.64	0.17	0.06	0.09
OsR498G0306174300.01	0.283	0.066	-2.04175	1.99E-08	3.68E-08	0.22	0.31	0.32	0.02	0.07	0.11
OsR498G0306181400.01	15.29	6.683	-1.18508	4.14E-49	3.75E-49	15.87	14.48	15.52	6.98	6.38	6.69
OsR498G0306183600.01	1.91	0.153	-3.64474	2.59E-19	3.55E-19	2.16	1.54	2.03	0.06	0.33	0.07
OsR498G0306186500.01	23.393	11.236	-1.04516	1.30E-65	1.01E-65	24.01	22.86	23.31	9.87	12.31	11.53
OsR498G0306190000.01	16.746	7.43	-1.15392	6.22E-66	4.83E-66	15.48	17.45	17.31	7.13	7.53	7.63
OsR498G0306191200.01	2.546	8.17	1.674996	2.55E-38	2.59E-38	2.59	2.55	2.5	8.25	8.57	7.69
OsR498G0306192500.01	7.48	0.27	-4.71703	6.36E-171	2.87E-171	6.53	8.04	7.87	0.31	0.27	0.23
OsR498G0306193300.01	24.796	10.846	-1.19829	1.01E-66	7.75E-67	21.28	27.36	25.75	10.68	9.69	12.17
OsR498G0306193900.01	18.96	53.906	1.497121	0	0	18.31	19.25	19.32	54.83	54.2	52.69
OsR498G0306200600.01	32.303	3.64	-3.15395	2.83E-201	1.15E-201	35.3	30.98	30.63	3.83	3.57	3.52
OsR498G0306205000.01	0.193	3.22	4.012993	1.49E-58	1.23E-58	0.19	0.19	0.2	3.82	3.1	2.74
OsR498G0306211200.01	2.176	0.09	-4.51141	3.20E-48	2.92E-48	2.49	1.83	2.21	0.21	0.04	0.02
OsR498G0306216600.01	0.44	0.043	-3.31779	3.28E-09	5.90E-09	0.45	0.47	0.4	0.03	0.07	0.03
OsR498G0306216800.01	148.7	67.32	-1.16567	0	0	152.69	150.25	143.2	68.36	65.5	68.1
OsR498G0306224500.01	13.59	40.47	1.569313	3.52E-253	1.22E-253	14.41	13.22	13.14	42.72	39.57	39.12
OsR498G0306232400.01	1.023	0.226	-2.17281	1.25E-10	2.13E-10	1.29	0.83	0.95	0.2	0.17	0.31
OsR498G0306232800.01	17.053	49.233	1.523049	0	0	17.04	17.37	16.75	47.49	51.99	48.22
OsR498G0306238300.01	85.096	27.573	-1.55781	0	0	83.26	85.05	86.98	26.87	29.17	26.68
OsR498G0306241900.01	10.62	2.23	-2.27745	9.07E-187	3.88E-187	11.07	10	10.79	1.85	2.35	2.49
OsR498G0306245800.01	0.216	1.343	2.489067	1.06E-11	1.76E-11	0.18	0.18	0.29	1.55	1	1.48
OsR498G0306249200.01	1.406	0.236	-2.47481	1.40E-12	2.26E-12	1.1	1.23	1.89	0.31	0.16	0.24
OsR498G0306253300.01	250.28	82.023	-1.61607	0	0	258.32	249.5	243	81.02	83.39	81.66
OsR498G0306261700.01	90.546	35.556	-1.3271	6.20E-217	2.40E-217	97.29	85.98	88.37	38.27	32.61	35.79
OsR498G0306262000.01	34.586	1.536	-4.54562	0	0	33.23	35.49	35.04	1.69	1.36	1.56
OsR498G0306263800.01	15.483	5.003	-1.64171	5.46E-42	5.33E-42	15.05	15.64	15.76	5.68	4.68	4.65
OsR498G0306264100.01	7.14	0.406	-4.18557	5.52E-46	5.16E-46	7.12	7.18	7.12	0.11	0.67	0.44
OsR498G0306269500.01	48.626	121.37	1.323239	0	0	48.74	48.81	48.33	119.52	123.13	121.45
OsR498G0306276700.01	0.833	1.75	1.028789	5.87E-07	1.16E-06	0.98	0.76	0.76	1.45	1.8	2
OsR498G0306282800.01	3.35	9.926	1.555775	1.45E-12	2.35E-12	2.17	5.02	2.86	10.16	8.97	10.65
OsR498G0306285400.01	0.056	20.396	8.564628	3.06E-222	1.17E-222	0	0.17	0	19.42	20.21	21.56
OsR498G0306291900.01	0.03	1.726	5.807944	6.45E-28	7.52E-28	0	0	0.09	1.87	1.74	1.57

OsR498G0306294300.01	1.226	0.363	-1.78336	7.37E-06	1.54E-05	1.12	1.01	1.55	0.41	0.68	0
OsR498G0306295300.01	171.65	63.656	-1.41699	0	0	177.96	171.77	165.2	68.52	60.68	61.77
OsR498G0306297500.01	210.93	551.46	1.379729	0	0	218.99	206.8	207	561.86	546.66	545.85
OsR498G0306305300.01	0.51	0.06	-2.78058	6.64E-07	1.32E-06	0.62	0.66	0.25	0	0.04	0.14
OsR498G0306321700.01	5.863	19.98	1.722853	3.10E-141	1.57E-141	5.44	5.97	6.18	19.49	19.99	20.46
OsR498G0306323800.01	0	0.796	6.624723	4.17E-13	6.62E-13	0	0	0	0.88	0.61	0.9
OsR498G0306333700.01	7.16	2.506	-1.67637	2.92E-68	2.22E-68	7.5	7.15	6.83	2.76	2.39	2.37
OsR498G0306338700.01	54.083	206.34	1.913892	0	0	51.46	55.69	55.1	200.66	210.04	208.33
OsR498G0306340300.01	0.103	0	-4.5452	0.0002138	0.0004889	0.18	0.03	0.1	0	0	0
OsR498G0306350200.01	8.44	3.273	-1.40081	1.53E-25	1.85E-25	9.67	7.58	8.07	3.05	3.01	3.76
OsR498G0306355200.01	26.883	7.06	-1.91927	2.15E-255	7.46E-256	28.04	27.75	24.86	6	8.28	6.9
OsR498G0306355400.01	3.286	0.753	-2.26594	2.40E-82	1.64E-82	2.67	3.63	3.56	0.53	0.66	1.07
OsR498G0306367200.01	5.6	2.52	-1.07508	1.93E-17	2.75E-17	5.68	6.07	5.05	2.3	2.56	2.7
OsR498G0306377200.01	11.973	5.883	-1.07694	7.96E-121	4.42E-121	12.31	12.15	11.46	5.92	5.84	5.89
OsR498G0306381500.01	0.116	0.796	2.702725	0.0001213	0.0002726	0.12	0	0.23	0.62	0.76	1.01
OsR498G0306382200.01	69.213	16.98	-2.01784	0	0	69.01	68.76	69.87	16.58	17.78	16.58
OsR498G0306387300.01	6.466	14.83	1.151653	2.57E-30	2.88E-30	6.02	6.14	7.24	15.26	15.16	14.07
OsR498G0306392300.01	409.12	144.98	-1.49623	0	0	424.14	401.75	401.5	146.09	142.71	146.14
OsR498G0306399600.01	11.116	62.32	2.470258	0	0	11.74	9.76	11.85	63.53	61.3	62.13
OsR498G0306402100.01	0.8	0.373	-1.10567	6.63E-05	0.0001466	0.82	0.82	0.76	0.3	0.38	0.44
OsR498G0306408900.01	5.096	0.53	-3.27707	2.94E-152	1.42E-152	5.37	4.64	5.28	0.46	0.66	0.47
OsR498G0306409400.01	11.256	5.36	-1.06958	8.09E-45	7.67E-45	11.83	11.45	10.49	5.67	5.17	5.24
OsR498G0306414400.01	16.07	5.68	-1.53482	0	0	15.66	16.59	15.96	5.07	6.29	5.68
OsR498G0306414800.01	9.883	1.336	-2.88682	5.25E-270	1.74E-270	8.16	11.31	10.18	1.42	1.57	1.02
OsR498G0306415800.01	25.553	71.156	1.469081	0	0	25.02	25.59	26.05	74.26	67.12	72.09
OsR498G0306419400.01	0	0.23	4.624723	0.0001711	0.0003885	0	0	0	0.28	0.35	0.06
OsR498G0306427000.01	50.31	277.48	2.459177	0	0	49.03	50.54	51.36	272.74	282.13	277.58
OsR498G0306428400.01	8.73	44.893	2.357863	0	0	9.32	8.12	8.75	44.17	45.35	45.16
OsR498G0306431400.01	0.58	0.22	-1.44137	3.74E-06	7.68E-06	0.6	0.46	0.68	0.32	0.24	0.1
OsR498G0306433000.01	6.466	0.46	-3.81822	1.70E-42	1.65E-42	6.59	6.66	6.15	0.48	0.55	0.35
OsR498G0306435600.01	7.133	2.34	-1.60757	2.29E-61	1.84E-61	6.43	7.2	7.77	2.12	2.07	2.83
OsR498G0306438900.01	0.633	0.013	-5.4838	1.58E-12	2.56E-12	0.33	0.82	0.75	0.04	0	0
OsR498G0306443900.01	1.48	0.326	-2.0605	3.28E-32	3.59E-32	1.45	1.53	1.46	0.54	0.23	0.21
OsR498G0306444400.01	0.676	0.026	-4.6041	6.01E-13	9.58E-13	0.76	0.8	0.47	0.04	0	0.04
OsR498G0306449100.01	2.86	1.156	-1.30904	1.31E-18	1.82E-18	3.12	3.03	2.43	0.99	1.23	1.25
OsR498G0306453400.01	20.546	68.84	1.750438	0	0	19.3	21.44	20.9	65.65	67.72	73.15
OsR498G0306461200.01	16.276	73.796	1.863786	0	0	15.48	15.76	17.59	68.1	77.73	75.56
OsR498G0306462000.01	4.626	2.266	-1.03003	1.63E-22	2.09E-22	5.17	4.69	4.02	1.88	2.48	2.44
OsR498G0306462500.01	0.103	0.906	3.134917	2.11E-18	2.95E-18	0.11	0.09	0.11	0.88	0.94	0.9
OsR498G0306466800.01	0.556	0.22	-1.3865	0.0002186	0.0005004	0.64	0.53	0.5	0.19	0.28	0.19
OsR498G0306466900.01	225.04	26.363	-3.0857	0	0	231.29	224.84	219	26.5	26.79	25.8
OsR498G0306484100.01	0.15	0	-5.66068	7.26E-08	1.38E-07	0.19	0.11	0.15	0	0	0
OsR498G0306485300.01	2373.7	300.08	-2.99008	0	0	2376	2364.9	2380	314.95	295.96	289.32
OsR498G0306488000.01	10.57	2.86	-1.90188	1.91E-69	1.44E-69	9.26	10.08	12.37	2.4	3.54	2.64
OsR498G0306490700.01	1.006	12.666	3.647345	5.76E-283	1.85E-283	1.09	0.98	0.95	13.6	12.47	11.93
OsR498G0306491600.01	1.67	0.646	-1.37809	3.00E-12	4.89E-12	1.61	1.62	1.78	0.68	0.62	0.64
OsR498G0306492900.01	50.446	20.436	-1.30841	1.40E-301	4.28E-302	51.24	49.04	51.06	20.7	20.86	19.75
OsR498G0306493800.01	220.28	73.206	-1.60281	0	0	224.06	220.69	216.1	73.59	68.94	77.09
OsR498G0306499400.01	10.843	5.14	-1.26231	1.32E-135	6.84E-136	10.62	10.99	10.92	5.55	4.83	5.04
OsR498G0306500500.01	0.706	0.313	-1.18263	8.85E-05	0.0001971	0.8	0.74	0.58	0.34	0.32	0.28
OsR498G0306505500.01	1.06	0.493	-1.10834	2.57E-05	5.53E-05	0.93	1.09	1.16	0.47	0.53	0.48
OsR498G0306508200.01	70.163	234.53	1.709797	0	0	69.23	70.16	71.1	243.96	229.18	230.44
OsR498G0306510800.01	2.916	1.74	-1.01538	7.78E-14	1.21E-13	2.93	2.7	3.12	1.99	1.64	1.59
OsR498G0306516100.01	3.743	29.173	3.017808	0	0	3.91	3.6	3.72	28.95	29.09	29.48
OsR498G0306516400.01	1.28	3.16	1.598346	9.54E-13	1.53E-12	0.99	1.5	1.35	3.56	2.78	3.14
OsR498G0306516900.01	6.596	2.19	-1.62571	5.43E-69	4.11E-69	6.32	6.86	6.61	2.4	1.84	2.33
OsR498G0306524700.01	0.1	3.2	5.021613	6.38E-39	6.43E-39	0.1	0.14	0.06	3.85	3.15	2.6
OsR498G0306526900.01	4.9	2.276	-1.10788	4.12E-35	4.35E-35	4.88	4.95	4.87	2.6	2.38	1.85
OsR498G0306531800.01	2.23	0.42	-2.41341	7.65E-17	1.11E-16	2.27	1.95	2.47	0.47	0.55	0.24
OsR498G0306534800.01	0.29	0.656	1.246211	5.62E-05	0.0001236	0.36	0.23	0.28	0.61	0.8	0.56
OsR498G0306540800.01	2.186	0.703	-1.71853	3.19E-15	4.81E-15	2.39	1.9	2.27	0.46	0.62	1.03
OsR498G0306542700.01	9.486	3.883	-1.21668	2.27E-43	2.18E-43	9.57	9.09	9.8	3.96	4.1	3.59
OsR498G0306544400.01	8.463	1.056	-3.01087	3.50E-78	2.47E-78	8.78	8.13	8.48	1	1.09	1.08
OsR498G0306547800.01	0.37	7.18	4.220102	6.21E-93	3.98E-93	0.29	0.21	0.61	7.62	6.89	7.03
OsR498G0306549300.01	4.173	1.91	-1.05848	1.21E-14	1.85E-14	4.89	3.68	3.95	2.29	2.03	1.41
OsR498G0306549800.01	6.69	2.213	-1.60056	3.35E-48	3.06E-48	6.21	6.69	7.17	2.39	2.24	2.01
OsR498G0306554100.01	0.28	2.94	3.354938	3.34E-12	5.45E-12	0.84	0	0	2.71	2.68	3.43
OsR498G0306562700.01	7.24	2.903	-1.30789	9.14E-56	7.74E-56	6.99	6.7	8.03	2.53	2.93	3.25
OsR498G0306563100.01	3.286	0.32	-3.38427	2.16E-119	1.21E-119	3.2	3.53	3.13	0.25	0.37	0.34

OsR498G0306563600.01	0.256	0.516	1.005813	6.01E-05	0.0001324	0.34	0.19	0.24	0.65	0.4	0.5
OsR498G0306566200.01	0.456	0.03	-4.1032	5.78E-13	9.22E-13	0.34	0.38	0.65	0	0.04	0.05
OsR498G0306568000.01	0.313	0.06	-2.46274	0.0005334	0.0012561	0.39	0.23	0.32	0.06	0	0.12
OsR498G0306568200.01	16.053	3.466	-2.20839	1.84E-195	7.65E-196	16.14	16.29	15.73	3.44	3.41	3.55
OsR498G0306580500.01	2.533	7.18	1.478502	1.70E-17	2.42E-17	2.29	2.03	3.28	6.23	6	9.31
OsR498G0306580700.01	2.913	6.34	1.11171	1.30E-26	1.55E-26	3.21	2.53	3	6.25	6.96	5.81
OsR498G0306586700.01	0.846	0.13	-2.69721	1.19E-13	1.85E-13	0.76	0.88	0.9	0.13	0.13	0.13
OsR498G0306595700.01	10.79	114.44	3.41602	0	0	10.94	11.06	10.37	111.27	113.44	118.62
OsR498G0306597000.01	4.04	36.04	3.159409	0	0	3.91	4.57	3.64	38.02	35.21	34.89
OsR498G0306604300.01	2.123	0.416	-2.53918	7.88E-35	8.34E-35	1.97	2.14	2.26	0.39	0.5	0.36
OsR498G0306611700.01	0.413	0.036	-3.4838	4.22E-06	8.70E-06	0.32	0.4	0.52	0.05	0	0.06
OsR498G0306612000.01	9.273	1.306	-2.84432	6.57E-119	3.68E-119	9.57	9.42	8.83	1.63	1.03	1.26
OsR498G0306612400.01	25.206	7.77	-1.70614	1.69E-91	1.09E-91	26.65	26.32	22.65	7.62	8.21	7.48
OsR498G0306624300.01	0.17	0.01	-4.08952	3.72E-09	6.71E-09	0.13	0.24	0.14	0	0	0.03
OsR498G0306624800.01	1.726	0.15	-3.5452	3.73E-104	2.25E-104	1.56	1.84	1.78	0.12	0.1	0.23
OsR498G0306626300.01	21.89	97.67	2.128391	0	0	22.38	21.11	22.18	96.42	99.49	97.1
OsR498G0306636000.01	11.526	31.42	1.445069	2.46E-150	1.20E-150	11.58	11.51	11.49	34.06	28.83	31.37
OsR498G0306637600.01	1.32	0.196	-2.78806	1.08E-12	1.74E-12	1.41	1.36	1.19	0.06	0.18	0.35
OsR498G0306643800.01	0.393	6.146	3.963649	1.28E-145	6.33E-146	0.37	0.41	0.4	5.98	6.58	5.88
OsR498G0306644400.01	2.81	0.583	-2.26868	2.40E-53	2.08E-53	2.65	2.65	3.13	0.66	0.64	0.45
OsR498G0306644700.01	1.81	0.166	-3.4838	4.22E-06	8.70E-06	0.93	2.71	1.79	0	0.25	0.25
OsR498G0306649900.01	2.073	0.953	-1.16333	2.03E-06	4.11E-06	1.79	2.51	1.92	0.3	1.72	0.84
OsR498G0306653600.01	15.326	33.133	1.093344	7.62E-141	3.87E-141	15.67	15.37	14.94	32.31	32.96	34.13
OsR498G0306653900.01	12.826	37.043	1.536461	4.30E-290	1.36E-290	12.72	12.45	13.31	35.68	38.26	37.19
OsR498G0306662200.01	0.1	2.05	4.355115	1.62E-50	1.44E-50	0.11	0.11	0.08	2.15	2.14	1.86
OsR498G0306662300.01	0.196	4.86	4.578919	5.05E-23	6.41E-23	0.45	0	0.14	4.51	5.2	4.87
OsR498G0306664100.01	0.81	0.063	-3.89884	1.49E-20	1.99E-20	0.99	0.68	0.76	0.1	0.09	0
OsR498G0306664800.01	0.533	0.053	-3.35256	1.36E-05	2.87E-05	0.68	0.64	0.28	0	0.16	0
OsR498G0306667400.01	0.56	1.276	1.175275	6.67E-09	1.21E-08	0.64	0.4	0.64	1.4	1.37	1.06
OsR498G0306667600.01	12.846	5.5	-1.23122	7.76E-80	5.41E-80	12.18	12.92	13.44	5.36	5.14	6
OsR498G0306669200.01	3.82	1.213	-1.65768	5.00E-21	6.61E-21	3.99	4.03	3.44	1.11	1	1.53
OsR498G0306670200.01	4.45	8.953	1.009528	3.10E-34	3.31E-34	4.54	4.29	4.52	8.48	9.36	9.02
OsR498G0306672200.01	47.723	113.68	1.247949	0	0	47.16	47.51	48.5	114.34	112.75	113.96
OsR498G0306675300.01	14.74	4.746	-1.75523	4.26E-77	3.02E-77	14.91	14.63	14.68	5.18	3.98	5.08
OsR498G0306679000.01	60.45	23.646	-1.35821	0	0	63.98	57.56	59.81	22.81	23.6	24.53
OsR498G0306680500.01	4.1	10.256	1.320072	9.47E-61	7.67E-61	4.22	4.17	3.91	10.51	9.93	10.33
OsR498G0306684400.01	37.25	5.266	-2.84168	1.89E-141	9.54E-142	38.79	37.28	35.68	4.48	5.61	5.71
OsR498G0306693800.01	5.74	17.466	1.635898	5.06E-158	2.38E-158	5.63	6.09	5.5	17.27	18.35	16.78
OsR498G0306694200.01	0.05	3.723	6.155237	2.52E-51	2.23E-51	0	0.15	0	3.54	4.48	3.15
OsR498G0306694400.01	11.263	121.56	3.419299	0	0	11.28	11.34	11.17	120.24	123.47	120.96
OsR498G0306694600.01	0	4.306	9.470213	2.11E-63	1.67E-63	0	0	0	4.26	4.53	4.13
OsR498G0306696800.01	15.473	34.4	1.145768	9.79E-105	5.88E-105	14.62	16.67	15.13	33.15	35.59	34.46
OsR498G0306696900.01	4.44	10.036	1.17193	1.10E-30	1.23E-30	4.2	4.72	4.4	10.44	10.9	8.77
OsR498G0306701000.01	1.993	0.25	-2.98772	3.32E-19	4.56E-19	2.23	2.4	1.35	0.17	0.29	0.29
OsR498G0306702100.01	1.86	4.01	1.111313	5.67E-24	7.09E-24	1.68	1.95	1.95	4.38	4.02	3.63
OsR498G0306704600.01	0.12	0.76	2.699584	3.40E-20	4.57E-20	0.02	0.14	0.2	0.95	0.69	0.64
OsR498G0306705400.01	1.306	0.146	-3.15006	2.08E-14	3.19E-14	1.33	1.56	1.03	0.22	0.22	0
OsR498G0306714800.01	10.61	38.846	1.882178	0	0	10.5	10.11	11.22	38.44	37.39	40.71
OsR498G0306718600.01	8.556	2.166	-1.99838	1.42E-25	1.72E-25	6.76	9.92	8.99	1.49	1.87	3.14
OsR498G0306721300.01	0	3.583	6.531613	1.92E-12	3.11E-12	0	0	0	4.29	2.63	3.83
OsR498G0306727100.01	2.223	12.516	2.49219	4.75E-130	2.52E-130	2	2.59	2.08	13.68	11.84	12.03
OsR498G0306728600.01	0.093	2.54	5.325162	6.68E-39	6.74E-39	0	0.1	0.18	3.27	1.99	2.36
OsR498G0306730500.01	6.63	3.323	-1.00792	2.77E-30	3.12E-30	6.45	7.03	6.41	3.05	3.77	3.15
OsR498G0306732000.01	0.793	3.353	2.061455	1.19E-16	1.73E-16	0.51	0.68	1.19	3.7	3.12	3.24
OsR498G0306732200.01	3.29	0.663	-2.32143	2.11E-20	2.83E-20	3.23	3.33	3.31	0.47	0.97	0.55
OsR498G0306735500.01	7.693	1.006	-2.87431	7.19E-176	3.19E-176	7.53	8.1	7.45	1.11	0.92	0.99
OsR498G0306738300.01	0.376	6.013	3.978359	7.54E-70	5.68E-70	0.54	0.28	0.31	5.72	6.19	6.13
OsR498G0306740400.01	1.716	0.06	-4.83798	4.77E-35	5.05E-35	1.54	1.75	1.86	0.07	0	0.11
OsR498G0306744000.01	1.323	0.496	-1.5325	2.67E-13	4.21E-13	1.54	1.12	1.31	0.45	0.54	0.5
OsR498G0306744300.01	4.106	1.746	-1.23033	2.61E-12	4.25E-12	4.53	3.62	4.17	1.73	1.84	1.67
OsR498G0306747400.01	5.3	1.56	-1.76416	2.36E-38	2.40E-38	5.97	4.7	5.23	1.97	1.48	1.23
OsR498G0306747800.01	9.106	2.156	-2.17025	2.09E-112	1.21E-112	9.18	8.57	9.57	2.24	2.02	2.21
OsR498G0306751800.01	0.28	0	-5.27639	2.14E-06	4.34E-06	0.33	0.35	0.16	0	0	0
OsR498G0306756300.01	6.063	1.736	-1.80481	1.42E-107	8.43E-108	5.74	6.28	6.17	1.95	1.68	1.58
OsR498G0306756900.01	3.966	0.95	-1.63831	1.73E-29	1.96E-29	3.84	4.14	3.92	1.4	0.78	0.67
OsR498G0306760300.01	1.356	0.95	-1.1032	0.0005273	0.001241	1.23	1.47	1.37	0.96	0.53	1.36
OsR498G0306761200.01	0.373	1.496	2.098654	2.60E-10	4.51E-10	0.23	0.5	0.39	1.71	1.88	0.9
OsR498G0306769300.01	0.97	0.116	-3.20817	4.33E-05	9.45E-05	1.43	0.94	0.54	0.23	0.12	0
OsR498G0306771400.01	3.326	1.326	-1.32702	4.21E-13	6.68E-13	3.06	3.65	3.27	1.34	1.22	1.42

OsR498G0306772900.01	2.103	63.736	4.911073	0	0	2.52	2.28	1.51	72.48	59.58	59.15
OsR498G0306774000.01	11.026	5.746	-1.01007	6.80E-50	6.11E-50	10.42	11.67	10.99	6.26	5.02	5.96
OsR498G0306780100.01	11.21	4.916	-1.2065	6.14E-18	8.67E-18	11.58	12.74	9.31	5.39	3.88	5.48
OsR498G0306781300.01	4.363	1.816	-1.27455	4.85E-12	7.96E-12	4.29	3.85	4.95	2.05	1.09	2.31
OsR498G0306781800.01	0.063	1.743	4.76768	4.17E-14	6.46E-14	0.19	0	0	1.95	1.5	1.78
OsR498G0306787500.01	2.683	0.773	-1.80198	1.26E-26	1.50E-26	2.53	2.62	2.9	0.71	0.64	0.97
OsR498G0306794200.01	0	0.253	5.361688	1.47E-06	2.96E-06	0	0	0	0.18	0.54	0.04
OsR498G0306796000.01	5.81	1.58	-1.75936	1.72E-37	1.76E-37	5.95	6.17	5.31	1.5	1.46	1.78
OsR498G0306797900.01	13.616	4.516	-1.8225	1.74E-201	7.09E-202	12.59	14.96	13.3	4.02	4.51	5.02
OsR498G0306799900.01	6.263	2.156	-1.58055	4.40E-51	3.90E-51	6.46	6.29	6.04	2.31	2.07	2.09
OsR498G0306806900.01	4.786	59.853	3.642128	0	0	3.98	5.34	5.04	64.38	58.95	56.23
OsR498G0306808200.01	0	0.226	6.325162	4.31E-11	7.27E-11	0	0	0	0.1	0.34	0.24
OsR498G0306813500.01	2.653	1.18	-1.16669	1.93E-07	3.72E-07	2.38	2.94	2.64	1.22	1.07	1.25
OsR498G0306814500.01	2.116	4.173	1.001618	9.58E-31	1.07E-30	1.84	2.42	2.09	4.22	4.23	4.07
OsR498G0306815000.01	268.24	2.08	-7.01439	0	0	279.64	264.64	260.4	2.2	1.99	2.05
OsR498G0306815100.01	15.703	2.356	-2.42769	3.87E-87	2.57E-87	15.85	15.32	15.94	2.29	2.35	2.43
OsR498G0306815500.01	87.936	30.963	-1.51364	0	0	92.94	86.74	84.13	31.66	30.26	30.97
OsR498G0306817500.01	6.83	26.7	1.960326	7.72E-155	3.69E-155	6.68	7.06	6.75	27.63	28.31	24.16
OsR498G0306818600.01	138.87	370.2	1.407038	0	0	136.93	145.64	134.1	395.64	357.71	357.26
OsR498G0306819800.01	19.506	55.2	1.497324	0	0	18.8	19.9	19.82	52.65	56.25	56.7
OsR498G0306820600.01	18.18	2.53	-2.84308	4.32E-145	2.15E-145	16.71	18.95	18.88	2.15	2.58	2.86
OsR498G0306820700.01	5.19	1.42	-1.88509	1.41E-50	1.25E-50	5.26	4.83	5.48	1.27	1.23	1.76
OsR498G0306821800.01	13.123	4.48	-1.55639	6.95E-115	3.97E-115	12.46	13.41	13.5	4.7	4.28	4.46
OsR498G0306824200.01	15.9	7.963	-1.00328	1.80E-55	1.53E-55	17.24	15.68	14.78	8.3	8.03	7.56
OsR498G0306827000.01	0.23	4.356	4.259809	6.63E-47	6.13E-47	0.25	0.26	0.18	5.08	5.11	2.88
OsR498G0306828300.01	0.546	1.556	1.487219	0.0007914	0.0018903	0.15	0.63	0.86	1.39	1.41	1.87
OsR498G0306829300.01	3.993	9.38	1.228301	7.50E-57	6.30E-57	3.91	3.82	4.25	10.41	8.81	8.92
OsR498G0306833100.01	18.026	3.04	-2.57806	1.32E-107	7.81E-108	19.6	17.75	16.73	2.99	3.3	2.83
OsR498G0306837400.01	0.76	0.346	-1.14466	0.000524	0.0012329	0.55	0.67	1.06	0.42	0.29	0.33
OsR498G0306839100.01	14.176	4.19	-1.73822	3.63E-36	3.78E-36	13.27	14.37	14.89	3.17	4.17	5.23
OsR498G0306845300.01	1.656	10.223	2.62087	4.60E-221	1.76E-221	1.57	1.54	1.86	10.08	10.59	10
OsR498G0306848200.01	0.786	0.076	-3.46274	7.09E-21	9.41E-21	0.78	0.79	0.79	0.1	0.08	0.05
OsR498G0306850400.01	17.406	142.78	3.029863	0	0	18.4	16.98	16.84	138.95	144.38	145
OsR498G0306851400.01	0.026	0.663	4.664251	7.91E-19	1.10E-18	0.05	0	0.03	0.66	0.68	0.65
OsR498G0306851600.01	10.69	61.48	2.518166	0	0	11.95	10.04	10.08	64.4	60.39	59.65
OsR498G0306853200.01	0	3.216	8.315884	1.06E-33	1.14E-33	0	0	0	3.74	2.72	3.19
OsR498G0306854200.01	0	0.24	4.269348	0.0008659	0.0020732	0	0	0	0.08	0.4	0.24
OsR498G0306854600.01	4.59	68.03	3.88283	0	0	4.27	4.83	4.67	70.19	67.72	66.18
OsR498G0306856500.01	2.083	0.763	-1.44567	2.25E-06	4.58E-06	2.61	2.11	1.53	0.87	0.38	1.04
OsR498G0306859100.01	39.763	17.773	-1.18147	2.85E-160	1.33E-160	40.95	38.49	39.85	17.6	18.43	17.29
OsR498G0306873300.01	0.566	3.233	2.524595	2.16E-70	1.62E-70	0.57	0.49	0.64	3.61	2.89	3.2
OsR498G0306875300.01	2.016	12.096	2.576523	6.99E-294	2.18E-294	1.5	2.44	2.11	12.05	12.44	11.8
OsR498G0306875700.01	16.973	7.866	-1.16498	6.64E-204	2.68E-204	15.65	17.68	17.59	7.72	8.3	7.58
OsR498G0306876900.01	0.99	9.396	3.209685	9.58E-107	5.69E-107	0.82	1	1.15	9.94	9.11	9.14
OsR498G0306880300.01	46.95	20.05	-1.24054	0	0	46.41	46.66	47.78	18.52	20.62	21.01
OsR498G0306884400.01	6.463	1.04	-2.68313	2.43E-101	1.49E-101	6.32	6.68	6.39	0.76	1.04	1.32
OsR498G0306884800.01	0.233	0.676	1.499192	1.76E-06	3.56E-06	0.18	0.27	0.25	0.81	0.69	0.53
OsR498G0306887100.01	8.366	17.58	1.094017	1.97E-52	1.73E-52	9.18	8.37	7.55	18.44	15.78	18.52
OsR498G0306887800.01	5.38	2.01	-1.43478	5.85E-36	6.11E-36	5.38	6.01	4.75	1.65	2.19	2.19
OsR498G0306900900.01	20.236	7.853	-1.30088	2.80E-140	1.43E-140	22.03	19.25	19.43	8.88	7.79	6.89
OsR498G0306903500.01	0.02	0.183	3.847115	0.0001179	0.0002648	0	0	0.06	0.27	0.24	0.04
OsR498G0306904700.01	107.23	323.75	1.588422	0	0	112.57	103.32	105.8	317.56	324.59	329.11
OsR498G0306907900.01	0.316	0.053	-2.52095	6.43E-10	1.13E-09	0.26	0.29	0.4	0.03	0.05	0.08
OsR498G0306908200.01	3.993	0.72	-2.47626	4.75E-21	6.28E-21	3.99	4.46	3.53	0.85	0.7	0.61
OsR498G0306910500.01	4.45	14.25	1.672276	3.82E-50	3.42E-50	4.07	4.72	4.56	13.14	15.69	13.92
OsR498G0306910800.01	3.703	9.293	1.101096	2.24E-60	1.82E-60	4.15	2.9	4.06	9.26	9.09	9.53
OsR498G0306911600.01	3.87	105.16	4.774907	0	0	4.05	3.53	4.03	104.75	105.88	104.85
OsR498G0306913700.01	105.19	31.906	-1.72397	0	0	110.5	101.92	103.2	34.46	29.61	31.65
OsR498G0306917500.01	0.163	1.436	3.132869	2.30E-24	2.85E-24	0.19	0.24	0.06	1.58	1.26	1.47
OsR498G0306918600.01	1.796	3.916	1.129515	1.12E-30	1.25E-30	1.61	1.74	2.04	4.32	3.72	3.71
OsR498G0306922400.01	0.646	0.086	-2.94521	0.0002294	0.0005261	0.74	0.75	0.45	0	0.26	0
OsR498G0306924000.01	0.583	6.983	3.544104	1.75E-103	1.06E-103	0.84	0.32	0.59	7.31	6.66	6.98
OsR498G0306931400.01	0.176	0.023	-3.01913	6.39E-06	1.33E-05	0.13	0.11	0.29	0.07	0	0
OsR498G0306934600.01	0.753	0.133	-2.50317	7.73E-21	1.03E-20	0.5	0.81	0.95	0.1	0.1	0.2
OsR498G0306938500.01	50.3	103.88	1.03976	0	0	50.03	51.89	48.98	103.15	103.6	104.89
OsR498G0306942100.01	18.096	1.696	-3.42179	1.24E-127	6.63E-128	19.68	18.2	16.41	2.35	1.75	0.99
OsR498G0306942400.01	2.203	0.13	-4.18631	2.52E-57	2.11E-57	1.84	2.09	2.68	0.1	0.13	0.16
OsR498G0306945600.01	15.276	69.036	2.197762	0	0	15.43	15.02	15.38	67.58	68.06	71.47
OsR498G0306949100.01	0.29	0	-4.56312	0.0001955	0.0004458	0.87	0	0	0	0	0

OsR498G0306951500.01	0.82	0	-7.35924	2.83E-20	3.80E-20	1.94	0	0.52	0	0	0
OsR498G0306951900.01	1.426	0.643	-1.17491	1.14E-05	2.40E-05	1.65	1.26	1.37	0.87	0.57	0.49
OsR498G0306954600.01	0.576	0.06	-3.21897	1.34E-13	2.10E-13	0.41	0.67	0.65	0.1	0.03	0.05
OsR498G0306954800.01	10.623	32.78	1.617129	1.37E-151	6.64E-152	9.52	10.46	11.89	36.04	31.61	30.69
OsR498G0306956600.01	9.533	1.536	-2.64931	1.63E-47	1.50E-47	8.85	8.84	10.91	1.67	1.62	1.32
OsR498G0306956900.01	0.673	0.253	-1.53282	4.12E-10	7.20E-10	0.56	0.74	0.72	0.19	0.31	0.26
OsR498G0306957100.01	0.113	0	-4.41967	0.0003895	0.0009082	0.06	0.16	0.12	0	0	0
OsR498G0306960100.01	0.093	2.583	4.946651	6.64E-30	7.51E-30	0.19	0.09	0	2.86	2.75	2.14
OsR498G0306960300.01	3.923	1.35	-1.54161	1.12E-30	1.25E-30	3.95	3.95	3.87	1.48	1.2	1.37
OsR498G0306960600.01	0.65	9.34	3.830752	3.56E-110	2.07E-110	0.51	0.75	0.69	10.11	9.14	8.77
OsR498G0306963400.01	1.26	11.386	3.171039	0	0	1.32	1.13	1.33	11	11.66	11.5
OsR498G0306965200.01	5.756	18.75	1.694345	1.71E-57	1.43E-57	6.32	5.87	5.08	19.47	17.82	18.96
OsR498G0306965500.01	5.33	13.353	1.31779	4.37E-48	4.00E-48	5.29	5.48	5.22	13.07	13.31	13.68
OsR498G0306971600.01	3.363	10.213	1.271989	7.67E-27	9.11E-27	3.33	4.01	2.75	11.19	9.02	10.43
OsR498G0306980400.01	7.013	0.333	-4.30404	1.06E-158	4.96E-159	7.05	6.53	7.46	0.43	0.34	0.23
OsR498G0306982000.01	97.716	23.903	-2.0151	0	0	98.38	97.36	97.41	23.09	24.43	24.19
OsR498G0306987700.01	8.986	4.47	-1.02575	2.02E-34	2.15E-34	9.14	8.48	9.34	4.07	4.78	4.56
OsR498G0306988300.01	0.123	0.02	-3.41967	0.0009442	0.002269	0.15	0.14	0.08	0	0	0.06
OsR498G0306990100.01	110.24	35.046	-1.6662	0	0	113.34	110.5	106.9	32.3	36.18	36.66
OsR498G0306991800.01	0.31	0.083	-1.89884	0.0006013	0.0014228	0.32	0.26	0.35	0.11	0.07	0.07
OsR498G0306994100.01	8.706	2.056	-2.07656	5.11E-186	2.19E-186	8.01	8.74	9.37	2.4	2.11	1.66
OsR498G0306995200.01	10.25	4.723	-1.13378	2.68E-15	4.02E-15	10.11	9.79	10.85	5.37	5.06	3.74
OsR498G0306995300.01	0	0.5	5.361688	1.47E-06	2.96E-06	0	0	0	0.14	0.43	0.93
OsR498G0307000500.01	0.033	17.6	9.031282	6.08E-98	3.78E-98	0	0	0.1	17.92	16.77	18.11
OsR498G0307000700.01	0.153	2.48	3.993956	5.24E-15	7.93E-15	0.34	0.12	0	2.13	2.3	3.01
OsR498G0307001100.01	0.9	10.99	3.608044	8.78E-70	6.61E-70	0.55	1.26	0.89	10.49	9.88	12.6
OsR498G0307001600.01	0.213	24.236	7.035245	9.07E-148	4.46E-148	0.43	0.11	0.1	29.28	23.26	20.17
OsR498G0307006100.01	0.91	0.243	-1.92371	1.75E-09	3.11E-09	0.98	0.84	0.91	0.18	0.26	0.29
OsR498G0307006400.01	6.22	2.513	-1.34011	2.25E-51	1.98E-51	5.91	6.26	6.49	2.47	2.59	2.48
OsR498G0307007000.01	28.676	8.256	-1.91735	2.39E-237	8.69E-238	28.77	29.12	28.14	8.54	7.77	8.46
OsR498G0307007700.01	43.233	15.223	-1.51686	6.59E-146	3.27E-146	44.88	39.67	45.15	16.62	14.42	14.63
OsR498G0307008000.01	47.85	18.9	-1.34343	0	0	52.31	44.98	46.26	20.42	18.35	17.93
OsR498G0307008400.01	72.256	7.093	-3.39942	0	0	71.21	71.83	73.73	6.17	7.82	7.29
OsR498G0307008800.01	2.39	18.733	2.965387	6.42E-131	3.40E-131	3.07	2.42	1.68	19.56	18.37	18.27
OsR498G0307010000.01	6.746	15.273	1.176365	1.82E-64	1.43E-64	6.89	7.05	6.3	15.4	15.01	15.41
OsR498G0307011100.01	4.133	1.603	-1.37791	4.15E-24	5.17E-24	4.24	4.03	4.13	1.44	1.36	2.01
OsR498G0307011500.01	2199.9	907.39	-1.28518	0	0	2230.3	2180.9	2188	897.05	914.49	910.63
OsR498G0307016500.01	2.256	72.78	5.005467	0	0	2.19	2.18	2.4	74.92	70.52	72.9
OsR498G0307019700.01	2.883	7.22	1.299254	4.28E-25	5.24E-25	3.09	2.98	2.58	6.4	7.61	7.65
OsR498G0307020100.01	4.55	77.26	3.706517	0	0	5.15	4.1	4.4	74.87	80.88	76.03
OsR498G0307023100.01	14.1	69.993	2.299482	0	0	16.32	11.76	14.22	73.9	69.83	66.25
OsR498G0307026600.01	19.026	1.916	-3.31372	5.96E-231	2.22E-231	19.87	18.77	18.44	2.2	2.01	1.54
OsR498G0307028900.01	3.02	0.76	-1.95552	6.74E-63	5.36E-63	3.16	2.72	3.18	0.86	0.73	0.69
OsR498G0307029700.01	24.803	102.29	2.030053	0	0	26.45	25.68	22.28	98.67	103.34	104.85
OsR498G0307030400.01	97.606	226.91	1.208989	0	0	99.91	94.07	98.84	232.18	225.93	222.62
OsR498G0307032000.01	88.756	43.373	-1.03793	1.51E-222	5.76E-223	90.29	87.44	88.54	48.18	42.54	39.4
OsR498G0307036700.01	4.81	9.893	1.048786	2.22E-21	2.91E-21	4.42	4.68	5.33	9.57	10.06	10.05
OsR498G0307037100.01	0.903	15.703	4.110523	3.83E-168	1.74E-168	1.17	0.92	0.62	16.73	14.53	15.85
OsR498G0307041000.01	3.933	7.696	1.034377	2.91E-44	2.78E-44	3.83	3.94	4.03	7.78	7.59	7.72
OsR498G0307042100.01	3.296	0.186	-4.13016	3.82E-32	4.19E-32	2.72	4.28	2.89	0.14	0.21	0.21
OsR498G0307045300.01	11.83	5.536	-1.11093	5.60E-39	5.64E-39	12.43	11.51	11.55	6.28	4.91	5.42
OsR498G0307046600.01	0.406	0.09	-2.14358	3.47E-05	7.54E-05	0.26	0.49	0.47	0.04	0.09	0.14
OsR498G0307048300.01	8.44	29.886	1.81074	4.10E-223	1.56E-223	8.36	9.41	7.55	28.99	31.04	29.63
OsR498G0307051700.01	3.37	0.79	-2.07338	2.88E-46	2.69E-46	3.6	2.77	3.74	0.68	0.86	0.83
OsR498G0307052800.01	62.936	18.293	-1.77516	0	0	62.84	66.31	59.66	18.35	18.09	18.44
OsR498G0307055400.01	26.79	8.663	-1.63482	5.44E-301	1.66E-301	25.98	26.8	27.59	8.68	8.27	9.04
OsR498G0307056700.01	10.276	22.093	1.10176	8.53E-48	7.83E-48	9.36	11.6	9.87	21.99	22.56	21.73
OsR498G0307057300.01	5.14	2.203	-1.25604	4.82E-22	6.25E-22	4.97	5	5.45	1.69	2.56	2.36
OsR498G0307058300.01	13.546	3.866	-1.80154	2.75E-267	9.20E-268	13.85	13.1	13.69	4.16	3.57	3.87
OsR498G0307058800.01	31.266	10.603	-1.59264	5.59E-234	2.06E-234	31.14	30.13	32.53	11.26	10.43	10.12
OsR498G0307064700.01	13.813	53.856	1.936269	0	0	13.22	13.94	14.28	52.38	55.5	53.69
OsR498G0307065300.01	36.103	136.71	1.946558	0	0	37.37	35.64	35.3	135.15	136.28	138.7
OsR498G0307070000.01	19.623	57.01	1.526012	0	0	18.9	19.62	20.35	55.3	57.33	58.4
OsR498G0307070600.01	0.383	1.02	1.387683	2.59E-05	5.58E-05	0.42	0.22	0.51	1.29	1	0.77
OsR498G0307071000.01	0.446	0.093	-2.17323	1.14E-07	2.17E-07	0.5	0.41	0.43	0.15	0	0.13
OsR498G0307072300.01	0.88	3.68	2.05601	4.95E-35	5.23E-35	0.74	0.88	1.02	3.7	3.86	3.48
OsR498G0307073200.01	1.4	0.55	-1.3449	2.55E-07	4.96E-07	1.77	1.4	1.03	0.81	0.56	0.28
OsR498G0307073400.01	40.42	8.66	-2.23175	0	0	40.23	38.99	42.04	9.67	8.46	7.85
OsR498G0307074100.01	0.05	0.323	2.645908	6.24E-08	1.18E-07	0.07	0.03	0.05	0.31	0.25	0.41

OsR498G0307076300.01	10.213	189.61	4.2088	0	0	9.91	9.5	11.23	199.89	184.26	184.67
OsR498G0307077000.01	7.01	47.34	2.825289	0	0	8.81	6.32	5.9	47.2	48.69	46.13
OsR498G0307078200.01	7.71	3.096	-1.32281	7.98E-80	5.56E-80	7.73	7.37	8.03	2.96	3.18	3.15
OsR498G0307079200.01	1.516	0.35	-2.13956	1.64E-10	2.83E-10	1.89	1.24	1.42	0.18	0.5	0.37
OsR498G0307081300.01	12.536	1.833	-2.79241	6.56E-154	3.15E-154	12.87	12.88	11.86	1.74	2.1	1.66
OsR498G0307081600.01	9.59	1.096	-3.15236	4.26E-204	1.72E-204	10.02	8.52	10.23	1.22	0.98	1.09
OsR498G0307082500.01	0.493	0.12	-2.04116	1.20E-07	2.30E-07	0.43	0.56	0.49	0.11	0.08	0.17
OsR498G0307083700.01	0.02	0.24	3.499192	4.83E-06	9.99E-06	0.06	0	0	0.44	0.05	0.23
OsR498G0307084100.01	0.983	2.84	1.535718	4.95E-18	6.97E-18	0.9	1.33	0.72	2.62	3.3	2.6
OsR498G0307084500.01	31.396	87.28	1.470272	0	0	32.85	29.91	31.43	90.43	85.63	85.78
OsR498G0307084700.01	2.52	1.143	-1.11979	5.08E-14	7.89E-14	2.35	2.64	2.57	0.96	1.38	1.09
OsR498G0307086600.01	16.45	83.82	2.34768	0	0	16.21	16.43	16.71	84.39	82.73	84.34
OsR498G0307086900.01	0.056	0.633	3.513691	3.02E-21	3.98E-21	0.04	0.06	0.07	0.71	0.6	0.59
OsR498G0307087600.01	0.696	2.53	1.854457	1.23E-39	1.23E-39	0.69	0.64	0.76	2.97	2.43	2.19
OsR498G0307094500.01	20.096	8.66	-1.27648	1.88E-97	1.17E-97	20.66	19.34	20.29	8.17	8.41	9.4
OsR498G0307096300.01	5.576	0.306	-4.18793	1.37E-136	7.07E-137	5.46	5.37	5.9	0.29	0.33	0.3
OsR498G0307096800.01	17.513	386.84	4.457545	0	0	17.03	17.03	18.48	410.17	378.03	372.33
OsR498G0307097600.01	17.156	66.336	1.949906	0	0	16.59	17.28	17.6	64.15	66.43	68.43
OsR498G0307104900.01	306.85	76.996	-2.0035	0	0	291.51	316.63	312.4	84.53	75.42	71.04
OsR498G0307107100.01	1.49	6.156	2.027049	2.92E-26	3.50E-26	1.73	0.99	1.75	6.12	5.54	6.81
OsR498G0307107300.01	47.653	1072.9	4.483654	0	0	47.69	49.2	46.07	1081.1	1032.2	1105.5
OsR498G0307107800.01	1.036	2.66	1.32333	3.12E-17	4.48E-17	0.99	0.99	1.13	3.14	2.74	2.1
OsR498G0307109100.01	0.06	0.27	2.155237	6.83E-08	1.29E-07	0.04	0.05	0.09	0.32	0.3	0.19
OsR498G0307109500.01	30.623	61.696	1.0051	2.68E-149	1.31E-149	30.81	31.27	29.79	60.85	62.95	61.29
OsR498G0307111200.01	0.706	0.1	-2.97519	1.14E-17	1.62E-17	0.75	0.6	0.77	0.05	0.15	0.1
OsR498G0307113100.01	7.656	23.58	1.628232	1.55E-264	5.21E-265	7.61	7.35	8.01	24.05	24.04	22.65
OsR498G0307114400.01	0.273	0.043	-2.69721	0.0001045	0.0002337	0.28	0.38	0.16	0.04	0.09	0
OsR498G0307114600.01	2.276	0.4	-2.51974	4.26E-41	4.19E-41	2.14	2.49	2.2	0.32	0.41	0.47
OsR498G0307114700.01	0.953	0.366	-1.34226	0.0001712	0.0003888	1.22	0.86	0.78	0.42	0.12	0.56
OsR498G0307120600.01	0.816	0.306	-1.32281	7.59E-05	0.0001682	1.06	0.54	0.85	0.56	0.27	0.09
OsR498G0307123800.01	47.323	15.783	-1.54043	3.80E-304	1.15E-304	48.59	45.61	47.77	15.4	16.48	15.47
OsR498G0307124500.01	11.313	48.886	2.107282	0	0	11.01	11.43	11.5	48.21	49.31	49.14
OsR498G0307127400.01	1.34	0.54	-1.31276	6.55E-10	1.15E-09	1.04	1.41	1.57	0.6	0.44	0.58
OsR498G0307128800.01	2.196	30.006	3.783673	0	0	2.16	2.24	2.19	29.03	30	30.99
OsR498G0307130100.01	3.36	13.94	2.048088	2.07E-210	8.20E-211	3.49	3.66	2.93	13.99	13.65	14.18
OsR498G0307131300.01	4.553	0.08	-6.57658	4.59E-203	1.85E-203	5.08	4.19	4.39	0.11	0.05	0.08
OsR498G0307131800.01	1835.1	692.71	-1.41503	0	0	1828.9	1829.7	1847	706.44	683.68	688.02
OsR498G0307133300.01	8.51	1.64	-2.40849	1.03E-138	5.27E-139	8.89	8.08	8.56	1.9	1.52	1.5
OsR498G0307135200.01	67.65	33.25	-1.02554	2.30E-125	1.25E-125	74.06	64.58	64.31	35.06	34.28	30.41
OsR498G0307136300.01	3.21	1.45	-1.13556	6.46E-20	8.76E-20	3.88	2.86	2.89	1.34	1.58	1.43
OsR498G0307136500.01	0.366	1.09	1.567007	5.57E-13	8.87E-13	0.36	0.43	0.31	0.98	1.13	1.16
OsR498G0307137500.01	0.393	0.093	-2.05335	1.15E-08	2.12E-08	0.36	0.35	0.47	0.12	0.05	0.11
OsR498G0307138100.01	38.683	15.29	-1.34538	1.07E-284	3.43E-285	37.97	39.96	38.12	15.45	14.66	15.76
OsR498G0307139600.01	0.21	1.966	3.246918	2.38E-30	2.67E-30	0.22	0.3	0.11	2.29	1.61	2
OsR498G0307140500.01	14.03	35.773	1.394371	1.76E-85	1.18E-85	13.88	13.83	14.38	36.08	35.09	36.15
OsR498G0307142500.01	2.246	14.846	2.646325	4.29E-211	1.69E-211	2.1	2.35	2.29	15.16	15.19	14.19
OsR498G0307145600.01	773.19	189.36	-2.04874	0	0	784.55	769.35	765.7	209.07	179.95	179.06
OsR498G0307151900.01	4.566	1.706	-1.44345	9.50E-36	9.96E-36	4.49	4.51	4.7	1.41	1.86	1.85
OsR498G0307152900.01	0.553	0.043	-3.6041	1.31E-06	2.63E-06	0.64	0.33	0.69	0.05	0	0.08
OsR498G0307153700.01	0.463	1.686	1.941973	9.18E-23	1.17E-22	0.45	0.36	0.58	1.45	1.97	1.64
OsR498G0307154200.01	65.556	9.28	-1.87599	0	0	64.5	66.16	66.01	9.06	9.37	9.41
OsR498G0307154800.01	14.08	6.813	-1.01927	1.01E-34	1.07E-34	14.41	13.82	14.01	6.64	6.37	7.43
OsR498G0307156300.01	4.19	0.05	-6.43597	4.14E-63	3.28E-63	4.77	3.61	4.19	0.1	0.05	0
OsR498G0307157700.01	241.73	66.723	-1.89186	0	0	242.8	236.93	245.5	65.69	68.23	66.25
OsR498G0307167400.01	73.56	24.71	-1.50349	0	0	73.89	71.14	75.65	25.25	23.98	24.9
OsR498G0307171400.01	15.57	1.136	-3.78072	5.97E-243	2.14E-243	19.53	14.01	13.17	1.09	1.13	1.19
OsR498G0307172400.01	3.186	0.913	-1.83278	9.86E-25	1.21E-24	2.89	2.88	3.79	0.73	1	1.01
OsR498G0307173400.01	0.603	24.446	5.311036	0	0	0.69	0.49	0.63	24.86	25.38	23.1
OsR498G0307173600.01	14.503	32.26	1.112424	5.23E-98	3.25E-98	14.58	14.52	14.41	34.5	30.97	31.31
OsR498G0307173800.01	1.326	0.19	-2.87672	1.18E-25	1.42E-25	1.33	1.42	1.23	0.19	0.16	0.22
OsR498G0307174300.01	203.73	421.98	1.044073	0	0	197.7	209.81	203.7	429.25	417.32	419.36
OsR498G0307174800.01	39.803	11.983	-1.73629	2.89E-160	1.35E-160	38.45	40.43	40.53	13.21	10.79	11.95
OsR498G0307176300.01	20.523	1.94	-3.4029	5.83E-237	2.13E-237	21.58	21.14	18.85	2.04	1.65	2.13
OsR498G0307176900.01	0.776	3.976	2.344758	1.85E-78	1.30E-78	0.72	0.61	1	3.96	3.97	4
OsR498G0307179100.01	0.593	1.986	1.747579	3.98E-06	8.19E-06	0.58	0.86	0.34	1.68	2.21	2.07
OsR498G0307180500.01	1.21	0.326	-1.89313	5.10E-22	6.61E-22	0.95	1.37	1.31	0.35	0.22	0.41
OsR498G0307182700.01	0.156	12.006	6.22238	2.76E-191	1.16E-191	0.13	0.09	0.25	13.34	10.95	11.73
OsR498G0307183500.01	3.256	0.966	-1.77669	7.23E-37	7.45E-37	3.6	3.19	2.98	1.05	1.05	0.8
OsR498G0307183800.01	0.646	2.943	2.191763	8.80E-83	6.01E-83	0.57	0.71	0.66	3.05	2.86	2.92

OsR498G0307184200.01	20.37	77.306	1.931229	0	0	17.37	22.23	21.51	78.21	77.58	76.13
OsR498G0307188500.01	1.486	0.553	-1.42973	0.0004759	0.0011163	1.59	1.93	0.94	0.63	0.39	0.64
OsR498G0307190300.01	0.14	4.466	4.974665	2.53E-123	1.38E-123	0.1	0.1	0.22	4.3	4.66	4.44
OsR498G0307191600.01	6.823	2.52	-1.25606	1.27E-20	1.69E-20	7.19	6.82	6.46	2.31	2.84	2.41
OsR498G0307191800.01	0.74	0.19	-1.89456	2.78E-05	6.01E-05	0.81	0.67	0.74	0.1	0.21	0.26
OsR498G0307194200.01	0.146	0.64	2.098654	0.000141	0.0003183	0.07	0.23	0.14	0.45	0.93	0.54
OsR498G0307195900.01	3.956	31.683	2.990924	3.02E-221	1.15E-221	3.64	4.04	4.19	32.65	32.26	30.14
OsR498G0307197200.01	32.546	85.826	1.407036	0	0	33.01	32.1	32.53	87.55	81.53	88.4
OsR498G0307197400.01	62.103	27.203	-1.19781	2.86E-219	1.10E-219	61.06	61.31	63.94	27.33	27.12	27.16
OsR498G0307197700.01	6.43	3.206	-1.13059	7.31E-40	7.28E-40	5.65	7.47	6.17	2.93	3.83	2.86
OsR498G0307199400.01	30.373	142.05	2.220309	0	0	29.74	30.91	30.47	151.09	139.27	135.78
OsR498G0307210400.01	2.32	0.316	-2.85909	6.90E-30	7.80E-30	2.27	2.94	1.75	0.51	0.24	0.2
OsR498G0307211000.01	0.21	0.026	-3.0121	8.51E-05	0.0001893	0.19	0.08	0.36	0.04	0.04	0
OsR498G0307219100.01	17.09	8.523	-1.01275	1.44E-44	1.37E-44	18.49	16	16.78	7.99	8.3	9.28
OsR498G0307219900.01	0	0.39	5.127223	8.48E-06	1.78E-05	0	0	0	0.47	0.42	0.28
OsR498G0307220700.01	19.826	67.85	1.769918	0	0	20.6	19.12	19.76	70.27	68.86	64.42
OsR498G0307221200.01	0.243	1.656	2.734347	1.17E-39	1.17E-39	0.2	0.3	0.23	1.72	1.64	1.61
OsR498G0307221600.01	6.73	34.01	2.335615	0	0	6.76	6.89	6.54	36.32	33.29	32.42
OsR498G0307222300.01	1.163	0.36	-2.10708	1.13E-08	2.07E-08	1.34	0.8	1.35	0.17	0.66	0.25
OsR498G0307223600.01	6.81	2.23	-1.62892	2.81E-54	2.42E-54	6.92	6.81	6.7	2.14	2.31	2.24
OsR498G0307224100.01	0.42	3.396	3.002393	1.00E-59	8.22E-60	0.46	0.33	0.47	3.62	3.05	3.52
OsR498G0307225500.01	6.58	13.956	1.071703	6.82E-25	8.37E-25	6.1	6.26	7.38	14.75	13.39	13.73
OsR498G0307226200.01	1.066	24.213	4.510502	0	0	1.03	1.33	0.84	24.9	23.18	24.56
OsR498G0307227600.01	0.423	0.08	-2.37528	0.0009088	0.0021805	0.71	0.33	0.23	0.08	0.08	0.08
OsR498G0307229200.01	0.98	2.223	1.185838	1.67E-08	3.08E-08	1.24	1	0.7	2.47	2.22	1.98
OsR498G0307229400.01	0.146	0.366	1.302794	0.000167	0.0003787	0.15	0.12	0.17	0.27	0.46	0.37
OsR498G0307230500.01	7.71	2.396	-1.69118	4.28E-41	4.22E-41	8.04	7.88	7.21	2.45	2.13	2.61
OsR498G0307231500.01	0.87	5.55	2.675349	4.82E-41	4.75E-41	0.92	1.1	0.59	5.52	5.37	5.76
OsR498G0307232300.01	0	0.243	4.499192	0.0003163	0.0007323	0	0	0	0.33	0.2	0.2
OsR498G0307234400.01	5.56	55.913	3.324954	0	0	6.1	5.42	5.16	55.46	56.27	56.01
OsR498G0307235100.01	206.79	91.87	-1.14587	0	0	208.4	206.78	205.2	93.7	92.32	89.59
OsR498G0307236300.01	0.04	0.156	2.361688	0.0002336	0.0005362	0	0.12	0	0.15	0.17	0.15
OsR498G0307239100.01	67.916	514.22	2.905236	0	0	71.99	65.25	66.51	498.39	522.57	521.69
OsR498G0307240900.01	1.396	0.153	-3.19471	1.05E-29	1.19E-29	1.36	1.37	1.46	0.24	0.14	0.08
OsR498G0307241700.01	0.14	0.82	1.755967	6.95E-06	1.45E-05	0.23	0.13	0.06	0.93	0.91	0.62
OsR498G0307242100.01	0.293	0.113	-1.455	0.0009085	0.0021791	0.39	0.23	0.26	0.11	0.06	0.17
OsR498G0307242800.01	12.026	4.976	-1.30676	7.98E-174	3.56E-174	11.23	12.49	12.36	4.62	5.42	4.89
OsR498G0307244500.01	305.71	883.89	1.521877	0	0	323.21	297.93	296	903.1	879.51	869.06
OsR498G0307245800.01	0.236	0.613	1.472719	3.58E-11	6.03E-11	0.23	0.21	0.27	0.64	0.55	0.65
OsR498G0307246200.01	31.453	87.88	1.463229	0	0	32.06	30.51	31.79	93.78	87.32	82.54
OsR498G0307248900.01	0.733	0.113	-2.83939	6.93E-18	9.80E-18	0.7	0.8	0.7	0.12	0.04	0.18
OsR498G0307249400.01	12.13	56.916	2.229327	0	0	12.38	12.73	11.28	54.99	58.74	57.02
OsR498G0307250200.01	3.343	7.056	1.119684	1.65E-39	1.65E-39	3.61	3.21	3.21	6.54	7.73	6.9
OsR498G0307251700.01	0.89	0.033	-4.76759	9.54E-21	1.27E-20	0.95	0.83	0.89	0	0.07	0.03
OsR498G0307253600.01	0.536	0.036	-3.86713	8.60E-05	0.0001913	0.43	0.56	0.62	0.11	0	0
OsR498G0307254000.01	0.16	0.56	1.817368	0.0005684	0.0013412	0.14	0.14	0.2	0.76	0.5	0.42
OsR498G0307254300.01	6.726	2.77	-1.32112	1.16E-29	1.32E-29	5.94	6.99	7.25	3.3	2.75	2.26
OsR498G0307258800.01	2.03	7.643	1.912023	3.25E-132	1.70E-132	1.74	1.97	2.38	7.04	8.15	7.74
OsR498G0307266400.01	4.69	10.816	1.257029	1.31E-52	1.15E-52	5.02	4.65	4.4	9.81	10.86	11.78
OsR498G0307266700.01	21.943	371.65	4.076761	0	0	21.83	22.51	21.49	384.38	369.82	360.75
OsR498G0307268700.01	0.473	1.993	2.093614	7.94E-10	1.40E-09	0.58	0.84	0	1.52	3.58	0.88
OsR498G0307269200.01	12.736	5.64	-1.1859	2.31E-44	2.20E-44	12.22	12.89	13.1	5.45	6.29	5.18
OsR498G0307271400.01	6.083	2.89	-1.07694	3.38E-34	3.62E-34	6	6.14	6.11	2.96	2.63	3.08
OsR498G0307272000.01	0.55	1.393	1.334381	3.45E-11	5.82E-11	0.53	0.67	0.45	1.43	1.33	1.42
OsR498G0307273300.01	2.166	0.306	-2.8936	2.06E-45	1.94E-45	2.47	1.74	2.29	0.38	0.22	0.32
OsR498G0307273500.01	0.036	0.273	2.946651	2.67E-09	4.78E-09	0.07	0.02	0.02	0.3	0.26	0.26
OsR498G0307274000.01	1.08	5.813	2.401528	4.74E-36	4.94E-36	1.24	0.55	1.45	6.41	5.92	5.11
OsR498G0307274900.01	18.376	5.28	-1.79978	0	0	17.44	18.5	19.19	5.21	5.45	5.18
OsR498G0307278600.01	0.466	0.053	-3.16969	3.21E-08	6.00E-08	0.45	0.55	0.4	0.04	0.08	0.04
OsR498G0307283400.01	9.236	2.023	-2.21985	3.82E-71	2.84E-71	8.44	10.27	9	1.58	2.27	2.22
OsR498G0307284200.01	31.443	10.706	-1.5556	2.70E-123	1.48E-123	30.85	31.65	31.83	10.92	10.51	10.69
OsR498G0307284700.01	25.503	60.393	1.23631	1.95E-188	8.28E-189	25.07	26.65	24.79	60.44	59.28	61.46
OsR498G0307285700.01	0.11	19.803	7.464346	2.05E-192	8.62E-193	0.13	0.07	0.13	21.45	18.86	19.1
OsR498G0307286100.01	0.143	211.53	11.06039	0	0	0	0.43	0	229.88	188.36	216.36
OsR498G0307286300.01	3.003	111.75	5.201717	0	0	2.79	2.77	3.45	121.75	103.18	110.33
OsR498G0307288900.01	77.106	8.506	-3.1955	0	0	83.28	70.54	77.5	8.64	7.62	9.26
OsR498G0307289800.01	1.33	0.276	-2.28217	8.27E-05	0.0001838	0.84	1.93	1.22	0.25	0.45	0.13
OsR498G0307290000.01	9.103	56.496	2.658429	0	0	9.07	9.12	9.12	57	57.18	55.31
OsR498G0307292500.01	14.166	38.666	1.441096	0	0	13.71	14.04	14.75	38.47	38.91	38.62

OsR498G0307298200.01	8.213	3.103	-1.48886	3.99E-72	2.94E-72	7.82	8.4	8.42	2.62	2.74	3.95
OsR498G0307298600.01	3.973	1.056	-1.90269	3.14E-68	2.39E-68	4.23	3.77	3.92	1.27	0.76	1.14
OsR498G0307298700.01	17.28	5.293	-1.71138	7.18E-110	4.19E-110	18.86	17.68	15.3	4.94	5.15	5.79
OsR498G0307301100.01	1.46	0.516	-1.49176	9.58E-13	1.54E-12	1.21	1.75	1.42	0.57	0.32	0.66
OsR498G0307301500.01	19.64	3.086	-2.7133	3.65E-264	1.23E-264	19.33	18.36	21.23	2.99	3	3.27
OsR498G0307301700.01	1.366	0.02	-6.09621	1.85E-34	1.97E-34	1.36	1.32	1.42	0.03	0	0.03
OsR498G0307302000.01	0.76	0	-4.3318	0.0005774	0.0013628	0.25	0.85	1.18	0	0	0
OsR498G0307302800.01	8.936	20.313	1.180022	2.53E-88	1.67E-88	8.24	9.53	9.04	19.03	20.67	21.24
OsR498G0307305000.01	0.023	0.413	4.127223	3.49E-09	6.28E-09	0.04	0	0.03	0.65	0.3	0.29
OsR498G0307305400.01	0.106	0.303	1.509245	0.0002521	0.0005801	0.1	0.13	0.09	0.43	0.23	0.25
OsR498G0307305700.01	111.03	498.44	2.162944	0	0	114.74	108.92	109.4	509.48	494.72	491.12
OsR498G0307310800.01	0	0.13	5.209685	4.72E-06	9.74E-06	0	0	0	0.15	0.11	0.13
OsR498G0307311400.01	0	1.073	7.531613	8.92E-22	1.16E-21	0	0	0	1.16	1.27	0.79
OsR498G0307312200.01	1.033	24.286	4.539606	2.91E-288	9.23E-289	1.28	0.7	1.12	26.36	22.47	24.03
OsR498G0307314400.01	0	0.276	4.49788	0.0003182	0.000737	0	0	0	0.3	0.23	0.3
OsR498G0307318800.01	0.543	0.043	-3.81822	1.78E-13	2.79E-13	0.44	0.73	0.46	0	0.04	0.09
OsR498G0307321700.01	3	1.223	-1.19855	9.46E-14	1.48E-13	3.06	2.98	2.96	0.9	1.5	1.27
OsR498G0307323500.01	0.386	0.013	-5.4838	1.58E-12	2.56E-12	0.26	0.45	0.45	0	0.04	0
OsR498G0307324500.01	4.736	2.21	-1.11911	1.64E-27	1.92E-27	4.84	4.92	4.45	2.11	2.52	2
OsR498G0307324800.01	44.863	18.7	-1.29375	1.52E-108	8.97E-109	46.23	42.94	45.42	17.21	19.11	19.78
OsR498G0307325300.01	34.896	106.43	1.341794	0	0	34.63	34.17	35.89	103.63	110.8	104.87
OsR498G0307329900.01	37.373	7.706	-2.28709	5.10E-225	1.93E-225	35.48	37.59	39.05	8.03	7.24	7.85
OsR498G0307330200.01	3.976	0.34	-3.55516	7.93E-25	9.75E-25	4.64	4.33	2.96	0.22	0.23	0.57
OsR498G0307330400.01	2.61	0.186	-3.96252	9.91E-58	8.26E-58	2.69	2.74	2.4	0.18	0.15	0.23
OsR498G0307330500.01	10.443	20.726	1.032591	2.81E-65	2.19E-65	10.12	10.33	10.88	20.89	20.7	20.59
OsR498G0307334500.01	0.476	0.096	-2.30943	9.13E-09	1.67E-08	0.33	0.53	0.57	0	0.16	0.13
OsR498G0307335000.01	0.17	0	-4.96024	2.02E-05	4.33E-05	0.22	0.17	0.12	0	0	0
OsR498G0307337900.01	34.53	8.613	-2.01919	0	0	33.9	35.24	34.45	8.96	8.08	8.8
OsR498G0307338600.01	9.456	1.413	-2.73897	5.76E-84	3.90E-84	9.72	9.36	9.29	1.88	0.92	1.44
OsR498G0307338700.01	73.566	11.863	-2.68136	0	0	74.44	73.04	73.22	11.14	11.91	12.54
OsR498G0307339000.01	3.423	19.866	2.524948	0	0	3	3.45	3.82	21.1	19.71	18.79
OsR498G0307339700.01	5.26	211.74	5.32575	0	0	5.1	5.15	5.53	226.83	203.47	204.92
OsR498G0307339900.01	0.176	5.533	4.927285	2.13E-36	2.21E-36	0.21	0.11	0.21	5.54	5.43	5.63
OsR498G0307340300.01	0.073	0.82	3.499192	2.42E-10	4.18E-10	0.11	0.06	0.05	0.88	0.68	0.9
OsR498G0307341100.01	2.196	0.853	-1.36715	4.88E-16	7.19E-16	2.03	2.33	2.23	0.76	0.82	0.98
OsR498G0307342100.01	51.66	26.99	-1.00326	2.46E-95	1.55E-95	51.48	51.87	51.63	28.33	24.38	28.26
OsR498G0307343400.01	1.93	0.9	-1.10567	6.63E-05	0.0001466	1.81	2.23	1.75	0.81	1.32	0.57
OsR498G0307344800.01	21.973	63.216	1.5191	0	0	22.61	21.6	21.71	61.44	65.44	62.77
OsR498G0307345400.01	19.646	4.976	-1.98442	1.07E-256	3.68E-257	19.21	20.09	19.64	4.94	5.09	4.9
OsR498G0307348400.01	0.13	2.76	4.432077	2.64E-58	2.19E-58	0.13	0.2	0.06	2.74	2.7	2.84
OsR498G0307353600.01	73.833	30.783	-1.39578	0	0	70.91	75.32	75.27	30.05	31.45	30.85
OsR498G0307354300.01	196.05	1.973	-6.18315	0	0	207.69	190.32	190.2	1.99	2.29	1.64
OsR498G0307357600.01	96.573	38.346	-1.34185	0	0	100.81	94.7	94.21	37.89	36.34	40.81
OsR498G0307357800.01	30.506	138.72	2.172906	0	0	28.49	33.3	29.73	146.26	132.22	137.67
OsR498G0307359200.01	13.33	26.96	1.006282	3.35E-145	1.67E-145	13.47	13.51	13.01	25.83	27.58	27.47
OsR498G0307363400.01	21.41	6.85	-1.64173	2.04E-101	1.25E-101	20.49	21.75	21.99	6.64	6.55	7.36
OsR498G0307366700.01	0.276	0	-5.30151	1.75E-06	3.54E-06	0.36	0.17	0.3	0	0	0
OsR498G0307367000.01	7.31	2.353	-1.63272	1.40E-91	9.05E-92	6.53	7.73	7.67	2.17	2.43	2.46
OsR498G0307367200.01	0.15	1.176	2.982275	1.65E-22	2.12E-22	0.19	0.15	0.11	1.12	1.17	1.24
OsR498G0307367700.01	14.9	45.56	1.611204	3.21E-227	1.21E-227	14.96	14.63	15.11	47.82	43.76	45.1
OsR498G0307369100.01	6.113	57.76	3.242732	0	0	5.57	6.7	6.07	61.2	57.28	54.8
OsR498G0307370400.01	0.183	0.57	1.702725	1.16E-06	2.32E-06	0.18	0.25	0.12	0.5	0.67	0.54
OsR498G0307370500.01	1.376	8.493	2.614875	6.08E-130	3.23E-130	1.13	1.15	1.85	8.93	8.25	8.3
OsR498G0307371900.01	14.926	2.18	-2.77993	1.68E-251	5.88E-252	14.95	13.24	16.59	1.67	1.96	2.91
OsR498G0307373000.01	80.543	31.306	-1.33019	0	0	84.33	77.26	80.04	31.39	32.48	30.05
OsR498G0307373200.01	20.436	5.69	-1.85235	1.54E-139	7.87E-140	21.3	19.39	20.62	5.71	5.78	5.58
OsR498G0307373800.01	3.233	0.706	-2.19587	7.68E-19	1.06E-18	3.84	2.92	2.94	1.01	0.35	0.76
OsR498G0307375500.01	8.026	2.533	-1.72915	7.86E-60	6.42E-60	7.69	8.53	7.86	2.87	2.43	2.3
OsR498G0307376600.01	28.613	54.376	1.052821	2.64E-241	9.52E-242	29.47	27.39	28.98	52.34	53.16	57.63
OsR498G0307377600.01	6.24	2.893	-1.11441	9.36E-15	1.42E-14	6.22	6.73	5.77	3.02	3.17	2.49
OsR498G0307378500.01	0.013	0.24	4.361688	3.01E-06	6.15E-06	0.04	0	0	0.39	0.22	0.11
OsR498G0307382800.01	27.803	65.89	1.236998	9.36E-187	4.00E-187	29.51	26.81	27.09	69.52	65.32	62.83
OsR498G0307383000.01	17.103	65.776	1.921072	0	0	17.53	17.25	16.53	68.41	63.11	65.81
OsR498G0307383200.01	5.773	1.903	-1.57437	5.23E-27	6.20E-27	5.85	6.3	5.17	2.11	2.07	1.53
OsR498G0307383500.01	3.743	0.673	-2.47365	1.41E-25	1.71E-25	4.28	3.97	2.98	0.84	0.46	0.72
OsR498G0307391500.01	1.703	0.406	-2.06219	1.95E-27	2.29E-27	1.81	1.61	1.69	0.64	0.42	0.16
OsR498G0307392200.01	0.836	0.25	-1.76155	1.85E-06	3.74E-06	1.09	0.71	0.71	0.1	0.25	0.4
OsR498G0307392900.01	19.5	2.536	-2.94275	3.37E-244	1.20E-244	18.83	19.24	20.43	2.79	1.85	2.97
OsR498G0307394300.01	0.81	0	-7.26402	3.21E-19	4.42E-19	0.79	0.96	0.68	0	0	0

OsR498G0307395500.01	3.61	0.163	-4.46274	9.48E-17	1.38E-16	3.62	4.32	2.89	0.16	0.33	0
OsR498G0307395900.01	2.396	0.913	-1.41481	1.75E-06	3.55E-06	3.12	1.44	2.63	0.8	1.33	0.61
OsR498G0307396400.01	622.5	122.26	-2.34354	0	0	623.97	610.83	632.7	121.94	127.58	117.25
OsR498G0307396600.01	1.656	0.046	-5.36963	1.55E-21	2.03E-21	1.24	1.24	2.49	0.14	0	0
OsR498G0307396700.01	5.656	0.926	-2.61596	2.06E-58	1.71E-58	6.57	4.88	5.52	1.09	0.94	0.75
OsR498G0307401600.01	0	0.353	4.946651	2.78E-05	6.01E-05	0	0	0	0.42	0.43	0.21
OsR498G0307403900.01	2.666	0.506	-2.39821	8.22E-25	1.01E-24	2.5	2.9	2.6	0.73	0.47	0.32
OsR498G0407413200.01	0.376	0.13	-1.50773	0.0002159	0.0004939	0.29	0.53	0.31	0.09	0.09	0.21
OsR498G0407414100.01	0.18	0	-4.41967	0.0003895	0.0009082	0.19	0.21	0.14	0	0	0
OsR498G0407414400.01	0.84	0.186	-2.00653	1.07E-11	1.77E-11	0.88	0.63	1.01	0.17	0.24	0.15
OsR498G0407421200.01	0.486	2.323	2.26644	1.08E-43	1.03E-43	0.4	0.63	0.43	2.41	2.4	2.16
OsR498G0407425700.01	1.773	0.066	-4.66068	7.90E-31	8.81E-31	2.03	1.92	1.37	0.12	0	0.08
OsR498G0407426700.01	15.08	32.243	1.093398	2.83E-144	1.41E-144	14.8	15.66	14.78	33.56	30.68	32.49
OsR498G0407428300.01	0.256	0.86	1.755967	6.95E-06	1.45E-05	0.32	0.4	0.05	0.77	1.08	0.73
OsR498G0407430700.01	15.3	33.913	1.142263	6.43E-128	3.45E-128	14.81	15.3	15.79	33.87	34.12	33.75
OsR498G0407430900.01	0.556	0	-6.20817	1.23E-10	2.11E-10	0.35	0.85	0.47	0	0	0
OsR498G0407438700.01	14.913	5.29	-1.49604	2.97E-63	2.35E-63	14.98	15.23	14.53	4.33	5.49	6.05
OsR498G0407442600.01	0.04	0.91	4.499192	1.06E-26	1.27E-26	0	0.03	0.09	0.92	0.96	0.85
OsR498G0407445000.01	0.026	1.293	5.466025	2.28E-22	2.94E-22	0.04	0	0.04	1.28	1.33	1.27
OsR498G0407447100.01	1.623	3.526	1.107223	1.42E-10	2.44E-10	2.02	1.2	1.65	3.27	3.27	4.04
OsR498G0407447600.01	29.036	74.15	1.329366	6.96E-287	2.21E-287	28.17	31.16	27.78	71.15	74.65	76.65
OsR498G0407449200.01	0.4	0.15	-1.40081	1.54E-06	3.11E-06	0.41	0.35	0.44	0.19	0.18	0.08
OsR498G0407453200.01	3.386	7.703	1.13877	3.47E-38	3.53E-38	3.53	3.53	3.1	7.28	8.38	7.45
OsR498G0407480900.01	1.28	0.026	-5.85506	1.58E-29	1.80E-29	1.35	1.24	1.25	0.04	0.04	0
OsR498G0407486900.01	1.033	1.43	-1.08147	9.16E-16	1.36E-15	1.08	1.04	0.98	1.41	1.45	1.43
OsR498G0407487300.01	7.79	1.213	-2.68775	2.53E-47	2.33E-47	7.41	8.48	7.48	1.61	0.78	1.25
OsR498G0407490300.01	8.276	0.126	-6.79443	4.25E-257	1.46E-257	7.87	9.16	7.8	0.19	0.1	0.09
OsR498G0407492700.01	4.813	1.106	-2.14588	1.04E-74	7.56E-75	4.77	4.42	5.25	1	1.08	1.24
OsR498G0407493300.01	6.32	1.77	-1.95577	5.54E-58	4.61E-58	6.77	5.96	6.23	1.46	1.77	2.08
OsR498G0407494400.01	4.653	1.323	-1.81615	8.25E-65	6.47E-65	4.46	4.98	4.52	1.32	1.16	1.49
OsR498G0407494600.01	0.053	0.223	2.03976	1.05E-05	2.22E-05	0.04	0.02	0.1	0.11	0.3	0.26
OsR498G0407495300.01	2.49	0.043	-5.36065	6.86E-80	4.77E-80	1.96	2.72	2.79	0.03	0.03	0.07
OsR498G0407495500.01	1.163	0	-6.28613	4.16E-11	7.01E-11	1.01	1.63	0.85	0	0	0
OsR498G0407498300.01	0.533	3.21	2.577266	3.03E-54	2.60E-54	0.61	0.34	0.65	3.43	3.18	3.02
OsR498G0407500300.01	0.32	2.026	2.672687	4.01E-30	4.52E-30	0.26	0.41	0.29	1.86	2.1	2.12
OsR498G0407506800.01	0.403	0.14	-1.59767	0.0007574	0.0018079	0.39	0.36	0.46	0.09	0.23	0.1
OsR498G0407507200.01	0.27	0.086	-1.67922	0.0006533	0.0015505	0.15	0.12	0.54	0.06	0	0.2
OsR498G0407514500.01	0.046	0	-4.28217	0.0007127	0.0016975	0.04	0.07	0.03	0	0	0
OsR498G0407520700.01	4.48	1.27	-1.82478	1.73E-19	2.36E-19	3.6	5.02	4.82	1.35	1.09	1.37
OsR498G0407526200.01	60.336	2.03	-4.91128	0	0	62.38	58.79	59.84	1.91	2.35	1.83
OsR498G0407550800.01	3.21	1.53	-1.0818	4.62E-10	8.08E-10	3.6	2.49	3.54	1.51	1.41	1.67
OsR498G0407554000.01	0.013	0.456	4.847115	7.07E-28	8.25E-28	0.02	0.01	0.01	0.47	0.4	0.5
OsR498G0407560200.01	0.926	0.403	-1.21022	3.62E-06	7.43E-06	0.88	0.96	0.94	0.32	0.4	0.49
OsR498G0407567400.01	4.85	0.44	-3.47356	4.57E-44	4.37E-44	3.34	5.91	5.3	0.39	0.46	0.47
OsR498G0407591100.01	1.443	0.466	-1.62464	6.80E-15	1.03E-14	1.59	1.5	1.24	0.57	0.38	0.45
OsR498G0407601700.01	2.076	0.256	-3.1393	4.16E-56	3.51E-56	1.93	2.04	2.26	0.2	0.31	0.26
OsR498G0407602000.01	0.136	0	-4.28217	0.0007127	0.0016975	0.08	0.13	0.2	0	0	0
OsR498G0407604600.01	214.81	42.273	-2.32429	0	0	214.9	211.81	217.7	42.73	42.36	41.73
OsR498G0407611500.01	0.506	0.083	-2.81822	2.84E-06	5.81E-06	0.45	0.75	0.32	0.07	0.06	0.12
OsR498G0407614400.01	3	8.453	1.485645	1.27E-42	1.24E-42	3.32	2.6	3.08	9.01	9.19	7.16
OsR498G0407615000.01	0.156	5.71	5.174529	3.92E-126	2.12E-126	0.16	0.13	0.18	5.45	5.88	5.8
OsR498G0407629800.01	2.756	0	-9.22703	4.52E-57	3.79E-57	2.57	2.91	2.79	0	0	0
OsR498G0407632800.01	9.59	3.566	-1.50081	1.11E-63	8.77E-64	10.5	9.44	8.83	3.35	3.74	3.61
OsR498G0407633300.01	1.386	0.586	-1.25259	1.89E-28	2.18E-28	1.44	1.3	1.42	0.58	0.44	0.74
OsR498G0407633800.01	0.316	0.823	1.548774	0.0001666	0.0003778	0.24	0.25	0.46	0.72	1.06	0.69
OsR498G0407633900.01	0.803	1.63	1.013765	0.0007763	0.0018533	0.68	0.99	0.74	1.93	1.8	1.16
OsR498G0407636100.01	9.093	3.36	-1.44609	5.94E-46	5.55E-46	10.14	7.62	9.52	3.37	3.31	3.4
OsR498G0407643700.01	1.4	14.27	3.34145	0	0	1.5	1.29	1.41	13.97	13.73	15.11
OsR498G0407644200.01	0.786	4.033	2.361688	9.47E-51	8.42E-51	0.97	0.88	0.51	3.99	4.16	3.95
OsR498G0407645200.01	0.21	3.613	4.0559	2.24E-43	2.16E-43	0.27	0	0.36	3.36	4.12	3.36
OsR498G0407647400.01	0.24	0.72	1.596978	7.48E-10	1.32E-09	0.1	0.38	0.24	0.57	0.79	0.8
OsR498G0407648100.01	0.573	3.373	2.556818	2.81E-49	2.54E-49	0.5	0.65	0.57	3.77	3.41	2.94
OsR498G0407654100.01	0.56	1.62	1.535639	1.90E-36	1.97E-36	0.54	0.63	0.51	1.4	1.72	1.74
OsR498G0407658300.01	0.146	0	-4.66068	0.0001179	0.0002647	0.3	0.04	0.1	0	0	0
OsR498G0407659900.01	2.573	1.17	-1.13588	3.20E-13	5.07E-13	2.76	2.79	2.17	1.48	0.96	1.07
OsR498G0407660100.01	0.54	6.66	3.730076	2.27E-145	1.13E-145	0.51	0.54	0.57	7.34	6.34	6.3
OsR498G0407661200.01	0.73	7.036	3.262152	3.15E-134	1.64E-134	1.06	0.56	0.57	7.25	6.78	7.08
OsR498G0407664200.01	0.043	7.896	7.813229	1.90E-237	6.92E-238	0.03	0.04	0.06	8.6	7.5	7.59
OsR498G0407666500.01	1.423	5.223	1.863934	7.44E-33	8.08E-33	0.98	1.25	2.04	6.92	4.38	4.37

OsR498G0407668300.01	0.59	20.616	5.124568	0	0	0.51	0.69	0.57	21.24	21.29	19.32
OsR498G0407673800.01	107.67	4.653	-4.54559	0	0	105.99	109.61	107.4	3.95	5.15	4.86
OsR498G0407674200.01	166.8	3.39	-5.63267	0	0	178.93	166.15	155.3	3.98	2.27	3.92
OsR498G0407682200.01	610.09	122.86	-2.27267	0	0	622.32	616.45	591.5	123.4	118.87	126.32
OsR498G0407683000.01	930.97	224.54	-2.06331	0	0	965.8	909.22	917.9	238.73	212.59	222.31
OsR498G0407699400.01	0.833	0.196	-1.99803	6.99E-22	9.08E-22	0.85	0.83	0.82	0.22	0.18	0.19
OsR498G0407700600.01	0.126	0	-4.41967	0.0003895	0.0009082	0.07	0.07	0.24	0	0	0
OsR498G0407701900.01	0.636	0.013	-5.71513	2.02E-14	3.11E-14	0.6	0.63	0.68	0	0.04	0
OsR498G0407703600.01	6.586	15.286	1.161224	4.66E-71	3.48E-71	5.52	6.84	7.4	15.57	15.52	14.77
OsR498G0407706500.01	0.956	0.243	-1.88624	6.41E-11	1.09E-10	0.85	1.14	0.88	0.31	0.15	0.27
OsR498G0407708700.01	1.99	0.403	-2.48178	1.09E-49	9.79E-50	1.87	2.04	2.06	0.38	0.44	0.39
OsR498G0407712200.01	2.946	0.473	-2.73147	1.50E-37	1.54E-37	2.23	3.82	2.79	0.49	0.39	0.54
OsR498G0407717000.01	51.026	3.543	-3.88098	0	0	53.4	49.58	50.1	3.8	3.48	3.35
OsR498G0407718900.01	0.843	2.883	1.799021	7.58E-40	7.55E-40	0.89	0.97	0.67	2.78	3	2.87
OsR498G0407719800.01	1.19	6.566	2.451574	1.04E-62	8.30E-63	1.61	1.02	0.94	6.31	6.52	6.87
OsR498G0407721400.01	1.863	50.793	4.765642	0	0	2.27	1.71	1.61	51.56	50.41	50.41
OsR498G0407721600.01	0.02	0.183	3.127223	9.61E-05	0.0002145	0.03	0	0.03	0.22	0.23	0.1
OsR498G0407721900.01	5.116	16.926	1.699539	3.93E-136	2.03E-136	4.57	5.77	5.01	16	18.05	16.73
OsR498G0407722200.01	0.103	0.443	2.287688	1.41E-06	2.84E-06	0	0.19	0.12	0.22	0.49	0.62
OsR498G0407734200.01	1.36	6.413	2.238321	4.85E-185	2.09E-185	1.33	1.43	1.32	6.11	6.7	6.43
OsR498G0407738100.01	0.216	1.476	2.762226	6.17E-07	1.22E-06	0.26	0.14	0.25	1.06	1.08	2.29
OsR498G0407764200.01	1174.4	2.85	-8.71834	0	0	1171.3	1177.6	1174	2.89	2.69	2.97
OsR498G0407764600.01	350.79	0.673	-9.04106	0	0	318.51	369.88	364	0.89	0.79	0.34
OsR498G0407772300.01	3	9.693	1.680688	3.94E-118	2.22E-118	2.97	2.7	3.33	9.23	9.9	9.95
OsR498G0407772900.01	0.113	1.356	3.254662	1.39E-08	2.56E-08	0.18	0.11	0.05	1.16	0.94	1.97
OsR498G0407773100.01	1.966	6.893	1.808795	8.30E-94	5.28E-94	2.15	1.87	1.88	6.59	7.21	6.88
OsR498G0407774900.01	0.493	2.523	2.371687	4.91E-75	3.54E-75	0.64	0.46	0.38	2.33	2.7	2.54
OsR498G0407776100.01	2.35	9.316	1.999977	9.46E-180	4.14E-180	2.08	2.62	2.35	9.04	9.41	9.5
OsR498G0407777300.01	34.62	15.946	-1.05427	2.23E-89	1.46E-89	35.72	34.55	33.59	15.66	16.97	15.21
OsR498G0407783600.01	1.823	4.95	1.438626	1.83E-29	2.09E-29	1.84	1.85	1.78	5.5	4.45	4.9
OsR498G0407801400.01	3.4	0.133	-4.97705	8.94E-61	7.24E-61	3.09	3.76	3.35	0.14	0.21	0.05
OsR498G0407816200.01	96.433	308.12	1.662268	0	0	95.42	96.27	97.61	302.07	310.44	311.84
OsR498G0407829400.01	1.766	0.683	-1.38108	7.19E-07	1.43E-06	1.89	1.93	1.48	0.91	0.6	0.54
OsR498G0407829800.01	84.86	22.45	-1.85221	0	0	81.88	87.39	85.31	22.2	20.66	24.49
OsR498G0407830500.01	11.27	3.246	-1.76764	8.24E-86	5.52E-86	11.43	11.69	10.69	3.02	3.32	3.4
OsR498G0407860000.01	19.763	45.6	1.192246	2.13E-174	9.49E-175	18.28	14	27.01	49	41.53	46.27
OsR498G0407861100.01	0.516	0	-4.46359	0.0003176	0.0007353	0.27	0.76	0.52	0	0	0
OsR498G0407863700.01	0	0.14	4.565829	0.0002295	0.0005263	0	0	0	0.34	0.08	0
OsR498G0407871000.01	0.043	0.28	2.720482	1.55E-18	2.16E-18	0.04	0.07	0.02	0.21	0.32	0.31
OsR498G0407886600.01	0.013	1.3	6.662398	6.44E-25	7.92E-25	0	0	0.04	1.51	1.29	1.1
OsR498G0407887400.01	26.7	7.586	-1.81119	1.04E-257	3.56E-258	26.01	26.45	27.64	6.67	8	8.09
OsR498G0407896300.01	4.65	15.04	1.686951	9.82E-34	1.05E-33	4.91	4.53	4.51	13.32	17.16	14.64
OsR498G0407897300.01	87.04	18.44	-2.18609	0	0	84.03	85.71	91.38	18.59	17.88	18.85
OsR498G0407898100.01	1.99	0.923	-1.2628	1.02E-05	2.15E-05	1.78	2.27	1.92	0.91	0.78	1.08
OsR498G0407902900.01	40.43	110.5	1.318513	0	0	41.1	38.31	41.88	109.58	112.11	109.81
OsR498G0407905000.01	1.1	0.186	-2.55928	3.31E-16	4.87E-16	1.14	1.3	0.86	0.13	0.2	0.23
OsR498G0407906700.01	2.56	0.29	-3.13449	6.22E-59	5.13E-59	2.75	2.22	2.71	0.32	0.24	0.31
OsR498G0407907100.01	2.753	0.53	-2.38564	7.84E-32	8.63E-32	2.27	2.78	3.21	0.68	0.52	0.39
OsR498G0407921500.01	6.28	3.106	-1.01965	5.22E-28	6.07E-28	6.28	6.21	6.35	2.96	3.1	3.26
OsR498G0407932400.01	324.43	11.71	-4.80192	0	0	336.3	323.73	313.3	11.42	11.42	12.29
OsR498G0407946500.01	1.323	10.26	2.936382	1.21E-59	9.88E-60	1.43	1.15	1.39	9.61	10.76	10.41
OsR498G0407946700.01	5.28	27.08	2.355971	0	0	5.36	5.47	5.01	26.7	27.72	26.82
OsR498G0407949900.01	34.863	104.62	1.58096	0	0	36.56	33.71	34.32	116.24	98.35	99.27
OsR498G0407951200.01	1.026	9.176	3.169373	1.04E-195	4.33E-196	0.8	1.19	1.09	8.76	9.93	8.84
OsR498G0407961500.01	10.483	3.083	-1.77283	1.20E-60	9.74E-61	10.77	10.72	9.96	2.66	2.9	3.69
OsR498G0407974700.01	13.85	29.016	1.090195	1.54E-187	6.59E-188	13.69	14.08	13.78	28.53	30.12	28.4
OsR498G0407993000.01	0.063	3.386	5.787953	3.33E-53	2.89E-53	0.14	0.05	0	3.81	3.17	3.18
OsR498G0407995000.01	7.62	21.253	1.429301	3.46E-147	1.71E-147	8.44	6.62	7.8	21.42	21.92	20.42
OsR498G0408009100.01	0.57	0.14	-2.01469	1.92E-07	3.71E-07	0.38	0.53	0.8	0.22	0.1	0.1
OsR498G0408011300.01	0.166	0.616	1.897741	0.0001258	0.0002831	0.19	0.13	0.18	0.76	0.58	0.51
OsR498G0408011600.01	21.793	10.416	-1.06137	8.89E-49	8.07E-49	22.34	21.14	21.9	10.27	10.5	10.48
OsR498G0408012200.01	26.613	13.066	-1.02042	8.06E-143	4.05E-143	26.15	26.31	27.38	12.75	13.58	12.87
OsR498G0408014500.01	70.286	203.72	1.51249	0	0	65.61	75.32	69.93	195.39	208.59	207.19
OsR498G0408019300.01	11.803	28.23	1.305444	2.58E-186	1.10E-186	12.22	11.99	11.2	28.49	29.7	26.5
OsR498G0408023500.01	636.66	204.94	-1.64067	0	0	692.59	595.6	621.8	207.33	207.56	199.93
OsR498G0408028500.01	7.303	17.363	1.243418	1.34E-88	8.84E-89	6.17	8.82	6.92	18.98	16.95	16.16
OsR498G0408030600.01	2.683	0.436	-2.60006	8.31E-40	8.28E-40	3	2.56	2.49	0.48	0.31	0.52
OsR498G0408031700.01	10.546	0.25	-5.4087	5.87E-123	3.22E-123	10.35	10.36	10.93	0.31	0.13	0.31
OsR498G0408034000.01	0.183	1.47	3.012993	4.48E-47	4.14E-47	0.19	0.17	0.19	1.26	1.58	1.57

OsR498G0408037100.01	0.333	0.02	-3.96024	4.73E-05	0.0001035	0.37	0.51	0.12	0	0.06	0
OsR498G0408048000.01	0.03	0.253	3.146611	8.38E-05	0.0001864	0	0.09	0	0.48	0.01	0.27
OsR498G0408050900.01	0.183	0.566	1.686989	9.46E-06	1.99E-05	0.26	0.06	0.23	0.55	0.64	0.51
OsR498G0408075800.01	0.903	0.336	-1.2806	8.84E-06	1.86E-05	0.9	1.39	0.42	0.29	0.39	0.33
OsR498G0408081100.01	4.363	11.413	1.327407	3.96E-105	2.37E-105	3.91	4.27	4.91	11.55	11.34	11.35
OsR498G0408082700.01	7.153	21.8	1.612531	0	0	5.92	8.99	6.55	21.36	21.7	22.34
OsR498G0408115100.01	0.3	0.04	-3.14984	2.13E-07	4.13E-07	0.29	0.2	0.41	0.12	0	0
OsR498G0408142400.01	109.32	12.503	-3.13601	0	0	109.99	108.5	109.5	13.54	13.06	10.91
OsR498G0408147600.01	13.333	5.203	-1.43514	4.98E-58	4.14E-58	15.18	12.34	12.48	5.42	4.89	5.3
OsR498G0408168600.01	6.376	1.13	-2.4635	2.22E-86	1.48E-86	6.7	6.02	6.41	0.92	1.2	1.27
OsR498G0408169700.01	0.516	1.62	1.572845	1.73E-10	2.97E-10	0.52	0.56	0.47	1.83	1.65	1.38
OsR498G0408170700.01	0.616	1.306	1.094243	0.0001795	0.000408	1.01	0.6	0.24	1.18	1.38	1.36
OsR498G0408171100.01	116.65	321.95	1.444587	0	0	118.85	112.24	118.9	317.28	323.7	324.86
OsR498G0408175300.01	4.49	11.41	1.359596	5.27E-44	5.04E-44	4.48	4.29	4.7	12.53	11.11	10.59
OsR498G0408175900.01	0.21	2.623	3.672028	4.21E-39	4.23E-39	0.33	0.26	0.04	2.49	2.75	2.63
OsR498G0408177400.01	0.543	2.076	2.094208	4.16E-20	5.61E-20	0.68	0.38	0.57	2.2	1.95	2.08
OsR498G0408197000.01	4.906	0.27	-4.20154	1.65E-139	8.41E-140	4.67	4.99	5.06	0.26	0.24	0.31
OsR498G0408211500.01	4.01	0.02	-7.7514	3.03E-95	1.91E-95	4.25	4.01	3.77	0.04	0	0.02
OsR498G0408211800.01	0.486	18.333	5.219695	0	0	0.32	0.64	0.5	19.03	18.95	17.02
OsR498G0408212000.01	1.89	65.836	5.087164	0	0	1.85	1.54	2.28	63.33	66.74	67.44
OsR498G0408216300.01	1.64	8.783	2.408405	2.80E-56	2.36E-56	1.99	1.56	1.37	9.7	8.25	8.4
OsR498G0408220300.01	3.726	1.66	-1.22327	2.97E-41	2.92E-41	3.71	3.66	3.81	1.71	1.66	1.61
OsR498G0408220900.01	1.393	0.63	-1.20715	1.18E-10	2.02E-10	1.63	1.16	1.39	0.67	0.52	0.7
OsR498G0408221600.01	1.973	0.91	-1.23109	1.75E-07	3.38E-07	2	1.99	1.93	0.9	0.79	1.04
OsR498G0408227200.01	12.05	4.513	-1.4224	1.04E-152	5.03E-153	11.28	12.52	12.35	4.55	4.21	4.78
OsR498G0408236200.01	15.386	1.326	-3.52858	0	0	14.97	15.3	15.89	1.57	1.01	1.4
OsR498G0408238800.01	0.243	1.333	2.406542	6.14E-15	9.30E-15	0.27	0.16	0.3	1.42	1.45	1.13
OsR498G0408240200.01	10.83	5.093	-1.1102	3.47E-12	5.68E-12	8.84	10.68	12.97	6.32	3.8	5.16
OsR498G0408253100.01	0.386	0.1	-1.79674	2.89E-06	5.90E-06	0.25	0.49	0.42	0.17	0.04	0.09
OsR498G0408266700.01	0.453	0	-5.91444	4.87E-09	8.81E-09	0.57	0.37	0.42	0	0	0
OsR498G0408272500.01	0.543	0.016	-4.96024	4.08E-09	7.36E-09	0.4	0.74	0.49	0.05	0	0
OsR498G0408312400.01	30.42	0.33	-6.53641	3.65E-263	1.23E-263	28.45	32.34	30.47	0.41	0	0.58
OsR498G0408332200.01	10.47	4.756	-1.1438	2.56E-37	2.62E-37	10.72	10.61	10.08	4.85	5.45	3.97
OsR498G0408332800.01	0.893	0.05	-4.18263	1.01E-13	1.57E-13	1.24	0.7	0.74	0	0.15	0
OsR498G0408336800.01	0.366	2.886	2.970497	2.90E-22	3.74E-22	0.41	0.29	0.4	3.22	2.66	2.78
OsR498G0408338800.01	0.32	0.673	1.075384	3.07E-05	6.64E-05	0.26	0.42	0.28	0.63	0.75	0.64
OsR498G0408339000.01	8.21	25.866	1.645481	2.70E-166	1.24E-166	7.79	7.68	9.16	25.53	25.96	26.11
OsR498G0408339100.01	1.666	18.626	3.491154	0	0	1.2	1.59	2.21	18.87	17.97	19.04
OsR498G0408346300.01	0.783	0.22	-1.84777	3.28E-05	7.12E-05	0.76	0.73	0.86	0.13	0.33	0.2
OsR498G0408348400.01	0.533	1.136	1.157277	1.85E-06	3.75E-06	0.52	0.58	0.5	1.52	1.07	0.82
OsR498G0408358300.01	20.366	139.39	2.720629	0	0	21.3	19.38	20.42	147.39	143.1	127.67
OsR498G0408359900.01	2.66	1.186	-1.14466	6.08E-11	1.03E-10	3.14	2.51	2.33	1.27	1.03	1.26
OsR498G0408366000.01	0.246	0.026	-3.13016	7.72E-05	0.0001712	0.16	0.26	0.32	0	0.04	0.04
OsR498G0408371100.01	0.156	0.05	-1.65315	0.000895	0.0021451	0.11	0.15	0.21	0.11	0.02	0.02
OsR498G0408382700.01	1.893	8.79	2.212582	8.55E-212	3.37E-212	1.48	2.07	2.13	8.57	8.82	8.98
OsR498G0408384700.01	6.39	2.293	-1.48867	3.42E-75	2.46E-75	6.54	6.19	6.44	2.31	2.07	2.5
OsR498G0408386100.01	4.756	24.77	2.37608	0	0	5.21	4.55	4.51	23.47	25.39	25.45
OsR498G0408386800.01	87.706	31.743	-1.49121	0	0	86.51	89.53	87.08	30.59	31.75	32.89
OsR498G0408388100.01	0.383	0.046	-2.86713	0.0004299	0.0010056	0.31	0.24	0.6	0	0.08	0.06
OsR498G0408397700.01	31.16	12.81	-1.28507	1.78E-137	9.16E-138	33.24	30.85	29.39	14.1	12.05	12.28
OsR498G0408401400.01	4.073	0.733	-2.37205	7.84E-59	6.47E-59	4.05	3.79	4.38	0.77	0.77	0.66
OsR498G0408412400.01	0.14	2.983	4.419532	4.29E-53	3.73E-53	0.11	0.16	0.15	3.69	2.9	2.36
OsR498G0408414800.01	0.093	0	-4.52218	0.0002393	0.0005497	0	0	0.28	0	0	0
OsR498G0408420100.01	58.546	21.58	-1.50512	1.46E-247	5.16E-248	62.26	57.23	56.15	21.82	23.14	19.78
OsR498G0408424300.01	25.043	4.613	-2.4566	4.48E-209	1.78E-209	24.2	26.95	23.98	5.26	4.81	3.77
OsR498G0408424900.01	25.076	4.816	-2.38902	5.03E-214	1.97E-214	24.68	24.56	25.99	4.9	5.11	4.44
OsR498G0408425200.01	7.17	0.583	-3.64206	1.42E-46	1.32E-46	9.66	5.48	6.37	0.48	0.49	0.78
OsR498G0408425900.01	9.13	0.32	-4.8451	3.58E-75	2.58E-75	8.97	9.43	8.99	0.17	0.09	0.7
OsR498G0408429300.01	3.403	0.896	-1.9399	1.09E-50	9.72E-51	3.32	3.32	3.57	0.81	1.17	0.71
OsR498G0408437300.01	72.723	171.38	1.239191	0	0	71.21	75	71.96	166.93	173.46	173.75
OsR498G0408441700.01	1.216	6.556	2.401344	3.78E-85	2.55E-85	1.4	1.09	1.16	6.35	6.69	6.63
OsR498G0408448900.01	0.053	2.94	5.930531	2.37E-44	2.26E-44	0.16	0	0	2.58	2.51	3.73
OsR498G0408449100.01	29.49	68.03	1.199979	0	0	29.14	34.39	24.94	65.63	72.15	66.31
OsR498G0408450800.01	0.266	0	-4.88624	3.23E-05	6.99E-05	0.59	0	0.21	0	0	0
OsR498G0408451300.01	16.376	72.896	2.156291	0	0	16.45	16.74	15.94	71.36	73.21	74.12
OsR498G0408451500.01	4.44	0.246	-4.09327	3.18E-42	3.10E-42	4.74	4.67	3.91	0.28	0.29	0.17
OsR498G0408452400.01	1.076	8.396	3.026256	1.82E-218	7.02E-219	0.96	1.02	1.25	7.73	8.66	8.8
OsR498G0408455300.01	0.063	1.463	4.454798	2.97E-16	4.36E-16	0.13	0	0.06	1.62	1.85	0.92
OsR498G0408462600.01	3.64	1.276	-1.5173	7.62E-20	1.03E-19	3.35	3.96	3.61	1.04	1.07	1.72

OsR498G0408463500.01	20.933	9.87	-1.10975	3.03E-35	3.19E-35	21.6	20.11	21.09	9.45	10.74	9.42
OsR498G0408465300.01	0.256	3.416	3.744304	1.54E-144	7.67E-145	0.28	0.31	0.18	3.39	3.38	3.48
OsR498G0408469200.01	1.84	0.14	-3.76383	1.29E-21	1.69E-21	2.24	1.26	2.02	0.1	0.15	0.17
OsR498G0408469400.01	8.91	3.676	-1.25697	1.19E-42	1.15E-42	9.99	9.45	7.29	3.87	3.45	3.71
OsR498G0408470900.01	0.55	22.2	4.833899	1.15E-303	3.49E-304	0.55	0.62	0.48	23.61	21.22	21.77
OsR498G0408473800.01	9.993	0.026	-8.66414	6.44E-82	4.43E-82	8.48	12.13	9.37	0	0.08	0
OsR498G0408477200.01	0.196	0	-4.66068	0.0001179	0.0002647	0.23	0	0.36	0	0	0
OsR498G0408481900.01	2.69	79.226	4.909963	0	0	2.51	2.78	2.78	81.1	78.35	78.23
OsR498G0408484600.01	0.046	0.28	2.624723	8.71E-10	1.54E-09	0.06	0.05	0.03	0.29	0.24	0.31
OsR498G0408485400.01	10.633	27.356	1.359287	1.98E-148	9.73E-149	9.55	11.53	10.82	27.61	27.42	27.04
OsR498G0408488100.01	4.876	10.303	1.069161	2.16E-16	3.16E-16	5.13	5.21	4.29	9.74	10.63	10.54
OsR498G0408492100.01	8.31	2.78	-1.5814	5.57E-53	4.84E-53	8.24	8.79	7.9	3.2	2.62	2.52
OsR498G0408494100.01	1.166	2.293	1.00033	9.13E-17	1.32E-16	1.02	1.18	1.3	2.02	2.48	2.38
OsR498G0408501100.01	76.686	184.38	1.223865	0	0	73.64	78.71	77.71	177.15	188.01	187.98
OsR498G0408504600.01	0.326	0.06	-2.44567	1.75E-05	3.73E-05	0.38	0.4	0.2	0.11	0.07	0
OsR498G0408507000.01	9.86	20.423	1.035709	3.11E-44	2.97E-44	9.93	10.45	9.2	19.54	19.58	22.15
OsR498G0408511100.01	1.336	10.743	3.005146	1.15E-154	5.51E-155	1.17	1.44	1.4	11.07	10.34	10.82
OsR498G0408517000.01	34.216	14.746	-1.22579	1.99E-77	1.41E-77	36.69	32.49	33.47	14.17	14.3	15.77
OsR498G0408518200.01	91.2	31.756	-1.53618	0	0	90.11	90.8	92.69	30.37	31.71	33.19
OsR498G0408520800.01	0.01	0.166	3.946651	6.37E-05	0.0001407	0	0.03	0	0.23	0.07	0.2
OsR498G0408524500.01	1.57	0.31	-2.35372	2.63E-18	3.69E-18	1.36	1.55	1.8	0.43	0.32	0.18
OsR498G0408525300.01	40	110.5	1.457984	0	0	39.74	38.68	41.58	112.66	109.41	109.44
OsR498G0408526800.01	4.703	1.723	-1.32627	3.19E-09	5.73E-09	4.07	5.03	5.01	1.05	1.7	2.42
OsR498G0408527700.01	104.66	19.803	-2.40706	0	0	104.13	106.19	103.7	20.23	19.34	19.84
OsR498G0408529000.01	2.45	1.093	-1.09114	1.46E-11	2.43E-11	2.2	2.56	2.59	1.27	1.06	0.95
OsR498G0408529300.01	1.01	0.033	-4.91444	7.22E-09	1.32E-08	1.26	1.02	0.75	0.1	0	0
OsR498G0408532700.01	0.72	1.613	1.10277	5.85E-07	1.16E-06	0.67	0.7	0.79	1.76	1.07	2.01
OsR498G0408535900.01	6.223	15.66	1.330241	8.70E-41	8.59E-41	5.57	6.92	6.18	16.3	15.29	15.39
OsR498G0408536200.01	0.063	0.503	3.03976	2.77E-07	5.40E-07	0.05	0.05	0.09	0.28	0.9	0.33
OsR498G0408537300.01	1809.1	371.87	-2.29044	0	0	1872.8	1801.4	1753	397.12	351.34	367.16
OsR498G0408540100.01	6.54	13.583	1.048373	4.23E-29	4.84E-29	7.42	6.5	5.7	13.24	12.17	15.34
OsR498G0408541500.01	3.866	1.243	-1.65212	7.28E-12	1.20E-11	4.04	3.57	3.99	1.32	0.68	1.73
OsR498G0408543100.01	0	0.406	5.127223	8.48E-06	1.78E-05	0	0	0	0.28	0.3	0.64
OsR498G0408544300.01	0.256	0.52	1.003234	0.0001062	0.0002377	0.29	0.16	0.32	0.37	0.63	0.56
OsR498G0408545200.01	3.183	0.243	-3.70274	5.82E-36	6.08E-36	3.44	3.1	3.01	0.33	0.17	0.23
OsR498G0408545900.01	2.923	71.69	4.601946	0	0	3.34	2.9	2.53	69.74	75.81	69.52
OsR498G0408546200.01	0	0.186	5.209685	4.72E-06	9.74E-06	0	0	0	0.18	0.16	0.22
OsR498G0408550800.01	0.836	0.14	-2.58319	4.52E-05	9.88E-05	0.36	1.03	1.12	0	0.11	0.31
OsR498G0408553000.01	0.606	0.033	-4.20817	7.92E-06	1.66E-05	0.57	0.7	0.55	0	0.1	0
OsR498G0408554900.01	2.256	11.153	2.501728	9.86E-121	5.47E-121	2.41	1.8	2.56	10.66	11.42	11.38
OsR498G0408558000.01	0	0.633	5.993956	3.10E-09	5.56E-09	0	0	0	0.66	0.93	0.31
OsR498G0408560500.01	59.316	8.85	-2.73474	0	0	57.4	61.28	59.27	8.9	8.66	8.99
OsR498G0408561300.01	19.573	0.746	-4.85831	0	0	18.57	20.1	20.05	0.62	1.05	0.57
OsR498G0408562300.01	0.453	0.01	-5.76759	6.90E-15	1.05E-14	0.56	0.43	0.37	0	0.03	0
OsR498G0408562900.01	7.633	1.213	-2.6571	2.65E-147	1.31E-147	7.34	7.91	7.65	0.98	1.07	1.59
OsR498G0408563300.01	6.403	1.276	-2.32971	9.71E-129	5.18E-129	6.34	6.36	6.51	1.24	1.42	1.17
OsR498G0408564200.01	0.546	0.236	-1.2031	2.86E-05	6.17E-05	0.6	0.48	0.56	0.21	0.24	0.26
OsR498G0408565900.01	42.4	17.54	-1.37753	2.10E-234	7.73E-235	42.85	41.89	42.46	15.83	18.58	18.21
OsR498G0408569000.01	2.166	0.693	-1.65731	2.20E-10	3.80E-10	2.07	2.43	2	0.93	0.39	0.76
OsR498G0408569200.01	56.83	6.033	-3.21912	0	0	56.78	56.62	57.09	5.94	5.97	6.19
OsR498G0408584200.01	0.016	0.533	5.069507	6.42E-25	7.88E-25	0.03	0.02	0	0.51	0.53	0.56
OsR498G0408584400.01	9.336	79.023	3.039744	0	0	8.99	9.95	9.07	76.68	81.9	78.49
OsR498G0408584900.01	7.286	36.043	2.331208	0	0	7.34	7.65	6.87	35.4	35.8	36.93
OsR498G0408585100.01	6.103	2.906	-1.1091	3.33E-06	6.82E-06	5.93	4.57	7.81	2.45	2.47	3.8
OsR498G0408586000.01	8.36	3.03	-1.45869	5.69E-49	5.15E-49	8.29	9.05	7.74	3.47	3.03	2.59
OsR498G0408587500.01	1.026	6.71	2.677886	1.50E-105	8.94E-106	1.01	0.96	1.11	7.16	5.9	7.07
OsR498G0408588300.01	0	0.093	4.741309	9.24E-05	0.0002061	0	0	0	0.14	0	0.14
OsR498G0408590400.01	12.296	5.47	-1.17325	1.46E-24	1.80E-24	12.33	13.76	10.8	6.39	5.79	4.23
OsR498G0408591800.01	1.17	0.64	-1.05345	0.0004769	0.0011188	1.25	1.29	0.97	0.6	0.77	0.55
OsR498G0408601300.01	1.12	0.24	-2.2557	2.58E-08	4.81E-08	1.12	0.85	1.39	0.26	0.13	0.33
OsR498G0408602600.01	21.866	60.183	1.455326	0	0	23.2	22.07	20.33	57.74	62.61	60.2
OsR498G0408605800.01	3.363	0.233	-3.90471	9.72E-152	4.71E-152	3.23	3.43	3.43	0.2	0.28	0.22
OsR498G0408606000.01	85.55	8.723	-3.30096	0	0	86.61	82.65	87.39	7.08	10.18	8.91
OsR498G0408606300.01	1.8	0.39	-2.25147	5.26E-13	8.37E-13	1.72	1.94	1.74	0.42	0.49	0.26
OsR498G0408606900.01	0.956	35.616	5.215018	0	0	0.87	0.97	1.03	37.37	36.65	32.83
OsR498G0408608400.01	7.67	24.13	1.655529	4.12E-141	2.09E-141	8.22	6.97	7.82	25.88	22.61	23.9
OsR498G0408612600.01	0.463	1.043	1.175415	1.91E-09	3.41E-09	0.33	0.57	0.49	1.12	0.9	1.11
OsR498G0408613900.01	0.01	0.293	4.847115	2.49E-08	4.62E-08	0	0	0.03	0.37	0.26	0.25
OsR498G0408615000.01	37.746	76.513	1.016689	0	0	37.44	39.1	36.7	75.4	76.37	77.77

OsR498G0408617400.01	1.03	0.233	-2.14997	1.74E-12	2.81E-12	1.21	0.93	0.95	0.12	0.24	0.34
OsR498G0408621300.01	36.53	78.083	1.093854	0	0	32.77	38.1	38.72	77.19	79.16	77.9
OsR498G0408622600.01	0.02	0.166	3.098654	3.81E-06	7.84E-06	0.04	0	0.02	0.16	0.2	0.14
OsR498G0408625400.01	0.776	0.21	-1.89514	4.53E-08	8.52E-08	0.7	0.73	0.9	0.15	0.18	0.3
OsR498G0408631200.01	32.42	15.093	-1.10057	5.77E-176	2.56E-176	32.43	32.98	31.85	15.02	15.24	15.02
OsR498G0408632900.01	38.96	18.553	-1.07834	1.83E-266	6.12E-267	36.39	39.79	40.7	18.05	18.97	18.64
OsR498G0408633300.01	16.82	38.56	1.19304	3.24E-183	1.41E-183	16.04	17.48	16.94	38.93	38.51	38.24
OsR498G0408633800.01	1.776	1.743	-1.31949	1.37E-09	2.44E-09	1.73	1.86	1.74	1.24	2.63	1.36
OsR498G0408637300.01	9.026	1.476	-2.63984	1.59E-110	9.23E-111	9.11	8.98	8.99	1.24	1.79	1.4
OsR498G0408638200.01	0.996	0.463	-1.10708	0.0002019	0.0004606	0.96	1.06	0.97	0.59	0.35	0.45
OsR498G0408645400.01	21.91	2.656	-3.06529	0	0	21.89	21.34	22.5	2.76	2.48	2.73
OsR498G0408648400.01	0	0.35	5.563322	2.64E-07	5.13E-07	0	0	0	0.18	0.37	0.5
OsR498G0408649100.01	40.48	9.653	-2.05146	6.68E-278	2.17E-278	42.06	38.73	40.65	9.33	9.94	9.69
OsR498G0408650500.01	1.683	0.806	-1.04597	7.08E-06	1.48E-05	1.41	1.73	1.91	1.02	0.46	0.94
OsR498G0408655500.01	1.793	7.676	2.078898	2.31E-93	1.48E-93	1.9	2.08	1.4	7.59	7.92	7.52
OsR498G0408658500.01	1.82	21.173	3.537845	0	0	1.58	2.03	1.85	21.72	21.6	20.2
OsR498G0408659900.01	0.81	0.393	-1.33875	8.72E-05	0.0001942	0.85	0.78	0.8	0.35	0.3	0.53
OsR498G0408660100.01	3.58	238.99	6.050335	0	0	3.61	3.13	4	242.71	243.7	230.57
OsR498G0408661900.01	1.66	0.246	-2.75775	6.74E-24	8.44E-24	1.98	1.32	1.68	0.24	0.32	0.18
OsR498G0408662700.01	2.443	7.16	1.544723	1.71E-52	1.49E-52	2.49	2.26	2.58	7.64	6.71	7.13
OsR498G0408664300.01	321.73	87.976	-1.87671	0	0	324.18	314	327	82.15	88.45	93.33
OsR498G0408664500.01	2.98	1.383	-1.12152	5.31E-11	8.99E-11	3	2.74	3.2	1.05	1.76	1.34
OsR498G0408665600.01	1.576	16.443	3.368298	0	0	1.41	1.45	1.87	14.33	16.63	18.37
OsR498G0408668500.01	0.323	2.086	2.669117	1.06E-16	1.54E-16	0.3	0.32	0.35	1.86	2.56	1.84
OsR498G0408669700.01	441.2	1444.2	1.702651	0	0	450.96	438.74	433.9	1453	1429.4	1450.1
OsR498G0408670700.01	2.336	9.933	2.280932	1.11E-101	6.79E-102	2.17	1.91	2.93	10.24	10.64	8.92
OsR498G0408670900.01	0.856	2.176	1.298736	5.18E-14	8.05E-14	0.72	1.01	0.84	1.81	2.36	2.36
OsR498G0408671100.01	0.53	17.153	5.010482	2.87E-207	1.15E-207	0.68	0.36	0.55	18.44	16.89	16.13
OsR498G0408672700.01	0.94	4.873	2.365804	1.01E-47	9.30E-48	0.76	0.89	1.17	4.95	4.4	5.27
OsR498G0408673300.01	4.463	1.5	-1.53515	1.08E-42	1.04E-42	4.53	4.28	4.58	1.24	1.56	1.7
OsR498G0408673500.01	0.396	0.943	1.257793	1.83E-06	3.71E-06	0.16	0.58	0.45	0.91	0.69	1.23
OsR498G0408673700.01	0.42	3.31	2.976566	1.02E-46	9.48E-47	0.39	0.56	0.31	2.83	3.01	4.09
OsR498G0408675200.01	11.696	1.15	-3.35746	2.12E-55	1.81E-55	11.42	13.78	9.89	0.97	1.49	0.99
OsR498G0408675800.01	34.043	168.72	2.292417	0	0	35.22	33.74	33.17	164.81	171.15	170.21
OsR498G0408676900.01	65.213	26.77	-1.34946	0	0	63.07	66.28	66.29	25.79	26.93	27.59
OsR498G0408678300.01	0.403	0.156	-1.37528	0.0003652	0.0008492	0.45	0.44	0.32	0.15	0.19	0.13
OsR498G0408679300.01	0.96	0.453	-1.07781	1.51E-06	3.04E-06	0.72	1.27	0.89	0.53	0.44	0.39
OsR498G0408685600.01	2.426	0.4	-2.58039	3.47E-25	4.24E-25	2.34	3	1.94	0.34	0.47	0.39
OsR498G0408685800.01	11.09	0.723	-3.95088	4.24E-96	2.66E-96	11.57	10.8	10.9	1.03	0.61	0.53
OsR498G0408688400.01	290.08	37.023	-2.97593	0	0	281.39	290.05	298.8	40.14	36.62	34.31
OsR498G0408689800.01	11.216	3.596	-1.66875	1.46E-113	8.38E-114	11.09	11.1	11.46	3.7	3.51	3.58
OsR498G0408690200.01	26.57	201.49	2.926374	0	0	26.55	27.28	25.88	197.79	201.21	205.46
OsR498G0408691700.01	1.736	0.473	-1.80046	8.40E-12	1.39E-11	1.45	1.87	1.89	0.36	0.51	0.55
OsR498G0408692700.01	0.01	0.256	4.499192	8.97E-07	1.79E-06	0	0	0.03	0.22	0.22	0.33
OsR498G0408693400.01	0.14	0	-4.76759	6.52E-05	0.0001441	0.21	0.12	0.09	0	0	0
OsR498G0408693600.01	0	0.07	4.624723	0.0001711	0.0003885	0	0	0	0.12	0.08	0.01
OsR498G0408696100.01	2098.2	611.66	-1.66257	0	0	2221.1	2039.3	2034	656.5	587.77	590.72
OsR498G0408699700.01	0.153	36.27	7.86249	0	0	0.15	0.1	0.21	37.95	36.43	34.43
OsR498G0408700100.01	0	0.246	4.499192	0.0003163	0.0007323	0	0	0	0.2	0.34	0.2
OsR498G0408701300.01	0.753	6.156	3.03976	5.00E-81	3.46E-81	0.94	0.78	0.54	6.32	5.48	6.67
OsR498G0408706800.01	1.753	0.26	-2.52502	1.37E-11	2.28E-11	1.46	1.54	2.26	0.5	0.19	0.09
OsR498G0408708600.01	11.626	26.666	1.22229	9.06E-94	5.77E-94	11.28	12.56	11.04	26.78	25.51	27.71
OsR498G0408711400.01	66.403	11.193	-2.58177	0	0	71.63	63.42	64.16	10.78	10.63	12.17
OsR498G0408711900.01	4.416	1.953	-1.18263	5.88E-17	8.49E-17	5.45	3.91	3.89	2.09	1.5	2.27
OsR498G0408712800.01	13.826	61.896	2.151921	0	0	13.69	13.57	14.22	61.97	59.92	63.8
OsR498G0408718000.01	4.693	1.933	-1.28629	7.68E-16	1.14E-15	5.81	4.06	4.21	2.56	1.43	1.81
OsR498G0408718100.01	5.02	1.183	-2.08673	5.01E-19	6.92E-19	4.16	7.57	3.33	0.63	1.8	1.12
OsR498G0408725100.01	3.39	0.863	-1.97202	1.66E-76	1.19E-76	2.74	3.9	3.53	0.92	0.85	0.82
OsR498G0408726700.01	45.753	131.97	1.529832	0	0	46.91	47.27	43.08	130.09	130.68	135.14
OsR498G0408732400.01	17.056	8.6	-1.00241	9.49E-11	1.62E-10	17.17	15.57	18.43	9.65	6.47	9.68
OsR498G0408734000.01	4.066	23.693	2.573244	2.23E-211	8.80E-212	3.07	4.6	4.53	24.37	22	24.71
OsR498G0408735400.01	16.946	247.05	3.853079	0	0	17.61	16.47	16.76	266.56	231.52	243.06
OsR498G0408737900.01	4.026	9.896	1.292901	7.62E-53	6.64E-53	3.98	4.13	3.97	8.98	10.07	10.64
OsR498G0408738100.01	10.663	5.293	-1.00523	3.20E-38	3.25E-38	11.12	9.81	11.06	3.85	5.61	6.42
OsR498G0408738400.01	0.89	0.15	-2.57064	2.34E-16	3.43E-16	0.76	1.12	0.79	0.15	0.18	0.12
OsR498G0408739900.01	19.496	64.27	1.706217	0	0	18.2	19.76	20.53	60.69	66.26	65.86
OsR498G0408741800.01	6.84	1.403	-2.26388	2.13E-77	1.51E-77	6.33	7.34	6.85	1.41	1.1	1.7
OsR498G0408743600.01	13.823	56.236	2.139296	0	0	13.87	13.36	14.24	56.26	57.64	54.81
OsR498G0408746400.01	0.126	0.006	-3.86713	8.60E-05	0.0001913	0.14	0.08	0.16	0	0	0.02

OsR498G0408748500.01	0.836	0.033	-5.13016	6.78E-35	7.18E-35	1	0.73	0.78	0.03	0.03	0.04
OsR498G0408755300.01	2.223	4.383	1.066465	6.70E-12	1.10E-11	1.87	2.6	2.2	5.67	3.91	3.57
OsR498G0408756800.01	5.02	15.89	1.65139	3.03E-135	1.57E-135	4.79	5.6	4.67	16.05	15.69	15.93
OsR498G0408758600.01	7.18	1.403	-2.46461	2.83E-173	1.26E-173	7.24	7.42	6.88	1.32	1.55	1.34
OsR498G0408759400.01	4.113	0.743	-2.47214	4.55E-43	4.39E-43	3.98	4.67	3.69	0.81	0.63	0.79
OsR498G0408760000.01	2.02	6.493	1.683616	4.73E-50	4.24E-50	1.8	2.2	2.06	6.88	6.2	6.4
OsR498G0408763700.01	0.02	0.236	3.702725	1.70E-09	3.02E-09	0.04	0	0.02	0.18	0.23	0.3
OsR498G0408765000.01	2.38	0.796	-1.5851	1.46E-26	1.74E-26	2.42	2.28	2.44	0.68	0.93	0.78
OsR498G0408765200.01	2.046	4.69	1.207798	2.57E-21	3.37E-21	2.36	1.97	1.81	4.07	5.03	4.97
OsR498G0408766900.01	3.79	1.763	-1.03793	7.57E-22	9.84E-22	4.42	3.24	3.71	1.39	1.66	2.24
OsR498G0408767400.01	8.156	25.72	1.591521	0	0	8.84	7.86	7.77	26.21	25.24	25.71
OsR498G0408770000.01	3.616	0.763	-2.25092	2.75E-18	3.86E-18	4.02	3.56	3.27	0.97	0.58	0.74
OsR498G0408770900.01	0.923	0.203	-2.21022	1.72E-22	2.21E-22	0.8	1	0.97	0.21	0.2	0.2
OsR498G0408772500.01	0.126	0.01	-3.66068	0.0002846	0.0006567	0.09	0.21	0.08	0.03	0	0
OsR498G0408773700.01	13.87	5.48	-1.34526	7.89E-54	6.81E-54	14.71	13.32	13.58	6.13	5.55	4.76
OsR498G0408774000.01	5.573	2.296	-1.28403	5.08E-23	6.46E-23	5.78	6.06	4.88	1.74	2.79	2.36
OsR498G0408776700.01	0.02	0.266	3.847115	0.0001179	0.0002648	0.06	0	0	0.34	0.17	0.29
OsR498G0408777000.01	92.863	200.02	1.101662	0	0	91.49	95.22	91.88	216.03	196.07	187.95
OsR498G0408778500.01	0.953	0.503	-1.49274	2.29E-07	4.44E-07	0.96	1.13	0.77	0.35	0.56	0.6
OsR498G0408782800.01	1.84	0.823	-1.16617	3.19E-22	4.12E-22	1.55	1.89	2.08	0.95	0.89	0.63
OsR498G0408789100.01	59.75	15.85	-1.93127	0	0	57.41	61.66	60.18	15.88	15.88	15.79
OsR498G0408789600.01	4.403	0.953	-2.25648	5.56E-42	5.43E-42	4.49	4.34	4.38	1.28	1.02	0.56
OsR498G0408790300.01	0.653	2.273	1.801149	1.12E-14	1.71E-14	0.78	0.66	0.52	2.09	2.15	2.58
OsR498G0408790600.01	11.956	27.693	1.244433	2.33E-214	9.12E-215	12.45	12.08	11.34	26.69	29.2	27.19
OsR498G0408792900.01	387.7	95.763	-2.01883	0	0	383.19	388.1	391.8	93.62	94.89	98.78
OsR498G0408793100.01	0.046	0.29	2.597755	1.50E-09	2.67E-09	0.06	0.03	0.05	0.31	0.2	0.36
OsR498G0408793600.01	0.036	5.3	7.135157	3.58E-126	1.93E-126	0.05	0.03	0.03	6.18	4.84	4.88
OsR498G0408794000.01	4.116	1.983	-1.1502	2.01E-33	2.16E-33	3.56	4.92	3.87	1.54	2.32	2.09
OsR498G0408794500.01	2.85	1.383	-1.04576	8.92E-13	1.43E-12	2.83	2.64	3.08	0.93	1.66	1.56
OsR498G0408795200.01	8.476	31.553	1.895881	0	0	8.72	8.51	8.2	34.18	29.23	31.25
OsR498G0408795300.01	0.343	6.333	4.267516	4.17E-156	1.98E-156	0	0.61	0.42	6.1	8.72	4.18
OsR498G0408795600.01	4.93	13.55	1.372866	3.87E-58	3.22E-58	5.14	5.03	4.62	14.6	13.55	12.5
OsR498G0408799300.01	0.446	1.243	1.481465	1.53E-14	2.34E-14	0.44	0.47	0.43	1.33	1.36	1.04
OsR498G0408800400.01	2.163	0.663	-1.75045	1.74E-41	1.70E-41	2.09	2.41	1.99	0.58	0.6	0.81
OsR498G0408800500.01	68.966	26.813	-1.38087	0	0	69.43	71.03	66.44	27.67	26.41	26.36
OsR498G0408800700.01	3.12	0.06	-5.73283	5.61E-40	5.58E-40	3.2	2.46	3.7	0.06	0.06	0.06
OsR498G0408802200.01	13.183	29.18	1.139832	3.03E-144	1.52E-144	13.49	12.2	13.86	29.57	29.67	28.3
OsR498G0408806900.01	0.08	0.286	1.876261	0.0003543	0.0008231	0.08	0	0.16	0.37	0.35	0.14
OsR498G0408807500.01	0.383	0	-6.91444	8.58E-16	1.27E-15	0.35	0.39	0.41	0	0	0
OsR498G0408810000.01	8.533	33.426	1.993685	0	0	8.51	9.03	8.06	32.34	32.86	35.08
OsR498G0408810900.01	2.426	5.013	1.047842	2.28E-16	3.34E-16	3.08	2.59	1.61	4.95	5.54	4.55
OsR498G0408811600.01	0.08	1.166	3.847115	0.0001179	0.0002648	0.24	0	0	1.75	0.75	1
OsR498G0408817100.01	23.39	177.64	2.925107	0	0	22.87	24.29	23.01	171.8	184.7	176.41
OsR498G0408817700.01	4.433	28.206	2.662795	5.87E-248	2.07E-248	5.09	4.25	3.96	28.96	28.55	27.11
OsR498G0408819400.01	1444	622.25	-1.21993	0	0	1471.4	1435.8	1425	647.85	621.06	597.84
OsR498G0408823400.01	58.83	29.583	-1.03625	8.85E-148	4.36E-148	59.26	58.44	58.79	29.86	29.93	28.96
OsR498G0408825500.01	1.626	0.12	-3.80154	1.97E-10	3.39E-10	1.46	2.01	1.41	0.12	0	0.24
OsR498G0408826800.01	0.25	0.783	1.646418	2.94E-07	5.72E-07	0.27	0.18	0.3	0.97	0.68	0.7
OsR498G0408830200.01	14.093	31.56	1.145842	7.96E-226	3.00E-226	12.87	14.82	14.59	30.53	32.4	31.75
OsR498G0408830600.01	2.183	6.676	1.612259	3.40E-38	3.45E-38	2.07	2.3	2.18	6.78	6.63	6.62
OsR498G0408832200.01	17.713	7.183	-1.31677	1.27E-86	8.44E-87	17.82	18.53	16.79	7.13	7.23	7.19
OsR498G0408832700.01	118.57	34.943	-1.76591	0	0	122.33	116.02	117.4	38.15	34.82	31.86
OsR498G0408832900.01	7.13	3.053	-1.24215	3.02E-43	2.91E-43	6.84	7.46	7.09	3.06	2.82	3.28
OsR498G0408834200.01	16.64	7.696	-1.16292	1.58E-41	1.54E-41	16.7	18.19	15.03	7.55	8.58	6.96
OsR498G0408834700.01	2.926	1.363	-1.09951	9.27E-16	1.38E-15	2.94	2.87	2.97	1.34	1.32	1.43
OsR498G0408837100.01	19	9.74	-1.00261	2.08E-47	1.91E-47	18.3	19.26	19.44	8.88	10.56	9.78
OsR498G0408838100.01	0.36	0	-4.66068	0.0001179	0.0002647	0.33	0.35	0.4	0	0	0
OsR498G0408838500.01	0	0.853	6.084154	1.05E-09	1.85E-09	0	0	0	0.61	0.86	1.09
OsR498G0408839800.01	0.16	26.493	7.24813	7.52E-302	2.29E-302	0.17	0.31	0	26.72	26.41	26.35
OsR498G0408841900.01	1.62	0.53	-1.61873	2.77E-14	4.26E-14	1.96	1.36	1.54	0.61	0.54	0.44
OsR498G0408846900.01	50.143	124.81	1.298675	0	0	52.13	47.03	51.27	122.75	125.49	126.18
OsR498G0408848300.01	12.47	51.883	2.07376	0	0	12.95	12.45	12.01	48.24	54.86	52.55
OsR498G0408852600.01	29.98	14.056	-1.09529	2.74E-32	2.99E-32	24.93	31.89	33.12	16.81	12.35	13.01
OsR498G0408852800.01	35.956	85.546	1.230304	0	0	36.58	34.78	36.51	84.69	87.28	84.67
OsR498G0408857000.01	3.356	13.856	2.072562	2.04E-307	6.12E-308	3.02	3.5	3.55	12.93	14.24	14.4
OsR498G0408860500.01	0.04	0.93	4.520887	4.99E-17	7.21E-17	0	0	0.12	1.23	0.85	0.71
OsR498G0408861800.01	1.22	4.913	2.061738	7.71E-104	4.66E-104	1.14	1.32	1.2	4.75	4.95	5.04
OsR498G0408864800.01	14.226	3.913	-1.88901	1.09E-71	8.04E-72	15.21	13.34	14.13	4.55	2.99	4.2
OsR498G0408869400.01	6.05	22.096	1.86788	0	0	6.34	6.2	5.61	22.32	21.78	22.19

OsR498G0408877400.01	3.876	1.736	-1.166	2.02E-17	2.89E-17	4.36	3.41	3.86	1.69	1.78	1.74
OsR498G0408878400.01	0.193	11.366	5.874758	3.62E-165	1.66E-165	0.19	0.25	0.14	10.1	12.37	11.63
OsR498G0408881200.01	0.426	2	2.224185	3.34E-32	3.66E-32	0.42	0.32	0.54	1.71	2.05	2.24
OsR498G0408883400.01	2.526	9.033	1.808838	5.99E-100	3.69E-100	2.94	1.97	2.67	9.65	8.18	9.27
OsR498G0408883800.01	1.853	0.186	-3.25792	3.40E-33	3.67E-33	1.96	2.04	1.56	0.29	0.17	0.1
OsR498G0408884400.01	2.973	0.286	-3.37838	2.08E-58	1.72E-58	2.98	3.38	2.56	0.2	0.24	0.42
OsR498G0408885400.01	1.39	2.82	1.024547	6.63E-13	1.06E-12	1.35	1.39	1.43	2.23	2.43	3.8
OsR498G0408886300.01	53.61	19.22	-1.4901	0	0	56.61	52.73	51.49	20.29	18.62	18.75
OsR498G0408886600.01	1.273	220.27	7.434202	0	0	1.01	1.56	1.25	215.99	220.85	223.96
OsR498G0408886800.01	0	2.023	8.406082	2.00E-35	2.10E-35	0	0	0	2.02	2.09	1.96
OsR498G0408888200.01	0.913	0.406	-1.16669	5.19E-06	1.07E-05	1.15	0.88	0.71	0.46	0.44	0.32
OsR498G0408897800.01	58.41	340.07	2.540554	0	0	60.2	59.52	55.51	344.64	336.71	338.87
OsR498G0408898700.01	0.6	2.816	2.225115	5.38E-19	7.44E-19	0.64	0.55	0.61	3.36	2.62	2.47
OsR498G0408899800.01	0.71	35.613	5.517022	0	0	0.81	0.57	0.75	37.26	34.5	35.08
OsR498G0408901100.01	19.313	41.79	1.10992	3.25E-197	1.34E-197	19.53	19.35	19.06	41.42	41.74	42.21
OsR498G0408906400.01	4.726	10.633	1.165822	4.45E-73	3.26E-73	4.64	4.71	4.83	10.48	11.05	10.37
OsR498G0408907700.01	5.236	0.023	-7.13016	9.08E-34	9.75E-34	5.36	4.33	6.02	0	0	0.07
OsR498G0408911500.01	1.06	3.853	1.870152	8.21E-51	7.29E-51	1	1.12	1.06	4.3	3.66	3.6
OsR498G0408913200.01	11.86	27.373	1.200664	1.70E-254	5.91E-255	11.69	11.7	12.19	25.19	28.62	28.31
OsR498G0408917900.01	3.316	6.856	1.027787	2.87E-11	4.82E-11	4.03	2.34	3.58	6.67	7.02	6.88
OsR498G0408919400.01	0.396	29.836	6.232988	0	0	0.65	0.38	0.16	29.48	30.23	29.8
OsR498G0408921900.01	1.85	0.3	-2.63597	1.49E-24	1.84E-24	2.28	1.25	2.02	0.42	0.17	0.31
OsR498G0408922200.01	10.03	3.263	-1.8457	1.41E-92	9.08E-93	9.26	10.52	10.31	2.27	3.34	4.18
OsR498G0408922800.01	0.156	0.006	-4.28217	2.00E-10	3.45E-10	0.15	0.17	0.15	0	0	0.02
OsR498G0408923500.01	0.046	9.593	7.906421	5.17E-251	1.81E-251	0.07	0.04	0.03	9.89	9.66	9.23
OsR498G0408924200.01	1.083	2.443	1.182204	2.25E-13	3.54E-13	0.79	1.46	1	2.74	2.07	2.52
OsR498G0408924600.01	0.74	20.816	4.814747	0	0	0.7	0.84	0.68	21.74	21.11	19.6
OsR498G0408927000.01	10.483	100.57	3.248913	0	0	8.75	11.56	11.14	101.81	101.88	98.01
OsR498G0408929200.01	0.5	0.04	-4.6041	6.01E-13	9.58E-13	0.42	0.47	0.61	0.09	0	0.03
OsR498G0408931100.01	6.226	2.113	-1.5681	6.24E-30	7.05E-30	5.81	6.55	6.32	2.45	1.95	1.94
OsR498G0408932400.01	8.793	1.016	-3.12347	1.66E-38	1.68E-38	7.78	10.26	8.34	1.13	1.03	0.89
OsR498G0408932500.01	44.87	12.47	-1.86755	0	0	46.71	42.64	45.26	12.28	11.75	13.38
OsR498G0408935400.01	0.57	1.283	1.194088	3.18E-05	6.88E-05	0.48	0.45	0.78	1.15	1.08	1.62
OsR498G0408935800.01	1.3	0.686	-1.07888	6.61E-05	0.000146	1.38	1.29	1.23	0.75	0.78	0.53
OsR498G0408938700.01	19.306	86.473	2.230259	0	0	18.95	19.16	19.81	81.15	88.7	89.57
OsR498G0408939100.01	10.666	55.2	2.351368	0	0	11.23	11.67	9.1	56.71	51.86	57.03
OsR498G0408941200.01	1.063	2.196	1.024379	7.35E-09	1.34E-08	0.87	1.36	0.96	2.73	1.95	1.91
OsR498G0408941400.01	2.173	0.763	-1.52318	2.17E-06	4.41E-06	1.59	2.52	2.41	0.62	0.73	0.94
OsR498G0408944800.01	10.303	2.67	-1.9569	4.71E-45	4.45E-45	10.51	9.84	10.56	3.59	1.96	2.46
OsR498G0408948900.01	11.13	32.866	1.552099	0	0	10.71	11.3	11.38	33.29	32.35	32.96
OsR498G0408950400.01	3.226	42.89	3.810016	0	0	3.29	3.09	3.3	43.88	41.97	42.82
OsR498G0408953600.01	40.933	14.85	-1.46954	3.80E-132	2.00E-132	40.31	43.85	38.64	14.58	15.16	14.81
OsR498G0408954500.01	0.283	0	-5.81822	1.42E-08	2.62E-08	0.27	0.06	0.52	0	0	0
OsR498G0408955300.01	2.59	1.016	-1.30914	2.02E-09	3.60E-09	2.92	2.93	1.92	1.24	1.15	0.66
OsR498G0408956200.01	2.196	6.943	1.656679	2.15E-65	1.68E-65	2.1	2.28	2.21	7.29	7.23	6.31
OsR498G0408956500.01	3.733	11.41	1.608633	9.82E-128	5.27E-128	3.51	3.68	4.01	12.19	10.98	11.06
OsR498G0408956900.01	110.48	29.143	-1.92225	0	0	110.28	108.47	112.7	28.31	29.56	29.56
OsR498G0408957400.01	12.906	32.683	1.334299	3.61E-126	1.95E-126	12.94	12.67	13.11	33.43	32.08	32.54
OsR498G0408958500.01	132.22	286.18	1.106068	0	0	127.1	132.56	137	292.08	285.3	281.16
OsR498G0408963000.01	44.77	232.4	2.363185	0	0	43.45	46.64	44.22	240.79	232.86	223.55
OsR498G0408964300.01	18.966	3.456	-2.47265	1.65E-281	5.32E-282	18.33	18.56	20.01	3.37	3.48	3.52
OsR498G0408964500.01	3.52	9.413	1.424576	4.53E-71	3.37E-71	3.64	3.55	3.37	10.09	8.55	9.6
OsR498G0408965700.01	67.076	17.306	-1.96122	0	0	68.6	66.7	65.93	16.92	17.29	17.71
OsR498G0408982500.01	4.453	22	2.398968	7.54E-294	2.36E-294	4.7	5.06	3.6	22.86	22.1	21.04
OsR498G0408982800.01	285.52	589.17	1.02643	0	0	281.44	288.7	286.4	584.36	593.41	589.75
OsR498G0408984200.01	0.12	1.276	3.42223	1.02E-15	1.52E-15	0.15	0.11	0.1	1.08	1.49	1.26
OsR498G0408986600.01	6.33	21.263	1.706403	0	0	6.33	6.69	5.97	21.12	22.15	20.52
OsR498G0408987300.01	0	0.473	5.127223	8.48E-06	1.78E-05	0	0	0	0.49	0.51	0.42
OsR498G0408987700.01	234.84	71.446	-1.72468	0	0	242.51	229.82	232.2	73.38	66.74	74.22
OsR498G0408993600.01	2.45	1.066	-1.2064	7.52E-07	1.49E-06	2.07	3	2.28	0.73	1.43	1.04
OsR498G0409002900.01	32.643	68.773	1.068218	8.78E-175	3.90E-175	33.78	32.26	31.89	69.65	68.76	67.91
OsR498G0409004300.01	0.436	0.923	1.08867	0.0002471	0.0005681	0.55	0.36	0.4	0.95	0.79	1.03
OsR498G0409006200.01	9.77	3.936	-1.36493	2.89E-104	1.74E-104	9.84	10.15	9.32	4.09	3.94	3.78
OsR498G0409007000.01	0.123	0.373	1.624723	0.0007	0.0016653	0.12	0.13	0.12	0.57	0.34	0.21
OsR498G0409007200.01	1.026	10.83	3.626314	1.01E-143	5.06E-144	0.79	0.87	1.42	9.63	11.88	10.98
OsR498G0409007600.01	0.26	6.1	4.647634	3.28E-32	3.59E-32	0	0.78	0	6.01	5.18	7.11
OsR498G0409008400.01	0.02	0.176	3.292993	2.80E-05	6.04E-05	0.03	0	0.03	0.22	0.17	0.14
OsR498G0409009300.01	12.716	40.46	1.663699	6.64E-181	2.90E-181	11.83	13.38	12.94	44.28	39.68	37.42
OsR498G0409010500.01	5.426	25.733	2.243058	0	0	5.63	4.62	6.03	25.7	26.11	25.39

OsR498G0409011100.01	1.983	8.393	2.066828	1.24E-31	1.37E-31	2.52	1.61	1.82	9.51	8.71	6.96
OsR498G0409012200.01	0.37	15.29	5.346421	3.57E-180	1.56E-180	0.59	0.18	0.34	14.99	15.58	15.3
OsR498G0409015600.01	0.75	3.02	2.024963	8.78E-23	1.12E-22	0.45	0.98	0.82	3.05	3.07	2.94
OsR498G0409019800.01	2.653	9.49	1.888933	5.74E-138	2.95E-138	2.39	2.67	2.9	10.21	8.96	9.3
OsR498G0409019900.01	4.21	20.17	2.230025	1.48E-74	1.08E-74	4.91	2.31	5.41	20.84	21.08	18.59
OsR498G0409023700.01	45.636	3.493	-3.71623	0	0	47.13	44.4	45.38	2.98	3.5	4
OsR498G0409024200.01	0.413	1.516	1.869292	1.84E-30	2.06E-30	0.51	0.36	0.37	1.33	1.55	1.67
OsR498G0409026000.01	5.56	1.13	-2.30669	9.03E-46	8.46E-46	4.3	5.88	6.5	1.09	1.28	1.02
OsR498G0409032100.01	0.646	0.02	-4.96024	4.08E-09	7.36E-09	0.48	0.82	0.64	0.06	0	0
OsR498G0409033700.01	3.72	1.793	-1.05632	4.04E-16	5.95E-16	3.68	3.69	3.79	1.83	1.61	1.94
OsR498G0409038800.01	0.03	0.513	4.209685	1.01E-05	2.14E-05	0	0.09	0	0.59	0.35	0.6
OsR498G0409043500.01	0.013	2.503	7.474388	2.79E-40	2.76E-40	0	0	0.04	2.47	2.95	2.09
OsR498G0409046600.01	0.596	0.063	-3.18263	1.16E-06	2.33E-06	0.85	0.35	0.59	0.04	0.07	0.08
OsR498G0409053200.01	42.706	151.48	1.82458	0	0	42.18	43.5	42.44	151.05	152.52	150.86
OsR498G0409055700.01	0.306	2.19	2.816821	1.48E-61	1.19E-61	0.2	0.3	0.42	2.25	2.15	2.17
OsR498G0409056100.01	304.21	1229.7	1.990363	0	0	314.68	301.8	296.1	1249.8	1220.7	1218.7
OsR498G0409056600.01	1.223	295.47	7.915519	0	0	1.06	1.37	1.24	309.95	291.65	284.81
OsR498G0409058200.01	1.023	29.79	4.863436	0	0	1.13	1.01	0.93	30.26	29.97	29.14
OsR498G0409058600.01	5.803	0.39	-3.89884	2.04E-104	1.23E-104	5.88	5.36	6.17	0.42	0.43	0.32
OsR498G0409059600.01	0.25	0.02	-3.76759	0.0001564	0.0003541	0.25	0.23	0.27	0	0	0.06
OsR498G0409059700.01	45.696	20.563	-1.21234	9.55E-172	4.29E-172	43.22	46.47	47.4	19.56	20.68	21.45
OsR498G0409061000.01	0	0.233	5.499192	4.66E-07	9.16E-07	0	0	0	0.38	0.16	0.16
OsR498G0409063000.01	1.8	3.673	1.01986	2.14E-07	4.16E-07	1.76	2.01	1.63	3.16	3.41	4.45
OsR498G0409063100.01	0.16	0.436	1.440298	3.33E-06	6.81E-06	0.17	0.18	0.13	0.41	0.38	0.52
OsR498G0409065000.01	11.26	3.986	-1.5452	1.85E-77	1.31E-77	10.34	11.02	12.42	3.6	4.4	3.96
OsR498G0409067400.01	3.14	1.446	-1.1202	4.81E-24	5.99E-24	3.67	2.67	3.08	1.64	1.27	1.43
OsR498G0409067600.01	2.623	6.516	1.307151	3.05E-36	3.17E-36	3.29	2.26	2.32	6.36	5.89	7.3
OsR498G0409067700.01	45.61	93.793	1.016007	0	0	46.1	45.69	45.04	91.14	95.96	94.28
OsR498G0409068000.01	4.73	1.516	-1.66633	2.23E-39	2.23E-39	4.59	4.74	4.86	1.5	1.14	1.91
OsR498G0409068300.01	31.68	15.53	-1.0456	2.27E-82	1.55E-82	29.58	32.42	33.04	16.01	16.52	14.06
OsR498G0409068700.01	0.076	0.35	2.132869	3.29E-09	5.91E-09	0.06	0.05	0.12	0.19	0.47	0.39
OsR498G0409069000.01	0.023	5.22	7.84066	7.62E-50	6.84E-50	0	0.07	0	5.6	5.05	5.01
OsR498G0409070000.01	1.533	0.566	-1.45357	6.44E-14	1.00E-13	1.57	1.62	1.41	0.55	0.56	0.59
OsR498G0409072400.01	0.036	0.25	2.847115	0.0005624	0.0013266	0	0.11	0	0.22	0.31	0.22
OsR498G0409073400.01	0.43	0	-4.41967	0.0003895	0.0009082	0.58	0.37	0.34	0	0	0
OsR498G0409073800.01	6.193	3.056	-1.05206	1.91E-31	2.12E-31	5.94	5.87	6.77	3.33	2.92	2.92
OsR498G0409081800.01	0.216	0.483	1.179938	0.0006673	0.0015856	0.22	0.27	0.16	0.43	0.46	0.56
OsR498G0409082600.01	3.513	28.356	3.01236	0	0	3.08	4.04	3.42	31.83	27.16	26.08
OsR498G0409083400.01	17.186	8.55	-1.01895	2.12E-26	2.53E-26	15.28	17.48	18.8	9.29	7.47	8.89
OsR498G0409083600.01	0.753	3.49	2.292052	8.61E-88	5.70E-88	0.93	0.7	0.63	3.27	2.93	4.27
OsR498G0409092500.01	39.006	138.9	1.801708	0	0	39.72	37.1	40.2	138.33	139.61	138.76
OsR498G0409098100.01	5.533	1.19	-2.22873	5.83E-27	6.91E-27	6.08	5.25	5.27	1.44	1.07	1.06
OsR498G0409098300.01	3.12	6.7	1.097093	4.92E-22	6.37E-22	3.48	3.04	2.84	6.88	6.85	6.37
OsR498G0409099500.01	0.63	2.993	2.257665	2.57E-28	2.97E-28	0.61	0.85	0.43	3.13	2.9	2.95
OsR498G0409099800.01	0.513	4.826	3.109604	4.91E-94	3.12E-94	0.65	0.48	0.41	3.85	5.05	5.58
OsR498G0409100900.01	0.45	1.62	1.822662	8.07E-13	1.29E-12	0.44	0.21	0.7	1.88	1.48	1.5
OsR498G0409101200.01	2.68	1.053	-1.36396	4.57E-17	6.58E-17	2.87	2.62	2.55	1.07	1.24	0.85
OsR498G0409109900.01	0.276	4.01	3.863127	1.11E-95	6.99E-96	0.33	0.3	0.2	4.48	4.19	3.36
OsR498G0409115900.01	1.186	0.573	-1.08618	2.32E-15	3.48E-15	1.15	1.25	1.16	0.62	0.52	0.58
OsR498G0409116500.01	4.11	0.686	-2.45582	3.30E-66	2.55E-66	4.28	4.05	4	0.75	0.55	0.76
OsR498G0409116700.01	2.456	19.576	2.929404	3.51E-132	1.84E-132	2.19	2.73	2.45	20.25	18.66	19.82
OsR498G0409117500.01	40.21	232.61	2.553434	0	0	39.75	40.36	40.52	235.97	229.94	231.92
OsR498G0409119100.01	3.09	17.876	2.534222	4.47E-246	1.58E-246	3.33	3.13	2.81	18.56	17.98	17.09
OsR498G0409125200.01	25.196	9.006	-1.54002	2.62E-121	1.45E-121	26.01	24.59	24.99	9.39	8.55	9.08
OsR498G0409131100.01	22.546	5.543	-2.0236	4.04E-185	1.74E-185	23.82	21.77	22.05	5.2	5.69	5.74
OsR498G0409132600.01	1.146	0.29	-1.93223	7.75E-07	1.54E-06	1.03	0.98	1.43	0.21	0.2	0.46
OsR498G0409132800.01	0.313	5.516	4.143631	1.20E-135	6.20E-136	0.31	0.43	0.2	5.74	5.41	5.4
OsR498G0409136200.01	0.716	0	-7.46803	1.50E-21	1.96E-21	0.64	0.93	0.58	0	0	0
OsR498G0409136800.01	0.256	0.073	-1.83471	8.50E-05	0.000189	0.19	0.22	0.36	0.12	0.05	0.05
OsR498G0409138800.01	2.926	6.226	1.129195	1.32E-18	1.83E-18	4.22	2.39	2.17	6.91	5.43	6.34
OsR498G0409141300.01	0.753	0.31	-1.28217	1.06E-06	2.12E-06	0.78	0.68	0.8	0.38	0.21	0.34
OsR498G0409144200.01	0.206	1.03	2.393397	6.41E-08	1.21E-07	0.37	0.13	0.12	0.68	1.28	1.13
OsR498G0409145800.01	0.863	0.33	-1.40081	2.50E-05	5.38E-05	1.13	0.76	0.7	0.37	0.24	0.38
OsR498G0409146200.01	0.06	0.996	4.03976	1.16E-08	2.14E-08	0.09	0.09	0	1.01	1.23	0.75
OsR498G0409147600.01	0.293	6.213	4.470045	6.07E-123	3.33E-123	0.34	0.25	0.29	6.03	6.31	6.3
OsR498G0409156600.01	0.45	0.036	-3.66764	2.43E-10	4.21E-10	0.38	0.52	0.45	0	0	0.11
OsR498G0409159600.01	0.646	0.073	-3.09725	1.03E-20	1.37E-20	0.63	0.74	0.57	0.12	0.07	0.03
OsR498G0409162700.01	1.88	0.39	-2.02152	2.86E-21	3.76E-21	1.74	1.54	2.36	0.6	0.21	0.36
OsR498G0409164200.01	2.71	1.406	-1.06477	8.32E-05	0.0001848	2.1	3.72	2.31	0.87	2.19	1.16

OsR498G0409164400.01	0.163	8.38	5.667836	2.30E-223	8.72E-224	0.08	0.19	0.22	8.08	8.67	8.39
OsR498G0409165000.01	0.406	1.51	1.708554	1.78E-12	2.88E-12	0.27	0.49	0.46	1.49	1.19	1.85
OsR498G0409166900.01	21.913	259.96	3.53722	0	0	22.09	21.26	22.39	260.65	257.63	261.6
OsR498G0409167200.01	0.153	0.556	1.873708	1.27E-07	2.43E-07	0.17	0.12	0.17	0.76	0.47	0.44
OsR498G0409167600.01	15.276	6.346	-1.22629	1.07E-79	7.47E-80	16.93	14.97	13.93	6.28	5.68	7.08
OsR498G0409168400.01	2.02	0.296	-2.76759	7.82E-19	1.08E-18	1.54	2.01	2.51	0.22	0.28	0.39
OsR498G0409169200.01	0.663	0.223	-1.60179	9.54E-08	1.82E-07	0.67	0.55	0.77	0.1	0.22	0.35
OsR498G0409169400.01	78.763	26.81	-1.59042	0	0	74.67	82.49	79.13	26.01	27.02	27.4
OsR498G0409169800.01	1.62	0.32	-2.32947	2.35E-17	3.36E-17	1.48	1.39	1.99	0.43	0.25	0.28
OsR498G0409171800.01	0.42	0	-5.41967	6.63E-07	1.31E-06	0.51	0.36	0.39	0	0	0
OsR498G0409177900.01	0.963	4.613	2.257351	2.95E-51	2.61E-51	1	0.95	0.94	4.96	3.63	5.25
OsR498G0409178500.01	1.383	4.523	1.708869	4.27E-32	4.69E-32	1.08	1.41	1.66	5.26	4.05	4.26
OsR498G0409180500.01	0.016	0.156	3.312779	3.62E-07	7.08E-07	0.02	0.01	0.02	0.19	0.12	0.16
OsR498G0409181900.01	24.336	100.95	2.049499	0	0	23.67	24.34	25	99.34	101.96	101.54
OsR498G0409182200.01	0.296	1.14	1.958981	1.31E-17	1.87E-17	0.19	0.27	0.43	1.13	1.33	0.96
OsR498G0409185200.01	5.68	77.41	3.777763	0	0	4.94	6.48	5.62	81.51	76.48	74.24
OsR498G0409185300.01	60.533	166.24	1.457495	0	0	60.26	63.23	58.11	172.73	158.38	167.61
OsR498G0409186100.01	0.026	0.37	3.7402	0.0002182	0.0004994	0	0	0.08	0.42	0.43	0.26
OsR498G0409189100.01	2.55	0.936	-1.4464	1.09E-27	1.27E-27	2.82	2.58	2.25	0.82	0.87	1.12
OsR498G0409190600.01	41.106	14.31	-1.19429	8.01E-179	3.52E-179	41.22	42.37	39.73	14.1	14.28	14.55
OsR498G0409191200.01	18.263	46.096	1.345609	8.98E-238	3.26E-238	17.99	20.12	16.68	47.46	45.15	45.68
OsR498G0409198500.01	60.786	3.32	-4.20345	0	0	63.91	59.44	59.01	4.26	3.37	2.33
OsR498G0409198900.01	6.743	17.246	1.348527	8.93E-137	4.60E-137	6.35	6.6	7.28	16.79	17.4	17.55
OsR498G0409200600.01	0	1.06	7.229585	2.17E-18	3.04E-18	0	0	0	0.83	0.86	1.49
OsR498G0409203000.01	3.396	26.996	2.994773	5.97E-299	1.84E-299	3.97	3.26	2.96	26.88	28.72	25.39
OsR498G0409205000.01	32.566	100.43	1.61765	0	0	31.67	32.27	33.76	96.89	103.91	100.5
OsR498G0409206800.01	8.82	2.126	-2.12478	2.97E-76	2.12E-76	9.07	8.43	8.96	2.55	1.95	1.88
OsR498G0409209900.01	0.01	1.436	7.158701	1.84E-33	1.98E-33	0	0	0.03	1.52	0.95	1.84
OsR498G0409215700.01	8.37	2.053	-2.10622	1.12E-159	5.23E-160	8.34	8.55	8.22	2.05	1.91	2.2
OsR498G0409218700.01	0.93	0.21	-2.1207	2.66E-10	4.61E-10	0.85	1.09	0.85	0.22	0.19	0.22
OsR498G0409218900.01	1.363	3.383	1.298736	3.66E-26	4.40E-26	1.31	1.11	1.67	3.24	3.68	3.23
OsR498G0409220100.01	2.923	8.46	1.76768	2.02E-49	1.82E-49	3.13	2.53	3.11	8.91	7.09	9.38
OsR498G0409220400.01	7.886	222.2	4.684165	0	0	8.6	6.97	8.09	226.77	218.89	220.93
OsR498G0409220600.01	1.736	354.13	7.659684	0	0	1.3	1.72	2.19	369.19	341.32	351.88
OsR498G0409222200.01	0.593	0.06	-3.33875	2.97E-11	4.98E-11	0.5	0.45	0.83	0.07	0.04	0.07
OsR498G0409223600.01	59.77	23.063	-1.41785	1.94E-164	8.92E-165	60.54	57.02	61.75	25.51	22.51	21.17
OsR498G0409223800.01	6.623	2.586	-1.41412	2.03E-26	2.43E-26	6.69	6.99	6.19	3.14	1.53	3.09
OsR498G0409224500.01	105.19	8.646	-3.60813	0	0	109.01	106.07	100.5	9.8	7.41	8.73
OsR498G0409225900.01	56.88	26.663	-1.03087	5.65E-255	1.96E-255	57.32	55.8	57.52	26.87	26.58	26.54
OsR498G0409226700.01	1.963	0.053	-5.09774	3.63E-42	3.54E-42	1.91	1.99	1.99	0	0.03	0.13
OsR498G0409227200.01	1.616	3.603	1.156336	2.93E-12	4.78E-12	1.25	1.72	1.88	3.3	3.58	3.93
OsR498G0409228300.01	36.263	10.02	-1.88127	9.17E-277	2.99E-277	36.54	35.97	36.28	9.16	10.71	10.19
OsR498G0409229200.01	13.616	48.28	1.82264	7.45E-185	3.21E-185	15.43	13.9	11.52	50.5	48.12	46.22
OsR498G0409230400.01	0.39	0	-7.10999	1.26E-17	1.79E-17	0.29	0.5	0.38	0	0	0
OsR498G0409230600.01	4.423	0.206	-4.7416	1.85E-63	1.47E-63	4.96	3.39	4.92	0.14	0.22	0.26
OsR498G0409231200.01	0.053	0.49	3.209685	1.18E-06	2.36E-06	0.16	0	0	0.37	0.44	0.66
OsR498G0409233900.01	11.473	46.86	2.026585	0	0	10.81	12.27	11.34	51.38	45.06	44.14
OsR498G0409237000.01	21.776	8.56	-1.34829	7.04E-267	2.36E-267	20.94	22.46	21.93	8.24	8.58	8.86
OsR498G0409239000.01	0.2	0.803	1.996691	8.09E-09	1.48E-08	0.25	0.18	0.17	0.68	0.89	0.84
OsR498G0409243900.01	3.296	0.893	-1.89553	9.67E-44	9.27E-44	3.22	2.99	3.68	0.83	0.86	0.99
OsR498G0409245200.01	16.916	43.473	1.327838	2.21E-289	7.00E-290	16.18	16.75	17.82	43.22	42.12	45.08
OsR498G0409245300.01	1.173	2.546	1.080732	9.64E-05	0.0002152	1.68	0.9	0.94	1.83	1.84	3.97
OsR498G0409246400.01	0.163	1.693	3.391745	2.21E-38	2.24E-38	0.1	0.18	0.21	1.63	1.72	1.73
OsR498G0409250300.01	3.21	1.366	-1.18646	4.41E-26	5.30E-26	3	3.13	3.5	1.3	1.41	1.39
OsR498G0409252300.01	14.06	48.606	1.789854	0	0	12.76	14.22	15.2	46.24	51.08	48.5
OsR498G0409255000.01	4.286	17.58	2.028445	1.68E-193	7.03E-194	4.27	4.6	3.99	17.34	17.02	18.38
OsR498G0409258900.01	5.253	181.09	5.099897	0	0	5.14	5.34	5.28	201.34	168.11	173.81
OsR498G0409260400.01	18.18	39.5	1.097776	1.56E-228	5.83E-229	19.74	17.4	17.4	38.72	38.96	40.82
OsR498G0409261700.01	1.16	0.366	-1.6821	3.22E-16	4.73E-16	1.13	1.04	1.31	0.26	0.41	0.43
OsR498G0409262000.01	2.756	0.336	-2.91292	3.17E-97	1.98E-97	2.39	3.02	2.86	0.29	0.34	0.38
OsR498G0409263100.01	1.226	0.446	-1.46067	1.55E-09	2.75E-09	1.1	1.45	1.13	0.45	0.46	0.43
OsR498G0409263500.01	73.213	34.793	-1.09302	7.86E-128	4.22E-128	77.81	69.6	72.23	35.66	34.14	34.58
OsR498G0409263700.01	1.98	6.84	1.786232	2.80E-94	1.77E-94	2.2	1.98	1.76	7.08	6.58	6.86
OsR498G0409265900.01	3.18	0.053	-5.89215	2.21E-30	2.48E-30	3.65	3.35	2.54	0.08	0	0.08
OsR498G0409266300.01	111.19	9.913	-3.49626	0	0	118.36	108.23	107	9.4	9.15	11.19
OsR498G0409266600.01	1.133	0.043	-4.79576	3.31E-14	5.11E-14	1.46	0.76	1.18	0	0.13	0
OsR498G0409267500.01	8.98	20.563	1.186828	8.68E-47	8.05E-47	9.48	8.93	8.53	22.54	21.28	17.87
OsR498G0409267900.01	0.69	1.51	1.370147	5.57E-13	8.86E-13	0.63	0.87	0.57	1.53	1.53	1.47
OsR498G0409268700.01	0.03	0.59	4.408994	9.77E-16	1.45E-15	0.06	0.03	0	0.76	0.35	0.66

OsR498G0409276700.01	39.983	104.76	1.386966	0	0	39.06	39.17	41.72	102.19	106.76	105.34
OsR498G0409276900.01	19.673	58.913	1.577219	0	0	19.04	19.79	20.19	57.65	59.84	59.25
OsR498G0409277900.01	18	7.44	-1.29204	1.05E-122	5.79E-123	18.03	17.26	18.71	7.32	6.99	8.01
OsR498G0409279500.01	0.006	0.066	3.624723	0.0004041	0.0009439	0	0	0.02	0.13	0.05	0.02
OsR498G0409282400.01	0.093	0.78	3.086217	1.48E-28	1.70E-28	0.13	0.05	0.1	0.73	0.82	0.79
OsR498G0409282800.01	0.46	1.2	1.382302	5.19E-20	7.02E-20	0.41	0.42	0.55	1.16	1.14	1.3
OsR498G0409287900.01	0.813	0.383	-1.0477	4.04E-05	8.80E-05	0.9	0.71	0.83	0.41	0.38	0.36
OsR498G0409288100.01	5.706	54.133	3.231855	0	0	5.53	5.95	5.64	53.81	55.3	53.29
OsR498G0409291200.01	0.013	0.526	5.76768	1.56E-14	2.39E-14	0	0.04	0	0.47	0.51	0.6
OsR498G0409292800.01	5.926	59.16	3.293219	0	0	5.69	5.92	6.17	59.98	58.82	58.68
OsR498G0409294000.01	0.73	3.566	2.29202	1.08E-85	7.21E-86	0.59	0.83	0.77	3.71	3.35	3.64
OsR498G0409298100.01	0.05	0	-4.65289	0.0001229	0.0002763	0	0.06	0.09	0	0	0
OsR498G0409300700.01	0.033	0.363	3.499192	0.0007491	0.0017869	0	0.1	0	0.49	0.1	0.5
OsR498G0409301900.01	2.32	10.586	2.176496	1.44E-90	9.41E-91	1.96	2.05	2.95	9.82	10.63	11.31
OsR498G0409305600.01	22.696	6.71	-1.79324	1.89E-202	7.64E-203	23.97	20.97	23.15	6.73	6.89	6.51
OsR498G0409306000.01	1.646	0	-6.96024	3.33E-16	4.89E-16	1.84	1.77	1.33	0	0	0
OsR498G0409307000.01	10.223	5.11	-1.02272	5.53E-26	6.66E-26	10.49	10.48	9.7	5.34	5.18	4.81
OsR498G0409307900.01	10.12	2.193	-2.26962	6.73E-154	3.23E-154	10.1	9.85	10.41	2.23	2.11	2.24
OsR498G0409308500.01	1224.2	149.58	-3.03778	0	0	1254.4	1207.9	1210	161.39	144.81	142.54
OsR498G0409312000.01	137.09	58.993	-1.28722	0	0	126.64	142.49	142.1	54.73	60.93	61.32
OsR498G0409312800.01	42.083	14.143	-1.58384	0	0	43.56	40.86	41.83	14.63	14.3	13.5
OsR498G0409313500.01	0.133	0.323	1.274225	0.0006025	0.0014258	0.14	0.15	0.11	0.38	0.27	0.32
OsR498G0409314500.01	2.606	6.94	1.418272	7.43E-30	8.40E-30	3.28	2.55	1.99	7.06	6.7	7.06
OsR498G0409317800.01	68.946	15.866	-2.16033	0	0	71.08	71.06	64.7	17.01	15.23	15.36
OsR498G0409317900.01	0.41	0.046	-3.17366	3.58E-07	7.00E-07	0.41	0.43	0.39	0	0.05	0.09
OsR498G0409318500.01	12.11	4.936	-1.22054	3.01E-77	2.14E-77	12.52	11.91	11.9	4.62	5.3	4.89
OsR498G0409319200.01	6.923	1.646	-2.04724	2.53E-85	1.70E-85	6.62	6.83	7.32	1.55	1.82	1.57
OsR498G0409322200.01	3.223	0	-8.13016	3.37E-31	3.75E-31	3	3.14	3.53	0	0	0
OsR498G0409323200.01	0.086	0.72	2.993956	4.94E-07	9.73E-07	0.13	0.07	0.06	0.55	0.91	0.7
OsR498G0409323700.01	188.58	577.23	1.599558	0	0	183.27	192.67	189.8	589.72	562.48	579.5
OsR498G0409325800.01	1.466	0.526	-1.39372	1.88E-12	3.05E-12	1.38	1.53	1.49	0.58	0.56	0.44
OsR498G0409326200.01	0.043	0.583	4.000589	5.41E-25	6.63E-25	0.04	0.03	0.06	0.52	0.64	0.59
OsR498G0409326900.01	5.963	2.583	-1.25989	1.56E-33	1.68E-33	6.04	5.66	6.19	2.83	2.45	2.47
OsR498G0409327400.01	0.523	0.096	-2.5452	3.79E-15	5.72E-15	0.67	0.38	0.52	0.1	0.07	0.12
OsR498G0409328500.01	4.586	1.043	-2.14384	1.02E-37	1.04E-37	4.79	3.84	5.13	1.46	0.76	0.91
OsR498G0409328900.01	7.046	14.67	1.054432	2.39E-50	2.13E-50	6.85	6.82	7.47	15.22	15.11	13.68
OsR498G0409329100.01	0.543	1.263	1.18415	6.18E-06	1.28E-05	0.48	0.61	0.54	1.15	1.69	0.95
OsR498G0409329700.01	1.366	0	-7.51483	4.01E-22	5.19E-22	1.9	1.17	1.03	0	0	0
OsR498G0409330100.01	1.013	7.7	2.924283	8.93E-144	4.48E-144	0.98	0.89	1.17	8.84	7.32	6.94
OsR498G0409332100.01	4.46	18.383	2.029158	1.61E-206	6.44E-207	4.98	4.46	3.94	19.03	17.76	18.36
OsR498G0409332800.01	7.323	21.94	1.597323	3.47E-289	1.10E-289	7.49	7.33	7.15	20.87	22.41	22.54
OsR498G0409334400.01	7.196	49.18	2.74622	0	0	7.65	7.05	6.89	47.88	50.51	49.15
OsR498G0409334600.01	71.493	235.88	1.703672	0	0	70.11	71.1	73.27	230.83	234.55	242.26
OsR498G0409334800.01	6.356	0.616	-3.37477	1.34E-174	5.94E-175	6.11	6.65	6.31	0.7	0.64	0.51
OsR498G0409335600.01	4.606	0.63	-2.87306	1.15E-97	7.18E-98	4.28	5.12	4.42	0.45	0.77	0.67
OsR498G0409337400.01	0.066	0.45	2.646952	0.0001135	0.0002544	0.1	0	0.1	0	1.04	0.31
OsR498G0409337500.01	4.916	0.676	-2.57432	2.47E-106	1.47E-106	4.97	5.25	4.53	0.78	0.63	0.62
OsR498G0409339000.01	1.743	0.02	-6.4142	3.06E-62	2.45E-62	1.65	1.59	1.99	0	0.06	0
OsR498G0409339700.01	11.573	45.416	1.982235	0	0	11.5	11.82	11.4	44.44	46.95	44.86
OsR498G0409340400.01	0.87	5.813	2.732782	1.16E-31	1.28E-31	0.78	0.91	0.92	6.38	6.55	4.51
OsR498G0409341800.01	3.296	0.213	-3.98999	5.94E-12	9.78E-12	3.23	2.57	4.09	0	0.21	0.43
OsR498G0409342400.01	7.35	0.21	-5.16969	7.22E-36	7.56E-36	8.43	7.19	6.43	0	0.16	0.47
OsR498G0409343000.01	11.893	1.03	-3.55086	6.76E-50	6.06E-50	11.48	12.13	12.07	0.73	1.33	1.03
OsR498G0409343100.01	40.26	4.82	-3.07406	1.94E-293	6.08E-294	42.01	37.52	41.25	5.24	4.96	4.26
OsR498G0409344100.01	4.613	43.873	3.244096	0	0	4.56	4.34	4.94	44.45	43.71	43.46
OsR498G0409345100.01	0.103	1.443	3.771564	7.40E-21	9.83E-21	0.13	0.14	0.04	1.19	1.32	1.82
OsR498G0409348400.01	1.573	0.436	-1.79031	2.21E-10	3.82E-10	1.54	1.35	1.83	0.75	0.16	0.4
OsR498G0409349400.01	57.14	4.47	-3.67549	0	0	56.42	54.82	60.18	4.33	4.12	4.96
OsR498G0409350700.01	0.1	3.113	4.897741	3.83E-22	4.96E-22	0.1	0	0.2	2.96	4.87	1.51
OsR498G0409350900.01	10.013	0.296	-5.07824	3.72E-89	2.44E-89	10.04	9.32	10.68	0.56	0	0.33
OsR498G0409351400.01	126.84	58.256	-1.16511	0	0	130.53	120.55	129.4	57.96	56.13	60.68
OsR498G0409353400.01	0.386	0.083	-2.22327	0.0001374	0.00031	0.24	0.36	0.56	0.05	0.1	0.1
OsR498G0409354400.01	0.036	0.293	2.624723	3.28E-06	6.72E-06	0.09	0.02	0	0.29	0.39	0.2
OsR498G0409358600.01	0.11	0.016	-2.4838	7.36E-05	0.0001629	0.06	0.12	0.15	0.04	0.01	0
OsR498G0409360500.01	0.746	0.3	-1.3449	0.0001665	0.0003777	0.86	0.92	0.46	0.15	0.3	0.45
OsR498G0409364100.01	6.873	14.676	1.096218	5.51E-62	4.42E-62	6.52	7.12	6.98	14.73	15	14.3
OsR498G0409365300.01	0.116	0	-4.66068	0.0001179	0.0002647	0.08	0.11	0.16	0	0	0
OsR498G0409368200.01	207.49	99.243	-1.0725	0	0	213.66	203.43	205.4	98.63	100.94	98.16
OsR498G0409368700.01	0.3	3.906	3.569013	1.26E-51	1.11E-51	0.43	0.18	0.29	3.57	4.01	4.14

OsR498G0409371600.01	7.543	0.613	-3.57762	3.85E-79	2.69E-79	8.26	7.32	7.05	0.55	0.23	1.06
OsR498G0409377600.01	14.99	31.896	1.085367	3.89E-164	1.80E-164	15.32	14.92	14.73	30.61	33.13	31.95
OsR498G0409378500.01	0.62	9.25	3.889759	1.27E-301	3.89E-302	0.31	0.68	0.87	8.93	9.41	9.41
OsR498G0409378900.01	0.083	1.193	3.7402	0.0002182	0.0004994	0	0	0.25	1.1	1.37	1.11
OsR498G0409380900.01	0.48	2.153	2.236157	1.88E-35	1.98E-35	0.5	0.55	0.39	2.2	1.83	2.43
OsR498G0409381900.01	31.68	75.496	1.248263	0	0	31.99	31.61	31.44	72.53	75.35	78.61
OsR498G0409388800.01	2.626	0.86	-1.6232	3.41E-09	6.13E-09	2.98	2.26	2.64	1.27	0.96	0.35
OsR498G0409389300.01	0.386	3.56	3.209685	4.26E-47	3.93E-47	0.37	0.43	0.36	3.83	3.1	3.75
OsR498G0409389600.01	103.06	443.84	2.102802	0	0	102.52	105.58	101.1	464.24	438.68	428.6
OsR498G0409390800.01	0	0.796	6.847115	7.65E-15	1.16E-14	0	0	0	1.14	0.78	0.47
OsR498G0409391400.01	2.433	1.026	-1.25275	7.13E-16	1.06E-15	2.54	2.26	2.5	1	0.95	1.13
OsR498G0409391900.01	12.42	4.073	-1.60904	2.69E-71	2.00E-71	13.85	12.23	11.18	4.48	4.35	3.39
OsR498G0409394600.01	0	0.246	5.127223	8.48E-06	1.78E-05	0	0	0	0.3	0.18	0.26
OsR498G0409395700.01	0.04	1.543	5.209685	2.30E-10	3.98E-10	0	0	0.12	1.14	2.07	1.42
OsR498G0409395900.01	0.133	2.57	4.259809	6.63E-47	6.13E-47	0.18	0.08	0.14	2.99	2.22	2.5
OsR498G0409396200.01	0.016	0.316	4.432077	1.64E-06	3.31E-06	0	0.05	0	0.31	0.23	0.41
OsR498G0409400300.01	4.25	38.35	3.165235	0	0	3.99	4	4.76	39.71	37.24	38.1
OsR498G0409400600.01	4.546	1.906	-1.27374	1.19E-15	1.78E-15	4.88	4.56	4.2	1.58	2.08	2.06
OsR498G0409402700.01	10.08	33.646	1.761573	2.21E-297	6.84E-298	10.62	9.7	9.92	32.63	33.98	34.33
OsR498G0409403100.01	0.05	2.73	5.7402	1.01E-26	1.20E-26	0.08	0	0.07	2.48	2.87	2.84
OsR498G0409406000.01	0.13	2.38	4.225627	8.55E-22	1.11E-21	0.16	0.08	0.15	2.16	2.38	2.6
OsR498G0409406200.01	0.21	91.413	8.739043	1.37E-245	4.85E-246	0.21	0.22	0.2	91.41	89.36	93.47
OsR498G0409409000.01	6.71	3	-1.18079	2.47E-14	3.80E-14	7.18	5.35	7.6	3.08	3.74	2.18
OsR498G0409409300.01	19.226	122.11	2.66678	0	0	17.12	19.44	21.12	123.02	120.87	122.43
OsR498G0409415100.01	1.283	0.313	-2.05978	5.95E-18	8.41E-18	1.46	1.23	1.16	0.21	0.38	0.35
OsR498G0409417800.01	418.22	176.94	-1.24014	0	0	431.48	415.4	407.8	176.72	177.2	176.89
OsR498G0409417900.01	152.56	47.506	-1.62748	0	0	139.29	154.22	164.2	49.26	48.23	45.03
OsR498G0409418500.01	81.166	12.87	-2.67097	0	0	82.69	83.41	77.4	14.1	11.71	12.8
OsR498G0409422600.01	3.91	0.576	-2.77967	3.98E-20	5.36E-20	3.98	4.19	3.56	0.3	0.82	0.61
OsR498G0409423800.01	1509.5	169.59	-3.15757	0	0	1533	1515	1481	170.7	170.37	167.71
OsR498G0409424400.01	4.213	1.913	-1.14466	3.85E-29	4.41E-29	4.53	4.39	3.72	1.77	2.14	1.83
OsR498G0409426500.01	0.05	0.576	3.499192	1.77E-12	2.86E-12	0.09	0.06	0	0.74	0.48	0.51
OsR498G0409431200.01	12.95	27.923	1.100419	2.28E-94	1.44E-94	12.7	12.65	13.5	26.55	27.59	29.63
OsR498G0409435200.01	0	0.136	4.361688	0.0005877	0.0013892	0	0	0	0.16	0.04	0.21
OsR498G0409438300.01	3.22	1.58	-1.03919	2.13E-08	3.96E-08	4	2.46	3.2	1.34	1.74	1.66
OsR498G0409445000.01	0	0.166	5.127223	8.48E-06	1.78E-05	0	0	0	0.2	0.12	0.18
OsR498G0409453400.01	0.946	0	-6.31779	2.64E-11	4.42E-11	0.96	1.22	0.66	0	0	0
OsR498G0409454600.01	2.786	6.286	1.089052	4.16E-37	4.28E-37	2.58	2.49	3.29	5.96	6.02	6.88
OsR498G0409455900.01	21.043	130.41	2.593308	0	0	21.81	19.46	21.86	135.56	125.18	130.5
OsR498G0409456500.01	4.036	1.183	-1.79952	1.85E-28	2.14E-28	4.52	3.87	3.72	1.15	1.19	1.21
OsR498G0409458800.01	69.056	28.566	-1.27914	0	0	72.09	68.13	66.95	28.21	30.49	27
OsR498G0409459100.01	235.97	492.33	1.05138	0	0	241.45	235.47	231	483.43	505.81	487.76
OsR498G0409459400.01	20.08	43.376	1.09931	1.11E-161	5.18E-162	20.06	20.95	19.23	40.52	43.87	45.74
OsR498G0409460000.01	0.393	1.093	1.490421	1.39E-07	2.68E-07	0.39	0.53	0.26	1.18	1.14	0.96
OsR498G0409460100.01	0.763	0.42	-1.09064	3.52E-05	7.64E-05	0.59	0.97	0.73	0.5	0.47	0.29
OsR498G0409460300.01	4.093	37.056	3.170118	0	0	3.49	4.68	4.11	40.76	38.34	32.07
OsR498G0409461000.01	46.613	110.52	1.183025	0	0	48.23	43.5	48.11	108.72	113	109.83
OsR498G0409461600.01	58.683	170.05	1.526368	0	0	57.53	59.72	58.8	168.73	175.38	166.04
OsR498G0409462200.01	1.98	8.34	2.079149	5.77E-130	3.07E-130	1.73	2.38	1.83	9.06	8.23	7.73
OsR498G0409465400.01	16.656	195.24	3.553893	0	0	17.72	15.98	16.27	195.07	194.56	196.1
OsR498G0409465600.01	29.37	266.36	3.175401	0	0	28.74	29.29	30.08	261.22	270.05	267.8
OsR498G0409466600.01	46.73	13.13	-1.83807	0	0	45.72	48.55	45.92	13.08	13.11	13.2
OsR498G0409467400.01	3.09	1.363	-1.21782	5.21E-31	5.80E-31	3.12	2.68	3.47	1.45	1.19	1.45
OsR498G0409471000.01	0.123	0.486	1.94158	4.45E-09	8.04E-09	0.09	0.12	0.16	0.25	0.72	0.49
OsR498G0409471500.01	5.22	12.81	1.29255	2.93E-73	2.14E-73	4.91	5.49	5.26	12.86	12.65	12.92
OsR498G0409477200.01	4.21	14.84	1.828122	5.68E-94	3.62E-94	4.2	3.78	4.65	15.14	15.23	14.15
OsR498G0409478900.01	12.97	126.97	3.277493	0	0	13.51	12.56	12.84	123.18	129.35	128.37
OsR498G0409480000.01	0.836	7.956	3.209685	4.20E-155	2.00E-155	0.94	0.85	0.72	8.34	8.05	7.48
OsR498G0409483700.01	0.056	0.903	3.963602	6.07E-18	8.58E-18	0.03	0.07	0.07	1.01	1.16	0.54
OsR498G0409485100.01	239.32	60.566	-1.99196	0	0	252.58	227.77	237.6	63.22	62.61	55.87
OsR498G0409486500.01	0.726	0.13	-2.36623	1.42E-08	2.62E-08	0.82	0.73	0.63	0.07	0.19	0.13
OsR498G0409490800.01	0.47	67.693	7.151765	0	0	0.63	0.22	0.56	73.85	63.53	65.7
OsR498G0409491400.01	0.25	2.646	3.403508	7.10E-68	5.44E-68	0.27	0.23	0.25	2.34	3.01	2.59
OsR498G0409493100.01	32.313	5.323	-2.60036	3.85E-288	1.22E-288	31.04	33.09	32.81	5.26	5.41	5.3
OsR498G0409494000.01	1.556	5.163	1.610856	2.38E-25	2.90E-25	1.59	1.58	1.5	4.49	6	5
OsR498G0409495300.01	12.036	0.16	-6.25235	1.66E-200	6.80E-201	11.19	12.17	12.75	0.17	0.09	0.22
OsR498G0409495700.01	0.01	1.333	6.910125	7.03E-29	8.06E-29	0	0.03	0	1.21	1.42	1.37
OsR498G0409497600.01	1.54	0.55	-1.5554	3.40E-12	5.55E-12	1.62	1.39	1.61	0.47	0.56	0.62
OsR498G0409503600.01	0.806	0.11	-2.91058	4.04E-19	5.57E-19	0.87	0.7	0.85	0.17	0.09	0.07

OsR498G0409505200.01	10.79	22.586	1.058471	4.18E-107	2.48E-107	10.8	10.44	11.13	22.05	23.02	22.69
OsR498G0409505900.01	13.87	33.923	1.278473	6.98E-132	3.67E-132	14.09	13.57	13.95	32.84	33.23	35.7
OsR498G0409512600.01	152.16	490.92	1.702468	0	0	148.58	153.74	154.2	532.45	474.08	466.23
OsR498G0409513100.01	17.213	0.053	-8.49382	7.75E-147	3.83E-147	18.25	17.68	15.71	0.08	0	0.08
OsR498G0409513400.01	42.453	22.89	-1.01246	1.77E-299	5.43E-300	39.1	43.95	44.31	24.26	21.95	22.46
OsR498G0409516300.01	0.776	0.283	-1.455	0.0009085	0.0021791	0.75	0.79	0.79	0.38	0.16	0.31
OsR498G0409516700.01	406.25	57.576	-2.82402	0	0	421.6	391.73	405.4	59.43	57.21	56.09
OsR498G0409516900.01	0.296	76.743	8.014696	0	0	0.32	0.34	0.23	83.03	73.79	73.41
OsR498G0409519400.01	1.51	120.03	6.306844	0	0	1.36	1.65	1.52	126.43	123.2	110.47
OsR498G0409520000.01	0.213	0.696	1.707185	2.03E-06	4.11E-06	0.19	0.16	0.29	0.84	0.51	0.74
OsR498G0409520400.01	16.95	43.773	1.366265	0	0	16.64	17.42	16.79	43.62	44.19	43.51
OsR498G0409520600.01	7.503	70.1	3.214596	0	0	7.42	7.06	8.03	68.75	75.72	65.83
OsR498G0409521200.01	1.923	0.86	-1.17069	6.94E-19	9.61E-19	1.87	1.66	2.24	0.94	0.84	0.8
OsR498G0409522400.01	0.03	0.863	4.7402	7.49E-20	1.02E-19	0.06	0.03	0	1.14	0.71	0.74
OsR498G0409524800.01	15.696	6.736	-1.23033	4.21E-104	2.54E-104	15.8	15.77	15.52	5.87	7.95	6.39
OsR498G0409526800.01	25.18	9.663	-1.38622	4.61E-99	2.86E-99	25.54	24.39	25.61	9.41	9.7	9.88
OsR498G0409529300.01	1.516	6.703	2.140561	3.37E-77	2.39E-77	1.58	1.56	1.41	6.99	6.68	6.44
OsR498G0409529600.01	3.22	1.493	-1.10673	2.36E-14	3.64E-14	3.51	3.5	2.65	1.54	1.53	1.41
OsR498G0409529800.01	0.11	0.686	2.691837	1.76E-08	3.25E-08	0.09	0.19	0.05	0.74	0.76	0.56
OsR498G0409533900.01	1.033	0.22	-2.24317	1.48E-10	2.54E-10	0.85	0.94	1.31	0.26	0.27	0.13
OsR498G0409534300.01	3.716	0.49	-2.90053	6.08E-47	5.62E-47	3.57	3.81	3.77	0.36	0.66	0.45
OsR498G0409535900.01	0.246	0	-5.4838	3.79E-07	7.43E-07	0.16	0.3	0.28	0	0	0
OsR498G0409537500.01	314.95	60.13	-2.39043	0	0	322.35	315.04	307.5	64.21	54.52	61.66
OsR498G0409537800.01	0.553	0.123	-2.1593	0.0001997	0.0004554	0.7	0.59	0.37	0	0.09	0.28
OsR498G0409541500.01	9.296	0.846	-3.45039	0	0	9.05	9.63	9.21	0.85	0.78	0.91
OsR498G0409542100.01	66.866	16.776	-1.98281	0	0	72.47	60.66	67.47	16.64	17.45	16.24
OsR498G0409542700.01	2.846	0.95	-1.59936	1.14E-30	1.27E-30	2.92	2.94	2.68	0.79	1.29	0.77
OsR498G0409545600.01	0.79	0.123	-2.69721	1.31E-16	1.91E-16	0.49	1.11	0.77	0.17	0.15	0.05
OsR498G0409546000.01	2	0.703	-1.43575	5.42E-15	8.19E-15	1.57	2.16	2.27	0.59	0.56	0.96
OsR498G0409548500.01	6.93	3.623	-1.044	5.51E-39	5.55E-39	6.83	6.81	7.15	4.03	3.56	3.28
OsR498G0409550500.01	3.026	1.25	-1.30787	2.84E-27	3.35E-27	2.64	3.15	3.29	1.2	1.34	1.21
OsR498G0509554900.01	13.193	5.343	-1.31667	1.19E-24	1.47E-24	13.61	13.2	12.77	5.75	4.44	5.84
OsR498G0509556900.01	2.766	55.526	4.317377	0	0	2.36	2.7	3.24	55.53	55.49	55.56
OsR498G0509557200.01	5.63	10.54	1.070016	3.50E-70	2.63E-70	4.83	6.25	5.81	10.35	11.16	10.11
OsR498G0509558600.01	0	0.333	4.775282	7.66E-05	0.0001698	0	0	0	0.52	0.19	0.29
OsR498G0509559600.01	0.81	0.1	-3.03063	8.58E-16	1.27E-15	0.84	0.46	1.13	0.06	0.12	0.12
OsR498G0509561800.01	7.51	3.95	-1.0867	2.99E-31	3.32E-31	7.06	7.75	7.72	3.48	4.17	4.2
OsR498G0509563100.01	0.21	0.633	1.549955	7.84E-09	1.43E-08	0.21	0.16	0.26	0.57	0.62	0.71
OsR498G0509563700.01	32.386	108.23	1.763569	0	0	32.13	32.49	32.54	106.01	108.7	109.97
OsR498G0509564700.01	78.246	14.566	-2.42369	0	0	86.23	76.73	71.78	13.96	14.86	14.88
OsR498G0509565100.01	29.743	6.333	-2.24752	5.36E-105	3.22E-105	31.39	27.32	30.52	7.11	5.21	6.68
OsR498G0509566100.01	0.083	0.006	-3.41967	0.0009442	0.002269	0.07	0.12	0.06	0.02	0	0
OsR498G0509566900.01	0	0.813	6.397312	1.51E-11	2.52E-11	0	0	0	1.12	0.54	0.78
OsR498G0509569600.01	101.35	260.11	1.357613	0	0	99.43	102.47	102.1	254.5	261.43	264.4
OsR498G0509569800.01	1.843	9.563	2.35275	2.67E-40	2.64E-40	1.67	1.53	2.33	9.18	9.46	10.05
OsR498G0509570500.01	6.676	0.843	-2.99341	1.83E-59	1.50E-59	5.84	6.9	7.29	1.04	0.72	0.77
OsR498G0509572300.01	114.18	22.256	-2.20487	0	0	117.63	110.32	114.6	22.02	22.72	22.03
OsR498G0509574600.01	46.64	22.376	-1.06368	2.51E-157	1.19E-157	47.6	44.81	47.51	23.38	21.11	22.64
OsR498G0509574900.01	5.366	3.686	-1.47214	2.39E-48	2.18E-48	6.79	4.74	4.57	2.48	4.04	4.54
OsR498G0509576000.01	1.57	0.71	-1.14141	4.02E-09	7.26E-09	1.78	1.46	1.47	1.15	0.43	0.55
OsR498G0509576200.01	0	0.196	5.499192	4.66E-07	9.16E-07	0	0	0	0.16	0.27	0.16
OsR498G0509577000.01	7.41	3.303	-1.17285	5.86E-99	3.63E-99	6.75	7.59	7.89	3.12	3.5	3.29
OsR498G0509577900.01	22.166	5.656	-1.97286	1.32E-129	7.03E-130	22.42	23.71	20.37	6.22	5.18	5.57
OsR498G0509578400.01	27.546	81.683	1.562004	0	0	27.16	26.99	28.49	82.15	83.71	79.19
OsR498G0509582000.01	2.166	0.96	-1.17597	2.24E-22	2.88E-22	1.97	2.11	2.42	1	0.97	0.91
OsR498G0509583500.01	1.923	25.243	3.707332	0	0	1.69	1.83	2.25	26.5	24.1	25.13
OsR498G0509584100.01	105.88	32.393	-1.71968	3.62E-291	1.14E-291	115.83	103.17	98.63	31.12	35.07	30.99
OsR498G0509588400.01	9.383	2.986	-1.66068	1.12E-22	1.44E-22	9.01	9.11	10.03	3.77	2.37	2.82
OsR498G0509588500.01	17.736	2.863	-2.62665	1.20E-121	6.60E-122	19.91	16.08	17.22	3.59	2.76	2.24
OsR498G0509589100.01	154.11	43.186	-1.84768	0	0	155.45	156.65	150.2	46.42	40.3	42.84
OsR498G0509591700.01	6.783	2.93	-1.18549	3.80E-26	4.56E-26	7.85	6.82	5.68	2.79	3.14	2.86
OsR498G0509592800.01	0.546	5.98	3.442519	1.44E-120	8.02E-121	0.61	0.47	0.56	6.6	6.23	5.11
OsR498G0509596400.01	5.496	1.073	-2.36309	8.82E-37	9.10E-37	5.97	5.21	5.31	1.34	1.01	0.87
OsR498G0509596900.01	0.27	0.193	-1.01469	0.0009694	0.0023312	0.27	0.29	0.25	0.14	0.12	0.32
OsR498G0509597100.01	198.64	80.183	-1.31959	0	0	195.11	202.58	198.2	79.64	80.36	80.55
OsR498G0509597500.01	2.236	0.763	-1.55347	2.66E-27	3.14E-27	2.1	2.42	2.19	0.6	1.02	0.67
OsR498G0509597700.01	664.18	117	-2.51393	0	0	663.78	668.84	659.9	120.82	112.14	118.05
OsR498G0509600000.01	0.02	1.163	5.922403	5.82E-16	8.60E-16	0	0	0.06	1.4	0.9	1.19
OsR498G0509604700.01	0.106	3.583	5.249213	1.21E-80	8.43E-81	0.22	0.04	0.06	3.54	3.85	3.36

OsR498G0509606200.01	9.496	4.726	-1.032	3.16E-42	3.07E-42	9.23	10.13	9.13	4.79	4.93	4.46
OsR498G0509606600.01	21.943	3.393	-2.69599	1.59E-293	4.97E-294	19.89	23.02	22.92	3.69	3.37	3.12
OsR498G0509606800.01	347.44	81.353	-2.09605	0	0	344.08	358.13	340.1	83.39	77.16	83.51
OsR498G0509611500.01	0.166	0.043	-1.89884	0.0006013	0.0014228	0.13	0.14	0.23	0.09	0.02	0.02
OsR498G0509614300.01	2.47	1.21	-1.0347	2.84E-11	4.77E-11	2.29	2.54	2.58	1.24	1.56	0.83
OsR498G0509615200.01	11.916	40.813	1.770221	4.66E-174	2.08E-174	11.93	12.59	11.23	44.42	39.62	38.4
OsR498G0509616100.01	1.006	0.04	-4.6949	2.84E-09	5.09E-09	0.98	1.12	0.92	0	0.12	0
OsR498G0509621700.01	3.143	49.09	3.896303	0	0	2.97	3.22	3.24	49.33	49.04	48.9
OsR498G0509626400.01	135.62	53.073	-1.36146	0	0	134.8	140.26	131.8	56.32	50.94	51.96
OsR498G0509629300.01	0.003	0.11	4.563322	4.91E-07	9.67E-07	0	0	0.01	0.1	0.1	0.13
OsR498G0509630100.01	0.883	0.073	-3.84288	1.33E-25	1.62E-25	0.76	0.83	1.06	0.08	0.03	0.11
OsR498G0509631100.01	33.083	12.78	-1.3781	2.78E-167	1.27E-167	30.97	33.56	34.72	12.71	13.61	12.02
OsR498G0509631600.01	0	0.533	4.847115	5.08E-05	0.0001113	0	0	0	0.68	0.46	0.46
OsR498G0509633500.01	4.876	1.51	-1.58342	3.77E-36	3.93E-36	4.32	5.26	5.05	1.24	1.79	1.5
OsR498G0509642200.01	0.173	0.04	-1.73785	0.0009028	0.0021644	0.26	0.14	0.12	0.05	0.02	0.05
OsR498G0509647100.01	0.193	0.916	2.454798	5.71E-15	8.65E-15	0.25	0.13	0.2	0.94	0.92	0.89
OsR498G0509647700.01	0.12	1.006	3.062128	3.08E-24	3.82E-24	0.07	0.12	0.17	0.93	1.13	0.96
OsR498G0509648000.01	30.936	65.223	1.075278	2.47E-232	9.14E-233	30.49	31.34	30.98	63.75	66.35	65.57
OsR498G0509652000.01	0.913	4.083	2.103671	7.54E-23	9.61E-23	0.83	1.09	0.82	4.27	3.82	4.16
OsR498G0509653300.01	2.286	0.166	-3.78621	6.54E-64	5.15E-64	2.44	2.04	2.38	0.2	0.19	0.11
OsR498G0509654700.01	0.096	4.186	5.442145	9.55E-173	4.27E-173	0.12	0.12	0.05	4.54	4.07	3.95
OsR498G0509655300.01	2.213	7.546	1.768214	2.22E-47	2.04E-47	2.33	2.44	1.87	8.51	7.58	6.55
OsR498G0509655800.01	2.293	5.16	1.168823	1.24E-21	1.61E-21	2.05	2.53	2.3	5.69	4.16	5.63
OsR498G0509658500.01	0.326	6.703	4.310864	1.06E-112	6.12E-113	0.24	0.43	0.31	6.12	7.28	6.71
OsR498G0509659300.01	37.71	3.216	-3.42721	0	0	39.9	36.24	36.99	3.29	3.19	3.17
OsR498G0509660500.01	32.766	135	2.036704	0	0	29.93	34.37	34	135.99	138.95	130.07
OsR498G0509662700.01	9.69	32.33	1.74094	1.63E-286	5.19E-287	9.36	10.52	9.19	32.39	32.41	32.19
OsR498G0509663700.01	81.723	179.68	1.107776	0	0	73.68	89.7	81.79	182.99	176.21	179.85
OsR498G0509666100.01	41.476	11.003	-1.93085	5.49E-192	2.31E-192	42.67	42.33	39.43	11	11.78	10.23
OsR498G0509666500.01	1.086	5.436	2.426091	1.52E-66	1.17E-66	1	0.96	1.3	5.13	5.34	5.84
OsR498G0509671000.01	22.04	8.646	-1.35538	2.27E-81	1.57E-81	24.13	20.74	21.25	9.78	7.85	8.31
OsR498G0509671200.01	8.93	1.38	-2.70468	5.11E-78	3.61E-78	9.24	9.37	8.18	1.16	1.63	1.35
OsR498G0509672600.01	9.8	3.886	-1.34782	2.89E-84	1.95E-84	10.15	9.04	10.21	3.35	4.33	3.98
OsR498G0509676800.01	34.933	12.576	-1.46692	1.54E-128	8.24E-129	34.44	35.77	34.59	14.02	12.63	11.08
OsR498G0509683500.01	0.19	0.063	-1.5301	0.0003053	0.0007055	0.16	0.23	0.18	0.09	0.05	0.05
OsR498G0509688700.01	35.236	73.666	1.058685	0	0	34.77	34.83	36.11	70.51	74.19	76.3
OsR498G0509689200.01	27.596	72.09	1.391116	0	0	28.62	27.66	26.51	70.68	73.42	72.17
OsR498G0509691000.01	11.01	4.296	-1.35129	2.02E-60	1.64E-60	10.28	12.09	10.66	5.02	4.53	3.34
OsR498G0509696100.01	16.386	2.813	-2.54599	0	0	15.3	16.57	17.29	2.82	2.7	2.92
OsR498G0509696800.01	2.616	10.253	1.971143	2.15E-74	1.56E-74	1.96	3.3	2.59	10.3	10.33	10.13
OsR498G0509699400.01	82.756	470.63	2.500313	0	0	91.41	79.07	77.79	490.05	445.4	476.45
OsR498G0509702300.01	5.056	14.53	1.475345	1.08E-81	7.44E-82	4.98	4.39	5.8	14.75	15.43	13.41
OsR498G0509711600.01	0.503	0.233	-1.11778	1.37E-05	2.90E-05	0.49	0.59	0.43	0.32	0.13	0.25
OsR498G0509712800.01	8.383	3.053	-1.46496	5.72E-38	5.83E-38	9.03	7.62	8.5	3.35	2.58	3.23
OsR498G0509713700.01	3.036	11.04	1.963975	9.19E-119	5.15E-119	3.37	3.08	2.66	11.65	10.2	11.27
OsR498G0509718400.01	0.783	6.833	3.12068	2.26E-40	2.24E-40	0.63	0.94	0.78	6.8	7.44	6.26
OsR498G0509718700.01	0.726	2.52	1.776726	2.54E-06	5.18E-06	1.01	0.61	0.56	2.1	2.43	3.03
OsR498G0509720300.01	10.81	5.07	-1.16638	1.96E-51	1.73E-51	11.14	10.69	10.6	4.43	5.31	5.47
OsR498G0509720600.01	308.72	129.91	-1.24989	0	0	320.88	302.44	302.8	129.69	133.02	127.01
OsR498G0509721500.01	131.57	271.98	1.04581	0	0	130.25	132.14	132.3	261.89	274.02	280.04
OsR498G0509722400.01	0.01	0.166	4.209685	1.01E-05	2.14E-05	0	0	0.03	0.11	0.17	0.22
OsR498G0509722700.01	27.73	117.06	2.111313	0	0	26.9	29.71	26.58	115.55	118.11	117.51
OsR498G0509723500.01	24.093	48.976	1.019099	0	0	22.94	24.05	25.29	46.79	49.89	50.25
OsR498G0509725700.01	2.12	0.55	-1.94585	5.41E-27	6.41E-27	1.99	2.3	2.07	0.62	0.52	0.51
OsR498G0509726000.01	4.976	15.1	1.461759	4.25E-174	1.90E-174	4.8	4.74	5.39	14.07	16.19	15.04
OsR498G0509726700.01	0.223	0	-4.65846	0.0001193	0.0002679	0.2	0.14	0.33	0	0	0
OsR498G0509733900.01	0.186	0.523	1.520397	3.78E-06	7.77E-06	0.15	0.31	0.1	0.63	0.45	0.49
OsR498G0509734200.01	0.306	1.293	2.069832	6.22E-13	9.92E-13	0.34	0.25	0.33	1.05	1.34	1.49
OsR498G0509736600.01	48.476	15.783	-1.61654	0	0	48.54	49.22	47.67	16.13	15.6	15.62
OsR498G0509736900.01	0.076	1.11	3.624723	1.13E-25	1.36E-25	0.05	0.05	0.13	1.02	1.2	1.11
OsR498G0509738400.01	1.99	0.903	-1.15118	5.80E-08	1.09E-07	2.05	1.63	2.29	1.01	0.91	0.79
OsR498G0509738700.01	25.15	122.01	2.271801	0	0	24.37	24.97	26.11	122.27	116.9	126.85
OsR498G0509739300.01	0.653	1.426	1.064719	6.72E-07	1.33E-06	0.73	0.58	0.65	1.77	1.29	1.22
OsR498G0509745300.01	79.043	34.133	-1.21753	1.05E-237	3.81E-238	82.59	76.42	78.12	34.11	34.41	33.88
OsR498G0509746000.01	0.113	0.28	1.289738	9.00E-07	1.79E-06	0.09	0.12	0.13	0.21	0.4	0.23
OsR498G0509747600.01	25.033	4.97	-2.32604	0	0	24.03	25.04	26.03	4.66	4.86	5.39
OsR498G0509751300.01	0.58	0.11	-2.40502	1.42E-14	2.18E-14	0.49	0.7	0.55	0.09	0.17	0.07
OsR498G0509768400.01	3.976	1.413	-1.54936	3.95E-52	3.46E-52	3.75	4.09	4.09	1.43	1.39	1.42
OsR498G0509769000.01	4.936	0.753	-2.71513	6.56E-54	5.66E-54	5.63	4.84	4.34	0.64	0.66	0.96

OsR498G0509773600.01	4.563	1.403	-1.70274	4.10E-45	3.87E-45	4.8	4.44	4.45	1.42	1.45	1.34
OsR498G0509775000.01	12.37	0.876	-4.0164	6.71E-256	2.32E-256	13.15	12.02	11.94	0.66	1.03	0.94
OsR498G0509775700.01	3.65	0.136	-4.73002	1.15E-99	7.09E-100	3.83	3.78	3.34	0.13	0.1	0.18
OsR498G0509776200.01	0.206	0	-5.76759	2.44E-08	4.54E-08	0.11	0.19	0.32	0	0	0
OsR498G0509781400.01	4.223	0.813	-2.35913	1.26E-50	1.12E-50	4.46	4.43	3.78	1.07	0.86	0.51
OsR498G0509785400.01	0.4	0.02	-4.35256	6.23E-11	1.06E-10	0.4	0.36	0.44	0.06	0	0
OsR498G0509788400.01	3.4	1.22	-1.46471	5.69E-10	9.96E-10	2.86	4.13	3.21	1.43	1.21	1.02
OsR498G0509793900.01	67.91	1.666	-5.35237	0	0	70.92	66.2	66.61	1.85	1.61	1.54
OsR498G0509796400.01	0.883	0.36	-1.30263	9.57E-06	2.01E-05	1.01	0.63	1.01	0.34	0.55	0.19
OsR498G0509799000.01	29.316	70.516	1.299379	0	0	28.51	30.52	28.92	70.64	70.54	70.37
OsR498G0509800400.01	22.27	116.18	2.369665	0	0	22.26	22.23	22.32	116.82	117.22	114.5
OsR498G0509801900.01	162.17	66.893	-1.2802	0	0	163.16	162.51	160.9	67.7	66.11	66.87
OsR498G0509802100.01	7.86	1.966	-2.00088	2.91E-61	2.35E-61	7.16	8.87	7.55	2.28	1.84	1.78
OsR498G0509804000.01	3.693	8.006	1.109421	2.93E-28	3.39E-28	3.91	3.28	3.89	8.05	8.34	7.63
OsR498G0509804600.01	3.123	7.17	1.210926	2.08E-74	1.51E-74	2.89	3.51	2.97	7.38	7.31	6.82
OsR498G0509809900.01	16.66	6.853	-1.24634	1.67E-150	8.11E-151	16.25	16.73	17	7.37	7.15	6.04
OsR498G0509813400.01	0.006	0.21	4.794648	4.50E-08	8.46E-08	0	0.02	0	0.23	0.19	0.21
OsR498G0509820600.01	15.326	48.236	1.651724	0	0	15.66	15.59	14.73	47.3	47.54	49.87
OsR498G0509822800.01	3.686	0.656	-2.49294	2.32E-71	1.72E-71	3.46	3.92	3.68	0.76	0.54	0.67
OsR498G0509823300.01	142.01	358.63	1.254428	0	0	141.01	143.49	141.5	368.21	369.53	338.14
OsR498G0509823500.01	0.363	1.723	2.197301	1.12E-22	1.44E-22	0.39	0.21	0.49	2.02	1.67	1.48
OsR498G0509827300.01	0.256	1.633	2.624723	3.19E-14	4.93E-14	0.27	0.29	0.21	1.82	1.69	1.39
OsR498G0509827700.01	0	0.05	4.361688	0.0005877	0.0013892	0	0	0	0.06	0.05	0.04
OsR498G0509830800.01	501.2	85.873	-2.53408	0	0	514.23	495.6	493.8	83.11	87.85	86.66
OsR498G0509831100.01	398.44	37.476	-3.40194	0	0	408.61	393.18	393.5	38.45	38.19	35.79
OsR498G0509831400.01	0.566	0.223	-1.35636	4.70E-07	9.25E-07	0.61	0.59	0.5	0.45	0.1	0.12
OsR498G0509831700.01	0.843	0	-6.13016	3.48E-10	6.05E-10	0.49	0.89	1.15	0	0	0
OsR498G0509832300.01	2.373	0.46	-2.41442	1.53E-37	1.57E-37	2.31	2.75	2.06	0.46	0.42	0.5
OsR498G0509835300.01	1.15	2.693	1.271086	8.92E-14	1.39E-13	1.52	0.85	1.08	2.57	2.38	3.13
OsR498G0509836000.01	0.976	0.073	-3.71513	4.06E-07	7.96E-07	1.29	0.91	0.73	0	0.11	0.11
OsR498G0509836400.01	0.246	0.08	-1.5452	5.16E-05	0.0001132	0.2	0.26	0.28	0.13	0.03	0.08
OsR498G0509837600.01	2.203	0.95	-1.21804	0.0001633	0.0003701	2.74	2.53	1.34	1.3	0.63	0.92
OsR498G0509838800.01	9.06	1.516	-2.92344	9.02E-169	4.09E-169	8.92	8.9	9.36	0.96	1.72	1.87
OsR498G0509840400.01	4.106	1.233	-1.74369	3.37E-35	3.55E-35	4.19	3.77	4.36	1.03	1.48	1.19
OsR498G0509847200.01	2.96	1.173	-1.33521	1.53E-39	1.53E-39	2.87	2.99	3.02	1.04	1.3	1.18
OsR498G0509848800.01	6.883	14.483	1.05841	2.64E-24	3.27E-24	7.2	6.2	7.25	14.45	14.28	14.72
OsR498G0509849600.01	0.13	0	-5.0477	1.13E-05	2.39E-05	0.07	0.19	0.13	0	0	0
OsR498G0509849900.01	2.59	0.553	-2.23033	5.26E-31	5.86E-31	2.58	2.73	2.46	0.54	0.5	0.62
OsR498G0509850100.01	5.076	2.4	-1.08825	1.22E-19	1.66E-19	5.12	4.92	5.19	2.33	2.71	2.16
OsR498G0509856700.01	4.29	11.776	1.440965	3.89E-71	2.90E-71	4.7	4.15	4.02	10.84	13.13	11.36
OsR498G0509860600.01	5.71	0.73	-2.9754	3.52E-95	2.22E-95	5.43	5.25	6.45	0.51	0.72	0.96
OsR498G0509865400.01	0.436	0.043	-3.31779	3.28E-09	5.90E-09	0.32	0.47	0.52	0.03	0.07	0.03
OsR498G0509867200.01	0.653	0.176	-1.93424	3.03E-07	5.91E-07	0.75	0.52	0.69	0.08	0.19	0.26
OsR498G0509868200.01	0.346	10.61	4.890617	7.14E-49	6.48E-49	0.45	0.16	0.43	10.94	9.67	11.22
OsR498G0509868500.01	8.513	16.726	1.013765	7.78E-101	4.78E-101	8.68	8.03	8.83	17.64	16.47	16.07
OsR498G0509869000.01	12.41	6.036	-1.06333	6.01E-30	6.79E-30	11.44	12.94	12.85	5.67	6.48	5.96
OsR498G0509869200.01	0.526	28.516	5.774339	0	0	0.47	0.61	0.5	28.05	28.03	29.47
OsR498G0509871400.01	1.016	59.89	5.953993	0	0	0.81	1.03	1.21	56.59	61.92	61.16
OsR498G0509871700.01	0.24	33.26	7.070427	3.18E-121	1.76E-121	0.72	0	0	34.53	33.3	31.95
OsR498G0509875200.01	24.926	166.21	2.70521	0	0	26.44	21.89	26.45	171.15	162.36	165.12
OsR498G0509880500.01	0.953	2.053	1.134708	2.44E-16	3.57E-16	1.04	1.07	0.75	1.71	2.45	2
OsR498G0509889700.01	32.83	74.28	1.176787	0	0	32.76	33.03	32.7	74.22	73.23	75.39
OsR498G0509895600.01	3.043	0.906	-1.70368	1.36E-26	1.62E-26	2.71	3.09	3.33	0.87	1.02	0.83
OsR498G0509901500.01	14.06	1.726	-3.0252	2.22E-159	1.04E-159	13.29	14.82	14.07	2.01	1.48	1.69
OsR498G0509902100.01	1.99	0.193	-3.43056	1.85E-26	2.21E-26	2.03	1.69	2.25	0.24	0.2	0.14
OsR498G0509907400.01	3.17	1.18	-1.44356	8.84E-11	1.51E-10	3.2	2.78	3.53	1.23	1.62	0.69
OsR498G0509914200.01	0.756	0	-6.28217	4.40E-11	7.43E-11	0.89	0.44	0.94	0	0	0
OsR498G0509916000.01	0.066	0.473	3.209685	2.71E-08	5.04E-08	0.1	0.05	0.05	0.4	0.35	0.67
OsR498G0509917000.01	0.843	22.796	4.759702	0	0	0.63	0.93	0.97	23.45	22.49	22.45
OsR498G0509918900.01	0.68	11.83	4.121629	4.61E-279	1.49E-279	0.53	0.87	0.64	12.06	11.58	11.85
OsR498G0509920800.01	23.103	3.223	-2.8142	2.09E-277	6.80E-278	24.76	22.91	21.64	2.85	3.59	3.23
OsR498G0509922600.01	25.486	10.883	-1.25023	1.08E-141	5.47E-142	23.27	27.19	26	11.14	10.3	11.21
OsR498G0509929800.01	24.85	3.03	-3.03496	0	0	25.96	24.18	24.41	2.87	2.7	3.52
OsR498G0509934500.01	0.89	0.183	-2.31779	6.73E-07	1.33E-06	0.82	0.64	1.21	0.21	0.06	0.28
OsR498G0509935200.01	2.243	17.153	2.945856	7.62E-302	2.32E-302	2.06	2.5	2.17	17.48	16.52	17.46
OsR498G0509943900.01	11.11	47.63	2.099659	3.15E-298	9.73E-299	11.64	10.57	11.12	50.87	45.8	46.22
OsR498G0509953400.01	0.32	0.046	-2.76759	6.01E-05	0.0001325	0.32	0.38	0.26	0.05	0	0.09
OsR498G0509955200.01	22.996	5.01	-2.43829	9.13E-243	3.28E-243	23.04	23.52	22.43	5.04	5.08	4.91
OsR498G0509955700.01	0.013	0.26	4.361688	3.01E-06	6.15E-06	0.04	0	0	0.42	0.2	0.16

OsR498G0509960000.01	10.846	21.866	1.002607	1.81E-49	1.63E-49	12.15	9.71	10.68	22.94	20.32	22.34
OsR498G0509961300.01	2.066	5.293	1.131842	1.68E-34	1.79E-34	2.17	2.17	1.86	4.33	5.69	5.86
OsR498G0509967100.01	0.36	0	-4.76759	6.52E-05	0.0001441	0.47	0.16	0.45	0	0	0
OsR498G0509968900.01	0.483	3.68	2.909176	5.67E-33	6.15E-33	0.34	0.41	0.7	3.37	3.59	4.08
OsR498G0509970000.01	239.06	25.72	-3.19969	0	0	234.48	240.71	242	25.27	25.12	26.77
OsR498G0509978600.01	29.816	3.863	-2.98326	0	0	28.8	30.22	30.43	4.09	3.8	3.7
OsR498G0509981600.01	6.58	0.646	-3.37322	2.46E-145	1.22E-145	5.95	6.86	6.93	0.41	0.69	0.84
OsR498G0509988600.01	0.353	0.053	-2.63831	2.09E-06	4.24E-06	0.2	0.51	0.35	0.1	0.03	0.03
OsR498G0509996700.01	9.94	33.666	1.746754	2.88E-94	1.83E-94	9.33	10.31	10.18	32.69	34.86	33.45
OsR498G0509997700.01	0.61	0.236	-1.38474	6.90E-05	0.0001526	0.5	0.6	0.73	0.22	0.19	0.3
OsR498G0510001600.01	0.053	0.746	3.847115	1.30E-07	2.49E-07	0.08	0.08	0	0.71	0.73	0.8
OsR498G0510005600.01	28.596	9.916	-1.47578	1.10E-173	4.93E-174	28.04	30.6	27.15	9.75	10.29	9.71
OsR498G0510013900.01	20.88	5.823	-1.99877	1.61E-216	6.27E-217	20.32	20.93	21.39	5.47	4.86	7.14
OsR498G0510015900.01	24.436	7.316	-1.71092	8.50E-266	2.85E-266	26.07	22.26	24.98	7.68	7.05	7.22
OsR498G0510017200.01	18.41	0	-10.6555	1.57E-122	8.62E-123	17.32	18.61	19.3	0	0	0
OsR498G0510020300.01	1.036	0.29	-2.0086	2.70E-11	4.53E-11	0.89	0.93	1.29	0.24	0.42	0.21
OsR498G0510026100.01	1.913	0.726	-1.4077	1.50E-05	3.20E-05	1.61	2.2	1.93	0.49	0.9	0.79
OsR498G0510044200.01	10.906	36.4	1.68624	7.55E-227	2.84E-227	12.32	10.67	9.73	37.61	35.47	36.12
OsR498G0510052200.01	5.393	2.05	-1.48554	3.30E-58	2.74E-58	5.18	5.86	5.14	2.45	1.71	1.99
OsR498G0510053600.01	80.663	221.63	1.460682	0	0	84.5	77.62	79.87	234.8	226.19	203.9
OsR498G0510054500.01	0.67	0.12	-2.47827	1.25E-05	2.64E-05	0.56	0.57	0.88	0.14	0.15	0.07
OsR498G0510066500.01	10.34	1.746	-2.54649	5.88E-157	2.79E-157	9.74	10.72	10.56	1.38	2.05	1.81
OsR498G0510069400.01	0.096	0.33	1.740633	0.0006281	0.0014885	0.11	0.11	0.07	0.35	0.2	0.44
OsR498G0510072400.01	0	1.423	7.982275	5.31E-28	6.18E-28	0	0	0	0.87	1.59	1.81
OsR498G0510076400.01	23.07	3.616	-2.68388	1.15E-290	3.63E-291	23.6	22.45	23.16	2.84	3.76	4.25
OsR498G0510083100.01	61.623	14.856	-2.06991	0	0	66.08	60.2	58.59	16.05	14.26	14.26
OsR498G0510087900.01	133.61	54.933	-1.32451	0	0	132.11	137.57	131.2	51.43	56.95	56.42
OsR498G0510107300.01	23.496	9.1	-1.38192	1.64E-134	8.56E-135	23.93	23.01	23.55	9.19	9.47	8.64
OsR498G0510111400.01	0	0.65	6.624723	4.17E-13	6.62E-13	0	0	0	0.68	0.62	0.65
OsR498G0510112800.01	0.343	0.033	-3.41967	7.56E-06	1.58E-05	0.23	0.67	0.13	0.05	0.05	0
OsR498G0510116600.01	0.94	3.326	1.822989	2.44E-46	2.27E-46	1.2	0.79	0.83	3.5	3.61	2.87
OsR498G0510118100.01	1.663	0.836	-1.02788	7.40E-12	1.22E-11	1.58	1.8	1.61	1.06	0.59	0.86
OsR498G0510123300.01	2.25	4.146	1.005791	5.88E-30	6.64E-30	1.85	2.94	1.96	4.25	3.92	4.27
OsR498G0510123500.01	0.983	0.45	-1.12685	1.13E-08	2.08E-08	1.25	1.02	0.68	0.53	0.52	0.3
OsR498G0510124100.01	11.933	1.746	-2.77653	3.15E-149	1.54E-149	12.58	12.22	11	1.61	1.82	1.81
OsR498G0510127200.01	2.523	0.896	-1.50161	3.96E-13	6.28E-13	3.11	2.22	2.24	1.27	0.68	0.74
OsR498G0510128800.01	0	1.066	7.01704	2.52E-16	3.69E-16	0	0	0	1.56	0.98	0.66
OsR498G0510130300.01	3.56	1.093	-1.75954	3.93E-25	4.80E-25	3.69	3.57	3.42	1.29	0.67	1.32
OsR498G0510131500.01	0.843	0.163	-2.19728	3.17E-17	4.56E-17	0.9	0.87	0.76	0.28	0.16	0.05
OsR498G0510133000.01	1.15	15.45	3.733657	2.75E-224	1.04E-224	1.09	1.31	1.05	14.52	16.4	15.43
OsR498G0510138100.01	0	1.113	6.993956	4.08E-16	6.02E-16	0	0	0	1.38	0.77	1.19
OsR498G0510167100.01	29.903	14.35	-1.02513	3.20E-212	1.26E-212	29.89	30.54	29.28	13.79	14.99	14.27
OsR498G0510174900.01	0.39	0.813	1.090386	4.32E-08	8.12E-08	0.37	0.38	0.42	0.74	0.83	0.87
OsR498G0510177300.01	0.193	2.163	3.499192	1.34E-31	1.48E-31	0.04	0.26	0.28	2.32	1.8	2.37
OsR498G0510177900.01	0.286	0.05	-2.5452	4.32E-05	9.42E-05	0.36	0.26	0.24	0.07	0.04	0.04
OsR498G0510192800.01	0.973	6.14	3.034183	1.48E-221	5.66E-222	1.34	0.9	0.68	5.61	6.37	6.44
OsR498G0510195400.01	0.016	0.376	4.425562	4.10E-27	4.84E-27	0	0.01	0.04	0.37	0.42	0.34
OsR498G0510210000.01	6.573	1.576	-1.9402	1.65E-58	1.36E-58	6.76	6.06	6.9	1.56	1.56	1.61
OsR498G0510210400.01	15.63	1.73	-3.03947	1.15E-175	5.10E-176	15.53	16.83	14.53	1.94	1.43	1.82
OsR498G0510215800.01	2.453	0.573	-2.04366	3.40E-30	3.82E-30	2.61	2.29	2.46	0.64	0.58	0.5
OsR498G0510220300.01	0.05	0.656	3.73534	1.30E-06	2.61E-06	0	0.15	0	0.58	0.61	0.78
OsR498G0510226700.01	2.73	1.186	-1.21178	2.64E-09	4.74E-09	2.45	3.03	2.71	1.41	1.21	0.94
OsR498G0510242400.01	4.763	0.616	-2.95572	1.96E-53	1.69E-53	5.07	5.09	4.13	0.45	0.52	0.88
OsR498G0510250400.01	10.133	20.64	1.020201	4.36E-67	3.35E-67	10.42	9.94	10.04	21.86	20.06	20
OsR498G0510266000.01	4.353	13.216	1.59536	8.21E-85	5.53E-85	4.65	4.16	4.25	13.6	13.48	12.57
OsR498G0510274800.01	0.01	0.143	3.847115	0.0001179	0.0002648	0.03	0	0	0.12	0.19	0.12
OsR498G0510277400.01	0.103	0	-5.13016	6.38E-06	1.33E-05	0.07	0.14	0.1	0	0	0
OsR498G0510282400.01	5.626	2.663	-1.13886	0.000368	0.000856	5.67	3.92	7.29	3.72	2.13	2.14
OsR498G0510322300.01	7.503	105.18	3.773325	0	0	6.67	7.62	8.22	101.14	107.45	106.96
OsR498G0510344200.01	252.17	43.006	-2.5571	0	0	248.4	251.03	257.1	43.11	43.62	42.29
OsR498G0510348500.01	291.68	121.91	-1.27007	0	0	288.71	293.89	292.4	126.12	117.6	122
OsR498G0510365500.01	21.606	48.08	1.147124	4.17E-129	2.22E-129	21.85	21.47	21.5	51.16	47	46.08
OsR498G0510370700.01	1.56	0.526	-1.58118	5.23E-17	7.54E-17	1.59	1.42	1.67	0.54	0.63	0.41
OsR498G0510378100.01	4.666	21.28	2.209685	1.20E-185	5.16E-186	5.61	3.69	4.7	21.59	20.33	21.92
OsR498G0510378400.01	0.886	0.206	-1.96024	2.61E-12	4.25E-12	0.98	0.69	0.99	0.18	0.19	0.25
OsR498G0510395600.01	10.786	23.203	1.107811	4.96E-190	2.10E-190	10.52	11.18	10.66	21.59	24.14	23.88
OsR498G0510408400.01	0.91	0.213	-2.09142	4.18E-09	7.55E-09	0.44	1.28	1.01	0.29	0.3	0.05
OsR498G0510408500.01	11.086	1.106	-3.33518	0	0	9.11	11.98	12.17	0.9	1.31	1.11
OsR498G0510414500.01	6.563	66.233	3.329768	0	0	6.14	6.8	6.75	61.97	68.46	68.27

OsR498G0510418900.01	1.823	0.31	-2.57384	8.02E-23	1.02E-22	1.8	1.89	1.78	0.41	0.19	0.33
OsR498G0510426700.01	0.043	0	-4.56431	0.0001944	0.000443	0.01	0.05	0.07	0	0	0
OsR498G0510434500.01	14.603	3.38	-2.05025	6.39E-183	2.78E-183	15.02	15.27	13.52	3.42	3.56	3.16
OsR498G0510436600.01	8.963	29.28	1.701161	1.61E-184	6.97E-185	9.3	8.64	8.95	29.78	29.24	28.82
OsR498G0510436900.01	0.7	0.233	-1.60077	0.0002686	0.0006189	0.87	0.48	0.75	0	0.29	0.41
OsR498G0510442200.01	0	1.156	6.897741	2.86E-15	4.30E-15	0	0	0	1.3	0.97	1.2
OsR498G0510446000.01	8.263	2.546	-1.70943	3.30E-28	3.83E-28	7.12	8.71	8.96	3.04	2.38	2.22
OsR498G0510448200.01	0.016	1.453	6.578919	1.05E-23	1.32E-23	0.05	0	0	1.57	1.74	1.05
OsR498G0510452500.01	34.833	9.076	-1.97842	3.16E-261	1.07E-261	36.38	33.2	34.92	9.58	8.63	9.02
OsR498G0510468900.01	9.956	32.83	1.704234	2.71E-273	8.91E-274	10.05	9.73	10.09	32.83	33.49	32.17
OsR498G0510477100.01	49.273	5.483	-2.69705	0	0	48.27	49.31	50.24	5.98	4.91	5.56
OsR498G0510478100.01	0.263	2.243	3.094668	1.02E-43	9.74E-44	0.23	0.21	0.35	2.32	2.38	2.03
OsR498G0510479400.01	9.036	21.106	1.165589	1.44E-149	7.04E-150	8.81	9.33	8.97	21.72	20.22	21.38
OsR498G0510480600.01	13.813	32.05	1.219037	6.11E-285	1.95E-285	12.59	14.65	14.2	33.48	31.49	31.18
OsR498G0510486800.01	27.84	193.28	2.867075	0	0	29.23	25.87	28.42	196.95	197.34	185.55
OsR498G0510489300.01	2.52	0.333	-2.91133	2.29E-20	3.07E-20	2.77	3.05	1.74	0.26	0.34	0.4
OsR498G0510489900.01	0.126	0.646	2.335216	8.74E-09	1.60E-08	0.24	0.07	0.07	0.67	0.55	0.72
OsR498G0510491900.01	20.746	79.93	1.92278	0	0	20.95	20.06	21.23	82.04	81.94	75.81
OsR498G0510492800.01	1.03	5.68	2.425818	8.00E-33	8.69E-33	1.03	0.78	1.28	6.08	4.81	6.15
OsR498G0510496300.01	92.946	267.42	1.520253	0	0	93.65	95.01	90.18	265.74	274.78	261.75
OsR498G0510497000.01	2.653	0.766	-1.75775	7.42E-21	9.86E-21	2.24	2.78	2.94	0.75	0.69	0.86
OsR498G0510498700.01	1.943	0.18	-3.46274	3.68E-08	6.90E-08	2.06	1.45	2.32	0	0.54	0
OsR498G0510500000.01	1.153	140.38	6.906326	0	0	0.89	1.03	1.54	142.54	135.96	142.63
OsR498G0510502500.01	1.84	0.436	-2.09774	1.24E-06	2.49E-06	1.74	2.1	1.68	0.52	0.53	0.26
OsR498G0510502800.01	5.826	0.92	-2.66433	5.66E-88	3.74E-88	5.23	5.61	6.64	1.05	0.9	0.81
OsR498G0510504100.01	1.393	0.366	-1.92547	6.85E-10	1.20E-09	1.31	1.17	1.7	0.26	0.32	0.52
OsR498G0510506900.01	0.226	0.086	-1.83471	8.50E-05	0.000189	0.2	0.25	0.23	0.03	0.13	0.1
OsR498G0510508400.01	1.276	2.973	1.197301	1.98E-16	2.90E-16	1.03	1.45	1.35	3.77	2.28	2.87
OsR498G0510525000.01	0.96	9.746	3.335457	8.44E-204	3.40E-204	0.96	0.92	1	9.28	9.79	10.17
OsR498G0510530600.01	9.413	21.483	1.185149	8.72E-107	5.18E-107	8.93	9.2	10.11	22.91	21.16	20.38
OsR498G0510534900.01	1.596	0.163	-3.24693	2.55E-44	2.43E-44	1.53	1.88	1.38	0.05	0.24	0.2
OsR498G0510544200.01	2.983	19.186	2.662882	0	0	2.87	2.98	3.1	19.83	19.03	18.7
OsR498G0510550000.01	14.683	3.903	-1.87407	1.71E-89	1.12E-89	14.39	16.08	13.58	4.08	4.52	3.11
OsR498G0510551800.01	0	0.46	5.210486	4.69E-06	9.68E-06	0	0	0	0.45	0.45	0.48
OsR498G0510554500.01	0.153	0.026	-2.5452	0.0003114	0.00072	0.1	0.11	0.25	0.05	0	0.03
OsR498G0510555700.01	7.41	30.623	1.992927	7.09E-168	3.22E-168	6.31	7.74	8.18	30.93	28.5	32.44
OsR498G0510560400.01	2.906	1.073	-1.60275	2.12E-25	2.58E-25	2.95	2.84	2.93	0.98	1.18	1.06
OsR498G0510563200.01	0.746	3.62	2.273557	1.05E-17	1.49E-17	0.49	1.04	0.71	3.96	4.15	2.75
OsR498G0510566200.01	1.05	0.44	-1.25302	3.85E-12	6.30E-12	1	0.91	1.24	0.47	0.47	0.38
OsR498G0510567700.01	6.7	242.19	5.177299	0	0	6.76	5.77	7.57	265.46	233.15	227.95
OsR498G0510578500.01	16.126	8.25	-1.02549	1.26E-49	1.13E-49	2.89	2.56	42.93	0.39	12.16	12.2
OsR498G0510579100.01	43.233	3.72	-3.5003	0	0	60.92	54.16	14.62	11.16	0	0
OsR498G0510580000.01	7.01	0.753	-3.23589	2.01E-57	1.69E-57	7.81	6.4	6.82	0.65	1.14	0.47
OsR498G0510581000.01	1.346	2.943	1.124649	3.47E-08	6.49E-08	1.34	1.53	1.17	3.1	3.07	2.66
OsR498G0510585900.01	6.136	14.13	1.198141	1.68E-81	1.16E-81	6.17	5.95	6.29	13.95	14.63	13.81
OsR498G0510598100.01	28.623	65.083	1.167704	4.54E-280	1.47E-280	29.53	28.38	27.96	64.66	65.4	65.19
OsR498G0510602900.01	16.026	39.353	1.289459	0	0	15.6	16.21	16.27	37.77	39.73	40.56
OsR498G0510610900.01	0.733	0.176	-2.07572	5.14E-06	1.06E-05	0.62	0.71	0.87	0.23	0.18	0.12
OsR498G0510611100.01	7.243	3.19	-1.14565	4.35E-38	4.43E-38	7.38	7.63	6.72	2.79	3.48	3.3
OsR498G0510613800.01	0.533	0.103	-2.35879	3.46E-09	6.22E-09	0.6	0.4	0.6	0.06	0.17	0.08
OsR498G0510624200.01	1.076	0.29	-1.89514	4.53E-08	8.52E-08	0.84	1.04	1.35	0.36	0.36	0.15
OsR498G0510625500.01	5.223	0.366	-3.83758	5.07E-52	4.45E-52	5.74	5.02	4.91	0.51	0.2	0.39
OsR498G0510625800.01	0.4	0.036	-4.61387	2.10E-07	4.07E-07	0.58	0.13	0.49	0.11	0	0
OsR498G0510638400.01	0.24	1.17	2.246211	1.30E-16	1.90E-16	0.23	0.22	0.27	1.42	0.9	1.19
OsR498G0510641200.01	16.253	58.333	1.844748	0	0	16.25	16.79	15.72	56.78	59.11	59.11
OsR498G0510645500.01	0.146	0	-4.86713	3.63E-05	7.88E-05	0.12	0.12	0.2	0	0	0
OsR498G0510653900.01	2.43	1.05	-1.22048	3.15E-12	5.14E-12	2.62	1.86	2.81	1.26	1.01	0.88
OsR498G0510658800.01	6.596	3.366	-1.00259	1.32E-19	1.80E-19	6.18	6.71	6.9	3.28	3.34	3.48
OsR498G0510659700.01	1.156	0.136	-3.48949	1.13E-25	1.37E-25	1.26	1.09	1.12	0.04	0.22	0.15
OsR498G0510662300.01	8.846	20.11	1.187099	2.46E-88	1.62E-88	7.99	9.01	9.54	19.44	21.34	19.55
OsR498G0510662600.01	11.756	230.45	4.307708	0	0	11.81	12.29	11.17	230.94	225.06	235.34
OsR498G0510668900.01	15.686	6.43	-1.30045	5.09E-79	3.57E-79	14.86	17.27	14.93	6.26	6.41	6.62
OsR498G0510671000.01	1.09	2.363	1.092227	0.0003626	0.0008428	0.6	1.15	1.52	2.25	2.81	2.03
OsR498G0510671900.01	12.88	5.91	-1.13079	2.15E-53	1.86E-53	13.47	11.57	13.6	6.4	5.82	5.51
OsR498G0510673100.01	2.363	1.146	-1.06353	5.84E-13	9.31E-13	2.31	2.39	2.39	1.05	1.37	1.02
OsR498G0510673800.01	3.19	0.09	-5.23326	3.87E-215	1.51E-215	2.62	3.59	3.36	0.09	0.08	0.1
OsR498G0510674700.01	0.456	1.19	1.377929	3.24E-10	5.64E-10	0.28	0.54	0.55	1.23	1.22	1.12
OsR498G0510676300.01	0	0.123	4.499192	0.0003163	0.0007323	0	0	0	0.15	0.13	0.09
OsR498G0510676700.01	1.243	0.24	-2.37528	0.0009088	0.0021805	1.82	1.69	0.22	0.24	0	0.48

OsR498G0510677300.01	0.343	1.553	2.167865	7.32E-20	9.92E-20	0.4	0.23	0.4	1.64	1.43	1.59
OsR498G0510677900.01	84.233	556.8	2.721664	0	0	87.83	82.21	82.66	551.46	559.31	559.64
OsR498G0510680400.01	0.526	0.033	-3.96024	1.06E-11	1.77E-11	0.59	0.44	0.55	0.03	0.07	0
OsR498G0510680800.01	2.676	15.6	2.521146	5.15E-132	2.71E-132	2.26	2.93	2.84	16.01	15.06	15.73
OsR498G0510682900.01	1.506	7.636	2.323968	1.68E-40	1.66E-40	1.61	1.15	1.76	8.15	6.53	8.23
OsR498G0510689400.01	0.256	0.573	1.165165	9.16E-05	0.0002041	0.21	0.3	0.26	0.45	0.64	0.63
OsR498G0510690000.01	0.013	0.083	2.624723	0.0002134	0.0004876	0.01	0.01	0.02	0.08	0.1	0.07
OsR498G0510694200.01	0.036	0.346	3.209685	5.30E-05	0.0001164	0.11	0	0	0.4	0.41	0.23
OsR498G0510699600.01	9.963	26.48	1.380204	9.40E-154	4.51E-154	10.73	10.21	8.95	26.82	25.76	26.86
OsR498G0510701800.01	2.896	1.286	-1.22237	5.70E-18	8.05E-18	3.23	2.76	2.7	1.32	1.25	1.29
OsR498G0510703100.01	1.833	7.353	2.003664	4.21E-73	3.09E-73	2.35	1.62	1.53	7.25	6.97	7.84
OsR498G0510705500.01	1.64	0.68	-1.27927	1.23E-12	1.99E-12	1.63	1.49	1.8	0.59	0.83	0.62
OsR498G0510710800.01	23.836	4.143	-2.52845	4.19E-295	1.30E-295	24.4	23.69	23.42	4.68	3.71	4.04
OsR498G0510715100.01	6.65	3.296	-1.17405	6.18E-38	6.31E-38	7.09	6.19	6.67	3.44	3.37	3.08
OsR498G0510717000.01	2.88	5.783	1.001625	2.87E-10	4.98E-10	3.13	3.06	2.45	6.18	5.65	5.52
OsR498G0510723600.01	0.45	1.51	1.731638	1.91E-05	4.08E-05	0.62	0.33	0.4	1.92	1.31	1.3
OsR498G0510726700.01	2.19	0.833	-1.38785	2.35E-42	2.28E-42	1.97	2.19	2.41	0.75	0.78	0.97
OsR498G0510730700.01	1.666	0.53	-1.64027	1.98E-15	2.97E-15	1.58	1.72	1.7	0.57	0.44	0.58
OsR498G0510731700.01	0.693	9.086	3.708139	1.15E-55	9.74E-56	0.98	0.72	0.38	10.08	7.95	9.23
OsR498G0510734900.01	51.896	28.4	-1.00974	8.05E-229	3.01E-229	53.12	50.33	52.24	27.83	29.97	27.4
OsR498G0510739700.01	0.386	2.35	2.581654	1.82E-30	2.04E-30	0.41	0.32	0.43	2.59	2.28	2.18
OsR498G0510740200.01	1.716	0.03	-5.78467	2.26E-41	2.22E-41	1.57	1.71	1.87	0.03	0	0.06
OsR498G0510740300.01	2.303	0.036	-5.78685	7.93E-42	7.75E-42	2.09	2.09	2.73	0	0	0.11
OsR498G0510740900.01	2.356	0.506	-2.32099	6.43E-16	9.51E-16	2.18	2.2	2.69	0.43	0.51	0.58
OsR498G0510747400.01	7.19	17.853	1.306394	5.01E-223	1.91E-223	6.93	7.15	7.49	16.2	19.52	17.84
OsR498G0510749100.01	0.076	1.713	4.499192	9.06E-17	1.31E-16	0.08	0.08	0.07	2.23	1.42	1.49
OsR498G0510755500.01	17.686	50.866	1.519419	0	0	17.96	16.72	18.38	53.22	49.82	49.56
OsR498G0510757800.01	0.203	0.973	2.267566	5.45E-24	6.80E-24	0.18	0.17	0.26	1.03	0.97	0.92
OsR498G0510759900.01	4.193	1.923	-1.13282	4.09E-20	5.52E-20	4.04	4.14	4.4	1.72	1.81	2.24
OsR498G0510762500.01	0.353	0.113	-1.64474	2.49E-05	5.37E-05	0.4	0.32	0.34	0.12	0.15	0.07
OsR498G0510763400.01	2.466	0.746	-1.71582	1.16E-23	1.46E-23	1.98	2.99	2.43	0.86	0.71	0.67
OsR498G0510763700.01	37.61	16.92	-1.12943	1.76E-218	6.80E-219	37.91	36.32	38.6	17.57	16.74	16.45
OsR498G0510765400.01	0.216	2.046	3.236157	8.96E-22	1.17E-21	0.27	0.28	0.1	1.92	2.09	2.13
OsR498G0510765500.01	1.196	9.79	3.005544	4.21E-44	4.02E-44	1.12	0.95	1.52	10.92	9.25	9.2
OsR498G0510767500.01	4.42	0.656	-2.76759	4.52E-23	5.74E-23	4.15	4.37	4.74	0.78	0.7	0.49
OsR498G0510767800.01	52.99	15.12	-1.82643	1.74E-144	8.70E-145	57.66	47.28	54.03	15.45	13.99	15.92
OsR498G0510770300.01	2.916	1.28	-1.18341	1.24E-31	1.37E-31	2.84	2.81	3.1	1.21	1.37	1.26
OsR498G0510770800.01	0.643	1.403	1.116381	2.85E-12	4.64E-12	0.56	0.64	0.73	1.35	1.41	1.45
OsR498G0510773300.01	3.753	1.71	-1.1772	1.83E-14	2.80E-14	3.64	3.99	3.63	1.99	1.44	1.7
OsR498G0510774100.01	0.95	0.23	-2.0554	5.70E-22	7.40E-22	1.08	0.95	0.82	0.2	0.31	0.18
OsR498G0510775200.01	17.213	52.463	1.586271	0	0	17.6	16.29	17.75	51.21	53.56	52.62
OsR498G0510775500.01	4.05	23.53	2.531897	0	0	4.35	3.87	3.93	22.97	24.37	23.25
OsR498G0510776500.01	83.406	31.523	-1.39379	0	0	78.65	86.51	85.06	33.42	30.37	30.78
OsR498G0510778400.01	0.55	1.266	1.206118	6.12E-07	1.21E-06	0.4	0.65	0.6	1.03	1.3	1.47
OsR498G0510783300.01	1.026	10.1	3.280321	1.88E-55	1.60E-55	0.99	0.84	1.25	12.37	8.68	9.25
OsR498G0510787600.01	0.09	0	-4.5452	0.0002138	0.0004889	0.09	0.05	0.13	0	0	0
OsR498G0510790000.01	9.18	31	1.741795	1.46E-206	5.85E-207	9.23	9.45	8.86	28.88	30.55	33.57
OsR498G0510790800.01	4.29	0.7	-2.61232	1.96E-39	1.96E-39	3.88	4.58	4.41	0.68	0.78	0.64
OsR498G0510793300.01	0.053	0.33	3.946651	6.37E-05	0.0001407	0	0.16	0	0.48	0.14	0.37
OsR498G0510794200.01	0.723	74.75	6.688297	0	0	0.65	0.79	0.73	75.79	76.28	72.18
OsR498G0510794300.01	1.473	0	-7.66068	5.25E-24	6.55E-24	1.47	1.85	1.1	0	0	0
OsR498G0510796000.01	180.34	21.833	-3.05216	0	0	187.95	175.16	177.9	22.57	20.74	22.19
OsR498G0510797600.01	14.073	66.276	2.245549	0	0	13.77	14.66	13.79	66.98	64.98	66.87
OsR498G0510800200.01	0.433	0.096	-2.20755	0.0001743	0.0003958	0.27	0.55	0.48	0	0.12	0.17
OsR498G0510802100.01	0.026	2.54	6.602002	3.68E-46	3.44E-46	0.04	0.04	0	2.53	2.46	2.63
OsR498G0510809900.01	13.716	28.773	1.052799	3.21E-246	1.14E-246	13.68	14.2	13.27	29.18	28.78	28.36
OsR498G0510810400.01	7.866	28.653	1.848846	0	0	7.93	7.9	7.77	26.42	30.88	28.66
OsR498G0510811600.01	0.413	6.316	3.980698	5.68E-219	2.19E-219	0.34	0.43	0.47	6.22	6.09	6.64
OsR498G0510815700.01	6.933	18.21	1.54811	2.67E-142	1.35E-142	7.62	6.26	6.92	19.12	17.6	17.91
OsR498G0510820700.01	0.35	0	-5.41967	6.63E-07	1.31E-06	0.47	0.35	0.23	0	0	0
OsR498G0510825900.01	0.92	0.08	-3.51204	3.59E-26	4.31E-26	0.96	0.85	0.95	0.06	0.09	0.09
OsR498G0510826100.01	0.633	0.083	-2.90926	5.15E-24	6.42E-24	0.48	0.7	0.72	0.07	0.1	0.08
OsR498G0510828200.01	8	3.656	-1.13249	2.13E-57	1.78E-57	7.91	7.81	8.28	3.84	3.84	3.29
OsR498G0510832000.01	15.44	5.63	-1.45545	2.56E-148	1.26E-148	16.05	15.23	15.04	5.39	5.52	5.98
OsR498G0510836400.01	0.04	1.086	4.644622	1.43E-18	1.99E-18	0.04	0	0.08	1.18	1.27	0.81
OsR498G0510842500.01	4.426	20.803	2.315063	0	0	4.51	4.54	4.23	19.97	20.96	21.48
OsR498G0510842800.01	8.46	33.353	1.931463	0	0	8.42	7.91	9.05	32.83	34.66	32.57
OsR498G0510843600.01	24.336	175.96	2.843108	0	0	24.17	25.68	23.16	172.8	176.5	178.59
OsR498G0510845300.01	0	0.143	5.03976	1.53E-05	3.26E-05	0	0	0	0.24	0.11	0.08

OsR498G0510849900.01	1.95	0.933	-1.0738	3.78E-12	6.19E-12	2.17	1.52	2.16	0.94	0.96	0.9
OsR498G0510850300.01	6.86	20.96	1.58749	8.01E-108	4.73E-108	6.89	7.32	6.37	20.79	21.07	21.02
OsR498G0510852000.01	4.47	11.3	1.323128	7.23E-28	8.44E-28	4.49	3.94	4.98	12.44	11.86	9.6
OsR498G0510860600.01	25.79	114.76	2.141455	0	0	25.47	26.4	25.5	115.51	114.05	114.73
OsR498G0510863900.01	140.62	433.64	1.623697	0	0	144.35	136.79	140.7	432.96	433.53	434.42
OsR498G0510865100.01	0.473	0.066	-2.57495	1.07E-08	1.96E-08	0.69	0.41	0.32	0.03	0.1	0.07
OsR498G0510866100.01	4.96	10.236	1.049833	5.24E-66	4.06E-66	5.33	5.35	4.2	9.74	10.82	10.15
OsR498G0510866900.01	7.59	126.86	4.18017	0	0	6.05	9.77	6.95	126.85	129.71	124.03
OsR498G0510872700.01	0.39	4.02	3.365635	1.53E-138	7.87E-139	0.34	0.37	0.46	4.1	4.15	3.81
OsR498G0510874300.01	10.756	26.703	1.305452	1.03E-157	4.84E-158	10	10.83	11.44	28.5	25.55	26.06
OsR498G0510875900.01	0.23	0	-4.28217	0.0007127	0.0016975	0.14	0	0.55	0	0	0
OsR498G0510877500.01	0.98	0.2	-2.37528	7.02E-06	1.47E-05	0.5	0.99	1.45	0.22	0.21	0.17
OsR498G0510880400.01	62.52	188.53	1.590892	0	0	60.52	63.75	63.29	179.89	192.82	192.89
OsR498G0510881100.01	1.82	4.533	1.331787	1.62E-24	2.00E-24	1.68	1.73	2.05	4.59	4.26	4.75
OsR498G0510884900.01	4.393	9.92	1.165826	5.49E-49	4.97E-49	3.62	4.76	4.8	10.95	9.55	9.26
OsR498G0510886400.01	1.193	32.026	4.742614	0	0	0.91	1.23	1.44	33.38	32.28	30.42
OsR498G0510888800.01	0.526	0	-6.16969	2.07E-10	3.57E-10	0.42	0.71	0.45	0	0	0
OsR498G0510889400.01	0.22	0	-5.6041	1.26E-07	2.41E-07	0.24	0.28	0.14	0	0	0
OsR498G0510892400.01	0.223	0.91	2.03976	0.0002325	0.0005334	0	0.35	0.32	1.7	0.46	0.57
OsR498G0510892600.01	21.956	48.76	1.145067	0	0	22.36	21.02	22.49	47.86	50.88	47.54
OsR498G0510893800.01	1.526	0.606	-1.34296	6.73E-14	1.05E-13	1.65	1.19	1.74	0.84	0.59	0.39
OsR498G0510894800.01	21.923	0.663	-5.06109	0	0	21.05	20.65	24.07	0.62	0.98	0.39
OsR498G0510897600.01	0.213	1.283	2.568139	1.25E-12	2.01E-12	0.1	0.16	0.38	1.62	1.04	1.19
OsR498G0510897900.01	4.443	1.276	-1.82216	1.46E-43	1.40E-43	4.57	3.74	5.02	1.21	1.35	1.27
OsR498G0510898800.01	2.72	0.553	-2.39209	3.15E-35	3.33E-35	2.35	2.86	2.95	0.44	0.55	0.67
OsR498G0510899100.01	0.413	0.143	-1.5452	3.09E-05	6.69E-05	0.36	0.33	0.55	0.05	0.22	0.16
OsR498G0510899400.01	0.163	0.03	-2.37528	0.0009088	0.0021805	0.12	0.19	0.18	0.06	0.03	0
OsR498G0510902800.01	3.64	13.216	1.854057	2.04E-131	1.08E-131	3.45	3.5	3.97	12.73	12.85	14.07
OsR498G0510909100.01	1.933	9.13	2.217882	1.56E-44	1.48E-44	2.31	1.33	2.16	10.02	8.8	8.57
OsR498G0510912100.01	5.583	2.533	-1.14757	3.10E-14	4.78E-14	6.08	5.1	5.57	2.75	2.8	2.05
OsR498G0510912500.01	0.736	0.066	-3.44567	1.18E-22	1.51E-22	0.71	0.62	0.88	0.1	0.1	0
OsR498G0510918600.01	0.123	1.583	3.624723	1.45E-06	2.92E-06	0.19	0	0.18	2.37	1.19	1.19
OsR498G0510923200.01	14.16	5.486	-1.32226	2.70E-66	2.09E-66	13.75	13.87	14.86	5.26	5.62	5.58
OsR498G0510924200.01	4.836	0.796	-2.61845	4.36E-45	4.11E-45	5.22	3.97	5.32	0.63	1.11	0.65
OsR498G0510924300.01	13.993	5.833	-1.27503	8.36E-25	1.03E-24	13.28	14.77	13.93	5.76	5.89	5.85
OsR498G0510924800.01	0.313	0.74	1.241394	1.90E-05	4.06E-05	0.31	0.42	0.21	0.98	0.72	0.52
OsR498G0510928200.01	6.546	17.343	1.157358	1.75E-94	1.11E-94	6.39	6.89	6.36	17.1	17.75	17.18
OsR498G0510935600.01	0.206	6.16	4.893992	1.90E-64	1.49E-64	0.4	0.14	0.08	5.88	5.43	7.17
OsR498G0510936200.01	0.533	4.25	2.98572	7.61E-46	7.13E-46	0.38	0.49	0.73	5.27	3.66	3.82
OsR498G0510937800.01	40.82	12.036	-1.76402	8.01E-212	3.15E-212	40.53	41.49	40.44	13.2	11.57	11.34
OsR498G0510938900.01	2.093	16.05	2.935063	4.51E-247	1.59E-247	2.25	2.1	1.93	16.7	15.72	15.73
OsR498G0510941100.01	0.16	5.31	5.005544	1.25E-89	8.18E-90	0.08	0.25	0.15	4.89	5.6	5.44
OsR498G0510945400.01	1.18	0.486	-1.29175	1.10E-24	1.35E-24	1.13	1.25	1.16	0.4	0.56	0.5
OsR498G0510952500.01	1.466	0.673	-1.13533	6.60E-05	0.0001458	1.63	1.38	1.39	0.77	0.53	0.72
OsR498G0510954600.01	0.303	0.05	-2.59251	3.58E-06	7.34E-06	0.35	0.22	0.34	0.06	0	0.09
OsR498G0510957400.01	12.363	3.75	-1.72263	2.00E-117	1.13E-117	12.74	12.75	11.6	4.25	3.79	3.21
OsR498G0510961900.01	19.81	8.516	-1.28337	2.68E-116	1.52E-116	20.93	18.81	19.69	8.34	8.88	8.33
OsR498G0510962400.01	5.62	2.21	-1.34999	2.05E-21	2.69E-21	5.54	6.13	5.19	2.15	2.34	2.14
OsR498G0510964700.01	15.386	7.816	-1.06241	9.44E-185	4.07E-185	15.99	14.38	15.79	7.71	7.91	7.83
OsR498G0510966100.01	5.19	0.463	-3.50105	1.79E-104	1.08E-104	5.46	4.67	5.44	0.29	0.69	0.41
OsR498G0510966400.01	14.586	35.22	1.262766	1.27E-172	5.66E-173	14.11	15.67	13.98	34.53	34.34	36.79
OsR498G0510969800.01	4.133	1.24	-1.89628	6.58E-38	6.72E-38	3.83	4.48	4.09	1.25	1.34	1.13
OsR498G0510970700.01	0.623	0.013	-5.63267	1.03E-13	1.61E-13	0.63	0.61	0.63	0	0	0.04
OsR498G0510970900.01	0	0.886	6.65447	2.51E-13	3.97E-13	0	0	0	0.64	0.76	1.26
OsR498G0510972600.01	23.64	7.85	-1.60957	7.50E-128	4.02E-128	23.84	24.1	22.98	7.8	8.49	7.26
OsR498G0510976700.01	22.196	47.46	1.087066	1.50E-133	7.85E-134	22.64	24.57	19.38	49.11	47.15	46.12
OsR498G0510977000.01	14.846	4.903	-1.6007	3.42E-82	2.34E-82	15.39	14.46	14.69	4.61	4.74	5.36
OsR498G0510977100.01	13.426	32.09	1.235206	3.50E-118	1.97E-118	14.19	12.31	13.78	32.13	32.94	31.2
OsR498G0510977300.01	0.083	0.42	2.241394	3.93E-13	6.23E-13	0.06	0.08	0.11	0.37	0.42	0.47
OsR498G0510978500.01	2.113	0.72	-1.55661	4.16E-11	7.01E-11	2.33	2.19	1.82	0.86	0.63	0.67
OsR498G0510979100.01	0.62	10.833	4.124864	3.47E-265	1.17E-265	0.62	0.62	0.62	10.87	10.87	10.76
OsR498G0510980700.01	2.06	5.503	1.413514	4.48E-45	4.23E-45	2.11	1.96	2.11	6.23	4.92	5.36
OsR498G0510982500.01	33.77	70.036	1.053886	0	0	32.28	34.48	34.55	67.72	72.72	69.67
OsR498G0510984600.01	0.87	3.303	1.93758	3.46E-46	3.23E-46	0.83	1	0.78	3.08	3.68	3.15
OsR498G0510986100.01	0.023	0.236	3.262152	6.54E-07	1.29E-06	0.07	0	0	0.17	0.36	0.18
OsR498G0510991000.01	18.16	38.203	1.06892	1.29E-91	8.36E-92	17.51	19.06	17.91	39.65	37.51	37.45
OsR498G0510994000.01	283.86	9.403	-4.93064	0	0	298.1	280.06	273.4	10.53	9.06	8.62
OsR498G0510996600.01	17.383	91.56	2.394124	0	0	17.99	17.35	16.81	88.28	91.35	95.05
OsR498G0510998400.01	0.133	0.02	-2.76759	0.0007566	0.0018055	0.08	0.18	0.14	0.03	0.03	0

OsR498G0510999400.01	0.246	0.01	-4.71513	7.27E-08	1.38E-07	0.33	0.2	0.21	0	0	0.03
OsR498G0511000600.01	9.896	21.326	1.102222	4.36E-243	1.56E-243	9.18	9.98	10.53	21.41	21.43	21.14
OsR498G0511001400.01	0.036	1.82	5.65447	5.68E-37	5.85E-37	0	0.08	0.03	1.97	1.96	1.53
OsR498G0511001900.01	0.26	1.763	2.702725	1.37E-07	2.64E-07	0.13	0.14	0.51	2.07	1.96	1.26
OsR498G0511003600.01	0.296	2.106	2.800572	1.82E-11	3.04E-11	0.2	0.31	0.38	2.56	1.47	2.29
OsR498G0511004700.01	0.066	0.446	2.770153	3.10E-13	4.90E-13	0.09	0.04	0.07	0.47	0.48	0.39
OsR498G0511005600.01	0	0.22	4.499192	0.0003163	0.0007323	0	0	0	0.06	0.36	0.24
OsR498G0511006000.01	0.076	0.283	1.847115	1.78E-06	3.60E-06	0.08	0.09	0.06	0.29	0.26	0.3
OsR498G0511006600.01	16.223	36.72	1.168782	3.93E-70	2.95E-70	17.06	15.65	15.96	41.34	32.09	36.73
OsR498G0511006800.01	21	43.59	1.03976	3.60E-101	2.21E-101	21.06	19.87	22.07	40.94	45.67	44.16
OsR498G0511007800.01	1.96	0.363	-2.41396	1.13E-34	1.20E-34	1.94	2	1.94	0.38	0.31	0.4
OsR498G0511009100.01	21.213	48.306	1.171032	1.26E-147	6.23E-148	22.66	21.31	19.67	44.78	51.67	48.47
OsR498G0511015200.01	0.04	0.483	3.694567	5.84E-05	0.0001285	0	0.12	0	1.12	0	0.33
OsR498G0511016100.01	0.24	9.783	5.383168	1.41E-194	5.89E-195	0.17	0.48	0.07	9.93	9.33	10.09
OsR498G0511019800.01	0.5	2.803	2.482402	3.54E-43	3.42E-43	0.65	0.43	0.42	3.18	2.63	2.6
OsR498G0511020400.01	4.33	11.236	1.376248	7.02E-93	4.50E-93	4.69	4.37	3.93	12.78	10.61	10.32
OsR498G0511020500.01	9.573	23.873	1.439536	1.97E-159	9.21E-160	9.46	9.37	9.89	20.78	24.38	26.46
OsR498G0511021200.01	6.75	36.916	2.421414	0	0	6.34	7.71	6.2	36	36.31	38.44
OsR498G0511021900.01	5.603	1.42	-1.99586	1.60E-14	2.45E-14	6.24	5.59	4.98	1.83	1.15	1.28
OsR498G0511022200.01	1.02	2.226	1.12025	1.21E-07	2.32E-07	1	1.05	1.01	2.65	2	2.03
OsR498G0511029000.01	6.893	50.33	2.863829	0	0	6.66	6.65	7.37	51.81	50.47	48.71
OsR498G0511031400.01	0.063	26.163	8.634551	0	0	0.02	0.08	0.09	25.69	25.49	27.31
OsR498G0511032000.01	14.586	59.573	2.026947	0	0	13.93	15.04	14.79	60.67	57.23	60.82
OsR498G0511033800.01	0.783	3.483	2.240296	1.06E-68	8.04E-69	0.72	0.56	1.07	3.2	4.02	3.23
OsR498G0511037900.01	0.143	0	-4.96024	2.02E-05	4.33E-05	0.11	0.14	0.18	0	0	0
OsR498G0511042100.01	1.17	14.056	3.59825	0	0	0.97	1.63	0.91	15.19	13.36	13.62
OsR498G0511044800.01	0.226	0	-5.0477	1.13E-05	2.39E-05	0.16	0.21	0.31	0	0	0
OsR498G0511045900.01	6.303	2.25	-1.49185	7.28E-22	9.46E-22	6.65	5.9	6.36	2.77	1.56	2.42
OsR498G0511047500.01	0.096	0.006	-4.0477	2.60E-05	5.61E-05	0.12	0.07	0.1	0.02	0	0
OsR498G0511052200.01	3.233	1.093	-1.57762	6.87E-12	1.13E-11	3.15	3.09	3.46	0.95	1.06	1.27
OsR498G0511052800.01	1.863	0.766	-1.18906	9.95E-13	1.60E-12	1.55	2.14	1.9	0.65	0.9	0.75
OsR498G0511054500.01	69.36	19.743	-1.80975	0	0	72.12	68.11	67.85	21.13	19.23	18.87
OsR498G0511055300.01	48.2	5.363	-3.15433	0	0	46.41	48.38	49.81	5.13	5.72	5.24
OsR498G0511061800.01	22.82	48.043	1.07075	1.56E-216	6.06E-217	20.69	23.62	24.15	46.13	47.78	50.22
OsR498G0511065200.01	3.506	0.87	-1.98919	2.42E-17	3.47E-17	3.59	3.73	3.2	0.79	1.2	0.62
OsR498G0511068200.01	0.29	5.806	4.322314	7.56E-58	6.29E-58	0.2	0.35	0.32	5.83	5.44	6.15
OsR498G0511072500.01	8.91	18.806	1.065378	7.92E-54	6.83E-54	9.68	8.34	8.71	19.22	18.9	18.3
OsR498G0511074200.01	0.243	0	-5.76759	2.44E-08	4.54E-08	0.16	0.19	0.38	0	0	0
OsR498G0511074600.01	0.01	0.866	6.397312	2.95E-21	3.88E-21	0	0	0.03	1.09	0.71	0.8
OsR498G0511075200.01	13.173	3.75	-1.97513	9.18E-219	3.54E-219	11.51	12.93	15.08	3.44	3.49	4.32
OsR498G0511075400.01	3.65	12.956	2.162043	2.06E-246	7.31E-247	3.12	3.61	4.22	11.87	13.3	13.7
OsR498G0511077900.01	5.533	18.473	1.748569	1.38E-70	1.03E-70	5.62	5.28	5.7	19.44	15.98	20
OsR498G0511080700.01	6.276	13.913	1.140407	6.85E-88	4.53E-88	6.08	6.34	6.41	13.61	14.83	13.3
OsR498G0511086400.01	8.486	25.803	1.602239	6.78E-136	3.51E-136	8.37	8.64	8.45	25.43	26.19	25.79
OsR498G0511086600.01	63.083	19.286	-1.70504	7.27E-303	2.21E-303	66.36	58.16	64.73	19.04	19.07	19.75
OsR498G0511087000.01	0.81	0.35	-1.36234	0.00061	0.0014443	0.44	1.19	0.8	0.45	0.19	0.41
OsR498G0511087400.01	192	92.57	-1.06046	0	0	199.73	189.9	186.4	95.8	88.19	93.72
OsR498G0511087600.01	2.636	0.606	-2.30538	4.69E-25	5.75E-25	2.12	2.49	3.3	0.63	0.81	0.38
OsR498G0511088400.01	43.166	19.946	-1.11561	2.47E-155	1.18E-155	43.71	42.97	42.82	20.35	21.51	17.98
OsR498G0511090500.01	0.616	1.446	1.221963	6.10E-08	1.15E-07	0.54	0.52	0.79	1.47	1.87	1
OsR498G0511092000.01	22.923	6.073	-1.97377	9.53E-208	3.80E-208	23.37	23.23	22.17	5.67	6.2	6.35
OsR498G0511092300.01	0.11	3.906	5.002393	3.90E-82	2.68E-82	0.09	0.16	0.08	3.73	3.55	4.44
OsR498G0511096000.01	71.94	183.75	1.323133	0	0	69.33	75.49	71	191.62	179.02	180.6
OsR498G0511097100.01	19.626	113.08	2.512805	0	0	20.15	20.96	17.77	115.7	113.67	109.86
OsR498G0511097400.01	0.016	0.42	4.563322	4.91E-07	9.67E-07	0	0	0.05	0.43	0.28	0.55
OsR498G0511099700.01	30.75	66.553	1.080625	0	0	29.78	30.63	31.84	63.16	66.76	69.74
OsR498G0511100700.01	0.813	5.706	2.799809	6.23E-101	3.82E-101	0.86	0.82	0.76	5.47	6.08	5.57
OsR498G0511102000.01	608	143.5	-2.091	0	0	624.44	597.92	601.6	146.66	141.81	142.03
OsR498G0511102100.01	1.913	7.896	2.048635	5.32E-37	5.48E-37	2.55	1.85	1.34	9.14	6.35	8.2
OsR498G0511102300.01	56.473	8.08	-2.81792	6.02E-266	2.02E-266	53.52	56.41	59.49	9.59	5.52	9.13
OsR498G0511103500.01	0.143	0.39	1.511828	5.36E-08	1.01E-07	0.16	0.19	0.08	0.33	0.46	0.38
OsR498G0511103800.01	1.17	3.226	1.47081	2.91E-12	4.74E-12	1.3	1.49	0.72	3.73	2.47	3.48
OsR498G0511105200.01	17.82	159.4	3.15193	0	0	17.34	18.28	17.84	171	152.9	154.29
OsR498G0511106300.01	6.853	25.106	1.871224	5.53E-259	1.89E-259	6.86	7.17	6.53	25.1	23.98	26.24
OsR498G0511111400.01	9.8	21.22	1.106054	1.58E-79	1.11E-79	8.84	9.45	11.11	21.62	21.84	20.2
OsR498G0511112500.01	3.47	1.503	-1.20877	1.00E-15	1.50E-15	3.36	3.26	3.79	2.01	1.24	1.26
OsR498G0511113100.01	0.39	2.193	2.492612	4.52E-39	4.55E-39	0.4	0.45	0.32	2.34	2.02	2.22
OsR498G0511113500.01	4.47	21.526	2.268713	0	0	4.09	5.1	4.22	22.1	21.28	21.2
OsR498G0511114100.01	0.083	1.396	3.90483	3.50E-23	4.43E-23	0.14	0.04	0.07	1.15	1.5	1.54

OsR498G0511114900.01	3.493	1.13	-1.6354	2.26E-17	3.24E-17	3.06	3.6	3.82	1.02	1.22	1.15
OsR498G0511118000.01	1.836	0.776	-1.24654	5.14E-07	1.01E-06	1.32	1.85	2.34	1.18	0.58	0.57
OsR498G0511118500.01	0.37	2.396	2.710114	1.38E-41	1.35E-41	0.47	0.3	0.34	2.53	2.29	2.37
OsR498G0511119100.01	1.843	4.003	1.047636	3.55E-24	4.41E-24	1.61	2.06	1.86	4.23	3.98	3.8
OsR498G0511119300.01	12.24	29.223	1.252648	2.83E-102	1.73E-102	11.79	13.27	11.66	32.84	28.59	26.24
OsR498G0511120900.01	6.453	22.493	1.794521	5.34E-263	1.81E-263	5.64	6.45	7.27	22.77	22.54	22.17
OsR498G0511123000.01	9.633	3.786	-1.3839	2.67E-46	2.49E-46	10.55	9.01	9.34	3.58	4.25	3.53
OsR498G0511130900.01	69.456	148.95	1.093728	0	0	70	69.01	69.36	153.51	141.61	151.74
OsR498G0511136400.01	1.626	4.433	1.438309	2.48E-17	3.54E-17	1.71	1.51	1.66	4.45	3.84	5.01
OsR498G0511137600.01	0	1.593	6.288826	7.18E-11	1.22E-10	0	0	0	1.62	1.52	1.64
OsR498G0511142400.01	0.05	1.71	5.024653	3.65E-24	4.54E-24	0.05	0	0.1	1.59	1.97	1.57
OsR498G0511143000.01	0.12	25.756	7.705096	0	0	0.09	0.05	0.22	27.32	24.13	25.82
OsR498G0511154300.01	23.63	10.256	-1.22858	3.03E-245	1.08E-245	25.05	22.13	23.71	10.15	10.44	10.18
OsR498G0511155500.01	10.71	48.986	2.178578	0	0	10.05	10.36	11.72	48.5	49.71	48.75
OsR498G0511158300.01	8.03	3.73	-1.11436	3.06E-51	2.70E-51	8.64	7.37	8.08	3.78	3.62	3.79
OsR498G0511158800.01	2.653	16.293	2.617292	7.46E-257	2.57E-257	2.39	2.92	2.65	16.03	16.61	16.24
OsR498G0511160200.01	1.883	4.133	1.148492	1.57E-14	2.40E-14	2.22	1.67	1.76	4.85	4.4	3.15
OsR498G0511161200.01	2.456	6.906	1.409194	9.51E-41	9.40E-41	2.57	2.45	2.35	6.57	6.58	7.57
OsR498G0511165600.01	23.833	65.593	1.457164	0	0	24.05	23.21	24.24	62.9	65.54	68.34
OsR498G0511166900.01	0.4	0.043	-3.41967	6.57E-08	1.24E-07	0.35	0.4	0.45	0	0.09	0.04
OsR498G0511167100.01	20.136	9.48	-1.06688	7.88E-68	6.03E-68	20.24	20.43	19.74	8.95	9.84	9.65
OsR498G0511169600.01	0.66	0.173	-1.93599	1.19E-07	2.27E-07	0.74	0.46	0.78	0.1	0.32	0.1
OsR498G0511172100.01	6.06	0.74	-3.0389	3.64E-83	2.48E-83	5.77	5.9	6.51	0.75	0.74	0.73
OsR498G0511173500.01	14.616	33.2	1.116961	3.34E-172	1.50E-172	15.43	13.67	14.75	32.89	33.69	33.02
OsR498G0511179700.01	1.483	6.6	2.188715	5.35E-83	3.65E-83	1.65	1.45	1.35	6.69	6.56	6.55
OsR498G0511180700.01	2.216	0.866	-1.36097	8.06E-20	1.09E-19	2.41	1.94	2.3	0.85	0.95	0.8
OsR498G0511182600.01	1.163	0	-5.81822	1.42E-08	2.62E-08	1.56	0.89	1.04	0	0	0
OsR498G0511182700.01	31.58	11.14	-1.693	1.15E-179	5.04E-180	34.04	30.15	30.55	10.67	11.06	11.69
OsR498G0511186000.01	19.866	9.396	-1.10801	1.09E-68	8.31E-69	20.36	20.23	19.01	9.71	8.51	9.97
OsR498G0511186500.01	4.93	1.23	-2.94811	6.70E-118	3.77E-118	5.41	4.53	4.85	1.06	1.1	1.53
OsR498G0511186800.01	8.956	0.103	-6.46428	1.21E-47	1.11E-47	10.3	8.06	8.51	0.31	0	0
OsR498G0511190900.01	24.346	9.25	-1.3474	4.82E-237	1.75E-237	24.47	23.17	25.4	9.58	9.82	8.35
OsR498G0511193300.01	2.033	5.49	1.42789	1.03E-21	1.34E-21	2.32	2.05	1.73	5.54	5.88	5.05
OsR498G0511194100.01	20.25	50.873	1.340877	0	0	19.94	20.5	20.31	48.67	52.57	51.38
OsR498G0511195200.01	36.26	167.58	2.201248	0	0	35.22	37.15	36.41	163.49	168.45	170.8
OsR498G0511196100.01	7.113	2.276	-1.56335	4.66E-37	4.80E-37	7.85	7.23	6.26	2.38	1.98	2.47
OsR498G0511202800.01	3.576	1.47	-1.28884	1.42E-70	1.06E-70	3.23	3.55	3.95	1.42	1.45	1.54
OsR498G0511203100.01	10.236	4.266	-1.31211	4.02E-73	2.95E-73	9.81	11.14	9.76	4.44	3.74	4.62
OsR498G0511203900.01	0.21	0.043	-2.34907	0.0001266	0.0002849	0.12	0.24	0.27	0.03	0.1	0
OsR498G0511204600.01	0.6	0.156	-2.01469	1.92E-07	3.71E-07	0.46	0.54	0.8	0.25	0.11	0.11
OsR498G0511207000.01	5.846	40.383	2.785977	0	0	4.73	6.23	6.58	41.2	40.78	39.17
OsR498G0511211300.01	1.26	3	1.238741	1.76E-10	3.02E-10	1.12	1.12	1.54	2.6	3.3	3.1
OsR498G0511211900.01	7.25	16.556	1.147956	4.57E-79	3.20E-79	7.95	6.87	6.93	15.9	15.99	17.78
OsR498G0511212100.01	8.626	2.726	-1.66687	2.86E-86	1.91E-86	9.66	7.99	8.23	2.75	3.12	2.31
OsR498G0511216000.01	0.143	0.566	1.999118	1.72E-05	3.66E-05	0.14	0.15	0.14	0.67	0.59	0.44
OsR498G0511218000.01	1.153	77.17	6.046186	0	0	0.74	1.21	1.51	84.54	77.93	69.04
OsR498G0511218600.01	0.053	14.296	8.086107	7.45E-230	2.78E-230	0.08	0.04	0.04	16.06	12.63	14.2
OsR498G0511218800.01	0.353	13.91	5.071876	0	0	0.43	0.31	0.32	13.95	14.48	13.3
OsR498G0511219800.01	0	3.573	9.045553	2.42E-50	2.16E-50	0	0	0	3.73	3.44	3.55
OsR498G0511221800.01	1.576	0.143	-3.455	6.26E-25	7.69E-25	1.37	1.67	1.69	0.15	0.16	0.12
OsR498G0511225400.01	13.186	36.25	1.449839	1.07E-138	5.49E-139	13.09	13.85	12.62	36.2	37.06	35.49
OsR498G0511228200.01	65.19	329.07	2.370984	0	0	64.93	65.71	64.93	324.49	332.93	329.8
OsR498G0511231300.01	4.443	35.996	3.010816	0	0	4.43	4.05	4.85	35.5	37.12	35.37
OsR498G0511234500.01	0.72	5.853	3.010614	4.83E-35	5.10E-35	0.86	0.72	0.58	6.18	5.89	5.49
OsR498G0511236100.01	5.053	15.666	1.619428	1.31E-152	6.34E-153	5.01	4.92	5.23	15.1	16.33	15.57
OsR498G0511245900.01	13.71	29.12	1.048267	1.94E-139	9.90E-140	13.71	13.69	13.73	29.2	29.12	29.04
OsR498G0511247700.01	0.083	0.693	3.009386	8.49E-10	1.50E-09	0.08	0.09	0.08	0.67	0.71	0.7
OsR498G0511248100.01	14.4	6.496	-1.14101	1.10E-66	8.50E-67	14.11	14.94	14.15	6.3	6.09	7.1
OsR498G0511248800.01	3.616	1.346	-1.44567	4.46E-10	7.80E-10	4.22	2.72	3.91	1.15	1.54	1.35
OsR498G0511249100.01	9.626	1.64	-2.56228	1.83E-96	1.15E-96	9.27	9.76	9.85	1.58	1.73	1.61
OsR498G0511249700.01	0.643	0.203	-2.18263	1.52E-11	2.54E-11	0.54	0.71	0.68	0.18	0.32	0.11
OsR498G0511251800.01	1.626	4.043	1.313325	2.14E-27	2.51E-27	1.47	1.87	1.54	4.11	4.2	3.82
OsR498G0511253300.01	8.813	159.27	4.16613	0	0	9.83	7.92	8.69	164.72	155.83	157.25
OsR498G0511254000.01	24.426	48.826	1.008923	1.67E-149	8.17E-150	26.43	24.17	22.68	50.25	48.09	48.14
OsR498G0511255300.01	0.23	1.93	3.079288	1.83E-27	2.14E-27	0.15	0.28	0.26	2.46	1.83	1.5
OsR498G0511257600.01	0.396	0.863	1.097093	3.92E-05	8.54E-05	0.3	0.5	0.39	0.76	0.86	0.97
OsR498G0511264900.01	4.59	21.036	2.18797	0	0	4.47	4.61	4.69	20.3	22.5	20.31
OsR498G0511265600.01	3.006	1.18	-1.35358	1.10E-22	1.41E-22	3.14	3.14	2.74	1.27	1.13	1.14
OsR498G0511266600.01	10.253	23.136	1.168738	7.66E-110	4.47E-110	10.59	9.67	10.5	24.77	22.14	22.5

OsR498G0511266700.01	5.886	29.893	2.3155	1.37E-87	9.11E-88	1.49	6.06	10.11	31.32	28.13	30.23
OsR498G0511267100.01	1.276	28.556	4.460752	0	0	1.52	0.66	1.65	29.14	28.96	27.57
OsR498G0511267500.01	19.753	57.123	1.480602	1.16E-214	4.53E-215	21.87	18.09	19.3	59.19	58.08	54.1
OsR498G0511269300.01	17.24	8.226	-1.08217	5.53E-51	4.91E-51	17.12	16.75	17.85	8.89	7.86	7.93
OsR498G0511270300.01	1.723	0.77	-1.23708	5.22E-05	0.0001145	1.53	1.34	2.3	0.83	0.6	0.88
OsR498G0511270900.01	3.606	67.7	4.151376	0	0	3.73	3.47	3.62	66.88	68.95	67.27
OsR498G0511271700.01	0	1.39	7.361688	8.41E-20	1.14E-19	0	0	0	1.59	1.43	1.15
OsR498G0511272700.01	2.306	0.02	-6.8306	2.92E-28	3.37E-28	2.24	2.29	2.39	0	0	0.06
OsR498G0511276400.01	3.2	1.153	-1.54018	1.81E-22	2.33E-22	3.11	3.22	3.27	0.79	1.37	1.3
OsR498G0511282400.01	0	0.246	5.127223	8.48E-06	1.78E-05	0	0	0	0.17	0.31	0.26
OsR498G0511283000.01	0.233	15.4	6.067666	3.87E-250	1.36E-250	0.13	0.36	0.21	16.61	14.25	15.34
OsR498G0511286100.01	0.18	5.793	5.03976	2.36E-09	4.22E-09	0	0.54	0	6.62	6.95	3.81
OsR498G0511286400.01	89.436	39.53	-1.22188	5.72E-210	2.27E-210	93.54	86.95	87.82	41.25	36.25	41.09
OsR498G0511287500.01	1.086	3.33	1.573873	9.41E-53	8.20E-53	0.89	1.23	1.14	3.12	3.33	3.54
OsR498G0511288600.01	0.383	1.013	1.372368	0.0009647	0.0023195	0.18	0.29	0.68	0.97	0.92	1.15
OsR498G0511289000.01	85.266	0.316	-8.0839	0	0	84.22	86.33	85.25	0.14	0.25	0.56
OsR498G0511289600.01	430.4	203.68	-1.08055	0	0	424.36	427.5	439.3	200.61	204.46	205.98
OsR498G0511291100.01	5.653	18.256	1.693545	1.98E-83	1.35E-83	6.93	5.09	4.94	18.28	18	18.49
OsR498G0511291300.01	2.696	0.89	-1.59601	1.03E-23	1.29E-23	2.84	2.83	2.42	1.13	0.64	0.9
OsR498G0511292500.01	4.116	28.04	2.762991	0	0	4.78	3.61	3.96	31.85	27.04	25.23
OsR498G0511293200.01	0.24	11.76	5.605142	1.17E-193	4.88E-194	0.25	0.22	0.25	12.62	10.95	11.71
OsR498G0511294900.01	0.883	0.06	-3.86713	7.00E-08	1.32E-07	1.06	0.74	0.85	0.09	0.09	0
OsR498G0511295400.01	1.85	9.006	2.298547	1.75E-154	8.36E-155	1.96	1.91	1.68	8.68	9.39	8.95
OsR498G0511296000.01	0	0.223	4.236682	0.0009874	0.0023758	0	0	0	0.32	0.35	0
OsR498G0511296500.01	0.256	15.596	6.037577	0	0	0.21	0.3	0.26	15.39	15.95	15.45
OsR498G0511302400.01	7.99	1.213	-2.72055	7.17E-190	3.04E-190	8.4	7.51	8.06	1.26	1.17	1.21
OsR498G0511302800.01	1.633	0.13	-3.66068	5.77E-12	9.49E-12	1.31	2.06	1.53	0.1	0	0.29
OsR498G0511303200.01	9.023	24.36	1.430335	2.58E-247	9.13E-248	8.37	9.68	9.02	24.4	24.68	24
OsR498G0511303400.01	2.63	16.43	2.640319	9.90E-167	4.52E-167	2.73	2.77	2.39	18.62	16.25	14.42
OsR498G0511307800.01	0.38	32.33	6.392613	5.38E-291	1.69E-291	0.38	0.32	0.44	34.06	32.6	30.33
OsR498G0511308800.01	0.08	0.326	2.191763	1.43E-06	2.88E-06	0.05	0.09	0.1	0.37	0.26	0.35
OsR498G0511311000.01	0.096	0.433	2.155237	8.50E-05	0.0001889	0.19	0.05	0.05	0.44	0.36	0.5
OsR498G0511313100.01	5.68	2.76	-1.04285	4.76E-25	5.83E-25	6.21	5.72	5.11	2.7	2.7	2.88
OsR498G0511318800.01	7.576	100.07	3.712712	0	0	6.75	7.83	8.15	105.82	95.61	98.77
OsR498G0511319100.01	9.44	68.81	2.873417	0	0	10.22	9.19	8.91	67.95	68.02	70.46
OsR498G0511319700.01	0.9	23.573	4.70698	0	0	0.99	0.86	0.85	23.5	22.41	24.81
OsR498G0511321100.01	1.89	4.37	1.189026	4.16E-25	5.09E-25	1.97	1.55	2.15	4.58	4.47	4.06
OsR498G0511324000.01	15.623	32.65	1.061988	2.97E-161	1.38E-161	15.32	16.04	15.51	32.56	30.88	34.51
OsR498G0511325000.01	38.37	223.98	2.536301	0	0	37.85	38.77	38.49	211.7	230.12	230.12
OsR498G0511325400.01	4.53	1.786	-1.35135	1.65E-19	2.26E-19	4.57	3.93	5.09	1.43	1.81	2.12
OsR498G0511331900.01	7.043	2.863	-1.29127	1.50E-45	1.41E-45	6.17	8.06	6.9	2.66	2.67	3.26
OsR498G0511332700.01	1776.3	399.44	-2.1613	0	0	1844.7	1772.8	1711	438.47	376.24	383.6
OsR498G0511334000.01	14.89	35.823	1.279317	1.11E-197	4.58E-198	15.18	14.98	14.51	34.71	36.31	36.45
OsR498G0511334500.01	6.923	16.236	1.401494	8.74E-99	5.42E-99	7.84	6.84	6.09	15.42	17.58	15.71
OsR498G0511334800.01	1.473	23.18	3.948679	6.01E-117	3.40E-117	1.8	1.03	1.59	23.27	24.32	21.95
OsR498G0511335100.01	8.133	55.056	2.755265	0	0	7.97	8.23	8.2	57.82	55.32	52.03
OsR498G0511339600.01	20.173	9.41	-1.10531	2.69E-59	2.21E-59	21.27	20.8	18.45	8.7	8.91	10.62
OsR498G0511339900.01	134.4	284.63	1.079443	0	0	132.93	136.27	134	291.2	279.18	283.51
OsR498G0511341800.01	7.676	25.343	1.719777	6.96E-194	2.90E-194	6.3	8.57	8.16	26.2	24.7	25.13
OsR498G0511342200.01	0	1.366	6.946651	1.08E-15	1.60E-15	0	0	0	1.28	1.18	1.64
OsR498G0511343400.01	9.836	24.15	1.292471	1.78E-253	6.18E-254	9.36	10.01	10.14	22.79	23.96	25.7
OsR498G0511346700.01	0.403	0.033	-3.4838	4.22E-06	8.70E-06	0.66	0.31	0.24	0	0.05	0.05
OsR498G0511347700.01	24.243	111.35	2.174273	2.03E-217	7.86E-218	23.05	24.58	25.1	117.32	103.65	113.07
OsR498G0511348700.01	23.56	51.216	1.104321	3.60E-243	1.29E-243	21.66	24.07	24.95	51.14	50.47	52.04
OsR498G0511349200.01	8.646	50.09	2.531112	0	0	7.5	10.03	8.41	50.83	48.35	51.09
OsR498G0511354100.01	0.176	5.913	5.019328	1.40E-149	6.81E-150	0.18	0.05	0.3	6.08	6.2	5.46
OsR498G0511356300.01	2.27	0.45	-2.41962	8.44E-19	1.17E-18	2.64	2.04	2.13	0.76	0.28	0.31
OsR498G0511356600.01	8.276	0.993	-3.14611	2.93E-64	2.31E-64	8.53	8.31	7.99	1.39	1.02	0.57
OsR498G0511358800.01	12.826	3.266	-1.99028	5.63E-126	3.04E-126	12.63	12.9	12.95	3.4	3.35	3.05
OsR498G0511359200.01	3.693	1.803	-1.11301	2.34E-27	2.76E-27	3.32	3.91	3.85	1.71	1.69	2.01
OsR498G0511359300.01	20.83	0.136	-7.25301	1.40E-145	6.95E-146	22.33	19.85	20.31	0.41	0	0
OsR498G0511363600.01	3.47	1.733	-1.02011	1.94E-06	3.93E-06	3.13	3.03	4.25	2.09	1.43	1.68
OsR498G0511364400.01	6.653	1.913	-1.8111	3.10E-29	3.54E-29	7.09	6.34	6.53	1.81	1.94	1.99
OsR498G0511364900.01	17.94	0.353	-5.49483	3.11E-184	1.34E-184	17.65	17.4	18.77	0.18	0.44	0.44
OsR498G0511366500.01	10.096	25.126	1.313066	4.30E-147	2.13E-147	9.86	10	10.43	25.26	25.28	24.84
OsR498G0511372100.01	61.023	12.176	-2.3287	0	0	63.51	59.05	60.51	13.33	12.12	11.08
OsR498G0511372500.01	0.323	3.483	3.411729	3.69E-35	3.90E-35	0.52	0.12	0.33	3.5	3.37	3.58
OsR498G0511373500.01	0.273	4.733	4.107505	8.28E-49	7.52E-49	0.31	0.33	0.18	5.39	4.36	4.45
OsR498G0511374600.01	3.61	18.586	2.360416	2.09E-149	1.03E-149	3.94	3.73	3.16	20.3	17.83	17.63

OsR498G0511376600.01	0.033	0.26	2.965759	3.62E-08	6.78E-08	0.02	0.04	0.04	0.28	0.23	0.27
OsR498G0511379100.01	1.946	0.84	-1.23204	7.23E-07	1.43E-06	2.11	2.03	1.7	0.9	0.74	0.88
OsR498G0511380000.01	139.73	30.663	-2.19152	0	0	138.91	139.81	140.5	32.13	29.99	29.87
OsR498G0511383100.01	40.99	17.83	-1.21171	1.84E-81	1.27E-81	39.7	42.52	40.75	17.53	18.49	17.47
OsR498G0511383800.01	182.52	14.523	-3.65554	0	0	180.09	182.78	184.7	13.79	14.26	15.52
OsR498G0511384200.01	0.016	11.793	9.566509	1.11E-245	3.94E-246	0	0.05	0	13.06	10.47	11.85
OsR498G0511391200.01	5.99	13.843	1.205022	1.24E-56	1.04E-56	6.12	6.11	5.74	14.22	12.92	14.39
OsR498G0511391600.01	7.45	397.32	5.727564	0	0	8.09	7.16	7.1	414.34	394.74	382.89
OsR498G0511392000.01	0.326	2.356	2.825256	1.06E-22	1.36E-22	0.21	0.27	0.5	2.17	2.68	2.22
OsR498G0511394400.01	0	0.553	5.624723	1.50E-07	2.88E-07	0	0	0	0.41	0.42	0.83
OsR498G0511395000.01	5.123	2.166	-1.23405	2.80E-25	3.41E-25	5.3	5.14	4.93	1.79	2.09	2.62
OsR498G0511400300.01	0.593	0.04	-4.0477	6.68E-09	1.22E-08	0.65	0.63	0.5	0.06	0	0.06
OsR498G0511401000.01	442.25	132.57	-1.75367	0	0	443.73	447.89	435.1	144.46	128.91	124.34
OsR498G0511403400.01	9.69	1.966	-2.29387	3.85E-138	1.98E-138	9.82	10.24	9.01	2.01	1.87	2.02
OsR498G0511404900.01	16.726	5.923	-1.42229	4.81E-107	2.86E-107	17.18	16.28	16.72	6.07	6.21	5.49
OsR498G0511405400.01	48.623	19.59	-1.31934	1.61E-163	7.44E-164	49.39	49.31	47.17	19.46	19.19	20.12
OsR498G0511409400.01	25.42	2.663	-3.26232	1.24E-282	3.99E-283	23.99	24.97	27.3	2.88	2.52	2.59
OsR498G0511410700.01	0	5.616	9.592199	1.02E-67	7.81E-68	0	0	0	6.09	5.66	5.1
OsR498G0511410900.01	0.046	1.136	4.550722	9.98E-28	1.17E-27	0.06	0	0.08	1.29	1.07	1.05
OsR498G0511411500.01	7.036	1.27	-2.47098	7.12E-88	4.71E-88	6.33	7.53	7.25	1.49	1.1	1.22
OsR498G0511411800.01	0.2	4.966	4.630362	2.66E-84	1.80E-84	0.08	0.21	0.31	4.64	5.37	4.89
OsR498G0511411900.01	0.02	0.233	3.574569	0.0005212	0.0012259	0.06	0	0	0.11	0.27	0.32
OsR498G0511415200.01	0.106	0.523	2.276799	6.51E-06	1.36E-05	0.09	0.19	0.04	0.51	0.44	0.62
OsR498G0511415500.01	0.043	0.796	4.169043	1.91E-09	3.41E-09	0.07	0	0.06	1.01	0.9	0.48
OsR498G0511416300.01	1.593	0.59	-1.44899	1.60E-07	3.08E-07	2.03	1.32	1.43	0.45	0.63	0.69
OsR498G0511417200.01	0.236	0.586	1.309849	0.0004155	0.0009709	0.21	0.3	0.2	0.46	0.74	0.56
OsR498G0511418600.01	9.266	3.05	-1.5983	6.49E-117	3.67E-117	8.76	9.39	9.65	2.9	3.11	3.14
OsR498G0511420500.01	0	0.19	5.127223	8.48E-06	1.78E-05	0	0	0	0.39	0.07	0.11
OsR498G0511420700.01	0.276	0.993	1.793672	0.0001234	0.0002776	0.18	0.1	0.55	0.88	1.21	0.89
OsR498G0511421500.01	0.423	0.026	-4.0477	6.68E-09	1.22E-08	0.58	0.32	0.37	0	0	0.08
OsR498G0511422000.01	15.896	52.123	1.702413	0	0	16.15	15.98	15.56	51.79	53.51	51.07
OsR498G0511423600.01	4.106	0.526	-2.97081	2.16E-46	2.01E-46	3.68	4	4.64	0.41	0.52	0.65
OsR498G0511425700.01	0.023	0.213	3.325162	4.65E-09	8.41E-09	0.02	0.03	0.02	0.29	0.12	0.23
OsR498G0511426100.01	0.153	3.076	4.371274	4.82E-69	3.65E-69	0.25	0.12	0.09	3.19	3	3.04
OsR498G0511426300.01	203.02	651.92	1.671467	0	0	191.25	206.89	210.9	670.27	643.36	642.14
OsR498G0511426500.01	0	2.213	7.683616	1.05E-23	1.31E-23	0	0	0	2.62	2.02	2
OsR498G0511427500.01	2.473	0.753	-1.7764	3.36E-45	3.16E-45	2.45	2.36	2.61	0.75	0.76	0.75
OsR498G0511428000.01	12.42	26.166	1.103382	1.41E-92	9.07E-93	13.53	11.78	11.95	25.9	26.29	26.31
OsR498G0511428600.01	0.71	0.013	-5.63267	1.43E-25	1.73E-25	0.62	0.82	0.69	0.02	0	0.02
OsR498G0511430400.01	7.336	1.036	-2.77715	8.49E-94	5.41E-94	7.13	7.62	7.26	1.33	0.92	0.86
OsR498G0511430900.01	8.963	2.053	-2.12875	1.36E-58	1.12E-58	8.21	9.78	8.9	2.47	1.61	2.08
OsR498G0511432600.01	0.993	0	-7.15006	5.00E-18	7.04E-18	1.09	1.23	0.66	0	0	0
OsR498G0511434200.01	4.21	25.59	2.60035	0	0	4.2	4.27	4.16	26.08	24.9	25.79
OsR498G0511435300.01	33.526	16.406	-1.03584	1.69E-117	9.50E-118	35.42	32.41	32.75	17.41	16.64	15.17
OsR498G0511435600.01	2.876	0.073	-5.17677	1.02E-95	6.42E-96	2.86	3.06	2.71	0.04	0.12	0.06
OsR498G0511436500.01	11.926	1.373	-3.11937	0	0	11.97	12.43	11.38	1.41	1.33	1.38
OsR498G0511436800.01	47.796	5.2	-3.20664	0	0	47.33	48.05	48.01	5.02	4.67	5.91
OsR498G0511441300.01	0.72	3.23	2.14164	2.98E-22	3.85E-22	0.75	0.44	0.97	4.09	2.91	2.69
OsR498G0511444000.01	260.24	120.01	-1.1211	0	0	257.43	260.81	262.5	118.06	119.45	122.52
OsR498G0511444700.01	4.2	0.956	-2.14545	2.49E-39	2.50E-39	3.88	4.83	3.89	0.72	1.09	1.06
OsR498G0511446800.01	3.933	10.53	1.414338	6.42E-59	5.30E-59	4.33	3.78	3.69	10.72	10.15	10.72
OsR498G0511448600.01	0.153	1.783	3.54226	5.34E-35	5.65E-35	0.24	0.13	0.09	1.98	1.6	1.77
OsR498G0511449000.01	81.746	219.7	1.433998	0	0	77.86	88.76	78.62	228.8	211.35	218.96
OsR498G0511449400.01	0.226	0.016	-3.81822	1.26E-07	2.41E-07	0.28	0.31	0.09	0	0.02	0.03
OsR498G0511449600.01	9.03	3.496	-1.31735	2.02E-106	1.20E-106	6.6	10.3	10.19	3.45	3.28	3.76
OsR498G0511452700.01	53.54	145.78	1.445958	0	0	52.66	54.06	53.9	148.43	143.36	145.56
OsR498G0511456100.01	33.803	12.766	-1.39139	7.92E-159	3.72E-159	33.97	33.1	34.34	12.53	12.55	13.22
OsR498G0511460400.01	12.313	4.87	-1.30128	1.06E-132	5.56E-133	12.55	12.64	11.75	4.97	5.23	4.41
OsR498G0511460800.01	0.33	0.09	-1.86713	0.0001398	0.0003156	0.23	0.38	0.38	0.13	0.07	0.07
OsR498G0611468200.01	8716.2	1667.5	-2.40994	0	0	8942.4	8642.9	8563	1787.5	1615.3	1599.7
OsR498G0611468300.01	2.483	0.396	-3.06125	8.22E-10	1.45E-09	3.9	1.06	2.49	0.5	0.62	0.07
OsR498G0611469100.01	23.766	54.976	1.206453	1.02E-278	3.31E-279	23.01	23.99	24.3	59.5	54.05	51.38
OsR498G0611470900.01	17.373	49.883	1.49249	0	0	17.35	18.75	16.02	50.15	49.76	49.74
OsR498G0611471700.01	1.596	4.293	1.413735	4.31E-10	7.53E-10	2.14	1.43	1.22	4.3	4.1	4.48
OsR498G0611473500.01	24.86	8.233	-1.56132	1.83E-269	6.08E-270	23.88	25.01	25.69	7.28	7.92	9.5
OsR498G0611475800.01	4.79	1.34	-1.81097	5.78E-40	5.75E-40	4.8	4.96	4.61	1.6	1.41	1.01
OsR498G0611477700.01	6.33	1.536	-2.12024	2.90E-82	1.99E-82	6.14	6.46	6.39	1.79	1.67	1.15
OsR498G0611478000.01	15.17	35.026	1.210068	1.54E-193	6.44E-194	15.42	14.82	15.27	35.29	36.35	33.44
OsR498G0611478800.01	0.146	0.703	2.115048	1.48E-08	2.73E-08	0.05	0.16	0.23	0.7	0.59	0.82

OsR498G0611482700.01	3.19	1.066	-1.59467	4.99E-22	6.47E-22	3.52	3.01	3.04	1.22	1.02	0.96
OsR498G0611482900.01	5.75	0.62	-3.19881	2.01E-95	1.27E-95	6.04	5.42	5.79	0.64	0.56	0.66
OsR498G0611484500.01	0.876	0.106	-3.08952	1.12E-13	1.75E-13	0.72	0.86	1.05	0	0.2	0.12
OsR498G0611486100.01	0.566	1.9	1.757989	6.11E-11	1.04E-10	0.84	0.29	0.57	1.68	1.78	2.24
OsR498G0611487900.01	16.866	3.38	-2.28334	1.52E-263	5.13E-264	16.96	16.89	16.75	3.72	3.44	2.98
OsR498G0611488400.01	0.736	0.053	-4.31779	1.11E-10	1.91E-10	1.24	0.61	0.36	0.11	0.05	0
OsR498G0611490100.01	1.413	0	-7.67449	3.42E-24	4.25E-24	1.41	1.31	1.52	0	0	0
OsR498G0611490300.01	9.85	3.716	-1.49535	1.40E-54	1.20E-54	9.91	9.53	10.11	3.5	3.63	4.02
OsR498G0611491500.01	22.863	5.62	-2.0338	8.09E-146	4.02E-146	24.16	22.92	21.51	5.41	5.62	5.83
OsR498G0611493500.01	1.283	0.233	-2.41396	2.79E-18	3.91E-18	1.57	1.2	1.08	0.22	0.24	0.24
OsR498G0611493700.01	40.663	14.996	-1.44946	3.27E-261	1.11E-261	41.52	40.38	40.09	14.82	15.79	14.38
OsR498G0611495800.01	14.623	6.8	-1.10968	4.90E-115	2.79E-115	14.9	14.36	14.61	6.85	6.5	7.05
OsR498G0611496000.01	8.72	19.906	1.178387	4.86E-190	2.06E-190	8.95	8.61	8.6	19.6	19.8	20.32
OsR498G0611497000.01	2.683	6.76	1.310348	2.69E-90	1.76E-90	2.75	2.53	2.77	6.95	6.41	6.92
OsR498G0611498500.01	0.306	0.836	1.443227	1.02E-09	1.81E-09	0.35	0.21	0.36	0.85	0.87	0.79
OsR498G0611500200.01	0.463	0.076	-2.6232	0.0001807	0.000411	0.66	0.23	0.5	0	0.08	0.15
OsR498G0611504100.01	3.226	0.916	-1.8456	1.59E-26	1.90E-26	3.46	2.97	3.25	1.02	1.01	0.72
OsR498G0611506700.01	0.51	0.076	-2.57915	7.37E-08	1.40E-07	0.51	0.5	0.52	0.06	0.1	0.07
OsR498G0611507200.01	7.71	0.803	-3.34271	1.56E-120	8.65E-121	6.77	7.88	8.48	1.11	0.51	0.79
OsR498G0611507600.01	95.793	13.45	-2.82767	0	0	97.25	89.91	100.2	14.22	12.76	13.37
OsR498G0611508100.01	0.216	1.453	2.711907	1.21E-21	1.57E-21	0	0.54	0.11	1.18	1.89	1.29
OsR498G0611510400.01	1036.6	483.24	-1.11519	0	0	1064.5	1038.6	1007	509.25	465.15	475.32
OsR498G0611510600.01	13.44	5.98	-1.15995	1.75E-57	1.46E-57	13.81	13.44	13.07	6.49	5.11	6.34
OsR498G0611513600.01	9.203	3.433	-1.43014	6.41E-53	5.57E-53	9.56	8.68	9.37	3.27	3.5	3.53
OsR498G0611514600.01	1.523	34.336	4.479405	9.51E-303	2.90E-303	1.43	1.37	1.77	37.39	33.82	31.8
OsR498G0611515500.01	0.013	0.196	3.946651	6.37E-05	0.0001407	0	0	0.04	0.27	0.2	0.12
OsR498G0611518800.01	1.243	27.76	4.462551	1.77E-128	9.47E-129	1.08	1.4	1.25	29.82	23.49	29.97
OsR498G0611524600.01	0.193	0.04	-2.18263	5.34E-05	0.0001174	0.2	0.26	0.12	0.04	0.06	0.02
OsR498G0611527900.01	3.21	8.486	1.377929	3.24E-10	5.64E-10	1.98	3.82	3.83	9.26	8.96	7.24
OsR498G0611538400.01	0.483	0.24	-1.4212	5.33E-06	1.10E-05	1.05	0.2	0.2	0.25	0.36	0.11
OsR498G0611538700.01	0.573	0	-6.88932	1.43E-15	2.13E-15	0	0.84	0.88	0	0	0
OsR498G0611544300.01	1.27	0	-6.35256	1.58E-11	2.64E-11	1.36	0.96	1.49	0	0	0
OsR498G0611547100.01	0.213	0	-4.5452	0.0002138	0.0004889	0.21	0.22	0.21	0	0	0
OsR498G0611547600.01	6.78	36.413	2.384766	0	0	7.09	6.89	6.36	36.01	35.96	37.27
OsR498G0611548300.01	0.59	1.266	1.131468	7.57E-05	0.0001677	0.76	0.88	0.13	0.98	1.17	1.65
OsR498G0611548600.01	37.313	6.363	-2.57392	0	0	35.89	38.41	37.64	6.79	6.55	5.75
OsR498G0611548700.01	3.456	1.276	-1.42606	7.44E-31	8.30E-31	3.54	3.36	3.47	1.5	1.27	1.06
OsR498G0611549900.01	0.106	0	-5.28217	2.04E-06	4.14E-06	0.02	0.18	0.12	0	0	0
OsR498G0611552700.01	0.033	0.463	4.209685	1.01E-05	2.14E-05	0	0.1	0	0.58	0.51	0.3
OsR498G0611553000.01	1.236	0.62	-1.02533	5.56E-07	1.10E-06	1.12	1.35	1.24	0.55	0.71	0.6
OsR498G0611555700.01	0.55	0.22	-1.32014	3.54E-06	7.26E-06	0.47	0.58	0.6	0.24	0.18	0.24
OsR498G0611556900.01	8.296	3.933	-1.08201	9.02E-18	1.28E-17	9.14	9.14	6.61	4.11	4.96	2.73
OsR498G0611557700.01	8.606	61.036	2.860954	0	0	8.33	9.21	8.28	62.57	61.05	59.49
OsR498G0611558100.01	0.15	9.95	6.0649	7.99E-126	4.32E-126	0	0.29	0.16	9.06	11.14	9.65
OsR498G0611558300.01	0.343	0.036	-3.18263	1.16E-06	2.33E-06	0.4	0.42	0.21	0	0.11	0
OsR498G0611559300.01	6.666	0.526	-3.64708	4.85E-104	2.93E-104	6.38	6.72	6.9	0.68	0.47	0.43
OsR498G0611560500.01	0.05	1.43	4.847115	7.15E-15	1.08E-14	0.15	0	0	1.28	1.86	1.15
OsR498G0611562900.01	0.14	0.023	-2.71513	8.51E-06	1.79E-05	0.09	0.23	0.1	0.02	0.02	0.03
OsR498G0611565400.01	416.19	2113.3	2.27846	0	0	423.37	407.75	417.5	2316.1	2025.5	1998.2
OsR498G0611566100.01	15.816	6.183	-1.42181	2.10E-109	1.23E-109	15.5	16.4	15.55	5.7	6.39	6.46
OsR498G0611569000.01	17.92	90.82	2.340016	0	0	16.65	18.93	18.18	97.39	89.32	85.75
OsR498G0611569100.01	0.64	3.033	2.223936	3.96E-35	4.18E-35	0.95	0.44	0.53	3.21	3.31	2.58
OsR498G0611573000.01	0.853	0.066	-4.15006	2.89E-17	4.14E-17	0.53	0.83	1.2	0.13	0.07	0
OsR498G0611574800.01	6.9	14.276	1.04667	5.14E-61	4.15E-61	6.95	7.34	6.41	15.33	14.12	13.38
OsR498G0611575200.01	3.626	1.606	-1.23	5.14E-15	7.77E-15	4.1	3.29	3.49	1.77	1.65	1.4
OsR498G0611575400.01	20.913	6.276	-1.73947	3.79E-110	2.21E-110	20.96	21.06	20.72	7.92	5.69	5.22
OsR498G0611576400.01	0.176	1	2.600475	1.94E-10	3.34E-10	0.15	0.33	0.05	0.85	1.28	0.87
OsR498G0611577100.01	2.523	1.23	-1.05757	5.53E-18	7.80E-18	2.56	2.66	2.35	1.22	1.25	1.22
OsR498G0611577400.01	7.96	3.38	-1.20789	1.19E-14	1.81E-14	7.14	8.64	8.1	2.9	3.67	3.57
OsR498G0611578100.01	0.203	46.41	7.790634	2.91E-234	1.07E-234	0.12	0.13	0.36	49.89	44.58	44.76
OsR498G0611579000.01	0.99	128.63	7.029133	0	0	0.71	1.58	0.68	135.63	121.21	129.06
OsR498G0611579900.01	0.58	0.013	-5.51483	9.14E-13	1.47E-12	0.74	0.39	0.61	0	0	0.04
OsR498G0611581900.01	2.57	1.026	-1.41569	2.55E-13	4.03E-13	2.15	2.99	2.57	1.31	0.87	0.9
OsR498G0611582200.01	328.1	133.98	-1.29573	0	0	332.57	322.75	329	131.57	137.63	132.73
OsR498G0611582700.01	17.28	41.543	1.260991	4.11E-223	1.56E-223	15.58	18.57	17.69	38.74	42.04	43.85
OsR498G0611583900.01	0.683	0	-5.76759	2.44E-08	4.54E-08	0.48	0.82	0.75	0	0	0
OsR498G0611587800.01	8.256	26.583	1.680018	7.02E-144	3.52E-144	8.03	8.94	7.8	25.94	25.41	28.4
OsR498G0611588800.01	74.88	27.783	-1.4517	0	0	77.71	74.29	72.64	26.27	29.43	27.65
OsR498G0611592000.01	2.626	0.983	-1.37211	2.48E-29	2.83E-29	2.55	2.94	2.39	1.09	0.78	1.08

OsR498G0611594100.01	9.94	3.73	-1.45656	1.66E-68	1.26E-68	9.71	9.71	10.4	4.22	3.04	3.93
OsR498G0611599500.01	0.85	2.716	1.665595	1.18E-16	1.71E-16	0.88	0.69	0.98	3.36	2.55	2.24
OsR498G0611602200.01	16.15	49.87	1.620889	0	0	15.82	15.88	16.75	50.29	49.88	49.44
OsR498G0611603000.01	0.196	4.576	4.502311	3.22E-106	1.92E-106	0.11	0.15	0.33	5.36	4.32	4.05
OsR498G0611603800.01	68.11	2.12	-5.01747	0	0	71.44	66.07	66.82	1.61	2.87	1.88
OsR498G0611606000.01	0.443	0	-5.0477	1.13E-05	2.39E-05	0.54	0.41	0.38	0	0	0
OsR498G0611608500.01	5.256	1.8	-1.53144	3.53E-33	3.82E-33	4.95	6.15	4.67	1.64	1.89	1.87
OsR498G0611608900.01	110.56	35.51	-1.69541	0	0	113.15	109.72	108.8	35.97	35.01	35.55
OsR498G0611610500.01	0.116	0.493	2.387683	3.08E-09	5.53E-09	0.13	0	0.22	0.42	0.49	0.57
OsR498G0611611000.01	3.166	12.833	2.037392	3.69E-168	1.68E-168	3.25	3.21	3.04	13.78	12.82	11.9
OsR498G0611614200.01	0.56	0	-6.57524	4.70E-13	7.47E-13	0.42	0.6	0.66	0	0	0
OsR498G0611616300.01	0.173	0	-5.0477	1.13E-05	2.39E-05	0.12	0.25	0.15	0	0	0
OsR498G0611617300.01	0.033	0.413	3.624723	2.24E-11	3.75E-11	0	0.05	0.05	0.48	0.37	0.39
OsR498G0611617900.01	0	0.13	4.499192	0.0003163	0.0007323	0	0	0	0.21	0.04	0.14
OsR498G0611619900.01	2.063	0.08	-4.72105	6.42E-111	3.73E-111	2.07	2.04	2.08	0.07	0.1	0.07
OsR498G0611621200.01	164.05	67.676	-1.2974	0	0	167.89	163.14	161.1	68.64	66.76	67.63
OsR498G0611623700.01	0.01	0.93	6.562224	1.81E-23	2.29E-23	0.03	0	0	0.98	0.83	0.98
OsR498G0611624700.01	0.21	12.213	5.843891	1.75E-108	1.03E-108	0.16	0.17	0.3	12.93	11.45	12.26
OsR498G0611625100.01	5.733	2.08	-1.47319	6.09E-19	8.43E-19	5.73	5.67	5.8	2.21	1.99	2.04
OsR498G0611627500.01	0.883	0.21	-1.65212	4.15E-05	9.06E-05	0.57	0.85	1.23	0.25	0.21	0.17
OsR498G0611630700.01	9.48	2.206	-2.26131	1.93E-93	1.23E-93	8.99	9.5	9.95	2.33	2.14	2.15
OsR498G0611631000.01	0.403	0.953	1.399243	2.11E-07	4.08E-07	0.41	0.41	0.39	1.36	0.91	0.59
OsR498G0611632500.01	51.686	169.96	1.713019	0	0	51.18	50.94	52.94	170.1	165.05	174.74
OsR498G0611633400.01	0.07	0.403	2.454798	0.0006489	0.0015399	0.07	0	0.14	0.45	0.46	0.3
OsR498G0611633600.01	8.58	3.916	-1.15429	5.89E-29	6.76E-29	9.18	8.6	7.96	3.27	4.46	4.02
OsR498G0611633800.01	0.77	1.613	1.076286	1.34E-08	2.46E-08	0.88	0.86	0.57	2.05	1.36	1.43
OsR498G0611636200.01	1	2.17	1.11191	3.77E-09	6.79E-09	0.88	1.01	1.11	2.69	2.22	1.6
OsR498G0611636900.01	3.053	1.193	-1.38802	1.74E-27	2.04E-27	3.55	3.12	2.49	0.88	1.42	1.28
OsR498G0611637700.01	33.24	13.81	-1.29025	1.01E-114	5.76E-115	36.64	32.03	31.05	13.74	13.82	13.87
OsR498G0611639500.01	24.513	2.273	-3.52	0	0	23.99	24.05	25.5	1.97	2.04	2.81
OsR498G0611643200.01	19.53	8.47	-1.25896	2.45E-78	1.73E-78	19.11	20.53	18.95	9.02	8.23	8.16
OsR498G0611645500.01	3.006	10.57	1.807543	5.53E-85	3.72E-85	3.06	2.55	3.41	11.01	10.24	10.46
OsR498G0611648200.01	0.126	0.756	2.570275	2.59E-09	4.64E-09	0.21	0.13	0.04	0.8	0.63	0.84
OsR498G0611650500.01	34.376	93.45	1.42701	0	0	32.79	34.98	35.36	93.86	93.37	93.12
OsR498G0611650800.01	3.36	11.643	1.786365	1.08E-165	4.95E-166	3.39	3.23	3.46	11	12.31	11.62
OsR498G0611651500.01	4.693	2.143	-1.13592	5.71E-16	8.44E-16	4.96	4.74	4.38	1.92	2.09	2.42
OsR498G0611652400.01	0.043	4.863	6.897741	2.03E-55	1.73E-55	0	0.13	0	4.7	5.54	4.35
OsR498G0611654200.01	114.79	22.116	-2.34626	0	0	115.2	113.87	115.3	24.42	20.36	21.57
OsR498G0611656300.01	144.3	7.326	-4.31427	0	0	136.51	151.44	144.9	7.51	8.29	6.18
OsR498G0611656900.01	120.92	18.283	-2.73332	0	0	121.8	124.14	116.8	19.47	17.07	18.31
OsR498G0611658300.01	0.49	8.62	4.138749	1.08E-164	4.97E-165	0.26	0.65	0.56	8.92	7.72	9.22
OsR498G0611659400.01	0.276	0.78	1.342829	2.86E-13	4.53E-13	0.29	0.2	0.34	0.98	0.64	0.72
OsR498G0611662800.01	12.093	24.52	1.012793	1.39E-54	1.19E-54	13.55	11.08	11.65	26.94	21.52	25.1
OsR498G0611665000.01	75.413	10.743	-2.81573	0	0	74.57	76.43	75.24	10.18	10.55	11.5
OsR498G0611665800.01	1.206	0.23	-2.43829	6.00E-07	1.19E-06	1.21	0.88	1.53	0.29	0.2	0.2
OsR498G0611668700.01	0.963	0.186	-2.38329	3.01E-18	4.22E-18	0.97	0.94	0.98	0.17	0.19	0.2
OsR498G0611672200.01	0	0.41	5.683616	8.53E-08	1.62E-07	0	0	0	0.48	0.3	0.45
OsR498G0611673700.01	53.14	10.733	-2.30651	0	0	54.61	52.02	52.79	10.33	10.89	10.98
OsR498G0611674100.01	1.743	0.75	-1.30263	9.32E-10	1.65E-09	1.78	1.58	1.87	0.63	0.77	0.85
OsR498G0611677500.01	1.04	15.066	3.929373	2.20E-262	7.46E-263	1.24	1.15	0.73	13.59	15.26	16.35
OsR498G0611679700.01	14.87	7.426	-1.00204	5.12E-96	3.22E-96	13.91	16.2	14.5	7.22	7.64	7.42
OsR498G0611681900.01	2.413	1.1	-1.13016	1.11E-12	1.78E-12	2.19	2.79	2.26	1.44	1.21	0.65
OsR498G0611682700.01	1.806	4.076	1.164456	5.94E-17	8.58E-17	1.92	1.54	1.96	4.48	3.92	3.83
OsR498G0611684100.01	12.156	32.353	1.441321	9.31E-194	3.89E-194	12.26	11.73	12.48	31.34	32.28	33.44
OsR498G0611685200.01	0.566	0.23	-1.32281	7.59E-05	0.0001682	0.6	0.43	0.67	0.32	0.17	0.2
OsR498G0611686700.01	5.14	2.46	-1.06716	1.21E-16	1.76E-16	5.05	5.61	4.76	2.14	2.44	2.8
OsR498G0611687100.01	2.113	1.05	-1.01469	7.46E-07	1.48E-06	2.24	2.48	1.62	1.15	1.18	0.82
OsR498G0611687600.01	4.92	47.213	3.263957	0	0	5.32	4.7	4.74	44.69	48.12	48.83
OsR498G0611687800.01	0.4	4.713	3.563322	1.11E-154	5.32E-155	0.44	0.35	0.41	4.46	4.81	4.87
OsR498G0611698300.01	69.293	17.963	-1.94847	0	0	67.39	68.76	71.73	17.29	19.21	17.39
OsR498G0611698500.01	3.386	1.11	-1.23326	3.64E-21	4.80E-21	3.43	3.14	3.59	1.27	1.07	0.99
OsR498G0611699100.01	33.76	93.013	1.455377	0	0	32.34	33.58	35.36	98.78	89.44	90.82
OsR498G0611702900.01	0.15	0.013	-3.41967	0.0009442	0.002269	0.16	0.21	0.08	0	0	0.04
OsR498G0611707700.01	0.053	0.006	-3.41967	0.0009442	0.002269	0.01	0.05	0.1	0	0.02	0
OsR498G0611712100.01	5.453	2.646	-1.04372	2.30E-21	3.01E-21	5.12	5.2	6.04	1.99	3.22	2.73
OsR498G0611713700.01	24.516	62.286	1.277041	6.47E-297	2.01E-297	24.43	25.42	23.7	61.62	63.2	62.04
OsR498G0611714100.01	11.7	62.62	1.965602	0	0	10.95	11.56	12.59	62.19	64.69	60.98
OsR498G0611716600.01	1.516	0.053	-4.76759	1.35E-39	1.35E-39	1.51	1.64	1.4	0.04	0.06	0.06
OsR498G0611717400.01	0.033	0.75	4.433398	0.0003419	0.000793	0	0	0.1	0	0.82	1.43

OsR498G0611718500.01	0.796	2.76	1.77059	4.41E-10	7.71E-10	2.39	0	0	1.96	3.29	3.03
OsR498G0611719000.01	5.743	1.913	-1.67466	1.11E-67	8.48E-68	6.27	5.56	5.4	1.54	1.79	2.41
OsR498G0611719200.01	1.6	6.053	1.918824	6.01E-65	4.71E-65	1.65	1.78	1.37	5.57	6.19	6.4
OsR498G0611722000.01	6.696	1.683	-2.02193	7.76E-47	7.19E-47	6.7	7.01	6.38	1.43	1.89	1.73
OsR498G0611728600.01	0.646	1.376	1.077235	4.48E-05	9.78E-05	0.51	0.64	0.79	1.25	1.5	1.38
OsR498G0611732100.01	3.883	0.963	-2	6.96E-74	5.07E-74	3.93	3.83	3.89	0.7	0.95	1.24
OsR498G0611733800.01	0.376	0.996	1.312033	1.29E-05	2.74E-05	0.35	0.15	0.63	1.23	1.08	0.68
OsR498G0611737800.01	17.443	64.713	1.867702	1.14E-147	5.59E-148	19.57	14	18.76	71.44	67.74	54.96
OsR498G0611738100.01	0.616	0.133	-2.20125	6.96E-08	1.32E-07	0.53	0.67	0.65	0.18	0.11	0.11
OsR498G0611738500.01	3.886	0.076	-5.67449	5.61E-75	4.05E-75	3.36	4.02	4.28	0.04	0.08	0.11
OsR498G0611739100.01	1.056	0	-6.45209	3.47E-12	5.67E-12	1.56	0.52	1.09	0	0	0
OsR498G0611740600.01	0.39	0.94	1.262152	3.69E-07	7.23E-07	0.39	0.38	0.4	0.73	1.11	0.98
OsR498G0611741100.01	0.266	1.216	2.155237	8.50E-05	0.0001889	0.4	0.14	0.26	1.39	1.13	1.13
OsR498G0611743600.01	1.663	0.843	-1.01411	5.77E-09	1.05E-08	1.6	1.6	1.79	0.88	0.76	0.89
OsR498G0611744100.01	0.08	0.266	1.731638	1.91E-05	4.08E-05	0.07	0.1	0.07	0.32	0.23	0.25
OsR498G0611746400.01	1.09	0.006	-7.14015	5.73E-34	6.13E-34	0.79	1.2	1.28	0	0	0.02
OsR498G0611748000.01	0.113	0	-4.96024	2.02E-05	4.33E-05	0.06	0.07	0.21	0	0	0
OsR498G0611748600.01	0.153	0.723	2.252754	4.11E-08	7.71E-08	0.25	0.13	0.08	1.05	0.56	0.56
OsR498G0611752400.01	20.296	10.106	-1.00723	3.19E-81	2.20E-81	20.35	20.69	19.85	9.74	9.9	10.68
OsR498G0611757800.01	0.656	0.166	-2.03063	6.55E-13	1.05E-12	0.64	0.51	0.82	0.2	0.16	0.14
OsR498G0611761500.01	0.18	23.476	7.016222	0	0	0.12	0.12	0.3	26.22	22.17	22.04
OsR498G0611762100.01	9.35	21.656	1.220803	5.76E-118	3.24E-118	9.53	9.78	8.74	20.81	21.67	22.49
OsR498G0611763000.01	1.733	0.026	-6.06876	7.11E-18	1.01E-17	1.53	1.65	2.02	0.08	0	0
OsR498G0611763200.01	20.88	60.486	1.493653	0	0	20.66	21.43	20.55	58.82	62.03	60.61
OsR498G0611765100.01	65.683	31.99	-1.04369	3.20E-233	1.18E-233	66.69	62.83	67.53	32.22	30.3	33.45
OsR498G0611769500.01	1.533	0.256	-2.71923	6.00E-15	9.08E-15	2.03	1.27	1.3	0.44	0.11	0.22
OsR498G0611772600.01	52.78	15.22	-1.77718	0	0	50.12	54.05	54.17	14.98	15.08	15.6
OsR498G0611777900.01	3.596	8.54	1.190861	2.15E-28	2.48E-28	3.68	3.82	3.29	8.55	8.75	8.32
OsR498G0611780800.01	3.06	1.273	-1.27544	3.45E-19	4.75E-19	3.08	3.04	3.06	1.3	1.47	1.05
OsR498G0611782500.01	2.053	10.653	2.364905	1.68E-294	5.25E-295	1.95	2.07	2.14	10.3	10.6	11.06
OsR498G0611782900.01	261.37	81.663	-1.68356	0	0	268.1	260.8	255.2	81.4	79.07	84.52
OsR498G0611788200.01	10.663	0.536	-4.3205	1.09E-116	6.20E-117	10.31	11.08	10.6	0.55	0.63	0.43
OsR498G0611789800.01	3.916	0.573	-2.8255	1.45E-170	6.52E-171	3.14	4	4.61	0.51	0.68	0.53
OsR498G0611790800.01	66.363	17.543	-1.92617	0	0	68.55	64.63	65.91	17.86	17.57	17.2
OsR498G0611790900.01	2.02	0.48	-2.67765	3.78E-52	3.31E-52	1.81	2	2.25	0.48	0.57	0.39
OsR498G0611792500.01	5.073	13.233	1.353463	2.91E-60	2.37E-60	5.11	5.1	5.01	13.59	13.46	12.65
OsR498G0611793200.01	1.756	22.63	3.787589	0	0	1.71	1.47	2.09	21.91	24.17	21.81
OsR498G0611794600.01	0.476	3.396	2.827352	6.08E-25	7.46E-25	0.61	0.36	0.46	3.39	3.7	3.1
OsR498G0611795300.01	0.746	12.103	4.014748	2.58E-135	1.34E-135	0.67	0.93	0.64	11.67	12.9	11.74
OsR498G0611795800.01	1.856	67.04	5.256129	0	0	1.88	2.11	1.58	70.75	64.97	65.4
OsR498G0611798900.01	0.643	0.153	-2.07572	0.0001433	0.0003236	0.59	0.77	0.57	0.15	0.31	0
OsR498G0611800600.01	0.563	2.593	2.200752	3.69E-21	4.87E-21	0.47	0.73	0.49	2.56	2.65	2.57
OsR498G0611801500.01	3.89	0.28	-3.79759	2.62E-50	2.34E-50	4.2	4.15	3.32	0.24	0.35	0.25
OsR498G0611801700.01	5.16	1.67	-1.63267	8.71E-22	1.13E-21	5.06	5.76	4.66	1.85	1.65	1.51
OsR498G0611802900.01	0.393	0.056	-2.96024	1.12E-05	2.37E-05	0.33	0.56	0.29	0.05	0.06	0.06
OsR498G0611807100.01	6.603	105.37	4.188729	0	0	6.22	7.34	6.25	102.53	107.64	105.94
OsR498G0611814100.01	8.546	1.873	-2.24377	2.52E-59	2.07E-59	9.66	8.5	7.48	2.3	1.61	1.71
OsR498G0611816700.01	0.836	0.12	-2.83471	3.45E-05	7.49E-05	0.56	1.3	0.65	0.12	0.24	0
OsR498G0611817500.01	3.196	0.79	-2.01682	1.44E-23	1.81E-23	3.17	3.31	3.11	0.68	0.99	0.7
OsR498G0611822700.01	5.186	21.693	2.065035	0	0	5.3	5.16	5.1	20.24	22.49	22.35
OsR498G0611823300.01	0	1.046	6.287688	7.29E-11	1.24E-10	0	0	0	1.14	1.17	0.83
OsR498G0611824500.01	0.5	0.22	-1.206	6.84E-06	1.43E-05	0.45	0.38	0.67	0.15	0.21	0.3
OsR498G0611826900.01	0.063	8.9	7.137792	2.23E-126	1.20E-126	0.09	0.1	0	9.58	8.51	8.61
OsR498G0611836500.01	0.526	3.153	2.792101	2.72E-46	2.54E-46	0.32	0.76	0.5	2.59	3.05	3.82
OsR498G0611841300.01	1.816	0.283	-2.72577	3.85E-22	4.98E-22	1.53	1.95	1.97	0.47	0.16	0.22
OsR498G0611846200.01	0.463	0.17	-1.44567	0.0001697	0.0003852	0.59	0.48	0.32	0.27	0.07	0.17
OsR498G0611849700.01	0.516	1.68	1.656431	1.49E-05	3.17E-05	0.31	0.33	0.91	1.62	2.1	1.32
OsR498G0611850100.01	0.623	4.13	2.712185	1.05E-40	1.04E-40	0.66	0.39	0.82	4.73	3.95	3.71
OsR498G0611853300.01	0.656	24.153	5.186117	0	0	0.69	0.45	0.83	24.46	23.04	24.96
OsR498G0611854600.01	0.026	0.626	4.624723	8.04E-13	1.29E-12	0	0.04	0.04	0.69	0.52	0.67
OsR498G0611854800.01	0.026	13.68	9.078679	3.75E-199	1.54E-199	0.08	0	0	14.25	12.92	13.87
OsR498G0611856400.01	2.896	25.9	3.155047	0	0	2.72	2.94	3.03	25.62	26.33	25.75
OsR498G0611857200.01	7.593	250.69	4.984851	0	0	8.06	7.6	7.12	265.09	244.95	242.04
OsR498G0611857400.01	1.3	68.51	5.724629	0	0	1.37	1.47	1.06	73.48	67.07	64.98
OsR498G0611857600.01	4.62	140.6	4.910957	0	0	4.78	4.44	4.64	147.7	136.13	137.96
OsR498G0611857800.01	0	0.256	5.7402	4.87E-08	9.17E-08	0	0	0	0.2	0.27	0.3
OsR498G0611858400.01	16.813	3.603	-2.22509	2.25E-95	1.42E-95	16.62	18.25	15.57	4.33	2.66	3.82
OsR498G0611858600.01	3.053	0.69	-2.15922	5.98E-39	6.02E-39	2.64	2.77	3.75	0.51	0.8	0.76
OsR498G0611862900.01	4.25	0.493	-3.12615	4.78E-63	3.80E-63	4.64	4.09	4.02	0.43	0.53	0.52

OsR498G0611865600.01	0.22	4.406	4.316918	5.28E-79	3.70E-79	0.18	0.27	0.21	4.57	4.85	3.8
OsR498G0611867800.01	0.036	0.14	2.209685	0.0006752	0.0016052	0.04	0.03	0.04	0.2	0.03	0.19
OsR498G0611870000.01	0.603	0.21	-1.5452	0.0002414	0.0005545	0.55	0.53	0.73	0.15	0.32	0.16
OsR498G0611874100.01	10.456	4.696	-1.22618	4.25E-37	4.37E-37	11.07	11.11	9.19	4.1	5.04	4.95
OsR498G0611877100.01	1.693	0.63	-1.43502	4.47E-14	6.93E-14	1.58	1.71	1.79	0.6	0.68	0.61
OsR498G0611880000.01	0.896	4.993	2.481866	4.14E-76	2.96E-76	0.97	1.04	0.68	5.22	5.01	4.75
OsR498G0611885800.01	1.75	0.573	-1.62558	8.40E-42	8.21E-42	1.77	1.66	1.82	0.5	0.5	0.72
OsR498G0611889400.01	188.7	39.993	-2.24401	0	0	174.07	201.94	190.1	39.69	38.47	41.82
OsR498G0611891900.01	0.19	0.01	-4.0631	6.59E-08	1.25E-07	0.14	0.18	0.25	0	0.03	0
OsR498G0611900200.01	18.203	1.696	-3.42554	0	0	18.14	18.19	18.28	1.54	1.1	2.45
OsR498G0611905300.01	3.686	1.78	-1.05576	2.19E-17	3.14E-17	4.42	3.45	3.19	1.39	2.03	1.92
OsR498G0611909200.01	0.97	0.406	-1.23708	5.22E-05	0.0001145	0.87	1.29	0.75	0.7	0.19	0.33
OsR498G0611910600.01	3.17	1.296	-1.28478	9.33E-24	1.17E-23	3.14	3.25	3.12	1.46	1.06	1.37
OsR498G0611911600.01	4.48	17.653	1.98058	2.85E-236	1.04E-236	4.17	4.62	4.65	17.88	18.02	17.06
OsR498G0611911800.01	5.13	2.566	-1.0747	1.52E-11	2.54E-11	5.1	5.52	4.77	2.84	2.75	2.11
OsR498G0611912000.01	2.78	15.9	2.365224	1.91E-108	1.12E-108	2.38	2.77	3.19	15.55	16.77	15.38
OsR498G0611912800.01	2.873	7.473	1.377948	4.20E-88	2.78E-88	2.82	3.01	2.79	7.16	7.6	7.66
OsR498G0611917700.01	0.366	0.06	-2.6232	3.00E-07	5.84E-07	0.26	0.48	0.36	0.06	0.06	0.06
OsR498G0611920000.01	1.106	21.163	4.254632	0	0	1.38	1.01	0.93	21.38	20.04	22.07
OsR498G0611923700.01	9.79	4.393	-1.22466	3.62E-17	5.21E-17	9.36	8.94	11.07	4.73	3.81	4.64
OsR498G0611933700.01	10.596	65.806	2.630364	0	0	10.32	11.13	10.34	63.97	66.67	66.78
OsR498G0611936300.01	1.096	0.133	-3.11552	1.53E-18	2.13E-18	1.06	1.05	1.18	0.17	0.15	0.08
OsR498G0611937400.01	0.13	0.833	2.64048	3.15E-21	4.14E-21	0.15	0.1	0.14	0.76	0.79	0.95
OsR498G0611939200.01	0.11	1.833	4.03976	2.33E-22	3.00E-22	0.07	0.07	0.19	2.4	1.62	1.48
OsR498G0611941700.01	31.483	6.5	-2.28112	0	0	31.27	31.55	31.63	6.36	6.84	6.3
OsR498G0611943700.01	0.803	2.416	1.597242	3.90E-26	4.68E-26	0.61	1.13	0.67	2.55	2.34	2.36
OsR498G0611945200.01	151.92	63.34	-1.26616	0	0	164.84	141.57	149.3	64.65	62.11	63.26
OsR498G0611947300.01	7.48	2.29	-1.72227	1.40E-29	1.59E-29	6.88	6.87	8.69	2.51	2.58	1.78
OsR498G0611947800.01	1.976	0.833	-1.24369	1.90E-11	3.18E-11	1.91	2.14	1.88	0.84	0.97	0.69
OsR498G0611949400.01	2.436	5.446	1.166785	5.04E-23	6.40E-23	2.14	3.07	2.1	5.83	5.3	5.21
OsR498G0611957000.01	0.77	0.133	-2.52792	2.96E-13	4.68E-13	0.61	0.87	0.83	0.11	0.09	0.2
OsR498G0611963700.01	0.7	1.78	1.331526	5.31E-06	1.10E-05	0.8	0.46	0.84	2	1.45	1.89
OsR498G0611965600.01	2.866	0.35	-2.98261	2.78E-44	2.65E-44	3.01	2.99	2.6	0.29	0.46	0.3
OsR498G0611966600.01	0.223	0.043	-2.37528	0.0009088	0.0021805	0.21	0.22	0.24	0.09	0.04	0
OsR498G0611967700.01	1.146	5.01	2.122842	6.81E-49	6.18E-49	1.17	1.11	1.16	5.85	4.95	4.23
OsR498G0611968200.01	38.243	15.526	-1.36862	1.23E-157	5.79E-158	40.45	37.46	36.82	16.89	15.27	14.42
OsR498G0611970300.01	0.04	0.886	4.499192	9.06E-17	1.31E-16	0.04	0.04	0.04	0.91	0.9	0.85
OsR498G0611970800.01	12.66	133.34	3.368352	0	0	16.45	10.38	11.15	131.73	136.88	131.41
OsR498G0611971500.01	0.226	0.486	1.094208	0.0004388	0.001027	0.21	0.22	0.25	0.53	0.36	0.57
OsR498G0611972300.01	3.88	1.686	-1.16506	2.64E-27	3.10E-27	4.04	4.06	3.54	1.65	1.85	1.56
OsR498G0611974900.01	1.09	0.366	-1.57495	9.29E-13	1.49E-12	1.06	1.27	0.94	0.36	0.37	0.37
OsR498G0611980100.01	0.113	0.646	2.624723	4.15E-07	8.14E-07	0.06	0	0.28	0.87	0.32	0.75
OsR498G0611980400.01	0.036	7.756	7.471606	9.53E-117	5.40E-117	0	0.07	0.04	7.84	7.82	7.61
OsR498G0611981600.01	6.77	1.683	-2.05488	1.13E-55	9.60E-56	6.46	6.31	7.54	1.69	1.62	1.74
OsR498G0611987200.01	12.63	3.836	-1.73798	2.14E-258	7.34E-259	12.06	13.33	12.5	3.98	3.74	3.79
OsR498G0611989000.01	286.14	130.77	-1.14187	0	0	301.15	278.55	278.7	145.16	127.39	119.75
OsR498G0611995400.01	33.83	10.01	-1.76156	0	0	34.2	34.66	32.63	9.45	10.38	10.2
OsR498G0611996700.01	27.663	60.7	1.122805	0	0	25.92	28.34	28.73	59.24	60.7	62.16
OsR498G0611997000.01	0.41	0.05	-2.92371	4.55E-08	8.55E-08	0.22	0.45	0.56	0.12	0	0.03
OsR498G0612000300.01	0.863	8.113	3.229585	3.87E-107	2.29E-107	0.72	1.05	0.82	8.27	8.04	8.03
OsR498G0612005300.01	0.16	6.973	5.388488	1.26E-40	1.25E-40	0.12	0	0.36	7.37	5.82	7.73
OsR498G0612008200.01	6.273	1.15	-2.44845	5.49E-139	2.81E-139	5.88	6.21	6.73	1.19	0.97	1.29
OsR498G0612009500.01	5.563	2.253	-1.32781	4.87E-35	5.14E-35	5.28	5.66	5.75	2.76	2.13	1.87
OsR498G0612010300.01	7.503	17.903	1.252204	1.33E-123	7.29E-124	6.57	8	7.94	18.85	17.47	17.39
OsR498G0612013800.01	4.386	20.58	2.227699	0	0	4.44	4.34	4.38	21.09	20.79	19.86
OsR498G0612015400.01	13.836	1.376	-3.34271	3.49E-94	2.22E-94	13.97	14.52	13.02	1.16	1.45	1.52
OsR498G0612016600.01	0.086	0.953	3.390257	4.50E-21	5.94E-21	0.05	0.03	0.18	0.85	1.22	0.79
OsR498G0612017700.01	0.01	0.13	3.624723	0.0004041	0.0009439	0.03	0	0	0.06	0.2	0.13
OsR498G0612020800.01	36.346	13.653	-1.4166	1.60E-208	6.34E-209	38.09	35.15	35.8	14.98	13.27	12.71
OsR498G0612021600.01	13.293	60.386	2.179772	0	0	13.28	13.69	12.91	67.53	55.17	58.46
OsR498G0612023100.01	0	0.983	4.361688	0.0005877	0.0013892	0	0	0	0.89	1.47	0.59
OsR498G0612024500.01	8.77	57.743	2.698236	0	0	9.13	8.91	8.27	56.47	57.52	59.24
OsR498G0612026500.01	1.786	7.903	2.127743	2.50E-81	1.73E-81	1.64	1.69	2.03	8.18	7.5	8.03
OsR498G0612027700.01	53.186	10.546	-2.3334	0	0	53.95	52.27	53.34	9.56	10.64	11.44
OsR498G0612031400.01	15.553	4.993	-1.68361	2.28E-119	1.27E-119	15.06	16.62	14.98	5.22	5.18	4.58
OsR498G0612033300.01	0.48	0.033	-3.91444	3.89E-08	7.29E-08	0.55	0.53	0.36	0.05	0.05	0
OsR498G0612038100.01	20.506	53.253	1.372239	0	0	19.42	20.21	21.89	51.52	53.35	54.89
OsR498G0612043200.01	6.476	34.656	2.41436	0	0	6.29	6.59	6.55	35.01	35.14	33.82
OsR498G0612054600.01	0.243	0.013	-4.20817	7.92E-06	1.66E-05	0.27	0.2	0.26	0	0.04	0

OsR498G0612055300.01	0.213	0.05	-2.18181	0.0001936	0.0004411	0.2	0.2	0.24	0.06	0.03	0.06
OsR498G0612058000.01	2.85	0.486	-2.55909	7.01E-12	1.16E-11	2.05	3.25	3.25	1.32	0.14	0
OsR498G0612058800.01	750.84	344.9	-1.13317	0	0	749.88	739.55	763.1	366.4	322.66	345.65
OsR498G0612059600.01	2.143	0.776	-1.46274	9.38E-10	1.66E-09	2.63	2.19	1.61	0.88	0.56	0.89
OsR498G0612066600.01	0.11	0.886	2.993956	2.14E-38	2.16E-38	0.13	0.12	0.08	0.9	0.81	0.95
OsR498G0612068000.01	0.033	0.476	3.914229	4.65E-11	7.87E-11	0	0.1	0	0.38	0.66	0.39
OsR498G0612068100.01	2.153	4.69	1.117321	3.45E-18	4.84E-18	2.46	1.97	2.03	4.24	4.5	5.33
OsR498G0612075300.01	108.74	18.886	-2.52673	0	0	105.33	112.25	108.6	18.96	19.31	18.39
OsR498G0612077800.01	2.186	1.013	-1.11552	4.55E-06	9.39E-06	1.93	2.58	2.05	0.95	1.19	0.9
OsR498G0612083600.01	4.376	0.226	-4.44406	6.15E-41	6.07E-41	2.91	5.97	4.25	0	0.17	0.51
OsR498G0612083900.01	1.406	4.966	1.814922	4.72E-41	4.65E-41	1.47	1.33	1.42	5.17	5.18	4.55
OsR498G0612084300.01	0.096	0.543	2.466025	1.02E-07	1.93E-07	0.07	0.08	0.14	0.52	0.69	0.42
OsR498G0612085100.01	7.746	2.966	-1.37368	4.46E-20	6.03E-20	8.63	6.83	7.78	3.1	2.95	2.85
OsR498G0612087300.01	36.536	10.366	-1.84906	1.33E-198	5.44E-199	38.29	35.95	35.37	11.08	9.17	10.85
OsR498G0612087700.01	5.013	1.986	-1.43383	6.71E-63	5.33E-63	4.16	5.4	5.48	1.98	1.77	2.21
OsR498G0612088200.01	0.15	0.426	1.525187	0.0009281	0.0022279	0.18	0.14	0.13	0.36	0.51	0.41
OsR498G0612090600.01	0.106	0.563	2.357106	5.82E-08	1.10E-07	0	0	0.32	0.8	0.47	0.42
OsR498G0612090700.01	0.27	1.35	2.311542	1.03E-15	1.54E-15	0.28	0.34	0.19	0.91	1.55	1.59
OsR498G0612116600.01	0.153	0	-4.5452	0.0002138	0.0004889	0.23	0.16	0.07	0	0	0
OsR498G0612131000.01	0.073	0.463	2.624723	3.28E-06	6.72E-06	0.13	0.05	0.04	0.32	0.7	0.37
OsR498G0612136200.01	0.193	0.956	2.2961	1.04E-12	1.67E-12	0.19	0.2	0.19	1.15	0.88	0.84
OsR498G0612143000.01	3.173	1.476	-1.11224	3.55E-26	4.26E-26	3.06	3.07	3.39	1.17	1.78	1.48
OsR498G0612146400.01	0.556	0.076	-3.06876	1.96E-13	3.08E-13	0.49	0.49	0.69	0.03	0.11	0.09
OsR498G0612148100.01	8.25	0.603	-3.78011	8.51E-117	4.81E-117	8.2	7.39	9.16	0.52	0.67	0.62
OsR498G0612148900.01	8.416	2.88	-1.53256	2.62E-42	2.55E-42	8.49	8.81	7.95	2.97	2.74	2.93
OsR498G0612153400.01	1.073	0.443	-1.28217	4.94E-05	0.0001082	0.86	1.07	1.29	0.38	0.34	0.61
OsR498G0612159500.01	91.61	305.88	1.7224	0	0	85.47	97.27	92.09	319.52	297.27	300.86
OsR498G0612160500.01	3.83	1.566	-1.41009	1.91E-32	2.08E-32	3.8	3.61	4.08	1.8	1.22	1.68
OsR498G0612161300.01	19.826	8.22	-1.26632	5.08E-235	1.86E-235	17.99	20.38	21.11	8.18	8.17	8.31
OsR498G0612162700.01	1.093	0.526	-1.05469	3.60E-11	6.06E-11	0.81	1.31	1.16	0.59	0.49	0.5
OsR498G0612166300.01	101.1	48.203	-1.10768	0	0	93.85	105.86	103.6	47.56	48.28	48.77
OsR498G0612167200.01	10.993	40.91	1.889335	0	0	9.97	10.83	12.18	44.2	38.97	39.56
OsR498G0612168000.01	11.97	1.92	-2.64292	1.95E-164	8.99E-165	11.38	12.95	11.58	2.12	2.16	1.48
OsR498G0612170100.01	3.37	59.08	4.04739	0	0	4.22	3.15	2.74	62.27	55.98	58.99
OsR498G0612176400.01	0.143	0.5	1.79719	2.37E-08	4.40E-08	0.14	0.17	0.12	0.5	0.58	0.42
OsR498G0612183000.01	0.146	0	-4.28217	0.0007127	0.0016975	0.31	0.09	0.04	0	0	0
OsR498G0612184400.01	0.623	0.02	-5.0477	1.30E-09	2.31E-09	0.72	0.46	0.69	0	0	0.06
OsR498G0612189500.01	2.1	17.12	3.063332	0	0	1.88	2.01	2.41	16.65	17.13	17.58
OsR498G0612190900.01	7.703	22.593	1.538438	0	0	7.72	7.76	7.63	22.58	22.26	22.94
OsR498G0612191400.01	0.743	0.17	-2.03824	8.29E-06	1.74E-05	0.98	0.64	0.61	0.34	0.12	0.05
OsR498G0612202200.01	3.976	1.71	-1.22077	1.91E-13	3.01E-13	4.12	4.43	3.38	1.52	1.68	1.93
OsR498G0612218200.01	0.306	0.146	-1.63831	0.0002956	0.0006829	0.38	0.15	0.39	0.29	0	0.15
OsR498G0612221700.01	0.126	0	-4.28217	0.0007127	0.0016975	0.11	0.2	0.07	0	0	0
OsR498G0612226600.01	0.29	0.126	-1.20417	0.0005981	0.0014149	0.33	0.28	0.26	0.06	0.18	0.14
OsR498G0612228200.01	0.873	0.07	-3.78042	2.40E-24	2.98E-24	0.81	0.92	0.89	0.09	0.05	0.07
OsR498G0612229300.01	18.09	37.71	1.049806	1.42E-159	6.64E-160	18.8	17.4	18.07	38.78	36.26	38.09
OsR498G0612234800.01	1.21	0.46	-1.34379	1.06E-15	1.58E-15	1.28	1.26	1.09	0.45	0.29	0.64
OsR498G0612251700.01	0.596	0.013	-5.3865	8.30E-12	1.37E-11	0.74	0.6	0.45	0.04	0	0
OsR498G0612253000.01	0.683	2	1.551406	1.33E-17	1.89E-17	0.52	0.73	0.8	1.88	1.8	2.32
OsR498G0612254400.01	1.923	18.426	3.232405	3.05E-94	1.94E-94	1.34	1.74	2.69	16.09	17.76	21.43
OsR498G0612260000.01	0.413	2.846	2.920178	1.71E-28	1.98E-28	0.3	0.32	0.62	2.18	3.24	3.12
OsR498G0612261200.01	0.28	7.126	4.7402	1.33E-91	8.61E-92	0.45	0.29	0.1	6.36	7.58	7.44
OsR498G0612262900.01	91.22	9.06	-3.34678	0	0	94.64	88.56	90.46	9.11	10.37	7.7
OsR498G0612264100.01	40.866	7.533	-2.44975	2.94E-245	1.04E-245	39.68	42.16	40.76	6.16	7.74	8.7
OsR498G0612264800.01	4.99	2.266	-1.15769	8.35E-10	1.47E-09	4.08	5.11	5.78	1.25	2.65	2.9
OsR498G0612266700.01	1.913	0.076	-4.66068	1.90E-13	2.98E-13	1.65	1.97	2.12	0	0	0.23
OsR498G0612267300.01	0.82	0.21	-1.96024	0.0001436	0.0003243	0.52	1.27	0.67	0	0.27	0.36
OsR498G0612271500.01	6.113	1.773	-1.78621	3.23E-50	2.89E-50	5.61	6.69	6.04	1.83	1.86	1.63
OsR498G0612271900.01	421.3	1096	1.377085	0	0	433.14	412.49	418.3	1074	1110	1104.1
OsR498G0612272400.01	24.373	625.56	4.669439	0	0	26.51	25.23	21.38	660.53	602.59	613.56
OsR498G0612273100.01	1.3	2.923	1.148284	5.21E-05	0.0001142	1.46	1.15	1.29	3.27	2.69	2.81
OsR498G0612274300.01	11.113	1.656	-2.75258	9.82E-74	7.17E-74	10.83	11.52	10.99	2.13	1.68	1.16
OsR498G0612275000.01	75.9	16.85	-2.18571	3.04E-290	9.60E-291	74.23	74.4	79.07	17.24	16.53	16.78
OsR498G0612275400.01	4.293	0.433	-3.42331	4.89E-75	3.53E-75	3.89	4.18	4.81	0.37	0.56	0.37
OsR498G0612275800.01	14.5	7.02	-1.05226	2.07E-47	1.91E-47	13.96	14.71	14.83	7.09	7.26	6.71
OsR498G0612275900.01	1316.5	96.243	-3.78061	0	0	1327.6	1302.9	1319	101.22	93.34	94.17
OsR498G0612281500.01	0.233	1.343	2.608603	7.16E-15	1.09E-14	0.2	0.25	0.25	1.46	1.24	1.33
OsR498G0612282000.01	31.186	5.646	-2.46493	3.12E-195	1.30E-195	31.87	32.75	28.94	6.45	6.17	4.32
OsR498G0612288800.01	21.033	8.153	-1.3774	1.08E-70	8.07E-71	18.51	22.18	22.41	7.93	8.51	8.02

OsR498G0612290300.01	68.606	12.923	-2.41156	0	0	71.44	66.56	67.82	13.44	12.89	12.44
OsR498G0612294900.01	14.83	30.976	1.046158	3.41E-111	1.98E-111	16.08	14.26	14.15	30.56	32.27	30.1
OsR498G0612296100.01	0.013	0.146	3.499192	0.0007491	0.0017869	0	0	0.04	0.2	0.2	0.04
OsR498G0612303700.01	0.23	0.016	-3.71513	4.06E-07	7.96E-07	0.23	0.24	0.22	0.05	0	0
OsR498G0612303900.01	0.22	0.57	1.372335	0.0003441	0.0007985	0.26	0.06	0.34	0.38	0.59	0.74
OsR498G0612304200.01	0.12	1.253	3.391515	9.49E-22	1.24E-21	0.36	0	0	1.33	1.07	1.36
OsR498G0612306400.01	2.346	6.703	1.507265	7.04E-18	9.96E-18	2.59	2.45	2	7.26	6.54	6.31
OsR498G0612308100.01	1.296	0.123	-3.37528	2.17E-19	2.98E-19	1.36	1.32	1.21	0.12	0.21	0.04
OsR498G0612309600.01	0.326	0.086	-2.20817	3.03E-06	6.20E-06	0.43	0.33	0.22	0.15	0.08	0.03
OsR498G0612310100.01	0.316	0.036	-3.13016	2.06E-06	4.17E-06	0.31	0.47	0.17	0	0.07	0.04
OsR498G0612310300.01	2.343	0.723	-1.91665	9.03E-18	1.28E-17	2.57	2.43	2.03	0.98	0.84	0.35
OsR498G0612311300.01	3.313	1.873	-1.02175	1.07E-22	1.36E-22	2.87	3.53	3.54	1.9	1.92	1.8
OsR498G0612311800.01	1.206	0.263	-2.19728	3.17E-17	4.56E-17	0.98	1.29	1.35	0.22	0.26	0.31
OsR498G0612311900.01	0.326	0.043	-2.96024	1.28E-09	2.27E-09	0.3	0.36	0.32	0.07	0.04	0.02
OsR498G0612314100.01	4.506	0.396	-3.51483	2.72E-37	2.79E-37	4.45	4.15	4.92	0.51	0.38	0.3
OsR498G0612317500.01	3.693	1.553	-1.2628	1.36E-17	1.94E-17	3.23	4.12	3.73	1.46	1.67	1.53
OsR498G0612318600.01	3.763	0.736	-2.40125	3.10E-62	2.48E-62	3.56	3.64	4.09	0.65	0.83	0.73
OsR498G0612321000.01	3.7	10.166	1.443357	2.68E-66	2.07E-66	3.36	3.6	4.14	10.42	10.11	9.97
OsR498G0612323800.01	3.34	0.046	-6.13683	4.42E-52	3.88E-52	4	3.18	2.84	0	0	0.14
OsR498G0612327800.01	0.793	2.553	1.644622	1.22E-19	1.67E-19	1.03	0.76	0.59	2.17	2.76	2.73
OsR498G0612331200.01	6.96	50.886	2.868514	0	0	7.8	6.9	6.18	53.89	49.64	49.13
OsR498G0612334700.01	0.33	0.74	1.218097	1.74E-08	3.22E-08	0.31	0.32	0.36	0.77	0.61	0.84
OsR498G0612340400.01	0.616	0.226	-1.45905	4.23E-06	8.71E-06	0.62	0.74	0.49	0.35	0.15	0.18
OsR498G0612354800.01	4.463	1.393	-1.70483	9.03E-86	6.05E-86	4.04	4.15	5.2	1.45	1.44	1.29
OsR498G0612358300.01	0.776	2.573	1.709611	9.23E-05	0.0002057	0.84	0.89	0.6	3.08	2.87	1.77
OsR498G0612358500.01	10.816	31.343	1.524037	0	0	10.9	11.09	10.46	29.86	32.46	31.71
OsR498G0612365000.01	13.536	2.196	-2.6334	2.87E-178	1.27E-178	13.41	13.75	13.45	2.15	2.33	2.11
OsR498G0612369500.01	26.843	9.46	-1.5204	7.62E-75	5.51E-75	27.82	23.14	29.57	10.65	9.47	8.26
OsR498G0612373900.01	15.056	5.87	-1.38231	4.98E-223	1.89E-223	14.33	15.37	15.47	5.85	6.02	5.74
OsR498G0612383300.01	6.833	1.986	-1.80333	4.80E-60	3.91E-60	6.58	6.97	6.95	2	2.33	1.63
OsR498G0612383600.01	99.413	12.2	-3.0406	0	0	104.32	96.72	97.2	14.57	11.03	11
OsR498G0612405100.01	1.276	0.366	-1.82274	2.00E-05	4.28E-05	2.09	1.1	0.64	0.2	0.4	0.5
OsR498G0612408600.01	0.373	13.53	5.299717	1.83E-201	7.44E-202	0.32	0.44	0.36	14.21	13.22	13.16
OsR498G0612409400.01	3.773	0.363	-3.31349	5.08E-77	3.61E-77	3.9	3.37	4.05	0.31	0.35	0.43
OsR498G0612410000.01	5.296	1.28	-2.16905	2.47E-66	1.91E-66	5.27	4.43	6.19	1.26	1.28	1.3
OsR498G0612412700.01	36.796	1.39	-5.22941	0	0	35.55	36.92	37.92	0.96	1.69	1.52
OsR498G0612416100.01	5.046	1.42	-1.84859	4.90E-18	6.90E-18	5.55	4.56	5.03	1.16	1.65	1.45
OsR498G0612416400.01	11.786	5.58	-1.33408	4.13E-41	4.06E-41	12.8	12.01	10.55	4.85	6.44	5.45
OsR498G0612423700.01	0.356	0.866	1.27478	2.53E-10	4.38E-10	0.21	0.41	0.45	0.77	0.93	0.9
OsR498G0612426800.01	5.63	77.86	3.79196	0	0	3.92	6.22	6.75	79.71	76.64	77.23
OsR498G0612429000.01	1.226	0.326	-1.91444	7.35E-08	1.39E-07	1.12	1.12	1.44	0.67	0.25	0.06
OsR498G0612442200.01	1.033	4.323	2.066945	9.64E-85	6.50E-85	0.84	1.39	0.87	4.24	4.4	4.33
OsR498G0612449600.01.'	0.603	1.25	1.03976	0.0007193	0.001714	0.97	0.41	0.43	1.33	1.37	1.05
OsR498G0612464700.01	3.306	1.28	-1.3201	1.40E-09	2.49E-09	3.9	3.34	2.68	1.64	1.04	1.16
OsR498G0612466400.01	0.56	1.883	1.73749	1.60E-13	2.51E-13	0.61	0.52	0.55	1.75	2.11	1.79
OsR498G0612478700.01	0.656	4.196	2.677834	2.79E-39	2.80E-39	0.68	0.72	0.57	5.43	3.6	3.56
OsR498G0612482600.01	2.38	0.243	-3.29322	2.80E-25	3.41E-25	1.84	2.9	2.4	0.22	0.23	0.28
OsR498G0612483400.01	5.7	2.81	-1.1227	9.25E-33	1.01E-32	5.37	6.16	5.57	3.22	2.5	2.71
OsR498G0612488000.01	81.69	572.76	2.792018	0	0	81.85	82.38	80.84	554.09	584.31	579.89
OsR498G0612489500.01	14.01	51.69	1.874862	0	0	14.25	14.39	13.39	50.23	51.46	53.38
OsR498G0612492500.01	1.623	0.323	-2.4	7.70E-35	8.16E-35	1.12	2.1	1.65	0.21	0.38	0.38
OsR498G0612499400.01	0.8	0	-6.91444	8.58E-16	1.27E-15	0.46	1.01	0.93	0	0	0
OsR498G0612507400.01	79.32	7.823	-3.29508	0	0	81.18	78.74	78.04	7.49	7.42	8.56
OsR498G0612512100.01	13.11	4.343	-1.61134	2.38E-34	2.54E-34	12.87	11.89	14.57	4.19	3.8	5.04
OsR498G0612514800.01	0.123	0	-4.5452	0.0002138	0.0004889	0.06	0.07	0.24	0	0	0
OsR498G0612522500.01	0.08	0.28	2.361688	2.29E-06	4.65E-06	0.11	0.06	0.07	0.22	0.22	0.4
OsR498G0612523700.01	2.28	5.446	1.253304	5.16E-34	5.53E-34	2.02	2.44	2.38	5.48	5.3	5.56
OsR498G0612525200.01	0.62	0.21	-1.55891	0.0002704	0.0006232	0.87	0.87	0.12	0.35	0.18	0.1
OsR498G0612529900.01	2.106	0.746	-1.32848	8.74E-23	1.12E-22	2.58	2.01	1.73	0.78	0.67	0.79
OsR498G0612533500.01	56.943	327.91	2.52189	0	0	55.76	56.74	58.33	320.19	329.19	334.34
OsR498G0612537000.01	7.48	2.856	-1.36905	2.35E-94	1.49E-94	6.46	7.89	8.09	2.39	2.91	3.27
OsR498G0612540200.01	0.04	0.31	3.127223	8.70E-08	1.65E-07	0.03	0.09	0	0.37	0.24	0.32
OsR498G0612546600.01	52.72	14.1	-1.89761	0	0	54.5	53.11	50.55	14.36	13.02	14.92
OsR498G0612550400.01	5.016	1.873	-1.3636	1.74E-21	2.27E-21	5.22	5.12	4.71	2.13	1.74	1.75
OsR498G0612552500.01	7.723	3.93	-1.07276	3.88E-35	4.10E-35	7.56	7.31	8.3	3.62	4.33	3.84
OsR498G0612553000.01	3.21	1.19	-1.44715	5.42E-40	5.39E-40	2.99	3.17	3.47	1.15	1.26	1.16
OsR498G0612571700.01	0.213	1.916	3.18415	8.01E-32	8.83E-32	0.1	0.38	0.16	2.05	1.91	1.79
OsR498G0612582000.01	4.58	2.213	-1.32222	1.15E-21	1.51E-21	4.22	4.66	4.86	1.95	2.06	2.63
OsR498G0612582600.01	0.433	0.11	-2.15289	2.08E-05	4.46E-05	0.42	0.46	0.42	0.23	0.05	0.05

OsR498G0612586700.01	0.76	2.326	1.597755	4.29E-10	7.50E-10	0.75	0.86	0.67	2.28	2.87	1.83
OsR498G0612587500.01	5.076	1.763	-1.49122	3.35E-25	4.09E-25	5.1	4.85	5.28	1.67	1.86	1.76
OsR498G0612588100.01	0.946	0.443	-1.09922	1.73E-11	2.88E-11	0.84	0.9	1.1	0.38	0.55	0.4
OsR498G0612589000.01	238.04	513.6	1.116165	0	0	237.7	238.49	237.9	519.41	503.36	518.02
OsR498G0612592000.01	0.036	0.386	3.499192	0.0007491	0.0017869	0	0.11	0	0.52	0.32	0.32
OsR498G0612594200.01	1.336	0.34	-1.96773	2.29E-21	3.01E-21	1.22	1.45	1.34	0.46	0.28	0.28
OsR498G0612611000.01	2.353	0.696	-1.6265	1.73E-07	3.34E-07	1.99	2.19	2.88	0.7	0.85	0.54
OsR498G0612630800.01	13.643	0.426	-4.50577	5.89E-89	3.87E-89	14.66	13.08	13.19	0.23	0.37	0.68
OsR498G0612632200.01	12.346	5.863	-1.08975	1.67E-18	2.33E-18	11.07	13.03	12.94	4.98	6.87	5.74
OsR498G0612638500.01	59.746	165.71	1.425162	0	0	53.75	65.22	60.27	166.51	161.15	169.47
OsR498G0612640000.01	106.83	41.296	-1.36677	3.12E-204	1.26E-204	108.09	104.82	107.6	42.51	40.57	40.81
OsR498G0612644700.01	6.763	18.343	1.432485	8.67E-207	3.47E-207	6.24	6.41	7.64	18.14	18.04	18.85
OsR498G0612647600.01	2.173	0.22	-3.32099	3.28E-22	4.23E-22	2.08	1.82	2.62	0.24	0.18	0.24
OsR498G0612653900.01	6.94	3.52	-1.00593	7.24E-52	6.37E-52	8.34	6.02	6.46	3.63	3.3	3.63
OsR498G0612658100.01	0.356	0.15	-1.2628	0.000293	0.0006764	0.32	0.4	0.35	0.08	0.1	0.27
OsR498G0612664400.01	1.74	0.503	-1.85332	5.53E-14	8.60E-14	1.62	1.97	1.63	0.41	0.33	0.77
OsR498G0612670100.01	0.28	0.056	-2.28217	8.27E-05	0.0001838	0.27	0.28	0.29	0.1	0.07	0
OsR498G0612671900.01	15.7	5.176	-1.60587	2.61E-118	1.47E-118	15.52	15.65	15.93	4.92	5.14	5.47
OsR498G0612676500.01	0.356	56.74	7.28972	0	0	0.46	0.24	0.37	77.76	54.46	38
OsR498G0612676800.01	0.673	1.623	1.266036	1.25E-08	2.31E-08	0.73	0.48	0.81	1.74	1.79	1.34
OsR498G0612679600.01	0.19	0.036	-2.41967	0.0001248	0.0002807	0.21	0.16	0.2	0.03	0.08	0
OsR498G0612680000.01	5.586	0.933	-2.84986	8.23E-51	7.31E-51	6.21	4.78	5.77	0.95	0.5	1.35
OsR498G0612680600.01	1.513	0.123	-3.59251	1.22E-25	1.48E-25	1.35	1.64	1.55	0.18	0.08	0.11
OsR498G0612690700.01	1.13	0.496	-1.19054	0.0001033	0.0002309	1.22	1.05	1.12	0.45	0.58	0.46
OsR498G0612693600.01	14.846	6.263	-1.29353	4.61E-72	3.40E-72	16.84	13.47	14.23	6.25	6.48	6.06
OsR498G0612699600.01	3.4	1.26	-1.43756	4.75E-11	8.02E-11	3.64	3.53	3.03	1.46	1.13	1.19
OsR498G0612707100.01	1.083	0.146	-2.85971	4.62E-17	6.67E-17	0.98	0.91	1.36	0.17	0.1	0.17
OsR498G0612711400.01	3.856	0.623	-2.62766	1.09E-63	8.62E-64	3.1	3.87	4.6	0.81	0.56	0.5
OsR498G0612714500.01	0.276	0.06	-2.20817	3.03E-06	6.20E-06	0.26	0.34	0.23	0.07	0.02	0.09
OsR498G0612718200.01	0.943	10.356	3.342091	1.62E-232	6.01E-233	0.94	0.81	1.08	10.36	10.46	10.25
OsR498G0612718500.01	1.85	0.833	-1.20817	2.56E-10	4.43E-10	1.91	1.75	1.89	0.65	1.08	0.77
OsR498G0612720200.01	103.83	12.396	-3.09014	0	0	103.94	103.2	104.4	13.38	12.28	11.53
OsR498G0612721800.01	1.933	0.446	-2.11699	2.07E-21	2.72E-21	2.2	1.89	1.71	0.35	0.48	0.51
OsR498G0612722200.01	2.436	0.916	-1.42114	1.80E-17	2.57E-17	2.15	2.16	3	0.76	0.97	1.02
OsR498G0612724200.01	0.02	0.283	3.7402	4.34E-07	8.52E-07	0	0.03	0.03	0.29	0.23	0.33
OsR498G0612725200.01	0.053	6.91	7.046507	8.28E-149	4.06E-149	0	0.07	0.09	7.69	6.82	6.22
OsR498G0612726400.01	0	4.92	10.07868	6.92E-88	4.58E-88	0	0	0	5.56	4.77	4.43
OsR498G0612727700.01	2.14	5.63	1.380797	6.39E-21	8.48E-21	2.68	1.52	2.22	5.68	6.08	5.13
OsR498G0612730900.01	0.736	0.353	-1.07869	6.63E-05	0.0001465	0.84	0.64	0.73	0.18	0.52	0.36
OsR498G0612733300.01	2.963	18.346	2.624723	5.81E-237	2.12E-237	2.49	3.1	3.3	18.95	18.05	18.04
OsR498G0612733600.01	8.426	20.86	1.30188	4.64E-128	2.48E-128	8.11	8.25	8.92	20.36	21.53	20.69
OsR498G0612736000.01	61.966	27.07	-1.20403	8.28E-139	4.24E-139	64.01	63.13	58.76	26.08	28.67	26.46
OsR498G0612737600.01	0.013	0.72	5.712185	4.71E-14	7.31E-14	0	0.04	0	0.89	0.64	0.63
OsR498G0612743000.01	24.15	1.483	-4.03626	9.18E-236	3.37E-236	24.44	22.24	25.77	1.24	1.48	1.73
OsR498G0612743600.01	1.423	0.673	-1.09351	1.46E-07	2.80E-07	1.55	1.29	1.43	0.7	0.6	0.72
OsR498G0612745200.01	20.09	7.353	-1.45438	4.57E-98	2.84E-98	20.2	20.19	19.88	8.02	6.74	7.3
OsR498G0612746400.01	0.12	0.61	2.361688	0.0002336	0.0005362	0	0.28	0.08	0.72	0.65	0.46
OsR498G0612746500.01	10.77	30	1.462265	0	0	10.49	11.17	10.65	27.6	31.55	30.85
OsR498G0612746800.01	45.306	17.426	-1.40433	0	0	44.78	44.83	46.31	16.33	18.48	17.47
OsR498G0612756500.01	2.3	13.21	2.517807	0	0	2.08	2.26	2.56	13.36	13.45	12.82
OsR498G0612758100.01	0.743	0.326	-1.18701	4.11E-07	8.08E-07	0.5	0.8	0.93	0.39	0.34	0.25
OsR498G0612763600.01	2.79	1.116	-1.34009	1.24E-22	1.59E-22	2.58	2.72	3.07	1.26	0.95	1.14
OsR498G0612764700.01	0.706	0.113	-2.63009	6.76E-12	1.11E-11	0.86	0.67	0.59	0.11	0.08	0.15
OsR498G0612766200.01	5.093	13.666	1.43781	2.66E-54	2.28E-54	5.28	5.28	4.72	13.64	13.13	14.23
OsR498G0612777500.01	54.26	9.163	-2.58234	0	0	55.26	53.51	54.01	9.31	9.13	9.05
OsR498G0612778200.01	4.673	2.23	-1.05527	7.05E-22	9.16E-22	4.89	4.89	4.24	2.1	2.52	2.07
OsR498G0612783200.01	0.526	0.19	-1.46154	7.18E-13	1.15E-12	0.63	0.5	0.45	0.16	0.27	0.14
OsR498G0612784100.01	2.713	0.566	-2.25925	3.53E-73	2.59E-73	4.05	1.95	2.14	0.97	0.73	0
OsR498G0612784300.01	82.663	21.77	-1.9377	7.95E-301	2.43E-301	93.65	74.33	80.01	23.71	20.6	21
OsR498G0612786600.01	0.49	0.183	-1.43829	0.0002839	0.0006549	0.52	0.63	0.32	0.27	0.12	0.16
OsR498G0612787500.01	0.433	0.156	-1.51044	1.55E-07	2.98E-07	0.26	0.46	0.58	0.1	0.2	0.17
OsR498G0612788600.01	1.11	0.473	-1.22322	0.0002079	0.0004747	0.92	1.32	1.09	0.57	0.2	0.65
OsR498G0612793900.01	0.43	0.13	-1.76759	0.0003459	0.0008029	0.64	0.25	0.4	0.09	0.2	0.1
OsR498G0612796500.01	0.396	0.14	-1.4109	0.0002493	0.0005735	0.33	0.4	0.46	0.24	0.12	0.06
OsR498G0612802600.01	3.143	0.896	-1.82805	2.11E-26	2.52E-26	2.77	3.5	3.16	0	1.74	0.95
OsR498G0612803900.01	0	0.186	4.361688	0.0005877	0.0013892	0	0	0	0.05	0.34	0.17
OsR498G0612804900.01	4.89	2.25	-1.13242	2.55E-23	3.23E-23	5.51	4.77	4.39	2.28	2.5	1.97
OsR498G0612807400.01	0.24	0.033	-2.86713	0.0004299	0.0010056	0.14	0.35	0.23	0	0.05	0.05
OsR498G0612810100.01	0.43	0.086	-2.46925	9.54E-12	1.58E-11	0.44	0.48	0.37	0.15	0.09	0.02

OsR498G0612810500.01	0.013	0.206	4.127223	1.87E-05	4.00E-05	0	0.04	0	0.29	0.07	0.26
OsR498G0612812000.01	2.076	10.653	2.360766	2.40E-205	9.64E-206	1.92	2.25	2.06	11.01	10.49	10.46
OsR498G0612813200.01	4.45	1.796	-1.30507	1.12E-65	8.72E-66	4.34	4.47	4.54	2.02	1.8	1.57
OsR498G0612814100.01	19.376	40.636	1.060041	1.11E-131	5.83E-132	19.48	19.18	19.47	41.89	38.83	41.19
OsR498G0612816500.01	27.556	5.013	-2.4664	0	0	27.16	27.68	27.83	4.54	5.46	5.04
OsR498G0612824500.01	2.133	0.06	-5.20817	1.36E-10	2.33E-10	3.18	1.77	1.45	0	0	0.18
OsR498G0612829300.01	0.12	2.51	4.54226	2.76E-17	3.96E-17	0.1	0	0.26	2.31	2.37	2.85
OsR498G0612834500.01	21.156	94.72	2.231584	0	0	20.65	21.91	20.91	92.91	96.91	94.34
OsR498G0612836900.01	1.56	7.526	2.229102	1.26E-24	1.55E-24	2.06	1.37	1.25	7.31	6.39	8.88
OsR498G0612840300.01	0.42	1.026	1.147563	1.10E-07	2.10E-07	0.42	0.42	0.42	0.96	1.24	0.88
OsR498G0612845300.01	0.956	0.2	-2.26559	9.18E-23	1.17E-22	0.81	1.02	1.04	0.2	0.23	0.17
OsR498G0612846600.01	4.593	16.753	1.817982	1.04E-59	8.50E-60	4.03	4.37	5.38	17.06	15.51	17.69
OsR498G0612846900.01	0.786	7.516	3.256335	1.09E-121	6.02E-122	0.66	1	0.7	7.1	8.15	7.3
OsR498G0612847100.01	0.336	4.613	3.75073	1.00E-57	8.36E-58	0.44	0.15	0.42	4.55	4.59	4.7
OsR498G0612847900.01	0.023	0.243	3.432077	8.80E-06	1.85E-05	0	0	0.07	0.31	0.21	0.21
OsR498G0612848100.01	0.09	0.54	2.624723	2.62E-05	5.65E-05	0.07	0.14	0.06	0.47	0.27	0.88
OsR498G0612852100.01	0.933	5.916	2.595518	6.02E-12	9.90E-12	0.25	0.44	2.11	5.03	6.49	6.23
OsR498G0612852500.01	1	3.063	1.79214	1.04E-12	1.67E-12	0.58	1.91	0.51	2.5	3.26	3.43
OsR498G0612856100.01	2.663	7.396	1.478427	9.28E-103	5.64E-103	2.12	3.18	2.69	7.76	6.98	7.45
OsR498G0612857000.01	0.26	1.14	2.0626	5.90E-23	7.51E-23	0.25	0.26	0.27	1.08	1.2	1.14
OsR498G0612863100.01	93.23	228.68	1.300355	0	0	90.95	94.09	94.65	225.46	228.8	231.78
OsR498G0612863300.01	0.343	0.97	1.472719	1.65E-05	3.50E-05	0.36	0.22	0.45	1.01	0.71	1.19
OsR498G0612863700.01	0.043	1.016	4.466025	3.19E-21	4.20E-21	0.03	0.07	0.03	1.04	0.94	1.07
OsR498G0612866100.01	2.893	1.28	-1.18002	1.25E-10	2.14E-10	2.96	2.91	2.81	1.29	1.43	1.12
OsR498G0612866900.01	1.03	2.463	1.230091	1.83E-06	3.69E-06	1.28	0.5	1.31	2.7	2.77	1.92
OsR498G0612870300.01	0.89	0.08	-3.4838	4.27E-19	5.89E-19	1.06	0.93	0.68	0.12	0.06	0.06
OsR498G0612871300.01	0.246	1.24	2.297558	9.31E-16	1.38E-15	0.16	0.17	0.41	1.66	1.24	0.82
OsR498G0612873900.01	101.62	30.36	-1.74104	0	0	100.85	104.13	99.88	30.1	30.58	30.4
OsR498G0612875900.01	6.433	0.716	-3.16984	7.49E-85	5.05E-85	5.92	6.34	7.04	0.73	0.8	0.62
OsR498G0612878300.01	0.736	1.59	1.095042	1.32E-07	2.53E-07	0.83	0.51	0.87	1.62	1.82	1.33
OsR498G0612878800.01	0.176	0.053	-1.77804	7.03E-05	0.0001556	0.14	0.13	0.26	0.05	0.08	0.03
OsR498G0612883100.01	6.033	1.38	-2.16766	8.65E-127	4.66E-127	5.44	6.07	6.59	1.44	1.51	1.19
OsR498G0612885700.01	0.993	13.09	3.718045	2.06E-275	6.76E-276	1.15	0.88	0.95	14.43	12.98	11.86
OsR498G0612887100.01	11.946	25.153	1.069617	1.03E-93	6.56E-94	13.07	11.68	11.09	24.83	25.05	25.58
OsR498G0612887300.01	2.056	0.863	-1.28886	9.65E-08	1.84E-07	1.95	2.31	1.91	0.4	1.31	0.88
OsR498G0612888300.01	0.716	5.766	2.999571	4.40E-121	2.44E-121	0.68	0.79	0.68	5.48	6.11	5.71
OsR498G0612890300.01	74.226	26.993	-1.46376	0	0	73.98	75.87	72.83	25.11	29.2	26.67
OsR498G0612891500.01	19.616	52.163	1.403851	2.26E-184	9.76E-185	19.87	19.79	19.19	52.13	51.4	52.96
OsR498G0612893400.01	5.086	15.063	1.558326	3.10E-169	1.41E-169	5.63	4.39	5.24	14.07	15.1	16.02
OsR498G0612894200.01	22.293	8.426	-1.43441	3.20E-130	1.70E-130	22.58	20.3	24	8.57	8.43	8.28
OsR498G0612895800.01	149.47	304.01	1.019945	0	0	146.96	153.01	148.4	310.91	299.75	301.37
OsR498G0612897800.01	12.386	131.01	3.396689	0	0	12.19	11.88	13.09	134.02	126.26	132.76
OsR498G0612900400.01	0.543	0.026	-4.40318	8.61E-21	1.15E-20	0.49	0.51	0.63	0	0.06	0.02
OsR498G0612901100.01	1.966	0.26	-2.93148	1.17E-49	1.05E-49	1.78	1.84	2.28	0.3	0.19	0.29
OsR498G0612903300.01	49.496	21.08	-1.23657	4.28E-108	2.52E-108	51.55	46.54	50.4	22.33	23.49	17.42
OsR498G0612906000.01	6.856	3.423	-1.00525	1.91E-28	2.20E-28	7.32	6.9	6.35	3.25	3.52	3.5
OsR498G0612911100.01	151.2	33.61	-2.1813	0	0	167.87	141.12	144.6	34.34	32.65	33.84
OsR498G0612911400.01	45.813	14.756	-1.64697	0	0	44.64	46.44	46.36	14.23	14.87	15.17
OsR498G0612914100.01	2.433	0.256	-3.24021	1.29E-28	1.49E-28	2.07	2.71	2.52	0.25	0.26	0.26
OsR498G0612917100.01	369.09	120.49	-1.65707	0	0	361	367.75	378.5	127.21	117.76	116.5
OsR498G0612917400.01	30.39	68.746	1.159916	0	0	30.8	29.52	30.85	70.74	67.39	68.11
OsR498G0612920700.01	0	0.2	4.627125	0.0001691	0.0003836	0	0	0	0	0.43	0.17
OsR498G0612921000.01	2.533	0.116	-4.45623	2.24E-53	1.94E-53	1.48	3.72	2.4	0.09	0.26	0
OsR498G0612921100.01	37.74	4.873	-2.77909	0	0	38.52	37.73	36.97	4.94	4.87	4.81
OsR498G0612921500.01	1.13	0.226	-2.31388	1.62E-07	3.12E-07	1.24	1.23	0.92	0.37	0.23	0.08
OsR498G0612928200.01	1099.2	487.58	-1.17721	0	0	1102.5	1101.7	1093	492.58	477.19	492.97
OsR498G0612933300.01	482.66	231.57	-1.06652	0	0	488.35	475.19	484.5	239.68	227.1	227.94
OsR498G0612934200.01	0.083	0.906	3.418272	1.39E-21	1.82E-21	0.05	0.08	0.12	1	0.93	0.79
OsR498G0612937400.01	4.546	74.026	4.023497	0	0	3.23	5.64	4.77	75.86	73.75	72.47
OsR498G0612959800.01	11.68	5.393	-1.12333	5.49E-39	5.53E-39	11.5	11.27	12.27	5.28	5.42	5.48
OsR498G0612961000.01	6.646	0.133	-5.62049	5.53E-84	3.75E-84	7.06	6.71	6.17	0.34	0	0.06
OsR498G0612961500.01	3.263	1.016	-1.66446	1.95E-55	1.66E-55	3.16	2.96	3.67	1.04	0.86	1.15
OsR498G0612970100.01	9.856	47.58	2.261062	0	0	10.22	9.67	9.68	49.69	47.91	45.14
OsR498G0612970500.01	4.396	1.856	-1.15289	4.55E-54	3.92E-54	4.52	3.88	4.79	1.79	1.86	1.92
OsR498G0612972800.01	4.99	27.82	2.45972	2.79E-282	8.99E-283	5.37	4.35	5.25	27.25	26.61	29.6
OsR498G0612975600.01	15.22	5.666	-1.43548	6.30E-148	3.10E-148	15.31	15.13	15.22	5.12	5.56	6.32
OsR498G0612991600.01	0.596	0.2	-1.60244	0.0002628	0.0006051	0.4	0.47	0.92	0.31	0.11	0.18
OsR498G0613005200.01	12.296	30.903	1.329422	1.20E-305	3.61E-306	11.98	11.58	13.33	30.24	31.25	31.22
OsR498G0613011200.01	4.25	2.053	-1.06333	1.35E-20	1.80E-20	4.25	4.48	4.02	1.92	1.89	2.35

OsR498G0613017200.01	2.133	5.503	1.361688	5.93E-24	7.42E-24	1.9	2.23	2.27	5.22	5.77	5.52
OsR498G0613017700.01	825.32	293.12	-1.48673	0	0	838.07	827.42	810.5	289.16	295.74	294.45
OsR498G0613018900.01	0.893	0.11	-2.41967	4.19E-06	8.63E-06	0.8	1.46	0.42	0.05	0.17	0.11
OsR498G0613021200.01	35.533	10.706	-1.73528	2.96E-294	9.23E-295	34.67	35.03	36.9	11.56	10.36	10.2
OsR498G0613026300.01	3.36	1.303	-1.5341	2.37E-20	3.18E-20	2.45	3.72	3.91	1.11	1.34	1.46
OsR498G0613029200.01	0.323	14.703	5.499192	1.34E-242	4.82E-243	0.25	0.35	0.37	15.63	14.74	13.74
OsR498G0613031800.01	46.853	18.156	-1.35941	1.56E-191	6.57E-192	48.22	46.17	46.17	18.53	17.31	18.63
OsR498G0613032100.01	381.13	176.34	-1.12333	0	0	395.42	369.85	378.1	188.39	174.6	166.03
OsR498G0613035300.01	0.183	0.583	1.589957	6.19E-05	0.0001364	0.33	0.13	0.09	0.68	0.61	0.46
OsR498G0613038900.01	13.54	0	-11.1113	1.69E-155	8.05E-156	12.32	13.81	14.49	0	0	0
OsR498G0613040900.01	0.09	0.953	3.432077	7.63E-18	1.08E-17	0.1	0.07	0.1	1.07	0.8	0.99
OsR498G0613042200.01	3.873	0.216	-4.14694	1.09E-74	7.87E-75	3.66	4.71	3.25	0.1	0.31	0.24
OsR498G0613048600.01	1.48	0.643	-1.23587	6.40E-09	1.16E-08	1.78	1.29	1.37	0.77	0.62	0.54
OsR498G0613048800.01	30.11	10.693	-1.4989	0	0	28.47	29.23	32.63	10.09	11.09	10.9
OsR498G0613049800.01	22	47.106	1.12936	3.68E-229	1.38E-229	21.53	19.78	24.69	45.68	48.91	46.73
OsR498G0613050500.01	97.14	36.19	-1.42248	0	0	95.92	96.66	98.84	34.62	35.91	38.04
OsR498G0613055600.01	160.12	402.08	1.327041	0	0	161.12	161.94	157.3	407.99	388.55	409.71
OsR498G0613055700.01	8.96	21.206	1.231798	2.57E-55	2.19E-55	9.07	8.58	9.23	21.18	23.4	19.04
OsR498G0613057800.01	136.61	57.523	-1.26632	0	0	141.99	133.47	134.4	58.04	56.32	58.21
OsR498G0613058600.01	15.273	32.093	1.051537	6.42E-52	5.64E-52	16.08	16.87	12.87	31.89	32.17	32.22
OsR498G0613059500.01	1.29	0.316	-2.22327	0.0001374	0.00031	0.96	0.65	2.26	0.5	0	0.45
OsR498G0613061000.01	4.92	13.673	1.509329	2.78E-96	1.74E-96	5.02	5.35	4.39	12.69	15.17	13.16
OsR498G0613061600.01	6.286	2.16	-1.53463	4.33E-89	2.84E-89	6.66	6.26	5.94	2.12	2.2	2.16
OsR498G0613062000.01	0	0.11	4.252329	0.0009276	0.0022265	0	0	0	0	0.33	0
OsR498G0613063400.01	4.146	1.87	-1.0377	1.73E-45	1.63E-45	4.18	4.37	3.89	1.65	2.14	1.82
OsR498G0613064000.01	7.376	2.943	-1.33073	6.38E-35	6.76E-35	7.18	7.45	7.5	3.12	2.95	2.76
OsR498G0613065200.01	0.88	21.92	4.649217	0	0	1.09	0.7	0.85	22.44	20.72	22.6
OsR498G0613066600.01	0.203	4.886	4.58364	3.88E-185	1.67E-185	0.26	0.2	0.15	4.48	4.93	5.25
OsR498G0613069100.01	0.393	2.803	2.837716	4.56E-75	3.29E-75	0.39	0.45	0.34	2.85	3.04	2.52
OsR498G0613070000.01	1.706	5.953	1.808277	1.30E-64	1.02E-64	1.79	1.55	1.78	6.31	5.23	6.32
OsR498G0613074000.01	59.21	206.92	1.831881	0	0	58.96	58.95	59.72	215.75	194.24	210.77
OsR498G0613074200.01	10.21	79.45	2.984739	0	0	10.55	9.12	10.96	84.49	76.11	77.75
OsR498G0613074600.01	4.59	0.966	-2.27903	1.24E-57	1.03E-57	4.46	4.31	5	0.61	1.2	1.09
OsR498G0613074700.01	0.846	6.883	3.003234	6.02E-48	5.52E-48	0.8	0.61	1.13	8.36	5.14	7.15
OsR498G0613075900.01	0	1.586	7.03976	1.56E-16	2.27E-16	0	0	0	1.98	1.96	0.82
OsR498G0613076100.01	14.92	3.796	-1.97811	1.05E-306	3.16E-307	14.12	14.99	15.65	3.53	4.01	3.85
OsR498G0613076600.01	0.256	6.086	4.518732	7.67E-153	3.70E-153	0.17	0.09	0.51	5.97	6.26	6.03
OsR498G0613076800.01	3.036	0.903	-1.76301	1.56E-15	2.33E-15	2.54	3.16	3.41	0.89	1.1	0.72
OsR498G0613079700.01	0.156	0	-4.28217	0.0007127	0.0016975	0.14	0.29	0.04	0	0	0
OsR498G0613087000.01	4.943	2.27	-1.09506	1.21E-24	1.50E-24	4.73	4.45	5.65	1.85	2.35	2.61
OsR498G0613088000.01	23.96	7.076	-1.76334	3.25E-222	1.24E-222	23.13	24.08	24.67	7.36	7.01	6.86
OsR498G0613090100.01	1.65	0.493	-1.71981	4.24E-18	5.97E-18	1.53	1.66	1.76	0.48	0.51	0.49
OsR498G0613091300.01	31.543	13.816	-1.19792	1.39E-80	9.66E-81	35.38	30.38	28.87	13.37	14.12	13.96
OsR498G0613100100.01	45.91	8.56	-2.43399	0	0	45.16	46.75	45.82	8.33	9.48	7.87
OsR498G0613102200.01	1.45	4.213	1.681554	2.53E-60	2.06E-60	1.4	1.32	1.63	3.93	4.01	4.7
OsR498G0613109100.01	7.923	25.226	1.675202	0	0	7.93	8.54	7.3	23.69	25.59	26.4
OsR498G0613110600.01	23.123	99.24	2.199524	0	0	24.36	22.75	22.26	100.4	98.88	98.44
OsR498G0613111800.01	17.99	75.433	2.060017	0	0	17.59	18.75	17.63	74.92	74.87	76.51
OsR498G0613112000.01	2.216	8.946	2.017442	5.08E-98	3.16E-98	1.98	2.1	2.57	8.36	9.04	9.44
OsR498G0613117200.01	4.596	0.72	-2.67017	9.05E-84	6.15E-84	4.21	4.82	4.76	0.57	0.7	0.89
OsR498G0613117300.01	6.666	3.133	-1.17443	1.61E-32	1.76E-32	6.88	6.28	6.84	3.14	2.96	3.3
OsR498G0613117700.01	0.16	0.736	2.155237	8.50E-05	0.0001889	0.08	0.17	0.23	0.66	0.54	1.01
OsR498G0613121600.01	4.893	1.92	-1.35197	4.33E-38	4.40E-38	5.15	4.81	4.72	1.5	1.84	2.42
OsR498G0613124300.01	21.84	71.166	1.69948	0	0	21.05	22.56	21.91	70.26	71.59	71.65
OsR498G0613128600.01	2.64	14.786	2.481005	0	0	2.72	2.51	2.69	14.26	15.24	14.86
OsR498G0613133500.01	16.673	96.703	2.528364	0	0	16.73	16.6	16.69	108.37	92.64	89.1
OsR498G0613134900.01	1.21	0.326	-1.90777	4.79E-07	9.42E-07	1.41	0.99	1.23	0.21	0.42	0.35
OsR498G0613137300.01	0.22	4.346	4.31386	2.36E-198	9.68E-199	0.1	0.27	0.29	5.18	3.88	3.98
OsR498G0613138900.01	0.536	3.32	2.627392	2.06E-81	1.42E-81	0.43	0.63	0.55	3.45	3.22	3.29
OsR498G0613139300.01	54.01	21.916	-1.01422	6.31E-139	3.23E-139	54.38	54.59	53.06	22.21	21.74	21.8
OsR498G0613141000.01	14.086	4.716	-1.60261	4.95E-103	3.00E-103	15.22	13.81	13.23	4.43	4.11	5.61
OsR498G0613145400.01	576.61	1744.8	1.62782	0	0	568.89	576.06	584.9	1704.2	1752.3	1777.8
OsR498G0613146300.01	13.52	36.33	1.430498	0	0	12.95	14.22	13.39	34.16	37.62	37.21
OsR498G0613148900.01	2.313	41.193	4.146215	0	0	2.28	2.12	2.54	42.3	38.49	42.79
OsR498G0613151300.01	2.043	10.273	2.418694	5.49E-185	2.37E-185	2.03	2.17	1.93	10.54	10.57	9.71
OsR498G0613153600.01	0.376	11.76	4.960609	1.69E-171	7.60E-172	0.42	0.44	0.27	10.86	11.76	12.66
OsR498G0613153800.01	0.246	2.833	3.525187	9.77E-13	1.57E-12	0	0.46	0.28	2.71	2.9	2.89
OsR498G0613155600.01	3.006	38.773	3.679764	3.47E-301	1.06E-301	3.89	2.6	2.53	47.55	36.5	32.27
OsR498G0613159800.01	11.023	5.316	-1.09238	1.65E-24	2.04E-24	11.5	10.5	11.07	4.14	4.72	7.09

OsR498G0613162800.01	7.48	3.44	-1.19433	5.89E-24	7.36E-24	7.79	7.25	7.4	3.62	4.03	2.67
OsR498G0613163400.01	0.26	0.803	1.468603	0.0003802	0.0008855	0.22	0.22	0.34	1.07	0.67	0.67
OsR498G0613163800.01	4.276	46.63	2.210641	0	0	4.24	4.22	4.37	47.59	45.23	47.07
OsR498G0613164400.01	11.856	198.97	4.101758	0	0	11.77	12.74	11.06	201.19	198.93	196.78
OsR498G0613166500.01	1.176	61.97	5.709003	0	0	1.27	1.04	1.22	59.36	65.73	60.82
OsR498G0613167000.01	0.28	0.08	-1.78127	1.76E-06	3.55E-06	0.22	0.38	0.24	0.06	0.05	0.13
OsR498G0613167600.01	9.96	39.026	1.968339	0	0	10.26	10.07	9.55	37.76	39.98	39.34
OsR498G0613169700.01	0.54	0.08	-2.71513	8.51E-06	1.79E-05	0.3	0.75	0.57	0.06	0.06	0.12
OsR498G0613171300.01	0.043	0.226	2.441858	7.92E-07	1.57E-06	0.04	0.02	0.07	0.27	0.26	0.15
OsR498G0613178200.01	16.636	48.556	1.562153	0	0	15.5	17.49	16.92	48.33	50.19	47.15
OsR498G0613180100.01	57.366	11.763	-2.2692	0	0	54.92	63.22	53.96	13.36	11.17	10.76
OsR498G0613181900.01	1.323	0.56	-1.25486	4.22E-10	7.36E-10	1.24	1.31	1.42	0.51	0.53	0.64
OsR498G0613193200.01	0.423	0.023	-4.24564	3.58E-10	6.23E-10	0.45	0.6	0.22	0	0.07	0
OsR498G0613193300.01	0.716	0.09	-3.03063	8.53E-09	1.56E-08	0.77	0.59	0.79	0	0.11	0.16
OsR498G0613194600.01	8.126	1.896	-1.97606	8.57E-30	9.71E-30	8.34	9.63	6.41	1.57	2.03	2.09
OsR498G0613197300.01	0.026	0.24	3.127223	9.61E-05	0.0002145	0.04	0.04	0	0.29	0.3	0.13
OsR498G0613197700.01	13.393	27.77	1.048468	3.70E-116	2.10E-116	13.17	13.62	13.39	27.7	27.76	27.85
OsR498G0613199600.01	8.86	1.606	-2.42334	6.33E-79	4.45E-79	8.22	8.96	9.4	1.75	1.42	1.65
OsR498G0613200200.01	0.076	0.443	2.525187	9.82E-06	2.07E-05	0.09	0.1	0.04	0.47	0.58	0.28
OsR498G0613200700.01	4.556	438.7	6.577538	0	0	3.94	4.72	5.01	468.32	416.77	431
OsR498G0613201000.01	0.2	4.186	4.35399	9.67E-20	1.31E-19	0.6	0	0	3.55	4.28	4.73
OsR498G0613201200.01	0.283	0	-4.96024	2.02E-05	4.33E-05	0.37	0.17	0.31	0	0	0
OsR498G0613202600.01	0.923	7.183	2.938383	5.30E-45	5.01E-45	1.35	0.42	1	6.58	7.1	7.87
OsR498G0613203000.01	0.103	0.286	1.454798	0.0006717	0.0015963	0.08	0.05	0.18	0.29	0.27	0.3
OsR498G0613203200.01	18.12	2.946	-2.61343	0	0	18.37	19.05	16.94	2.74	2.79	3.31
OsR498G0613204600.01	0.753	0.116	-2.73665	1.04E-06	2.09E-06	0.35	0.87	1.04	0	0.08	0.27
OsR498G0613204700.01	0.146	0	-5.28217	2.04E-06	4.14E-06	0.04	0.21	0.19	0	0	0
OsR498G0613207700.01	5.686	12.85	1.161224	5.77E-25	7.07E-25	6.29	4.92	5.85	13.52	12.77	12.26
OsR498G0613207900.01	1.36	0.546	-1.45565	2.88E-15	4.33E-15	1.47	1.15	1.46	0.37	0.57	0.7
OsR498G0613208200.01	10.793	3.153	-1.78496	7.33E-39	7.40E-39	10.57	10.55	11.26	3.64	2.76	3.06
OsR498G0613210800.01	2.713	1.1	-1.30128	1.13E-16	1.64E-16	2.6	2.52	3.02	1.12	1.41	0.77
OsR498G0613211900.01	3.373	0.363	-3.2112	1.90E-43	1.83E-43	3.25	3.4	3.47	0.43	0.35	0.31
OsR498G0613212900.01	9.756	30.046	1.619175	7.56E-269	2.52E-269	8.91	10.17	10.19	30.54	30.04	29.56
OsR498G0613213400.01	9.01	0.516	-4.14276	1.94E-74	1.41E-74	8.5	9.34	9.19	0.4	0.66	0.49
OsR498G0613214400.01	4.123	34.463	3.043522	0	0	3.97	3.53	4.87	34.02	34.75	34.62
OsR498G0613215600.01	2.24	25.69	3.4578	0	0	1.95	2.41	2.36	24.12	27.77	25.18
OsR498G0613219600.01	0.356	0.896	1.317745	1.78E-07	3.44E-07	0.32	0.36	0.39	0.9	0.82	0.97
OsR498G0613222500.01	1.44	0.656	-1.3865	2.08E-12	3.38E-12	1.43	1.67	1.22	0.75	0.67	0.55
OsR498G0613225600.01	0.166	0.756	2.03976	7.24E-12	1.19E-11	0.15	0.14	0.21	0.68	0.95	0.64
OsR498G0613226300.01	12.153	30.933	1.359922	2.45E-201	9.96E-202	13.17	12.39	10.9	29.52	31.95	31.33
OsR498G0613226800.01	7.526	2.643	-1.14356	8.00E-45	7.58E-45	8.42	6.9	7.26	2.58	3.11	2.24
OsR498G0613227500.01	0.256	0	-5.35256	1.16E-06	2.33E-06	0.18	0.38	0.21	0	0	0
OsR498G0613228500.01	18.126	43.046	1.242619	2.37E-208	9.42E-209	17.42	18.36	18.6	41.95	43.76	43.43
OsR498G0613230200.01	6.546	1.06	-2.04158	7.23E-58	6.01E-58	6.86	6.5	6.28	1.36	0.95	0.87
OsR498G0613230500.01	104.29	27.82	-1.90827	0	0	111.83	97.59	103.5	29.12	27.24	27.1
OsR498G0613231400.01	376.78	120.19	-1.66988	0	0	380.24	368.92	381.2	118.39	120.59	121.59
OsR498G0613231900.01	2.926	0.483	-2.61232	2.15E-14	3.30E-14	3.39	2.31	3.08	0.51	0.63	0.31
OsR498G0613233900.01	1.056	0.466	-1.18851	9.55E-06	2.01E-05	0.97	0.93	1.27	0.63	0.29	0.48
OsR498G0613235300.01	0.016	0.273	4.084154	6.36E-09	1.16E-08	0	0.05	0	0.27	0.26	0.29
OsR498G0613237300.01	0.206	0	-4.41967	0.0003895	0.0009082	0.17	0.19	0.26	0	0	0
OsR498G0613238700.01	6.706	3.076	-1.13106	7.60E-20	1.03E-19	6.56	6.42	7.14	2.66	3.11	3.46
OsR498G0613242700.01	0.026	0.38	3.847115	1.30E-07	2.49E-07	0	0.08	0	0.48	0.25	0.41
OsR498G0613243200.01	0.896	2.053	1.204391	3.40E-12	5.55E-12	0.88	1.1	0.71	2.49	1.97	1.7
OsR498G0613244800.01	4.733	11.173	1.165123	2.15E-39	2.15E-39	4.9	4.07	5.23	12.3	10.63	10.59
OsR498G0613245900.01	2.69	10.39	2.065295	4.42E-159	2.07E-159	2.4	2.33	3.34	10.17	9.72	11.28
OsR498G0613246700.01	3.973	0.79	-2.27235	2.31E-37	2.37E-37	3.59	3.76	4.57	0.75	0.79	0.83
OsR498G0613247300.01	3.703	1.283	-1.33593	3.77E-18	5.29E-18	4.1	3.69	3.32	1	1.11	1.74
OsR498G0613247600.01	0.45	8.953	4.127863	0	0	0.48	0.45	0.42	8.78	9.04	9.04
OsR498G0613248700.01	1.146	0.08	-3.98261	3.03E-15	4.55E-15	1.21	0.87	1.36	0.06	0.18	0
OsR498G0613249800.01	18.633	4.54	-2.05067	1.08E-66	8.34E-67	19.09	18.55	18.26	4.7	4.24	4.68
OsR498G0613250700.01	21.813	45.066	1.041631	0	0	21.85	21.69	21.9	44.57	45.76	44.87
OsR498G0613250900.01	0.503	3.476	2.914229	6.07E-56	5.14E-56	0.44	0.62	0.45	3.16	3.91	3.36
OsR498G0613251700.01	1.15	0.296	-1.94544	3.57E-21	4.70E-21	1.11	1.26	1.08	0.41	0.2	0.28
OsR498G0613253600.01	0.873	5.49	3.03976	1.93E-191	8.12E-192	0.92	0.69	1.01	5.15	5.54	5.78
OsR498G0613264700.01	4.056	8.813	1.119208	1.02E-91	6.58E-92	3.77	4.31	4.09	8.52	9.14	8.78
OsR498G0613265800.01	0.153	0	-4.5452	0.0002138	0.0004889	0.19	0.16	0.11	0	0	0
OsR498G0613266700.01	0.2	0.056	-1.79031	0.0001333	0.0003003	0.15	0.26	0.19	0.06	0.09	0.02
OsR498G0613267000.01	3.243	0.7	-2.06777	7.62E-41	7.52E-41	3.11	3.69	2.93	0.56	0.78	0.76
OsR498G0613272900.01	82.463	9.946	-3.05005	0	0	84.29	81.82	81.28	10.56	9.84	9.44

OsR498G0613275100.01	6.666	0.88	-2.92707	6.37E-112	3.69E-112	4.97	7.82	7.21	1	0.66	0.98
OsR498G0613278600.01	2.936	0.97	-1.61067	2.93E-22	3.77E-22	2.95	2.72	3.14	0.61	1.18	1.12
OsR498G0613279000.01	0	0.283	5.847115	1.60E-08	2.96E-08	0	0	0	0.3	0.28	0.27
OsR498G0613279800.01	0.886	0.443	-1.04149	1.03E-09	1.83E-09	0.78	0.9	0.98	0.37	0.48	0.48
OsR498G0613280600.01	2.386	0.363	-2.46544	1.71E-14	2.62E-14	2.13	2.72	2.31	0.38	0.17	0.54
OsR498G0613282300.01	19.993	75.65	1.913465	0	0	20.63	19.56	19.79	80.59	70.35	76.01
OsR498G0613283500.01	0	0.343	5.325162	1.97E-06	3.98E-06	0	0	0	0.2	0	0.83
OsR498G0613285800.01	50.02	155.35	1.77767	0	0	51.03	50.11	48.92	159.57	152.04	154.43
OsR498G0613286600.01	0	5.52	9.850332	8.60E-78	6.08E-78	0	0	0	5.79	5.36	5.41
OsR498G0613287200.01	0.796	2.023	1.349088	3.37E-12	5.51E-12	0.52	0.97	0.9	1.95	1.91	2.21
OsR498G0613287700.01	30.426	184.91	2.602482	0	0	31.38	30.06	29.84	194.01	181.06	179.67
OsR498G0613287900.01	4.16	8.93	1.086054	4.23E-14	6.55E-14	3.7	3.99	4.79	9.18	8.84	8.77
OsR498G0613288400.01	1.98	5.323	1.422704	3.04E-23	3.85E-23	1.99	1.94	2.01	6.6	5.18	4.19
OsR498G0613289800.01	0.023	1.88	6.466025	3.75E-22	4.85E-22	0	0.07	0	2.16	1.85	1.63
OsR498G0613291300.01	3.546	11.056	1.633493	2.85E-81	1.97E-81	3.95	3.19	3.5	11.84	11.34	9.99
OsR498G0613292600.01	13.5	35.166	1.375265	6.33E-307	1.90E-307	13.15	13.39	13.96	34.33	34.77	36.4
OsR498G0613296500.01	1.61	0.496	-1.76134	7.48E-12	1.24E-11	1.33	1.39	2.11	0.59	0.46	0.44
OsR498G0613297400.01	3.55	16.983	2.251544	3.91E-135	2.03E-135	3.63	3.7	3.32	17.18	16.72	17.05
OsR498G0613297500.01	0.196	0.48	1.40233	0.0004802	0.0011268	0.11	0.23	0.25	0.54	0.3	0.6
OsR498G0613298600.01	18.763	45.913	1.260537	4.54E-228	1.70E-228	19.37	18.87	18.05	45.06	47.12	45.56
OsR498G0613301000.01	0.646	0.133	-2.28217	4.66E-06	9.62E-06	0.35	0.65	0.94	0.13	0.11	0.16
OsR498G0613301300.01	26.563	61.816	1.171864	2.19E-288	6.94E-289	26.67	25.59	27.43	64.26	59.78	61.41
OsR498G0613302100.01	0.916	0.113	-3.03553	1.19E-11	1.98E-11	0.74	0.93	1.08	0.14	0.1	0.1
OsR498G0613303400.01	1.086	0.193	-2.50773	3.38E-12	5.53E-12	1.06	1.13	1.07	0.22	0.31	0.05
OsR498G0613303600.01	12.673	3.123	-2.01568	2.30E-247	8.14E-248	13.11	12.26	12.65	3.51	2.73	3.13
OsR498G0613304400.01	0.49	1.813	1.730076	3.06E-14	4.72E-14	0.37	0.57	0.53	2.3	1.26	1.88
OsR498G0613305900.01	26.43	81.44	1.613077	0	0	26.26	26.66	26.37	81.93	80.64	81.75
OsR498G0613306500.01	22.683	2.636	-3.10906	1.09E-231	4.06E-232	23.75	23.04	21.26	3	2.36	2.55
OsR498G0613307600.01	68.836	30.936	-1.17801	0	0	66.61	67.82	72.08	30.99	31.34	30.48
OsR498G0613307800.01	51.4	116.13	1.181083	0	0	50.55	52.39	51.26	117.43	113.26	117.7
OsR498G0613309200.01	2.61	1.236	-1.10125	2.08E-12	3.37E-12	2.8	2.55	2.48	1.14	1.21	1.36
OsR498G0613309900.01	4.373	1.956	-1.17046	1.50E-18	2.09E-18	3.82	5.18	4.12	2.26	1.74	1.87
OsR498G0613311100.01	5.09	34.586	2.763713	0	0	5.28	4.77	5.22	35.19	33.94	34.63
OsR498G0613312200.01	0	0.23	5.127223	8.48E-06	1.78E-05	0	0	0	0.32	0.25	0.12
OsR498G0613312700.01	0.92	1.94	1.070787	9.88E-06	2.08E-05	1.13	0.88	0.75	2.13	1.95	1.74
OsR498G0613327500.01	0.433	2.186	2.401216	1.76E-26	2.11E-26	0.47	0.39	0.44	2.34	1.91	2.31
OsR498G0613327700.01	2.446	0.576	-2.03281	8.62E-30	9.76E-30	2.13	2.89	2.32	0.62	0.5	0.61
OsR498G0613329300.01	14.266	6.686	-1.10346	2.26E-21	2.97E-21	14.31	14.48	14.01	6.64	7.37	6.05
OsR498G0613331200.01	288.34	1371.4	2.245256	0	0	291.16	287.84	286	1375.9	1381.8	1356.4
OsR498G0613332400.01	20.326	167.79	3.041033	0	0	18.48	21.88	20.62	185.96	158.97	158.43
OsR498G0613335700.01	0.326	0.083	-1.96024	0.0001436	0.0003243	0.45	0.29	0.24	0.11	0.07	0.07
OsR498G0613336100.01	0.033	0.266	3.127223	9.61E-05	0.0002145	0	0.1	0	0.23	0.24	0.33
OsR498G0613341500.01	6.316	17.97	1.503896	9.64E-264	3.25E-264	6.09	6.27	6.59	17.71	17.49	18.71
OsR498G0613342600.01	10.533	1.5	-2.82824	0	0	9.21	11.51	10.88	1.48	1.64	1.38
OsR498G0613346000.01	0.956	5.45	2.478333	4.41E-16	6.50E-16	1.06	0.64	1.17	7.09	4.96	4.3
OsR498G0613346900.01	7.833	1.326	-2.6276	1.07E-73	7.83E-74	8.06	6.6	8.84	1.22	1.3	1.46
OsR498G0613347100.01	40.313	98.16	1.272804	0	0	40.37	41.2	39.37	94.51	98.89	101.08
OsR498G0613348100.01	10.466	5.41	-1.03572	1.51E-43	1.45E-43	10.27	9.56	11.57	4.83	5.63	5.77
OsR498G0613349200.01	4.796	2.256	-1.1163	3.78E-48	3.46E-48	5.16	4.38	4.85	1.72	2.25	2.8
OsR498G0613349400.01	19.7	4.033	-2.29659	4.60E-108	2.71E-108	21.09	18.52	19.49	4.67	3.21	4.22
OsR498G0613351300.01	1.333	0.6	-1.13016	5.05E-07	9.96E-07	1.13	1.35	1.52	0.48	0.63	0.69
OsR498G0713353700.01	63.916	162	1.312948	0	0	63.79	64.38	63.58	158.78	164.61	162.62
OsR498G0713354100.01	0.113	0.34	1.580328	0.0003419	0.0007932	0.25	0.03	0.06	0.44	0.29	0.29
OsR498G0713355400.01	2.71	1.056	-1.36078	1.15E-13	1.80E-13	2.99	2.45	2.69	1.29	1.03	0.85
OsR498G0713357100.01	6.48	2.94	-1.03431	1.69E-82	1.16E-82	5.67	6.71	7.06	3.07	2.96	2.79
OsR498G0713359500.01	8.63	1.263	-2.76859	4.75E-222	1.82E-222	7.71	9.57	8.61	1.32	1.39	1.08
OsR498G0713370800.01	1.533	3.836	1.330275	7.04E-49	6.38E-49	1.48	1.78	1.34	3.52	3.67	4.32
OsR498G0713371200.01	4.92	24.866	2.336953	0	0	4.97	4.93	4.86	25.72	24.6	24.28
OsR498G0713372900.01	0.82	0.17	-2.30263	1.36E-10	2.34E-10	0.83	0.76	0.87	0.18	0.15	0.18
OsR498G0713375700.01	1.42	8.05	2.347748	1.82E-47	1.67E-47	1.74	1.52	1	7.77	7.5	8.88
OsR498G0713376000.01	0.273	2.31	3.069495	4.57E-34	4.89E-34	0.25	0.18	0.39	2.57	2.28	2.08
OsR498G0713382300.01	0.8	0.17	-2.32399	1.16E-23	1.46E-23	0.79	0.69	0.92	0.11	0.16	0.24
OsR498G0713387500.01	5.66	0.703	-3.01335	1.53E-56	1.29E-56	6.25	5.92	4.81	0.78	0.7	0.63
OsR498G0713392100.01	2.943	0.21	-3.77738	1.22E-60	9.91E-61	2.51	3.42	2.9	0.17	0.19	0.27
OsR498G0713392900.01	1.653	0.19	-3.12011	3.18E-26	3.81E-26	1.42	1.53	2.01	0.25	0.18	0.14
OsR498G0713394500.01	0.54	0.16	-1.88624	2.95E-07	5.75E-07	0.45	0.58	0.59	0.18	0.15	0.15
OsR498G0713394700.01	0.42	0	-4.28217	0.0007127	0.0016975	0.38	0.4	0.48	0	0	0
OsR498G0713401800.01	0.706	4.083	2.535841	3.50E-44	3.34E-44	0.48	0.87	0.77	4.8	3.54	3.91
OsR498G0713405700.01	0.74	0	-6.91444	8.58E-16	1.27E-15	0.82	0.71	0.69	0	0	0

OsR498G0713406500.01	25.97	10.03	-1.38053	9.59E-58	7.99E-58	27.42	25.98	24.51	10.77	10.88	8.44
OsR498G0713407400.01	0.27	0.006	-5.08952	7.37E-10	1.30E-09	0.37	0.24	0.2	0	0.02	0
OsR498G0713409200.01	1.056	0.27	-1.99694	1.22E-05	2.59E-05	1.61	0.39	1.17	0.1	0.2	0.51
OsR498G0713410800.01	1.633	0.5	-1.71156	2.79E-10	4.84E-10	1.5	1.72	1.68	0.44	0.51	0.55
OsR498G0713412200.01	0.44	1.84	2.334868	2.49E-35	2.62E-35	0.59	0.47	0.26	1.99	1.82	1.71
OsR498G0713412600.01	2.05	10.243	2.316804	4.46E-74	3.24E-74	2.56	2.08	1.51	9.36	10.93	10.44
OsR498G0713413600.01	0.32	1.256	2.394635	2.25E-13	3.55E-13	0.33	0.38	0.25	0.78	1.52	1.47
OsR498G0713414200.01	0.113	1.186	3.361688	4.47E-13	7.11E-13	0.06	0.06	0.22	0.93	1.74	0.89
OsR498G0713419000.01	3.25	1.516	-1.10982	1.04E-24	1.29E-24	3.31	2.89	3.55	1.75	1.39	1.41
OsR498G0713421700.01	112.2	13.14	-3.12729	0	0	107.35	112.81	116.4	12.29	13.1	14.03
OsR498G0713422500.01	0.16	0	-5.20817	3.61E-06	7.40E-06	0.2	0.08	0.2	0	0	0
OsR498G0713423300.01	0.476	4.13	3.110149	7.23E-24	9.05E-24	0.57	0.5	0.36	4.95	3.88	3.56
OsR498G0713425600.01	0.24	0.616	1.346421	0.000137	0.000309	0.34	0.12	0.26	0.7	0.52	0.63
OsR498G0713426100.01	6.683	3.31	-1.01419	2.55E-10	4.41E-10	7.13	6.69	6.23	3.27	2.95	3.71
OsR498G0713426900.01	0.036	0.743	4.466025	3.19E-21	4.20E-21	0.03	0.08	0	0.76	0.74	0.73
OsR498G0713428100.01	1.74	23.8	3.610312	0	0	1.44	2.13	1.65	24.25	22.59	24.56
OsR498G0713428300.01	0.053	1.43	4.769857	3.98E-14	6.16E-14	0.08	0.08	0	1.43	1.64	1.22
OsR498G0713428500.01	0.11	0.873	2.963738	3.96E-11	6.67E-11	0.09	0.1	0.14	1.07	0.64	0.91
OsR498G0713428800.01	0.143	1.566	3.429158	3.32E-56	2.80E-56	0.14	0.15	0.14	1.43	1.61	1.66
OsR498G0713428900.01	8.97	59.336	2.708574	1.67E-289	5.28E-290	7.38	9.55	9.98	60.21	57.15	60.65
OsR498G0713429300.01	2.17	67.953	4.929844	0	0	2.23	2.42	1.86	67.15	68.5	68.21
OsR498G0713429500.01	3.246	30.523	3.225719	0	0	3.37	2.86	3.51	30.52	31.22	29.83
OsR498G0713429700.01	0.773	3.063	1.996125	7.07E-53	6.16E-53	0.85	0.82	0.65	3.06	2.92	3.21
OsR498G0713430000.01	0.746	2.856	2.144317	3.87E-23	4.91E-23	0.82	0.98	0.44	3	2.61	2.96
OsR498G0713432900.01	0	0.406	5.499192	4.66E-07	9.16E-07	0	0	0	0.27	0.28	0.67
OsR498G0713433200.01	20.89	7.636	-1.49944	6.22E-81	4.31E-81	19.93	22.91	19.83	7.51	7.97	7.43
OsR498G0713435600.01	0.28	3.433	3.602696	1.54E-27	1.81E-27	0.31	0.16	0.37	3.67	3.05	3.58
OsR498G0713436800.01	20.643	116.19	2.476102	0	0	22.62	18.38	20.93	119.72	116.98	111.86
OsR498G0713453500.01	4.773	18.173	2.031156	1.37E-84	9.28E-85	4.28	5.56	4.48	18.94	19.23	16.35
OsR498G0713458800.01	0.07	0.376	2.361688	4.96E-07	9.78E-07	0.05	0.03	0.13	0.39	0.46	0.28
OsR498G0713459800.01	0.043	0.256	2.624723	2.62E-05	5.65E-05	0.03	0.07	0.03	0.28	0.2	0.29
OsR498G0713460400.01	0.04	0.21	2.31769	3.76E-05	8.17E-05	0.05	0.05	0.02	0.21	0.19	0.23
OsR498G0713461200.01	0.083	0.343	2.03976	3.78E-06	7.78E-06	0.1	0.03	0.12	0.35	0.39	0.29
OsR498G0713461300.01	1.733	6.34	1.638118	1.65E-132	8.67E-133	1.73	1.76	1.71	6	7.21	5.81
OsR498G0713461500.01	6.04	24.313	1.967842	1.05E-269	3.48E-270	5.95	5.93	6.24	24.23	24.25	24.46
OsR498G0713461800.01	4.876	51.13	3.390606	0	0	5.03	5.38	4.22	50.56	52.25	50.58
OsR498G0713462000.01	0.316	3.7	3.53352	2.95E-124	1.61E-124	0.32	0.21	0.42	3.82	3.5	3.78
OsR498G0713462200.01	40.036	12.373	-1.69641	0	0	38.55	42.38	39.18	11.98	12.07	13.07
OsR498G0713462300.01	11.386	2.24	-2.35158	6.04E-187	2.58E-187	10.52	11.84	11.8	2.02	2.63	2.07
OsR498G0713462600.01	62.77	198.44	1.656163	0	0	62.41	62.66	63.24	193.78	196.76	204.77
OsR498G0713463000.01	9.593	129.4	3.750292	0	0	9.32	9.96	9.5	127.57	131.12	129.5
OsR498G0713464300.01	1.046	12.88	4.019463	1.89E-286	6.02E-287	0.76	1.19	1.19	13.34	12.01	13.29
OsR498G0713465300.01	0.11	0.01	-3.5452	0.0005186	0.0012198	0.03	0.17	0.13	0	0.03	0
OsR498G0713471500.01	7.013	28.726	2.037404	0	0	7.21	6.6	7.23	28.03	28.87	29.28
OsR498G0713472300.01	0.226	3.87	4.058206	1.55E-119	8.63E-120	0.12	0.23	0.33	4.21	3.92	3.48
OsR498G0713472500.01	6.92	2.27	-1.59299	6.97E-52	6.13E-52	6.54	7.06	7.16	2.31	2.22	2.28
OsR498G0713472800.01	36.616	86.36	1.055715	0	0	36.72	37.76	35.37	83.5	87.98	87.6
OsR498G0713483300.01	0.126	37.163	8.169043	0	0	0.13	0	0.25	39.12	37.33	35.04
OsR498G0713492600.01	0.123	7.01	5.085316	1.39E-153	6.69E-154	0.09	0.1	0.18	7.31	7.64	6.08
OsR498G0713493200.01	1693	237.6	-2.82461	0	0	1686.6	1712.4	1680	252.59	226.05	234.15
OsR498G0713493600.01	7.24	2.29	-1.72668	5.95E-109	3.49E-109	6.33	7.58	7.81	2.37	2	2.5
OsR498G0713495300.01	5.006	0	-8.22703	6.33E-33	6.87E-33	5.12	5.6	4.3	0	0	0
OsR498G0713495500.01	0.05	0.48	3.209685	5.30E-05	0.0001164	0	0	0.15	0.71	0.41	0.32
OsR498G0713496900.01	121.41	5.256	-4.54319	0	0	120.27	124.39	119.6	5.08	5.79	4.9
OsR498G0713498600.01	2.386	1.096	-1.68816	3.89E-06	8.00E-06	1.74	2.37	3.05	1.49	0.55	1.25
OsR498G0713510500.01	1023.5	7.11	-7.21192	0	0	1052.4	1013.3	1005	7.59	7.06	6.68
OsR498G0713515500.01	3524.4	510.62	-2.79439	0	0	3496.6	3614.1	3463	534.96	479.09	517.82
OsR498G0713516800.01	13.89	70.773	2.338468	4.24E-303	1.29E-303	16.19	13.37	12.11	74.74	71.25	66.33
OsR498G0713518200.01	0.44	1.146	1.300885	1.76E-10	3.03E-10	0.46	0.53	0.33	1.13	1.23	1.08
OsR498G0713518400.01	16.853	5.71	-1.55955	3.95E-186	1.69E-186	16.67	17.07	16.82	6.23	5.55	5.35
OsR498G0713521400.01	27.853	86.016	1.620533	0	0	27.34	28.16	28.06	82.04	88.13	87.88
OsR498G0713526100.01	0.16	1.523	3.224185	6.59E-20	8.93E-20	0.04	0.18	0.26	1.3	1.9	1.37
OsR498G0713530800.01	0.15	0.37	1.302794	1.28E-06	2.57E-06	0.13	0.15	0.17	0.38	0.34	0.39
OsR498G0713538700.01	0.026	1.19	5.594349	1.60E-35	1.68E-35	0	0.08	0	0.99	1.49	1.09
OsR498G0713544200.01	1.06	0	-5.66068	7.26E-08	1.38E-07	0.87	0.76	1.55	0	0	0
OsR498G0713544300.01	4.503	0.08	-5.79641	1.83E-77	1.30E-77	4.55	4.45	4.51	0.13	0.11	0
OsR498G0713550300.01	0.906	2.456	1.431211	8.47E-11	1.44E-10	0.97	0.84	0.91	1.82	3.14	2.41
OsR498G0713550700.01	3.04	7.256	1.198011	1.45E-23	1.83E-23	3.27	2.99	2.86	7.4	6.26	8.11
OsR498G0713551000.01	49.473	19.6	-1.33883	1.85E-210	7.30E-211	50.39	50.64	47.39	19.15	20.69	18.96

OsR498G0713551200.01	0.633	1.763	1.454798	1.40E-05	2.97E-05	0.63	0.48	0.79	1.3	2.57	1.42
OsR498G0713554800.01	0.573	0.146	-1.96024	7.49E-08	1.42E-07	0.63	0.45	0.64	0.14	0.15	0.15
OsR498G0713560300.01	1.273	5.216	2.021382	2.08E-35	2.18E-35	1.55	0.96	1.31	5.27	5.14	5.24
OsR498G0713560700.01	1.16	3.363	1.535455	2.93E-43	2.82E-43	1.16	1.23	1.09	3.17	3.6	3.32
OsR498G0713561300.01	1.996	0.206	-3.25092	4.45E-26	5.35E-26	2.32	1.69	1.98	0.16	0.29	0.17
OsR498G0713563400.01	1259	617.83	-1.03608	0	0	1275.7	1230.7	1270	637.63	592.65	623.2
OsR498G0713563700.01	10.413	2.686	-1.97484	1.37E-122	7.54E-123	11.1	9.79	10.35	2.74	2.71	2.61
OsR498G0713565000.01	93.86	3.043	-5.01047	0	0	93.17	91.37	97.04	2.75	2.93	3.45
OsR498G0713568400.01	97.11	361.67	1.885872	0	0	103.1	91.54	96.69	395.33	354.95	334.73
OsR498G0713568600.01	1.876	6.65	1.816088	7.12E-32	7.83E-32	2.39	1.51	1.73	7.93	6.35	5.67
OsR498G0713569400.01	4.426	20.066	2.171252	3.98E-165	1.83E-165	4.39	4.12	4.77	21.8	20.12	18.28
OsR498G0713570600.01	0.516	2.843	2.466025	3.00E-37	3.08E-37	0.45	0.64	0.46	3.25	2.69	2.59
OsR498G0713571400.01	0.56	0.066	-3.13016	1.58E-09	2.81E-09	0.41	0.55	0.72	0.08	0.04	0.08
OsR498G0713572200.01	0.76	0.18	-2.10708	1.13E-08	2.07E-08	0.96	0.46	0.86	0.34	0.12	0.08
OsR498G0713572600.01	1.323	4.856	1.839461	1.30E-28	1.50E-28	1.08	1.74	1.15	4.78	4.48	5.31
OsR498G0713574400.01	0.02	0.543	4.76768	4.17E-14	6.46E-14	0	0	0.06	0.63	0.41	0.59
OsR498G0713574600.01	1.853	0.256	-2.8564	3.26E-54	2.80E-54	2.07	1.65	1.84	0.32	0.16	0.29
OsR498G0713575100.01	0.876	0.283	-1.6148	1.02E-06	2.03E-06	0.95	1.05	0.63	0.46	0.18	0.21
OsR498G0713575400.01	33.91	10.61	-1.67939	1.31E-277	4.27E-278	34.9	32.69	34.14	11.06	11.79	8.98
OsR498G0713578800.01	14.853	67.28	2.175787	0	0	14.14	15.22	15.2	66.1	68.93	66.81
OsR498G0713581400.01	3.67	0.97	-1.93948	3.27E-29	3.74E-29	3.23	3.43	4.35	0.61	1.22	1.08
OsR498G0713583000.01	0.253	0.89	1.817368	0.0005684	0.0013412	0.32	0.34	0.1	0.99	0.56	1.12
OsR498G0713584300.01	1.166	0.243	-2.27169	5.84E-30	6.60E-30	1.17	0.97	1.36	0.29	0.19	0.25
OsR498G0713584600.01	1.453	0.543	-1.20444	6.08E-09	1.11E-08	1.93	1.1	1.33	0.42	0.47	0.74
OsR498G0713585900.01	1.99	7.393	1.86655	8.79E-34	9.44E-34	2.45	1.58	1.94	8.16	7.38	6.64
OsR498G0713586200.01	40.263	142.04	1.651653	0	0	36.27	41.48	43.04	139.13	143.89	143.1
OsR498G0713588000.01	21.123	10.086	-1.07102	4.70E-160	2.20E-160	20.81	21.96	20.6	9.66	10.37	10.23
OsR498G0713588900.01	1.013	0.403	-1.22327	0.0003637	0.0008453	1.31	1.08	0.65	0.54	0.43	0.24
OsR498G0713590100.01	0.936	0.123	-2.93424	1.51E-19	2.07E-19	0.84	1.01	0.96	0.13	0.08	0.16
OsR498G0713590600.01	72.67	185.3	1.339599	0	0	70.57	75.88	71.56	193.85	183.79	178.27
OsR498G0713594700.01	0.076	7.27	6.558085	2.14E-66	1.65E-66	0	0.16	0.07	9.24	6.11	6.46
OsR498G0713594900.01	0.203	0.7	1.762226	0.0001037	0.0002319	0.3	0.13	0.18	0.44	0.83	0.83
OsR498G0713598700.01	0.246	0.823	1.344615	0.0002847	0.0006572	0.25	0.35	0.14	0.45	0.76	1.26
OsR498G0713600500.01	0.42	7.123	4.064706	1.80E-151	8.74E-152	0.35	0.43	0.48	7.4	6.83	7.14
OsR498G0713603300.01	4.163	0.543	-2.9463	1.15E-51	1.01E-51	4.67	4.01	3.81	0.41	0.68	0.54
OsR498G0713603500.01	5.81	2.516	-1.18503	7.81E-31	8.71E-31	5.87	5.41	6.15	2.23	3.06	2.26
OsR498G0713609200.01	5.073	2	-1.35965	5.85E-11	9.91E-11	5.35	4.9	4.97	1.94	1.88	2.18
OsR498G0713609400.01	2.506	0.27	-3.21147	3.92E-40	3.89E-40	2.74	2.37	2.41	0.38	0.18	0.25
OsR498G0713610900.01	1.726	11.95	2.788117	3.79E-88	2.50E-88	1.5	2.17	1.51	12.87	11.61	11.37
OsR498G0713613000.01	2.673	1.013	-1.40928	5.88E-28	6.85E-28	2.72	2.53	2.77	0.84	1.12	1.08
OsR498G0713615700.01	0.123	0.863	3.019582	6.40E-14	9.96E-14	0.18	0.1	0.09	0.79	1	0.8
OsR498G0713618700.01	0.59	0	-7.24564	5.05E-19	6.99E-19	0.46	0.55	0.76	0	0	0
OsR498G0713620600.01	441.09	141.5	-1.64874	0	0	451.78	423.91	447.6	146.49	135.07	142.94
OsR498G0713621600.01	17.24	52.033	1.586669	1.22E-237	4.45E-238	16.75	17.71	17.26	55.96	52.03	48.11
OsR498G0713621900.01	1.726	0.51	-1.78127	1.76E-06	3.55E-06	0.97	1.95	2.26	0.1	0.82	0.61
OsR498G0713625700.01	244.95	1099.4	2.157814	0	0	253.6	242.12	239.1	1187.4	1061.6	1049.2
OsR498G0713625900.01	511.7	5578.2	3.437037	0	0	493.22	521.79	520.1	6086.1	5287.3	5361.3
OsR498G0713627600.01	7.77	43.033	2.444179	0	0	7.72	8.49	7.1	43.39	42.17	43.54
OsR498G0713628000.01	11.296	5.066	-1.16529	3.06E-57	2.57E-57	11.63	10.6	11.66	4.53	5.59	5.08
OsR498G0713630600.01	81.306	26.846	-1.49305	0	0	73.8	85.86	84.26	26.15	27.87	26.52
OsR498G0713631300.01	63.02	24.226	-1.32917	0	0	61.65	62.57	64.84	23.25	24.49	24.94
OsR498G0713633700.01	0.77	2.2	1.569421	1.67E-25	2.03E-25	0.95	0.68	0.68	2.38	2.17	2.05
OsR498G0713634400.01	15.85	37.146	1.224734	3.20E-138	1.65E-138	15.81	15.73	16.01	36.75	40.75	33.94
OsR498G0713637100.01	0.11	0.026	-1.96024	5.52E-05	0.0001214	0.1	0.11	0.12	0.02	0.03	0.03
OsR498G0713643700.01	10.43	3.31	-1.68282	2.03E-107	1.20E-107	9.62	10.22	11.45	3.27	3.4	3.26
OsR498G0713645200.01	20.996	9.9	-1.07999	1.24E-92	7.95E-93	21.85	20.54	20.6	10.21	9.92	9.57
OsR498G0713653700.01	7.39	29.17	1.93601	0	0	7.47	6.42	8.28	27.13	29.43	30.95
OsR498G0713654200.01	80.126	517.05	2.687306	0	0	81.88	81.98	76.52	506.2	525.67	519.28
OsR498G0713656400.01	0.51	1.516	1.571142	6.42E-16	9.50E-16	0.5	0.5	0.53	1.4	1.67	1.48
OsR498G0713658300.01	136.84	39.953	-1.76616	0	0	141	138.67	130.8	38.29	39.44	42.13
OsR498G0713661100.01	2.25	0.323	-2.49988	8.83E-19	1.23E-18	2.63	2.36	1.76	0.41	0.17	0.39
OsR498G0713669500.01	31.196	203.92	2.705667	0	0	31.34	30.97	31.28	199.27	205.4	207.1
OsR498G0713671700.01	0.056	0.23	2.03976	2.34E-08	4.35E-08	0.08	0.05	0.04	0.28	0.21	0.2
OsR498G0713672300.01	4.796	2.24	-1.08978	7.53E-46	7.06E-46	4.82	4.9	4.67	2.3	2.13	2.29
OsR498G0713673300.01	0.423	18.386	5.434252	0	0	0.42	0.42	0.43	19.87	17.81	17.48
OsR498G0713675100.01	1.356	0.6	-1.4259	2.92E-09	5.25E-09	1.1	1.54	1.43	0.47	0.6	0.73
OsR498G0713681500.01	1.243	2.543	1.01686	1.72E-11	2.87E-11	1.14	1.08	1.51	2.91	1.96	2.76
OsR498G0713682400.01	2.123	0.336	-2.09774	3.47E-21	4.58E-21	1.38	2.79	2.2	0.35	0.22	0.44
OsR498G0713684800.01	1.58	31.16	4.298748	0	0	1.69	1.46	1.59	29.83	32.26	31.39

OsR498G0713685000.01	0.256	0.656	1.368086	8.16E-09	1.49E-08	0.27	0.34	0.16	0.56	0.8	0.61
OsR498G0713686000.01	62.423	182.26	1.532195	0	0	63.99	60.05	63.23	187.36	187.51	171.91
OsR498G0713686200.01	1.153	0.466	-1.30354	2.44E-11	4.09E-11	1.17	1.19	1.1	0.41	0.38	0.61
OsR498G0713687000.01	0.196	0	-5.4838	3.79E-07	7.43E-07	0.23	0.21	0.15	0	0	0
OsR498G0713690400.01	79.25	36.19	-1.20107	8.25E-218	3.19E-218	78.47	80.08	79.2	37.01	36.27	35.29
OsR498G0713690500.01	0.306	0.07	-2.13016	8.77E-05	0.0001953	0.44	0.25	0.23	0.03	0.14	0.04
OsR498G0713693700.01	1.373	0.1	-3.81822	6.72E-31	7.49E-31	1.24	1.71	1.17	0.09	0.12	0.09
OsR498G0713697500.01	27.54	11.623	-1.25387	2.37E-73	1.74E-73	30.09	25.44	27.09	12.34	10.88	11.65
OsR498G0713710700.01	0.586	1.183	1.094208	1.70E-06	3.44E-06	0.49	0.7	0.57	1.31	1.3	0.94
OsR498G0713711600.01	3.203	1.23	-1.33662	1.14E-09	2.01E-09	2.92	4.59	2.1	1.2	1	1.49
OsR498G0713711700.01	7.363	53.71	2.859336	0	0	7.66	7.62	6.81	52.19	56.9	52.04
OsR498G0713719700.01	0.043	0.39	3.209685	5.30E-05	0.0001164	0	0.07	0.06	0.51	0.33	0.33
OsR498G0713720700.01	0.04	0.373	2.118724	5.24E-05	0.0001149	0.08	0.04	0	0.2	0.92	0
OsR498G0713721600.01	90.506	14.94	-2.60505	0	0	94.28	88.91	88.33	14.63	15.37	14.82
OsR498G0713721900.01	1.57	0.273	-2.5452	6.09E-06	1.27E-05	1.71	1.65	1.35	0	0.49	0.33
OsR498G0713723400.01	0.433	0.03	-3.75032	2.73E-18	3.83E-18	0.45	0.49	0.36	0.06	0.03	0
OsR498G0713731400.01	2.903	0.216	-3.74729	3.86E-127	2.07E-127	2.74	3.28	2.69	0.18	0.29	0.18
OsR498G0713732500.01	6.59	2.53	-1.38778	5.70E-28	6.65E-28	6.94	6.7	6.13	2.36	2.92	2.31
OsR498G0713741900.01	6.113	1.1	-2.47721	1.48E-108	8.69E-109	6.27	6.15	5.92	1.02	1.16	1.12
OsR498G0713744000.01	4.6	10.71	1.209224	3.47E-161	1.62E-161	3.86	5.32	4.62	11.19	10.1	10.84
OsR498G0713751900.01	89.323	39.226	-1.14629	0	0	86.48	90.24	91.25	41.63	37.5	38.55
OsR498G0713753400.01	2.936	0.57	-2.41155	8.60E-57	7.23E-57	3	2.67	3.14	0.6	0.52	0.59
OsR498G0713759600.01	10.326	1.416	-2.85903	4.66E-279	1.51E-279	10.44	10.27	10.27	1.32	1.59	1.34
OsR498G0713761600.01	4.233	0.09	-5.57495	5.40E-36	5.64E-36	3.33	5.57	3.8	0	0.18	0.09
OsR498G0713783500.01	18.416	10.416	-1.06688	1.07E-66	8.28E-67	16.71	17.59	20.95	12.3	9.69	9.26
OsR498G0713793200.01	0.07	0	-4.51851	0.0002436	0.00056	0	0.13	0.08	0	0	0
OsR498G0713795500.01	0	1.306	4.361688	0.0005877	0.0013892	0	0	0	0.79	1.95	1.18
OsR498G0713805100.01	0.363	0.916	1.272421	5.28E-07	1.04E-06	0.33	0.34	0.42	1.07	0.74	0.94
OsR498G0713805700.01	2.636	60.723	4.5262	0	0	1.63	3.56	2.72	63.42	59.11	59.64
OsR498G0713819100.01	5.28	1.196	-2.11824	2.33E-55	1.98E-55	5.51	4.64	5.69	1.35	1.11	1.13
OsR498G0713820300.01	4.96	12.193	1.289236	3.86E-28	4.48E-28	4.68	5.24	4.96	13.31	11.53	11.74
OsR498G0713822000.01	1.166	0.24	-2.28217	2.86E-15	4.30E-15	1.05	1.16	1.29	0.29	0.23	0.2
OsR498G0713822600.01	1.163	0.236	-2.31074	1.76E-14	2.70E-14	1.23	1.11	1.15	0.24	0.29	0.18
OsR498G0713823200.01	1.873	0.9	-1.06142	5.91E-12	9.72E-12	1.78	2.01	1.83	0.93	0.96	0.81
OsR498G0713823800.01	0.916	0.313	-1.56015	6.29E-09	1.14E-08	0.87	1.06	0.82	0.29	0.33	0.32
OsR498G0713824800.01	7.816	2.253	-1.82585	5.22E-71	3.90E-71	8.24	6.95	8.26	2.2	2.2	2.36
OsR498G0713826600.01	14.776	63.943	2.105197	0	0	15.11	14.57	14.65	62.5	65.06	64.27
OsR498G0713828700.01	1.89	7.75	2.018795	1.55E-27	1.81E-27	2.03	1.67	1.97	7.67	8.85	6.73
OsR498G0713833100.01	64.74	134.73	1.050539	0	0	63.83	62.72	67.67	139.23	133.58	131.38
OsR498G0713833600.01	14.403	38.98	1.412576	3.98E-55	3.39E-55	14.59	13.07	15.55	39.21	40.08	37.65
OsR498G0713843700.01	3.61	1.733	-1.06876	1.02E-18	1.42E-18	3.87	3.72	3.24	1.85	1.52	1.83
OsR498G0713855000.01	12.056	1.07	-3.51044	9.29E-110	5.42E-110	12.11	12.06	12	0.77	1.52	0.92
OsR498G0713856800.01	0.18	0.413	1.219084	9.12E-06	1.92E-05	0.17	0.23	0.14	0.38	0.33	0.53
OsR498G0713859300.01	8.74	1.486	-2.56321	2.78E-125	1.51E-125	9.25	9.06	7.91	1.31	1.69	1.46
OsR498G0713860700.01	5.253	0.57	-3.26823	3.92E-93	2.51E-93	5.75	4.99	5.02	0.59	0.38	0.74
OsR498G0713864300.01	0.37	6.466	4.105849	6.16E-74	4.49E-74	0.5	0.29	0.32	6.6	6.52	6.28
OsR498G0713869100.01	101.66	39.59	-1.37632	2.35E-190	9.94E-191	105.98	94.97	104	42.25	38.95	37.57
OsR498G0713875100.01	22.906	104.56	2.18341	0	0	22.8	22.8	23.12	99.65	103.16	110.88
OsR498G0713875600.01	2.856	1.213	-1.27218	2.41E-17	3.45E-17	3.11	2.63	2.83	1.06	1.09	1.49
OsR498G0713885800.01	4.163	1.25	-1.8216	5.61E-65	4.39E-65	4.46	3.7	4.33	1.02	1.41	1.32
OsR498G0713886300.01	1.23	0.066	-4.26402	8.86E-19	1.23E-18	1.16	1.29	1.24	0.05	0.1	0.05
OsR498G0713888200.01	0.446	4.416	3.282121	7.87E-73	5.78E-73	0.49	0.24	0.61	4.59	4.2	4.46
OsR498G0713889300.01	17.256	81.996	2.244807	0	0	17.08	18.33	16.36	85.99	81.46	78.54
OsR498G0713892000.01	0.496	1.313	1.398841	1.49E-05	3.16E-05	0.45	0.61	0.43	1.31	1.42	1.21
OsR498G0713895900.01	1.436	0.58	-1.31388	4.63E-20	6.25E-20	1.75	1.19	1.37	0.65	0.47	0.62
OsR498G0713897300.01	12.683	1.49	-3.10176	6.22E-87	4.14E-87	14.15	12.26	11.64	1.16	1.74	1.57
OsR498G0713898100.01	12.546	2.556	-2.35396	6.82E-67	5.24E-67	12.2	12.95	12.49	3.29	2.31	2.07
OsR498G0713916200.01	0.313	0.04	-3.20817	1.82E-08	3.36E-08	0.32	0.38	0.24	0.03	0.05	0.04
OsR498G0713920700.01	3.73	1.856	-1.01506	6.86E-13	1.10E-12	3.64	3.49	4.06	1.73	1.83	2.01
OsR498G0713940400.01	0.84	0.393	-1.09215	3.89E-06	8.00E-06	1	0.79	0.73	0.57	0.33	0.28
OsR498G0713948900.01	0.08	0.86	3.461224	3.15E-16	4.63E-16	0.07	0.07	0.1	0.98	0.91	0.69
OsR498G0713959500.01	2.413	0.033	-6.03349	1.06E-48	9.61E-49	2.58	2.55	2.11	0.07	0.03	0
OsR498G0713969200.01	4.563	24.296	2.40225	0	0	4.66	4.32	4.71	25.16	24.3	23.43
OsR498G0713970900.01	1.846	3.893	1.072476	3.12E-11	5.25E-11	1.77	1.96	1.81	4.59	3.25	3.84
OsR498G0713971700.01	5.96	14.68	1.291807	1.92E-93	1.23E-93	5.51	5.55	6.82	14.16	14.35	15.53
OsR498G0713982500.01	0.97	0.43	-1.19244	4.36E-07	8.56E-07	0.87	0.66	1.38	0.58	0.34	0.37
OsR498G0713983000.01	1.45	0.213	-2.71259	1.40E-12	2.27E-12	1.56	1.65	1.14	0.22	0.12	0.3
OsR498G0713984000.01	4.856	12.64	1.389344	1.35E-106	8.04E-107	5.1	4.55	4.92	12.07	13.15	12.7
OsR498G0713984400.01	16.65	158.47	3.232588	0	0	19.39	13.92	16.64	165.92	153.79	155.69

OsR498G0713988000.01	4.136	0.873	-2.26402	6.15E-21	8.16E-21	4.08	3.71	4.62	0.97	0.83	0.82
OsR498G0713988700.01	5.35	2.363	-1.36749	6.19E-50	5.55E-50	4.58	5.43	6.04	1.94	2.94	2.21
OsR498G0713992100.01	9.033	0.97	-3.20513	6.18E-167	2.82E-167	8.62	9.44	9.04	1.12	0.88	0.91
OsR498G0713999000.01	30.023	78.956	1.392622	0	0	29.87	30.4	29.8	78.07	77.3	81.5
OsR498G0713999800.01	16.893	33.306	1.023841	1.29E-138	6.62E-139	17.27	16.93	16.48	32.51	35.07	32.34
OsR498G0714006500.01	0.763	0.25	-1.57691	3.43E-05	7.45E-05	0.81	0.94	0.54	0.32	0.16	0.27
OsR498G0714008000.01	0.91	0.29	-1.70346	3.25E-08	6.08E-08	1.2	0.81	0.72	0.23	0.3	0.34
OsR498G0714014300.01	0.296	0.026	-3.4838	4.22E-06	8.70E-06	0.22	0.35	0.32	0.04	0.04	0
OsR498G0714022900.01	10.716	1.9	-2.48593	1.66E-184	7.16E-185	10.5	11.12	10.53	1.87	1.99	1.84
OsR498G0714023300.01	17.753	4.44	-2.01056	1.77E-71	1.31E-71	18.41	17.37	17.48	5.27	4.25	3.8
OsR498G0714031400.01	1.413	0.106	-3.70918	1.29E-25	1.56E-25	1.43	1.5	1.31	0.18	0.07	0.07
OsR498G0714033000.01	74.223	30.226	-1.31481	5.27E-99	3.27E-99	77.35	71.85	73.47	34.69	31.92	24.07
OsR498G0714035300.01	5.853	17.72	1.428872	2.14E-131	1.13E-131	5.82	5.69	6.05	18.07	18.54	16.55
OsR498G0714035600.01	4.433	1.7	-1.43772	2.53E-25	3.08E-25	4.56	4.7	4.04	1.43	1.88	1.79
OsR498G0714039400.01	3.7	1.196	-1.60623	9.30E-15	1.41E-14	3.8	4.33	2.97	1.1	1.13	1.36
OsR498G0714046100.01	0.59	0.143	-2.5452	6.09E-06	1.27E-05	1.03	0.27	0.47	0.34	0	0.09
OsR498G0714073200.01	71.43	18.87	-1.93981	0	0	70.22	72.88	71.19	18.53	18.94	19.14
OsR498G0714075300.01	46.51	21.88	-1.09951	3.28E-252	1.14E-252	47.47	44.25	47.81	20.54	22.49	22.61
OsR498G0714084500.01	16.66	6.913	-1.30009	3.04E-87	2.02E-87	15.91	17.41	16.66	7.31	6.39	7.04
OsR498G0714086300.01	0	0.296	4.847115	5.08E-05	0.0001113	0	0	0	0.12	0.32	0.45
OsR498G0714089600.01	0.183	1.296	2.776726	4.50E-08	8.46E-08	0	0.1	0.45	1.44	1.38	1.07
OsR498G0714091600.01	0.033	0.403	3.54226	1.85E-08	3.43E-08	0.1	0	0	0.49	0.25	0.47
OsR498G0714096400.01	456.95	77.846	-2.55818	0	0	439.67	467.72	463.5	75.96	78.16	79.42
OsR498G0714097600.01	4.053	1.76	-1.21322	3.92E-16	5.78E-16	3.78	3.83	4.55	1.99	1.52	1.77
OsR498G0714098100.01	4.63	2.21	-1.07398	3.20E-13	5.06E-13	3.55	5.03	5.31	2.34	2.26	2.03
OsR498G0714111400.01	81.31	25.573	-1.70374	0	0	82.68	78.56	82.69	25.55	25.08	26.09
OsR498G0714112900.01	0.03	2.043	6.176717	4.84E-52	4.24E-52	0.03	0.03	0.03	1.68	2	2.45
OsR498G0714117000.01	0.91	1.923	1.072574	2.28E-16	3.34E-16	0.87	0.91	0.95	1.93	2.13	1.71
OsR498G0714121500.01	15.416	5.75	-1.38751	4.25E-101	2.60E-101	14.29	15.89	16.07	4.94	6.3	6.01
OsR498G0714126600.01	1.343	0.61	-1.13271	3.57E-08	6.68E-08	1.27	1.53	1.23	0.62	0.49	0.72
OsR498G0714154600.01	16.556	5.666	-1.62409	2.49E-128	1.33E-128	16	15.77	17.9	5.74	5.64	5.62
OsR498G0714154900.01	1060.3	109.38	-3.29111	0	0	1113.1	1058.3	1010	113.39	108	106.74
OsR498G0714158400.01	1.113	0.376	-1.5452	0.0004052	0.0009466	1.01	0.96	1.37	0.31	0.64	0.18
OsR498G0714159000.01	0.796	0	-7.26402	3.21E-19	4.42E-19	0.88	0.57	0.94	0	0	0
OsR498G0714165900.01	1.21	3.34	1.403165	9.91E-18	1.41E-17	1.63	1.1	0.9	3.54	3.25	3.23
OsR498G0714174100.01	20.233	2.333	-3.29088	0	0	21.02	19.72	19.96	2.51	2.28	2.21
OsR498G0714174300.01	0.37	36.81	6.625405	0	0	0.5	0.32	0.29	40.6	35.23	34.6
OsR498G0714178700.01	5.87	0.276	-4.42621	5.33E-50	4.78E-50	5.79	5.84	5.98	0.33	0.33	0.17
OsR498G0714181800.01	2.826	0.606	-2.22221	1.04E-65	8.12E-66	2.84	2.65	2.99	0.76	0.43	0.63
OsR498G0714182900.01	0.316	0	-5.4838	3.79E-07	7.43E-07	0.37	0.3	0.28	0	0	0
OsR498G0714183600.01	18.993	57.143	1.581902	0	0	19.13	18.37	19.48	59.26	55.89	56.28
OsR498G0714188400.01	26.553	110.07	2.111175	0	0	28.94	25.93	24.79	111.01	105.78	113.41
OsR498G0714189600.01	0.11	1.336	3.594349	6.53E-39	6.58E-39	0.18	0.09	0.06	1.23	1.3	1.48
OsR498G0714192500.01	0.716	2.683	1.911245	5.93E-18	8.37E-18	0.63	0.91	0.61	2.95	2.54	2.56
OsR498G0714195500.01	171.26	348.1	1.012942	0	0	167.57	174.23	172	362.16	346.4	335.75
OsR498G0714200800.01	63.05	11.553	-2.42027	0	0	66.33	63.19	59.63	11.46	11.87	11.33
OsR498G0714219700.01	6.056	2.33	-1.28857	5.23E-39	5.26E-39	6.56	6.13	5.48	2.4	2.13	2.46
OsR498G0714229100.01	0.576	0.05	-3.5452	0.0005186	0.0012198	0.42	0.9	0.41	0.15	0	0
OsR498G0714233700.01	19.56	0.933	-4.41581	5.96E-83	4.06E-83	19.18	15.96	23.54	0.98	1.16	0.66
OsR498G0714236700.01	0.256	0.74	1.525187	0.0009281	0.0022279	0.38	0.24	0.15	0.86	0.64	0.72
OsR498G0714240800.01	2.453	0.783	-1.65292	6.58E-13	1.05E-12	2.53	2.38	2.45	0.81	0.83	0.71
OsR498G0714248200.01	106.19	36.593	-1.54063	0	0	105.45	106.98	106.2	37.55	34.86	37.37
OsR498G0714260700.01	6.986	2.47	-1.60706	1.12E-43	1.08E-43	7.25	6.26	7.45	2.42	2.7	2.29
OsR498G0714261100.01	16.84	7.796	-1.12001	1.81E-39	1.82E-39	18.46	15.13	16.93	8.89	6.66	7.84
OsR498G0714263100.01	181.04	43.466	-2.14555	0	0	194.74	175.48	172.9	46.63	43.41	40.36
OsR498G0714263300.01	3.556	0.333	-3.41967	3.94E-20	5.30E-20	3.75	3.61	3.31	0.55	0	0.45
OsR498G0714263900.01	4.12	0.353	-3.5651	8.78E-16	1.30E-15	3.19	4.8	4.37	0.7	0	0.36
OsR498G0714265800.01	1.71	0.526	-1.73076	9.99E-07	2.00E-06	2.08	1.5	1.55	0.55	0.47	0.56
OsR498G0714267700.01	53.733	20.38	-1.39224	1.70E-270	5.64E-271	53.44	53.02	54.74	19.16	21.26	20.72
OsR498G0714268000.01	6.99	17.76	1.345889	5.49E-62	4.40E-62	7.03	7.11	6.83	17.97	17.78	17.53
OsR498G0714268500.01	12.56	38.116	1.599522	0	0	12.17	12.67	12.84	36.99	38.65	38.71
OsR498G0714269700.01	0.72	2.373	1.616109	2.75E-28	3.18E-28	0.56	0.68	0.92	2.5	2.78	1.84
OsR498G0714273300.01	0.593	0.14	-2.07572	7.16E-09	1.31E-08	0.41	0.85	0.52	0.14	0.17	0.11
OsR498G0714275200.01	0.806	0.303	-1.34415	4.38E-08	8.23E-08	0.74	0.94	0.74	0.28	0.24	0.39
OsR498G0714275700.01	2.49	6.886	1.460336	6.11E-73	4.48E-73	2.64	2.22	2.61	6.63	7.1	6.93
OsR498G0714283300.01	48.246	116.31	1.244586	0	0	48.4	48.76	47.58	115.17	115.64	118.12
OsR498G0714283900.01	102.42	4.253	-4.56153	0	0	103.86	103.17	100.2	4.47	3.95	4.34
OsR498G0714285400.01	1.263	0.21	-2.85836	1.06E-14	1.62E-14	1.48	1.05	1.26	0.23	0.22	0.18
OsR498G0714289000.01	1.136	3.716	1.728227	8.59E-36	8.99E-36	0.77	1.37	1.27	4.03	3.5	3.62

OsR498G0714294600.01	385.91	13.423	-4.85032	0	0	360.43	396.1	401.2	14.78	12.99	12.5
OsR498G0714294700.01	276.88	7.13	-5.2898	0	0	330.69	255.74	244.2	7.03	6.85	7.51
OsR498G0714302100.01	15.33	45.206	1.477674	2.28E-157	1.08E-157	17.28	13.96	14.75	47.97	44.46	43.19
OsR498G0714308600.01	0.46	0.173	-1.39689	8.00E-05	0.0001777	0.49	0.45	0.44	0.19	0.17	0.16
OsR498G0714308700.01	5.55	12.226	1.145113	6.21E-55	5.31E-55	5.49	6.22	4.94	12.51	12.16	12.01
OsR498G0714308900.01	1.37	4.56	1.73562	1.80E-77	1.27E-77	1.29	1.44	1.38	4.49	4.57	4.62
OsR498G0714309200.01	6.24	12.676	1.01949	6.47E-103	3.93E-103	5.49	6.61	6.62	12.17	13.06	12.8
OsR498G0714310200.01	0.853	11.513	3.750521	4.53E-201	1.85E-201	0.88	0.86	0.82	12.99	10.66	10.89
OsR498G0714316600.01	1.836	0.14	-3.78467	8.58E-19	1.19E-18	2.2	1.83	1.48	0.14	0.13	0.15
OsR498G0714317800.01	14.246	30.656	1.102411	1.37E-118	7.66E-119	13.83	15.09	13.82	30.23	30.01	31.73
OsR498G0714327600.01	1.073	0.136	-2.99586	1.49E-08	2.75E-08	1.33	0.91	0.98	0.16	0	0.25
OsR498G0714327900.01	1.123	0.04	-4.70171	8.64E-26	1.04E-25	1.03	1.5	0.84	0.06	0.03	0.03
OsR498G0714329200.01	2.743	0.516	-2.40246	3.12E-34	3.33E-34	2.88	3.01	2.34	0.6	0.58	0.37
OsR498G0714331100.01	1.25	6.606	2.385535	4.13E-42	4.02E-42	1.38	1.05	1.32	8.14	6.21	5.47
OsR498G0714336100.01	0.566	0.053	-3.44137	2.76E-14	4.25E-14	0.35	0.71	0.64	0.03	0	0.13
OsR498G0714336900.01	3.45	1.553	-1.16756	8.82E-08	1.68E-07	3.65	3.43	3.27	1.58	1.58	1.5
OsR498G0714341900.01	0.703	0.053	-3.66068	5.77E-12	9.49E-12	0.57	0.68	0.86	0.08	0.04	0.04
OsR498G0714343800.01	0.693	0.193	-1.84288	2.84E-07	5.53E-07	0.78	0.52	0.78	0.21	0.15	0.22
OsR498G0714344900.01	0.026	0.556	4.325162	1.73E-10	2.97E-10	0	0	0.08	0.38	0.65	0.64
OsR498G0714345300.01	1.023	0.296	-1.78621	9.56E-09	1.75E-08	0.78	1.27	1.02	0.32	0.33	0.24
OsR498G0714350300.01	41.29	9.383	-2.1578	0	0	42.67	39.36	41.84	9.11	9.89	9.15
OsR498G0714351200.01	3.213	1.463	-1.2389	1.62E-29	1.85E-29	3.48	2.69	3.47	1.39	1.54	1.46
OsR498G0714357500.01	15.526	43.316	1.463802	0	0	15.83	15.24	15.51	42.71	42.99	44.25
OsR498G0714359600.01	0.15	0.01	-3.28217	2.42E-05	5.21E-05	0.11	0.07	0.27	0	0.03	0
OsR498G0714368300.01	48.703	4.29	-3.51369	0	0	50.73	47.53	47.85	4.33	4.47	4.07
OsR498G0714374800.01	30.04	118.01	2.121731	0	0	28.64	30.39	31.09	120.74	118.76	114.53
OsR498G0714384000.01	2	0.156	-3.5585	1.29E-22	1.65E-22	2.13	1.99	1.88	0.1	0.16	0.21
OsR498G0714390500.01	0.816	0.36	-1.11603	2.33E-07	4.52E-07	0.65	0.93	0.87	0.37	0.29	0.42
OsR498G0714398700.01	6.753	15.54	1.244208	7.08E-114	4.06E-114	7.75	6.21	6.3	15.84	15.16	15.62
OsR498G0714401700.01	0.083	0.33	1.978359	0.0003815	0.0008887	0.08	0.09	0.08	0.3	0.39	0.3
OsR498G0714403500.01	20.616	6.486	-1.68352	5.10E-147	2.52E-147	21.26	19.14	21.45	6.86	6.7	5.9
OsR498G0714407100.01	0.046	1.053	4.51008	6.47E-32	7.11E-32	0.05	0.02	0.07	1.21	1.09	0.86
OsR498G0714418300.01	142.23	65.106	-1.11982	0	0	144.17	141.16	141.4	69.91	61.84	63.57
OsR498G0714419600.01	1.6	0.416	-2.16275	1.05E-15	1.56E-15	1.7	1.45	1.65	0.25	0.62	0.38
OsR498G0714419700.01	0.363	0.043	-3.09885	2.97E-10	5.16E-10	0.48	0.31	0.3	0.04	0.04	0.05
OsR498G0714421000.01	34.56	16.126	-1.08538	3.24E-170	1.46E-170	34.04	33.76	35.88	15.3	16.39	16.69
OsR498G0714425300.01	1.183	0.45	-1.46772	1.52E-11	2.54E-11	1.27	1.04	1.24	0.36	0.56	0.43
OsR498G0714425700.01	4.233	1.63	-1.38194	4.12E-15	6.22E-15	4.42	4.4	3.88	1.9	1.29	1.7
OsR498G0714427000.01	3.813	19.94	2.379357	3.87E-142	1.95E-142	3.13	4.3	4.01	19.37	20.78	19.67
OsR498G0714428500.01	0	0.413	4.847115	5.08E-05	0.0001113	0	0	0	0.44	0.4	0.4
OsR498G0714430800.01	14.66	6.63	-1.15397	4.40E-98	2.73E-98	14.93	14.76	14.29	6.72	6.73	6.44
OsR498G0714431500.01	16.933	0.686	-4.64273	1.76E-161	8.20E-162	17.31	16.53	16.96	0.57	0.75	0.74
OsR498G0714432100.01	0.523	0.176	-1.5452	5.29E-07	1.04E-06	0.39	0.7	0.48	0.22	0.2	0.11
OsR498G0714435900.01	0.06	1.416	4.54226	1.09E-32	1.19E-32	0.12	0.03	0.03	1.32	1.52	1.41
OsR498G0714438700.01	2.27	0.06	-5.27614	6.01E-57	5.05E-57	2.35	2.26	2.2	0.09	0	0.09
OsR498G0714440500.01	0.173	0.023	-2.96024	0.000243	0.0005585	0.06	0.24	0.22	0	0	0.07
OsR498G0714443100.01	0.51	0	-6.5649	5.59E-13	8.90E-13	0.48	0.6	0.45	0	0	0
OsR498G0714446100.01	5.67	0.713	-3.0969	8.04E-60	6.57E-60	5.77	5.64	5.6	0.75	0.77	0.62
OsR498G0714446500.01	13.393	3.313	-2.02703	4.44E-69	3.36E-69	14.25	12.34	13.59	3.26	2.9	3.78
OsR498G0714447600.01	0.053	0.813	3.973872	1.78E-24	2.20E-24	0	0.05	0.11	0.85	0.72	0.87
OsR498G0714450000.01	0.203	0.023	-3.0477	0.0001372	0.0003095	0.18	0.26	0.17	0	0.07	0
OsR498G0714454900.01	14.9	162.59	3.444959	0	0	13.98	15.69	15.03	154.52	166.06	167.2
OsR498G0714455300.01	1.323	0.383	-1.78515	7.81E-24	9.79E-24	1.15	1.64	1.18	0.3	0.41	0.44
OsR498G0714461000.01	0.21	2.253	3.460649	8.43E-35	8.93E-35	0.3	0.19	0.14	2.45	1.97	2.34
OsR498G0714462100.01	0.35	0.99	1.44415	8.85E-05	0.0001971	0.42	0.28	0.35	1.38	0.74	0.85
OsR498G0714465200.01	0.95	0.41	-1.22703	1.07E-05	2.26E-05	0.96	0.89	1	0.53	0.39	0.31
OsR498G0714466600.01	0.273	0.026	-3.6041	1.31E-06	2.63E-06	0.26	0.31	0.25	0.05	0	0.03
OsR498G0714469600.01	1.293	0.193	-2.75032	2.37E-14	3.64E-14	1.26	1.27	1.35	0.28	0.15	0.15
OsR498G0714472000.01	0.06	1.22	4.376043	2.22E-24	2.76E-24	0.07	0.04	0.07	1.17	1.51	0.98
OsR498G0714472500.01	0.01	1.046	6.578919	1.05E-23	1.32E-23	0.03	0	0	1.26	0.86	1.02
OsR498G0714473100.01	1.903	0.276	-2.97519	1.14E-17	1.62E-17	1.78	1.75	2.18	0.3	0.38	0.15
OsR498G0714480600.01	0	15.046	11.85834	2.33E-222	8.89E-223	0	0	0	16.18	14.8	14.16
OsR498G0714482300.01	12.846	2.546	-2.34337	1.02E-119	5.68E-120	12.37	12.28	13.89	2.71	2.63	2.3
OsR498G0714483400.01	2.156	0.65	-1.69538	1.53E-23	1.92E-23	2.14	2.07	2.26	0.61	0.73	0.61
OsR498G0714483700.01	6.95	2.493	-1.48031	8.15E-30	9.23E-30	6.92	7.63	6.3	2.71	1.95	2.82
OsR498G0714484500.01	66.886	18.296	-1.9155	0	0	66.53	66.67	67.46	18.05	18.5	18.34
OsR498G0714484800.01	18.603	2.016	-3.17859	2.87E-282	9.24E-283	18.92	17.5	19.39	1.89	1.87	2.29
OsR498G0714490800.01	4.776	1.476	-1.51687	1.23E-26	1.46E-26	4.5	4.98	4.85	1.08	1.66	1.69
OsR498G0714490900.01	0.873	0.15	-2.5452	1.25E-07	2.39E-07	0.81	0.91	0.9	0.25	0.07	0.13

OsR498G0714491100.01	1.1	0.143	-2.96024	1.12E-05	2.37E-05	0.95	1.43	0.92	0	0.29	0.14
OsR498G0714494400.01	0.493	1.673	1.742559	2.45E-15	3.68E-15	0.38	0.43	0.67	1.74	1.93	1.35
OsR498G0714494900.01	0.186	0.646	1.755967	0.0009051	0.0021704	0.24	0.09	0.23	0.75	0.43	0.76
OsR498G0714495300.01	0.73	4.14	2.576894	2.44E-40	2.42E-40	0.83	0.51	0.85	3.76	4.64	4.02
OsR498G0714496300.01	0.343	5.08	3.895251	2.11E-122	1.16E-122	0.13	0.39	0.51	5.47	4.87	4.9
OsR498G0714497100.01	2.466	5.583	1.249077	3.22E-37	3.31E-37	2.65	2.27	2.48	6.17	5.14	5.44
OsR498G0714497700.01	1.193	0.206	-2.5578	7.21E-18	1.02E-17	1.19	1.44	0.95	0.19	0.16	0.27
OsR498G0714500100.01	1.436	0.573	-1.30338	1.76E-09	3.12E-09	1.81	1.18	1.32	0.56	0.65	0.51
OsR498G0714500300.01	1.13	2.7	1.218324	3.68E-19	5.06E-19	1.13	1.14	1.12	2.43	2.83	2.84
OsR498G0714501100.01	1.84	8.013	2.116111	4.79E-35	5.07E-35	2.3	1.92	1.3	7.9	6.94	9.2
OsR498G0714501300.01	3.553	1.45	-1.34027	6.33E-13	1.01E-12	3.77	3.31	3.58	1.78	1.27	1.3
OsR498G0714501900.01	0.176	0.45	1.332542	0.0001149	0.0002578	0.16	0.11	0.26	0.38	0.31	0.66
OsR498G0714502100.01	0.86	4.743	2.50044	2.67E-82	1.83E-82	0.59	0.97	1.02	4.76	4.74	4.73
OsR498G0714502600.01	0.033	0.183	2.683616	1.50E-05	3.19E-05	0	0.03	0.07	0.12	0.18	0.25
OsR498G0714502900.01	0	0.08	4.499192	0.0003163	0.0007323	0	0	0	0.11	0.04	0.09
OsR498G0714503300.01	0.136	0.766	2.491456	8.91E-15	1.36E-14	0.14	0.13	0.14	0.73	0.6	0.97
OsR498G0714503600.01	0	0.216	5.499192	4.66E-07	9.16E-07	0	0	0	0.17	0.36	0.12
OsR498G0714504000.01	0.876	2.673	1.604713	6.76E-30	7.64E-30	0.78	0.91	0.94	2.52	2.76	2.74
OsR498G0714504400.01	0.406	3.36	3.053566	7.75E-74	5.65E-74	0.4	0.49	0.33	3.57	3.09	3.42
OsR498G0714504900.01	1.4	3.083	1.263212	1.20E-28	1.38E-28	1.13	1.77	1.3	3.04	3.1	3.11
OsR498G0714506800.01	1.666	0.756	-1.02798	5.93E-06	1.23E-05	2.22	1.29	1.49	1	0.5	0.77
OsR498G0714507100.01	0.996	3.66	1.864967	2.16E-34	2.30E-34	0.88	0.99	1.12	3.1	4.29	3.59
OsR498G0714508400.01	0.01	0.18	4.361688	3.01E-06	6.15E-06	0.03	0	0	0.21	0.08	0.25
OsR498G0714510100.01	2.016	4.143	1.065912	9.80E-23	1.25E-22	2.18	2.08	1.79	3.86	3.78	4.79
OsR498G0714510800.01	0.026	0.206	2.914229	2.18E-05	4.67E-05	0	0	0.08	0.22	0.12	0.28
OsR498G0714513000.01	1.193	0.496	-1.27404	2.65E-05	5.71E-05	1.17	1.26	1.15	0.32	0.66	0.51
OsR498G0714515000.01	9.576	37.683	2.081295	0	0	9.23	9.65	9.85	38.47	38.56	36.02
OsR498G0714515300.01	1.14	10.453	3.199808	0	0	0.73	1.5	1.19	10.37	10.64	10.35
OsR498G0714516300.01	0.06	0.29	2.209685	3.95E-06	8.13E-06	0.07	0.02	0.09	0.31	0.17	0.39
OsR498G0714516900.01	7.103	2.753	-1.37909	1.18E-55	1.00E-55	6.87	7.15	7.29	2.81	2.56	2.89
OsR498G0714517800.01	0.626	1.743	1.504723	7.70E-16	1.14E-15	0.55	0.69	0.64	1.73	1.8	1.7
OsR498G0714518300.01	0.6	2.933	2.30535	1.64E-30	1.83E-30	0.68	0.71	0.41	2.35	3.32	3.13
OsR498G0714519200.01	0.956	0.173	-2.3992	4.45E-13	7.07E-13	0.95	1.14	0.78	0.19	0.2	0.13
OsR498G0714521700.01	7.04	2.696	-1.44033	2.25E-54	1.93E-54	5.95	7.58	7.59	2.36	3.17	2.56
OsR498G0714524300.01	845.87	37.796	-4.46757	0	0	897.78	801.77	838.1	41.5	36.48	35.41
OsR498G0714524600.01	19.313	0.71	-4.76867	0	0	19.45	19.29	19.2	0.9	0.57	0.66
OsR498G0714525100.01	0.27	0	-4.76759	6.52E-05	0.0001441	0.11	0.26	0.44	0	0	0
OsR498G0714527300.01	18.99	44.89	1.229182	0	0	17.86	19.94	19.17	44.3	43.68	46.69
OsR498G0714527700.01	91.423	25.743	-1.87981	0	0	92.99	91.33	89.95	25.96	25.48	25.79
OsR498G0714527900.01	900.12	107.46	-3.09161	0	0	905.1	888.5	906.8	105.91	113.01	103.45
OsR498G0714528300.01	1.966	0.2	-3.30601	8.50E-13	1.36E-12	2.13	1.43	2.34	0.5	0.1	0
OsR498G0714533900.01	24.35	3.95	-2.63272	0	0	23.38	24.63	25.04	3.53	4.28	4.04
OsR498G0714534700.01	19.606	9.41	-1.07789	2.95E-70	2.22E-70	23.11	18.1	17.61	8.67	9.65	9.91
OsR498G0714535000.01	34.823	17.44	-1.00206	4.08E-90	2.67E-90	35.48	34.36	34.63	16.97	17.95	17.4
OsR498G0714535600.01	4.54	1.703	-1.42737	6.01E-20	8.14E-20	4.87	4	4.75	1.52	1.83	1.76
OsR498G0714537700.01	30	14.116	-1.10077	7.77E-40	7.75E-40	29.7	30.79	29.51	15.47	13.78	13.1
OsR498G0714539100.01	0	0.433	6.209685	2.10E-10	3.63E-10	0	0	0	0.39	0.33	0.58
OsR498G0714541400.01	0.366	9.623	4.764164	0	0	0.36	0.45	0.29	9.08	10.22	9.57
OsR498G0714549300.01	4.243	14.793	1.981194	0	0	3.69	4.4	4.64	14.42	14.79	15.17
OsR498G0714549600.01	0.766	2.113	1.441858	0.0003225	0.0007473	0.82	0.69	0.79	2.04	1.55	2.75
OsR498G0714549800.01	1.023	9.53	3.210477	3.36E-54	2.89E-54	0.48	1.42	1.17	7.67	10.08	10.84
OsR498G0714550000.01	2.673	11.06	2.044952	3.87E-177	1.71E-177	3.26	2.34	2.42	11.47	11.63	10.08
OsR498G0714554300.01	1.596	0.77	-1.05488	7.93E-07	1.58E-06	1.86	1.39	1.54	1	0.72	0.59
OsR498G0714556700.01	1.02	0.343	-1.57799	1.90E-08	3.53E-08	0.68	1.32	1.06	0.31	0.32	0.4
OsR498G0714559100.01	2370.2	957.01	-1.32474	0	0	2415.3	2377.6	2318	1022.9	915.3	932.87
OsR498G0714559300.01	4.123	1.99	-1.05187	2.48E-17	3.55E-17	3.86	4.14	4.37	2.18	1.69	2.1
OsR498G0714565000.01	12.08	29.726	1.296572	6.16E-233	2.28E-233	13	11.94	11.3	28.89	29.95	30.34
OsR498G0714565800.01	0.073	6.273	6.421736	3.52E-100	2.17E-100	0	0.18	0.04	5.93	6.55	6.34
OsR498G0714567400.01	21.016	3.053	-2.87466	0	0	21.75	20.36	20.94	2.93	3.19	3.04
OsR498G0714568000.01	1611.8	18.016	-6.49694	0	0	1649	1592.7	1594	18.47	18.18	17.4
OsR498G0714569500.01	0.8	0.163	-2.28217	0.0003536	0.0008216	0.83	1	0.57	0.37	0	0.12
OsR498G0714569800.01	5.606	18.026	1.678992	9.09E-106	5.43E-106	6.37	5.14	5.31	19.27	17.68	17.13
OsR498G0714575800.01	0.53	30.7	5.828388	0	0	0.71	0.28	0.6	29.35	31.69	31.06
OsR498G0714577600.01	0.696	0.136	-2.36234	3.01E-11	5.06E-11	0.59	0.82	0.68	0.2	0.09	0.12
OsR498G0714578700.01	1468.6	463.04	-1.67204	0	0	1529.1	1442.5	1434	496.04	451.64	441.43
OsR498G0714582200.01	0.106	3.006	4.782724	1.57E-57	1.31E-57	0.14	0.04	0.14	2.94	3.08	3
OsR498G0714583800.01	88.366	200.85	1.181502	0	0	87.42	91.63	86.05	203.61	196.79	202.16
OsR498G0714584800.01	0.086	2.12	4.598727	1.92E-39	1.93E-39	0.04	0.08	0.14	2.16	2.23	1.97
OsR498G0714585900.01	0.293	11.463	5.277603	2.28E-305	6.90E-306	0.19	0.45	0.24	11.07	12.71	10.61

OsR498G0714588000.01	2.173	6.423	1.555681	1.21E-47	1.11E-47	2.32	2.15	2.05	6.89	6.69	5.69
OsR498G0714588200.01	0.956	0.406	-1.18541	3.67E-10	6.39E-10	0.72	1	1.15	0.37	0.5	0.35
OsR498G0714591300.01	0.456	9.003	4.268579	2.41E-51	2.13E-51	0.34	0.36	0.67	9.97	8.48	8.56
OsR498G0714591800.01	12.96	58.983	2.090386	0	0	14.11	11.65	13.12	56.38	61.63	58.94
OsR498G0714592800.01	0.38	0.896	1.226638	2.13E-07	4.12E-07	0.41	0.38	0.35	1.16	1	0.53
OsR498G0714594600.01	3.696	7.933	1.071238	2.22E-19	3.04E-19	4.43	3.58	3.08	7.28	8.82	7.7
OsR498G0714594900.01	0.883	0.22	-2.0571	4.38E-10	7.64E-10	1.1	0.72	0.83	0.28	0.18	0.2
OsR498G0714596900.01	14.456	42.71	1.562013	0	0	14.36	13.62	15.39	42.74	42.29	43.1
OsR498G0714598000.01	16.346	126.48	2.951544	0	0	17.19	16.09	15.76	122.67	132.6	124.17
OsR498G0714598700.01	0.54	3.773	2.951704	1.27E-25	1.54E-25	0.46	0.61	0.55	3.2	3.61	4.51
OsR498G0714599800.01	0.156	0	-4.5452	0.0002138	0.0004889	0.24	0.12	0.11	0	0	0
OsR498G0714601400.01	7.846	16.363	1.056997	1.07E-53	9.27E-54	7.35	7.48	8.71	15.56	16.22	17.31
OsR498G0714602000.01	163.75	6.876	-4.37778	0	0	170.16	158.18	162.9	6.79	7.71	6.13
OsR498G0714602100.01	27.536	1.78	-4.04316	0	0	26.31	26.97	29.33	1.81	1.71	1.82
OsR498G0714602700.01	10.306	22.586	1.125992	1.88E-68	1.43E-68	10.26	10.44	10.22	22.58	21.55	23.63
OsR498G0714602900.01	0.093	3.81	5.367687	2.96E-116	1.68E-116	0.09	0.1	0.09	4	3.57	3.86
OsR498G0714603200.01	2.34	5.87	1.326463	5.13E-59	4.23E-59	2.35	2.35	2.32	5.37	6.15	6.09
OsR498G0714606200.01	0.466	3.476	2.876965	2.80E-49	2.53E-49	0.34	0.43	0.63	3.39	3.07	3.97
OsR498G0714606400.01	56.35	114.28	1.017571	0	0	53.19	57.86	58	114.28	111.02	117.54
OsR498G0714606800.01	3.01	9.523	1.653482	4.00E-39	4.02E-39	3.26	2.85	2.92	10.36	8.77	9.44
OsR498G0714608200.01	0.846	0.34	-1.33875	8.72E-05	0.0001942	0.69	0.57	1.28	0.3	0.26	0.46
OsR498G0714609700.01	0.113	0.553	2.54226	1.64E-11	2.74E-11	0.17	0.12	0.05	0.74	0.45	0.47
OsR498G0714610500.01	1.746	0.433	-2.03063	1.49E-19	2.03E-19	1.84	1.66	1.74	0.35	0.46	0.49
OsR498G0714611100.01	1.313	0.37	-1.85062	5.19E-16	7.66E-16	1.27	1.22	1.45	0.37	0.26	0.48
OsR498G0714613300.01	0.15	28.853	7.530009	3.07E-200	1.25E-200	0.09	0.1	0.26	34.71	27.55	24.3
OsR498G0714613700.01	13.81	33.036	1.262462	2.97E-171	1.34E-171	14.41	13.31	13.71	33.13	34.42	31.56
OsR498G0714614600.01	5.18	2.44	-1.10585	2.25E-23	2.85E-23	4.92	5.66	4.96	2.72	2.26	2.34
OsR498G0714615200.01	93.306	221	1.310449	0	0	94.99	92.69	92.24	222.84	214.72	225.44
OsR498G0714615300.01	16.963	596.5	5.117703	0	0	17.29	16.9	16.7	622.95	567.53	599.01
OsR498G0714620200.01	3.98	0.17	-4.57167	5.60E-99	3.47E-99	3.6	4.08	4.26	0.19	0.26	0.06
OsR498G0714620600.01	4.653	24.706	2.402575	0	0	4.66	4.48	4.82	23.54	26.91	23.67
OsR498G0714621000.01	4.873	40.37	3.04796	0	0	4.79	4.8	5.03	42.64	38.51	39.96
OsR498G0714621700.01	0.026	1.586	5.77447	9.97E-53	8.70E-53	0.02	0.02	0.04	1.58	1.7	1.48
OsR498G0714622900.01	1.163	0.3	-1.93148	1.49E-16	2.17E-16	1.23	1.27	0.99	0.24	0.33	0.33
OsR498G0714623100.01	13.126	6.79	-1.04664	1.46E-81	1.01E-81	13.28	14.21	11.89	6.97	6.6	6.8
OsR498G0714623900.01	1525.4	254.45	-2.59434	0	0	1487.6	1553.1	1536	277.9	243.21	242.25
OsR498G0714624100.01	25.016	52.36	1.059895	4.05E-177	1.79E-177	25.36	25.31	24.38	51.38	53.22	52.48
OsR498G0714629300.01	1.556	0.486	-1.68816	1.57E-10	2.71E-10	1.19	1.57	1.91	0.36	0.46	0.64
OsR498G0714631800.01	0.59	2.106	1.984618	6.28E-10	1.10E-09	0.98	0.33	0.46	1.61	2.9	1.81
OsR498G0714632200.01	2.25	6.773	1.573992	5.10E-38	5.19E-38	2.42	2.15	2.18	7.65	6.35	6.32
OsR498G0714634400.01	8.293	17.156	1.029113	1.35E-49	1.22E-49	9.56	8.31	7.01	18.08	16.92	16.47
OsR498G0714634800.01	10.613	87.436	3.058734	0	0	10.3	11.16	10.38	89.37	87.93	85.01
OsR498G0714641300.01	117.54	1270.7	3.43028	0	0	112.59	118.24	121.8	1346.6	1248.9	1216.7
OsR498G0714641600.01	0	0.12	4.361688	0.0005877	0.0013892	0	0	0	0.11	0.11	0.14
OsR498G0714644200.01	0.37	0.826	1.168084	2.45E-06	4.99E-06	0.28	0.4	0.43	0.74	0.73	1.01
OsR498G0714646900.01	8.686	24.59	1.443241	1.26E-101	7.69E-102	7.47	9.35	9.24	25.61	23.06	25.1
OsR498G0714647100.01	2.853	32.78	3.517807	0	0	2.64	3.02	2.9	32.5	33.83	32.01
OsR498G0714647700.01	0.046	0.433	3.186601	6.34E-13	1.01E-12	0.06	0.02	0.06	0.39	0.43	0.48
OsR498G0714651300.01	11.493	24.31	1.073614	2.34E-94	1.49E-94	10.77	11.67	12.04	24.08	25.86	22.99
OsR498G0714653900.01	45.263	141.09	1.625879	0	0	48.76	43.48	43.55	146.06	143.82	133.38
OsR498G0714654200.01	2.69	5.806	1.10598	2.06E-32	2.25E-32	2.67	3.09	2.31	5.42	5.8	6.2
OsR498G0714655900.01	0.25	0.826	1.750253	5.89E-05	0.0001297	0.07	0.42	0.26	1.09	0.7	0.69
OsR498G0714656800.01	0.853	0.016	-6.26402	4.08E-20	5.51E-20	0.89	0.8	0.87	0	0	0.05
OsR498G0714664400.01	0.1	4.483	5.466025	1.33E-32	1.45E-32	0	0.11	0.19	5.15	4.11	4.19
OsR498G0714665300.01	1.646	6.156	1.899678	3.43E-57	2.87E-57	1.47	1.71	1.76	7.01	5.38	6.08
OsR498G0714667100.01	35.783	91.89	1.359001	0	0	35.38	37.04	34.93	88.48	94.49	92.7
OsR498G0714667400.01	0.033	0.293	3.069507	2.69E-10	4.66E-10	0.05	0.02	0.03	0.33	0.37	0.18
OsR498G0714670300.01	9.93	2.016	-2.28416	2.94E-90	1.92E-90	9.51	10.33	9.95	2.3	1.83	1.92
OsR498G0714670500.01	0.996	0.473	-1.09326	0.0002218	0.0005081	1.25	0.79	0.95	0.29	0.75	0.38
OsR498G0714676400.01	0.05	0.306	3.127223	9.61E-05	0.0002145	0	0.08	0.07	0.47	0.08	0.37
OsR498G0714681100.01	4.73	2.496	-1.05548	2.62E-34	2.79E-34	4.51	4.9	4.78	2.28	2.66	2.55
OsR498G0714682600.01	0.046	17.696	8.529608	0	0	0.05	0.02	0.07	19.93	16.49	16.67
OsR498G0714684600.01	2.036	0.79	-1.35256	1.67E-05	3.56E-05	2.09	2.59	1.43	0.87	1	0.5
OsR498G0714685300.01	0.183	10.343	5.787953	1.10E-155	5.23E-156	0.23	0.1	0.22	11.26	10.43	9.34
OsR498G0714691200.01	0.596	0.206	-1.47214	5.58E-07	1.10E-06	0.47	0.66	0.66	0.18	0.19	0.25
OsR498G0714695200.01	0.93	4.8	2.390615	3.76E-55	3.21E-55	1.08	0.76	0.95	4.39	4.86	5.15
OsR498G0714696400.01	0.09	4.216	5.521963	1.27E-214	4.96E-215	0.08	0.07	0.12	4.24	4.29	4.12
OsR498G0714700300.01	1.573	0.623	-1.32947	1.74E-21	2.27E-21	1.28	1.55	1.89	0.62	0.66	0.59
OsR498G0714703200.01	0.113	0.503	1.832318	5.29E-11	8.96E-11	0.05	0.14	0.15	0.34	0.41	0.76

OsR498G0714704400.01	0.716	3.57	2.342952	1.20E-21	1.57E-21	0.72	0.36	1.07	3.62	3.13	3.96
OsR498G0714708100.01	2.21	0.136	-4.03919	6.20E-37	6.39E-37	2.34	2.29	2	0.08	0.25	0.08
OsR498G0714708800.01	59.863	14.736	-1.99247	0	0	57.58	61.62	60.39	15.61	14.17	14.43
OsR498G0714709600.01	0	0.54	5.355906	1.54E-06	3.11E-06	0	0	0	0.4	0.66	0.56
OsR498G0714713800.01	0.28	0.103	-1.96024	0.0003753	0.0008736	0.25	0.22	0.37	0.13	0.07	0.11
OsR498G0714720100.01	1.473	11.463	2.935721	5.79E-132	3.05E-132	1.37	1.32	1.73	11.39	11.18	11.82
OsR498G0714721100.01	6.01	1.626	-1.85412	7.29E-74	5.32E-74	5.68	6.36	5.99	1.71	1.27	1.9
OsR498G0714722100.01	4.126	9.77	1.257351	3.23E-45	3.04E-45	4.23	3.87	4.28	9.76	9.95	9.6
OsR498G0714723300.01	2.693	6.27	1.214392	2.21E-33	2.38E-33	2.37	2.91	2.8	5.82	6.63	6.36
OsR498G0714724500.01	8.213	3.38	-1.30751	1.17E-35	1.22E-35	8.28	8.57	7.79	3.46	3.33	3.35
OsR498G0714725500.01	367.18	141.17	-1.45502	0	0	373.83	363.19	364.5	147.55	137.04	138.92
OsR498G0714726200.01	1.92	0.716	-1.44659	3.29E-22	4.25E-22	1.88	1.81	2.07	0.91	0.65	0.59
OsR498G0714726900.01	51.64	11.69	-2.14323	1.04E-207	4.13E-208	53.4	51.24	50.28	13.5	10.27	11.3
OsR498G0714729500.01	5.753	2.77	-1.11478	1.06E-59	8.67E-60	5.29	6.33	5.64	2.6	2.78	2.93
OsR498G0714732800.01	2.416	1.026	-1.2536	2.16E-08	4.01E-08	2.08	2.08	3.09	1.32	0.94	0.82
OsR498G0714733400.01	0	0.3	5.624723	1.50E-07	2.88E-07	0	0	0	0.3	0.34	0.26
OsR498G0714734900.01	15.59	4.67	-1.73785	2.92E-114	1.67E-114	15.38	16.38	15.01	5.21	4.03	4.77
OsR498G0714735400.01	23.563	11.156	-1.08924	3.22E-81	2.23E-81	22.43	23.72	24.54	12.33	10.52	10.62
OsR498G0714737500.01	7.456	4.016	-1.06612	3.26E-32	3.58E-32	7.49	7.75	7.13	4.01	3.91	4.13
OsR498G0714739200.01	82.563	10.566	-3.00924	2.36E-272	7.78E-273	77.68	84.96	85.05	9.36	12.18	10.16
OsR498G0714740700.01	2.09	0.313	-2.7313	2.57E-25	3.13E-25	2.31	2.04	1.92	0.24	0.29	0.41
OsR498G0714743600.01	1.033	41.163	5.311453	0	0	1.29	0.93	0.88	39.76	40.76	42.97
OsR498G0714745500.01	0.263	1.326	2.287688	1.41E-06	2.84E-06	0.2	0.11	0.48	1.24	1.8	0.94
OsR498G0714746100.01	7.41	23.766	1.672649	2.47E-98	1.54E-98	7.44	7.26	7.53	25.39	23.81	22.1
OsR498G0714748200.01	6.656	16.883	1.332505	4.69E-81	3.25E-81	6.39	6.63	6.95	16.36	15.63	18.66
OsR498G0714749100.01	3.913	14.226	1.862134	1.29E-201	5.24E-202	3.98	3.82	3.94	14.06	15.01	13.61
OsR498G0714751200.01	17.52	124.67	2.833882	0	0	17.55	17.36	17.65	131.89	121.33	120.79
OsR498G0714751700.01	0.306	0.133	-1.21963	9.23E-06	1.94E-05	0.44	0.24	0.24	0.17	0.06	0.17
OsR498G0714752500.01	112.21	356.31	1.662061	0	0	111.45	114.22	111	373.33	344.28	351.33
OsR498G0714753100.01	8.813	1.496	-2.64254	6.29E-110	3.67E-110	8.85	9.22	8.37	1.1	1.38	2.01
OsR498G0714755000.01	0.51	0.073	-2.74451	3.70E-11	6.23E-11	0.44	0.57	0.52	0.05	0.05	0.12
OsR498G0714756300.01	29.903	1.076	-4.80339	0	0	30.24	28.68	30.79	1.29	0.87	1.07
OsR498G0714761900.01	1.38	6.63	2.251264	8.08E-67	6.21E-67	1.71	0.95	1.48	7.55	6.32	6.02
OsR498G0714763000.01	0.956	0	-6.61244	2.49E-13	3.93E-13	1.14	0.78	0.95	0	0	0
OsR498G0714763500.01	0.216	0.03	-3.00463	3.06E-07	5.98E-07	0.2	0.25	0.2	0	0.07	0.02
OsR498G0714764100.01	0.3	1.19	2.122222	4.36E-10	7.62E-10	0.35	0.31	0.24	1.77	1.25	0.55
OsR498G0714765100.01	12.29	24.94	1.041792	4.32E-144	2.16E-144	11.86	12.76	12.25	23.94	25.48	25.4
OsR498G0714766900.01	0.966	20.103	4.377828	0	0	0.76	1.06	1.08	21.75	18.49	20.07
OsR498G0714768100.01	2.74	0.64	-2.09386	8.93E-75	6.46E-75	2.46	3.1	2.66	0.58	0.55	0.79
OsR498G0714768600.01	10.596	3.546	-1.60781	3.76E-61	3.03E-61	11.9	9.47	10.42	3.57	3.77	3.3
OsR498G0714768800.01	14.34	0.98	-3.86937	0	0	14.3	14.22	14.5	0.91	1.12	0.91
OsR498G0714772900.01	0.353	1.526	2.105849	1.18E-24	1.46E-24	0.15	0.42	0.49	1.38	1.53	1.67
OsR498G0714773800.01	3.06	21.543	2.808723	4.61E-148	2.26E-148	3.02	3.31	2.85	21.48	20.13	23.02
OsR498G0714774900.01	32.176	78.846	1.281607	0	0	31.75	32.2	32.58	79.16	77.71	79.67
OsR498G0714775600.01	6.783	64.03	3.22659	0	0	6.45	6.78	7.12	63.31	63.87	64.91
OsR498G0714777700.01	0.013	0.433	5.209685	2.30E-10	3.98E-10	0	0.04	0	0.57	0.44	0.29
OsR498G0714778600.01	13.013	48.336	1.887252	1.03E-169	4.65E-170	12.77	14.72	11.55	53	45.34	46.67
OsR498G0714779700.01	24.08	6.066	-2.00272	1.53E-108	9.03E-109	27.11	23.74	21.39	5.89	6.14	6.17
OsR498G0714783700.01	0.026	0.523	4.361688	9.48E-11	1.62E-10	0.04	0.04	0	0.42	0.28	0.87
OsR498G0714785000.01	27.726	92.6	1.732483	0	0	28.53	28.98	25.67	91.53	92.46	93.81
OsR498G0714788600.01	3.5	1.133	-1.6435	4.94E-15	7.46E-15	4.08	2.9	3.52	0.9	1.65	0.85
OsR498G0714789300.01	6.103	1.55	-1.98028	8.91E-120	4.97E-120	6.27	6.01	6.03	1.59	1.62	1.44
OsR498G0714791800.01	0.493	0.016	-4.96024	4.08E-09	7.36E-09	0.46	0.76	0.26	0.05	0	0
OsR498G0714796600.01	38.886	16.893	-1.22095	9.51E-183	4.14E-183	37.92	38.51	40.23	17.13	17.17	16.38
OsR498G0714798400.01	0.17	0.59	1.789782	3.74E-05	8.14E-05	0.14	0.15	0.22	0.61	0.39	0.77
OsR498G0714808300.01	1.6	25.856	3.99714	1.01E-241	3.63E-242	1.65	1.17	1.98	27.55	27.57	22.45
OsR498G0714808700.01	35.18	8.706	-2.0393	2.14E-241	7.72E-242	36.76	33.95	34.83	8.97	8.46	8.69
OsR498G0714811700.01	1.656	8.036	2.05984	1.05E-74	7.57E-75	2.13	1.18	1.66	8.04	8.27	7.8
OsR498G0714814400.01	0.176	0.046	-2.83471	3.45E-05	7.49E-05	0.19	0.15	0.19	0	0.03	0.11
OsR498G0714814500.01	17.316	5.98	-1.53745	3.36E-68	2.57E-68	18.59	17.28	16.08	6.28	5.84	5.82
OsR498G0714816600.01	6.796	2.72	-1.30873	5.00E-28	5.82E-28	5.86	7.43	7.1	2.76	2.81	2.59
OsR498G0714817700.01	1.133	0.053	-5.03706	1.07E-32	1.16E-32	1.03	1.08	1.29	0.09	0.07	0
OsR498G0714820100.01	0.726	13.98	4.262505	7.17E-301	2.19E-301	0.68	0.75	0.75	14.67	13.61	13.66
OsR498G0714820500.01	15.3	86.213	2.48955	0	0	16.76	14.4	14.74	85.56	84.85	88.23
OsR498G0714821100.01	0.043	26.563	9.347644	0	0	0.1	0.03	0	27.39	26.75	25.55
OsR498G0714822200.01	2.57	1.386	-1.06047	7.78E-14	1.21E-13	2.08	3.07	2.56	1.23	1.12	1.81
OsR498G0714826400.01	0.186	0.403	1.109994	0.0001192	0.0002677	0.18	0.19	0.19	0.31	0.45	0.45
OsR498G0714826500.01	4.71	18.08	1.928528	1.60E-184	6.92E-185	4.78	4.88	4.47	17.96	18	18.28
OsR498G0714827200.01	3.106	0.44	-2.82613	1.42E-79	9.94E-80	3.06	2.55	3.71	0.45	0.35	0.52

OsR498G0714827600.01	439.57	953.82	1.075798	0	0	447.94	431.09	439.7	947.45	972.63	941.38
OsR498G0714828500.01	2.833	0.85	-1.73581	4.44E-19	6.13E-19	2.54	3.46	2.5	0.98	0.7	0.87
OsR498G0714830500.01	0.16	0.4	1.315394	0.0001997	0.0004555	0.18	0.13	0.17	0.46	0.5	0.24
OsR498G0714836300.01	1.11	0.533	-1.03706	4.85E-07	9.55E-07	1.14	1.34	0.85	0.49	0.58	0.53
OsR498G0714837500.01	1.703	3.926	1.233036	3.01E-36	3.13E-36	1.55	1.7	1.86	3.69	3.78	4.31
OsR498G0714838600.01	0.88	0.36	-1.36905	1.96E-06	3.97E-06	0.88	0.68	1.08	0.31	0.52	0.25
OsR498G0714840200.01	6.67	2.776	-1.26663	2.01E-31	2.23E-31	6.18	7.04	6.79	2.69	2.43	3.21
OsR498G0714844600.01	1.73	0.6	-1.53178	1.86E-09	3.32E-09	1.84	1.62	1.73	0.49	0.71	0.6
OsR498G0714845800.01	0	1.503	8.423464	9.07E-36	9.50E-36	0	0	0	1.48	1.46	1.57
OsR498G0714846900.01	0.723	0.266	-1.42973	0.0004759	0.0011163	0.65	1	0.52	0.24	0.25	0.31
OsR498G0714848100.01	5.586	28.27	2.339015	0	0	5.56	6.15	5.05	27.89	27.75	29.17
OsR498G0714848300.01	0.463	2.89	2.721584	3.50E-37	3.59E-37	0.56	0.56	0.27	3.23	2.72	2.72
OsR498G0714851500.01	38.193	12.56	-1.63566	2.76E-294	8.62E-295	40.43	37.5	36.65	12.3	13.39	11.99
OsR498G0714853900.01	55.756	147.01	1.398061	0	0	52.88	58.76	55.63	149.34	145.47	146.23
OsR498G0714854200.01	0.95	0.226	-2.08362	1.82E-08	3.37E-08	1.13	0.95	0.77	0.19	0.25	0.24
OsR498G0714855100.01	0.47	1.05	1.134225	6.05E-15	9.16E-15	0.38	0.54	0.49	1.23	1.07	0.85
OsR498G0714858600.01	62.766	20.886	-1.58902	0	0	63.6	66.66	58.04	21.36	20.91	20.39
OsR498G0714860400.01	0.193	0	-5.59309	1.39E-07	2.68E-07	0.12	0	0.46	0	0	0
OsR498G0714862300.01	12.98	4.18	-1.66978	4.08E-101	2.51E-101	13.55	12.56	12.83	4.41	3.95	4.18
OsR498G0714864200.01	1.97	0.606	-1.69458	6.11E-17	8.83E-17	2.28	2.05	1.58	0.55	0.84	0.43
OsR498G0714865400.01	5.463	2.6	-1.13016	1.08E-14	1.64E-14	4.95	6.65	4.79	2.15	2.82	2.83
OsR498G0714866700.01	0.413	0.84	1.015098	2.95E-06	6.03E-06	0.38	0.33	0.53	0.85	0.91	0.76
OsR498G0714874500.01	3.36	1.65	-1.04203	9.14E-07	1.82E-06	2.29	3.74	4.05	1.05	2.08	1.82
OsR498G0714874800.01	7.363	3.176	-1.16815	2.28E-48	2.08E-48	7.51	7.21	7.37	3.15	3.23	3.15
OsR498G0714875900.01	0.803	6.823	3.22874	4.46E-194	1.86E-194	0.64	0.95	0.82	7.04	7	6.43
OsR498G0714876300.01	3.48	35.65	3.3533	0	0	3.51	3.35	3.58	35.93	35.11	35.91
OsR498G0714876900.01	0.796	0.363	-1.13793	0.0005908	0.0013971	0.49	1.16	0.74	0.15	0.58	0.36
OsR498G0714879900.01	8.533	27.963	1.707147	2.04E-192	8.55E-193	9.42	7.62	8.56	28.37	27.46	28.06
OsR498G0714880900.01	3.516	0.53	-2.74493	1.05E-58	8.70E-59	3.45	3.56	3.54	0.45	0.5	0.64
OsR498G0714881900.01	0.303	0.97	1.652737	4.84E-06	1.00E-05	0.32	0.28	0.31	0.88	0.85	1.18
OsR498G0714882500.01	31.276	15.333	-1.23057	4.72E-129	2.52E-129	32.01	30.4	31.42	16.16	15.26	14.58
OsR498G0714882800.01	7.36	3.616	-1.02539	3.89E-31	4.32E-31	7.62	6.57	7.89	4.54	3.28	3.03
OsR498G0714886900.01	0	0.42	6.18666	2.84E-10	4.93E-10	0	0	0	0.83	0.43	0
OsR498G0714888000.01	620.15	228.33	-1.38675	0	0	591.65	635.36	633.5	218.06	231.08	235.86
OsR498G0714888800.01	0.373	0.013	-4.96024	1.98E-16	2.89E-16	0.37	0.35	0.4	0.04	0	0
OsR498G0714890900.01	7.95	24.156	1.592677	4.66E-81	3.23E-81	7.9	7.59	8.36	26.35	22.74	23.38
OsR498G0714891600.01	0	0.423	5.835215	1.82E-08	3.37E-08	0	0	0	0.53	0.42	0.32
OsR498G0714893000.01	15.153	37.2	1.29003	1.08E-114	6.19E-115	14.08	15.96	15.42	41.05	35.35	35.2
OsR498G0714901400.01	0.236	0.64	1.446935	6.81E-06	1.42E-05	0.33	0.26	0.12	0.62	0.51	0.79
OsR498G0714901600.01	27.893	12.933	-1.14053	9.76E-125	5.30E-125	28.42	26.83	28.43	11.08	14.05	13.67
OsR498G0714902300.01	131.34	35.38	-1.88585	0	0	129.59	131.94	132.5	34.25	34.28	37.61
OsR498G0714904000.01	0.963	0.046	-4.3865	1.54E-20	2.05E-20	1.12	0.78	0.99	0.07	0.04	0.03
OsR498G0714905900.01	5.436	1.196	-2.19005	1.61E-54	1.38E-54	5.77	4.8	5.74	1.46	0.98	1.15
OsR498G0714906900.01	1.03	0.1	-3.37528	1.54E-13	2.41E-13	0.86	1.26	0.97	0.05	0.15	0.1
OsR498G0714910700.01	0.01	0.863	6.03976	3.89E-17	5.60E-17	0	0	0.03	1.24	0.79	0.56
OsR498G0714911700.01	1.463	0.62	-1.24782	4.02E-06	8.28E-06	1.63	1.49	1.27	0.7	0.61	0.55
OsR498G0714913200.01	3.32	0.806	-2.13956	1.64E-10	2.83E-10	3.31	3.09	3.56	1.06	0.96	0.4
OsR498G0714913300.01	6.626	3.063	-1.11284	3.05E-25	3.72E-25	6.88	6.38	6.62	2.74	2.5	3.95
OsR498G0714919400.01	1.396	0.246	-2.49629	1.43E-17	2.03E-17	1.44	1.32	1.43	0.29	0.3	0.15
OsR498G0714924900.01	3.746	1.786	-1.07021	1.19E-19	1.61E-19	3.67	3.9	3.67	1.93	1.67	1.76
OsR498G0714925200.01	7.276	22.68	1.633076	5.22E-210	2.07E-210	7.29	6.79	7.75	21.97	22.73	23.34
OsR498G0714925800.01	56.48	26.02	-1.1239	8.70E-278	2.83E-278	57.4	55.15	56.89	25.56	26.13	26.37
OsR498G0714928600.01	20.4	54.613	1.424909	0	0	19.73	21.14	20.33	55.75	54.55	53.54
OsR498G0714930700.01	1.13	0.113	-3.32281	7.84E-15	1.19E-14	0.98	1.51	0.9	0.05	0.1	0.19
OsR498G0714931500.01	3.856	0.256	-3.5452	5.15E-36	5.38E-36	3.73	3.87	3.97	0.25	0.4	0.12
OsR498G0714931700.01	110.74	6.876	-3.85988	0	0	111.34	108.38	112.5	5.94	8.35	6.34
OsR498G0714934800.01	0.543	0.093	-2.66919	2.98E-14	4.59E-14	0.37	0.48	0.78	0.13	0.05	0.1
OsR498G0714939200.01	1.476	0.646	-1.6041	2.27E-11	3.80E-11	1.44	1.45	1.54	0.57	0.47	0.9
OsR498G0714939500.01	1.356	0.506	-1.435	6.68E-06	1.39E-05	0.88	1.59	1.6	0.57	0.47	0.48
OsR498G0714939800.01	1121.7	286.92	-1.97961	0	0	1131.3	1088.9	1145	310.85	278.41	271.51
OsR498G0714940500.01	2.686	99.81	5.191993	0	0	3.7	2.84	1.52	105.76	96.07	97.6
OsR498G0714941700.01	11.923	2.703	-2.15255	1.83E-141	9.28E-142	12.46	11.85	11.46	2.75	2.71	2.65
OsR498G0714943300.01	0.296	0	-4.28217	0.0007127	0.0016975	0.36	0.19	0.34	0	0	0
OsR498G0714944900.01	17.483	6.693	-1.30111	2.99E-157	1.41E-157	16.16	18.79	17.5	5.91	6.88	7.29
OsR498G0714947000.01	0.22	1.47	2.697641	1.68E-05	3.57E-05	0.17	0.18	0.31	1.97	1.36	1.08
OsR498G0714951100.01	3.566	0.563	-2.6756	2.34E-37	2.40E-37	3.95	3.36	3.39	0.45	0.78	0.46
OsR498G0714952500.01	0.12	1.403	3.604545	6.46E-30	7.30E-30	0.09	0.21	0.06	1.28	1.65	1.28
OsR498G0714955800.01	120	638.73	2.4054	0	0	117.81	123.05	119.1	621.32	649.02	645.86
OsR498G0714959300.01	1.106	32.643	4.83032	0	0	0.98	0.88	1.46	30.79	33.81	33.33

OsR498G0714959600.01	0.266	0.546	1.054115	6.38E-06	1.33E-05	0.27	0.36	0.17	0.49	0.58	0.57
OsR498G0714961800.01	2.873	7.236	1.324969	1.43E-56	1.21E-56	2.79	2.72	3.11	7.46	7.26	6.99
OsR498G0714963400.01	1.61	21.183	3.761095	0	0	1.38	2.21	1.24	23.96	19.72	19.87
OsR498G0714963900.01	9.21	25.426	1.321867	1.34E-209	5.31E-210	9.94	9.44	8.25	24.4	27.05	24.83
OsR498G0714964600.01	4.306	8.74	1.010165	5.43E-46	5.07E-46	4.33	4.25	4.34	8.57	9.25	8.4
OsR498G0714966300.01	44.763	7.446	-2.59896	0	0	49.01	42.19	43.09	7.65	6.67	8.02
OsR498G0714968400.01	19.453	2.933	-2.74135	0	0	19.36	19.44	19.56	2.99	2.63	3.18
OsR498G0714973300.01	4.08	19.46	2.254442	8.07E-270	2.68E-270	4.18	3.89	4.17	18.61	20.76	19.01
OsR498G0714974500.01	0.28	222.43	9.655473	0	0	0.37	0.19	0.28	232.98	216.5	217.82
OsR498G0714974900.01	3.576	1.506	-1.27076	9.09E-19	1.26E-18	3.37	4.03	3.33	1.6	1.32	1.6
OsR498G0714976300.01	556.23	104.42	-2.41939	0	0	556.49	554.82	557.4	106.36	103.93	102.96
OsR498G0714976700.01	1550.7	658.57	-1.24364	0	0	1612.4	1527.4	1512	693.1	640.66	641.94
OsR498G0714977900.01	1.826	0.426	-2.45751	1.29E-50	1.15E-50	1.61	2.07	1.8	0.33	0.51	0.44
OsR498G0714978600.01	10.883	153.37	3.801654	0	0	11.1	9.94	11.61	161.43	147.08	151.59
OsR498G0714982400.01	20.29	4.606	-2.15176	2.73E-188	1.16E-188	20.31	20.76	19.8	3.6	4.52	5.7
OsR498G0714983100.01	2.393	23.203	3.272421	0	0	1.77	2.68	2.73	24.79	22.33	22.49
OsR498G0714984400.01	22.423	88.33	1.982423	0	0	19.67	24.24	23.36	95.72	84.16	85.11
OsR498G0714987100.01	3.476	0.223	-4.07572	3.02E-45	2.84E-45	3.71	3.19	3.53	0.04	0.17	0.46
OsR498G0714989100.01	4.576	0.133	-5.0807	4.80E-147	2.37E-147	4.54	4.51	4.68	0.16	0.11	0.13
OsR498G0714989400.01	5.006	0.196	-4.67782	1.24E-29	1.41E-29	5.32	4.31	5.39	0.33	0.01	0.25
OsR498G0714990300.01	0.293	1.503	2.361688	5.98E-13	9.53E-13	0.15	0.48	0.25	1.51	1.67	1.33
OsR498G0714991100.01	0.463	16.636	5.164351	0	0	0.47	0.56	0.36	18.71	16.27	14.93
OsR498G0714991900.01	0.41	1.333	1.685095	4.21E-12	6.90E-12	0.47	0.49	0.27	1.35	1.53	1.12
OsR498G0714992600.01	0.033	0.856	4.712185	1.36E-13	2.13E-13	0.05	0.05	0	0.74	0.97	0.86
OsR498G0714992800.01	0.56	10.156	4.163881	2.20E-62	1.76E-62	0.31	0.66	0.71	9.7	9.81	10.96
OsR498G0714994000.01	1.06	10.61	3.321458	1.62E-145	8.05E-146	0.83	1.33	1.02	11.01	11.21	9.61
OsR498G0714995400.01	0.36	1.33	1.886643	2.73E-27	3.21E-27	0.4	0.34	0.34	1.44	1.15	1.4
OsR498G0714995900.01	0.17	0.553	1.731638	1.91E-05	4.08E-05	0.08	0.28	0.15	0.54	0.76	0.36
OsR498G0714999700.01	15.01	5.46	-1.59401	4.42E-219	1.70E-219	14.58	15.41	15.04	5.47	5	5.91
OsR498G0715003600.01	5.336	2.333	-1.20399	4.48E-30	5.05E-30	5.96	4.8	5.25	1.64	2.86	2.5
OsR498G0715003800.01	7.986	3.9	-1.00231	1.47E-70	1.10E-70	7.22	8.65	8.09	3.79	3.68	4.23
OsR498G0715004000.01	11.103	27.403	1.290926	1.13E-240	4.08E-241	11.34	11.16	10.81	27.31	27.12	27.78
OsR498G0715005400.01	0.263	1.023	1.970497	1.48E-14	2.27E-14	0.15	0.38	0.26	1.01	0.95	1.11
OsR498G0715007500.01	185.92	14.683	-3.66362	0	0	178.81	187.98	191	14.31	15.46	14.28
OsR498G0715007900.01	11.706	38.893	1.720841	1.89E-134	9.83E-135	12.64	10.83	11.65	42.06	37.86	36.76
OsR498G0715009300.01	2.493	0.183	-3.72577	4.58E-82	3.14E-82	2.51	2.85	2.12	0.14	0.19	0.22
OsR498G0715011100.01	2.213	0.56	-1.99977	1.79E-09	3.19E-09	2.32	2.16	2.16	0.46	0.66	0.56
OsR498G0715013300.01	13.973	38.04	1.528437	0	0	14.36	13.37	14.19	33.89	39.73	40.5
OsR498G0715013600.01	2.323	18.236	2.964134	0	0	2.16	2.67	2.14	18.38	18.52	17.81
OsR498G0715015100.01	3.146	22.973	2.862414	1.41E-306	4.24E-307	3.44	2.98	3.02	22.9	21.61	24.41
OsR498G0715018400.01	5.23	1.813	-1.53249	4.68E-34	5.00E-34	5.34	5.32	5.03	1.53	2.07	1.84
OsR498G0715018600.01	838.76	344.85	-1.30953	0	0	848.39	839.47	828.4	355.31	339.67	339.56
OsR498G0715019000.01	22.143	7.393	-1.59271	1.26E-134	6.56E-135	23.72	21.16	21.55	8.05	6.98	7.15
OsR498G0715020100.01	149.36	29.31	-2.32744	0	0	142.48	154.55	151.1	27.77	30.39	29.77
OsR498G0715020600.01	2.803	16.98	2.606922	0	0	2.6	2.77	3.04	15.94	17.3	17.7
OsR498G0815024100.01	4.48	2.176	-1.12427	1.53E-17	2.18E-17	5.49	4.27	3.68	1.42	2.82	2.29
OsR498G0815026500.01	3	10.35	1.78684	2.13E-123	1.16E-123	2.86	3.16	2.98	11.14	10.29	9.62
OsR498G0815026900.01	15.033	4.706	-1.68336	2.09E-55	1.78E-55	15.85	14.93	14.32	5.17	4.6	4.35
OsR498G0815028800.01	2.276	0.17	-3.76759	3.41E-35	3.60E-35	2.15	2.21	2.47	0.21	0.13	0.17
OsR498G0815032100.01	0.223	0	-4.27783	0.0007259	0.0017298	0.43	0.24	0	0	0	0
OsR498G0815035200.01	6156.7	1402.1	-2.1451	0	0	6381.8	6019.1	6069	1506.5	1363.9	1335.8
OsR498G0815035700.01	1.843	12.356	2.740978	0	0	1.84	1.84	1.85	11.88	12.28	12.91
OsR498G0815037400.01	10.446	41.443	1.9818	0	0	10.82	9.91	10.61	42.44	40.96	40.93
OsR498G0815038000.01	1.226	6.42	2.384589	3.09E-102	1.89E-102	1.05	1.55	1.08	6.29	7.01	5.96
OsR498G0815040500.01	5.426	1.216	-2.16126	2.99E-55	2.55E-55	5.89	4.69	5.7	1.55	1.2	0.9
OsR498G0815040800.01	0	0.26	5.03976	1.53E-05	3.26E-05	0	0	0	0.14	0.2	0.44
OsR498G0815041800.01	19.436	67.85	1.799182	0	0	21.17	19.05	18.09	73.02	66.04	64.49
OsR498G0815042400.01	20.02	49.913	1.312826	1.02E-295	3.17E-296	20.06	19.11	20.89	47.04	50.94	51.76
OsR498G0815045300.01	3.46	12.966	1.864649	1.82E-165	8.36E-166	3.17	3.7	3.51	12.6	12.8	13.5
OsR498G0815051500.01	0.093	0.663	2.847115	8.02E-20	1.09E-19	0.09	0.07	0.12	0.66	0.67	0.66
OsR498G0815055200.01	6.076	23.733	1.838877	1.39E-183	6.01E-184	6.05	5.81	6.37	22.86	23.92	24.42
OsR498G0815055900.01	11.286	37.916	1.745038	0	0	11.41	11.25	11.2	35.96	38.01	39.78
OsR498G0815056100.01	0.366	1.2	1.695112	3.79E-07	7.41E-07	0.5	0.12	0.48	0.84	1.33	1.43
OsR498G0815056400.01	0.15	5.73	5.249213	3.64E-28	4.22E-28	0.15	0.16	0.14	5.68	5.76	5.75
OsR498G0815056600.01	8.63	21.873	1.344952	2.69E-228	1.01E-228	8.55	8.62	8.72	21.6	21.36	22.66
OsR498G0815057200.01	2.876	1.24	-1.2124	1.64E-29	1.86E-29	2.34	2.85	3.44	1.32	1.1	1.3
OsR498G0815058200.01	0.123	2.103	4.076849	4.14E-44	3.95E-44	0.06	0.19	0.12	2.07	2.54	1.7
OsR498G0815060400.01	0.096	8.816	6.464136	1.52E-163	7.02E-164	0.08	0.08	0.13	8.52	8.47	9.46
OsR498G0815061000.01	8.82	25.066	1.498838	2.78E-290	8.75E-291	8.67	8.88	8.91	24.5	26.7	24

OsR498G0815061700.01	8.353	2.52	-1.73586	7.14E-46	6.69E-46	8.13	8.61	8.32	2.6	2.34	2.62
OsR498G0815062700.01	0.653	6.133	3.22595	3.32E-82	2.28E-82	0.52	0.67	0.77	6.28	5.74	6.38
OsR498G0815062900.01	16.746	5.626	-1.57109	4.34E-93	2.78E-93	18.07	16.16	16.01	6.31	4.95	5.62
OsR498G0815064300.01	32.903	9.063	-1.80863	0	0	33.7	34.39	30.62	9.89	9.35	7.95
OsR498G0815065200.01	0.793	0.2	-2.28217	0.0003536	0.0008216	0.67	0.87	0.84	0	0.45	0.15
OsR498G0815068800.01	0.006	0.656	6.639673	1.40E-24	1.73E-24	0	0	0.02	0.74	0.56	0.67
OsR498G0815073900.01	1.143	0.033	-5.78042	3.87E-28	4.49E-28	0.76	1.28	1.39	0	0.04	0.06
OsR498G0815078400.01	0.066	0.693	3.361688	2.58E-09	4.62E-09	0	0.05	0.15	0.72	0.42	0.94
OsR498G0815079100.01	15.35	7.46	-1.04984	1.33E-38	1.35E-38	14.67	14.92	16.46	7.35	6.91	8.12
OsR498G0815079800.01	15.913	32.76	1.033608	2.31E-94	1.47E-94	18.54	14.1	15.1	37.81	30.87	29.6
OsR498G0815080800.01	18.596	42.803	1.213918	1.08E-154	5.15E-155	19.35	18.26	18.18	43.2	42.55	42.66
OsR498G0815081500.01	1.88	4.62	1.382648	5.53E-28	6.44E-28	1.91	1.72	2.01	4.59	4.74	4.53
OsR498G0815084900.01	63.156	18.606	-1.7594	0	0	65.07	59.42	64.98	17.39	20.53	17.9
OsR498G0815087200.01	201.09	18.986	-3.33673	0	0	190.18	196.7	216.4	20.55	17.93	18.48
OsR498G0815087400.01	50.87	156.27	1.595265	0	0	50.19	51.36	51.06	167.08	150.81	150.92
OsR498G0815088900.01	0.073	0.006	-3.41967	0.0009442	0.002269	0.06	0.12	0.04	0.02	0	0
OsR498G0815089200.01	5.473	2.316	-1.24106	9.42E-39	9.53E-39	5.19	6.36	4.87	2.28	2.23	2.44
OsR498G0815089600.01	18.66	5.406	-1.82895	2.31E-268	7.70E-269	19.03	19.22	17.73	5.48	5.94	4.8
OsR498G0815090000.01	1.553	3.693	1.199631	2.84E-16	4.17E-16	1.16	1.68	1.82	3.93	3.74	3.41
OsR498G0815097400.01	0.21	0.713	1.782985	7.85E-09	1.43E-08	0.16	0.31	0.16	0.96	0.54	0.64
OsR498G0815100800.01	0.046	0.346	2.935709	3.24E-09	5.82E-09	0.02	0.05	0.07	0.41	0.26	0.37
OsR498G0815102700.01	697.84	310.16	-1.17339	0	0	689.05	691.76	712.7	308.19	304.19	318.11
OsR498G0815103200.01	0.013	0.166	3.624723	0.0004041	0.0009439	0.04	0	0	0.16	0.26	0.08
OsR498G0815103300.01	12.343	1.26	-3.31074	0	0	11.67	12.22	13.14	1.28	1.14	1.36
OsR498G0815104300.01	44.616	897.32	4.325253	0	0	45.21	43.26	45.38	918.97	891.17	881.82
OsR498G0815105100.01	3.133	9.573	1.588197	5.92E-38	6.03E-38	3.7	2.5	3.2	10.16	9.43	9.13
OsR498G0815106700.01	0.216	0.033	-2.69721	0.0001045	0.0002337	0.2	0.32	0.13	0.03	0.07	0
OsR498G0815111500.01	26.633	115.34	2.100646	0	0	26.72	25.26	27.92	109.03	116.38	120.62
OsR498G0815115800.01	0.093	0.53	2.525187	9.81E-10	1.73E-09	0.03	0.17	0.08	0.53	0.43	0.63
OsR498G0815117900.01	0.166	0.636	1.624723	3.24E-10	5.63E-10	0.17	0.2	0.13	0.86	0.4	0.65
OsR498G0815119500.01	2.03	0.67	-1.6041	1.58E-07	3.03E-07	1.88	3.01	1.2	0.71	0.76	0.54
OsR498G0815126300.01	0.2	0.043	-2.13016	0.0009788	0.0023546	0.2	0.21	0.19	0.1	0	0.03
OsR498G0815133600.01	0.936	0.383	-1.30477	1.21E-12	1.95E-12	0.88	1.05	0.88	0.43	0.43	0.29
OsR498G0815137300.01	1	25.68	4.667421	6.99E-245	2.49E-245	0.91	0.88	1.21	28.49	23.8	24.75
OsR498G0815137500.01	0.133	2.603	4.287688	5.13E-27	6.07E-27	0.2	0.07	0.13	2.02	2.91	2.88
OsR498G0815139100.01	1.08	0.556	-1.02633	1.71E-09	3.05E-09	1.07	1	1.17	0.46	0.52	0.69
OsR498G0815139400.01	0.346	0.833	1.241394	1.90E-05	4.06E-05	0.34	0.37	0.33	0.82	0.62	1.06
OsR498G0815140200.01	32.003	13.08	-1.29555	1.46E-79	1.02E-79	36.59	30.31	29.11	14.81	12.26	12.17
OsR498G0815140400.01	70.773	10.763	-2.69942	0	0	64.3	72.98	75.04	10.38	11.06	10.85
OsR498G0815140600.01	18.406	6.11	-1.57083	1.84E-127	9.87E-128	19.59	18.66	16.97	6.73	5.52	6.08
OsR498G0815142800.01	0.696	0	-7.13016	7.93E-18	1.12E-17	0.49	0.76	0.84	0	0	0
OsR498G0815143900.01	0.406	0	-5.5452	2.18E-07	4.22E-07	0.51	0.27	0.44	0	0	0
OsR498G0815145300.01	0.01	1.466	7.219669	1.10E-34	1.16E-34	0.03	0	0	1.46	1.48	1.46
OsR498G0815146000.01	0.626	2.666	2.08477	1.20E-34	1.28E-34	0.66	0.53	0.69	3	2.67	2.33
OsR498G0815148100.01	66.69	151.98	1.206619	0	0	70.72	65.99	63.36	149.74	158.14	148.06
OsR498G0815148400.01	1.9	26.163	3.775664	0	0	1.96	2.05	1.69	25.25	26.64	26.6
OsR498G0815152100.01	7.473	2.99	-1.26354	1.28E-36	1.33E-36	7.19	7.62	7.61	3.26	2.94	2.77
OsR498G0815167000.01	26.806	5.696	-1.93363	3.94E-134	2.05E-134	25.97	26.92	27.53	6.51	5.1	5.48
OsR498G0815171800.01	1.076	0.013	-6.35256	3.22E-21	4.24E-21	1.03	1.2	1	0	0.04	0
OsR498G0815188200.01	24.616	9.546	-1.37652	2.50E-166	1.14E-166	25.18	24.16	24.51	9.98	9.15	9.51
OsR498G0815197100.01	4.483	50.823	3.438898	0	0	4.07	4.62	4.76	53.91	50.01	48.55
OsR498G0815200700.01	55.056	18.123	-1.63064	0	0	55.33	54.43	55.41	17.81	18.89	17.67
OsR498G0815203800.01	10.12	22.016	1.067067	7.79E-82	5.36E-82	10.42	9.62	10.32	20.12	23.36	22.57
OsR498G0815206200.01	0.86	0.23	-2.01913	4.91E-07	9.67E-07	0.74	0.98	0.86	0.18	0.25	0.26
OsR498G0815209400.01	2.95	0.663	-2.15754	2.80E-62	2.24E-62	2.82	2.85	3.18	0.55	0.68	0.76
OsR498G0815210200.01	7.673	2.91	-1.39816	2.93E-45	2.76E-45	7.78	8.56	6.68	3.07	2.66	3
OsR498G0815224500.01	21.01	142.23	2.756436	0	0	21.6	21.16	20.27	144.2	140.64	141.84
OsR498G0815224800.01	24.426	51.033	1.055057	7.21E-126	3.90E-126	26.72	23.53	23.03	50.86	51.68	50.56
OsR498G0815225900.01	7.833	3.5	-1.1695	2.08E-35	2.19E-35	7.84	7.55	8.11	3.3	3.5	3.7
OsR498G0815226800.01	5.363	1.433	-1.90595	1.13E-34	1.19E-34	5.29	5.6	5.2	1.44	1.24	1.62
OsR498G0815231800.01	0.33	1.2	1.398857	0.000304	0.0007025	0.49	0.05	0.45	0.96	1.55	1.09
OsR498G0815233600.01	27.36	77.466	1.498013	0	0	26.6	27.65	27.83	76.24	77.21	78.95
OsR498G0815235000.01	19.8	44.703	1.171648	5.45E-188	2.32E-188	18.97	20.73	19.7	46.16	44.22	43.73
OsR498G0815239000.01	274.08	8.616	-4.99416	0	0	283.72	268.54	270	8.35	8.33	9.17
OsR498G0815243400.01	0.763	0.156	-2.33108	1.28E-20	1.71E-20	0.69	0.91	0.69	0.16	0.05	0.26
OsR498G0815247400.01	5.953	1.406	-2.09372	6.74E-49	6.11E-49	5.3	6.61	5.95	1	1.62	1.6
OsR498G0815251300.01	43.92	90.596	1.04345	0	0	42.06	45.08	44.62	90.3	90.59	90.9
OsR498G0815253500.01	713.34	268.28	-1.41649	0	0	712.15	712.37	715.5	274.31	264.6	265.94
OsR498G0815254000.01	406.31	17.1	-4.57304	0	0	414.26	398.98	405.7	16.55	17.02	17.73

OsR498G0815255400.01	0.566	0.033	-4.13016	1.44E-05	3.05E-05	0.47	0.5	0.73	0.1	0	0
OsR498G0815255900.01	174.96	24.25	-2.83459	0	0	176.58	177.47	170.8	22.92	27.16	22.67
OsR498G0815257100.01	14.006	6.973	-1.01066	6.13E-57	5.15E-57	12.06	14.89	15.07	7.57	6.19	7.16
OsR498G0815261700.01	17.15	3.07	-2.49467	7.63E-74	5.56E-74	17.65	17.58	16.22	2.96	3.54	2.71
OsR498G0815270100.01	53.94	26.593	-1.02991	1.41E-131	7.46E-132	55.61	51.88	54.33	26.42	26.64	26.72
OsR498G0815270900.01	0.16	0.03	-2.35256	0.0002107	0.0004813	0.23	0.07	0.18	0.02	0.02	0.05
OsR498G0815273900.01	0.04	1.18	5.209685	2.30E-10	3.98E-10	0	0.12	0	1.4	1.3	0.84
OsR498G0815275500.01	3.683	0.03	-6.80573	8.89E-54	7.68E-54	3.82	3.6	3.63	0.05	0.04	0
OsR498G0815285700.01	12.856	5.86	-1.1281	2.51E-48	2.29E-48	13.53	12.58	12.46	5.94	6.04	5.6
OsR498G0815291700.01	70.126	13.473	-2.46606	0	0	72.2	69.67	68.51	12.83	13.56	14.03
OsR498G0815292800.01	12.093	6.103	-1.0027	8.11E-15	1.23E-14	12.84	11.72	11.72	5.37	6.16	6.78
OsR498G0815295300.01	1.03	20.25	4.273491	1.26E-179	5.53E-180	0.65	0.76	1.68	22.5	17.28	20.97
OsR498G0815297600.01	3.403	20.706	2.600244	1.79E-273	5.90E-274	3.69	3.26	3.26	21.38	20.66	20.08
OsR498G0815297700.01	0.986	7.066	2.837026	1.01E-92	6.46E-93	0.93	1.02	1.01	6.49	7.36	7.35
OsR498G0815301600.01	38.416	132.56	1.784473	0	0	38.21	39.61	37.43	134.49	128.65	134.55
OsR498G0815302500.01	33.796	16.243	-1.07683	2.64E-108	1.56E-108	35.19	33.36	32.84	16.57	15.84	16.32
OsR498G0815304200.01	2.053	20.966	3.354356	0	0	1.75	2.45	1.96	21.81	21.17	19.92
OsR498G0815306300.01	0.16	1.293	3.03285	1.25E-19	1.70E-19	0.18	0.23	0.07	1.18	1.55	1.15
OsR498G0815310000.01	17.9	3.83	-2.18147	4.51E-145	2.25E-145	19.29	17.11	17.3	4.31	2.82	4.36
OsR498G0815310900.01	2.516	0.113	-4.43093	8.45E-142	4.27E-142	2.2	2.88	2.47	0.15	0.09	0.1
OsR498G0815318300.01	21.693	7.353	-1.56752	6.07E-62	4.87E-62	20.25	22.32	22.51	8.32	6.74	7
OsR498G0815319100.01	0	0.276	4.847115	5.08E-05	0.0001113	0	0	0	0.23	0.18	0.42
OsR498G0815320200.01	0.236	0.09	-1.87158	0.0001963	0.0004474	0.27	0.27	0.17	0.04	0.08	0.15
OsR498G0815325400.01	0.41	0	-5.5452	2.18E-07	4.22E-07	0.41	0.22	0.6	0	0	0
OsR498G0815328600.01	171.85	71.106	-1.28623	0	0	167.49	172.88	175.2	69.13	72.31	71.88
OsR498G0815329300.01	1.3	0.193	-2.76759	1.19E-15	1.78E-15	1.41	1.08	1.41	0.13	0.32	0.13
OsR498G0815332900.01	14.27	5.703	-1.35339	6.13E-155	2.93E-155	12.88	15.19	14.74	6.24	5.94	4.93
OsR498G0815333900.01	0.59	4.403	2.881038	2.11E-33	2.27E-33	1.26	0	0.51	9.07	4.14	0
OsR498G0815335100.01	5.25	1.293	-2.04513	9.67E-17	1.40E-16	5.4	4.38	5.97	1.51	1.19	1.18
OsR498G0815339300.01	0.186	2.256	3.636695	6.23E-26	7.51E-26	0.16	0.4	0	2.31	2.55	1.91
OsR498G0815340500.01	0.823	2.173	1.37961	7.06E-07	1.40E-06	0.93	0.57	0.97	1.82	2.6	2.1
OsR498G0815341300.01	0.333	1.143	1.76768	1.46E-06	2.93E-06	0.31	0.33	0.36	1.66	0.99	0.78
OsR498G0815341700.01	0.023	0.34	3.946651	6.37E-05	0.0001407	0	0	0.07	0.5	0.13	0.39
OsR498G0815344500.01	0.67	0.026	-4.6232	9.37E-19	1.30E-18	0.58	0.69	0.74	0.03	0	0.05
OsR498G0815345700.01	0.173	0.03	-2.69721	0.0001045	0.0002337	0.18	0.11	0.23	0.03	0.03	0.03
OsR498G0815345900.01	0.463	0.093	-2.33528	2.98E-12	4.85E-12	0.45	0.47	0.47	0.1	0.12	0.06
OsR498G0815347600.01	0.133	0.01	-3.5452	0.0005186	0.0012198	0.13	0.14	0.13	0	0	0.03
OsR498G0815347700.01	19.393	1.453	-3.74054	0	0	19.91	19.46	18.81	1.43	1.2	1.73
OsR498G0815352500.01	3.493	0.04	-6.5502	7.38E-68	5.65E-68	4.29	3.24	2.95	0	0.03	0.09
OsR498G0815353900.01	1.39	0.51	-1.45905	1.86E-10	3.20E-10	1.47	1.34	1.36	0.52	0.54	0.47
OsR498G0815361000.01	9.03	1.586	-2.51584	8.32E-75	6.01E-75	8.72	8.98	9.39	1.59	1.91	1.26
OsR498G0815365500.01	0.153	0.45	1.411729	0.0001314	0.000296	0.13	0.25	0.08	0.35	0.62	0.38
OsR498G0815366500.01	0.05	0.343	2.624723	5.29E-08	9.97E-08	0.02	0.02	0.11	0.29	0.25	0.49
OsR498G0815367900.01	0.156	0.426	1.397312	0.0002302	0.0005279	0.09	0.19	0.19	0.43	0.39	0.46
OsR498G0815372600.01	2.03	0.203	-3.3395	2.17E-71	1.61E-71	1.7	2.17	2.22	0.26	0.17	0.18
OsR498G0815373700.01	1.49	0.39	-1.96024	8.27E-06	1.73E-05	1.57	1.06	1.84	0.35	0.12	0.7
OsR498G0815379800.01	0.506	28.446	5.784594	0	0	0.47	0.49	0.56	31.18	27.52	26.64
OsR498G0815380800.01	11399	2727.8	-2.07061	0	0	11530	11219	11448	2867.7	2640.1	2675.7
OsR498G0815381500.01	3.6	0.833	-2.09508	1.86E-37	1.91E-37	3.67	3.72	3.41	0.65	0.91	0.94
OsR498G0815382000.01	41.956	5.916	-3.13143	0	0	43.54	38.87	43.46	6.13	6.32	5.3
OsR498G0815382600.01	0.2	1.713	3.079288	1.15E-14	1.75E-14	0.2	0.21	0.19	1.92	1.34	1.88
OsR498G0815385700.01	34.083	14.813	-1.15079	4.03E-262	1.37E-262	33.39	34.63	34.23	14.71	14.08	15.65
OsR498G0815386000.01	4.543	14.52	1.722727	7.11E-252	2.48E-252	4.86	4.04	4.73	14.06	15.1	14.4
OsR498G0815386500.01	0.273	2.336	3.216638	1.29E-76	9.17E-77	0.27	0.3	0.25	2.02	2.53	2.46
OsR498G0815386700.01	37.326	6.726	-2.47507	0	0	38.3	37.26	36.42	6.1	6.68	7.4
OsR498G0815387500.01	2.133	0.193	-3.48728	1.42E-75	1.02E-75	2.23	1.8	2.37	0.09	0.2	0.29
OsR498G0815393200.01	18.02	46.616	1.369246	3.57E-232	1.32E-232	19.12	18.71	16.23	49.96	46.12	43.77
OsR498G0815394100.01	0.666	29.723	5.455161	0	0	0.73	0.63	0.64	30.79	30.19	28.19
OsR498G0815397300.01	0.863	2.583	1.58749	3.08E-37	3.16E-37	0.78	0.98	0.83	2.43	2.56	2.76
OsR498G0815402500.01	2.74	1.156	-1.25622	6.25E-15	9.46E-15	2.8	2.18	3.24	1.24	1.11	1.12
OsR498G0815403000.01	0.426	0.083	-2.31779	6.73E-07	1.33E-06	0.31	0.52	0.45	0.09	0.03	0.13
OsR498G0815441000.01	16.253	6.376	-1.43153	2.93E-182	1.27E-182	14.75	17.14	16.87	7.1	5.87	6.16
OsR498G0815443700.01	20.5	8.523	-1.35672	1.76E-99	1.09E-99	20.94	21.35	19.21	9.32	8.14	8.11
OsR498G0815454900.01	22.536	6.243	-1.90229	1.76E-129	9.39E-130	23.26	22.37	21.98	6.16	6.26	6.31
OsR498G0815461300.01	1.046	0.45	-1.12478	1.24E-16	1.80E-16	1.01	1.09	1.04	0.36	0.49	0.5
OsR498G0815463200.01	5.986	1.443	-2.16003	1.23E-90	7.99E-91	6.05	6.1	5.81	1.35	1.62	1.36
OsR498G0815464100.01	0.49	0.226	-1.10708	0.0002019	0.0004606	0.54	0.54	0.39	0.22	0.12	0.34
OsR498G0815464500.01	19.883	40.683	1.042316	6.14E-296	1.91E-296	18.9	21.03	19.72	41.49	40.22	40.34
OsR498G0815470100.01	2.78	0.25	-3.60479	6.63E-26	8.00E-26	3.11	3.02	2.21	0.11	0.58	0.06

OsR498G0815489000.01	0.476	0.116	-2.07572	0.0001433	0.0003236	0.22	0.52	0.69	0.06	0.23	0.06
OsR498G0815493900.01	9.426	20.166	1.258119	5.92E-118	3.33E-118	8.57	9.22	10.49	21.53	19.71	19.26
OsR498G0815494700.01	14.163	5.473	-1.40796	2.48E-114	1.42E-114	14.53	14.21	13.75	5.34	5.27	5.81
OsR498G0815499500.01	2.626	1.026	-1.35879	3.17E-05	6.87E-05	2.82	2.98	2.08	2.09	0.14	0.85
OsR498G0815500900.01	0.923	0.413	-1.17323	0.0003755	0.000874	1.08	0.8	0.89	0.5	0.4	0.34
OsR498G0815501800.01	1.22	0.61	-1.01757	0.0001195	0.0002685	1.38	0.9	1.38	0.34	0.9	0.59
OsR498G0815504000.01	7.29	3.173	-1.21774	2.72E-12	4.43E-12	7.96	6.62	7.29	3.03	2.87	3.62
OsR498G0815506900.01	4.53	1.92	-1.24403	2.64E-19	3.62E-19	5.09	3.94	4.56	2.18	1.4	2.18
OsR498G0815509300.01	1.036	0.14	-2.83208	8.68E-10	1.53E-09	1.45	0.87	0.79	0.18	0	0.24
OsR498G0815509700.01	0.24	0	-4.5512	0.0002077	0.0004741	0.27	0.09	0.36	0	0	0
OsR498G0815511900.01	0.233	1.266	2.485062	1.25E-21	1.63E-21	0.27	0.28	0.15	1.17	1.23	1.4
OsR498G0815517200.01	1.386	0.343	-1.97183	3.47E-27	4.09E-27	1.11	1.41	1.64	0.34	0.34	0.35
OsR498G0815520600.01	797.03	165.93	-2.27521	0	0	819.48	769.5	802.1	164.53	168.36	164.91
OsR498G0815521100.01	80.143	13.98	-2.52712	0	0	78.38	80.07	81.98	13.19	13.81	14.94
OsR498G0815523900.01	100.37	214.16	1.101219	0	0	101.55	98.12	101.4	205.25	215.6	221.64
OsR498G0815524200.01	0.356	1.026	1.499192	0.0004464	0.0010454	0.18	0.28	0.61	0.64	1.04	1.4
OsR498G0815527100.01	0.9	0.233	-1.93223	6.51E-12	1.07E-11	1.08	0.88	0.74	0.29	0.19	0.22
OsR498G0815527400.01	1.82	0.583	-1.67433	1.66E-25	2.01E-25	1.51	1.96	1.99	0.64	0.68	0.43
OsR498G0815534900.01	365.14	9.23	-5.36068	0	0	360.44	368.79	366.2	9.39	8.94	9.36
OsR498G0815535500.01	25.4	0.726	-5.08994	0	0	24.83	26.11	25.26	0.66	0.58	0.94
OsR498G0815535700.01	9.143	0.3	-4.9171	0	0	8.36	9.52	9.55	0.33	0.27	0.3
OsR498G0815537800.01	2.466	0.586	-2.07824	1.39E-22	1.79E-22	2.56	2.15	2.69	0.51	0.73	0.52
OsR498G0815539300.01	11.173	47.25	1.981657	0	0	12.3	10.23	10.99	52.91	45.37	43.47
OsR498G0815539600.01	1.923	0.083	-4.53973	8.82E-60	7.21E-60	1.77	2.17	1.83	0.07	0.11	0.07
OsR498G0815540400.01	13.186	3.93	-1.90433	0	0	12.85	13.52	13.19	3.8	3.8	4.19
OsR498G0815540600.01	11.293	5.3	-1.10495	1.14E-23	1.43E-23	9.38	11.49	13.01	4.77	6.1	5.03
OsR498G0815545200.01	1.153	4.063	1.81201	3.37E-27	3.97E-27	0.99	1.22	1.25	4.09	4.36	3.74
OsR498G0815559000.01	0.443	0.086	-2.34365	1.87E-07	3.61E-07	0.48	0.43	0.42	0.08	0.12	0.06
OsR498G0815561600.01	0.913	11.873	3.687458	5.97E-115	3.41E-115	0.97	0.89	0.88	13.3	12.36	9.96
OsR498G0815564400.01	3.513	1.57	-1.21263	3.78E-34	4.04E-34	2.81	3.8	3.93	1.49	1.27	1.95
OsR498G0815571100.01	0.28	0	-4.96024	2.02E-05	4.33E-05	0.21	0.33	0.3	0	0	0
OsR498G0815581600.01	3.74	1.116	-1.73705	2.76E-38	2.81E-38	3.42	4.36	3.44	0.78	1.07	1.5
OsR498G0815584300.01	80.19	17.94	-2.11543	0	0	82.3	77.19	81.08	19.02	17.57	17.23
OsR498G0815585300.01	1.74	0.823	-1.09195	4.20E-08	7.88E-08	1.74	1.95	1.53	0.86	0.7	0.91
OsR498G0815588200.01	1.08	0.393	-1.47043	4.00E-08	7.50E-08	1.1	0.94	1.2	0.42	0.47	0.29
OsR498G0815589400.01	0.61	0.066	-3.23326	4.66E-12	7.65E-12	0.6	0.62	0.61	0.07	0.03	0.1
OsR498G0815589600.01	2.166	1.066	-1.0477	0.0009913	0.0023856	2.03	2.01	2.46	0.66	1.61	0.93
OsR498G0815591300.01	11.663	4.86	-1.26576	2.98E-90	1.95E-90	11.53	11.3	12.16	4.66	5.32	4.6
OsR498G0815593200.01	22.83	1.39	-4.04616	1.69E-195	7.03E-196	22.65	22.12	23.72	1.72	1.23	1.22
OsR498G0815593600.01	59.823	3.01	-4.32131	0	0	59.12	62.67	57.68	3.11	2.97	2.95
OsR498G0815593800.01	33.206	15.523	-1.1036	1.58E-90	1.03E-90	34.72	32.46	32.44	15.37	14.41	16.79
OsR498G0815597800.01	8.67	2.543	-1.99546	1.50E-112	8.66E-113	8.37	9.24	8.4	2.01	2.73	2.89
OsR498G0815599900.01	1.28	0.713	-1.02164	2.08E-05	4.46E-05	1.44	1.21	1.19	0.72	0.98	0.44
OsR498G0815603900.01	5.283	2.046	-1.33495	1.26E-23	1.59E-23	5.82	3.75	6.28	2.21	1.95	1.98
OsR498G0815613300.01	0.126	0.556	2.110149	0.0003986	0.0009301	0	0.08	0.3	0.71	0.4	0.56
OsR498G0815613500.01	0.426	1.023	1.241394	0.0003467	0.0008049	0.45	0.27	0.56	1.05	1.15	0.87
OsR498G0815617800.01	0.35	0.053	-2.69721	1.08E-10	1.85E-10	0.28	0.33	0.44	0.09	0.02	0.05
OsR498G0815626400.01	13.306	27.64	1.036661	7.44E-39	7.51E-39	13.24	12.62	14.06	28.8	27.09	27.03
OsR498G0815643600.01	12.2	2.75	-2.15885	3.45E-45	3.25E-45	11.09	12.58	12.93	4.03	2.72	1.5
OsR498G0815659600.01	11.936	67.456	2.457094	0	0	11.76	12.01	12.04	65.04	69.16	68.17
OsR498G0815661100.01	1.42	6.303	2.14194	5.20E-56	4.40E-56	1.44	1.31	1.51	6.03	6.07	6.81
OsR498G0815689000.01	3.16	1.316	-1.26509	3.56E-33	3.85E-33	3.16	3.13	3.19	1.32	1.22	1.41
OsR498G0815691300.01	1.006	0.11	-3.21897	1.34E-13	2.10E-13	0.9	1.08	1.04	0.14	0.05	0.14
OsR498G0815694100.01	0.396	0.95	1.332542	1.24E-07	2.36E-07	0.34	0.38	0.47	0.89	0.95	1.01
OsR498G0815698400.01	4.48	1.6	-1.52318	3.14E-20	4.22E-20	4.21	4.79	4.44	1.42	2.08	1.3
OsR498G0815699300.01	0.023	1.87	6.361688	1.03E-39	1.02E-39	0	0.07	0	1.74	1.91	1.96
OsR498G0815705100.01	3.01	0.36	-3.13016	4.36E-77	3.10E-77	2.65	2.96	3.42	0.28	0.46	0.34
OsR498G0815709200.01	4.06	1.6	-1.44067	4.19E-13	6.66E-13	5.2	3.38	3.6	1.97	1.44	1.39
OsR498G0815726700.01	0.61	1.71	1.453696	5.10E-05	0.0001118	0	0.54	1.29	1.37	1.74	2.02
OsR498G0815732100.01	0.21	0.02	-3.45647	8.89E-06	1.87E-05	0.22	0.21	0.2	0	0	0.06
OsR498G0815742700.01	51.84	22.036	-1.25181	5.79E-185	2.50E-185	55.13	49.82	50.57	23.23	22.07	20.81
OsR498G0815746700.01	0.356	21.77	5.945577	1.48E-187	6.30E-188	0.32	0.34	0.41	21.31	20.4	23.6
OsR498G0815765100.01	4.306	1.603	-1.24832	1.88E-19	2.58E-19	4.14	4.75	4.03	0.9	2.49	1.42
OsR498G0815767600.01	0.383	0.09	-2.11224	3.17E-06	6.49E-06	0.33	0.5	0.32	0.1	0.12	0.05
OsR498G0815772600.01	0.12	0.32	1.557273	0.0001528	0.0003455	0.11	0.15	0.1	0.25	0.45	0.26
OsR498G0815777800.01	73.916	27.87	-1.34038	2.92E-269	9.72E-270	76.13	72.46	73.16	32.03	26.64	24.94
OsR498G0815786100.01	0	0.096	4.499192	0.0003163	0.0007323	0	0	0	0.16	0.08	0.05
OsR498G0815787600.01	4.096	2	-1.03292	1.47E-07	2.82E-07	4.85	4.29	3.15	1.96	1.57	2.47
OsR498G0815788300.01	6.616	1.796	-1.87651	8.11E-86	5.43E-86	6.13	7.16	6.56	1.91	1.73	1.75

OsR498G0815789900.01	4.44	2.046	-1.12634	1.42E-18	1.98E-18	4.6	3.5	5.22	2.04	2.06	2.04
OsR498G0815799800.01	1.98	4.693	1.229238	1.23E-08	2.27E-08	2.51	1.95	1.48	5.15	4.23	4.7
OsR498G0815800700.01	0.506	4.71	3.151268	3.44E-151	1.67E-151	0.52	0.52	0.48	4.6	4.94	4.59
OsR498G0815804200.01	63.246	12.536	-2.42502	0	0	63.26	61.42	65.06	13.27	12.97	11.37
OsR498G0815805400.01	0.023	0.183	2.946651	0.0003132	0.0007244	0	0.07	0	0.22	0.11	0.22
OsR498G0815812500.01	0.01	1.82	7.632217	3.61E-44	3.45E-44	0	0	0.03	1.55	2.04	1.87
OsR498G0815813300.01	19.733	57.306	1.496342	0	0	19.04	18.41	21.75	57.37	56.44	58.11
OsR498G0815818800.01	5.193	1.716	-1.6041	2.61E-51	2.30E-51	5.53	5.06	4.99	1.72	1.55	1.88
OsR498G0815837200.01	0.54	1.91	2.109394	3.13E-45	2.95E-45	0.59	0.62	0.41	1.89	1.72	2.12
OsR498G0815848100.01	23.113	6.33	-1.88176	3.71E-197	1.53E-197	23.62	21.71	24.01	6.44	6.34	6.21
OsR498G0815850400.01	146.8	39.443	-1.87749	0	0	148.52	148.54	143.4	38.33	39.38	40.62
OsR498G0815855600.01	0.15	3.456	4.609616	1.55E-23	1.95E-23	0.22	0.23	0	3.82	3.4	3.15
OsR498G0815858000.01	47.056	7.813	-2.66287	0	0	47.98	46.77	46.42	8.91	7.42	7.11
OsR498G0815866300.01	9.836	0.12	-6.47001	1.06E-168	4.80E-169	9.43	10.54	9.54	0.18	0.15	0.03
OsR498G0815869800.01	0.806	0.11	-2.86713	0.0004299	0.0010056	0.79	1.17	0.46	0	0	0.33
OsR498G0815873600.01	0.39	4.553	3.552935	1.23E-28	1.41E-28	0.38	0.7	0.09	4.43	4.33	4.9
OsR498G0815875600.01	3.546	0.936	-1.92303	4.35E-43	4.20E-43	3.23	3.51	3.9	1	0.95	0.86
OsR498G0815877000.01	2.183	0.996	-1.13016	1.11E-12	1.78E-12	1.63	2.67	2.25	1.09	1.01	0.89
OsR498G0815877200.01	40.646	1.556	-4.72292	0	0	40.72	39.65	41.57	1.37	1.69	1.61
OsR498G0815885000.01	29.813	6.443	-2.22611	0	0	30.71	28.45	30.28	6.55	6.37	6.41
OsR498G0815887400.01	26.586	4.18	-2.64919	0	0	26.54	26.74	26.48	4.01	3.89	4.64
OsR498G0815887900.01	1.09	11.366	3.37015	1.32E-193	5.52E-194	0.92	1.27	1.08	11.33	11.68	11.09
OsR498G0815893300.01	0.56	0.243	-1.35054	8.70E-06	1.83E-05	0.6	0.56	0.52	0.4	0.06	0.27
OsR498G0815912200.01	58.953	122.3	1.081819	0	0	56.18	61.71	58.97	120.01	122.92	123.97
OsR498G0815912500.01	955.71	315.33	-1.61125	0	0	943.64	964.7	958.8	311.01	313.01	321.96
OsR498G0815926300.01	0.95	0.483	-1.02011	1.94E-06	3.93E-06	0.98	0.92	0.95	0.53	0.31	0.61
OsR498G0815932400.01	15.683	0.9	-4.09571	0	0	16.21	15	15.84	0.99	0.88	0.83
OsR498G0815937300.01	0.58	0	-6.51483	1.27E-12	2.05E-12	0.63	0.39	0.72	0	0	0
OsR498G0815941000.01	1.143	0.02	-5.84264	1.39E-15	2.08E-15	1.27	1.21	0.95	0.06	0	0
OsR498G0815946800.01	0.013	3.696	8.100456	6.90E-58	5.74E-58	0	0	0.04	3.23	3.65	4.21
OsR498G0815947600.01	26.22	85.703	1.723822	0	0	25.89	26.45	26.32	84.66	86	86.45
OsR498G0815955000.01	0.156	0.02	-3.18263	1.16E-06	2.33E-06	0.17	0.12	0.18	0.02	0.02	0.02
OsR498G0815960700.01	0.11	1.636	3.851617	8.15E-20	1.11E-19	0.06	0.06	0.21	1.28	2.05	1.58
OsR498G0815961000.01	16.806	7.51	-1.17167	2.99E-49	2.70E-49	16.7	16.52	17.2	6.88	8	7.65
OsR498G0815962200.01	198.32	72.786	-1.45055	0	0	197.31	198.89	198.8	74.77	71.64	71.95
OsR498G0815970500.01	35.413	8.416	-2.08771	3.54E-304	1.07E-304	34.27	36.71	35.26	8.09	8.77	8.39
OsR498G0815971100.01	2.78	0.79	-1.83471	3.74E-27	4.42E-27	2.79	3.01	2.54	0.92	0.91	0.54
OsR498G0815974500.01	5.056	13.423	1.402611	7.32E-49	6.64E-49	4.92	5.44	4.81	13.11	14.65	12.51
OsR498G0815975800.01	2.95	31.43	3.408871	0	0	2.77	3.01	3.07	30.39	32.35	31.55
OsR498G0815976400.01	6.166	3	-1.0427	4.08E-13	6.48E-13	6.12	6.06	6.32	2.57	2.77	3.66
OsR498G0815976900.01	1.023	3.723	1.80968	1.33E-09	2.35E-09	0.35	0.52	2.2	4.03	4.25	2.89
OsR498G0815977100.01	0.876	0.316	-1.26603	7.42E-07	1.47E-06	0.62	1.15	0.86	0.33	0.27	0.35
OsR498G0815979300.01	2.263	124.91	5.779639	0	0	1.84	2.38	2.57	135.04	123.35	116.35
OsR498G0815983000.01	0.026	6.056	7.881104	3.34E-198	1.37E-198	0	0.04	0.04	6.17	5.84	6.16
OsR498G0815983900.01	1.386	0.45	-1.7369	2.03E-06	4.11E-06	1.18	1.33	1.65	1.05	0.27	0.03
OsR498G0815985400.01	2.05	1.026	-1.01271	2.22E-14	3.40E-14	1.88	2.2	2.07	1	0.99	1.09
OsR498G0815986100.01	0.246	0.04	-2.57915	7.37E-08	1.40E-07	0.22	0.28	0.24	0.02	0.05	0.05
OsR498G0815992000.01	0.453	0.063	-2.86713	0.0004299	0.0010056	0.54	0.29	0.53	0	0.19	0
OsR498G0815992300.01	8.46	3.576	-1.28793	8.58E-57	7.22E-57	8.45	8.13	8.8	3.19	3.71	3.83
OsR498G0815995400.01	0.013	0.71	5.7402	2.71E-14	4.17E-14	0	0	0.04	0.6	0.75	0.78
OsR498G0815998500.01	0.296	2.393	3.02634	2.89E-38	2.94E-38	0.26	0.41	0.22	2.5	2.42	2.26
OsR498G0815999400.01	2.48	13.993	2.490222	4.70E-135	2.45E-135	2.11	2.52	2.81	14.05	13.56	14.37
OsR498G0816026100.01	130.44	43.02	-1.60044	0	0	136.99	129.06	125.3	46.86	39.99	42.21
OsR498G0816026400.01	5.78	41.163	2.828386	0	0	5.96	5.68	5.7	40.39	41.84	41.26
OsR498G0816026500.01	0.87	20.903	4.576701	0	0	0.79	0.81	1.01	18.94	21.91	21.86
OsR498G0816026900.01	0.05	0.223	2.209685	5.09E-05	0.0001115	0.07	0.08	0	0.19	0.23	0.25
OsR498G0816031300.01	1.89	13.363	2.820643	2.72E-241	9.80E-242	1.92	2.09	1.66	11.84	14.11	14.14
OsR498G0816031500.01	0.413	2.126	2.565222	4.06E-29	4.64E-29	0.55	0.31	0.38	2.23	1.96	2.19
OsR498G0816031900.01	24.513	7.666	-1.68968	8.54E-221	3.27E-221	24.25	25.8	23.49	7.38	8.7	6.92
OsR498G0816038800.01	0.053	1.31	4.651523	2.06E-07	3.98E-07	0	0.16	0	0.75	1.26	1.92
OsR498G0816038900.01	0	0.54	6.108001	7.78E-10	1.37E-09	0	0	0	0.63	0.5	0.49
OsR498G0816039300.01	0.906	0.323	-1.50673	1.49E-09	2.65E-09	1.12	0.94	0.66	0.3	0.44	0.23
OsR498G0816041700.01	2.11	31.37	3.888628	0	0	1.92	2.24	2.17	32.9	29.33	31.88
OsR498G0816044700.01	2.056	4.863	1.243291	1.07E-21	1.39E-21	1.79	2.61	1.77	4.37	5.32	4.9
OsR498G0816045500.01	2.323	0.973	-1.25819	2.74E-09	4.91E-09	2.87	2.18	1.92	1.29	1.08	0.55
OsR498G0816047300.01	0.04	1.856	5.515493	4.23E-23	5.36E-23	0.06	0.06	0	2.16	1.27	2.14
OsR498G0816052700.01	0.276	0	-4.86713	3.63E-05	7.88E-05	0.22	0.4	0.21	0	0	0
OsR498G0816054900.01	67.916	187.74	1.461365	0	0	66.44	68.95	68.36	180.24	188.89	194.08
OsR498G0816057100.01	10.08	4.463	-1.1856	2.45E-56	2.07E-56	10.74	9.51	9.99	4.3	4.77	4.32

OsR498G0816059800.01	176.1	44.556	-1.98747	0	0	177.08	169.64	181.6	49.2	42.38	42.09
OsR498G0816061900.01	34.87	109.63	1.607764	0	0	31.93	39.22	33.46	116.55	107.51	104.84
OsR498G0816064500.01	88.143	5.33	-4.03866	0	0	87.4	89.71	87.32	5.23	5.48	5.28
OsR498G0816068700.01	0.703	26.233	5.208602	0	0	0.8	0.7	0.61	24.57	27.59	26.54
OsR498G0816074700.01	0.03	0.22	3.03976	6.82E-06	1.42E-05	0.03	0.06	0	0.13	0.28	0.25
OsR498G0816077400.01	2.476	0.91	-1.47043	4.00E-08	7.50E-08	1.57	2.75	3.11	0.72	0.71	1.3
OsR498G0816077600.01	5.326	14.343	1.290951	6.98E-80	4.86E-80	4.5	5.02	6.46	13.16	15.42	14.45
OsR498G0816077700.01	0.06	47.446	9.598659	0	0	0.05	0	0.13	49.5	47.94	44.9
OsR498G0816080900.01	2.883	6.396	1.119824	2.02E-19	2.76E-19	3.05	3.06	2.54	6.69	6.8	5.7
OsR498G0816082900.01	1.253	0.46	-1.56169	2.85E-08	5.31E-08	1.36	1.03	1.37	0.6	0.29	0.49
OsR498G0816083200.01	0.083	0	-4.27639	0.0007304	0.0017407	0.17	0.02	0.06	0	0	0
OsR498G0816088300.01	250.77	756.79	1.582583	0	0	258.92	245.12	248.3	777.74	750.43	742.21
OsR498G0816088900.01	12.016	24.043	1.045209	2.65E-87	1.76E-87	12.83	11.85	11.37	23.83	25.7	22.6
OsR498G0816099200.01	3.97	0.8	-2.32947	4.27E-25	5.23E-25	4.29	3.36	4.26	0.59	0.74	1.07
OsR498G0816099700.01	7.613	2.87	-1.42364	2.66E-19	3.65E-19	8.84	6.67	7.33	2.65	3.08	2.88
OsR498G0816109100.01	29.54	13.39	-1.15218	1.98E-149	9.68E-150	28.68	30.13	29.81	13.81	12.88	13.48
OsR498G0816110100.01	4.583	22.823	2.352643	1.18E-269	3.91E-270	4.59	5.11	4.05	20.45	25.23	22.79
OsR498G0816110700.01	19.5	3.403	-2.52871	6.28E-145	3.13E-145	19.55	18.82	20.13	3.08	3.76	3.37
OsR498G0816112000.01	1.626	0.63	-1.37205	7.63E-11	1.30E-10	1.6	1.64	1.64	0.6	0.58	0.71
OsR498G0816112500.01	0.026	0.45	3.563322	2.65E-06	5.40E-06	0	0	0.08	0.59	0.53	0.23
OsR498G0816115300.01	1.543	4.65	1.574932	9.73E-52	8.56E-52	1.59	1.58	1.46	4.13	5.42	4.4
OsR498G0816116900.01	4.356	1.363	-1.68686	3.11E-13	4.93E-13	4.88	3.66	4.53	2.51	0.89	0.69
OsR498G0816119300.01	7.416	108.28	3.867054	0	0	6.81	8.66	6.78	109.08	106.24	109.53
OsR498G0816121400.01	13.38	36.083	1.429762	9.51E-238	3.45E-238	13.24	13.51	13.39	36.08	35.47	36.7
OsR498G0816124800.01	15.45	5.97	-1.38217	8.97E-49	8.15E-49	16.88	14.26	15.21	6.14	6.64	5.13
OsR498G0816126000.01	30.433	15.11	-1.01071	0	0	30.87	29.87	30.56	15.38	14.85	15.1
OsR498G0816128800.01	1.186	0.26	-2.20817	9.63E-11	1.64E-10	1.36	1.03	1.17	0.24	0.25	0.29
OsR498G0816134200.01	2.2	0.966	-1.358	1.02E-11	1.68E-11	1.99	2.47	2.14	1.1	0.54	1.26
OsR498G0816135600.01	16.776	4.603	-1.85809	5.89E-91	3.83E-91	18.5	16.04	15.79	5.21	4.1	4.5
OsR498G0816135800.01	9.2	2.82	-1.74578	8.08E-78	5.71E-78	9.41	9.24	8.95	2.54	3.03	2.89
OsR498G0816137300.01	32.296	80.476	1.313348	0	0	30.55	33.68	32.66	78	82.32	81.11
OsR498G0816137700.01	8.773	1.863	-2.22011	5.11E-34	5.47E-34	6.9	9.82	9.6	1.83	1.7	2.06
OsR498G0816137900.01	1.006	0.23	-2.07213	2.40E-16	3.52E-16	1.04	1.1	0.88	0.27	0.2	0.22
OsR498G0816138200.01	3.253	1.07	-1.60974	2.35E-26	2.81E-26	3.8	2.9	3.06	0.96	1.18	1.07
OsR498G0816139100.01	2.276	1.07	-1.12251	6.22E-06	1.29E-05	2.4	2.24	2.19	1.24	1.22	0.75
OsR498G0816148700.01	42.323	127.35	1.585208	0	0	44.49	40.53	41.95	127.37	125.27	129.42
OsR498G0816149600.01	1.293	0.296	-2.17576	1.85E-16	2.70E-16	1.57	0.84	1.47	0.33	0.17	0.39
OsR498G0816150900.01	0.09	0.636	2.810278	3.23E-20	4.34E-20	0.12	0.08	0.07	0.75	0.59	0.57
OsR498G0816151100.01	3.873	60.886	3.96655	0	0	4.08	3.68	3.86	65.57	60.8	56.29
OsR498G0816151600.01	37.053	392.61	3.379413	0	0	34.87	38.27	38.02	398.16	394.48	385.18
OsR498G0816151700.01	0	0.506	5.180539	5.82E-06	1.21E-05	0	0	0	0.47	0	1.05
OsR498G0816153900.01	2.78	1.163	-1.2645	5.50E-17	7.94E-17	2.86	2.76	2.72	1.07	1.05	1.37
OsR498G0816154100.01	0.92	0.51	-1.06115	1.13E-10	1.94E-10	0.85	0.75	1.16	0.51	0.52	0.5
OsR498G0816159000.01	1432.1	101.81	-3.82123	0	0	1371	1442.9	1482	100.29	102.92	102.22
OsR498G0816161300.01	0.213	0	-5.13016	6.38E-06	1.33E-05	0.22	0.11	0.31	0	0	0
OsR498G0816166000.01	3.17	1.01	-1.63343	2.57E-20	3.45E-20	3	2.6	3.91	1.43	0.83	0.77
OsR498G0816166700.01	1.083	0.246	-2.15754	5.41E-17	7.82E-17	1.06	1.09	1.1	0.23	0.26	0.25
OsR498G0816167400.01	0.51	3.71	2.847115	4.61E-50	4.13E-50	0.73	0.37	0.43	3.51	4.01	3.61
OsR498G0816171100.01	36.79	74.206	1.00286	1.74E-132	9.10E-133	36.04	36.35	37.98	77.8	73.41	71.41
OsR498G0816171500.01	1.896	4.563	1.303836	1.66E-49	1.49E-49	1.79	1.67	2.23	4.55	4.74	4.4
OsR498G0816173800.01	7.41	18.923	1.355347	6.78E-71	5.07E-71	7.51	6.64	8.08	18.41	18.76	19.6
OsR498G0816180400.01	2.356	38.253	4.015277	0	0	2.44	2.35	2.28	41.81	38.49	34.46
OsR498G0816182700.01	0	0.26	4.7402	9.30E-05	0.0002074	0	0	0	0.18	0.12	0.48
OsR498G0816184400.01	0.703	0.196	-1.85775	1.39E-05	2.96E-05	0.58	0.79	0.74	0.33	0.23	0.03
OsR498G0816189900.01	0.016	0.713	5.287688	7.27E-11	1.24E-10	0.05	0	0	1	0.8	0.34
OsR498G0816196900.01	0.156	3.176	4.588197	3.41E-108	2.01E-108	0.15	0.13	0.19	3.59	2.95	2.99
OsR498G0816198200.01	0.463	0.16	-1.53099	5.09E-09	9.22E-09	0.55	0.39	0.45	0.17	0.14	0.17
OsR498G0816201300.01	25.316	3.093	-3.03862	6.60E-212	2.60E-212	24.11	26.19	25.65	3.47	3.2	2.61
OsR498G0816205500.01	0.066	0.386	3.105562	1.32E-10	2.26E-10	0	0.17	0.03	0.33	0.41	0.42
OsR498G0816205800.01	0.42	7.893	4.226965	5.94E-181	2.59E-181	0.53	0.41	0.32	8.53	7.9	7.25
OsR498G0816206100.01	0	0.806	6.084154	1.05E-09	1.85E-09	0	0	0	0.72	0.67	1.03
OsR498G0816212400.01	1.133	0.123	-3.06458	4.87E-09	8.82E-09	1.17	1.08	1.15	0.2	0.17	0
OsR498G0816213300.01	19.913	4.606	-2.12069	1.80E-119	1.01E-119	18.92	21.32	19.5	3.6	4.68	5.54
OsR498G0816218100.01	57.15	21.733	-1.4015	2.37E-188	1.01E-188	58.46	55.32	57.67	22.36	21.77	21.07
OsR498G0816223900.01	144.19	5.343	-4.79021	0	0	154.3	139.29	139	5.73	4.58	5.72
OsR498G0816230800.01	1.48	0.15	-3.41967	4.89E-14	7.59E-14	1.55	1.77	1.12	0.13	0.19	0.13
OsR498G0816232300.01	0.04	1.596	5.209685	2.03E-27	2.38E-27	0.04	0.04	0.04	1.26	1.8	1.73
OsR498G0816234700.01	1.623	0.263	-2.66068	1.75E-07	3.38E-07	1.76	0.93	2.18	0.39	0.4	0
OsR498G0816238400.01	0.45	9.27	4.364821	1.33E-206	5.31E-207	0.44	0.49	0.42	9.26	8.98	9.57

OsR498G0816240500.01	43.416	97.196	1.160348	0	0	43.52	42.75	43.98	96.25	99.37	95.97
OsR498G0816242700.01	2.166	4.753	1.131683	5.21E-13	8.29E-13	1.58	2.85	2.07	4.9	5.12	4.24
OsR498G0816246400.01	0.366	1.29	1.820273	8.67E-22	1.13E-21	0.23	0.48	0.39	1.32	1.35	1.2
OsR498G0816252800.01	1.693	4.56	1.413884	3.14E-40	3.11E-40	1.68	1.71	1.69	4.39	4.13	5.16
OsR498G0816253400.01	9.58	4.163	-1.17806	1.15E-47	1.06E-47	10.05	9.28	9.41	3.86	4.32	4.31
OsR498G0816253600.01	0.313	0.866	1.736833	3.15E-11	5.29E-11	0.3	0.23	0.41	0.94	0.91	0.75
OsR498G0816256800.01	0.326	0.093	-1.85332	0.0003648	0.0008482	0.34	0.35	0.29	0.08	0.12	0.08
OsR498G0816257600.01	0.16	0.67	2.03976	3.78E-06	7.78E-06	0.34	0.05	0.09	0.79	0.31	0.91
OsR498G0816258300.01	4.54	1.443	-1.66965	1.30E-11	2.17E-11	4.15	4.49	4.98	1.62	1.77	0.94
OsR498G0816259500.01	67.65	21.763	-1.64944	2.30E-234	8.45E-235	68.93	67.09	66.93	22.19	20.26	22.84
OsR498G0816265000.01	66.223	14.466	-2.15684	0	0	66.18	65.04	67.45	14.55	13.5	15.35
OsR498G0816265900.01	0.336	0.026	-3.73283	6.30E-10	1.11E-09	0.27	0.43	0.31	0	0.05	0.03
OsR498G0816271500.01	0	0.13	4.499192	0.0003163	0.0007323	0	0	0	0.18	0.07	0.14
OsR498G0816272100.01	3.273	0.26	-3.71026	6.49E-31	7.23E-31	3.48	3.1	3.24	0.11	0.47	0.2
OsR498G0816274900.01	146.39	60.076	-1.28589	0	0	134.82	151.31	153.1	64.82	58.72	56.69
OsR498G0816276200.01	3.583	0.473	-3.03162	6.46E-76	4.63E-76	3.66	4.91	2.18	0.67	0.41	0.34
OsR498G0816278300.01	0.96	0.183	-2.3932	2.67E-12	4.34E-12	0.92	1.04	0.92	0.14	0.3	0.11
OsR498G0816279300.01	2.273	0.11	-4.41967	3.02E-16	4.43E-16	2.48	1.74	2.6	0.11	0	0.22
OsR498G0816280200.01	0.283	0.053	-2.35256	0.0002107	0.0004813	0.29	0.21	0.35	0.12	0	0.04
OsR498G0816284600.01	5.373	0.036	-7.19864	2.16E-68	1.65E-68	5.41	5.79	4.92	0.05	0	0.06
OsR498G0816288300.01	0.01	0.116	3.7402	0.0002182	0.0004994	0.03	0	0	0.07	0.16	0.12
OsR498G0816288900.01	6.376	17.71	1.480531	6.36E-74	4.64E-74	6.96	6.72	5.45	16.08	19.26	17.79
OsR498G0816292400.01	13.19	5.133	-1.38702	1.61E-87	1.07E-87	13.22	12.86	13.49	5.44	4.69	5.27
OsR498G0816295500.01	0.15	0	-5.0477	1.13E-05	2.39E-05	0.13	0.11	0.21	0	0	0
OsR498G0816299200.01	1	0.146	-2.76759	2.15E-11	3.60E-11	1.24	0.8	0.96	0.24	0.05	0.15
OsR498G0816299500.01	1.12	4.336	1.951016	7.78E-36	8.14E-36	1.1	1.31	0.95	4.9	3.88	4.23
OsR498G0816305600.01	1.766	4.706	1.409306	9.48E-27	1.13E-26	1.42	1.82	2.06	5.26	4.44	4.42
OsR498G0816308800.01	0.376	2.15	2.509245	6.23E-12	1.03E-11	0.52	0.36	0.25	2.47	2.18	1.8
OsR498G0816310400.01	28.036	57.346	1.030258	0	0	27.39	29.48	27.24	54.96	59.9	57.18
OsR498G0816312000.01	102.88	413.61	2.028832	0	0	102.18	103.38	103.1	445.29	405.12	390.42
OsR498G0816314700.01	5.113	0.113	-5.46803	3.39E-128	1.81E-128	4.86	5.64	4.84	0.09	0.09	0.16
OsR498G0816319500.01	44.263	135.53	1.604542	0	0	44.81	44.13	43.85	137.64	134.76	134.19
OsR498G0816319800.01	147.65	348.96	1.238624	0	0	145.19	149.23	148.5	340.66	354.06	352.17
OsR498G0816322700.01	8.033	20.8	1.36822	1.82E-122	1.00E-122	8.43	7.96	7.71	20.98	20.35	21.07
OsR498G0816324000.01	2.06	0.333	-2.62857	2.17E-08	4.03E-08	1.69	3.08	1.41	0.55	0.31	0.14
OsR498G0816325700.01	12.87	4.556	-1.43331	8.26E-50	7.42E-50	12.31	14.16	12.14	4.26	5	4.41
OsR498G0816326400.01	13.44	5.803	-1.21348	1.96E-76	1.40E-76	13.37	14.26	12.69	6.01	6.28	5.12
OsR498G0816327100.01	11.616	5.09	-1.20333	3.41E-73	2.50E-73	11.65	11.23	11.97	4.9	4.76	5.61
OsR498G0816328500.01	0.663	4.81	2.823522	7.44E-34	7.98E-34	0	0	1.99	5.37	2.29	6.77
OsR498G0816331200.01	0.246	1.623	2.721815	1.26E-14	1.92E-14	0.28	0.35	0.11	1.72	1.39	1.76
OsR498G0816331600.01	24.556	0.413	-5.65563	0	0	25.24	24.34	24.09	0.35	0.36	0.53
OsR498G0816334900.01	2.203	0.76	-1.61862	4.42E-22	5.73E-22	2.45	2.15	2.01	0.5	1.06	0.72
OsR498G0816335900.01	0.07	0.436	2.664251	2.39E-07	4.64E-07	0.07	0.04	0.1	0.52	0.36	0.43
OsR498G0816340500.01	3.646	18.236	2.382152	6.34E-198	2.61E-198	3.68	3.67	3.59	18.66	17.59	18.46
OsR498G0816343000.01	0.316	0	-4.41967	0.0003895	0.0009082	0.35	0.22	0.38	0	0	0
OsR498G0816343400.01	23.766	7.72	-1.65164	5.12E-150	2.50E-150	25.64	22.55	23.11	8.74	7.12	7.3
OsR498G0816344600.01	13.266	3.93	-1.76824	6.36E-100	3.92E-100	13.33	13.72	12.75	3.91	3.47	4.41
OsR498G0816347700.01	4.363	0.353	-3.72046	4.09E-55	3.49E-55	4.82	3.29	4.98	0.45	0.47	0.14
OsR498G0816352400.01	12.316	0.403	-4.93887	7.59E-245	2.70E-245	11.96	11.93	13.06	0.48	0.55	0.18
OsR498G0816355900.01	0.283	0.043	-2.20817	0.0005899	0.0013949	0.28	0.22	0.35	0	0.13	0
OsR498G0816362100.01	0.126	0	-4.28217	0.0007127	0.0016975	0.04	0.16	0.18	0	0	0
OsR498G0816366800.01	17.69	36.916	1.070396	2.53E-129	1.35E-129	18.6	16.93	17.54	36.31	37.09	37.35
OsR498G0816367700.01	1.293	9.506	2.933404	2.25E-305	6.80E-306	1.42	1.24	1.22	8.96	10.38	9.18
OsR498G0816373700.01	0.386	0.846	1.117763	0.0002607	0.0006001	0.3	0.45	0.41	0.7	0.86	0.98
OsR498G0816373900.01	0.543	1.34	1.331526	5.31E-06	1.10E-05	0.56	0.7	0.37	1.45	1.16	1.41
OsR498G0816379300.01	41.793	111.07	1.391025	0	0	42.38	42.58	40.42	110.1	110.6	112.52
OsR498G0816386000.01	21.013	7.976	-1.39979	1.86E-186	7.98E-187	21.06	21.47	20.51	8.6	7.88	7.45
OsR498G0816386100.01	0	0.91	7.833006	9.09E-26	1.10E-25	0	0	0	0	1.26	1.47
OsR498G0816389500.01	33.436	15.943	-1.07069	2.90E-132	1.52E-132	34.79	32.54	32.98	16.19	15	16.64
OsR498G0816390800.01	42.11	187.07	2.162749	0	0	41.23	43.69	41.41	198.38	175.39	187.44
OsR498G0816395800.01	0.77	0.253	-1.50081	1.84E-08	3.41E-08	0.81	0.81	0.69	0.12	0.27	0.37
OsR498G0816397400.01	5.366	2.273	-1.25073	2.49E-38	2.52E-38	5.25	4.92	5.93	2.5	2.37	1.95
OsR498G0816397900.01	2.773	11.633	2.070698	2.16E-158	1.02E-158	2.38	2.97	2.97	11.59	11.28	12.03
OsR498G0816401700.01	0.04	0.176	2.209685	0.0006752	0.0016052	0.09	0.03	0	0.2	0.24	0.09
OsR498G0816404600.01	0.493	0.07	-2.76759	6.01E-05	0.0001325	0.28	0.52	0.68	0.07	0.07	0.07
OsR498G0816405000.01	3.066	0.993	-1.61629	7.05E-34	7.56E-34	3.07	3.32	2.81	0.92	1.17	0.89
OsR498G0816405900.01	0.533	0	-7.08952	2.00E-17	2.86E-17	0.6	0.4	0.6	0	0	0
OsR498G0816407600.01	0.173	0	-5.27566	2.15E-06	4.36E-06	0.16	0.2	0.16	0	0	0
OsR498G0816407700.01	0.346	0.026	-4.13736	1.36E-05	2.89E-05	0.42	0.25	0.37	0	0	0.08

OsR498G0816415100.01	25.316	7.873	-1.68931	6.77E-285	2.17E-285	26.26	24.31	25.38	7.85	7.98	7.79
OsR498G0816418200.01	12.066	4.936	-1.2938	6.42E-50	5.76E-50	12.36	12.26	11.58	4.72	5.49	4.6
OsR498G0816418300.01	19.396	0.276	-6.06727	3.97E-225	1.50E-225	18.07	20.29	19.83	0.41	0.12	0.3
OsR498G0816419100.01	59.166	165.88	1.472883	0	0	59.47	57.32	60.71	179.08	161.05	157.51
OsR498G0816420800.01	0.44	0.943	1.092871	2.23E-05	4.78E-05	0.66	0.24	0.42	1.07	0.73	1.03
OsR498G0816422700.01	2.276	0.29	-3.09215	2.32E-71	1.72E-71	2.46	2.12	2.25	0.3	0.24	0.33
OsR498G0816425400.01	0.266	0	-5.4838	3.79E-07	7.43E-07	0.31	0.29	0.2	0	0	0
OsR498G0816430400.01	0.023	0.52	4.499192	8.67E-12	1.43E-11	0	0.07	0	0.59	0.47	0.5
OsR498G0816432100.01	0.873	0.036	-4.61502	1.20E-18	1.67E-18	0.93	0.87	0.82	0	0.11	0
OsR498G0816434300.01	0.013	1.29	6.648569	1.03E-24	1.27E-24	0.04	0	0	1.1	1.41	1.36
OsR498G0816434700.01	0	1.123	7.630721	5.13E-23	6.52E-23	0	0	0	1.19	0.99	1.19
OsR498G0816435000.01	0.69	0.15	-2.21263	2.35E-11	3.93E-11	0.61	0.8	0.66	0.26	0.11	0.08
OsR498G0816438400.01	0.3	0.683	1.346421	0.000137	0.000309	0.51	0.19	0.2	0.6	0.76	0.69
OsR498G0816439300.01	2.466	10.976	2.133272	1.00E-166	4.58E-167	2.41	2.24	2.75	11.27	10.49	11.17
OsR498G0816439800.01	0.61	48.613	6.310359	0	0	0.57	0.57	0.69	51.21	48.51	46.12
OsR498G0816440900.01	3.006	73.716	4.611275	0	0	2.61	3.06	3.35	76.62	72.35	72.18
OsR498G0816441200.01	0.91	0.01	-6.64674	2.54E-25	3.09E-25	0.87	0.86	1	0.03	0	0
OsR498G0816445800.01	1.06	4.123	1.768276	1.24E-42	1.20E-42	1.02	1.25	0.91	4.24	4.39	3.74
OsR498G0816450400.01	1.06	4.276	2.008733	1.69E-21	2.21E-21	1.28	1.06	0.84	5.02	4.17	3.64
OsR498G0816453100.01	207.3	16.31	-3.67239	0	0	223.23	191.49	207.2	17.24	16.54	15.15
OsR498G0816457300.01	8.82	19.233	1.210863	2.97E-189	1.26E-189	8.26	9.21	8.99	17.32	20.09	20.29
OsR498G0816459900.01	0	1.386	8.116576	3.58E-30	4.03E-30	0	0	0	1.45	1.44	1.27
OsR498G0816464400.01	0.946	6.176	2.690311	1.23E-42	1.20E-42	1.12	0.76	0.96	7.31	4.67	6.55
OsR498G0816465500.01	2.283	0.873	-1.39158	2.64E-07	5.13E-07	1.84	2.57	2.44	1.33	0.81	0.48
OsR498G0816466200.01	9.073	27.593	1.687889	0	0	8.64	9.03	9.55	26.67	27.5	28.61
OsR498G0816466800.01	16.596	35.05	1.074779	7.54E-218	2.91E-218	16.9	15.78	17.11	34.62	35.58	34.95
OsR498G0816467000.01	3.83	1.523	-1.33528	4.25E-21	5.61E-21	4.03	3.76	3.7	1.65	1.49	1.43
OsR498G0816467200.01	5.986	2.756	-1.11395	3.11E-18	4.36E-18	5.42	6.47	6.07	2.59	2.8	2.88
OsR498G0816468300.01	1.856	29.333	3.918374	0	0	1.89	2.04	1.64	29.88	30.32	27.8
OsR498G0816468800.01	6.66	2.97	-1.16714	1.91E-13	3.01E-13	7.35	7.05	5.58	3.6	3.03	2.28
OsR498G0816469100.01	16.213	59.103	1.863404	0	0	16.44	16.64	15.56	57.23	62.54	57.54
OsR498G0816475800.01	29.033	58.433	1.002107	1.64E-220	6.30E-221	27.73	28.53	30.84	59.12	58.36	57.82
OsR498G0816476700.01	3.516	0.526	-2.76416	3.75E-65	2.93E-65	3.74	3.19	3.62	0.5	0.52	0.56
OsR498G0816477500.01	0.47	1.746	1.917504	2.35E-31	2.60E-31	0.33	0.71	0.37	1.89	1.83	1.52
OsR498G0816481100.01	5.46	1.773	-1.62909	2.86E-62	2.29E-62	6.09	5.1	5.19	1.68	1.76	1.88
OsR498G0816482400.01	0.713	0.06	-3.6041	4.33E-21	5.72E-21	0.62	0.76	0.76	0.04	0.07	0.07
OsR498G0816482800.01	4.67	1.166	-2.00228	2.02E-41	1.98E-41	4.67	4.51	4.83	1.22	1.07	1.21
OsR498G0816484000.01	1.803	6.206	1.818728	1.12E-83	7.59E-84	1.92	1.95	1.54	6.69	5.21	6.72
OsR498G0816484100.01	0.636	2.343	1.860419	4.40E-10	7.69E-10	0.41	0.66	0.84	2.04	2.24	2.75
OsR498G0816484600.01	7.336	1.41	-2.42452	9.45E-80	6.59E-80	6.94	6.87	8.2	1.05	1.32	1.86
OsR498G0816484800.01	5.126	2.03	-1.36094	9.42E-24	1.18E-23	5.31	4.74	5.33	2.38	1.85	1.86
OsR498G0816493500.01	0.26	0.02	-3.6041	1.31E-06	2.63E-06	0.28	0.23	0.27	0	0.03	0.03
OsR498G0816496500.01	1.046	0.386	-1.44023	1.02E-08	1.87E-08	0.97	1.26	0.91	0.3	0.46	0.4
OsR498G0816496700.01	144.95	46.816	-1.62977	0	0	149.39	141.97	143.5	46.87	47.82	45.76
OsR498G0816497000.01	1.493	0.56	-1.42519	4.88E-20	6.60E-20	1.35	1.29	1.84	0.64	0.45	0.59
OsR498G0816498900.01	420.32	142.41	-1.57917	0	0	439.62	413.89	407.5	141.37	136.44	149.41
OsR498G0816499600.01	11.236	56.763	2.328964	0	0	9.67	11.35	12.69	61.69	55.51	53.09
OsR498G0816501100.01	0.37	0.14	-1.57915	5.73E-05	0.0001261	0.29	0.34	0.48	0.06	0.19	0.17
OsR498G0816506400.01	0.08	0.613	2.914229	2.18E-05	4.67E-05	0.08	0.08	0.08	0.66	0.59	0.59
OsR498G0816507800.01	0	0.206	4.361688	0.0005877	0.0013892	0	0	0	0.26	0.16	0.2
OsR498G0816509400.01	0.023	0.42	4.084154	6.36E-09	1.16E-08	0.04	0	0.03	0.36	0.48	0.42
OsR498G0816510300.01	3.483	13.273	1.921226	4.53E-71	3.38E-71	3.51	3.39	3.55	14.5	12.26	13.06
OsR498G0816513900.01	0.35	0.016	-4.4838	7.49E-07	1.49E-06	0.45	0.29	0.31	0.05	0	0
OsR498G0816515500.01	2.68	6.926	1.36257	4.80E-52	4.21E-52	2.9	2.44	2.7	6.64	7.18	6.96
OsR498G0816516200.01	0.836	0.336	-1.33177	0.0006092	0.0014422	0.79	1.05	0.67	0.15	0.46	0.4
OsR498G0816519700.01	7.716	0.263	-4.83982	5.04E-108	2.97E-108	7.16	7.99	8	0.29	0.2	0.3
OsR498G0816521800.01	16.266	6.743	-1.27713	3.74E-48	3.42E-48	17.46	15.78	15.56	6.82	6.62	6.79
OsR498G0816524700.01	7.346	1.556	-2.22474	9.11E-95	5.77E-95	6.98	7.32	7.74	1.7	1.4	1.57
OsR498G0816530400.01	23.07	51.506	1.156214	1.95E-258	6.67E-259	23.07	23.7	22.44	51.85	49.27	53.4
OsR498G0816530900.01	1.383	7.306	2.330592	4.98E-72	3.68E-72	1.04	1.47	1.64	7.22	7.24	7.46
OsR498G0816531200.01	2.54	0.343	-2.8394	1.67E-39	1.67E-39	2.32	2.74	2.56	0.5	0.23	0.3
OsR498G0816534200.01	0	0.27	5.432077	8.26E-07	1.64E-06	0	0	0	0.23	0.27	0.31
OsR498G0816534500.01	0.303	1.51	2.298494	2.61E-10	4.52E-10	0.22	0.42	0.27	1.2	1.69	1.64
OsR498G0816540800.01	4.04	1.71	-1.3731	6.49E-42	6.34E-42	3.43	4.15	4.54	1.71	1.78	1.64
OsR498G0816541900.01	9.83	3.856	-1.35476	2.20E-84	1.49E-84	10.15	9.76	9.58	3.84	3.88	3.85
OsR498G0816542500.01	11.686	37.496	1.676242	0	0	11.62	12.13	11.31	37.08	37.69	37.72
OsR498G0816544000.01	0.376	0.066	-2.6232	3.00E-07	5.84E-07	0.52	0.51	0.1	0.09	0	0.11
OsR498G0816548900.01	3.07	1.053	-1.54943	2.88E-51	2.55E-51	3.08	2.85	3.28	1.06	0.98	1.12
OsR498G0816554000.01	0.61	0.173	-1.82006	9.21E-11	1.57E-10	0.6	0.49	0.74	0.15	0.14	0.23

OsR498G0816554800.01	8.24	25.39	1.64206	3.75E-191	1.59E-191	8.17	8.37	8.18	24.75	26.22	25.2
OsR498G0816558700.01	1.846	0.856	-1.1104	1.05E-20	1.40E-20	1.71	1.93	1.9	0.77	0.85	0.95
OsR498G0816559900.01	1.816	0.806	-1.11427	1.04E-08	1.90E-08	1.82	1.81	1.82	0.86	0.91	0.65
OsR498G0816563700.01	0.086	0.373	2.397682	3.31E-12	5.41E-12	0.1	0.12	0.04	0.39	0.28	0.45
OsR498G0816564400.01	3.266	9.046	1.46909	1.37E-64	1.07E-64	3.11	3.55	3.14	9.77	8.95	8.42
OsR498G0816564900.01	23.196	71.466	1.477788	0	0	21.1	25.56	22.93	68.94	76.07	69.39
OsR498G0816565700.01	28.01	3.313	-3.09093	0	0	25.75	29.11	29.17	3.51	3.09	3.34
OsR498G0816566000.01	1.43	0.26	-2.49658	7.08E-13	1.13E-12	2.17	0.88	1.24	0	0.78	0
OsR498G0816567100.01	2.5	0.256	-3.28784	7.37E-37	7.60E-37	2.97	2.56	1.97	0.21	0.38	0.18
OsR498G0816567100.01.'	31.12	8.05	-1.95454	8.62E-204	3.48E-204	31.15	31.3	30.91	8.01	7.7	8.44
OsR498G0816568800.01	0.373	0.086	-2.16187	4.69E-07	9.23E-07	0.39	0.4	0.33	0.05	0.13	0.08
OsR498G0816569900.01	11.746	1.326	-2.55573	5.40E-74	3.93E-74	11.75	12.65	10.84	1.16	1.37	1.45
OsR498G0816570400.01	1.37	0.166	-3.20125	5.28E-20	7.14E-20	1.39	1.16	1.56	0.17	0.12	0.21
OsR498G0816570600.01	0.75	4.41	2.547815	3.20E-51	2.83E-51	0.81	0.73	0.71	4.83	4.34	4.06
OsR498G0816572100.01	3.966	1.693	-1.22498	1.54E-27	1.81E-27	4.25	3.8	3.85	1.7	1.59	1.79
OsR498G0816573200.01	0.976	0.02	-5.92217	2.75E-45	2.59E-45	1.03	0.87	1.03	0	0	0.06
OsR498G0816578500.01	6.126	1.623	-1.92263	8.05E-28	9.40E-28	5.47	5.96	6.95	2.12	1.38	1.37
OsR498G0816584800.01	3.24	22.606	2.798906	2.08E-228	7.79E-229	3.45	3.24	3.03	24.59	22.21	21.02
OsR498G0816587400.01	0.243	0.053	-2.20817	0.0005899	0.0013949	0.32	0.13	0.28	0.08	0	0.08
OsR498G0816587600.01	0.396	0.103	-1.86713	0.0001398	0.0003156	0.5	0.3	0.39	0.08	0.15	0.08
OsR498G0816587800.01	2.313	1.276	-1.05022	2.39E-10	4.13E-10	2.36	2.36	2.22	0.87	1.36	1.6
OsR498G0816588400.01	0.643	0.063	-3.52095	5.36E-13	8.53E-13	0.42	0.75	0.76	0.11	0.08	0
OsR498G0816588600.01	0.296	0.11	-1.68013	2.35E-06	4.78E-06	0.29	0.28	0.32	0.11	0.11	0.11
OsR498G0816589600.01	0.313	0.056	-2.23326	3.24E-05	7.02E-05	0.25	0.19	0.5	0.02	0.1	0.05
OsR498G0816589700.01	0.953	4.306	2.174061	4.14E-23	5.25E-23	0.76	1.23	0.87	4.17	4.36	4.39
OsR498G0816591100.01	0.316	0.773	1.334548	3.01E-08	5.62E-08	0.39	0.3	0.26	0.72	0.74	0.86
OsR498G0816591500.01	0	0.24	5.742972	4.73E-08	8.91E-08	0	0	0	0.31	0.25	0.16
OsR498G0816594900.01	0.596	1.796	1.606107	5.51E-20	7.46E-20	0.67	0.64	0.48	1.65	1.88	1.86
OsR498G0816597300.01	14.2	2.436	-2.55113	1.87E-69	1.41E-69	14.05	15.06	13.49	2.76	2.28	2.27
OsR498G0816598100.01	7.396	1.756	-2.04804	5.48E-117	3.10E-117	6.35	7.97	7.87	1.75	1.81	1.71
OsR498G0816598800.01	12.366	28.346	1.175368	3.55E-231	1.32E-231	12	12.09	13.01	27.74	28.18	29.12
OsR498G0816599500.01	0.383	6.643	4.10362	3.95E-106	2.35E-106	0.44	0.25	0.46	7.02	6.97	5.94
OsR498G0816600000.01	141.37	54.086	-1.39064	0	0	146.36	140.1	137.7	54.01	54.9	53.35
OsR498G0816600400.01	11.54	1.546	-2.90234	3.88E-176	1.72E-176	11.83	11.7	11.09	1.62	1.55	1.47
OsR498G0816600800.01	125.67	40.79	-1.63115	0	0	130.34	120.96	125.7	41.58	39.24	41.55
OsR498G0816601500.01	18.773	199.33	3.629072	0	0	18.52	18.69	19.11	199.71	198.08	200.19
OsR498G0816602800.01	2.543	12.013	2.271887	7.79E-89	5.13E-89	2.47	2.32	2.84	12.56	13.41	10.07
OsR498G0816603500.01	248.55	92.24	-1.43489	0	0	254.73	241.36	249.6	93.37	92.99	90.36
OsR498G0816604000.01	0.113	0.38	1.762226	1.01E-07	1.93E-07	0.1	0.1	0.14	0.43	0.42	0.29
OsR498G0816606000.01	18.106	7.013	-1.37399	5.79E-47	5.36E-47	19.87	15.93	18.52	7.1	7	6.94
OsR498G0816613300.01	2.886	0.553	-2.37093	5.05E-22	6.55E-22	2.59	3.9	2.17	0.59	0.33	0.74
OsR498G0816613600.01	1876	353.64	-2.41452	0	0	1885.3	1904.7	1838	380.69	337.94	342.29
OsR498G0816615600.01	1.213	0.053	-4.6514	2.30E-36	2.39E-36	1.01	1.19	1.44	0.05	0.08	0.03
OsR498G0816616100.01	124.6	41.503	-1.59829	0	0	128.76	121.84	123.2	41.75	39.86	42.9
OsR498G0816616300.01	0.323	0	-5.0477	1.13E-05	2.39E-05	0.33	0.53	0.11	0	0	0
OsR498G0816616900.01	294.74	110.75	-1.15916	0	0	306.71	289.71	287.8	114.68	107.27	110.3
OsR498G0816617200.01	32.833	186.57	2.717579	0	0	31.97	33.07	33.46	186.39	184.08	189.25
OsR498G0816618000.01	0.32	1.893	2.545651	4.55E-22	5.89E-22	0.23	0.36	0.37	2	1.83	1.85
OsR498G0816630800.01	0.186	0.013	-3.99242	2.77E-06	5.66E-06	0.3	0.11	0.15	0	0.04	0
OsR498G0816631100.01	0.53	0.146	-1.83746	1.85E-07	3.56E-07	0.32	0.57	0.7	0.21	0.07	0.16
OsR498G0816631800.01	9.82	27.786	1.508699	3.79E-306	1.14E-306	9.44	9.82	10.2	26.63	28.59	28.14
OsR498G0816633600.01	1.493	0.356	-2.07572	5.14E-06	1.06E-05	1.7	1.91	0.87	0.35	0.6	0.12
OsR498G0916657400.01	12.136	32.06	1.393143	1.21E-201	4.90E-202	13.33	12.15	10.93	34.31	30.76	31.11
OsR498G0916674400.01	17.88	128.35	2.833322	0	0	18.13	16.34	19.17	131.57	128.29	125.19
OsR498G0916678000.01	0.336	0.01	-5.00463	2.30E-09	4.12E-09	0.49	0.19	0.33	0	0	0.03
OsR498G0916692600.01	80.366	588.44	2.840682	0	0	82.39	80.87	77.84	592.23	588.36	584.74
OsR498G0916693600.01	4.253	1.423	-1.5301	1.39E-08	2.56E-08	4.29	4.03	4.44	1.54	1.4	1.33
OsR498G0916693700.01	0.976	0.4	-1.29727	0.0002081	0.0004753	0.62	0.94	1.37	0.37	0.27	0.56
OsR498G0916716100.01	1.016	0	-5.66068	7.26E-08	1.38E-07	1.17	0.86	1.02	0	0	0
OsR498G0916723200.01	1.686	0.393	-2.17402	4.84E-21	6.40E-21	1.61	1.78	1.67	0.37	0.33	0.48
OsR498G0916727400.01	0.696	1.433	1.019582	0.0001676	0.0003803	0.99	0.43	0.67	1.13	1.23	1.94
OsR498G0916732700.01	0.163	0	-5.81822	1.42E-08	2.62E-08	0.12	0.14	0.23	0	0	0
OsR498G0916735500.01	1.373	0.63	-1.12463	0.0001249	0.000281	1.57	1.52	1.03	0.9	0.6	0.39
OsR498G0916736800.01	0.556	15.946	4.826574	0	0	0.49	0.4	0.78	16.58	16.69	14.57
OsR498G0916742000.01	17.55	9.323	-1.05072	1.53E-91	9.89E-92	17.17	16.74	18.74	9.27	8.93	9.77
OsR498G0916742900.01	6.623	1.853	-1.93078	1.25E-61	1.01E-61	6.76	6.16	6.95	2.04	1.67	1.85
OsR498G0916743300.01	3.046	13.816	2.181893	2.93E-105	1.76E-105	3.51	2.81	2.82	13.89	13.46	14.1
OsR498G0916743600.01	0.163	0.65	2.003234	6.16E-06	1.28E-05	0.15	0.2	0.14	0.64	0.76	0.55
OsR498G0916747900.01	2.4	6.343	1.397608	2.45E-21	3.22E-21	2.64	2.59	1.97	6.44	7.08	5.51

OsR498G0916749400.01	5.986	1.47	-2.01964	4.81E-51	4.27E-51	6.18	5.73	6.05	1.3	1.86	1.25
OsR498G0916756400.01	48.193	20.06	-1.27334	2.15E-207	8.59E-208	47.85	47.1	49.63	21.62	19	19.56
OsR498G0916769500.01	12.836	37.683	1.552408	4.60E-193	1.93E-193	13.02	12.07	13.42	40.03	36.21	36.81
OsR498G0916776200.01	84.146	35.616	-1.25083	2.98E-288	9.46E-289	85.41	82.53	84.5	35.71	36.12	35.02
OsR498G0916781000.01	14.46	198.3	3.689832	0	0	14.45	15	13.93	202.98	196.42	195.5
OsR498G0916782600.01	0.046	0.006	-2.96024	0.000243	0.0005585	0.07	0.02	0.05	0	0	0.02
OsR498G0916791700.01	0.846	0.233	-2.0477	3.06E-07	5.97E-07	1.21	0.44	0.89	0.14	0.29	0.27
OsR498G0916800100.01	16.176	7.916	-1.03103	1.36E-37	1.39E-37	17.35	15.83	15.35	7.02	7.91	8.82
OsR498G0916807400.01	7.856	3.8	-1.06531	1.50E-33	1.62E-33	8.28	7.17	8.12	3.72	4.13	3.55
OsR498G0916823600.01	0.903	0.17	-2.46274	6.52E-11	1.11E-10	1.11	0.95	0.65	0.08	0.22	0.21
OsR498G0916824900.01	0.796	0.29	-1.46274	3.65E-05	7.93E-05	0.88	0.88	0.63	0.19	0.39	0.29
OsR498G0916838900.01	11.496	26.923	1.212535	2.19E-131	1.16E-131	12.43	11.51	10.55	26.58	27.53	26.66
OsR498G0916843600.01	3.41	7.123	1.053156	5.82E-24	7.27E-24	3.03	3.5	3.7	6.95	6.55	7.87
OsR498G0916844700.01	0.986	0.336	-1.56114	1.72E-08	3.18E-08	1	1.02	0.94	0.33	0.55	0.13
OsR498G0916852900.01	0.673	0.136	-2.28217	1.96E-05	4.18E-05	1.14	0.49	0.39	0.27	0	0.14
OsR498G0916857600.01	7.973	2.656	-1.65566	7.70E-42	7.52E-42	8.05	8.17	7.7	2.61	2.69	2.67
OsR498G0916858200.01	1.316	0.25	-2.41967	4.19E-06	8.63E-06	1.66	1.37	0.92	0.25	0.13	0.37
OsR498G0916873200.01	1.643	5.246	1.700554	2.74E-58	2.27E-58	1.4	1.71	1.82	5.92	4.88	4.94
OsR498G0916877900.01	0.746	0.356	-1.21505	4.63E-07	9.10E-07	0.66	0.78	0.8	0.13	0.67	0.27
OsR498G0916878800.01	18.466	39.146	1.0796	2.62E-176	1.16E-176	19.88	17.73	17.79	38.02	39.15	40.27
OsR498G0916879300.01	13.47	6.27	-1.25218	1.52E-91	9.82E-92	12.7	13.54	14.17	6.37	6.52	5.92
OsR498G0916883200.01	17.05	8.456	-1.01362	3.83E-52	3.36E-52	16.34	17.96	16.85	8.16	9.11	8.1
OsR498G0916885600.01	7.98	0.203	-5.37528	4.32E-120	2.40E-120	7.9	9.17	6.87	0.37	0.12	0.12
OsR498G0916887300.01	2.486	0.816	-1.61066	7.24E-21	9.62E-21	2.73	2.03	2.7	0.91	0.79	0.75
OsR498G0916889000.01	0.07	0.006	-2.96024	0.000243	0.0005585	0.01	0.07	0.13	0.01	0	0.01
OsR498G0916892200.01	2.33	1.056	-1.15006	6.90E-12	1.14E-11	2.81	1.9	2.28	1.17	1.01	0.99
OsR498G0916897000.01	3.426	0.01	-8.39903	1.66E-70	1.25E-70	3.49	3.31	3.48	0.03	0	0
OsR498G0916897600.01	12.24	3.776	-1.6041	4.55E-93	2.91E-93	10.96	13.53	12.23	3.97	4.31	3.05
OsR498G0916899900.01	2.61	1.093	-1.26323	6.31E-14	9.82E-14	2.18	2.78	2.87	1.05	1.09	1.14
OsR498G0916906700.01	165.32	37.493	-2.1458	0	0	171.42	163.98	160.6	39.12	35.43	37.93
OsR498G0916907100.01	9.096	3.72	-1.24865	7.64E-63	6.08E-63	8.79	9.45	9.05	4.02	3.75	3.39
OsR498G0916914400.01	6.163	2.263	-1.45435	3.12E-28	3.62E-28	5	6.88	6.61	2.19	2.72	1.88
OsR498G0916917100.01	0.753	0.33	-1.19728	5.43E-05	0.0001192	0.86	0.5	0.9	0.31	0.36	0.32
OsR498G0916921200.01	3.06	1.02	-1.58827	1.71E-09	3.03E-09	3.65	3.38	2.15	1.01	1.03	1.02
OsR498G0916926100.01	1.42	0.333	-2.10529	1.53E-14	2.34E-14	1.37	1.09	1.8	0.19	0.35	0.46
OsR498G0916928900.01	7.073	3.133	-1.09681	5.06E-15	7.64E-15	7.17	6.77	7.28	3.52	2.75	3.13
OsR498G0916942000.01	3.926	1.153	-1.79251	7.15E-28	8.35E-28	3.71	3.95	4.12	1.04	1.23	1.19
OsR498G0916948000.01	7.19	2.013	-1.85659	3.08E-43	2.97E-43	7.56	7.67	6.34	1.82	2.18	2.04
OsR498G0917002400.01	0.046	0.133	1.534525	0.0005222	0.0012286	0.05	0.04	0.05	0.11	0.2	0.09
OsR498G0917003600.01	12.98	0.136	-6.59621	0	0	12.69	13.09	13.16	0.12	0.11	0.18
OsR498G0917004800.01	2.31	0.216	-3.44702	8.15E-81	5.66E-81	2.18	2.33	2.42	0.21	0.15	0.29
OsR498G0917012300.01	13.63	40.426	1.55753	0	0	13.88	13.72	13.29	39.17	40.36	41.75
OsR498G0917014900.01	5.123	1.38	-1.86538	6.42E-60	5.23E-60	4.37	5.23	5.77	1.28	1.39	1.47
OsR498G0917015400.01	7.9	3.526	-1.17071	1.68E-43	1.61E-43	8.54	7.91	7.25	2.96	3.96	3.66
OsR498G0917019700.01	80.876	179.01	1.148147	0	0	83.44	84.08	75.11	178.4	171.99	186.63
OsR498G0917039600.01	18.086	293.8	4.014012	0	0	18.51	18.24	17.51	299.36	301.79	280.24
OsR498G0917044700.01	139.3	65.74	-1.08738	0	0	135.9	143.8	138.2	63.57	65.69	67.96
OsR498G0917045000.01	7.29	2.553	-1.52303	2.02E-64	1.59E-64	7.57	6.28	8.02	2.43	3.33	1.9
OsR498G0917060400.01	17.683	6.726	-1.31048	2.41E-81	1.66E-81	19.08	17.18	16.79	6.54	6.82	6.82
OsR498G0917061100.01	0.356	10.42	4.855402	3.05E-204	1.23E-204	0.48	0.29	0.3	9.58	11.23	10.45
OsR498G0917061900.01	2.87	5.833	1.011746	1.62E-15	2.42E-15	2.84	2.72	3.05	6.37	6.15	4.98
OsR498G0917079600.01	5.036	11.53	1.192259	1.68E-114	9.62E-115	4.63	5.34	5.14	10.54	12.66	11.39
OsR498G0917083400.01	17.486	47.556	1.437135	4.78E-239	1.73E-239	19.25	16.14	17.07	50.56	45.94	46.17
OsR498G0917088600.01	8.206	32.143	1.990108	0	0	8.45	8.06	8.11	30.67	32.56	33.2
OsR498G0917090600.01	8.923	27.716	1.630825	0	0	8.77	8.98	9.02	27.06	27.52	28.57
OsR498G0917094200.01	0.68	0.063	-3.76759	3.52E-10	6.13E-10	0.85	0.8	0.39	0.05	0.05	0.09
OsR498G0917137100.01	3.833	1.066	-1.85788	1.06E-52	9.26E-53	3.24	4.62	3.64	0.72	1.34	1.14
OsR498G0917144600.01	2.43	0.743	-1.73396	7.27E-11	1.24E-10	2.96	1.74	2.59	0.92	1.09	0.22
OsR498G0917148400.01	4.273	12.05	1.432993	4.26E-93	2.73E-93	3.49	4.47	4.86	12.71	11.67	11.77
OsR498G0917151200.01	3.163	50.736	4.01649	0	0	3	3.09	3.4	49.07	52.31	50.83
OsR498G0917156200.01	0.516	1.39	1.415269	4.54E-08	8.54E-08	0.63	0.48	0.44	1.15	1.8	1.22
OsR498G0917156400.01	0.346	0	-5.28217	2.04E-06	4.14E-06	0.75	0.2	0.09	0	0	0
OsR498G0917159900.01	0.453	5.116	3.471933	3.39E-72	2.50E-72	0.4	0.34	0.62	5.59	5.14	4.62
OsR498G0917162200.01	0.313	2.146	2.755967	2.94E-23	3.72E-23	0.36	0.19	0.39	2.34	2.13	1.97
OsR498G0917162400.01	0.04	0.936	4.97522	1.16E-37	1.19E-37	0.02	0.05	0.05	0.84	1.16	0.81
OsR498G0917162500.01	9.283	45.32	2.265959	0	0	9.18	9.58	9.09	45.26	45.42	45.28
OsR498G0917163000.01	6.876	14.733	1.093375	5.64E-123	3.09E-123	6.54	6.48	7.61	15.01	14.45	14.74
OsR498G0917172500.01	1.176	0.06	-4.33875	2.53E-24	3.14E-24	1.22	1.17	1.14	0.14	0.04	0
OsR498G0917174700.01	3.606	0.083	-4.76759	4.07E-08	7.64E-08	4.1	3.18	3.54	0	0.25	0

OsR498G0917182100.01	8.626	72.956	3.060114	0	0	8.11	8.92	8.85	74.85	72.33	71.69
OsR498G0917182700.01	0.223	1.163	2.262152	3.74E-26	4.49E-26	0.22	0.2	0.25	1.18	1.25	1.06
OsR498G0917187600.01	2.606	1.106	-1.24564	6.63E-08	1.25E-07	2.6	2.45	2.77	1.09	1.26	0.97
OsR498G0917189500.01	1.716	0.59	-1.78897	5.20E-23	6.61E-23	1.76	2.02	1.37	0.48	0.77	0.52
OsR498G0917193100.01	2.436	0.896	-1.42235	4.36E-19	6.02E-19	2.28	2.65	2.38	0.93	0.75	1.01
OsR498G0917194500.01	0.423	0.17	-1.35617	0.0001002	0.0002239	0.38	0.37	0.52	0.21	0.11	0.19
OsR498G0917195700.01	1.486	0.59	-1.33464	6.05E-10	1.06E-09	1.65	1.4	1.41	0.61	0.5	0.66
OsR498G0917197000.01	7.653	2.05	-1.91311	3.12E-28	3.62E-28	8.34	7.62	7	1.82	2.48	1.85
OsR498G0917198200.01	0.06	0.39	2.717832	1.08E-06	2.16E-06	0.04	0.04	0.1	0.43	0.26	0.48
OsR498G0917198400.01	0.07	0.963	3.783921	1.41E-15	2.11E-15	0.04	0.09	0.08	1.23	0.79	0.87
OsR498G0917198600.01	29.02	1.963	-4.19476	0	0	29.17	27.7	30.19	1.78	1.63	2.48
OsR498G0917202600.01	0.013	0.116	3.127223	9.61E-05	0.0002145	0.04	0	0	0.04	0.08	0.23
OsR498G0917203100.01	8.146	1.273	-2.71783	1.92E-64	1.51E-64	7.36	8.61	8.47	1.09	1.34	1.39
OsR498G0917203700.01	5.46	1.423	-1.90502	6.29E-96	3.96E-96	5.62	5.36	5.4	1.29	1.22	1.76
OsR498G0917205200.01	0.076	0.296	1.93788	5.79E-06	1.20E-05	0.08	0.09	0.06	0.34	0.38	0.17
OsR498G0917206700.01	2528.5	1163.4	-1.12491	0	0	2560.5	2500	2525	1258	1144.5	1087.8
OsR498G0917209900.01	2.57	18.5	2.839735	0	0	2.73	2.16	2.82	19.52	17.66	18.32
OsR498G0917216200.01	0.416	0.043	-3.5452	3.01E-13	4.76E-13	0.53	0.35	0.37	0	0.08	0.05
OsR498G0917219200.01	0.16	0	-5.5452	2.18E-07	4.22E-07	0.14	0.15	0.19	0	0	0
OsR498G0917223000.01	0.2	0.036	-2.49629	1.03E-05	2.18E-05	0.17	0.23	0.2	0.02	0.07	0.02
OsR498G0917227800.01	3.46	0.253	-3.77571	5.52E-108	3.26E-108	3.16	3.1	4.12	0.18	0.3	0.28
OsR498G0917239600.01	0	0.13	5.306547	2.27E-06	4.62E-06	0	0	0	0.06	0.17	0.16
OsR498G0917240000.01	13.37	2.55	-3.05532	0	0	13.33	14.08	12.7	2.83	2.18	2.64
OsR498G0917242100.01	28.06	0.126	-7.93106	0	0	26.61	29.5	28.07	0.17	0.13	0.08
OsR498G0917242800.01	14.603	4.366	-1.74959	6.18E-108	3.65E-108	15.62	13.9	14.29	4.32	4.75	4.03
OsR498G0917245900.01	3.973	14.016	1.821179	2.06E-143	1.03E-143	3.33	4.49	4.1	14.06	13.36	14.63
OsR498G0917246300.01	0	0.173	4.499192	0.0003163	0.0007323	0	0	0	0.14	0.24	0.14
OsR498G0917247600.01	18.13	45.333	1.24945	2.00E-165	9.17E-166	18.63	18.27	17.49	46.17	43.56	46.27
OsR498G0917249500.01	11.366	24.75	1.119487	5.90E-186	2.53E-186	11.92	10.97	11.21	23.36	25.49	25.4
OsR498G0917251500.01	0.006	0.1	3.624723	0.0004041	0.0009439	0	0	0.02	0.07	0.08	0.15
OsR498G0917251900.01	31.213	104.18	1.71892	0	0	31.01	32.04	30.59	104.21	103.77	104.57
OsR498G0917253000.01	0.313	0.873	1.400758	3.10E-10	5.39E-10	0.23	0.37	0.34	1.07	0.73	0.82
OsR498G0917255100.01	0.093	1.886	4.337441	4.75E-28	5.53E-28	0.09	0.1	0.09	2.12	1.9	1.64
OsR498G0917260400.01	0.016	1.673	6.64709	1.80E-47	1.65E-47	0.05	0	0	1.82	1.39	1.81
OsR498G0917267800.01	0.023	0.656	4.993956	4.24E-09	7.66E-09	0	0	0.07	0.64	0.63	0.7
OsR498G0917268500.01	115.66	33.143	-1.8124	0	0	125.91	111.08	110	37.98	30.37	31.08
OsR498G0917274700.01	0.033	0.563	4.127223	1.87E-05	4.00E-05	0.1	0	0	0.49	0.7	0.5
OsR498G0917276700.01	94.57	277.82	1.551817	0	0	91.8	95.22	96.69	299.89	268.71	264.87
OsR498G0917279900.01	18.45	8.23	-1.17489	4.02E-75	2.89E-75	18.9	17.6	18.85	8.13	8.1	8.46
OsR498G0917282000.01	4.98	18.266	1.878479	1.01E-137	5.22E-138	4.91	4.71	5.32	17.15	20.49	17.16
OsR498G0917283500.01	2.34	24.503	3.361011	0	0	2.17	2.71	2.14	26.68	24.3	22.53
OsR498G0917285100.01	1.756	28.96	4.044993	0	0	1.83	1.91	1.53	31.99	28.06	26.83
OsR498G0917285900.01	0.796	2.55	1.665202	9.20E-23	1.18E-22	0.73	0.67	0.99	2.19	2.79	2.67
OsR498G0917291500.01	2.616	5.926	1.139897	8.56E-18	1.21E-17	2.11	2.61	3.13	6.42	5.07	6.29
OsR498G0917296600.01	1.533	3.206	1.089126	4.55E-30	5.13E-30	1.39	1.77	1.44	2.93	3.25	3.44
OsR498G0917303400.01	14.053	5.74	-1.27901	3.88E-125	2.10E-125	13.4	14.44	14.32	5.63	6.02	5.57
OsR498G0917313200.01	0.836	4.636	2.462643	4.17E-111	2.42E-111	0.94	0.72	0.85	4.38	4.64	4.89
OsR498G0917313700.01	17.34	4.133	-2.09486	3.60E-209	1.43E-209	17.89	16.43	17.7	3.49	4.27	4.64
OsR498G0917315900.01	0.416	0.146	-1.51824	1.66E-05	3.54E-05	0.47	0.49	0.29	0.07	0.22	0.15
OsR498G0917320700.01	4.1	1.656	-1.1513	2.39E-26	2.86E-26	4.03	4.22	4.05	1.6	1.78	1.59
OsR498G0917326800.01	1.976	0.74	-1.41543	1.28E-12	2.07E-12	1.93	1.91	2.09	0.84	0.46	0.92
OsR498G0917327200.01	7.146	3.18	-1.17545	1.57E-14	2.41E-14	6.3	8.13	7.01	4.01	3.28	2.25
OsR498G0917335000.01	0	0.47	6.084154	1.05E-09	1.85E-09	0	0	0	0.46	0.48	0.47
OsR498G0917339500.01	0.19	7.263	5.249213	3.70E-63	2.94E-63	0.24	0.17	0.16	7.8	7.15	6.84
OsR498G0917355600.01	0.013	0.106	3.178923	1.68E-05	3.59E-05	0.04	0	0	0.09	0.14	0.09
OsR498G0917367500.01	20.313	62.023	1.607121	0	0	21.53	19.74	19.67	61.81	62.19	62.07
OsR498G0917377700.01	53.88	9.55	-2.51292	0	0	57.01	51.08	53.55	8.49	10.32	9.84
OsR498G0917379000.01	28.003	50.86	1.096699	3.96E-221	1.52E-221	29.88	25.71	28.42	51.48	50.31	50.79
OsR498G0917389500.01	3.446	9.923	1.522295	3.37E-45	3.17E-45	2.26	4.28	3.8	9.73	10.6	9.44
OsR498G0917393900.01	2.49	0.463	-2.42839	6.20E-78	4.39E-78	2.59	2.47	2.41	0.47	0.42	0.5
OsR498G0917394200.01	2.12	0.803	-1.3974	7.89E-17	1.14E-16	2.05	2.28	2.03	0.74	0.99	0.68
OsR498G0917404400.01	29.733	1.486	-4.32783	0	0	27.8	29.08	32.32	1.72	1.66	1.08
OsR498G0917404500.01	0.15	0	-4.21272	0.0009496	0.0022826	0.31	0	0.14	0	0	0
OsR498G0917404800.01	0.52	0.163	-2.37999	2.66E-09	4.77E-09	0.29	0.91	0.36	0	0	0.49
OsR498G0917404900.01	31.926	0.913	-5.12971	0	0	29.2	33.04	33.54	1.04	1.29	0.41
OsR498G0917406000.01	0.436	10.253	4.565687	1.55E-278	5.03E-279	0.27	0.59	0.45	11.27	10.25	9.24
OsR498G0917407400.01	73.796	346.98	2.191649	0	0	74.27	75.48	71.64	369.66	336.33	334.95
OsR498G0917410000.01	85.69	272.54	1.665983	0	0	84.09	86.96	86.02	279.9	274.38	263.33
OsR498G0917411600.01	8.673	21.883	1.333395	4.17E-117	2.35E-117	8.75	9.2	8.07	23	20.98	21.67

OsR498G0917411900.01	0.84	0.326	-1.36078	1.01E-05	2.13E-05	0.87	0.92	0.73	0.32	0.2	0.46
OsR498G0917412600.01	18	6.536	-1.46296	0	0	16.92	18.8	18.28	6.15	6.52	6.94
OsR498G0917415600.01	1.493	0.426	-1.83471	6.18E-10	1.08E-09	1.66	1.59	1.23	0.37	0.59	0.32
OsR498G0917417400.01	1.436	0.436	-1.73785	4.28E-12	7.02E-12	1.51	1.24	1.56	0.33	0.72	0.26
OsR498G0917420500.01	0.216	0.866	1.987293	0.000135	0.0003042	0.28	0.19	0.18	0.57	1.16	0.87
OsR498G0917421600.01	1.113	4.416	1.946651	7.80E-05	0.000173	0.83	1.32	1.19	4.02	5.24	3.99
OsR498G0917422600.01	31.863	69.97	1.128378	6.01E-187	2.57E-187	31.04	32.65	31.9	71.09	69.49	69.33
OsR498G0917427900.01	1.586	13.196	3.069148	3.41E-232	1.27E-232	1.37	1.95	1.44	14.8	12.13	12.66
OsR498G0917428200.01	0	1.526	6.847115	7.65E-15	1.16E-14	0	0	0	2.02	1.41	1.15
OsR498G0917434200.01	1.71	0.7	-1.42839	3.87E-07	7.59E-07	1.02	1.79	2.32	1.25	0.48	0.37
OsR498G0917438000.01	8.28	659.71	6.112553	0	0	9.3	7.48	8.06	702.16	672.85	604.11
OsR498G0917438600.01	3.163	0.09	-5.15006	2.21E-35	2.33E-35	3.3	3.13	3.06	0.07	0	0.2
OsR498G0917439300.01	226.45	79.026	-1.52904	0	0	230.47	228.31	220.6	81.5	74.22	81.36
OsR498G0917441500.01	28.023	9.266	-1.65834	1.98E-157	9.36E-158	28.18	27.95	27.94	8.78	9.31	9.71
OsR498G0917445700.01	2.196	0.403	-2.36706	3.84E-24	4.78E-24	2.38	2.13	2.08	0.55	0.25	0.41
OsR498G0917447400.01	1.266	0.013	-6.4838	5.80E-23	7.38E-23	1.12	1.08	1.6	0.04	0	0
OsR498G0917449000.01	40.106	6.4	-2.609	0	0	42.45	39.55	38.32	6.53	6.06	6.61
OsR498G0917451500.01	0	0.396	5.897741	9.23E-09	1.69E-08	0	0	0	0.57	0.25	0.37
OsR498G0917453500.01	42.746	118.17	1.489735	0	0	44.43	41.88	41.93	123.34	115.45	115.72
OsR498G0917456300.01	0.93	4.06	2.111804	1.51E-41	1.48E-41	1.01	0.74	1.04	4.66	4.15	3.37
OsR498G0917457600.01	1.743	4.796	1.452542	2.82E-16	4.14E-16	1.82	1.84	1.57	4.52	4.45	5.42
OsR498G0917460800.01	1.206	0.416	-1.5452	2.56E-08	4.77E-08	1.31	1.07	1.24	0.34	0.59	0.32
OsR498G0917462400.01	4.393	28.356	2.687498	0	0	4.11	4.77	4.3	30.52	26.78	27.77
OsR498G0917463600.01	2.17	17.483	3.010506	2.72E-183	1.18E-183	1.96	1.91	2.64	17.73	15.89	18.83
OsR498G0917465900.01	1.25	0.243	-2.3865	6.55E-13	1.05E-12	0.87	1.41	1.47	0.18	0.14	0.41
OsR498G0917467800.01	187.48	41.813	-2.17149	0	0	185.9	187.53	189	43.82	41.34	40.28
OsR498G0917467900.01	10.11	1.403	-2.85683	8.69E-222	3.32E-222	10.58	10.57	9.18	1.5	1.35	1.36
OsR498G0917469500.01	0.186	1.946	3.353177	2.21E-33	2.39E-33	0.2	0.1	0.26	2.07	2	1.77
OsR498G0917469700.01	0.1	0.64	2.683616	1.50E-05	3.19E-05	0.07	0.16	0.07	0.76	0.7	0.46
OsR498G0917471700.01	1.793	0.82	-1.13568	1.06E-07	2.02E-07	1.83	1.97	1.58	0.75	0.73	0.98
OsR498G0917473300.01	1148.4	377.19	-1.61564	0	0	1151.4	1139.6	1154	377.67	367.94	385.96
OsR498G0917475600.01	0.133	0.02	-2.69721	0.0001045	0.0002337	0.12	0.15	0.13	0.02	0.04	0
OsR498G0917476600.01	0.21	0.553	1.361688	0.0007094	0.0016883	0.23	0.14	0.26	0.51	0.34	0.81
OsR498G0917480900.01	10.216	2.523	-2.04472	4.37E-283	1.40E-283	9.8	10.19	10.66	2.12	2.8	2.65
OsR498G0917483300.01	5.29	14.55	1.463272	3.18E-91	2.06E-91	5.55	4.9	5.42	14.47	15.51	13.67
OsR498G0917483900.01	2.74	11.006	1.999794	2.95E-77	2.09E-77	2.68	2.82	2.72	10.89	10.4	11.73
OsR498G0917484800.01	1.413	3.303	1.216309	1.56E-21	2.04E-21	1.43	1.31	1.5	3.2	3.9	2.81
OsR498G0917485600.01	1.92	0.926	-1.02841	9.73E-22	1.27E-21	1.9	2.03	1.83	0.84	0.88	1.06
OsR498G0917486200.01	0.013	0.183	3.7402	4.34E-07	8.52E-07	0.02	0.02	0	0.23	0.15	0.17
OsR498G0917486500.01	2.026	0.44	-2.22327	1.75E-07	3.38E-07	2.78	1.47	1.83	0.39	0.8	0.13
OsR498G0917488500.01	48.263	231.16	2.25265	0	0	48.06	46.94	49.79	243.84	226.18	223.45
OsR498G0917489000.01	1.426	17.916	3.703415	4.45E-282	1.43E-282	1.54	1.61	1.13	18.27	17.65	17.83
OsR498G0917489200.01	0.183	0.023	-3.5452	0.0005186	0.0012198	0.08	0.12	0.35	0.07	0	0
OsR498G0917490000.01	7.206	0.133	-5.7178	2.18E-127	1.17E-127	6.75	7.27	7.6	0.12	0.2	0.08
OsR498G0917491900.01	1.086	0.166	-2.73785	1.97E-23	2.49E-23	0.97	1.18	1.11	0.27	0.09	0.14
OsR498G0917499400.01	4.7	10.943	1.212227	3.30E-96	2.07E-96	4.28	4.72	5.1	10.3	10.85	11.68
OsR498G0917508100.01	0.04	3.156	6.306547	2.18E-129	1.16E-129	0.02	0.07	0.03	3.15	3.36	2.96
OsR498G0917508700.01	5.666	36.363	2.790675	0	0	6.16	5.28	5.56	35.21	36.29	37.59
OsR498G0917512100.01	0.49	0	-5.41967	6.63E-07	1.31E-06	0.53	0.42	0.52	0	0	0
OsR498G0917513200.01	3.13	1.406	-1.16306	1.65E-07	3.19E-07	2.84	2.91	3.64	1.73	1.29	1.2
OsR498G0917515100.01	0.453	3.456	2.938831	1.75E-47	1.61E-47	0.36	0.61	0.39	4.58	2.95	2.84
OsR498G0917518300.01	3.246	14.23	2.130393	1.28E-86	8.50E-87	2.99	3.76	2.99	16.22	12.49	13.98
OsR498G0917518600.01	0.216	2.793	3.666438	6.36E-31	7.09E-31	0.23	0.06	0.36	2.83	2.71	2.84
OsR498G0917519700.01	98.9	22.65	-2.13747	0	0	98.81	93.25	104.6	22.84	22.73	22.38
OsR498G0917520500.01	160.7	22.763	-2.78819	0	0	168.2	156.67	157.2	23.12	21.08	24.09
OsR498G0917523000.01	0.413	1.183	1.554905	5.03E-08	9.47E-08	0.34	0.49	0.41	1.29	0.9	1.36
OsR498G0917527500.01	3.496	19.89	2.509617	0	0	3.25	3.79	3.45	19.61	19.72	20.34
OsR498G0917527700.01	44.226	103.48	1.200384	0	0	47.82	43.08	41.78	103.11	105	102.33
OsR498G0917531100.01	11.92	1.716	-2.80534	1.07E-108	6.30E-109	11.34	12.6	11.82	1.42	1.69	2.04
OsR498G0917532800.01	9.013	24.32	1.422815	7.98E-77	5.68E-77	8.96	8.89	9.19	25.2	24.67	23.09
OsR498G0917535200.01	40.066	16.726	-1.26681	9.52E-139	4.88E-139	39.77	39.02	41.41	16.87	17.35	15.96
OsR498G0917535900.01	8.103	2.956	-1.89916	3.72E-144	1.86E-144	6.59	9.42	8.3	3.17	2.89	2.81
OsR498G0917537200.01	1.52	3.39	1.149059	8.15E-13	1.31E-12	1.51	1.63	1.42	2.87	3.8	3.5
OsR498G0917537500.01	6.59	13.086	1.030509	6.19E-57	5.20E-57	6.51	6.74	6.52	13.06	13.31	12.89
OsR498G0917537700.01	2.64	0.903	-1.55247	2.56E-16	3.76E-16	2.77	2.7	2.45	1.01	1.08	0.62
OsR498G0917538800.01	3.316	1.586	-1.04384	1.79E-16	2.62E-16	3.69	3.43	2.83	1.59	1.56	1.61
OsR498G0917542200.01	54.246	110.64	1.035673	0	0	55.81	53.47	53.46	104.73	111.93	115.25
OsR498G0917545900.01	39.823	160.05	2.007771	0	0	37.28	39.81	42.38	167.48	154.63	158.03
OsR498G0917547300.01	0.226	0.01	-3.86713	8.60E-05	0.0001913	0.29	0.33	0.06	0.03	0	0

OsR498G0917550700.01	32.306	458.1	3.827861	0	0	33.79	30.97	32.16	469.19	453.04	452.07
OsR498G0917555100.01	1.626	0.166	-3.28217	2.26E-30	2.54E-30	1.86	1.53	1.49	0.21	0.07	0.22
OsR498G0917557600.01	1.523	104.76	6.095084	0	0	1.2	1.57	1.8	111.11	102.99	100.18
OsR498G0917561300.01	0.52	0.103	-2.35256	4.05E-07	7.94E-07	0.52	0.54	0.5	0.19	0.08	0.04
OsR498G0917566900.01	30.733	3.676	-3.06256	0	0	30.21	31.1	30.89	3.67	3.54	3.82
OsR498G0917567800.01	1.63	0.506	-1.65483	1.20E-11	1.99E-11	1.11	2.11	1.67	0.59	0.45	0.48
OsR498G0917570000.01	0.173	0.906	2.380797	7.24E-12	1.20E-11	0.24	0.18	0.1	0.91	0.91	0.9
OsR498G0917576100.01	22.036	7.996	-1.45027	3.74E-125	2.03E-125	24.11	20.71	21.29	7.23	8.73	8.03
OsR498G0917580500.01	19.153	2.393	-3.00265	0	0	19.7	18.38	19.38	2.59	2.13	2.46
OsR498G0917581500.01	8.876	0.77	-3.50567	5.27E-83	3.59E-83	9.27	9.24	8.12	0.6	1.05	0.66
OsR498G0917582600.01	4.513	2.24	-1.02869	1.95E-27	2.29E-27	4.21	4.84	4.49	2.02	2.35	2.35
OsR498G0917586800.01	1.373	0.343	-1.98659	1.75E-12	2.84E-12	1.37	1.54	1.21	0.85	0.18	0
OsR498G0917587500.01	1.753	0.28	-2.63009	6.76E-12	1.11E-11	1.63	2.34	1.29	0.3	0.23	0.31
OsR498G0917588300.01	6.51	0.483	-3.75944	2.34E-102	1.43E-102	6.47	6.69	6.37	0.38	0.51	0.56
OsR498G0917589100.01	17.686	6.226	-1.5354	2.09E-127	1.12E-127	18.49	16.77	17.8	6.07	6.04	6.57
OsR498G0917591100.01	25.986	5.68	-2.2295	2.97E-259	1.02E-259	26.09	25.04	26.83	5.71	5.62	5.71
OsR498G0917592100.01	2.733	0.193	-3.83939	1.42E-22	1.82E-22	2.79	2.1	3.31	0.33	0.08	0.17
OsR498G0917592600.01	7.323	69.54	3.243308	0	0	7.89	7.12	6.96	69.46	67.86	71.3
OsR498G0917593000.01	14.073	6.44	-1.11666	1.81E-85	1.22E-85	14.76	12.87	14.59	5.9	6.81	6.61
OsR498G0917593400.01	0.303	0.08	-1.91444	0.0005351	0.0012602	0.36	0.17	0.38	0	0.2	0.04
OsR498G0917593800.01	12.223	187.89	3.947358	0	0	12.25	12.54	11.88	190.56	188.53	184.59
OsR498G0917594200.01	7.956	22.31	1.426615	9.60E-132	5.06E-132	8.05	8.09	7.73	21.92	21.39	23.62
OsR498G0917595200.01	6.76	2.696	-1.33194	2.02E-38	2.04E-38	6.72	7.06	6.5	2.14	3.03	2.92
OsR498G0917598500.01	7.773	93.47	3.579697	0	0	7.23	8.14	7.95	96.26	94.81	89.34
OsR498G0917599400.01	0.16	0.466	1.549955	7.84E-09	1.43E-08	0.14	0.14	0.2	0.45	0.56	0.39
OsR498G0917604800.01	6.023	16.993	1.485786	1.48E-78	1.04E-78	6.05	5.31	6.71	18.52	17.12	15.34
OsR498G0917605400.01	3.21	0.73	-2.14958	4.92E-23	6.25E-23	2.93	3.23	3.47	0.63	0.97	0.59
OsR498G0917606100.01	0.08	0.006	-3.41967	0.0009442	0.002269	0.09	0.13	0.02	0.02	0	0
OsR498G0917608700.01	3.263	100.71	5.004601	0	0	3.63	2.84	3.32	102.77	103.01	96.36
OsR498G0917613600.01	0.016	1.136	5.946651	3.38E-16	4.97E-16	0	0	0.05	0.67	1.38	1.36
OsR498G0917614700.01	0.123	0.01	-3.76759	0.0001564	0.0003541	0.03	0.22	0.12	0.03	0	0
OsR498G0917615600.01	17.806	3.313	-2.43117	1.07E-92	6.85E-93	18.79	17.66	16.97	4.32	2.34	3.28
OsR498G0917615700.01	0.1	0	-4.5452	0.0002138	0.0004889	0.1	0.08	0.12	0	0	0
OsR498G0917615900.01	161.62	72.766	-1.17632	9.06E-265	3.05E-265	164.44	153.84	166.6	73.68	73.55	71.07
OsR498G0917619800.01	0.38	1.06	1.480021	2.11E-06	4.28E-06	0.26	0.53	0.35	0.97	0.83	1.38
OsR498G0917621300.01	8.71	1.893	-2.20774	3.30E-94	2.10E-94	9.08	9.13	7.92	1.29	2.05	2.34
OsR498G0917622100.01	51.11	15.453	-1.85379	9.25E-290	2.92E-290	51.71	50.8	50.82	17.17	15.71	13.48
OsR498G0917622300.01	18.603	53.193	1.512887	1.23E-244	4.39E-245	17.42	20.37	18.02	57.46	51.34	50.78
OsR498G0917622500.01	0.856	0.4	-1.09774	0.0004488	0.0010511	0.89	0.73	0.95	0.62	0.29	0.29
OsR498G0917623600.01	1.023	0	-7.35256	3.37E-20	4.54E-20	1.24	0.95	0.88	0	0	0
OsR498G0917625800.01	2.606	12.203	2.219084	1.34E-85	9.03E-86	2.5	2.46	2.86	13.56	11.69	11.36
OsR498G0917626000.01	0.323	0.706	1.139296	0.0008198	0.0019603	0.5	0.38	0.09	0.6	0.62	0.9
OsR498G0917626300.01	0.393	1.963	2.375047	2.31E-30	2.59E-30	0.29	0.42	0.47	1.61	2.09	2.19
OsR498G0917629000.01	0.146	0.04	-1.80799	6.46E-05	0.0001426	0.19	0.17	0.08	0.05	0.01	0.06
OsR498G0917640700.01	13.92	41.363	1.531927	1.40E-133	7.31E-134	14.33	12.84	14.59	41.91	41.13	41.05
OsR498G0917641000.01	18.826	4.136	-2.13054	3.14E-299	9.65E-300	18.56	19.7	18.22	4.05	4.14	4.22
OsR498G0917645800.01	3.833	1.086	-1.82585	1.79E-36	1.86E-36	4.08	3.86	3.56	1.23	1.24	0.79
OsR498G0917648500.01	113.67	288.71	1.361147	0	0	117.38	110.91	112.7	302.08	283.31	280.74
OsR498G0917648800.01	0.173	1.47	3.084154	5.06E-19	6.99E-19	0.09	0.22	0.21	1.31	1.76	1.34
OsR498G0917649000.01	15.21	33.696	1.306926	1.59E-138	8.19E-139	15.27	14.89	15.47	34.61	32.78	33.7
OsR498G0917651200.01	0.596	11.09	4.208048	7.37E-194	3.08E-194	0.44	0.5	0.85	11.64	10.82	10.81
OsR498G0917656700.01	12.76	2.396	-2.42667	2.00E-177	8.81E-178	13.29	12.61	12.38	2.65	2.42	2.12
OsR498G0917660200.01	10.853	3.233	-1.74982	1.03E-74	7.44E-75	11.15	10.41	11	4.01	2.58	3.11
OsR498G0917661300.01	1.8	0.303	-2.03481	7.08E-08	1.34E-07	1.71	1.85	1.84	0.68	0.23	0
OsR498G0917661600.01	24.043	5.436	-2.14966	0	0	23.64	23.42	25.07	5.45	5.04	5.82
OsR498G0917665300.01	0.486	23.963	5.623797	0	0	0.65	0.51	0.3	23.97	23.64	24.28
OsR498G0917668500.01	0.796	0.246	-1.85801	1.68E-07	3.25E-07	0.66	0.58	1.15	0.3	0.13	0.31
OsR498G0917669900.01	1.356	0.193	-2.81573	8.95E-11	1.53E-10	2.24	0.95	0.88	0	0.07	0.51
OsR498G0917677100.01	27.613	176.97	2.667818	0	0	28.14	26.21	28.49	178.91	173.59	178.4
OsR498G0917677600.01	2.06	50.21	4.604772	0	0	1.76	2.36	2.06	50.19	51.07	49.37
OsR498G0917678200.01	6.73	2.67	-1.20061	5.31E-52	4.66E-52	6.98	6.4	6.81	2.84	2.74	2.43
OsR498G0917678300.01	4.31	1.363	-1.67138	2.89E-18	4.06E-18	4.39	3.87	4.67	1.62	1.41	1.06
OsR498G0917683100.01	0.266	0.876	1.710452	3.01E-15	4.53E-15	0.24	0.39	0.17	0.71	0.94	0.98
OsR498G0917683800.01	0.873	0.26	-1.74451	1.71E-12	2.76E-12	0.78	1.03	0.81	0.3	0.18	0.3
OsR498G0917684000.01	1.546	8.89	2.53145	4.04E-158	1.90E-158	1.51	1.56	1.57	9.5	8	9.17
OsR498G0917684200.01	46.373	99.19	1.093149	0	0	46.24	45.95	46.93	96.36	100.58	100.63
OsR498G0917686300.01	5.056	1.39	-1.85554	1.41E-62	1.12E-62	5.53	5.11	4.53	1.58	1.39	1.2
OsR498G0917689300.01	5.833	2.003	-1.53033	5.29E-43	5.11E-43	6.13	5.3	6.07	2.13	1.95	1.93
OsR498G0917694300.01	1.93	0.1	-4.19119	1.10E-37	1.12E-37	1.81	1.89	2.09	0.03	0.1	0.17

OsR498G0917697100.01	0.763	0.12	-2.68013	2.88E-18	4.04E-18	0.81	0.85	0.63	0.08	0.17	0.11
OsR498G0917697300.01	1471.9	420.51	-1.81799	0	0	1553.5	1410.8	1451	466.72	411.35	383.46
OsR498G0917698300.01	0.176	0.66	1.893909	8.80E-16	1.31E-15	0.19	0.17	0.17	0.62	0.7	0.66
OsR498G0917700000.01	10.266	1.993	-2.36832	1.39E-89	9.10E-90	11.31	10.7	8.79	1.94	1.82	2.22
OsR498G0917700900.01	1.556	5.233	1.748711	1.91E-39	1.91E-39	1.32	1.83	1.52	5.66	4.88	5.16
OsR498G0917701900.01	0.11	0.54	2.304899	3.67E-13	5.81E-13	0.12	0.12	0.09	0.42	0.46	0.74
OsR498G0917703700.01	0.32	4.843	3.894353	5.38E-132	2.83E-132	0.32	0.18	0.46	5.26	5	4.27
OsR498G0917707100.01	3.306	13.116	1.973872	1.87E-46	1.74E-46	3.35	3.24	3.33	15.97	11.13	12.25
OsR498G0917713300.01	12.766	241.94	4.246168	0	0	11.37	14.13	12.8	252	234.66	239.17
OsR498G0917714500.01	9.193	49.773	2.430657	0	0	9.6	9.27	8.71	51.91	50.86	46.55
OsR498G0917716900.01	1.063	2.996	1.467181	3.61E-07	7.07E-07	1.32	0.81	1.06	2.16	3.6	3.23
OsR498G0917717300.01	41.263	112.89	1.448869	0	0	42.52	41.95	39.32	116.1	112.62	109.94
OsR498G0917720000.01	0.563	0.243	-1.42737	7.57E-08	1.44E-07	0.51	0.55	0.63	0.24	0.23	0.26
OsR498G0917722500.01	0.1	0.233	2.110149	0.0003986	0.0009301	0.12	0.12	0.06	0.27	0.25	0.18
OsR498G0917724200.01	0.056	0.866	3.993956	5.24E-15	7.93E-15	0	0.09	0.08	0.8	0.9	0.9
OsR498G0917725400.01	14.953	41.02	1.488793	0	0	14.48	15.56	14.82	41.49	40.01	41.56
OsR498G0917732800.01	2.986	35.376	3.566824	0	0	2.97	2.62	3.37	34.88	35.8	35.45
OsR498G0917733200.01	7.45	24.3	1.702566	3.72E-259	1.27E-259	7.64	7.07	7.64	24.01	23.82	25.07
OsR498G0917733800.01	4.05	1.3	-1.64941	1.30E-24	1.60E-24	3.77	3.55	4.83	1.64	1.11	1.15
OsR498G0917734800.01	108.87	13.226	-3.03833	0	0	109.2	109.88	107.5	12.72	13.61	13.35
OsR498G0917736100.01	0.376	2.026	2.425582	3.89E-39	3.91E-39	0.34	0.36	0.43	2.13	2.1	1.85
OsR498G0917737100.01	0.236	24.743	6.705742	6.24E-262	2.12E-262	0.26	0.2	0.25	25.7	24.38	24.15
OsR498G0917737700.01	1.723	0.746	-1.221	1.16E-14	1.77E-14	1.33	2.01	1.83	0.92	0.64	0.68
OsR498G0917738400.01	6.03	25.103	2.042662	0	0	6.09	6.25	5.75	25.29	25.69	24.33
OsR498G0917739300.01	0.913	1.98	1.065295	5.99E-09	1.09E-08	1.04	0.82	0.88	1.86	2.02	2.06
OsR498G0917742800.01	3.823	0.306	-3.64474	9.85E-37	1.02E-36	3.87	3.8	3.8	0.19	0.27	0.46
OsR498G0917743800.01	3.063	0.9	-1.76924	1.42E-116	8.07E-117	3.09	3.24	2.86	0.77	0.93	1
OsR498G0917744200.01	0.693	0.213	-1.71121	2.21E-13	3.48E-13	0.71	0.57	0.8	0.14	0.19	0.31
OsR498G0917745500.01	0.696	0.126	-2.44962	1.60E-11	2.66E-11	0.8	0.54	0.75	0.17	0.03	0.18
OsR498G0917746500.01	9.986	4.706	-1.2183	3.06E-109	1.79E-109	10.47	10.09	9.4	5.06	4.09	4.97
OsR498G0917747400.01	0.253	1.393	2.486016	5.78E-17	8.34E-17	0.19	0.39	0.18	1.62	1.25	1.31
OsR498G0917749800.01	0.393	0.063	-2.66068	1.75E-07	3.38E-07	0.36	0.29	0.53	0.1	0.06	0.03
OsR498G0917750600.01	11.823	5.61	-1.08349	2.18E-35	2.29E-35	11.82	11.98	11.67	5.38	6.01	5.44
OsR498G0917755000.01	0.42	0.04	-3.46274	3.68E-08	6.90E-08	0.48	0.35	0.43	0.08	0.04	0
OsR498G0917757400.01	1.653	0.196	-3.56309	1.86E-13	2.92E-13	2.93	0.12	1.91	0.35	0.12	0.12
OsR498G0917759000.01	0.13	0.68	2.408994	3.99E-10	6.97E-10	0.13	0.17	0.09	0.61	0.74	0.69
OsR498G0917759700.01	0.15	14.666	6.668852	8.18E-256	2.83E-256	0.08	0.29	0.08	15.48	13.87	14.65
OsR498G0917761100.01	1.436	0.476	-1.60794	5.12E-09	9.28E-09	1.1	1.58	1.63	0.47	0.58	0.38
OsR498G0917763200.01	0.1	1.393	3.794648	1.55E-29	1.76E-29	0.03	0.13	0.14	1.64	1.45	1.09
OsR498G0917769100.01	7.966	2.116	-1.9814	1.00E-92	6.43E-93	7.71	7.66	8.53	2.02	2.22	2.11
OsR498G0917774600.01	1.363	0.486	-1.46925	9.25E-07	1.85E-06	1.2	1.32	1.57	0.17	0.59	0.7
OsR498G0917776400.01	1.003	0.193	-2.38345	2.08E-09	3.71E-09	0.72	1.19	1.1	0.42	0.05	0.11
OsR498G0917779100.01	37.313	84.08	1.135257	6.74E-295	2.10E-295	36.11	37.41	38.42	83.82	85.06	83.36
OsR498G0917780400.01	4.56	103.21	4.498112	0	0	4.94	4.22	4.52	100.16	104.13	105.33
OsR498G0917780800.01	0.02	0.4	4.361688	9.48E-11	1.62E-10	0.03	0	0.03	0.56	0.34	0.3
OsR498G0917787800.01	107.1	400.68	1.900702	0	0	102.94	107.73	110.6	398.51	402.42	401.1
OsR498G0917791400.01	0.103	0.39	1.892911	7.52E-08	1.43E-07	0.11	0.05	0.15	0.49	0.38	0.3
OsR498G0917791700.01	1.526	28.82	4.156246	0	0	1.54	1.53	1.51	25.55	30.07	30.84
OsR498G0917792000.01	1.496	0.69	-1.13016	3.31E-06	6.78E-06	1.45	1.76	1.28	0.55	0.89	0.63
OsR498G0917792400.01	26.62	0.976	-5.00446	0	0	26.07	28.38	25.41	0.64	1.32	0.97
OsR498G0917792500.01	2.87	0.593	-2.42865	7.76E-39	7.84E-39	2.75	2.97	2.89	0.58	0.65	0.55
OsR498G0917794500.01	40.816	18.34	-1.15604	1.60E-131	8.45E-132	44.63	40.6	37.22	17.96	17.17	19.89
OsR498G0917797100.01	28.98	7.2	-2.11997	5.59E-221	2.14E-221	29.3	28.31	29.33	6.57	7.69	7.34
OsR498G0917797500.01	5.986	2.493	-1.21531	8.40E-40	8.38E-40	5.68	6.08	6.2	2.4	2.9	2.18
OsR498G0917797700.01	0.17	1.246	2.914229	2.18E-05	4.67E-05	0	0.35	0.16	1.56	1.75	0.43
OsR498G0917800600.01	3.586	1.743	-1.0714	4.72E-20	6.37E-20	3.54	3.14	4.08	1.42	1.63	2.18
OsR498G0917800800.01	305.68	142.42	-1.11412	0	0	305.44	311.51	300.1	143.44	142.14	141.69
OsR498G0917802500.01	11.08	0.94	-3.56361	1.36E-262	4.61E-263	10.58	10.99	11.67	0.91	1.03	0.88
OsR498G0917803000.01	0.106	27.143	8.13089	0	0	0.06	0.16	0.1	26.82	26.18	28.43
OsR498G0917803700.01	0.716	0.04	-4.14346	7.79E-25	9.58E-25	0.69	0.66	0.8	0.02	0.04	0.06
OsR498G0917804000.01	9.58	23.723	1.3037	1.36E-129	7.26E-130	10.39	9.24	9.11	23.78	24.05	23.34
OsR498G0917804300.01	0.726	0.243	-1.65056	1.89E-07	3.66E-07	0.67	0.75	0.76	0.17	0.25	0.31
OsR498G0917804500.01	1.276	5.08	1.989134	5.24E-49	4.74E-49	1.09	1.34	1.4	5.71	4.81	4.72
OsR498G0917805300.01	0.01	8.46	9.586655	4.59E-133	2.40E-133	0.03	0	0	7.38	9.57	8.43
OsR498G0917808000.01	0.67	0.23	-1.37528	3.63E-05	7.90E-05	0.6	0.77	0.64	0.13	0.3	0.26
OsR498G0917809200.01	13.486	1.5	-3.66754	1.81E-288	5.73E-289	13.18	14.16	13.12	1.13	2.01	1.36
OsR498G0917811300.01	23.93	102.7	2.10244	0	0	25.57	23.19	23.03	101.94	99.82	106.35
OsR498G0917812400.01	5.256	1.953	-1.40158	3.25E-35	3.43E-35	6.21	4.62	4.94	2.1	2.19	1.57
OsR498G0917815700.01	3.186	16.833	2.388514	5.70E-231	2.12E-231	2.82	3.34	3.4	16.1	17.64	16.76

OsR498G0917816500.01	2.16	0.666	-1.70712	3.65E-26	4.38E-26	2.11	1.86	2.51	0.58	0.89	0.53
OsR498G0917816800.01	0.07	1.863	4.697972	4.50E-25	5.50E-25	0.05	0.06	0.1	2.34	1.91	1.34
OsR498G0917827400.01	32.283	82.746	1.344923	0	0	31.23	33.66	31.96	83.76	82.74	81.74
OsR498G0917828200.01	0.44	0.093	-2.24416	0.000462	0.0010827	0.75	0.11	0.46	0.14	0.07	0.07
OsR498G0917829600.01	0.016	2.2	6.922403	4.31E-29	4.94E-29	0	0	0.05	2.98	1.71	1.91
OsR498G0917831400.01	26.583	11.96	-1.16144	5.37E-95	3.39E-95	25.94	25.31	28.5	12.4	11.36	12.12
OsR498G0917831800.01	8.633	2.683	-1.68948	8.29E-58	6.90E-58	9.1	8.53	8.27	3.04	2.44	2.57
OsR498G0917834500.01	0.933	0.066	-4.02633	1.26E-29	1.43E-29	1.22	0.83	0.75	0.07	0.02	0.11
OsR498G0917835700.01	20.283	7.266	-1.48862	4.80E-104	2.90E-104	21.41	20.08	19.36	6.33	8.12	7.35
OsR498G0917836900.01	35.123	1.66	-4.40511	0	0	34.18	34.42	36.77	1.65	1.75	1.58
OsR498G0917837400.01	0.6	0	-6.0477	9.92E-10	1.75E-09	0.63	0.66	0.51	0	0	0
OsR498G0917839400.01	79.033	29.58	-1.44672	0	0	82.81	75.43	78.86	29.08	27.51	32.15
OsR498G0917840700.01	394.35	9.733	-5.34588	0	0	402.89	391.46	388.7	10.02	9.08	10.1
OsR498G0917842100.01	2.086	0.343	-2.61232	2.15E-14	3.30E-14	2.47	2.08	1.71	0.14	0.52	0.37
OsR498G0917844300.01	0.453	1.683	1.990178	6.10E-17	8.82E-17	0.39	0.71	0.26	1.63	1.21	2.21
OsR498G0917844500.01	12.04	24.89	1.031586	1.19E-29	1.35E-29	13.02	10.82	12.28	29.35	22.84	22.48
OsR498G0917844800.01	44.18	16.123	-1.45449	5.47E-237	1.99E-237	45.9	42.25	44.39	15.55	16.24	16.58
OsR498G0917848400.01	1.22	0.393	-1.66806	9.85E-10	1.74E-09	0.79	1.48	1.39	0.23	0.56	0.39
OsR498G0917848800.01	1.426	0.47	-1.6029	1.01E-15	1.51E-15	1.82	1.39	1.07	0.35	0.62	0.44
OsR498G0917849600.01	7.126	576.06	6.333269	0	0	6.4	7.55	7.43	607.37	558.02	562.79
OsR498G0917851900.01	0.753	0.13	-2.52415	2.15E-12	3.50E-12	0.75	0.73	0.78	0.12	0.12	0.15
OsR498G0917852100.01	95.666	21.843	-2.17328	0	0	96.24	96.41	94.35	20.46	22.17	22.9
OsR498G0917852800.01	1.246	2.593	1.007666	1.92E-20	2.57E-20	1.11	1.38	1.25	2.62	2.69	2.47
OsR498G0917854200.01	1.263	5.67	2.145412	9.17E-53	8.00E-53	1.1	1.21	1.48	5.84	5.75	5.42
OsR498G0917855800.01	2.003	0.4	-2.33257	5.29E-31	5.89E-31	2.29	1.73	1.99	0.47	0.46	0.27
OsR498G0917858600.01	431.19	120.31	-1.8436	0	0	439.79	426.98	426.8	119.64	119.6	121.68
OsR498G0917860900.01	11.19	1.956	-2.53365	3.60E-32	3.95E-32	10.17	12	11.4	2.52	1.92	1.43
OsR498G0917861100.01	5.66	1.11	-2.36759	7.35E-91	4.77E-91	5.26	6.04	5.68	1.09	1.23	1.01
OsR498G0917861400.01	7.943	2.69	-1.56699	2.28E-53	1.98E-53	8.25	7.43	8.15	2.92	2.43	2.72
OsR498G0917863900.01	0.253	0.61	1.247868	2.28E-05	4.89E-05	0.24	0.19	0.33	0.51	0.77	0.55
OsR498G0917866300.01	2.096	0.36	-2.46577	4.32E-23	5.48E-23	2.31	2.03	1.95	0.61	0.24	0.23
OsR498G0917875600.01	1.126	0.096	-3.5651	8.78E-16	1.30E-15	1.3	0.92	1.16	0.05	0.1	0.14
OsR498G0917876200.01	2.616	11.46	2.114107	2.38E-126	1.28E-126	2.77	2.45	2.63	10.92	11.86	11.6
OsR498G0917878200.01	0.063	1.376	4.449151	5.78E-21	7.65E-21	0.14	0	0.05	2.02	0.74	1.37
OsR498G0917878700.01	0.94	2.633	1.474561	5.59E-29	6.41E-29	0.94	1.09	0.79	2.57	2.67	2.66
OsR498G0917880200.01	14.233	4.593	-1.64025	1.10E-48	1.00E-48	16.1	13.77	12.83	5.33	3.91	4.54
OsR498G0917882300.01	0.146	0.783	2.408994	3.99E-10	6.97E-10	0.15	0.15	0.14	0.82	0.73	0.8
OsR498G0917884300.01	148.81	2.833	-5.72032	0	0	147.56	148.56	150.3	3.07	2.66	2.77
OsR498G0917886700.01	7.993	1.863	-2.13427	2.52E-41	2.48E-41	7.62	8.4	7.96	2.05	1.35	2.19
OsR498G0917887400.01	25.483	10.666	-1.30059	2.14E-148	1.05E-148	26.24	24.13	26.08	10.66	9.96	11.38
OsR498G0917889100.01	2.093	0.906	-1.21855	4.06E-14	6.29E-14	1.98	1.94	2.36	0.84	0.76	1.12
OsR498G0917890300.01	0.283	4.206	3.897741	2.82E-56	2.38E-56	0.33	0.29	0.23	4.47	4.17	3.98
OsR498G0917897700.01	0.68	1.6	1.403607	2.45E-43	2.36E-43	0.67	0.77	0.6	1.5	1.51	1.79
OsR498G0917900200.01	21.59	57.233	1.406667	1.73E-237	6.27E-238	20.6	22.45	21.72	56.41	56.86	58.43
OsR498G0917903500.01	1.376	0.026	-5.46803	5.71E-23	7.26E-23	1.83	1.15	1.15	0	0	0.08
OsR498G0917904600.01	0.843	0.36	-1.206	6.84E-06	1.43E-05	0.89	0.83	0.81	0.41	0.3	0.37
OsR498G0917905000.01	1.996	0.533	-1.81268	1.65E-19	2.25E-19	2.42	1.46	2.11	0.55	0.47	0.58
OsR498G0917905800.01	5.783	0.113	-5.6699	2.42E-38	2.45E-38	6.7	5.56	5.09	0.11	0	0.23
OsR498G0917910100.01	1.786	20.446	3.509952	8.62E-184	3.73E-184	1.94	1.91	1.51	20.11	21.24	19.99
OsR498G0917912100.01	0.286	0.096	-1.59251	0.0004506	0.0010554	0.14	0.34	0.38	0.11	0.06	0.12
OsR498G0917913000.01	7.976	0.276	-4.89288	3.53E-195	1.47E-195	9.04	6.99	7.9	0.38	0.35	0.1
OsR498G0917915800.01	0	0.39	6.03976	1.80E-09	3.20E-09	0	0	0	0.54	0.41	0.22
OsR498G0917916100.01	2.86	0.6	-2.2552	1.11E-33	1.19E-33	2.71	3.07	2.8	0.49	0.64	0.67
OsR498G0917917200.01	0.283	0.78	1.441858	0.0003225	0.0007473	0.24	0.32	0.29	0.81	1.02	0.51
OsR498G0917917800.01	15.84	33.746	1.100386	5.47E-172	2.45E-172	15.69	15.72	16.11	34.54	31.84	34.86
OsR498G0917923200.01	1.306	0.573	-1.15955	0.0001234	0.0002775	1.57	1.75	0.6	0.77	0.32	0.63
OsR498G0917925200.01	0.17	0.016	-3.41967	0.0009442	0.002269	0.09	0.24	0.18	0.05	0	0
OsR498G0917927000.01	114.3	419.67	1.862397	0	0	117.55	108.41	116.9	452.14	401.9	404.98
OsR498G0917928600.01	19.903	52.153	1.381355	1.55E-260	5.28E-261	19.29	18.57	21.85	53.72	52.99	49.75
OsR498G0917929400.01	7.796	49.31	2.643208	0	0	8.1	6.93	8.36	46.44	49.96	51.53
OsR498G0917931100.01	2.016	12.416	2.650258	2.06E-179	9.04E-180	2.33	1.64	2.08	12.35	12.42	12.48
OsR498G0917934600.01	0.113	1.71	3.941297	2.42E-58	2.01E-58	0.17	0.08	0.09	1.61	2.09	1.43
OsR498G0917935000.01	7.156	27.463	1.96012	1.14E-259	3.91E-260	7.41	6.59	7.47	28.18	26.51	27.7
OsR498G0917935800.01	1.53	0.573	-1.40604	1.02E-16	1.48E-16	1.51	1.7	1.38	0.49	0.63	0.6
OsR498G0917939500.01	5.34	22.09	2.048932	0	0	5.62	5.4	5	21.98	22.09	22.2
OsR498G0917939700.01	6.636	2.183	-1.60544	2.23E-22	2.88E-22	6.02	7.79	6.1	2.86	1.62	2.07
OsR498G0917940000.01	21.22	93.76	2.166345	0	0	21.24	21.19	21.23	90.39	95.85	95.04
OsR498G0917940100.01	1.09	0.086	-3.77207	6.26E-66	4.86E-66	0.9	1.25	1.12	0.12	0.09	0.05
OsR498G0917941600.01	1.113	0.063	-4.18906	9.04E-18	1.28E-17	0.98	1.29	1.07	0.09	0.05	0.05

OsR498G0917941800.01	1.393	5.153	1.887126	1.83E-91	1.19E-91	1.49	1.42	1.27	5.22	5.18	5.06
OsR498G0917942100.01	19.46	61.373	1.65409	0	0	19.96	19.66	18.76	60.09	64.66	59.37
OsR498G0917948900.01	4.183	2.133	-1.01233	2.35E-12	3.82E-12	4.28	4.36	3.91	1.67	2.59	2.14
OsR498G0917949100.01	1.776	0.576	-1.59912	3.87E-14	5.98E-14	1.76	1.89	1.68	0.56	0.61	0.56
OsR498G0917952100.01	0.013	1.883	7.258929	1.69E-35	1.78E-35	0.04	0	0	1.86	2	1.79
OsR498G0917952300.01	0.11	6.786	5.935386	1.27E-214	4.97E-215	0.09	0.16	0.08	7.04	6.92	6.4
OsR498G0917953300.01	0.053	5.25	6.661812	9.49E-186	4.08E-186	0.06	0.06	0.04	5.46	5.25	5.04
OsR498G0917953800.01	5.713	27.2	2.24557	3.61E-256	1.25E-256	5.13	5.81	6.2	30.74	25.66	25.2
OsR498G0917957000.01	56.356	6.863	-3.04752	0	0	55.98	55.14	57.95	6.29	7.33	6.97
OsR498G0917960200.01	114.34	38.906	-1.12337	0	0	119.08	114.23	109.7	41.69	37.89	37.14
OsR498G0917961200.01	0.973	3.47	1.745177	3.11E-33	3.36E-33	0.68	1.3	0.94	4.74	2.81	2.86
OsR498G0917961300.01	0.97	3.916	2.001506	1.45E-43	1.39E-43	1.09	0.92	0.9	3.83	4.33	3.59
OsR498G0917962700.01	3.55	7.893	1.139543	1.49E-23	1.87E-23	3.45	2.94	4.26	8.18	8.06	7.44
OsR498G0917971800.01	0.3	2.53	3.08417	1.68E-41	1.64E-41	0.36	0.37	0.17	2.09	3.24	2.26
OsR498G0917973000.01	0.156	0.95	2.584081	7.18E-07	1.42E-06	0.47	0	0	0.96	0.66	1.23
OsR498G0917973100.01	0.413	19.38	5.635324	0	0	0.39	0.39	0.46	18.08	19.56	20.5
OsR498G0917973700.01	3.386	35.323	3.199124	0	0	2.86	3.85	3.45	34.87	34.81	36.29
OsR498G0917976000.01	0.043	0.306	2.847115	2.00E-07	3.86E-07	0.05	0.03	0.05	0.26	0.4	0.26
OsR498G0917976300.01	0.643	1.736	1.432897	1.08E-08	1.99E-08	0.54	0.75	0.64	1.55	1.99	1.67
OsR498G0917977900.01	1.82	92.513	5.663501	0	0	1.58	1.83	2.05	100.19	87.52	89.83
OsR498G0917978100.01	17.366	54.243	1.622525	4.25E-158	2.00E-158	17.16	15.86	19.08	57.23	53.03	52.47
OsR498G0917980300.01	0	1.093	6.361688	2.55E-11	4.28E-11	0	0	0	1.21	1.08	0.99
OsR498G0917981400.01	10.55	64.4	2.619573	0	0	9.48	10.84	11.33	63.66	63.14	66.4
OsR498G0917982900.01	32.273	130.54	2.008183	0	0	33.71	32.01	31.1	144.15	119.81	127.67
OsR498G0917983600.01	13.86	66.696	2.246422	0	0	13.46	13.9	14.22	63.62	68.36	68.11
OsR498G0917984000.01	1.233	0.523	-1.24789	0.0001347	0.0003037	1.32	1.28	1.1	0.33	0.59	0.65
OsR498G1017994000.01	41.696	13.953	-1.55183	2.65E-286	8.44E-287	42.09	42.11	40.89	13.97	14.16	13.73
OsR498G1017994900.01	249.58	491.26	1.115271	0	0	252.38	257.53	238.8	544.47	463.42	465.9
OsR498G1017995500.01	0	3.59	8.885187	4.07E-46	3.80E-46	0	0	0	5.8	2.54	2.43
OsR498G1017996700.01	3	7.603	1.341563	4.61E-70	3.47E-70	2.68	3.19	3.13	8.48	7.65	6.68
OsR498G1017997800.01	27.45	174.19	2.662473	0	0	27.04	28.52	26.79	185.08	169.16	168.33
OsR498G1018004500.01	0.24	0.996	2.03976	4.18E-17	6.02E-17	0.37	0.11	0.24	0.98	1.15	0.86
OsR498G1018010600.01	0.923	0.373	-1.32023	1.65E-05	3.52E-05	0.84	0.83	1.1	0.35	0.18	0.59
OsR498G1018016300.01	0.456	2.99	2.65447	3.92E-09	7.07E-09	0.52	0.18	0.67	3.46	2.39	3.12
OsR498G1018022900.01	1.04	0.463	-1.13427	9.91E-06	2.09E-05	1.29	0.87	0.96	0.56	0.44	0.39
OsR498G1018029600.01	0.383	0	-6.38348	9.97E-12	1.65E-11	0.37	0.5	0.28	0	0	0
OsR498G1018031600.01	8.803	3.716	-1.23708	1.19E-53	1.03E-53	8.55	8.9	8.96	3.51	3.47	4.17
OsR498G1018034200.01	15.353	0.9	-3.75159	0	0	15.48	15.08	15.5	0.97	0.84	0.89
OsR498G1018036600.01	47	160.67	1.771918	0	0	47.71	46.26	47.03	171.24	153.24	157.53
OsR498G1018037200.01	4.846	10.833	1.161947	1.11E-63	8.80E-64	4.62	4.39	5.53	10.94	10.46	11.1
OsR498G1018037600.01	3.813	33.313	3.120482	0	0	4.2	3.43	3.81	32.58	32.5	34.86
OsR498G1018038500.01	6.833	1.626	-2.07572	2.83E-48	2.58E-48	6.81	6.79	6.9	1.74	1.65	1.49
OsR498G1018039500.01	0.023	0.456	4.325162	3.71E-19	5.11E-19	0	0	0.07	0.52	0.41	0.44
OsR498G1018042100.01	9.276	2.996	-1.63882	7.99E-47	7.40E-47	8.93	9.38	9.52	2.5	3.35	3.14
OsR498G1018043000.01	7.453	15.53	1.052929	3.41E-51	3.02E-51	7.43	7.55	7.38	15.71	14.88	16
OsR498G1018061000.01	0.176	1.556	3.132206	8.97E-53	7.81E-53	0.16	0.16	0.21	1.67	1.43	1.57
OsR498G1018061800.01	0.746	0.1	-3.84777	9.12E-17	1.32E-16	0.9	0.62	0.72	0.26	0.02	0.02
OsR498G1018062600.01	15.44	2.226	-2.80002	3.01E-98	1.87E-98	17.54	15.44	13.34	2.69	2.08	1.91
OsR498G1018069100.01	0.203	2.913	3.82112	3.45E-13	5.47E-13	0.3	0.16	0.15	3.15	2.88	2.71
OsR498G1018069300.01	0.306	0.1	-1.59767	0.0007574	0.0018079	0.39	0.24	0.29	0.17	0.1	0.03
OsR498G1018070300.01	4.243	1.793	-1.24975	6.86E-10	1.21E-09	4.54	3.73	4.46	1.95	1.71	1.72
OsR498G1018080200.01	0.043	0.566	3.7402	0.0002182	0.0004994	0	0.13	0	0.39	0.92	0.39
OsR498G1018080600.01	0.063	0.353	2.454798	3.66E-06	7.50E-06	0.06	0.07	0.06	0.29	0.47	0.3
OsR498G1018081200.01	0	0.19	5.209685	4.72E-06	9.74E-06	0	0	0	0.31	0.1	0.16
OsR498G1018086000.01	0.143	0.373	1.361688	0.0007094	0.0016883	0.03	0.1	0.3	0.22	0.55	0.35
OsR498G1018086400.01	0.146	0.44	1.587248	0.0001092	0.0002446	0.13	0.21	0.1	0.38	0.35	0.59
OsR498G1018087000.01	0.16	0.543	1.755967	6.95E-06	1.45E-05	0.21	0.14	0.13	0.52	0.58	0.53
OsR498G1018091000.01	2.816	1.366	-1.04134	4.01E-26	4.82E-26	2.88	2.89	2.68	1.57	1.39	1.14
OsR498G1018091500.01	1	0.216	-2.20817	1.95E-17	2.79E-17	0.89	1.22	0.89	0.18	0.26	0.21
OsR498G1018095600.01	0.466	0.063	-2.83471	1.58E-15	2.36E-15	0.38	0.58	0.44	0.06	0.08	0.05
OsR498G1018098400.01	7.626	1.51	-2.36234	4.04E-44	3.86E-44	7.48	7.32	8.08	1.6	1.55	1.38
OsR498G1018099300.01	0.073	0	-4.96024	2.02E-05	4.33E-05	0.06	0.07	0.09	0	0	0
OsR498G1018101300.01	0.05	1.016	4.361688	9.48E-11	1.62E-10	0	0.15	0	1.51	0.62	0.92
OsR498G1018102000.01	0.15	7.896	5.709611	2.04E-135	1.06E-135	0.16	0.13	0.16	7.64	8.37	7.68
OsR498G1018103400.01	0.283	0.016	-4.0477	2.60E-05	5.61E-05	0.5	0.05	0.3	0.05	0	0
OsR498G1018104800.01	6.98	40.506	2.525443	0	0	7.12	6.75	7.07	40.58	41.56	39.38
OsR498G1018105500.01	0.406	2	2.285236	2.59E-20	3.48E-20	0.47	0.33	0.42	1.64	2.23	2.13
OsR498G1018109000.01	6.58	1.25	-2.32	6.46E-93	4.14E-93	6.11	7.21	6.42	1.15	1.07	1.53
OsR498G1018109100.01	14.75	0.383	-5.19081	0	0	14.64	15.57	14.04	0.39	0.34	0.42

OsR498G1018111400.01	0.97	2.273	1.227832	2.96E-11	4.97E-11	1.1	1.07	0.74	2.34	2.35	2.13
OsR498G1018112500.01	3.363	18.836	2.483622	0	0	2.94	3.53	3.62	18.46	19.13	18.92
OsR498G1018116900.01	0.776	0.136	-2.50081	1.58E-10	2.72E-10	0.61	1.24	0.48	0.15	0.15	0.11
OsR498G1018117600.01	0.163	4.663	4.834176	1.06E-104	6.40E-105	0.12	0.13	0.24	5.13	4.65	4.21
OsR498G1018119400.01	0.873	0	-6.79313	9.42E-15	1.43E-14	1.06	0.67	0.89	0	0	0
OsR498G1018122500.01	0.603	0.083	-2.90777	1.91E-10	3.29E-10	0.67	0.49	0.65	0.07	0.11	0.07
OsR498G1018123500.01	8.306	32.63	1.971143	3.00E-219	1.16E-219	8.99	7.73	8.2	32.74	31.42	33.73
OsR498G1018125300.01	11.023	53.656	2.279783	0	0	11.61	10.95	10.51	51.65	54.31	55.01
OsR498G1018131600.01	1.206	0	-6.86713	2.23E-15	3.34E-15	1.2	1.2	1.22	0	0	0
OsR498G1018133600.01	0.04	0.326	3.03976	6.82E-06	1.42E-05	0.04	0	0.08	0.24	0.29	0.45
OsR498G1018140100.01	0.046	0.443	3.209685	2.71E-08	5.04E-08	0	0	0.14	0.36	0.34	0.63
OsR498G1018141300.01	0.046	0.396	3.209685	3.55E-13	5.62E-13	0.04	0.06	0.04	0.39	0.5	0.3
OsR498G1018141500.01	4.743	1.756	-1.25282	1.46E-16	2.13E-16	3.99	5.87	4.37	1.93	1.5	1.84
OsR498G1018144800.01	4.22	10.026	1.598624	9.60E-117	5.44E-117	3.27	5.45	3.94	10.25	9.86	9.97
OsR498G1018147200.01	0.793	0.116	-2.79031	1.25E-11	2.08E-11	0.97	0.47	0.94	0.08	0.08	0.19
OsR498G1018169000.01	12.486	3.34	-1.91124	4.06E-100	2.50E-100	12.72	12.03	12.71	3.53	3.65	2.84
OsR498G1018182700.01	4.046	1.216	-1.74451	2.03E-36	2.11E-36	3.86	3.83	4.45	1.23	1.06	1.36
OsR498G1018185400.01	7.116	2.76	-1.55718	1.61E-19	2.19E-19	6.86	6.8	7.69	3.27	2.3	2.71
OsR498G1018188600.01	15.766	4.233	-1.89364	8.23E-148	4.05E-148	15.33	15.06	16.91	3.67	4.43	4.6
OsR498G1018201600.01	0.91	5.27	2.631819	6.22E-35	6.58E-35	0.68	1.05	1	5.11	5.84	4.86
OsR498G1018201700.01	0.14	0.04	-2.01913	0.0002324	0.0005331	0.15	0.13	0.14	0.06	0.03	0.03
OsR498G1018206100.01	0.976	0.206	-2.26649	5.57E-24	6.96E-24	0.89	1.01	1.03	0.18	0.21	0.23
OsR498G1018216900.01	3.793	7.806	1.02481	2.46E-09	4.41E-09	5.37	3.08	2.93	8.08	7.02	8.32
OsR498G1018217100.01	68.943	21.84	-1.66047	0	0	71.14	68.39	67.3	22.58	21.58	21.36
OsR498G1018218000.01	0.21	0.506	1.860051	4.69E-06	9.69E-06	0.01	0.29	0.33	0.38	0.63	0.51
OsR498G1018224900.01	11.45	23.4	1.01282	6.28E-77	4.47E-77	10.88	11.28	12.19	23.57	23.77	22.86
OsR498G1018228800.01	0.4	1.856	2.237699	2.17E-11	3.63E-11	0.62	0.58	0	2.54	1.74	1.29
OsR498G1018238100.01	3.02	0.336	-3.17907	1.32E-48	1.20E-48	3.54	2.87	2.65	0.27	0.46	0.28
OsR498G1018238900.01	0.1	0.01	-3.41967	0.0009442	0.002269	0.08	0.03	0.19	0	0	0.03
OsR498G1018239200.01	181.4	81.23	-1.16453	0	0	182.77	180.23	181.2	78.61	81.63	83.45
OsR498G1018255800.01	16.433	2.07	-3.13527	1.24E-291	3.91E-292	16.37	15.57	17.36	2.11	2.11	1.99
OsR498G1018265600.01	11.33	4.606	-1.30419	9.14E-61	7.40E-61	11.9	10.87	11.22	4.54	5.02	4.26
OsR498G1018269500.01	4.646	1.12	-2.05422	1.39E-25	1.68E-25	4.11	5.5	4.33	1.08	1.18	1.1
OsR498G1018281600.01	85.576	18.983	-2.21213	0	0	85.05	86.34	85.34	17.93	19	20.02
OsR498G1018288800.01	2.26	1.076	-1.06765	5.45E-20	7.38E-20	2.5	2.17	2.11	0.87	1.3	1.06
OsR498G1018293300.01	1.15	3.166	1.454798	3.80E-31	4.22E-31	1.05	1.47	0.93	3.33	3.11	3.06
OsR498G1018302000.01	0	0.28	4.361688	0.0005877	0.0013892	0	0	0	0.25	0.34	0.25
OsR498G1018310500.01	39.473	18.433	-1.1081	6.96E-245	2.48E-245	40.32	39.32	38.78	17.21	19.03	19.06
OsR498G1018314600.01	102.97	36.806	-1.49706	0	0	106.53	100.92	101.5	35.81	36.53	38.08
OsR498G1018317800.01	4.453	16.346	1.828624	2.97E-169	1.34E-169	4.23	4.19	4.94	16.32	16.83	15.89
OsR498G1018323000.01	0.423	0.066	-2.72577	7.08E-07	1.41E-06	0.45	0.35	0.47	0.08	0.04	0.08
OsR498G1018333000.01	0.39	0.113	-2.05335	1.15E-08	2.12E-08	0.42	0.38	0.37	0.06	0.22	0.06
OsR498G1018338500.01	0.59	0.063	-3.15805	7.65E-12	1.26E-11	0.49	0.54	0.74	0.06	0.06	0.07
OsR498G1018339300.01	2.8	1.233	-1.1958	4.52E-13	7.18E-13	2.74	2.04	3.62	1.49	1.05	1.16
OsR498G1018343700.01	16.75	4.413	-1.92689	2.17E-211	8.56E-212	16.78	16.29	17.18	3.95	4.57	4.72
OsR498G1018362000.01	0.06	0.54	3.155237	2.12E-06	4.31E-06	0.12	0.06	0	0.55	0.32	0.75
OsR498G1018371000.01	0.63	0.146	-2.08735	6.71E-11	1.14E-10	0.7	0.73	0.46	0.21	0.14	0.09
OsR498G1018374400.01	3.883	17.12	2.127489	3.13E-157	1.48E-157	3.97	3.67	4.01	17.19	17.63	16.54
OsR498G1018375100.01	3.28	1.17	-1.46564	4.33E-57	3.64E-57	2.84	3.52	3.48	1.19	1.23	1.09
OsR498G1018378900.01	29.623	12.083	-1.29769	1.22E-102	7.45E-103	30.74	28.43	29.7	12.3	12.14	11.81
OsR498G1018387300.01	2.066	1.566	-1.10512	5.75E-36	6.00E-36	1.93	2.09	2.18	1.7	1.57	1.43
OsR498G1018391500.01	303.98	107.99	-1.50406	0	0	308.21	302.78	300.9	108.35	106.36	109.27
OsR498G1018393600.01	4.84	34.77	2.808945	0	0	5.19	4.83	4.5	35.43	35.45	33.43
OsR498G1018399800.01	47.7	16.45	-1.54667	1.36E-143	6.84E-144	50	45.92	47.18	17.51	16.18	15.66
OsR498G1018420100.01	2.113	0.73	-1.7459	1.04E-21	1.36E-21	2.24	2.02	2.08	0.65	0.61	0.93
OsR498G1018427100.01	40.256	121.75	1.592178	0	0	37.83	41.58	41.36	124.43	122.37	118.46
OsR498G1018431000.01	9.55	4.183	-1.21933	7.18E-46	6.73E-46	10.02	10.05	8.58	3.96	4.24	4.35
OsR498G1018432500.01	0.203	1.086	2.378562	3.55E-13	5.63E-13	0.29	0.11	0.21	1.26	1.13	0.87
OsR498G1018435100.01	3.41	1.666	-1.10093	5.63E-29	6.46E-29	3.28	3.41	3.54	1.46	1.91	1.63
OsR498G1018439200.01	0.163	0.85	2.344094	5.96E-23	7.59E-23	0.12	0.14	0.23	0.8	0.84	0.91
OsR498G1018440800.01	0.566	1.523	1.418272	4.91E-06	1.02E-05	0.34	0.71	0.65	1.18	1.49	1.9
OsR498G1018443700.01	0.533	0.09	-3.0477	1.75E-07	3.37E-07	0.42	0.74	0.44	0.07	0.13	0.07
OsR498G1018449400.01	1.1	3.693	1.71924	2.86E-37	2.94E-37	1.17	0.93	1.2	3.98	3.65	3.45
OsR498G1018450400.01	2.43	5.476	1.143265	4.03E-26	4.85E-26	2.09	2.43	2.77	5.14	5.7	5.59
OsR498G1018453300.01	1.203	0.423	-1.4994	3.05E-08	5.69E-08	1.46	1.24	0.91	0.39	0.48	0.4
OsR498G1018461300.01	100.37	1.136	-6.45768	0	0	102.43	98.74	99.94	1.35	1.04	1.02
OsR498G1018461600.01	4.266	0.04	-6.78574	3.90E-22	5.04E-22	2.42	4.65	5.73	0.01	0.11	0
OsR498G1018472400.01	0.646	0.2	-1.68702	9.18E-09	1.68E-08	0.68	0.67	0.59	0.19	0.18	0.23
OsR498G1018473300.01	0.923	0.403	-1.18968	0.0002329	0.0005345	0.91	1.37	0.49	0.26	0.53	0.42

OsR498G1018484800.01	0.53	0	-4.41967	0.0003895	0.0009082	1.01	0.3	0.28	0	0	0
OsR498G1018485600.01	0.11	0.9	3.048332	7.12E-26	8.59E-26	0.11	0.15	0.07	0.89	1.06	0.75
OsR498G1018499500.01	10.64	4.436	-1.23946	6.93E-62	5.57E-62	10.44	11.08	10.4	4.6	4.7	4.01
OsR498G1018501800.01	23.376	7.086	-1.80236	3.48E-143	1.75E-143	22.03	22.26	25.84	6.63	6.26	8.37
OsR498G1018502800.01	20.963	64.04	1.603589	0	0	22.59	20.46	19.84	62.76	64.44	64.92
OsR498G1018504700.01	0	0.11	4.361688	0.0005877	0.0013892	0	0	0	0.06	0.17	0.1
OsR498G1018505600.01	9.386	47.803	2.356506	0	0	9.36	9.63	9.17	48.56	46.98	47.87
OsR498G1018511200.01	0	0.116	4.847115	5.08E-05	0.0001113	0	0	0	0.12	0.13	0.1
OsR498G1018518700.01	0.296	0	-5.13016	6.38E-06	1.33E-05	0.34	0.41	0.14	0	0	0
OsR498G1018527900.01	0.226	0	-5.63606	9.24E-08	1.76E-07	0.1	0.43	0.15	0	0	0
OsR498G1018528200.01	1.916	0.726	-1.42231	6.26E-26	7.54E-26	1.97	2.11	1.67	0.71	0.74	0.73
OsR498G1018533200.01	0.01	0.43	5.084154	1.31E-09	2.33E-09	0	0	0.03	0.45	0.38	0.46
OsR498G1018534900.01	0.686	1.98	1.525786	3.72E-28	4.32E-28	0.76	0.7	0.6	1.9	2.04	2
OsR498G1018540900.01	6.47	0.613	-3.41119	3.50E-64	2.76E-64	6.49	6.87	6.05	0.35	0.84	0.65
OsR498G1018542500.01	3.013	7.203	1.232651	1.19E-10	2.03E-10	1.6	3.05	4.39	7.04	6.95	7.62
OsR498G1018545900.01	14.426	1.8	-3.02202	5.06E-57	4.25E-57	16.13	12.94	14.21	2.01	1.63	1.76
OsR498G1018549300.01	34.076	9.926	-1.7815	4.75E-291	1.49E-291	33.58	33.47	35.18	9.61	9.77	10.4
OsR498G1018549600.01	2.926	1	-1.55085	1.76E-57	1.47E-57	2.87	2.93	2.98	0.91	1.1	0.99
OsR498G1018550000.01	0.79	0.28	-1.48107	2.87E-06	5.86E-06	0.65	0.68	1.04	0.29	0.36	0.19
OsR498G1018551500.01	37.74	13.133	-1.51259	1.20E-108	7.06E-109	42.54	34.25	36.43	13.49	13	12.91
OsR498G1018551700.01	0.17	1.366	3.01704	1.51E-12	2.44E-12	0.19	0.26	0.06	1.67	1.52	0.91
OsR498G1018552900.01	0.84	16.68	4.299322	1.62E-166	7.39E-167	0.45	0.95	1.12	18.54	15.08	16.42
OsR498G1018555200.01	17.003	5.346	-1.69796	2.05E-54	1.76E-54	17.62	16.02	17.37	5.48	5.34	5.22
OsR498G1018558500.01	0.066	0.31	2.169043	6.56E-06	1.37E-05	0.05	0.03	0.12	0.26	0.35	0.32
OsR498G1018558800.01	44.203	14.35	-1.63118	0	0	45.05	43.06	44.5	15.37	14.17	13.51
OsR498G1018560400.01	0.24	2.97	3.624723	2.60E-61	2.10E-61	0.23	0.3	0.19	3.09	2.95	2.87
OsR498G1018561400.01	21.94	10.383	-1.1288	6.01E-119	3.37E-119	20.64	22.86	22.32	10.55	10.36	10.24
OsR498G1018562700.01	1.353	0.253	-2.4077	1.25E-09	2.21E-09	1.54	1.48	1.04	0.27	0.28	0.21
OsR498G1018567200.01	0.603	0.116	-2.13016	7.63E-07	1.52E-06	0.72	0.63	0.46	0.11	0.12	0.12
OsR498G1018573900.01	4.103	1.593	-1.36541	3.47E-54	2.99E-54	3.86	3.78	4.67	1.28	1.79	1.71
OsR498G1018574700.01	0.173	0.006	-5.0477	1.30E-09	2.31E-09	0.17	0.19	0.16	0	0	0.02
OsR498G1018576500.01	53.966	131.95	1.295533	0	0	51.06	55.06	55.78	129.11	131.31	135.43
OsR498G1018583800.01	20.63	41.77	1.022546	2.16E-105	1.29E-105	20.84	19.9	21.15	44.47	39.4	41.44
OsR498G1018586200.01	56.573	6.446	-3.37412	0	0	58.25	53.01	58.46	5.76	6.7	6.88
OsR498G1018590600.01	0	0.85	7.189507	5.56E-18	7.85E-18	0	0	0	0.99	0.73	0.83
OsR498G1018591600.01	0	0.25	5.361688	1.47E-06	2.96E-06	0	0	0	0.37	0.15	0.23
OsR498G1018595800.01	4.593	9.7	1.071598	8.88E-54	7.67E-54	4.19	4.61	4.98	9.47	9.57	10.06
OsR498G1018617600.01	3.983	0.19	-4.34567	5.43E-47	5.02E-47	3.95	3.77	4.23	0.22	0.16	0.19
OsR498G1018621200.01	1.286	13.076	3.295017	1.87E-110	1.09E-110	1.74	0.86	1.26	12.88	13.27	13.08
OsR498G1018621400.01	15.883	32.47	1.024455	1.38E-78	9.73E-79	16.37	14.95	16.33	31.29	33.47	32.65
OsR498G1018622000.01	3.486	0.7	-2.33837	8.75E-21	1.16E-20	3.27	2.83	4.36	0.57	0.66	0.87
OsR498G1018622100.01	2.03	0.633	-1.60911	5.02E-36	5.24E-36	2	2.22	1.87	0.57	0.82	0.51
OsR498G1018622300.01	1.483	0.313	-2.24975	1.12E-17	1.59E-17	1.37	1.75	1.33	0.24	0.28	0.42
OsR498G1018622600.01	5.576	72.256	3.693719	0	0	6	5.55	5.18	73.85	71.41	71.51
OsR498G1018623200.01	4.67	19.95	2.321675	0	0	4.28	4.89	4.84	19.29	19.75	20.81
OsR498G1018623600.01	2.423	89.506	5.753053	0	0	2.88	1.51	2.88	97.37	89.35	81.8
OsR498G1018624000.01	0.293	3.196	3.624723	1.09E-44	1.03E-44	0.21	0.22	0.45	2.87	3.65	3.07
OsR498G1018625400.01	2.013	0.12	-4.09195	4.00E-64	3.15E-64	2.17	1.68	2.19	0.1	0.11	0.15
OsR498G1018626300.01	9.083	44.263	2.283868	0	0	8.56	9.9	8.79	42.89	44.49	45.41
OsR498G1018626700.01	2.136	4.653	1.121998	3.72E-36	3.88E-36	2.16	2.25	2	4.47	4.66	4.83
OsR498G1018631200.01	0	2.823	7.970497	8.08E-28	9.44E-28	0	0	0	3.24	2.39	2.84
OsR498G1018635200.01	0.606	48.84	6.490971	0	0	0.23	0.89	0.7	53.57	48.15	44.8
OsR498G1018636500.01	0.02	1.326	6.194781	8.10E-19	1.12E-18	0	0.06	0	1.38	0.91	1.69
OsR498G1018638500.01	0.013	0.366	4.794648	4.50E-08	8.46E-08	0	0	0.04	0.24	0.41	0.45
OsR498G1018638900.01	0	0.56	6.397312	1.51E-11	2.52E-11	0	0	0	0.56	0.71	0.41
OsR498G1018641100.01	2.406	0.976	-1.35785	5.18E-43	5.01E-43	2.21	2.69	2.32	0.97	1.07	0.89
OsR498G1018641400.01	9.12	1.3	-3.12249	7.94E-63	6.32E-63	9.15	8.94	9.27	1.71	1.29	0.9
OsR498G1018643000.01	1.41	7.893	2.482089	7.17E-83	4.90E-83	1.36	1.59	1.28	8.68	6.89	8.11
OsR498G1018644000.01	0.17	1.176	2.930531	7.08E-46	6.63E-46	0.16	0.22	0.13	1.02	1.54	0.97
OsR498G1018648600.01	1.416	0	-6.31779	2.64E-11	4.42E-11	1.78	1.24	1.23	0	0	0
OsR498G1018652000.01	4.7	0.246	-3.73283	3.44E-34	3.67E-34	4.14	4.75	5.21	0.43	0.05	0.26
OsR498G1018654200.01	0.013	0.19	3.908323	0.0001806	0.0004107	0	0.04	0	0.26	0.09	0.22
OsR498G1018676700.01	7.193	0.913	-3.08715	8.72E-130	4.64E-130	7.09	6.55	7.94	1.04	0.7	1
OsR498G1018685200.01	21.013	5.15	-2.01897	7.42E-305	2.24E-305	21.69	19.96	21.39	5.02	5.11	5.32
OsR498G1018699100.01	17.213	8.533	-1.02237	1.32E-133	6.88E-134	17.11	17.57	16.96	8.35	8.53	8.72
OsR498G1018700800.01	1.97	0	-7.98261	9.78E-29	1.12E-28	1.95	1.95	2.01	0	0	0
OsR498G1018701100.01	0.996	4.113	1.997725	1.09E-62	8.71E-63	1.06	1.13	0.8	3.55	4.36	4.43
OsR498G1018707600.01	2.993	1.173	-1.37528	2.22E-17	3.17E-17	3.15	3.15	2.68	1.08	1.3	1.14
OsR498G1018709100.01	15.26	6.04	-1.33672	6.27E-82	4.31E-82	15.65	14.72	15.41	5.72	5.98	6.42

OsR498G1018711500.01	0.113	0.443	1.996914	5.33E-05	0.0001171	0.18	0.16	0	0.41	0.33	0.59
OsR498G1018711900.01	0.586	0.27	-1.12448	0.0007443	0.0017751	0.46	0.81	0.49	0.24	0.32	0.25
OsR498G1018717500.01	0.563	0.02	-4.71513	5.31E-20	7.18E-20	0.58	0.65	0.46	0.02	0.04	0
OsR498G1018717900.01	0.593	1.156	1.073707	2.10E-05	4.50E-05	0.37	0.71	0.7	1.22	1.01	1.24
OsR498G1018718400.01	0.706	0.143	-2.29466	4.27E-16	6.30E-16	0.51	0.8	0.81	0.09	0.13	0.21
OsR498G1018718600.01	0.346	3.303	3.213897	1.80E-63	1.42E-63	0.33	0.15	0.56	3.38	3.39	3.14
OsR498G1018720700.01	122.24	276.65	1.093474	0	0	123.51	120.03	123.2	269.35	276.92	283.69
OsR498G1018721400.01	0.116	0.01	-3.41967	0.0009442	0.002269	0	0.13	0.22	0	0	0.03
OsR498G1018723700.01	4.566	23.236	2.343359	1.62E-193	6.76E-194	5.3	4.37	4.03	25.22	22.3	22.19
OsR498G1018725300.01	0.15	0.506	1.776726	0.0001826	0.0004153	0.05	0.21	0.19	0.7	0.31	0.51
OsR498G1018728100.01	24.176	296.7	3.614549	0	0	23.14	26.51	22.88	301.57	292.5	296.02
OsR498G1018729400.01	8.88	3.94	-1.17895	4.09E-39	4.11E-39	9.25	8.25	9.14	4.12	4.12	3.58
OsR498G1018732200.01	0.006	0.383	5.946651	3.38E-16	4.97E-16	0	0.02	0	0.34	0.44	0.37
OsR498G1018736600.01	2.553	19.346	2.934385	2.81E-156	1.33E-156	2.55	2.61	2.5	19.65	18.86	19.53
OsR498G1018737400.01	4.426	11.686	1.394979	2.00E-53	1.73E-53	4.17	4.64	4.47	11.71	12.29	11.06
OsR498G1018744600.01	5.64	2.26	-1.33436	3.80E-70	2.85E-70	5.66	5.43	5.83	2.35	2.18	2.25
OsR498G1018745900.01	0.096	0.586	2.589957	9.15E-08	1.74E-07	0.08	0.17	0.04	0.63	0.57	0.56
OsR498G1018748200.01	1.793	0.31	-2.6265	8.81E-23	1.13E-22	1.86	1.92	1.6	0.42	0.29	0.22
OsR498G1018763200.01	183.13	475.49	1.371019	0	0	174.6	186.72	188.1	495.64	460.15	470.69
OsR498G1018763500.01	1.953	0.336	-2.52095	5.08E-18	7.15E-18	2.04	1.82	2	0.57	0.15	0.29
OsR498G1018768400.01	9.373	4.02	-1.27497	1.01E-55	8.59E-56	9.14	9.98	9	3.72	3.93	4.41
OsR498G1018774500.01	0.823	0.313	-1.40955	2.91E-06	5.94E-06	0.75	0.64	1.08	0.4	0.27	0.27
OsR498G1018776400.01	1.363	0.243	-2.87216	3.74E-25	4.57E-25	1.6	1.2	1.29	0.31	0.32	0.1
OsR498G1018776600.01	0.413	0.106	-1.98477	1.15E-05	2.44E-05	0.42	0.41	0.41	0.16	0	0.16
OsR498G1018781700.01	0.243	0.59	1.290304	1.11E-05	2.34E-05	0.1	0.34	0.29	0.73	0.6	0.44
OsR498G1018782300.01	1.386	0.426	-1.45378	6.97E-09	1.27E-08	1.3	1.29	1.57	0.39	0.48	0.41
OsR498G1018783200.01	10.666	5.303	-1.01505	5.65E-70	4.25E-70	10.12	11.1	10.78	5.47	5.33	5.11
OsR498G1018787000.01	7.436	3.463	-1.14623	1.31E-28	1.51E-28	8.68	7.16	6.47	3.94	3.47	2.98
OsR498G1018787300.01	3.866	0.966	-2.01208	1.49E-19	2.04E-19	3.2	3.93	4.47	1.04	0.72	1.14
OsR498G1018793600.01	1.146	3.05	1.403723	6.88E-21	9.13E-21	1.61	0.88	0.95	2.82	2.95	3.38
OsR498G1018794000.01	2.876	5.81	1.036202	5.63E-18	7.95E-18	2.46	3.15	3.02	6.14	5.23	6.06
OsR498G1018794500.01	15.096	118.9	2.941422	0	0	14.56	15.06	15.67	125.15	117.59	113.97
OsR498G1018797700.01	15.37	38.296	1.308521	7.18E-127	3.87E-127	15.92	15.6	14.59	37.49	39.16	38.24
OsR498G1018797900.01	0.186	5.246	4.818932	2.75E-154	1.32E-154	0.09	0.22	0.25	5.53	4.91	5.3
OsR498G1018798100.01	0.133	1.84	3.785187	1.74E-48	1.58E-48	0.07	0.17	0.16	1.95	1.82	1.75
OsR498G1018798600.01	1.21	3.61	1.480884	1.12E-69	8.42E-70	1.34	1.18	1.11	3.36	3.96	3.51
OsR498G1018800500.01	3.746	31.14	3.104404	0	0	3.22	3.93	4.09	29.78	31.67	31.97
OsR498G1018804000.01	16.873	37.93	1.165619	8.32E-175	3.69E-175	16.28	17.22	17.12	42.04	36.85	34.9
OsR498G1018809400.01	1.866	0.443	-2.19157	2.19E-14	3.36E-14	1.12	2.8	1.68	0.41	0.48	0.44
OsR498G1018810600.01	9.516	4.16	-1.2017	1.63E-43	1.56E-43	6.65	10.94	10.96	3.09	4.67	4.72
OsR498G1018810900.01	1.08	52.05	5.601739	0	0	1.15	1.56	0.53	53.39	51.73	51.03
OsR498G1018811700.01	4.64	1.916	-1.3448	1.28E-46	1.19E-46	4.79	4.77	4.36	1.93	1.75	2.07
OsR498G1018825800.01	18.26	5.146	-1.82106	2.89E-94	1.84E-94	18.04	19.67	17.07	5.64	4.87	4.93
OsR498G1018836900.01	0.523	1.906	1.854728	5.80E-11	9.82E-11	0.52	0.43	0.62	2.25	2.08	1.39
OsR498G1018837500.01	3.623	9.3	1.355867	1.34E-69	1.01E-69	3.37	3.66	3.84	10.3	9.54	8.06
OsR498G1018841600.01	8.716	1.263	-2.78784	2.99E-100	1.84E-100	9.58	8.26	8.31	1.33	1.29	1.17
OsR498G1018854200.01	2.973	6.083	1.030023	2.58E-19	3.53E-19	2.95	3.01	2.96	6.64	5.32	6.29
OsR498G1018854900.01	17.326	6.85	-1.35928	7.01E-18	9.92E-18	17.36	20.43	14.19	5.81	7.47	7.27
OsR498G1018856000.01	7.53	0.753	-3.28771	2.10E-48	1.92E-48	7.62	6.56	8.41	1.12	0.39	0.75
OsR498G1018858200.01	12.456	2.28	-2.57248	8.96E-114	5.14E-114	14.67	11.39	11.31	1.77	2.53	2.54
OsR498G1018861400.01	5.866	58.213	3.295006	0	0	6.68	5.62	5.3	56.29	56.7	61.65
OsR498G1018861800.01	0.266	4.226	3.960326	8.14E-69	6.18E-69	0.19	0.24	0.37	4.54	4.34	3.8
OsR498G1018863100.01	0.036	2.786	6.334381	4.87E-39	4.90E-39	0	0.06	0.05	3.04	2.66	2.66
OsR498G1018863400.01	30.353	5.476	-2.48132	0	0	30.43	30.35	30.28	5.66	5.39	5.38
OsR498G1018865300.01	7.236	22.89	1.657155	4.60E-241	1.66E-241	7.14	6.88	7.69	24.16	22.08	22.43
OsR498G1018865700.01	3.46	0.52	-2.81066	1.81E-38	1.83E-38	3.42	4.03	2.93	0.4	0.7	0.46
OsR498G1018872900.01	1.326	12.373	3.217887	0	0	1.38	1.29	1.31	12.45	12.07	12.6
OsR498G1018874400.01	0.41	8.5	4.382047	6.29E-225	2.38E-225	0.2	0.58	0.45	8.77	8.87	7.86
OsR498G1018874800.01	16.553	71.546	2.11006	0	0	17.7	16.68	15.28	70.98	70.81	72.85
OsR498G1018878300.01	7.906	31.46	1.982763	0	0	7.69	8.19	7.84	33.22	31.56	29.6
OsR498G1018879300.01	0.176	0.01	-4.28793	4.19E-06	8.62E-06	0.13	0.22	0.18	0	0.03	0
OsR498G1018881800.01	0.186	0.01	-4.4838	7.49E-07	1.49E-06	0.21	0.24	0.11	0.03	0	0
OsR498G1018893600.01	0.623	0.063	-3.30009	2.27E-16	3.32E-16	0.53	0.58	0.76	0.02	0.15	0.02
OsR498G1018898300.01	0.236	0.763	1.652737	4.84E-06	1.00E-05	0.08	0.22	0.41	0.69	0.94	0.66
OsR498G1018898500.01	2.606	6.036	1.201141	3.13E-25	3.81E-25	3.05	2.13	2.64	6.92	5.37	5.82
OsR498G1018898700.01	0.533	0.15	-1.98409	4.71E-08	8.86E-08	0.42	0.6	0.58	0.13	0.17	0.15
OsR498G1018899100.01	0.656	1.416	1.06873	8.10E-09	1.48E-08	0.7	0.7	0.57	1.65	1.4	1.2
OsR498G1018903500.01	0.586	0.236	-1.29727	0.0002081	0.0004753	0.33	0.8	0.63	0.22	0.11	0.38
OsR498G1018906500.01	324.11	145.3	-1.16131	0	0	334.8	323.19	314.4	146.79	145.34	143.78

OsR498G1018912700.01	1.813	0.296	-2.6066	2.52E-22	3.25E-22	1.88	1.74	1.82	0.34	0.28	0.27
OsR498G1018914100.01	8.256	39.246	2.266383	0	0	9.03	8.06	7.68	38.02	39.86	39.86
OsR498G1018920300.01	0.103	1.21	3.499192	4.83E-06	9.99E-06	0.31	0	0	1.64	0.5	1.49
OsR498G1018923400.01	0.02	0.313	4.03976	1.16E-08	2.14E-08	0.03	0	0.03	0.29	0.3	0.35
OsR498G1018923600.01	19.643	43.16	1.1244	1.54E-65	1.20E-65	20.1	20.11	18.72	43.47	39.64	46.37
OsR498G1018931200.01	1.263	2.756	1.168746	1.47E-16	2.15E-16	1.39	1.22	1.18	2.51	2.81	2.95
OsR498G1018934100.01	0.543	0.06	-3.14787	5.98E-16	8.84E-16	0.48	0.54	0.61	0.08	0.08	0.02
OsR498G1018936200.01	3.37	1.376	-1.28582	1.67E-23	2.11E-23	4.17	2.58	3.36	1.42	1.61	1.1
OsR498G1018936400.01	0.246	0.523	1.063607	0.0002819	0.0006502	0.27	0.18	0.29	0.5	0.58	0.49
OsR498G1018937800.01	9.43	4.17	-1.18589	2.13E-37	2.18E-37	9.18	9.53	9.58	3.92	4.13	4.46
OsR498G1018938500.01	2.713	1.003	-1.44427	2.26E-15	3.39E-15	3.61	2.18	2.35	1.2	0.91	0.9
OsR498G1018940100.01	3.58	12.056	1.749642	1.09E-96	6.86E-97	3.24	3.99	3.51	12.47	12.17	11.53
OsR498G1018940300.01	0.633	0.26	-1.28623	1.39E-11	2.32E-11	0.6	0.47	0.83	0.29	0.27	0.22
OsR498G1018940800.01	0.256	2.79	3.406542	7.76E-76	5.57E-76	0.12	0.34	0.31	2.71	3.01	2.65
OsR498G1018950800.01	5.293	1.736	-1.58534	6.46E-31	7.20E-31	4.92	5.36	5.6	1.63	1.92	1.66
OsR498G1018951400.01	7.09	3.456	-1.07072	9.49E-53	8.28E-53	7.24	7.18	6.85	3.28	3.25	3.84
OsR498G1018952600.01	41.173	0.843	-5.62836	0	0	38.3	41.5	43.72	0.96	0.91	0.66
OsR498G1018954400.01	5.543	0.56	-3.30741	7.57E-64	5.97E-64	5.5	5.82	5.31	0.93	0.15	0.6
OsR498G1018957400.01	2.026	0.23	-3.1618	3.55E-16	5.23E-16	2.78	1.61	1.69	0.15	0.16	0.38
OsR498G1018957600.01	4.796	2.126	-1.18851	9.74E-14	1.52E-13	5.09	3.87	5.43	2.21	2.34	1.83
OsR498G1018962600.01	1078.7	142.31	-3.00201	0	0	1113.8	1059.6	1063	137.63	144.44	144.86
OsR498G1018963700.01	4.183	14.853	1.83298	6.55E-112	3.79E-112	3.98	3.92	4.65	15.06	14.51	14.99
OsR498G1018966500.01	11.086	0.423	-4.68734	1.40E-196	5.79E-197	9.85	11.64	11.77	0.4	0.19	0.68
OsR498G1018968700.01	23.743	6.226	-1.98114	6.11E-214	2.39E-214	24.09	23.82	23.32	7.26	6.09	5.33
OsR498G1018969000.01	38.7	5.193	-2.89917	0	0	38.32	39.27	38.51	5.22	5.1	5.26
OsR498G1018975500.01	0.473	0.1	-2.20355	7.61E-10	1.34E-09	0.75	0.52	0.15	0.15	0	0.15
OsR498G1018978600.01	18.566	40.66	1.12808	1.60E-300	4.90E-301	19.24	18.25	18.21	42.52	39.51	39.95
OsR498G1018978800.01	0.706	1.186	1.062298	1.28E-08	2.37E-08	0.66	1.09	0.37	1.09	1.5	0.97
OsR498G1018979500.01	2.143	7.866	1.901881	1.75E-36	1.81E-36	2.24	1.63	2.56	7.22	8.15	8.23
OsR498G1018982300.01	142.71	9.586	-3.89972	0	0	142.52	140.7	144.9	9.86	9.52	9.38
OsR498G1018982900.01	12.583	6.55	-1.06093	6.49E-52	5.71E-52	12.71	12.81	12.23	6.8	6.73	6.12
OsR498G1018985300.01	0.696	0.086	-3.35256	4.12E-17	5.93E-17	0.84	0.65	0.6	0.05	0.05	0.16
OsR498G1018986200.01	3.973	0.306	-3.7784	4.24E-55	3.62E-55	5.01	3.83	3.08	0.19	0.6	0.13
OsR498G1018986700.01	36.576	12.68	-1.56951	3.83E-211	1.51E-211	37.72	34.39	37.62	12.57	12.18	13.29
OsR498G1018991500.01	4.713	1.21	-1.77987	2.83E-38	2.87E-38	4.9	4.1	5.14	1.64	1.03	0.96
OsR498G1018994500.01	86.19	185.33	1.201154	0	0	88.95	86.74	82.88	184.88	186.26	184.84
OsR498G1018996000.01	7.176	3.53	-1.03241	5.10E-18	7.19E-18	7.12	7.17	7.24	3.2	3.93	3.46
OsR498G1018998500.01	0.126	0.383	1.575813	0.0006066	0.0014356	0.19	0.12	0.07	0.43	0.4	0.32
OsR498G1018998800.01	54.193	11.896	-2.24151	0	0	56.87	54.36	51.35	12.69	11.65	11.35
OsR498G1018999600.01	1.666	0.35	-2.2557	2.58E-08	4.81E-08	1.48	1.65	1.87	0.47	0.29	0.29
OsR498G1019002900.01	1.476	4.98	1.744562	2.36E-38	2.40E-38	1.64	1.24	1.55	4.99	5.35	4.6
OsR498G1019004000.01	1.303	0.476	-1.47269	2.42E-08	4.49E-08	1.21	1.62	1.08	0.33	0.55	0.55
OsR498G1019004800.01	224.89	47.79	-2.2655	0	0	232.73	227.89	214.1	48.01	48.41	46.95
OsR498G1019005800.01	0.143	1.063	2.876261	6.13E-10	1.08E-09	0.12	0.13	0.18	0.63	0.9	1.66
OsR498G1019008200.01	100.42	18.363	-2.47582	0	0	108.12	89.15	104	20.44	19.09	15.56
OsR498G1019008700.01	0.066	0.41	2.54226	1.24E-06	2.49E-06	0.03	0	0.17	0.28	0.55	0.4
OsR498G1019010900.01	0.56	0.166	-1.72577	4.19E-06	8.64E-06	0.5	0.52	0.66	0.13	0.13	0.24
OsR498G1019021500.01	54.066	14.47	-1.90502	0	0	54.45	53.73	54.02	15.82	12.7	14.89
OsR498G1019030000.01	1.036	15.743	3.921159	0	0	1.18	1.02	0.91	16.4	16.13	14.7
OsR498G1019031400.01	15.493	5.653	-1.45676	2.31E-110	1.35E-110	15.56	15.42	15.5	4.84	6.17	5.95
OsR498G1019033100.01	1.42	0.39	-1.87004	1.46E-13	2.28E-13	1.23	1.68	1.35	0.42	0.25	0.5
OsR498G1019033400.01	0	1.35	8.209685	8.95E-32	9.87E-32	0	0	0	1.3	1.17	1.58
OsR498G1019034900.01	17.786	56.97	1.675591	0	0	18.34	17.49	17.53	61.24	55.88	53.79
OsR498G1019036300.01	751.2	30.456	-4.65074	0	0	781.18	731.39	741	31.88	29.6	29.89
OsR498G1019043400.01	3.586	0.216	-4.07175	4.18E-27	4.94E-27	3.88	3.9	2.98	0	0.37	0.28
OsR498G1019047400.01	0.493	18.236	5.197563	0	0	0.23	0.46	0.79	19.9	18.1	16.71
OsR498G1019048900.01	21.153	43.433	1.023704	1.85E-196	7.65E-197	20.98	21.56	20.92	42.37	43.78	44.15
OsR498G1019052200.01	6.05	13.643	1.16464	1.46E-69	1.10E-69	5.61	5.44	7.1	14.22	14.51	12.2
OsR498G1019053700.01	0	0.956	6.87265	4.68E-15	7.06E-15	0	0	0	0.99	1.07	0.81
OsR498G1019056900.01	0.633	0.136	-2.73283	5.97E-08	1.13E-07	0.88	0.66	0.36	0.05	0.16	0.2
OsR498G1019060300.01	1.216	30.243	4.636982	0	0	1.08	1.46	1.11	30	29.17	31.56
OsR498G1019064500.01	1.9	0.2	-3.23758	4.13E-41	4.06E-41	1.89	1.71	2.1	0.18	0.05	0.37
OsR498G1019065900.01	4.416	0.073	-5.92602	6.63E-31	7.39E-31	5.29	3.46	4.5	0.11	0.11	0
OsR498G1019069600.01	0.15	7.436	5.603093	8.74E-81	6.06E-81	0.19	0.2	0.06	6.83	7.3	8.18
OsR498G1019070200.01	2.08	9.236	2.147385	1.25E-241	4.51E-242	2.11	2.18	1.95	8.95	9.37	9.39
OsR498G1019070300.01	0.013	0.126	2.974277	0.0002637	0.0006075	0.02	0	0.02	0.05	0.23	0.1
OsR498G1019071400.01	0.113	0	-4.86713	3.63E-05	7.88E-05	0.04	0.19	0.11	0	0	0
OsR498G1019073200.01	0.84	13.486	4.031947	6.23E-276	2.04E-276	0.82	1.01	0.69	12.23	14.12	14.11
OsR498G1019074000.01	7.306	0.7	-3.39701	2.75E-71	2.05E-71	6.87	7.03	8.02	0.76	0.61	0.73

OsR498G1019074400.01	1.693	3.82	1.161751	6.45E-09	1.17E-08	2.38	1.41	1.29	4.35	4	3.11
OsR498G1019074700.01	0.053	0.91	4.03976	1.16E-08	2.14E-08	0	0	0.16	1.09	0.78	0.86
OsR498G1019075200.01	3.32	87.43	4.089982	0	0	3.22	4	2.74	91.67	83.52	87.1
OsR498G1019075300.01	0.033	38.57	10.12656	0	0	0	0	0.1	41.9	37.48	36.33
OsR498G1019075700.01	0.063	6.363	6.619704	4.97E-69	3.76E-69	0	0.08	0.11	6.77	5.47	6.85
OsR498G1019075900.01	0.253	35.9	7.126407	0	0	0.35	0.18	0.23	39.34	35.37	32.99
OsR498G1019076300.01	12.856	41.106	1.669528	4.89E-183	2.12E-183	11.12	13.72	13.73	41.77	41.16	40.39
OsR498G1019076500.01	0.836	5.81	2.788221	3.23E-28	3.74E-28	0.6	0.95	0.96	5.71	6.39	5.33
OsR498G1019078700.01	6.123	0.5	-3.61845	8.83E-41	8.72E-41	6.58	6.74	5.05	0.83	0.48	0.19
OsR498G1019078900.01	9.89	36.27	1.866611	4.21E-160	1.97E-160	9.01	10.6	10.06	36.51	36.19	36.11
OsR498G1019079100.01	0.483	19.25	5.308484	1.31E-266	4.40E-267	0.55	0.42	0.48	19.24	18.76	19.75
OsR498G1019079300.01	0.34	2.92	3.105849	3.12E-25	3.81E-25	0.44	0.4	0.18	2.7	3.7	2.36
OsR498G1019080300.01	1.126	0.036	-4.91444	5.36E-30	6.05E-30	1.05	1.13	1.2	0.05	0.06	0
OsR498G1019080500.01	3.196	59.376	4.211663	0	0	3.13	3.46	3	62.51	58.59	57.03
OsR498G1019080700.01	0.17	10.113	5.88211	2.56E-111	1.48E-111	0.25	0.2	0.06	9.68	10.58	10.08
OsR498G1019080900.01	51.166	158.08	1.622451	0	0	50.57	53.05	49.88	167.7	153.3	153.23
OsR498G1019083200.01	169.26	60.623	-1.48857	0	0	169.25	176	162.5	61.47	62.33	58.07
OsR498G1019083900.01	11.65	3.906	-1.59059	7.52E-36	7.87E-36	10.34	11.19	13.42	4.27	3.82	3.63
OsR498G1019085000.01	6.64	14.093	1.081961	5.76E-38	5.87E-38	6.65	6.98	6.29	15.17	13.95	13.16
OsR498G1019088700.01	0	4.27	8.500748	2.50E-37	2.57E-37	0	0	0	1.71	9.43	1.67
OsR498G1019091500.01	2.056	5.82	1.307009	2.65E-48	2.41E-48	1.86	2.09	2.22	5.96	5.56	5.94
OsR498G1019091800.01	10.263	0	-9.78272	4.60E-77	3.27E-77	10.68	11.16	8.95	0	0	0
OsR498G1019092300.01	3.353	1.443	-1.22723	5.57E-13	8.86E-13	3.07	3.36	3.63	1.26	1.64	1.43
OsR498G1019092700.01	3.06	0.066	-5.67449	1.23E-50	1.09E-50	2.8	3.1	3.28	0.09	0.06	0.05
OsR498G1019100000.01	2.27	14.066	2.636051	7.14E-134	3.73E-134	2.52	2.16	2.13	13.35	14.4	14.45
OsR498G1019100500.01	1.79	7.45	2.048901	8.41E-54	7.27E-54	1.6	1.9	1.87	8.37	7.37	6.61
OsR498G1019100900.01	129.09	52.87	-1.28831	0	0	129.36	128.65	129.3	54.48	52.46	51.67
OsR498G1019102500.01	25.883	398.43	3.942142	0	0	23.7	27.1	26.85	408.76	392.33	394.21
OsR498G1019102900.01	16.183	42.05	1.356064	1.15E-118	6.47E-119	15.53	15.73	17.29	42.19	40.42	43.54
OsR498G1019107900.01	0.106	1.1	3.37961	4.44E-17	6.39E-17	0.12	0.12	0.08	1.57	0.42	1.31
OsR498G1019110800.01	0.17	0.41	1.262152	3.27E-05	7.10E-05	0.13	0.16	0.22	0.48	0.34	0.41
OsR498G1019112900.01	5.953	15.366	1.36784	1.07E-146	5.27E-147	4.73	6.62	6.51	15.9	15.31	14.89
OsR498G1019114700.01	18.89	6.95	-1.4046	4.27E-192	1.80E-192	18.12	19.31	19.24	7.14	6.91	6.8
OsR498G1019115200.01	6.083	1.413	-2.04181	6.29E-98	3.92E-98	5.4	6.94	5.91	1.26	1.4	1.58
OsR498G1019115300.01	13.626	1.96	-2.6281	3.36E-281	1.09E-281	11.54	15.51	13.83	1.73	2.1	2.05
OsR498G1019116500.01	0.326	0.103	-1.66068	0.0008332	0.0019932	0.3	0.43	0.25	0.11	0.12	0.08
OsR498G1019117300.01	0.356	0.156	-1.23326	9.95E-05	0.0002222	0.3	0.28	0.49	0.15	0.26	0.06
OsR498G1019118600.01	1.683	5.563	1.677716	1.00E-52	8.73E-53	1.51	1.8	1.74	5.48	5.49	5.72
OsR498G1019122600.01	87.073	206.64	1.242431	0	0	90.67	86.12	84.43	206.21	200.64	213.07
OsR498G1019123300.01	0.316	0.08	-1.96024	0.0009885	0.0023786	0.33	0.25	0.37	0.14	0.1	0
OsR498G1019124000.01	0.13	0.573	2.155237	2.37E-06	4.82E-06	0.09	0.22	0.08	0.65	0.45	0.62
OsR498G1019125100.01	12.573	42.443	1.739592	0	0	12.33	12.79	12.6	41.18	44.13	42.02
OsR498G1019125300.01	23.906	69.46	1.4624	4.70E-205	1.89E-205	22.43	24.76	24.53	76.29	65.88	66.21
OsR498G1019125600.01	5.346	57.2	3.55452	0	0	4.37	5.96	5.71	57.45	57.66	56.49
OsR498G1019125700.01	1.453	3.75	1.371604	1.65E-24	2.04E-24	1.58	1.63	1.15	4.42	3.33	3.5
OsR498G1019131700.01	6.163	30.19	2.302714	0	0	5.89	6.4	6.2	28.69	29.91	31.97
OsR498G1019134400.01	1.7	5.7	1.752478	1.01E-43	9.70E-44	1.26	2.28	1.56	6.12	5.45	5.53
OsR498G1019139800.01	0.963	4.6	2.177801	2.78E-65	2.17E-65	1.3	0.99	0.6	4.11	4.85	4.84
OsR498G1019140000.01	72.636	23.136	-1.69122	0	0	73.31	73.57	71.03	23.11	23.63	22.67
OsR498G1019140200.01	0.153	0.85	2.484545	1.30E-08	2.40E-08	0.15	0.21	0.1	1.08	0.53	0.94
OsR498G1019140600.01	22.813	96.34	2.020279	0	0	21.28	24.44	22.72	94.38	97	97.64
OsR498G1019140700.01	1.02	0.053	-4.28217	9.81E-15	1.49E-14	0.91	1.32	0.83	0.05	0.11	0
OsR498G1019141200.01	3.826	1.836	-1.06096	6.93E-30	7.84E-30	3.87	3.78	3.83	1.94	1.86	1.71
OsR498G1019148500.01	3.786	40.393	3.423898	0	0	3.55	4.02	3.79	43.96	39.51	37.71
OsR498G1019155100.01	13.27	173.46	3.696028	0	0	13.53	13.68	12.6	173.28	174.51	172.58
OsR498G1019156700.01	0.006	0.123	3.966579	2.93E-06	5.99E-06	0	0.02	0	0.15	0.22	0
OsR498G1019158200.01	2.436	14.74	2.587043	1.38E-236	5.05E-237	2.23	2.28	2.8	14.27	15.69	14.26
OsR498G1019160600.01	1.053	0.01	-6.57495	2.96E-24	3.68E-24	1.06	0.98	1.12	0	0	0.03
OsR498G1019162400.01	0.293	2.116	2.869226	2.56E-44	2.44E-44	0.37	0.37	0.14	2.25	2.23	1.87
OsR498G1019163600.01	0.923	0.23	-2.0477	4.53E-09	8.19E-09	0.99	0.72	1.06	0.24	0.32	0.13
OsR498G1019166100.01	0.706	30.703	5.478837	0	0	0.63	0.67	0.82	31.12	31.03	29.96
OsR498G1019172000.01	0.516	0.01	-5.5452	5.29E-13	8.42E-13	0.32	0.64	0.59	0	0	0.03
OsR498G1019181700.01	7.84	2.673	-1.55698	9.67E-71	7.23E-71	7.63	8.64	7.25	2.53	2.4	3.09
OsR498G1019186800.01	11.293	4.923	-1.19674	1.13E-103	6.81E-104	10.99	11.28	11.61	4.96	4.84	4.97
OsR498G1019187100.01	0.503	0.183	-1.46274	3.65E-05	7.93E-05	0.5	0.44	0.57	0.18	0.22	0.15
OsR498G1019187400.01	2.893	0.87	-1.68461	1.72E-20	2.30E-20	3.51	2.41	2.76	0.89	1.04	0.68
OsR498G1019189600.01	6.396	2.903	-1.0623	7.59E-58	6.32E-58	6.37	6.29	6.53	2.8	2.73	3.18
OsR498G1019190000.01	0.286	1.173	2.020967	3.06E-20	4.11E-20	0.19	0.26	0.41	1.07	1.19	1.26
OsR498G1019190600.01	0.5	2.503	2.314241	9.65E-44	9.25E-44	0.49	0.59	0.42	2.56	2.31	2.64

OsR498G1019191600.01	0.34	0.97	1.624723	4.04E-05	8.80E-05	0.38	0.46	0.18	0.81	0.81	1.29
OsR498G1019192600.01	0.26	0.67	1.361688	0.0007094	0.0016883	0.28	0.23	0.27	0.68	0.58	0.75
OsR498G1019196100.01	1.25	0.183	-2.77967	3.98E-20	5.36E-20	1.24	1.52	0.99	0.16	0.13	0.26
OsR498G1019196500.01	33.86	87.876	1.36258	0	0	34.4	33.6	33.58	87.36	89.44	86.83
OsR498G1019198300.01	0.146	0.98	2.724258	1.01E-08	1.85E-08	0.06	0.2	0.18	1.16	0.8	0.98
OsR498G1019198700.01	12.02	85.283	2.821593	0	0	12.93	11.56	11.57	93.57	83.27	79.01
OsR498G1019201200.01	247.31	86.846	-1.50932	0	0	250.27	242.87	248.8	87.84	87.02	85.68
OsR498G1019201700.01	0.836	0.086	-3.30263	1.39E-14	2.12E-14	0.78	0.6	1.13	0.04	0.11	0.11
OsR498G1019201900.01	0.69	0.353	-1.31779	8.19E-09	1.50E-08	0.59	0.79	0.69	0.22	0.49	0.35
OsR498G1019202400.01	1	0.206	-2.06178	6.48E-13	1.03E-12	1.09	0.95	0.96	0.08	0.32	0.22
OsR498G1019203700.01	0.503	78.65	7.265162	0	0	0.49	0.36	0.66	82.73	77.59	75.63
OsR498G1019206100.01	0.44	1.303	1.580328	4.97E-19	6.87E-19	0.52	0.3	0.5	1.2	1.6	1.11
OsR498G1019206600.01	15.39	7.73	-1.00076	1.31E-25	1.58E-25	16.56	16.23	13.38	8.6	6.57	8.02
OsR498G1019206800.01	0.956	12.173	3.657889	4.04E-107	2.39E-107	0.73	1.05	1.09	13.35	12.27	10.9
OsR498G1019209400.01	3.45	0.77	-2.17456	5.90E-36	6.17E-36	3.64	3.56	3.15	0.46	0.77	1.08
OsR498G1019210800.01	0.776	3.873	2.40511	2.84E-51	2.51E-51	0.64	0.85	0.84	3.83	3.79	4
OsR498G1019218100.01	0.013	0.29	4.563322	4.91E-07	9.67E-07	0	0.04	0	0.34	0.15	0.38
OsR498G1019219100.01	42.516	10.96	-1.96877	3.67E-207	1.47E-207	40.36	42.84	44.35	11.36	11.46	10.06
OsR498G1019221100.01	1.636	0.7	-1.23134	1.11E-14	1.70E-14	1.82	1.37	1.72	0.65	0.58	0.87
OsR498G1019222200.01	34.276	3.243	-3.38862	0	0	33.68	33.47	35.68	3.75	3.15	2.83
OsR498G1019223000.01	0.646	0.12	-2.29322	7.19E-10	1.26E-09	0.57	0.52	0.85	0.07	0.21	0.08
OsR498G1019223100.01	0.553	0.16	-1.73202	6.64E-10	1.17E-09	0.53	0.57	0.56	0.11	0.2	0.17
OsR498G1019223400.01	0.623	0.23	-1.42888	1.36E-05	2.89E-05	0.7	0.5	0.67	0.13	0.43	0.13
OsR498G1019225500.01	118.86	693.24	2.540748	0	0	121.08	117.97	117.5	693.31	684.26	702.15
OsR498G1019226300.01	6.733	16.536	1.271271	4.97E-121	2.75E-121	7.44	6.29	6.47	15.27	17.2	17.14
OsR498G1019228300.01	0	0.436	4.792509	6.95E-05	0.0001537	0	0	0	0.43	0.73	0.15
OsR498G1019228500.01	584.81	124.67	-2.24214	0	0	614.08	579.51	560.8	129.89	114.18	129.94
OsR498G1019230700.01	69.423	25.44	-1.46204	2.62E-139	1.34E-139	70.28	68.65	69.34	28.53	23.29	24.5
OsR498G1019232500.01	0.526	0.12	-1.75165	1.14E-05	2.42E-05	0.46	0.48	0.64	0.13	0.13	0.1
OsR498G1019235000.01	15.446	3.866	-2.00116	2.14E-189	9.08E-190	15.33	15.11	15.9	3.97	3.69	3.94
OsR498G1019235500.01	9.616	30.57	1.704311	4.68E-273	1.54E-273	9.76	9.32	9.77	30.11	29.88	31.72
OsR498G1019236000.01	27.383	67.536	1.312016	0	0	25.56	29.06	27.53	65.63	67.59	69.39
OsR498G1019237200.01	0.22	0.543	1.312779	3.89E-05	8.48E-05	0.22	0.31	0.13	0.54	0.54	0.55
OsR498G1019239100.01	14.13	5.92	-1.24387	2.28E-41	2.24E-41	15.76	12.81	13.82	6.08	6.1	5.58
OsR498G1019242000.01	25.58	57.743	1.175766	8.60E-256	2.98E-256	24.8	25.83	26.11	59.57	56.31	57.35
OsR498G1019242100.01	0.45	1.623	1.841071	6.48E-17	9.37E-17	0.34	0.52	0.49	1.07	1.74	2.06
OsR498G1019243900.01	7.876	26.253	1.733589	0	0	6.64	8.08	8.91	27.52	25.76	25.48
OsR498G1019246300.01	91.416	37.92	-1.27519	0	0	95.94	90.21	88.1	41.11	37.4	35.25
OsR498G1019249000.01	4.796	1.186	-2.02553	2.06E-37	2.11E-37	5.07	5	4.32	1.12	1.47	0.97
OsR498G1019249900.01	1.853	5.033	1.413218	4.57E-24	5.70E-24	1.76	1.85	1.95	4.82	5.02	5.26
OsR498G1019250500.01	0.083	0.3	1.865731	1.49E-05	3.16E-05	0.09	0.07	0.09	0.2	0.38	0.32
OsR498G1019250800.01	7.423	20.21	1.661086	4.40E-170	1.99E-170	7.63	6.62	8.02	21.1	19.56	19.97
OsR498G1019252100.01	0.226	0	-4.55479	0.000204	0.0004654	0.1	0.33	0.25	0	0	0
OsR498G1019253100.01	3.533	138.65	5.294754	0	0	3.34	3.81	3.45	135.21	139.96	140.79
OsR498G1019253800.01	128.36	257.8	1.005205	0	0	127.38	128.96	128.7	247.42	266.81	259.18
OsR498G1019253900.01	24.833	6.766	-1.94654	1.12E-303	3.40E-304	23.94	26.34	24.22	7.13	6.68	6.49
OsR498G1019255800.01	4.103	16.37	1.995965	2.27E-248	8.00E-249	3.9	3.93	4.48	15.5	16.71	16.9
OsR498G1019256000.01	24.156	205.84	3.085276	0	0	23.73	25.22	23.52	202.23	204.33	210.97
OsR498G1019256300.01	0.563	0.04	-3.86713	7.00E-08	1.32E-07	0.43	0.6	0.66	0	0.06	0.06
OsR498G1019256600.01	17.886	7.273	-1.308	4.30E-46	4.01E-46	19.43	17.42	16.81	7.41	8.13	6.28
OsR498G1019258300.01	4.653	0.316	-3.86713	9.69E-114	5.56E-114	4.71	4.57	4.68	0.25	0.18	0.52
OsR498G1019260300.01	7.09	101.02	3.8315	0	0	7.05	7.32	6.9	106.47	98.25	98.34
OsR498G1019262800.01	5.32	130.84	4.587648	0	0	4.77	5.83	5.36	130.05	129.79	132.69
OsR498G1019263400.01	1.173	9.566	3.023272	2.04E-267	6.82E-268	1.28	1.06	1.18	9.46	9.57	9.67
OsR498G1019269700.01	14.213	6.916	-1.04542	6.00E-46	5.61E-46	15.08	14.02	13.54	6.27	7.94	6.54
OsR498G1119270400.01	0	0.73	6.499192	3.21E-12	5.23E-12	0	0	0	0.61	0.61	0.97
OsR498G1119271000.01	11.586	1.97	-2.58691	3.25E-98	2.02E-98	11.71	10.85	12.2	1.95	2.01	1.95
OsR498G1119271100.01	1.436	0.336	-2.1032	1.18E-07	2.26E-07	1.62	1.28	1.41	0.42	0.34	0.25
OsR498G1119279200.01	3.013	7.293	1.26478	3.02E-45	2.85E-45	2.47	3.24	3.33	6.24	8.31	7.33
OsR498G1119279400.01	0.23	1.403	2.607974	5.21E-34	5.58E-34	0	0.69	0	1.45	0.67	2.09
OsR498G1119284300.01	19.296	8.716	-1.14825	2.27E-192	9.53E-193	18.96	19.7	19.23	8.54	8.97	8.64
OsR498G1119284600.01	1.103	0.396	-1.51245	5.91E-09	1.07E-08	1.33	1.04	0.94	0.47	0.34	0.38
OsR498G1119285800.01	0.783	0.21	-1.9196	3.40E-05	7.37E-05	0.59	0.71	1.05	0.29	0.27	0.07
OsR498G1119287500.01	12.62	26.013	1.035374	2.47E-297	7.65E-298	9.65	14.42	13.79	23.24	27.44	27.36
OsR498G1119289600.01	60.146	12.973	-2.25103	0	0	64.47	57.1	58.87	12.39	13.12	13.41
OsR498G1119291800.01	2.276	22.206	3.28888	0	0	1.77	2.44	2.62	22.16	22.26	22.2
OsR498G1119292200.01	49.146	19.603	-1.15838	1.22E-274	4.01E-275	43.61	52.71	51.12	18.64	20.37	19.8
OsR498G1119293000.01	27.466	400.98	3.845993	0	0	26.98	27.3	28.12	406.31	384.77	411.85
OsR498G1119293100.01	1.006	2.76	1.348534	1.98E-30	2.22E-30	0.8	1.08	1.14	2.5	2.83	2.95

OsR498G1119296300.01	12.156	1.776	-2.77753	4.33E-217	1.68E-217	12.98	11.1	12.39	1.4	2.15	1.78
OsR498G1119296700.01	0.266	0.743	1.466025	0.0001042	0.0002331	0.2	0.31	0.29	0.56	1.1	0.57
OsR498G1119298400.01	4.696	1.29	-1.91133	5.01E-75	3.61E-75	4.33	4.92	4.84	1.13	1.4	1.34
OsR498G1119301300.01	3.243	8.016	1.308422	7.20E-63	5.73E-63	3.5	3.16	3.07	7.8	8.4	7.85
OsR498G1119303200.01	0	0.313	5.432077	8.26E-07	1.64E-06	0	0	0	0.22	0.41	0.31
OsR498G1119303400.01	0.026	0.263	3.209685	8.46E-15	1.29E-14	0.03	0.03	0.02	0.28	0.3	0.21
OsR498G1119304900.01	11.67	5.156	-1.18792	2.08E-45	1.96E-45	11.79	12.11	11.11	4.93	5.06	5.48
OsR498G1119309800.01	3.606	0.336	-3.37809	4.43E-33	4.79E-33	3.96	3.46	3.4	0.32	0.24	0.45
OsR498G1119310500.01	0.24	0.78	1.596153	1.13E-05	2.40E-05	0.21	0.18	0.33	0.82	0.77	0.75
OsR498G1119313200.01	24.873	11.366	-1.17533	7.19E-109	4.22E-109	24.73	25.55	24.34	10.94	11.01	12.15
OsR498G1119315600.01	1.35	0.513	-1.40591	5.51E-12	9.06E-12	1.54	1.1	1.41	0.46	0.6	0.48
OsR498G1119321500.01	171.31	14.936	-3.56369	0	0	176.03	163.75	174.1	15.61	13.08	16.12
OsR498G1119322300.01	512.37	45.833	-3.44088	0	0	533.14	505.81	498.2	50.42	43.35	43.73
OsR498G1119323300.01	105.29	1.076	-6.39309	0	0	102.78	108.5	104.6	0.83	1.24	1.16
OsR498G1119323800.01	60.423	166.94	1.496723	0	0	61.38	60.67	59.22	168.61	161.82	170.38
OsR498G1119323900.01	8.576	2.386	-1.88532	1.59E-43	1.53E-43	10.19	7.7	7.84	2.22	2.15	2.79
OsR498G1119324200.01	0.243	0.076	-1.60753	0.0002837	0.0006544	0.02	0.22	0.49	0	0	0.23
OsR498G1119324400.01	5.623	2.096	-1.27167	8.24E-43	7.98E-43	5.29	5.93	5.65	1.87	2.24	2.18
OsR498G1119325200.01	19.04	115.36	2.593824	0	0	17.85	20.71	18.56	121.38	106.44	118.27
OsR498G1119327000.01	0.413	2.62	2.625841	7.26E-21	9.65E-21	0.41	0.23	0.6	2.55	2.55	2.76
OsR498G1119327400.01	2.276	0.613	-1.89884	3.68E-15	5.55E-15	2.37	2.07	2.39	0.86	0.26	0.72
OsR498G1119329400.01	0.066	0.406	3.219875	2.54E-11	4.25E-11	0.07	0.08	0.05	0.35	0.42	0.45
OsR498G1119329500.01	0.393	6.356	3.951656	2.63E-75	1.90E-75	0.33	0.26	0.59	6.35	6.74	5.98
OsR498G1119330000.01	0.183	1.186	2.624723	2.46E-13	3.88E-13	0.17	0.13	0.25	1.33	1.17	1.06
OsR498G1119330200.01	11.96	39.166	1.69747	0	0	10.49	12.79	12.6	41.29	37.37	38.84
OsR498G1119330300.01	0.95	0.373	-1.52409	1.28E-10	2.20E-10	0.85	1.19	0.81	0.3	0.43	0.39
OsR498G1119330600.01	0.226	0.05	-2.13076	0.0001836	0.0004177	0.16	0.35	0.17	0.08	0	0.07
OsR498G1119331000.01	0.613	0.076	-3.02633	5.97E-13	9.51E-13	0.82	0.57	0.45	0.08	0.06	0.09
OsR498G1119332100.01	10.84	2.186	-2.30968	1.09E-153	5.24E-154	11.54	10.17	10.81	2.16	2.71	1.69
OsR498G1119333300.01	0.193	0.04	-2.37167	0.0009285	0.0022289	0.18	0.23	0.17	0	0.08	0.04
OsR498G1119336000.01	19.166	6.123	-1.65462	9.11E-82	6.27E-82	18.44	19.65	19.41	5.87	6.21	6.29
OsR498G1119339100.01	1.24	0.313	-2.22327	0.0001374	0.00031	1.35	1.24	1.13	0.28	0.66	0
OsR498G1119339400.01	3.943	1.086	-1.66285	3.56E-15	5.36E-15	4.06	3.71	4.06	1.04	0.99	1.23
OsR498G1119347500.01	3.196	6.486	1.020753	4.52E-41	4.45E-41	3.48	3.22	2.89	6.93	6.88	5.65
OsR498G1119348100.01	0.196	0.693	1.762226	0.0001037	0.0002319	0.3	0.06	0.23	0.68	1.08	0.32
OsR498G1119348600.01	0.91	0.1	-3.20717	1.76E-24	2.18E-24	0.77	0.9	1.06	0.14	0.12	0.04
OsR498G1119349400.01	5.973	2.356	-1.28901	2.08E-20	2.79E-20	5.58	6.36	5.98	2.47	2.49	2.11
OsR498G1119350300.01	4.146	0.206	-4.19054	1.51E-53	1.30E-53	3.79	4.31	4.34	0.35	0.18	0.09
OsR498G1119352100.01	0.146	31.48	7.733981	0	0	0.09	0.12	0.23	33.24	29.89	31.31
OsR498G1119354300.01	230.58	11.44	-4.33465	0	0	237.1	232.95	221.7	11.77	11.33	11.22
OsR498G1119356300.01	1.03	5.17	2.308211	6.82E-24	8.54E-24	0.94	0.99	1.16	5.47	4.52	5.52
OsR498G1119359600.01	19.866	95.69	2.283847	0	0	18.1	20.97	20.53	89.54	98.7	98.83
OsR498G1119360200.01	0.166	0.503	1.509387	3.97E-05	8.64E-05	0.1	0.13	0.27	0.52	0.4	0.59
OsR498G1119361700.01	10.226	21.606	1.081066	2.93E-64	2.31E-64	11.71	8.7	10.27	22.33	21.5	20.99
OsR498G1119362400.01	5.616	14.51	1.437904	9.00E-117	5.09E-117	5.71	5.54	5.6	14.1	14.71	14.72
OsR498G1119363500.01	0.146	0.846	2.658184	2.38E-11	3.98E-11	0.04	0.2	0.2	1.31	0.6	0.63
OsR498G1119364000.01	0.363	1.386	1.932845	9.46E-10	1.67E-09	0.16	0.38	0.55	1.84	1.41	0.91
OsR498G1119365300.01	0	0.343	5.359522	1.50E-06	3.02E-06	0	0	0	0.22	0.43	0.38
OsR498G1119366100.01	8.906	38.053	2.08152	3.52E-142	1.78E-142	10.53	8.04	8.15	38.8	38.24	37.12
OsR498G1119366500.01	1.996	5.953	1.594171	2.72E-48	2.49E-48	2.04	1.86	2.09	6.13	5.71	6.02
OsR498G1119370400.01	5.406	16.15	1.556683	9.88E-141	5.02E-141	5.36	5.76	5.1	16.22	15.84	16.39
OsR498G1119371300.01	0.013	3.936	8.025982	4.61E-118	2.59E-118	0.02	0	0.02	3.31	4.13	4.37
OsR498G1119371800.01	0.043	0.31	2.847115	2.76E-06	5.63E-06	0.1	0.03	0	0.23	0.3	0.4
OsR498G1119374800.01	3.073	28.023	3.141603	0	0	2.91	2.79	3.52	26.98	28	29.09
OsR498G1119375500.01	0.136	1.19	3.123103	2.03E-18	2.84E-18	0.15	0.12	0.14	1.5	1.18	0.89
OsR498G1119377100.01	62.22	130.15	1.044692	0	0	62.45	61.07	63.14	127.31	129.02	134.12
OsR498G1119377800.01	0.103	0.613	2.554333	1.58E-07	3.03E-07	0.13	0.05	0.13	0.72	0.61	0.51
OsR498G1119380000.01	3.726	10.023	1.421876	6.05E-44	5.79E-44	3.93	3.72	3.53	11.4	9.66	9.01
OsR498G1119380200.01	11.973	47.68	1.948023	0	0	11.52	12.23	12.17	44.73	49.29	49.02
OsR498G1119382000.01	22.346	1.746	-3.61167	0	0	22.81	22.59	21.64	1.82	1.8	1.62
OsR498G1119384300.01	13.723	33.073	1.285819	4.33E-186	1.85E-186	13.84	13.67	13.66	32.25	33.43	33.54
OsR498G1119390000.01	6.52	17.343	1.398915	9.47E-56	8.02E-56	7.36	5.61	6.59	16.36	17.74	17.93
OsR498G1119390600.01	7.536	22.006	1.563227	8.97E-72	6.63E-72	7.23	7.9	7.48	20.94	22.4	22.68
OsR498G1119394100.01	13.426	31.966	1.026307	1.07E-149	5.23E-150	13.61	13.57	13.1	29.65	31.44	34.81
OsR498G1119398200.01	0.45	73.41	7.332382	0	0	0.53	0.37	0.45	80.84	68.84	70.55
OsR498G1119402100.01	9.75	24.143	1.304758	2.26E-167	1.03E-167	9.74	9.55	9.96	23.5	24.14	24.79
OsR498G1119404600.01	0.06	0.326	2.54226	0.0003732	0.0008685	0.06	0.12	0	0.23	0.29	0.46
OsR498G1119405700.01	0.093	1.233	3.732782	3.68E-41	3.62E-41	0.11	0.08	0.09	1.12	1.22	1.36
OsR498G1119406500.01	334.62	158.08	-1.08924	0	0	330.49	342.84	330.5	158.7	149.32	166.21

OsR498G1119406700.01	2.16	1.006	-1.10056	1.35E-10	2.32E-10	2.13	2.5	1.85	0.92	1.22	0.88
OsR498G1119408900.01	6.083	24.72	2.014885	2.45E-149	1.20E-149	6.7	6.15	5.4	23.12	25.12	25.92
OsR498G1119409200.01	0.316	20.293	6.052004	8.64E-171	3.89E-171	0.38	0.31	0.26	18.03	21.81	21.04
OsR498G1119410000.01	0.283	0.733	1.387683	2.59E-05	5.58E-05	0.23	0.27	0.35	0.71	0.79	0.7
OsR498G1119411100.01	0.19	0.046	-2.3932	2.95E-05	6.38E-05	0.19	0.2	0.18	0.05	0.04	0.05
OsR498G1119413400.01	611.25	223.35	-1.46992	0	0	623.7	598.67	611.4	222.72	224.36	222.96
OsR498G1119414400.01	1.706	4.203	1.305033	4.16E-24	5.19E-24	1.78	1.71	1.63	4.32	4.11	4.18
OsR498G1119418100.01	4.293	0.02	-7.86111	8.60E-52	7.57E-52	5.14	4.27	3.47	0	0.06	0
OsR498G1119419700.01	51.88	14.286	-1.80624	0	0	50.83	51.81	53	13.83	14.74	14.29
OsR498G1119420600.01	0.423	0.016	-4.20817	7.92E-06	1.66E-05	0.51	0.43	0.33	0.05	0	0
OsR498G1119421800.01	20.45	6.61	-1.64237	5.90E-125	3.20E-125	20.75	20.83	19.77	6.54	6.27	7.02
OsR498G1119425200.01	0	3.53	9.410447	2.19E-61	1.77E-61	0	0	0	4.27	3.21	3.11
OsR498G1119426800.01	0.06	0.22	1.914229	0.000621	0.0014712	0.12	0.03	0.03	0.45	0.1	0.11
OsR498G1119427900.01	0	0.473	6.084154	1.05E-09	1.85E-09	0	0	0	0.34	0.78	0.3
OsR498G1119433200.01	4.333	19.51	2.164773	2.12E-120	1.18E-120	3.51	4.91	4.58	20.82	19.42	18.29
OsR498G1119433900.01	12.633	1.086	-3.37737	8.52E-253	2.97E-253	13.93	11.55	12.42	1.21	1.09	0.96
OsR498G1119434100.01	6.043	2.07	-1.5489	3.74E-30	4.21E-30	6.6	5.91	5.62	2.54	1.99	1.68
OsR498G1119437300.01	0.08	0.673	3.03976	6.58E-17	9.51E-17	0.02	0.16	0.06	0.63	0.79	0.6
OsR498G1119440800.01	5.146	182.36	5.15121	0	0	4.86	5.68	4.9	171.8	188.56	186.72
OsR498G1119444400.01	4.396	27.243	2.627315	2.40E-247	8.48E-248	4.39	4.56	4.24	28.27	23.99	29.47
OsR498G1119445300.01	0.28	2.72	3.287688	8.79E-23	1.12E-22	0.28	0.36	0.2	2.89	2.61	2.66
OsR498G1119446100.01	0.51	1.076	1.076754	1.61E-08	2.98E-08	0.53	0.58	0.42	1.14	1.02	1.07
OsR498G1119449200.01	52.786	17.743	-1.48182	1.83E-155	8.70E-156	51.65	59.97	46.74	20.08	16.07	17.08
OsR498G1119450600.01	3.103	13.06	2.058924	2.20E-193	9.22E-194	2.76	2.98	3.57	13.35	12.9	12.93
OsR498G1119451500.01	17.833	64.566	1.85728	0	0	17.47	17.23	18.8	64.54	64.9	64.26
OsR498G1119453700.01	58.913	162.26	1.456296	0	0	59.05	58.55	59.14	159.97	163.5	163.32
OsR498G1119461400.01	11.543	32.553	1.60137	0	0	12.07	11.17	11.39	31.7	31.72	34.24
OsR498G1119462300.01	0	0.85	5.361688	1.47E-06	2.96E-06	0	0	0	1.01	0.9	0.64
OsR498G1119463100.01	22.756	12.336	-1.03402	3.80E-82	2.60E-82	22.22	22.55	23.5	13.06	11.62	12.33
OsR498G1119465500.01	0.576	0.073	-2.92724	1.18E-15	1.76E-15	0.67	0.61	0.45	0.04	0.04	0.14
OsR498G1119465700.01	13.456	28.996	1.113892	3.33E-260	1.14E-260	13.68	12.99	13.7	29.9	28.35	28.74
OsR498G1119468000.01	6.763	2.153	-1.5452	6.26E-16	9.26E-16	5.95	6.81	7.53	2.57	2.21	1.68
OsR498G1119469900.01	6.8	13.276	1.005885	2.08E-54	1.78E-54	7.92	6.27	6.21	13.08	13.56	13.19
OsR498G1119470500.01	2.293	6.826	1.569684	5.45E-51	4.84E-51	2.48	2.07	2.33	7.38	6.23	6.87
OsR498G1119476600.01	629.58	140.92	-2.16871	0	0	606.29	644.02	638.4	148.64	133.07	141.04
OsR498G1119478500.01	2.696	0.933	-1.53627	2.26E-13	3.56E-13	3.07	2.45	2.57	0.79	1.26	0.75
OsR498G1119484800.01	2.803	1.146	-1.56424	2.23E-63	1.77E-63	2.65	2.72	3.04	0.98	0.91	1.55
OsR498G1119485200.01	1.923	4.346	1.133052	4.28E-26	5.14E-26	2.12	1.88	1.77	4.32	4.7	4.02
OsR498G1119486400.01	9239.3	3828	-1.30703	0	0	8371.9	9679.9	9666	3944.4	3727.8	3811.9
OsR498G1119486600.01	6.52	2.37	-1.46093	9.00E-55	7.69E-55	6.18	6.65	6.73	2.77	2.31	2.03
OsR498G1119487000.01	7.576	27.33	1.837155	0	0	7.33	7.52	7.88	26.84	28.43	26.72
OsR498G1119488200.01	0.376	1.02	1.429466	1.55E-15	2.31E-15	0.29	0.34	0.5	0.98	0.95	1.13
OsR498G1119488900.01	1.223	0.226	-2.55089	2.85E-40	2.82E-40	1.07	1.51	1.09	0.2	0.27	0.21
OsR498G1119489300.01	2.63	0.02	-6.91417	5.10E-113	2.94E-113	2.36	2.55	2.98	0	0.03	0.03
OsR498G1119492700.01	0.653	0.026	-4.5452	2.47E-28	2.86E-28	0.71	0.65	0.6	0.03	0	0.05
OsR498G1119493500.01	0.1	0.013	-2.96024	0.000243	0.0005585	0.13	0.1	0.07	0.02	0.02	0
OsR498G1119494900.01	12.4	2.363	-2.42603	0	0	12.25	12.76	12.19	2.24	2.61	2.24
OsR498G1119500400.01	0.16	0.03	-2.37528	0.0009088	0.0021805	0.18	0.16	0.14	0	0.06	0.03
OsR498G1119504300.01	1.14	3.133	1.446266	9.35E-21	1.24E-20	1.24	0.85	1.33	2.94	2.91	3.55
OsR498G1119509000.01	24.22	7.836	-1.65959	1.93E-81	1.33E-81	21.77	25.89	25	8.47	7.47	7.57
OsR498G1119518700.01	1.066	0.103	-3.35256	2.72E-13	4.30E-13	0.8	1.32	1.08	0.15	0.05	0.11
OsR498G1119519800.01	19.09	41.093	1.096424	7.24E-159	3.40E-159	19.52	18.08	19.67	39.45	43.7	40.13
OsR498G1119520500.01	0.276	0.116	-1.14466	3.49E-05	7.58E-05	0.32	0.3	0.21	0.14	0.05	0.16
OsR498G1119522500.01	0.47	0.13	-1.88081	7.54E-07	1.50E-06	0.41	0.61	0.39	0.05	0.2	0.14
OsR498G1119522900.01	45.496	272.18	2.582696	0	0	46.39	45.19	44.91	264.29	275.72	276.53
OsR498G1119523700.01	0.97	8.143	2.952929	1.58E-135	8.20E-136	0.86	1.01	1.04	7.35	8.55	8.53
OsR498G1119524400.01	0.91	6.533	2.83494	2.74E-58	2.27E-58	0.96	0.95	0.82	6.86	6.85	5.89
OsR498G1119525200.01	0	3.603	8.697972	1.30E-41	1.27E-41	0	0	0	3.68	3.69	3.44
OsR498G1119526200.01	0.31	13.423	5.433222	2.04E-301	6.23E-302	0.43	0.23	0.27	13.28	13.24	13.75
OsR498G1119527200.01	7.3	17.186	1.236527	3.93E-139	2.01E-139	7.69	7.56	6.65	18.1	16.7	16.76
OsR498G1119529700.01	1.303	0.183	-2.83278	5.50E-79	3.86E-79	1.22	1.4	1.29	0.16	0.21	0.18
OsR498G1119529900.01	2.246	13.936	2.624723	4.03E-221	1.54E-221	2.05	2.37	2.32	14.09	13.48	14.24
OsR498G1119531400.01	41.79	153.15	1.86798	0	0	42.35	39.86	43.16	148.63	154.91	155.92
OsR498G1119536300.01	0.66	4.26	2.67893	4.21E-71	3.14E-71	0.7	0.48	0.8	4.4	4.13	4.25
OsR498G1119550500.01	25.476	12.806	-1.00641	5.90E-30	6.66E-30	27.04	24.91	24.48	13.36	12.1	12.96
OsR498G1119551000.01	11.49	4.653	-1.30434	5.98E-86	4.01E-86	11.11	12.33	11.03	4.67	4.06	5.23
OsR498G1119553700.01	11.323	99.863	3.167783	0	0	11.21	11.12	11.64	103.34	97.51	98.74
OsR498G1119554100.01	3.103	11.94	1.945773	9.75E-128	5.23E-128	3.15	3.39	2.77	13.72	11.62	10.48
OsR498G1119556300.01	4.52	1.596	-1.51	3.19E-14	4.92E-14	4.59	4.92	4.05	1.67	1.75	1.37

OsR498G1119560700.01	1.393	0.553	-1.35617	0.0001002	0.0002239	1.26	1.06	1.86	0.6	0.53	0.53
OsR498G1119561000.01	4.816	1.826	-1.40081	1.23E-32	1.34E-32	5.55	5.03	3.87	1.89	2.05	1.54
OsR498G1119563500.01	0.01	0.443	5.287688	7.27E-11	1.24E-10	0	0	0.03	0.69	0.36	0.28
OsR498G1119567600.01	24.186	7.1	-1.77592	4.51E-238	1.63E-238	23.39	25.26	23.91	6.57	6.94	7.79
OsR498G1119569000.01	12.213	36.17	1.565556	0	0	11.75	12.8	12.09	35.73	35.72	37.06
OsR498G1119569800.01	3.216	7.59	1.231808	4.42E-38	4.50E-38	3.29	2.91	3.45	7.64	7.08	8.05
OsR498G1119570600.01	0.203	0.843	1.78419	1.11E-06	2.21E-06	0.18	0.22	0.21	0.87	0.65	1.01
OsR498G1119570700.01	0.083	0.606	3.346946	6.40E-13	1.02E-12	0.04	0.21	0	0.74	0.49	0.59
OsR498G1119571200.01	2.54	1.223	-1.08302	1.04E-19	1.42E-19	2.73	2.37	2.52	0.97	1.34	1.36
OsR498G1119571600.01	0.27	0.813	1.567007	1.73E-05	3.69E-05	0.24	0.15	0.42	0.88	0.71	0.85
OsR498G1119573100.01	18.45	3.69	-2.33117	5.26E-196	2.18E-196	18.3	18.23	18.82	3.53	3.44	4.1
OsR498G1119575200.01	14.66	0.783	-4.2306	0	0	14.72	14.35	14.91	0.73	0.9	0.72
OsR498G1119575300.01	1.41	0.346	-2.02027	6.89E-07	1.37E-06	0.95	1.84	1.44	0.35	0.2	0.49
OsR498G1119583000.01	3.25	1.163	-1.52485	9.27E-27	1.10E-26	3.24	3.87	2.64	1.25	1.11	1.13
OsR498G1119585000.01	241.92	83.26	-1.54625	0	0	249.1	224.34	252.3	81.54	83.29	84.95
OsR498G1119592200.01	0.156	4.51	4.829837	1.46E-59	1.20E-59	0.16	0.16	0.15	4.43	4.3	4.8
OsR498G1119592400.01	1.106	27.09	4.71202	0	0	1.31	1.03	0.98	27.44	28.22	25.61
OsR498G1119592500.01	1.313	39.783	4.922506	0	0	1.21	1.5	1.23	38.75	38.4	42.2
OsR498G1119594000.01	2.16	0.473	-2.18487	1.06E-26	1.27E-26	1.83	2.48	2.17	0.36	0.38	0.68
OsR498G1119596600.01	0.31	0.653	1.062128	0.0001924	0.0004384	0.29	0.27	0.37	0.56	0.49	0.91
OsR498G1119597400.01	0.07	0.623	3.003234	2.04E-08	3.78E-08	0	0.09	0.12	0.78	0.53	0.56
OsR498G1119597600.01	0.02	0.616	4.776726	2.31E-20	3.10E-20	0.02	0.02	0.02	0.52	0.68	0.65
OsR498G1119598400.01	30.606	1.73	-4.17034	0	0	41.98	19.11	30.73	0	1.25	3.94
OsR498G1119599000.01	82.256	24.73	-1.76001	0	0	74.72	87.46	84.59	24.61	25.44	24.14
OsR498G1119599500.01	232.74	78.583	-1.58157	0	0	227.38	246.02	224.8	83.71	71.55	80.49
OsR498G1119600000.01	1.006	2.256	1.156274	7.06E-13	1.13E-12	1.01	1.03	0.98	2.06	2.25	2.46
OsR498G1119605600.01	4.236	559.97	7.028942	0	0	4.54	3.7	4.47	568.08	570.39	541.43
OsR498G1119605800.01	11.07	31.903	1.489518	7.11E-166	3.26E-166	11.57	10.16	11.48	32.85	32.18	30.68
OsR498G1119607600.01	94.693	0.223	-8.79881	0	0	91.27	95.43	97.38	0.31	0.17	0.19
OsR498G1119608900.01	0.063	0.516	3.127223	9.61E-05	0.0002145	0.09	0.1	0	0.47	0.45	0.63
OsR498G1119614900.01	0.43	0.96	1.152654	3.43E-06	7.03E-06	0.48	0.35	0.46	0.85	0.88	1.15
OsR498G1119615600.01	7.796	24.1	1.82944	1.47E-283	4.71E-284	7.88	8.12	7.39	23.45	23.79	25.06
OsR498G1119617900.01	3.263	0.33	-3.31074	3.49E-20	4.70E-20	3.36	2.53	3.9	0.49	0.1	0.4
OsR498G1119619100.01	6.513	17.57	1.424468	1.03E-153	4.97E-154	6.46	6.66	6.42	16.86	17.55	18.3
OsR498G1119619600.01	279.46	567.45	1.019758	0	0	274.84	283.26	280.3	549.18	575.56	577.6
OsR498G1119621400.01	10.31	2.1	-2.29979	4.65E-113	2.68E-113	10.55	10.09	10.29	2	2.36	1.94
OsR498G1119622100.01	76.92	35.703	-1.10707	4.08E-274	1.34E-274	75.55	75.49	79.72	35.44	35.5	36.17
OsR498G1119632800.01	18.383	0.366	-5.64674	6.38E-193	2.68E-193	16.11	20	19.04	0.82	0.14	0.14
OsR498G1119633500.01	10.46	4.436	-1.26097	6.24E-27	7.40E-27	10.9	10.14	10.34	5	4.41	3.9
OsR498G1119644200.01	23.706	93.77	1.981569	0	0	23.41	23.24	24.47	92.27	95.37	93.67
OsR498G1119648000.01	1.603	5.316	1.739377	9.43E-79	6.63E-79	1.6	1.56	1.65	5	6.05	4.9
OsR498G1119648200.01	5.776	15.013	1.36612	4.90E-42	4.78E-42	4.71	5.87	6.75	14.88	14.83	15.33
OsR498G1119648600.01	9.99	33.003	1.718921	3.00E-93	1.92E-93	10.89	9.49	9.59	33.2	32.34	33.47
OsR498G1119649900.01	4.166	42.893	3.344321	0	0	3.83	4.15	4.52	42.02	42.42	44.24
OsR498G1119662400.01	2.466	0.253	-3.38526	7.44E-55	6.36E-55	2.65	1.83	2.92	0.03	0.18	0.55
OsR498G1119662800.01	0.843	0.243	-1.91444	8.81E-05	0.0001963	0.49	1.37	0.67	0.35	0.15	0.23
OsR498G1119667700.01	2.046	0.083	-4.70803	6.60E-26	7.96E-26	2.42	1.85	1.87	0	0	0.25
OsR498G1119669800.01	3.323	7.53	1.170344	6.30E-12	1.04E-11	4.05	3.61	2.31	6.54	7.56	8.49
OsR498G1119671000.01	25.113	10.42	-1.28407	1.08E-84	7.28E-85	25.97	23.74	25.63	10.02	10.47	10.77
OsR498G1119677800.01	1.003	0.21	-2.26402	2.19E-11	3.67E-11	1.29	0.99	0.73	0.35	0.08	0.2
OsR498G1119681000.01	5.296	2.416	-1.13712	1.38E-20	1.85E-20	5.56	5.26	5.07	2.48	2.2	2.57
OsR498G1119683100.01	8.086	3.646	-1.15261	7.81E-37	8.06E-37	8.36	7.73	8.17	3.61	3.24	4.09
OsR498G1119686500.01	21.553	45.716	1.088564	0	0	21.49	21.23	21.94	43.66	48.06	45.43
OsR498G1119688900.01	0.843	2.92	1.803462	2.06E-45	1.94E-45	0.73	0.89	0.91	2.71	3.39	2.66
OsR498G1119689900.01	1735.4	485.66	-1.85949	0	0	1664.9	1758	1783	514.98	476.8	465.21
OsR498G1119691400.01	0.273	0.05	-2.76759	6.01E-05	0.0001325	0.36	0.24	0.22	0.1	0.05	0
OsR498G1119698200.01	1.19	0.193	-2.61796	5.56E-28	6.48E-28	1.38	1.12	1.07	0.2	0.18	0.2
OsR498G1119701700.01	4.66	1.443	-1.83965	7.64E-38	7.80E-38	4.99	4.5	4.49	1.42	1.15	1.76
OsR498G1119707600.01	3.043	0.353	-3.11405	4.00E-32	4.39E-32	3.01	3.11	3.01	0.42	0.21	0.43
OsR498G1119712400.01	31.966	15.68	-1.02183	6.58E-113	3.80E-113	31.96	32.15	31.79	16.51	15.21	15.32
OsR498G1119714500.01	6.226	49.726	2.861693	0	0	5.93	7.15	5.6	49.33	50.46	49.39
OsR498G1119717400.01	2.66	0.733	-1.86713	9.38E-36	9.83E-36	2.66	2.68	2.64	0.71	0.83	0.66
OsR498G1119719100.01	0.17	0.02	-3.02359	8.36E-05	0.0001857	0.09	0.24	0.18	0	0	0.06
OsR498G1119729900.01	0.016	0.27	4.03976	3.45E-05	7.49E-05	0	0.05	0	0.4	0.31	0.1
OsR498G1119733000.01	10.2	1.85	-1.49963	2.00E-126	1.08E-126	10.99	10.34	9.27	1.86	1.79	1.9
OsR498G1119741400.01	2.75	12.836	2.20238	2.14E-190	9.04E-191	2.76	2.53	2.96	12.6	12.86	13.05
OsR498G1119744400.01	0.583	0.043	-3.76759	0.0001564	0.0003541	0.63	0.27	0.85	0	0	0.13
OsR498G1119761300.01	2.506	6.513	1.397312	1.61E-51	1.42E-51	1.99	2.86	2.67	6.84	6.06	6.64
OsR498G1119765500.01	4.923	1.926	-1.34875	1.69E-32	1.85E-32	5.02	4.93	4.82	1.87	2.06	1.85

OsR498G1119768400.01	2.88	0.273	-3.40081	8.02E-16	1.19E-15	2.81	3.55	2.28	0.58	0.24	0
OsR498G1119770300.01	85.253	28.536	-1.5824	0	0	85.06	90.98	79.72	30.29	26.88	28.44
OsR498G1119793700.01	4.883	2.48	-1.00482	5.31E-20	7.18E-20	4.77	5.03	4.85	2.61	2.47	2.36
OsR498G1119797800.01	0.45	0.043	-3.31074	5.25E-11	8.89E-11	0.5	0.52	0.33	0.05	0	0.08
OsR498G1119799200.01	0.616	0.04	-3.98261	3.03E-15	4.55E-15	0.5	0.75	0.6	0	0	0.12
OsR498G1119802100.01	0.67	0.063	-3.4418	4.89E-08	9.20E-08	0.79	0.47	0.75	0	0	0.19
OsR498G1119802700.01	3.84	1.83	-1.06595	4.97E-25	6.09E-25	4.15	3.49	3.88	2.1	1.84	1.55
OsR498G1119803900.01	1.686	0.17	-3.34987	1.88E-17	2.68E-17	1.16	1.54	2.36	0.14	0.37	0
OsR498G1119807900.01	2.743	0.95	-1.53023	9.16E-13	1.47E-12	3.31	2.77	2.15	1.25	1	0.6
OsR498G1119808900.01	2.713	0.086	-5.8185	3.81E-26	4.58E-26	2.92	1.82	3.4	0.23	0.03	0
OsR498G1119820100.01	0.96	0.036	-4.7416	1.55E-26	1.85E-26	1.05	0.9	0.93	0.03	0.05	0.03
OsR498G1119821500.01	8.95	3.666	-1.27808	5.30E-59	4.37E-59	8.44	9.53	8.88	3.92	3.43	3.65
OsR498G1119823100.01	13.906	0.07	-7.52357	3.56E-286	1.14E-286	12.37	15.59	13.76	0.12	0	0.09
OsR498G1119844100.01	2.163	0.573	-1.92547	5.75E-18	8.12E-18	2.83	2.02	1.64	0.48	0.46	0.78
OsR498G1119848800.01	0.523	1.263	1.39205	3.79E-15	5.71E-15	0.55	0.55	0.47	0.98	1.58	1.23
OsR498G1119862900.01	5.34	19.756	1.883497	1.82E-127	9.80E-128	5.79	5.28	4.95	21.07	20.28	17.92
OsR498G1119877600.01	0.136	0.58	2.088434	1.55E-25	1.89E-25	0.14	0.14	0.13	0.56	0.59	0.59
OsR498G1119883200.01	0	0.846	5.209685	4.72E-06	9.74E-06	0	0	0	0.14	1.84	0.56
OsR498G1119883300.01	0.393	4.9	3.631675	8.72E-167	3.98E-167	0.34	0.41	0.43	4.57	4.48	5.65
OsR498G1119886100.01	6.343	3.276	-1.06905	6.35E-21	8.42E-21	6.9	5.61	6.52	3.25	3.72	2.86
OsR498G1119887800.01	0.013	0.213	3.946651	6.37E-05	0.0001407	0	0	0.04	0.29	0.26	0.09
OsR498G1119892200.01	5.23	1.3	-2.10651	7.95E-82	5.47E-82	5.39	5.6	4.7	1.34	1.15	1.41
OsR498G1119898700.01	5.316	2.146	-1.28551	2.43E-19	3.33E-19	6.2	5.46	4.29	2.04	2.52	1.88
OsR498G1119901000.01	0.14	6.96	6.440878	2.01E-101	1.23E-101	0.24	0.18	0	7.09	6.85	6.94
OsR498G1119916100.01	41.736	19.85	-1.06927	3.27E-115	1.86E-115	41.34	42.89	40.98	20.24	20.33	18.98
OsR498G1119923300.01	19.373	5.996	-1.57162	2.66E-221	1.02E-221	18.75	20.33	19.04	5.64	5.8	6.55
OsR498G1119953400.01	1.956	0.453	-2.10888	7.32E-13	1.17E-12	0.77	3.48	1.62	0.24	0.17	0.95
OsR498G1119959400.01	126.2	52.223	-1.28129	7.13E-269	2.38E-269	128.17	125.6	124.8	52.24	50.38	54.05
OsR498G1119966200.01	0.283	0.026	-4.13016	1.44E-05	3.05E-05	0.26	0.33	0.26	0	0.08	0
OsR498G1119967700.01	0.433	0.136	-1.67073	0.0001155	0.0002591	0.32	0.74	0.24	0.11	0.15	0.15
OsR498G1119993800.01	87.253	29.916	-1.53697	0	0	88.32	87.1	86.34	28.18	29.97	31.6
OsR498G1119995500.01	0.01	0.173	4.209685	1.01E-05	2.14E-05	0	0.03	0	0.14	0.18	0.2
OsR498G1120008500.01	6.226	0.176	-5.14611	1.26E-43	1.21E-43	6.55	5.71	6.42	0.21	0.11	0.21
OsR498G1120014500.01	213.04	74.993	-1.51211	0	0	213.73	211.86	213.5	76.66	73.88	74.44
OsR498G1120016500.01	0.02	0.446	4.563322	2.64E-12	4.29E-12	0.03	0.03	0	0.34	0.56	0.44
OsR498G1120018500.01	3.423	1.173	-1.54155	3.03E-30	3.41E-30	3.26	3.76	3.25	1.23	0.92	1.37
OsR498G1120019200.01	3.09	13.286	2.094584	3.36E-68	2.56E-68	3.97	2.66	2.64	12.63	12.93	14.3
OsR498G1120020500.01	0.763	0.023	-5.16969	2.38E-10	4.12E-10	0.75	0.67	0.87	0	0	0.07
OsR498G1120026400.01	12.896	4.556	-1.50642	5.38E-84	3.64E-84	12.36	12.86	13.47	4.48	4.29	4.9
OsR498G1120026500.01	17.14	5.84	-1.56175	1.62E-80	1.13E-80	18.2	15.52	17.7	6.54	5.71	5.27
OsR498G1120027100.01	0.876	0	-7.10999	1.26E-17	1.79E-17	0.97	0.92	0.74	0	0	0
OsR498G1120029400.01	0.013	0.573	5.209685	2.30E-10	3.98E-10	0	0	0.04	0.66	0.58	0.48
OsR498G1120029500.01	3.263	8.566	1.380797	6.39E-21	8.48E-21	3.9	2.99	2.9	8.83	8.04	8.83
OsR498G1120030900.01	0.603	3.776	2.61265	2.72E-19	3.74E-19	1.18	0.19	0.44	3.93	4.55	2.85
OsR498G1120041800.01	8.676	1.696	-2.35427	5.20E-108	3.07E-108	8.25	9.39	8.39	1.68	1.87	1.54
OsR498G1120042400.01	1.48	0.196	-2.83471	1.58E-15	2.36E-15	1.22	1.81	1.41	0.23	0.12	0.24
OsR498G1120044700.01	0.55	3.436	2.437352	2.03E-20	2.72E-20	0.77	0.59	0.29	2.94	4.06	3.31
OsR498G1120048900.01	100.31	40.273	-1.3303	5.04E-307	1.51E-307	100.61	98.61	101.7	44.33	38.27	38.22
OsR498G1120049700.01	118.7	54.703	-1.1241	0	0	115.46	117.66	123	60.67	52.24	51.2
OsR498G1120056000.01	3.89	0.63	-2.35437	5.07E-27	6.01E-27	4.8	2.84	4.03	0.6	0.43	0.86
OsR498G1120058300.01	1.073	3.09	1.600777	1.60E-20	2.14E-20	1.35	0.98	0.89	2.82	2.94	3.51
OsR498G1120059500.01	2.566	5.226	1.021899	3.60E-36	3.74E-36	2.58	2.36	2.76	5.2	5.24	5.24
OsR498G1120060200.01	0.4	0.126	-1.67073	0.0001155	0.0002591	0.57	0.21	0.42	0.17	0.14	0.07
OsR498G1120079200.01	100.5	46.196	-1.12421	0	0	98.4	103.25	99.85	47.64	45.16	45.79
OsR498G1120079300.01	3.88	0	-7.34135	4.51E-20	6.09E-20	11.64	0	0	0	0	0
OsR498G1120082000.01	8.406	0.026	-8.50713	5.79E-75	4.18E-75	8.14	8.17	8.91	0.08	0	0
OsR498G1120082200.01	104.63	37.77	-1.47618	0	0	107.19	104.45	102.2	36.72	38.75	37.84
OsR498G1120086500.01	0.32	0.086	-1.81822	0.0002205	0.0005049	0.12	0.6	0.24	0.11	0.09	0.06
OsR498G1120106700.01	2.54	0.323	-3.01913	6.39E-06	1.33E-05	0.91	2.91	3.8	0	0	0.97
OsR498G1120113700.01	2.143	0.583	-1.92023	0.0001829	0.0004161	2.3	1.63	2.5	0.98	0.77	0
OsR498G1120115000.01	6.106	1.543	-1.97961	4.22E-78	2.98E-78	5.84	6.47	6.01	1.69	1.44	1.5
OsR498G1120116900.01	2	1.02	-1.12643	2.13E-10	3.68E-10	2.31	2.11	1.58	0.77	1.12	1.17
OsR498G1120147800.01	0.18	2.076	3.624723	6.43E-21	8.53E-21	0.25	0.23	0.06	1.9	1.83	2.5
OsR498G1120150400.01	0.09	1.46	4.003234	5.78E-35	6.11E-35	0.05	0.14	0.08	1.57	1.51	1.3
OsR498G1120152900.01	37.553	5.38	-2.81277	0	0	36.41	39.97	36.28	5.69	5.68	4.77
OsR498G1120153300.01	1.196	0.233	-2.4994	1.80E-14	2.77E-14	1.2	1.24	1.15	0.13	0.28	0.29
OsR498G1120153700.01	18.423	0.12	-7.25256	0	0	18.96	18.35	17.96	0.12	0.18	0.06
OsR498G1120154200.01	0.266	1.21	2.177264	7.19E-15	1.09E-14	0.29	0.23	0.28	1.2	1.21	1.22
OsR498G1120155000.01	3.853	1.73	-1.16138	1.87E-15	2.80E-15	4.42	3.56	3.58	1.81	1.66	1.72

OsR498G1120157800.01	0.343	0.093	-1.85277	0.0003665	0.0008522	0.2	0.37	0.46	0.08	0.04	0.16
OsR498G1120160300.01	14.386	5.366	-1.43287	1.05E-33	1.13E-33	15.2	14.53	13.43	6.16	4.76	5.18
OsR498G1120167900.01	2.203	35.296	3.995022	0	0	2.66	2.06	1.89	38.69	33.7	33.5
OsR498G1120170600.01	6.15	1.25	-2.3016	1.87E-185	8.05E-186	6.22	5.95	6.28	1.17	1.29	1.29
OsR498G1120171200.01	26.643	69.486	1.451076	0	0	26.77	27.02	26.14	68.69	69.7	70.07
OsR498G1120177600.01	5.95	1.363	-2.13016	3.06E-69	2.31E-69	5.81	5.26	6.78	1.44	1.43	1.22
OsR498G1120183000.01	1.003	0.216	-2.3817	1.81E-11	3.03E-11	1.26	0.92	0.83	0.29	0.18	0.18
OsR498G1120203700.01	0.036	0.466	3.702725	1.70E-09	3.02E-09	0.04	0	0.07	0.69	0.34	0.37
OsR498G1120205300.01	0.02	0.28	3.683616	7.93E-07	1.58E-06	0	0	0.06	0.33	0.24	0.27
OsR498G1120210200.01	1.486	15.243	3.354575	7.64E-228	2.87E-228	1.38	1.6	1.48	16.57	14.82	14.34
OsR498G1120213300.01	0.72	0	-4.76759	6.52E-05	0.0001441	0.61	0.81	0.74	0	0	0
OsR498G1120214000.01	1.313	0.66	-1.02307	1.73E-17	2.47E-17	0.9	1.63	1.41	0.59	0.74	0.65
OsR498G1120215800.01	25.053	71.413	1.503318	4.84E-238	1.75E-238	26.72	25.2	23.24	70.7	70.5	73.04
OsR498G1120216000.01	1.533	0.083	-4.15464	3.45E-21	4.55E-21	1.62	1.47	1.51	0.2	0.05	0
OsR498G1120216800.01	4.61	2.116	-1.1513	6.85E-69	5.19E-69	4.26	4.55	5.02	2.1	2.05	2.2
OsR498G1120217400.01	3.223	1.41	-1.24487	7.50E-12	1.24E-11	3.68	3.1	2.89	1.04	1.51	1.68
OsR498G1120222300.01	20.39	109.72	2.4219	0	0	19.27	20.23	21.67	108.33	109.33	111.49
OsR498G1120222700.01	0.16	1.723	3.318619	2.28E-32	2.49E-32	0.29	0.1	0.09	1.6	1.86	1.71
OsR498G1120227300.01	0.553	0.11	-2.41967	0.0001248	0.0002807	0.62	0.24	0.8	0	0.22	0.11
OsR498G1120235000.01	43.253	112.52	1.362754	2.70E-292	8.45E-293	44.4	42.32	43.04	114.02	114.19	109.34
OsR498G1120235900.01	3.403	24.813	2.885562	0	0	3.03	3.13	4.05	23.47	25.53	25.44
OsR498G1120253100.01	5.38	1.71	-1.71147	4.31E-47	3.98E-47	6.08	5.38	4.68	1.44	1.85	1.84
OsR498G1120253800.01	0.046	0.42	3.209685	5.30E-05	0.0001164	0.07	0.07	0	0.41	0.71	0.14
OsR498G1120258000.01	0.886	0.186	-2.4065	5.17E-16	7.63E-16	0.85	0.98	0.83	0.25	0.12	0.19
OsR498G1120259100.01	1.25	0.443	-1.51278	7.72E-05	0.0001711	0.77	1.25	1.73	0.44	0.45	0.44
OsR498G1120263000.01	312.18	108.61	-1.52609	0	0	341.57	298.41	296.6	148.8	95.21	81.81
OsR498G1120269200.01	39.9	8.813	-2.35943	0	0	39.52	39.13	41.05	9.12	9.21	8.11
OsR498G1120278000.01	22.346	2.893	-2.93274	0	0	22.91	21.56	22.57	2.57	2.9	3.21
OsR498G1120278400.01	0.33	1.88	2.489067	1.06E-11	1.76E-11	0.23	0.32	0.44	1.96	1.61	2.07
OsR498G1120279100.01	0.063	0.6	3.312779	3.62E-07	7.08E-07	0.06	0.13	0	0.55	0.44	0.81
OsR498G1120279400.01	26.06	7.24	-1.8521	0	0	25.73	25.91	26.54	7.07	7.51	7.14
OsR498G1120285000.01	0.603	12.35	4.344615	3.51E-225	1.33E-225	0.6	0.45	0.76	13.53	12.48	11.04
OsR498G1120287000.01	3.72	9.366	1.326223	7.17E-67	5.52E-67	4.28	3.59	3.29	8.98	9.08	10.04
OsR498G1120288100.01	0.1	0.67	2.683616	1.50E-05	3.19E-05	0.15	0	0.15	0.55	0.81	0.65
OsR498G1120288600.01	24	67.016	1.478301	0	0	23.26	23.73	25.01	67.56	68.13	65.36
OsR498G1120293500.01	0.283	0	-5.13016	6.38E-06	1.33E-05	0.42	0.2	0.23	0	0	0
OsR498G1120294800.01	0.616	0.096	-2.68271	1.22E-06	2.44E-06	0.78	0.64	0.43	0.11	0.06	0.12
OsR498G1120295700.01	21.706	3.643	-2.58046	0	0	20.98	21.69	22.45	3.77	3.6	3.56
OsR498G1120296600.01	6.436	0.873	-3.00426	3.84E-160	1.79E-160	6.08	6.76	6.47	0.73	0.85	1.04
OsR498G1120297100.01	11.623	4.376	-1.41022	4.97E-64	3.92E-64	12.47	11.34	11.06	5.6	3.66	3.87
OsR498G1120297300.01	2.583	0.466	-2.46274	1.84E-15	2.75E-15	2.63	3.01	2.11	0.46	0.55	0.39
OsR498G1120297500.01	90.156	2.91	-4.53793	0	0	89.19	95.08	86.2	2.86	2.94	2.93
OsR498G1120304700.01	0.646	0.123	-1.99586	5.18E-06	1.07E-05	0.49	0.3	1.15	0.15	0.11	0.11
OsR498G1120304900.01	0	0.256	4.361688	0.0005877	0.0013892	0	0	0	0.23	0.23	0.31
OsR498G1120310400.01	1.673	6.236	1.897156	2.54E-32	2.78E-32	1.41	1.91	1.7	7.72	6.72	4.27
OsR498G1120311400.01	12.38	5.613	-1.21184	4.16E-58	3.46E-58	11.95	12.79	12.4	5.51	5.23	6.1
OsR498G1120312000.01	0.33	0.086	-1.94294	0.0001489	0.0003365	0.47	0.28	0.24	0	0.07	0.19
OsR498G1120321000.01	5.163	17.99	1.789698	1.95E-144	9.74E-145	5.69	4.78	5.02	17.69	18.44	17.84
OsR498G1120324400.01	0.24	1.696	2.748711	3.87E-28	4.49E-28	0.18	0.13	0.41	1.66	1.73	1.7
OsR498G1120327700.01	0.013	0.33	4.993956	4.24E-09	7.66E-09	0	0.04	0	0.38	0.31	0.3
OsR498G1120336900.01	1.016	0.373	-1.30669	8.59E-07	1.71E-06	1.25	0.78	1.02	0.28	0.42	0.42
OsR498G1120337200.01	2.18	0.776	-1.48961	5.32E-21	7.04E-21	2.55	2.33	1.66	0.78	0.63	0.92
OsR498G1120348100.01	5.203	0.14	-5.23077	2.71E-46	2.53E-46	3.86	5.92	5.83	0.17	0.08	0.17
OsR498G1120348700.01	0.043	0.56	3.604545	6.77E-16	1.00E-15	0.02	0	0.11	0.65	0.53	0.5
OsR498G1120350200.01	0.88	0.163	-2.44137	1.09E-10	1.87E-10	0.69	0.94	1.01	0.08	0.16	0.25
OsR498G1120350400.01	11.933	4.603	-1.37836	7.98E-59	6.59E-59	11.56	11.87	12.37	4.7	4.23	4.88
OsR498G1120352700.01	0.066	0.343	2.337441	1.92E-09	3.42E-09	0.02	0.07	0.11	0.43	0.3	0.3
OsR498G1120353500.01	0.263	0.106	-1.33018	0.0001698	0.0003853	0.3	0.2	0.29	0.08	0.17	0.07
OsR498G1120370300.01	1.34	0.456	-1.55748	4.35E-19	5.99E-19	1.33	1.26	1.43	0.52	0.48	0.37
OsR498G1120370600.01	1.383	0.046	-4.81822	1.32E-47	1.21E-47	1.39	1.22	1.54	0.02	0.06	0.06
OsR498G1120373800.01	0.5	1.276	1.03976	0.0001801	0.0004094	0.54	0.6	0.36	0.84	1.63	1.36
OsR498G1120374500.01	0.656	0.183	-1.82697	1.74E-07	3.35E-07	0.82	0.79	0.36	0.19	0.26	0.1
OsR498G1120374700.01	2.973	0.6	-2.32947	1.17E-05	2.48E-05	2.26	4.21	2.45	0.6	0.6	0.6
OsR498G1120379000.01	0.866	0.376	-1.23486	1.24E-05	2.63E-05	0.69	1.01	0.9	0.32	0.38	0.43
OsR498G1120380000.01	0.793	0.18	-2.2896	3.26E-09	5.86E-09	0.86	0.85	0.67	0.17	0.32	0.05
OsR498G1120383600.01	1.47	0.39	-1.84777	4.75E-19	6.56E-19	1.73	1.38	1.3	0.31	0.44	0.42
OsR498G1120383800.01	3.656	1.583	-1.76054	8.17E-64	6.44E-64	4.16	3.77	3.04	1.84	1.3	1.61
OsR498G1120384900.01	0.026	0.183	2.847115	2.76E-06	5.63E-06	0.04	0.04	0	0.19	0.18	0.18
OsR498G1120393200.01	0.103	0.866	3.088123	1.18E-17	1.68E-17	0.11	0.09	0.11	0.7	1.1	0.8

OsR498G1120393600.01	0.5	1.206	1.272421	3.04E-12	4.96E-12	0.37	0.53	0.6	1.34	1.1	1.18
OsR498G1120396500.01	1.8	0.73	-1.30128	1.97E-12	3.20E-12	1.47	2.13	1.8	0.83	0.68	0.68
OsR498G1120404900.01	0.06	0.006	-3.66068	0.0002846	0.0006567	0.04	0.03	0.11	0	0	0.02
OsR498G1120405900.01	6.31	2.253	-1.49866	6.78E-23	8.64E-23	6.64	6.15	6.14	2.75	1.96	2.05
OsR498G1120407400.01	36.716	17.946	-1.0315	9.35E-102	5.71E-102	37.74	37.37	35.04	18.62	17.03	18.19
OsR498G1120411100.01	3.38	1.546	-1.13016	4.52E-11	7.64E-11	3.5	3.73	2.91	1.69	1.48	1.47
OsR498G1120411600.01	19.69	121.07	2.612709	0	0	18.87	19.55	20.65	124.55	120.16	118.51
OsR498G1120413700.01	0.11	0.436	2.018386	5.00E-09	9.07E-09	0.06	0.18	0.09	0.34	0.46	0.51
OsR498G1120415500.01	3.15	0.776	-2.02325	1.29E-38	1.31E-38	2.81	3.48	3.16	0.71	0.65	0.97
OsR498G1120416500.01	1.75	0.603	-1.44567	2.25E-06	4.58E-06	2.22	1.44	1.59	0.79	0.4	0.62
OsR498G1120418500.01	6.193	15.336	1.304266	3.72E-159	1.74E-159	6.42	5.93	6.23	15.98	14.71	15.32
OsR498G1120419800.01	5.143	15.593	1.56723	4.93E-117	2.79E-117	5.77	4.02	5.64	16.08	15.71	14.99
OsR498G1120425000.01	0.013	3.87	8.01274	2.78E-214	1.09E-214	0.02	0.01	0.01	3.64	3.95	4.02
OsR498G1120430300.01	3.076	0.96	-1.58473	3.01E-21	3.96E-21	2.95	3.09	3.19	1.46	0.63	0.79
OsR498G1120434100.01	1.876	4.323	1.192839	2.84E-09	5.09E-09	1.92	1.85	1.86	4.55	4.49	3.93
OsR498G1120434500.01	0.443	2.54	2.491456	8.91E-15	1.36E-14	0.39	0.33	0.61	2.5	2.49	2.63
OsR498G1120437200.01	14.92	5.34	-1.48653	6.12E-128	3.27E-128	14.55	14.94	15.27	5.34	5.36	5.32
OsR498G1120437900.01	0.036	0.47	3.624723	0.0004041	0.0009439	0.11	0	0	0.58	0.12	0.71
OsR498G1120440500.01	0.336	0.97	1.51039	8.72E-09	1.59E-08	0.26	0.36	0.39	0.97	1.01	0.93
OsR498G1120444000.01	0	0.146	4.361688	0.0005877	0.0013892	0	0	0	0.22	0.09	0.13
OsR498G1120446600.01	0.04	0.453	3.445752	5.76E-12	9.47E-12	0.02	0	0.1	0.73	0.42	0.21
OsR498G1120448400.01	0.616	2.203	1.831105	5.02E-66	3.89E-66	0.64	0.58	0.63	2	2.11	2.5
OsR498G1120452300.01	2.04	6.01	1.488624	1.74E-55	1.48E-55	1.96	1.36	2.8	5.52	6.07	6.44
OsR498G1120452900.01	0.04	0.446	3.572255	9.13E-18	1.29E-17	0.03	0.04	0.05	0.5	0.41	0.43
OsR498G1120454300.01	0	0.33	6.794648	2.06E-14	3.16E-14	0	0	0	0.29	0.3	0.4
OsR498G1120454800.01	42.766	117.65	1.456444	0	0	42.02	42.77	43.51	124.61	113.31	115.04
OsR498G1120466600.01	0.193	13.666	6.084154	2.17E-80	1.51E-80	0.24	0	0.34	14.13	12.66	14.21
OsR498G1120466900.01	23.126	87.123	1.906951	0	0	23.72	23.26	22.4	88.62	86.8	85.95
OsR498G1120467700.01	5.09	82.03	4.058901	0	0	4.79	4.74	5.74	87.26	75.03	83.8
OsR498G1120471000.01	15.83	70.376	2.15108	0	0	16.45	16.67	14.37	72.08	71.49	67.56
OsR498G1120475400.01	1.64	4.25	1.3438	3.55E-33	3.84E-33	1.47	1.69	1.76	4.34	3.94	4.47
OsR498G1120475600.01	9.12	24.213	1.396292	1.29E-181	5.62E-182	9.8	8.07	9.49	22.61	25.03	25
OsR498G1120477000.01	1.77	0.506	-1.80552	6.07E-21	8.05E-21	1.79	1.93	1.59	0.35	0.63	0.54
OsR498G1120478400.01	0.663	0.14	-2.22327	0.0001374	0.00031	0.81	1.02	0.16	0.17	0.17	0.08
OsR498G1120482500.01	20.59	45.486	1.134917	5.46E-164	2.52E-164	20.03	20.34	21.4	45.19	44.46	46.81
OsR498G1120483200.01	4.733	17.7	1.895279	1.97E-225	7.44E-226	4.83	4.59	4.78	17.59	17.54	17.97
OsR498G1120483500.01	0.04	2.033	5.999799	5.40E-95	3.41E-95	0.06	0.02	0.04	1.84	1.86	2.4
OsR498G1120484000.01	0.406	0.093	-2.10708	1.13E-08	2.07E-08	0.37	0.41	0.44	0.08	0.12	0.08
OsR498G1120484100.01	0.406	0.026	-3.41967	5.86E-10	1.03E-09	0.36	0.66	0.2	0.04	0.04	0
OsR498G1120486300.01	1.513	0.586	-1.34567	4.11E-14	6.37E-14	1.41	1.27	1.86	0.56	0.65	0.55
OsR498G1120489000.01	1.433	0.31	-2.56792	9.34E-20	1.27E-19	1.21	1.72	1.37	0.3	0.19	0.44
OsR498G1120489600.01	0.383	0.166	-1.26836	0.0001555	0.0003518	0.35	0.42	0.38	0.21	0.1	0.19
OsR498G1120491500.01	13.923	4.646	-1.60992	2.58E-113	1.49E-113	14.56	14.31	12.9	4.09	4.96	4.89
OsR498G1120503300.01	0.93	0.27	-1.77653	5.84E-09	1.06E-08	0.82	1.08	0.89	0.42	0.14	0.25
OsR498G1120505100.01	32.586	11.15	-1.56015	8.50E-179	3.74E-179	32.98	31.88	32.9	11.76	10.12	11.57
OsR498G1120505900.01	14.303	47.423	1.720478	0	0	12.91	14.63	15.37	44.97	49.48	47.82
OsR498G1120506100.01	8.093	1.523	-2.27818	1.29E-89	8.48E-90	8.05	7.94	8.29	1.34	1.75	1.48
OsR498G1120514100.01	0.166	0.033	-2.76759	0.0007566	0.0018055	0.08	0.27	0.15	0	0.05	0.05
OsR498G1120523800.01	119.26	59.813	-1.00573	0	0	125.67	113.57	118.6	61.62	60.07	57.75
OsR498G1120525600.01	1.97	9.24	2.311874	4.66E-103	2.83E-103	2.01	1.88	2.02	8.95	9.39	9.38
OsR498G1120526500.01	47.57	16.45	-1.5341	1.18E-204	4.74E-205	47.07	48.66	46.98	15.52	16.67	17.16
OsR498G1120543200.01	1.92	9.413	2.353911	7.70E-122	4.25E-122	2.06	1.84	1.86	10.23	9.05	8.96
OsR498G1120562000.01	22.663	3.086	-2.88699	4.87E-123	2.67E-123	23.51	22.88	21.6	3.8	2.51	2.95
OsR498G1120576800.01	3.14	0.746	-2.1032	3.29E-25	4.02E-25	2.96	3.27	3.19	0.54	0.88	0.82
OsR498G1120580100.01	2.733	0.55	-2.37785	8.54E-26	1.03E-25	2.7	2.85	2.65	0.63	0.64	0.38
OsR498G1120581500.01	14.54	4.643	-1.77198	0	0	14.5	14.35	14.77	4.24	5.21	4.48
OsR498G1120584800.01	40.636	15.426	-1.40202	2.75E-171	1.24E-171	38.97	42.14	40.8	16.52	14.45	15.31
OsR498G1120588100.01	0.243	0.033	-2.86713	0.0004299	0.0010056	0.34	0.25	0.14	0	0.1	0
OsR498G1120591500.01	21.353	0.02	-9.89393	0	0	20.76	20.28	23.02	0	0.03	0.03
OsR498G1120617100.01	0.566	0.11	-2.35256	0.0002107	0.0004813	0.97	0.26	0.47	0.08	0.17	0.08
OsR498G1120617700.01	0.033	11.71	8.449151	1.40E-276	4.58E-277	0	0.1	0	12.31	11.35	11.47
OsR498G1120631700.01	0.28	0	-4.96024	2.02E-05	4.33E-05	0.37	0.22	0.25	0	0	0
OsR498G1120633100.01	3.133	1.15	-1.50598	1.65E-19	2.26E-19	3.33	2.78	3.29	0.86	1.45	1.14
OsR498G1120633800.01	2.713	0.973	-1.39836	4.96E-07	9.77E-07	2.96	1.61	3.57	0.75	1.18	0.99
OsR498G1120635000.01	17.8	58.19	1.70708	0	0	17.7	17.56	18.14	56.98	59.28	58.31
OsR498G1120635400.01	1.336	0.31	-1.85248	1.07E-24	1.32E-24	1.37	1.38	1.26	0.37	0.31	0.25
OsR498G1120641000.01	10.596	22.14	1.03184	2.41E-66	1.86E-66	10.17	11.35	10.27	22.29	21.83	22.3
OsR498G1120646200.01	8.24	1.726	-2.21488	4.05E-46	3.79E-46	8.7	7.89	8.13	2.04	1.69	1.45
OsR498G1120669300.01	13.363	31.63	1.212837	5.07E-119	2.84E-119	12.9	13.93	13.26	30.78	31.81	32.3

OsR498G1120669500.01	0.906	0	-5.6041	1.26E-07	2.41E-07	0.97	1.13	0.62	0	0	0
OsR498G1120673600.01	0.073	1.146	3.946651	6.37E-05	0.0001407	0.22	0	0	1.83	0.46	1.15
OsR498G1120680000.01	1.626	13.493	3.043675	9.17E-254	3.19E-254	1.53	1.61	1.74	12.95	13.17	14.36
OsR498G1120681300.01	0.03	5.513	7.370677	8.85E-146	4.39E-146	0.02	0.03	0.04	5.44	5.6	5.5
OsR498G1120682400.01	8.396	25.293	1.578146	1.75E-181	7.62E-182	8.46	8.39	8.34	24.12	26.39	25.37
OsR498G1120683400.01	19.35	95.35	2.371469	0	0	20.13	18.8	19.12	95.51	92.74	97.8
OsR498G1120685800.01	903.91	283.41	-1.68428	0	0	947.18	867.95	896.6	290.7	275.92	283.6
OsR498G1120686800.01	11.886	5.676	-1.10664	1.92E-42	1.86E-42	12.36	13.17	10.13	4.92	6.21	5.9
OsR498G1120688900.01	0.533	1.09	1.013288	6.25E-06	1.30E-05	0.61	0.43	0.56	1.22	1.14	0.91
OsR498G1120691200.01	4.01	39.5	3.286721	0	0	3.32	4.79	3.92	40.34	38.62	39.54
OsR498G1120691300.01	0.216	3.43	3.982275	6.93E-28	8.08E-28	0.48	0.17	0	3.13	3.89	3.27
OsR498G1120694900.01	0.033	0.166	3.499192	0.0007491	0.0017869	0	0.1	0	0.09	0.23	0.18
OsR498G1120695800.01	0.05	0.42	3.432077	7.90E-10	1.39E-09	0.04	0.08	0.03	0.64	0.36	0.26
OsR498G1120696900.01	0.023	0.59	4.466025	1.58E-11	2.63E-11	0	0.07	0	0.56	0.42	0.79
OsR498G1120697900.01	0.436	1.923	2.178524	5.93E-29	6.80E-29	0.31	0.59	0.41	1.88	2.26	1.63
OsR498G1120700800.01	44.693	2.86	-3.97399	0	0	45.49	44.27	44.32	3.18	2.68	2.72
OsR498G1120703700.01	0.316	1.053	1.730656	1.04E-11	1.73E-11	0.25	0.35	0.35	0.92	1.03	1.21
OsR498G1120704100.01	12.17	29.05	1.241121	8.22E-114	4.71E-114	11.24	12.5	12.77	26.74	30.64	29.77
OsR498G1120707000.01	0.13	0.406	1.67719	1.79E-06	3.62E-06	0.11	0.13	0.15	0.36	0.49	0.37
OsR498G1120709100.01	2.973	0.836	-1.85142	4.73E-76	3.39E-76	2.87	3.13	2.92	0.85	0.82	0.84
OsR498G1120709400.01	0.36	0	-5.08622	8.69E-06	1.83E-05	0.32	0.32	0.44	0	0	0
OsR498G1120711700.01	57.943	11.313	-2.4286	0	0	56.9	57.62	59.31	10.87	10.97	12.1
OsR498G1120712900.01	44.103	17.4	-1.40465	0	0	43.9	43.49	44.92	16.73	16.66	18.81
OsR498G1120713500.01	81.603	24.6	-1.70793	0	0	80.54	82.95	81.32	24.82	23.91	25.07
OsR498G1120714100.01	2.116	0.653	-1.61366	2.48E-17	3.55E-17	1.8	2.33	2.22	0.75	0.67	0.54
OsR498G1120714500.01	0.43	0.08	-2.32775	5.64E-09	1.02E-08	0.33	0.58	0.38	0.15	0.02	0.07
OsR498G1120716000.01	0.123	0	-4.28217	0.0007127	0.0016975	0.18	0.15	0.04	0	0	0
OsR498G1120717200.01	3.133	0.35	-3.19546	2.07E-80	1.44E-80	2.36	3.77	3.27	0.33	0.41	0.31
OsR498G1120723800.01	0.206	1.19	2.830075	2.39E-16	3.49E-16	0.42	0.13	0.07	0.81	1.26	1.5
OsR498G1120723900.01	3.726	1	-1.90224	4.57E-144	2.29E-144	3.67	3.68	3.83	0.93	1.11	0.96
OsR498G1120726800.01	0.423	0.013	-5.5452	5.29E-13	8.42E-13	0.33	0.43	0.51	0.04	0	0
OsR498G1120729800.01	69.703	20.216	-1.87454	0	0	68.73	70.03	70.35	19.82	20.65	20.18
OsR498G1120730600.01	1.28	11.313	3.122319	7.07E-156	3.36E-156	1.67	0.85	1.32	10.47	11.09	12.38
OsR498G1120733900.01	2.113	0.633	-1.74101	2.10E-41	2.07E-41	2.22	2.05	2.07	0.65	0.55	0.7
OsR498G1120735400.01	16.156	7.306	-1.12436	9.16E-196	3.80E-196	14.03	17.92	16.52	6.93	7.63	7.36
OsR498G1120740500.01	11.263	2.28	-2.33221	1.39E-155	6.61E-156	10	11.57	12.22	1.88	2.26	2.7
OsR498G1120741700.01	4.946	30.826	2.670382	0	0	4.65	5.25	4.94	29.41	31.8	31.27
OsR498G1120742400.01	54.186	19.706	-1.50144	1.26E-158	5.91E-159	53.89	53.3	55.37	17.3	20.55	21.27
OsR498G1120743300.01	125.46	41.2	-1.79095	0	0	127.39	125.83	123.2	43.57	38.84	41.19
OsR498G1120745500.01	1.73	0.336	-2.36661	4.60E-51	4.08E-51	1.78	1.6	1.81	0.38	0.34	0.29
OsR498G1120745900.01	0.466	0.04	-3.54331	8.72E-18	1.24E-17	0.45	0.47	0.48	0.05	0.02	0.05
OsR498G1120747000.01	0.833	0.026	-5.04218	2.38E-17	3.41E-17	1.12	1.05	0.33	0	0.08	0
OsR498G1120747200.01	1.203	0	-8.78208	9.55E-45	9.05E-45	1.14	1.07	1.4	0	0	0
OsR498G1120747600.01	0.603	0.016	-6.35256	3.22E-21	4.24E-21	0.6	0.47	0.74	0	0	0.05
OsR498G1120749400.01	0.02	0.103	2.499598	0.0003218	0.0007455	0.02	0.02	0.02	0.14	0.1	0.07
OsR498G1120756400.01	0.176	0.01	-3.98483	1.56E-08	2.89E-08	0.13	0.21	0.19	0.03	0	0
OsR498G1120756900.01	8.23	1.486	-2.47809	1.93E-128	1.03E-128	8.62	7.94	8.13	1.68	1.62	1.16
OsR498G1120760100.01	9.163	31.45	1.748916	0	0	9.72	8.81	8.96	29.76	31.53	33.06
OsR498G1120760800.01	64.336	16.14	-2.00342	0	0	64.35	64.61	64.05	14.92	17.94	15.56
OsR498G1120761400.01	0.023	0.85	5.209685	2.30E-10	3.98E-10	0	0	0.07	1.19	0.79	0.57
OsR498G1120762900.01	0.18	0.75	2.03976	3.78E-06	7.78E-06	0.11	0.17	0.26	0.66	0.91	0.68
OsR498G1120764000.01	0	2.033	8.028445	9.92E-29	1.14E-28	0	0	0	2.03	1.95	2.12
OsR498G1120764600.01	0.236	4.436	4.22109	1.19E-29	1.35E-29	0.1	0.32	0.29	4.45	4.34	4.52
OsR498G1120767700.01	647.74	233.68	-1.47589	0	0	621.13	665.24	656.9	236.01	233.39	231.64
OsR498G1120768100.01	1.17	12.233	3.370868	2.49E-90	1.62E-90	1.42	0.94	1.15	11.76	13.27	11.67
OsR498G1120769500.01	361.65	1568.2	2.107166	0	0	351.94	370.73	362.3	1644.6	1501.8	1558.1
OsR498G1120770000.01	5.96	14.036	1.226485	1.65E-43	1.58E-43	6.04	5.96	5.88	14.42	14.01	13.68
OsR498G1120770600.01	0.19	2.996	3.970497	2.44E-53	2.11E-53	0.21	0.22	0.14	3.81	2.36	2.82
OsR498G1120771100.01	2.26	15.623	2.783921	1.83E-284	5.85E-285	2.43	2	2.35	16.39	15.44	15.04
OsR498G1120775000.01	2.963	0.34	-3.13016	4.50E-31	5.00E-31	3.52	2.82	2.55	0.37	0.33	0.32
OsR498G1120776600.01	53.886	192.63	1.841884	0	0	54.97	52.18	54.51	191.27	187.25	199.38
OsR498G1120777600.01	0.283	0.063	-2.13016	8.77E-05	0.0001953	0.31	0.36	0.18	0.03	0.03	0.13
OsR498G1120778500.01	11.553	2.723	-2.09117	1.96E-97	1.22E-97	11.08	11.41	12.17	2.42	2.89	2.86
OsR498G1120778700.01	1.326	0.243	-2.44853	3.07E-15	4.62E-15	0.94	1.68	1.36	0.32	0.33	0.08
OsR498G1220780400.01	1.436	0.106	-3.72024	1.69E-20	2.26E-20	1.25	1.5	1.56	0.17	0.05	0.1
OsR498G1220781400.01	1.286	24.006	3.863002	2.95E-262	1.00E-262	1.52	0.81	1.53	25.93	22.12	23.97
OsR498G1220782600.01	2.103	35.576	4.069347	0	0	2.13	1.91	2.27	33.04	37.19	36.5
OsR498G1220783400.01	10.786	51.27	2.243598	0	0	11.57	10.85	9.94	53.69	48.86	51.26
OsR498G1220784400.01	0.023	0.673	4.82112	1.29E-14	1.96E-14	0	0.04	0.03	0.72	0.34	0.96

OsR498G1220789200.01	18.326	47.63	1.3433	0	0	16.57	20.07	18.34	45.42	48.53	48.94
OsR498G1220789800.01	10.383	23.453	1.172304	1.99E-141	1.01E-141	9.37	10.93	10.85	24.26	24.45	21.65
OsR498G1220798200.01	3.493	61.083	4.13982	0	0	3.42	3.46	3.6	61.52	62.77	58.96
OsR498G1220798800.01	30.233	79.396	1.41561	0	0	29.35	31.24	30.11	77.8	77.63	82.76
OsR498G1220799800.01	57.096	1268.9	4.462996	0	0	58.71	55.34	57.24	1290.4	1223.6	1292.7
OsR498G1220802100.01	22.323	4.57	-2.29309	0	0	21.5	23.19	22.28	3.99	5	4.72
OsR498G1220806200.01	0.903	0.256	-1.60076	3.95E-09	7.12E-09	1.05	0.98	0.68	0.36	0.24	0.17
OsR498G1220808300.01	5.503	11.626	1.077461	4.82E-66	3.73E-66	5.02	5.97	5.52	10.55	12.72	11.61
OsR498G1220810700.01	1.093	0.186	-2.59965	2.48E-13	3.91E-13	0.77	1.14	1.37	0.13	0.25	0.18
OsR498G1220813400.01	0.073	1.07	4.137792	1.65E-31	1.82E-31	0	0.14	0.08	1.12	1.07	1.02
OsR498G1220815000.01	3.626	8.29	1.167656	5.37E-57	4.51E-57	3.78	3.5	3.6	8.37	8.29	8.21
OsR498G1220815500.01	9.32	4.146	-1.31985	2.52E-50	2.25E-50	9.91	8.97	9.08	3.87	3.91	4.66
OsR498G1220816400.01	0.796	0.096	-3.0477	1.75E-07	3.37E-07	0.78	0.45	1.16	0.07	0.15	0.07
OsR498G1220817300.01	2.073	0.726	-1.52054	4.06E-14	6.28E-14	2.04	2.26	1.92	0.55	0.85	0.78
OsR498G1220817600.01	10.693	3.336	-1.45232	1.38E-27	1.62E-27	10.93	10.79	10.36	2.72	4.49	2.8
OsR498G1220818100.01	0.296	0.06	-2.28217	0.0003536	0.0008216	0.31	0.28	0.3	0.09	0.09	0
OsR498G1220819400.01	12.146	5.183	-1.2309	2.35E-62	1.88E-62	12.28	12.21	11.95	4.93	4.91	5.71
OsR498G1220819900.01	27.046	60.506	1.149934	8.27E-119	4.63E-119	28.13	25.5	27.51	60.52	58.35	62.65
OsR498G1220822800.01	3.223	0.676	-2.2483	1.29E-46	1.20E-46	3.16	3.13	3.38	0.78	0.68	0.57
OsR498G1220824700.01	4.456	1.85	-1.26052	1.84E-25	2.24E-25	4.36	4.36	4.65	1.67	1.62	2.26
OsR498G1220827000.01	11.786	0.733	-4.10603	5.12E-284	1.64E-284	12.58	12.19	10.59	0.58	0.67	0.95
OsR498G1220827300.01	16.426	5.113	-1.68933	6.59E-55	5.63E-55	16.8	17.5	14.98	6.04	4.57	4.73
OsR498G1220827700.01	1.333	0.583	-1.21082	2.70E-06	5.51E-06	1.03	1.23	1.74	0.47	0.72	0.56
OsR498G1220830400.01	1.82	3.973	1.12724	9.97E-12	1.65E-11	1.53	2.17	1.76	4.53	3.72	3.67
OsR498G1220830600.01	7.9	3.803	-1.1598	4.74E-47	4.38E-47	7.91	7.5	8.29	3.73	4.19	3.49
OsR498G1220834300.01	1.256	0.14	-3.27183	1.44E-26	1.72E-26	1.41	1	1.36	0.09	0.21	0.12
OsR498G1220835700.01	7.033	21.943	1.629054	1.30E-193	5.44E-194	6.27	6.65	8.18	23.83	20.41	21.59
OsR498G1220836000.01	0	0.326	6.126798	6.15E-10	1.08E-09	0	0	0	0.53	0.45	0
OsR498G1220836100.01	0.11	1.156	3.312779	6.19E-18	8.74E-18	0.04	0.08	0.21	1.34	0.89	1.24
OsR498G1220836800.01	0.096	6.246	5.926213	2.08E-72	1.53E-72	0.06	0	0.23	5.95	7.11	5.68
OsR498G1220841100.01	9.53	59.71	2.643083	0	0	9.9	9.86	8.83	67.22	54.61	57.3
OsR498G1220842300.01	11.556	4.27	-1.40561	8.95E-109	5.25E-109	11.23	11.61	11.83	4.13	4.3	4.38
OsR498G1220842500.01	0.116	0.363	1.608249	2.10E-05	4.49E-05	0.28	0	0.07	0.24	0.36	0.49
OsR498G1220842800.01	9.066	4.13	-1.07243	2.28E-22	2.93E-22	10.48	8	8.72	4.68	4.4	3.31
OsR498G1220843600.01	184.13	1.543	-6.75708	0	0	183.87	184.61	183.9	1.64	1.18	1.81
OsR498G1220844000.01	0.706	1.443	1.049475	7.16E-08	1.36E-07	0.56	0.71	0.85	1.58	1.12	1.63
OsR498G1220844800.01	442.44	17.956	-4.63313	0	0	468.21	424.49	434.6	19.8	15.95	18.12
OsR498G1220845200.01	285.8	15.67	-4.1976	0	0	285.71	280.68	291	18.44	13.88	14.69
OsR498G1220845600.01	1.613	0	-7.27149	2.67E-19	3.66E-19	1.48	1.68	1.68	0	0	0
OsR498G1220854200.01	0.546	0.08	-2.73368	1.69E-14	2.59E-14	0.56	0.57	0.51	0.1	0.06	0.08
OsR498G1220855500.01	10.366	4.946	-1.00604	2.19E-21	2.88E-21	10.72	9.52	10.86	5.6	5.46	3.78
OsR498G1220856100.01	13.56	1.686	-3.0872	1.37E-202	5.53E-203	13.11	14.08	13.49	1.44	1.9	1.72
OsR498G1220858100.01	0.556	30.473	5.780243	0	0	0.67	0.55	0.45	31.69	28.59	31.14
OsR498G1220859100.01	0.06	12.226	7.699399	8.59E-222	3.28E-222	0.18	0	0	13.29	11.58	11.81
OsR498G1220859700.01	327.55	33.74	-3.27864	0	0	347.19	317.41	318	35.38	33.16	32.68
OsR498G1220861200.01	4.806	2.29	-1.07298	7.52E-20	1.02E-19	4.48	5.17	4.77	2.19	2.2	2.48
OsR498G1220862500.01	1.383	6.286	2.167906	1.13E-31	1.25E-31	1.36	1.2	1.59	5.12	7.07	6.67
OsR498G1220864800.01	0.87	0.283	-1.63009	0.0001766	0.0004013	0.96	0.86	0.79	0.23	0.39	0.23
OsR498G1220866400.01	0.983	5.266	2.424898	5.13E-70	3.85E-70	0.89	1.26	0.8	5.37	5.3	5.13
OsR498G1220867600.01	9.893	23.013	1.206595	7.27E-127	3.91E-127	10.51	9.51	9.66	22.23	23.35	23.46
OsR498G1220869900.01	0.276	1.146	2.107943	3.40E-11	5.72E-11	0.12	0.22	0.49	1.24	1.4	0.8
OsR498G1220870600.01	0.45	1.77	1.965759	1.10E-13	1.73E-13	0.45	0.47	0.43	1.65	1.42	2.24
OsR498G1220871400.01	0.316	0.1	-1.63955	3.34E-06	6.84E-06	0.27	0.32	0.36	0.08	0.12	0.1
OsR498G1220872300.01	7.633	25.31	1.717287	1.66E-78	1.17E-78	7.46	7.44	8	27.85	24.01	24.07
OsR498G1220875300.01	0.533	1.786	1.680866	8.55E-24	1.07E-23	0.44	0.54	0.62	1.8	1.85	1.71
OsR498G1220875600.01	6.866	2.016	-1.78545	5.35E-63	4.25E-63	6.59	7.3	6.71	1.9	2.27	1.88
OsR498G1220879500.01	0.593	1.66	1.585194	3.73E-10	6.51E-10	0.91	0.44	0.43	1.53	1.71	1.74
OsR498G1220880200.01	0.053	1.883	5.079731	1.67E-09	2.97E-09	0.16	0	0	1.65	1.39	2.61
OsR498G1220880500.01	0.116	1.54	3.726752	1.03E-45	9.65E-46	0.12	0.11	0.12	1.55	1.23	1.84
OsR498G1220885200.01	2.866	26.336	3.167625	0	0	2.37	3.22	3.01	25.64	26.43	26.94
OsR498G1220885500.01	0.113	0.933	3.072182	2.10E-17	3.00E-17	0.09	0.19	0.06	0.68	1.12	1
OsR498G1220886700.01	3.44	1.43	-1.22266	8.54E-20	1.16E-19	3.06	3.41	3.85	1.05	1.63	1.61
OsR498G1220891400.01	7.513	19.923	1.401347	1.04E-89	6.83E-90	7.17	8.24	7.13	20.67	19.86	19.24
OsR498G1220892600.01	16.176	0.636	-5.02574	0	0	16.39	15.43	16.71	0.53	0.55	0.83
OsR498G1220897400.01	7.53	19.2	1.269314	2.97E-47	2.73E-47	7.74	7.37	7.48	19.98	17.47	20.15
OsR498G1220901400.01	0.68	0.14	-2.28217	1.96E-05	4.18E-05	1.08	0.5	0.46	0	0.28	0.14
OsR498G1220901700.01	9.356	34.73	1.921176	0	0	9.34	9.43	9.3	33.98	34.8	35.41
OsR498G1220901900.01	0.136	1.363	3.262152	6.54E-07	1.29E-06	0.14	0	0.27	0.72	1.03	2.34
OsR498G1220903700.01	1.26	0.613	-1.0402	0.0001949	0.0004442	1.2	1.15	1.43	0.76	0.4	0.68

OsR498G1220905600.01	1.476	25.273	4.070522	1.32E-204	5.30E-205	0.92	1.21	2.3	28.86	23.58	23.38
OsR498G1220908900.01	0.393	0	-5.4838	3.79E-07	7.43E-07	0.61	0.32	0.25	0	0	0
OsR498G1220910200.01	4.566	1.206	-1.93108	1.52E-49	1.37E-49	4.15	4.51	5.04	1.29	1.15	1.18
OsR498G1220910900.01	189.78	64.22	-1.55967	0	0	191.43	193.85	184.1	68.31	62.59	61.76
OsR498G1220913000.01	3.536	1.48	-1.31997	2.27E-18	3.18E-18	3.21	3.32	4.08	1.49	1.58	1.37
OsR498G1220913400.01	1.143	2.713	1.244119	5.29E-16	7.81E-16	1.22	1.03	1.18	2.83	2.47	2.84
OsR498G1220919200.01	1.11	0.303	-1.63343	4.51E-11	7.61E-11	1.33	1.22	0.78	0.35	0.37	0.19
OsR498G1220920300.01	134.4	64.143	-1.02584	3.32E-199	1.36E-199	135.48	138.01	129.7	66.62	61.45	64.36
OsR498G1220924800.01	0.176	1.556	3.236157	8.96E-22	1.17E-21	0.13	0.14	0.26	1.79	1.47	1.41
OsR498G1220926300.01	2.41	0.53	-2.37809	9.41E-24	1.18E-23	2.3	2.46	2.47	0.55	0.71	0.33
OsR498G1220930000.01	262.96	42.71	-2.63501	0	0	267.42	257.68	263.8	42.44	42.04	43.65
OsR498G1220931300.01	8.906	4.163	-1.09774	4.12E-41	4.05E-41	8.22	9.05	9.45	4.26	4.17	4.06
OsR498G1220938900.01	21.436	223.98	3.380583	0	0	19.67	23.81	20.83	221.78	225.79	224.38
OsR498G1220940200.01	0.05	0.386	2.978359	1.22E-05	2.58E-05	0.05	0.05	0.05	0.35	0.51	0.3
OsR498G1220940400.01	0.013	0.183	3.563322	2.65E-06	5.40E-06	0	0	0.04	0.23	0.17	0.15
OsR498G1220941200.01	0.036	0.446	3.084154	1.56E-07	2.99E-07	0.08	0	0.03	0.41	0.31	0.62
OsR498G1220943800.01	0.053	0.313	2.472719	1.69E-05	3.61E-05	0.03	0.07	0.06	0.38	0.46	0.1
OsR498G1220945200.01	53.58	129.97	1.273925	0	0	53.11	54.76	52.87	134.67	125.96	129.28
OsR498G1220946600.01	0.01	8	9.553488	1.13E-130	6.01E-131	0	0	0.03	8.54	7.81	7.65
OsR498G1220947700.01	1.026	0.266	-1.96024	3.22E-06	6.59E-06	1.19	0.88	1.01	0.14	0.59	0.07
OsR498G1220949700.01	9.73	4.15	-1.23521	6.17E-43	5.97E-43	8.62	9.64	10.93	4.58	4.17	3.7
OsR498G1220950900.01	3.763	0.826	-2.09285	2.93E-34	3.12E-34	3.78	3.64	3.87	0.63	1.1	0.75
OsR498G1220952600.01	0.023	1.256	5.683616	8.23E-14	1.28E-13	0	0.07	0	1.77	0.69	1.31
OsR498G1220953200.01	2.786	9.226	1.702725	5.48E-115	3.13E-115	2.94	2.62	2.8	8.81	9.14	9.73
OsR498G1220955300.01	1.323	26.463	4.298893	7.55E-116	4.29E-116	2.05	0.93	0.99	28.01	29.2	22.18
OsR498G1220957500.01	0.15	0.84	2.499192	2.13E-06	4.32E-06	0.15	0.23	0.07	0.83	0.85	0.84
OsR498G1220963600.01	15.986	5.22	-1.62601	1.18E-61	9.49E-62	15.04	14.55	18.37	6.03	4.71	4.92
OsR498G1220963800.01	25.53	3.72	-2.93073	0	0	25.8	24.84	25.95	3.64	3.95	3.57
OsR498G1220969300.01	0.136	1.956	3.824031	2.03E-27	2.39E-27	0.18	0.19	0.04	2.19	1.87	1.81
OsR498G1220972100.01	0.823	0.256	-1.74046	1.39E-08	2.55E-08	0.91	0.86	0.7	0.22	0.25	0.3
OsR498G1220972700.01	0.28	1.163	2.129397	5.89E-21	7.80E-21	0.31	0.26	0.27	1.17	1.22	1.1
OsR498G1220973100.01	0.176	1.293	2.932845	4.84E-19	6.69E-19	0.32	0.18	0.03	1.21	1.27	1.4
OsR498G1220981300.01	1.73	10.81	2.521996	4.26E-154	2.04E-154	2.08	1.46	1.65	11.11	10.91	10.41
OsR498G1220982600.01	3.46	8.47	1.271933	7.52E-51	6.68E-51	3.34	3.41	3.63	7.82	8.98	8.61
OsR498G1220983000.01	4.63	33.196	2.835792	3.84E-224	1.45E-224	4.81	4.83	4.25	37.24	33.69	28.66
OsR498G1220984200.01	2.14	0.506	-2.07437	1.05E-37	1.07E-37	2	2.36	2.06	0.57	0.32	0.63
OsR498G1220984300.01	1.89	0.686	-1.48227	1.49E-07	2.87E-07	1.29	1.56	2.82	0.74	0.64	0.68
OsR498G1220987300.01	0.05	0.44	3.177264	1.13E-09	2.00E-09	0.03	0.03	0.09	0.41	0.52	0.39
OsR498G1220988600.01	1.726	0.303	-2.49398	1.61E-35	1.69E-35	1.7	1.94	1.54	0.35	0.29	0.27
OsR498G1220991100.01	0.243	1.546	2.664251	2.39E-07	4.64E-07	0	0.38	0.35	1.74	0.89	2.01
OsR498G1220994200.01	0.313	1.19	1.847115	3.14E-17	4.51E-17	0.49	0.22	0.23	1.37	1.23	0.97
OsR498G1220995600.01	2.183	5.28	1.213212	4.12E-28	4.78E-28	2.08	2.24	2.23	5.6	5.01	5.23
OsR498G1221005700.01	11.866	4.753	-1.32295	1.06E-85	7.11E-86	12.05	11.65	11.9	4.97	5.11	4.18
OsR498G1221006800.01	1.08	2.513	1.220332	2.06E-12	3.34E-12	1.29	1.16	0.79	2.43	2.44	2.67
OsR498G1221008600.01	65.106	31.3	-1.07587	1.12E-244	3.97E-245	65.31	62.17	67.84	31.74	30.71	31.45
OsR498G1221009500.01	7.603	2.39	-1.65491	4.71E-106	2.81E-106	7.75	7.12	7.94	2.74	2.17	2.26
OsR498G1221014600.01	0.1	1.306	3.711172	2.02E-23	2.56E-23	0.11	0.13	0.06	1.4	1.54	0.98
OsR498G1221014700.01	0.043	0.56	3.751799	4.11E-06	8.45E-06	0	0.12	0.01	0.63	0.62	0.43
OsR498G1221019400.01	114.94	344.09	1.55936	0	0	115.27	113.5	116.1	350.98	339.99	341.3
OsR498G1221020200.01	0.31	0.08	-1.93887	1.82E-08	3.37E-08	0.36	0.29	0.28	0.08	0.09	0.07
OsR498G1221020400.01	250.52	105.02	-1.27672	0	0	254.2	240.34	257	105.48	106.56	103.01
OsR498G1221020700.01	102.17	2.216	-5.53148	0	0	94.61	103.56	108.4	2.2	2.4	2.05
OsR498G1221022400.01	11.513	2.58	-2.16746	1.24E-84	8.38E-85	12.56	9.98	12	2.86	2.4	2.48
OsR498G1221022700.01	5.993	15.78	1.383714	2.18E-105	1.30E-105	4.9	7	6.08	15.79	16.02	15.53
OsR498G1221022900.01	5.776	14.093	1.33779	6.18E-84	4.19E-84	6.1	5.84	5.39	14.62	14.2	13.46
OsR498G1221023500.01	0.926	2.333	1.329267	2.49E-08	4.63E-08	1.1	0.97	0.71	2.5	2.84	1.66
OsR498G1221025700.01	8.963	17.686	1.088298	3.32E-42	3.23E-42	9.04	8.39	9.46	19.21	16.21	17.64
OsR498G1221028300.01	0.24	0.06	-2.02374	0.0006394	0.0015165	0.21	0.08	0.43	0.11	0.07	0
OsR498G1221028600.01	0.65	0.07	-3.18778	1.10E-06	2.20E-06	0.55	0.72	0.68	0.14	0	0.07
OsR498G1221030800.01	0.073	0.62	3.03976	6.82E-06	1.42E-05	0.07	0.08	0.07	0.46	0.47	0.93
OsR498G1221033500.01	6.173	12.55	1.010614	7.89E-17	1.14E-16	5.06	6.88	6.58	11.84	12.81	13
OsR498G1221036900.01	0.503	2.216	2.134917	3.91E-18	5.49E-18	0.55	0.48	0.48	2.41	2.25	1.99
OsR498G1221038000.01	15.09	4.24	-1.83572	1.67E-89	1.10E-89	14.88	15.51	14.88	4.86	3.53	4.33
OsR498G1221038900.01	0.403	0	-5.35256	1.16E-06	2.33E-06	0.46	0.48	0.27	0	0	0
OsR498G1221041300.01	6.853	15.456	1.168183	1.57E-67	1.20E-67	6.24	6.81	7.51	15.12	15.99	15.26
OsR498G1221042000.01	2.45	1.123	-1.1323	2.18E-09	3.89E-09	2.9	2.32	2.13	1.15	1.23	0.99
OsR498G1221044900.01	0.366	0.04	-3.00463	3.06E-07	5.98E-07	0.29	0.47	0.34	0.03	0	0.09
OsR498G1221049100.01	0.096	0.543	2.447845	3.07E-11	5.16E-11	0.11	0.09	0.09	0.53	0.6	0.5
OsR498G1221050000.01	4.846	1.636	-1.57135	7.18E-39	7.25E-39	5.36	4.37	4.81	1.56	1.49	1.86

OsR498G1221050600.01	14.66	28.336	1.070503	3.40E-214	1.33E-214	16.44	14.62	12.92	28.03	29.41	27.57
OsR498G1221051500.01	59.073	16.233	-1.90357	0	0	57.31	59.59	60.32	16.76	15.42	16.52
OsR498G1221056000.01	0	2.973	7.88525	1.57E-26	1.88E-26	0	0	0	2.43	2.56	3.93
OsR498G1221058200.01	1643.6	396.03	-2.06051	0	0	1714.7	1635.5	1581	420.59	385.49	382.01
OsR498G1221059300.01	1533.9	331.61	-2.21665	0	0	1609.5	1515.8	1476	354.2	314.78	325.84
OsR498G1221060900.01	37.81	11.96	-1.63076	9.57E-171	4.32E-171	42.17	36.66	34.6	12.16	11.02	12.7
OsR498G1221061600.01	512.6	3230.1	2.653201	0	0	509.84	516.33	511.6	3320	3139.2	3231
OsR498G1221062400.01	0	0.576	7.105849	3.70E-17	5.32E-17	0	0	0	0.53	0.39	0.81
OsR498G1221072900.01	9.896	3.976	-1.28966	6.01E-39	6.06E-39	9.82	9.58	10.29	3.74	3.58	4.61
OsR498G1221078700.01	5.52	1.74	-1.65635	1.26E-41	1.24E-41	5.41	4.98	6.17	1.91	1.8	1.51
OsR498G1221087400.01	6.166	1.37	-2.18069	4.22E-87	2.81E-87	6.23	5.82	6.45	1.19	1.6	1.32
OsR498G1221087800.01	7.966	1.053	-2.93581	6.56E-171	2.96E-171	7.35	7.61	8.94	0.96	1.22	0.98
OsR498G1221088200.01	2.446	18.906	2.934438	1.60E-108	9.43E-109	2.02	2.39	2.93	21.45	17.93	17.34
OsR498G1221088800.01	0.666	3.03	2.168235	4.33E-28	5.04E-28	0.79	0.49	0.72	2.52	3.1	3.47
OsR498G1221090600.01	3.956	1.843	-1.21101	8.74E-15	1.33E-14	3.32	3.79	4.76	1.67	2.1	1.76
OsR498G1221091800.01	0.013	0.186	3.624723	0.0004041	0.0009439	0	0	0.04	0.09	0.38	0.09
OsR498G1221093800.01	62.616	6.113	-3.3638	0	0	63.5	62.96	61.39	5.74	6.96	5.64
OsR498G1221094300.01	10.256	0.016	-9.13267	1.84E-106	1.10E-106	10.62	9.32	10.83	0	0	0.05
OsR498G1221094600.01	1.383	9.68	2.778278	0	0	1.4	1.4	1.35	9.05	10.27	9.72
OsR498G1221095700.01	4.626	1.316	-1.86687	5.83E-134	3.04E-134	4.42	4.66	4.8	1.39	1.15	1.41
OsR498G1221096300.01	13.76	1	-3.7906	4.99E-115	2.85E-115	14.79	13.73	12.76	0.96	0.91	1.13
OsR498G1221097200.01	3.123	0.216	-4.32014	3.93E-68	3.00E-68	3.48	2.61	3.28	0.25	0.27	0.13
OsR498G1221097900.01	0.176	0.03	-2.69721	0.0001045	0.0002337	0.11	0.14	0.28	0.03	0.06	0
OsR498G1221102500.01	8.033	3.42	-1.20045	1.42E-20	1.89E-20	6.85	8.63	8.62	3.56	3.32	3.38
OsR498G1221102800.01	2.856	1.283	-1.16748	8.55E-11	1.46E-10	2.73	2.73	3.11	1.14	1.43	1.28
OsR498G1221105700.01	3.213	0.65	-2.32481	1.58E-27	1.85E-27	2.37	4.01	3.26	0.85	0.53	0.57
OsR498G1221106400.01	13.326	3.746	-1.91286	5.38E-124	2.93E-124	13.67	12.87	13.44	3.81	3.85	3.58
OsR498G1221122200.01	0.01	0.16	4.127223	1.87E-05	4.00E-05	0.03	0	0	0.2	0.17	0.11
OsR498G1221126000.01	0.626	0.033	-4.20817	7.92E-06	1.66E-05	0.58	1.02	0.28	0	0.1	0
OsR498G1221129600.01	0.27	0.03	-3.21275	0.0003163	0.0007319	0	0.35	0.46	0	0	0.09
OsR498G1221135300.01	66.08	31.46	-1.07954	7.07E-132	3.72E-132	65.55	65.78	66.91	30.18	33	31.2
OsR498G1221137200.01	2.49	8.646	1.80306	1.09E-120	6.07E-121	2.44	2.77	2.26	8.01	9.26	8.67
OsR498G1221142800.01	3.456	0.14	-4.70843	6.44E-50	5.78E-50	2.75	4.09	3.53	0.04	0.38	0
OsR498G1221146700.01	43.463	181.9	2.064544	0	0	44.88	41.96	43.55	184.1	177.82	183.79
OsR498G1221146900.01	153.83	25.196	-2.60295	0	0	154.38	156.08	151	23.27	25.97	26.35
OsR498G1221156800.01	0	0.18	4.361688	0.0005877	0.0013892	0	0	0	0.02	0.14	0.38
OsR498G1221156900.01	0.31	0	-6.28217	4.40E-11	7.43E-11	0.21	0.29	0.43	0	0	0
OsR498G1221157700.01	0.973	7.246	2.894421	1.78E-72	1.31E-72	1.06	1.07	0.79	8.53	6.92	6.29
OsR498G1221158500.01	30.483	1.723	-4.13883	0	0	30.15	31.41	29.89	1.85	1.55	1.77
OsR498G1221161500.01	3.823	0.053	-6.20817	5.31E-37	5.47E-37	4.15	2.94	4.38	0.08	0.08	0
OsR498G1221165900.01	31.936	15.353	-1.04866	4.53E-76	3.24E-76	33.36	32.29	30.16	15.37	14.52	16.17
OsR498G1221169700.01	12.523	4.226	-1.65522	1.15E-147	5.65E-148	12.39	11.51	13.67	3.94	4.62	4.12
OsR498G1221171700.01	7.26	3.213	-1.17239	5.15E-35	5.45E-35	6.91	7.66	7.21	3.35	3.29	3
OsR498G1221182500.01	0.966	0.15	-2.66919	1.21E-26	1.44E-26	0.85	0.95	1.1	0.19	0.14	0.12
OsR498G1221184600.01	5.98	12.04	1.014885	2.79E-81	1.93E-81	5.89	5.75	6.3	11.38	12.92	11.82
OsR498G1221185500.01	0.01	0.156	4.03976	3.45E-05	7.49E-05	0	0.03	0	0.15	0.11	0.21
OsR498G1221188800.01	0.526	1.306	1.327741	1.84E-20	2.47E-20	0.47	0.65	0.46	1.2	1.28	1.44
OsR498G1221192400.01	65.086	19.24	-1.64667	0	0	66.05	64.56	64.65	17.58	20.03	20.11
OsR498G1221199100.01	0.53	2.346	2.139296	9.57E-16	1.42E-15	0.51	0.48	0.6	2.89	1.73	2.42
OsR498G1221199400.01	0.056	0.293	2.361688	2.29E-06	4.65E-06	0.07	0.07	0.03	0.3	0.28	0.3
OsR498G1221201400.01	13.3	4.52	-1.56398	6.76E-53	5.88E-53	12.37	14.54	12.99	4.5	4.37	4.69
OsR498G1221203100.01	2.566	0.893	-1.6192	1.97E-06	3.99E-06	2.89	2.62	2.19	1	0.97	0.71
OsR498G1221203700.01	0.796	0.293	-1.455	0.0009085	0.0021791	1.31	0.68	0.4	0.28	0.34	0.26
OsR498G1221216700.01	1.14	0	-5.56372	1.84E-07	3.54E-07	0.94	0.62	1.86	0	0	0
OsR498G1221222200.01	33.053	91.113	1.462408	0	0	32.61	33.21	33.34	90.01	91.61	91.72
OsR498G1221222700.01	0.453	0	-5.35256	1.16E-06	2.33E-06	0.38	0.68	0.3	0	0	0
OsR498G1221223500.01	0	4.546	10.03694	5.95E-86	3.98E-86	0	0	0	4.79	4.4	4.45
OsR498G1221227900.01	0	7.52	10.66547	6.99E-120	3.89E-120	0	0	0	8.21	7.18	7.17
OsR498G1221234700.01	1.32	0.34	-2.14112	2.36E-05	5.08E-05	1.53	1.5	0.93	0.41	0.11	0.5
OsR498G1221234900.01	1.896	0.066	-4.88994	1.06E-77	7.49E-78	1.93	1.99	1.77	0.09	0.07	0.04
OsR498G1221239800.01	13.683	1.77	-2.97313	4.78E-239	1.73E-239	14.81	12.98	13.26	1.55	1.73	2.03
OsR498G1221241800.01	2.033	0.846	-1.28239	2.58E-05	5.57E-05	2.15	1.87	2.08	0.87	1.08	0.59
OsR498G1221242000.01	76.536	24.256	-1.70136	0	0	77.86	75.33	76.42	25.04	24.33	23.4
OsR498G1221242800.01	0.656	0.246	-1.4316	0.000288	0.0006648	1.18	0.32	0.47	0.27	0.34	0.13
OsR498G1221257500.01	1.33	0.343	-1.95401	5.50E-25	6.74E-25	1.32	1.26	1.41	0.47	0.27	0.29
OsR498G1221260600.01	0.603	0.183	-1.92509	4.51E-05	9.86E-05	0.52	0.53	0.76	0.07	0.28	0.2
OsR498G1221260700.01	0.27	0.01	-4.93353	1.06E-11	1.75E-11	0.24	0.36	0.21	0	0	0.03
OsR498G1221261800.01	0.583	12.516	4.436728	5.44E-280	1.76E-280	0.56	0.77	0.42	13.58	12.26	11.71
OsR498G1221277200.01	0.266	2.27	2.862882	1.63E-31	1.81E-31	0.15	0.3	0.35	1.9	2.6	2.31

OsR498G1221281600.01	13214	2037.6	-2.70423	0	0	13300	13330	13013	2126.4	1993.9	1992.6
OsR498G1221303800.01	196.57	606.87	1.657141	0	0	198.49	201.8	189.4	601.23	615.79	603.58
OsR498G1221305400.01	0.676	1.443	1.216638	0.0001988	0.0004533	0.71	0.58	0.74	1.45	1.56	1.32
OsR498G1221320300.01	0.016	0.67	5.249213	1.29E-10	2.21E-10	0.05	0	0	0.53	0.88	0.6
OsR498G1221321500.01	7.743	23.526	1.598436	1.53E-221	5.85E-222	8.27	7.43	7.53	22.15	24.17	24.26
OsR498G1221325200.01	127.36	15.52	-2.80947	0	0	133.13	123.76	125.2	14.45	16.45	15.66
OsR498G1221325700.01	2.073	0	-6.5452	7.75E-13	1.24E-12	1.28	2.76	2.18	0	0	0
OsR498G1221326600.01	7.34	2.483	-1.55854	4.15E-81	2.87E-81	7.45	8	6.57	2.5	2.33	2.62
OsR498G1221327100.01	0.933	0.233	-2.0477	2.14E-05	4.58E-05	1	0.44	1.36	0.09	0	0.61
OsR498G1221334200.01	5.62	2.306	-1.30538	4.55E-07	8.94E-07	6.58	5.47	4.81	3.54	1.69	1.69
OsR498G1221346400.01	8.043	18.663	1.253216	9.56E-109	5.61E-109	8.3	8.3	7.53	18.92	18.8	18.27
OsR498G1221348500.01	8498.2	1005.4	-3.09057	0	0	8350.1	8517.5	8627	1007.7	1007.4	1001
OsR498G1221349500.01	2961.1	629.48	-2.24171	0	0	3026.4	2886.3	2970	648.28	617.72	622.44
OsR498G1221349800.01	26.23	4.58	-2.55177	2.63E-76	1.88E-76	24.08	24.62	29.99	5.17	4.52	4.05
OsR498G1221350300.01	2521.1	140.6	-4.15557	0	0	2339.1	2541.4	2683	139.24	141.08	141.49
OsR498G1221351500.01	122.94	42.873	-1.55208	0	0	123.57	122.41	122.9	41.48	44.08	43.06
OsR498G1221351800.01	5.693	2.48	-1.26602	1.21E-84	8.16E-85	5.88	5.56	5.64	2.52	2.52	2.4
OsR498G1221356400.01	17.6	8.363	-1.20847	7.55E-69	5.73E-69	19.17	17.41	16.22	8.54	7.92	8.63
OsR498G1221368800.01	5.986	23.606	1.985772	1.63E-257	5.59E-258	5.55	6.07	6.34	22.89	23.89	24.04
OsR498G1221375600.01	0.826	0.22	-1.9217	1.97E-05	4.21E-05	0.3	1.25	0.93	0	0.66	0
OsR498G1221382800.01	0.09	0	-4.41967	0.0003895	0.0009082	0.06	0.21	0	0	0	0
OsR498G1221402700.01	2.783	0.92	-1.6041	1.88E-09	3.35E-09	3.47	2.96	1.92	0.51	1.65	0.6
OsR498G1221404900.01	0.62	0.086	-2.84777	1.37E-07	2.63E-07	0.65	0.68	0.53	0.05	0.11	0.1
OsR498G1221412700.01	1168.4	511.29	-1.19574	0	0	1154.6	1170.5	1180	532.17	498.77	502.94
OsR498G1221413300.01	1552.6	160.01	-3.27695	0	0	1588.6	1526	1543	173.25	156.42	150.37
OsR498G1221425300.01	1.2	0.32	-1.92987	9.50E-16	1.41E-15	1.22	0.99	1.39	0.18	0.46	0.32
OsR498G1221431000.01	0.113	0	-4.86713	3.63E-05	7.88E-05	0.07	0.16	0.11	0	0	0
OsR498G1221435000.01	127.92	12.23	-3.42423	0	0	138.14	126.34	119.3	12.43	13.71	10.55
OsR498G1221448500.01	0.066	22.04	8.352643	1.24E-196	5.13E-197	0.13	0.07	0	23.96	19.54	22.62
OsR498G1221450500.01	0.343	0.046	-2.86713	0.0004299	0.0010056	0.41	0.22	0.4	0.07	0	0.07
OsR498G1221451200.01	0.64	1.953	1.591899	7.54E-39	7.61E-39	0.52	0.75	0.65	1.96	1.74	2.16
OsR498G1221453900.01	1.11	37.54	5.074322	0	0	1.12	0.99	1.22	34.77	39.5	38.35
OsR498G1221464500.01	11.186	1.703	-2.74451	1.74E-20	2.32E-20	13.63	11.94	7.99	1.99	1.98	1.14
OsR498G1221464700.01	5.663	2.46	-1.20817	7.11E-18	1.01E-17	4.93	5.83	6.23	2.67	1.76	2.95
OsR498G1221467600.01	0.38	5.42	3.881062	1.04E-28	1.20E-28	0.17	0.13	0.84	5.22	5.42	5.62
OsR498G1221467900.01	4.543	0.136	-5.07357	1.03E-17	1.46E-17	4.31	4.36	4.96	0	0	0.41
OsR498G1221468700.01	0.866	4.82	2.472034	1.01E-59	8.24E-60	1.09	0.87	0.64	4.41	4.99	5.06
OsR498G1221469100.01	260.4	6.646	-5.34532	0	0	261.3	265.3	254.6	6.23	7.15	6.56
OsR498G1221471600.01	1.746	0.186	-3.23878	5.60E-48	5.13E-48	1.76	1.51	1.97	0.16	0.13	0.27
OsR498G1221488200.01	8.146	3.306	-1.30381	9.95E-34	1.07E-33	8.74	7.96	7.74	4.16	2.83	2.93
OsR498G1221499700.01	4.216	2.043	-1.08819	1.41E-24	1.74E-24	4.3	4.43	3.92	1.73	2.17	2.23
OsR498G1221501200.01	5.956	13.3	1.158429	9.12E-81	6.33E-81	6.41	6.02	5.44	13.06	13.54	13.3
OsR498G1221505500.01	0.326	0.733	1.159059	9.15E-05	0.0002038	0.27	0.35	0.36	0.62	1.05	0.53
OsR498G1221506900.01	0.496	0.106	-2.31074	3.92E-08	7.35E-08	0.52	0.41	0.56	0.13	0.04	0.15
OsR498G1221512900.01	6.516	1.01	-2.69549	2.97E-37	3.05E-37	6.43	7.65	5.47	1.3	0.94	0.79
OsR498G1221536100.01	0.53	0.166	-1.68722	1.07E-09	1.89E-09	0.46	0.57	0.56	0.1	0.21	0.19
OsR498G1221541200.01	3.623	7.613	1.056015	3.31E-32	3.62E-32	4.22	3.64	3.01	7.84	7.31	7.69
OsR498G1221562700.01	8.44	3.53	-1.55572	6.57E-112	3.80E-112	8.04	9.24	8.04	3.21	4.34	3.04
OsR498G1221572700.01	0.02	0.186	3.287688	2.92E-05	6.31E-05	0.03	0	0.03	0.17	0.21	0.18
OsR498G1221574600.01	0.15	0.753	2.329267	1.83E-07	3.52E-07	0.3	0.05	0.1	1.11	0.63	0.52
OsR498G1221587200.01	9.253	23.6	1.343608	9.58E-83	6.54E-83	8.26	9.6	9.9	23.68	24.3	22.82
OsR498G1221589400.01	0.703	2.063	1.491221	2.95E-27	3.47E-27	0.59	0.76	0.76	2.18	2.1	1.91
OsR498G1221589500.01	11.593	70.63	2.604924	0	0	11.88	12.37	10.53	76.25	72.02	63.62
OsR498G1221591800.01	0.416	6.49	3.955368	1.06E-71	7.81E-72	0.62	0.47	0.16	7.15	7.26	5.06
OsR498G1221602100.01	20.983	3.256	-2.638	1.52E-264	5.12E-265	21.51	19.64	21.8	3.52	3.15	3.1
OsR498G1221602400.01	4.49	0.763	-2.55502	4.59E-104	2.77E-104	4.57	4.39	4.51	0.83	0.65	0.81
OsR498G1221606400.01	0.193	0.123	-1.70761	1.31E-05	2.78E-05	0.09	0.3	0.19	0.1	0.16	0.11
OsR498G1221607600.01	5.82	2.133	-1.49504	1.54E-41	1.51E-41	5.23	5.7	6.53	1.54	2.12	2.74
OsR498G1221610800.01	2.076	0.626	-1.71155	2.84E-35	2.99E-35	1.95	2.16	2.12	0.72	0.46	0.7
OsR498G1221620100.01	7.87	1.56	-2.33326	3.99E-113	2.30E-113	7.59	7.07	8.95	1.79	1.44	1.45
OsR498G1221620800.01	20.36	10.163	-1.01212	1.43E-35	1.50E-35	22.17	19.44	19.47	9.24	10.65	10.6
OsR498G1221621600.01	19.953	4.893	-2.13417	3.45E-244	1.23E-244	20.02	20.19	19.65	4.71	5	4.97
OsR498G1221624200.01	0.146	0.01	-4.0477	2.60E-05	5.61E-05	0.23	0.08	0.13	0	0.03	0
OsR498G1221645800.01	0.123	0.283	1.2961	0.000347	0.0008056	0.08	0.2	0.09	0.29	0.38	0.18
OsR498G1221647700.01	4.686	2.193	-1.10963	3.78E-12	6.19E-12	5.17	3.66	5.23	2.47	2.14	1.97
OsR498G1221648900.01	0.6	0.13	-1.96024	0.0003753	0.0008736	0.75	0.59	0.46	0.1	0.19	0.1
OsR498G1221649100.01	0.333	0.08	-2.05335	1.15E-08	2.12E-08	0.34	0.31	0.35	0.06	0.08	0.1
OsR498G1221651400.01	13.753	3.253	-2.06437	1.19E-223	4.53E-224	12.66	14.89	13.71	3.4	3.16	3.2
OsR498G1221655300.01	0.43	0.106	-2.6232	0.0001807	0.000411	0.59	0.22	0.48	0.07	0.13	0.12

OsR498G1221663400.01	104.25	25.343	-2.12782	0	0	114.19	95.69	102.9	27.19	25.59	23.25
OsR498G1221670300.01	59.2	27.956	-1.09606	2.40E-139	1.23E-139	61.02	59.29	57.29	26.89	27.86	29.12
OsR498G1221671000.01	0.266	0.603	1.204147	4.61E-05	0.0001007	0.24	0.25	0.31	0.74	0.51	0.56
OsR498G1221672600.01	33.146	322.2	3.273318	0	0	32.72	33.6	33.12	320.1	321.37	325.12
OsR498G1221673100.01	3.216	7.5	1.204777	6.93E-54	5.98E-54	2.74	3.4	3.51	5.97	8.82	7.71
OsR498G1221685300.01	0.023	0.883	5.107082	8.33E-20	1.13E-19	0	0	0.07	1.39	0.78	0.48
OsR498G1221687500.01	78.64	30.56	-1.36999	3.36E-271	1.11E-271	79.87	79.88	76.17	30	29.62	32.06
OsR498G1221688500.01	12.336	31.746	1.360817	2.07E-204	8.34E-205	13.63	12.54	10.84	32.1	32.96	30.18
OsR498G1221689700.01	2.8	1.643	-1.07278	3.47E-20	4.67E-20	2.69	2.6	3.11	1.61	1.41	1.91
OsR498G1221690000.01	33.17	0.39	-6.35394	1.06E-282	3.42E-283	34.44	32.36	32.71	0.63	0.08	0.46
OsR498G1221690700.01	10.543	4.91	-1.10888	1.42E-26	1.69E-26	10.12	11.37	10.14	4.26	5.03	5.44
OsR498G1221691000.01	15.23	2.44	-2.65198	2.58E-121	1.43E-121	15.39	16.29	14.01	2.43	2.06	2.83
OsR498G1221698200.01	1.01	2.393	1.244691	1.50E-13	2.35E-13	1.33	1.03	0.67	2.13	2.85	2.2
OsR498G1221698700.01	0.296	0	-4.66068	0.0001179	0.0002647	0.27	0.29	0.33	0	0	0
OsR498G1221700900.01	0.15	0.006	-4.66068	1.30E-07	2.49E-07	0.19	0.11	0.15	0.02	0	0
OsR498G1221701800.01	0.19	8.296	5.462666	6.58E-104	3.97E-104	0.22	0.24	0.11	9.94	7.19	7.76
OsR498G1221702600.01	8.013	23.733	1.563515	1.68E-142	8.44E-143	7.71	8.63	7.7	23.19	24.77	23.24
OsR498G1221702900.01	0	0.47	6.287688	7.29E-11	1.24E-10	0	0	0	0.4	0.38	0.63
OsR498G1221703100.01	7.453	17.163	1.206785	3.89E-83	2.65E-83	7.41	7.3	7.65	17.7	16.65	17.14
OsR498G1221705000.01	7.093	45.54	2.674283	0	0	7.34	6.82	7.12	47.61	44.97	44.04
OsR498G1221706400.01	0.096	2.043	4.383168	4.14E-47	3.82E-47	0.03	0.09	0.17	2.1	2.12	1.91
OsR498G1221708200.01	9.256	3.46	-1.45774	1.87E-68	1.43E-68	8.35	9.81	9.61	4.1	3.5	2.78
OsR498G1221709000.01	67.95	31.56	-1.11672	1.18E-106	7.01E-107	68.75	67.66	67.44	30.09	32.46	32.13
OsR498G1221710000.01	13.673	29.256	1.13208	1.05E-163	4.85E-164	13.81	13.16	14.05	29.21	30.17	28.39
OsR498G1221710800.01	57.346	26.91	-1.10105	0	0	57.74	56.33	57.97	26.05	25.78	28.9
OsR498G1221715900.01	1.913	0.51	-1.92071	4.94E-30	5.57E-30	2.33	1.57	1.84	0.67	0.38	0.48
OsR498G1221716600.01	16.373	86.86	2.39692	0	0	17.33	15.77	16.02	87.73	86.07	86.78
OsR498G1221722100.01	13.02	0.506	-4.69592	4.44E-85	2.99E-85	13.36	12.09	13.61	0.75	0.33	0.44
OsR498G1221723300.01	0.05	1.17	4.588197	1.70E-28	1.96E-28	0.06	0.03	0.06	0.97	1.22	1.32
OsR498G1221724500.01	0.466	0.093	-2.214	4.09E-09	7.37E-09	0.47	0.41	0.52	0.11	0.11	0.06
OsR498G1221725400.01	1.283	2.73	1.091599	6.74E-13	1.08E-12	1.21	1.53	1.11	2.58	2.62	2.99
OsR498G1221726100.01	0	0.106	4.847115	5.08E-05	0.0001113	0	0	0	0.09	0.14	0.09
OsR498G1221728100.01	2.303	2.24	1.013355	1.97E-27	2.31E-27	2.36	2.24	2.31	2.36	1.91	2.45
OsR498G1221728300.01	24.6	12.586	-1.04221	6.84E-62	5.49E-62	24.62	23.06	26.12	11.88	13.52	12.36
OsR498G1221728800.01	20.896	6.646	-1.65658	9.38E-121	5.20E-121	20.77	21.29	20.63	7.27	6.24	6.43
OsR498G1221729000.01	2.443	0.553	-2.17174	6.06E-12	9.97E-12	2.58	2.01	2.74	0.26	0.53	0.87
OsR498G1221757300.01	40.276	15.663	-1.38077	3.65E-276	1.19E-276	41.12	40.87	38.84	15.25	15.92	15.82
OsR498G1221759000.01	84.67	33.673	-1.34814	2.20E-231	8.19E-232	89.29	83.96	80.76	35.69	30.72	34.61
OsR498G1221773900.01	1.48	0	-5.41967	6.63E-07	1.31E-06	1.79	1.69	0.96	0	0	0
OsR498G1221774300.01	39.65	1.28	-4.96624	0	0	43	38.51	37.44	0.88	1.55	1.41
OsR498G1221774900.01	0.74	0.143	-2.39881	3.50E-15	5.27E-15	0.69	0.73	0.8	0.11	0.17	0.15
OsR498G1221776800.01	38.993	15.5	-1.36139	3.85E-92	2.48E-92	35.23	41.33	40.42	16.19	14.33	15.98
OsR498G1221785200.01	24.08	10.83	-1.15925	4.68E-101	2.87E-101	24.39	24.58	23.27	10.44	11.81	10.24
OsR498G1221786300.01	9.39	2.853	-1.72807	3.88E-55	3.31E-55	10.21	8.63	9.33	2.77	3.16	2.63
OsR498G1221818600.01	3.59	1.403	-1.42216	1.25E-25	1.51E-25	3.8	3.47	3.5	1.47	1.57	1.17
OsR498G1221837500.01	0.056	0.266	2.584081	7.18E-07	1.42E-06	0.03	0.06	0.08	0.26	0.27	0.27
OsR498G1221837800.01	12.423	202.41	4.029589	0	0	12.77	11.98	12.52	200.53	206.85	199.86
OsR498G1221841100.01	2.956	1.166	-1.34201	1.02E-27	1.19E-27	2.97	2.99	2.91	1.27	1.17	1.06
OsR498G1221847800.01	0.68	0	-6.00463	1.68E-09	3.00E-09	0.8	0.58	0.66	0	0	0
OsR498G1221852200.01	0.073	0.583	2.99085	2.17E-21	2.85E-21	0.09	0.05	0.08	0.45	0.6	0.7
OsR498G1221854600.01	0.756	0.166	-2.20069	1.61E-18	2.25E-18	0.92	0.74	0.61	0.12	0.2	0.18
OsR498G1221856800.01	22.22	8.156	-1.44402	5.85E-282	1.89E-282	21.2	22.23	23.23	8.6	8.25	7.62
OsR498G1221863300.01	1.24	0.306	-2.02252	2.85E-09	5.12E-09	1.48	1.02	1.22	0.48	0.28	0.16
OsR498G1221863500.01	141.46	41.14	-1.78973	0	0	144.92	141.05	138.4	43.7	39.8	39.92
OsR498G1221866100.01	2.503	39.266	3.960157	0	0	2.53	2.44	2.54	37.45	38.73	41.62
OsR498G1221868800.01	3.426	1.346	-1.35698	1.32E-40	1.30E-40	3.37	3.34	3.57	1.21	1.35	1.48
OsR498G1221868900.01	5.383	1.25	-2.05431	4.29E-66	3.32E-66	5.42	5.11	5.62	1.22	1.23	1.3
OsR498G1221874400.01	17.906	4.256	-2.06856	3.29E-277	1.07E-277	16.82	18.13	18.77	4.7	4.03	4.04
OsR498G1221877200.01	22.93	65.396	1.49637	5.52E-227	2.08E-227	23.06	22.73	23	68.53	64.95	62.71
OsR498G1221881500.01	0.426	31.893	6.441172	0	0	0.59	0.28	0.41	33.63	30.79	31.26
OsR498G1221882000.01	35.35	12.856	-1.47223	1.60E-284	5.13E-285	34.78	35.15	36.12	12.13	13.38	13.06
OsR498G1221882700.01	24.6	77.186	1.657626	0	0	24.58	25.59	23.63	77.87	76.92	76.77
OsR498G1221883500.01	4.823	2.366	-1.03619	5.44E-23	6.92E-23	4.92	5.14	4.41	2.29	2.34	2.47
OsR498G1221888700.01	49.456	1.136	-5.3809	0	0	51.57	48.16	48.64	1.14	1.28	0.99
OsR498G1221890200.01	0	11.736	10.57504	2.58E-114	1.48E-114	0	0	0	10.7	11.3	13.21
OsR498G1221892000.01	0.72	0.293	-1.35676	1.71E-09	3.04E-09	0.62	0.92	0.62	0.3	0.33	0.25
OsR498G1221893900.01	1.123	4.016	1.862712	1.34E-64	1.05E-64	1.06	0.88	1.43	4.04	4.08	3.93
OsR498G1221894200.01	0.793	0.123	-2.69296	1.69E-18	2.36E-18	0.94	0.75	0.69	0.19	0.09	0.09
OsR498G1221894900.01	1.873	0.26	-3.11155	4.31E-41	4.24E-41	1.96	1.58	2.08	0.36	0.25	0.17

OsR498G1221896800.01	62.543	25.8	-1.28283	7.68E-299	2.36E-299	61.92	62.14	63.57	27.07	23.92	26.41
OsR498G1221902200.01	0	0.286	4.361688	0.0005877	0.0013892	0	0	0	0.43	0.17	0.26
OsR498G1221905700.01	9.043	0.2	-5.54063	8.50E-124	4.64E-124	10.03	8.52	8.58	0.27	0.22	0.11
OsR498G1221909300.01	0.236	1.626	2.782264	1.74E-15	2.60E-15	0.33	0.22	0.16	1.91	1.8	1.17
OsR498G1221909500.01	0.27	0.106	-1.33221	0.0002767	0.000638	0.26	0.24	0.31	0.07	0.17	0.08
OsR498G1221910700.01	0.106	0.98	3.155237	2.12E-06	4.31E-06	0	0.11	0.21	1.12	0.8	1.02
OsR498G1221910900.01	0.113	0.69	2.563322	4.56E-05	9.98E-05	0.26	0	0.08	1.16	0.64	0.27
OsR498G1221916500.01	2.46	0.77	-1.82052	2.42E-23	3.06E-23	2.52	2.67	2.19	0.6	0.98	0.73
OsR498G1221918700.01	11.95	25.836	1.105516	4.36E-169	1.98E-169	11.47	11.69	12.69	27.11	25.3	25.1
OsR498G1221922000.01	0.073	0.75	3.332542	7.09E-22	9.21E-22	0.08	0.04	0.1	0.75	0.83	0.67
OsR498G1221924600.01	227.41	111.68	-1.03319	0	0	233.87	217.08	231.3	112.76	112.64	109.64
OsR498G1221927000.01	11.58	2.343	-2.28987	2.64E-185	1.14E-185	11.33	11.57	11.84	2.47	2.11	2.45
OsR498G1221928600.01	0.026	0.18	2.847115	3.87E-05	8.44E-05	0.05	0.03	0	0.1	0.13	0.31
OsR498G1221932100.01	89.976	11.22	-3.00726	0	0	88.15	90.66	91.12	10.34	11.04	12.28
OsR498G1221932600.01	0.166	0.016	-3.35256	1.36E-05	2.87E-05	0.19	0.08	0.23	0.02	0.03	0
OsR498G1221932800.01	13.426	1.43	-3.23511	0	0	12.1	14.12	14.06	1.3	1.45	1.54
OsR498G1221934600.01	2.086	0.77	-1.43897	2.12E-21	2.78E-21	1.76	2.3	2.2	0.69	0.83	0.79
OsR498G1221936100.01	0.613	0.246	-1.6655	7.55E-08	1.43E-07	0.39	0.91	0.54	0.18	0.22	0.34
OsR498G1221936400.01	3.62	11.966	1.718498	4.53E-151	2.20E-151	3.51	3.42	3.93	12.06	12.27	11.57
OsR498G1221937000.01	0.61	0.093	-2.63267	3.69E-09	6.65E-09	0.52	0.55	0.76	0.14	0.07	0.07
OsR498G1221945700.01	10.333	3.063	-1.75785	2.32E-59	1.90E-59	10.66	10.05	10.29	3.74	3.13	2.32
OsR498G1221947400.01	0.73	0.106	-2.86713	1.23E-13	1.92E-13	0.9	0.6	0.69	0.08	0.16	0.08
OsR498G1221950700.01	49.3	15.746	-1.6508	0	0	48.6	48.38	50.92	15.37	16.97	14.9
OsR498G1221951400.01	72.283	21.73	-1.74407	0	0	72.25	71.61	72.99	23.89	22.08	19.22
OsR498G1221955200.01	0.24	0.016	-3.86713	8.60E-05	0.0001913	0.19	0.3	0.23	0	0.05	0
OsR498G1221957900.01	5.206	0.033	-7.15598	1.18E-163	5.43E-164	5.29	5.18	5.15	0.06	0	0.04
OsR498G1221958100.01	84.746	40.483	-1.15729	6.42E-199	2.63E-199	89.67	83.84	80.73	40.76	43.68	37.01
OsR498G1221958400.01	14.11	3.35	-2.08248	8.04E-257	2.77E-257	13.12	15.09	14.12	3.44	3.04	3.57
OsR498G1221960100.01	0.01	1.356	7.890509	2.67E-51	2.36E-51	0	0.03	0	1.6	1.27	1.2
OsR498G1221963400.01	17.253	1.103	-3.89678	5.19E-191	2.19E-191	18.78	15.57	17.41	1.51	0.93	0.87
OsR498G1221987100.01	37.68	166.68	2.142421	0	0	36.41	38.76	37.87	167.76	164.24	168.04
OsR498G1221987400.01	1.51	5.816	1.933109	2.17E-52	1.90E-52	1.32	1.72	1.49	6.08	5.51	5.86
OsR498G1221989900.01	12.19	3.99	-1.61005	2.01E-71	1.49E-71	12.64	11.85	12.08	3.88	3.93	4.16
OsR498G1221990700.01	13.48	47.493	1.810498	0	0	13.04	13.52	13.88	46.32	48.77	47.39
OsR498G1221999600.01	0.186	0	-4.66068	0.0001179	0.0002647	0.22	0.08	0.26	0	0	0
OsR498G1222011000.01	0.896	0.143	-2.81822	8.48E-11	1.44E-10	0.99	0.85	0.85	0.13	0.1	0.2
OsR498G1222022000.01	0.07	6.26	6.432077	1.64E-41	1.61E-41	0.11	0	0.1	5.96	7.55	5.27
OsR498G1222025700.01	0.973	6.813	2.794085	1.90E-102	1.16E-102	1.33	0.61	0.98	7.1	7.13	6.21
OsR498G1222029400.01	17.65	41.94	1.225238	2.45E-228	9.17E-229	18.28	17.81	16.86	41.68	41.51	42.63
OsR498G1222031800.01	2.816	0.583	-2.28217	7.98E-13	1.28E-12	2.71	2.76	2.98	0.67	0.3	0.78
OsR498G1222032500.01	0.216	2.166	3.32133	5.39E-54	4.65E-54	0.2	0.3	0.15	2.33	2.06	2.11
OsR498G1222033300.01	1.543	0.396	-1.97584	2.69E-11	4.51E-11	1.35	1.31	1.97	0.61	0.37	0.21
OsR498G1222033700.01	1.636	0.45	-1.80206	8.93E-23	1.14E-22	1.48	1.69	1.74	0.55	0.39	0.41
OsR498G1222034300.01	8.303	3.9	-1.11421	2.91E-29	3.33E-29	8.95	6.79	9.17	3.91	4.22	3.57
OsR498G1222046700.01	1.293	0.206	-3.11745	1.70E-59	1.39E-59	1.53	1.26	1.09	0.22	0.27	0.13
OsR498G1222052100.01	133.89	14.293	-3.39412	0	0	128.79	139.56	133.3	14.64	13.87	14.37
OsR498G1222067000.01	0.18	0.04	-2.15664	4.48E-08	8.42E-08	0.18	0.15	0.21	0.03	0.04	0.05
OsR498G1222071300.01	0.856	0.463	-1.13776	1.26E-08	2.32E-08	0.7	1	0.87	0.48	0.64	0.27
OsR498G1222074600.01	24.04	10.546	-1.25502	7.42E-125	4.03E-125	25.31	22.85	23.96	9.79	11.08	10.77
OsR498G1222079400.01	12.06	2.886	-2.01682	7.19E-186	3.09E-186	11.84	12.62	11.72	2.75	2.95	2.96
OsR498G1222081400.01	0.073	0.41	2.683616	2.26E-09	4.04E-09	0.16	0.03	0.03	0.36	0.65	0.22
OsR498G1222082600.01	10.17	20.62	1.017198	6.98E-46	6.53E-46	10.58	10.57	9.36	21.12	21.34	19.4
OsR498G1222085300.01	3.603	84.926	4.555357	0	0	3.57	3.79	3.45	87.12	82.8	84.86
OsR498G1222088000.01	1.14	0.35	-1.72577	1.83E-10	3.15E-10	0.74	0.99	1.69	0.24	0.46	0.35
OsR498G1222094400.01	2.373	1.06	-1.17139	6.66E-12	1.10E-11	2.19	2.45	2.48	0.77	0.95	1.46
OsR498G1222095000.01	2.276	0.813	-1.4838	2.02E-28	2.34E-28	2.38	2.02	2.43	0.93	0.86	0.65
OsR498G1222099100.01	1.563	0.603	-1.38474	2.36E-14	3.63E-14	1.71	1.42	1.56	0.62	0.74	0.45
OsR498G1222104500.01	6.143	0.686	-3.05158	2.51E-125	1.36E-125	6.71	5.57	6.15	0.83	0.66	0.57
OsR498G1222106300.01	5.23	1.976	-1.4187	1.98E-25	2.41E-25	5.32	4.61	5.76	1.81	2.27	1.85
OsR498G1222108900.01	0	0.573	7.268579	8.52E-19	1.18E-18	0	0	0	0.72	0.61	0.39
OsR498G1222111000.01	19.886	3.68	-2.43931	1.24E-186	5.30E-187	18.28	21.96	19.42	3.52	4.09	3.43
OsR498G1222111900.01	14.9	6.3	-1.24208	1.01E-69	7.62E-70	15.19	16.43	13.08	6.27	6.97	5.66
OsR498G1222114300.01	2.26	17.59	2.953983	0	0	2.2	2.12	2.46	18.27	17.26	17.24
OsR498G1222118000.01	87.983	202.7	1.209372	0	0	91.35	85.26	87.34	198.77	202.86	206.46
OsR498G1222120400.01	15.666	2.873	-2.43914	3.44E-193	1.44E-193	14.52	16.34	16.14	2.93	2.99	2.7
OsR498G1222122200.01	3.503	0.673	-2.39001	2.69E-48	2.45E-48	3.14	3.26	4.11	0.53	0.64	0.85
OsR498G1222125700.01	7.49	2.123	-1.73237	2.41E-113	1.39E-113	8.6	6.65	7.22	2.35	2.11	1.91
OsR498G1222126800.01	5.373	2.203	-1.29727	8.10E-10	1.43E-09	3.88	6.67	5.57	1.83	1.99	2.79
OsR498G1222131700.01	0.466	0	-5.28217	2.04E-06	4.14E-06	0.42	0.37	0.61	0	0	0

OsR498G1222132700.01	0.043	0.3	2.7402	7.88E-08	1.50E-07	0.02	0.09	0.02	0.27	0.33	0.3
OsR498G1222134700.01	0.48	0.143	-2.02437	1.26E-06	2.53E-06	0.52	0.42	0.5	0.21	0.11	0.11
OsR498G1222135100.01	2.206	0.906	-1.30441	1.81E-05	3.87E-05	2.8	1.69	2.13	0.72	1.06	0.94
OsR498G1222137200.01	33.89	105.61	1.636369	0	0	34.38	33.42	33.87	103.59	105.86	107.37
OsR498G1222139900.01	0.09	1.696	4.201648	6.50E-41	6.41E-41	0.11	0.03	0.13	1.81	1.45	1.83
OsR498G1222141000.01	2.56	1.2	-1.1458	2.02E-12	3.27E-12	2.15	2.75	2.78	1.09	1.24	1.27
OsR498G1222141300.01	5.78	0.563	-3.42513	1.46E-99	9.05E-100	5.22	6.49	5.63	0.4	1.09	0.2
OsR498G1222144900.01	255.73	676.4	1.401902	0	0	262.61	253.68	250.9	668.32	683.4	677.47
OsR498G1222146100.01	8.503	4.026	-1.09484	4.82E-27	5.70E-27	8.56	7.91	9.04	4.38	4.24	3.46
OsR498G1222149200.01	353.64	67.61	-2.39874	0	0	377.24	342.43	341.3	67.62	66.86	68.35
OsR498G1222150400.01	51.476	1.74	-4.89772	0	0	53.29	50.7	50.44	1.84	1.98	1.4
OsR498G1222150600.01	1.17	0	-5.81822	1.42E-08	2.62E-08	0.96	1.39	1.16	0	0	0
OsR498G1222151400.01	0.31	0.043	-2.80154	2.01E-08	3.72E-08	0.3	0.23	0.4	0.02	0.07	0.04
OsR498G1222152600.01	8.386	0.473	-4.16481	4.04E-64	3.18E-64	8.26	7.35	9.55	0.61	0.63	0.18
OsR498G1222153000.01	0.423	0.056	-2.89884	1.97E-05	4.22E-05	0.38	0.52	0.37	0.06	0	0.11
OsR498G1222159300.01	1.283	18.176	3.815785	0	0	1.1	1.21	1.54	19.56	17.81	17.16
OsR498G1222159600.01	3.446	0.776	-2.13876	3.59E-49	3.25E-49	3.74	3.5	3.1	0.84	0.79	0.7
OsR498G1222161300.01	2.553	12.39	2.38912	4.91E-303	1.49E-303	2.45	3.17	2.04	11.88	12.81	12.48
OsR498G1222162300.01	0.593	0.213	-1.49629	8.07E-10	1.42E-09	0.72	0.48	0.58	0.2	0.26	0.18
OsR498G1222164400.01	23.553	104.41	2.141465	0	0	24.35	23.89	22.42	101.4	104.24	107.58
OsR498G1222164700.01	1.286	0.433	-1.22703	1.07E-05	2.26E-05	1.34	1.35	1.17	0.61	0.37	0.32
OsR498G1222165100.01	0.476	5.876	3.639078	4.42E-62	3.54E-62	0.45	0.71	0.27	6.12	6.55	4.96
OsR498G1222167600.01	5.066	0.48	-3.41967	2.73E-32	2.99E-32	4.85	5.4	4.95	0.57	0.58	0.29
OsR498G1222168100.01	5.856	1.506	-1.99266	4.30E-11	7.26E-11	7.22	4.14	6.21	2.25	1.44	0.83
OsR498G1222168600.01	0.19	1.01	2.472719	5.23E-13	8.33E-13	0.22	0.15	0.2	0.95	1.03	1.05
OsR498G1222169600.01	18.54	170.89	3.192038	0	0	18.84	17.63	19.15	181.17	170	161.5
OsR498G1222170100.01	3.663	24.286	2.713259	0	0	3.64	3.87	3.48	25.8	23.3	23.76
OsR498G1222173400.01	0.826	6.726	2.98572	9.85E-90	6.45E-90	0.76	0.81	0.91	6.44	7.16	6.58
OsR498G1222174600.01	4.586	1.833	-1.32601	1.05E-27	1.23E-27	4.92	4.55	4.29	1.97	2.01	1.52
OsR498G1222175000.01	4.69	0.236	-4.31276	4.82E-94	3.06E-94	4.9	4.82	4.35	0.4	0.07	0.24
OsR498G1222176000.01	0.376	0.04	-3.25792	2.65E-12	4.31E-12	0.4	0.4	0.33	0.02	0.08	0.02
OsR498G1222180100.01	3.633	1.26	-1.53762	8.80E-23	1.12E-22	3.85	3.26	3.79	1.19	1.13	1.46
OsR498G1222180900.01	1.59	0.683	-1.22943	1.99E-11	3.33E-11	1.65	1.28	1.84	0.85	0.43	0.77
OsR498G1222181700.01	48.076	6.653	-2.90743	0	0	45.22	49.3	49.71	7.01	6.34	6.61
OsR498G1222182200.01	23.353	105.27	2.17039	0	0	22.24	24.14	23.68	104.57	104.33	106.92
OsR498G1222182500.01	5.696	31.246	2.454154	0	0	5.29	6.15	5.65	32.12	30.87	30.75
OsR498G1222185500.01	3.213	21.756	2.876421	9.94E-211	3.93E-211	2.79	3.64	3.21	22.57	22.31	20.39
OsR498G1222188300.01	0.086	2.9	5.053566	2.16E-100	1.33E-100	0.04	0.08	0.14	2.98	3.12	2.6
OsR498G1222188900.01	0.026	0.266	3.325162	4.65E-09	8.41E-09	0.02	0.02	0.04	0.32	0.25	0.23
OsR498G1222189700.01	2.043	0.673	-1.67645	2.97E-24	3.69E-24	1.85	2.23	2.05	0.56	0.83	0.63

Differentially expressed genes in YN0 vs YN30

Gene ID	YN0 FPKM	YN30 FPKM	log2(YN30/YN0)	Qvalue(YN0 vs YN30)	Pvalue(YN0 vs YN30)	YN0a FPKM	YN0b FPKM	YN0c FPKM	YN30a FPKM	YN30b FPKM	YN30c FPKM
OsR498G0022198300.01	0.126	0.333	1.3989078	6.80E-05	0.0001305	0.08	0.22	0.08	0.29	0.35	0.36
OsR498G0022198700.01	0.066	0.226	1.879906	3.59E-05	6.77E-05	0.04	0.04	0.12	0.27	0.34	0.07
OsR498G0022202400.01	0.04	0.103	1.3231319	0.0009738	0.0020278	0.07	0.04	0.01	0.08	0.16	0.07
OsR498G0022217100.01	0	0.11	4.4338416	0.0004331	0.0008746	0	0	0	0.15	0	0.18
OsR498G0022222500.01	0.173	0.543	1.6342966	0.0004544	0.000919	0	0.3	0.22	0	0.77	0.86
OsR498G0022222800.01	1.983	0.076	-4.72603	2.57E-33	2.45E-33	0.22	0.2	5.53	0.06	0.1	0.07
OsR498G0022225600.01	0.063	0.166	1.3960351	0.0007418	0.0015265	0.04	0.09	0.06	0.25	0.11	0.14
OsR498G0022230500.01	0.173	0.543	1.6342966	0.0004544	0.000919	0	0.3	0.22	0	0.77	0.86
OsR498G0022235600.01	38.436	15.3	-1.332345	4.87E-43	4.06E-43	35.99	37.65	41.67	17.56	12.09	16.25
OsR498G0022236200.01	14.11	5.846	-1.270373	9.25E-12	1.35E-11	11.43	17.39	13.51	6.82	6.65	4.07
OsR498G0022236400.01	182.426	19.023	-3.262788	0	0	189.34	187.49	170.45	18.85	18.06	20.16
OsR498G0022237000.01	44.873	18.526	-1.280526	1.15E-252	2.80E-253	46.75	42.45	45.42	16.69	15.34	23.55
OsR498G0022237700.01	79.186	16.596	-2.255633	0	0	68.79	97.33	71.44	15.8	17.46	16.53
OsR498G0022238000.01	8.64	3.53	-1.290192	1.22E-101	6.08E-102	8.25	9.52	8.15	3.79	3.66	3.14
OsR498G0022238900.01	44.873	18.526	-1.280526	1.15E-252	2.80E-253	46.75	42.45	45.42	16.69	15.34	23.55
OsR498G0022240800.01	155.836	46.18	-1.752293	0	0	153.45	159.36	154.7	46.64	43.85	48.05
OsR498G0100002300.01	5.746	30.166	2.4157893	9.96E-183	3.22E-183	5.68	5.79	5.77	29.33	31.87	29.3
OsR498G0100004300.01	0.13	0.403	1.2070816	5.31E-05	0.0001011	0.06	0.15	0.18	0.42	0.4	0.39
OsR498G0100006700.01	139.1	67.033	-1.054352	1.76E-281	3.91E-282	133.9	141.51	141.89	67.45	65.85	67.8
OsR498G0100011900.01	32.136	81.246	1.2690254	1.07E-227	2.87E-228	32.27	33.31	30.83	84.42	78.36	80.96
OsR498G0100012800.01	19.036	55.893	1.5541454	4.25E-240	1.09E-240	18.43	19.65	19.03	54.5	54.92	58.26
OsR498G0100013000.01	0.07	0.313	3.1672354	1.01E-09	1.58E-09	0.08	0.13	0	0.24	0.38	0.32
OsR498G0100013300.01	3.203	0.816	-1.975549	2.41E-35	2.24E-35	3.29	2.66	3.66	0.74	0.87	0.84
OsR498G0100014200.01	8.866	29.386	1.7421641	1.52E-157	5.51E-158	9.72	9.1	7.78	29.29	27.54	31.33
OsR498G0100014300.01	0.283	2.2	2.6994744	2.71E-43	2.25E-43	0.31	0.2	0.34	2.35	2.09	2.16
OsR498G0100016300.01	99.58	46.166	-1.10839	5.09E-267	1.18E-267	98.66	97.17	102.91	47.61	40.65	50.24
OsR498G0100017900.01	0.8	3.95	2.3104132	1.06E-65	6.97E-66	0.84	0.72	0.84	4	4.04	3.81
OsR498G0100021200.01	6.14	15.776	1.287634	3.72E-38	3.31E-38	5.97	5.47	6.98	15.77	15.23	16.33
OsR498G0100022900.01	22.33	45.223	1.0170652	3.16E-101	1.58E-101	23.49	21.56	21.94	44.95	42.67	48.05
OsR498G0100023400.01	2.873	29.84	3.375471	0	0	3.09	2.37	3.16	30.25	27.64	31.63
OsR498G0100023600.01	0.813	2.69	1.7209616	1.39E-14	1.88E-14	0.71	0.97	0.76	2.6	2.8	2.67
OsR498G0100023900.01	0.233	0.48	1.1647412	6.48E-06	1.18E-05	0.28	0.16	0.26	0.41	0.65	0.38
OsR498G0100024800.01	506.943	236.59	-1.111409	0	0	513.25	510.85	496.73	234.92	242.57	232.29
OsR498G0100029700.01	10.66	3.173	-1.741494	3.08E-61	2.11E-61	9.22	11.59	11.17	3.24	2.72	3.56
OsR498G0100030100.01	1.86	0.03	-6.141067	4.98E-51	3.79E-51	1.91	1.66	2.01	0.03	0.03	0.03
OsR498G0100031500.01	0.1	0.63	2.2223375	0.0006354	0.0012994	0.07	0.23	0	0.14	1.25	0.5
OsR498G0100033300.01	7.85	3.19	-1.403088	2.23E-74	1.36E-74	7.36	8.81	7.38	2.96	3.45	3.16
OsR498G0100033900.01	5.75	20.193	1.8184188	1.12E-159	4.03E-160	5.73	6.65	4.87	20.61	20.4	19.57
OsR498G0100034600.01	0.78	0.213	-1.847947	1.37E-15	1.82E-15	0.86	0.79	0.69	0.2	0.28	0.16
OsR498G0100035200.01	0.06	0.216	1.9130094	0.0006406	0.0013103	0	0.15	0.03	0.26	0.19	0.2
OsR498G0100035600.01	0.573	0.05	-3.569911	1.31E-08	2.13E-08	0.84	0.34	0.54	0.1	0	0.05
OsR498G0100035900.01	1.493	0.466	-1.713646	1.03E-06	1.80E-06	1.26	1.49	1.73	0.77	0.31	0.32
OsR498G0100036300.01	5.093	10.283	1.0189411	1.63E-51	1.23E-51	5.08	5.17	5.03	10.87	9.76	10.22
OsR498G0100044700.01	0.803	0.363	-1.163094	0.0002603	0.0005177	0.53	0.96	0.92	0.52	0.39	0.18
OsR498G0100045000.01	3.983	9.023	1.0908519	2.01E-33	1.92E-33	3.69	4.58	3.68	8.95	9.1	9.02
OsR498G0100045100.01	0.09	1.55	4.0851715	9.15E-42	7.77E-42	0.05	0.1	0.12	1.61	1.4	1.64
OsR498G0100045700.01	0.19	0.386	1.1191749	0.000828	0.0017124	0.11	0.29	0.17	0.44	0.33	0.39
OsR498G0100048100.01	0.086	0.393	2.2233804	3.62E-09	5.75E-09	0.08	0.14	0.04	0.34	0.38	0.46
OsR498G0100050400.01	0.053	0.393	2.8813006	0.000469	0.0009493	0	0.08	0.08	0.24	0.7	0.24
OsR498G0100050900.01	109.103	275.07	1.3340522	0	0	109.57	109.14	108.6	279.95	275.37	269.9
OsR498G0100053700.01	16.13	6.633	-1.229107	1.73E-43	1.43E-43	15.35	16.65	16.39	6.53	7.13	6.24
OsR498G0100054000.01	1.573	4.7	1.7565994	3.88E-31	3.84E-31	1.93	1.64	1.15	3.63	4.27	6.2
OsR498G0100056000.01	1.116	4.06	1.8103411	2.89E-68	1.85E-68	1.13	1.07	1.15	3.9	4.12	4.16
OsR498G0100056100.01	1.023	5.4	2.3887112	1.91E-57	1.36E-57	1.26	0.84	0.97	5.6	5.5	5.1
OsR498G0100056600.01	3.596	1.406	-1.351131	1.18E-26	1.26E-26	3.48	3.93	3.38	1.72	1.45	1.05
OsR498G0100057000.01	0.143	0.403	1.5716878	1.04E-07	1.75E-07	0.07	0.2	0.16	0.42	0.36	0.43
OsR498G0100057500.01	7.57	15.996	1.0757707	9.03E-58	6.40E-58	7.59	8.22	6.9	16.13	15.71	16.15
OsR498G0100060600.01	2.053	5.343	1.4331688	1.58E-36	1.44E-36	2.29	1.58	2.29	5.75	4.97	5.31
OsR498G0100062400.01	0.133	0.013	-3.195515	6.51E-05	0.0001248	0.09	0.12	0.19	0.02	0.02	0
OsR498G0100063300.01	2.633	5.933	1.1736189	2.96E-27	3.13E-27	2.35	2.5	3.05	5.32	6.29	6.19
OsR498G0100065000.01	2.333	27.953	3.6586439	0	0	2.39	2.49	2.12	26.21	30.43	27.22
OsR498G0100065900.01	5.12	14.066	1.4660934	5.98E-73	3.68E-73	5.4	5.07	4.89	14.26	14.45	13.49
OsR498G0100066400.01	10.08	29.43	1.5440203	0	0	10.43	10.01	9.8	30.99	30.2	27.1
OsR498G0100070800.01	0.126	0.913	2.8619352	1.36E-07	2.30E-07	0.08	0.15	0.15	0.73	0.66	1.35
OsR498G0100080200.01	8.93	0.306	-4.84085	3.44E-86	1.92E-86	9.45	8.63	8.71	0.07	0.42	0.43
OsR498G0100081000.01	0.99	5.47	2.4642152	1.92E-72	1.18E-72	1.11	1.04	0.82	5.11	4.81	6.49

OsR498G0100082800.01	0.666	3.86	2.6984365	3.17E-29	3.23E-29	0.79	0.51	0.7	4.23	3.84	3.51
OsR498G0100085200.01	1.633	3.68	1.1679475	1.05E-12	1.49E-12	1.67	1.82	1.41	3.39	4.04	3.61
OsR498G0100089700.01	2.076	5.46	1.3604684	2.33E-29	2.37E-29	1.72	2.25	2.26	5.53	5.81	5.04
OsR498G0100091500.01	2.003	34.166	4.0980611	0	0	2.28	1.72	2.01	34.01	33.12	35.37
OsR498G0100091900.01	0.45	2.513	2.5104629	1.47E-33	1.40E-33	0.57	0.44	0.34	2.37	2.74	2.43
OsR498G0100093200.01	0	1.646	8.0404991	1.72E-29	1.74E-29	0	0	0	1.17	1.82	1.95
OsR498G0100094400.01	0.1	1.64	4.2075311	5.94E-62	4.03E-62	0.14	0.08	0.08	1.59	1.67	1.66
OsR498G0100094500.01	1.073	61.14	5.8956229	0	0	1.02	1.11	1.09	61.36	60.93	61.13
OsR498G0100094800.01	2.183	5.116	1.2450575	1.09E-17	1.37E-17	1.86	2.54	2.15	5.3	4.82	5.23
OsR498G0100095000.01	8.846	25.273	1.5118379	1.70E-229	4.53E-230	8.3	9.12	9.12	25.36	24.83	25.63
OsR498G0100095800.01	1.263	3.273	1.3591988	5.40E-21	6.36E-21	1.05	1.1	1.64	3.11	3.23	3.48
OsR498G0100102600.01	5.89	22.673	1.9609439	3.67E-238	9.51E-239	6.05	5.54	6.08	22.92	22.44	22.66
OsR498G0100103600.01	16.363	4.08	-2.00396	9.77E-72	6.06E-72	15.28	15.97	17.84	4.2	4.18	3.86
OsR498G0100104900.01	0.096	0.013	-2.994945	0.0001144	0.0002227	0.13	0.11	0.05	0	0.04	0
OsR498G0100106000.01	0.573	0.17	-1.762556	0.0002331	0.000462	0.52	0.51	0.69	0.06	0.45	0
OsR498G0100110900.01	4.69	12.09	1.3463787	1.45E-22	1.65E-22	4.39	4.83	4.85	11.33	12.92	12.02
OsR498G0100111600.01	0.32	3.073	3.2474285	2.11E-22	2.42E-22	0.16	0.4	0.4	3.48	3.39	2.35
OsR498G0100114400.01	10.9	36.98	1.4654468	8.97E-157	3.27E-157	10.11	10.96	11.63	36.55	37.25	37.14
OsR498G0100117900.01	0.066	0.306	2.2598122	1.83E-06	3.24E-06	0.1	0.05	0.05	0.42	0.09	0.41
OsR498G0100119500.01	0.246	29.283	6.8845337	0	0	0.2	0.34	0.2	28.18	30.55	29.12
OsR498G0100120800.01	5.143	12.843	1.3199678	1.79E-33	1.70E-33	4.84	5.5	5.09	12.5	13.48	12.55
OsR498G0100122800.01	3.51	17.503	2.3188901	1.91E-101	9.51E-102	4.88	3.45	2.2	14.57	19.88	18.06
OsR498G0100122900.01	96.183	641.68	2.7358469	0	0	91.09	100.19	97.27	638.55	598.7	687.79
OsR498G0100125200.01	23.69	53.85	1.2138474	7.35E-297	1.57E-297	23.73	23.13	24.21	54.54	55.5	51.51
OsR498G0100126200.01	0.043	1.233	4.7972501	1.07E-10	1.62E-10	0	0	0.13	0.97	1.43	1.3
OsR498G0100127300.01	1.463	0.31	-1.993799	2.24E-07	3.82E-07	2.75	1.25	0.39	0.4	0	0.53
OsR498G0100127900.01	0.253	0.666	1.5786711	1.91E-07	3.24E-07	0.31	0.24	0.21	0.63	0.86	0.51
OsR498G0100132900.01	3.056	29.02	3.2174032	0	0	3.13	3.07	2.97	29.91	29.44	27.71
OsR498G0100135100.01	11.15	25.7	1.1772732	8.71E-86	4.87E-86	10.84	11.01	11.6	25.51	25.16	26.43
OsR498G0100139600.01	0.103	0.73	2.97441	1.18E-05	2.17E-05	0.11	0.2	0	0.91	0.52	0.76
OsR498G0100141500.01	0.086	0.276	1.7244317	7.64E-05	0.0001469	0.13	0.06	0.07	0.38	0.23	0.22
OsR498G0100141700.01	3.976	10.2	1.3575704	4.39E-24	4.88E-24	3.77	4.08	4.08	9.61	10.22	10.77
OsR498G0100149000.01	62.956	139.52	1.1486274	0	0	60.52	62.79	65.56	138.44	141.73	138.39
OsR498G0100150400.01	7.203	19.55	1.4306229	5.28E-211	1.51E-211	6.52	7.7	7.39	19.61	20.24	18.8
OsR498G0100152200.01	18.793	55.816	1.6421479	0	0	17.31	19.53	19.54	54.42	56.37	56.66
OsR498G0100152900.01	3.576	25.443	2.7391125	0	0	3.49	3.59	3.65	24.74	24.93	26.66
OsR498G0100162200.01	0.48	41.776	6.4338416	0	0	0.21	0.46	0.77	41.86	41.24	42.23
OsR498G0100171500.01	1.386	0.303	-2.185865	4.39E-16	5.74E-16	1.21	1.44	1.51	0.44	0.23	0.24
OsR498G0100176800.01	0.08	0.223	1.2444991	0.0008329	0.0017234	0.07	0.13	0.04	0.29	0.13	0.25
OsR498G0100177000.01	0.026	0.216	3.1173679	7.33E-11	1.10E-10	0.03	0.04	0.01	0.22	0.23	0.2
OsR498G0100178400.01	0.57	1.09	1.1602765	2.73E-10	4.18E-10	0.88	0.4	0.43	1.19	0.95	1.13
OsR498G0100184500.01	81.643	24.29	-1.706896	0	0	82.59	80.83	81.51	24.16	25.71	23
OsR498G0100184700.01	0.266	1.256	2.2337966	1.15E-20	1.36E-20	0.29	0.17	0.34	1.42	1.25	1.1
OsR498G0100188900.01	0.286	0.766	1.4310484	3.93E-10	6.04E-10	0.22	0.33	0.31	0.71	0.99	0.6
OsR498G0100191900.01	5.826	13.4	1.2063796	3.02E-74	1.84E-74	5.94	5.91	5.63	12.67	14.33	13.2
OsR498G0100193000.01	0.766	0.326	-1.695441	1.20E-10	1.82E-10	0.79	0.96	0.55	0.26	0.41	0.31
OsR498G0100194700.01	2.413	0.336	-2.893487	1.75E-25	1.90E-25	2.5	2.78	1.96	0.4	0.31	0.3
OsR498G0100195800.01	0.026	0.293	3.2963381	2.59E-05	4.84E-05	0.04	0	0.04	0.3	0.09	0.49
OsR498G0100196800.01	1.006	5.64	2.4874795	2.22E-20	2.65E-20	0.63	1.26	1.13	7.13	5.39	4.4
OsR498G0100197000.01	0.846	5.216	2.6289134	2.61E-26	2.80E-26	1.04	0.47	1.03	5.77	5.56	4.32
OsR498G0100198300.01	1.276	44.826	5.1322621	0	0	1.25	0.86	1.72	47.42	41.54	45.52
OsR498G0100203600.01	1.286	6.153	2.2582029	6.87E-29	7.04E-29	1.26	1.26	1.34	5.74	7.18	5.54
OsR498G0100203700.01	11.333	46.236	2.0278012	3.23E-300	6.84E-301	11.51	11.55	10.94	49.2	44.84	44.67
OsR498G0100204500.01	24.42	74.696	1.6328112	0	0	24.11	24.69	24.46	71.68	72.88	79.53
OsR498G0100207000.01	12.073	24.28	1.0064298	2.93E-74	1.78E-74	11.52	11.85	12.85	22.24	25.04	25.56
OsR498G0100208600.01	1.886	5.826	1.620218	1.33E-80	7.76E-81	1.72	2.18	1.76	5.76	6.03	5.69
OsR498G0100209200.01	37.92	97.67	1.3756374	0	0	37.1	38.77	37.89	98.77	98.69	95.55
OsR498G0100209800.01	3.696	10.33	1.4706435	6.97E-74	4.26E-74	3.87	3.08	4.14	9.97	10.21	10.81
OsR498G0100209900.01	0.546	0	-4.347518	0.0007014	0.0014405	0.33	0.82	0.49	0	0	0
OsR498G0100210300.01	0.373	1.846	2.310013	5.54E-15	7.42E-15	0.37	0.43	0.32	1.77	2.01	1.76
OsR498G0100211200.01	2.593	8.083	1.6408001	1.94E-79	1.14E-79	3	2.57	2.21	7.75	8.47	8.03
OsR498G0100212300.01	0.44	0.11	-1.984948	4.25E-09	6.77E-09	0.38	0.56	0.38	0.11	0.05	0.17
OsR498G0100212900.01	0.183	0.46	1.3154469	4.64E-06	8.38E-06	0.2	0.18	0.17	0.34	0.45	0.59
OsR498G0100213800.01	0.246	17.766	6.3402463	0	0	0.21	0.18	0.35	18.09	18.11	17.1
OsR498G0100215400.01	1.696	5.436	1.6785856	4.09E-49	3.18E-49	1.85	1.49	1.75	5.59	5.28	5.44
OsR498G0100215600.01	269.766	130.12	-1.060242	0	0	266.65	276.81	265.84	129.49	133.9	126.97
OsR498G0100215700.01	10.363	22.116	1.0970163	4.11E-78	2.43E-78	11.21	9.67	10.21	21.55	21.99	22.81
OsR498G0100216100.01	0.973	4.776	2.3215595	2.88E-92	1.53E-92	0.78	0.9	1.24	5.37	4.48	4.48
OsR498G0100218400.01	2.433	5.44	1.0986128	2.35E-31	2.31E-31	2.31	2.64	2.35	5.11	5.76	5.45

OsR498G0100219300.01	0.88	2.136	1.3165159	4.72E-12	6.84E-12	0.85	0.94	0.85	2.05	2.33	2.03
OsR498G0100219700.01	0.073	0.45	2.5593725	4.55E-05	8.61E-05	0	0.22	0	0.4	0.49	0.46
OsR498G0100221200.01	1.706	5.29	1.621795	1.75E-49	1.35E-49	1.55	1.61	1.96	4.94	5.68	5.25
OsR498G0100221500.01	16.876	28.303	1.256414	2.34E-188	7.36E-189	17.58	16.18	16.87	27.64	29.17	28.1
OsR498G0100221800.01	5.203	21.056	2.0038686	2.26E-244	5.73E-245	5.01	5.59	5.01	21.26	21.48	20.43
OsR498G0100222500.01	1.16	0.243	-2.347518	4.07E-12	5.89E-12	1.31	0.92	1.25	0.36	0.18	0.19
OsR498G0100225000.01	94.656	228.31	1.2663684	0	0	94.3	96.45	93.22	226.31	234.71	223.92
OsR498G0100225200.01	31.5	99.44	1.6674575	0	0	31.46	32.31	30.73	97.9	96.57	103.85
OsR498G0100226300.01	1.163	5.526	2.2566738	5.18E-71	3.23E-71	1.01	1.54	0.94	5.07	5.39	6.12
OsR498G0100233200.01	0.606	2.46	2.0170543	3.38E-34	3.18E-34	0.56	0.7	0.56	2.51	2.36	2.51
OsR498G0100235000.01	1.723	0.826	-1.07712	2.07E-11	3.06E-11	1.5	1.48	2.19	0.78	0.89	0.81
OsR498G0100238400.01	3.796	0.763	-2.310267	1.44E-64	9.56E-65	4.24	3.53	3.62	0.89	0.6	0.8
OsR498G0100238600.01	0.74	1.676	1.0225042	2.20E-06	3.91E-06	0.91	0.74	0.57	1.54	1.87	1.62
OsR498G0100242100.01	16.163	68.42	2.0847182	0	0	16.67	15.5	16.32	65.98	68.38	70.9
OsR498G0100242400.01	2.92	6.086	1.0707887	3.10E-39	2.72E-39	2.63	3.04	3.09	5.86	6.2	6.2
OsR498G0100243800.01	26.443	80.763	1.613288	2.08E-274	4.72E-275	27.58	25.37	26.38	83.07	77.44	81.78
OsR498G0100246500.01	6.106	26.706	2.1306664	0	0	6.06	6.01	6.25	26.49	27.38	26.25
OsR498G0100246800.01	2.143	5.59	1.3817858	5.15E-21	6.06E-21	2.52	1.63	2.28	4.46	5.87	6.44
OsR498G0100251400.01	0.26	0.913	1.9504467	6.58E-09	1.05E-08	0.46	0.24	0.08	1.11	0.65	0.98
OsR498G0100252000.01	53.733	2.003	-4.741774	0	0	55.63	53.12	52.45	2.27	2.05	1.69
OsR498G0100253200.01	0.066	1.4	4.4073694	1.98E-25	2.15E-25	0.08	0.04	0.08	1.54	1.35	1.31
OsR498G0100253300.01	0.793	5.706	2.8589167	9.17E-89	5.01E-89	0.7	1.01	0.67	5.42	6.1	5.6
OsR498G0100254100.01	0.823	2.683	1.7061303	1.60E-25	1.74E-25	0.71	1.08	0.68	2.54	2.86	2.65
OsR498G0100258600.01	0.29	4.506	3.9643563	1.05E-60	7.24E-61	0.39	0.14	0.34	4.73	3.83	4.96
OsR498G0100263800.01	7.943	2	-1.990337	5.31E-44	4.37E-44	7.29	8	8.54	1.84	2.3	1.86
OsR498G0100264200.01	5.623	0.63	-3.159173	3.44E-105	1.67E-105	5.86	5.33	5.68	0.84	0.47	0.58
OsR498G0100264600.01	39.04	18.776	-1.104724	2.59E-96	1.33E-96	39.17	37.47	40.48	17.75	17.34	21.24
OsR498G0100267100.01	0.85	6.133	2.7163151	1.28E-248	3.17E-249	0.88	0.82	0.85	6.48	6.7	5.22
OsR498G0100267300.01	0.503	4.046	3.8901238	1.50E-29	1.52E-29	0.23	1.28	0	2.97	3.49	5.68
OsR498G0100268100.01	11.803	24.096	1.0272618	3.01E-76	1.80E-76	11.68	12.25	11.48	24.71	23.66	23.92
OsR498G0100269800.01	23.79	68.82	1.5436247	5.12E-291	1.11E-291	24.15	24.65	22.57	66.61	70.32	69.53
OsR498G0100270600.01	0.46	1.03	1.449748	2.67E-09	4.22E-09	0.5	0.39	0.49	1.21	0.95	0.93
OsR498G0100274000.01	1.296	4.036	1.6490097	7.76E-35	7.24E-35	1.4	1.06	1.43	4.26	4.39	3.46
OsR498G0100276000.01	0.966	2.256	1.2307497	3.79E-06	6.80E-06	0.72	1.31	0.87	2.49	2.05	2.23
OsR498G0100277300.01	0.53	1.07	1.0197339	2.15E-05	4.01E-05	0.58	0.5	0.51	1.22	1.21	0.78
OsR498G0100278000.01	0.473	0.026	-4.113053	2.28E-05	4.25E-05	0.42	0.42	0.58	0.08	0	0
OsR498G0100278600.01	1.976	4.726	1.2841376	1.28E-27	1.34E-27	2.13	1.95	1.85	4.39	4.87	4.92
OsR498G0100279000.01	39.92	82.976	1.0543868	0	0	40.26	39.23	40.27	83.22	84.9	80.81
OsR498G0100282100.01	0.786	4.903	2.6684576	4.45E-84	2.52E-84	0.93	0.77	0.66	4.86	4.76	5.09
OsR498G0100285700.01	0.766	4.553	2.475212	6.59E-40	5.74E-40	0.77	0.48	1.05	4.44	4	5.22
OsR498G0100286200.01	0.94	3.096	1.7154917	2.32E-31	2.29E-31	0.79	1.01	1.02	2.89	3	3.4
OsR498G0100286900.01	0.03	0.49	4.103693	3.75E-09	5.96E-09	0.09	0	0	0.75	0.42	0.3
OsR498G0100287200.01	22.046	48.45	1.1347132	0	0	21.92	22.27	21.95	50.69	49.11	45.55
OsR498G0100287600.01	0	0.3	4.7745333	7.47E-05	0.0001437	0	0	0	0.45	0.15	0.3
OsR498G0100287700.01	1.106	10.853	3.2868008	1.13E-161	4.02E-162	1.41	0.99	0.92	11.18	9.83	11.55
OsR498G0100288900.01	0.01	0.16	3.97441	5.14E-05	9.78E-05	0	0.03	0	0.09	0.18	0.21
OsR498G0100293900.01	4.976	16.776	1.9616346	1.70E-160	6.11E-161	4.18	4.69	6.06	16.2	16.08	18.05
OsR498G0100294100.01	0.3	0.953	1.6748497	5.73E-06	1.04E-05	0.51	0.22	0.17	1.37	0.76	0.73
OsR498G0100297400.01	0	0.173	4.8813006	3.98E-05	7.52E-05	0	0	0	0.25	0.17	0.1
OsR498G0100300100.01	0.55	1.55	1.6647255	1.71E-07	2.90E-07	0.64	0.38	0.63	1.87	1.08	1.7
OsR498G0100303400.01	8.44	16.87	1.0635277	4.81E-62	3.26E-62	9.01	7.55	8.76	17.91	16.14	16.56
OsR498G0100305000.01	3.43	9.013	1.3932562	1.09E-56	7.78E-57	3.69	3.21	3.39	8.67	9.08	9.29
OsR498G0100305100.01	22.183	45.463	1.0298833	1.44E-101	7.17E-102	21.29	23.37	21.89	46.33	43.56	46.5
OsR498G0100309400.01	1.143	2.893	1.2991367	3.29E-16	4.29E-16	1.57	1	0.86	3.17	2.86	2.65
OsR498G0100309500.01	4.706	29.546	2.6423339	2.82E-198	8.50E-199	4.84	4.47	4.81	29.74	30.99	27.91
OsR498G0100310200.01	16.276	1.896	-2.970125	6.24E-150	2.34E-150	17.23	14.53	17.07	1.79	1.47	2.43
OsR498G0100313800.01	0.783	0.293	-1.260055	0.0007821	0.0016115	0.84	0.93	0.58	0.26	0.2	0.42
OsR498G0100319600.01	0.01	0.12	3.5593725	0.0005724	0.0011661	0	0	0.03	0.15	0.06	0.15
OsR498G0100325200.01	1.826	4.14	1.1699608	2.48E-25	2.70E-25	2.05	1.77	1.66	4.54	3.86	4.02
OsR498G0100328800.01	2.82	16.683	2.565019	3.94E-288	8.59E-289	3.13	2.37	2.96	15.8	16.62	17.63
OsR498G0100331100.01	1.64	5.913	1.8506746	7.09E-27	7.53E-27	2.03	1.41	1.48	5.59	6.55	5.6
OsR498G0100335400.01	0.176	0.63	1.832391	0.0002304	0.0004563	0.27	0.13	0.13	0.65	0.64	0.6
OsR498G0100335600.01	1.19	0.156	-2.951589	2.25E-14	3.06E-14	1.74	0.61	1.22	0.27	0.05	0.15
OsR498G0100336900.01	9.173	4.333	-1.076422	5.92E-65	3.91E-65	8.92	9.38	9.22	4.24	4.59	4.17
OsR498G0100339300.01	0	1.22	7.7688258	2.45E-25	2.66E-25	0	0	0	1.02	1.25	1.39
OsR498G0100340100.01	1.166	2.486	1.0939171	5.95E-12	8.65E-12	1.28	1.2	1.02	2.63	2.19	2.64
OsR498G0100341300.01	2.38	13.72	2.5656862	5.44E-194	1.67E-194	2.18	2.19	2.77	14.17	13.48	13.51
OsR498G0100343500.01	8.396	19.973	1.2302128	7.00E-63	4.72E-63	8.96	7.85	8.38	19.68	19.9	20.34
OsR498G0100345000.01	0.493	1.953	1.989207	3.87E-42	3.27E-42	0.43	0.54	0.51	1.82	2.19	1.85

OsR498G0100347500.01	70.063	27.763	-1.339321	4.20E-210	1.21E-210	69.28	70.16	70.75	26.19	25.56	31.54
OsR498G0100348800.01	82.766	228.88	1.4610637	0	0	83.86	80.69	83.75	228.68	229.96	228.01
OsR498G0100349600.01	3.103	12.513	2.0052474	3.10E-36	2.84E-36	2.54	3.1	3.67	9.89	11.2	16.45
OsR498G0100356900.01	0.12	0.866	2.8224069	1.37E-18	1.69E-18	0.02	0.19	0.15	0.69	0.83	1.08
OsR498G0100364900.01	0.013	0.273	4.3667274	2.59E-06	4.61E-06	0	0	0.04	0.27	0.23	0.32
OsR498G0100368300.01	8.19	0.233	-5.139332	9.74E-67	6.32E-67	8.55	9.2	6.82	0.26	0.17	0.27
OsR498G0100370600.01	6.443	16.893	1.3719745	1.19E-122	5.18E-123	6.95	6.16	6.22	17.64	16.24	16.8
OsR498G0100373100.01	3.066	12.176	1.9804421	2.84E-75	1.71E-75	3.94	2.65	2.61	11.77	12.23	12.53
OsR498G0100373300.01	3.373	5.46	1.0745928	7.60E-39	6.72E-39	3.19	3.33	3.6	4.59	5.49	6.3
OsR498G0100374100.01	27.363	1.88	-3.868182	2.30E-232	6.06E-233	26.27	28.1	27.72	2.73	1.73	1.18
OsR498G0100374300.01	6.763	26.81	1.9899588	5.34E-136	2.16E-136	6.83	6.91	6.55	27.1	26.8	26.53
OsR498G0100376800.01	1.67	4.023	1.2654587	6.05E-13	8.54E-13	1.72	1.76	1.53	3.97	3.32	4.78
OsR498G0100377600.01	70.61	23.98	-1.558843	0	0	67.39	72.36	72.08	22.81	24.6	24.53
OsR498G0100380200.01	0.883	0.213	-2.072569	3.67E-14	5.02E-14	0.64	1	1.01	0	0.36	0.28
OsR498G0100384500.01	36.863	17.996	-1.063374	0	0	37.06	34.64	38.89	18.52	17.26	18.21
OsR498G0100389000.01	0.053	2.036	5.0333036	1.15E-47	9.05E-48	0.05	0.07	0.04	2.37	1.96	1.78
OsR498G0100389600.01	4.503	9.673	1.1004178	6.39E-49	4.97E-49	3.86	4.64	5.01	9.31	9.24	10.47
OsR498G0100390700.01	0.026	0.71	4.7817649	4.23E-08	6.99E-08	0	0	0.08	0.75	0.45	0.93
OsR498G0100392000.01	39.94	80.22	1.0029086	4.44E-152	1.65E-152	39.17	40.2	40.45	77.26	77.31	86.09
OsR498G0100392200.01	47.09	19.493	-1.273518	7.44E-148	2.82E-148	48.27	48.33	44.67	18.08	19.86	20.54
OsR498G0100393600.01	12.276	5.803	-1.196708	3.19E-92	1.70E-92	12.14	12.6	12.09	5.33	5.96	6.12
OsR498G0100402300.01	5.91	2.08	-1.516576	1.52E-46	1.22E-46	5.6	6.13	6	2.35	2.07	1.82
OsR498G0100405900.01	0.016	0.456	4.6297618	5.94E-30	5.98E-30	0.02	0.02	0.01	0.51	0.32	0.54
OsR498G0100407600.01	0.2	0.013	-4.72603	1.01E-07	1.69E-07	0.17	0.22	0.21	0	0.04	0
OsR498G0100410000.01	6.193	31.893	2.3638216	1.28E-238	3.30E-239	6.47	6.36	5.75	31.2	32.9	31.58
OsR498G0100410700.01	0.333	6.013	4.1629164	6.18E-83	3.53E-83	0.24	0.33	0.43	5.28	6.93	5.83
OsR498G0100411100.01	0.676	1.723	1.3781321	1.53E-09	2.39E-09	0.67	0.62	0.74	1.94	1.53	1.7
OsR498G0100411900.01	37.256	98.246	1.3978627	0	0	35.9	36.68	39.19	95.87	96.81	102.06
OsR498G0100412800.01	0.636	1.506	1.2428988	0.0001883	0.0003711	0.61	0.78	0.52	1.78	1.44	1.3
OsR498G0100420900.01	1.426	0.393	-1.84662	7.34E-12	1.07E-11	1.89	1.31	1.08	0.43	0.47	0.28
OsR498G0100424900.01	1.67	14.243	3.1295075	2.65E-250	6.54E-251	1.44	1.4	2.17	14.5	13.72	14.51
OsR498G0100426100.01	0.31	3.676	3.5424986	8.40E-99	4.26E-99	0.37	0.3	0.26	3.68	3.81	3.54
OsR498G0100431000.01	194.836	492.38	1.1581893	0	0	195.24	199.02	190.25	493.32	498.99	484.82
OsR498G0100432300.01	10.256	29.65	1.4609701	5.26E-82	3.03E-82	10.86	9.34	10.57	30.75	27.58	30.62
OsR498G0100437400.01	0.356	0.12	-1.610553	1.75E-05	3.24E-05	0.35	0.41	0.31	0.19	0.13	0.04
OsR498G0100439900.01	66.003	207.09	1.6520016	0	0	70.69	63.7	63.62	202.91	208.22	210.13
OsR498G0100440200.01	0.526	1.84	1.7457856	2.46E-10	3.76E-10	0.58	0.42	0.58	1.7	2.09	1.73
OsR498G0100441000.01	1.303	3.403	1.1740493	4.02E-15	5.37E-15	0.87	1.39	1.65	3.2	3.4	3.61
OsR498G0100443200.01	0.903	0.22	-1.973123	2.32E-07	3.95E-07	0.8	1.01	0.9	0.28	0.21	0.17
OsR498G0100443500.01	0.1	0.69	2.8423064	3.17E-13	4.44E-13	0.02	0.07	0.21	0.74	0.69	0.64
OsR498G0100444800.01	15.293	4.59	-1.755408	1.46E-102	7.20E-103	15.05	14.46	16.37	4.58	4.43	4.76
OsR498G0100446400.01	0.053	0.343	2.7817649	0.0008272	0.0017105	0.08	0	0.08	0.37	0.43	0.23
OsR498G0100446600.01	1.31	9.516	2.8079073	4.34E-139	1.73E-139	1.42	1.12	1.39	9.36	9	10.19
OsR498G0100447600.01	8.503	2.073	-2.058313	8.58E-192	2.66E-192	8.77	8.25	8.49	1.92	2.4	1.9
OsR498G0100452900.01	1.776	4.446	1.3170966	8.98E-12	1.31E-11	1.86	1.85	1.62	3.55	4.29	5.5
OsR498G0100453200.01	0.04	0.363	3.144335	8.30E-05	0.00016	0.06	0	0.06	0.48	0.42	0.19
OsR498G0100454000.01	11.07	31.086	1.4842296	5.06E-105	2.46E-105	10.18	10.99	12.04	31.31	29.97	31.98
OsR498G0100456800.01	1.02	28.303	4.7205112	0	0	1.11	1.1	0.85	27.96	26.7	30.25
OsR498G0100462300.01	1.406	2.883	1.0364709	3.59E-09	5.70E-09	1.49	1.29	1.44	3.21	2.93	2.51
OsR498G0100462700.01	2.976	6.91	1.2204174	4.92E-35	4.58E-35	3.44	2.54	2.95	6.81	7.25	6.67
OsR498G0100463300.01	8.586	3.946	-1.126638	2.35E-57	1.67E-57	8.94	8.14	8.68	4.1	3.43	4.31
OsR498G0100466700.01	55.51	135.97	1.2953018	0	0	56.56	55.02	54.95	137.7	135.25	134.95
OsR498G0100467900.01	13.28	52.453	1.9952934	0	0	13.12	13.93	12.79	51.92	52.56	52.88
OsR498G0100468100.01	56.446	133.65	1.2413692	0	0	57.73	58.56	53.05	137.53	131.34	132.07
OsR498G0100469500.01	13.576	32.99	1.2801767	1.09E-158	3.95E-159	14.53	12.74	13.46	32.67	32.77	33.53
OsR498G0100471400.01	0.043	0.423	3.6039609	5.96E-09	9.54E-09	0	0.08	0.05	0.57	0.41	0.29
OsR498G0100473000.01	4.286	15.056	1.8080703	2.62E-139	1.04E-139	4.43	4.74	3.69	14.39	15.3	15.48
OsR498G0100475200.01	1.72	0.38	-2.145884	2.33E-13	3.25E-13	2.06	1.22	1.88	0.25	0.54	0.35
OsR498G0100476300.01	4.243	1.393	-1.321046	5.64E-16	7.39E-16	5.78	3.24	3.71	1.54	1.26	1.38
OsR498G0100477800.01	6.723	20.093	1.5818035	9.27E-162	3.30E-162	6.68	6.85	6.64	21.1	19.21	19.97
OsR498G0100479700.01	25.33	11.843	-1.096542	9.13E-113	4.23E-113	25.13	26.09	24.77	11.37	11.04	13.12
OsR498G0100481300.01	0.12	0.526	2.0898872	0.0001502	0.0002945	0.12	0.12	0.12	0.54	0.36	0.68
OsR498G0100481600.01	2.536	13.156	2.3729593	3.09E-61	2.11E-61	3.1	2.25	2.26	13.46	11.09	14.92
OsR498G0100482300.01	5.413	0.726	-2.921253	4.20E-54	3.08E-54	6.17	5.15	4.92	0.54	0.89	0.75
OsR498G0100487200.01	29.89	61.326	1.0338142	2.39E-147	9.08E-148	29.39	29.91	30.37	63.1	62.67	58.21
OsR498G0100490700.01	1.09	0.49	-1.159891	5.18E-06	9.38E-06	0.95	0.83	1.49	0.39	0.53	0.55
OsR498G0100493800.01	4.69	11.526	1.2951647	6.41E-70	4.04E-70	4.59	4.65	4.83	11.43	12.3	10.85
OsR498G0100494600.01	0.81	2.16	1.4120962	6.17E-29	6.32E-29	0.74	0.75	0.94	2.16	2.17	2.15
OsR498G0100500300.01	0.263	1.043	2.0618728	5.76E-11	8.63E-11	0.34	0.18	0.27	1.34	1.11	0.68

OsR498G0100505800.01	0.016	0.24	3.97441	5.14E-05	9.78E-05	0.05	0	0	0.36	0.13	0.23
OsR498G0100506800.01	59.446	20.473	-1.527244	5.04E-166	1.76E-166	56.78	63.65	57.91	19.57	19.56	22.29
OsR498G0100507700.01	46.233	1.06	-5.388995	0	0	46.64	45.89	46.17	1.03	0.92	1.23
OsR498G0100508800.01	1.113	3.966	1.857053	6.84E-13	9.67E-13	0.95	1.43	0.96	3.33	3.87	4.7
OsR498G0100510600.01	244.826	107.29	-1.193399	0	0	248.49	244.6	241.39	106.07	110.02	105.77
OsR498G0100519100.01	0.386	0.956	1.2963381	9.36E-07	1.64E-06	0.55	0.16	0.45	0.57	1.1	1.2
OsR498G0100519400.01	1.75	6.143	1.8017333	3.35E-54	2.46E-54	2.1	1.61	1.54	7.16	6.06	5.21
OsR498G0100521900.01	8	17.27	1.1149682	4.66E-39	4.11E-39	8.4	7.67	7.93	18.2	17.86	15.75
OsR498G0100523100.01	2.783	0.306	-3.195515	6.51E-05	0.0001248	2.3	2.83	3.22	0	0	0.92
OsR498G0100524200.01	0.933	3.106	1.7352223	1.07E-22	1.22E-22	0.94	0.73	1.13	3.18	2.6	3.54
OsR498G0100524900.01	1.37	2.996	1.142477	2.54E-28	2.63E-28	1.55	1.16	1.4	3.03	3.28	2.68
OsR498G0100525200.01	10.526	30.266	1.4541491	3.77E-219	1.04E-219	10.37	10.48	10.73	30.09	31.2	29.51
OsR498G0100529000.01	0.226	3.393	4.6297618	4.15E-114	1.91E-114	0.31	0.18	0.19	3.98	4.12	2.08
OsR498G0100539500.01	256.26	4161.3	4.0188242	0	0	275.62	240.6	252.56	4174.1	4010.9	4299
OsR498G0100540000.01	2.58	8.33	1.7346907	2.60E-53	1.92E-53	2.47	2.26	3.01	7.97	8.12	8.9
OsR498G0100540400.01	0	0.393	4.2963381	0.0007942	0.0016388	0	0	0	0.24	0.7	0.24
OsR498G0100541900.01	5.703	48.27	3.0821238	0	0	6.17	5.52	5.42	45.73	47.46	51.62
OsR498G0100549900.01	18.54	43.74	1.2505133	6.28E-282	1.40E-282	18.84	19.22	17.56	42.14	46.68	42.4
OsR498G0100556300.01	2.206	20.13	3.1861551	3.18E-166	1.11E-166	1.96	2.46	2.2	18.5	18.93	22.96
OsR498G0100557300.01	0.643	2.43	1.9184536	3.43E-19	4.18E-19	0.76	0.54	0.63	2.44	2.63	2.22
OsR498G0100559700.01	119.34	263.75	1.1450969	0	0	116.24	118.63	123.15	260.72	261.4	269.13
OsR498G0100560700.01	15.93	7.143	-1.147295	2.58E-43	2.14E-43	16.04	14.72	17.03	6.13	7.4	7.9
OsR498G0100564900.01	1.053	10.806	3.313751	6.98E-257	1.67E-257	1.01	0.91	1.24	11.1	10.76	10.56
OsR498G0100565800.01	69.08	148.55	1.0969857	0	0	66.14	69.66	71.44	152.99	140.62	152.04
OsR498G0100573900.01	0.05	0.936	5.103693	7.53E-10	1.17E-09	0	0	0.15	1.2	0.71	0.9
OsR498G0100578400.01	0.836	0.37	-1.174453	3.39E-09	5.38E-09	0.6	0.82	1.09	0.4	0.38	0.33
OsR498G0100589500.01	0.54	2.37	2.0347105	5.94E-20	7.13E-20	0.76	0.31	0.55	2.56	2.65	1.9
OsR498G0100589700.01	0.53	3.336	2.8900178	5.15E-15	6.89E-15	0.51	0.46	0.62	2.65	3.49	3.87
OsR498G0100590400.01	0.03	0.613	4.4118153	4.93E-16	6.44E-16	0.06	0	0.03	0.73	0.56	0.55
OsR498G0100591100.01	0.04	0.646	4.2162501	7.04E-10	1.09E-09	0.06	0	0.06	0.68	0.43	0.83
OsR498G0100591500.01	0.78	0.366	-1.092316	4.39E-17	5.61E-17	0.82	0.8	0.72	0.3	0.45	0.35
OsR498G0100594800.01	20.53	100.63	2.2922836	0	0	20.6	20.41	20.58	103.76	100.52	97.62
OsR498G0100602700.01	14.66	3	-2.290445	7.54E-101	3.77E-101	15.83	12.64	15.51	3.35	2.37	3.28
OsR498G0100605400.01	0.326	1.36	2.0602117	4.20E-37	3.80E-37	0.27	0.35	0.36	1.16	1.55	1.37
OsR498G0100607700.01	1.49	0.57	-1.529252	7.43E-17	9.54E-17	1.48	1.46	1.53	0.51	0.59	0.61
OsR498G0100609500.01	45.02	114.77	1.3536885	2.10E-269	4.83E-270	45.72	44.23	45.11	117.67	108.62	118.03
OsR498G0100611700.01	6.98	17.45	1.3026243	4.06E-66	2.65E-66	6.73	6.98	7.23	17.01	17.77	17.57
OsR498G0100612600.01	4.146	12.18	1.5616075	1.12E-96	5.74E-97	4.43	3.77	4.24	11.98	12.39	12.17
OsR498G0100612800.01	0.54	0.2	-1.41896	7.43E-06	1.35E-05	0.63	0.57	0.42	0.17	0.2	0.23
OsR498G0100613400.01	7.926	3.093	-1.427319	9.00E-75	5.45E-75	8.71	7.76	7.31	3.46	3.09	2.73
OsR498G0100616100.01	0.913	0.296	-1.543438	2.20E-06	3.92E-06	0.87	1	0.87	0.35	0.19	0.35
OsR498G0100618400.01	16.173	34.226	1.0818264	9.15E-85	5.15E-85	16.32	15.78	16.42	34.53	34.36	33.79
OsR498G0100622100.01	44.286	145.39	1.7166667	0	0	41.53	45.85	45.48	141.14	148.22	146.8
OsR498G0100622500.01	0	1.45	7.4979719	9.14E-22	1.06E-21	0	0	0	1.17	1.4	1.78
OsR498G0100622800.01	4.976	10.163	1.0325129	2.58E-25	2.81E-25	4.68	5.41	4.84	10.25	10.23	10.01
OsR498G0100623200.01	0.543	2.05	1.9116742	2.11E-19	2.56E-19	0.59	0.55	0.49	2.03	1.82	2.3
OsR498G0100625600.01	71.706	264.26	1.8795897	0	0	73.04	70.21	71.87	265.02	265.56	262.21
OsR498G0100627500.01	13.47	28.666	1.0479171	6.33E-172	2.14E-172	13.62	12.81	13.98	29.52	28.22	28.26
OsR498G0100627900.01	0.163	1.993	3.4399174	1.54E-61	1.05E-61	0.03	0.18	0.28	2	1.94	2.04
OsR498G0100628700.01	18.366	44.006	1.2917823	2.75E-202	8.17E-203	17.68	18.82	18.6	44.65	43.63	43.74
OsR498G0100629200.01	5.686	14.096	1.3090686	1.48E-75	8.93E-76	5.32	5.88	5.86	14.46	13.85	13.98
OsR498G0100632000.01	25.733	88.436	1.7801947	0	0	26.25	26.33	24.62	87.25	88.45	89.61
OsR498G0100634700.01	14.82	29.71	1.0025293	9.11E-101	4.56E-101	15.32	15.5	13.64	28.54	31.51	29.08
OsR498G0100635600.01	5.386	14.61	1.4361011	2.60E-43	2.16E-43	5.18	5.9	5.08	13.87	13.55	16.41
OsR498G0100638600.01	1.34	16.05	3.5808735	7.19E-261	1.70E-261	1.35	1.44	1.23	15.87	16.15	16.13
OsR498G0100639700.01	2.59	0.48	-2.381071	3.26E-13	4.57E-13	2.54	2.77	2.46	0.67	0.53	0.24
OsR498G0100640000.01	0.486	0.05	-3.258251	2.64E-10	4.03E-10	0.75	0.34	0.37	0.09	0.06	0
OsR498G0100642500.01	0.966	2.326	1.2658728	1.40E-10	2.12E-10	1.22	0.86	0.82	2.15	2.06	2.77
OsR498G0100651200.01	9.81	25.7	1.3890075	3.01E-271	6.90E-272	9.33	9.12	10.98	25.86	26.38	24.86
OsR498G0100653000.01	16.14	59.67	1.8582169	8.96E-220	2.46E-220	16.31	16.57	15.54	61.16	56.79	61.06
OsR498G0100653900.01	26.253	58.763	1.155776	1.06E-231	2.79E-232	26.53	25.48	26.75	58.12	61.03	57.14
OsR498G0100654200.01	0.42	1.09	1.5414505	1.53E-07	2.60E-07	0.17	0.55	0.54	1.06	1.21	1
OsR498G0100655000.01	1.236	2.736	1.1428636	5.69E-13	8.02E-13	1.3	1.08	1.33	2.33	2.84	3.04
OsR498G0100655300.01	0.45	0.086	-2.347518	2.97E-06	5.31E-06	0.7	0.38	0.27	0.07	0.11	0.08
OsR498G0100660500.01	3.016	7.596	1.3303855	1.14E-21	1.33E-21	2.57	2.72	3.76	6.59	8.1	8.1
OsR498G0100660800.01	2.356	5.53	1.226952	8.56E-16	1.13E-15	2.29	2.2	2.58	5.16	4.72	6.71
OsR498G0100663000.01	3.93	9.693	1.3580071	9.72E-124	4.21E-124	4.29	3.66	3.84	9.94	9.93	9.21
OsR498G0100668500.01	1052.1	373.85	-1.494701	0	0	1039.1	1049	1068.2	379.23	367.6	374.72
OsR498G0100668900.01	57.356	189.71	1.7249649	0	0	56.21	57.41	58.45	187.19	188.92	193.02

OsR498G0100669200.01	3.773	14.9	1.9798153	2.90E-138	1.16E-138	3.86	3.47	3.99	15.52	15.13	14.05
OsR498G0100672300.01	0.263	0.75	1.5087198	2.59E-08	4.25E-08	0.3	0.22	0.27	0.65	0.83	0.77
OsR498G0100674900.01	203.683	85.67	-1.245485	0	0	207.62	198.69	204.74	86.17	83.58	87.26
OsR498G0100676000.01	18.99	45.78	1.2530732	0	0	18.36	18.87	19.74	45.92	45.34	46.08
OsR498G0100676800.01	1.076	3.996	1.8822918	1.28E-36	1.17E-36	0.94	1.29	1	4	4.05	3.94
OsR498G0100678600.01	0.846	0.256	-1.990825	2.14E-10	3.26E-10	0.74	1	0.8	0.22	0.34	0.21
OsR498G0100681000.01	11.5	26	1.1694774	3.02E-106	1.45E-106	12.65	10.76	11.09	26.61	24.24	27.15
OsR498G0100683300.01	1.586	0	-7.468534	4.59E-21	5.40E-21	1.87	1.2	1.69	0	0	0
OsR498G0100687900.01	3.383	14.303	2.0644422	1.08E-92	5.71E-93	3.78	3	3.37	14.53	13.63	14.75
OsR498G0100688400.01	1.886	6.16	1.7039307	2.12E-39	1.86E-39	1.72	1.82	2.12	5.93	5.66	6.89
OsR498G0100694000.01	13.233	62.446	2.2341079	0	0	12.82	13.03	13.85	60.94	62.77	63.63
OsR498G0100695700.01	18.273	37.94	1.0844635	1.72E-127	7.32E-128	17.38	18.52	18.92	37.79	37.49	38.54
OsR498G0100701100.01	0.423	1.106	1.3853431	5.53E-09	8.84E-09	0.46	0.49	0.32	1.27	1.04	1.01
OsR498G0100708900.01	0.496	15.486	4.9641505	0	0	0.59	0.4	0.5	13.73	16.56	16.17
OsR498G0100709200.01	0.96	19.906	4.3745098	2.28E-234	5.98E-235	0.85	1.35	0.68	20.7	16.84	22.18
OsR498G0100721100.01	14.726	7.403	-1.000557	8.22E-34	7.79E-34	14.4	14.68	15.1	6.89	8.32	7
OsR498G0100721700.01	17.683	38.596	1.1174809	1.00E-281	2.24E-282	17.48	16.93	18.64	38.1	39.19	38.5
OsR498G0100726000.01	0.183	0	-4.72603	0.0001114	0.0002167	0.13	0.08	0.34	0	0	0
OsR498G0100727600.01	28.156	62.34	1.0553961	2.11E-169	7.21E-170	30.4	27.89	26.18	64.52	61.93	60.57
OsR498G0100728600.01	0.25	0.656	1.3894475	0.0001142	0.0002223	0.29	0.25	0.21	0.57	0.73	0.67
OsR498G0100728900.01	2.25	7.426	1.7244317	7.89E-49	6.15E-49	2.18	1.79	2.78	7.89	7.06	7.33
OsR498G0100731500.01	0.01	0.463	5.4979719	1.58E-12	2.25E-12	0.03	0	0	0.51	0.3	0.58
OsR498G0100734400.01	0.19	3.746	4.3494494	7.50E-38	6.71E-38	0.22	0.15	0.2	3.82	3.19	4.23
OsR498G0100734500.01	0.36	0.076	-2.273518	0.0004936	0.0010012	0.29	0.28	0.51	0	0.06	0.17
OsR498G0100736700.01	0.163	0.533	1.7023304	3.74E-06	6.71E-06	0.19	0.15	0.15	0.48	0.81	0.31
OsR498G0100742000.01	7.726	19.143	1.3073349	3.42E-242	8.71E-243	7.7	8.15	7.33	19.58	20.43	17.42
OsR498G0100745200.01	0.14	0.49	1.7933968	1.62E-05	3.00E-05	0.07	0.21	0.14	0.25	0.65	0.57
OsR498G0100746600.01	15.86	7.18	-1.142508	8.95E-33	8.62E-33	15.82	16.4	15.36	8.27	6.92	6.35
OsR498G0100749600.01	0.65	1.443	1.0962309	5.94E-08	9.88E-08	0.65	0.38	0.92	1.27	1.34	1.72
OsR498G0100750800.01	0.04	2.42	5.8692277	1.31E-29	1.33E-29	0	0.12	0	2.18	2.46	2.62
OsR498G0100751600.01	0.223	2.606	3.5157832	1.09E-32	1.05E-32	0.24	0.24	0.19	2.45	2.68	2.69
OsR498G0100753000.01	16.916	70.026	2.0546322	3.70E-257	8.86E-258	15.17	16.99	18.59	70.35	67.06	72.67
OsR498G0100753700.01	0.453	2.366	2.3964747	2.83E-46	2.27E-46	0.42	0.25	0.69	2.38	2.61	2.11
OsR498G0100756500.01	19.54	46.316	1.3084221	3.21E-123	1.39E-123	20.41	20.19	18.02	45.83	47.46	45.66
OsR498G0100761500.01	1.753	22.476	3.6714828	0	0	1.72	2.07	1.47	21.95	23.13	22.35
OsR498G0100762200.01	52.66	20.99	-1.32786	1.67E-251	4.08E-252	52.3	55.34	50.34	20.66	21.67	20.64
OsR498G0100765000.01	0.376	0.093	-1.958476	4.74E-06	8.57E-06	0.49	0.16	0.48	0.05	0.1	0.13
OsR498G0100768200.01	0.833	36.37	5.4399764	0	0	0.68	0.83	0.99	34.92	37.42	36.77
OsR498G0100776800.01	6.05	23.613	1.9431793	0	0	5.98	6.26	5.91	24.22	24.48	22.14
OsR498G0100778500.01	1.796	6.24	1.7925716	8.23E-20	9.90E-20	1.9	1.89	1.6	5.54	5.89	7.29
OsR498G0100785100.01	0.03	0.753	5.0618728	1.33E-09	2.09E-09	0	0.09	0	0.86	0.72	0.68
OsR498G0100786600.01	4.21	2.1	-1.126676	8.49E-08	1.42E-07	4.38	4.1	4.15	2.74	1.64	1.92
OsR498G0100789400.01	306.796	679.43	1.1452378	0	0	320.56	292.72	307.11	643.04	664.43	730.82
OsR498G0100791300.01	0.14	0.42	1.589276	1.98E-07	3.36E-07	0.15	0.15	0.12	0.7	0.2	0.36
OsR498G0100791600.01	9.493	42.39	2.1506887	0	0	10.44	8.83	9.21	42.26	42.21	42.7
OsR498G0100794900.01	63.65	135.09	1.1282248	2.78E-171	9.43E-172	63.92	63.97	63.06	131.21	126.03	148.03
OsR498G0100797300.01	5.366	1.35	-1.991907	1.76E-28	1.82E-28	5.35	5.18	5.57	1.11	1.29	1.65
OsR498G0100798100.01	3.363	1.62	-1.056092	1.48E-11	2.18E-11	3.23	3.44	3.42	1.23	1.77	1.86
OsR498G0100800300.01	8.586	36.206	2.0374431	0	0	8.71	8.51	8.54	36.55	35.89	36.18
OsR498G0100817200.01	5.62	12.583	1.1708072	3.12E-64	2.07E-64	5.38	5.51	5.97	12.95	12.42	12.38
OsR498G0100833100.01	0.54	1.693	1.6442614	4.08E-10	6.28E-10	0.54	0.49	0.59	1.53	1.24	2.31
OsR498G0100834600.01	6.793	3.086	-1.143198	1.39E-20	1.65E-20	6.26	7.04	7.08	3.01	2.55	3.7
OsR498G0100834900.01	18.163	98.766	2.4656297	0	0	20.1	16.22	18.17	98.51	96.21	101.58
OsR498G0100836600.01	0.08	0.416	2.4338416	1.41E-07	2.38E-07	0.12	0.06	0.06	0.34	0.33	0.58
OsR498G0100840600.01	16.576	36.83	1.1602249	2.13E-164	7.54E-165	16.51	17.09	16.13	36.32	37.4	36.77
OsR498G0100841100.01	2.256	7.473	1.7192807	1.31E-104	6.37E-105	2.13	2.56	2.08	7.2	8.13	7.09
OsR498G0100841700.01	0.38	0.066	-2.569911	9.30E-07	1.63E-06	0.34	0.37	0.43	0.07	0.03	0.1
OsR498G0100850400.01	170.083	10.483	-4.016009	0	0	171.23	171.77	167.25	11.53	10.24	9.68
OsR498G0100850700.01	6.83	2.54	-1.415785	1.11E-88	6.05E-89	6.76	7.26	6.47	2.24	2.91	2.47
OsR498G0100862300.01	8.676	24.346	1.4856936	2.54E-185	8.10E-186	8.26	8.75	9.02	23.94	25.51	23.59
OsR498G0100863300.01	22.543	52.01	1.2087499	7.09E-271	1.63E-271	22.72	23.38	21.53	53.52	52.27	50.24
OsR498G0100866200.01	4.32	0.596	-2.85958	1.82E-80	1.06E-80	4.57	3.91	4.48	0.64	0.39	0.76
OsR498G0100879300.01	5.316	1.726	-1.581601	1.12E-35	1.03E-35	5.11	5.49	5.35	2.15	1.29	1.74
OsR498G0100880900.01	0.256	0.706	1.573872	3.16E-09	5.01E-09	0.08	0.36	0.33	0.65	0.82	0.65
OsR498G0100884100.01	10.123	2.353	-2.109654	1.23E-61	8.33E-62	9.16	10.79	10.42	2.24	2.12	2.7
OsR498G0100886700.01	0.016	0.51	4.8813006	1.33E-08	2.15E-08	0	0	0.05	0.71	0.25	0.57
OsR498G0100887200.01	1.076	0.033	-4.932481	9.49E-09	1.53E-08	1.4	1.29	0.54	0	0.1	0
OsR498G0100897400.01	8.913	27.466	1.6339933	5.34E-280	1.20E-280	9.1	8.56	9.08	28.31	28.16	25.93
OsR498G0100901500.01	14.656	58.863	2.0041073	4.01E-251	9.84E-252	12.31	14.37	17.29	57.15	58.94	60.5

OsR498G0100909100.01	0.953	2.12	1.154231	1.16E-13	1.61E-13	0.97	1.03	0.86	1.85	2.28	2.23
OsR498G0100909500.01	5.146	10.193	1.0400193	3.64E-64	2.42E-64	5.01	4.89	5.54	10.06	10.76	9.76
OsR498G0100913700.01	5.456	32.776	2.5857066	0	0	5.54	5.36	5.47	33.82	30.83	33.68
OsR498G0100918400.01	5.9	14.106	1.2637947	5.47E-142	2.14E-142	5.81	5.79	6.1	13.66	15.04	13.62
OsR498G0100930500.01	30.343	5.416	-2.484835	0	0	30.17	29.26	31.6	5.27	5.1	5.88
OsR498G0100933400.01	6.833	14.976	1.1309677	4.32E-27	4.58E-27	7	5.98	7.52	15.03	15.05	14.85
OsR498G0100943000.01	24.63	4.023	-2.625239	0	0	25.03	24.93	23.93	4.45	3.94	3.68
OsR498G0100949500.01	7.54	15.36	1.005085	3.57E-28	3.71E-28	7.64	7.85	7.13	16.56	13.79	15.73
OsR498G0100954400.01	11.653	32.21	1.4670458	1.63E-302	3.44E-303	11.63	11.73	11.6	32.25	32.82	31.56
OsR498G0100956900.01	0.55	0.163	-1.901541	3.99E-10	6.14E-10	0.67	0.52	0.46	0.05	0.28	0.16
OsR498G0100964000.01	17.263	43.556	1.3295007	0	0	18.22	16.19	17.38	44.48	44.82	41.37
OsR498G0100966100.01	0.726	0.23	-1.650081	0.0001057	0.0002052	0.36	0.82	1	0.4	0.17	0.12
OsR498G0100966900.01	22.71	2.393	-3.246141	8.46E-143	3.29E-143	22.99	23.26	21.88	2.63	2.2	2.35
OsR498G0100968500.01	0.183	0.53	1.5407568	2.57E-07	4.38E-07	0.21	0.13	0.21	0.51	0.43	0.65
OsR498G0100969800.01	1.82	16.176	3.1538452	5.05E-133	2.08E-133	1.63	1.88	1.95	17.36	16.6	14.57
OsR498G0100970600.01	3.163	1.133	-1.491254	4.05E-22	4.67E-22	3.65	2.96	2.88	1.69	0.83	0.88
OsR498G0100971200.01	0	0.156	4.6748497	0.0001298	0.0002536	0	0	0	0.07	0.29	0.11
OsR498G0100978100.01	0.18	2.776	3.9352394	3.40E-24	3.77E-24	0.16	0.15	0.23	2.65	2.49	3.19
OsR498G0100978400.01	0	0.123	4.2963381	0.0007942	0.0016388	0	0	0	0.15	0.11	0.11
OsR498G0100980000.01	0.08	3.453	5.4501434	1.50E-22	1.72E-22	0.12	0	0.12	3.24	3.45	3.67
OsR498G0100981200.01	0.146	0.356	1.286354	1.10E-05	2.03E-05	0.14	0.21	0.09	0.27	0.4	0.4
OsR498G0100984500.01	10.423	22.89	1.1348746	2.00E-80	1.17E-80	9.61	10.55	11.11	23.36	23.5	21.81
OsR498G0100984800.01	2.246	10.85	2.2822205	3.06E-163	1.09E-163	2.31	2.14	2.29	10.18	12.04	10.33
OsR498G0100985300.01	0.983	2.09	1.2018205	2.80E-11	4.16E-11	1.12	0.72	1.11	2.08	1.97	2.22
OsR498G0100997400.01	29.61	6.05	-2.291627	5.28E-122	2.30E-122	29.68	30.4	28.75	5.77	6.56	5.82
OsR498G0101005100.01	3.716	1.826	-1.02773	4.83E-16	6.32E-16	4	3.36	3.79	2.18	1.43	1.87
OsR498G0101007700.01	14.23	37.113	1.3820084	1.40E-92	7.44E-93	15.8	13.93	12.96	37.79	35.63	37.92
OsR498G0101010400.01	0.02	0.163	3.0618728	0.0001482	0.0002902	0	0.03	0.03	0.14	0.17	0.18
OsR498G0101021000.01	0.93	0.04	-4.688555	3.50E-19	4.27E-19	0.87	1.34	0.58	0	0.04	0.08
OsR498G0101022400.01	0.453	1.813	1.8003806	2.44E-12	3.50E-12	0.4	0.33	0.63	1.71	1.9	1.83
OsR498G0101023400.01	1.766	0.403	-2.141067	2.76E-12	3.96E-12	1.64	1.74	1.92	0.46	0.17	0.58
OsR498G0101025500.01	0.153	9.606	5.9828386	1.08E-121	4.73E-122	0.06	0.23	0.17	9.1	9.4	10.32
OsR498G0101026700.01	0.45	66	7.1977222	0	0	0.32	0.58	0.45	66.18	62.91	68.91
OsR498G0101029700.01	18.713	50.523	1.4802607	0	0	20.27	17.93	17.94	50.99	51.48	49.1
OsR498G0101038400.01	1	1.25	1.001377	1.07E-05	1.96E-05	1.14	0.86	1	1.49	0.96	1.3
OsR498G0101039100.01	24.5	71.246	1.5598985	8.96E-203	2.66E-203	24.74	24.06	24.7	70.72	67.43	75.59
OsR498G0101042900.01	29.97	11.27	-1.366845	5.61E-110	2.64E-110	29.19	28.88	31.84	12.04	11.56	10.21
OsR498G0101052000.01	0.09	5.03	5.8624767	5.17E-126	2.22E-126	0.09	0.06	0.12	4.69	5	5.4
OsR498G0101055400.01	0.713	0.283	-1.02559	0.000629	0.0012854	1.06	0.66	0.42	0.16	0.43	0.26
OsR498G0101057900.01	3.573	0.643	-2.398542	4.85E-36	4.45E-36	3.35	3.68	3.69	0.92	0.68	0.33
OsR498G0101059100.01	2.81	5.626	1.0217157	5.15E-23	5.83E-23	3.5	2.45	2.48	5.99	5.41	5.48
OsR498G0101060700.01	2984.44	667.24	-2.159495	0	0	3000.9	2939.5	3012.9	665.14	653.4	683.19
OsR498G0101068400.01	6.15	2.863	-1.250945	6.18E-54	4.54E-54	6.24	6.41	5.8	3.22	2.63	2.74
OsR498G0101070200.01	2.79	1.043	-1.417907	4.44E-14	6.09E-14	2.98	2.38	3.01	1.08	1.03	1.02
OsR498G0101071600.01	2.24	11.636	2.3797273	2.33E-166	8.12E-167	2.01	2.43	2.28	11.57	10.89	12.45
OsR498G0101075900.01	0.54	4.023	2.8488791	1.00E-39	8.77E-40	0.64	0.53	0.45	4.28	3.9	3.89
OsR498G0101078100.01	0.71	2.39	1.7673137	5.70E-28	5.95E-28	0.62	0.91	0.6	2.67	2.07	2.43
OsR498G0101078300.01	0.083	0.326	1.9997009	0.0002597	0.0005163	0.06	0.14	0.05	0.24	0.39	0.35
OsR498G0101080700.01	19.906	46.323	1.2172906	1.59E-202	4.71E-203	20.78	20.13	18.81	44.88	45.1	48.99
OsR498G0101084100.01	83.123	29.966	-1.474051	0	0	85.68	82.68	81.01	32.33	28.7	28.87
OsR498G0101084700.01	20.53	55.223	1.4462787	2.91E-261	6.86E-262	20.48	21.52	19.59	55.18	57.37	53.12
OsR498G0101085300.01	0.403	0.076	-2.762556	5.92E-08	9.85E-08	0.42	0.45	0.34	0.06	0.1	0.07
OsR498G0101085900.01	0.12	1.346	3.4769103	3.70E-08	6.10E-08	0.36	0	0	2.12	0.47	1.45
OsR498G0101089100.01	2.67	0.206	-3.693969	1.23E-19	1.49E-19	2.35	3.32	2.34	0.18	0.26	0.18
OsR498G0101095500.01	25.116	64.116	1.3448132	0	0	23.98	26.19	25.18	64.4	63.68	64.27
OsR498G0101096000.01	0.146	4.9	5.0296924	2.51E-32	2.44E-32	0.11	0.22	0.11	5.6	4.49	4.61
OsR498G0101098100.01	1.16	3.01	1.3581142	8.91E-12	1.30E-11	1.03	1.65	0.8	3.27	3.14	2.62
OsR498G0101102400.01	1.223	23.033	4.2302489	4.12E-275	9.34E-276	1.54	1.04	1.09	22.81	23.89	22.4
OsR498G0101102600.01	1.503	7.75	2.3661193	1.79E-77	1.07E-77	1.92	1.27	1.32	7.85	7.28	8.12
OsR498G0101102800.01	14.146	29.15	1.0592486	1.05E-106	5.05E-107	13.57	14.33	14.54	28.36	29.43	29.66
OsR498G0101108400.01	5.093	12.34	1.2736088	4.10E-68	2.62E-68	4.84	4.84	5.6	12.45	12.07	12.5
OsR498G0101110800.01	1.056	2.743	1.5928472	2.49E-18	3.09E-18	0.63	1.27	1.27	2.44	2.79	3
OsR498G0101111200.01	10.333	27.486	1.4284564	7.58E-88	4.17E-88	11.37	10.35	9.28	26.24	24.84	31.38
OsR498G0101111400.01	3.763	17.93	2.2763003	1.78E-158	6.45E-159	3.5	3.72	4.07	17.69	17.76	18.34
OsR498G0101112100.01	8.203	22.613	1.3921597	2.65E-232	6.98E-233	8.33	8.28	8	23.23	23.01	21.6
OsR498G0101114500.01	0.306	0.936	1.2523947	7.28E-07	1.27E-06	0.37	0.33	0.22	1.03	1.2	0.58
OsR498G0101117500.01	0.246	0.733	1.5810675	9.00E-07	1.57E-06	0.24	0.27	0.23	0.95	0.65	0.6
OsR498G0101140500.01	0	0.173	5.3667274	1.24E-06	2.19E-06	0	0	0	0.1	0.17	0.25
OsR498G0101142900.01	13.57	3.936	-1.784852	1.76E-87	9.71E-88	15.11	12.05	13.55	4.56	3.42	3.83

OsR498G0101147900.01	10.44	1.436	-2.86581	1.98E-37	1.78E-37	11.18	10.72	9.42	1.52	0.96	1.83
OsR498G0101155200.01	0.61	0.183	-1.748056	2.01E-13	2.80E-13	0.7	0.59	0.54	0.23	0.11	0.21
OsR498G0101164000.01	0.833	4.77	2.5198441	4.20E-61	2.87E-61	1.09	0.97	0.44	5.23	4.65	4.43
OsR498G0101176700.01	0.006	0.193	4.331962	1.10E-10	1.67E-10	0.01	0	0.01	0.25	0.14	0.19
OsR498G0101178700.01	4.816	16.72	1.8031738	0	0	5.16	4.83	4.46	17.37	17.31	15.48
OsR498G0101179100.01	13.256	39.313	1.568591	0	0	13.74	12.48	13.55	39.84	39.77	38.33
OsR498G0101179400.01	2.893	1.4	-1.102115	1.57E-25	1.70E-25	2.89	3.19	2.6	1.27	1.46	1.47
OsR498G0101185300.01	1.796	4.273	1.3046774	2.64E-21	3.09E-21	1.75	2	1.64	4.27	3.71	4.84
OsR498G0101185700.01	1.16	0.256	-2.195515	5.13E-06	9.29E-06	0.87	1.16	1.45	0.19	0.19	0.39
OsR498G0101188200.01	0.04	4.086	6.6859049	3.37E-121	1.48E-121	0.02	0.03	0.07	4.23	4.15	3.88
OsR498G0101225100.01	0.396	1.693	2.103693	1.98E-13	2.76E-13	0.3	0.64	0.25	1.44	1.72	1.92
OsR498G0101225400.01	36.133	100.84	1.445721	0	0	36.54	36.77	35.09	99.31	104.22	99
OsR498G0101226100.01	0.356	1.213	1.8748743	1.83E-12	2.63E-12	0.25	0.53	0.29	1.17	0.97	1.5
OsR498G0101246700.01	0.036	0.513	4.31426	2.41E-19	2.93E-19	0.06	0.03	0.02	0.47	0.41	0.66
OsR498G0101257300.01	1.146	0.233	-2.28655	3.49E-19	4.26E-19	1.12	1.21	1.11	0.27	0.26	0.17
OsR498G0101257500.01	2.643	0.71	-1.892324	3.83E-26	4.13E-26	3.11	2.81	2.01	0.7	0.87	0.56
OsR498G0101258700.01	3.153	10.593	1.8033431	1.18E-77	6.99E-78	2.68	3.08	3.7	10.6	10.97	10.21
OsR498G0101259400.01	0.12	0	-4.347518	0.0007014	0.0014405	0.11	0.04	0.21	0	0	0
OsR498G0101268300.01	1.443	0.7	-1.043077	1.02E-08	1.65E-08	1.34	1.49	1.5	0.72	0.63	0.75
OsR498G0101271400.01	0.2	2.323	3.5593725	2.19E-45	1.78E-45	0.19	0.24	0.17	2.13	2.62	2.22
OsR498G0101273200.01	8.07	20.173	1.2805826	1.70E-145	6.50E-146	7.96	8.28	7.97	19.49	20.66	20.37
OsR498G0101276700.01	16.246	47.85	1.5237938	0	0	16.65	15.78	16.31	45.69	48.1	49.76
OsR498G0101278700.01	41.03	135.12	1.7929768	0	0	40.32	39.98	42.79	136.29	135.95	133.12
OsR498G0101279600.01	21.056	55.606	1.4007694	0	0	20.96	20.87	21.34	56.24	57.54	53.04
OsR498G0101280600.01	438.956	1433.8	1.7071515	0	0	438.05	438.06	440.76	1424.2	1400.7	1476.5
OsR498G0101280700.01	0.23	0.906	1.9878892	5.63E-16	7.38E-16	0	0.24	0.45	1.37	0.98	0.37
OsR498G0101288000.01	0.526	0.206	-1.348103	6.07E-07	1.05E-06	0.42	0.61	0.55	0.34	0.2	0.08
OsR498G0101288200.01	2.02	0.743	-1.444812	7.71E-08	1.29E-07	3.01	1.85	1.2	0.52	0.71	1
OsR498G0101288300.01	0.21	1.193	2.5989008	5.08E-07	8.79E-07	0.2	0.23	0.2	1.61	1.01	0.96
OsR498G0101311500.01	9.91	4.003	-1.233573	1.64E-43	1.36E-43	10.77	9.51	9.45	3.67	3.49	4.85
OsR498G0101312600.01	0.84	2.346	1.4874795	3.49E-11	5.19E-11	0.92	0.77	0.83	2.43	2.66	1.95
OsR498G0101321600.01	2.546	5.32	1.1326215	5.58E-39	4.92E-39	2.52	2.86	2.26	5.53	5.12	5.31
OsR498G0101322400.01	10.023	26.933	1.4240081	8.20E-112	3.82E-112	9.28	10.52	10.27	24.3	27.45	29.05
OsR498G0101330600.01	0.616	31.943	5.6952886	0	0	0.72	0.71	0.42	31.16	29.81	34.86
OsR498G0101330900.01	9.86	20.533	1.0699534	8.72E-120	3.86E-120	10.37	9.69	9.52	19.51	21.05	21.04
OsR498G0101331200.01	18.076	42.73	1.225138	2.56E-152	9.48E-153	19.11	18.14	16.98	42.86	42.23	43.1
OsR498G0101333000.01	118.926	287.55	1.2765596	0	0	117.54	121.37	117.87	292.35	286.97	283.33
OsR498G0101333500.01	0.23	1.436	2.6468353	3.48E-09	5.52E-09	0.26	0.26	0.17	1.34	1.51	1.46
OsR498G0101333900.01	8.443	22.766	1.4191603	1.84E-297	3.91E-298	8.6	8.41	8.32	23.01	23.24	22.05
OsR498G0101334700.01	7.203	23.633	1.737121	3.83E-211	1.10E-211	6.78	7.99	6.84	23.98	23.62	23.3
OsR498G0101336300.01	9.92	26.803	1.4480476	5.42E-181	1.77E-181	9.81	9.63	10.32	27.27	24.99	28.15
OsR498G0101337500.01	109.036	280.99	1.3645026	0	0	108.37	112.15	106.59	287.66	281.58	273.72
OsR498G0101339800.01	6.836	14.816	1.115135	1.22E-60	8.40E-61	6.7	7.16	6.65	14.85	14.33	15.27
OsR498G0101341800.01	0.01	0.12	3.5593725	0.0005724	0.0011661	0.03	0	0	0.15	0.06	0.15
OsR498G0101347500.01	0.213	0.036	-2.195515	0.0008316	0.0017206	0.11	0.16	0.37	0.05	0	0.06
OsR498G0101352700.01	7.296	16.173	1.146027	1.07E-34	9.97E-35	6.79	7.13	7.97	15.49	15.88	17.15
OsR498G0101353000.01	0.223	0.636	1.5025696	3.88E-06	6.97E-06	0.17	0.33	0.17	0.68	0.84	0.39
OsR498G0101356600.01	1.876	7.553	2.0058882	9.55E-31	9.50E-31	2.08	1.9	1.65	7.77	7.17	7.72
OsR498G0101357200.01	1.45	0.633	-1.183131	6.88E-09	1.11E-08	1.46	1.07	1.82	0.81	0.64	0.45
OsR498G0101357600.01	1.87	0.093	-4.334343	7.08E-50	5.45E-50	2.07	1.74	1.8	0.03	0.07	0.18
OsR498G0101359400.01	10.833	26.5	1.2877289	1.79E-61	1.22E-61	10.69	11.27	10.54	25.84	23.57	30.09
OsR498G0101369200.01	7.056	21.863	1.6493871	1.14E-223	3.10E-224	7.42	7.06	6.69	23.3	20.82	21.47
OsR498G0101369900.01	1.513	0.49	-1.637025	4.80E-15	6.42E-15	1.63	1.59	1.32	0.54	0.43	0.5
OsR498G0101370900.01	0.126	1.2	3.2374444	8.33E-19	1.02E-18	0.15	0.15	0.08	1.3	1.15	1.15
OsR498G0101379800.01	2.45	0.673	-1.621693	4.35E-12	6.29E-12	3.04	2.42	1.89	0.64	0.75	0.63
OsR498G0101382500.01	0.19	13.15	6.1183397	0	0	0.11	0.24	0.22	13.05	13.44	12.96
OsR498G0101384900.01	2.803	0.55	-2.347518	1.81E-11	2.67E-11	2.59	3.47	2.35	0.77	0.66	0.22
OsR498G0101399300.01	0.676	0.183	-1.93986	1.02E-06	1.78E-06	0.6	0.52	0.91	0.09	0.17	0.29
OsR498G0101399700.01	3.786	15.56	2.0310994	2.20E-59	1.53E-59	3.57	3.46	4.33	16.37	15.12	15.19
OsR498G0101400300.01	2.643	6.926	1.4222652	2.55E-65	1.68E-65	2.25	2.7	2.98	6.78	7.58	6.42
OsR498G0101400900.01	0.853	1.78	1.0305777	1.07E-14	1.44E-14	0.86	0.77	0.93	1.57	1.8	1.97
OsR498G0101401400.01	0.22	0.066	-1.762556	0.0002331	0.000462	0.2	0.28	0.18	0.02	0.09	0.09
OsR498G0101403700.01	0.223	0.08	-1.485022	0.0005725	0.0011665	0.25	0.26	0.16	0.08	0.1	0.06
OsR498G0101407900.01	7.933	16.49	1.0798309	6.61E-82	3.81E-82	7.58	7.71	8.51	17.4	14.65	17.42
OsR498G0101409500.01	11.896	28.09	1.2383257	4.46E-137	1.79E-137	11.57	12.3	11.82	28.6	27.51	28.16
OsR498G0101410200.01	1.15	5.1	2.1311896	6.43E-51	4.90E-51	1.1	0.91	1.44	5.75	4.89	4.66
OsR498G0101413800.01	0.18	0.76	2.0767717	9.79E-10	1.53E-09	0.19	0.22	0.13	0.82	0.72	0.74
OsR498G0101415900.01	0.363	1.566	2.1152411	8.31E-86	4.64E-86	0.38	0.31	0.4	1.88	1.78	1.04
OsR498G0101416100.01	0.773	2.793	1.8532777	2.44E-53	1.80E-53	0.68	0.8	0.84	2.38	2.94	3.06

OsR498G0101416500.01	3.94	65.92	4.0639114	0	0	4	3.79	4.03	64.81	61.95	71
OsR498G0101417500.01	0	2.276	7.3838009	2.06E-20	2.45E-20	0	0	0	2.24	2.38	2.21
OsR498G0101418800.01	160.063	67.6	-1.242312	0	0	173.41	144.57	162.21	65.28	60.59	76.93
OsR498G0101418900.01	0.63	1.73	1.4483411	5.30E-05	0.000101	0.63	0.84	0.42	1.03	1.64	2.52
OsR498G0101426100.01	0.423	0.193	-1.115227	2.01E-05	3.74E-05	0.49	0.4	0.38	0.21	0.22	0.15
OsR498G0101428600.01	0.063	1.363	4.4662631	1.28E-21	1.49E-21	0.19	0	0	1.58	1.21	1.3
OsR498G0101433200.01	349.323	120.58	-1.556755	0	0	344.57	360.32	343.08	122.34	118.02	121.38
OsR498G0101435800.01	15.226	7.503	-1.007389	2.51E-52	1.88E-52	15.22	15.4	15.06	7.17	7.59	7.75
OsR498G0101437100.01	2.993	7.33	1.2911763	1.23E-32	1.19E-32	2.66	3.07	3.25	7.31	7.44	7.24
OsR498G0101437600.01	0.163	0.556	1.8099313	1.44E-10	2.19E-10	0.13	0.18	0.18	0.64	0.53	0.5
OsR498G0101442100.01	0.286	0.073	-1.92371	7.63E-06	1.39E-05	0.25	0.31	0.3	0.12	0.08	0.02
OsR498G0101443900.01	59.106	129.04	1.1253029	0	0	59.38	57.37	60.57	127.88	125.42	133.83
OsR498G0101445100.01	12.993	52.536	2.005035	0	0	13.55	13.07	12.36	51.14	52.93	53.54
OsR498G0101449900.01	0.166	1.826	3.4598368	2.79E-12	4.01E-12	0.2	0.2	0.1	1.85	1.94	1.69
OsR498G0101450600.01	0.193	0.456	1.2138759	5.32E-06	9.64E-06	0.18	0.2	0.2	0.43	0.44	0.5
OsR498G0101455200.01	0.39	1.09	1.4803009	1.41E-06	2.48E-06	0.19	0.42	0.56	1.19	1	1.08
OsR498G0101455300.01	8.23	23.47	1.5103019	4.64E-127	1.98E-127	7.31	9.19	8.19	22.25	23.44	24.72
OsR498G0101459000.01	0.606	0.186	-1.72603	0.0006425	0.0013145	0.49	0.77	0.56	0.21	0.07	0.28
OsR498G0101461100.01	18.236	48.09	1.3353358	8.92E-162	3.18E-162	18.74	16.88	19.09	47.95	44.11	52.21
OsR498G0101462100.01	8.723	74.96	3.0838235	0	0	8.62	8.02	9.53	69.46	71.95	83.47
OsR498G0101462300.01	0.523	1.206	1.2582029	1.04E-07	1.74E-07	0.4	0.52	0.65	1.41	0.96	1.25
OsR498G0101466500.01	0.246	3.27	3.7445311	4.64E-45	3.77E-45	0.37	0.14	0.23	3.01	3.15	3.65
OsR498G0101469100.01	0.36	2.803	2.961919	2.89E-39	2.54E-39	0.34	0.37	0.37	3.48	2.02	2.91
OsR498G0101469300.01	0.67	7.25	3.4387823	1.81E-110	8.50E-111	0.69	0.68	0.64	8.01	6.76	6.98
OsR498G0101471100.01	0.026	0.67	4.5891198	1.03E-12	1.47E-12	0	0.08	0	0.57	0.59	0.85
OsR498G0101471900.01	2.193	0.366	-2.583585	2.62E-32	2.55E-32	2.03	2.41	2.14	0.33	0.36	0.41
OsR498G0101475400.01	2.143	32.09	3.9048707	0	0	1.95	2	2.48	33.02	31.55	31.7
OsR498G0101476300.01	572.64	36.69	-3.955537	0	0	619.22	544.18	554.52	36.95	34.71	38.41
OsR498G0101476600.01	6.346	1.963	-1.638133	7.91E-15	1.07E-14	8.65	5.27	5.12	2.1	1.83	1.96
OsR498G0101477000.01	3.363	0.95	-1.830334	1.08E-27	1.13E-27	3.57	3.53	2.99	0.86	0.96	1.03
OsR498G0101477700.01	460.2	30.61	-3.907941	0	0	409.47	438.04	533.09	26.15	28.5	37.18
OsR498G0101479800.01	0.046	0.343	2.9484147	1.02E-10	1.54E-10	0.04	0.06	0.04	0.26	0.44	0.33
OsR498G0101480400.01	1.74	7.643	2.1343509	1.65E-147	6.28E-148	1.85	1.84	1.53	7.87	8.01	7.05
OsR498G0101482200.01	10.91	65.38	2.5852147	0	0	10.79	10.8	11.14	66.13	68.57	61.44
OsR498G0101482400.01	15.24	141.1	3.2152522	0	0	15.17	15.77	14.78	138.34	140.16	144.79
OsR498G0101483000.01	14.013	69.143	2.2990833	0	0	13.74	14.58	13.72	67.11	74.4	65.92
OsR498G0101483200.01	0.033	0.423	3.6748497	7.57E-07	1.32E-06	0.1	0	0	0.53	0.34	0.4
OsR498G0101485500.01	7.563	3.08	-1.294648	9.26E-67	6.00E-67	7.55	7.59	7.55	3.11	2.96	3.17
OsR498G0101486800.01	4.556	9.356	1.036049	1.81E-32	1.75E-32	4.73	4.43	4.51	8.99	10.04	9.04
OsR498G0101487100.01	0.053	0.213	1.9752574	3.44E-05	6.48E-05	0	0	0.16	0.16	0.48	0
OsR498G0101488300.01	1.673	4.306	1.3698597	3.74E-40	3.25E-40	2.03	1.47	1.52	4.51	4.62	3.79
OsR498G0101497700.01	0.453	1.36	1.5712204	1.34E-10	2.02E-10	0.36	0.29	0.71	1.27	1.22	1.59
OsR498G0101497900.01	0.023	0.293	3.7610063	1.47E-15	1.95E-15	0.01	0.03	0.03	0.32	0.27	0.29
OsR498G0101498300.01	4.66	9.423	1.0155738	3.23E-55	2.34E-55	4.66	4.38	4.94	9.35	9.58	9.34
OsR498G0101499300.01	0.25	0.02	-3.72603	0.0002637	0.0005248	0.23	0.29	0.23	0	0	0.06
OsR498G0101499500.01	2.536	12.77	2.3183644	2.27E-59	1.58E-59	3.01	2.61	1.99	14.15	12.69	11.47
OsR498G0101500000.01	0.776	2.756	1.8239183	2.50E-19	3.04E-19	1.06	0.7	0.57	3.18	2.53	2.56
OsR498G0101503700.01	1.713	9.91	2.535245	7.34E-138	2.94E-138	1.91	1.6	1.63	10.45	9.76	9.52
OsR498G0101506700.01	5.693	2.576	-1.144578	3.88E-24	4.30E-24	6.16	5.98	4.94	2.5	2.73	2.5
OsR498G0101507200.01	0.146	0	-5.273518	3.18E-06	5.68E-06	0.12	0.08	0.24	0	0	0
OsR498G0101515200.01	1.973	4.756	1.271027	1.04E-68	6.59E-69	1.91	2.21	1.8	4.91	4.96	4.4
OsR498G0101517800.01	0.19	4.156	4.4662631	1.10E-238	2.85E-239	0.18	0.12	0.27	4.4	4.19	3.88
OsR498G0101520100.01	1.88	6.386	1.7592629	1.17E-30	1.16E-30	1.92	1.68	2.04	7.04	6.13	5.99
OsR498G0101521200.01	8.426	20.643	1.2981924	2.20E-88	1.20E-88	8.56	8.24	8.48	21.13	20.93	19.87
OsR498G0101522300.01	0.203	0.036	-2.610553	0.0002631	0.0005232	0.24	0.06	0.31	0.06	0	0.05
OsR498G0101522700.01	7.913	22.14	1.4855793	1.03E-96	5.31E-97	8.41	7.75	7.58	22.96	21.7	21.76
OsR498G0101526300.01	0.6	0.036	-4.134115	1.14E-16	1.47E-16	0.45	0.65	0.7	0.08	0	0.03
OsR498G0101528100.01	14.246	32.893	1.2049334	6.56E-87	3.63E-87	11.91	13.52	17.31	31	33.22	34.46
OsR498G0101531300.01	0.5	2.963	2.832594	6.99E-13	9.89E-13	0.39	0	1.11	6.29	0.19	2.41
OsR498G0101532700.01	0.486	0.23	-1.065854	2.94E-06	5.25E-06	0.43	0.37	0.66	0.22	0.22	0.25
OsR498G0101536000.01	0.346	1.086	1.656234	6.15E-08	1.02E-07	0.52	0.3	0.22	1.05	1.17	1.04
OsR498G0101540400.01	2.636	89.036	5.0721095	0	0	2.48	3.18	2.25	84.88	85.27	96.96
OsR498G0101540500.01	0.063	7.5	6.9051473	7.88E-57	5.64E-57	0	0.19	0	7.24	7.66	7.6
OsR498G0101541200.01	2.956	9.953	1.7505969	5.05E-77	3.01E-77	2.99	2.81	3.07	10.28	9.53	10.05
OsR498G0101546500.01	1.946	9.673	2.2918226	3.58E-80	2.09E-80	2.02	1.59	2.23	8.67	9.35	11
OsR498G0101550800.01	3.406	15.39	2.1869268	1.82E-97	9.32E-98	3.2	3.06	3.96	17.47	16.22	12.48
OsR498G0101552600.01	0.263	0.053	-2.28193	4.08E-07	7.02E-07	0.24	0.31	0.24	0	0.07	0.09
OsR498G0101552900.01	3.5	37.07	3.4181281	0	0	3.55	3.66	3.29	38.66	37.87	34.68
OsR498G0101553100.01	6.01	18.876	1.6565555	3.43E-166	1.20E-166	5.77	5.62	6.64	17.56	20.23	18.84

OsR498G0101557500.01	4.76	0.443	-3.899168	9.32E-153	3.45E-153	4.35	5.16	4.77	0.26	0.44	0.63
OsR498G0101557800.01	51.466	0.423	-6.939412	0	0	48.44	53.62	52.34	0.39	0.55	0.33
OsR498G0101558500.01	1.786	0.716	-1.43693	6.65E-13	9.40E-13	2	1.37	1.99	0.64	0.87	0.64
OsR498G0101558800.01	0.806	0	-6.84398	7.91E-15	1.07E-14	0.82	1.23	0.37	0	0	0
OsR498G0101559000.01	1.333	0.04	-5.010483	5.47E-24	6.09E-24	1.57	0.96	1.47	0.04	0.04	0.04
OsR498G0101561600.01	0.256	1.24	2.240304	6.69E-14	9.21E-14	0.29	0.14	0.34	1.14	1.39	1.19
OsR498G0101562000.01	29.566	123.23	2.0559042	0	0	30.4	30.11	28.19	124.89	126.29	118.52
OsR498G0101563100.01	12.103	37.616	1.6299575	9.94E-146	3.80E-146	12.95	11.63	11.73	38.32	37.1	37.43
OsR498G0101564900.01	42.75	88.606	1.0529076	0	0	44.89	42.72	40.64	89.07	89.15	87.6
OsR498G0101565900.01	10.346	24.61	1.2637905	2.66E-137	1.07E-137	10.28	10.37	10.39	24.67	23.85	25.31
OsR498G0101571300.01	43.946	113.09	1.354262	0	0	44.47	43.47	43.9	114.34	114.25	110.67
OsR498G0101573400.01	4.41	9.5	1.1059074	3.92E-72	2.42E-72	4.42	4.86	3.95	9.96	9.47	9.07
OsR498G0101576700.01	0.293	0.08	-1.900059	0.0007746	0.0015958	0.32	0.32	0.24	0.04	0.08	0.12
OsR498G0101577900.01	0.15	1.033	2.9192684	3.51E-14	4.80E-14	0.08	0.22	0.15	1.04	1.07	0.99
OsR498G0101580700.01	34.986	71.196	1.0369333	0	0	36.06	34.83	34.07	69.97	72.45	71.17
OsR498G0101581000.01	55.62	233.22	2.0889446	0	0	54.84	57.08	54.94	227.54	243.39	228.74
OsR498G0101581900.01	0	0.52	7.0135484	1.43E-16	1.85E-16	0	0	0	0.63	0.53	0.4
OsR498G0101582200.01	0.08	12.936	7.3122994	1.49E-285	3.29E-286	0.06	0.06	0.12	13.36	12.09	13.36
OsR498G0101582300.01	0.27	5.92	4.4603138	1.79E-134	7.34E-135	0.27	0.27	0.27	5.94	5.74	6.08
OsR498G0101582500.01	0.52	86.13	7.3780222	0	0	0.6	0.43	0.53	89.71	85.32	83.36
OsR498G0101585100.01	3.146	14.733	2.228935	2.57E-190	8.01E-191	3.82	2.39	3.23	15.46	14.96	13.78
OsR498G0101585300.01	2.066	52.773	4.6736429	0	0	2.2	1.81	2.19	52.22	53.67	52.43
OsR498G0101590100.01	0.043	0	-4.347518	0.0007014	0.0014405	0.04	0.05	0.04	0	0	0
OsR498G0101590900.01	0.223	0.093	-1.237406	0.0002203	0.0004357	0.18	0.21	0.28	0.07	0.05	0.16
OsR498G0101594500.01	0.346	0.673	1.0431227	4.31E-05	8.15E-05	0.28	0.39	0.37	0.52	0.7	0.8
OsR498G0101594900.01	0.336	15.056	5.4874795	1.44E-65	9.42E-66	0.34	0.17	0.5	14.27	15.68	15.22
OsR498G0101599100.01	1.873	7.376	1.9685809	6.41E-52	4.83E-52	1.82	2.05	1.75	7.12	7.58	7.43
OsR498G0101603100.01	108.5	28.68	-1.909405	0	0	114.41	104.47	106.62	28.61	28.86	28.57
OsR498G0101603500.01	0.98	2.363	1.2684012	2.03E-11	2.99E-11	0.93	1.04	0.97	2.48	2.39	2.22
OsR498G0101603800.01	4.683	14.21	1.4528425	3.53E-37	3.19E-37	4.49	3.63	5.93	13.34	14.39	14.9
OsR498G0101609600.01	1.203	8.056	2.7249585	3.12E-76	1.87E-76	1.61	0.79	1.21	7.84	8.51	7.82
OsR498G0101610600.01	1.27	6.92	2.3958737	3.42E-89	1.86E-89	1.18	1.2	1.43	6.51	8.19	6.06
OsR498G0101613100.01	0.53	1.663	1.6524819	1.89E-09	2.97E-09	0.53	0.58	0.48	1.7	1.59	1.7
OsR498G0101614100.01	0.253	31.876	6.9717024	0	0	0.12	0.18	0.46	29.57	32.52	33.54
OsR498G0101615700.01	5.026	0.84	-2.588526	5.83E-58	4.12E-58	5.05	4.85	5.18	0.87	0.68	0.97
OsR498G0101621700.01	4183.39	318.4	-3.730808	0	0	4209	4032	4309.2	311.65	307.59	335.95
OsR498G0101624900.01	10.413	2.163	-2.264881	1.19E-114	5.45E-115	10.7	10.21	10.33	2.1	2.65	1.74
OsR498G0101626900.01	0.126	0.616	2.2639166	4.30E-07	7.42E-07	0.17	0.17	0.04	0.71	0.5	0.64
OsR498G0101631500.01	0.046	0.39	3.144335	8.30E-05	0.00016	0.07	0	0.07	0.45	0.39	0.33
OsR498G0101632000.01	0.473	2.113	2.144335	4.68E-14	6.42E-14	0.48	0.53	0.41	1.75	1.73	2.86
OsR498G0101636800.01	4.113	10.296	1.3590738	2.45E-66	1.60E-66	4.22	4.64	3.48	10.1	10.34	10.45
OsR498G0101637100.01	2.073	93.403	5.4984015	0	0	2.23	1.72	2.27	100	88.41	91.8
OsR498G0101644400.01	0.346	0.913	1.3871012	1.12E-06	1.97E-06	0.28	0.38	0.38	1.09	0.84	0.81
OsR498G0101644900.01	0.063	0.8	3.7023304	1.06E-22	1.21E-22	0	0.14	0.05	0.63	0.91	0.86
OsR498G0101648200.01	2.413	0.346	-3.479968	2.90E-56	2.09E-56	2.07	2.68	2.49	0.61	0.25	0.18
OsR498G0101654400.01	0.606	1.68	1.4719096	3.01E-08	4.95E-08	0.7	0.53	0.59	1.93	1.4	1.71
OsR498G0101654900.01	1.55	5.733	1.8966288	5.35E-68	3.42E-68	1.72	1.28	1.65	6.28	5.52	5.4
OsR498G0101655100.01	15.583	32.9	1.0631559	3.49E-104	1.70E-104	15.53	15.7	15.52	31.21	33.63	33.86
OsR498G0101655500.01	6.873	31.003	2.1504014	1.17E-258	2.79E-259	6.48	6.78	7.36	30.74	29.17	33.1
OsR498G0101656100.01	0.503	0.18	-1.454433	0.0004899	0.0009933	0.68	0.55	0.28	0	0.29	0.25
OsR498G0101656300.01	0.72	0.176	-1.832945	0.0002564	0.0005097	0.95	0.74	0.47	0.26	0.13	0.14
OsR498G0101658300.01	3.736	8.9	1.1770717	2.48E-35	2.30E-35	4	3.48	3.73	10.84	7.47	8.39
OsR498G0101660900.01	0.186	0.643	1.8692277	4.38E-13	6.15E-13	0.19	0.23	0.14	0.63	0.52	0.78
OsR498G0101661500.01	1.63	0.2	-2.995216	1.40E-17	1.77E-17	1.79	1.35	1.75	0.09	0.32	0.19
OsR498G0101665700.01	44.346	19.033	-1.213311	3.11E-263	7.26E-264	44.88	44.99	43.17	18.98	19.51	18.61
OsR498G0101676300.01	8.863	2.033	-2.134037	1.54E-134	6.27E-135	8.59	8.86	9.14	1.97	2.05	2.08
OsR498G0101680500.01	0.096	0.616	2.7161168	2.27E-07	3.87E-07	0.27	0.02	0	0.81	0.36	0.68
OsR498G0101685000.01	2.433	6.73	1.4643759	1.35E-14	1.83E-14	2.8	1.5	3	5.87	6.27	8.05
OsR498G0101685700.01	12.476	35.78	1.5253795	0	0	12.13	13.32	11.98	36.57	36	34.77
OsR498G0101686000.01	4.406	12.416	1.4999252	1.42E-120	6.23E-121	4.72	4.54	3.96	13.3	13.27	10.68
OsR498G0101690800.01	8.526	43.603	2.3397405	0	0	4.99	19.07	1.52	45.13	47.64	38.04
OsR498G0101691600.01	2.923	34.726	3.5633414	0	0	2.64	2.75	3.38	35.38	33.28	35.52
OsR498G0101694200.01	0.063	0.553	3.144335	5.64E-08	9.36E-08	0.09	0.05	0.05	0.27	0.59	0.8
OsR498G0101694700.01	10.136	26.056	1.3102154	2.04E-78	1.20E-78	9.33	10.32	10.76	29.79	22.68	25.7
OsR498G0101697900.01	1.356	3.993	1.5463163	8.91E-18	1.12E-17	1.49	1.04	1.54	3.63	4.78	3.57
OsR498G0101698500.01	6.8	0.81	-3.073623	6.64E-70	4.18E-70	6.61	6.65	7.14	0.85	0.7	0.88
OsR498G0101702400.01	31.326	2.536	-3.626742	3.13E-231	8.30E-232	31.69	31.45	30.84	2.76	2.51	2.34
OsR498G0101706400.01	0.38	0.143	-1.410254	9.96E-05	0.0001931	0.39	0.31	0.44	0.09	0.17	0.17
OsR498G0101708600.01	0.03	0.366	3.5593725	5.57E-11	8.35E-11	0.07	0.02	0	0.41	0.41	0.28

OsR498G0101710100.01	1.816	0.793	-1.200364	2.94E-09	4.66E-09	1.91	2	1.54	0.64	0.74	1
OsR498G0101711000.01	0.726	4.403	2.5989008	1.25E-59	8.69E-60	0.83	0.46	0.89	4.18	4.24	4.79
OsR498G0101713800.01	0.74	2.106	1.5078422	1.43E-05	2.65E-05	0.59	0.58	1.05	1.72	2.73	1.87
OsR498G0101714600.01	0.13	0.623	2.2639166	4.30E-07	7.42E-07	0.13	0.17	0.09	0.46	0.76	0.65
OsR498G0101715400.01	5.446	12.19	1.1633642	8.65E-36	7.97E-36	4.59	5.03	6.72	11.75	12.13	12.69
OsR498G0101715500.01	567.706	251.33	-1.176615	0	0	568.82	573.09	561.21	250.15	249.37	254.47
OsR498G0101717400.01	14.09	50.666	1.845523	1.66E-214	4.65E-215	14.18	12.92	15.17	50.93	49.54	51.53
OsR498G0101720100.01	11.63	26.883	1.1995296	3.42E-71	2.13E-71	13.12	10.18	11.59	24.45	26.74	29.46
OsR498G0101721200.01	5.39	11.1	1.0286239	3.08E-38	2.74E-38	5.26	5.7	5.21	10.16	10.43	12.71
OsR498G0101722000.01	4.773	21.123	2.144335	8.61E-114	3.97E-114	4.91	4.41	5	20.2	20.94	22.23
OsR498G0101724900.01	0.366	0.233	-1.189089	0.0001846	0.0003638	0.43	0.34	0.33	0.15	0.07	0.48
OsR498G0101727800.01	1.02	0.243	-2.07157	2.10E-18	2.60E-18	1.11	1.09	0.86	0.28	0.18	0.27
OsR498G0101730000.01	0.236	1.006	2.0862843	2.58E-18	3.20E-18	0.22	0.31	0.18	1.03	0.9	1.09
OsR498G0101730500.01	0.79	2.253	1.4807626	2.68E-10	4.10E-10	0.98	0.9	0.49	2.42	2.38	1.96
OsR498G0101735200.01	0.07	0.413	2.5593725	0.0003356	0.0006725	0	0	0.21	0.69	0.27	0.28
OsR498G0101735400.01	0.126	0.596	2.2545179	5.40E-10	8.35E-10	0.16	0.11	0.11	0.6	0.57	0.62
OsR498G0101736500.01	54.253	110.16	1.0220604	0	0	55.5	53.98	53.28	112.87	110.65	106.96
OsR498G0101741200.01	26.573	115.89	2.1359437	0	0	27.33	25.75	26.64	119.26	117.43	110.98
OsR498G0101745200.01	4.423	13.57	1.6154065	5.56E-99	2.81E-99	4.23	4.41	4.63	12.85	13.75	14.11
OsR498G0101746700.01	9.496	92.703	3.2749945	0	0	8.67	9.42	10.4	94.2	90.24	93.67
OsR498G0101747500.01	2.77	8.323	1.5772291	9.32E-105	4.52E-105	2.65	2.93	2.73	8.12	8.71	8.14
OsR498G0101751200.01	1.586	5.013	1.6548386	1.76E-28	1.82E-28	1.55	1.66	1.55	5.3	4.6	5.14
OsR498G0101752900.01	7.73	1.32	-2.547359	1.15E-58	8.06E-59	8.41	7.12	7.66	0.73	1.96	1.27
OsR498G0101758800.01	4.743	1.65	-1.521689	1.75E-58	1.23E-58	4.84	4.62	4.77	1.51	1.84	1.6
OsR498G0101760500.01	76.753	198.99	1.35589	0	0	79.94	74.35	75.97	201.61	195.01	200.35
OsR498G0101763800.01	31.8	100.55	1.6603361	3.71E-291	8.03E-292	31.8	33.52	30.08	95.47	105.2	100.97
OsR498G0101769600.01	1.03	0.45	-1.179375	6.83E-07	1.19E-06	1.19	1.08	0.82	0.57	0.51	0.27
OsR498G0101770000.01	1.16	2.906	1.3228103	1.36E-17	1.72E-17	1.05	1.13	1.3	2.84	3.04	2.84
OsR498G0101770300.01	0.053	0.36	2.7399447	7.12E-07	1.24E-06	0.02	0.14	0	0.54	0.28	0.26
OsR498G0101770600.01	2.276	15.853	2.7900682	0	0	2.14	2.47	2.22	16.15	15.5	15.91
OsR498G0101771700.01	153.37	52.196	-1.55458	0	0	150.04	154.56	155.51	52.74	54.71	49.14
OsR498G0101772000.01	5.26	1.73	-1.610553	3.75E-16	4.89E-16	6.58	4.47	4.73	1.94	1.09	2.16
OsR498G0101772400.01	3.613	8.166	1.2149632	1.14E-160	4.09E-161	3.88	3.59	3.37	8.3	8.46	7.74
OsR498G0101774600.01	3.916	10.233	1.385818	2.10E-99	1.06E-99	3.92	4.03	3.8	10.76	9.95	9.99
OsR498G0101778900.01	1.136	8.47	2.8958979	6.43E-73	3.96E-73	1.22	1.38	0.81	9.08	7.81	8.52
OsR498G0101781600.01	6.053	13.52	1.160024	1.28E-68	8.13E-69	6.08	6.34	5.74	13.63	13.26	13.67
OsR498G0101782000.01	0.176	0	-4.347518	0.0007014	0.0014405	0.2	0.14	0.19	0	0	0
OsR498G0101785700.01	0.48	0.086	-2.473169	9.19E-11	1.39E-10	0.47	0.44	0.53	0.11	0.13	0.02
OsR498G0101787300.01	0.526	0.18	-1.549152	4.05E-05	7.66E-05	0.51	0.51	0.56	0.2	0.1	0.24
OsR498G0101788000.01	12.693	26.463	1.0614059	9.82E-80	5.74E-80	13.74	11.32	13.02	27.87	26.26	25.26
OsR498G0101788300.01	9.313	37.703	2.0135216	1.41E-282	3.14E-283	9.61	8.59	9.74	38.04	35.14	39.93
OsR498G0101791800.01	3.16	1.206	-1.489994	4.66E-17	5.95E-17	3.28	3.17	3.03	1.33	1.05	1.24
OsR498G0101800300.01	1.863	7.53	1.9863469	1.69E-76	1.01E-76	1.81	1.78	2	6.85	8.22	7.52
OsR498G0101803200.01	0.47	7.22	3.9169245	3.44E-77	2.05E-77	0.53	0.18	0.7	7.47	6.91	7.28
OsR498G0101803400.01	0.553	8.57	3.9562626	5.23E-83	2.99E-83	0.67	0.26	0.73	7.88	8.94	8.89
OsR498G0101806500.01	7.336	71.436	3.3169521	0	0	7.31	8.16	6.54	72.33	72.76	69.22
OsR498G0101808700.01	1.763	15.536	3.1382709	1.90E-225	5.12E-226	1.3	1.77	2.22	15.52	14.97	16.12
OsR498G0101817300.01	4.216	9.786	1.2558971	5.38E-65	3.55E-65	4.22	4.09	4.34	9.39	10.04	9.93
OsR498G0101819100.01	72.23	9.94	-2.828819	0	0	73.59	73.94	69.16	9.87	10.44	9.51
OsR498G0101819800.01	6.803	120.74	4.1453684	0	0	6.72	6.97	6.72	123.41	124.57	114.25
OsR498G0101820100.01	0	0.203	4.4338416	0.0004331	0.0008746	0	0	0	0.11	0.22	0.28
OsR498G0101820800.01	6.266	1.543	-2.133312	8.50E-40	7.42E-40	6.89	5.69	6.22	1.85	0.86	1.92
OsR498G0101823100.01	3.123	14.393	2.2379625	1.17E-134	4.79E-135	3.36	2.96	3.05	13.97	13.31	15.9
OsR498G0101823600.01	0.353	0.763	1.0964005	7.35E-05	0.0001412	0.25	0.34	0.47	0.95	0.49	0.85
OsR498G0101831600.01	0.343	2.036	2.5697891	2.36E-21	2.77E-21	0.27	0.4	0.36	2.22	1.87	2.02
OsR498G0101832600.01	0.09	0.986	3.4555366	2.46E-14	3.35E-14	0.05	0.09	0.13	1.01	1.14	0.81
OsR498G0101838400.01	4.113	11.416	1.4598368	3.62E-104	1.77E-104	3.92	4.21	4.21	11.94	11.35	10.96
OsR498G0101838600.01	3.983	22.62	2.4915003	1.63E-212	4.65E-213	4.38	3.68	3.89	23.57	22.31	21.98
OsR498G0101844100.01	422.08	164.7	-1.358791	0	0	430.07	428.24	407.93	165.5	164.36	164.24
OsR498G0101845100.01	8.673	1.753	-2.307906	1.66E-119	7.36E-120	8.55	8.7	8.77	1.57	1.73	1.96
OsR498G0101846200.01	2.06	0.366	-2.489246	2.04E-25	2.22E-25	2.61	1.87	1.7	0.21	0.56	0.33
OsR498G0101847400.01	0	2.396	6.0188041	1.75E-09	2.75E-09	0	0	0	1.52	2.59	3.08
OsR498G0101849200.01	0.27	4.293	4.0018907	9.58E-46	7.72E-46	0.25	0.25	0.31	4.04	4.32	4.52
OsR498G0101850000.01	17.14	46.083	1.3608049	1.19E-170	4.05E-171	17.04	18.04	16.34	44.33	43.54	50.38
OsR498G0101853300.01	0.97	2.703	1.4785414	7.53E-22	8.72E-22	0.76	0.95	1.2	2.51	2.66	2.94
OsR498G0101856100.01	10.273	34.656	1.7581698	0	0	10.86	10.12	9.84	35.32	35.69	32.96
OsR498G0101856600.01	7.67	18.603	1.356747	2.51E-157	9.11E-158	8.12	7.36	7.53	18.13	19.14	18.54
OsR498G0101856700.01	0.033	0.48	3.8611227	2.56E-05	4.79E-05	0.1	0	0	0.28	0.71	0.45
OsR498G0101857000.01	0.756	3.086	2.0294078	9.84E-20	1.19E-19	0.7	0.78	0.79	3.4	2.79	3.07

OsR498G0101864100.01	3.756	23.633	2.5941184	1.29E-257	3.09E-258	4.13	3.33	3.81	21.76	23.61	25.53
OsR498G0101865600.01	147.553	40.68	-1.824562	0	0	147.85	147.84	146.97	41.41	41.54	39.09
OsR498G0101867800.01	2.59	1.06	-1.294079	8.95E-14	1.24E-13	2.8	2.44	2.53	0.93	1	1.25
OsR498G0101868400.01	3.23	6.34	1.0204433	1.22E-22	1.39E-22	2.98	3.44	3.27	6.51	6.7	5.81
OsR498G0101869400.01	17.076	4.086	-2.117658	1.46E-179	4.79E-180	16.88	17.76	16.59	3.56	3.85	4.85
OsR498G0101877100.01	2.18	0.113	-4.230704	5.60E-38	4.99E-38	2.18	1.92	2.44	0.15	0.15	0.04
OsR498G0101880600.01	2.326	37.65	4.1410298	0	0	2.02	2.44	2.52	36.97	38.69	37.29
OsR498G0101881000.01	0	0.883	5.8813006	8.71E-09	1.40E-08	0	0	0	1.23	0.7	0.72
OsR498G0101882900.01	2.423	9.01	1.6490097	1.34E-54	9.77E-55	2.07	2.36	2.84	8.2	9.54	9.29
OsR498G0101884600.01	0.026	0.613	4.4338416	1.91E-11	2.81E-11	0	0.04	0.04	0.66	0.5	0.68
OsR498G0101885900.01	0.04	0.4	3.3436438	2.11E-07	3.59E-07	0	0.04	0.08	0.19	0.38	0.63
OsR498G0101886400.01	0.046	0.193	2.0447993	0.0006522	0.0013355	0	0.11	0.03	0.14	0.16	0.28
OsR498G0101896900.01	14.706	0.12	-6.967465	0	0	13.77	15.15	15.2	0.16	0.13	0.07
OsR498G0101902900.01	0.603	4.3	2.832391	2.25E-15	2.99E-15	0.76	0.6	0.45	4.72	4.41	3.77
OsR498G0101903500.01	0.933	7.1	2.9290494	1.12E-132	4.64E-133	1.15	0.82	0.83	7.63	6.38	7.29
OsR498G0101906500.01	0.756	1.926	1.3362768	6.86E-19	8.41E-19	0.84	0.66	0.77	1.78	1.8	2.2
OsR498G0101909600.01	26.666	3.466	-2.95019	0	0	27.11	25.89	27	3.4	3.56	3.44
OsR498G0101911900.01	0.996	2.94	1.5593725	1.79E-15	2.37E-15	1.21	1.01	0.77	3.15	2.15	3.52
OsR498G0101914200.01	8.58	19.853	1.2127471	3.47E-85	1.94E-85	8.09	8.5	9.15	18.81	19.01	21.74
OsR498G0101917800.01	2.796	15.423	2.4568027	1.94E-90	1.04E-90	2.41	2.74	3.24	15.69	14.34	16.24
OsR498G0101919300.01	0.403	0.133	-1.559022	7.65E-06	1.40E-05	0.44	0.37	0.4	0.13	0.14	0.13
OsR498G0101919800.01	0.926	2.97	1.6795755	5.96E-58	4.21E-58	0.9	1.04	0.84	2.85	3.07	2.99
OsR498G0101922300.01	2.526	5.04	1.0062857	3.92E-50	3.01E-50	2.44	2.6	2.54	5.16	4.91	5.05
OsR498G0101922900.01	0.036	1.08	4.9286063	7.45E-09	1.20E-08	0	0	0.11	0.93	1.14	1.17
OsR498G0101925700.01	0.216	3.403	3.577751	3.77E-32	3.66E-32	0.29	0.24	0.12	3.14	3.47	3.6
OsR498G0101926800.01	25.786	10.78	-1.261399	1.38E-121	6.03E-122	24.84	26.67	25.85	8.82	10.96	12.56
OsR498G0101929800.01	0.12	1.993	4.0641074	3.75E-53	2.79E-53	0.12	0.17	0.07	1.94	1.9	2.14
OsR498G0101931000.01	3.706	1.593	-1.222432	9.67E-30	9.77E-30	3.91	3.74	3.47	1.58	1.26	1.94
OsR498G0101931300.01	0.933	1.903	1.0462006	8.45E-06	1.55E-05	0.94	1.19	0.67	2.59	1.53	1.59
OsR498G0101932000.01	0.086	3.303	5.2963381	3.76E-122	1.64E-122	0.02	0.11	0.13	3.41	3.19	3.31
OsR498G0101932200.01	9.086	80.186	3.1389382	0	0	8.5	9.5	9.26	80.79	81.37	78.4
OsR498G0101936400.01	1.24	2.733	1.1429157	2.20E-13	3.07E-13	1.52	1.21	0.99	2.85	2.65	2.7
OsR498G0101937700.01	126.626	19.073	-2.729735	0	0	124.98	126.88	128.02	19.21	19.84	18.17
OsR498G0101938600.01	0.036	1.61	5.4338416	1.54E-32	1.49E-32	0.04	0	0.07	1.59	1.3	1.94
OsR498G0101939600.01	0.97	11.61	3.5816655	0	0	0.88	1.06	0.97	11.55	12.39	10.89
OsR498G0101941000.01	0.213	1.533	2.8268528	6.52E-12	9.48E-12	0.29	0.21	0.14	1.47	1.33	1.8
OsR498G0101941400.01	0.723	0.373	-1.105025	1.14E-06	1.99E-06	0.57	0.54	1.06	0.33	0.54	0.25
OsR498G0101942100.01	12.516	32.693	1.3849878	2.11E-160	7.58E-161	12.54	12.85	12.16	33.39	32.17	32.52
OsR498G0101942300.01	0	0.373	5.6748497	7.66E-08	1.28E-07	0	0	0	0.21	0.34	0.57
OsR498G0101946900.01	1.17	0.196	-2.569911	7.76E-17	9.96E-17	1.04	1.2	1.27	0.2	0.22	0.17
OsR498G0101947500.01	0.053	5.503	6.6794663	3.42E-73	2.10E-73	0	0	0.16	5.18	5.42	5.91
OsR498G0101949900.01	49.376	22.49	-1.137543	1.48E-214	4.17E-215	49.38	50.47	48.28	23.51	21.61	22.35
OsR498G0101950700.01	12.726	158.79	3.6399791	0	0	12.25	12.33	13.6	154.09	156.32	165.97
OsR498G0101951500.01	2.913	17.663	2.5995296	6.50E-219	1.79E-219	2.73	2.73	3.28	17.45	17.59	17.95
OsR498G0101953400.01	3.44	16.62	2.2722664	7.55E-168	2.61E-168	3.21	3.88	3.23	16.75	15.52	17.59
OsR498G0101953800.01	2.086	7.163	1.7772775	1.63E-58	1.14E-58	2.36	2.17	1.73	6.77	6.74	7.98
OsR498G0101954500.01	0.163	1.95	3.5743228	1.95E-20	2.32E-20	0.25	0.24	0	1.92	2.15	1.78
OsR498G0101957100.01	1.52	3.896	1.4055257	2.01E-26	2.15E-26	1.48	1.66	1.42	3.91	3.94	3.84
OsR498G0101957300.01	1.913	4.606	1.2483327	9.81E-23	1.12E-22	2.01	1.84	1.89	4.27	4.55	5
OsR498G0101957700.01	0.33	0	-4.347518	0.0007014	0.0014405	0.4	0.39	0.2	0	0	0
OsR498G0101958100.01	1.946	7.566	1.9488533	2.81E-119	1.25E-119	2.26	1.59	1.99	6.98	8.18	7.54
OsR498G0101960700.01	3.786	9.593	1.3331141	3.92E-84	2.22E-84	3.41	3.84	4.11	9.97	9.91	8.9
OsR498G0101962600.01	6.08	13.243	1.1194224	3.12E-66	2.04E-66	6.72	5.53	5.99	14.06	11.97	13.7
OsR498G0101964800.01	70.986	23.463	-1.597836	2.34E-259	5.55E-260	68.39	75.38	69.19	22.26	24.99	23.14
OsR498G0101968200.01	2.916	30.546	3.4075623	0	0	2.73	3.17	2.85	32.21	29.35	30.08
OsR498G0101968800.01	8.273	27.823	1.7803583	0	0	8.2	8.63	7.99	27.5	27.7	28.27
OsR498G0101971900.01	3.44	14.55	2.1864604	9.29E-147	3.53E-147	3.21	3.23	3.88	15.6	14.01	14.04
OsR498G0101972400.01	0.72	4.88	2.7569437	1.48E-71	9.18E-72	0.74	0.61	0.81	5.04	4.49	5.11
OsR498G0101972900.01	0.186	0.786	2.1009424	2.73E-16	3.54E-16	0.25	0.11	0.2	0.95	0.77	0.64
OsR498G0101973900.01	4.086	46.766	3.5170778	0	0	4.3	3.82	4.14	46.58	47.31	46.41
OsR498G0101977300.01	0.046	0.33	2.8113701	2.55E-07	4.36E-07	0.08	0	0.06	0.38	0.22	0.39
OsR498G0101978300.01	24.313	8.17	-1.516611	6.09E-136	2.46E-136	25.47	24.58	22.89	7.36	8.44	8.71
OsR498G0101978600.01	1.06	0.41	-1.384671	2.04E-05	3.80E-05	0.7	0.97	1.51	0.37	0.16	0.7
OsR498G0101986700.01	3.936	14.276	1.828916	1.78E-87	9.80E-88	4.33	3.5	3.98	15.09	14.09	13.65
OsR498G0101987200.01	23.523	8.243	-1.512905	1.88E-263	4.38E-264	23.54	23.55	23.48	8.54	7.8	8.39
OsR498G0101989600.01	0.2	0.5	1.3258823	5.37E-06	9.73E-06	0.21	0.25	0.14	0.36	0.64	0.5
OsR498G0101990600.01	0.8	2.056	1.3656007	1.95E-11	2.88E-11	0.87	0.9	0.63	1.84	2.4	1.93
OsR498G0101992300.01	147.26	22.5	-2.71126	0	0	143.18	149.69	148.91	22.07	23.84	21.59
OsR498G0101993100.01	10.58	28.826	1.5218645	7.17E-238	1.86E-238	9.83	11.19	10.72	27.35	30.59	28.54

OsR498G0101994100.01	53.12	160.3	1.5965654	0	0	54.14	52.46	52.76	164.98	161.57	154.34
OsR498G0101994300.01	12.713	27.436	1.1094309	1.36E-122	5.93E-123	12.73	13.72	11.69	28.05	27.81	26.45
OsR498G0101997600.01	5.68	21.193	1.8989262	9.21E-171	3.13E-171	5.73	5.66	5.65	21.05	21.14	21.39
OsR498G0101999600.01	0.083	0.293	1.7520175	0.0009565	0.0019907	0.07	0.07	0.11	0.4	0.18	0.3
OsR498G0102006800.01	5.273	15.736	1.5508555	2.46E-122	1.07E-122	5.17	5.4	5.25	15.03	16.19	15.99
OsR498G0102007200.01	12.403	74.186	2.5787267	0	0	12.25	13.17	11.79	76.66	71.25	74.65
OsR498G0102008000.01	1.753	5.013	1.5065206	7.89E-86	4.40E-86	1.9	1.69	1.67	4.7	4.45	5.89
OsR498G0102008100.01	2.53	8.33	1.7196908	1.36E-99	6.85E-100	2.54	2.43	2.62	8.45	8.8	7.74
OsR498G0102009100.01	20.5	102.99	2.3324439	0	0	20.54	19.94	21.02	103.1	104.7	101.18
OsR498G0102009300.01	0.593	1.706	1.526951	7.72E-05	0.0001486	0.24	0.83	0.71	1.5	1.73	1.89
OsR498G0102009600.01	0.48	1.803	1.9178264	1.31E-11	1.92E-11	0.61	0.44	0.39	1.77	1.87	1.77
OsR498G0102010200.01	13	4.84	-1.422717	8.77E-134	3.60E-134	12.67	13.03	13.3	4.63	5.26	4.63
OsR498G0102011100.01	0.11	0.856	3.0188041	3.09E-07	5.29E-07	0.1	0.06	0.17	0.71	1.17	0.69
OsR498G0102012800.01	0.103	0.386	1.8949755	7.18E-07	1.25E-06	0.11	0.09	0.11	0.28	0.48	0.4
OsR498G0102021500.01	0.01	5.02	8.8009584	5.84E-88	3.21E-88	0	0	0.03	4.91	5.41	4.74
OsR498G0102025000.01	2.73	11.296	2.0462124	2.16E-87	1.19E-87	3	2.39	2.8	10.91	11.65	11.33
OsR498G0102025100.01	0.863	2.066	1.2617507	1.11E-16	1.44E-16	1	0.87	0.72	1.94	2.34	1.92
OsR498G0102030600.01	0.45	0.106	-2.503637	3.55E-07	6.10E-07	0.49	0.45	0.41	0.07	0.06	0.19
OsR498G0102038700.01	0.693	1.93	1.4637948	1.82E-16	2.36E-16	0.76	0.67	0.65	1.87	2.19	1.73
OsR498G0102040900.01	966.89	295.16	-1.702973	0	0	960.92	961.26	978.49	295.69	290.44	299.35
OsR498G0102044500.01	0.443	0.17	-1.330445	0.0003731	0.0007493	0.51	0.6	0.22	0.18	0.24	0.09
OsR498G0102045200.01	2.426	11.29	2.207333	2.36E-66	1.54E-66	1.51	3.35	2.42	10.97	11.17	11.73
OsR498G0102045300.01	0.413	2.663	2.7185711	7.81E-22	9.05E-22	0.44	0.31	0.49	2.72	2.79	2.48
OsR498G0102046000.01	5.116	1.35	-1.902156	4.26E-47	3.38E-47	5.1	5.14	5.11	1.34	1.26	1.45
OsR498G0102049700.01	2.66	7.95	1.5820925	1.54E-54	1.13E-54	2.75	2.53	2.7	7.51	7.97	8.37
OsR498G0102050400.01	99.233	29.2	-1.82658	0	0	99.4	102.08	96.22	29.33	27.93	30.34
OsR498G0102050900.01	1.106	2.843	1.4254894	1.43E-15	1.89E-15	1.14	0.99	1.19	3.04	2.97	2.52
OsR498G0102051900.01	9.776	62.71	2.6951368	0	0	10.01	9.2	10.12	60.6	60.16	67.37
OsR498G0102052400.01	0.353	3.803	3.4064478	1.49E-59	1.04E-59	0.5	0.11	0.45	3.05	4.56	3.8
OsR498G0102053200.01	0.036	0.686	4.1823681	4.27E-13	6.00E-13	0	0.11	0	0.89	0.42	0.75
OsR498G0102054200.01	7.823	36.09	2.2060547	4.92E-301	1.04E-301	7.47	7.24	8.76	36.19	35.48	36.6
OsR498G0102055700.01	0.176	0.95	2.5289988	2.92E-08	4.80E-08	0.19	0.22	0.12	0.96	0.9	0.99
OsR498G0102058600.01	6.756	17.253	1.4225741	1.90E-145	7.29E-146	6.01	7.21	7.05	16.7	17.33	17.73
OsR498G0102060600.01	0.15	0.6	1.97441	5.68E-05	0.0001084	0.11	0.17	0.17	0.5	0.61	0.69
OsR498G0102061700.01	0.82	1.933	1.3260587	2.84E-34	2.67E-34	0.95	0.73	0.78	1.87	2.31	1.62
OsR498G0102064700.01	1.746	23.363	3.7719418	0	0	1.96	1.33	1.95	22.92	24.59	22.58
OsR498G0102065000.01	31.973	3.37	-3.241754	0	0	32.4	32.67	30.85	3.47	3.37	3.27
OsR498G0102065600.01	1.276	0.01	-6.979088	3.56E-30	3.58E-30	1.16	1.34	1.33	0	0	0.03
OsR498G0102066700.01	5.01	12.823	1.3547092	6.13E-33	5.89E-33	5.13	4.66	5.24	12.47	12.79	13.21
OsR498G0102067000.01	1.256	2.91	1.2063924	2.33E-07	3.97E-07	0.84	0.98	1.95	2.7	2.52	3.51
OsR498G0102067800.01	0.246	2.823	3.506905	1.93E-32	1.87E-32	0.16	0.21	0.37	2.59	2.63	3.25
OsR498G0102068600.01	0.416	1.01	1.2739702	3.54E-05	6.66E-05	0.34	0.38	0.53	1.13	0.98	0.92
OsR498G0102068700.01	17.15	48.623	1.5022702	6.01E-165	2.12E-165	17.74	16.08	17.63	48.54	46.93	50.4
OsR498G0102072200.01	18.54	37.45	1.0192085	2.08E-94	1.09E-94	16.75	19.54	19.33	37.9	35.85	38.6
OsR498G0102072700.01	3.556	16.023	2.1925529	8.05E-94	4.22E-94	3.63	3.17	3.87	16.13	16.42	15.52
OsR498G0102074300.01	19.81	43.016	1.119168	2.73E-210	7.83E-211	19.66	20.55	19.22	43.46	43.47	42.12
OsR498G0102074800.01	0	0.483	6.103693	6.09E-10	9.42E-10	0	0	0	0.27	0.71	0.47
OsR498G0102075000.01	0.183	1.833	3.696876	1.09E-53	8.04E-54	0.25	0.3	0	2.07	1.64	1.79
OsR498G0102075300.01	1.583	16.513	3.3778404	0	0	1.61	1.48	1.66	16.59	16.74	16.21
OsR498G0102075600.01	3.613	7.286	1.0026871	5.57E-35	5.19E-35	3.61	3.5	3.73	8.01	7.19	6.66
OsR498G0102077400.01	2214.22	642.26	-1.787451	0	0	2232.1	2202.5	2208.1	624.6	618.35	683.82
OsR498G0102078100.01	0	2.323	7.857053	1.27E-26	1.36E-26	0	0	0	2.52	1.98	2.47
OsR498G0102083800.01	0.033	0.276	3.144335	2.13E-06	3.77E-06	0.04	0.03	0.03	0.33	0.23	0.27
OsR498G0102085800.01	22.953	46.146	1.0071999	3.20E-107	1.53E-107	23.8	22.47	22.59	43.15	48.24	47.05
OsR498G0102088400.01	2.376	6.433	1.5023415	2.81E-36	2.56E-36	2	2.45	2.68	6.89	6.01	6.4
OsR498G0102088500.01	0.016	0.683	5.8813006	7.87E-16	1.03E-15	0	0.05	0	0.43	0.96	0.66
OsR498G0102089000.01	2.55	11.186	2.1431729	4.68E-142	1.83E-142	2.73	2.43	2.49	11.64	11.34	10.58
OsR498G0102091500.01	0.793	2.473	1.6433348	3.08E-24	3.42E-24	0.54	0.88	0.96	2.32	2.58	2.52
OsR498G0102091900.01	10.846	37.156	1.7748607	3.81E-121	1.67E-121	10.17	11.35	11.02	34.47	38.31	38.69
OsR498G0102095500.01	23.5	56.563	1.2608264	0	0	23.29	23.41	23.8	56.92	56.52	56.25
OsR498G0102095700.01	3.87	53.986	3.8032449	0	0	4.11	3.67	3.83	54.23	54.95	52.78
OsR498G0102096000.01	10.7	0.38	-4.821311	1.17E-68	7.47E-69	11.24	10.56	10.3	0.45	0.37	0.32
OsR498G0102096500.01	0.07	0.32	2.2966003	1.46E-08	2.36E-08	0.06	0.09	0.06	0.38	0.33	0.25
OsR498G0102097100.01	0.53	1.786	1.7475246	1.31E-21	1.53E-21	0.48	0.33	0.78	1.9	1.62	1.84
OsR498G0102099700.01	19.516	45.62	1.2154181	2.51E-223	6.82E-224	19.03	19.92	19.6	45.83	47.37	43.66
OsR498G0102100200.01	3.73	16.313	2.13592	8.89E-176	2.96E-176	3.7	4.26	3.23	17.25	15.56	16.13
OsR498G0102101700.01	13.81	27.986	1.0167829	8.08E-54	5.95E-54	13.47	13.16	14.8	26.63	26.4	30.93
OsR498G0102101900.01	0.273	1.983	2.8607532	3.38E-40	2.93E-40	0.3	0.25	0.27	1.86	2	2.09
OsR498G0102102500.01	0	1.236	4.4338416	0.0004331	0.0008746	0	0	0	1.35	1.68	0.68

OsR498G0102106700.01	3.003	1.21	-1.312309	1.57E-27	1.65E-27	3.08	3.36	2.57	1.24	1.18	1.21
OsR498G0102107500.01	11.286	37.96	1.4969203	2.25E-276	5.07E-277	12.87	10.55	10.44	38.2	38.71	36.97
OsR498G0102108100.01	4.036	10.18	1.3290267	3.53E-30	3.55E-30	4.5	4.15	3.46	9.42	11.33	9.79
OsR498G0102109300.01	0.09	0.4	2.1838633	4.98E-06	9.00E-06	0.1	0.07	0.1	0.55	0.32	0.33
OsR498G0102109500.01	0.75	3.303	2.1170802	1.04E-30	1.04E-30	0.74	0.61	0.9	3.11	3.23	3.57
OsR498G0102109700.01	2.306	16.7	2.7798101	0	0	2.24	2.46	2.22	16.05	18.57	15.48
OsR498G0102110200.01	13.303	4.623	-1.536951	7.69E-147	2.92E-147	13.61	13.01	13.29	4.16	5.16	4.55
OsR498G0102111100.01	92.966	397.4	2.0739373	0	0	95.57	91.93	91.4	398.33	390.13	403.75
OsR498G0102115700.01	0.696	3.9	2.5104629	2.38E-53	1.76E-53	0.49	0.61	0.99	3.68	4.11	3.91
OsR498G0102119400.01	9.536	2.86	-1.728389	5.97E-68	3.83E-68	9.43	10	9.18	3.38	2.85	2.35
OsR498G0102120400.01	0.723	10.22	3.8005347	4.58E-172	1.55E-172	0.62	0.61	0.94	10.4	9.74	10.52
OsR498G0102121700.01	5.636	11.103	1.0097582	4.71E-31	4.67E-31	5.29	5.86	5.76	11.94	11.1	10.27
OsR498G0102122700.01	0.323	0.826	1.3463787	0.00028	0.0005582	0.17	0.57	0.23	0.56	1	0.92
OsR498G0102124700.01	1.003	2.096	1.0541371	1.93E-05	3.59E-05	0.69	1.23	1.09	2.01	1.53	2.75
OsR498G0102125500.01	0.316	1.69	2.4535778	2.35E-26	2.52E-26	0.34	0.22	0.39	1.77	1.38	1.92
OsR498G0102125800.01	1.196	3.6	1.5780182	2.61E-48	2.05E-48	1.14	1.27	1.18	3.31	3.91	3.58
OsR498G0102127000.01	1.963	9.583	2.2825323	1.09E-52	8.12E-53	1.55	1.75	2.59	9.8	8.33	10.62
OsR498G0102127400.01	0.673	1.883	1.479938	1.29E-12	1.84E-12	0.68	0.73	0.61	2.05	1.38	2.22
OsR498G0102128300.01	0.596	1.663	1.4637948	1.45E-11	2.13E-11	0.44	0.84	0.51	1.68	1.61	1.7
OsR498G0102129600.01	18.45	56.116	1.544462	0	0	18.03	18.96	18.36	57.12	58.2	53.03
OsR498G0102129800.01	0	2.596	7.5891198	6.58E-23	7.46E-23	0	0	0	2.69	2.75	2.35
OsR498G0102130200.01	5.343	65.373	3.6114243	0	0	5.54	4.64	5.85	65.22	63.27	67.63
OsR498G0102130600.01	0.83	18.023	4.4459346	5.77E-133	2.38E-133	1.02	1.01	0.46	17.94	19.3	16.83
OsR498G0102131700.01	0	0.33	5.4338416	7.08E-07	1.23E-06	0	0	0	0.18	0.35	0.46
OsR498G0102133700.01	0.13	2.39	4.149042	9.45E-67	6.13E-67	0.14	0.16	0.09	2.24	2.69	2.24
OsR498G0102134700.01	1.606	22.653	3.8030607	0	0	1.27	1.7	1.85	21.87	23.6	22.49
OsR498G0102135900.01	1.606	4.046	1.3348122	2.76E-07	4.71E-07	2.35	1.3	1.17	3.94	4.43	3.77
OsR498G0102137100.01	0.393	2.056	2.4006747	2.36E-07	4.03E-07	0.88	0.15	0.15	2.43	1.99	1.75
OsR498G0102137700.01	0.97	20.18	4.331962	3.17E-108	1.51E-108	0.94	1.21	0.76	19.01	17.68	23.85
OsR498G0102139500.01	25.37	54.733	1.0869057	3.84E-157	1.40E-157	24.83	26.12	25.16	57.06	52.98	54.16
OsR498G0102148800.01	5.243	20.966	2.1027159	2.65E-205	7.80E-206	4.98	5.24	5.51	19.44	19.92	23.54
OsR498G0102148900.01	0.22	0.66	1.5862892	1.83E-07	3.10E-07	0.12	0.4	0.14	0.69	0.53	0.76
OsR498G0102149100.01	28.706	96.8	1.8039987	0	0	29.75	27.03	29.34	97.47	96.02	96.91
OsR498G0102151700.01	0.553	2.966	2.5218978	1.34E-43	1.11E-43	0.54	0.44	0.68	2.75	3.12	3.03
OsR498G0102154100.01	33.933	16.693	-1.008634	1.68E-78	9.91E-79	32.37	33.32	36.11	16.45	16.94	16.69
OsR498G0102159600.01	4.623	0.066	-6.091679	6.27E-97	3.22E-97	4.48	5.15	4.24	0.03	0.03	0.14
OsR498G0102162700.01	0.98	3.583	1.8725303	5.79E-33	5.56E-33	1.54	0.73	0.67	3.9	3.34	3.51
OsR498G0102165600.01	18.743	52.093	1.4728281	1.65E-132	6.84E-133	16.37	19.35	20.51	51.97	51.27	53.04
OsR498G0102166500.01	0.313	2.096	2.7501948	5.80E-36	5.33E-36	0.23	0.48	0.23	2.12	1.84	2.33
OsR498G0102166700.01	4.563	0.726	-2.651425	9.97E-55	7.27E-55	4.73	4.6	4.36	0.78	0.48	0.92
OsR498G0102167600.01	11.056	4.86	-1.134257	7.29E-31	7.25E-31	11.34	11.27	10.56	4.14	5.14	5.3
OsR498G0102167900.01	0.493	0.203	-1.42998	0.0001169	0.0002277	0.69	0.43	0.36	0.08	0.37	0.16
OsR498G0102168600.01	1.97	0.943	-1.030236	5.32E-14	7.31E-14	2.35	1.67	1.89	1.06	1	0.77
OsR498G0102176800.01	0.536	1.776	1.7113756	1.78E-24	1.97E-24	0.49	0.53	0.59	1.96	1.56	1.81
OsR498G0102177100.01	0.076	0.683	3.144335	8.30E-05	0.00016	0	0	0.23	0.79	0.68	0.58
OsR498G0102180100.01	0.78	0.07	-3.466163	2.60E-16	3.37E-16	0.96	0.61	0.77	0.15	0.06	0
OsR498G0102182300.01	92.796	257.71	1.445562	0	0	91.3	97.37	89.72	266.77	253.09	253.27
OsR498G0102184800.01	0.346	0.79	1.1608342	6.63E-06	1.21E-05	0.2	0.37	0.47	0.92	0.71	0.74
OsR498G0102186800.01	2.423	9.3	1.9433831	3.70E-75	2.23E-75	2.41	2.55	2.31	9.7	9.59	8.61
OsR498G0102187200.01	0.25	0.67	1.33698	6.21E-06	1.13E-05	0.11	0.24	0.4	0.82	0.6	0.59
OsR498G0102187500.01	11.976	5.53	-1.118846	3.59E-31	3.55E-31	10.15	12.68	13.1	4.6	5.28	6.71
OsR498G0102193100.01	1.976	4.676	1.2357965	7.96E-11	1.20E-10	1.88	1.94	2.11	3.33	4.19	6.51
OsR498G0102198400.01	0.646	8.926	3.7077643	3.43E-54	2.52E-54	0.58	0.68	0.68	8.33	7.99	10.46
OsR498G0102199200.01	2.576	0.15	-4.227224	7.22E-22	8.36E-22	2.59	2.3	2.84	0.17	0.28	0
OsR498G0102200100.01	0.023	2.07	6.7688258	4.57E-27	4.84E-27	0	0	0.07	1.98	2.12	2.11
OsR498G0102200300.01	151.583	304.31	1.0548935	0	0	152.37	151.35	151.03	305.49	301.53	305.91
OsR498G0102201400.01	0.123	0	-4.610553	0.0002051	0.000405	0.14	0.13	0.1	0	0	0
OsR498G0102202600.01	0.083	0.27	1.7817649	8.23E-05	0.0001585	0.05	0.12	0.08	0.27	0.25	0.29
OsR498G0102202700.01	0.14	0.576	2.0524125	6.12E-10	9.48E-10	0.07	0.19	0.16	0.45	0.69	0.59
OsR498G0102203600.01	0.226	2.46	3.3634522	5.32E-42	4.50E-42	0.52	0.09	0.07	2.65	1.93	2.8
OsR498G0102204200.01	23.733	90.323	1.9236493	0	0	24.39	22.58	24.23	91.32	90.71	88.94
OsR498G0102206900.01	29.29	166.95	2.5155657	0	0	31.97	27.37	28.53	163.77	167.31	169.78
OsR498G0102207400.01	0.15	1.51	3.3154469	2.88E-29	2.93E-29	0.18	0.09	0.18	1.52	1.78	1.23
OsR498G0102207500.01	0.043	0.853	4.3389824	3.62E-24	4.02E-24	0.08	0.05	0	0.94	0.96	0.66
OsR498G0102207800.01	1.126	5.966	2.4338416	1.64E-55	1.19E-55	1.06	1.29	1.03	5.89	5.47	6.54
OsR498G0102210700.01	0.106	0.333	1.4598368	1.88E-05	3.48E-05	0.16	0.12	0.04	0.4	0.38	0.22
OsR498G0102215800.01	0.143	0.413	1.5420191	3.42E-07	5.88E-07	0.15	0.17	0.11	0.37	0.48	0.39
OsR498G0102216100.01	0.646	2.056	1.694302	1.03E-15	1.35E-15	0.83	0.59	0.52	1.86	2.1	2.21
OsR498G0102217000.01	7.296	18.91	1.3847973	1.62E-204	4.76E-205	7.63	6.78	7.48	18.19	19.03	19.51

OsR498G0102222100.01	0.316	2.063	2.7469995	9.29E-33	8.95E-33	0.12	0.51	0.32	1.84	2.25	2.1
OsR498G0102222400.01	0.056	0.353	2.6589081	2.45E-08	4.01E-08	0.07	0.1	0	0.49	0.33	0.24
OsR498G0102223200.01	0.53	9.513	4.1726796	2.95E-165	1.04E-165	0.57	0.38	0.64	9.52	10.51	8.51
OsR498G0102228800.01	11.02	27.453	1.2963381	1.58E-128	6.67E-129	12.03	11.41	9.62	26.78	28.56	27.02
OsR498G0102234200.01	0.68	0	-5.932481	6.55E-09	1.05E-08	1.31	0.27	0.46	0	0	0
OsR498G0102236100.01	2.153	0.026	-6.263995	1.85E-37	1.66E-37	1.98	1.96	2.52	0	0.08	0
OsR498G0102236300.01	5.24	1.273	-2.097601	1.19E-61	8.06E-62	5.52	5.31	4.89	1.09	1.24	1.49
OsR498G0102236500.01	5.596	33.21	2.5689585	3.66E-192	1.13E-192	7.42	4.53	4.84	33.62	31.1	34.91
OsR498G0102236900.01	0.396	1.176	1.5748025	8.80E-09	1.42E-08	0.61	0.27	0.31	1.04	1.17	1.32
OsR498G0102239000.01	0.1	0.373	1.933768	1.01E-08	1.63E-08	0.13	0.09	0.08	0.49	0.32	0.31
OsR498G0102239800.01	1.313	2.686	1.0282327	1.93E-15	2.56E-15	1.39	1.35	1.2	2.7	2.51	2.85
OsR498G0102242100.01	7.02	19.46	1.5685447	3.20E-223	8.71E-224	6.45	7.18	7.43	19.25	20.41	18.72
OsR498G0102242700.01	2.59	8.446	1.7145221	4.63E-66	3.02E-66	2.31	2.89	2.57	8.47	8.4	8.47
OsR498G0102247400.01	4.14	48.97	3.5579819	0	0	4.64	3.54	4.24	48.99	46.93	50.99
OsR498G0102247500.01	1.816	5.663	1.6955768	6.83E-57	4.88E-57	1.83	1.94	1.68	5.44	5.78	5.77
OsR498G0102247900.01	23.08	47.283	1.0310961	9.49E-67	6.16E-67	24.34	22.96	21.94	49.42	43.16	49.27
OsR498G0102249300.01	4.45	46.236	3.3770817	0	0	4.99	3.99	4.37	44.51	45.79	48.41
OsR498G0102251200.01	2.496	0.256	-3.266598	1.06E-20	1.26E-20	2.32	2.51	2.66	0.35	0.28	0.14
OsR498G0102254000.01	0.783	2.2	1.3628681	9.39E-20	1.13E-19	0.77	0.77	0.81	2.12	2.15	2.33
OsR498G0102257700.01	0.983	11.47	3.5413896	1.79E-168	6.15E-169	0.75	0.89	1.31	13.31	10.2	10.9
OsR498G0102262400.01	2.41	9.26	1.9418053	1.69E-71	1.05E-71	2.4	2.29	2.54	9.34	9.57	8.87
OsR498G0102265100.01	1.806	14.7	3.0304628	1.39E-254	3.37E-255	1.82	2.13	1.47	14.39	13.99	15.72
OsR498G0102265800.01	1.243	0.403	-1.620537	2.87E-13	4.02E-13	1.06	1.36	1.31	0.38	0.37	0.46
OsR498G0102268500.01	19.273	40.833	1.0730796	2.85E-109	1.34E-109	19.73	19.04	19.05	40.05	41.2	41.25
OsR498G0102268800.01	0.623	1.696	1.4293119	4.59E-12	6.64E-12	0.7	0.44	0.73	1.52	1.61	1.96
OsR498G0102270000.01	19.653	45.746	1.2230716	3.19E-126	1.37E-126	19.75	18.99	20.22	45.61	46.47	45.16
OsR498G0102270900.01	1.906	0.376	-2.279347	5.78E-25	6.33E-25	1.83	2.07	1.82	0.46	0.24	0.43
OsR498G0102271500.01	0.023	7.7	8.4722618	3.29E-144	1.27E-144	0	0	0.07	7.67	7.51	7.92
OsR498G0102273300.01	0.626	5.303	3.0886671	4.28E-135	1.74E-135	0.6	0.64	0.64	5.44	5.84	4.63
OsR498G0102274300.01	2.016	8.883	2.1919333	3.55E-59	2.47E-59	2.33	1.65	2.07	7.98	9.4	9.27
OsR498G0102275200.01	27.273	54.686	1.0038696	2.56E-209	7.39E-210	26.53	28.11	27.18	54.21	55.04	54.81
OsR498G0102276700.01	13.576	28.486	1.0429072	6.19E-181	2.02E-181	13.89	13.87	12.97	28.36	30.03	27.07
OsR498G0102279000.01	9.903	28.246	1.5125186	4.81E-110	2.26E-110	10.5	10.36	8.85	27.53	28.74	28.47
OsR498G0102279400.01	26.266	110.94	2.0657484	0	0	27.18	26.02	25.6	109.67	108.44	114.71
OsR498G0102281000.01	2.183	5.176	1.2467822	1.57E-11	2.31E-11	2.65	1.61	2.29	5.14	5.29	5.1
OsR498G0102283700.01	45.26	16.893	-1.392243	0	0	44.16	47.42	44.2	17.42	16.59	16.67
OsR498G0102284000.01	16.553	35.343	1.1249005	2.37E-175	7.93E-176	16.12	16.35	17.19	35.14	35.03	35.86
OsR498G0102284100.01	0.126	0.483	1.933768	3.47E-05	6.52E-05	0.17	0.17	0.04	0.53	0.37	0.55
OsR498G0102286500.01	59.056	27.733	-1.097204	1.24E-88	6.78E-89	61.52	58.79	56.86	27.32	24.75	31.13
OsR498G0102289000.01	14.656	31.643	1.2599201	6.15E-169	2.11E-169	14.05	15.09	14.83	31.87	30.37	32.69
OsR498G0102289100.01	0.54	1.253	1.2157026	0.0001027	0.0001992	0	1.46	0.16	2.92	0.84	0
OsR498G0102290000.01	0.97	23.98	4.681363	0	0	0.88	0.93	1.1	24.19	24.95	22.8
OsR498G0102290700.01	0.476	1.343	1.4979719	1.60E-06	2.82E-06	0.42	0.71	0.3	1.16	1.68	1.19
OsR498G0102291100.01	8.233	19.07	1.2070707	1.97E-83	1.12E-83	8.33	8.29	8.08	18.84	18.21	20.16
OsR498G0102291300.01	10.673	21.576	1.0277395	9.30E-78	5.52E-78	10.98	10.48	10.56	20.83	23.39	20.51
OsR498G0102297100.01	12.363	25.943	1.0754498	5.30E-64	3.53E-64	11.48	12.99	12.62	29.14	23.59	25.1
OsR498G0102297400.01	0.806	2.2	1.4453007	3.58E-12	5.16E-12	0.6	0.93	0.89	2.32	2.4	1.88
OsR498G0102298800.01	0.023	3.54	7.331962	8.42E-38	7.53E-38	0.07	0	0	2.96	4.16	3.5
OsR498G0102299000.01	0.61	0.013	-5.6403	1.86E-13	2.59E-13	0.56	0.56	0.71	0	0.04	0
OsR498G0102299300.01	0.67	5.49	3.0374198	7.59E-41	6.54E-41	0.6	0.84	0.57	5.93	4.91	5.63
OsR498G0102301300.01	0.21	1.106	2.3743406	5.27E-14	7.23E-14	0.21	0.28	0.14	1.01	1.06	1.25
OsR498G0102306200.01	6.7	24.813	1.850976	1.73E-87	9.55E-88	6.79	7.22	6.09	22.79	24.94	26.71
OsR498G0102306400.01	0.746	1.716	1.2734352	6.16E-08	1.03E-07	0.71	0.92	0.61	1.66	1.69	1.8
OsR498G0102308400.01	13.706	27.49	1.0126024	3.94E-136	1.59E-136	13.93	13.67	13.52	27.16	28.87	26.44
OsR498G0102309500.01	10.746	27.133	1.3413532	1.87E-222	5.10E-223	10.42	11.28	10.54	27.57	28.46	25.37
OsR498G0102309700.01	6.69	17.713	1.4114909	8.67E-84	4.92E-84	7.27	5.61	7.19	17.99	17.3	17.85
OsR498G0102309900.01	3.69	110.15	4.8981165	0	0	3.53	4.26	3.28	107.11	109.41	113.92
OsR498G0102311900.01	71.346	161.86	1.1783104	0	0	73.04	70.96	70.04	163.43	166.68	155.46
OsR498G0102318300.01	4.896	31.953	2.7422549	0	0	4.56	4.58	5.55	32.53	32.61	30.72
OsR498G0102318600.01	3.706	9.28	1.3805802	2.04E-51	1.54E-51	3.41	4.09	3.62	9.08	9.24	9.52
OsR498G0102319600.01	0.38	0.136	-1.440628	0.0008564	0.0017736	0.54	0.25	0.35	0.17	0.03	0.21
OsR498G0102322000.01	0.3	3.053	3.3328309	1.88E-42	1.58E-42	0.14	0.48	0.28	2.74	2.5	3.92
OsR498G0102322100.01	0.12	1.72	3.8399267	1.15E-31	1.13E-31	0.09	0.04	0.23	1.49	1.62	2.05
OsR498G0102328800.01	2.756	10.596	1.9425739	6.00E-51	4.57E-51	3.23	2.42	2.62	10.57	11.23	9.99
OsR498G0102329500.01	60.523	245.72	2.0226148	0	0	62.64	59.29	59.64	248.73	244.6	243.84
OsR498G0102331100.01	11.78	3.163	-1.893354	1.83E-209	5.27E-210	12.06	11.79	11.49	3.09	3.15	3.25
OsR498G0102333600.01	0.99	3.716	1.906023	7.30E-22	8.45E-22	0.94	1.26	0.77	4.46	3.27	3.42
OsR498G0102338900.01	4.383	23.2	2.4179366	7.70E-285	1.70E-285	4.8	3.77	4.58	22.79	23.18	23.63
OsR498G0102339600.01	0.026	0.4	3.7817649	2.33E-07	3.97E-07	0.04	0.04	0	0.42	0.3	0.48

OsR498G0102339900.01	0.346	34.883	6.61979	0	0	0.33	0.33	0.38	34.84	33.54	36.27
OsR498G0102340200.01	0.066	7.71	6.8242842	5.60E-28	5.83E-28	0.2	0	0	10.47	5.53	7.13
OsR498G0102340300.01	0.196	0.046	-2.163094	0.0002982	0.0005957	0.22	0.21	0.16	0	0.03	0.11
OsR498G0102341300.01	168.896	961.67	2.508218	0	0	172.97	164.59	169.13	948.81	943.62	992.58
OsR498G0102341500.01	0.6	1.906	1.6512113	6.31E-20	7.59E-20	0.59	0.63	0.58	1.81	1.71	2.2
OsR498G0102343200.01	0.2	1.003	2.2963381	2.60E-07	4.44E-07	0.13	0.21	0.26	1.46	0.73	0.82
OsR498G0102343400.01	2.923	8.67	1.5658565	3.53E-51	2.68E-51	2.62	3.03	3.12	9.37	7.68	8.96
OsR498G0102345000.01	40.24	19.846	-1.01728	5.65E-247	1.42E-247	40.79	39.43	40.5	19.9	20.32	19.32
OsR498G0102354500.01	11.693	3.52	-1.734099	9.53E-111	4.46E-111	11.47	12.04	11.57	3.41	3.28	3.87
OsR498G0102357800.01	0.486	0.946	1.2739702	5.98E-07	1.04E-06	0.48	0.28	0.7	1.07	0.8	0.97
OsR498G0102360100.01	6.253	14.71	1.2326434	1.51E-112	7.03E-113	6.04	6.34	6.38	13.76	15.21	15.16
OsR498G0102361800.01	0.566	3.06	2.4319932	3.16E-53	2.35E-53	0.58	0.62	0.5	3.39	2.95	2.84
OsR498G0102368400.01	2.176	0.723	-1.587469	2.60E-11	3.85E-11	2.01	1.94	2.58	0.98	0.66	0.53
OsR498G0102369300.01	0.343	0.08	-2.247982	6.81E-07	1.18E-06	0.35	0.13	0.55	0.08	0.13	0.03
OsR498G0102369600.01	0.303	1.243	2.0345309	1.33E-22	1.52E-22	0.39	0.33	0.19	1.3	1.32	1.11
OsR498G0102370400.01	1.89	5.946	1.6501379	2.01E-21	2.34E-21	2.15	1.17	2.35	4.75	6.3	6.79
OsR498G0102370800.01	3.706	8.183	1.083067	2.17E-44	1.77E-44	3.54	3.32	4.26	7.72	8.46	8.37
OsR498G0102372900.01	0.546	1.66	1.6051761	1.60E-16	2.07E-16	0.53	0.63	0.48	2.01	1.59	1.38
OsR498G0102374400.01	0.03	0.18	2.4769103	0.0005775	0.0011774	0.06	0	0.03	0.19	0.22	0.13
OsR498G0102377500.01	9.053	19.206	1.1170592	2.23E-63	1.50E-63	8.15	9.57	9.44	21.09	19.63	16.9
OsR498G0102377800.01	2.073	0.72	-1.456131	4.73E-14	6.49E-14	2.08	2.02	2.12	0.84	0.55	0.77
OsR498G0102379100.01	2.52	17.463	2.7991995	3.18E-155	1.16E-155	2.48	2.19	2.89	16.46	18.65	17.28
OsR498G0102379300.01	1.116	0.44	-1.347518	3.02E-06	5.39E-06	0.99	0.84	1.52	0.44	0.52	0.36
OsR498G0102381400.01	0.696	2.5	2.4904251	3.22E-21	3.77E-21	0.68	0.69	0.72	2.89	2.37	2.24
OsR498G0102383900.01	98.17	35.626	-1.463638	1.27E-256	3.04E-257	98.03	98.88	97.6	38.32	32.38	36.18
OsR498G0102384500.01	0.736	2.783	1.923426	1.67E-15	2.21E-15	0.43	0.8	0.98	2.72	2.49	3.14
OsR498G0102385500.01	0.333	5.06	3.9182271	1.97E-58	1.38E-58	0.28	0.5	0.22	4.73	5.64	4.81
OsR498G0102387900.01	0.963	6.923	2.8464023	1.73E-62	1.17E-62	1.04	0.76	1.09	6.95	6.08	7.74
OsR498G0102390500.01	3.686	36.363	3.2984897	1.93E-242	4.91E-243	3.4	3.54	4.12	37.68	32.86	38.55
OsR498G0102391600.01	0.98	148.76	7.2451774	0	0	0.88	0.87	1.19	151.46	142.26	152.56
OsR498G0102391800.01	4.723	1.563	-1.617821	1.81E-64	1.20E-64	4.85	4.52	4.8	1.4	1.75	1.54
OsR498G0102395000.01	3.816	14.373	1.9329178	1.16E-113	5.37E-114	4.06	3.94	3.45	14.7	14.56	13.86
OsR498G0102395800.01	0.496	1.763	1.832391	3.44E-15	4.58E-15	0.45	0.52	0.52	1.74	1.84	1.71
OsR498G0102399300.01	0	0.673	5.832391	1.49E-08	2.43E-08	0	0	0	0.94	0.6	0.48
OsR498G0102401800.01	9.01	20.766	1.2155321	2.68E-46	2.15E-46	9.73	9.76	7.54	21.5	18.61	22.19
OsR498G0102403700.01	0.95	2.63	1.42121	1.51E-14	2.05E-14	0.89	1	0.96	2.44	2.87	2.58
OsR498G0102404000.01	1.793	15.886	3.1546875	0	0	1.51	1.97	1.9	14.55	16.3	16.81
OsR498G0102404900.01	5.64	11.896	1.0493148	4.52E-55	3.29E-55	5.98	5.05	5.89	11.65	11.73	12.31
OsR498G0102405100.01	8.623	17.496	1.0193065	1.58E-42	1.33E-42	8.72	9.13	8.02	16.92	17.14	18.43
OsR498G0102406000.01	0.46	3.11	3.144335	2.76E-31	2.72E-31	0.69	0.61	0.08	2.05	3.27	4.01
OsR498G0102408600.01	0.113	0.613	2.4598368	2.74E-24	3.03E-24	0.12	0.06	0.16	0.68	0.54	0.62
OsR498G0102411000.01	0.003	0.183	5.4979719	1.58E-12	2.25E-12	0	0.01	0	0.28	0.16	0.11
OsR498G0102411200.01	0.086	8.216	6.5213044	2.04E-173	6.86E-174	0.1	0.13	0.03	8.2	7.55	8.9
OsR498G0102412100.01	26.866	88.66	1.6538421	0	0	26.95	26.68	26.97	90.62	86.01	89.35
OsR498G0102416900.01	457.986	227.24	-1.01016	0	0	450.33	471.68	451.95	231.03	227.3	223.39
OsR498G0102417100.01	4.4	16.043	1.8673678	5.16E-155	1.89E-155	4.59	4.43	4.18	16.16	16.53	15.44
OsR498G0102417400.01	7.456	16.283	1.1271452	7.96E-41	6.86E-41	7.25	7.24	7.88	16.23	17.43	15.19
OsR498G0102418100.01	2.053	0.61	-1.745482	1.72E-21	2.01E-21	1.92	2.27	1.97	0.49	0.77	0.57
OsR498G0102421700.01	0.636	0.296	-1.148972	0.0001501	0.0002942	0.69	0.64	0.58	0.34	0.29	0.26
OsR498G0102422400.01	9.783	19.726	1.0233559	8.29E-158	3.01E-158	10.22	8.94	10.19	20.09	19.87	19.22
OsR498G0102423600.01	21.173	5.84	-1.858853	1.83E-232	4.83E-233	22.08	20.7	20.74	6.25	5.15	6.12
OsR498G0102429100.01	5.153	17.81	1.7877699	1.64E-87	9.05E-88	5.33	4.78	5.35	17.84	16.99	18.6
OsR498G0102431700.01	1.37	5.32	1.9330317	1.94E-66	1.26E-66	1.44	1.5	1.17	4.71	5.58	5.67
OsR498G0102432600.01	1.783	8.456	2.286354	5.65E-90	3.06E-90	2.21	1.41	1.73	8.59	8.38	8.4
OsR498G0102433100.01	27.783	72.45	1.3526209	0	0	27.41	28.34	27.6	72.72	71.5	73.13
OsR498G0102435700.01	1.093	0.55	-1.000499	0.0008534	0.001767	0.92	0.92	1.44	0.57	0.45	0.63
OsR498G0102437500.01	0.553	0.08	-2.803198	2.56E-09	4.04E-09	0.38	0.66	0.62	0.07	0.03	0.14
OsR498G0102439000.01	2.736	16.65	2.5978768	0	0	2.86	2.38	2.97	17.21	17.11	15.63
OsR498G0102440700.01	0.86	4.103	2.258713	3.30E-61	2.26E-61	0.69	0.79	1.1	3.96	4	4.35
OsR498G0102446100.01	34.39	12.236	-1.492141	0	0	34.53	35.03	33.61	12.93	12.19	11.59
OsR498G0102446400.01	1.383	9.276	2.7473955	5.81E-129	2.45E-129	1.45	1.47	1.23	9.7	9.8	8.33
OsR498G0102447400.01	1.246	12.513	3.3280469	4.59E-168	1.58E-168	1.17	1.2	1.37	11.61	12.41	13.52
OsR498G0102448800.01	0.54	2.51	2.227176	1.95E-28	2.01E-28	0.62	0.52	0.48	2.5	2.54	2.49
OsR498G0102453500.01	9.923	2.45	-2.007888	4.44E-156	1.62E-156	10.19	9.88	9.7	2.53	2.5	2.32
OsR498G0102454200.01	0.07	0.386	2.524607	2.07E-07	3.52E-07	0.03	0.06	0.12	0.45	0.36	0.35
OsR498G0102458300.01	15.386	6.923	-1.158506	2.76E-58	1.94E-58	13.82	15.84	16.5	6.67	7.55	6.55
OsR498G0102459900.01	2.663	0.39	-2.780478	3.16E-31	3.12E-31	2.47	2.48	3.04	0.54	0.16	0.47
OsR498G0102460200.01	9.136	2.583	-1.760578	1.75E-66	1.13E-66	8.76	9.39	9.26	2.54	2.55	2.66
OsR498G0102460500.01	0.216	1.013	2.2474285	3.36E-05	6.31E-05	0.11	0.22	0.32	1.04	1.14	0.86

OsR498G0102462100.01	12.466	29.55	1.2510378	2.95E-166	1.03E-166	13.16	12.42	11.82	27.51	32.08	29.06
OsR498G0102466800.01	0.99	4.866	2.3176853	1.75E-103	8.60E-104	0.96	0.87	1.14	4.81	4.64	5.15
OsR498G0102467600.01	148.506	487.14	1.7150841	0	0	151.25	141.87	152.4	476.5	473.9	511.01
OsR498G0102469100.01	0.706	4.1	2.5727583	2.82E-85	1.58E-85	0.64	0.91	0.57	4.4	3.69	4.21
OsR498G0102470900.01	1.04	0.393	-1.404102	0.0003553	0.0007128	0.72	1.04	1.36	0.31	0.47	0.4
OsR498G0102471600.01	11.493	26.776	1.2199757	1.25E-132	5.17E-133	10.56	12.04	11.88	26.15	27.35	26.83
OsR498G0102472000.01	0.59	0.036	-4.007443	1.63E-18	2.01E-18	0.5	0.57	0.7	0.04	0.03	0.04
OsR498G0102473900.01	0.006	0.106	3.97441	5.14E-05	9.78E-05	0	0.02	0	0.16	0.1	0.06
OsR498G0102483900.01	2.006	0.923	-1.117333	3.08E-11	4.58E-11	2.04	1.96	2.02	1.19	0.58	1
OsR498G0102484600.01	1.836	3.843	1.144335	1.30E-48	1.02E-48	1.71	1.83	1.97	3.8	3.83	3.9
OsR498G0102485400.01	5.06	11.69	1.0159563	2.72E-48	2.14E-48	5.04	5.39	4.75	12.1	11.35	11.62
OsR498G0102488800.01	0.373	0	-6.85848	5.99E-15	8.03E-15	0.4	0.27	0.45	0	0	0
OsR498G0102489700.01	0.04	0.246	2.718793	1.33E-06	2.34E-06	0.06	0	0.06	0.06	0.2	0.48
OsR498G0102492500.01	4.366	40.08	3.2107176	0	0	4.27	5.11	3.72	41.66	39.51	39.07
OsR498G0102492800.01	1.95	12.303	2.764931	4.77E-136	1.93E-136	2.45	1.77	1.63	12.26	12.3	12.35
OsR498G0102495900.01	0.433	0.033	-3.72603	0.0002637	0.0005248	0.4	0.4	0.5	0.1	0	0
OsR498G0102496500.01	2.436	0.87	-1.603463	1.28E-17	1.61E-17	2.78	2.2	2.33	1.05	0.88	0.68
OsR498G0102496800.01	0.296	0.923	1.656234	6.15E-08	1.02E-07	0.26	0.15	0.48	0.82	1.07	0.88
OsR498G0102498100.01	0.326	0.68	1.0829344	0.0001402	0.0002742	0.39	0.13	0.46	0.8	0.7	0.54
OsR498G0102502500.01	17.433	37.746	1.0685804	3.30E-107	1.58E-107	17.24	17.11	17.95	38.86	37.79	36.59
OsR498G0102503900.01	1.096	2.516	1.1968024	8.55E-05	0.0001651	1.1	1.46	0.73	2.26	2.37	2.92
OsR498G0102505400.01	0	0.336	5.8047667	2.01E-08	3.29E-08	0	0	0	0	0.09	0.92
OsR498G0102505500.01	0.036	0.63	4.1573697	1.44E-09	2.25E-09	0	0	0.11	0.49	0.99	0.41
OsR498G0102506100.01	0.276	0.613	1.1168542	6.83E-07	1.19E-06	0.32	0.32	0.19	0.55	0.72	0.57
OsR498G0102506400.01	280.783	77.376	-1.855137	0	0	288.07	279.89	274.39	75.51	78.19	78.43
OsR498G0102506700.01	28.72	176.82	2.6236154	0	0	29.71	26.5	29.95	171.95	183.31	175.2
OsR498G0102506900.01	8.353	58.776	2.8132941	0	0	8.29	7.64	9.13	63.61	54.67	58.05
OsR498G0102507600.01	4.563	11.87	1.3712369	6.92E-48	5.45E-48	4.17	4.61	4.91	12.73	11.28	11.6
OsR498G0102508000.01	18.023	36.84	1.0354655	1.90E-107	9.10E-108	17.01	19.02	18.04	35.41	38.13	36.98
OsR498G0102508400.01	2.086	0.86	-1.219763	1.01E-08	1.62E-08	2.18	1.87	2.21	0.86	0.67	1.05
OsR498G0102509100.01	36.4	16.006	-1.201243	9.42E-179	3.10E-179	35.1	38.28	35.82	16.12	16.29	15.61
OsR498G0102513000.01	17.816	41.656	1.2288569	0	0	17.86	18.03	17.56	41.51	42.01	41.45
OsR498G0102514700.01	120.376	49.426	-1.289675	0	0	126.92	115.55	118.66	47.16	48.04	53.08
OsR498G0102514900.01	5.75	12.956	1.1747086	1.50E-68	9.59E-69	5.63	6.19	5.43	12.2	13.06	13.61
OsR498G0102520500.01	1.446	4.05	1.5104629	1.78E-46	1.42E-46	1.42	1.39	1.53	4.14	4.13	3.88
OsR498G0102525300.01	11.086	5.466	-1.02474	3.26E-49	2.53E-49	10.82	11.22	11.22	5.1	5.46	5.84
OsR498G0102525600.01	65.553	16.616	-1.965065	0	0	66.39	70.22	60.05	17.22	17.73	14.9
OsR498G0102526000.01	1.203	0.49	-1.299238	1.19E-12	1.69E-12	1.07	1.1	1.44	0.38	0.68	0.41
OsR498G0102527000.01	0.863	2.293	1.3594349	4.30E-15	5.74E-15	0.79	0.93	0.87	2.24	2.45	2.19
OsR498G0102527700.01	11.306	26.08	1.2091816	1.50E-139	5.94E-140	10.74	11.04	12.14	26.58	25.03	26.63
OsR498G0102528100.01	0.756	8.116	3.3558391	5.80E-182	1.88E-182	0.58	0.72	0.97	8.58	8.16	7.61
OsR498G0102528600.01	2.193	30.73	3.8644588	0	0	2.16	2.23	2.19	30.66	29.88	31.65
OsR498G0102529000.01	2.163	0.28	-2.955588	7.76E-99	3.93E-99	1.91	2.56	2.02	0.28	0.34	0.22
OsR498G0102529600.01	3.083	8.19	1.4089556	1.80E-53	1.33E-53	3.01	3.43	2.81	8.15	8.03	8.39
OsR498G0102530100.01	1.223	0.333	-1.884728	4.90E-17	6.26E-17	1.3	1.43	0.94	0.37	0.39	0.24
OsR498G0102535900.01	0.286	2.266	2.97441	4.75E-41	4.07E-41	0.14	0.37	0.35	2.33	1.93	2.54
OsR498G0102536100.01	0.123	0.946	3.1282153	4.34E-17	5.54E-17	0.2	0.08	0.09	0.8	1.05	0.99
OsR498G0102539200.01	2.323	17.703	2.8940971	1.49E-262	3.49E-263	2.72	2.12	2.13	17.12	18.37	17.62
OsR498G0102547300.01	7.28	23.753	1.6944025	2.80E-141	1.10E-141	7.04	7.14	7.66	24.05	22.48	24.73
OsR498G0102548800.01	4.386	36.703	3.0695964	0	0	4.5	4.22	4.44	37.95	37.47	34.69
OsR498G0102550900.01	0.956	2.65	1.4705632	1.53E-14	2.07E-14	1.13	0.94	0.8	2.3	2.95	2.7
OsR498G0102552200.01	6.08	22.5	1.899445	1.25E-182	4.04E-183	6.31	5.97	5.96	23.31	21.97	22.22
OsR498G0102552300.01	0.586	1.213	1.0483783	3.88E-11	5.78E-11	0.58	0.68	0.5	1.18	1.14	1.32
OsR498G0102553600.01	3.793	8.29	1.1341536	1.04E-17	1.31E-17	3.26	4.52	3.6	8.82	7.95	8.1
OsR498G0102553700.01	2.68	6.83	1.3497013	2.15E-12	3.08E-12	3.25	2.1	2.69	6.35	6.69	7.45
OsR498G0102553900.01	55.813	16.27	-1.779001	0	0	56.51	54.49	56.44	16.25	15.67	16.89
OsR498G0102554600.01	0.08	17.426	7.7817649	2.04E-96	1.05E-96	0.24	0	0	15.3	17.66	19.32
OsR498G0102555700.01	10.033	25.803	1.364632	1.66E-218	4.59E-219	10.66	9.44	10	26.15	26.34	24.92
OsR498G0102558400.01	1.906	4.986	1.4008884	3.08E-28	3.20E-28	1.52	2.15	2.05	5.23	5.16	4.57
OsR498G0102560100.01	849.666	196.3	-2.111955	0	0	845.18	862.03	841.79	192.87	197.39	198.64
OsR498G0102562000.01	1.903	0.733	-1.38816	2.03E-07	3.45E-07	2.01	1.93	1.77	0.57	0.81	0.82
OsR498G0102562800.01	0.393	2.206	2.2579667	2.34E-22	2.68E-22	0.25	0.62	0.31	2.3	1.74	2.58
OsR498G0102563800.01	0.033	0.356	3.8813006	9.36E-05	0.0001812	0	0.1	0	0.34	0.14	0.59
OsR498G0102565000.01	0.04	0.5	3.9484147	1.90E-24	2.10E-24	0.03	0.05	0.04	0.51	0.49	0.5
OsR498G0102565100.01	0.08	0.626	3.0618728	0.0001482	0.0002902	0.1	0	0.14	0.83	0.36	0.69
OsR498G0102565700.01	20.75	41.833	1.0244019	3.12E-187	9.88E-188	22.1	19.91	20.24	43.34	41.6	40.56
OsR498G0102568600.01	25.923	54.08	1.0878771	1.33E-242	3.37E-243	25.58	26.39	25.8	53.52	52.27	56.45
OsR498G0102571400.01	0.176	3.423	4.4726608	1.70E-51	1.29E-51	0.22	0.23	0.08	3.72	3.23	3.32
OsR498G0102571600.01	0	0.12	4.2963381	0.0007942	0.0016388	0	0	0	0.14	0.07	0.15

OsR498G0102571800.01	0.033	0.883	4.7023304	1.02E-13	1.41E-13	0.05	0	0.05	0.89	0.89	0.87
OsR498G0102572600.01	0.176	1.743	3.3037441	5.07E-72	3.14E-72	0.19	0.26	0.08	2	1.47	1.76
OsR498G0102572700.01	45.926	151.49	1.7206653	0	0	45.76	45.99	46.03	152.8	150.89	150.78
OsR498G0102573300.01	1.06	4.56	2.2612911	2.37E-31	2.34E-31	1.42	0.93	0.83	4.02	5.89	3.77
OsR498G0102573500.01	0.286	0.793	1.4691746	0.0009479	0.0019719	0.16	0.39	0.31	0.84	0.45	1.09
OsR498G0102575500.01	10.34	2.463	-2.106236	1.98E-138	7.92E-139	9.95	10.7	10.37	2.56	2.34	2.49
OsR498G0102576200.01	107.536	31.1	-1.784445	0	0	108.03	105.54	109.04	30.87	32.18	30.25
OsR498G0102576500.01	2.873	1.263	-1.189977	3.62E-37	3.27E-37	2.79	3.19	2.64	1.02	1.24	1.53
OsR498G0102581400.01	2.803	6.12	1.0685321	4.06E-25	4.44E-25	2.76	2.78	2.87	6.18	6.01	6.17
OsR498G0102585300.01	11.97	37.5	1.6382969	1.75E-186	5.57E-187	11.81	11.66	12.44	35.12	36.93	40.45
OsR498G0102586700.01	43.403	22.24	-1.127016	2.83E-268	6.54E-269	43.11	41.75	45.35	21.59	22.13	23
OsR498G0102589900.01	5.866	14.306	1.2844313	6.74E-91	3.62E-91	5.32	6	6.28	14.61	13.69	14.62
OsR498G0102592100.01	1.233	0.06	-4.417907	3.28E-25	3.57E-25	1.56	1.37	0.77	0.07	0	0.11
OsR498G0102593100.01	3.366	8.62	1.327378	9.04E-71	5.66E-71	3.49	3.4	3.21	8.46	8.8	8.6
OsR498G0102597800.01	4.38	17.293	1.9708416	1.38E-206	4.04E-207	4.2	4.63	4.31	17.52	16.33	18.03
OsR498G0102601900.01	3.566	1.15	-1.629853	8.35E-26	9.02E-26	3.35	3.61	3.74	1.16	1.1	1.19
OsR498G0102605100.01	0.763	2.063	1.4390782	3.06E-06	5.48E-06	0.83	0.64	0.82	1.96	2.13	2.1
OsR498G0102607100.01	12.613	33.376	1.4104187	0	0	12.85	12.05	12.94	33.86	34.26	32.01
OsR498G0102612100.01	12.286	26.196	1.088788	1.39E-84	7.86E-85	11.54	11.66	13.66	26.5	26.43	25.66
OsR498G0102612600.01	5.83	17.673	1.5908627	1.57E-134	6.40E-135	5.65	6	5.84	17.03	18.27	17.72
OsR498G0102613700.01	40.876	18.966	-1.100557	4.45E-190	1.39E-190	40.73	40.37	41.53	18.15	19.38	19.37
OsR498G0102617400.01	1.36	3.193	1.2122738	7.61E-27	8.09E-27	1.36	1.3	1.42	3.13	3.12	3.33
OsR498G0102619200.01	12.236	186.96	3.9339909	0	0	12.47	11.46	12.78	187.88	186.87	186.14
OsR498G0102622000.01	2.163	0.436	-2.31909	2.35E-27	2.48E-27	1.87	2.61	2.01	0.38	0.31	0.62
OsR498G0102622700.01	85.32	237.57	1.475795	0	0	80.34	88.31	87.31	234.38	235.74	242.59
OsR498G0102623300.01	0.2	0.543	1.4073694	7.33E-07	1.28E-06	0.18	0.18	0.24	0.53	0.57	0.53
OsR498G0102624400.01	0.416	10.363	4.5526778	5.55E-97	2.85E-97	0.19	0.51	0.55	11.57	9.97	9.55
OsR498G0102627800.01	22.64	10.713	-1.0824	1.35E-77	8.04E-78	23.28	21.53	23.11	10.83	10.66	10.65
OsR498G0102630300.01	1.136	3.24	1.5494228	7.61E-23	8.64E-23	1.18	0.92	1.31	3.85	3.35	2.52
OsR498G0102634000.01	2.793	6.803	1.2831224	2.32E-46	1.86E-46	2.79	2.89	2.7	7.08	6.58	6.75
OsR498G0102635500.01	54.943	125.28	1.1937929	0	0	55.11	54.83	54.89	123.89	123.3	128.65
OsR498G0102636800.01	0.203	1.153	2.8268528	6.52E-12	9.48E-12	0.17	0.32	0.12	1.58	0.99	0.89
OsR498G0102639100.01	9.103	32.25	1.8165303	1.30E-28	1.34E-28	10.31	6.4	10.6	29.26	34.35	33.14
OsR498G0102640200.01	2.876	5.983	1.0294513	3.36E-16	4.38E-16	2.56	2.88	3.19	6.33	5.26	6.36
OsR498G0102642800.01	37.11	20.443	-1.023375	0	0	39.36	36.81	35.16	20.87	20.5	19.96
OsR498G0102646200.01	19.88	40.403	1.0036645	6.11E-132	2.54E-132	19.45	19.08	21.11	39.39	40.34	41.48
OsR498G0102646700.01	2.896	6.413	1.1487401	4.13E-13	5.81E-13	3.26	3.35	2.08	5.76	5.74	7.74
OsR498G0102648200.01	3.883	9.173	1.2368928	7.79E-29	8.00E-29	3.92	3.94	3.79	8.71	8.52	10.29
OsR498G0102648800.01	11.48	30.27	1.4007941	2.03E-190	6.32E-191	10.8	11.64	12	30.88	30.4	29.53
OsR498G0102649000.01	20.103	68.656	1.7673522	0	0	22.01	18.57	19.73	66.35	63.63	75.99
OsR498G0102650800.01	0.276	1.3	1.9382057	1.01E-10	1.53E-10	0.31	0.17	0.35	1.02	1.19	1.69
OsR498G0102654200.01	3.396	0.886	-1.944453	1.98E-26	2.11E-26	3.81	2.93	3.45	0.82	0.78	1.06
OsR498G0102655100.01	24.05	78.33	1.7157313	0	0	25.11	24.47	22.57	78.24	79.24	77.51
OsR498G0102657500.01	18.926	58.296	1.6196694	0	0	19.63	19.21	17.94	59.22	57.89	57.78
OsR498G0102666800.01	1.366	0.596	-1.190928	1.46E-09	2.28E-09	1.52	1.35	1.23	0.61	0.81	0.37
OsR498G0102667500.01	1.876	6.096	1.7188507	1.33E-23	1.49E-23	2.15	1.78	1.7	5.15	6.61	6.53
OsR498G0102667700.01	0.056	0.253	2.1017092	1.37E-07	2.32E-07	0.04	0.09	0.04	0.29	0.24	0.23
OsR498G0102668500.01	2.696	11.123	2.0462006	1.70E-133	6.97E-134	2.63	2.68	2.78	12.06	10.67	10.64
OsR498G0102668700.01	0.583	0.243	-1.239715	9.15E-05	0.0001771	0.66	0.5	0.59	0.27	0.18	0.28
OsR498G0102672000.01	1.6	6.753	1.7407533	1.36E-64	9.02E-65	1.64	1.56	1.6	6.15	7.5	6.61
OsR498G0102674100.01	10.013	0.326	-4.835396	1.77E-137	7.11E-138	10.45	9.77	9.82	0.17	0.33	0.48
OsR498G0102674300.01	0.176	0.55	1.6442614	0.0001813	0.0003571	0.19	0.05	0.29	0.33	0.6	0.72
OsR498G0102681100.01	30.42	62.64	1.0511989	0	0	31.3	30.29	29.67	61.43	63.49	63
OsR498G0102681300.01	4.486	19.446	2.1157658	6.51E-127	2.78E-127	4.57	4.53	4.36	18.38	19.01	20.95
OsR498G0102682500.01	5.16	38.85	3.0873396	0	0	4.52	4.23	6.73	38.52	39.26	38.77
OsR498G0102686000.01	0.516	10.65	4.3687465	4.73E-157	1.72E-157	0.64	0.14	0.77	10.2	10.95	10.8
OsR498G0102686200.01	0.92	2.936	1.6928034	3.34E-25	3.64E-25	0.79	1.09	0.88	3.22	2.83	2.76
OsR498G0102686800.01	0.096	1.166	3.5459519	3.13E-22	3.60E-22	0.1	0.06	0.13	1.11	0.96	1.43
OsR498G0102687000.01	2.93	7.486	1.3744273	5.95E-49	4.63E-49	3.27	2.69	2.83	8.37	6.26	7.83
OsR498G0102687600.01	0.433	4.396	3.417582	1.14E-77	6.80E-78	0.3	0.45	0.55	3.71	4.49	4.99
OsR498G0102688000.01	0.123	9.623	6.2918402	2.25E-191	6.97E-192	0.12	0.07	0.18	8.7	10.11	10.06
OsR498G0102688200.01	36.066	73.19	1.0210699	5.69E-261	1.34E-261	35.71	35	37.49	74.24	73.58	71.75
OsR498G0102692900.01	1.863	4.863	1.3894475	1.83E-33	1.74E-33	2.14	1.83	1.62	4.85	4.08	5.66
OsR498G0102694000.01	2.09	9.626	2.1888269	2.92E-162	1.04E-162	2.12	2.38	1.77	9.89	9.25	9.74
OsR498G0102699200.01	1.276	3.916	1.6583143	1.17E-29	1.18E-29	1.4	1.29	1.14	3.28	4.45	4.02
OsR498G0102700100.01	14.303	73.426	2.3588709	0	0	15.15	13.87	13.89	71.23	70.95	78.1
OsR498G0102700400.01	1.283	15.353	3.58259	3.88E-87	2.14E-87	0.75	3.1	0	13.4	21.94	10.72
OsR498G0102700800.01	141.493	345.95	1.2877678	0	0	137.33	142.37	144.78	335.03	327.38	375.45
OsR498G0102701900.01	1.99	10.5	2.3917398	2.85E-165	1.00E-165	2.04	1.95	1.98	10.22	10.64	10.64

OsR498G0102702500.01	0.176	2.333	3.7216439	2.10E-20	2.50E-20	0	0.15	0.38	2.51	2.06	2.43
OsR498G0102702900.01	0.33	3.98	3.6048871	6.68E-33	6.42E-33	0.46	0.45	0.08	4.42	3.89	3.63
OsR498G0102704000.01	6.786	14.203	1.0828324	1.03E-38	9.12E-39	7.45	6.29	6.62	14.68	13.48	14.45
OsR498G0102704700.01	0.243	0.04	-3.417907	1.09E-05	2.00E-05	0.22	0.35	0.16	0	0.05	0.07
OsR498G0102705300.01	2.896	6.053	1.0618728	1.62E-24	1.78E-24	3.04	2.79	2.86	5.63	5.92	6.61
OsR498G0102710200.01	1.193	0.296	-1.685515	2.23E-08	3.64E-08	1.28	0.88	1.42	0.44	0.36	0.09
OsR498G0102712500.01	0.1	0.24	1.2953358	0.0004484	0.0009065	0.08	0.16	0.06	0.28	0.18	0.26
OsR498G0102712800.01	8.336	21.61	1.423187	1.53E-132	6.33E-133	9.3	7.73	7.98	20.36	20.54	23.93
OsR498G0102717800.01	1.056	5.503	2.3618119	2.03E-76	1.21E-76	1.04	1.08	1.05	5.12	5.53	5.86
OsR498G0102720600.01	2	0.596	-1.748056	3.05E-19	3.72E-19	2.12	1.98	1.9	0.55	0.59	0.65
OsR498G0102721000.01	1.046	72.293	6.1117353	0	0	1.1	1.09	0.95	73.45	68.38	75.05
OsR498G0102722700.01	0.51	8.423	4.04814	1.58E-68	1.01E-68	0.81	0.32	0.4	7.2	7.8	10.27
OsR498G0102723000.01	0	0.623	4.7879346	6.92E-05	0.0001328	0	0	0	0.53	0.66	0.68
OsR498G0102723100.01	16.876	44.713	1.3989468	0	0	17.11	16.62	16.9	46.02	43.24	44.88
OsR498G0102726000.01	0.163	1.12	2.802229	9.46E-13	1.34E-12	0.29	0.1	0.1	1.36	0.84	1.16
OsR498G0102726800.01	2.213	5.706	1.362164	4.66E-38	4.16E-38	2.34	2.03	2.27	5.97	5.2	5.95
OsR498G0102730100.01	0.02	0.576	5.3246714	3.16E-30	3.17E-30	0	0.03	0.03	0.52	0.45	0.76
OsR498G0102733900.01	0.323	1.353	2.085054	2.08E-34	1.95E-34	0.18	0.44	0.35	1.37	1.49	1.2
OsR498G0102734800.01	33.553	100.53	1.5673505	0	0	34.42	32.96	33.28	99.59	101.79	100.21
OsR498G0102736100.01	29.063	64.57	1.1515638	0	0	27.54	29.41	30.24	63	67.83	62.88
OsR498G0102736400.01	12.283	6.46	-1.006944	1.29E-34	1.20E-34	12.34	13.09	11.42	7.14	6.08	6.16
OsR498G0102736500.01	0.973	2.306	1.3181659	1.35E-21	1.57E-21	1.13	0.86	0.93	2.57	2.08	2.27
OsR498G0102737000.01	0.08	36.896	8.9051473	1.44E-184	4.62E-185	0.12	0.12	0	34.14	38.04	38.51
OsR498G0102737100.01	0.066	11.403	7.3795514	7.65E-76	4.60E-76	0.1	0.1	0	13.34	10.09	10.78
OsR498G0102738500.01	0.08	0.523	2.7185711	8.27E-12	1.21E-11	0.17	0.05	0.02	0.56	0.56	0.45
OsR498G0102738900.01	7.66	194.97	4.6655719	0	0	6.77	7.47	8.74	179.96	201.47	203.47
OsR498G0102739900.01	12.353	35.13	1.5060038	2.96E-105	1.44E-105	13.33	12.29	11.44	35.61	32.02	37.76
OsR498G0102740300.01	0.783	4.743	2.582705	6.35E-81	3.69E-81	0.64	0.87	0.84	4.82	4.98	4.43
OsR498G0102742000.01	33.493	80.816	1.2623788	0	0	34.03	33.32	33.13	81.8	83.13	77.52
OsR498G0102742200.01	0.066	0.506	3.0100339	1.46E-08	2.37E-08	0.04	0.08	0.08	0.51	0.59	0.42
OsR498G0102742400.01	1.203	4.093	1.7614162	3.41E-17	4.34E-17	1.14	1.27	1.2	3.94	3.51	4.83
OsR498G0102743100.01	7.45	33.29	2.1637621	0	0	7.16	7.85	7.34	31.97	34.32	33.58
OsR498G0102744500.01	0	1.316	6.8073	9.39E-15	1.27E-14	0	0	0	1.03	1.37	1.55
OsR498G0102744800.01	5.773	13.153	1.1879179	1.07E-69	6.76E-70	6.05	5.78	5.49	13.37	12.53	13.56
OsR498G0102745800.01	1.096	9.136	3.0874726	3.70E-69	2.34E-69	1.3	0.96	1.03	8.7	8.99	9.72
OsR498G0102746100.01	0.213	1.196	2.4501434	1.17E-13	1.62E-13	0.16	0.28	0.2	1.16	0.95	1.48
OsR498G0102748300.01	0.046	0.243	2.3894475	0.0009846	0.0020515	0.05	0.09	0	0.5	0.18	0.05
OsR498G0102749600.01	0.183	0	-4.485022	0.0003787	0.0007614	0.25	0.15	0.15	0	0	0
OsR498G0102750100.01	3.983	2.196	-1.321791	1.35E-32	1.30E-32	4.7	3.65	3.6	2.81	1.9	1.88
OsR498G0102750400.01	11.55	35.633	1.6268004	2.72E-155	9.97E-156	12.77	10.95	10.93	34.5	35.85	36.55
OsR498G0102755800.01	67.95	379.11	2.4728635	0	0	66.24	67.89	69.72	374.77	382.39	380.18
OsR498G0102757100.01	0.78	6.44	3.0430138	1.39E-109	6.56E-110	0.55	0.9	0.89	6.74	6.13	6.45
OsR498G0102760100.01	0.39	1.08	1.4598368	2.65E-08	4.36E-08	0.3	0.33	0.54	1.05	1.08	1.11
OsR498G0102760300.01	2.25	6.54	1.6453457	2.45E-59	1.70E-59	2.62	2.05	2.08	7.28	5.62	6.72
OsR498G0102761500.01	0.843	1.906	1.1859141	2.67E-10	4.08E-10	0.92	0.69	0.92	1.83	1.92	1.97
OsR498G0102764600.01	1.73	7.216	2.0557983	2.76E-27	2.92E-27	1.86	1.57	1.76	7.52	6.23	7.9
OsR498G0102767200.01	0.246	0.61	1.3154469	0.0007909	0.0016307	0.1	0.4	0.24	0.64	0.66	0.53
OsR498G0102770700.01	21.233	53.446	1.3310333	1.38E-175	4.61E-176	19.31	22.72	21.67	57.06	49.56	53.72
OsR498G0102771100.01	4.72	24.286	2.3617847	2.61E-122	1.14E-122	4.95	4.29	4.92	22.6	24.41	25.85
OsR498G0102772400.01	0.286	0.826	1.5593725	4.73E-09	7.54E-09	0.21	0.35	0.3	0.66	0.86	0.96
OsR498G0102773200.01	63.566	155.32	1.2603212	0	0	64.78	62.79	63.13	159.33	158.91	147.72
OsR498G0102774400.01	0.55	1.823	1.5689585	5.39E-13	7.59E-13	0.64	0.53	0.48	1.71	1.83	1.93
OsR498G0102774800.01	7.976	17.093	1.0605666	2.41E-46	1.93E-46	8.27	7.48	8.18	15.83	16.69	18.76
OsR498G0102775500.01	0.463	4.12	3.149042	9.83E-55	7.17E-55	0.45	0.41	0.53	4.04	4.7	3.62
OsR498G0102776000.01	0.273	3.136	3.5324054	4.25E-42	3.59E-42	0.23	0.36	0.23	3.13	2.89	3.39
OsR498G0102779300.01	19.083	0.55	-5.25334	0	0	18.74	20.82	17.69	0.84	0.33	0.48
OsR498G0102780000.01	30.766	86.41	1.4862931	0	0	30.31	31.58	30.41	85.82	87	86.41
OsR498G0102781100.01	0.156	0.546	2.206856	0.0004797	0.0009717	0.22	0.11	0.14	0.46	0.88	0.3
OsR498G0102781300.01	147.413	24.473	-2.595146	0	0	145.94	150.55	145.75	23.04	23.4	26.98
OsR498G0102782500.01	5.33	1.833	-1.547543	6.47E-35	6.03E-35	4.88	5.54	5.57	1.71	1.53	2.26
OsR498G0102782800.01	1.06	0.306	-1.730134	2.04E-09	3.21E-09	1.07	0.89	1.22	0.36	0.16	0.4
OsR498G0102782900.01	3.146	0.93	-1.733409	2.58E-10	3.95E-10	2.78	3.66	3	0.95	0.97	0.87
OsR498G0102783200.01	8.473	25.8	1.5736144	1.15E-190	3.57E-191	8.17	9.02	8.23	25.84	25.41	26.15
OsR498G0102784200.01	0.376	8.24	4.4501434	2.84E-138	1.14E-138	0.41	0.32	0.4	7.67	8.35	8.7
OsR498G0102787800.01	77.346	17.386	-2.155279	0	0	74.6	74.93	82.51	15.46	17.66	19.04
OsR498G0102789000.01	0	0.816	5.144335	6.91E-06	1.26E-05	0	0	0	0.54	0.81	1.1
OsR498G0102789100.01	0.18	0.73	1.9967778	2.89E-15	3.84E-15	0.12	0.28	0.14	0.57	0.8	0.82
OsR498G0102789400.01	4.113	10.2	1.2545179	5.57E-44	4.59E-44	4.57	3.75	4.02	9.84	10.63	10.13
OsR498G0102790200.01	1.213	3.806	1.6508708	4.33E-16	5.66E-16	1.44	1.23	0.97	3.61	4.1	3.71

OsR498G0102791300.01	14.85	33.036	1.1121784	4.18E-133	1.72E-133	15.76	14.4	14.39	34.07	34.26	30.78
OsR498G0102791900.01	0.08	0.403	2.3249072	3.71E-08	6.12E-08	0.02	0.17	0.05	0.35	0.35	0.51
OsR498G0102795600.01	8.553	20.08	1.1101431	2.61E-47	2.07E-47	9.08	7.54	9.04	19.6	20.43	20.21
OsR498G0102795800.01	1.52	0.646	-1.2491	1.42E-06	2.50E-06	1.03	1.67	1.86	0.72	0.18	1.04
OsR498G0202796800.01	3.79	8.803	1.2257441	4.88E-53	3.63E-53	4.3	3.19	3.88	8.72	8.54	9.15
OsR498G0202798700.01	4.526	2.17	-1.062302	5.43E-10	8.40E-10	3.98	4.69	4.91	2.73	1.66	2.12
OsR498G0202799200.01	0.94	1.99	1.085054	5.15E-08	8.54E-08	0.62	1.18	1.02	1.95	2.06	1.96
OsR498G0202803900.01	16.53	4.606	-1.866247	7.26E-145	2.79E-145	17.04	15.72	16.83	3.83	5.15	4.84
OsR498G0202804800.01	0.683	2.82	2.0434818	8.33E-37	7.56E-37	0.61	0.67	0.77	2.46	2.84	3.16
OsR498G0202805400.01	10.703	32.726	1.612252	1.92E-121	8.40E-122	10.78	10.3	11.03	32.97	34.96	30.25
OsR498G0202807400.01	0.47	1.033	1.1543736	0.0003611	0.0007245	0.74	0.47	0.2	1.87	1.12	0.11
OsR498G0202807600.01	880.923	362.56	-1.266668	0	0	882.02	849.6	911.15	349.62	360.53	377.52
OsR498G0202807800.01	0.313	1.866	2.5832192	2.81E-36	2.56E-36	0.28	0.26	0.4	1.94	2	1.66
OsR498G0202810200.01	0.703	0	-6.273518	9.05E-11	1.36E-10	0.61	0.87	0.63	0	0	0
OsR498G0202813500.01	5.526	13.093	1.2258164	5.68E-81	3.30E-81	5.48	6.41	4.69	14.18	13.16	11.94
OsR498G0202814300.01	2.52	5.643	1.1616215	1.45E-23	1.62E-23	2.57	2.69	2.3	5.85	5.25	5.83
OsR498G0202815200.01	0.316	1.586	2.2963381	2.53E-20	3.01E-20	0.34	0.27	0.34	1.85	1.48	1.43
OsR498G0202815400.01	33.583	188.8	2.4880831	0	0	33.71	33.17	33.87	192.21	189.25	184.95
OsR498G0202817800.01	0.066	0.31	2.2963381	6.15E-08	1.02E-07	0.06	0.06	0.08	0.13	0.37	0.43
OsR498G0202819600.01	1.73	0.2	-3.129927	2.32E-24	2.57E-24	2.02	1.24	1.93	0.24	0.16	0.2
OsR498G0202832500.01	3.826	0.48	-2.993169	1.06E-60	7.29E-61	3.74	3.34	4.4	0.38	0.44	0.62
OsR498G0202833200.01	0.43	1.28	1.5676401	9.17E-15	1.24E-14	0.18	0.71	0.4	1.5	1.09	1.25
OsR498G0202833400.01	1.383	0.05	-4.822603	7.14E-34	6.76E-34	1.26	1.17	1.72	0.06	0.03	0.06
OsR498G0202835200.01	0.16	2.203	3.8131097	8.63E-48	6.81E-48	0.1	0.2	0.18	2.27	1.93	2.41
OsR498G0202835300.01	8.55	44.786	2.3836452	3.79E-225	1.02E-225	9.81	7.55	8.29	44.78	42.33	47.25
OsR498G0202842400.01	2.156	5.12	1.2779985	2.55E-27	2.69E-27	2.65	1.89	1.93	5.36	4.92	5.08
OsR498G0202843500.01	1.9	7.673	2.007942	7.86E-88	4.33E-88	2.1	1.82	1.78	7.61	8.14	7.27
OsR498G0202846700.01	11	66.283	2.5918279	0	0	11.37	10.37	11.26	66.54	67.86	64.45
OsR498G0202849600.01	6.33	19.986	1.657292	5.71E-170	1.95E-170	6.32	6.9	5.77	20.69	20.12	19.15
OsR498G0202850900.01	0.05	0.23	2.4979719	7.77E-05	0.0001495	0.05	0.03	0.07	0.3	0.15	0.24
OsR498G0202851500.01	5.19	10.49	1.0063528	2.94E-32	2.86E-32	5.52	5.03	5.02	10.94	10.29	10.24
OsR498G0202851700.01	8.486	18.846	1.1560168	2.90E-92	1.54E-92	8.79	8.35	8.32	19.02	19.23	18.29
OsR498G0202852100.01	1.546	4.43	1.5083139	5.03E-58	3.55E-58	1.49	1.47	1.68	4.18	5.02	4.09
OsR498G0202854300.01	15.686	79.583	2.3398274	0	0	16.4	15.82	14.84	80.01	80.41	78.33
OsR498G0202858300.01	2.38	0.553	-2.102949	7.54E-30	7.61E-30	1.99	2.51	2.64	0.5	0.5	0.66
OsR498G0202859900.01	0.246	8.293	5.0692335	4.90E-182	1.59E-182	0.19	0.26	0.29	8.32	7.59	8.97
OsR498G0202860400.01	0.25	0.586	1.2963381	0.0006801	0.0013943	0.24	0.24	0.27	0.56	0.67	0.53
OsR498G0202860800.01	1.723	5.28	1.6724232	5.01E-59	3.50E-59	1.87	1.25	2.05	5.48	5.22	5.14
OsR498G0202862500.01	0.156	1.69	3.5420945	1.23E-17	1.55E-17	0.06	0.3	0.11	1.77	1.12	2.18
OsR498G0202863800.01	2.07	0.45	-2.200364	1.40E-19	1.70E-19	2.39	2.07	1.75	0.37	0.4	0.58
OsR498G0202864000.01	1.68	5.34	1.6725831	1.56E-40	1.35E-40	1.69	1.74	1.61	5.6	5.75	4.67
OsR498G0202864400.01	10.613	21.663	1.0301935	1.49E-85	8.31E-86	10.69	10.43	10.72	21.58	22.7	20.71
OsR498G0202865800.01	3.67	7.83	1.120653	8.35E-40	7.30E-40	4.11	3.21	3.69	8.11	7.44	7.94
OsR498G0202867800.01	29.293	4.23	-2.757157	0	0	29.15	28.33	30.4	4.44	4.29	3.96
OsR498G0202869700.01	0.886	1.783	1.0071255	4.61E-10	7.11E-10	0.69	0.97	1	1.55	2.01	1.79
OsR498G0202870800.01	15.843	36.8	1.2191639	6.41E-122	2.80E-122	14.71	16.26	16.56	37.62	37.13	35.65
OsR498G0202871600.01	21.933	51.703	1.2343546	1.81E-102	8.91E-103	23.59	20.45	21.76	53.16	43.16	58.79
OsR498G0202873700.01	3.85	17.21	2.1613912	0	0	3.91	3.56	4.08	16.78	17.83	17.02
OsR498G0202874000.01	7.603	15.19	1.0101044	6.26E-78	3.71E-78	7.72	8.01	7.08	15.24	15.39	14.94
OsR498G0202874400.01	13.49	37.403	1.4270898	2.41E-176	8.02E-177	13.64	13.39	13.44	38.01	36.94	37.26
OsR498G0202875100.01	2.723	10.47	1.9526056	2.28E-41	1.95E-41	2.87	3.34	1.96	10.71	10.16	10.54
OsR498G0202878700.01	4.993	25.086	2.333571	1.10E-198	3.32E-199	5.53	5.17	4.28	23.93	26.29	25.04
OsR498G0202882700.01	8.3	105.45	3.662306	0	0	8.05	8.64	8.21	107.57	104.83	103.94
OsR498G0202882900.01	0.563	1.436	1.4405863	2.53E-19	3.08E-19	0.54	0.52	0.63	1.66	1.3	1.35
OsR498G0202885300.01	0.463	1.733	1.8919478	5.12E-28	5.33E-28	0.29	0.54	0.56	1.65	1.85	1.7
OsR498G0202885600.01	6.056	13.13	1.0905679	1.48E-46	1.19E-46	6.31	5.4	6.46	12.74	12.56	14.09
OsR498G0202886100.01	3.48	12.716	1.866462	3.80E-77	2.27E-77	3.94	3.16	3.34	12.46	11.54	14.15
OsR498G0202895000.01	3.923	1.493	-1.308368	1.47E-19	1.78E-19	4.21	4.31	3.25	1.09	1.31	2.08
OsR498G0202895300.01	2.563	6.46	1.3211023	1.58E-50	1.21E-50	1.97	3.19	2.53	6.77	6.53	6.08
OsR498G0202897100.01	18.27	314.08	4.1008904	0	0	17.66	17.14	20.01	298.07	297.85	346.32
OsR498G0202897600.01	1.31	3.893	1.5680897	4.22E-14	5.78E-14	1.22	1.14	1.57	4.04	2.99	4.65
OsR498G0202901300.01	1.09	2.26	1.044312	1.84E-13	2.55E-13	1.15	0.92	1.2	2.32	2.19	2.27
OsR498G0202903900.01	1.623	5.913	1.8627646	3.12E-18	3.88E-18	1.77	1.45	1.65	5.87	5.75	6.12
OsR498G0202905400.01	0.21	0.58	1.5120667	4.35E-08	7.19E-08	0.3	0.18	0.15	0.45	0.74	0.55
OsR498G0202905600.01	3.196	1.553	-1.042868	3.00E-15	3.99E-15	3.16	3.35	3.08	1.78	1.39	1.49
OsR498G0202907000.01	99.296	45.673	-1.093532	5.36E-220	1.47E-220	99.06	98.2	100.63	48.15	45.53	43.34
OsR498G0202911600.01	0.246	0.68	1.4691746	0.0009479	0.0019719	0.2	0.27	0.27	0.33	1.04	0.67
OsR498G0202912200.01	0.153	0.473	1.6849033	1.12E-07	1.89E-07	0.12	0.14	0.2	0.53	0.44	0.45
OsR498G0202912300.01	38.043	93.45	1.2943863	0	0	36.84	37.87	39.42	93.8	92.15	94.4

OsR498G0202912700.01	5.2	20.873	1.9579218	1.72E-220	4.71E-221	5.1	4.88	5.62	20.63	20.28	21.71
OsR498G0202914200.01	14.64	71.006	2.2823312	0	0	13.42	16.24	14.26	72.38	68.81	71.83
OsR498G0202916400.01	6.133	22.526	1.9229302	1.05E-247	2.63E-248	6.19	6.36	5.85	21.84	24.97	20.77
OsR498G0202917300.01	6.58	28.536	2.1122737	0	0	6.61	6.56	6.57	28.92	28.1	28.59
OsR498G0202918500.01	0.683	2.76	2.0062187	1.55E-68	9.92E-69	0.67	0.79	0.59	2.79	2.88	2.61
OsR498G0202921500.01	4.413	0.843	-2.384044	4.51E-28	4.70E-28	4.19	3.83	5.22	0.71	1.29	0.53
OsR498G0202925400.01	70.29	180.7	1.3900801	0	0	72.22	67.16	71.49	187.31	174.4	180.4
OsR498G0202926700.01	0.456	2.236	2.2891065	2.84E-26	3.05E-26	0.62	0.34	0.41	2.31	2.4	2
OsR498G0202929500.01	5.73	11.593	1.0137535	1.55E-50	1.18E-50	5.71	5.55	5.93	11.15	11.94	11.69
OsR498G0202932700.01	0.216	3.613	4.2426409	7.15E-64	4.78E-64	0.41	0.1	0.14	2.93	3.87	4.04
OsR498G0202933600.01	16.76	48.223	1.5051631	7.99E-189	2.51E-189	17.21	16.89	16.18	49.42	46.22	49.03
OsR498G0202933800.01	1.046	2.49	1.2755795	8.80E-17	1.13E-16	1	1.01	1.13	1.94	2.64	2.89
OsR498G0202937800.01	26.42	10.23	-1.445637	1.85E-200	5.54E-201	26.04	25.25	27.97	10.16	9.63	10.9
OsR498G0202938400.01	0.223	0.033	-2.762556	8.51E-05	0.0001642	0.1	0.33	0.24	0.07	0.03	0
OsR498G0202938500.01	1.44	3.013	1.0675194	2.65E-08	4.34E-08	1.39	1.49	1.44	3.03	3.13	2.88
OsR498G0202938800.01	4.113	0.426	-3.034052	1.24E-117	5.57E-118	4.5	3.53	4.31	0.37	0.54	0.37
OsR498G0202940300.01	0.056	4.153	6.154319	3.62E-69	2.29E-69	0.09	0.04	0.04	4.36	4.85	3.25
OsR498G0202941400.01	1.92	5.553	1.5468507	2.20E-88	1.21E-88	1.9	2.01	1.85	5.23	6.02	5.41
OsR498G0202945800.01	0.74	1.76	1.243345	1.92E-09	3.02E-09	0.73	0.76	0.73	1.78	1.49	2.01
OsR498G0202950300.01	0.536	1.48	1.5164098	1.01E-29	1.02E-29	0.47	0.51	0.63	1.42	1.62	1.4
OsR498G0202953500.01	0.053	0.166	1.696876	0.0001991	0.0003928	0.06	0.1	0	0.15	0.24	0.11
OsR498G0202958200.01	3.626	7.85	1.1492438	1.23E-35	1.13E-35	3.86	3.48	3.54	7.79	7.17	8.59
OsR498G0202961100.01	1	3.43	1.7067142	1.21E-27	1.26E-27	1.04	0.75	1.21	3.8	3.02	3.47
OsR498G0202966500.01	17.38	37.676	1.1053911	3.84E-206	1.12E-206	17.79	17.67	16.68	37.5	38.85	36.68
OsR498G0202966700.01	18.093	39.463	1.127253	5.05E-180	1.65E-180	17.37	18.32	18.59	39.81	39.74	38.84
OsR498G0202967300.01	12.133	42.413	1.8035654	0	0	12.17	12.14	12.09	43.67	42.58	40.99
OsR498G0202967600.01	1.026	2.316	1.172981	2.57E-09	4.06E-09	0.85	1.29	0.94	1.61	2.85	2.49
OsR498G0202967700.01	14.24	30.073	1.0251097	3.21E-96	1.66E-96	15.34	11.22	16.16	29.08	29.15	31.99
OsR498G0202969600.01	0.21	0.45	1.0981319	3.76E-05	7.10E-05	0.14	0.27	0.22	0.48	0.32	0.55
OsR498G0202976900.01	0.136	0.41	1.6024412	0.0002771	0.0005524	0.15	0.11	0.15	0.32	0.39	0.52
OsR498G0202979100.01	0.436	0.023	-4.273518	6.73E-06	1.22E-05	0.56	0.41	0.34	0	0.07	0
OsR498G0202980900.01	0.533	1.183	1.1484394	8.97E-06	1.64E-05	0.58	0.53	0.49	1.28	1.24	1.03
OsR498G0202983400.01	1.16	3.066	1.3724943	4.99E-36	4.58E-36	1.14	1.26	1.08	2.57	3.44	3.19
OsR498G0202986300.01	0.11	0.283	1.5593725	8.67E-05	0.0001674	0.1	0.17	0.06	0.24	0.38	0.23
OsR498G0202988400.01	1.693	18.273	3.4853719	9.83E-216	2.74E-216	1.12	2.28	1.68	18.89	17.97	17.96
OsR498G0202988500.01	0.376	0.093	-1.879739	2.60E-06	4.64E-06	0.27	0.55	0.31	0.15	0.04	0.09
OsR498G0202990800.01	0.55	4.563	3.0653325	4.84E-142	1.89E-142	0.55	0.49	0.61	4.32	4.81	4.56
OsR498G0202991600.01	0.966	4.273	2.1434775	6.60E-97	3.39E-97	0.84	0.94	1.12	4.89	4.11	3.82
OsR498G0202998200.01	0.536	10.95	4.3441848	2.86E-194	8.77E-195	0.3	0.6	0.71	10.41	10.91	11.53
OsR498G0203001700.01	1.246	0.07	-4.126769	9.11E-15	1.23E-14	1.37	0.81	1.56	0	0.16	0.05
OsR498G0203003800.01	0.113	0	-4.485022	0.0003787	0.0007614	0.18	0	0.16	0	0	0
OsR498G0203003900.01	0.596	0.1	-2.610553	0.0002631	0.0005232	0.8	0.3	0.69	0.1	0.1	0.1
OsR498G0203004900.01	1.063	0.213	-2.310992	1.14E-11	1.67E-11	0.78	1.14	1.27	0.24	0.24	0.16
OsR498G0203005400.01	0.406	0.933	1.1968024	3.79E-05	7.16E-05	0.41	0.2	0.61	0.79	0.95	1.06
OsR498G0203005900.01	0.1	0.513	2.3223333	8.82E-09	1.42E-08	0.11	0.11	0.08	0.52	0.41	0.61
OsR498G0203007700.01	38.303	11.853	-1.69574	0	0	39.37	38.27	37.27	11.47	12.19	11.9
OsR498G0203008600.01	1.163	5.99	2.3221221	2.69E-79	1.58E-79	1.11	0.97	1.41	6.3	5.98	5.69
OsR498G0203011500.01	0.056	0.27	2.2963381	2.01E-05	3.75E-05	0.06	0.05	0.06	0.11	0.45	0.25
OsR498G0203019400.01	498.763	1241.6	1.3087282	0	0	482.61	500.38	513.3	1246.3	1223.9	1254.5
OsR498G0203020900.01	0.48	0.043	-3.485022	5.98E-06	1.09E-05	0.66	0.39	0.39	0	0.06	0.07
OsR498G0203021600.01	30.946	86.39	1.4810054	0	0	33.52	30.69	28.63	88.48	86.97	83.72
OsR498G0203023100.01	2.57	81.623	4.9865982	0	0	2.83	0.65	4.23	79.66	74.53	90.68
OsR498G0203030300.01	5.166	28.213	2.4602877	0	0	4.98	4.74	5.78	27.4	29.32	27.92
OsR498G0203032000.01	0.046	2.606	5.7817649	5.50E-28	5.74E-28	0	0.14	0	2.81	1.87	3.14
OsR498G0203036700.01	1.01	2.556	1.33698	6.21E-06	1.13E-05	1.19	1.19	0.65	2.54	2.32	2.81
OsR498G0203043100.01	6.1	20.52	1.7465038	1.21E-43	9.97E-44	5.27	6.53	6.5	21.49	19.02	21.05
OsR498G0203044600.01	0	0.623	5.9286063	5.09E-09	8.13E-09	0	0	0	0.66	0.72	0.49
OsR498G0203045700.01	10.136	22.93	1.1150633	5.24E-110	2.46E-110	9.37	9.89	11.15	23.61	22.93	22.25
OsR498G0203046000.01	22.303	44.43	1.0084401	4.24E-94	2.22E-94	22.16	22.39	22.36	43.9	45.8	43.59
OsR498G0203046700.01	16.163	34.736	1.0953318	2.17E-116	9.83E-117	17.88	15.04	15.57	34.09	35.36	34.76
OsR498G0203051200.01	1.956	4.56	1.2073668	3.80E-38	3.38E-38	1.78	2.41	1.68	4.18	4.8	4.7
OsR498G0203054600.01	1.806	0.106	-4.113053	2.28E-05	4.25E-05	1.27	1.93	2.22	0	0	0.32
OsR498G0203064100.01	2.49	9.72	1.97441	2.21E-119	9.79E-120	2.37	2.84	2.26	9.99	9.88	9.29
OsR498G0203064500.01	2.38	38.87	4.0293636	0	0	2.99	1.99	2.16	35.12	41.02	40.47
OsR498G0203065300.01	1.283	3.71	1.4579272	6.71E-22	7.75E-22	1.02	1.17	1.66	4.54	3.58	3.01
OsR498G0203065800.01	10.66	32.866	1.6202547	1.38E-226	3.71E-227	10.43	10.22	11.33	31.1	33.63	33.87
OsR498G0203068700.01	0.42	0.093	-2.195515	6.44E-05	0.0001235	0.66	0.23	0.37	0.14	0	0.14
OsR498G0203071700.01	0.146	1.04	2.8455596	6.92E-08	1.15E-07	0	0	0.44	0.96	0.68	1.48
OsR498G0203072000.01	11.266	33.86	1.5855929	3.46E-91	1.86E-91	12.46	10.12	11.22	31.8	33	36.78

OsR498G0203072200.01	0.03	0.26	3.2228436	8.90E-07	1.56E-06	0.03	0.03	0.03	0.39	0.19	0.2
OsR498G0203075000.01	1.57	3.463	1.1477416	5.44E-31	5.40E-31	1.6	1.41	1.7	3.76	2.87	3.76
OsR498G0203078000.01	6.026	16.02	1.4155911	1.01E-173	3.39E-174	5.8	6.32	5.96	15.77	16.63	15.66
OsR498G0203081200.01	27.303	73.963	1.4309241	3.75E-145	1.44E-145	28.15	27.62	26.14	78.6	69.04	74.25
OsR498G0203081900.01	4.74	22.37	2.2390127	3.21E-287	7.02E-288	4.52	4.78	4.92	22.74	21.76	22.61
OsR498G0203082200.01	8.903	1.15	-3.035393	6.77E-77	4.04E-77	8.33	9.72	8.66	1.94	0.62	0.89
OsR498G0203085500.01	71.04	234.12	1.7185711	0	0	68.96	72.31	71.85	228.17	229.7	244.48
OsR498G0203089100.01	7.576	17.173	1.1813624	4.27E-76	2.56E-76	7.29	7.85	7.59	17.55	17.42	16.55
OsR498G0203091200.01	8.02	16.44	1.0474436	1.91E-51	1.44E-51	7.56	9.12	7.38	15.98	16.86	16.48
OsR498G0203091300.01	18.17	69.816	1.9220862	0	0	17.41	18.72	18.38	67.29	71.01	71.15
OsR498G0203096300.01	5.483	46.55	3.098711	0	0	5.16	6.23	5.06	46.85	44.92	47.88
OsR498G0203098800.01	0.033	0.183	2.4073694	3.07E-05	5.77E-05	0.06	0.02	0.02	0.16	0.18	0.21
OsR498G0203103600.01	18.333	32.7	1.1306958	1.36E-167	4.69E-168	17.44	19.41	18.15	32.48	32.51	33.11
OsR498G0203105400.01	0.253	0.773	1.8029295	4.32E-11	6.45E-11	0.21	0.26	0.29	0.69	0.69	0.94
OsR498G0203106200.01	8.563	21.583	1.3139404	1.52E-153	5.61E-154	8.98	8.54	8.17	21.96	22.53	20.26
OsR498G0203106300.01	19.623	39.33	1.0014782	6.89E-71	4.30E-71	20.18	18.47	20.22	41.44	38.1	38.45
OsR498G0203106600.01	62.673	24.803	-1.31906	0	0	62.53	62.74	62.75	24.6	24.99	24.82
OsR498G0203108600.01	0.223	0.713	1.6572198	1.22E-06	2.14E-06	0.32	0.21	0.14	0.84	0.69	0.61
OsR498G0203109500.01	2.076	9.083	2.1614085	3.12E-89	1.70E-89	2.1	2.09	2.04	8.92	9.27	9.06
OsR498G0203111900.01	1.966	5.28	1.4203554	8.23E-33	7.91E-33	1.7	1.88	2.32	5.44	5	5.4
OsR498G0203112700.01	7.063	88.853	3.6526197	0	0	6.91	7.9	6.38	90.05	87.06	89.45
OsR498G0203116200.01	5.673	19.956	1.8369859	3.18E-154	1.17E-154	4.99	5.65	6.38	20.46	19.06	20.35
OsR498G0203116700.01	3.226	9.726	1.5972406	8.55E-46	6.88E-46	3.15	3.17	3.36	10.68	8.92	9.58
OsR498G0203117500.01	11.073	33.23	1.5751892	5.76E-145	2.21E-145	11.52	10.07	11.63	32.68	32.29	34.72
OsR498G0203119400.01	73.496	32.973	-1.15433	3.09E-142	1.21E-142	70.37	76.5	73.62	31.31	34.27	33.34
OsR498G0203119900.01	0.276	5.153	4.2096264	3.86E-98	1.96E-98	0.17	0.28	0.38	4.95	5.03	5.48
OsR498G0203120200.01	2.46	57.406	4.5607945	0	0	2.22	2.1	3.06	59.17	57.56	55.49
OsR498G0203122000.01	19.773	43.303	1.1198344	3.36E-174	1.12E-174	19.85	19.1	20.37	42.72	41.2	45.99
OsR498G0203124200.01	0.046	1.106	4.6748497	2.63E-19	3.20E-19	0.1	0	0.04	1.28	1.12	0.92
OsR498G0203126600.01	7.18	17.36	1.2664144	1.57E-46	1.26E-46	7.66	7.15	6.73	16.66	18.34	17.08
OsR498G0203127800.01	3.713	8.373	1.2098104	1.53E-42	1.29E-42	3.9	3.86	3.38	8.31	7.33	9.48
OsR498G0203128900.01	0.086	2.163	4.5949964	1.64E-56	1.17E-56	0.05	0.13	0.08	2.28	2.11	2.1
OsR498G0203129600.01	0.07	0.25	1.8908513	0.0001181	0.0002301	0.15	0	0.06	0.29	0.13	0.33
OsR498G0203132100.01	0.116	0.35	1.6601523	3.90E-06	7.02E-06	0.06	0.12	0.17	0.27	0.4	0.38
OsR498G0203132400.01	20.6	9.133	-1.149957	6.88E-78	4.08E-78	20.47	19.27	22.06	9.33	8.72	9.35
OsR498G0203134100.01	0.06	0.293	2.2963381	2.60E-07	4.44E-07	0.04	0.04	0.1	0.35	0.12	0.41
OsR498G0203134400.01	1.126	3.46	1.5721965	5.19E-27	5.51E-27	1.04	1.31	1.03	3.79	3.37	3.22
OsR498G0203134700.01	0.53	3.836	2.8278613	1.48E-26	1.58E-26	0.65	0.5	0.44	3.37	4.22	3.92
OsR498G0203139800.01	39.993	4	-3.323755	0	0	40.72	38.07	41.19	4.71	2.93	4.36
OsR498G0203139900.01	1.99	6.116	1.6211014	4.53E-61	3.10E-61	2.07	1.73	2.17	5.98	5.95	6.42
OsR498G0203141200.01	8.263	17.456	1.0764985	3.67E-33	3.52E-33	8.08	7.67	9.04	16.65	16.38	19.34
OsR498G0203141800.01	3.456	15.9	2.2592896	9.79E-102	4.86E-102	3.1	3.86	3.41	16.32	15.68	15.7
OsR498G0203143200.01	4.123	11.593	1.5188746	6.62E-237	1.72E-237	4.13	3.96	4.28	11.9	11.9	10.98
OsR498G0203143300.01	10.153	26.736	1.3965655	1.81E-94	9.49E-95	10.32	10.16	9.98	26.19	26.14	27.88
OsR498G0203144000.01	12.753	31.643	1.3215013	7.76E-182	2.52E-182	12.58	13	12.68	32.54	32.22	30.17
OsR498G0203146000.01	43.646	0.31	-7.140902	0	0	42.75	43.68	44.51	0.29	0.38	0.26
OsR498G0203147600.01	4.813	9.87	1.0053234	1.92E-40	1.66E-40	4.79	4.91	4.74	9.58	10.27	9.76
OsR498G0203148600.01	39.303	86.85	1.1381622	1.24E-217	3.45E-218	41.28	38.55	38.08	87	84.32	89.23
OsR498G0203150600.01	5.186	15.593	1.581315	4.63E-180	1.51E-180	5.85	5.16	4.55	15.63	15.13	16.02
OsR498G0203156400.01	3.103	8.513	1.4622313	4.76E-42	4.03E-42	2.66	2.65	4	9.33	8.09	8.12
OsR498G0203156600.01	3962.24	1319.7	-1.582588	0	0	3944.2	3831.2	4111.4	1292.5	1278.8	1387.7
OsR498G0203156800.01	2.796	0.85	-1.696282	5.67E-15	7.60E-15	2.88	2.4	3.11	0.85	1.04	0.66
OsR498G0203158700.01	9.463	22.41	1.2190871	2.16E-88	1.19E-88	8.73	9.15	10.51	23.83	21.13	22.27
OsR498G0203159400.01	0.163	0.323	1.0496981	0.0005112	0.0010377	0.22	0.18	0.09	0.33	0.31	0.33
OsR498G0203160100.01	7.853	32.29	2.0343644	0	0	8.46	7.18	7.92	31.33	33.26	32.28
OsR498G0203160400.01	0.396	1.39	1.832391	1.51E-20	1.80E-20	0.39	0.47	0.33	1.51	1.33	1.33
OsR498G0203161400.01	1.64	5.686	1.7957041	1.85E-34	1.74E-34	2.11	1.52	1.29	5.3	5.69	6.07
OsR498G0203163200.01	6.17	1.863	-1.646067	2.09E-28	2.16E-28	6.56	5.8	6.15	2.17	1.52	1.9
OsR498G0203165000.01	0.39	0.116	-1.79818	2.75E-09	4.35E-09	0.42	0.38	0.37	0.13	0.12	0.1
OsR498G0203169800.01	2.82	13.243	2.2246366	3.33E-76	2.00E-76	2.77	2.24	3.45	12.71	11.19	15.83
OsR498G0203173700.01	15.066	99.383	2.7212058	0	0	16.1	15.52	13.58	100.76	98.67	98.72
OsR498G0203174000.01	2.42	6.97	1.5168309	2.20E-126	9.42E-127	2.87	2.24	2.15	6.25	7.84	6.82
OsR498G0203174600.01	23.633	54.173	1.1961265	3.82E-106	1.84E-106	25.68	23.63	21.59	51.41	54.66	56.45
OsR498G0203175400.01	0.556	1.713	1.6235028	3.95E-07	6.80E-07	0.23	0.53	0.91	2.07	1.55	1.52
OsR498G0203176200.01	73.86	204.7	1.4695811	0	0	62.2	78.31	81.07	206.82	207.97	199.31
OsR498G0203179000.01	2.753	6.46	1.2300917	7.22E-58	5.10E-58	2.68	2.45	3.13	6.58	6.78	6.02
OsR498G0203181500.01	8.89	52.93	2.5267912	0	0	8.35	8.99	9.33	51.84	52.53	54.42
OsR498G0203183800.01	0.03	0.566	4.3201848	5.08E-15	6.80E-15	0.03	0	0.06	0.61	0.52	0.57
OsR498G0203186200.01	6.103	17.01	1.4629734	2.16E-69	1.36E-69	6.22	5.91	6.18	15.77	18.83	16.43

OsR498G0203187400.01	51.166	116.03	1.1805031	0	0	50.6	52.36	50.54	119.94	113.85	114.29
OsR498G0203188000.01	0.663	40.306	5.8959203	0	0	0.67	0.63	0.69	39.95	39.14	41.83
OsR498G0203190000.01	0.883	3.92	2.1615845	7.27E-53	5.41E-53	1.15	0.76	0.74	3.96	4.2	3.6
OsR498G0203190800.01	1.553	8.31	2.4169491	1.51E-110	7.09E-111	1.83	1.31	1.52	9.23	7.81	7.89
OsR498G0203191700.01	0.01	0.136	3.6748497	0.0003128	0.0006259	0	0.03	0	0.09	0.16	0.16
OsR498G0203193100.01	12.19	2.003	-2.605736	3.41E-45	2.76E-45	12.28	11	13.29	2.63	1.55	1.83
OsR498G0203195500.01	1.74	7.283	2.2340651	7.29E-215	2.04E-215	1.87	1.61	1.74	7.38	7.18	7.29
OsR498G0203196000.01	10.516	0.38	-4.788675	6.62E-83	3.78E-83	10.42	11.14	9.99	0.09	0.58	0.47
OsR498G0203197400.01	0.29	0.013	-4.417907	2.01E-06	3.56E-06	0.33	0.29	0.25	0	0	0.04
OsR498G0203199800.01	7.036	22.916	1.7148183	0	0	6.74	6.64	7.73	21.78	24	22.97
OsR498G0203200200.01	12.136	27.513	1.1793753	3.84E-236	1.00E-236	12.48	12.23	11.7	27.31	29.54	25.69
OsR498G0203200600.01	1.09	47.746	5.4533818	3.18E-157	1.15E-157	1.31	1.31	0.65	45.19	47.35	50.7
OsR498G0203200800.01	0.203	5.106	4.7062138	1.41E-43	1.17E-43	0	0.31	0.3	5.09	5.8	4.43
OsR498G0203202200.01	0.46	1.23	1.4188742	0.0006633	0.0013589	0.63	0.64	0.11	1.16	1.67	0.86
OsR498G0203202700.01	0.043	0.176	2.0713804	1.54E-06	2.72E-06	0.05	0.04	0.04	0.14	0.23	0.16
OsR498G0203203700.01	6.27	2.806	-1.161422	5.13E-51	3.90E-51	6.38	6.49	5.94	2.73	2.86	2.83
OsR498G0203205200.01	0.163	0.696	2.0925914	2.18E-21	2.54E-21	0.12	0.23	0.14	0.62	0.72	0.75
OsR498G0203209400.01	0.07	0.263	1.9286063	9.10E-05	0.000176	0.03	0.1	0.08	0.18	0.25	0.36
OsR498G0203210500.01	1.06	2.473	1.2223375	1.60E-08	2.61E-08	1.14	1.3	0.74	2.66	2.54	2.22
OsR498G0203211300.01	0.253	3.15	3.6468353	1.08E-21	1.26E-21	0.19	0.19	0.38	4.05	2.57	2.83
OsR498G0203214300.01	1.36	0.226	-2.417907	7.88E-16	1.04E-15	1.54	0.99	1.55	0.08	0.42	0.18
OsR498G0203216700.01	3.276	8.01	1.3098303	4.63E-23	5.23E-23	3.24	3.42	3.17	7.75	6.42	9.86
OsR498G0203218700.01	0.126	4.653	5.2622762	2.91E-137	1.17E-137	0.09	0.16	0.13	4.26	4.81	4.89
OsR498G0203220700.01	0.423	0.156	-1.440628	1.91E-05	3.55E-05	0.41	0.45	0.41	0.2	0.18	0.09
OsR498G0203220900.01	105.34	162.76	1.1419575	0	0	111.12	106.72	98.18	160.23	161.15	166.89
OsR498G0203222800.01	0.13	5.643	5.4726608	2.11E-54	1.54E-54	0.31	0	0.08	5.43	5.48	6.02
OsR498G0203226300.01	9.836	35.956	1.8593048	0	0	10.04	9.64	9.83	37.07	36.7	34.1
OsR498G0203227300.01	0.323	1.02	1.7077643	6.21E-13	8.76E-13	0.33	0.44	0.2	0.85	1.29	0.92
OsR498G0203230600.01	3.386	9.796	1.4810877	1.15E-56	8.23E-57	3.73	3.1	3.33	9.56	9.85	9.98
OsR498G0203233000.01	51.82	11.203	-2.198638	0	0	53.53	51.81	50.12	11.01	10.88	11.72
OsR498G0203233300.01	40.813	126.51	1.6250877	0	0	40.53	41.2	40.71	127.4	129.76	122.37
OsR498G0203237200.01	20.96	43.026	1.0402662	6.00E-168	2.07E-168	20.85	20.83	21.2	44.94	43.38	40.76
OsR498G0203237300.01	32.046	87.77	1.4240429	0	0	31.28	33.29	31.57	83.4	89.11	90.8
OsR498G0203238300.01	3.693	10.906	1.4911242	2.49E-61	1.70E-61	3.41	3.85	3.82	11.19	11.19	10.34
OsR498G0203240400.01	1.406	2.886	1.0339871	1.89E-10	2.88E-10	1.14	1.62	1.46	3.02	3.1	2.54
OsR498G0203240500.01	3.836	12.426	1.3586034	3.85E-100	1.94E-100	3.35	3.93	4.23	11.99	12.9	12.39
OsR498G0203243500.01	85.653	35.756	-1.287832	8.97E-246	2.26E-246	81.75	82.44	92.77	36.04	33.71	37.52
OsR498G0203254500.01	6.076	22.083	1.8703601	6.95E-179	2.28E-179	6.41	6.34	5.48	23.18	21.96	21.11
OsR498G0203254900.01	19.123	52.523	1.4717247	2.85E-262	6.70E-263	19.77	19.55	18.05	52.71	53.71	51.15
OsR498G0203255400.01	21.333	4.333	-2.300393	1.96E-164	6.91E-165	20.51	21.12	22.37	4.24	4.37	4.39
OsR498G0203255500.01	0.673	0.076	-3.124897	4.57E-08	7.57E-08	0.62	0.47	0.93	0	0.1	0.13
OsR498G0203258100.01	0.336	0.073	-2.195515	6.44E-05	0.0001235	0.19	0.52	0.3	0.14	0.04	0.04
OsR498G0203258800.01	20.42	50.566	1.2928519	5.73E-173	1.93E-173	20.06	22.2	19	48.17	52.47	51.06
OsR498G0203259700.01	7.486	15.2	1.0200068	6.90E-36	6.35E-36	7.35	7.08	8.03	14.62	14.46	16.52
OsR498G0203259900.01	12.86	49.763	1.9508114	0	0	13.92	12.74	11.92	50.87	49.07	49.35
OsR498G0203260200.01	6.466	2.763	-1.230572	1.42E-17	1.79E-17	5.88	6.72	6.8	2.44	2.31	3.54
OsR498G0203262700.01	18.02	4.726	-1.994988	0	0	18	18.08	17.98	4.78	4.77	4.63
OsR498G0203262900.01	1.896	4.523	1.2549063	2.98E-38	2.65E-38	1.96	1.78	1.95	4.54	4.62	4.41
OsR498G0203263200.01	0	0.216	5.0703344	1.16E-05	2.14E-05	0	0	0	0.15	0.29	0.21
OsR498G0203263400.01	18.726	101.32	2.4356049	0	0	18.96	18.6	18.62	100.13	96.73	107.11
OsR498G0203265800.01	2.303	16.16	2.8090557	0	0	2.39	2.26	2.26	17.25	16.13	15.1
OsR498G0203266200.01	1.75	6.11	1.6820417	9.16E-44	7.56E-44	1.69	2.11	1.45	6.68	6.32	5.33
OsR498G0203268600.01	44.08	94.99	1.1102722	0	0	43.68	43.25	45.31	95.78	95.98	93.21
OsR498G0203271400.01	11.5	71.1	2.6225026	0	0	10.12	12.95	11.43	70.93	71.62	70.75
OsR498G0203272500.01	22.063	75.703	1.7603912	0	0	21.16	22.78	22.25	77.14	77.9	72.07
OsR498G0203274600.01	0.55	2.883	2.331962	4.17E-12	6.03E-12	0.84	0.62	0.19	2.83	2.96	2.86
OsR498G0203274700.01	7.663	19.703	1.3571632	3.84E-99	1.94E-99	8.49	7.45	7.05	18.62	20.17	20.32
OsR498G0203282300.01	0.046	0.51	3.4662631	3.18E-10	4.88E-10	0.07	0.07	0	0.54	0.47	0.52
OsR498G0203282600.01	0.146	0.303	1.0524125	0.0006046	0.0012343	0.16	0.2	0.08	0.35	0.25	0.31
OsR498G0203284600.01	2.053	4.893	1.2642303	3.91E-29	3.99E-29	1.82	1.73	2.61	4.58	5.37	4.73
OsR498G0203286800.01	0.683	0.106	-2.72603	1.02E-09	1.60E-09	0.65	0.6	0.8	0.05	0.22	0.05
OsR498G0203293400.01	0.483	0.126	-1.900059	0.0007746	0.0015958	0.53	0.46	0.46	0.06	0.06	0.26
OsR498G0203294900.01	44.146	110.08	1.3124519	0	0	44.59	42.61	45.24	109.88	110.63	109.73
OsR498G0203297900.01	6.846	14.503	1.0836618	2.35E-61	1.60E-61	6.22	6.74	7.58	15.35	14.23	13.93
OsR498G0203300400.01	37.46	92.176	1.2971927	0	0	34.97	40.2	37.21	92.77	93.64	90.12
OsR498G0203302700.01	0.896	0.13	-2.832945	8.48E-12	1.24E-11	1.2	0.79	0.7	0.14	0.05	0.2
OsR498G0203306400.01	2.36	6.246	1.2938907	1.89E-34	1.78E-34	2.52	2.42	2.14	5.63	6.73	6.38
OsR498G0203308700.01	0.736	0.016	-5.517443	1.74E-12	2.48E-12	0.74	0.78	0.69	0	0	0.05
OsR498G0203310700.01	3.18	1	-1.668851	8.59E-27	9.13E-27	3.2	3.13	3.21	0.98	1.1	0.92

OsR498G0203314800.01	0.066	0.866	3.7185711	4.72E-15	6.31E-15	0.12	0.04	0.04	1.08	0.69	0.83
OsR498G0203315000.01	5.583	0.85	-2.737465	6.44E-89	3.51E-89	5.42	5.46	5.87	0.65	0.9	1
OsR498G0203318800.01	15.33	34.686	1.1772673	0	0	15.42	15.75	14.82	35.5	34.51	34.05
OsR498G0203323700.01	1.793	4	1.1396125	1.19E-15	1.57E-15	1.37	2.12	1.89	4.52	3.67	3.81
OsR498G0203336700.01	17.146	56.373	1.6846824	0	0	18.07	16.61	16.76	56.59	53.64	58.89
OsR498G0203340000.01	0.9	12.153	3.7524871	6.29E-89	3.43E-89	0.68	0.84	1.18	10.96	12.4	13.1
OsR498G0203343200.01	3.226	18.786	2.5415612	1.53E-135	6.19E-136	3.04	3.79	2.85	16.86	21.23	18.27
OsR498G0203343400.01	2.233	13.346	2.5775198	2.32E-69	1.46E-69	2.81	2.2	1.69	12.34	13.69	14.01
OsR498G0203345000.01	2.893	12.19	2.0735757	3.09E-62	2.09E-62	3.62	1.66	3.4	11.34	12.72	12.51
OsR498G0203347200.01	0.13	0.81	2.7377097	3.13E-21	3.67E-21	0.13	0.18	0.08	1.1	0.6	0.73
OsR498G0203348200.01	5.05	12.406	1.2963381	3.18E-23	3.59E-23	5.72	4.52	4.91	14.98	10.43	11.81
OsR498G0203351800.01	49.023	17.503	-1.469941	0	0	48.81	49.17	49.09	17.58	18.33	16.6
OsR498G0203352400.01	0.37	5.4	3.8687005	4.05E-71	2.52E-71	0.39	0.48	0.24	5.12	5.62	5.46
OsR498G0203353700.01	39.7	13.266	-1.582092	8.52E-300	1.80E-300	40.33	39.1	39.67	13.09	12.51	14.2
OsR498G0203376500.01	65.146	131.66	1.0114315	1.29E-282	2.87E-283	63.23	68.45	63.76	128.87	133.32	132.8
OsR498G0203376700.01	2.253	4.576	1.0309935	7.54E-16	9.90E-16	2.37	2.42	1.97	4.25	5.26	4.22
OsR498G0203377000.01	5.363	15.06	1.488095	2.56E-65	1.68E-65	5.13	5.37	5.59	14.64	15.3	15.24
OsR498G0203377100.01	0.543	1.22	1.162855	5.57E-11	8.34E-11	0.52	0.6	0.51	1.43	1.22	1.01
OsR498G0203378100.01	0.326	1.03	1.6772089	2.73E-14	3.73E-14	0.29	0.32	0.37	0.81	1.14	1.14
OsR498G0203378400.01	14.936	37.346	1.320707	8.23E-140	3.26E-140	15.45	15.34	14.02	39.01	34.95	38.08
OsR498G0203379300.01	1.046	5.46	2.3870084	1.23E-52	9.17E-53	0.98	0.86	1.3	5.45	4.94	5.99
OsR498G0203385800.01	0.016	0.776	5.5442656	5.55E-24	6.17E-24	0	0.05	0	0.95	0.73	0.65
OsR498G0203389600.01	0.036	0.306	2.97441	2.56E-08	4.19E-08	0.02	0	0.09	0.27	0.34	0.31
OsR498G0203400100.01	18.303	54.013	1.5597153	0	0	18.68	18.73	17.5	56.1	54.14	51.8
OsR498G0203400800.01	1.153	3	1.3729593	1.14E-17	1.44E-17	1	1.23	1.23	3.04	3.2	2.76
OsR498G0203402100.01	1.856	7.336	1.9855505	3.78E-55	2.75E-55	1.65	1.89	2.03	7.11	7.87	7.03
OsR498G0203408600.01	6.693	0.9	-2.895006	8.16E-171	2.77E-171	6.81	6.7	6.57	0.99	0.84	0.87
OsR498G0203410200.01	4.913	1.39	-1.944786	1.47E-70	9.24E-71	4.83	5.72	4.19	1.17	1.69	1.31
OsR498G0203410500.01	1.106	0.286	-1.944972	1.20E-10	1.82E-10	0.92	1.33	1.07	0.3	0.33	0.23
OsR498G0203410800.01	0.18	0	-5.72603	5.93E-08	9.87E-08	0.09	0.19	0.26	0	0	0
OsR498G0203417000.01	162.583	48.49	-1.73349	0	0	168.61	159.46	159.68	47.06	50.27	48.14
OsR498G0203417300.01	13.91	38.453	1.4836858	1.22E-229	3.25E-230	14.83	13.14	13.76	38.02	39.67	37.67
OsR498G0203420100.01	4.136	11.296	1.4532581	3.27E-146	1.25E-146	4.32	3.95	4.14	11.49	11.63	10.77
OsR498G0203420700.01	0.176	6.033	5.1018181	2.08E-178	6.84E-179	0.19	0.17	0.17	5.7	6.58	5.82
OsR498G0203421300.01	1.136	9.446	1.9512927	4.75E-70	2.99E-70	1.06	1.06	1.29	10.24	8.82	9.28
OsR498G0203421800.01	0	0.096	4.6748497	0.0001298	0.0002536	0	0	0	0.04	0.2	0.05
OsR498G0203428600.01	29.143	117.81	2.0134329	0	0	31.08	26.43	29.92	109.61	116.1	127.71
OsR498G0203429400.01	0.686	1.523	1.1512877	0.0004478	0.0009052	0.18	0.54	1.34	1.31	1.74	1.52
OsR498G0203435500.01	3.273	0.103	-5.02559	1.32E-46	1.06E-46	3.46	3.17	3.19	0.05	0.2	0.06
OsR498G0203438600.01	1.366	7.583	2.4698941	3.56E-75	2.15E-75	1.45	1.47	1.18	7.8	7.19	7.76
OsR498G0203438800.01	1.643	4.806	1.5555201	3.53E-29	3.60E-29	1.8	1.64	1.49	4.17	5.14	5.11
OsR498G0203439500.01	3.543	7.603	1.1016097	6.11E-39	5.39E-39	3.7	3.66	3.27	7.56	7.87	7.38
OsR498G0203451100.01	0.22	0.706	1.6264867	3.78E-05	7.14E-05	0.24	0.09	0.33	0.79	0.71	0.62
OsR498G0203456000.01	1.906	7.54	1.8560478	2.02E-77	1.20E-77	1.64	2.29	1.79	7.85	8.18	6.59
OsR498G0203459900.01	0.173	0.57	1.4937841	0.0001153	0.0002245	0.04	0.27	0.21	0.44	0.31	0.96
OsR498G0203476400.01	9.53	35.936	1.9332526	8.78E-248	2.19E-248	10.5	9.74	8.35	36.39	32.41	39.01
OsR498G0203476700.01	0.02	2.486	6.826159	3.95E-54	2.90E-54	0.03	0.03	0	2.37	2.42	2.67
OsR498G0203478900.01	27.103	61.893	1.1838098	0	0	27.73	25.78	27.8	63.74	59.84	62.1
OsR498G0203479900.01	5.136	53.92	3.390834	0	0	4.53	5.43	5.45	54.49	54.15	53.12
OsR498G0203480200.01	0.15	0.923	2.6066782	3.65E-06	6.54E-06	0.18	0.18	0.09	0.8	1.06	0.91
OsR498G0203485500.01	0.273	0.603	1.2114492	5.20E-05	9.90E-05	0.44	0.06	0.32	0.8	0.45	0.56
OsR498G0203486100.01	0.466	0.183	-1.353617	0.0002048	0.0004043	0.59	0.31	0.5	0.15	0.15	0.25
OsR498G0203486400.01	0.28	0.696	1.333491	3.86E-05	7.28E-05	0.33	0.18	0.33	0.64	0.83	0.62
OsR498G0203495200.01	35.816	10.53	-1.776651	5.47E-251	1.34E-251	36.64	35.4	35.41	10.07	10.1	11.42
OsR498G0203498400.01	0.11	1.04	3.2545179	5.41E-26	5.82E-26	0.15	0.07	0.11	1.1	1.01	1.01
OsR498G0203501200.01	5.636	1.593	-1.797784	8.85E-113	4.10E-113	5.57	5.65	5.69	1.41	1.87	1.5
OsR498G0203502900.01	0.29	0.62	1.0854413	0.0006171	0.0012604	0.21	0.35	0.31	0.58	0.44	0.84
OsR498G0203504400.01	2.216	0.56	-1.97668	8.52E-08	1.43E-07	2.76	1.83	2.06	0.34	1	0.34
OsR498G0203510000.01	0.633	1.33	1.0922464	4.65E-06	8.40E-06	0.67	0.58	0.65	1.36	1.41	1.22
OsR498G0203512500.01	23.346	65.146	1.5605725	2.32E-206	6.78E-207	24.46	25.16	20.42	58.41	66.76	70.27
OsR498G0203512900.01	0.08	0.54	2.7532415	0.0001688	0.000332	0.24	0	0	0.63	0.68	0.31
OsR498G0203514900.01	4.043	16.003	1.977604	2.23E-101	1.11E-101	3.44	4.55	4.14	15.69	14.63	17.69
OsR498G0203517300.01	13.953	50.006	1.87265	1.43E-183	4.60E-184	12.59	13.51	15.76	54.41	44.59	51.02
OsR498G0203517500.01	0.206	0	-4.550406	0.0002767	0.0005514	0.22	0.09	0.31	0	0	0
OsR498G0203521400.01	70.05	156.93	1.1652207	0	0	72.44	68.28	69.43	152.4	160.13	158.27
OsR498G0203521900.01	2.666	5.793	1.1179233	7.27E-21	8.58E-21	2.79	2.8	2.41	5.69	5.71	5.98
OsR498G0203525300.01	7.2	15.826	1.1346291	1.80E-48	1.41E-48	7.72	7.18	6.7	15.12	15.1	17.26
OsR498G0203539200.01	1.94	3.843	1.0011273	8.54E-10	1.33E-09	2.17	1.93	1.72	4.04	3.87	3.62
OsR498G0203539800.01	0.626	1.17	1.0188041	9.65E-07	1.69E-06	0.8	0.5	0.58	1.12	1.29	1.1

OsR498G0203542600.01	5.113	45.466	3.1539795	0	0	5.11	4.96	5.27	46.63	47.28	42.49
OsR498G0203544900.01	0.646	7.356	3.5119471	1.03E-148	3.87E-149	0.73	0.4	0.81	7.34	6.86	7.87
OsR498G0203550500.01	0.24	0.796	1.7292975	0.0005682	0.0011565	0.36	0.27	0.09	0.62	1.05	0.72
OsR498G0203559000.01	6.87	0.086	-6.221689	0	0	6.44	6.72	7.45	0.07	0.1	0.09
OsR498G0203564100.01	5.9	12.253	1.1070713	3.95E-55	2.87E-55	4.99	5.79	6.92	12.08	12.81	11.87
OsR498G0203566300.01	1.196	0.5	-1.260055	4.54E-06	8.19E-06	1.22	1.03	1.34	0.39	0.48	0.63
OsR498G0203567200.01	0.156	1.086	2.8813006	5.59E-28	5.83E-28	0.15	0.15	0.17	1.14	1.28	0.84
OsR498G0203567900.01	2.116	4.623	1.1282153	9.97E-14	1.38E-13	1.97	1.9	2.48	4.9	4.73	4.24
OsR498G0203568300.01	7.11	50.536	2.8354138	0	0	7.78	6.41	7.14	50.52	49.25	51.84
OsR498G0203573900.01	0.37	0	-6.195515	2.60E-10	3.98E-10	0.28	0.4	0.43	0	0	0
OsR498G0203580700.01	4.573	23.823	2.3901436	4.27E-180	1.40E-180	4.86	3.97	4.89	23.37	23.44	24.66
OsR498G0203585900.01	0.2	1.023	2.4173535	3.27E-13	4.58E-13	0.35	0.18	0.07	0.92	1.25	0.9
OsR498G0203590900.01	9.71	21.34	1.1342261	1.12E-58	7.85E-59	10.38	8.5	10.25	21.13	19.86	23.03
OsR498G0203592300.01	219.4	67.27	-1.735415	0	0	223.03	219.6	215.57	67.7	68.68	65.43
OsR498G0203594100.01	8.483	27.903	1.7228205	3.24E-194	9.94E-195	7.95	8.8	8.7	28.12	29.14	26.45
OsR498G0203595100.01	0.076	15.346	7.6079242	1.47E-299	3.12E-300	0.1	0.03	0.1	14.06	15.93	16.05
OsR498G0203609300.01	2.43	5.48	1.1702717	2.83E-44	2.32E-44	2.28	2.35	2.66	5.06	5.46	5.92
OsR498G0203620800.01	5.226	11.856	1.1801749	8.72E-64	5.83E-64	5.2	5.81	4.67	11.09	11.53	12.95
OsR498G0203621100.01	0	1.32	6.234812	1.07E-10	1.62E-10	0	0	0	0.92	1.36	1.68
OsR498G0203639000.01	5.29	16.503	1.6411967	3.00E-96	1.55E-96	5.25	5.36	5.26	17.19	16.3	16.02
OsR498G0203643200.01	35.79	167.74	2.2153136	0	0	34.67	37.88	34.82	170.39	165.24	167.6
OsR498G0203648900.01	0.72	0.11	-2.683802	5.79E-17	7.41E-17	0.62	0.66	0.88	0.1	0.12	0.11
OsR498G0203653400.01	23.046	54.403	1.23492	6.99E-266	1.62E-266	22.98	22.47	23.69	55.68	53.52	54.01
OsR498G0203675100.01	0.05	0.373	2.9286063	9.53E-07	1.67E-06	0.07	0.04	0.04	0.25	0.21	0.66
OsR498G0203677700.01	4.303	23.886	2.4784868	4.30E-207	1.25E-207	4.79	3.48	4.64	22.46	22.61	26.59
OsR498G0203686900.01	0.083	0.006	-4.113053	2.28E-05	4.25E-05	0.05	0.1	0.1	0	0	0.02
OsR498G0203707200.01	19.213	40.176	1.0641796	2.15E-239	5.54E-240	19.03	19.51	19.1	40.26	41.06	39.21
OsR498G0203719700.01	0.326	1.223	1.9093149	4.14E-08	6.83E-08	0.35	0.34	0.29	0.89	1.06	1.72
OsR498G0203723000.01	1.25	3.503	1.5485657	8.18E-31	8.13E-31	1.27	1.39	1.09	3.04	4.14	3.33
OsR498G0203737200.01	0.59	1.973	1.7399447	4.05E-06	7.29E-06	0.12	0.94	0.71	1.73	2.07	2.12
OsR498G0203738600.01	126	61.553	-1.034572	1.84E-285	4.05E-286	128.01	128.46	121.53	60.56	60.74	63.36
OsR498G0203745800.01	2.126	4.286	1.0015461	1.21E-13	1.67E-13	2.35	2.05	1.98	4.05	3.96	4.85
OsR498G0203746600.01	22.806	10.843	-1.174769	8.10E-98	4.13E-98	24.45	20.51	23.46	10.83	11.94	9.76
OsR498G0203746900.01	5.876	27.026	2.1791964	1.93E-86	1.07E-86	7.12	4.79	5.72	24.91	27.88	28.29
OsR498G0203747100.01	0.04	6.036	7.2270754	3.62E-69	2.29E-69	0.06	0	0.06	6	5.22	6.89
OsR498G0203747500.01	11.066	24.113	1.1486454	1.80E-59	1.25E-59	13.1	10.23	9.87	23.69	23.79	24.86
OsR498G0203755100.01	38.966	94.58	1.1564559	9.84E-247	2.47E-247	38.34	38.56	40	92.49	94.67	96.58
OsR498G0203756600.01	16.876	37.363	1.1499636	1.11E-175	3.71E-176	15.95	16.56	18.12	38.57	36.65	36.87
OsR498G0203757400.01	0.013	1.443	6.6609105	2.26E-25	2.46E-25	0.04	0	0	1.59	1.37	1.37
OsR498G0203758400.01	26.186	85.826	1.8346015	0	0	24.55	28.4	25.61	85.47	87	85.01
OsR498G0203764700.01	0	0.276	5.8123532	1.85E-08	3.03E-08	0	0	0	0.33	0.26	0.24
OsR498G0203767000.01	1.413	6.063	2.0932462	4.78E-49	3.72E-49	0.99	1.24	2.01	5.77	6.07	6.35
OsR498G0203767300.01	89.36	37.92	-1.226555	0	0	86.12	92.76	89.2	37.18	39.98	36.6
OsR498G0203772600.01	0.39	0.82	1.0767717	8.23E-08	1.38E-07	0.33	0.4	0.44	0.77	0.75	0.94
OsR498G0203772700.01	3.21	6.81	1.0844994	2.44E-22	2.80E-22	3.5	3.46	2.67	7.03	6.59	6.81
OsR498G0203773700.01	0.473	3.276	2.9009676	3.50E-84	1.98E-84	0.52	0.48	0.42	3.64	3.06	3.13
OsR498G0203775300.01	81.903	242.38	1.5698415	0	0	82.17	80.48	83.06	247.68	234.15	245.32
OsR498G0203777900.01	0.073	0.206	1.5289988	4.59E-05	8.69E-05	0.07	0.07	0.08	0.22	0.16	0.24
OsR498G0203781300.01	0.466	0.04	-3.485022	4.31E-08	7.12E-08	0.56	0.28	0.56	0.08	0	0.04
OsR498G0203785700.01	0.853	1.843	1.1477416	1.58E-11	2.33E-11	0.97	0.83	0.76	1.81	1.75	1.97
OsR498G0203791300.01	5.76	12.533	1.130855	6.78E-115	3.10E-115	6.35	5.47	5.46	12.21	13.65	11.74
OsR498G0203794900.01	15.34	35.353	1.220797	3.46E-226	9.32E-227	16.4	14.08	15.54	35.55	35.42	35.09
OsR498G0203805100.01	20.14	63.12	1.6462805	1.02E-207	2.96E-208	18.83	21.7	19.89	62.82	61.05	65.49
OsR498G0203805300.01	31.316	123.68	1.9784568	0	0	29.26	31.11	33.58	118.37	125.48	127.19
OsR498G0203807200.01	0.433	1.18	1.4400735	1.65E-05	3.05E-05	0.62	0.43	0.25	1.21	0.9	1.43
OsR498G0203808800.01	2.75	1.096	-1.149972	7.43E-32	7.27E-32	3.46	2.54	2.25	1.15	0.95	1.19
OsR498G0203812000.01	2.24	6.256	1.4716436	2.50E-23	2.82E-23	2.03	2.37	2.32	6.01	6.62	6.14
OsR498G0203816400.01	6.663	16.46	1.3088783	7.02E-145	2.70E-145	6.82	6.6	6.57	16.48	16.96	15.94
OsR498G0203816900.01	9.366	1.99	-2.305299	1.15E-196	3.49E-197	9.19	9.88	9.03	2.15	1.74	2.08
OsR498G0203819600.01	0.413	3.5	3.0778395	1.74E-55	1.26E-55	0.47	0.5	0.27	3.7	3.03	3.77
OsR498G0203829100.01	1.273	0.303	-2.072896	1.59E-15	2.11E-15	1.3	1.29	1.23	0.42	0.12	0.37
OsR498G0203835200.01	46.95	105.75	1.1809205	0	0	45.85	49.65	45.35	107.15	108.36	101.74
OsR498G0203838500.01	7.383	40.473	2.449442	9.23E-292	2.00E-292	7.54	7.8	6.81	41.41	39.06	40.95
OsR498G0203840900.01	0.06	0.173	1.4691746	0.0009479	0.0019719	0.08	0.03	0.07	0.2	0.15	0.17
OsR498G0203841100.01	0.036	0.23	2.6748497	1.53E-05	2.83E-05	0.03	0.03	0.05	0.29	0.26	0.14
OsR498G0203842300.01	0.773	0.353	-1.127952	5.01E-05	9.53E-05	0.61	0.82	0.89	0.28	0.37	0.41
OsR498G0203852800.01	0.143	0.716	2.2183355	1.89E-12	2.71E-12	0.14	0.17	0.12	0.78	0.54	0.83
OsR498G0203857000.01	1.936	4.023	1.0568721	6.40E-12	9.31E-12	2.21	1.55	2.05	4.09	4.12	3.86
OsR498G0203861100.01	7.916	23.273	1.5530862	6.66E-52	5.02E-52	8.5	7.01	8.24	21.38	21.59	26.85

OsR498G0203863600.01	0.42	1.62	1.9481443	5.97E-40	5.20E-40	0.44	0.4	0.42	1.61	1.75	1.5
OsR498G0203880900.01	3.313	7.326	1.1448857	1.38E-61	9.43E-62	3.32	3.26	3.36	7.25	7.26	7.47
OsR498G0203881300.01	11.07	38.743	1.8408535	0	0	11.21	11.57	10.43	38	37.39	40.84
OsR498G0203881900.01	36.51	75.553	1.0453814	2.32E-99	1.17E-99	34.3	39.73	35.5	80.46	68.33	77.87
OsR498G0203882700.01	0.41	0.14	-1.534604	0.0002162	0.0004275	0.5	0.33	0.4	0.19	0.1	0.13
OsR498G0203883100.01	1.746	34.373	4.2986436	0	0	1.69	1.57	1.98	36.55	30.86	35.71
OsR498G0203883500.01	4.356	62.256	3.7394958	0	0	4.03	4.37	4.67	60.73	58.96	67.08
OsR498G0203884300.01	0.836	3.566	1.8271139	3.81E-58	2.69E-58	0.61	0.96	0.94	3.62	3.5	3.58
OsR498G0203887100.01	0.206	0.55	1.3894475	0.0001142	0.0002223	0.14	0.24	0.24	0.48	0.54	0.63
OsR498G0203899300.01	2.206	16.313	2.9054466	6.15E-194	1.89E-194	2.31	2.39	1.92	15.93	16.47	16.54
OsR498G0203900100.01	1.59	0.736	-1.113053	2.82E-06	5.03E-06	1.74	1.16	1.87	0.96	0.41	0.84
OsR498G0203901800.01	8.38	24.876	1.567884	1.54E-39	1.35E-39	6.42	9.53	9.19	23.43	26.38	24.82
OsR498G0203902500.01	0.463	5.506	3.6210115	1.02E-52	7.64E-53	0.53	0.46	0.4	6.29	4.98	5.25
OsR498G0203903900.01	6.77	17.56	1.3738334	7.45E-93	3.94E-93	6.88	6.38	7.05	17.38	17.68	17.62
OsR498G0203907000.01	0	0.203	4.4364623	0.0004281	0.0008637	0	0	0	0.14	0.06	0.41
OsR498G0203907900.01	0.62	2.943	2.2474285	2.93E-12	4.22E-12	0.83	0.82	0.21	2.72	2.91	3.2
OsR498G0203912300.01	6.046	18.266	1.6005641	7.60E-156	2.78E-156	6.59	6.27	5.28	17.25	19.55	18
OsR498G0203912800.01	0	0.446	6.144335	3.60E-10	5.53E-10	0	0	0	0.44	0.48	0.42
OsR498G0203917700.01	3.446	51.54	3.8530278	0	0	3.56	3.03	3.75	52.98	52.74	48.9
OsR498G0203927400.01	1.66	0.62	-1.41959	1.86E-09	2.92E-09	1.7	1.38	1.9	0.53	0.78	0.55
OsR498G0203931400.01	0.213	1.216	2.7292975	8.81E-06	1.61E-05	0.25	0.1	0.29	0.99	0.78	1.88
OsR498G0203939000.01	4.596	71.82	4.0220163	0	0	4.15	4.61	5.03	72.4	75.27	67.79
OsR498G0203939200.01	6.54	71.58	3.4513229	0	0	6.69	6.13	6.8	69.92	70.32	74.5
OsR498G0203939400.01	27.81	81.253	1.5422278	0	0	27.26	29.3	26.87	83.66	81.38	78.72
OsR498G0203948900.01	33.96	94.656	1.4793229	0	0	33.55	34.93	33.4	96.36	94.23	93.38
OsR498G0203953400.01	10.49	21.28	1.0162301	1.40E-67	8.97E-68	11.18	9.32	10.97	21.97	21.51	20.36
OsR498G0203954400.01	0.053	1.206	4.5442656	7.08E-23	8.04E-23	0.08	0.04	0.04	1.25	1.28	1.09
OsR498G0203954800.01	31.363	83.766	1.3963006	0	0	30.91	30.63	32.55	86.8	81.9	82.6
OsR498G0203961800.01	15.03	31.22	1.054432	9.67E-246	2.44E-246	14.81	15.18	15.1	32.37	32.86	28.43
OsR498G0203962500.01	1.51	16.403	3.4318972	1.21E-276	2.71E-277	1.2	1.71	1.62	16.21	16.02	16.98
OsR498G0203968800.01	4.06	1.083	-1.906946	1.09E-15	1.43E-15	4.28	3.91	3.99	1.02	1.1	1.13
OsR498G0203971900.01	0.276	1.103	1.9910887	9.31E-20	1.12E-19	0.25	0.23	0.35	1.04	1.07	1.2
OsR498G0203972200.01	1.253	3.313	1.4025829	1.39E-22	1.59E-22	1.09	0.96	1.71	3.68	2.87	3.39
OsR498G0203973700.01	6.773	311.06	5.5207658	0	0	6.98	6.61	6.73	316.71	315.19	301.27
OsR498G0203975000.01	0.626	0.073	-3.076216	8.93E-12	1.30E-11	0.42	0.68	0.78	0.03	0.16	0.03
OsR498G0203976100.01	1.046	5.423	2.3726133	4.65E-44	3.82E-44	0.86	1.03	1.25	6.55	3.79	5.93
OsR498G0203976400.01	6.456	45.506	2.8827203	0	0	7.61	4.95	6.81	44.08	42.13	50.31
OsR498G0203977800.01	1.54	9.89	2.6698291	1.66E-148	6.29E-149	1.42	1.51	1.69	10.04	10.07	9.56
OsR498G0203980700.01	0.426	1.126	1.4043978	7.27E-08	1.21E-07	0.43	0.64	0.21	1.04	1.1	1.24
OsR498G0203983400.01	0.02	0.28	4.4758491	8.85E-17	1.14E-16	0.03	0	0.03	0.3	0.26	0.28
OsR498G0203986400.01	0.083	0.86	3.3765084	5.87E-29	6.00E-29	0.16	0.07	0.02	0.86	0.83	0.89
OsR498G0203987700.01	1.9	5.146	1.4358579	1.93E-13	2.69E-13	2.11	1.75	1.84	4.97	5.29	5.18
OsR498G0203990200.01	1.406	4.503	1.677292	5.47E-62	3.71E-62	1.47	1.4	1.35	4.61	4.55	4.35
OsR498G0203996200.01	0.643	1.936	1.5865939	7.58E-10	1.18E-09	0.56	0.49	0.88	1.94	1.82	2.05
OsR498G0203998700.01	11.36	46.49	1.9978778	4.78E-202	1.42E-202	11.56	10.08	12.44	46.57	46.95	45.95
OsR498G0203998900.01	7.736	13.753	1.0917869	5.50E-52	4.14E-52	7.99	7.45	7.77	12.85	13.2	15.21
OsR498G0203999500.01	0.443	0	-5.610553	1.82E-07	3.08E-07	0.39	0.61	0.33	0	0	0
OsR498G0204005600.01	10.836	43.41	1.9968622	2.63E-222	7.18E-223	9.51	10.47	12.53	44.8	40.23	45.2
OsR498G0204009900.01	0.283	0.756	1.3343059	3.23E-06	5.79E-06	0.43	0.11	0.31	0.72	0.87	0.68
OsR498G0204010400.01	0	6.863	8.9856372	1.01E-49	7.75E-50	0	0	0	7.37	6.23	6.99
OsR498G0204019800.01	34.973	73.033	1.0751388	0	0	34.89	35.72	34.31	71.58	76.33	71.19
OsR498G0204021100.01	0.25	1.146	2.0280479	2.78E-16	3.61E-16	0.26	0.19	0.3	1.26	1.21	0.97
OsR498G0204022400.01	0.27	0.81	1.580131	5.38E-07	9.30E-07	0.21	0.21	0.39	0.65	0.68	1.1
OsR498G0204023600.01	0.05	0.726	3.9516899	8.83E-15	1.19E-14	0.07	0.04	0.04	0.96	0.44	0.78
OsR498G0204025500.01	0.79	3.97	2.3336915	3.81E-41	3.27E-41	0.82	0.62	0.93	3.73	3.3	4.88
OsR498G0204031200.01	0	0.32	5.4979719	4.04E-07	6.96E-07	0	0	0	0.33	0.29	0.34
OsR498G0204033800.01	1.286	4.403	1.7706814	4.99E-36	4.59E-36	1.49	1.27	1.1	4.24	4.16	4.81
OsR498G0204036700.01	6.313	12.733	1.0203758	2.80E-45	2.27E-45	6.09	6.97	5.88	13.25	12.57	12.38
OsR498G0204038000.01	14.406	32.116	1.1544976	4.90E-66	3.20E-66	14.81	13.77	14.64	30.83	31.96	33.56
OsR498G0204042000.01	86.516	27.793	-1.646625	1.71E-289	3.72E-290	81.26	90.7	87.59	27.47	26.65	29.26
OsR498G0204065700.01	2.196	10.496	2.2622682	0	0	2.17	2.15	2.27	10.36	10.87	10.26
OsR498G0204068400.01	3.413	8.783	1.354812	1.51E-53	1.12E-53	3.66	3.03	3.55	8.34	9.4	8.61
OsR498G0204078500.01	0.21	0.08	-1.394177	0.0002858	0.0005703	0.19	0.15	0.29	0.03	0.09	0.12
OsR498G0204080800.01	0.13	0.463	1.7817649	8.23E-05	0.0001585	0.12	0.15	0.12	0.32	0.37	0.7
OsR498G0204085100.01	5.56	23.206	2.0594305	1.82E-144	7.01E-145	5.22	6.06	5.4	22.2	23.29	24.13
OsR498G0204086500.01	0.386	0.143	-1.447054	9.01E-07	1.58E-06	0.38	0.31	0.47	0.08	0.24	0.11
OsR498G0204090800.01	3.963	10.08	1.347403	8.73E-85	4.92E-85	3.8	3.8	4.29	10.43	10.43	9.38
OsR498G0204091600.01	1.58	5.22	1.7165558	5.74E-41	4.93E-41	1.25	2.09	1.4	5.53	5.53	4.6
OsR498G0204091800.01	0.513	1.36	1.4014095	2.13E-09	3.36E-09	0.44	0.54	0.56	1.31	1.6	1.17

OsR498G0204091900.01	0.866	3.653	1.9443924	3.88E-29	3.96E-29	1.11	1.01	0.48	3.72	3.94	3.3
OsR498G0204092600.01	6.46	20.816	1.6990562	6.24E-150	2.34E-150	6.25	5.9	7.23	20.99	21.03	20.43
OsR498G0204095000.01	0.23	0.846	1.9614709	5.01E-13	7.06E-13	0.22	0.22	0.25	0.82	0.74	0.98
OsR498G0204096700.01	6.31	19.366	1.6221918	3.64E-180	1.19E-180	6.72	6.07	6.14	19.79	20.17	18.14
OsR498G0204097400.01	0.073	0.36	2.3529216	5.18E-05	9.85E-05	0.09	0	0.13	0.33	0.41	0.34
OsR498G0204099800.01	7.43	16.483	1.319086	5.36E-142	2.10E-142	7.43	7.8	7.06	17	16.42	16.03
OsR498G0204100600.01	201.383	58.433	-1.793126	0	0	209.36	197.54	197.25	60.04	59.98	55.28
OsR498G0204103500.01	33.083	1.893	-4.105545	0	0	32.52	33.18	33.55	2.05	1.88	1.75
OsR498G0204104000.01	10.616	31.816	1.5422666	7.92E-304	1.66E-304	11.25	10.04	10.56	32.79	32.07	30.59
OsR498G0204107300.01	0.046	1.79	5.2223375	3.39E-28	3.51E-28	0.14	0	0	2.06	1.68	1.63
OsR498G0204107700.01	10.743	3.433	-1.674493	1.33E-87	7.31E-88	9.67	11.67	10.89	3.84	3.3	3.16
OsR498G0204108000.01	1.956	0.446	-2.154873	3.84E-22	4.42E-22	1.95	1.87	2.05	0.56	0.24	0.54
OsR498G0204109800.01	0.666	3.77	2.6331504	5.76E-51	4.38E-51	0.84	0.5	0.66	3.84	3.62	3.85
OsR498G0204111700.01	36.743	85.753	1.2287618	0	0	36.92	38.67	34.64	84.7	86.46	86.1
OsR498G0204117500.01	0.74	11.526	3.9097043	0	0	0.66	0.94	0.62	10.93	12.19	11.46
OsR498G0204117800.01	10.31	25.53	1.3065929	7.98E-109	3.78E-109	9.9	10.45	10.58	25.59	24.53	26.47
OsR498G0204120800.01	2.706	5.56	1.0326246	1.28E-08	2.08E-08	2.76	2.62	2.74	5.5	5.55	5.63
OsR498G0204121100.01	11.79	37.11	1.5931752	1.32E-145	5.04E-146	11.61	11.85	11.91	37.1	35.72	38.51
OsR498G0204121600.01	0.066	7.926	6.8813006	4.38E-110	2.06E-110	0.15	0.05	0	7.37	7.68	8.73
OsR498G0204124000.01	0.02	0.556	5.7557697	1.15E-14	1.56E-14	0	0.06	0	0.45	0.57	0.65
OsR498G0204130800.01	11.276	25.373	1.1562483	3.20E-65	2.11E-65	11.27	11.97	10.59	24.87	25.59	25.66
OsR498G0204131200.01	0.14	0.426	1.5910813	3.41E-05	6.42E-05	0.17	0.14	0.11	0.39	0.38	0.51
OsR498G0204132900.01	2.276	4.96	1.1153351	1.03E-27	1.08E-27	2.31	2.22	2.3	5.38	4.81	4.69
OsR498G0204134300.01	0.073	0.356	2.2963381	8.68E-05	0.0001676	0.09	0	0.13	0.34	0.64	0.09
OsR498G0204135900.01	8.01	74.203	3.210624	0	0	7.56	8.18	8.29	70.55	73.34	78.72
OsR498G0204137000.01	3.846	14.726	1.9243693	1.54E-160	5.53E-161	3.75	4.23	3.56	15.47	14.47	14.24
OsR498G0204137400.01	8.976	19.09	1.0944496	3.29E-33	3.14E-33	9.57	8.84	8.52	19.63	18.65	18.99
OsR498G0204139100.01	0.473	2.033	2.0970292	1.07E-16	1.37E-16	0.65	0.27	0.5	1.7	1.87	2.53
OsR498G0204139900.01	9.376	37.086	1.9803146	3.80E-289	8.28E-290	8.79	9.94	9.4	36.96	37.56	36.74
OsR498G0204140800.01	0.11	0.006	-4.062972	3.25E-05	6.10E-05	0.16	0.12	0.05	0	0	0.02
OsR498G0204143300.01	0.69	0.143	-2.232041	4.64E-09	7.40E-09	0.56	0.82	0.69	0.13	0.23	0.07
OsR498G0204145300.01	0	0.106	4.2963381	0.0007942	0.0016388	0	0	0	0.1	0.06	0.16
OsR498G0204148100.01	3.553	10.83	1.6068271	5.83E-79	3.43E-79	3.77	3.52	3.37	11.27	10.02	11.2
OsR498G0204149200.01	0.62	4	2.688119	9.47E-60	6.55E-60	0.55	0.68	0.63	4.01	4.23	3.76
OsR498G0204150700.01	5.293	0.99	-2.417907	1.18E-113	5.46E-114	5.4	5.09	5.39	1.05	0.84	1.08
OsR498G0204151800.01	0.406	0	-6.86706	5.07E-15	6.79E-15	0.51	0.41	0.3	0	0	0
OsR498G0204152600.01	4.936	0.013	-8.609914	2.43E-153	8.97E-154	4.78	5.11	4.92	0	0.04	0
OsR498G0204154400.01	0.02	0.16	2.8813006	0.000469	0.0009493	0.03	0	0.03	0.22	0.13	0.13
OsR498G0204155600.01	1.413	7.43	2.3885082	9.44E-47	7.52E-47	1.01	1.55	1.68	7.54	6.59	8.16
OsR498G0204157800.01	1.78	4.203	1.2396828	6.92E-22	8.01E-22	1.9	1.72	1.72	4.07	3.7	4.84
OsR498G0204160000.01	12.7	27.493	1.1106577	9.81E-104	4.80E-104	13.09	12.17	12.84	27.46	28.62	26.4
OsR498G0204160200.01	19.106	62.916	1.7569257	0	0	18.77	17.79	20.76	63.4	64.06	61.29
OsR498G0204162100.01	0.03	0.743	4.579272	4.74E-18	5.91E-18	0.03	0.06	0	0.64	0.69	0.9
OsR498G0204164900.01	14.37	33.856	1.2226317	7.93E-250	1.96E-250	15.1	13.85	14.16	33.49	35.57	32.51
OsR498G0204165200.01	9.443	203.21	4.4257405	0	0	10.51	10.04	7.78	197.34	199.63	212.67
OsR498G0204167700.01	0.176	1.21	2.7899854	8.04E-29	8.25E-29	0.17	0.17	0.19	1.18	1.16	1.29
OsR498G0204170600.01	6.336	26.416	2.0539006	1.93E-209	5.56E-210	6.58	6.72	5.71	26.68	24.74	27.83
OsR498G0204171000.01	1.79	3.803	1.0755888	3.57E-12	5.15E-12	2.07	1.41	1.89	4.03	4.03	3.35
OsR498G0204171700.01	5.37	2.27	-1.417384	3.61E-99	1.83E-99	5.1	5.16	5.85	2.15	2.14	2.52
OsR498G0204172200.01	3.516	1.163	-1.571581	5.27E-17	6.74E-17	3.62	3.92	3.01	1.3	1.2	0.99
OsR498G0204173100.01	0.06	1.326	4.4431794	8.21E-36	7.57E-36	0.05	0.08	0.05	1.5	1.04	1.44
OsR498G0204175500.01	0.173	15.746	6.4132018	1.47E-241	3.75E-242	0.13	0.26	0.13	16.29	13.83	17.12
OsR498G0204179700.01	0.523	1.543	1.5593725	6.14E-08	1.02E-07	0.39	0.56	0.62	1.32	2.07	1.24
OsR498G0204180400.01	3.096	6.976	1.167055	4.92E-07	8.51E-07	2.66	3.8	2.83	6.5	5.92	8.51
OsR498G0204182200.01	1.85	5.996	1.696876	3.58E-29	3.65E-29	1.62	2.32	1.61	5.96	6.52	5.51
OsR498G0204182400.01	0.353	0.876	1.310013	0.0001073	0.0002083	0.36	0.35	0.35	0.84	1.03	0.76
OsR498G0204184100.01	245.576	30.46	-3.013865	0	0	225.92	244.96	265.85	31.04	27.4	32.94
OsR498G0204185600.01	15.396	37.29	1.3028514	7.24E-188	2.28E-188	15.44	14.6	16.15	35.14	36.84	39.89
OsR498G0204186700.01	1.99	0.33	-2.593275	8.42E-14	1.16E-13	2.38	1.72	1.87	0.21	0.35	0.43
OsR498G0204187800.01	7.643	19.09	1.3198776	8.37E-69	5.32E-69	8.03	7.56	7.34	17.52	19.81	19.94
OsR498G0204188600.01	0.033	0.7	4.2223375	6.41E-10	9.93E-10	0.05	0	0.05	0.88	0.58	0.64
OsR498G0204189200.01	0.116	2.89	4.656234	2.88E-36	2.63E-36	0.06	0.06	0.23	2.57	3.06	3.04
OsR498G0204190000.01	1.77	0.023	-6.235043	2.59E-19	3.15E-19	1.87	1.79	1.65	0	0.07	0
OsR498G0204192200.01	5.27	37.473	2.8284729	0	0	4.82	4.91	6.08	36.51	35.97	39.94
OsR498G0204192400.01	19.826	171.9	3.1056066	0	0	19.81	19.57	20.1	169.8	171.96	173.95
OsR498G0204193000.01	0.36	2.233	2.637375	1.50E-15	1.99E-15	0.43	0.29	0.36	2.24	2.23	2.23
OsR498G0204193200.01	4.026	11.51	1.5147034	2.30E-35	2.13E-35	3.85	4.26	3.97	11.72	11.74	11.07
OsR498G0204196300.01	37.926	76.46	1.0086455	2.61E-196	7.95E-197	38.23	38.76	36.79	75.86	77.14	76.38
OsR498G0204196600.01	13.22	36.276	1.4540647	7.91E-91	4.25E-91	12.13	12.45	15.08	37.85	32.66	38.32

OsR498G0204197700.01	1.833	7.613	2.0156326	3.23E-61	2.21E-61	1.94	2.04	1.52	7.26	8.03	7.55
OsR498G0204203500.01	46.626	14.5	-1.686415	4.22E-189	1.32E-189	47.07	45.87	46.94	14.17	14	15.33
OsR498G0204203700.01	1.303	0.383	-1.762556	2.81E-05	5.26E-05	1.47	1.27	1.17	0.38	0.28	0.49
OsR498G0204207000.01	22.61	5.24	-2.110299	1.17E-193	3.60E-194	22.5	22.89	22.44	4.94	5.34	5.44
OsR498G0204209400.01	7.55	1.176	-2.699245	3.44E-90	1.86E-90	7.92	7.66	7.07	1.08	1.13	1.32
OsR498G0204216600.01	11.273	59.116	2.3855757	0	0	10.44	11.32	12.06	56.8	58.99	61.56
OsR498G0204216800.01	1.166	7.806	2.7020101	3.49E-85	1.96E-85	1.05	1.21	1.24	7.97	7.35	8.1
OsR498G0204218200.01	2.603	6.673	1.3672682	4.99E-42	4.22E-42	2.38	2.91	2.52	6.81	7.01	6.2
OsR498G0204218400.01	0.583	1.873	1.6758865	3.69E-30	3.71E-30	0.56	0.54	0.65	1.86	1.86	1.9
OsR498G0204218900.01	30.12	114.89	1.9281899	0	0	29.85	31.91	28.6	111.37	111.73	121.58
OsR498G0204219200.01	7.756	15.67	1.0256353	3.15E-41	2.69E-41	8.13	7.81	7.33	15.76	16.34	14.91
OsR498G0204223300.01	294.596	126.88	-1.164263	0	0	295.6	297.06	291.13	128.7	127.68	124.26
OsR498G0204224400.01	62.74	212.63	1.7521316	0	0	61.35	62.08	64.79	210.99	212.33	214.58
OsR498G0204224700.01	4.256	8.503	1.0059588	4.54E-24	5.04E-24	4.64	3.59	4.54	8.96	8.02	8.53
OsR498G0204225600.01	0.496	0.896	1.4260359	3.26E-07	5.60E-07	0.19	0.69	0.61	0.52	1.11	1.06
OsR498G0204228500.01	4.026	1.346	-1.561643	1.02E-38	9.00E-39	4.15	4.23	3.7	1.38	1.46	1.2
OsR498G0204230000.01	0.04	0.333	2.97441	5.43E-07	9.41E-07	0.03	0.06	0.03	0.25	0.43	0.32
OsR498G0204236200.01	75.526	19.07	-2.027414	0	0	78.9	75.09	72.59	19.16	18.15	19.9
OsR498G0204238700.01	1.183	3.11	1.3801696	8.50E-21	1.00E-20	1.05	1.06	1.44	3.07	3.56	2.7
OsR498G0204242200.01	7.41	15.356	1.0508231	7.13E-42	6.05E-42	6.41	7.78	8.04	13.38	16.89	15.8
OsR498G0204244900.01	2.57	6.206	1.2115129	4.78E-47	3.80E-47	2.51	2.83	2.37	6.27	5.97	6.38
OsR498G0204245100.01	0.676	1.39	1.0113996	5.74E-08	9.54E-08	0.5	0.72	0.81	1.43	1.41	1.33
OsR498G0204248100.01	1.493	4.173	1.4903021	5.08E-34	4.80E-34	1.43	1.71	1.34	4.35	4.03	4.14
OsR498G0204252400.01	34.676	81.366	1.2238895	0	0	35.57	34.03	34.43	83.35	82.16	78.59
OsR498G0204255200.01	3.86	1.293	-1.577024	5.88E-33	5.64E-33	4.26	3.85	3.47	0.99	1.61	1.28
OsR498G0204255800.01	0.813	0.35	-1.221987	0.000156	0.000306	0.8	0.84	0.8	0.35	0.52	0.18
OsR498G0204258800.01	723.276	319.37	-1.176637	0	0	735.53	731.1	703.2	331.23	325.58	301.31
OsR498G0204265100.01	0.136	0.386	1.5197394	0.0008986	0.0018646	0.17	0.11	0.13	0.34	0.52	0.3
OsR498G0204267500.01	6.373	19.15	1.5767545	2.72E-101	1.35E-101	6.42	6.66	6.04	19.32	20.04	18.09
OsR498G0204274200.01	1.47	0.58	-1.341092	1.87E-08	3.05E-08	1.98	1.37	1.06	0.54	0.57	0.63
OsR498G0204275400.01	14.38	32.326	1.1741215	2.53E-247	6.34E-248	14.3	13.97	14.87	33.25	32.96	30.77
OsR498G0204275900.01	5.94	1.703	-1.808283	6.93E-58	4.90E-58	7.03	5.19	5.6	1.86	1.6	1.65
OsR498G0204278200.01	1.16	0.153	-3.21545	6.26E-11	9.40E-11	1.92	0.86	0.7	0.14	0.16	0.16
OsR498G0204279000.01	1.043	0.083	-3.575787	7.66E-18	9.60E-18	1.23	1.06	0.84	0.07	0.07	0.11
OsR498G0204279800.01	1.226	0.56	-1.053408	1.59E-08	2.59E-08	1.21	0.93	1.54	0.61	0.61	0.46
OsR498G0204282000.01	3.16	6.54	1.0467242	1.72E-28	1.78E-28	3.08	2.87	3.53	6.74	6.51	6.37
OsR498G0204285300.01	592.95	1794.9	1.5979016	0	0	598.09	600.02	580.74	1816.8	1819.1	1748.6
OsR498G0204285700.01	0.543	1.323	1.2764385	1.43E-11	2.11E-11	0.52	0.62	0.49	1.31	1.2	1.46
OsR498G0204288500.01	2.756	6.753	1.2896122	1.31E-13	1.81E-13	2.61	2.59	3.07	6.02	7.59	6.65
OsR498G0204288800.01	0.706	5.263	2.8981744	2.15E-22	2.46E-22	0.5	1	0.62	4.63	6.55	4.61
OsR498G0204291800.01	0.43	1.5	1.7954398	2.62E-11	3.88E-11	0.39	0.51	0.39	1.47	1.13	1.9
OsR498G0204292000.01	20.006	40.533	1.0155791	4.51E-251	1.11E-251	20.65	20	19.37	39.75	44.17	37.68
OsR498G0204292600.01	23.003	587.19	4.6700259	0	0	23.66	22.08	23.27	587.6	565.51	608.46
OsR498G0204296100.01	0.393	1.48	1.9219425	9.32E-10	1.45E-09	0.56	0.17	0.45	1.58	1.41	1.45
OsR498G0204296700.01	8.793	24.9	1.5101548	1.07E-132	4.41E-133	8.62	8.86	8.9	25.03	23.38	26.29
OsR498G0204297200.01	16.226	55.946	1.7849225	9.86E-205	2.90E-205	15.38	17.42	15.88	55.83	57.05	54.96
OsR498G0204298500.01	12.516	28.71	1.1890568	8.10E-77	4.84E-77	13.2	12.28	12.07	30.18	27.33	28.62
OsR498G0204298800.01	24.596	112.38	2.2176168	0	0	25.98	25.1	22.71	113.85	114.02	109.28
OsR498G0204299100.01	0.14	0.03	-2.485022	9.86E-05	0.0001911	0.09	0.16	0.17	0	0.05	0.04
OsR498G0204300600.01	1.713	3.59	1.0611604	0.0002391	0.0004743	1.66	1.83	1.65	3.44	2.95	4.38
OsR498G0204301900.01	0.256	1.113	2.0628625	8.01E-13	1.13E-12	0.14	0.23	0.4	1.31	0.88	1.15
OsR498G0204307500.01	0.213	0.593	1.4588098	3.52E-07	6.06E-07	0.26	0.19	0.19	0.6	0.45	0.73
OsR498G0204310500.01	0.326	0.97	1.5762755	2.13E-07	3.63E-07	0.17	0.6	0.21	0.89	0.96	1.06
OsR498G0204322800.01	0.176	0.006	-4.832945	3.08E-08	5.06E-08	0.19	0.28	0.06	0.02	0	0
OsR498G0204323600.01	0.073	0.37	2.4338416	4.77E-09	7.60E-09	0.04	0.11	0.07	0.35	0.54	0.22
OsR498G0204325700.01	0.746	2.78	1.8950111	1.20E-43	9.93E-44	0.62	0.87	0.75	2.5	2.73	3.11
OsR498G0204326200.01	0.136	0.92	2.8187591	2.54E-26	2.73E-26	0.08	0.17	0.16	1.21	0.78	0.77
OsR498G0204327100.01	8.093	17.5	1.1123448	5.37E-31	5.32E-31	9.03	7	8.25	17.41	16.36	18.73
OsR498G0204327700.01	3.683	9.526	1.335467	3.33E-57	2.37E-57	3.05	3.59	4.41	10.25	9	9.33
OsR498G0204328000.01	2.68	10.733	2.0014158	1.98E-74	1.20E-74	2.49	2.75	2.8	9.62	11.35	11.23
OsR498G0204331600.01	5.876	2.853	-1.10534	4.38E-42	3.70E-42	5.66	6.2	5.77	2.54	2.79	3.23
OsR498G0204331700.01	0.14	0.05	-1.588487	0.0003174	0.0006352	0.14	0.09	0.19	0.06	0.03	0.06
OsR498G0204333300.01	26.893	74.406	1.4667449	1.11E-214	3.13E-215	25.81	26.38	28.49	72.41	73.22	77.59
OsR498G0204335200.01	0.906	3.65	2.0310994	2.20E-59	1.53E-59	1.01	0.9	0.81	3.35	3.73	3.87
OsR498G0204336300.01	0.07	0.306	2.2963381	2.60E-07	4.44E-07	0.14	0.07	0	0.21	0.38	0.33
OsR498G0204336600.01	0.03	0.256	3.2963381	3.77E-07	6.48E-07	0	0	0.09	0.32	0.28	0.17
OsR498G0204337400.01	5.323	10.316	1.0822132	2.65E-38	2.35E-38	5.25	6.52	4.2	10.12	10.41	10.42
OsR498G0204337500.01	25.35	57.96	1.1922587	9.64E-300	2.04E-300	25.82	25.86	24.37	57.7	58.96	57.22
OsR498G0204339400.01	12.923	82.05	2.6656041	0	0	11.95	13.31	13.51	81.87	79.39	84.89

OsR498G0204343600.01	12.336	42.813	1.7854377	0	0	12.34	12.27	12.4	41.38	43.76	43.3
OsR498G0204345600.01	0.12	0.653	2.4338416	5.63E-12	8.18E-12	0.1	0.21	0.05	0.58	0.68	0.7
OsR498G0204346000.01	0.1	4.31	5.4338416	5.71E-53	4.25E-53	0.12	0.12	0.06	3.96	4.18	4.79
OsR498G0204347900.01	4.073	15.94	1.9751955	1.12E-188	3.52E-189	4.34	3.86	4.02	15.47	16.31	16.04
OsR498G0204349900.01	13.753	35.483	1.3640162	0	0	14.67	12.65	13.94	36.79	35.36	34.3
OsR498G0204350900.01	1.06	18.44	4.117726	2.58E-284	5.70E-285	1.18	1.08	0.92	18.55	17.74	19.03
OsR498G0204351800.01	11.443	36.763	1.687128	1.09E-214	3.07E-215	11.48	11.61	11.24	35.32	38.36	36.61
OsR498G0204353600.01	7.666	18.49	1.3039998	6.97E-134	2.86E-134	7.55	7.54	7.91	18.03	18.39	19.05
OsR498G0204354400.01	13.8	151.16	3.4516469	0	0	13.98	13.74	13.68	147.49	148.61	157.37
OsR498G0204354900.01	0.033	0.946	5.9861963	6.40E-32	6.25E-32	0	0	0.1	1.06	0.69	1.09
OsR498G0204355400.01	0.83	6.343	2.9294522	5.87E-139	2.34E-139	0.71	0.9	0.88	6.3	6.08	6.65
OsR498G0204362700.01	0.49	2.146	2.1336086	2.48E-09	3.91E-09	0.69	0.49	0.29	2.19	1.71	2.54
OsR498G0204364800.01	1.096	4.616	2.0887934	5.31E-98	2.71E-98	1.12	1.29	0.88	4.51	4.34	5
OsR498G0204369900.01	15.783	47.553	1.6035054	0	0	17.93	15.61	13.81	46.81	48.88	46.97
OsR498G0204376600.01	1.873	0.83	-1.176877	5.87E-16	7.69E-16	2.33	1.57	1.72	0.77	0.84	0.88
OsR498G0204380600.01	5.17	13.933	1.3575502	2.39E-61	1.63E-61	4.61	5.29	5.61	13.1	14.44	14.26
OsR498G0204381800.01	0	0.25	5.0618728	1.23E-05	2.27E-05	0	0	0	0.31	0.26	0.18
OsR498G0204382100.01	95.586	265.96	1.4880565	0	0	95.51	88.94	102.31	248.1	253.28	296.49
OsR498G0204382300.01	0.183	0.926	2.5593725	7.13E-12	1.04E-11	0.24	0.21	0.1	0.92	0.94	0.92
OsR498G0204382700.01	28.51	14.11	-1.014209	3.01E-93	1.59E-93	29.03	27.29	29.21	13.77	14.32	14.24
OsR498G0204387000.01	0.293	0.026	-3.485022	0.000899	0.0018658	0.32	0.24	0.32	0	0	0.08
OsR498G0204387200.01	5.3	11.63	1.1238832	2.68E-38	2.38E-38	4.75	5.89	5.26	13.02	10.07	11.8
OsR498G0204391000.01	6.31	26.926	2.0916292	3.99E-274	9.07E-275	6.13	6.3	6.5	28.22	26.15	26.41
OsR498G0204391300.01	16.526	34.676	1.0525063	8.36E-70	5.27E-70	15.63	18.82	15.13	35.36	33.03	35.64
OsR498G0204391600.01	1.626	4.7	1.5593725	6.23E-27	6.61E-27	1.56	1.53	1.79	4.42	4.42	5.26
OsR498G0204392800.01	0.896	5.443	2.5920035	2.00E-90	1.08E-90	0.69	1.05	0.95	4.76	5.69	5.88
OsR498G0204395600.01	0.963	0.186	-2.366627	1.08E-11	1.58E-11	1.3	0.79	0.8	0.19	0.22	0.15
OsR498G0204398300.01	8.023	17.35	1.1019276	5.20E-50	4.00E-50	7.95	7.43	8.69	18.84	17.19	16.02
OsR498G0204400400.01	1.463	0.203	-2.853188	2.81E-13	3.93E-13	1.67	1.37	1.35	0.12	0.12	0.37
OsR498G0204402500.01	0	0.306	5.2963381	2.20E-06	3.91E-06	0	0	0	0.23	0.41	0.28
OsR498G0204404000.01	3.07	0.303	-3.337868	2.31E-29	2.35E-29	2.79	3.1	3.32	0.48	0.24	0.19
OsR498G0204405600.01	0.196	0.07	-1.515916	5.75E-06	1.04E-05	0.22	0.22	0.15	0.08	0.06	0.07
OsR498G0204410500.01	0.226	4.533	4.3106933	1.87E-45	1.52E-45	0.34	0.2	0.14	3.67	4.18	5.75
OsR498G0204418800.01	7.666	0.243	-5.00784	5.68E-171	1.93E-171	7.13	8.5	7.37	0.22	0.32	0.19
OsR498G0204420900.01	6.233	0.086	-6.134115	1.46E-148	5.51E-149	6.19	6.17	6.34	0.06	0.09	0.11
OsR498G0204424900.01	0.196	1.13	2.5218978	2.92E-12	4.21E-12	0.23	0.18	0.18	1.2	1.19	1
OsR498G0204425600.01	5.94	2.073	-1.547693	4.41E-20	5.28E-20	6.3	5.86	5.66	2.53	1.58	2.11
OsR498G0204430300.01	0.05	0.366	2.9378841	4.48E-08	7.41E-08	0.12	0.03	0	0.39	0.36	0.35
OsR498G0204432200.01	2.77	7.87	1.5271681	2.35E-33	2.25E-33	3.26	2.02	3.03	7.07	8.09	8.45
OsR498G0204432800.01	11.573	5.323	-1.092422	8.83E-23	1.00E-22	11.9	11.91	10.91	4.33	6.59	5.05
OsR498G0204435000.01	1.743	0.106	-4.485022	1.71E-26	1.83E-26	1.45	2.12	1.66	0.05	0.18	0.09
OsR498G0204439300.01	42.486	126.1	1.567352	0	0	40.98	43.67	42.81	127.28	126.32	124.69
OsR498G0204441300.01	4.316	10.356	1.2900971	5.70E-40	4.96E-40	4.91	3.71	4.33	11.13	10.1	9.84
OsR498G0204443200.01	0.783	10.726	3.7797024	3.77E-141	1.48E-141	0.85	0.61	0.89	10.54	9.68	11.96
OsR498G0204446300.01	13.666	36.003	1.4018964	6.40E-288	1.40E-288	13.59	14.56	12.85	36.01	36.23	35.77
OsR498G0204447000.01	0.373	0.133	-1.495075	0.0003264	0.0006536	0.41	0.49	0.22	0.06	0.09	0.25
OsR498G0204448900.01	0.74	0.26	-1.505583	1.74E-05	3.22E-05	1.01	0.67	0.54	0.16	0.24	0.38
OsR498G0204452600.01	9.39	19.343	1.0035149	1.43E-132	5.93E-133	8.86	9.45	9.86	19.93	18.19	19.91
OsR498G0204455300.01	6.276	23.173	1.8481337	6.52E-62	4.42E-62	5.65	6.93	6.25	24.49	20.67	24.36
OsR498G0204455600.01	0	0.526	4.6748497	0.0001298	0.0002536	0	0	0	0.6	0.24	0.74
OsR498G0204457300.01	0.573	0	-6.347518	3.18E-11	4.73E-11	0.65	0.47	0.6	0	0	0
OsR498G0204458200.01	5.006	11.106	1.1198404	2.03E-40	1.75E-40	5.31	4.87	4.84	12.02	12.09	9.21
OsR498G0204458500.01	0	0.103	4.6748497	0.0001298	0.0002536	0	0	0	0.17	0.02	0.12
OsR498G0204462100.01	1.953	0.393	-2.318372	5.41E-08	8.98E-08	2.04	1.79	2.03	0.47	0.47	0.24
OsR498G0204464600.01	0.376	0.096	-2.02559	1.37E-05	2.53E-05	0.48	0.37	0.28	0.09	0.07	0.13
OsR498G0204465900.01	3.393	6.58	1.1769229	8.38E-49	6.54E-49	3.65	3.26	3.27	6.73	6.76	6.25
OsR498G0204467100.01	0.286	1.213	2.1187999	3.52E-11	5.24E-11	0.3	0.29	0.27	1.05	1.19	1.4
OsR498G0204468900.01	5.826	13.133	1.1946539	1.29E-34	1.20E-34	5.87	6.26	5.35	13.15	13.54	12.71
OsR498G0204472600.01	0.646	3.66	2.5074686	4.23E-251	1.04E-251	0.6	0.64	0.7	3.73	3.64	3.61
OsR498G0204474000.01	0.08	1.04	3.6397459	2.83E-41	2.42E-41	0.06	0.09	0.09	0.93	0.99	1.2
OsR498G0204474300.01	31.573	123.03	1.9657424	0	0	29.85	32.09	32.78	116.96	127.24	124.9
OsR498G0204477800.01	5.716	0.233	-4.601956	1.63E-29	1.65E-29	5.97	4.8	6.38	0.27	0.19	0.24
OsR498G0204481600.01	0.216	0.646	1.5844634	4.25E-06	7.66E-06	0.14	0.17	0.34	0.46	0.76	0.72
OsR498G0204481800.01	27.17	54.793	1.0115026	4.40E-172	1.49E-172	26.48	27.39	27.64	54.37	57.29	52.72
OsR498G0204487100.01	1.833	0.883	-1.042664	4.13E-05	7.81E-05	1.85	2.03	1.62	0.74	0.56	1.35
OsR498G0204488200.01	1.32	0.296	-2.16024	1.42E-13	1.97E-13	1.26	1.25	1.45	0.38	0.27	0.24
OsR498G0204490700.01	7.336	17.133	1.2206826	2.44E-62	1.65E-62	7.23	6.59	8.19	16.2	15.88	19.32
OsR498G0204491200.01	0.506	1.833	1.8252665	4.58E-11	6.84E-11	0.3	0.61	0.61	2.06	1.57	1.87
OsR498G0204492800.01	0.306	0.086	-1.862091	0.0004461	0.0009016	0.22	0.44	0.26	0.04	0.11	0.11

OsR498G0204493300.01	35.08	10.783	-1.699723	2.81E-162	1.00E-162	34.06	33.64	37.54	9.84	9.48	13.03
OsR498G0204493600.01	1.466	2.09	1.0866195	6.05E-08	1.01E-07	1.4	1.81	1.19	1.87	1.53	2.87
OsR498G0204495300.01	30.543	14.82	-1.044529	6.74E-97	3.46E-97	31.94	28.84	30.85	14.77	14	15.69
OsR498G0204495600.01	1.5	0.493	-1.581406	5.07E-13	7.13E-13	1.19	1.59	1.72	0.5	0.47	0.51
OsR498G0204496000.01	9.156	3.553	-1.573699	7.00E-110	3.29E-110	9.02	8.62	9.83	3.91	3.26	3.49
OsR498G0204500600.01	0.79	0.096	-3.069984	2.00E-07	3.39E-07	0.72	0.86	0.79	0	0.07	0.22
OsR498G0204500800.01	2.79	6.406	1.0959719	4.05E-27	4.29E-27	2.67	2.96	2.74	6.54	5.95	6.73
OsR498G0204501000.01	0.036	0.323	3.103693	9.95E-08	1.67E-07	0	0.03	0.08	0.36	0.33	0.28
OsR498G0204501500.01	0.64	1.316	1.0471663	0.0004324	0.0008727	0.86	0.46	0.6	0.83	1.53	1.59
OsR498G0204502500.01	0.15	0.516	1.7954398	1.68E-06	2.97E-06	0.09	0.21	0.15	0.49	0.46	0.6
OsR498G0204505500.01	5.326	23.336	2.1246625	0	0	5.11	5.61	5.26	24.1	24.29	21.62
OsR498G0204505700.01	11.713	42.586	1.8669388	1.22E-211	3.49E-212	11.55	12.39	11.2	41.64	42.59	43.53
OsR498G0204515700.01	15.263	31.283	1.0285951	4.20E-115	1.91E-115	13.83	16.79	15.17	32.37	31.34	30.14
OsR498G0204519700.01	0.02	0.16	2.7817649	0.0008272	0.0017105	0	0.03	0.03	0.17	0.17	0.14
OsR498G0204522800.01	26.87	9.683	-1.474348	6.65E-99	3.37E-99	25.85	26.57	28.19	9.79	9.2	10.06
OsR498G0204527000.01	0.226	0.63	1.4691746	0.0009479	0.0019719	0.31	0.12	0.25	0.3	1.15	0.44
OsR498G0204529300.01	13.89	32.736	1.2335784	2.92E-166	1.02E-166	14.62	14.68	12.37	33.2	33.87	31.14
OsR498G0204535200.01	2.83	8.813	1.6367029	3.79E-86	2.11E-86	3.17	2.79	2.53	8.25	8.34	9.85
OsR498G0204536600.01	0.226	1.543	2.7817649	8.23E-17	1.06E-16	0.29	0.29	0.1	1.55	1.35	1.73
OsR498G0204538000.01	4.13	19.48	2.237856	1.91E-85	1.07E-85	4.01	4.82	3.56	19.88	20.13	18.43
OsR498G0204545100.01	1.393	4.593	1.7683582	4.13E-44	3.39E-44	1.52	1.4	1.26	4.52	4.31	4.95
OsR498G0204545400.01	33.023	9.1	-1.858126	6.12E-172	2.07E-172	35.96	30.12	32.99	9.97	9.37	7.96
OsR498G0204545500.01	9.13	2.753	-1.727668	1.27E-41	1.08E-41	9.22	10.59	7.58	2.21	3.49	2.56
OsR498G0204546200.01	5.24	2.626	-1.001862	3.12E-09	4.95E-09	4.84	5.36	5.52	2.47	2.07	3.34
OsR498G0204550600.01	1.603	4.296	1.3086164	1.84E-18	2.28E-18	1.47	2.06	1.28	3.19	5.17	4.53
OsR498G0204551100.01	1.706	4.643	1.4489965	1.16E-39	1.02E-39	1.94	1.67	1.51	5.46	4.53	3.94
OsR498G0204553900.01	6.83	17.506	1.3594202	7.26E-140	2.87E-140	7.25	6.82	6.42	18.27	16.64	17.61
OsR498G0204554400.01	2.253	10.2	2.186487	2.55E-121	1.12E-121	2.45	1.96	2.35	10.65	10.47	9.48
OsR498G0204555400.01	0.05	1.166	4.5891198	1.03E-12	1.47E-12	0.15	0	0	1.56	0.92	1.02
OsR498G0204565000.01	7.603	15.91	1.0890886	3.68E-65	2.43E-65	8.16	7.19	7.46	15.7	15.75	16.28
OsR498G0204565900.01	15.853	47.216	1.5734235	4.85E-262	1.14E-262	14.17	16.68	16.71	48.04	46.32	47.29
OsR498G0204569800.01	2.09	5.62	1.4166752	2.59E-48	2.03E-48	1.94	2.05	2.28	6.04	4.75	6.07
OsR498G0204570000.01	0.666	1.933	1.5289988	4.59E-05	8.69E-05	0.5	1.13	0.37	1.47	1.83	2.5
OsR498G0204572300.01	18.526	51.57	1.4762377	0	0	19.02	19.74	16.82	51.85	54.24	48.62
OsR498G0204574100.01	0	0.186	4.8813006	3.98E-05	7.52E-05	0	0	0	0.26	0.07	0.23
OsR498G0204575600.01	2.606	0.493	-2.620783	4.31E-48	3.39E-48	2.09	1.93	3.8	0.7	0.4	0.38
OsR498G0204577600.01	22.673	47.75	1.0643132	1.29E-142	5.03E-143	22.86	22.62	22.54	46.31	46.81	50.13
OsR498G0204579800.01	4.476	13.32	1.5703603	1.21E-50	9.21E-51	3.96	4.3	5.17	12.87	12.03	15.06
OsR498G0204580300.01	0.593	5.696	3.3057984	2.75E-139	1.09E-139	0.61	0.56	0.61	5.81	5.97	5.31
OsR498G0204580600.01	198.516	84.566	-1.230931	0	0	195.32	205.23	195	83.11	85.47	85.12
OsR498G0204582800.01	17.42	35.44	1.0279171	1.39E-78	8.21E-79	17.05	16.93	18.28	35.72	35.88	34.72
OsR498G0204583700.01	0.02	0.21	3.4338416	7.99E-06	1.46E-05	0	0	0.06	0.17	0.2	0.26
OsR498G0204585000.01	0.073	0.25	1.7520175	0.0009565	0.0019907	0.03	0.16	0.03	0.31	0.28	0.16
OsR498G0204586500.01	0.996	0.256	-1.900059	4.46E-06	8.04E-06	1.33	0.51	1.15	0.08	0.56	0.13
OsR498G0204589700.01	4.676	9.453	1.031834	5.59E-68	3.58E-68	4.66	4.57	4.8	9.43	9.77	9.16
OsR498G0204592100.01	4.333	11.6	1.4230782	2.19E-57	1.56E-57	3.54	5.08	4.38	11.09	12.37	11.34
OsR498G0204592300.01	75.153	243.54	1.6902779	0	0	74.33	78.17	72.96	241.96	245.73	242.94
OsR498G0204592600.01	7.353	47.743	2.6983489	0	0	7.29	7.74	7.03	47.31	46.08	49.84
OsR498G0204592900.01	0.583	2.716	2.2298043	3.98E-42	3.36E-42	0.67	0.49	0.59	2.58	3.02	2.55
OsR498G0204593700.01	5844.83	2499.6	-1.224842	0	0	5648.8	5967.2	5918.6	2519.1	2513.6	2466.3
OsR498G0204597500.01	25.096	91.6	1.8592769	0	0	24.49	26.28	24.52	92.9	92.81	89.09
OsR498G0204598100.01	0.183	2.803	3.9401942	1.05E-78	6.21E-79	0.14	0.3	0.11	3.3	2.91	2.2
OsR498G0204598300.01	10.756	46.873	2.1335234	1.12E-255	2.70E-256	10.91	10.12	11.24	44.55	47.75	48.32
OsR498G0204598600.01	0.07	0.193	1.4544029	3.15E-05	5.93E-05	0.06	0.12	0.03	0.25	0.15	0.18
OsR498G0204599700.01	0.54	1.216	1.2194947	1.44E-09	2.26E-09	0.42	0.85	0.35	1.01	1.36	1.28
OsR498G0204600600.01	39.313	15.82	-1.314296	2.88E-165	1.01E-165	43.53	37.42	36.99	15.25	15.25	16.96
OsR498G0204601200.01	0.936	0.22	-2.041892	1.89E-11	2.79E-11	0.95	0.82	1.04	0.25	0.25	0.16
OsR498G0204605700.01	5.553	11.613	1.0662024	3.47E-61	2.38E-61	5.63	5.25	5.78	12.91	11.52	10.41
OsR498G0204612300.01	150.013	6.42	-4.548803	0	0	154.06	146.02	149.96	5.97	5.92	7.37
OsR498G0204612600.01	13.853	0.7	-4.305694	9.24E-231	2.45E-231	15.7	9.94	15.92	0.67	0.44	0.99
OsR498G0204615400.01	197.08	411.54	1.1340457	0	0	210.81	187.75	192.68	407.85	402.21	424.57
OsR498G0204616300.01	4.446	13.636	1.6133281	1.97E-58	1.39E-58	4.65	3.99	4.7	13.34	13.4	14.17
OsR498G0204620100.01	0.383	2.773	2.9318495	1.27E-55	9.23E-56	0.32	0.35	0.48	2.52	2.92	2.88
OsR498G0204622400.01	332.546	148.17	-1.159299	0	0	334.66	336.85	326.13	147.17	150.3	147.03
OsR498G0204624800.01	2.493	5.42	1.0754399	6.40E-19	7.84E-19	2.49	2.49	2.5	5.39	4.87	6
OsR498G0204626200.01	3.853	1.773	-1.122946	2.20E-27	2.31E-27	3.9	4.58	3.08	1.27	2.02	2.03
OsR498G0204628700.01	5.543	0.53	-3.3944	7.98E-72	4.94E-72	5.05	6	5.58	0.51	0.54	0.54
OsR498G0204635700.01	2.033	8.166	1.9906963	1.50E-84	8.48E-85	1.9	1.94	2.26	8.26	8.02	8.22
OsR498G0204636000.01	0.05	0.476	3.2531028	3.43E-07	5.89E-07	0.08	0	0.07	0.58	0.39	0.46

OsR498G0204636900.01	0.533	3.296	2.6290814	3.01E-93	1.59E-93	0.65	0.58	0.37	3.16	3.57	3.16
OsR498G0204640500.01	2.126	4.413	1.0519709	2.79E-16	3.63E-16	1.98	2.03	2.37	3.73	4.75	4.76
OsR498G0204640800.01	0.14	1.653	3.5664272	4.65E-41	3.99E-41	0.15	0.17	0.1	1.73	1.6	1.63
OsR498G0204641800.01	0.05	0.31	3.4555366	2.46E-14	3.35E-14	0.09	0.06	0	0.38	0.21	0.34
OsR498G0204646800.01	0.136	0.476	1.7520175	6.76E-06	1.23E-05	0.17	0.16	0.08	0.4	0.49	0.54
OsR498G0204648100.01	6.896	13.876	1.0101292	2.43E-114	1.11E-114	6.75	6.94	7	13.91	13.82	13.9
OsR498G0204648300.01	1.093	3.456	1.6881058	8.26E-29	8.48E-29	0.91	0.9	1.47	3.02	3.55	3.8
OsR498G0204649300.01	0.49	2.42	2.2963381	1.73E-26	1.85E-26	0.37	0.51	0.59	1.99	2.76	2.51
OsR498G0204651300.01	3.93	8.036	1.0398838	2.90E-13	4.06E-13	4.39	2.73	4.67	7.27	8.39	8.45
OsR498G0204653400.01	103.463	24.15	-2.09269	0	0	100.97	105.4	104.02	24.56	23.4	24.49
OsR498G0204656700.01	2.5	17.3	2.5099686	9.57E-241	2.45E-241	2.23	2.84	2.43	16.49	17.7	17.71
OsR498G0204657000.01	0.753	5.766	2.8786988	2.15E-89	1.17E-89	0.73	0.55	0.98	5.83	5.7	5.77
OsR498G0204657300.01	2.183	5.39	1.3106933	3.13E-13	4.38E-13	1.88	2.63	2.04	5.07	4.52	6.58
OsR498G0204657600.01	21.23	110.08	2.3697591	0	0	21	21.46	21.23	107.05	104.4	118.8
OsR498G0204658200.01	0.83	23.7	4.832391	3.84E-146	1.46E-146	0.34	0.91	1.24	23.71	25.05	22.34
OsR498G0204658500.01	2.993	103.01	5.1024801	0	0	2.92	3.43	2.63	105.04	101.13	102.87
OsR498G0204659500.01	67.23	159.23	1.2437834	0	0	67.39	69.16	65.14	159.78	161.52	156.39
OsR498G0204663500.01	3.38	6.876	1.0243509	4.63E-20	5.55E-20	3.22	3.55	3.37	7.35	6.3	6.98
OsR498G0204663700.01	0.04	1.81	5.4979719	1.58E-12	2.25E-12	0	0.12	0	2.34	1.17	1.92
OsR498G0204664300.01	0.93	3.3	1.8216209	2.22E-35	2.06E-35	1.1	0.93	0.76	3.23	3.1	3.57
OsR498G0204670000.01	13.45	36.676	1.4465224	2.29E-140	9.05E-141	13.63	13.23	13.49	36.26	36.78	36.99
OsR498G0204672700.01	0.81	0.313	-1.218235	5.37E-05	0.0001024	0.94	0.64	0.85	0.3	0.33	0.31
OsR498G0204673000.01	6.596	18.08	1.4535778	5.84E-93	3.09E-93	6.89	6.59	6.31	19.54	17.07	17.63
OsR498G0204674100.01	56.313	176.27	1.6673575	0	0	55.53	58.06	55.35	176.07	175.7	177.03
OsR498G0204674400.01	2.22	5.416	1.2870002	1.64E-14	2.22E-14	2.8	2	1.86	5.31	5.21	5.73
OsR498G0204677500.01	0.526	0.026	-4.832945	3.08E-08	5.06E-08	0.64	0.54	0.4	0	0.08	0
OsR498G0204681600.01	0.226	1.186	2.4006747	2.36E-07	4.03E-07	0.51	0.17	0	0.9	1.23	1.43
OsR498G0204682100.01	4.013	10.54	1.3960217	2.15E-88	1.18E-88	4	4.05	3.99	10.5	11.14	9.98
OsR498G0204682900.01	7.453	2.566	-1.572931	4.97E-59	3.47E-59	7.76	7.92	6.68	2.58	2.48	2.64
OsR498G0204683700.01	59.75	143.31	1.2626164	0	0	60.74	61.05	57.46	145.33	144.85	139.75
OsR498G0204684400.01	6.153	1.023	-2.582808	1.95E-31	1.92E-31	5.85	6.17	6.44	0.87	1.39	0.81
OsR498G0204684900.01	0.266	1.58	2.5593725	1.32E-18	1.63E-18	0.24	0.32	0.24	1.53	1.56	1.65
OsR498G0204687800.01	27.993	11.12	-1.337534	1.84E-108	8.73E-109	28.38	26.3	29.3	10.48	10.69	12.19
OsR498G0204689800.01	0.083	6.006	6.1608746	8.83E-20	1.06E-19	0	0	0.25	4.42	2.9	10.7
OsR498G0204693200.01	1.526	5.136	1.8058104	1.06E-23	1.19E-23	1.22	1.71	1.65	4.31	5.84	5.26
OsR498G0204693400.01	27.966	60.306	1.1014726	0	0	28.71	27.72	27.47	59.03	62.5	59.39
OsR498G0204695000.01	7.27	19.413	1.4286453	2.86E-64	1.90E-64	7.37	7.17	7.27	16.73	20.48	21.03
OsR498G0204695100.01	2.883	7.066	1.2920251	1.60E-29	1.63E-29	2.37	2.99	3.29	7.28	7.04	6.88
OsR498G0204696500.01	1.81	7.23	2.0060556	1.61E-54	1.18E-54	1.9	1.62	1.91	6.86	7.86	6.97
OsR498G0204697000.01	0.13	0.636	2.2963381	4.69E-06	8.47E-06	0.22	0.17	0	0.54	0.59	0.78
OsR498G0204698800.01	211.733	84.773	-1.350202	0	0	209.99	214.6	210.61	85.41	83.84	85.07
OsR498G0204699100.01	0.51	1.27	1.3132119	0.0004058	0.0008174	0.36	0.36	0.81	1.41	1.05	1.35
OsR498G0204702700.01	0.57	15.14	4.7241996	1.18E-128	5.00E-129	0.49	0.57	0.65	13.81	16.38	15.23
OsR498G0204702900.01	0	0.546	4.97441	2.21E-05	4.12E-05	0	0	0	0.41	0.61	0.62
OsR498G0204703200.01	0.143	0.386	1.524607	0.0001302	0.0002545	0.1	0.13	0.2	0.58	0.27	0.31
OsR498G0204704700.01	5.016	34.06	2.7616637	0	0	5.19	5.27	4.59	34.59	34.8	32.79
OsR498G0204705500.01	0.11	0.343	1.6442614	0.0001813	0.0003571	0.12	0.15	0.06	0.44	0.2	0.39
OsR498G0204705800.01	2.836	10.526	1.8989577	2.01E-59	1.40E-59	2.68	2.82	3.01	10.58	10.84	10.16
OsR498G0204706400.01	49.55	141.42	1.5132133	0	0	48.42	50.12	50.11	143.77	139.75	140.73
OsR498G0204706700.01	2.17	4.963	1.2523947	2.28E-27	2.40E-27	2.15	2.28	2.08	4.64	5.56	4.69
OsR498G0204707300.01	45.113	94.82	1.0029642	4.52E-259	1.08E-259	45.77	45.49	44.08	96.03	94.32	94.11
OsR498G0204710500.01	8.94	3.813	-1.220914	1.53E-33	1.46E-33	8.4	8.52	9.9	3.58	4.03	3.83
OsR498G0204714200.01	9.813	23.46	1.2551857	0	0	10.01	9.62	9.81	23.96	24.18	22.24
OsR498G0204714800.01	0.783	0.09	-3.141067	2.67E-06	4.76E-06	0.82	0.54	0.99	0.09	0.09	0.09
OsR498G0204720900.01	8.87	47.126	2.4038601	0	0	8.33	8.53	9.75	46.59	47.44	47.35
OsR498G0204722300.01	13.55	55.356	2.0128841	5.32E-192	1.65E-192	15.17	12	13.48	56.68	52.37	57.02
OsR498G0204722500.01	0.24	0.91	1.9093149	4.14E-08	6.83E-08	0.17	0.25	0.3	0.92	0.95	0.86
OsR498G0204725800.01	21.14	44.94	1.0820855	1.27E-147	4.80E-148	20.02	21.98	21.42	45.6	43.94	45.28
OsR498G0204726900.01	1.886	4.26	1.2027516	7.69E-30	7.76E-30	1.78	1.62	2.26	4.48	4.14	4.16
OsR498G0204727300.01	3.23	17.25	2.4161474	0	0	3.2	3.3	3.19	17.45	17.94	16.36
OsR498G0204727600.01	0.743	5.553	2.8890779	2.42E-90	1.31E-90	0.79	0.76	0.68	5.5	5.38	5.78
OsR498G0204727800.01	13.613	57.13	2.0445057	0	0	13.36	12.68	14.8	56.57	57.56	57.26
OsR498G0204728000.01	12.28	30.053	1.2816148	4.16E-230	1.11E-230	12.86	12.06	11.92	30.4	30.88	28.88
OsR498G0204728100.01	2.79	13.006	2.574353	0	0	2.79	2.81	2.77	13.52	13.33	12.17
OsR498G0204728400.01	0	3.14	7.9805958	1.60E-28	1.65E-28	0	0	0	3	2.34	4.08
OsR498G0204728500.01	0.043	0.743	4.1063934	3.02E-06	5.39E-06	0	0.13	0	0	0	2.23
OsR498G0204729000.01	0.993	5.626	2.4933749	1.01E-33	9.59E-34	0.73	1.16	1.09	5.77	4.75	6.36
OsR498G0204732200.01	0.1	2.196	4.4901098	3.96E-42	3.34E-42	0.11	0.15	0.04	2.32	2.03	2.24
OsR498G0204732500.01	0.8	6.523	3.0306782	1.10E-62	7.44E-63	1.15	0.76	0.49	5.83	6.23	7.51

OsR498G0204735300.01	2.343	8.706	1.8994479	8.66E-89	4.72E-89	1.94	1.89	3.2	9.46	8.51	8.15
OsR498G0204736200.01	8.323	17.276	1.056264	3.28E-130	1.37E-130	8.39	8.68	7.9	17.21	18.1	16.52
OsR498G0204737000.01	0.763	5.106	2.7441079	2.71E-131	1.13E-131	0.82	0.72	0.75	5.16	5.45	4.71
OsR498G0204738600.01	1.09	12.2	3.5593725	4.09E-210	1.17E-210	1.24	1.03	1	11.54	12.49	12.57
OsR498G0204741800.01	0.166	0.006	-4.549152	6.03E-07	1.05E-06	0.2	0.15	0.15	0	0	0.02
OsR498G0204743200.01	0	0.2	5.0618728	1.23E-05	2.27E-05	0	0	0	0.21	0.14	0.25
OsR498G0204743700.01	0.95	2.513	1.4088128	4.37E-08	7.23E-08	1.16	0.54	1.15	2.25	2.68	2.61
OsR498G0204744000.01	5.69	20.016	1.8126589	1.14E-44	9.29E-45	5.19	5.82	6.06	19.09	19.89	21.07
OsR498G0204744400.01	0.513	7.203	3.8026907	6.25E-127	2.66E-127	0.42	0.38	0.74	7.41	6.76	7.44
OsR498G0204746400.01	0.513	0.093	-2.458549	2.15E-05	4.00E-05	0.52	0.51	0.51	0.11	0.06	0.11
OsR498G0204749000.01	21.063	46.98	1.1514469	8.40E-147	3.20E-147	20.43	20.82	21.94	48.14	48.38	44.42
OsR498G0204755400.01	4.673	19.353	2.0494552	2.12E-273	4.82E-274	4.79	4.57	4.66	20.13	18.07	19.86
OsR498G0204758000.01	0.666	1.883	1.4834236	6.70E-12	9.76E-12	0.54	0.88	0.58	1.66	1.81	2.18
OsR498G0204758500.01	66.99	135.14	1.0091754	1.53E-260	3.62E-261	66.04	69.83	65.1	138.4	133.42	133.61
OsR498G0204761200.01	0.416	0.24	-1.084484	0.0008487	0.001757	0.5	0.37	0.38	0.18	0.15	0.39
OsR498G0204761600.01	74.776	25.613	-1.517734	0	0	75.29	73.37	75.67	25.55	25.88	25.41
OsR498G0204762300.01	334.42	106.53	-1.650885	0	0	347.77	322.72	332.77	108.12	102.6	108.88
OsR498G0204764100.01	19.693	72.23	1.868651	0	0	18.69	20.9	19.49	69.19	70.42	77.08
OsR498G0204765300.01	0.843	3.063	1.8567584	1.51E-30	1.50E-30	0.89	0.82	0.82	2.98	2.84	3.37
OsR498G0204771300.01	0.103	0	-4.72603	0.0001114	0.0002167	0.12	0.07	0.12	0	0	0
OsR498G0204772800.01	12.366	25.076	1.0240237	1.38E-98	7.03E-99	12.43	11.99	12.68	26.85	24.16	24.22
OsR498G0204775600.01	4.766	10.98	1.20531	8.68E-75	5.26E-75	4.78	5.2	4.32	10.63	11.06	11.25
OsR498G0204776600.01	4.73	36.98	2.9587616	0	0	4.07	5.33	4.79	36.97	38.41	35.56
OsR498G0204781000.01	2.196	4.743	1.1058939	3.24E-14	4.43E-14	2.63	1.8	2.16	4.07	4.06	6.1
OsR498G0204781600.01	5.353	11.593	1.1286002	5.19E-77	3.10E-77	6.03	5.11	4.92	11.62	12.2	10.96
OsR498G0204782700.01	308.983	128.07	-1.264768	0	0	286.43	294.4	346.12	130.64	122.75	130.83
OsR498G0204783300.01	3.61	8	1.1474747	1.04E-38	9.16E-39	3.75	3.47	3.61	7.84	8.07	8.09
OsR498G0204783600.01	1.293	4.656	1.8468122	4.00E-45	3.25E-45	1.48	1.06	1.34	4.62	4.75	4.6
OsR498G0204790000.01	0.003	0.063	3.6748497	0.0003128	0.0006259	0	0	0.01	0.09	0.04	0.06
OsR498G0204794900.01	0.22	1.603	2.8674948	3.13E-18	3.89E-18	0.19	0.28	0.19	1.7	1.37	1.74
OsR498G0204795300.01	0.89	0.213	-2.095979	3.23E-11	4.80E-11	0.94	1	0.73	0.25	0.2	0.19
OsR498G0204795900.01	17.806	52.54	1.559069	0	0	17.93	17.2	18.29	51.27	48.82	57.53
OsR498G0204800300.01	1.033	2.043	1.0064221	1.03E-11	1.51E-11	1.25	1.04	0.81	1.68	1.87	2.58
OsR498G0204802800.01	0.026	0.26	3.2223375	4.64E-05	8.81E-05	0.04	0.04	0	0.2	0.37	0.21
OsR498G0204803600.01	1.596	58.563	5.1914347	0	0	1.32	1.86	1.61	57.49	54.97	63.23
OsR498G0204807600.01	29.756	62.633	1.0465597	5.34E-144	2.07E-144	28.72	28.94	31.61	62.37	61.29	64.24
OsR498G0204807700.01	7.706	16.513	1.097452	1.94E-53	1.43E-53	8.78	6.66	7.68	16.15	15.35	18.04
OsR498G0204808100.01	0.233	7.74	5.0524125	1.58E-71	9.84E-72	0.16	0.31	0.23	7.05	7.93	8.24
OsR498G0204811000.01	3.603	1.68	-1.101941	1.53E-14	2.08E-14	4.48	3.66	2.67	1.92	1.33	1.79
OsR498G0204812100.01	6.38	13.933	1.1336472	1.09E-57	7.72E-58	6.32	5.81	7.01	14.09	13.81	13.9
OsR498G0204815600.01	386.4	159.79	-1.291972	0	0	388.94	392.46	377.8	150.17	161.48	167.73
OsR498G0204815800.01	8.06	25.146	1.4377474	6.22E-92	3.32E-92	7.6	8.28	8.3	27.09	23.61	24.74
OsR498G0204819000.01	3.41	6.986	1.0310994	7.80E-23	8.86E-23	3.92	3.11	3.2	7.17	6.71	7.08
OsR498G0204819800.01	10.596	4.963	-1.085637	1.26E-19	1.53E-19	10.44	12.06	9.29	4.93	4.17	5.79
OsR498G0204820200.01	5.406	16.283	1.6023688	2.95E-101	1.47E-101	5.6	5.2	5.42	17.34	16.1	15.41
OsR498G0204823700.01	5.746	39.276	2.7635209	0	0	5.98	5.11	6.15	39.44	39.79	38.6
OsR498G0204825100.01	2.763	0.16	-4.273518	1.78E-18	2.21E-18	3.63	2.7	1.96	0.2	0.1	0.18
OsR498G0204825700.01	0.806	2.22	1.4625109	3.02E-16	3.93E-16	0.54	0.97	0.91	2.29	1.92	2.45
OsR498G0204826000.01	227.423	54.056	-2.07045	0	0	225.07	227.78	229.42	53.34	51.5	57.33
OsR498G0204826400.01	0	0.226	4.2963381	0.0007942	0.0016388	0	0	0	0.07	0.33	0.28
OsR498G0204827600.01	3.966	1.45	-1.455005	1.97E-31	1.94E-31	3.71	4.18	4.01	1.48	1.29	1.58
OsR498G0204829100.01	33.793	11.57	-1.547127	5.22E-133	2.16E-133	33.89	33.81	33.68	10.95	12.12	11.64
OsR498G0204829400.01	7.323	16.673	1.1874413	5.63E-86	3.14E-86	7.44	6.9	7.63	16.75	16.34	16.93
OsR498G0204831100.01	1.573	4.156	1.4110497	1.56E-41	1.33E-41	1.31	1.82	1.59	4.31	4.42	3.74
OsR498G0204833000.01	6.49	16.84	1.3713827	3.03E-72	1.87E-72	6.83	6.3	6.34	17.1	16.35	17.07
OsR498G0204833100.01	0.026	1.203	5.4501434	1.50E-22	1.72E-22	0	0.04	0.04	1.33	1.2	1.08
OsR498G0204835000.01	1.023	25.933	4.6575044	0	0	1.09	0.94	1.04	23.24	25.78	28.78
OsR498G0204835200.01	8.483	21.943	1.3696868	3.60E-79	2.11E-79	7.67	8.9	8.88	20.4	23.52	21.91
OsR498G0204835400.01	1.956	0.593	-1.711836	6.09E-17	7.80E-17	1.6	1.98	2.29	0.71	0.74	0.33
OsR498G0204835800.01	0.733	0.18	-1.832945	1.10E-07	1.85E-07	0.67	0.7	0.83	0.11	0.16	0.27
OsR498G0204839800.01	0.083	0.006	-3.507913	0.0005349	0.0010869	0.08	0.1	0.07	0	0.02	0
OsR498G0204841500.01	1.776	4.416	1.276447	2.81E-52	2.11E-52	1.72	1.73	1.88	4.17	4.51	4.57
OsR498G0204842000.01	32.1	66.55	1.051751	1.92E-165	6.73E-166	33.42	32.1	30.78	64.61	66.16	68.88
OsR498G0204842400.01	1.076	3.01	1.491201	2.14E-27	2.26E-27	0.99	1.13	1.11	3.33	3.19	2.51
OsR498G0204843700.01	0	0.17	4.6748497	0.0001298	0.0002536	0	0	0	0.23	0.04	0.24
OsR498G0204845000.01	0.083	0.853	3.3473661	1.72E-27	1.81E-27	0.09	0.07	0.09	0.9	0.92	0.74
OsR498G0204846200.01	3.056	6.696	1.1317349	7.84E-25	8.60E-25	3.33	3.05	2.79	6.49	6.93	6.67
OsR498G0204849000.01	0.626	0.153	-2.227224	3.87E-10	5.95E-10	0.6	0.52	0.76	0.08	0.19	0.19
OsR498G0204849400.01	19.666	72.976	1.8919478	0	0	20.17	19.68	19.15	73.08	74.57	71.28

OsR498G0204850500.01	0.693	1.986	1.526951	3.37E-11	5.02E-11	0.57	0.73	0.78	1.9	1.96	2.1
OsR498G0204854200.01	0.193	0.863	2.167055	2.13E-05	3.97E-05	0.08	0.25	0.25	0.97	1.12	0.5
OsR498G0204855900.01	0.4	2.513	2.8488791	2.10E-29	2.14E-29	0.5	0.45	0.25	1.95	3.03	2.56
OsR498G0204857800.01	6.696	13.573	1.0950693	2.21E-44	1.81E-44	7.34	6.7	6.05	13.29	14.07	13.36
OsR498G0204858200.01	1.046	3.773	1.8490557	1.28E-19	1.55E-19	1.2	1.06	0.88	4.92	2.71	3.69
OsR498G0204858400.01	0.386	1.103	1.4803009	2.64E-16	3.43E-16	0.32	0.36	0.48	1.23	1.1	0.98
OsR498G0204860400.01	5.92	2.76	-1.102714	8.42E-18	1.06E-17	5.19	5.62	6.95	2.94	2.22	3.12
OsR498G0204866800.01	3.45	7.286	1.0798932	1.19E-43	9.87E-44	3.2	3.5	3.65	8.13	7.56	6.17
OsR498G0204869500.01	0.07	0	-4.610553	0.0002051	0.000405	0.1	0.03	0.08	0	0	0
OsR498G0204870400.01	8.48	1.11	-2.935125	4.19E-136	1.69E-136	8.9	8.47	8.07	1.18	1.15	1
OsR498G0204873100.01	1.5	3.823	1.3726298	5.60E-26	6.03E-26	1.41	1.81	1.28	3.89	3.99	3.59
OsR498G0204875400.01	2.416	5.903	1.2850522	2.80E-12	4.03E-12	2.08	1.88	3.29	5.16	6.23	6.32
OsR498G0204887100.01	0.706	3.766	2.4197502	3.47E-115	1.58E-115	0.72	0.73	0.67	3.54	4.12	3.64
OsR498G0204889600.01	32.166	16.03	-1.090542	3.63E-96	1.88E-96	31.02	31.38	34.1	15.19	16.43	16.47
OsR498G0204893200.01	2.74	6.843	1.340652	6.47E-84	3.67E-84	2.66	2.76	2.8	7.24	7.09	6.2
OsR498G0204894500.01	0	0.276	4.4338416	0.0004331	0.0008746	0	0	0	0.15	0.45	0.23
OsR498G0204900900.01	25.613	75.603	1.5593725	2.32E-222	6.32E-223	25.84	26.47	24.53	74.72	70.38	81.71
OsR498G0204901100.01	0.92	2.19	1.3024642	1.20E-21	1.40E-21	0.87	1.16	0.73	2.21	2.41	1.95
OsR498G0204905300.01	1.156	0.073	-5.36544	5.49E-21	6.46E-21	1.64	0.7	1.13	0.11	0.11	0
OsR498G0204906600.01	2.313	5.96	1.3614331	1.91E-36	1.74E-36	2.4	2.53	2.01	5.64	5.49	6.75
OsR498G0204908700.01	0.653	2.8	2.1200153	1.30E-57	9.22E-58	0.75	0.6	0.61	2.69	2.74	2.97
OsR498G0204910500.01	13.026	46.166	1.824849	2.31E-261	5.43E-262	12.53	13.2	13.35	50.04	43.02	45.44
OsR498G0204911000.01	2.306	4.826	1.0660404	2.16E-12	3.10E-12	2.51	2.37	2.04	4.75	4.73	5
OsR498G0204911800.01	0	0.106	4.8813006	3.98E-05	7.52E-05	0	0	0	0.15	0.04	0.13
OsR498G0204913700.01	3.153	6.513	1.0451627	4.17E-33	3.99E-33	3	3.26	3.2	6.59	6.39	6.56
OsR498G0204919300.01	2.26	5.916	1.387892	6.14E-40	5.35E-40	2.15	2.35	2.28	6.07	5.5	6.18
OsR498G0204925200.01	0.486	0.103	-2.258251	1.51E-07	2.55E-07	0.42	0.47	0.57	0.16	0.05	0.1
OsR498G0204925300.01	186.736	24.546	-2.932007	0	0	173.42	187.44	199.35	21.03	21.48	31.13
OsR498G0204925700.01	0.106	1.813	4.1342813	4.79E-32	4.67E-32	0.08	0.12	0.12	1.8	1.81	1.83
OsR498G0204928300.01	0.276	0.603	1.2519737	5.98E-08	9.95E-08	0.39	0.29	0.15	0.67	0.51	0.63
OsR498G0204928600.01	4.186	16.703	1.9937076	1.47E-50	1.12E-50	3.96	4.4	4.2	15.95	15.76	18.4
OsR498G0204932400.01	1.063	6.593	2.6204671	3.02E-119	1.34E-119	1.09	1.08	1.02	6.73	6.71	6.34
OsR498G0204934100.01	0.146	1.606	3.4338416	7.99E-06	1.46E-05	0	0	0.44	2.84	1.31	0.67
OsR498G0204936900.01	4.176	9.093	1.1784676	8.87E-22	1.03E-21	3.64	4.34	4.55	9.17	9.02	9.09
OsR498G0204938700.01	10.583	44.493	2.0696905	7.31E-117	3.30E-117	11.9	8.54	11.31	44.49	42.82	46.17
OsR498G0204940000.01	8.003	20.196	1.3025212	5.16E-152	1.92E-152	7.87	7.55	8.59	19.8	20.25	20.54
OsR498G0204944700.01	5.993	22.596	1.9205664	1.53E-192	4.73E-193	5.8	6.73	5.45	23.37	22.22	22.2
OsR498G0204946100.01	1.003	0.33	-1.653621	4.34E-10	6.68E-10	1.41	0.69	0.91	0.23	0.46	0.3
OsR498G0204947800.01	5.53	0.976	-2.507242	1.16E-33	1.10E-33	4.66	5.92	6.01	0.92	0.99	1.02
OsR498G0204958300.01	4.396	0.8	-2.454194	1.42E-64	9.39E-65	4.47	4.28	4.44	0.97	0.8	0.63
OsR498G0204959200.01	43.66	108.71	1.3150186	0	0	44.64	40.97	45.37	112.7	108.21	105.21
OsR498G0204959400.01	0.5	0.126	-2.006481	9.75E-10	1.52E-09	0.4	0.48	0.62	0.14	0.12	0.12
OsR498G0204960500.01	8.61	23.94	1.5167787	1.40E-196	4.26E-197	9.66	8.46	7.71	24.9	23.27	23.65
OsR498G0204961100.01	1.09	3.156	1.6392258	4.59E-12	6.65E-12	0.76	1.08	1.43	2.92	3.62	2.93
OsR498G0204961500.01	2.326	6.336	1.4223121	1.03E-39	9.00E-40	2.21	2.59	2.18	6.61	6.86	5.54
OsR498G0204961700.01	2.593	6.11	1.2189588	7.72E-45	6.28E-45	2.44	2.42	2.92	6.19	5.79	6.35
OsR498G0204964600.01	11.546	51.046	2.1409203	1.09E-144	4.20E-145	10.6	11.79	12.25	49.36	46.17	57.61
OsR498G0204964700.01	2.46	9.256	1.8892426	1.20E-58	8.45E-59	2.59	2.61	2.18	9.35	8.86	9.56
OsR498G0204965200.01	10.87	24.453	1.1694115	1.19E-125	5.12E-126	11.35	9.71	11.55	24.49	24.4	24.47
OsR498G0204965900.01	0.08	0.423	2.4237174	3.91E-11	5.84E-11	0.06	0.07	0.11	0.55	0.39	0.33
OsR498G0204968000.01	0.393	2.98	2.8166705	1.11E-40	9.60E-41	0.49	0.51	0.18	2.81	3.3	2.83
OsR498G0204968900.01	18.146	47.226	1.3797855	1.42E-201	4.23E-202	18.08	18.29	18.07	49.07	46.22	46.39
OsR498G0204969200.01	3.793	7.856	1.0683861	2.01E-15	2.66E-15	2.94	4.74	3.7	7.81	7.57	8.19
OsR498G0204969400.01	61.98	30.37	-1.040667	0	0	63.28	62.48	60.18	30.4	31.07	29.64
OsR498G0204972300.01	2.506	18.71	2.9013702	0	0	2.39	2.77	2.36	17.87	18.44	19.82
OsR498G0204976100.01	2.486	6.96	1.4872714	3.34E-51	2.54E-51	2.47	2.25	2.74	7.33	7.24	6.31
OsR498G0204976400.01	2.163	7.776	1.8469907	3.20E-49	2.48E-49	2.58	1.72	2.19	7.86	6.83	8.64
OsR498G0204977000.01	0	0.263	5.2671917	2.76E-06	4.93E-06	0	0	0	0.22	0.22	0.35
OsR498G0204977600.01	0.063	0.583	3.2411965	1.87E-15	2.48E-15	0.05	0.09	0.05	0.54	0.56	0.65
OsR498G0204980200.01	0.59	3.626	2.6859049	1.14E-59	7.92E-60	0.6	0.57	0.6	2.92	4.21	3.75
OsR498G0204983100.01	3.693	10.396	1.4912815	1.10E-132	4.56E-133	3.61	3.65	3.82	10.27	10.54	10.38
OsR498G0204985000.01	1	0.313	-1.724822	0.000157	0.0003081	1.15	0.75	1.1	0.3	0.35	0.29
OsR498G0204985100.01	24.013	81.38	1.7392735	0	0	22.96	24.17	24.91	79.86	86.14	78.14
OsR498G0204994100.01	4.343	9.173	1.078607	4.91E-66	3.20E-66	4.39	4.12	4.52	9.16	9.61	8.75
OsR498G0204995100.01	0.11	0.776	2.8113478	2.40E-19	2.91E-19	0.08	0.11	0.14	0.84	0.65	0.84
OsR498G0204995300.01	0.316	1.223	1.9456336	4.17E-06	7.50E-06	0.18	0.57	0.2	1.48	1.16	1.03
OsR498G0204995900.01	49.863	218.73	2.1364778	0	0	51.02	49.37	49.2	221.04	220.91	214.24
OsR498G0204996300.01	0.07	0.356	2.4149825	9.99E-07	1.75E-06	0.15	0.03	0.03	0.31	0.36	0.4
OsR498G0204998600.01	62.84	275.91	2.1348417	0	0	65.66	61.95	60.91	275.11	269.7	282.92

OsR498G0204998800.01	11.673	26.173	1.1798043	1.92E-152	7.11E-153	12.33	11.78	10.91	26.81	27.19	24.52
OsR498G0205000400.01	0.433	1.086	1.345352	1.26E-11	1.85E-11	0.45	0.48	0.37	1.2	1.14	0.92
OsR498G0205000500.01	16.293	97.206	2.5756743	0	0	15.11	15.06	18.71	97.3	95.82	98.5
OsR498G0205001300.01	2.94	13.05	2.1580969	9.48E-217	2.64E-217	2.89	2.84	3.09	12.88	13.13	13.14
OsR498G0205001700.01	6.773	19.51	1.5240054	7.12E-99	3.61E-99	6.2	7.39	6.73	19.96	18.27	20.3
OsR498G0205001900.01	89.096	25.28	-1.819139	0	0	98.12	81.59	87.58	23.17	24.62	28.05
OsR498G0205002100.01	103.896	10.763	-3.245765	0	0	104.66	103.32	103.71	11.35	9.96	10.98
OsR498G0205003000.01	0.553	1.663	1.6126118	3.01E-11	4.47E-11	0.49	0.62	0.55	1.59	1.83	1.57
OsR498G0205009000.01	10.863	22.256	1.0247198	2.74E-76	1.64E-76	10.71	10.98	10.9	21.81	22.15	22.81
OsR498G0205009300.01	7.516	30.483	2.0167208	8.53E-219	2.35E-219	8.41	7.21	6.93	29.37	29.58	32.5
OsR498G0205010200.01	22.196	52.52	1.2387801	4.05E-303	8.51E-304	22.25	22.5	21.84	53.19	52.83	51.54
OsR498G0205010700.01	294.663	66.76	-2.15993	0	0	300.17	288.88	294.94	68.49	64.62	67.17
OsR498G0205016600.01	0.243	0.61	1.3298906	8.76E-07	1.53E-06	0.22	0.32	0.19	0.69	0.58	0.56
OsR498G0205018400.01	6.716	23.383	1.7103719	4.82E-202	1.43E-202	5.92	7.27	6.96	23.53	24.15	22.47
OsR498G0205021600.01	4.396	11.986	1.4466152	8.61E-69	5.48E-69	4.26	4.42	4.51	12.16	12.4	11.4
OsR498G0205023600.01	2.586	15.583	2.579272	1.45E-104	7.03E-105	2.21	2.32	3.23	14.44	14.34	17.97
OsR498G0205024600.01	0.536	1.28	1.2474285	7.66E-08	1.28E-07	0.51	0.43	0.67	1.38	0.98	1.48
OsR498G0205025100.01	1.366	7.29	2.397909	6.20E-61	4.25E-61	1.36	1.44	1.3	7.87	6.22	7.78
OsR498G0205027800.01	43.63	97.026	1.1517858	3.36E-245	8.49E-246	47.76	44.8	38.33	93.52	95.79	101.77
OsR498G0205028600.01	5.506	14.97	1.412983	1.28E-112	5.95E-113	5.16	5.9	5.46	16.22	14.46	14.23
OsR498G0205029100.01	94.133	291.38	1.6289233	0	0	96.73	91.8	93.87	284.74	283.11	306.28
OsR498G0205029500.01	0.27	0.013	-4.347518	3.68E-06	6.60E-06	0.08	0.4	0.33	0	0	0.04
OsR498G0205031900.01	17.183	3.86	-2.156037	1.12E-91	6.00E-92	16.51	17.17	17.87	3.83	3.75	4
OsR498G0205032200.01	2.283	0.966	-1.228028	4.00E-39	3.53E-39	2.35	2.34	2.16	1.06	1	0.84
OsR498G0205033600.01	10.41	21.173	1.022534	4.18E-33	4.01E-33	10.72	11.03	9.48	21.14	19.6	22.78
OsR498G0205037100.01	3.826	0.643	-2.477286	2.41E-27	2.54E-27	4.28	3.29	3.91	0.72	0.78	0.43
OsR498G0205037700.01	0.326	0	-4.485022	0.0003787	0.0007614	0.27	0.44	0.27	0	0	0
OsR498G0205038400.01	26.9	61.046	1.1818433	0	0	27.97	26.21	26.52	61.22	63.11	58.81
OsR498G0205042800.01	0.536	8.903	4.0590412	4.68E-108	2.23E-108	0.21	0.76	0.64	8.67	8.7	9.34
OsR498G0205043200.01	8.103	2.756	-1.575647	1.65E-57	1.17E-57	7.77	8.24	8.3	2.85	2.81	2.61
OsR498G0205043500.01	15.606	34.933	1.2273303	2.16E-123	9.35E-124	14.63	16.71	15.48	34.52	34.08	36.2
OsR498G0205045700.01	1.243	0.12	-3.447054	1.71E-44	1.40E-44	1.34	1.2	1.19	0.13	0.13	0.1
OsR498G0205046200.01	0.713	0.176	-1.879739	2.60E-06	4.64E-06	0.29	1.12	0.73	0.28	0.09	0.16
OsR498G0205048000.01	0.086	0.366	2.1212513	3.50E-05	6.59E-05	0.11	0.04	0.11	0.28	0.49	0.33
OsR498G0205056400.01	6.756	14.316	1.1097481	6.62E-77	3.95E-77	5.49	7.2	7.58	11.62	13.59	17.74
OsR498G0205056800.01	13.32	31.42	1.2405988	1.43E-134	5.85E-135	12.99	14.1	12.87	31.51	32.43	30.32
OsR498G0205060400.01	7.46	2.716	-1.45583	1.53E-12	2.18E-12	8.89	7.74	5.75	3.16	2.6	2.39
OsR498G0305064700.01	0.01	0.13	3.6748497	0.0003128	0.0006259	0	0	0.03	0.15	0.09	0.15
OsR498G0305065200.01	1.7	0.246	-2.791125	9.31E-18	1.17E-17	1.86	1.79	1.45	0.29	0.15	0.3
OsR498G0305067900.01	0.866	1.763	1.0356971	2.67E-23	3.01E-23	0.92	0.82	0.86	1.63	1.74	1.92
OsR498G0305070200.01	4.336	1.276	-1.759243	4.61E-66	3.01E-66	4.5	4.37	4.14	1.37	1.39	1.07
OsR498G0305073700.01	0.193	0	-4.610553	0.0002051	0.000405	0.29	0.19	0.1	0	0	0
OsR498G0305077600.01	1.396	8.18	2.7870793	1.94E-53	1.43E-53	1.11	1.35	1.73	6.4	9.6	8.54
OsR498G0305080300.01	0	0.61	4.7817649	7.17E-05	0.0001377	0	0	0	0.78	0.39	0.66
OsR498G0305081700.01	11.4	5.563	-1.00066	2.73E-48	2.14E-48	11.25	11.46	11.49	5.22	4.94	6.53
OsR498G0305082200.01	3.163	6.453	1.0009906	6.35E-20	7.63E-20	2.89	3.48	3.12	6.95	6.54	5.87
OsR498G0305083300.01	0.533	2.28	2.0922464	3.07E-24	3.40E-24	0.55	0.54	0.51	1.99	2.05	2.8
OsR498G0305093800.01	4.386	29.5	2.7478471	8.93E-173	3.01E-173	4.38	4.19	4.59	27.62	28.74	32.14
OsR498G0305095300.01	2.003	4.056	1.0775034	3.09E-16	4.02E-16	2.06	1.72	2.23	4.1	3.94	4.13
OsR498G0305096500.01	11.48	24.936	1.1693893	9.71E-177	3.22E-177	10.85	11.57	12.02	24.88	26.15	23.78
OsR498G0305101600.01	1.636	13.596	3.0520474	2.73E-75	1.65E-75	2.37	1.32	1.22	11.71	13.96	15.12
OsR498G0305101800.01	33.186	97.366	1.5497464	0	0	32.8	32.3	34.46	97	97.36	97.74
OsR498G0305102600.01	51.783	24.343	-1.059932	1.40E-144	5.38E-145	52.83	50.47	52.05	24.05	23.94	25.04
OsR498G0305102700.01	35.783	11.97	-1.579188	1.09E-91	5.82E-92	37.52	34.01	35.82	11.67	13.15	11.09
OsR498G0305103900.01	3.746	9.153	1.2740636	4.77E-66	3.11E-66	3.88	3.75	3.61	8.46	10.67	8.33
OsR498G0305106100.01	111.52	27.013	-1.994669	0	0	110.91	112.92	110.73	28.16	26.51	26.37
OsR498G0305107600.01	5.826	100.91	4.145757	0	0	5.43	6.49	5.56	97.69	106.3	98.74
OsR498G0305108300.01	0.603	1.82	1.6259062	1.66E-29	1.68E-29	0.66	0.56	0.59	1.78	1.82	1.86
OsR498G0305108900.01	21.45	43.376	1.0399575	0	0	21.84	21.53	20.98	43.41	46.25	40.47
OsR498G0305109200.01	1.13	2.576	1.1822148	1.35E-17	1.70E-17	1.35	1.08	0.96	2.65	2.44	2.64
OsR498G0305110000.01	0.39	0.023	-4.069984	8.08E-09	1.30E-08	0.36	0.28	0.53	0	0.03	0.04
OsR498G0305110300.01	4.473	13.86	1.6943934	1.58E-78	9.31E-79	5.13	3.79	4.5	13.63	13.48	14.47
OsR498G0305114400.01	5.29	12.43	1.2379625	1.26E-30	1.25E-30	5.64	5.55	4.68	11.64	12.31	13.34
OsR498G0305116100.01	1.333	4.133	1.6255627	1.40E-36	1.28E-36	1.16	1.23	1.61	3.81	4.38	4.21
OsR498G0305116400.01	4.42	14.403	1.7026583	2.03E-70	1.28E-70	4.28	4.19	4.79	13.41	14.8	15
OsR498G0305118700.01	7.646	15.636	1.0280892	2.19E-123	9.48E-124	7.95	7.29	7.7	15.86	16.5	14.55
OsR498G0305119300.01	23.716	64.693	1.4575303	9.39E-234	2.46E-234	23.98	24.64	22.53	62.3	64.03	67.75
OsR498G0305119600.01	0.806	25.116	4.9389113	0	0	0.65	0.84	0.93	24.38	23.59	27.38
OsR498G0305121100.01	0.486	1.613	1.7442902	2.86E-13	4.00E-13	0.44	0.34	0.68	1.65	1.62	1.57

OsR498G0305121500.01	30.863	131.59	2.0924314	0	0	32.05	30.45	30.09	131.93	134.51	128.34
OsR498G0305122600.01	20.663	61.003	1.5814543	0	0	20.74	20.33	20.92	62.07	60.74	60.2
OsR498G0305124000.01	0.243	0.016	-3.447054	0.0005962	0.0012167	0.28	0.28	0.17	0	0	0.05
OsR498G0305126100.01	8.67	1.156	-2.854938	2.86E-107	1.37E-107	8.75	8.91	8.35	1.25	0.97	1.25
OsR498G0305129000.01	9.773	25.89	1.4045909	6.32E-80	3.69E-80	9.12	10.12	10.08	26.58	26.16	24.93
OsR498G0305130200.01	1.513	0.286	-2.388688	1.51E-29	1.53E-29	1.3	1.5	1.74	0.29	0.29	0.28
OsR498G0305130300.01	1.296	0.506	-1.370901	3.93E-06	7.08E-06	1.73	0.89	1.27	0	0.41	1.11
OsR498G0305133100.01	9.346	2.09	-2.168968	2.76E-32	2.68E-32	9.42	8.83	9.79	1.79	1.37	3.11
OsR498G0305135000.01	4.29	0.626	-2.855665	3.88E-63	2.61E-63	4.33	4.3	4.24	0.42	0.62	0.84
OsR498G0305136000.01	0.953	0.44	-1.125126	0.0002099	0.0004146	0.91	1.14	0.81	0.42	0.28	0.62
OsR498G0305140000.01	4.236	1.723	-1.303254	4.75E-21	5.59E-21	3.91	4.58	4.22	1.73	1.43	2.01
OsR498G0305143000.01	24.193	79.226	1.708979	0	0	23.43	23.58	25.57	78.57	77.26	81.85
OsR498G0305143200.01	0.373	1.29	1.7893781	2.83E-10	4.33E-10	0.24	0.44	0.44	1.03	1.44	1.4
OsR498G0305150500.01	3.773	24.643	2.7044592	8.28E-228	2.22E-228	3.32	3.33	4.67	24.07	22.91	26.95
OsR498G0305151900.01	13.273	28.976	1.1947903	1.20E-141	4.71E-142	13.2	13.26	13.36	29	28.23	29.7
OsR498G0305153800.01	3.653	8	1.1325274	8.95E-28	9.37E-28	4.35	2.93	3.68	8.53	7.18	8.29
OsR498G0305155500.01	0.12	0.036	-1.704851	0.00061	0.0012456	0.09	0.18	0.09	0.08	0.03	0
OsR498G0305155800.01	8.51	2.723	-1.66423	5.39E-25	5.89E-25	7.89	8.97	8.67	2.86	3	2.31
OsR498G0305156900.01	9.066	23.796	1.389942	1.76E-98	8.95E-99	8.26	9.02	9.92	24.31	21.28	25.8
OsR498G0305157800.01	13.65	46.563	1.761423	0	0	13.57	13.65	13.73	48.32	47.25	44.12
OsR498G0305158900.01	1.283	3.4	1.4073694	5.12E-12	7.42E-12	1.61	1.09	1.15	3.81	2.99	3.4
OsR498G0305161100.01	0.51	0.13	-1.918675	0.000277	0.0005521	0.13	0.58	0.82	0.1	0.16	0.13
OsR498G0305162900.01	0.2	0	-5.02559	1.85E-05	3.43E-05	0.19	0.07	0.34	0	0	0
OsR498G0305164000.01	0.276	1.366	2.3292527	1.66E-18	2.05E-18	0.31	0.29	0.23	1.16	1.56	1.38
OsR498G0305165100.01	0	0.133	5.1110935	8.75E-06	1.60E-05	0	0	0	0	0.4	0
OsR498G0305166000.01	3.823	15.77	2.0544696	1.18E-179	3.87E-180	3.56	3.46	4.45	16.54	15.48	15.29
OsR498G0305166900.01	1644.36	367.3	-2.162761	0	0	1635.6	1606	1691.5	361.95	362.93	377.02
OsR498G0305170100.01	0.1	2.316	4.5391946	1.51E-17	1.90E-17	0	0.3	0	2.13	1.74	3.08
OsR498G0305170500.01	9.503	30.563	1.6841359	1.69E-183	5.44E-184	9.65	9.64	9.22	29.54	31	31.15
OsR498G0305173100.01	0.633	6.156	3.3689418	2.12E-61	1.45E-61	0.45	0.79	0.66	6	6.11	6.36
OsR498G0305173400.01	15.986	6.716	-1.314677	7.13E-156	2.60E-156	16.11	16.42	15.43	6.55	6.84	6.76
OsR498G0305175400.01	1.1	4.386	1.97441	3.94E-53	2.93E-53	0.87	1.15	1.28	4.49	4.29	4.38
OsR498G0305175900.01	3.19	1.233	-1.374318	6.81E-12	9.92E-12	3.07	2.63	3.87	1.78	0.86	1.06
OsR498G0305176100.01	2.163	0.213	-3.395156	5.96E-29	6.10E-29	2.47	1.83	2.19	0.22	0.28	0.14
OsR498G0305179900.01	6.123	15.703	1.3549658	1.85E-102	9.12E-103	6.14	6.28	5.95	16.52	15.53	15.06
OsR498G0305180300.01	30.22	5.4	-2.51677	7.58E-226	2.05E-226	31.64	29.16	29.86	4.55	5.9	5.75
OsR498G0305180600.01	5.57	22.263	1.9985224	2.06E-151	7.66E-152	5.38	5.56	5.77	22.62	22.57	21.6
OsR498G0305181500.01	5.436	18.533	1.7685687	4.37E-140	1.73E-140	5.05	5.46	5.8	17.72	18.58	19.3
OsR498G0305182200.01	0.31	0.713	1.2022159	3.89E-06	7.00E-06	0.44	0.29	0.2	0.6	0.9	0.64
OsR498G0305186600.01	0.296	0.72	1.2819672	3.08E-05	5.79E-05	0.14	0.4	0.35	0.63	0.66	0.87
OsR498G0305190100.01	1.37	11.833	3.1119135	2.47E-54	1.81E-54	2	0.94	1.17	9.26	11.85	14.39
OsR498G0305190300.01	0.833	2.476	1.5700989	7.58E-32	7.41E-32	0.77	0.98	0.75	2.24	2.59	2.6
OsR498G0305191400.01	7.2	19.3	1.4192401	3.76E-227	1.01E-227	7.28	7.15	7.17	19.16	18.85	19.89
OsR498G0305194500.01	20.16	54.16	1.4148124	4.14E-232	1.09E-232	20.11	20.93	19.44	54.55	53.55	54.38
OsR498G0305194700.01	43.933	172.83	1.9709261	0	0	42.19	46.13	43.48	166.68	166.92	184.88
OsR498G0305197900.01	19.836	51.913	1.3931192	0	0	21.18	20.02	18.31	51.48	54.08	50.18
OsR498G0305201500.01	23.4	131.23	2.4873055	0	0	24.06	22.81	23.33	124.17	127.13	142.39
OsR498G0305207300.01	1.723	0.29	-2.572485	1.37E-28	1.41E-28	1.78	1.51	1.88	0.3	0.21	0.36
OsR498G0305207800.01	0.893	0.633	-1.247982	1.33E-06	2.34E-06	0.93	0.96	0.79	0.99	0.57	0.34
OsR498G0305210300.01	0.67	2.083	1.5756743	9.98E-22	1.16E-21	0.62	0.75	0.64	2.04	2.19	2.02
OsR498G0305210500.01	11.463	38.96	1.7626619	1.70E-142	6.64E-143	10.95	10.94	12.5	36.74	38.09	42.05
OsR498G0305214100.01	0.046	0.936	4.3469641	2.72E-33	2.59E-33	0.04	0.06	0.04	0.88	0.86	1.07
OsR498G0305215500.01	9.153	21.053	1.1823361	6.37E-251	1.57E-251	9.31	9.35	8.8	21.86	21.33	19.97
OsR498G0305215800.01	17.583	67.87	1.9311522	0	0	18.14	17.29	17.32	68.04	68.48	67.09
OsR498G0305217600.01	0.793	4.25	2.4153907	7.87E-68	5.05E-68	0.98	0.66	0.74	5.15	4.09	3.51
OsR498G0305220700.01	0.273	0.776	1.5149783	0.0006351	0.0012984	0.3	0.15	0.37	0.87	0.79	0.67
OsR498G0305220900.01	0.56	1.36	1.4400735	3.64E-13	5.11E-13	0.52	0.62	0.54	1.17	1.48	1.43
OsR498G0305223500.01	2.593	5.223	1.0807619	1.30E-34	1.22E-34	2.15	2.75	2.88	4.51	5.8	5.36
OsR498G0305223800.01	0.41	2.266	2.3083107	7.75E-17	9.95E-17	0.74	0.33	0.16	2.45	2.26	2.09
OsR498G0305224700.01	46.94	178.19	1.9249457	0	0	46.42	50.06	44.34	181.37	178.27	174.92
OsR498G0305225000.01	87.626	383.57	2.1383891	0	0	83.91	85.28	93.69	376.27	368.1	406.35
OsR498G0305225100.01	0.063	0.6	3.0815719	1.57E-05	2.91E-05	0.06	0	0.13	0.6	0.47	0.73
OsR498G0305226500.01	41.553	103.86	1.3207196	0	0	41.32	41.6	41.74	102.18	104.98	104.42
OsR498G0305226900.01	4.14	8.356	1.0253649	2.20E-97	1.12E-97	4.32	4.28	3.82	8.7	8.84	7.53
OsR498G0305231800.01	0.55	1.413	1.336118	9.74E-16	1.28E-15	0.57	0.54	0.54	1.22	1.48	1.54
OsR498G0305231900.01	1.323	3.653	1.4922583	8.00E-15	1.08E-14	1.57	0.95	1.45	3.4	3.08	4.48
OsR498G0305237100.01	0.413	1.73	2.0564457	3.26E-23	3.68E-23	0.23	0.49	0.52	1.7	1.62	1.87
OsR498G0305239500.01	13.406	40.613	1.6349099	2.54E-248	6.32E-249	13.31	13.74	13.17	42.29	38.11	41.44
OsR498G0305239700.01	0.69	2.136	1.8819416	9.88E-15	1.33E-14	0.44	0.86	0.77	1.69	2.32	2.4

OsR498G0305241400.01	0.34	0.813	1.2444991	0.0008329	0.0017234	0.48	0.24	0.3	0.58	1.15	0.71
OsR498G0305243800.01	0.573	2.19	1.8879952	2.22E-17	2.81E-17	0.36	0.8	0.56	2.18	2.22	2.17
OsR498G0305245400.01	154.963	59.866	-1.39834	0	0	161.42	150.66	152.81	61.56	58.72	59.32
OsR498G0305245700.01	6.813	13.23	1.0019758	1.39E-73	8.47E-74	6.34	6.68	7.42	13.8	12.84	13.05
OsR498G0305245900.01	0.256	0.516	1.1503934	1.01E-06	1.76E-06	0.3	0.24	0.23	0.38	0.5	0.67
OsR498G0305247700.01	0.013	1.403	6.7817649	2.81E-27	2.97E-27	0.04	0	0	1.59	1.61	1.01
OsR498G0305248100.01	1.043	2.98	1.5120667	9.01E-21	1.07E-20	1.18	0.77	1.18	2.82	2.61	3.51
OsR498G0305248900.01	23.04	110.38	2.2600813	0	0	24.7	20.46	23.96	110.95	111.98	108.21
OsR498G0305252200.01	18.496	109.36	2.559966	0	0	19.13	18.56	17.8	109.39	109	109.7
OsR498G0305254100.01	1.153	10.33	3.1383857	1.93E-209	5.55E-210	1.19	1.1	1.17	10.94	9.8	10.25
OsR498G0305254600.01	108.083	273.08	1.3240546	0	0	103.95	113.19	107.11	276.37	274.34	268.54
OsR498G0305255700.01	5.27	2.063	-1.333019	1.01E-42	8.50E-43	4.97	5.1	5.74	2.03	1.93	2.23
OsR498G0305255900.01	2.226	4.576	1.0385403	4.49E-17	5.74E-17	1.87	2.49	2.32	4.6	4.23	4.9
OsR498G0305256500.01	9.166	26.22	1.5131226	5.90E-246	1.49E-246	9	9.43	9.07	26.11	25.82	26.73
OsR498G0305266900.01	0.676	0.013	-5.780478	1.17E-14	1.59E-14	0.55	0.91	0.57	0.04	0	0
OsR498G0305270500.01	237.99	80.8	-1.625946	0	0	242.96	237.61	233.4	82.48	83.42	76.5
OsR498G0305271700.01	0.763	2.313	1.8095991	2.77E-30	2.78E-30	0.53	0.72	1.04	1.79	2.33	2.82
OsR498G0305274100.01	1.1	0.1	-3.558085	1.38E-17	1.74E-17	1.27	1.01	1.02	0.04	0.13	0.13
OsR498G0305276500.01	11.2	24.316	1.1157975	5.99E-119	2.67E-119	11.72	11.03	10.85	24.62	25.09	23.24
OsR498G0305276900.01	0.23	0.093	-1.347518	0.0009086	0.0018872	0.2	0.21	0.28	0.06	0.1	0.12
OsR498G0305280600.01	0.106	0.43	1.9954716	6.50E-09	1.04E-08	0.1	0.09	0.13	0.39	0.5	0.4
OsR498G0305281500.01	0.29	0.136	-1.131508	0.0009524	0.0019817	0.23	0.39	0.25	0.14	0.15	0.12
OsR498G0305284600.01	0.07	3.856	5.7766032	6.84E-67	4.42E-67	0.08	0	0.13	3.99	3.48	4.1
OsR498G0305285000.01	0.32	0.853	1.3976214	2.11E-05	3.93E-05	0.26	0.48	0.22	0.82	0.9	0.84
OsR498G0305287900.01	7.756	17.653	1.1844446	1.46E-19	1.77E-19	9.53	6.36	7.38	17.52	17.29	18.15
OsR498G0305291100.01	0.02	0.143	2.7817649	0.0008272	0.0017105	0.06	0	0	0.06	0.15	0.22
OsR498G0305291500.01	5.606	29.81	2.4106417	3.23E-280	7.22E-281	6.24	5.61	4.97	29.39	29.3	30.74
OsR498G0305291800.01	0.366	1.48	1.9947851	4.61E-19	5.63E-19	0.32	0.41	0.37	1.51	1.4	1.53
OsR498G0305292600.01	0.496	1.016	1.0317431	0.0001132	0.0002203	0.33	0.72	0.44	0.99	1.29	0.77
OsR498G0305293000.01	0.073	0.306	2.0787466	1.97E-06	3.49E-06	0.07	0.11	0.04	0.26	0.4	0.26
OsR498G0305294500.01	1371.69	636.53	-1.108408	0	0	1333.1	1433.8	1348.2	635.11	649.44	625.05
OsR498G0305295300.01	0.133	0.486	1.7629059	4.92E-05	9.35E-05	0.22	0.05	0.13	0.46	0.41	0.59
OsR498G0305297000.01	10.323	21.603	1.0634664	4.17E-43	3.48E-43	11.63	9.14	10.2	18.98	21.1	24.73
OsR498G0305297400.01	1.38	3.63	1.3894475	1.06E-07	1.77E-07	1.04	1.61	1.49	3.49	3.14	4.26
OsR498G0305300200.01	0.38	0.053	-2.832945	2.58E-07	4.41E-07	0.48	0.33	0.33	0.13	0.03	0
OsR498G0305304300.01	30.75	97.39	1.630797	0	0	31.16	30.98	30.11	100.73	95.93	95.51
OsR498G0305306100.01	1.126	2.496	1.0562482	1.22E-23	1.37E-23	1.08	1.29	1.01	2.49	2.8	2.2
OsR498G0305310300.01	2.22	6.81	1.577254	2.27E-68	1.45E-68	2.25	2.15	2.26	6.53	6.83	7.07
OsR498G0305311800.01	23.29	11.31	-1.061932	1.12E-59	7.78E-60	23.69	23.48	22.7	10.89	10.79	12.25
OsR498G0305313900.01	8.213	1.65	-1.903998	4.10E-50	3.15E-50	7.38	8.17	9.09	1.06	2.33	1.56
OsR498G0305320100.01	13.286	31.11	1.2297926	2.43E-134	9.94E-135	13.48	13.47	12.91	33.68	31.24	28.41
OsR498G0305320300.01	119.85	325.64	1.4473407	0	0	116.9	120.33	122.32	322.27	326.71	327.93
OsR498G0305321100.01	52.376	209.48	1.99783	0	0	52.56	54.72	49.85	214.14	204.61	209.7
OsR498G0305321500.01	88.313	284.69	1.6878328	0	0	88.16	87.75	89.03	283.98	287.06	283.02
OsR498G0305321800.01	102.22	39.713	-1.330963	0	0	103.84	104.09	98.73	38.92	39.74	40.48
OsR498G0305323000.01	12.443	6.183	-1.092932	1.68E-36	1.53E-36	10.88	13.7	12.75	5.4	6.36	6.79
OsR498G0305323100.01	3.78	1.366	-1.469164	9.43E-20	1.14E-19	3.84	3.04	4.46	1.36	1.03	1.71
OsR498G0305324300.01	0.29	1.003	1.8134737	1.85E-16	2.39E-16	0.31	0.27	0.29	0.89	0.99	1.13
OsR498G0305326300.01	1.856	5.946	1.7139044	9.09E-50	7.00E-50	2.43	1.63	1.51	6.03	5.23	6.58
OsR498G0305326600.01	0	0.483	5.4979719	4.04E-07	6.96E-07	0	0	0	0.38	0.56	0.51
OsR498G0305327800.01	5.54	11.886	1.1013828	2.50E-60	1.72E-60	5.99	5.26	5.37	11.51	12.52	11.63
OsR498G0305328400.01	4.166	1.793	-1.211457	4.63E-11	6.91E-11	3.66	3.97	4.87	2.68	1.53	1.17
OsR498G0305329000.01	57.606	127.72	1.1484394	0	0	57.82	58.42	56.58	125.78	128.17	129.2
OsR498G0305330200.01	0.08	0.513	2.6748497	1.53E-05	2.83E-05	0.24	0	0	0.59	0.65	0.3
OsR498G0305330900.01	0.903	19.66	4.4426424	1.24E-253	2.99E-254	0.58	1.35	0.78	19.58	19.65	19.75
OsR498G0305331700.01	1.91	14.496	2.9204898	1.76E-115	8.02E-116	1.63	2.23	1.87	13.44	13.32	16.73
OsR498G0305333100.01	0.076	0.266	1.832391	0.0002304	0.0004563	0.06	0.06	0.11	0.33	0.16	0.31
OsR498G0305335100.01	0.403	0.13	-1.610553	0.000882	0.0018289	0.4	0.36	0.45	0.13	0.13	0.13
OsR498G0305337000.01	12.953	58.976	2.1840871	0	0	11.75	13.57	13.54	59.48	57.62	59.83
OsR498G0305337200.01	11.29	37.46	1.715076	1.45E-176	4.84E-177	11.69	11.66	10.52	38.29	37.91	36.18
OsR498G0305337400.01	0.29	3.906	3.7557697	1.87E-56	1.34E-56	0.34	0.23	0.3	3.86	3.85	4.01
OsR498G0305340600.01	1.493	6.893	2.205089	5.84E-36	5.37E-36	1.67	1.44	1.37	6.49	7.24	6.95
OsR498G0305343300.01	0.013	0.276	4.2963381	4.68E-06	8.46E-06	0.04	0	0	0.19	0.45	0.19
OsR498G0305348700.01	1.15	2.376	1.0533983	2.61E-21	3.06E-21	1.22	1.14	1.09	2.6	2.54	1.99
OsR498G0305350600.01	1.486	3.593	1.3583249	1.34E-14	1.82E-14	2.07	1.15	1.24	4.1	3.55	3.13
OsR498G0305351100.01	12.15	32.27	1.404154	2.05E-125	8.83E-126	11.86	12.65	11.94	34.04	32.8	29.97
OsR498G0305352100.01	1.823	4.26	1.2209562	5.01E-15	6.71E-15	2.5	1.04	1.93	4.17	4.1	4.51
OsR498G0305354100.01	3.326	9.62	1.4894704	7.61E-31	7.57E-31	2.29	4.01	3.68	8.82	9.47	10.57
OsR498G0305356500.01	31.316	229.99	2.852279	0	0	30.72	31.15	32.08	235.16	216.48	238.32

OsR498G0305356700.01	19.686	89.46	2.1811313	0	0	19.62	20.1	19.34	91.64	89.41	87.33
OsR498G0305358500.01	2.64	44.593	4.0944015	0	0	2.74	2.4	2.78	42.96	46.49	44.33
OsR498G0305361600.01	1.136	30.066	4.7171812	5.46E-188	1.72E-188	0.66	0.99	1.76	28.47	26.85	34.88
OsR498G0305362700.01	1.346	13.536	3.3284707	6.49E-207	1.89E-207	1.36	1.01	1.67	13.74	13.72	13.15
OsR498G0305363900.01	23.4	104.46	2.1760438	0	0	26.15	21.28	22.77	100.56	101.81	111.01
OsR498G0305364400.01	5.95	14.913	1.3580149	1.79E-69	1.13E-69	6	5.67	6.18	13.59	15.3	15.85
OsR498G0305366800.01	7.87	17.923	1.1867287	2.38E-114	1.09E-114	8.31	7.79	7.51	17.59	18.03	18.15
OsR498G0305367600.01	7.973	26.763	1.7646048	2.07E-224	5.61E-225	8.26	8.35	7.31	27.75	26.95	25.59
OsR498G0305368500.01	0.216	0.703	1.1088745	0.0004154	0.0008374	0.33	0.16	0.16	0.42	0.65	1.04
OsR498G0305369000.01	9.48	2.79	-1.766988	5.90E-60	4.07E-60	9.87	9.42	9.15	2.6	2.97	2.8
OsR498G0305369900.01	4.6	17.533	1.8681605	1.33E-89	7.19E-90	4.82	4.48	4.5	17.65	17.57	17.38
OsR498G0305371500.01	0.333	7.196	4.4248714	6.43E-107	3.08E-107	0.23	0.45	0.32	7.3	6.69	7.6
OsR498G0305371800.01	3.646	7.393	1.0335047	5.77E-25	6.32E-25	3.76	3.71	3.47	6.9	7.67	7.61
OsR498G0305375300.01	10.113	24.103	1.2521791	1.44E-177	4.76E-178	9.84	10.45	10.05	23.99	25.15	23.17
OsR498G0305375500.01	4.606	10.113	1.1230326	6.66E-25	7.31E-25	5.21	3.96	4.65	10.53	8.87	10.94
OsR498G0305379200.01	1.603	4.016	1.3258823	4.05E-23	4.58E-23	1.53	1.95	1.33	4.43	3.7	3.92
OsR498G0305379500.01	10.37	33.97	1.7119716	5.82E-137	2.34E-137	10.37	10.45	10.29	35.17	32.62	34.12
OsR498G0305379700.01	2.283	12.506	2.4537897	3.36E-84	1.90E-84	2.34	2.49	2.02	13.47	10.31	13.74
OsR498G0305380500.01	1.99	50.81	4.671242	0	0	2.03	1.85	2.09	47.55	48.52	56.36
OsR498G0305381400.01	1.936	3.926	1.0202136	6.17E-14	8.49E-14	2.01	2.03	1.77	4.46	2.96	4.36
OsR498G0305382000.01	14.12	30.726	1.1180904	4.02E-166	1.40E-166	13.54	14	14.82	29.22	32.69	30.27
OsR498G0305382100.01	1.95	5.85	1.5820925	1.51E-06	2.66E-06	2.78	2.52	0.55	6.29	5.45	5.81
OsR498G0305389700.01	26.053	150.99	2.5336094	0	0	27.73	24.33	26.1	149.01	152.01	151.95
OsR498G0305394200.01	61.676	148.38	1.2528118	2.19E-306	4.57E-307	63.25	64.13	57.65	151.25	139.33	154.55
OsR498G0305397100.01	5.206	137.94	4.7272833	0	0	5.26	5.31	5.05	139.9	137.17	136.76
OsR498G0305397500.01	1.56	53.433	5.1117946	0	0	1.5	1.5	1.68	53.86	53.81	52.63
OsR498G0305399100.01	2.246	6.62	1.5570016	2.58E-46	2.06E-46	2.59	2.14	2.01	6.23	6.79	6.84
OsR498G0305401000.01	0.176	0.536	1.6118399	0.0007971	0.0016457	0.18	0.23	0.12	0.68	0.34	0.59
OsR498G0305402000.01	0.12	1.516	3.4338416	5.67E-10	8.77E-10	0.09	0.18	0.09	1.31	1.99	1.25
OsR498G0305402300.01	4.983	10.583	1.1060068	6.41E-44	5.28E-44	4.67	5.18	5.1	10.55	10.09	11.11
OsR498G0305408900.01	0.063	1.22	4.2781907	7.73E-19	9.49E-19	0	0.14	0.05	1.29	1.33	1.04
OsR498G0305409500.01	0.27	3.643	3.757786	2.01E-37	1.81E-37	0.19	0.31	0.31	3.27	3.93	3.73
OsR498G0305410500.01	22.16	109.76	2.3051323	0	0	20.86	23.16	22.46	111.17	110.8	107.31
OsR498G0305411900.01	1.603	7.943	2.3096964	1.20E-55	8.68E-56	2.39	1.21	1.21	7.4	8.52	7.91
OsR498G0305413000.01	4.16	12.366	1.565456	4.36E-76	2.62E-76	3.98	4.56	3.94	11.97	12.65	12.48
OsR498G0305413100.01	0.653	2.83	2.1332894	1.30E-27	1.36E-27	0.85	0.61	0.5	2.57	3.19	2.73
OsR498G0305415900.01	0.706	3.163	1.9929459	4.50E-26	4.84E-26	0.81	0.64	0.67	3.31	2.9	3.28
OsR498G0305416600.01	2.103	5.523	1.3845981	3.54E-26	3.81E-26	1.75	2.09	2.47	5.05	5.31	6.21
OsR498G0305416700.01	0.343	1.686	2.2333861	1.50E-52	1.12E-52	0.24	0.42	0.37	1.65	1.75	1.66
OsR498G0305418900.01	0.106	0.346	1.6849033	1.12E-07	1.89E-07	0.15	0.1	0.07	0.3	0.37	0.37
OsR498G0305424000.01	5.64	12.46	1.1375554	1.26E-97	6.44E-98	5.5	5.5	5.92	12.35	11.96	13.07
OsR498G0305424200.01	10.17	34.95	1.786824	3.84E-154	1.42E-154	9.25	10.58	10.68	32.39	37.09	35.37
OsR498G0305425700.01	2.34	7.113	1.6079657	1.08E-148	4.08E-149	2.45	2.38	2.19	7.01	7.88	6.45
OsR498G0305425900.01	5.806	11.786	1.0374791	2.19E-29	2.23E-29	5	5.81	6.61	11.89	12.11	11.36
OsR498G0305430600.01	50.81	16.706	-1.606745	0	0	52.53	47.66	52.24	17.05	15.58	17.49
OsR498G0305431800.01	1.253	5.75	2.1907279	2.07E-29	2.10E-29	1.33	1.25	1.18	5.02	6.17	6.06
OsR498G0305432300.01	0.896	6.543	2.8657811	2.19E-96	1.13E-96	1.17	0.96	0.56	6.71	6.32	6.6
OsR498G0305434900.01	0.04	0.216	2.5593725	4.55E-05	8.61E-05	0.03	0.06	0.03	0.35	0.08	0.22
OsR498G0305437700.01	15.21	31.176	1.0120975	2.07E-68	1.32E-68	14.36	16.44	14.83	31.99	32.28	29.26
OsR498G0305438000.01	4.133	0.406	-3.309044	1.89E-103	9.30E-104	3.98	3.81	4.61	0.48	0.34	0.4
OsR498G0305438500.01	11.226	31.256	1.4610844	4.98E-248	1.24E-248	11.22	10.86	11.6	31.94	31.9	29.93
OsR498G0305440700.01	3.513	20.06	2.3756339	1.04E-152	3.83E-153	3.74	3.48	3.32	21.11	18.43	20.64
OsR498G0305441000.01	2.753	7.833	1.5071543	1.23E-42	1.03E-42	3.08	2.36	2.82	7.58	7.86	8.06
OsR498G0305441300.01	21.693	70.866	1.6656445	0	0	23.71	20.84	20.53	72.6	69.33	70.67
OsR498G0305445400.01	0.913	2.836	1.6490097	1.21E-21	1.41E-21	1.02	0.84	0.88	3.04	2.86	2.61
OsR498G0305446300.01	3.143	19.456	2.6226984	1.14E-188	3.58E-189	4.02	2.63	2.78	21.13	18.52	18.72
OsR498G0305447400.01	1.156	0.39	-1.754044	9.28E-17	1.19E-16	1.17	1.14	1.16	0.25	0.33	0.59
OsR498G0305448900.01	0.55	1.54	1.3804023	2.90E-08	4.77E-08	0.62	0.54	0.49	1.59	1.49	1.54
OsR498G0305450400.01	0.356	0.88	1.2870002	5.72E-06	1.04E-05	0.32	0.27	0.48	1.02	0.77	0.85
OsR498G0305452300.01	25.646	68.35	1.4182592	0	0	25.53	26.8	24.61	67.96	67.96	69.13
OsR498G0305452800.01	4.01	10.903	1.4523456	2.37E-77	1.41E-77	4.18	3.68	4.17	11.3	10.66	10.75
OsR498G0305456700.01	1.323	3.643	1.4309241	9.71E-34	9.22E-34	1.17	1.54	1.26	3.7	3.75	3.48
OsR498G0305457100.01	7.833	17.06	1.1253645	2.13E-76	1.27E-76	8.41	7.61	7.48	16.21	18.76	16.21
OsR498G0305457500.01	0.016	0.273	3.5593725	0.0005724	0.0011661	0	0.05	0	0.48	0.1	0.24
OsR498G0305460300.01	8.11	16.626	1.0382611	1.88E-111	8.77E-112	7.78	8.54	8.01	16.95	16.84	16.09
OsR498G0305463800.01	157.693	387.33	1.2929526	0	0	161.02	153.15	158.91	379.74	379.69	402.57
OsR498G0305466600.01	163.8	66.63	-1.301445	0	0	156.4	173.55	161.45	65.02	65.22	69.65
OsR498G0305467400.01	7.37	22.91	1.6305056	2.21E-206	6.44E-207	7.84	7.41	6.86	21.79	23.46	23.48
OsR498G0305468600.01	6.836	3.08	-1.153494	1.20E-35	1.11E-35	6.8	6.72	6.99	3.08	3.3	2.86

OsR498G0305472100.01	0.47	2.676	2.4524573	8.31E-28	8.69E-28	0.26	0.66	0.49	3.23	2.09	2.71
OsR498G0305476500.01	0.46	0.076	-3.247982	8.24E-07	1.44E-06	0.55	0.39	0.44	0.18	0.05	0
OsR498G0305477500.01	0.353	0.143	-1.318372	0.0001529	0.0002998	0.52	0.19	0.35	0.17	0.17	0.09
OsR498G0305483100.01	13.98	27.966	1.0259067	8.02E-151	2.99E-151	13.67	14.19	14.08	28.74	28.98	26.18
OsR498G0305483400.01	4.346	14.733	1.7650235	2.89E-256	6.94E-257	4.61	3.88	4.55	14.31	14.47	15.42
OsR498G0305484900.01	15.68	98.526	2.6363998	0	0	14.89	16.24	15.91	99.94	97.29	98.35
OsR498G0305485800.01	3.156	9.563	1.5977863	5.64E-51	4.29E-51	3.13	3.05	3.29	9.98	8.45	10.26
OsR498G0305487700.01	1.44	3.06	1.0894266	7.71E-24	8.61E-24	1.8	1.27	1.25	2.83	3.52	2.83
OsR498G0305487900.01	8.966	20.263	1.1777327	2.14E-140	8.43E-141	8.92	9	8.98	20.74	21.13	18.92
OsR498G0305488100.01	0.553	2.976	2.4299789	6.08E-51	4.63E-51	0.59	0.53	0.54	2.78	3.41	2.74
OsR498G0305488900.01	0	0.926	7.6037666	4.25E-23	4.81E-23	0	0	0	0.81	1.08	0.89
OsR498G0305489600.01	0.106	0.743	2.7817649	5.07E-06	9.19E-06	0.16	0	0.16	0.95	0.63	0.65
OsR498G0305489900.01	0.666	0.08	-3.02559	1.22E-12	1.74E-12	0.88	0.62	0.5	0.15	0.03	0.06
OsR498G0305490500.01	212.203	62.486	-1.765875	0	0	208.19	215.25	213.17	62.35	63.12	61.99
OsR498G0305491400.01	4.37	9.703	1.1481166	4.04E-28	4.20E-28	4.67	4.42	4.02	9.67	8.98	10.46
OsR498G0305496100.01	0.286	1.106	1.97441	8.12E-13	1.15E-12	0.3	0.12	0.44	0.96	1.13	1.23
OsR498G0305501000.01	0.066	0	-4.832945	6.10E-05	0.0001167	0.07	0.04	0.09	0	0	0
OsR498G0305505200.01	4.613	1.46	-1.659462	2.94E-53	2.18E-53	4.77	4.74	4.33	1.56	1.32	1.5
OsR498G0305506100.01	0.48	1.7	1.8176845	2.51E-07	4.29E-07	0.51	0.76	0.17	1.08	1.98	2.04
OsR498G0305507900.01	3.823	11.643	1.6051761	7.45E-90	4.03E-90	4	3.74	3.73	12.09	10.9	11.94
OsR498G0305520600.01	21.936	6.693	-1.714359	1.96E-251	4.80E-252	21.44	21.17	23.2	6.49	6.16	7.43
OsR498G0305527400.01	59.516	155.02	1.3951377	0	0	59.93	57.56	61.06	155.5	149.54	160.02
OsR498G0305528700.01	21.76	47.523	1.1383585	4.21E-135	1.71E-135	22.73	21.72	20.83	46.83	48.62	47.12
OsR498G0305530900.01	0.586	11.72	4.2592056	1.33E-256	3.19E-257	0.44	0.63	0.69	11.81	11.38	11.97
OsR498G0305533900.01	1.543	7.076	2.1782823	4.04E-91	2.17E-91	1.6	1.68	1.35	7.31	7.04	6.88
OsR498G0305538800.01	1.256	3.203	1.3511305	2.44E-11	3.61E-11	1.53	1.27	0.97	3.32	3.01	3.28
OsR498G0305541500.01	27.243	227.46	3.0615283	0	0	27.64	27.55	26.54	228.88	230.61	222.88
OsR498G0305541800.01	0.643	2.04	1.6606142	5.24E-16	6.86E-16	0.76	0.71	0.46	1.98	2.11	2.03
OsR498G0305542900.01	11.643	34.17	1.552322	0	0	11.89	11.93	11.11	34.12	34.9	33.49
OsR498G0305546400.01	0.096	0.55	2.5289988	2.92E-08	4.80E-08	0.11	0.11	0.07	0.7	0.52	0.43
OsR498G0305546600.01	23.31	75.173	1.6876427	2.45E-257	5.86E-258	24.85	23.51	21.57	74.87	75.67	74.98
OsR498G0305546700.01	5.413	12.686	1.2279742	2.78E-31	2.74E-31	4.85	5.14	6.25	12.56	13.08	12.42
OsR498G0305547700.01	8.263	42.143	2.344126	2.71E-253	6.59E-254	7.81	8.9	8.08	42.72	40.99	42.72
OsR498G0305548400.01	24.3	57.193	1.3168981	0	0	23.22	23.67	26.01	56.73	57.82	57.03
OsR498G0305550600.01	20.543	53.626	1.3836461	3.27E-298	6.95E-299	19.87	19.55	22.21	53.67	52.75	54.46
OsR498G0305553600.01	5.153	10.323	1.0203758	1.42E-23	1.59E-23	5.21	5.48	4.77	9.95	11.49	9.53
OsR498G0305556500.01	120.04	59.47	-1.013204	2.27E-244	5.74E-245	123.37	116.89	119.86	62.15	59.41	56.85
OsR498G0305559200.01	15.873	36.206	1.1877002	9.50E-109	4.50E-109	16.16	16.25	15.21	37.16	33.22	38.24
OsR498G0305559500.01	9.173	20.683	1.1154001	6.45E-78	3.83E-78	9	9.9	8.62	19.46	22.06	20.53
OsR498G0305565900.01	0.163	0.073	-1.52809	0.0003791	0.0007623	0.21	0.22	0.06	0.1	0.03	0.09
OsR498G0305570600.01	0.226	2.086	3.2135393	1.08E-83	6.13E-84	0.38	0	0.3	2.28	1.93	2.05
OsR498G0305571800.01	1.283	7.54	2.5561186	8.12E-237	2.11E-237	1.26	1.38	1.21	6.75	8.59	7.28
OsR498G0305572700.01	2.373	0.67	-1.762556	1.27E-19	1.54E-19	2.3	2.43	2.39	0.69	0.72	0.6
OsR498G0305573700.01	1.363	10.42	2.9412431	2.27E-168	7.83E-169	1.59	1.39	1.11	9.88	10.69	10.69
OsR498G0305574600.01	20.323	43.143	1.093837	0	0	21.14	20.53	19.3	44.2	43.46	41.77
OsR498G0305575000.01	5.176	21.92	2.080391	1.80E-221	4.92E-222	5.06	5.86	4.61	20.45	22.34	22.97
OsR498G0305575800.01	0.08	0.433	2.4149825	9.99E-07	1.75E-06	0.14	0.07	0.03	0.54	0.24	0.52
OsR498G0305576600.01	11.086	37.626	1.7648378	0	0	11.36	10.25	11.65	38.46	37.59	36.83
OsR498G0305578100.01	3.263	6.76	1.0566676	1.78E-18	2.20E-18	3.6	3.47	2.72	6.64	6.77	6.87
OsR498G0305578300.01	1.61	10.363	2.6681081	3.20E-64	2.13E-64	1.34	1.79	1.7	9.25	10.57	11.27
OsR498G0305578700.01	2.153	5.206	1.2416938	4.34E-30	4.37E-30	2.09	2.19	2.18	5.37	5.03	5.22
OsR498G0305579400.01	0.24	0.013	-4.195515	1.24E-05	2.28E-05	0.28	0.28	0.16	0	0.04	0
OsR498G0305580000.01	112.473	229.86	1.0356035	0	0	110.44	112.65	114.33	224.24	226.62	238.72
OsR498G0305581000.01	26.34	51.95	1.0234017	1.21E-208	3.49E-209	27.35	25.14	26.53	50.91	51.41	53.53
OsR498G0305586800.01	36.42	1.703	-4.476702	0	0	35.17	38.21	35.88	2.05	1.44	1.62
OsR498G0305587100.01	19.07	87.08	2.2045669	0	0	19.01	19.38	18.82	87.74	89.38	84.12
OsR498G0305593200.01	9.343	23.773	1.3859483	2.46E-128	1.04E-128	10.01	9.18	8.84	24.6	23.08	23.64
OsR498G0305597100.01	1.326	2.933	1.144335	1.30E-16	1.68E-16	1.28	1.29	1.41	2.99	2.49	3.32
OsR498G0305598600.01	18.756	57.13	1.6212563	0	0	17.22	18.91	20.14	56.16	56.59	58.64
OsR498G0305600000.01	6.876	30.463	2.1681964	0	0	7.32	6.76	6.55	31	30.96	29.43
OsR498G0305600400.01	5.533	17.943	1.6917153	8.07E-172	2.73E-172	4.78	5.58	6.24	16.65	18.38	18.8
OsR498G0305602400.01	2.693	12.41	2.1986251	1.79E-63	1.20E-63	1.8	2.78	3.5	11.18	11.35	14.7
OsR498G0305607600.01	0.39	0.826	1.0840344	3.81E-05	7.20E-05	0.28	0.4	0.49	0.63	1.05	0.8
OsR498G0305612100.01	0.033	0.243	2.7817649	0.0008272	0.0017105	0	0.05	0.05	0.26	0.15	0.32
OsR498G0305615900.01	21.28	1.383	-4.122163	4.52E-240	1.16E-240	24.56	20.44	18.84	0.61	1.24	2.3
OsR498G0305619800.01	1.026	3.25	1.6664603	1.05E-53	7.74E-54	0.87	1.23	0.98	3.19	3.55	3.01
OsR498G0305619900.01	0	0.166	5.2223375	3.89E-06	7.00E-06	0	0	0	0.14	0.21	0.15
OsR498G0305621800.01	31.823	64.686	1.0293524	1.04E-113	4.81E-114	29.96	32.54	32.97	62.69	64.5	66.87
OsR498G0305622000.01	7.52	17.39	1.2711773	1.09E-164	3.86E-165	7.83	7.36	7.37	17.65	17.69	16.83

OsR498G0305623100.01	12.95	31.42	1.2555133	1.95E-198	5.87E-199	12.81	13.39	12.65	32.38	30.88	31
OsR498G0305625900.01	11.526	5.393	-1.364051	6.27E-93	3.32E-93	12.25	11.51	10.82	5.56	5.56	5.06
OsR498G0305626500.01	47.963	203.4	2.0826172	0	0	47.6	50.1	46.19	199.35	198.35	212.5
OsR498G0305627100.01	31.06	6.5	-2.283201	0	0	31.02	31.56	30.6	6.64	6.46	6.4
OsR498G0305627400.01	4.483	10.006	1.0425815	5.61E-35	5.22E-35	4.42	4.53	4.5	9.61	10.46	9.95
OsR498G0305629300.01	5.57	19.533	1.8091954	2.31E-251	5.65E-252	5.56	5.27	5.88	19.09	19.41	20.1
OsR498G0305629600.01	3.936	10.89	1.4667335	1.18E-91	6.32E-92	3.61	4.09	4.11	11.31	10.7	10.66
OsR498G0305631400.01	0.176	0.83	2.23537	2.05E-18	2.54E-18	0.2	0.13	0.2	0.85	0.75	0.89
OsR498G0305632600.01	0.906	0.246	-1.872626	2.88E-21	3.38E-21	0.97	0.79	0.96	0.28	0.21	0.25
OsR498G0305632700.01	48.886	23.443	-1.08593	4.80E-198	1.45E-198	49.5	47.81	49.35	23.69	23.23	23.41
OsR498G0305633000.01	3.7	7.823	1.0807189	4.67E-17	5.96E-17	4.33	3.58	3.19	7.07	8.75	7.65
OsR498G0305633900.01	18.106	39.823	1.1341954	1.33E-119	5.88E-120	18.02	17.56	18.74	39.34	36.75	43.38
OsR498G0305638300.01	0.916	0.353	-1.382393	0.000439	0.0008868	0.96	0.48	1.31	0.35	0.07	0.64
OsR498G0305641600.01	13.213	5.953	-1.151818	1.80E-116	8.15E-117	13.95	13.35	12.34	6.35	5.86	5.65
OsR498G0305642700.01	1.29	3.67	1.5008464	1.28E-23	1.44E-23	1.44	1.13	1.3	4.53	3.68	2.8
OsR498G0305645600.01	2.853	9.293	1.6903651	3.81E-69	2.42E-69	3.01	2.63	2.92	9.44	9.19	9.25
OsR498G0305651500.01	0.06	0.503	3.103693	9.95E-08	1.67E-07	0.09	0.09	0	0.64	0.34	0.53
OsR498G0305652000.01	1.743	3.586	1.0439509	1.82E-08	2.98E-08	2.59	1.09	1.55	4.15	3.38	3.23
OsR498G0305652300.01	3.82	16.343	2.0825037	4.64E-207	1.35E-207	4.19	3.44	3.83	16.78	17	15.25
OsR498G0305653700.01	1.233	2.613	1.0772584	7.03E-20	8.45E-20	1.01	1.33	1.36	2.28	2.75	2.81
OsR498G0305653900.01	0.01	0.173	3.9496066	2.81E-05	5.27E-05	0.03	0	0	0.09	0.27	0.16
OsR498G0305654200.01	0.87	1.716	1.1489863	1.94E-11	2.87E-11	0.76	0.99	0.86	1.85	1.83	1.47
OsR498G0305655100.01	4.956	10.88	1.1368496	1.05E-38	9.26E-39	5.01	4.65	5.21	10.81	10.92	10.91
OsR498G0305659100.01	9.516	19.12	1.030553	2.63E-67	1.69E-67	9.16	10.12	9.27	18.44	19.89	19.03
OsR498G0305660600.01	42.28	14.206	-1.586305	1.80E-42	1.51E-42	41.15	43.34	42.35	9.49	16.75	16.38
OsR498G0305661600.01	0.123	0	-4.347518	0.0007014	0.0014405	0.16	0.13	0.08	0	0	0
OsR498G0305665500.01	39.14	97.02	1.3067018	0	0	38.35	38.76	40.31	96.62	99.62	94.82
OsR498G0305665700.01	0.19	0.986	2.2963381	8.68E-05	0.0001676	0	0.35	0.22	0.84	1.3	0.82
OsR498G0305667700.01	67.21	27.536	-1.287053	0	0	68.46	68.07	65.1	28.33	27.6	26.68
OsR498G0305669400.01	8.8	28.23	1.6805381	7.79E-248	1.94E-248	9.27	8.54	8.59	30.02	26.87	27.8
OsR498G0305672500.01	8.036	21.483	1.3419463	5.51E-155	2.02E-155	8.15	8.29	7.67	22.04	21.31	21.1
OsR498G0305678500.01	194.37	512.26	1.3986879	0	0	190.1	194.84	198.17	509.76	506.9	520.13
OsR498G0305678800.01	0.316	1.143	1.8633548	5.87E-06	1.06E-05	0.38	0.38	0.19	1.58	1.28	0.57
OsR498G0305679000.01	1.713	3.57	1.0410077	3.19E-12	4.60E-12	1.71	1.6	1.83	3.72	3.7	3.29
OsR498G0305679100.01	1.046	5.176	2.3053268	8.96E-22	1.04E-21	1.28	0.88	0.98	5.27	5.54	4.72
OsR498G0305682600.01	0	0.183	4.4338416	0.0004331	0.0008746	0	0	0	0.3	0.2	0.05
OsR498G0305684300.01	14.853	78.49	2.3909011	0	0	14.32	16.01	14.23	79.89	78.38	77.2
OsR498G0305684900.01	0.256	0.686	1.4223256	1.49E-11	2.19E-11	0.31	0.22	0.24	0.86	0.71	0.49
OsR498G0305687200.01	1040.32	466.56	-1.160321	0	0	1034.8	1009.8	1076.4	459.01	444.21	496.46
OsR498G0305688000.01	44.716	9.2	-2.282057	1.55E-199	4.64E-200	47.17	41.21	45.77	9.3	9.02	9.28
OsR498G0305690800.01	0.023	0.606	4.7292975	7.57E-08	1.26E-07	0	0.07	0	0.81	0.6	0.41
OsR498G0305692900.01	0.16	0.506	1.7488503	2.20E-07	3.75E-07	0.16	0.22	0.1	0.28	0.64	0.6
OsR498G0305693600.01	17.793	50.093	1.4937084	0	0	18.18	17.84	17.36	50.25	52.11	47.92
OsR498G0305694300.01	3.146	0.223	-3.832945	1.46E-30	1.45E-30	2.51	3.16	3.77	0.07	0.33	0.27
OsR498G0305706500.01	3.366	8.993	1.3996367	2.23E-34	2.10E-34	3.21	3.59	3.3	9.46	8.76	8.76
OsR498G0305706800.01	0.203	158.41	9.9210913	0	0	0.23	0.2	0.18	159.44	157.74	158.05
OsR498G0305707200.01	90.81	508.52	2.5121704	0	0	89.28	88.7	94.45	500.67	503.07	521.81
OsR498G0305707600.01	2.143	4.7	1.1311355	5.30E-14	7.27E-14	1.7	2.68	2.05	4.83	3.99	5.28
OsR498G0305709600.01	0.3	0.836	1.4937841	1.08E-07	1.81E-07	0.39	0.3	0.21	0.72	0.75	1.04
OsR498G0305709700.01	1.32	5.843	2.3111858	3.26E-38	2.90E-38	1.96	1.21	0.79	5.88	6.1	5.55
OsR498G0305711600.01	37.833	78.373	1.0609234	1.69E-292	3.65E-293	38.89	37.83	36.78	80.03	79.2	75.89
OsR498G0305713000.01	1.413	7.54	2.4365894	4.27E-262	1.00E-262	1.44	1.49	1.31	7.1	7.38	8.14
OsR498G0305714300.01	0.163	6.383	5.2999403	1.19E-94	6.23E-95	0.15	0.19	0.15	6.59	6.42	6.14
OsR498G0305715700.01	85.883	233.37	1.4388967	0	0	85.2	87.27	85.18	230.64	233.24	236.23
OsR498G0305716200.01	1.983	21.893	3.4432352	0	0	2.11	1.99	1.85	21.21	22.31	22.16
OsR498G0305718500.01	20.503	59.92	1.562571	0	0	19.12	20.74	21.65	60.63	60.6	58.53
OsR498G0305721200.01	0.403	34.156	6.3978761	3.75E-239	9.68E-240	0.2	0.4	0.61	34.08	32.27	36.12
OsR498G0305721600.01	49.646	176.83	1.8323506	0	0	51.44	50.47	47.03	181.85	177.54	171.11
OsR498G0305723500.01	0.983	0.483	-1.043292	2.46E-08	4.02E-08	1.22	0.88	0.85	0.38	0.46	0.61
OsR498G0305724100.01	0.39	0.086	-2.02559	0.0002922	0.0005834	0.49	0.39	0.29	0.21	0	0.05
OsR498G0305724400.01	13.493	28.656	1.0724212	3.25E-46	2.61E-46	11.8	14.02	14.66	31.05	27.56	27.36
OsR498G0305724500.01	1.016	110.88	6.7674922	0	0	1.02	1.25	0.78	112.87	108.07	111.7
OsR498G0305726100.01	18.333	38.29	1.0618326	5.70E-183	1.84E-183	18.89	18.88	17.23	39.68	38.29	36.9
OsR498G0305726500.01	0.556	3.88	2.7684065	1.58E-34	1.48E-34	0.77	0.66	0.24	4.23	3.77	3.64
OsR498G0305727200.01	8.66	27.55	1.6686822	6.84E-148	2.59E-148	8.83	7.91	9.24	26.74	27.08	28.83
OsR498G0305727500.01	1.626	4.606	1.4970164	6.31E-41	5.42E-41	1.57	1.85	1.46	4.82	4.34	4.66
OsR498G0305728500.01	23.61	251.82	3.4169157	0	0	23.48	24.36	22.99	254.83	258.55	242.09
OsR498G0305728900.01	8.45	16.96	1.0024243	1.07E-47	8.42E-48	8.02	8.81	8.52	16.66	15.66	18.56
OsR498G0305729500.01	0.133	0.303	1.5149783	0.0006351	0.0012984	0.17	0.06	0.17	0.34	0.24	0.33

OsR498G0305731000.01	0.01	0.616	6.103693	4.02E-18	5.00E-18	0	0	0.03	0.6	0.62	0.63
OsR498G0305736400.01	0	0.39	6.9286063	8.50E-16	1.12E-15	0	0	0	0.42	0.33	0.42
OsR498G0305739300.01	14.7	47.913	1.7039786	0	0	14.31	15.5	14.29	45.81	49.25	48.68
OsR498G0305741500.01	17.486	6.963	-1.33051	1.17E-78	6.87E-79	16.31	17.02	19.13	6.81	6.63	7.45
OsR498G0305741800.01	16.416	35.01	1.0934926	1.78E-89	9.67E-90	17.31	17.01	14.93	33.99	35.45	35.59
OsR498G0305742000.01	0.016	0.14	3.2963381	2.59E-05	4.84E-05	0.02	0.03	0	0.25	0.09	0.08
OsR498G0305746100.01	0.563	0.243	-1.220511	0.0002382	0.0004725	0.53	0.42	0.74	0.26	0.22	0.25
OsR498G0305747000.01	0.283	0.67	1.249417	4.02E-08	6.63E-08	0.36	0.27	0.22	0.51	0.87	0.63
OsR498G0305747600.01	13.98	5.66	-1.299024	7.48E-84	4.24E-84	14.71	13.21	14.02	5.74	5.8	5.44
OsR498G0305749200.01	1.483	5.446	1.8954217	2.22E-46	1.78E-46	1.91	1.68	0.86	5.35	6.07	4.92
OsR498G0305749600.01	0.866	0.373	-1.224899	3.41E-08	5.61E-08	0.93	0.83	0.84	0.33	0.31	0.48
OsR498G0305752600.01	4.29	9.576	1.1621947	1.34E-25	1.46E-25	3.63	5.32	3.92	9.3	10.82	8.61
OsR498G0305753100.01	0.083	0.383	2.167055	2.13E-05	3.97E-05	0.07	0.18	0	0.36	0.46	0.33
OsR498G0305753300.01	0.24	1.18	2.2963381	4.69E-06	8.47E-06	0.21	0.31	0.2	0.6	1.5	1.44
OsR498G0305753500.01	3.626	8.186	1.3431323	3.57E-43	2.98E-43	3.46	4.28	3.14	7.72	8.23	8.61
OsR498G0305754100.01	20.166	49.6	1.2974515	6.22E-130	2.61E-130	20.04	21.75	18.71	50.54	46.64	51.62
OsR498G0305754500.01	15.336	35.58	1.2136822	8.09E-96	4.19E-96	16.8	15.67	13.54	37.42	34.14	35.18
OsR498G0305756400.01	7.263	1.213	-2.585678	6.13E-68	3.93E-68	7.48	7.21	7.1	0.88	1.15	1.61
OsR498G0305761900.01	0.39	5.096	3.700235	2.02E-69	1.27E-69	0.45	0.36	0.36	4.81	5.18	5.3
OsR498G0305766000.01	1.16	2.316	1.0004052	5.23E-10	8.08E-10	1.18	1.13	1.17	2.81	2.43	1.71
OsR498G0305766400.01	0.373	1.476	2.0033613	1.64E-17	2.08E-17	0.26	0.45	0.41	1.19	1.28	1.96
OsR498G0305766500.01	0.75	2.066	1.473981	1.91E-18	2.37E-18	0.77	1	0.48	1.96	2.15	2.09
OsR498G0305767800.01	2.533	24.686	3.2839188	9.71E-250	2.40E-250	2.51	3.31	1.78	24.28	25.59	24.19
OsR498G0305769300.01	0.33	1.106	1.72777	7.75E-12	1.13E-11	0.37	0.28	0.34	1.14	0.86	1.32
OsR498G0305770700.01	0.173	1.54	3.1588345	2.04E-36	1.86E-36	0.14	0.26	0.12	1.81	1.39	1.42
OsR498G0305773700.01	18.463	59.06	1.6830395	0	0	19.04	18.26	18.09	58.16	59.95	59.07
OsR498G0305774300.01	4.3	8.97	1.0605107	7.57E-18	9.48E-18	4.85	3.71	4.34	8.75	9.25	8.91
OsR498G0305774900.01	0.106	0.563	2.3894475	7.16E-06	1.30E-05	0.05	0.11	0.16	0.58	0.57	0.54
OsR498G0305780400.01	0.723	0.28	-1.422926	7.93E-09	1.28E-08	0.69	0.82	0.66	0.28	0.29	0.27
OsR498G0305782200.01	8.13	1.23	-2.709406	1.65E-47	1.30E-47	7.64	9.22	7.53	0.98	1.53	1.18
OsR498G0305784900.01	0.253	1.993	2.9882158	4.47E-19	5.46E-19	0.12	0.29	0.35	1.98	2.14	1.86
OsR498G0305786100.01	1.916	4.676	1.2852586	1.01E-26	1.07E-26	1.8	2.38	1.57	4.56	4.36	5.11
OsR498G0305786300.01	0.553	1.533	1.4289758	3.93E-06	7.07E-06	0.49	0.7	0.47	1.84	1.4	1.36
OsR498G0305786700.01	2.243	5.386	1.2617336	2.76E-64	1.83E-64	2.31	2.28	2.14	5.29	5.16	5.71
OsR498G0305788600.01	0.016	0.1	2.637375	0.0001939	0.0003825	0.02	0	0.03	0.11	0.09	0.1
OsR498G0305790400.01	54.346	123.64	1.1836892	1.71E-275	3.87E-276	54.42	56.13	52.49	121.65	118.76	130.5
OsR498G0305790900.01	17.916	59.276	1.721671	0	0	17.2	18.36	18.19	59.84	55.39	62.6
OsR498G0305792900.01	0.52	1.556	1.503769	4.59E-11	6.86E-11	0.67	0.4	0.49	1.47	1.68	1.52
OsR498G0305794200.01	6.756	19.783	1.5333569	3.51E-92	1.87E-92	6.17	7.57	6.53	20.07	19.38	19.9
OsR498G0305794600.01	4.783	9.963	1.0528087	2.29E-82	1.31E-82	4.67	5.26	4.42	10.12	10.16	9.61
OsR498G0305794900.01	4.99	10.516	1.0732701	1.70E-64	1.13E-64	5.16	5.35	4.46	11.36	9.79	10.4
OsR498G0305796000.01	0.296	1.34	2.1593788	2.23E-29	2.27E-29	0.22	0.2	0.47	1.4	1.38	1.24
OsR498G0305796200.01	7.05	29.96	2.0876321	3.21E-281	7.16E-282	6.79	7.47	6.89	30.14	31.29	28.45
OsR498G0305801100.01	7.736	16.11	1.0537275	6.68E-104	3.26E-104	7.6	7.45	8.16	17.66	15.57	15.1
OsR498G0305801300.01	0.096	3.36	5.1393169	6.76E-68	4.33E-68	0.07	0.11	0.11	2.99	3.4	3.69
OsR498G0305804100.01	200.15	81.186	-1.344029	0	0	203.22	199.26	197.97	81.87	82.46	79.23
OsR498G0305804600.01	3.96	0.72	-2.454292	1.46E-113	6.74E-114	3.96	4.43	3.49	0.7	0.85	0.61
OsR498G0305805500.01	0.076	0.526	2.7817649	5.07E-06	9.19E-06	0.17	0	0.06	0.34	0.67	0.57
OsR498G0305809900.01	9.703	20.973	1.0946696	9.71E-79	5.72E-79	11.2	9.93	7.98	19.91	21.88	21.13
OsR498G0305815000.01	10.13	36.996	1.8713518	0	0	10.48	10.19	9.72	37.6	37.96	35.43
OsR498G0305816700.01	72.266	32.6	-1.155928	4.85E-191	1.51E-191	71.56	73.22	72.02	31.3	31.93	34.57
OsR498G0305817600.01	17.43	63.67	1.8704489	0	0	17.55	17.61	17.13	63.76	66.01	61.24
OsR498G0305824900.01	10.87	61.936	2.5590925	0	0	9.19	11.03	12.39	61.49	63.01	61.31
OsR498G0305825700.01	0.85	2.34	1.4798745	2.05E-29	2.08E-29	0.56	0.95	1.04	2.53	2.23	2.26
OsR498G0305826500.01	0.066	0.596	3.1760438	8.63E-10	1.34E-09	0.04	0.12	0.04	0.62	0.46	0.71
OsR498G0305829400.01	2.543	14.296	2.5026034	8.47E-246	2.13E-246	2.48	2.43	2.72	15.49	14.83	12.57
OsR498G0305829600.01	29.03	81.83	1.4813312	0	0	28.77	30.11	28.21	83.39	84.2	77.9
OsR498G0305830700.01	2.333	88.616	5.246262	0	0	2.42	1.89	2.69	85.78	85.42	94.65
OsR498G0305832300.01	285.016	612.05	1.1022055	0	0	277.54	289.16	288.35	598.21	613.67	624.28
OsR498G0305839500.01	4.64	1.706	-1.468824	4.92E-23	5.57E-23	3.68	4.75	5.49	1.72	2.02	1.38
OsR498G0305839700.01	8.873	21.073	1.246719	9.38E-64	6.27E-64	8.61	8.42	9.59	21.32	20.49	21.41
OsR498G0305840900.01	2.276	0.593	-2.016766	3.26E-19	3.98E-19	2.63	1.85	2.35	0.49	0.81	0.48
OsR498G0305842400.01	21.146	46.356	1.1067887	3.47E-169	1.19E-169	22.72	20.97	19.75	45.01	47.17	46.89
OsR498G0305842800.01	9.513	32.123	1.7463259	0	0	9.6	9.38	9.56	33	33.28	30.09
OsR498G0305844700.01	2.2	105.62	5.5850866	0	0	2.22	2.52	1.86	104.82	106.39	105.65
OsR498G0305845000.01	0.5	6.32	3.7550683	1.72E-137	6.89E-138	0.66	0.53	0.31	6.31	5.75	6.9
OsR498G0305845100.01	2.69	7.096	1.3115598	3.94E-29	4.02E-29	2.75	2.17	3.15	7.82	7.22	6.25
OsR498G0305846000.01	0.286	1.976	2.8073	1.35E-10	2.04E-10	0.43	0.11	0.32	2.17	1.54	2.22
OsR498G0305846500.01	69.286	143.09	1.0445518	6.78E-208	1.96E-208	69.28	70.7	67.88	142.01	146.03	141.24

OsR498G0305847900.01	6.29	34.563	2.4632825	0	0	5.57	6.73	6.57	34.87	34.22	34.6
OsR498G0305854800.01	0.306	3.806	3.6367908	2.15E-161	7.67E-162	0.27	0.27	0.38	3.98	4.03	3.41
OsR498G0305855900.01	1.146	12.023	3.4042396	2.42E-188	7.60E-189	1.44	1.13	0.87	12.03	10.65	13.39
OsR498G0305859800.01	1.37	5.546	2.0193717	6.28E-135	2.56E-135	1.48	1.42	1.21	5.44	6.04	5.16
OsR498G0305860200.01	0.15	0.656	2.1212513	3.32E-12	4.79E-12	0.15	0.15	0.15	0.59	0.67	0.71
OsR498G0305862400.01	379.626	176.35	-1.110284	0	0	385.84	378.56	374.48	169.29	175.65	184.12
OsR498G0305863600.01	1.033	25.68	4.6321703	0	0	1.28	0.94	0.88	25.02	26.41	25.61
OsR498G0305867600.01	5.966	50.623	3.0829281	0	0	5.73	6.13	6.04	51.72	47.4	52.75
OsR498G0305870500.01	4.153	17.346	2.057201	0	0	4.1	4.23	4.13	17.37	18.42	16.25
OsR498G0305876600.01	1.056	0.303	-1.780798	5.30E-14	7.28E-14	0.99	1.39	0.79	0.25	0.38	0.28
OsR498G0305879300.01	1.55	0.373	-2.044449	1.52E-18	1.88E-18	1.7	1.71	1.24	0.41	0.29	0.42
OsR498G0305882100.01	103.5	270.49	1.3851192	0	0	104.23	103.9	102.37	266.73	271.36	273.39
OsR498G0305882800.01	12.763	41.583	1.7294726	1.13E-176	3.77E-177	12.22	12.18	13.89	41.94	40.37	42.44
OsR498G0305884200.01	8.74	2.096	-2.055915	4.66E-70	2.93E-70	8.12	8.15	9.95	2.1	1.94	2.25
OsR498G0305884500.01	1.73	0.583	-1.571558	2.80E-07	4.79E-07	2.07	1.13	1.99	0.49	0.83	0.43
OsR498G0305887500.01	0.313	0.1	-1.610553	0.0001627	0.0003196	0.29	0.37	0.28	0.05	0.15	0.1
OsR498G0305887800.01	6.646	19.543	1.5439574	1.20E-149	4.52E-150	7.14	6.56	6.24	20.52	19.43	18.68
OsR498G0305887900.01	0.61	3.626	2.5779084	5.39E-101	2.69E-101	0.62	0.61	0.6	4.23	3.46	3.19
OsR498G0305888500.01	18.346	36.97	1.0113966	1.39E-94	7.27E-95	17.84	18.68	18.52	36.06	38.69	36.16
OsR498G0305888900.01	142.64	42.22	-1.757945	0	0	144.28	133.93	149.71	43.08	39.03	44.55
OsR498G0305890800.01	4.796	27.94	2.5498268	0	0	4.96	5.08	4.35	28.07	28.85	26.9
OsR498G0305891500.01	1.103	7.413	2.7292975	2.40E-163	8.50E-164	0.96	1.34	1.01	7.29	8.29	6.66
OsR498G0305893800.01	15.07	37.413	1.3137106	8.06E-262	1.90E-262	14.78	15.21	15.22	36.47	39.55	36.22
OsR498G0305894900.01	0.05	2.266	5.3581142	1.35E-40	1.16E-40	0.04	0.11	0	2.29	2.2	2.31
OsR498G0305896800.01	1.696	3.816	1.1637953	2.66E-40	2.31E-40	1.66	1.77	1.66	4	3.84	3.61
OsR498G0305899400.01	4	9.546	1.2584414	9.84E-30	9.94E-30	4.31	3.44	4.25	8.52	10.12	10
OsR498G0305903000.01	20.71	10.676	-1.040515	1.63E-94	8.53E-95	20.86	21	20.27	10.72	10.92	10.39
OsR498G0305903800.01	99.756	253.75	1.3498968	0	0	102.28	103.7	93.29	254.29	255.99	250.96
OsR498G0305904000.01	8.796	30.246	1.7844921	1.43E-283	3.18E-284	8.68	8.83	8.88	31.38	29.28	30.08
OsR498G0305907000.01	1.03	7.216	2.7993232	4.25E-116	1.92E-116	1.05	1.09	0.95	6.74	6.91	8
OsR498G0305910100.01	0.216	0	-5.417907	9.99E-07	1.75E-06	0.22	0.17	0.26	0	0	0
OsR498G0305915600.01	2.356	8.72	1.9219425	2.40E-126	1.03E-126	2.33	2.76	1.98	8.6	9.21	8.35
OsR498G0305918400.01	5.49	28.526	2.355604	1.16E-165	4.06E-166	6.47	5.36	4.64	25.81	31.32	28.45
OsR498G0305919100.01	1.203	0.143	-3.273518	3.60E-12	5.21E-12	0.89	1.96	0.76	0	0.25	0.18
OsR498G0305920400.01	0.146	1.326	3.1894228	3.71E-13	5.20E-13	0.06	0.31	0.07	1.46	1.27	1.25
OsR498G0305920600.01	0.106	5.073	5.5593725	9.85E-58	6.98E-58	0.13	0.13	0.06	4.03	5.51	5.68
OsR498G0305921800.01	124.156	38.996	-1.698659	0	0	123.43	124.32	124.72	39.09	39.58	38.32
OsR498G0305923300.01	0.653	4.033	2.6366969	4.75E-104	2.32E-104	0.8	0.67	0.49	3.69	4.52	3.89
OsR498G0305923500.01	35.946	11.376	-1.662631	1.41E-271	3.24E-272	32.08	38.38	37.38	11.79	11.22	11.12
OsR498G0305923800.01	8.59	45.753	2.4126077	2.83E-239	7.31E-240	10.02	7.87	7.88	46.71	45.17	45.38
OsR498G0305924100.01	1.833	5.143	1.5349711	1.88E-35	1.74E-35	1.45	2.17	1.88	5.35	5.33	4.75
OsR498G0305924900.01	397.24	38.163	-3.356647	0	0	367.22	423.35	401.15	35.32	36.15	43.02
OsR498G0305928500.01	26.47	83.873	1.6609391	2.90E-254	7.02E-255	25.79	27.28	26.34	80.51	80.53	90.58
OsR498G0305937300.01	5.913	14.51	1.2930071	4.40E-38	3.93E-38	5.84	7.07	4.83	14.24	13.63	15.66
OsR498G0305937600.01	6.13	13.143	1.1020639	6.21E-84	3.52E-84	6.25	6.09	6.05	13.35	14.01	12.07
OsR498G0305940700.01	1.116	2.593	1.0865203	8.75E-11	1.32E-10	1.17	1.16	1.02	2.39	2.56	2.83
OsR498G0305942000.01	178.333	86.123	-1.050335	0	0	176.25	184.33	174.42	88.03	85.9	84.44
OsR498G0305945800.01	2.323	7.543	1.6863979	7.73E-74	4.72E-74	2.39	2.06	2.52	7.76	7.1	7.77
OsR498G0305946800.01	6.536	17.006	1.3762402	1.32E-118	5.87E-119	6.02	6.97	6.62	16.39	17.29	17.34
OsR498G0305947800.01	0.586	0.213	-1.466163	0.0002798	0.000558	0.79	0.6	0.37	0.23	0.18	0.23
OsR498G0305954400.01	1.293	5.393	2.0541371	1.29E-61	8.74E-62	1.3	1.49	1.09	5.29	5.13	5.76
OsR498G0305955800.01	13.266	41.346	1.6389896	4.13E-119	1.83E-119	12.51	12.67	14.62	40.85	42.58	40.61
OsR498G0305955900.01	1.103	0.173	-2.688555	1.80E-07	3.05E-07	0.96	1.48	0.87	0.17	0.17	0.18
OsR498G0305957500.01	71.133	174.9	1.2835788	0	0	69.47	70.15	73.78	167.66	168.9	188.13
OsR498G0305959300.01	0.51	1.1	1.0524125	1.35E-08	2.19E-08	0.47	0.62	0.44	0.98	1.18	1.14
OsR498G0305966400.01	29.303	68.643	1.2251286	0	0	29.38	29.85	28.68	67.21	71.9	66.82
OsR498G0305967800.01	2.733	5.926	1.0273844	5.08E-22	5.86E-22	2.78	2.26	3.16	6.32	5.68	5.78
OsR498G0305969000.01	19.863	108.52	2.4485372	0	0	19.14	21.15	19.3	109.36	106.32	109.88
OsR498G0305969400.01	0.95	3.8	2.0496981	2.21E-33	2.11E-33	0.91	1.2	0.74	3.72	4.21	3.47
OsR498G0305972400.01	1.016	2.246	1.144335	7.65E-06	1.40E-05	1	1.06	0.99	2.38	2.37	1.99
OsR498G0305973900.01	1.703	0.31	-2.459686	2.26E-36	2.06E-36	1.86	1.4	1.85	0.33	0.22	0.38
OsR498G0305983800.01	0	0.176	4.6748497	0.0001298	0.0002536	0	0	0	0.04	0.16	0.33
OsR498G0305986500.01	0.013	0.693	5.7817649	6.72E-15	9.03E-15	0	0	0.04	0.63	0.62	0.83
OsR498G0305990000.01	2.16	0.88	-1.295679	2.22E-06	3.94E-06	2.22	2.13	2.13	0.46	1.23	0.95
OsR498G0305990700.01	54.133	17.576	-1.397292	8.25E-213	2.34E-213	49.69	53.85	58.86	16.57	17.08	19.08
OsR498G0305991100.01	60.27	292.08	2.2186162	0	0	61.74	61.73	57.34	292.09	280.72	303.44
OsR498G0305997700.01	0.44	1.09	1.2963381	1.09E-10	1.64E-10	0.56	0.37	0.39	0.87	1.13	1.27
OsR498G0305999000.01	23.85	8.303	-1.519495	6.01E-166	2.10E-166	22.89	23.63	25.03	8.8	8.23	7.88
OsR498G0305999600.01	0.553	1.336	1.3093942	2.39E-14	3.26E-14	0.35	0.64	0.67	1.42	1.37	1.22

OsR498G0305999900.01	8.746	22.57	1.354586	4.80E-115	2.19E-115	9.06	8.21	8.97	22.52	22.71	22.48
OsR498G0306000200.01	0.186	4.59	4.6932282	2.61E-90	1.41E-90	0.21	0.17	0.18	4.92	4.72	4.13
OsR498G0306001300.01	11.356	55.166	2.279155	0	0	11.55	10.89	11.63	54.54	52.83	58.13
OsR498G0306002500.01	2.743	7.616	1.4821394	7.62E-49	5.94E-49	2.75	2.9	2.58	7.59	7.39	7.87
OsR498G0306009000.01	3.27	0.813	-2.013908	4.40E-41	3.77E-41	3.32	3.17	3.32	0.7	0.83	0.91
OsR498G0306012500.01	0.87	2.42	1.4769103	7.43E-10	1.15E-09	0.75	0.93	0.93	1.82	2.77	2.67
OsR498G0306019600.01	15.52	34.103	1.1407869	3.01E-158	1.09E-158	15.38	16.11	15.07	36.46	33.05	32.8
OsR498G0306020500.01	5.813	2.523	-1.212245	1.11E-64	7.34E-65	6.05	5.67	5.72	2.6	2.23	2.74
OsR498G0306023500.01	0.1	0.436	2.1588345	1.76E-07	2.99E-07	0.16	0.11	0.03	0.49	0.24	0.58
OsR498G0306025900.01	27.46	13.563	-1.035558	1.08E-110	5.08E-111	27.77	26.96	27.65	13.51	13.45	13.73
OsR498G0306030900.01	2.813	7.076	1.3304744	7.59E-44	6.26E-44	2.81	3.13	2.5	7.5	6.78	6.95
OsR498G0306032700.01	2.833	9.11	1.6677486	1.87E-75	1.13E-75	2.64	3.15	2.71	8.44	9.47	9.42
OsR498G0306037100.01	11.256	23.873	1.0824615	3.13E-49	2.43E-49	12.35	11.26	10.16	24.23	21.57	25.82
OsR498G0306039800.01	1.14	4.036	1.8389465	1.43E-37	1.28E-37	1.14	1.22	1.06	3.75	3.82	4.54
OsR498G0306040800.01	55.386	115.7	1.0606887	0	0	55.71	56.65	53.8	117.98	114.84	114.27
OsR498G0306042000.01	1.746	20.873	3.670575	6.75E-265	1.57E-265	1.69	1.41	2.14	20.41	20.18	22.03
OsR498G0306047600.01	1.306	0.106	-3.610553	7.25E-09	1.16E-08	1.64	0.87	1.41	0.21	0	0.11
OsR498G0306048300.01	3.15	7.273	1.2077086	2.12E-52	1.58E-52	2.88	3.23	3.34	7.17	8.2	6.45
OsR498G0306048900.01	0.68	4.28	2.6579363	1.03E-32	9.94E-33	0.81	0.49	0.74	3.79	4.73	4.32
OsR498G0306049200.01	0.03	0.746	4.6182661	2.44E-07	4.16E-07	0	0	0.09	1.25	0.62	0.37
OsR498G0306052900.01	0.853	3.606	2.0732956	3.12E-36	2.85E-36	0.65	0.99	0.92	3.66	3.42	3.74
OsR498G0306060000.01	1.7	0.546	-1.657858	0.0003249	0.0006505	1.65	2.37	1.08	0.46	0.52	0.66
OsR498G0306062300.01	5.61	12.73	1.1228354	3.90E-65	2.57E-65	5.9	5.06	5.87	12.96	12.81	12.42
OsR498G0306070300.01	0.086	0.466	2.4338416	4.24E-06	7.65E-06	0.13	0	0.13	0.59	0.29	0.52
OsR498G0306076100.01	30.923	212.58	2.7836995	0	0	30.49	31.7	30.58	215.74	218.6	203.39
OsR498G0306078400.01	116.946	58.433	-1.001986	0	0	120.99	114.42	115.43	56.39	57.48	61.43
OsR498G0306080600.01	0.476	0.05	-3.347518	2.54E-07	4.34E-07	0.67	0.47	0.29	0.05	0.05	0.05
OsR498G0306085000.01	18.786	67.566	1.8419528	0	0	18.25	19.7	18.41	65.16	67.82	69.72
OsR498G0306085400.01	61.536	130.03	1.0902412	0	0	62.45	63.47	58.69	130.54	130.75	128.8
OsR498G0306087800.01	2.356	7.013	1.5575288	4.71E-59	3.29E-59	2.24	2.43	2.4	7.63	6.77	6.64
OsR498G0306098600.01	1.683	6.12	1.8383484	2.45E-52	1.83E-52	1.55	1.64	1.86	6.18	6.39	5.79
OsR498G0306105000.01	2.183	6.64	1.5987211	1.10E-52	8.19E-53	1.82	2.28	2.45	6.66	7.07	6.19
OsR498G0306105600.01	4.3	17.296	2.0082348	2.33E-293	5.01E-294	4.51	4.55	3.84	17.44	17.91	16.54
OsR498G0306110200.01	0.223	0.62	1.4637948	1.24E-06	2.18E-06	0.21	0.28	0.18	0.84	0.58	0.44
OsR498G0306113300.01	2.873	6.32	1.1423044	3.95E-34	3.72E-34	2.84	2.79	2.99	6.47	5.81	6.68
OsR498G0306113700.01	2.966	8.75	1.5543015	5.56E-64	3.71E-64	2.97	3.03	2.9	8.7	8.93	8.62
OsR498G0306118900.01	0.443	0.123	-2.227224	0.000178	0.0003504	0.42	0.36	0.55	0	0.18	0.19
OsR498G0306125100.01	5.273	57.146	3.4033717	0	0	5.81	3.75	6.26	51.98	58.29	61.17
OsR498G0306125700.01	29.43	68.466	1.3262042	1.37E-161	4.89E-162	31.41	29.85	27.03	67.06	65.9	72.44
OsR498G0306127400.01	2.11	0.563	-1.911419	3.05E-10	4.68E-10	2.54	1.56	2.23	0.44	0.36	0.89
OsR498G0306129200.01	2.04	0.89	-1.195515	1.10E-06	1.93E-06	1.99	1.85	2.28	1.21	0.78	0.68
OsR498G0306131300.01	159.333	45.513	-1.788923	0	0	155.96	160.86	161.18	46.49	46.73	43.32
OsR498G0306140800.01	0.27	0.9	1.7352223	5.65E-07	9.79E-07	0.23	0.13	0.45	1.23	0.57	0.9
OsR498G0306141500.01	0.233	5.256	4.6182661	1.24E-68	7.92E-69	0.19	0.25	0.26	5.54	4.49	5.74
OsR498G0306142300.01	29.44	78.7	1.4164429	1.42E-306	2.95E-307	31	28.71	28.61	75.22	74.42	86.46
OsR498G0306144200.01	26.043	95.11	1.8599999	0	0	25.73	27.8	24.6	94.36	94.05	96.92
OsR498G0306144900.01	1.743	4.25	1.2826775	1.77E-53	1.31E-53	1.66	1.76	1.81	4.08	4.74	3.93
OsR498G0306152500.01	5.91	22.15	1.8968949	5.06E-152	1.88E-152	6.18	5.58	5.97	21.03	21.49	23.93
OsR498G0306153100.01	1.14	2.24	1.023136	4.04E-12	5.84E-12	0.83	1.31	1.28	2.01	2.43	2.28
OsR498G0306157700.01	4.196	17.68	2.0744353	5.48E-230	1.46E-230	4.64	4.13	3.82	18.17	17.22	17.65
OsR498G0306167000.01	0.24	1.68	2.8084	3.73E-27	3.94E-27	0.22	0.25	0.25	1.8	1.55	1.69
OsR498G0306169900.01	23.823	97.603	2.0322979	0	0	22.02	23.43	26.02	92.56	93.95	106.3
OsR498G0306170200.01	0.02	0.363	4.144335	1.55E-05	2.86E-05	0	0	0.06	0.42	0.42	0.25
OsR498G0306174200.01	0.746	0.153	-2.336035	6.12E-11	9.19E-11	0.59	1.04	0.61	0.06	0.12	0.28
OsR498G0306177300.01	0.603	2.16	1.8285591	4.11E-19	5.02E-19	0.49	0.52	0.8	2.24	2.29	1.95
OsR498G0306181500.01	1.433	9.2	2.6774282	6.40E-43	5.34E-43	1.41	1.69	1.2	7.72	9.16	10.72
OsR498G0306186500.01	6.866	21.113	1.6293166	1.26E-115	5.73E-116	6.68	7.28	6.64	21.3	20.41	21.63
OsR498G0306186700.01	1.65	5.376	1.7029633	1.44E-25	1.56E-25	2.02	1.49	1.44	5.16	4.52	6.45
OsR498G0306188000.01	7.763	27.336	1.8150885	8.54E-177	2.83E-177	7.12	7.99	8.18	28.53	25.46	28.02
OsR498G0306192500.01	1.746	0.556	-1.64511	1.83E-15	2.43E-15	1.71	1.61	1.92	0.48	0.62	0.57
OsR498G0306193100.01	4.556	12.243	1.4189538	1.00E-107	4.78E-108	4.02	4.59	5.06	12.75	11.54	12.44
OsR498G0306200600.01	24.496	6.536	-1.892123	1.31E-84	7.39E-85	26.39	24.36	22.74	5.8	6.35	7.46
OsR498G0306205000.01	0.103	0.496	2.1968024	1.17E-06	2.06E-06	0.07	0.09	0.15	0.52	0.49	0.48
OsR498G0306209100.01	0.22	0.01	-4.576491	4.63E-07	7.99E-07	0.39	0.07	0.2	0.03	0	0
OsR498G0306211200.01	0.853	0.136	-3.011651	3.51E-19	4.29E-19	0.9	0.72	0.94	0.08	0.16	0.17
OsR498G0306216600.01	0.613	0.01	-5.80695	6.78E-15	9.12E-15	0.54	0.6	0.7	0	0.03	0
OsR498G0306223200.01	0.533	1.336	1.3215185	1.91E-06	3.38E-06	0.72	0.44	0.44	1.2	1.19	1.62
OsR498G0306223900.01	5.693	11.7	1.0449701	2.28E-49	1.77E-49	6.06	5.82	5.2	11.15	12.85	11.1
OsR498G0306232800.01	13	38.706	1.5695769	5.36E-264	1.25E-264	13.54	13.54	11.92	40.61	38.09	37.42

OsR498G0306235500.01	0.41	1.226	1.5338374	7.48E-14	1.03E-13	0.3	0.4	0.53	1.32	1.3	1.06
OsR498G0306236600.01	11.296	24.573	1.1348746	4.48E-94	2.35E-94	11.97	10.86	11.06	25.18	24.05	24.49
OsR498G0306236800.01	0.146	1.173	3.022773	6.12E-17	7.84E-17	0.19	0.18	0.07	1.02	1.27	1.23
OsR498G0306240900.01	24.263	109.47	2.1804186	0	0	25.36	22.68	24.75	115.14	107.89	105.39
OsR498G0306241200.01	2.156	6.323	1.5547111	1.81E-69	1.14E-69	2.21	2.25	2.01	6.73	6.21	6.03
OsR498G0306241700.01	16.143	90.73	2.4887611	0	0	15.1	16.9	16.43	91.1	90.29	90.8
OsR498G0306242300.01	5.896	20.463	1.6540947	4.90E-116	2.22E-116	5.79	5.69	6.21	19.86	20.48	21.05
OsR498G0306249200.01	2.47	1.063	-1.001743	3.12E-06	5.58E-06	2.34	2.81	2.26	1.4	0.85	0.94
OsR498G0306250100.01	10.776	22.733	1.0775402	4.14E-182	1.34E-182	10.61	10.92	10.8	23.1	22.49	22.61
OsR498G0306253900.01	5.313	14.32	1.4287883	1.47E-38	1.31E-38	5.62	5.73	4.59	14.5	12.36	16.1
OsR498G0306261700.01	107.46	46.21	-1.204037	1.34E-217	3.71E-218	104.85	112.26	105.27	47.29	43.7	47.64
OsR498G0306262000.01	25.77	2.446	-3.360762	0	0	26.09	25.61	25.61	2.45	2.5	2.39
OsR498G0306263800.01	19.49	7.153	-1.446887	5.40E-43	4.51E-43	18.88	20.65	18.94	6.95	7.39	7.12
OsR498G0306264100.01	0.716	0.143	-2.361873	0.0002609	0.0005189	0.51	1.09	0.55	0.11	0.21	0.11
OsR498G0306265800.01	0.066	0.433	2.6524819	4.15E-11	6.19E-11	0.12	0.02	0.06	0.44	0.32	0.54
OsR498G0306276700.01	0.96	2.396	1.3436438	9.82E-13	1.39E-12	1.06	1.21	0.61	2.36	2.57	2.26
OsR498G0306282800.01	3.273	7.696	1.227176	1.77E-07	3.00E-07	3.35	2.31	4.16	6.77	7.56	8.76
OsR498G0306285400.01	0.11	2.336	4.3894475	9.68E-30	9.78E-30	0	0.22	0.11	2.77	1.62	2.62
OsR498G0306291900.01	0.05	0.283	2.5593725	0.0003356	0.0006725	0.1	0	0.05	0.28	0.14	0.43
OsR498G0306295300.01	343.633	133.86	-1.338974	0	0	348.86	337.63	344.41	131.25	131.96	138.38
OsR498G0306297500.01	141.763	460.71	1.6944832	0	0	141.41	141.43	142.45	461.64	454.72	465.77
OsR498G0306299600.01	6.54	15.533	1.2931715	5.76E-115	2.63E-115	6.51	7.2	5.91	15.36	15.33	15.91
OsR498G0306321700.01	3.85	16.73	2.1373485	1.32E-152	4.90E-153	4.07	3.55	3.93	15.88	17.39	16.92
OsR498G0306323800.01	0.016	1.1	6.0404991	1.93E-17	2.44E-17	0	0	0.05	0.68	1.36	1.26
OsR498G0306326100.01	0.463	0	-5.485022	5.65E-07	9.78E-07	0.38	0.76	0.25	0	0	0
OsR498G0306338700.01	81.62	258.56	1.6783198	0	0	83.75	79.62	81.49	260.04	266.28	249.36
OsR498G0306350200.01	6.813	3.363	-1.050085	8.04E-14	1.11E-13	6.12	7.03	7.29	3.7	3.28	3.11
OsR498G0306370000.01	0.623	0	-4.34896	0.0006975	0.0014313	0.37	0.94	0.56	0	0	0
OsR498G0306381300.01	0.616	0.12	-2.371365	9.55E-10	1.49E-09	0.52	0.51	0.82	0.06	0.12	0.18
OsR498G0306382200.01	54.95	18.353	-1.546471	0	0	55.12	54.62	55.11	18.41	17.51	19.14
OsR498G0306383300.01	0.133	0.31	1.2444991	0.0008329	0.0017234	0.09	0.12	0.19	0.2	0.36	0.37
OsR498G0306387300.01	4.383	23.496	2.4666905	8.80E-115	4.02E-115	5.01	3.5	4.64	22.74	24.21	23.54
OsR498G0306391400.01	7.313	15.26	1.0693415	2.59E-49	2.01E-49	7.54	6.99	7.41	14.88	14.23	16.67
OsR498G0306392300.01	350.16	119.09	-1.555224	0	0	353.54	352.79	344.15	120	118.28	119
OsR498G0306393900.01	6.246	17.176	1.4535957	3.21E-163	1.14E-163	6.01	6.59	6.14	17.22	17	17.31
OsR498G0306394200.01	6.633	16.643	1.2141845	2.21E-60	1.52E-60	6.44	7.01	6.45	16.96	17.84	15.13
OsR498G0306397400.01	11.71	24.16	1.0393012	1.79E-82	1.02E-82	11.99	11.86	11.28	24.62	23.26	24.6
OsR498G0306399600.01	13.326	44.41	1.7467308	0	0	12.78	13.29	13.91	46.21	43.67	43.35
OsR498G0306399900.01	0.406	1.113	1.4524573	0.0002901	0.000579	0.44	0.26	0.52	1.11	1.53	0.7
OsR498G0306409400.01	1.893	4.966	1.3862802	7.51E-30	7.58E-30	1.65	1.89	2.14	4.96	5.32	4.62
OsR498G0306414400.01	0.05	0	-4.832945	6.10E-05	0.0001167	0	0	0.15	0	0	0
OsR498G0306415400.01	4.72	10.04	1.0447993	2.02E-32	1.96E-32	5.06	5.02	4.08	11.37	8.53	10.22
OsR498G0306415800.01	22.113	67.466	1.6101633	0	0	21.14	21.92	23.28	66.24	66.59	69.57
OsR498G0306419400.01	0	0.36	5.2223375	3.89E-06	7.00E-06	0	0	0	0.45	0.28	0.35
OsR498G0306425400.01	0.586	0.126	-2.610553	0.0002631	0.0005232	1.01	0.75	0	0	0.12	0.26
OsR498G0306427000.01	54.666	270.59	2.3032821	0	0	56.12	54.29	53.59	275.29	274.35	262.12
OsR498G0306428400.01	7.47	29.98	2.0048026	0	0	7.28	7.21	7.92	29.64	31.38	28.92
OsR498G0306432300.01	7.23	14.56	1.0123778	1.35E-53	9.92E-54	8.46	6.58	6.65	14.3	14.71	14.67
OsR498G0306434000.01	36.066	124.96	1.7941669	0	0	37.08	35.19	35.93	122.34	123	129.54
OsR498G0306434900.01	84.243	200.29	1.2455205	0	0	83.6	86.72	82.41	197.47	206.78	196.61
OsR498G0306435600.01	6.54	2.813	-1.217939	1.44E-37	1.30E-37	6.74	6.43	6.45	2.55	2.66	3.23
OsR498G0306438900.01	0.6	0.04	-3.832945	2.03E-10	3.09E-10	0.39	0.81	0.6	0.04	0.04	0.04
OsR498G0306442600.01	19.246	84.896	2.1394767	0	0	19.77	17.93	20.04	82.98	83.55	88.16
OsR498G0306444400.01	0.596	0.096	-2.6445	4.06E-08	6.71E-08	0.67	0.58	0.54	0.08	0.08	0.13
OsR498G0306448100.01	2.406	5.97	1.22755	3.24E-14	4.43E-14	2.25	2.8	2.17	5.11	5.75	7.05
OsR498G0306453400.01	11.676	39.23	1.7293955	0	0	12.23	11.2	11.6	38.84	38.88	39.97
OsR498G0306461200.01	13.083	71.196	2.02429	0	0	12.35	13.39	13.51	72.28	72.7	68.61
OsR498G0306462000.01	5.846	2.653	-1.122788	6.40E-31	6.36E-31	5.72	4.85	6.97	2.71	2.22	3.03
OsR498G0306466900.01	81.516	19.116	-2.074922	0	0	77.46	85.26	81.83	19.09	18.53	19.73
OsR498G0306468000.01	2.51	12.013	2.2664116	6.98E-181	2.27E-181	2.47	2.23	2.83	12.58	11.56	11.9
OsR498G0306468100.01	7.536	19.763	1.4115086	9.44E-163	3.35E-163	7.42	7.32	7.87	19.77	20.6	18.92
OsR498G0306483100.01	1.073	4.19	2.0629201	4.85E-76	2.91E-76	0.97	1.02	1.23	3.91	4.11	4.55
OsR498G0306484100.01	0.326	0	-6.832945	9.77E-15	1.32E-14	0.3	0.3	0.38	0	0	0
OsR498G0306485300.01	2283.45	406.99	-2.489453	0	0	2286.1	2217.9	2346.3	403.44	391.34	426.18
OsR498G0306488000.01	13.993	3.443	-2.031013	9.51E-98	4.86E-98	14.01	13.08	14.89	3.22	3.61	3.5
OsR498G0306490700.01	0.773	14.986	4.2743634	0	0	0.73	0.67	0.92	15.35	14.68	14.93
OsR498G0306492900.01	82.44	38.623	-1.094153	0	0	80.48	84.96	81.88	38.71	39.03	38.13
OsR498G0306495200.01	0.893	5.18	2.5933198	3.95E-20	4.72E-20	1.08	0.87	0.73	5.48	5.26	4.8
OsR498G0306496600.01	0.51	0.956	1.0917669	3.54E-07	6.09E-07	0.43	0.47	0.63	0.91	0.87	1.09

OsR498G0306498400.01	2.826	0.543	-2.388673	2.92E-10	4.47E-10	2.08	3.71	2.69	0.47	0.53	0.63
OsR498G0306500800.01	2.203	7.536	1.7786388	1.35E-42	1.13E-42	2.03	1.98	2.6	7.18	7.42	8.01
OsR498G0306503000.01	1.32	4.176	1.6327456	1.04E-58	7.30E-59	1.23	1.09	1.64	3.89	4.55	4.09
OsR498G0306506000.01	9.75	45.59	2.2544258	4.21E-271	9.65E-272	9.4	8.64	11.21	45.72	42.95	48.1
OsR498G0306507000.01	0.186	0.763	1.9004094	1.06E-14	1.43E-14	0.21	0.17	0.18	0.71	1.01	0.57
OsR498G0306508200.01	48.996	177.8	1.8564168	0	0	48.02	48.92	50.05	172.75	179	181.66
OsR498G0306509200.01	3.863	9.406	1.2857244	7.82E-28	8.17E-28	3.66	3.81	4.12	10.47	9.43	8.32
OsR498G0306511400.01	6.41	14.493	1.1701855	2.61E-70	1.64E-70	6.54	5.95	6.74	12.99	15.55	14.94
OsR498G0306516100.01	1.593	38.686	4.6149572	0	0	1.59	1.36	1.83	38.42	37.93	39.71
OsR498G0306516400.01	0.673	3.096	2.527459	1.40E-19	1.69E-19	0.76	0.83	0.43	3.68	2.65	2.96
OsR498G0306516900.01	0.293	2.7	3.1058608	1.73E-58	1.22E-58	0.32	0.32	0.24	2.95	2.89	2.26
OsR498G0306524700.01	0.09	1.24	3.9051473	2.84E-14	3.88E-14	0.2	0	0.07	1.22	1.25	1.25
OsR498G0306526900.01	5.37	1.726	-1.631926	7.62E-67	4.93E-67	5.64	5.22	5.25	1.96	1.74	1.48
OsR498G0306531800.01	3.76	0.46	-3.041187	1.91E-33	1.82E-33	3.78	3.81	3.69	0.24	0.71	0.43
OsR498G0306534800.01	0.186	0.536	1.3788002	0.0001923	0.0003792	0.2	0.23	0.13	0.55	0.56	0.5
OsR498G0306544400.01	2.61	0.823	-1.674683	3.07E-13	4.30E-13	2.51	2.94	2.38	0.78	0.61	1.08
OsR498G0306547800.01	0.423	4.523	3.4127028	6.81E-52	5.13E-52	0.56	0.3	0.41	4.27	4.2	5.1
OsR498G0306549800.01	7.7	3.503	-1.136111	5.90E-33	5.66E-33	7.57	8.02	7.51	3.8	3.3	3.41
OsR498G0306554000.01	11.933	41.596	1.8055978	1.73E-198	5.21E-199	11.2	11.87	12.73	40.88	43.22	40.69
OsR498G0306554100.01	0.953	2.336	1.284177	0.0004175	0.0008417	0.87	1.99	0	0.29	1.83	4.89
OsR498G0306559300.01	0.386	0.03	-3.655529	0.0003862	0.000777	0.46	0.54	0.16	0	0	0.09
OsR498G0306564400.01	10.22	22.446	1.0600161	5.13E-217	1.43E-217	9.77	10.55	10.34	21.96	23.72	21.66
OsR498G0306571500.01	7.956	22.586	1.5124828	5.56E-182	1.80E-182	7.54	8.27	8.06	24.16	22.24	21.36
OsR498G0306573300.01	0.316	0.71	1.1699608	4.53E-05	8.57E-05	0.37	0.31	0.27	0.77	0.69	0.67
OsR498G0306575100.01	0.396	1.003	1.3348122	2.76E-07	4.71E-07	0.52	0.48	0.19	1.07	0.97	0.97
OsR498G0306579600.01	0.326	1.873	2.5373462	8.42E-11	1.27E-10	0.27	0.27	0.44	1.56	2.36	1.7
OsR498G0306580500.01	2.853	5.923	1.0532654	2.73E-09	4.32E-09	2.9	2.78	2.88	6.29	5.23	6.25
OsR498G0306581200.01	0.333	1.79	2.4338416	1.41E-07	2.38E-07	0.25	0.5	0.25	2.06	1.57	1.74
OsR498G0306586700.01	1.07	0.303	-1.811465	4.85E-11	7.24E-11	1.13	0.96	1.12	0.31	0.41	0.19
OsR498G0306593100.01	0.443	1.47	1.7327041	2.17E-15	2.88E-15	0.4	0.45	0.48	1.55	1.79	1.07
OsR498G0306593400.01	0.1	0.673	2.7629059	2.36E-13	3.29E-13	0.05	0.11	0.14	0.79	0.55	0.68
OsR498G0306595700.01	15.33	62.206	2.0118321	0	0	15.59	16.04	14.36	64.25	63.99	58.38
OsR498G0306597000.01	4.696	41.843	3.1540074	0	0	4.53	4.5	5.06	39.65	40.89	44.99
OsR498G0306597900.01	20.076	52.213	1.4302777	9.62E-256	2.32E-256	20.78	20.82	18.63	51.13	54.04	51.47
OsR498G0306604300.01	4.246	0.693	-2.66476	2.72E-70	1.71E-70	4.56	3.79	4.39	0.67	0.55	0.86
OsR498G0306604800.01	22.34	47.59	1.0893497	3.99E-77	2.38E-77	22.01	22.09	22.92	44.45	49.08	49.24
OsR498G0306611700.01	0.446	0.126	-1.803198	0.0007121	0.001463	0.39	0.17	0.78	0.11	0.05	0.22
OsR498G0306612000.01	0.653	2.203	1.7334019	7.46E-17	9.58E-17	0.34	0.79	0.83	2.17	2.24	2.2
OsR498G0306612400.01	2.786	12.81	2.2003823	1.06E-64	7.00E-65	2.7	2.1	3.56	10.78	14.62	13.03
OsR498G0306624300.01	0.236	0.053	-2.151121	2.53E-07	4.32E-07	0.3	0.25	0.16	0.09	0.04	0.03
OsR498G0306624800.01	1.623	0.303	-2.428946	1.04E-67	6.69E-68	1.92	1.6	1.35	0.3	0.36	0.25
OsR498G0306636000.01	10.753	23.273	1.1044541	3.12E-73	1.92E-73	10.36	9.95	11.95	23.19	22.58	24.05
OsR498G0306637600.01	0.79	0.346	-1.213217	0.0009328	0.0019394	0.77	0.61	0.99	0.23	0.58	0.23
OsR498G0306640800.01	5.616	11.453	1.0242291	2.01E-16	2.60E-16	4.67	6.91	5.27	10.76	10.01	13.59
OsR498G0306643800.01	0.48	3.223	2.7503152	1.29E-56	9.26E-57	0.49	0.54	0.41	2.88	3.39	3.4
OsR498G0306644400.01	2.503	0.6	-2.065556	4.67E-42	3.94E-42	2.5	2.2	2.81	0.48	0.62	0.7
OsR498G0306649900.01	3.7	1.68	-1.126391	3.60E-09	5.73E-09	4.5	3.02	3.58	1.86	1.37	1.81
OsR498G0306653600.01	6.083	19.966	1.719491	5.23E-167	1.81E-167	5.99	5.81	6.45	19.26	20.89	19.75
OsR498G0306653900.01	10.993	30.043	1.4648202	1.15E-218	3.16E-219	9.72	10.96	12.3	30.53	30.78	28.82
OsR498G0306662200.01	0.263	1.063	2.0125451	7.83E-14	1.08E-13	0.2	0.2	0.39	1.24	0.99	0.96
OsR498G0306662300.01	0.156	2.226	3.8157122	1.92E-10	2.92E-10	0.16	0	0.31	2.16	1.38	3.14
OsR498G0306664100.01	0.656	0.136	-2.254409	5.24E-11	7.85E-11	0.79	0.55	0.63	0.18	0.1	0.13
OsR498G0306667400.01	0.186	0.983	2.4022715	1.60E-16	2.07E-16	0.21	0.16	0.19	1.17	0.93	0.85
OsR498G0306670200.01	3.823	7.893	1.0540942	2.62E-32	2.54E-32	4.01	3.61	3.85	7.82	7.58	8.28
OsR498G0306671900.01	5.183	11.936	1.1957967	1.38E-48	1.08E-48	5.32	5.49	4.74	12.08	11.6	12.13
OsR498G0306675300.01	11.566	4.82	-1.312211	3.43E-40	2.98E-40	11.5	11.17	12.03	4.4	4.85	5.21
OsR498G0306680500.01	2.373	9.6	2.0172958	7.86E-102	3.90E-102	2.22	2.35	2.55	10.08	9.83	8.89
OsR498G0306680800.01	0.75	0.323	-1.172431	0.000107	0.0002078	0.89	0.74	0.62	0.32	0.29	0.36
OsR498G0306684400.01	47.593	11.6	-2.040103	1.51E-120	6.66E-121	48.37	49.61	44.8	12.96	9.11	12.73
OsR498G0306694200.01	0	0.516	5.8813006	8.71E-09	1.40E-08	0	0	0	0.37	0.52	0.66
OsR498G0306694400.01	6.45	25.76	2.0044596	4.59E-208	1.33E-208	6.57	6.4	6.38	25.07	26.21	26
OsR498G0306694600.01	0	0.616	6.6182661	2.90E-13	4.06E-13	0	0	0	0.88	0.33	0.64
OsR498G0306696300.01	2.04	0.806	-1.347518	4.14E-11	6.18E-11	2.38	1.61	2.13	0.58	0.77	1.07
OsR498G0306696800.01	15.986	37.736	1.2374444	4.59E-128	1.95E-128	16.21	15.68	16.07	37.65	37.68	37.88
OsR498G0306696900.01	2.613	9.006	1.7938377	3.90E-50	2.99E-50	2.15	2.75	2.94	9.45	9.44	8.13
OsR498G0306699300.01	8.593	19.966	1.2153463	1.97E-93	1.04E-93	9.03	8.67	8.08	19.66	20.8	19.44
OsR498G0306700700.01	1.876	0.596	-1.650081	8.45E-11	1.27E-10	1.84	2.12	1.67	0.54	0.64	0.61
OsR498G0306701000.01	0.873	0.34	-1.347518	0.0002154	0.0004259	0.88	0.93	0.81	0.28	0.57	0.17
OsR498G0306703500.01	0.613	0.086	-2.797501	1.13E-10	1.70E-10	0.78	0.52	0.54	0	0.18	0.08

OsR498G0306703700.01	4.74	46.863	3.3060377	0	0	4.4	5.57	4.25	47.72	44.16	48.71
OsR498G0306704600.01	0.196	0.426	1.1142385	0.0001089	0.0002116	0.26	0.23	0.1	0.38	0.48	0.42
OsR498G0306705400.01	1.976	0.016	-6.767057	1.35E-26	1.44E-26	1.34	2.37	2.22	0	0.05	0
OsR498G0306714400.01	10.94	34.773	1.6673348	2.86E-137	1.15E-137	10.5	10.83	11.49	36.01	33.01	35.3
OsR498G0306714800.01	12.51	36.096	1.5278315	0	0	12.54	12.36	12.63	35.69	35.96	36.64
OsR498G0306718600.01	12.093	4.343	-1.478102	2.26E-23	2.54E-23	12.14	11.27	12.87	4.66	4.53	3.84
OsR498G0306721300.01	0	1.103	4.7817649	7.17E-05	0.0001377	0	0	0	1.18	1.17	0.96
OsR498G0306727100.01	2.623	7.1	1.434521	2.96E-36	2.71E-36	2.46	2.64	2.77	6.69	7.45	7.16
OsR498G0306728600.01	0.05	0.766	3.8813006	5.93E-11	8.90E-11	0.1	0	0.05	0.72	0.89	0.69
OsR498G0306729500.01	0.23	0.916	2.0139383	1.58E-09	2.47E-09	0.19	0.23	0.27	1.22	0.92	0.61
OsR498G0306732000.01	0.503	1.136	1.1760438	0.0007294	0.0015004	0.3	0.53	0.68	1.18	1.1	1.13
OsR498G0306732200.01	4.64	0.18	-4.683802	6.84E-48	5.39E-48	4.78	4.26	4.88	0.27	0.13	0.14
OsR498G0306738200.01	3.6	7.49	1.055241	8.29E-27	8.81E-27	3.75	3.14	3.91	7.47	6.86	8.14
OsR498G0306738300.01	0.133	2.176	4.0496981	1.79E-26	1.92E-26	0.17	0.17	0.06	2.47	1.58	2.48
OsR498G0306740400.01	1.686	0.19	-3.077679	4.47E-26	4.81E-26	1.79	1.74	1.53	0.18	0.14	0.25
OsR498G0306741600.01	0.516	0.073	-2.809861	1.48E-11	2.18E-11	0.35	0.8	0.4	0.15	0.02	0.05
OsR498G0306744000.01	4.206	0.893	-2.289323	2.07E-53	1.53E-53	4.32	3.59	4.71	0.9	0.82	0.96
OsR498G0306744300.01	0.81	2.136	1.3804023	2.90E-08	4.77E-08	0.61	0.91	0.91	1.92	2.37	2.12
OsR498G0306751800.01	0.23	0	-5.03637	1.72E-05	3.19E-05	0.24	0.17	0.28	0	0	0
OsR498G0306753800.01	3.646	10.593	1.5377424	1.24E-25	1.34E-25	4.46	3.27	3.21	9.55	11.26	10.97
OsR498G0306756300.01	5.016	2.266	-1.145153	1.19E-45	9.61E-46	4.74	5.46	4.85	2.19	2.4	2.21
OsR498G0306756900.01	7.066	1.663	-1.289191	2.28E-30	2.28E-30	6.25	8.95	6	1.37	1.75	1.87
OsR498G0306759300.01	3.633	0.86	-2.079457	5.07E-23	5.75E-23	3.84	3.35	3.71	0.95	0.89	0.74
OsR498G0306760600.01	3.196	10.036	1.607871	5.48E-76	3.29E-76	3.35	3.03	3.21	9.34	10.29	10.48
OsR498G0306761200.01	0.76	2.956	2.0447993	1.66E-17	2.10E-17	0.79	0.82	0.67	2.59	3.21	3.07
OsR498G0306762100.01	7.816	16.36	1.0638002	3.36E-32	3.26E-32	7.5	8.1	7.85	16.64	14.43	18.01
OsR498G0306763700.01	5.596	15.553	1.4794042	3.97E-41	3.40E-41	6.72	4.9	5.17	15.2	13.71	17.75
OsR498G0306765200.01	0.313	0.756	1.2747482	0.0003103	0.0006204	0.57	0.12	0.25	0.47	1.12	0.68
OsR498G0306772900.01	2.526	78.15	4.9486333	0	0	2.79	1.77	3.02	75.68	72.42	86.35
OsR498G0306779000.01	0.796	0.16	-2.347518	0.0002903	0.0005793	0.84	1.19	0.36	0.12	0.12	0.24
OsR498G0306781800.01	0	0.423	4.6748497	0.0001298	0.0002536	0	0	0	0.48	0.19	0.6
OsR498G0306787500.01	3.326	1.233	-1.437716	1.08E-23	1.21E-23	3.98	3.04	2.96	1.03	1.12	1.55
OsR498G0306797900.01	1.75	3.613	1.0964005	6.74E-24	7.52E-24	1.94	1.75	1.56	3.84	3.48	3.52
OsR498G0306806900.01	5.923	47.206	2.9936266	0	0	6.4	5.72	5.65	46.29	45.44	49.89
OsR498G0306811900.01	4.46	11.28	1.33698	3.54E-144	1.37E-144	4.68	4.39	4.31	11.24	11.01	11.59
OsR498G0306814500.01	1.743	4.446	1.5036999	6.42E-60	4.43E-60	1.49	1.95	1.79	4.61	4.59	4.14
OsR498G0306815000.01	115.683	3.646	-4.989883	0	0	118.56	116.38	112.11	3.52	3.51	3.91
OsR498G0306815100.01	6.12	2.09	-1.510441	2.15E-20	2.56E-20	6.21	6.15	6	2.43	2.42	1.42
OsR498G0306815700.01	0.736	2.533	1.7870398	5.07E-14	6.96E-14	0.74	0.51	0.96	2.53	2.3	2.77
OsR498G0306817500.01	4.563	9.74	1.0920787	4.55E-25	4.97E-25	4.48	3.98	5.23	8.87	10.11	10.24
OsR498G0306818600.01	105.74	381.93	1.8513915	0	0	105.53	107.03	104.66	378.14	367.25	400.41
OsR498G0306818800.01	5.416	14.546	1.4403549	2.23E-81	1.29E-81	4.73	5.13	6.39	14.64	14.42	14.58
OsR498G0306820600.01	20.3	3.93	-2.388783	3.78E-132	1.57E-132	21.92	19.21	19.77	3.09	4.52	4.18
OsR498G0306820700.01	5.826	2.48	-1.254385	5.62E-34	5.31E-34	6.66	5.1	5.72	2.23	2.25	2.96
OsR498G0306827000.01	0.426	15.703	5.1974463	4.79E-174	1.61E-174	0.32	0.51	0.45	16.07	13.51	17.53
OsR498G0306828100.01	8.796	18.796	1.1003296	5.35E-74	3.26E-74	8.76	8.4	9.23	18.95	18.52	18.92
OsR498G0306828300.01	0.26	1.073	2.0447993	0.0006522	0.0013355	0.16	0.31	0.31	0.76	1.37	1.09
OsR498G0306837400.01	0.76	0.233	-1.703662	8.32E-06	1.52E-05	1.1	0.8	0.38	0.23	0.28	0.19
OsR498G0306839100.01	14.27	6.606	-1.146285	7.88E-20	9.49E-20	17.73	13.16	11.92	6.05	7.22	6.55
OsR498G0306843600.01	32.523	76.45	1.2315943	7.64E-206	2.24E-206	33.22	32.74	31.61	75.84	72.57	80.94
OsR498G0306845300.01	1.226	8.36	2.7667186	6.40E-190	2.00E-190	0.91	1.49	1.28	8.43	8.53	8.12
OsR498G0306848200.01	0.366	0.036	-3.258251	2.64E-10	4.03E-10	0.33	0.4	0.37	0.02	0.04	0.05
OsR498G0306850400.01	15.63	63.36	2.0144332	0	0	15.47	16.39	15.03	62.95	63.9	63.23
OsR498G0306851400.01	0.016	0.503	4.8073	1.01E-14	1.37E-14	0.05	0	0	0.55	0.55	0.41
OsR498G0306851600.01	8.216	41.486	2.3340912	0	0	7.95	8.15	8.55	39.71	40.8	43.95
OsR498G0306853200.01	0	0.49	5.5593725	2.32E-07	3.95E-07	0	0	0	0.24	0.67	0.56
OsR498G0306854600.01	1.873	10.37	2.4658355	4.43E-74	2.70E-74	1.65	1.22	2.75	10.01	9.95	11.15
OsR498G0306856700.01	6.466	19.07	1.5605468	4.60E-272	1.05E-272	6.52	6.7	6.18	19.53	20.36	17.32
OsR498G0306859400.01	22.423	48.71	1.1240004	2.10E-116	9.51E-117	22.24	22.29	22.74	46.3	49.43	50.4
OsR498G0306860100.01	9.703	22.723	1.0574657	4.16E-82	2.39E-82	9.89	9.64	9.58	20.41	23.34	24.42
OsR498G0306870500.01	3.433	9.933	1.5308973	3.01E-66	1.96E-66	3.55	3.3	3.45	10.55	9.65	9.6
OsR498G0306870800.01	5.153	10.866	1.0715391	2.05E-26	2.19E-26	4.97	5.25	5.24	10.54	11.14	10.92
OsR498G0306873300.01	0.456	2.756	2.6156799	1.31E-61	8.94E-62	0.41	0.45	0.51	2.83	2.26	3.18
OsR498G0306876900.01	0.566	3.423	2.5933198	2.54E-32	2.46E-32	0.43	0.57	0.7	3.32	3.71	3.24
OsR498G0306877500.01	20.693	42.573	1.0464364	6.16E-61	4.23E-61	21.03	22.31	18.74	45.98	41.72	40.02
OsR498G0306878800.01	2.13	5.2	1.2861182	2.14E-13	2.99E-13	1.74	2.47	2.18	5.36	4.82	5.42
OsR498G0306884400.01	4.44	0.816	-2.442748	5.00E-63	3.36E-63	4.47	4.76	4.09	0.77	0.98	0.7
OsR498G0306884800.01	0.15	0.746	2.6748497	2.94E-13	4.12E-13	0.23	0.05	0.17	0.81	0.63	0.8
OsR498G0306887100.01	7.43	15.19	1.0597318	1.65E-42	1.39E-42	7.24	6.8	8.25	13.99	15.18	16.4

OsR498G0306887300.01	85.136	184.77	1.1146631	0	0	90.48	84.48	80.45	182.85	184.54	186.92
OsR498G0306893700.01	4.646	1.906	-1.278159	1.14E-27	1.19E-27	4.86	4.49	4.59	1.81	1.86	2.05
OsR498G0306899500.01	45.63	21.24	-1.097418	7.06E-241	1.81E-241	45.63	46	45.26	22.89	20.98	19.85
OsR498G0306904700.01	152.973	356.07	1.2229777	0	0	155.86	154.94	148.12	355.21	362.01	350.99
OsR498G0306906900.01	0.533	1.51	1.5169372	3.00E-11	4.46E-11	0.44	0.58	0.58	1.55	1.52	1.46
OsR498G0306907200.01	10.863	54.186	2.3168256	0	0	11.49	10.32	10.78	53.91	54.73	53.92
OsR498G0306908200.01	2.35	1.166	-1.007888	9.14E-05	0.0001767	2.71	1.91	2.43	1.36	1.18	0.96
OsR498G0306911600.01	9.083	38.446	2.0841899	3.35E-268	7.74E-269	9.1	9.19	8.96	37.35	39.12	38.87
OsR498G0306916100.01	2.153	5.473	1.3821574	5.28E-22	6.10E-22	1.84	2.5	2.12	5.23	5.26	5.93
OsR498G0306917500.01	0.303	1.213	2.0139383	2.01E-13	2.80E-13	0.24	0.4	0.27	0.71	1.6	1.33
OsR498G0306918600.01	1.283	3.583	1.5291578	3.07E-42	2.59E-42	1.29	1.18	1.38	3.43	3.44	3.88
OsR498G0306924000.01	0.513	2.116	2.0484105	6.21E-19	7.60E-19	0.46	0.63	0.45	1.74	2.55	2.06
OsR498G0306934300.01	0.08	0	-4.620139	0.0001953	0.0003852	0	0	0.24	0	0	0
OsR498G0306937800.01	4.083	13.866	1.7590723	3.49E-73	2.14E-73	4.62	4.47	3.16	13.9	13.93	13.77
OsR498G0306938500.01	52.536	118.19	1.1683254	0	0	52.49	51.16	53.96	119.36	119.5	115.7
OsR498G0306941400.01	6.946	15.84	1.1396252	6.15E-40	5.36E-40	7.88	6.78	6.18	15.89	15.76	15.87
OsR498G0306942100.01	1.103	2.813	1.3529216	6.05E-08	1.01E-07	1.33	0.91	1.07	2.5	3.21	2.73
OsR498G0306942400.01	1.986	0.44	-2.179713	1.49E-28	1.53E-28	1.84	2.08	2.04	0.54	0.41	0.37
OsR498G0306945600.01	9.813	30.883	1.6456963	0	0	10.11	10.18	9.15	30.86	31.72	30.07
OsR498G0306951500.01	0	0.326	6.0126705	1.89E-09	2.97E-09	0	0	0	0.98	0	0
OsR498G0306954300.01	3.303	11.16	1.7573367	0	0	3.36	3.3	3.25	11.48	11.8	10.2
OsR498G0306954800.01	8.206	23.743	1.5315978	6.12E-101	3.06E-101	8.33	8.31	7.98	23.92	22.45	24.86
OsR498G0306955700.01	0.926	2.023	1.1212513	0.0001972	0.0003891	0.7	0.89	1.19	2.23	1.55	2.29
OsR498G0306956300.01	13.346	27.693	1.056364	3.28E-182	1.06E-182	13.71	14.11	12.22	27.06	28.31	27.71
OsR498G0306957500.01	0.926	3.816	2.0447993	8.02E-49	6.25E-49	0.82	1.22	0.74	3.83	3.65	3.97
OsR498G0306960100.01	0.03	0.96	5.4662631	2.74E-12	3.94E-12	0	0.09	0	1.27	0.63	0.98
OsR498G0306960300.01	2.553	1.07	-1.247982	2.96E-15	3.95E-15	2.9	2.3	2.46	1.3	1.21	0.7
OsR498G0306960600.01	6.55	21.923	1.7401759	9.29E-109	4.40E-109	6.73	6.18	6.74	21.72	18.25	25.8
OsR498G0306963400.01	2.456	8.633	1.8088921	4.08E-140	1.61E-140	2.53	2.47	2.37	9.12	8.36	8.42
OsR498G0306965200.01	2.406	17.706	2.8790087	3.04E-101	1.51E-101	2.33	2.23	2.66	18.37	17.21	17.54
OsR498G0306966100.01	8.956	18.906	1.0638192	3.75E-28	3.89E-28	9.74	8.79	8.34	17.38	20.6	18.74
OsR498G0306966900.01	1.866	6.716	1.5220688	9.27E-48	7.31E-48	1.66	2.03	1.91	7.23	7.09	5.83
OsR498G0306967100.01	0.013	0.193	3.7817649	0.0001711	0.0003365	0	0.04	0	0.36	0.11	0.11
OsR498G0306970700.01	58.81	151.1	1.3607988	0	0	56.79	60.6	59.04	155.73	148.54	149.04
OsR498G0306971600.01	4.116	11.14	1.2215563	4.03E-28	4.19E-28	4.25	4.03	4.07	11.76	10.59	11.07
OsR498G0306978500.01	0.813	0.34	-1.240603	3.78E-05	7.14E-05	0.68	0.71	1.05	0.4	0.25	0.37
OsR498G0306980000.01	0.8	0.27	-1.332251	0.0001815	0.0003575	1.19	0.69	0.52	0.21	0.38	0.22
OsR498G0306980100.01	2.093	5.773	1.4554535	1.05E-49	8.10E-50	2.06	1.9	2.32	5.78	6.05	5.49
OsR498G0306980400.01	6.89	2.04	-1.831866	2.08E-64	1.38E-64	6.37	7.51	6.79	1.78	2.69	1.65
OsR498G0306981600.01	0.606	2.426	2.0032072	4.19E-28	4.36E-28	0.59	0.47	0.76	2.77	2.42	2.09
OsR498G0306985700.01	1.5	7.586	2.3378147	5.74E-64	3.83E-64	1.79	1.07	1.64	7.3	7.4	8.06
OsR498G0306990100.01	120.766	56.243	-1.10328	1.52E-203	4.49E-204	127.7	123.36	111.24	56.84	55.49	56.4
OsR498G0306994100.01	7.333	1.963	-1.897976	9.48E-138	3.80E-138	8.01	6.3	7.69	1.9	2.05	1.94
OsR498G0307000500.01	0.206	7.93	5.2474285	1.78E-55	1.29E-55	0.21	0.31	0.1	7.84	7.5	8.45
OsR498G0307000700.01	0.2	1.34	2.7399447	7.12E-07	1.24E-06	0.24	0.24	0.12	1.53	1.05	1.44
OsR498G0307001100.01	0.286	3.723	3.687128	5.42E-25	5.93E-25	0	0.67	0.19	3.2	4.21	3.76
OsR498G0307001600.01	0.606	4.756	2.9630946	1.60E-22	1.83E-22	0.57	0.91	0.34	3.68	5.11	5.48
OsR498G0307002000.01	7.373	15.88	1.063751	1.98E-52	1.48E-52	8.08	7.06	6.98	15.22	17.06	15.36
OsR498G0307002900.01	5.016	15.273	1.5447257	1.49E-157	5.43E-158	5.77	4.9	4.38	16.4	15.11	14.31
OsR498G0307003200.01	0.023	0.15	2.7817649	0.0008272	0.0017105	0	0	0.07	0.22	0.16	0.07
OsR498G0307007000.01	1.826	19.843	3.2755795	2.34E-272	5.35E-273	1.98	1.16	2.34	20.83	19.89	18.81
OsR498G0307008400.01	19.506	7.823	-1.457619	5.50E-96	2.85E-96	20.46	18.84	19.22	7.72	7.8	7.95
OsR498G0307008600.01	2.61	8.626	1.7006491	3.30E-135	1.34E-135	2.59	2.64	2.6	8.63	9.19	8.06
OsR498G0307008800.01	1.19	14.163	3.5715884	2.82E-115	1.28E-115	1.46	1.16	0.95	14.15	13.67	14.67
OsR498G0307011100.01	0.46	2.806	2.6138202	1.39E-36	1.26E-36	0.36	0.56	0.46	2.71	3.18	2.53
OsR498G0307011500.01	3619.05	1259.6	-1.524703	0	0	3679.7	3641.1	3536.4	1271.1	1277.3	1230.6
OsR498G0307014300.01	0.303	0.08	-1.910113	1.63E-06	2.88E-06	0.31	0.3	0.3	0.11	0.13	0
OsR498G0307019700.01	1.14	3.856	1.7565643	1.41E-21	1.65E-21	1.41	1.08	0.93	2.58	4.73	4.26
OsR498G0307020100.01	4.62	22.986	1.6873953	0	0	4.46	4.36	5.04	22.17	22.61	24.18
OsR498G0307022300.01	15.076	33.966	1.1934267	4.12E-168	1.42E-168	15.97	15.39	13.87	34.57	34.54	32.79
OsR498G0307023100.01	11.57	34.236	1.5575	1.09E-170	3.72E-171	11.72	10.02	12.97	32.21	32.03	38.47
OsR498G0307026600.01	8.873	2.576	-1.784779	8.44E-54	6.22E-54	8.94	9.22	8.46	2.25	2.92	2.56
OsR498G0307029700.01	16.3	63.696	1.9311278	0	0	17.93	14.74	16.23	60.06	62.3	68.73
OsR498G0307030100.01	0.006	0.13	4.5289988	3.31E-12	4.78E-12	0.02	0	0	0.17	0.12	0.1
OsR498G0307030400.01	58.716	148.26	1.3056925	0	0	58.47	58.84	58.84	147.35	147.55	149.88
OsR498G0307034000.01	35.27	104.86	1.5829593	0	0	34.31	35.8	35.7	108.32	102.87	103.4
OsR498G0307037100.01	1.04	10.95	3.392097	6.34E-102	3.14E-102	0.92	1.53	0.67	10.22	10.42	12.21
OsR498G0307041000.01	1.99	8.016	2.0850731	1.48E-121	6.49E-122	2.07	1.79	2.11	8.46	8.14	7.45
OsR498G0307041400.01	7.78	3.49	-1.158787	1.57E-33	1.50E-33	7.93	8.26	7.15	3.47	3.29	3.71

OsR498G0307041800.01	1.463	9.493	2.7159848	3.12E-126	1.34E-126	1.35	1.6	1.44	9.83	9.98	8.67
OsR498G0307042100.01	3.596	0.516	-3.02559	1.50E-27	1.58E-27	4.06	3.54	3.19	0.55	0.62	0.38
OsR498G0307044800.01	6.703	14.26	1.0859566	5.19E-30	5.22E-30	7.21	6.31	6.59	14.21	14.09	14.48
OsR498G0307045500.01	3.073	6.216	1.0073496	1.88E-67	1.21E-67	2.94	3.11	3.17	6.21	5.93	6.51
OsR498G0307047300.01	0.573	1.76	1.6182661	1.41E-07	2.39E-07	0.72	0.57	0.43	1.96	1.6	1.72
OsR498G0307047600.01	14.396	34.623	1.257476	3.87E-175	1.29E-175	15.49	13.15	14.55	33.69	36.29	33.89
OsR498G0307048300.01	7.69	22.503	1.4903457	2.90E-126	1.24E-126	7.71	7.67	7.69	24.27	22.1	21.14
OsR498G0307051700.01	4.96	0.513	-3.313456	3.06E-109	1.44E-109	5.34	5.04	4.5	0.4	0.57	0.57
OsR498G0307052200.01	4.15	8.36	1.015255	1.52E-47	1.20E-47	4.16	4.05	4.24	8.58	8.29	8.21
OsR498G0307055400.01	46.736	17.38	-1.438852	0	0	45.31	46.61	48.29	17.09	17.35	17.7
OsR498G0307056700.01	7.186	40.726	2.523249	1.71E-272	3.91E-273	7.19	7.84	6.53	38.55	42.13	41.5
OsR498G0307057300.01	13.66	3.503	-1.994624	3.29E-108	1.56E-108	12.85	14.14	13.99	3.7	2.82	3.99
OsR498G0307058800.01	75.513	15.243	-2.38938	0	0	74.94	77.29	74.31	14.53	15.93	15.27
OsR498G0307059100.01	357.163	27.743	-3.686591	0	0	365.75	346.13	359.61	27.82	28.3	27.11
OsR498G0307062800.01	0.356	1.07	1.579272	3.29E-09	5.21E-09	0.43	0.57	0.07	1.15	0.97	1.09
OsR498G0307063500.01	9.973	26.813	1.4170853	1.17E-76	7.01E-77	11.55	10.01	8.36	26.58	25.38	28.48
OsR498G0307063700.01	0.213	0.03	-3.469987	0.0004798	0.000972	0.1	0.1	0.44	0	0	0.09
OsR498G0307064700.01	8.036	31.683	1.9636179	1.66E-297	3.52E-298	8.57	8.58	6.96	31.94	32.11	31
OsR498G0307065100.01	4.293	11.916	1.4725844	7.65E-129	3.22E-129	4.5	3.98	4.4	11.57	12.47	11.71
OsR498G0307065300.01	47.5	130.55	1.4601839	0	0	48.5	50.02	43.98	133.73	130.52	127.41
OsR498G0307066700.01	7.283	3.17	-1.149272	5.33E-31	5.29E-31	7.72	7.11	7.02	3.04	3.23	3.24
OsR498G0307068000.01	1.336	0.61	-1.080038	0.0005852	0.0011936	1.41	1.37	1.23	0.19	1.07	0.57
OsR498G0307069700.01	36.156	130.19	1.8459326	0	0	34.24	37.38	36.85	129.67	127.99	132.91
OsR498G0307072300.01	0.32	2.433	2.9106526	3.95E-34	3.72E-34	0.38	0.3	0.28	2.58	2.02	2.7
OsR498G0307073400.01	85.973	18.373	-2.228173	0	0	82.84	89.49	85.59	18.8	17.37	18.95
OsR498G0307074100.01	0.02	0.153	3.1023868	5.33E-05	0.0001015	0.03	0.03	0	0.03	0.27	0.16
OsR498G0307076300.01	6.88	78.116	3.5053422	0	0	7.03	5.83	7.78	77.64	76.74	79.97
OsR498G0307077000.01	3.786	44.326	3.6425024	0	0	3.6	4.1	3.66	45.09	42.21	45.68
OsR498G0307078200.01	1.07	4.583	2.1246086	3.65E-91	1.96E-91	1.02	1.03	1.16	4.7	4.39	4.66
OsR498G0307078700.01	8.593	19.15	1.1590333	1.66E-124	7.19E-125	8.94	8.76	8.08	19.28	19.73	18.44
OsR498G0307081100.01	121.11	269.7	1.1554528	0	0	117.2	124.55	121.58	273.11	264.86	271.13
OsR498G0307081300.01	1.123	2.986	1.3580149	2.26E-15	3.01E-15	1.19	1.54	0.64	3.34	2.91	2.71
OsR498G0307084100.01	0.556	2.996	2.4246621	1.02E-32	9.85E-33	0.55	0.5	0.62	2.88	2.83	3.28
OsR498G0307084500.01	9.38	23.766	1.3396435	7.12E-142	2.79E-142	9.26	9.07	9.81	22.48	22.24	26.58
OsR498G0307085000.01	7.546	18.056	1.2773299	5.40E-104	2.64E-104	7.89	7.53	7.22	17.12	18.48	18.57
OsR498G0307085100.01	0.776	5.513	2.8341054	2.38E-67	1.53E-67	0.86	0.63	0.84	5.18	5.26	6.1
OsR498G0307086400.01	0.023	1.106	5.5593725	5.24E-13	7.38E-13	0.07	0	0	0.89	1.58	0.85
OsR498G0307086600.01	17.363	57.736	1.730509	0	0	17.34	17.09	17.66	58.44	56.65	58.12
OsR498G0307086900.01	0.096	0.736	2.6221082	2.55E-15	3.40E-15	0.08	0.13	0.08	0.56	1.04	0.61
OsR498G0307087600.01	0.486	3.763	2.9550446	7.65E-98	3.90E-98	0.47	0.39	0.6	3.27	3.72	4.3
OsR498G0307091500.01	1.806	0.623	-1.537489	2.12E-07	3.61E-07	1.98	1.4	2.04	0.55	0.55	0.77
OsR498G0307096300.01	6.236	0.746	-3.051125	3.01E-118	1.35E-118	6.54	6.77	5.4	0.53	1	0.71
OsR498G0307096800.01	51.166	115.63	1.1749116	0	0	52.27	49.92	51.31	113.65	111.76	121.47
OsR498G0307097600.01	14.43	39.623	1.4583293	0	0	14.43	14.28	14.58	40.38	40.27	38.22
OsR498G0307104900.01	425.7	134.2	-1.666814	0	0	430.15	424.22	422.73	131.43	132.15	139.03
OsR498G0307107300.01	132.24	409.66	1.6299346	0	0	129.38	134.81	132.53	420.81	392.87	415.29
OsR498G0307108800.01	161.1	376	1.2236561	0	0	162.5	164.8	156	375.01	387.53	365.47
OsR498G0307109100.01	0.093	0.38	1.8919478	2.52E-08	4.14E-08	0.11	0.09	0.08	0.36	0.33	0.45
OsR498G0307111200.01	0.75	0.26	-1.566158	5.29E-09	8.46E-09	0.64	0.73	0.88	0.1	0.32	0.36
OsR498G0307112000.01	34.486	109.21	1.6603954	0	0	35.14	35.65	32.67	107.85	110.57	109.22
OsR498G0307113100.01	6.066	19.553	1.6894231	1.19E-228	3.19E-229	6.12	6.2	5.88	20.25	20.57	17.84
OsR498G0307113800.01	3.013	25.123	3.1163954	0	0	3.38	2.66	3	25.44	24.02	25.91
OsR498G0307114400.01	0.21	0.026	-2.932481	0.0003821	0.0007687	0.17	0.29	0.17	0.04	0.04	0
OsR498G0307114600.01	1.266	0.486	-1.369831	2.48E-11	3.67E-11	1.53	1.13	1.14	0.47	0.62	0.37
OsR498G0307124500.01	4.783	20.1	2.0718758	3.07E-260	7.28E-261	4.84	4.63	4.88	19.69	20.78	19.83
OsR498G0307127400.01	3.043	0.776	-1.966511	6.60E-34	6.25E-34	3.31	3.09	2.73	0.6	0.71	1.02
OsR498G0307128600.01	21.643	77.81	1.8450097	1.00E-197	3.03E-198	22.26	19.45	23.22	70.89	72.71	89.83
OsR498G0307128800.01	2.95	13.17	2.1624288	1.45E-235	3.78E-236	3.49	2.85	2.51	13.36	13.76	12.39
OsR498G0307130100.01	4.49	16.063	1.8456609	8.52E-210	2.45E-210	4.48	4.73	4.26	15.73	16.4	16.06
OsR498G0307131300.01	4.58	0.126	-5.149918	4.68E-206	1.37E-206	4.55	4.57	4.62	0.07	0.22	0.09
OsR498G0307131800.01	2260.69	1083.4	-1.043675	0	0	2241.4	2252.7	2288	1098.6	1057.3	1094.3
OsR498G0307133000.01	113.336	53.736	-1.086882	0	0	114.66	113.93	111.42	53.05	56.66	51.5
OsR498G0307133300.01	7.566	2.753	-1.552905	2.31E-66	1.50E-66	7.58	6.78	8.34	2.51	2.82	2.93
OsR498G0307133800.01	0.06	0.326	2.3894475	0.0009846	0.0020515	0.06	0	0.12	0.61	0.18	0.19
OsR498G0307136500.01	0.263	1.003	1.9536514	1.09E-15	1.43E-15	0.27	0.27	0.25	0.93	1.23	0.85
OsR498G0307139600.01	0.166	0.553	1.7658233	1.13E-05	2.08E-05	0.19	0.23	0.08	0.77	0.44	0.45
OsR498G0307140500.01	7.243	31.993	2.1780181	9.86E-137	3.97E-137	7.67	6.74	7.32	32.09	31.95	31.94
OsR498G0307142500.01	0.523	6.153	3.5235717	7.40E-115	3.38E-115	0.53	0.46	0.58	5.76	6.51	6.19
OsR498G0307145300.01	6.826	18.303	1.2231197	3.16E-127	1.35E-127	6.87	6.63	6.98	17.55	19.27	18.09

OsR498G0307145600.01	1138.5	302.78	-1.913934	0	0	1156.9	1086.7	1172	291.64	283.97	332.73
OsR498G0307153200.01	0.946	2.463	1.3298906	7.26E-12	1.06E-11	0.85	1.14	0.85	2.85	2.53	2.01
OsR498G0307153400.01	1.936	12.733	2.7160356	5.45E-171	1.85E-171	1.7	2.24	1.87	12.11	14.02	12.07
OsR498G0307153700.01	0.26	0.826	1.8152114	5.62E-11	8.42E-11	0.43	0.23	0.12	0.68	0.89	0.91
OsR498G0307154200.01	49.133	11.38	-1.473942	3.72E-228	9.96E-229	51.53	47.2	48.67	11.19	12.19	10.76
OsR498G0307154400.01	14.606	67.973	2.2283877	2.58E-299	5.47E-300	14.01	14.69	15.12	60.6	65.66	77.66
OsR498G0307156300.01	3	0.176	-4.105317	1.17E-41	9.99E-42	3.45	2.36	3.19	0.24	0.1	0.19
OsR498G0307157700.01	249.876	61.306	-2.042253	0	0	244.25	253.99	251.39	59.74	61.8	62.38
OsR498G0307165000.01	8.693	19.29	1.1128567	7.34E-42	6.23E-42	8.52	8.85	8.71	19.12	18.86	19.89
OsR498G0307166900.01	20.55	60.02	1.5440291	0	0	19.52	22.25	19.88	63.16	59.26	57.64
OsR498G0307167200.01	31.283	82.293	1.3906623	0	0	30.98	32.35	30.52	81.85	86.46	78.57
OsR498G0307167400.01	9.876	24.873	1.3668138	2.27E-127	9.66E-128	8.86	10.05	10.72	25.05	25.17	24.4
OsR498G0307171400.01	10.24	2.95	-1.795998	4.11E-72	2.54E-72	9.86	10.15	10.71	3.23	3.02	2.6
OsR498G0307172700.01	1.54	8.923	2.5366102	1.82E-149	6.85E-150	1.59	1.42	1.61	8.8	9.25	8.72
OsR498G0307173400.01	0.993	9.56	3.2000598	6.57E-131	2.74E-131	0.91	0.98	1.09	8.68	9.37	10.63
OsR498G0307173600.01	5.796	24.386	2.0125451	1.16E-169	3.96E-170	6.04	5.47	5.88	23.65	23.68	25.83
OsR498G0307174800.01	29.33	14.69	-1.000228	3.38E-51	2.57E-51	28.95	28.26	30.78	12.13	15.63	16.31
OsR498G0307176300.01	11.12	4.246	-1.385701	8.96E-44	7.39E-44	10.13	10.39	12.84	4.36	3.71	4.67
OsR498G0307179100.01	0.486	1.92	1.97441	1.21E-06	2.13E-06	0.12	0.85	0.49	2.15	1.9	1.71
OsR498G0307182700.01	0.05	3.046	6.0689276	1.56E-49	1.20E-49	0.05	0.05	0.05	3.27	2.82	3.05
OsR498G0307183800.01	0.6	3.423	2.5097417	5.03E-111	2.35E-111	0.6	0.69	0.51	3.17	3.51	3.59
OsR498G0307184200.01	22.79	67.673	1.5686045	0	0	23.55	22.39	22.43	68.7	67.21	67.11
OsR498G0307188500.01	2.303	0.17	-3.773514	1.00E-12	1.42E-12	2.58	2.57	1.76	0.25	0.13	0.13
OsR498G0307189300.01	5.16	16.27	1.6517287	9.35E-124	4.04E-124	4.66	5.33	5.49	15.92	16.64	16.25
OsR498G0307189800.01	1.676	3.98	1.2627317	1.29E-23	1.45E-23	1.36	1.5	2.17	3.56	4.56	3.82
OsR498G0307190300.01	0.086	1.06	3.6182661	4.97E-26	5.36E-26	0.13	0.03	0.1	1	0.93	1.25
OsR498G0307191800.01	0.51	0.14	-1.758914	0.0008992	0.0018664	0.51	0.46	0.56	0.1	0.17	0.15
OsR498G0307195900.01	5.02	20.213	2.0072983	1.87E-89	1.01E-89	5.8	5.1	4.16	20.4	17.04	23.2
OsR498G0307196600.01	15.29	45.216	1.5600275	2.97E-163	1.05E-163	13.76	16.21	15.9	41.38	45.65	48.62
OsR498G0307197700.01	6.7	3.443	-1.183503	9.18E-46	7.40E-46	6.37	7.46	6.27	3.82	3.17	3.34
OsR498G0307198000.01	1.403	0.556	-1.340101	1.53E-07	2.59E-07	1.31	1.6	1.3	0.51	0.42	0.74
OsR498G0307199400.01	25.36	108.61	2.0982769	0	0	24.52	27.09	24.47	110.88	108.02	106.93
OsR498G0307207900.01	3.393	8.17	1.2606832	2.40E-62	1.62E-62	3.71	3.13	3.34	8.26	8.51	7.74
OsR498G0307209000.01	9.966	20.14	1.0645106	4.23E-60	2.92E-60	9.57	10.53	9.8	19.6	19.54	21.28
OsR498G0307210400.01	11.543	0.523	-4.461885	4.43E-195	1.36E-195	11.66	11.46	11.51	0.31	0.58	0.68
OsR498G0307216100.01	0.383	0.04	-2.832945	4.82E-05	9.16E-05	0.28	0.14	0.73	0	0	0.12
OsR498G0307216400.01	19.61	99.47	2.3413677	0	0	18.11	19.99	20.73	98.85	101.78	97.78
OsR498G0307217700.01	194.13	67.103	-1.530592	0	0	196.86	188.26	197.27	67.52	64.39	69.4
OsR498G0307218900.01	5.683	11.8	1.0538444	1.14E-28	1.18E-28	5.82	5.18	6.05	10.92	12.13	12.35
OsR498G0307219300.01	8.64	22.64	1.3653576	3.63E-136	1.47E-136	8.39	8.38	9.15	22.99	22.85	22.08
OsR498G0307220100.01	0.666	1.643	1.2906693	8.60E-09	1.39E-08	0.79	0.7	0.51	1.66	2.06	1.21
OsR498G0307220700.01	14.923	82.963	2.4729438	0	0	14.51	15.82	14.44	80.55	82.22	86.12
OsR498G0307220900.01	14.993	30.093	1.0056995	6.94E-143	2.70E-143	13.88	15.7	15.4	30.61	31.4	28.27
OsR498G0307221200.01	0.193	1.686	3.1586336	8.03E-46	6.46E-46	0.1	0.29	0.19	1.71	1.79	1.56
OsR498G0307221600.01	5.496	18.736	1.76753	2.21E-178	7.30E-179	5.36	5.66	5.47	18.65	17.38	20.18
OsR498G0307223700.01	0.543	3.1	2.5110094	5.70E-74	3.48E-74	0.56	0.53	0.54	3.11	3.17	3.02
OsR498G0307224100.01	0.726	2.096	1.5260524	4.81E-17	6.15E-17	0.81	0.83	0.54	2.48	1.83	1.98
OsR498G0307225500.01	4.556	10.643	1.2231904	2.70E-23	3.04E-23	5.09	4.29	4.29	10.42	10.9	10.61
OsR498G0307226200.01	0.243	5.23	4.4043978	1.09E-128	4.60E-129	0.3	0.16	0.27	5.16	5.19	5.34
OsR498G0307226800.01	2.256	14.61	2.5992414	2.70E-278	6.06E-279	2.26	2.37	2.14	14.93	14.49	14.41
OsR498G0307227200.01	0.456	1.033	1.1608231	8.68E-05	0.0001677	0.52	0.43	0.42	1.36	1.12	0.62
OsR498G0307229600.01	5.513	10.516	1.0753804	5.59E-209	1.61E-209	6.33	5.35	4.86	11.31	10.35	9.89
OsR498G0307230100.01	1.56	5.833	1.8886801	2.30E-25	2.50E-25	1.53	1.52	1.63	5.41	6.68	5.41
OsR498G0307230500.01	7.36	3.056	-1.267791	7.19E-26	7.76E-26	8.02	5.74	8.32	2.98	2.92	3.27
OsR498G0307231000.01	10.89	23.993	1.1374936	1.22E-89	6.61E-90	10.51	11.83	10.33	24.83	22.89	24.26
OsR498G0307231500.01	0.833	5.843	2.8079488	1.03E-44	8.40E-45	0.71	1.15	0.64	5.69	5.36	6.48
OsR498G0307232500.01	9.38	19.29	1.036537	1.30E-85	7.29E-86	9.41	9.26	9.47	20.06	19.01	18.8
OsR498G0307233600.01	17.673	36.993	1.0546729	3.26E-197	9.90E-198	18.02	17.6	17.4	37.05	38.11	35.82
OsR498G0307234400.01	5.436	21.123	1.9535894	8.08E-202	2.40E-202	5.65	5.5	5.16	20.94	22.36	20.07
OsR498G0307235100.01	295.446	120.98	-1.301109	0	0	298.04	293.12	295.18	118.96	119.13	124.86
OsR498G0307238800.01	0.55	1.62	1.4568027	4.43E-08	7.33E-08	0.58	0.66	0.41	1.32	2.08	1.46
OsR498G0307239100.01	162.053	341.68	1.0736845	0	0	166.59	164.82	154.75	340.35	338.44	346.26
OsR498G0307240900.01	2.286	0.326	-2.79913	1.96E-41	1.67E-41	2.05	2.37	2.44	0.5	0.26	0.22
OsR498G0307241700.01	0.023	0.203	2.8813006	0.000469	0.0009493	0	0	0.07	0.21	0.2	0.2
OsR498G0307242400.01	4.983	12.56	1.35039	1.12E-55	8.13E-56	4.98	4.73	5.24	12.43	13.11	12.14
OsR498G0307244300.01	11.703	74.266	2.6638516	0	0	11	11.48	12.63	70.31	74.45	78.04
OsR498G0307245800.01	0.086	1.226	4.0671671	6.51E-59	4.56E-59	0.11	0.05	0.1	1.46	1.39	0.83
OsR498G0307246200.01	23.776	66.336	1.47145	0	0	23.45	24.35	23.53	64.57	69.78	64.66
OsR498G0307247900.01	14.453	39.28	1.445769	0	0	14.19	15.04	14.13	39.04	39.39	39.41

OsR498G0307248900.01	0.256	0.013	-3.979786	2.69E-08	4.41E-08	0.15	0.2	0.42	0	0	0.04
OsR498G0307249400.01	5.84	31.176	2.4141095	0	0	6.16	6.25	5.11	29.94	32.55	31.04
OsR498G0307250200.01	1.293	5.746	2.1247062	2.63E-75	1.59E-75	1.11	1.47	1.3	5.56	5.69	5.99
OsR498G0307251300.01	0.49	4.573	3.2781907	3.63E-44	2.98E-44	0.53	0.47	0.47	4.69	4.79	4.24
OsR498G0307251700.01	0.803	0.086	-3.215415	6.97E-15	9.37E-15	0.83	0.76	0.82	0.13	0	0.13
OsR498G0307253200.01	14.536	7.376	-1.02056	6.00E-35	5.59E-35	14.8	14.93	13.88	7.55	7.27	7.31
OsR498G0307253800.01	10.733	21.886	1.02831	2.01E-110	9.45E-111	10.65	10.94	10.61	21.91	21.65	22.1
OsR498G0307254000.01	0.046	0.65	3.7817649	2.33E-07	3.97E-07	0	0.07	0.07	0.76	0.55	0.64
OsR498G0307258800.01	0.34	14.01	5.3631696	0	0	0.28	0.51	0.23	14.26	14.41	13.36
OsR498G0307266700.01	69.736	144.5	1.0502609	0	0	70.4	67.79	71.02	145.94	140.19	147.37
OsR498G0307267000.01	16.903	43.1	1.3474769	1.79E-297	3.82E-298	17.66	15.93	17.12	43.19	43.47	42.64
OsR498G0307267300.01	67.21	139.2	1.0495834	2.27E-286	4.99E-287	65.66	69.54	66.43	138.71	137.63	141.27
OsR498G0307268700.01	2.2	5.22	1.2406071	1.44E-11	2.12E-11	1.23	2.16	3.21	4.26	4.69	6.71
OsR498G0307270900.01	3.37	10.546	1.6219674	7.72E-55	5.63E-55	3.1	3.15	3.86	10.22	10.07	11.35
OsR498G0307272000.01	0.15	1.313	3.1157658	1.85E-27	1.95E-27	0.08	0.15	0.22	1.44	1.04	1.46
OsR498G0307272300.01	0.506	3.353	2.7231527	4.67E-63	3.14E-63	0.35	0.65	0.52	3.73	3.37	2.96
OsR498G0307273300.01	1.28	0.103	-3.804563	1.16E-32	1.12E-32	0.96	1.15	1.73	0.09	0.17	0.05
OsR498G0307273500.01	0.026	0.233	3.2598122	1.01E-08	1.64E-08	0	0.04	0.04	0.25	0.14	0.31
OsR498G0307274000.01	0.406	1.703	2.0524125	6.12E-10	9.48E-10	0.41	0.61	0.2	1.2	1.72	2.19
OsR498G0307275500.01	1.283	0.076	-4.127128	5.73E-24	6.38E-24	1.24	1.15	1.46	0.04	0.11	0.08
OsR498G0307278600.01	0.56	0.126	-2.163094	6.89E-07	1.20E-06	0.78	0.46	0.44	0.11	0.11	0.16
OsR498G0307279400.01	10.893	22.276	1.0010365	5.61E-78	3.32E-78	11.01	10.6	11.07	22.18	23.77	20.88
OsR498G0307279700.01	6.39	12.9	1.0121649	3.27E-32	3.18E-32	5.79	6.7	6.68	12.66	12.89	13.15
OsR498G0307283400.01	6.786	3.09	-1.129581	8.36E-21	9.88E-21	6.86	6.17	7.33	3.07	3.26	2.94
OsR498G0307284700.01	23.566	48.346	1.0401852	6.84E-114	3.15E-114	24.32	24.39	21.99	46.84	49.8	48.4
OsR498G0307285700.01	0.116	3.423	4.8813006	6.27E-36	5.77E-36	0	0.21	0.14	3.19	3.04	4.04
OsR498G0307286100.01	0.303	45.903	7.2690307	5.30E-209	1.53E-209	0	0.07	0.84	45.04	44.67	48
OsR498G0307286300.01	2.623	53.983	4.3617611	0	0	1.88	2.83	3.16	54.67	53.57	53.71
OsR498G0307286500.01	63.913	130.81	1.0227629	2.80E-249	6.93E-250	65.44	62.31	63.99	130.51	128.76	133.15
OsR498G0307287500.01	28.756	68.016	1.2422441	0	0	30.01	27.81	28.45	68.55	68.33	67.17
OsR498G0307288900.01	107.91	16.413	-2.718736	0	0	115.46	104.15	104.12	15.07	17.12	17.05
OsR498G0307290000.01	14.776	33.57	1.1610045	1.71E-193	5.26E-194	14.65	14.52	15.16	34.23	33.03	33.45
OsR498G0307291800.01	0.26	0.596	1.1968024	1.49E-06	2.62E-06	0.26	0.22	0.3	0.71	0.52	0.56
OsR498G0307292200.01	4.343	10.316	1.2404375	7.59E-42	6.44E-42	4.46	4.43	4.14	10.11	10.74	10.1
OsR498G0307292500.01	12.85	29.513	1.198949	9.14E-178	3.02E-178	13.41	12.68	12.46	30.02	31.45	27.07
OsR498G0307293900.01	2.19	4.656	1.092296	8.75E-33	8.42E-33	2.35	2.14	2.08	4.84	4.62	4.51
OsR498G0307294300.01	1.02	6.723	2.7859649	1.89E-54	1.38E-54	0.99	0.89	1.18	6.55	7.71	5.91
OsR498G0307294700.01	24.83	52.55	1.0742867	8.44E-176	2.81E-176	24.01	26.52	23.96	54.17	52.26	51.22
OsR498G0307298600.01	0.273	1.173	2.0947042	7.80E-24	8.72E-24	0.27	0.3	0.25	1.15	1.22	1.15
OsR498G0307299100.01	0.463	3.566	2.9412431	5.11E-99	2.59E-99	0.5	0.51	0.38	3.34	3.78	3.58
OsR498G0307299300.01	7.353	17.476	1.2753125	1.80E-64	1.19E-64	8.69	6.16	7.21	17.19	17.13	18.11
OsR498G0307301400.01	3.353	9.13	1.4512237	7.11E-24	7.94E-24	3.6	2.74	3.72	9.68	8.5	9.21
OsR498G0307301500.01	2.043	4.763	1.1434491	2.21E-20	2.63E-20	2.2	1.67	2.26	5.25	4.72	4.32
OsR498G0307301700.01	1.773	0.04	-5.486742	1.40E-43	1.16E-43	1.61	1.79	1.92	0.06	0	0.06
OsR498G0307302000.01	1.306	0	-5.09512	1.16E-05	2.13E-05	0.7	1.98	1.24	0	0	0
OsR498G0307302500.01	11.686	25.14	1.1024404	2.52E-184	8.06E-185	12.09	11.44	11.53	25.76	25.74	23.92
OsR498G0307302800.01	7.853	17.103	1.120973	2.95E-68	1.89E-68	7.32	8.42	7.82	18.1	16.13	17.08
OsR498G0307304400.01	3.25	1.523	-1.092989	1.26E-22	1.44E-22	3.16	3.81	2.78	1.34	1.67	1.56
OsR498G0307305000.01	0.013	0.263	4.4338416	1.43E-06	2.52E-06	0	0.04	0	0.36	0.21	0.22
OsR498G0307305400.01	2.036	6.79	1.7319229	4.45E-72	2.75E-72	2.12	2.09	1.9	6.22	6.49	7.66
OsR498G0307305700.01	111.396	450.22	2.0144364	0	0	109.78	110.61	113.8	453.37	444.42	452.87
OsR498G0307306000.01	6.66	22.1	1.7310127	5.15E-136	2.08E-136	5.91	6.85	7.22	22.24	22.77	21.29
OsR498G0307308200.01	16.193	33.25	1.059626	1.01E-114	4.61E-115	15.61	15.98	16.99	32.16	33.51	34.08
OsR498G0307311600.01	59.176	160.2	1.4355773	0	0	57.52	61.92	58.09	160.44	158.79	161.38
OsR498G0307312000.01	7.003	16.39	1.2248075	8.69E-54	6.40E-54	6.19	7.2	7.62	15.93	16.43	16.81
OsR498G0307312200.01	0.656	8.25	3.6518187	1.80E-86	1.00E-86	0.58	0.75	0.64	6.65	8.54	9.56
OsR498G0307315600.01	0.843	3.06	1.8878223	4.69E-39	4.13E-39	0.7	1.15	0.68	3.37	2.95	2.86
OsR498G0307319400.01	8.683	24.146	1.4710527	1.05E-81	6.04E-82	8.98	8.25	8.82	24.21	23.19	25.04
OsR498G0307321200.01	12.48	27.64	1.1500486	1.25E-187	3.95E-188	11.83	12.43	13.18	29.14	28.19	25.59
OsR498G0307323500.01	0.356	0.09	-2.02559	4.97E-06	8.99E-06	0.48	0.33	0.26	0.15	0.04	0.08
OsR498G0307323800.01	2.11	8.153	1.9366079	4.50E-69	2.85E-69	2.24	1.98	2.11	8.64	7.4	8.42
OsR498G0307330200.01	2.64	0.81	-1.705756	2.45E-08	4.01E-08	0.71	4.43	2.78	0.71	1.23	0.49
OsR498G0307330400.01	1.5	0.213	-2.883571	3.06E-25	3.34E-25	1.48	1.26	1.76	0.15	0.18	0.31
OsR498G0307330500.01	4.923	11.303	1.1875526	1.87E-44	1.53E-44	5.14	5.49	4.14	12.6	10.56	10.75
OsR498G0307330800.01	0.51	1.626	1.6678704	2.89E-07	4.94E-07	0.52	0.41	0.6	1.02	1.64	2.22
OsR498G0307332500.01	20.456	69.776	1.7682955	3.11E-277	7.00E-278	20.95	20.21	20.21	69.76	66.75	72.82
OsR498G0307333400.01	15.44	45.793	1.4632377	2.47E-194	7.56E-195	16.87	13.54	15.91	44.32	44.73	48.33
OsR498G0307333700.01	1.813	5.75	1.6647255	9.79E-25	1.08E-24	1.8	1.85	1.79	5.61	6.19	5.45
OsR498G0307335000.01	0.78	0.03	-4.590375	6.64E-18	8.30E-18	0.86	0.92	0.56	0.06	0.03	0

OsR498G0307337900.01	48.25	19.8	-1.279996	4.52E-295	9.68E-296	48.56	49.55	46.64	20.4	19.45	19.55
OsR498G0307338600.01	1.753	4.773	1.447163	1.05E-19	1.27E-19	2	2.03	1.23	4.01	4.49	5.82
OsR498G0307338700.01	45.396	19.246	-1.247667	0	0	46.27	46.14	43.78	19.37	19.03	19.34
OsR498G0307339700.01	2.196	135.04	5.9709904	0	0	1.96	2.48	2.15	132.86	127.16	145.1
OsR498G0307339900.01	0.036	3.506	6.5442656	1.18E-23	1.32E-23	0	0.11	0	3.19	4.06	3.27
OsR498G0307340300.01	0.056	0.296	2.3894475	0.0009846	0.0020515	0.11	0	0.06	0.27	0.11	0.51
OsR498G0307343400.01	0.163	0.646	1.97441	0.0003981	0.0008013	0.25	0.08	0.16	0.64	0.56	0.74
OsR498G0307344100.01	5.04	10.523	1.0333279	1.45E-05	2.68E-05	6.32	2.7	6.1	10.29	9.07	12.21
OsR498G0307344800.01	4.04	12.316	1.614068	2.50E-187	7.90E-188	4.04	4.11	3.97	12.05	12.96	11.94
OsR498G0307345500.01	0.126	0	-4.758944	9.29E-05	0.0001799	0	0.38	0	0	0	0
OsR498G0307346800.01	0.083	0.44	2.4218689	1.14E-09	1.78E-09	0.09	0.09	0.07	0.46	0.41	0.45
OsR498G0307348000.01	11.376	23.45	1.0413166	1.07E-40	9.22E-41	11.72	11.05	11.36	20.24	23.72	26.39
OsR498G0307348200.01	0.083	0.896	3.3621401	1.67E-30	1.67E-30	0.03	0.05	0.17	0.86	1.02	0.81
OsR498G0307348400.01	0.02	0.563	4.6748497	1.82E-13	2.53E-13	0.03	0.03	0	0.32	0.67	0.7
OsR498G0307348600.01	4.03	14.98	1.8941179	2.51E-94	1.31E-94	3.21	4.36	4.52	15.46	15.26	14.22
OsR498G0307353300.01	31.923	85.56	1.4201984	7.41E-282	1.65E-282	30.44	33.6	31.73	84.31	80.26	92.11
OsR498G0307354300.01	314.566	25.38	-3.593497	0	0	319.59	310.12	313.99	25.54	24.49	26.11
OsR498G0307355700.01	0.123	0.356	1.5453295	0.0003348	0.0006704	0.07	0.15	0.15	0.39	0.47	0.21
OsR498G0307356100.01	0.316	2.706	3.1595214	7.21E-35	6.72E-35	0.21	0.32	0.42	2.7	2.88	2.54
OsR498G0307356900.01	5.496	23.693	2.1001955	2.40E-285	5.30E-286	5.53	5.59	5.37	24.54	23.87	22.67
OsR498G0307357800.01	41.053	121.46	1.5588409	0	0	38.97	41	43.19	120.44	116.68	127.26
OsR498G0307358600.01	0.06	0.373	2.6264867	4.19E-08	6.93E-08	0.08	0.05	0.05	0.51	0.3	0.31
OsR498G0307359200.01	11.563	30.173	1.3002251	7.71E-240	1.98E-240	12.02	11.8	10.87	29.54	31.84	29.14
OsR498G0307365000.01	0.686	0.213	-1.328254	0.0001718	0.0003379	0.64	0.57	0.85	0.31	0.07	0.26
OsR498G0307366700.01	1.89	0.24	-2.899325	3.19E-25	3.48E-25	1.83	1.61	2.23	0.26	0.29	0.17
OsR498G0307367200.01	0.143	1.056	2.8748743	1.54E-19	1.86E-19	0.06	0.23	0.14	1.01	1.12	1.04
OsR498G0307367600.01	0.666	0.31	-1.084484	0.0008487	0.001757	0.52	0.83	0.65	0.48	0.19	0.26
OsR498G0307367700.01	15.926	33.353	1.0735574	4.50E-89	2.45E-89	14.42	17.44	15.92	34.49	31.48	34.09
OsR498G0307369100.01	5.556	45.92	3.0439706	0	0	5.91	5.32	5.44	44.67	44.87	48.22
OsR498G0307370400.01	0.113	0.866	2.97441	3.25E-16	4.23E-16	0.13	0.06	0.15	1	0.82	0.78
OsR498G0307370500.01	1.636	14.403	3.1357218	1.72E-259	4.08E-260	1.88	1.77	1.26	14.71	13.6	14.9
OsR498G0307371900.01	1.506	0.506	-1.566158	7.59E-14	1.05E-13	1.64	1.42	1.46	0.41	0.6	0.51
OsR498G0307373800.01	3.57	1.38	-1.370977	2.33E-11	3.44E-11	3.89	3.37	3.45	1.35	1.48	1.31
OsR498G0307374300.01	1.766	17.876	3.3448215	4.33E-150	1.62E-150	1.83	1.6	1.87	17.73	16.15	19.75
OsR498G0307374600.01	1.263	9.283	2.8619352	4.14E-147	1.57E-147	1.37	1.03	1.39	8.95	9.29	9.61
OsR498G0307376800.01	0.06	0.363	2.5593725	4.55E-05	8.61E-05	0	0.09	0.09	0.22	0.45	0.42
OsR498G0307377000.01	12.963	28.423	1.126413	4.06E-87	2.25E-87	12.66	13.57	12.66	27.92	28.54	28.81
OsR498G0307377600.01	0.983	2.94	1.5805889	4.60E-12	6.66E-12	1.38	0.98	0.59	2.81	3.25	2.76
OsR498G0307378800.01	0.72	1.483	1.0425815	1.93E-09	3.03E-09	0.84	0.72	0.6	1.7	1.49	1.26
OsR498G0307382300.01	5.636	12.32	1.1276562	2.58E-36	2.35E-36	5.42	5.42	6.07	12.75	12.08	12.13
OsR498G0307382600.01	1.116	3.493	1.6569278	1.16E-22	1.33E-22	1	1.2	1.15	3.22	3.25	4.01
OsR498G0307382800.01	44.113	107.91	1.2884111	0	0	42.66	44.18	45.5	107.02	99.69	117.01
OsR498G0307383000.01	18.953	55.596	1.5401581	0	0	18.18	19.29	19.39	58.52	56.02	52.25
OsR498G0307388900.01	3.913	0.13	-4.96105	2.21E-108	1.05E-108	3.82	4.34	3.58	0.13	0.23	0.03
OsR498G0307392200.01	1.593	0.296	-2.420815	6.31E-15	8.47E-15	1.79	1.35	1.64	0.29	0.34	0.26
OsR498G0307394300.01	0.6	0.08	-2.85848	6.34E-11	9.52E-11	0.64	0.72	0.44	0.06	0.12	0.06
OsR498G0307395900.01	1.903	0.806	-1.247982	0.0001094	0.0002125	2.56	1.83	1.32	1	0.4	1.02
OsR498G0307396400.01	347.61	122.22	-1.501292	0	0	352.03	344.39	346.41	125.64	121.92	119.11
OsR498G0307396600.01	0.576	0.02	-4.780478	5.56E-08	9.24E-08	0.93	0.33	0.47	0	0	0.06
OsR498G0307396700.01	5.756	0.856	-2.746573	1.70E-61	1.16E-61	5.4	5.75	6.12	1.17	0.47	0.93
OsR498G0307398100.01	5.54	11.206	1.0146471	5.16E-40	4.50E-40	5.14	5.84	5.64	11.54	10.76	11.32
OsR498G0307400000.01	6.026	13.633	1.1813195	1.91E-71	1.19E-71	6.57	5.61	5.9	13.21	14.82	12.87
OsR498G0307400600.01	11.116	29.15	1.3819036	2.15E-122	9.36E-123	10.22	11.24	11.89	29.03	28.8	29.62
OsR498G0307401100.01	9.096	21.723	1.2478812	5.52E-99	2.79E-99	8.45	9.58	9.26	21.86	22.45	20.86
OsR498G0307403900.01	1.3	0.55	-1.239715	3.79E-06	6.81E-06	1.55	1.14	1.21	0.77	0.32	0.56
OsR498G0407412900.01	11.72	5.58	-1.199689	1.39E-81	8.02E-82	11.84	11.24	12.08	5.43	6.2	5.11
OsR498G0407413200.01	0.36	0.12	-1.569911	0.0002493	0.000495	0.46	0.34	0.28	0.03	0.24	0.09
OsR498G0407414400.01	1.263	0.09	-3.554843	1.59E-26	1.70E-26	1.41	1.07	1.31	0.07	0.07	0.13
OsR498G0407417700.01	6.236	21.483	1.763879	2.72E-131	1.13E-131	4.9	6.85	6.96	22.4	20.59	21.46
OsR498G0407419600.01	8.463	27.856	1.7261168	7.60E-213	2.16E-213	8.96	8.51	7.92	28.96	27.01	27.6
OsR498G0407420000.01	43.826	98.926	1.1556173	0	0	44.33	44.8	42.35	96.61	101.51	98.66
OsR498G0407421200.01	0.303	1.303	2.0999408	5.08E-23	5.75E-23	0.31	0.25	0.35	1.19	1.35	1.37
OsR498G0407425700.01	0.366	0.013	-4.72603	1.01E-07	1.69E-07	0.34	0.34	0.42	0	0	0.04
OsR498G0407425900.01	1.786	5.523	1.6255046	1.86E-83	1.06E-83	2	1.88	1.48	5.7	5.56	5.31
OsR498G0407426700.01	7.243	45.863	2.661788	0	0	6.87	6.73	8.13	45.19	44.79	47.61
OsR498G0407428300.01	0.413	1.683	2.022773	2.24E-11	3.32E-11	0.28	0.45	0.51	1.65	1.31	2.09
OsR498G0407429000.01	51.526	127.52	1.2107336	0	0	49.3	51.82	53.46	128.7	124.69	129.17
OsR498G0407430700.01	9.496	19.57	1.023463	1.81E-62	1.22E-62	8.4	10.93	9.16	20.03	18.9	19.78
OsR498G0407430900.01	0.18	0	-4.370419	0.0006357	0.0013002	0.14	0.13	0.27	0	0	0

OsR498G0407435800.01	0.976	0.173	-2.485022	6.90E-11	1.04E-10	0.89	1.15	0.89	0.3	0.13	0.09
OsR498G0407442600.01	0.01	0.446	5.7557697	1.15E-14	1.56E-14	0.03	0	0	0.46	0.41	0.47
OsR498G0407447400.01	0.34	0.693	1.0288577	0.0009716	0.0020229	0.2	0.39	0.43	0.92	0.57	0.59
OsR498G0407448100.01	0.466	0.226	-1.02559	8.12E-05	0.0001563	0.51	0.42	0.47	0.22	0.2	0.26
OsR498G0407453200.01	2.73	6.516	1.2900971	5.70E-40	4.96E-40	3.01	2.46	2.72	6.34	6.5	6.71
OsR498G0407480900.01	0.833	0.093	-3.292377	6.88E-16	9.03E-16	0.88	0.81	0.81	0.1	0.11	0.07
OsR498G0407484100.01	1.983	4.873	1.2963381	2.01E-40	1.74E-40	2.09	1.98	1.88	5.03	4.82	4.77
OsR498G0407484900.01	16.776	34.033	1.0128046	1.24E-106	5.94E-107	17.07	15.86	17.4	33.2	33.33	35.57
OsR498G0407490300.01	16.613	0.253	-7.013495	0	0	16.4	16.85	16.59	0.11	0.22	0.43
OsR498G0407492700.01	18.503	1.303	-3.854382	0	0	17.72	19.47	18.32	1.29	1.25	1.37
OsR498G0407493000.01	1.563	3.283	1.0264062	6.46E-18	8.08E-18	2.01	1.27	1.41	3.11	3.73	3.01
OsR498G0407495300.01	12.686	0.01	-9.807024	2.12E-299	4.50E-300	13.17	11.86	13.03	0	0	0.03
OsR498G0407495500.01	3.436	0	-7.869951	2.25E-26	2.41E-26	4.69	3.31	2.31	0	0	0
OsR498G0407498300.01	0.153	1.45	3.2462442	2.52E-31	2.48E-31	0.15	0.13	0.18	1.29	1.45	1.61
OsR498G0407499700.01	0.026	0.133	2.4769103	0.0005775	0.0011774	0.05	0	0.03	0.23	0.12	0.05
OsR498G0407500300.01	0.083	0.856	3.3488453	1.70E-16	2.20E-16	0.08	0.03	0.14	0.89	0.78	0.9
OsR498G0407505200.01	0.05	0.273	2.5104629	1.06E-05	1.96E-05	0.06	0.06	0.03	0.28	0.25	0.29
OsR498G0407510000.01	0.12	0.02	-2.688555	0.0001499	0.0002937	0.1	0.11	0.15	0	0.06	0
OsR498G0407513700.01	4.083	12.226	1.5406452	1.82E-73	1.11E-73	3.99	3.79	4.47	12.05	12.66	11.97
OsR498G0407514500.01	0.056	0.003	-3.72603	0.0002637	0.0005248	0.08	0.05	0.04	0	0.01	0
OsR498G0407532600.01	0.216	0.67	1.6177128	1.44E-07	2.43E-07	0.11	0.44	0.1	0.78	0.13	1.1
OsR498G0407534800.01	0.543	0	-6.80695	1.60E-14	2.17E-14	0.48	0.53	0.62	0	0	0
OsR498G0407559800.01	11.82	24.376	1.0480073	1.16E-83	6.59E-84	11.5	12.05	11.91	25.63	24.23	23.27
OsR498G0407574100.01	0.17	0.036	-2.195515	0.0008316	0.0017206	0.11	0.26	0.14	0.03	0.08	0
OsR498G0407588200.01	0.146	0.013	-3.72603	0.0002637	0.0005248	0.17	0.1	0.17	0	0.04	0
OsR498G0407589100.01	0.833	1.756	1.0796842	8.01E-17	1.03E-16	0.82	0.9	0.78	1.83	1.86	1.58
OsR498G0407591100.01	1.39	0.4	-1.796649	3.63E-16	4.74E-16	1.15	1.53	1.49	0.44	0.41	0.35
OsR498G0407601700.01	1.916	0.766	-1.4591	1.42E-19	1.72E-19	2.18	1.87	1.7	0.82	0.75	0.73
OsR498G0407603300.01	7.93	17.74	1.1609315	4.75E-66	3.10E-66	8.68	7.78	7.33	17.04	18.5	17.68
OsR498G0407603600.01	13.346	33.483	1.3128336	1.17E-128	4.95E-129	12.68	14.13	13.23	34.31	34.01	32.13
OsR498G0407604600.01	126.203	23.313	-2.384887	0	0	125.02	125.62	127.97	22.81	23.94	23.19
OsR498G0407614400.01	1.996	7.966	1.9953189	3.35E-59	2.33E-59	2.02	1.7	2.27	7.84	7.43	8.63
OsR498G0407615000.01	0.186	2.036	3.4719096	8.19E-38	7.32E-38	0.42	0.07	0.07	1.71	2.03	2.37
OsR498G0407624000.01	1.126	2.526	1.1773062	3.29E-18	4.09E-18	1.16	1.12	1.1	2.37	2.62	2.59
OsR498G0407629800.01	1.78	0.01	-7.610553	9.39E-44	7.76E-44	1.71	1.82	1.81	0	0	0.03
OsR498G0407636100.01	0.266	0.026	-3.273518	3.60E-05	6.79E-05	0.17	0.25	0.38	0.04	0.04	0
OsR498G0407639200.01	0.29	0.013	-4.610553	3.31E-07	5.68E-07	0.47	0.25	0.15	0	0	0.04
OsR498G0407639800.01	3	0.523	-2.521743	1.12E-54	8.20E-55	3.02	3.1	2.88	0.51	0.37	0.69
OsR498G0407643700.01	0.37	1.306	1.8255466	1.05E-18	1.29E-18	0.24	0.41	0.46	1.33	1.31	1.28
OsR498G0407645200.01	0.036	0.256	2.7817649	0.0008272	0.0017105	0	0	0.11	0.27	0.22	0.28
OsR498G0407651000.01	23.126	72.783	1.646936	1.08E-285	2.38E-286	21.54	23.05	24.79	69.3	74.81	74.24
OsR498G0407654100.01	0.273	1.13	2.0500076	1.91E-37	1.71E-37	0.24	0.27	0.31	1.15	1.1	1.14
OsR498G0407661200.01	0.08	0.486	2.6468353	3.48E-09	5.52E-09	0.09	0.03	0.12	0.65	0.4	0.41
OsR498G0407666500.01	4.383	1.23	-1.817293	1.03E-26	1.10E-26	4.37	4.68	4.1	1.32	0.88	1.49
OsR498G0407668300.01	0.063	2.29	5.1681817	4.92E-44	4.05E-44	0.04	0.11	0.04	2.49	1.93	2.45
OsR498G0407673800.01	46.103	3.523	-3.708049	5.45E-305	1.14E-305	45.73	48.7	43.88	4.28	3.73	2.56
OsR498G0407674200.01	72.926	6.79	-3.425782	0	0	75.79	76.5	66.49	5.47	8.35	6.55
OsR498G0407681200.01	1.753	0.726	-1.269516	0.0003809	0.0007662	1.76	1.52	1.98	0.68	0.45	1.05
OsR498G0407682200.01	473.3	177.77	-1.357031	0	0	439.52	492.58	487.8	179.84	170.87	182.61
OsR498G0407683000.01	945.7	400.67	-1.240584	0	0	957.95	947.7	931.45	401.11	384.46	416.45
OsR498G0407696900.01	1.02	15.653	4.1999168	0	0	0.95	1.18	0.93	15.54	16.38	15.04
OsR498G0407701900.01	0.563	0.153	-1.848712	4.13E-06	7.43E-06	0.33	0.81	0.55	0.14	0.25	0.07
OsR498G0407706000.01	12.266	64.79	2.3990451	9.76E-217	2.72E-217	13.15	10.59	13.06	62.5	63.64	68.23
OsR498G0407706500.01	0.71	0.23	-1.674683	1.77E-07	3.00E-07	0.78	0.59	0.76	0.25	0.28	0.16
OsR498G0407712200.01	10.09	3.61	-1.607624	7.23E-58	5.12E-58	9.55	9.63	11.09	3.61	3.24	3.98
OsR498G0407716800.01	0.353	1.173	1.7216439	9.99E-18	1.26E-17	0.36	0.35	0.35	1.09	1.17	1.26
OsR498G0407718200.01	0.083	0.396	2.2800625	2.49E-09	3.93E-09	0.25	0	0	0.34	0.55	0.3
OsR498G0407718900.01	1.2	2.656	1.1712673	2.12E-20	2.53E-20	1.15	1.31	1.14	2.82	2.55	2.6
OsR498G0407719800.01	0.06	2.186	5.2128147	1.00E-36	9.09E-37	0.09	0.04	0.05	2.5	1.98	2.08
OsR498G0407720100.01	0.37	1.293	1.804485	1.52E-07	2.57E-07	0.31	0.31	0.49	1.44	1.08	1.36
OsR498G0407721400.01	0.226	11.063	5.631781	1.32E-201	3.92E-202	0.28	0.12	0.28	10.32	11.43	11.44
OsR498G0407721900.01	3.62	14.926	2.049961	3.11E-169	1.07E-169	3.96	3.4	3.5	15.35	15.4	14.03
OsR498G0407722200.01	0.076	0.54	2.3765084	1.68E-06	2.97E-06	0.03	0.1	0.1	0.25	0.43	0.94
OsR498G0407728500.01	5.266	0.456	-3.619115	2.40E-68	1.54E-68	4.96	5	5.84	0.42	0.38	0.57
OsR498G0407734200.01	1.05	10.78	3.3602636	0	0	1.28	0.97	0.9	10.9	10.89	10.55
OsR498G0407756000.01.	2.81	6.4	1.1898435	9.41E-45	7.67E-45	2.4	2.98	3.05	6.83	6.62	5.75
OsR498G0407764200.01	319.303	11.426	-4.805542	0	0	329.61	317.04	311.26	11.45	11.92	10.91
OsR498G0407764600.01	157.336	4.326	-5.187615	0	0	172.37	153.03	146.61	4.18	3.73	5.07
OsR498G0407771500.01	1.52	10.336	2.7667887	4.19E-89	2.28E-89	1.6	1.48	1.48	11.52	10.57	8.92

OsR498G0407771700.01	2.13	18.053	3.0818335	2.16E-112	1.01E-112	1.78	2.43	2.18	19.97	17.51	16.68
OsR498G0407771900.01	0.03	0.406	3.7292975	4.20E-07	7.23E-07	0.09	0	0	0.45	0.4	0.37
OsR498G0407772300.01	1.39	17.146	3.6299705	0	0	1.44	1.45	1.28	17.75	17.52	16.17
OsR498G0407772900.01	0	0.18	4.2992206	0.0007853	0.0016183	0	0	0	0.25	0.12	0.17
OsR498G0407773100.01	5.09	92.6	4.1851239	0	0	5.17	4.82	5.28	93.4	93.99	90.41
OsR498G0407774900.01	0.853	2.743	1.6874807	4.16E-51	3.16E-51	0.83	0.89	0.84	2.69	2.66	2.88
OsR498G0407776100.01	1.33	5.103	1.8876983	1.89E-88	1.03E-88	1.34	1.29	1.36	4.76	5.4	5.15
OsR498G0407777300.01	19.466	9.76	-1.027889	8.48E-51	6.47E-51	18.68	19.25	20.47	11.15	9.25	8.88
OsR498G0407787000.01	0.113	0.546	2.2598122	3.10E-11	4.60E-11	0.15	0.08	0.11	0.71	0.5	0.43
OsR498G0407801400.01	2.193	0.276	-3.366627	4.04E-30	4.07E-30	2.25	2.17	2.16	0.28	0.34	0.21
OsR498G0407806800.01	0	0.443	4.7630957	7.98E-05	0.0001536	0	0	0	0.19	0.65	0.49
OsR498G0407814900.01	2.426	5.55	1.1386852	5.02E-22	5.79E-22	2.58	2.95	1.75	6.6	4.8	5.25
OsR498G0407815300.01	4.013	8.26	1.0601858	1.10E-27	1.15E-27	4.18	3.95	3.91	8.98	7.8	8
OsR498G0407816200.01	106.7	237.2	1.1524083	0	0	110.47	107.92	101.71	230.09	241.74	239.77
OsR498G0407829800.01	100.793	43.78	-1.211421	0	0	100.87	100.16	101.35	43.92	43.26	44.16
OsR498G0407830500.01	9.35	2.41	-1.946023	1.15E-80	6.70E-81	8.85	9.97	9.23	2.83	2.11	2.29
OsR498G0407830900.01	4.22	17.27	2.03238	9.01E-112	4.19E-112	3.85	4.67	4.14	17.09	16.81	17.91
OsR498G0407841500.01	2887.41	791.59	-1.869148	0	0	2855.4	2822.1	2984.8	760.87	749.1	864.79
OsR498G0407860000.01	13.163	33.176	1.3386001	1.30E-149	4.88E-150	8.02	17.31	14.16	31.27	35.9	32.36
OsR498G0407862700.01	0	0.74	6.1908648	1.95E-10	2.96E-10	0	0	0	0.36	0.59	1.27
OsR498G0407867300.01	0.206	0.93	1.933768	3.47E-05	6.52E-05	0.34	0.2	0.08	1.03	0.76	1
OsR498G0407886600.01	0.013	0.21	3.9762122	5.08E-05	9.66E-05	0	0.04	0	0.23	0.12	0.28
OsR498G0407891700.01	0.093	0.793	3.103693	9.83E-14	1.36E-13	0	0.07	0.21	0.91	0.74	0.73
OsR498G0407896300.01	2.93	13.61	2.2317978	1.99E-56	1.43E-56	3.19	3.06	2.54	11.96	15.04	13.83
OsR498G0407897300.01	50.953	19.126	-1.402158	0	0	50.06	50.55	52.25	19.21	19.57	18.6
OsR498G0407900000.01	8.443	22.79	1.4252034	3.09E-95	1.61E-95	8.6	8.04	8.69	22.67	23.02	22.68
OsR498G0407902900.01	43.3	101.35	1.1053477	0	0	41.82	44.25	43.83	98.95	106.73	98.36
OsR498G0407906700.01	0.836	0.39	-1.095979	2.70E-06	4.82E-06	1.2	0.5	0.81	0.35	0.51	0.31
OsR498G0407923500.01	0.29	0.023	-3.610553	0.0004877	0.0009887	0.14	0.35	0.38	0	0	0.07
OsR498G0407926900.01	0.883	5.113	2.5463163	2.10E-32	2.04E-32	0.28	1.31	1.06	5.21	3.98	6.15
OsR498G0407932400.01	49.313	21.72	-1.18458	5.35E-92	2.85E-92	48.72	48.41	50.81	22.37	20.31	22.48
OsR498G0407935100.01	0.226	0.75	1.7292975	0.0005682	0.0011565	0.17	0.34	0.17	0.75	0.99	0.51
OsR498G0407941200.01	1.18	2.423	1.0345309	2.20E-09	3.47E-09	1.55	0.97	1.02	2.26	2.74	2.27
OsR498G0407949900.01	20.03	59.606	1.5707598	1.38E-293	2.97E-294	21.1	18.36	20.63	57.5	56.39	64.93
OsR498G0407951200.01	0.24	3.23	3.7200553	1.51E-78	8.91E-79	0.2	0.15	0.37	3.12	3.21	3.36
OsR498G0407953300.01	3.043	8.26	1.3736109	1.70E-38	1.51E-38	3.38	2.87	2.88	7.85	9.71	7.22
OsR498G0407961500.01	7.31	2.16	-1.756949	9.39E-42	7.98E-42	7.72	6.57	7.64	1.66	2.54	2.28
OsR498G0407974200.01	19.15	57.876	1.590637	0	0	18.66	18.73	20.06	56.31	58.83	58.49
OsR498G0407993000.01	0.236	1.65	2.7954398	4.06E-18	5.06E-18	0.19	0.19	0.33	1.99	1.25	1.71
OsR498G0407995000.01	4.91	18.006	1.8483745	3.11E-179	1.02E-179	4.78	4.85	5.1	19.75	16.99	17.28
OsR498G0408000400.01	0.173	0.403	1.2281665	7.11E-05	0.0001366	0.14	0.16	0.22	0.54	0.33	0.34
OsR498G0408002900.01	11.303	26.646	1.2360677	3.90E-88	2.14E-88	11.56	11.11	11.24	26.3	25.44	28.2
OsR498G0408003400.01	1.423	3.243	1.1888536	1.40E-10	2.13E-10	1.27	1.91	1.09	3.42	3.24	3.07
OsR498G0408009100.01	0.67	0.196	-1.786402	2.72E-07	4.65E-07	0.83	0.69	0.49	0.13	0.26	0.2
OsR498G0408014500.01	32.5	154.04	2.2503762	0	0	32.64	32.9	31.96	157.99	157.29	146.84
OsR498G0408019300.01	4.486	23.363	2.4219484	0	0	4.97	4.08	4.41	22.71	25	22.38
OsR498G0408023500.01	718.426	239.46	-1.585521	0	0	724.57	716.49	714.22	248.37	230.2	239.81
OsR498G0408031700.01	18.99	2.14	-3.151699	4.77E-161	1.71E-161	18.23	20.19	18.55	2.02	2.07	2.33
OsR498G0408034000.01	0.14	0.463	1.6833612	9.54E-09	1.54E-08	0.08	0.16	0.18	0.47	0.45	0.47
OsR498G0408050900.01	0.116	0.77	2.9279018	1.59E-12	2.27E-12	0.07	0.13	0.15	0.59	1.15	0.57
OsR498G0408052500.01	0.31	0.87	1.4550027	1.91E-06	3.38E-06	0.25	0.43	0.25	0.7	0.64	1.27
OsR498G0408081100.01	1.733	19.103	3.4108272	0	0	1.78	1.61	1.81	19.59	18.16	19.56
OsR498G0408091200.01	1.383	0.09	-3.932481	7.73E-05	0.0001487	1.38	1.39	1.38	0	0.27	0
OsR498G0408115100.01	0.016	0.14	3.9406558	6.41E-05	0.0001228	0	0.05	0	0.31	0.11	0
OsR498G0408139600.01	0.786	1.856	1.2020208	9.11E-10	1.42E-09	0.81	0.74	0.81	2.36	1.82	1.39
OsR498G0408142400.01	53.646	18.26	-1.556232	2.32E-202	6.88E-203	51.21	53.88	55.85	17.81	18.26	18.71
OsR498G0408147600.01	14.54	6.5	-1.187966	2.22E-47	1.76E-47	15.52	13.84	14.26	6.61	6	6.89
OsR498G0408168600.01	7.76	2.2	-1.74938	6.73E-69	4.28E-69	7.66	7.02	8.6	2.2	2.5	1.9
OsR498G0408171100.01	99.683	374.29	1.9051015	0	0	103.89	96.14	99.02	374.39	368.67	379.8
OsR498G0408171400.01	0	0.043	4.4338416	0.0004331	0.0008746	0	0	0	0.01	0.06	0.06
OsR498G0408175300.01	1.173	2.953	1.3127325	7.77E-12	1.13E-11	1.15	1.34	1.03	3.72	2.29	2.85
OsR498G0408175900.01	0.026	0.74	4.7023304	1.02E-13	1.41E-13	0	0	0.08	0.58	0.87	0.77
OsR498G0408197000.01	1.223	0.283	-2.100931	2.05E-19	2.49E-19	1.11	1.2	1.36	0.31	0.4	0.14
OsR498G0408211500.01	0.633	0.063	-3.485022	1.87E-14	2.54E-14	0.64	0.79	0.47	0.08	0.07	0.04
OsR498G0408211800.01	0.223	2.933	3.9076253	2.61E-47	2.07E-47	0.22	0.15	0.3	3.27	3.09	2.44
OsR498G0408212000.01	0.356	9.966	5.2213885	3.68E-163	1.31E-163	0.48	0.35	0.24	9.26	10.96	9.68
OsR498G0408213900.01	22.993	65.326	1.4929477	2.55E-191	7.92E-192	21.92	22.62	24.44	61.69	59.89	74.4
OsR498G0408216300.01	2.516	10.02	2.002364	1.85E-50	1.41E-50	3.27	2.07	2.21	9.27	9.53	11.26
OsR498G0408219900.01	6.933	16.23	1.2266176	5.20E-122	2.27E-122	6.93	7.1	6.77	16.05	17.2	15.44

OsR498G0408220300.01	5.203	1.776	-1.671779	4.12E-91	2.21E-91	4.91	5.56	5.14	1.88	1.88	1.57
OsR498G0408221600.01	0.51	1.576	1.6399909	4.76E-09	7.60E-09	0.37	0.88	0.28	1.53	1.37	1.83
OsR498G0408225600.01	49.683	121.93	1.2939096	0	0	49.53	52.69	46.83	124.39	119.57	121.83
OsR498G0408236200.01	5.58	2.56	-1.111141	4.56E-31	4.51E-31	5.94	5.26	5.54	2.55	2.32	2.81
OsR498G0408238800.01	0.173	1.156	2.7169137	8.46E-15	1.14E-14	0.12	0.12	0.28	1.03	1.18	1.26
OsR498G0408255600.01	0.093	0.2	1.0249697	0.0004757	0.0009632	0.09	0.1	0.09	0.15	0.2	0.25
OsR498G0408258100.01	0	0.08	4.5593725	0.0002368	0.0004696	0	0	0	0.13	0.06	0.05
OsR498G0408266700.01	0.503	0.03	-4.069984	8.08E-09	1.30E-08	0.74	0.27	0.5	0	0.04	0.05
OsR498G0408272500.01	0.4	0.086	-2.227224	0.000178	0.0003504	0.37	0.52	0.31	0.05	0.1	0.11
OsR498G0408305400.01	1.21	3.406	1.4710859	1.07E-15	1.41E-15	1.48	0.89	1.26	3.16	4.16	2.9
OsR498G0408312400.01	18.263	0.326	-5.809134	1.34E-158	4.85E-159	18.57	17.36	18.86	0.49	0.32	0.17
OsR498G0408332200.01	1.36	2.876	1.0799682	1.45E-10	2.20E-10	0.88	1.39	1.81	2.56	3.2	2.87
OsR498G0408332800.01	0.56	0.146	-1.943128	3.61E-05	6.79E-05	0.5	0.49	0.69	0.15	0.14	0.15
OsR498G0408336800.01	0.166	4.446	4.7369106	1.42E-44	1.16E-44	0.22	0.21	0.07	3.97	4.79	4.58
OsR498G0408337400.01	2.643	0.386	-2.776361	3.95E-56	2.84E-56	2.51	2.75	2.67	0.31	0.4	0.45
OsR498G0408339000.01	4.44	22.186	2.2963381	1.14E-217	3.15E-218	4.86	4.14	4.32	22.64	22.61	21.31
OsR498G0408339100.01	2.29	32.92	3.8572585	0	0	2.37	2.11	2.39	32.77	33.72	32.27
OsR498G0408346900.01	0.843	1.673	1.0109358	8.52E-09	1.37E-08	0.8	0.89	0.84	1.96	1.45	1.61
OsR498G0408358300.01	14.643	96.786	2.7378283	0	0	14.39	14.15	15.39	97.18	96.29	96.89
OsR498G0408361700.01	4.02	9.153	1.1896779	7.97E-51	6.08E-51	4.43	3.7	3.93	8.66	9.13	9.67
OsR498G0408366000.01	1.46	0.196	-2.904736	1.40E-18	1.73E-18	1.37	1.65	1.36	0.12	0.21	0.26
OsR498G0408373000.01	5.466	27.72	2.3404109	9.18E-161	3.29E-161	4.98	5.27	6.15	28.02	25.17	29.97
OsR498G0408376600.01	12.67	35.83	1.5027693	1.13E-197	3.41E-198	12.74	13.08	12.19	36.37	34.52	36.6
OsR498G0408382700.01	1.823	5.376	1.5593725	3.46E-81	2.01E-81	1.6	2.2	1.67	5.47	5.76	4.9
OsR498G0408386100.01	1.856	13.31	2.8426376	1.13E-205	3.32E-206	2.1	1.76	1.71	13.92	13.28	12.73
OsR498G0408395400.01	0.876	2.3	1.3823301	1.69E-22	1.93E-22	0.85	0.9	0.88	2.38	1.98	2.54
OsR498G0408397700.01	27.766	13.49	-1.040484	5.74E-87	3.18E-87	28.61	26.41	28.28	13.83	13.44	13.2
OsR498G0408412400.01	0.303	0.853	1.4952421	2.68E-06	4.77E-06	0.4	0.27	0.24	0.81	0.84	0.91
OsR498G0408424900.01	17.976	5.076	-1.824121	4.88E-108	2.32E-108	19.48	16.23	18.22	4.65	5.32	5.26
OsR498G0408425900.01	11.523	1.463	-2.975217	2.47E-67	1.59E-67	12.6	10.24	11.73	1.8	1.19	1.4
OsR498G0408430000.01	2.946	4.73	1.1150083	4.49E-14	6.15E-14	3.93	2.58	2.33	4.39	4.98	4.82
OsR498G0408441700.01	1.103	2.68	1.2936389	1.30E-16	1.67E-16	1.41	1.05	0.85	2.04	2.62	3.38
OsR498G0408448900.01	0.05	0.523	3.3436438	2.11E-07	3.59E-07	0	0	0.15	0.63	0.54	0.4
OsR498G0408449100.01	16.836	41.073	1.2885449	4.68E-212	1.33E-212	20.2	13.89	16.42	42.55	39.51	41.16
OsR498G0408450800.01	0.096	0.753	2.9019831	1.07E-09	1.67E-09	0	0	0.29	0	0	2.26
OsR498G0408451300.01	23.273	61.446	1.4073164	0	0	22.54	23.37	23.91	61.18	60.4	62.76
OsR498G0408451500.01	12.033	0.266	-5.164354	3.33E-109	1.57E-109	13.68	11.62	10.8	0.34	0.11	0.35
OsR498G0408452400.01	0.673	4.15	2.5977188	2.25E-88	1.24E-88	0.67	0.76	0.59	3.84	4.12	4.49
OsR498G0408454800.01	1.016	2.036	1.0029791	1.76E-05	3.26E-05	1.29	0.79	0.97	2.08	1.6	2.43
OsR498G0408461900.01	22.383	62.146	1.4616953	1.33E-177	4.40E-178	20.91	24.09	22.15	61.35	57.89	67.2
OsR498G0408462600.01	3.3	0.953	-1.788551	3.64E-22	4.18E-22	3.64	3.52	2.74	1.38	0.64	0.84
OsR498G0408465300.01	0.196	1.193	2.6124838	2.02E-37	1.82E-37	0.21	0.16	0.22	1.21	1.12	1.25
OsR498G0408469900.01	18.996	72.883	1.9265058	1.21E-246	3.05E-247	18.1	18.3	20.59	68.32	66.38	83.95
OsR498G0408470900.01	1.03	24.886	4.4474796	0	0	0.88	1.41	0.8	25.24	24.5	24.92
OsR498G0408473800.01	15.116	0.413	-5.188426	1.04E-144	4.01E-145	15.19	14.77	15.39	0.29	0.58	0.37
OsR498G0408477200.01	0.433	0	-5.610553	1.82E-07	3.08E-07	0.34	0.39	0.57	0	0	0
OsR498G0408481900.01	1.09	13.006	3.5561195	0	0	1.13	0.96	1.18	12.7	12.75	13.57
OsR498G0408484600.01	0.016	0.253	4.0333036	3.17E-12	4.57E-12	0	0.02	0.03	0.2	0.36	0.2
OsR498G0408488100.01	5.393	11.47	1.0855889	3.22E-18	4.01E-18	5.03	5.71	5.44	11.79	10.18	12.44
OsR498G0408492100.01	11.413	4.746	-1.268183	1.43E-51	1.08E-51	10.21	13.43	10.6	4.69	4.31	5.24
OsR498G0408502400.01	3.396	12.736	1.8978421	2.06E-181	6.69E-182	3.29	3.5	3.4	13.19	13.22	11.8
OsR498G0408511100.01	1.68	6.883	2.0320753	7.49E-64	5.01E-64	1.26	1.53	2.25	6.27	6.49	7.89
OsR498G0408516600.01	0.23	0.02	-3.642653	0.0004133	0.0008331	0.25	0.16	0.28	0	0	0.06
OsR498G0408520300.01	1.27	6	2.2619865	5.42E-42	4.59E-42	0.83	1.33	1.65	6.52	6.08	5.4
OsR498G0408520500.01	0.04	0.39	3.2474285	6.71E-07	1.17E-06	0	0.04	0.08	0.44	0.28	0.45
OsR498G0408521900.01	0.043	1.02	4.5593725	1.85E-12	2.65E-12	0	0.13	0	0.95	1.07	1.04
OsR498G0408524500.01	0.816	0.233	-1.793075	1.06E-07	1.78E-07	0.89	0.71	0.85	0.26	0.35	0.09
OsR498G0408525300.01	49.97	125.8	1.3313639	0	0	51.96	49.01	48.94	122.03	126.99	128.39
OsR498G0408525900.01	9.223	20.356	1.1030244	1.30E-36	1.18E-36	9.67	7.99	10.01	20.49	19.8	20.78
OsR498G0408527700.01	86.523	24.196	-1.84801	0	0	85.44	84.38	89.75	22.97	24.55	25.07
OsR498G0408530800.01	8.586	59.083	2.7788995	0	0	8.44	8.9	8.42	58.17	60.52	58.56
OsR498G0408532700.01	0.613	1.476	1.1880054	4.85E-07	8.38E-07	0.65	0.38	0.81	1.71	1.19	1.53
OsR498G0408535900.01	3.723	12.4	1.8175246	3.41E-50	2.61E-50	4.17	3.48	3.52	12.63	10.88	13.69
OsR498G0408536200.01	0.05	0.493	3.3894475	1.18E-07	1.99E-07	0.05	0.05	0.05	0.64	0.37	0.47
OsR498G0408537300.01	3476.8	540.6	-2.68622	0	0	3467.4	3510	3453	534.36	528.3	559.15
OsR498G0408537500.01	0.496	1.406	1.4698209	1.78E-15	2.35E-15	0.47	0.6	0.42	1.33	1.37	1.52
OsR498G0408538100.01	9.726	20.043	1.0414339	2.12E-36	1.93E-36	9.79	9.5	9.89	20.15	18.57	21.41
OsR498G0408540100.01	3.083	10.546	1.7708766	3.12E-48	2.45E-48	3.06	3.09	3.1	10.11	10.54	10.99
OsR498G0408541500.01	2.883	1.166	-1.30799	6.42E-07	1.12E-06	2.32	2.78	3.55	1.03	1.22	1.25

OsR498G0408545200.01	1.386	0.24	-2.514975	5.39E-12	7.82E-12	1.49	1.36	1.31	0.11	0.44	0.17
OsR498G0408545900.01	2.06	26.883	3.7140058	0	0	2.36	1.83	1.99	26.57	27.54	26.54
OsR498G0408550000.01	0.323	0.853	1.4032533	0.0007247	0.0014903	0.22	0.3	0.45	1.09	0.58	0.89
OsR498G0408553000.01	1.16	0.1	-3.569911	1.31E-08	2.13E-08	1.4	0.89	1.19	0.1	0.1	0.1
OsR498G0408554900.01	2.79	10.656	1.8529902	1.82E-78	1.08E-78	2.83	2.65	2.89	10.12	10.2	11.65
OsR498G0408558000.01	0	0.646	5.97441	2.98E-09	4.72E-09	0	0	0	0.54	0.6	0.8
OsR498G0408560500.01	103.56	28.713	-1.804721	0	0	99.99	107.83	102.86	29.23	28.8	28.11
OsR498G0408561300.01	8.423	2.606	-1.702548	1.80E-89	9.75E-90	8.2	8.3	8.77	3	2.57	2.25
OsR498G0408563300.01	7.48	2.59	-1.529975	3.61E-85	2.03E-85	7.72	7.55	7.17	2.38	2.82	2.57
OsR498G0408569200.01	29.406	8.043	-1.913827	0	0	28.79	29.84	29.59	8.29	7.94	7.9
OsR498G0408584200.01	0	1.116	8.566867	1.69E-39	1.48E-39	0	0	0	1.1	1.06	1.19
OsR498G0408584400.01	4.573	116.93	4.5796545	0	0	4.99	4.42	4.31	118.32	118.14	114.34
OsR498G0408584900.01	8.436	27.543	1.7032226	1.21E-270	2.77E-271	8.82	8.69	7.8	28.15	28.18	26.3
OsR498G0408585700.01	20.263	87.51	2.1081631	0	0	21.55	19.88	19.36	86.05	86.75	89.73
OsR498G0408587500.01	0.336	8.516	4.6326214	2.64E-204	7.78E-205	0.27	0.22	0.52	7.53	8.71	9.31
OsR498G0408589700.01	14.376	35.93	1.3204014	0	0	13.66	14.8	14.67	36.55	36.13	35.11
OsR498G0408595100.01	0.25	1.436	2.5989008	1.31E-17	1.65E-17	0.22	0.32	0.21	1.47	1.3	1.54
OsR498G0408599000.01	5.39	12.753	1.2422938	1.01E-63	6.77E-64	5.48	5.72	4.97	13.29	13.17	11.8
OsR498G0408601300.01	1.216	0.366	-1.745482	1.15E-06	2.01E-06	1.24	1.04	1.37	0.32	0.19	0.59
OsR498G0408603200.01	0.316	0.933	1.5312214	2.48E-16	3.22E-16	0.22	0.3	0.43	0.9	1.06	0.84
OsR498G0408604400.01	3.026	9.32	1.6121351	2.46E-85	1.38E-85	2.84	3.03	3.21	9.04	9.04	9.88
OsR498G0408606300.01	0.836	0.353	-1.247982	0.0006539	0.0013392	0.98	0.92	0.61	0.12	0.51	0.43
OsR498G0408606900.01	0.466	12.573	4.7648416	5.21E-307	1.08E-307	0.63	0.37	0.4	12.6	11.7	13.42
OsR498G0408608400.01	4.423	19.263	2.1188551	5.72E-155	2.10E-155	4.3	4.07	4.9	20.25	18.9	18.64
OsR498G0408612600.01	0.253	1.263	2.3114449	1.09E-25	1.18E-25	0.26	0.24	0.26	1.63	1.17	0.99
OsR498G0408614800.01	0.266	0.013	-4.273518	6.73E-06	1.22E-05	0.34	0.25	0.21	0	0.04	0
OsR498G0408617000.01	1.316	4.256	1.6874375	3.99E-23	4.51E-23	1.16	1.4	1.39	3.55	4.93	4.29
OsR498G0408617100.01	0.143	0	-4.898419	4.14E-05	7.82E-05	0.21	0	0.22	0	0	0
OsR498G0408632900.01	86.273	33.876	-1.345998	0	0	87.11	86.61	85.1	35.42	33.93	32.28
OsR498G0408633800.01	3.64	3.076	-1.457564	1.54E-19	1.86E-19	3.36	2.89	4.67	3.54	3.31	2.38
OsR498G0408637300.01	3.95	1.436	-1.446728	5.83E-23	6.61E-23	3.84	4.13	3.88	1.3	1.38	1.63
OsR498G0408638800.01	0.633	1.46	1.2047076	0.0001002	0.0001943	0.66	0.73	0.51	1.78	1.14	1.46
OsR498G0408639600.01	3.63	9.016	1.2845783	1.27E-38	1.12E-38	3.61	3.14	4.14	9.63	8.64	8.78
OsR498G0408645400.01	1.72	3.603	1.0295929	3.03E-19	3.69E-19	1.68	1.46	2.02	3.36	3.64	3.81
OsR498G0408648400.01	0.033	0.403	3.7292975	4.20E-07	7.23E-07	0.05	0.05	0	0.35	0.31	0.55
OsR498G0408649100.01	77.043	15.61	-2.278571	0	0	76.32	79.85	74.96	15.95	14.73	16.15
OsR498G0408651600.01	0.2	2.833	3.8173888	1.79E-59	1.24E-59	0.15	0.21	0.24	2.79	2.66	3.05
OsR498G0408655500.01	2.073	7.28	1.7972534	3.48E-73	2.14E-73	1.87	2.71	1.64	7.41	7.25	7.18
OsR498G0408658500.01	3.09	12.623	2.027487	3.01E-223	8.18E-224	3.25	2.87	3.15	11.88	12.47	13.52
OsR498G0408660100.01	1.493	168.45	6.8241225	0	0	1.52	1.2	1.76	169.46	164.07	171.82
OsR498G0408661100.01	0.266	0.08	-1.780478	0.0004075	0.0008212	0.27	0.32	0.21	0.12	0.09	0.03
OsR498G0408661900.01	2.02	0.34	-2.585462	1.93E-26	2.07E-26	2.07	2.04	1.95	0.31	0.21	0.5
OsR498G0408662700.01	1.576	8.246	2.3840272	2.29E-104	1.12E-104	1.3	1.88	1.55	8.65	7.48	8.61
OsR498G0408663900.01	0.66	4.713	2.7165189	1.65E-85	9.23E-86	0.8	0.35	0.83	4.98	4.6	4.56
OsR498G0408665600.01	0.866	8.426	3.2545179	1.27E-182	4.11E-183	0.87	0.86	0.87	8.52	8.83	7.93
OsR498G0408668500.01	0.17	1.253	2.8813006	2.59E-11	3.84E-11	0.32	0.13	0.06	1.18	1.05	1.53
OsR498G0408670900.01	0.416	1.55	1.8519259	1.32E-16	1.71E-16	0.45	0.31	0.49	1.94	1.37	1.34
OsR498G0408671100.01	0.37	3.993	3.4199694	3.74E-40	3.25E-40	0.59	0.29	0.23	4.42	4.02	3.54
OsR498G0408672700.01	1.02	3.08	1.5983666	6.03E-19	7.38E-19	1.05	0.92	1.09	2.94	3.41	2.89
OsR498G0408673500.01	0.416	1.05	1.328947	2.56E-07	4.37E-07	0.28	0.45	0.52	0.97	0.88	1.3
OsR498G0408673700.01	0.496	2.79	2.4916856	6.95E-33	6.67E-33	0.45	0.26	0.78	2.87	2.85	2.65
OsR498G0408675200.01	20.326	4.746	-2.100669	3.60E-57	2.57E-57	18.87	21.39	20.72	4.84	4.58	4.82
OsR498G0408675800.01	27.803	150.32	2.4448981	0	0	29.86	27.1	26.45	153.6	148.69	148.68
OsR498G0408685600.01	1.07	0.53	-1.068659	0.000159	0.0003121	1.09	1.19	0.93	0.25	0.46	0.88
OsR498G0408685800.01	2.953	0.613	-2.264377	2.20E-16	2.85E-16	3.24	2.62	3	0.66	0.58	0.6
OsR498G0408688400.01	459.37	67.176	-2.7747	0	0	470.46	439.7	467.95	67.06	64.22	70.25
OsR498G0408689800.01	5.226	1.993	-1.4182	7.69E-42	6.52E-42	5.09	4.84	5.75	2.17	1.59	2.22
OsR498G0408690200.01	30.343	119.85	1.9803622	0	0	30.28	30.68	30.07	119.24	122.18	118.13
OsR498G0408692700.01	0.01	0.183	4.144335	1.55E-05	2.86E-05	0	0.03	0	0.15	0.37	0.03
OsR498G0408693600.01	0.003	0.116	4.6748497	1.36E-07	2.29E-07	0	0.01	0	0.14	0.08	0.13
OsR498G0408696100.01	2610.66	882.39	-1.566813	0	0	2546.6	2530.3	2755.1	853.36	828.75	965.05
OsR498G0408696400.01	12.5	28.76	1.2084012	9.04E-91	4.86E-91	13.16	13.13	11.21	30.31	27.79	28.18
OsR498G0408696800.01	0.89	5.743	2.676866	2.46E-54	1.80E-54	0.78	0.91	0.98	6.13	5.35	5.75
OsR498G0408697000.01	1.126	2.546	1.2382908	1.81E-19	2.19E-19	1.13	1.09	1.16	2.6	2.53	2.51
OsR498G0408699700.01	0.046	25.443	9.2068309	0	0	0.08	0	0.06	26.5	23.93	25.9
OsR498G0408700100.01	0	0.22	4.2963381	0.0007942	0.0016388	0	0	0	0.13	0.13	0.4
OsR498G0408700300.01	6.18	14.886	1.2639166	4.98E-64	3.32E-64	6.8	5.16	6.58	13.99	15.1	15.57
OsR498G0408701300.01	0.6	3.963	2.694022	1.39E-46	1.11E-46	0.59	0.66	0.55	3.62	4.27	4
OsR498G0408707400.01	0.893	1.463	1.0557101	1.29E-07	2.17E-07	0.51	1.16	1.01	2.07	1.07	1.25

OsR498G0408708600.01	14.863	42.296	1.6000432	1.43E-217	3.96E-218	13.97	15.9	14.72	42.48	42.23	42.18
OsR498G0408709700.01	45.303	156.32	1.7816878	0	0	45.06	46.28	44.57	161.05	154.87	153.03
OsR498G0408710000.01	1.53	4.263	1.4748389	1.64E-17	2.07E-17	1.69	1.56	1.34	4.49	4.09	4.21
OsR498G0408710200.01	0.393	1.723	2.1357602	7.01E-11	1.05E-10	0.46	0.52	0.2	1.78	1.33	2.06
OsR498G0408710300.01	0.233	1.213	2.3743406	5.27E-14	7.23E-14	0.16	0.23	0.31	1.29	0.91	1.44
OsR498G0408710600.01	5.756	20.44	1.8492682	5.58E-117	2.51E-117	4.97	5.6	6.7	18.97	21.13	21.22
OsR498G0408711400.01	72.783	19.186	-1.925604	1.82E-258	4.33E-259	74.85	75.08	68.42	17.55	19.13	20.88
OsR498G0408712400.01	21.753	9.566	-1.187773	4.79E-54	3.52E-54	22.32	21.14	21.8	10.76	7.39	10.55
OsR498G0408712800.01	10.616	39.79	1.9047491	2.99E-221	8.17E-222	10.1	11.44	10.31	40.72	39.74	38.91
OsR498G0408718100.01	5.84	1.703	-1.788105	6.13E-18	7.66E-18	9.32	3.68	4.52	1.39	0.71	3.01
OsR498G0408719400.01	2.723	10.093	1.9147507	1.12E-163	3.97E-164	2.91	2.9	2.36	9.99	10.18	10.11
OsR498G0408720200.01	1.073	3.083	1.5174002	6.10E-21	7.19E-21	1.08	1.1	1.04	3.11	2.9	3.24
OsR498G0408726700.01	54.103	109.72	1.0124077	0	0	54.65	55.47	52.19	108.58	112.15	108.42
OsR498G0408727000.01	0.523	2.85	2.4603547	1.57E-95	8.13E-96	0.51	0.44	0.62	2.89	2.92	2.74
OsR498G0408732400.01	22.466	8.146	-1.435874	7.28E-22	8.43E-22	24.82	20.3	22.28	8.11	7.7	8.63
OsR498G0408734000.01	0.693	10.453	3.8255911	1.90E-129	7.96E-130	0.76	0.69	0.63	9.85	9.67	11.84
OsR498G0408735400.01	28.273	132.86	2.230897	0	0	26.28	29.08	29.46	136.16	130.35	132.07
OsR498G0408737900.01	2.996	8.766	1.5487252	1.30E-60	8.97E-61	3.29	2.63	3.07	8.77	9.21	8.32
OsR498G0408739900.01	18.25	60.673	1.7574581	0	0	19.57	18.66	16.52	60.97	60.24	60.81
OsR498G0408741800.01	7.706	2.793	-1.536654	2.99E-53	2.22E-53	7.71	7.62	7.79	2.67	2.81	2.9
OsR498G0408746400.01	0.376	0.066	-2.213217	1.13E-06	1.99E-06	0.33	0.52	0.28	0.04	0.1	0.06
OsR498G0408748500.01	0.25	0.056	-2.466163	6.07E-07	1.05E-06	0.24	0.31	0.2	0.08	0.05	0.04
OsR498G0408749700.01	0.173	0.31	1.6009789	0.000871	0.0018049	0.29	0.06	0.17	0.35	0.23	0.35
OsR498G0408753000.01	7.303	43.94	2.5902099	0	0	7.32	6.16	8.43	45.4	42.91	43.51
OsR498G0408755300.01	2.066	4.766	1.1759356	1.58E-14	2.15E-14	2.22	2.28	1.7	4.77	4.4	5.13
OsR498G0408756000.01	2.11	13.586	2.1273071	1.61E-189	5.04E-190	1.99	2.47	1.87	12.41	14.86	13.49
OsR498G0408756800.01	3.51	11.653	1.7149481	1.17E-103	5.72E-104	2.84	4.05	3.64	11.77	11.19	12
OsR498G0408760000.01	1.02	2.786	1.4483411	2.81E-18	3.49E-18	0.92	1.12	1.02	3.02	2.61	2.73
OsR498G0408760600.01	0.413	1.596	1.8919478	1.26E-11	1.85E-11	0.33	0.44	0.47	1.72	1.76	1.31
OsR498G0408762000.01	6.543	17.116	1.3903295	3.76E-170	1.28E-170	6.53	6.43	6.67	17.02	17.32	17.01
OsR498G0408763100.01	1.066	7.3	2.7528522	1.99E-76	1.19E-76	1.09	0.9	1.21	7.39	7.47	7.04
OsR498G0408763700.01	0.02	0.11	2.5593725	0.0003356	0.0006725	0.04	0.02	0	0.11	0.09	0.13
OsR498G0408765200.01	1.046	5.45	2.4163541	8.25E-61	5.66E-61	1	0.86	1.28	5.35	5.61	5.39
OsR498G0408767400.01	6.23	15.16	1.3847648	2.83E-158	1.02E-158	6.48	6.27	5.94	14.65	14.82	16.01
OsR498G0408770900.01	1.26	0.333	-1.912496	5.34E-25	5.84E-25	1.11	1.29	1.38	0.36	0.35	0.29
OsR498G0408772500.01	0.476	0.02	-4.6403	5.89E-13	8.31E-13	0.41	0.58	0.44	0	0	0.06
OsR498G0408773400.01	1.87	5.003	1.4202097	2.39E-67	1.54E-67	1.95	1.76	1.9	5.56	5.19	4.26
OsR498G0408777000.01	70.12	216.31	1.6234143	0	0	69.69	71.52	69.15	212.1	203.67	233.17
OsR498G0408780600.01	0.923	5.016	2.450848	6.83E-36	6.28E-36	1.02	1.16	0.59	4.7	5.5	4.85
OsR498G0408783400.01	1.493	4.523	1.5832192	1.47E-47	1.16E-47	1.6	1.37	1.51	4.77	4.35	4.45
OsR498G0408787400.01	1.576	3.42	1.1003189	1.08E-16	1.39E-16	1.91	1.5	1.32	3.4	3.24	3.62
OsR498G0408787500.01	11.28	37.353	1.7246048	3.76E-258	8.97E-259	11.06	11.33	11.45	39.1	36.15	36.81
OsR498G0408790300.01	0.246	1.933	2.9501624	5.65E-21	6.66E-21	0.31	0.15	0.28	1.95	2.2	1.65
OsR498G0408793100.01	0.043	0.423	3.4708358	1.23E-16	1.59E-16	0.05	0.03	0.05	0.59	0.32	0.36
OsR498G0408793600.01	0.053	0.526	3.4338416	4.96E-12	7.19E-12	0.09	0.03	0.04	0.66	0.55	0.37
OsR498G0408795200.01	6.51	18.62	1.5156851	4.56E-230	1.21E-230	5.52	7.1	6.91	18.3	19.35	18.21
OsR498G0408795300.01	1.663	3.956	1.3042547	1.57E-28	1.62E-28	2.28	1.21	1.5	4.82	3.08	3.97
OsR498G0408795600.01	1.826	8.01	2.1301592	2.45E-64	1.63E-64	1.78	1.75	1.95	7.71	8.05	8.27
OsR498G0408795800.01	0.1	0.41	2.0524125	8.30E-06	1.52E-05	0.07	0.03	0.2	0.38	0.32	0.53
OsR498G0408799300.01	0.3	1.436	2.2485849	7.89E-27	8.38E-27	0.29	0.4	0.21	1.26	1.74	1.31
OsR498G0408800700.01	3.343	0.293	-3.511017	3.61E-34	3.40E-34	4.02	3.21	2.8	0.58	0.12	0.18
OsR498G0408806900.01	0.036	0.406	3.5593725	1.15E-08	1.87E-08	0.04	0.07	0	0.4	0.2	0.62
OsR498G0408809200.01	0.133	0.483	1.832391	0.0002304	0.0004563	0.2	0.05	0.15	0.3	0.69	0.46
OsR498G0408809700.01	17.126	36.53	1.1741015	1.23E-147	4.66E-148	17.33	16.34	17.71	34.99	36.18	38.42
OsR498G0408810000.01	8.013	19.526	1.3449939	7.19E-117	3.24E-117	8.41	8.09	7.54	19.14	20.95	18.49
OsR498G0408816100.01	1.193	5.056	2.0055005	4.45E-104	2.17E-104	1.01	1	1.57	5.07	5.82	4.28
OsR498G0408817100.01	19.51	83.16	2.0673487	0	0	20.65	19.74	18.14	83.75	85.67	80.06
OsR498G0408817300.01	1.096	3.956	1.9016286	1.83E-93	9.61E-94	1.07	1	1.22	4.43	4.07	3.37
OsR498G0408817700.01	7.243	17.88	1.3017976	5.58E-61	3.83E-61	7.25	7.84	6.64	16.73	17.71	19.2
OsR498G0408820400.01	13.153	103.19	2.9890663	0	0	13.32	13.86	12.28	102.92	103.6	103.04
OsR498G0408821200.01	2.513	6.993	1.4288704	1.66E-34	1.56E-34	2.34	2.4	2.8	6.27	7.42	7.29
OsR498G0408826800.01	0.236	0.996	2.0954254	2.22E-11	3.28E-11	0.39	0.11	0.21	1.02	0.91	1.06
OsR498G0408830200.01	8.45	21.74	1.3232726	1.20E-193	3.69E-194	8.44	8.43	8.48	21.94	22.2	21.08
OsR498G0408832700.01	169.31	67.67	-1.32794	0	0	172.99	158.37	176.57	67.02	62.69	73.3
OsR498G0408834200.01	16.376	8.153	-1.058065	4.30E-35	4.00E-35	16.26	15.6	17.27	8.31	8.16	7.99
OsR498G0408834700.01	0.516	1.03	1.0637574	2.09E-06	3.70E-06	0.62	0.54	0.39	1.28	0.91	0.9
OsR498G0408837800.01	0.293	0.496	2.4979719	7.77E-05	0.0001495	0.07	0.27	0.54	0.45	0.51	0.53
OsR498G0408838500.01	0.183	0.813	2.167055	2.13E-05	3.97E-05	0.16	0.31	0.08	1.14	0.83	0.47
OsR498G0408839800.01	0.2	5.696	4.9698945	1.20E-74	7.29E-75	0.21	0.21	0.18	5.02	6.38	5.69

OsR498G0408841600.01	46.773	156.85	1.7405722	0	0	48.36	46.34	45.62	155.65	152.36	162.55
OsR498G0408845300.01	5.79	11.786	1.039744	1.36E-99	6.86E-100	6.32	5.14	5.91	12.16	12.25	10.95
OsR498G0408845600.01	23.703	54.773	1.208298	1.20E-118	5.35E-119	23.59	25.65	21.87	54.4	56.8	53.12
OsR498G0408846900.01	58.306	158.77	1.4948352	0	0	59.97	59.32	55.63	155.82	165.06	155.43
OsR498G0408848300.01	7.893	24.736	1.6704493	6.03E-207	1.76E-207	8.33	8.8	6.55	24.93	25	24.28
OsR498G0408848700.01	11.726	28.933	1.2952807	2.11E-77	1.25E-77	10.97	12.99	11.22	28.69	28.49	29.62
OsR498G0408850300.01	29.173	63.393	1.0545654	8.82E-222	2.41E-222	28.85	29.27	29.4	63.28	65.52	61.38
OsR498G0408852600.01	55.3	25.69	-1.102168	3.01E-57	2.15E-57	57.92	51.89	56.09	26.29	23.51	27.27
OsR498G0408852800.01	17.363	66.79	1.9329125	0	0	16.95	17.77	17.37	68.93	66.16	65.28
OsR498G0408857000.01	2.543	8.256	1.7015119	5.93E-139	2.36E-139	2.78	2.43	2.42	8.33	8.35	8.09
OsR498G0408860500.01	0.126	0.456	1.8488791	8.72E-05	0.0001686	0.17	0	0.21	0.25	0.41	0.71
OsR498G0408861800.01	0.763	2.716	1.8257659	4.02E-49	3.12E-49	0.83	0.65	0.81	2.91	2.49	2.75
OsR498G0408865500.01	33.77	83.936	1.3386504	4.66E-160	1.68E-160	33.67	34.72	32.92	88.05	83.05	80.71
OsR498G0408866100.01	129.893	298.1	1.1939352	0	0	128.9	130.27	130.51	302.33	292.11	299.87
OsR498G0408870000.01	1.413	3.316	1.1968024	7.34E-15	9.88E-15	1.79	1.24	1.21	3.07	3.6	3.28
OsR498G0408872400.01	4.95	12.136	1.3001694	2.66E-201	7.92E-202	5.23	5.16	4.46	12.22	13.07	11.12
OsR498G0408876800.01	2.21	8.003	1.8504776	4.21E-114	1.94E-114	2.42	2.35	1.86	8.11	8.17	7.73
OsR498G0408877600.01	0.75	1.826	1.286354	1.07E-09	1.67E-09	0.74	0.66	0.85	1.89	2.11	1.48
OsR498G0408878400.01	0.1	1.663	4.0898872	1.12E-23	1.26E-23	0	0.15	0.15	1.94	1.46	1.59
OsR498G0408881200.01	0.416	1.896	2.1868596	8.16E-30	8.23E-30	0.36	0.41	0.48	1.66	2.17	1.86
OsR498G0408883400.01	1.296	5.32	1.7358545	5.25E-56	3.79E-56	1.07	1.26	1.56	5.55	4.93	5.48
OsR498G0408883800.01	0.786	0.323	-1.195515	2.62E-05	4.91E-05	0.66	0.82	0.88	0.45	0.23	0.29
OsR498G0408884400.01	8.176	1.166	-2.824259	1.90E-132	7.88E-133	8.82	8.3	7.41	1.25	1.07	1.18
OsR498G0408885400.01	1.67	5.88	1.8946309	1.93E-59	1.34E-59	2.11	1.47	1.43	5.7	6.09	5.85
OsR498G0408886600.01	1.056	98.13	6.5337064	0	0	0.83	1.08	1.26	98.45	101.88	94.06
OsR498G0408886800.01	0.023	0.7	4.832391	5.71E-15	7.66E-15	0.07	0	0	0.79	0.68	0.63
OsR498G0408890800.01	34.023	94.613	1.4704579	1.97E-276	4.45E-277	32.5	36.92	32.65	92.63	94.03	97.18
OsR498G0408897800.01	117.163	397.11	1.7493585	0	0	117.69	117.76	116.04	392.31	398.9	400.12
OsR498G0408898700.01	0.16	0.97	2.5891198	1.01E-08	1.64E-08	0.24	0.18	0.06	1.06	0.94	0.91
OsR498G0408899800.01	0.48	5.433	3.3997158	4.85E-71	3.03E-71	0.6	0.37	0.47	5.22	5.6	5.48
OsR498G0408901100.01	14.93	34.096	1.1764824	4.41E-174	1.48E-174	14.73	15.2	14.86	34.47	34.53	33.29
OsR498G0408901300.01	0.86	0.016	-5.72603	3.53E-14	4.83E-14	0.95	0.69	0.94	0	0	0.05
OsR498G0408903800.01	8.93	20.91	1.2160474	4.59E-75	2.78E-75	8.76	8.42	9.61	20.75	21.36	20.62
OsR498G0408904400.01	0.083	0.58	3.4841102	2.77E-14	3.78E-14	0.17	0	0.08	0.15	1.25	0.34
OsR498G0408907400.01	2.363	4.84	1.0100339	1.25E-18	1.55E-18	2.38	2.45	2.26	4.85	4.82	4.85
OsR498G0408907700.01	4.066	0.046	-5.845769	1.10E-28	1.13E-28	3.33	4.31	4.56	0	0.07	0.07
OsR498G0408911500.01	1.556	4.83	1.6332546	4.41E-52	3.32E-52	1.73	1.38	1.56	4.76	4.72	5.01
OsR498G0408913200.01	8.483	20.736	1.2862766	4.09E-213	1.16E-213	8.43	8.22	8.8	20.44	21.07	20.7
OsR498G0408913800.01	1.656	3.806	1.1685825	1.46E-14	1.97E-14	1.82	1.72	1.43	4.17	3.67	3.58
OsR498G0408916400.01	5.486	1.7	-1.690854	3.50E-28	3.64E-28	6.69	5.06	4.71	1.69	1.78	1.63
OsR498G0408919400.01	0.353	32.1	6.5042309	0	0	0.38	0.51	0.17	30.53	29.79	35.98
OsR498G0408921900.01	1.303	0.63	-1.04629	5.01E-06	9.06E-06	1.3	1.36	1.25	0.59	0.58	0.72
OsR498G0408923500.01	0.083	0.94	3.3939488	1.57E-21	1.83E-21	0.03	0.15	0.07	1.11	1.09	0.62
OsR498G0408924200.01	0.723	2.18	1.5869847	1.03E-17	1.29E-17	0.63	0.94	0.6	1.9	2.05	2.59
OsR498G0408924600.01	1.26	12.753	3.3355086	1.45E-204	4.25E-205	1.36	1.17	1.25	11.48	12.64	14.14
OsR498G0408927000.01	1.97	49.356	4.674017	0	0	1.76	2	2.15	50.45	49.83	47.79
OsR498G0408929200.01	0.27	0	-5.72603	5.93E-08	9.87E-08	0.28	0.31	0.22	0	0	0
OsR498G0408931100.01	1.153	2.573	1.1537337	6.62E-09	1.06E-08	1.48	0.81	1.17	2.53	2.17	3.02
OsR498G0408932400.01	3.576	1.46	-1.288624	1.87E-06	3.31E-06	3.59	3.7	3.44	1.87	1.49	1.02
OsR498G0408935400.01	1.943	0.566	-1.771833	4.02E-11	5.99E-11	1.92	2.51	1.4	0.49	0.65	0.56
OsR498G0408935800.01	2.963	0.426	-2.824332	3.14E-28	3.25E-28	3.37	2.49	3.03	0.28	0.41	0.59
OsR498G0408938700.01	9.593	43.47	2.1905161	0	0	10.17	9.48	9.13	44.08	44.14	42.19
OsR498G0408939000.01	4.156	9.4	1.1659168	7.33E-36	6.75E-36	4.39	3.61	4.47	9.04	8.99	10.17
OsR498G0408939100.01	3.383	30.593	3.156362	2.39E-230	6.34E-231	3.91	3.4	2.84	31.72	30.97	29.09
OsR498G0408939300.01	1.913	7.476	1.9628033	2.48E-64	1.65E-64	1.81	2.28	1.65	7.68	7.57	7.18
OsR498G0408940100.01	18.356	38.836	1.005304	4.14E-54	3.04E-54	18.52	19.39	17.16	39.63	34.01	42.87
OsR498G0408941200.01	0.616	1.776	1.4468238	4.24E-11	6.33E-11	0.91	0.42	0.52	1.96	1.58	1.79
OsR498G0408941400.01	1.806	0.646	-1.478102	2.60E-05	4.87E-05	1.15	2.08	2.19	1.02	0.61	0.31
OsR498G0408950400.01	1.523	23.136	4.0083202	0	0	1.82	1.42	1.33	22.63	23.45	23.33
OsR498G0408954500.01	0.216	0.03	-2.832945	4.82E-05	9.16E-05	0.06	0.37	0.22	0	0.06	0.03
OsR498G0408956900.01	132.346	28.223	-2.172598	0	0	132.32	131.4	133.32	29.16	27.74	27.77
OsR498G0408957100.01	1.013	4.453	2.1201811	8.40E-45	6.84E-45	1.18	0.86	1	4.71	4.63	4.02
OsR498G0408957400.01	3.556	18.096	2.3540448	5.73E-151	2.14E-151	3.63	3.37	3.67	19.26	18.15	16.88
OsR498G0408963000.01	36.886	283.44	2.9528443	0	0	37.37	36.24	37.05	282.61	280.41	287.3
OsR498G0408964300.01	10.883	4.633	-1.256753	5.45E-64	3.64E-64	10.71	11.06	10.88	4.85	4.31	4.74
OsR498G0408964500.01	2.25	7.36	1.70677	2.11E-71	1.31E-71	2.28	2.32	2.15	7.02	7.53	7.53
OsR498G0408965100.01	21.943	56.61	1.3881769	1.75E-211	4.98E-212	21.29	23.27	21.27	57.93	56.33	55.57
OsR498G0408967400.01	3.613	8.303	1.1100206	1.08E-21	1.26E-21	4.3	3.34	3.2	7.53	8.11	9.27
OsR498G0408982000.01	11.596	32.356	1.3623202	2.84E-244	7.19E-245	11.23	11.98	11.58	33.01	33.5	30.56

OsR498G0408982500.01	4.576	20.453	2.50699	3.00E-276	6.77E-277	4.14	5.39	4.2	20.49	20.84	20.03
OsR498G0408986600.01	4.773	23.553	2.2876592	0	0	4.8	4.91	4.61	24.4	24.31	21.95
OsR498G0408987700.01	288.79	140.24	-1.043287	0	0	273.33	301.33	291.71	140.07	139.09	141.55
OsR498G0408991600.01	25.966	52.936	1.0256577	9.11E-179	2.99E-179	26.39	25.45	26.06	52.95	53.37	52.49
OsR498G0408993200.01	12.076	4.983	-1.298345	2.24E-79	1.32E-79	12.68	11.93	11.62	4.74	5.18	5.03
OsR498G0408993300.01	6.22	2.556	-1.461795	3.67E-56	2.64E-56	5.85	6.46	6.35	2.4	2.33	2.94
OsR498G0408993600.01	2.566	0.973	-1.390162	2.92E-08	4.79E-08	2.78	2.53	2.39	1.09	1.23	0.6
OsR498G0409002900.01	38.83	79.743	1.0373827	5.99E-191	1.86E-191	38.44	38.41	39.64	81.07	78.06	80.1
OsR498G0409006200.01	7.693	3.716	-1.051403	2.67E-54	1.95E-54	7.44	7.52	8.12	3.41	4.46	3.28
OsR498G0409006400.01	0.573	2.683	2.2315678	3.60E-25	3.93E-25	0.61	0.52	0.59	3.04	2.27	2.74
OsR498G0409007200.01	0.153	2.053	3.6876894	2.67E-29	2.72E-29	0.06	0.15	0.25	1.8	2.14	2.22
OsR498G0409007600.01	0.11	1.12	3.342045	4.11E-06	7.40E-06	0	0	0.33	1.28	0.57	1.51
OsR498G0409008000.01	13.086	33.803	1.3826262	6.85E-286	1.51E-286	12.51	13.37	13.38	34.7	32.58	34.13
OsR498G0409010500.01	3.773	8.793	1.2162986	9.49E-47	7.57E-47	3.8	3.83	3.69	7.71	9.38	9.29
OsR498G0409010900.01	6.253	28.22	2.1402188	7.82E-81	4.55E-81	6.53	6.27	5.96	29.03	27.54	28.09
OsR498G0409011100.01	1.57	8.77	2.4803009	3.94E-40	3.42E-40	1.89	1.22	1.6	8.36	7.87	10.08
OsR498G0409012200.01	0.286	5.203	4.1838633	7.20E-57	5.14E-57	0.31	0.37	0.18	4.72	5.12	5.77
OsR498G0409012400.01	8.826	46.48	2.3972284	0	0	8.43	8.66	9.39	47.93	47.89	43.62
OsR498G0409012600.01	8.35	24.636	1.5613811	9.89E-213	2.81E-213	7.82	9.15	8.08	24.34	25.18	24.39
OsR498G0409015600.01	0.766	2.37	1.637375	8.67E-14	1.20E-13	0.94	0.71	0.65	2.14	2.4	2.57
OsR498G0409023700.01	10.673	2.746	-1.965583	1.92E-108	9.11E-109	10.04	10.8	11.18	2.6	2.91	2.73
OsR498G0409024300.01	0.75	2.986	2.0007941	1.92E-127	8.16E-128	0.74	0.75	0.76	3.16	3.2	2.6
OsR498G0409024600.01	3.063	7.1	1.2110443	2.96E-27	3.13E-27	2.57	3.3	3.32	7.26	6.61	7.43
OsR498G0409026000.01	6.163	1.466	-2.071884	9.32E-44	7.70E-44	4.8	6.93	6.76	1.99	0.95	1.46
OsR498G0409029500.01	1337.7	567.86	-1.223068	0	0	1340.9	1363.5	1308.7	584.72	576.08	542.77
OsR498G0409039700.01	3.193	15.2	2.2666077	3.74E-211	1.07E-211	3.41	2.94	3.23	15.86	15.22	14.52
OsR498G0409043500.01	0.09	2.19	4.6468353	5.11E-36	4.70E-36	0.09	0.09	0.09	2.3	2.08	2.19
OsR498G0409048200.01	3.99	10.32	1.372506	6.57E-72	4.07E-72	4.03	4.23	3.71	10.57	10.82	9.57
OsR498G0409055000.01	1.896	10.113	2.4135216	1.82E-75	1.10E-75	2.37	2.07	1.25	11.06	10.1	9.18
OsR498G0409055200.01	4.493	11.043	1.2889206	1.57E-44	1.29E-44	4.4	4.89	4.19	11.98	10.57	10.58
OsR498G0409055700.01	0.613	1.716	1.4889831	2.11E-22	2.41E-22	0.63	0.75	0.46	1.67	2.02	1.46
OsR498G0409056100.01	304.03	1273.7	2.0459839	0	0	298.53	311.66	301.9	1291.1	1268	1261.9
OsR498G0409056600.01	0.36	122.1	8.4093101	0	0	0.24	0.44	0.4	119.13	120.85	126.33
OsR498G0409058200.01	0.633	11.38	4.1724722	1.42E-215	3.97E-216	0.66	0.55	0.69	12.73	10.62	10.79
OsR498G0409058300.01	0.016	0.193	3.5487773	2.09E-06	3.72E-06	0.05	0	0	0.12	0.34	0.12
OsR498G0409058600.01	18.26	1.136	-4.007819	0	0	18.4	17.17	19.21	1.43	0.8	1.18
OsR498G0409060800.01	0	0.313	5.97441	2.98E-09	4.72E-09	0	0	0	0.17	0.35	0.42
OsR498G0409061000.01	0	0.376	6.144335	3.60E-10	5.53E-10	0	0	0	0.34	0.31	0.48
OsR498G0409062400.01	0.21	0.6	1.5218978	0.0002209	0.0004369	0.24	0.29	0.1	0.75	0.47	0.58
OsR498G0409065000.01	10.276	4.686	-1.168677	1.43E-46	1.15E-46	9.88	10.87	10.08	4.59	4.92	4.55
OsR498G0409066100.01	491.946	1037.4	1.0753067	0	0	489.85	476.76	509.23	1010.7	1016.3	1085.1
OsR498G0409067600.01	2.306	4.793	1.057826	1.03E-19	1.24E-19	2.58	2	2.34	5.01	4.82	4.55
OsR498G0409069000.01	0.023	1.04	5.4662631	2.74E-12	3.94E-12	0.07	0	0	1.03	1.1	0.99
OsR498G0409069500.01	82.286	179.87	1.1629189	0	0	81.62	81.73	83.51	181.01	182.44	176.15
OsR498G0409069700.01	3.91	8.41	1.0944657	3.57E-24	3.96E-24	2.98	4.14	4.61	8.1	7.97	9.16
OsR498G0409070000.01	0.336	0.813	1.4719096	3.01E-08	4.95E-08	0.36	0.36	0.29	0.7	0.87	0.87
OsR498G0409074600.01	7.183	15.393	1.0939399	7.61E-75	4.61E-75	7.57	6.83	7.15	14.54	15.87	15.77
OsR498G0409074700.01	2.69	5.36	1.0010998	7.46E-20	8.98E-20	2.71	2.66	2.7	5.24	5.5	5.34
OsR498G0409079500.01	1.496	4.433	1.5754921	6.01E-22	6.95E-22	1.38	1.59	1.52	5.35	3.88	4.07
OsR498G0409079700.01	164.92	523.07	1.6644183	0	0	167.71	159.7	167.35	522.23	506.82	540.15
OsR498G0409082600.01	5.42	25.676	2.243631	6.85E-259	1.63E-259	5.61	5.33	5.32	24.98	25.59	26.46
OsR498G0409090000.01	1.733	4.926	1.4489666	1.03E-49	7.90E-50	1.8	1.55	1.85	4.67	5.42	4.69
OsR498G0409092500.01	37.27	102.96	1.4712174	0	0	38.16	37.23	36.42	100.49	99.33	109.05
OsR498G0409098300.01	2.87	6.736	1.2288751	7.45E-26	8.04E-26	3.47	3.09	2.05	5.63	6.87	7.71
OsR498G0409099500.01	0.576	2.933	2.3447784	1.26E-28	1.30E-28	0.55	0.63	0.55	2.96	2.5	3.34
OsR498G0409099800.01	0.946	3.62	1.9851898	3.70E-43	3.08E-43	1.01	0.75	1.08	3.7	3.95	3.21
OsR498G0409100900.01	0.53	1.48	1.4813699	3.89E-09	6.19E-09	0.67	0.46	0.46	1.51	1.42	1.51
OsR498G0409109900.01	1.22	3.293	1.4347986	2.73E-26	2.93E-26	0.97	1.22	1.47	3.12	3.39	3.37
OsR498G0409116700.01	2.45	18.42	2.8783449	3.66E-123	1.59E-123	2.59	2.43	2.33	16.92	17.64	20.7
OsR498G0409117500.01	104.353	307.14	1.5744055	0	0	103.17	105.36	104.53	308.57	310.37	302.47
OsR498G0409119100.01	1.063	10.546	3.3033245	1.19E-187	3.76E-188	1.02	1.06	1.11	10.74	10.08	10.82
OsR498G0409125200.01	1.903	8.17	2.1413634	1.25E-56	8.97E-57	1.82	1.84	2.05	8.48	6.85	9.18
OsR498G0409129500.01	7.64	17.753	1.1991293	9.02E-63	6.08E-63	6.49	8.77	7.66	18.11	17.26	17.89
OsR498G0409132600.01	2.386	0.5	-2.174453	8.65E-14	1.19E-13	2.94	1.88	2.34	0.52	0.52	0.46
OsR498G0409132800.01	0.17	1.003	2.5593725	9.16E-18	1.15E-17	0.19	0.16	0.16	0.86	1.23	0.92
OsR498G0409134300.01	1.376	0.12	-3.511017	1.35E-12	1.93E-12	1.19	1.32	1.62	0	0.29	0.07
OsR498G0409135500.01	40.77	90.28	1.1423254	2.71E-140	1.07E-140	41.97	39.74	40.6	93.1	87.29	90.45
OsR498G0409136200.01	0.84	0	-7.72603	2.47E-24	2.73E-24	0.91	0.82	0.79	0	0	0
OsR498G0409141300.01	0.643	0.17	-1.913115	3.79E-09	6.03E-09	0.71	0.57	0.65	0.25	0.05	0.21

OsR498G0409144200.01	0.206	1.876	3.162037	5.03E-16	6.59E-16	0.07	0.47	0.08	1.82	1.95	1.86
OsR498G0409145800.01	0.646	0.213	-1.575787	8.13E-05	0.0001565	0.76	0.71	0.47	0.37	0.18	0.09
OsR498G0409147300.01	1.846	4.06	1.1593277	3.25E-14	4.44E-14	1.62	2.22	1.7	3.86	3.63	4.69
OsR498G0409147600.01	0.186	1	2.4006747	5.46E-13	7.69E-13	0.24	0.08	0.24	0.99	0.94	1.07
OsR498G0409148600.01	0.56	3.193	2.5091867	1.01E-32	9.72E-33	0.56	0.47	0.65	3.11	2.93	3.54
OsR498G0409149800.01	0.23	0.506	1.1232733	0.000623	0.0012729	0.19	0.2	0.3	0.43	0.62	0.47
OsR498G0409155600.01	0.19	1.006	2.3991935	6.15E-15	8.26E-15	0.31	0.02	0.24	0.92	1.09	1.01
OsR498G0409156200.01	0.216	0.943	2.1348746	5.87E-19	7.18E-19	0.21	0.29	0.15	0.9	1.09	0.84
OsR498G0409162700.01	0.373	0.853	1.0879419	2.63E-05	4.93E-05	0.41	0.37	0.34	0.71	1	0.85
OsR498G0409164400.01	1.52	7.696	2.3435246	3.54E-111	1.65E-111	1.77	1.52	1.27	7.61	7.91	7.57
OsR498G0409166900.01	20.43	110.03	2.3654443	0	0	20.64	20.87	19.78	112.2	110.29	107.61
OsR498G0409167200.01	0.106	0.563	2.4005856	1.12E-09	1.75E-09	0.14	0.15	0.03	0.75	0.52	0.42
OsR498G0409168400.01	0.906	0.25	-1.848712	4.13E-06	7.43E-06	1.07	0.99	0.66	0.11	0.32	0.32
OsR498G0409169200.01	1.113	0.376	-1.599325	9.97E-12	1.46E-11	1.18	1.09	1.07	0.41	0.38	0.34
OsR498G0409169600.01	1.746	3.933	1.1344872	1.51E-21	1.76E-21	1.97	1.55	1.72	3.83	3.77	4.2
OsR498G0409174400.01	3.966	12.406	1.6456049	2.84E-48	2.22E-48	4.55	3.93	3.42	12.04	12.69	12.49
OsR498G0409177900.01	0.486	2.27	2.2187172	2.33E-25	2.53E-25	0.32	0.55	0.59	2.24	2.3	2.27
OsR498G0409178100.01	3.59	7.863	1.1307245	3.63E-30	3.65E-30	3.47	3.96	3.34	7.54	8.25	7.8
OsR498G0409178500.01	0.51	1.476	1.5228466	4.34E-10	6.68E-10	0.58	0.56	0.39	1.04	1.35	2.04
OsR498G0409180500.01	0.016	0.083	2.3894475	0.0009846	0.0020515	0.04	0.01	0	0.12	0.05	0.08
OsR498G0409181900.01	23.183	120.87	2.3828859	0	0	22.98	23.44	23.13	123.73	123.8	115.09
OsR498G0409182200.01	0.426	1.446	1.8182981	2.96E-20	3.54E-20	0.43	0.32	0.53	1.45	1.58	1.31
OsR498G0409185200.01	18.26	44.746	1.3013082	0	0	16.91	17.89	19.98	42.31	44.2	47.73
OsR498G0409185300.01	103.636	227.14	1.1330047	0	0	97.45	106.33	107.13	238.22	220.28	222.93
OsR498G0409191200.01	14.716	45.896	1.6393237	0	0	13.68	14.64	15.83	46.49	44.14	47.06
OsR498G0409196100.01	20.69	6.2	-1.739412	3.65E-280	8.15E-281	20.6	22	19.47	6.34	6	6.26
OsR498G0409196800.01	24.083	49.34	1.0358479	6.04E-132	2.51E-132	25.11	23.6	23.54	48.33	50.81	48.88
OsR498G0409198000.01	0.923	0.12	-2.932481	1.12E-06	1.96E-06	0.56	1.38	0.83	0.18	0.09	0.09
OsR498G0409198900.01	3.47	9.026	1.3801593	2.46E-74	1.50E-74	3.04	3.63	3.74	8.9	9.49	8.69
OsR498G0409199100.01	0.706	3.02	2.0231693	1.23E-36	1.12E-36	0.72	0.95	0.45	2.6	2.88	3.58
OsR498G0409199300.01	0.01	0.203	4.2963381	4.68E-06	8.46E-06	0	0.03	0	0.15	0.24	0.22
OsR498G0409200000.01	0.296	0.686	1.2195225	6.08E-05	0.0001163	0.33	0.29	0.27	0.6	0.47	0.99
OsR498G0409203000.01	2.143	23.39	3.5248696	5.17E-299	1.10E-299	2.11	2.46	1.86	22.47	24.08	23.62
OsR498G0409205000.01	7.216	30.68	2.0875556	1.58E-249	3.91E-250	6.98	6.66	8.01	30.98	29.61	31.45
OsR498G0409205200.01	50.75	112	1.1376168	2.12E-270	4.86E-271	52.78	49.54	49.93	108.29	111.6	116.1
OsR498G0409206800.01	8.893	3.673	-1.300641	8.09E-39	7.15E-39	9.26	8.73	8.69	3.72	3.56	3.74
OsR498G0409209900.01	0	0.193	5.2223375	3.89E-06	7.00E-06	0	0	0	0.21	0.18	0.19
OsR498G0409212700.01	3.49	29.06	3.056204	1.07E-235	2.80E-236	3	3.86	3.61	28.19	29.61	29.38
OsR498G0409217700.01	1.346	4.99	1.9030712	6.54E-44	5.39E-44	1.17	1.41	1.46	5.53	4.94	4.5
OsR498G0409218900.01	0.893	1.913	1.0977924	3.17E-12	4.57E-12	0.94	0.78	0.96	1.59	2.3	1.85
OsR498G0409220100.01	0.556	2.716	2.3667274	1.28E-20	1.52E-20	0.66	0.63	0.38	2.49	2.7	2.96
OsR498G0409220400.01	2.546	51.933	4.2835519	0	0	2.58	2.68	2.38	52.05	51.62	52.13
OsR498G0409220600.01	1.373	57.986	5.4010316	0	0	1.5	1.22	1.4	56.86	57.47	59.63
OsR498G0409221000.01	2.113	4.326	1.0041573	1.17E-12	1.67E-12	1.93	2.29	2.12	4.73	4.36	3.89
OsR498G0409221600.01	7.393	17.33	1.1942303	6.32E-93	3.34E-93	7.44	7.79	6.95	17.22	18.16	16.61
OsR498G0409222200.01	1.12	0.223	-2.332251	7.80E-14	1.08E-13	1.29	0.92	1.15	0.25	0.13	0.29
OsR498G0409227200.01	1.04	2.61	1.5011578	1.29E-13	1.79E-13	1.08	1.33	0.71	2.13	3.27	2.43
OsR498G0409228300.01	28.22	6.413	-2.152211	3.33E-252	8.12E-253	26.52	28.77	29.37	6.57	6.52	6.15
OsR498G0409229200.01	15.61	35.523	1.1839871	6.17E-72	3.82E-72	15.67	15.65	15.51	32.43	35.68	38.46
OsR498G0409231700.01	2.65	9.886	1.9033269	3.06E-40	2.66E-40	2.55	2.82	2.58	9.44	8.77	11.45
OsR498G0409234500.01	0.746	2.2	1.5593725	1.03E-07	1.73E-07	1.08	0.33	0.83	1.94	2.09	2.57
OsR498G0409238500.01	10.19	62.426	2.616358	0	0	10.28	9.89	10.4	63.46	63.15	60.67
OsR498G0409239000.01	0.11	0.503	2.1968024	1.17E-06	2.06E-06	0	0	0.33	0.57	0.35	0.59
OsR498G0409242100.01	15.866	32.866	1.0426267	1.99E-82	1.14E-82	14.47	15.31	17.82	33.59	31.62	33.39
OsR498G0409245200.01	12.96	40.35	1.5511321	0	0	12.75	12.64	13.49	40.73	38.93	41.39
OsR498G0409245300.01	1.01	4.38	2.1360803	1.76E-15	2.33E-15	0.43	1.04	1.56	3.12	4.29	5.73
OsR498G0409246400.01	0.08	0.54	2.9286063	8.57E-12	1.25E-11	0.15	0	0.09	0.83	0.43	0.36
OsR498G0409249700.01	0	0.306	4.6748497	0.0001298	0.0002536	0	0	0	0.14	0.28	0.5
OsR498G0409250300.01	0.773	1.626	1.1130635	9.08E-13	1.29E-12	0.86	0.87	0.59	1.6	1.86	1.42
OsR498G0409251200.01	1.54	4.496	1.5732446	6.74E-33	6.47E-33	1.39	1.46	1.77	3.77	5.08	4.64
OsR498G0409251300.01	0.033	0.276	2.97441	0.0002639	0.0005255	0	0.05	0.05	0.31	0.41	0.11
OsR498G0409252300.01	8.433	40.45	2.2536138	0	0	7.76	8.79	8.75	42.14	39.96	39.25
OsR498G0409252600.01	3.766	8.236	1.1351572	2.15E-42	1.81E-42	3.58	4.17	3.55	7.99	8.91	7.81
OsR498G0409255000.01	3.87	11.176	1.5320573	5.19E-84	2.94E-84	3.92	3.98	3.71	11.98	11.29	10.26
OsR498G0409257100.01	0.05	0.213	2.109299	0.0005317	0.0010802	0	0.03	0.12	0.13	0.18	0.33
OsR498G0409258900.01	15.196	73.603	2.2711218	0	0	15.71	15.45	14.43	70.07	68.81	81.93
OsR498G0409259400.01	4.793	31.616	2.7202359	0	0	4.36	5.19	4.83	31.01	31.92	31.92
OsR498G0409260900.01	10.796	38.53	1.8361718	0	0	11.31	10.36	10.72	39.41	38.06	38.12
OsR498G0409261700.01	1.37	0.5	-1.443928	3.80E-15	5.07E-15	1.58	1.19	1.34	0.61	0.47	0.42

OsR498G0409263100.01	1.276	0.353	-1.851561	5.99E-13	8.45E-13	1.58	1.4	0.85	0.35	0.22	0.49
OsR498G0409263500.01	68.296	32.906	-1.06482	1.37E-112	6.35E-113	66.29	69.97	68.63	34.47	28.13	36.12
OsR498G0409263700.01	1.136	4.586	2.0136602	5.07E-74	3.09E-74	1.3	1.19	0.92	4.44	4.73	4.59
OsR498G0409264000.01	3.213	8.403	1.393896	1.66E-91	8.90E-92	3.5	2.94	3.2	8.75	8.51	7.95
OsR498G0409265900.01	1.376	0	-6.70197	1.09E-13	1.50E-13	1.71	1.29	1.13	0	0	0
OsR498G0409266800.01	40.58	90.62	1.1622542	0	0	38.63	41.02	42.09	90.41	87.47	93.98
OsR498G0409267100.01	0.433	0.986	1.0802046	4.99E-10	7.70E-10	0.45	0.32	0.53	1.07	1	0.89
OsR498G0409267500.01	6.053	16.76	1.4679494	4.63E-52	3.48E-52	5.9	6.33	5.93	18.86	14.73	16.69
OsR498G0409267800.01	0.1	0.47	2.3079821	1.38E-05	2.55E-05	0.14	0.11	0.05	0.52	0.46	0.43
OsR498G0409267900.01	0.493	1.19	1.5152799	3.23E-12	4.66E-12	0.38	0.66	0.44	1.03	1.37	1.17
OsR498G0409268700.01	0.07	0.396	2.5593725	1.22E-07	2.05E-07	0.12	0.03	0.06	0.42	0.42	0.35
OsR498G0409269300.01	11.356	63.056	2.4704372	0	0	9.51	11.84	12.72	61.08	55.98	72.11
OsR498G0409269800.01	0.403	0.096	-1.989064	8.07E-06	1.47E-05	0.31	0.42	0.48	0.12	0.09	0.08
OsR498G0409270400.01	11.363	27.886	1.294738	2.41E-176	8.04E-177	12.27	10.69	11.13	28.6	27.73	27.33
OsR498G0409274100.01	34.19	68.47	1.0035718	4.06E-281	9.05E-282	34.65	33.86	34.06	68.47	68.4	68.54
OsR498G0409276700.01	67.036	136.76	1.0398285	0	0	67.58	66.5	67.03	137.92	139.21	133.16
OsR498G0409276900.01	19.47	44.31	1.1849027	3.75E-269	8.63E-270	19.14	19.93	19.34	43.43	44.83	44.67
OsR498G0409277400.01	1.073	2.183	1.0238786	4.18E-05	7.91E-05	0.97	1.19	1.06	2.11	2.18	2.26
OsR498G0409282200.01	0.76	1.596	1.0647251	2.17E-14	2.96E-14	0.76	0.62	0.9	1.54	1.66	1.59
OsR498G0409282400.01	0.073	0.776	3.3671002	3.87E-30	3.88E-30	0.12	0.08	0.02	0.71	0.79	0.83
OsR498G0409282800.01	0.253	1.51	2.6903817	4.40E-56	3.17E-56	0.27	0.29	0.2	1.6	1.37	1.56
OsR498G0409288100.01	4.77	26.673	2.5081599	0	0	5.15	4.63	4.53	26.83	27.67	25.52
OsR498G0409288800.01	17.74	54.273	1.6211975	4.99E-288	1.09E-288	17.97	18.18	17.07	54.51	55.78	52.53
OsR498G0409291200.01	0	0.086	4.2963381	0.0007942	0.0016388	0	0	0	0.08	0.1	0.08
OsR498G0409292800.01	4.143	22.96	2.461801	1.71E-276	3.85E-277	4.22	3.89	4.32	22.21	24.08	22.59
OsR498G0409294000.01	0.643	3.346	2.3781321	2.61E-83	1.49E-83	0.71	0.72	0.5	3.55	3.5	2.99
OsR498G0409296700.01	4.016	15.693	1.9622514	3.46E-228	9.25E-229	4.36	3.76	3.93	15.77	16.11	15.2
OsR498G0409298500.01	0.13	0.32	1.3132119	0.0004058	0.0008174	0.28	0.02	0.09	0.29	0.51	0.16
OsR498G0409300400.01	0.196	1.23	2.6371952	1.60E-09	2.51E-09	0.09	0.21	0.29	1.61	1.19	0.89
OsR498G0409300700.01	0.033	0.393	3.5593725	0.0005724	0.0011661	0	0.1	0	0.39	0.49	0.3
OsR498G0409301900.01	1.33	6.97	2.3913647	1.11E-66	7.23E-67	1.24	1.39	1.36	7.19	6.92	6.8
OsR498G0409306000.01	0.936	0	-6.154873	4.43E-10	6.82E-10	1.45	0.64	0.72	0	0	0
OsR498G0409307900.01	5.843	2.13	-1.499746	3.80E-52	2.85E-52	6.2	5.58	5.75	2.17	1.94	2.28
OsR498G0409308500.01	1088.6	282.44	-1.946608	0	0	1074.3	1095.4	1096.1	276.85	279.59	290.89
OsR498G0409314500.01	3.186	7.956	1.319702	2.85E-30	2.85E-30	3.24	3.06	3.26	7.85	8.1	7.92
OsR498G0409315000.01	0.446	1.846	2.2529443	1.38E-57	9.78E-58	0.55	0.4	0.39	1.86	1.78	1.9
OsR498G0409315200.01	0.063	0.223	2.1025374	8.32E-08	1.39E-07	0.08	0.02	0.09	0.22	0.2	0.25
OsR498G0409321000.01	0.173	0.423	1.4032533	0.0007247	0.0014903	0.31	0.07	0.14	0.61	0.35	0.31
OsR498G0409322200.01	0.83	0	-6.195515	2.60E-10	3.98E-10	0.9	1.04	0.55	0	0	0
OsR498G0409323600.01	1.053	3.17	1.5907854	2.19E-26	2.34E-26	1.02	1.1	1.04	3.2	3.55	2.76
OsR498G0409323700.01	187.82	405.97	1.1089717	0	0	183.16	187.98	192.32	411.71	403.86	402.34
OsR498G0409325300.01	10.66	4.906	-1.188553	2.04E-22	2.34E-22	11.39	10.32	10.27	5.26	3.45	6.01
OsR498G0409326200.01	0.013	0.226	3.6748497	2.00E-09	3.14E-09	0.01	0.03	0	0.17	0.26	0.25
OsR498G0409327400.01	0.616	0.17	-1.84662	7.34E-12	1.07E-11	0.69	0.62	0.54	0.14	0.18	0.19
OsR498G0409327700.01	2.18	6.866	1.6544653	3.74E-83	2.13E-83	2.35	2.25	1.94	6.76	6.8	7.04
OsR498G0409328900.01	5.573	18.63	1.7458182	3.78E-133	1.55E-133	6.32	4.67	5.73	18.95	19.26	17.68
OsR498G0409329100.01	0.196	0.856	2.1319512	2.66E-08	4.36E-08	0.14	0.27	0.18	0.79	0.79	0.99
OsR498G0409329700.01	0.706	0	-6.580179	8.73E-13	1.24E-12	0.9	0.67	0.55	0	0	0
OsR498G0409332600.01	1.253	2.57	1.2029969	4.67E-21	5.49E-21	1.38	1.5	0.88	2.42	2.78	2.51
OsR498G0409332800.01	7.766	21.416	1.4878129	2.03E-254	4.92E-255	8.49	7.3	7.51	22.04	21.94	20.27
OsR498G0409334400.01	6.51	44.356	2.76336	0	0	6.77	6.1	6.66	43.82	46.83	42.42
OsR498G0409334600.01	56.38	215.84	1.9244966	0	0	57.07	56.72	55.35	221.3	220.45	205.78
OsR498G0409334800.01	5.436	1.246	-2.157897	6.89E-92	3.67E-92	6.29	5.85	4.17	1.22	1.38	1.14
OsR498G0409335600.01	3.236	1.12	-1.523496	5.28E-32	5.15E-32	3.39	3.33	2.99	1.01	1.1	1.25
OsR498G0409339000.01	0.18	0.023	-3.298609	4.58E-07	7.90E-07	0.12	0.27	0.15	0.07	0	0
OsR498G0409339700.01	5.966	19.04	1.7557697	1.52E-173	5.12E-174	5.9	5.16	6.84	18.74	19.08	19.3
OsR498G0409340400.01	0.783	2.226	1.5027889	5.68E-07	9.85E-07	1	0.63	0.72	2.38	1.41	2.89
OsR498G0409340800.01	3.886	0.286	-3.757394	1.16E-20	1.37E-20	3.65	3.76	4.25	0.37	0.24	0.25
OsR498G0409341800.01	5.996	0.14	-5.417907	1.01E-21	1.17E-21	4.93	7.52	5.54	0.42	0	0
OsR498G0409342400.01	6.553	0.36	-4.195515	3.71E-29	3.79E-29	7.04	6.4	6.22	0	0.61	0.47
OsR498G0409343000.01	8.703	1.31	-2.738227	6.33E-29	6.49E-29	7.99	8.12	10	1.73	0.73	1.47
OsR498G0409343500.01	3.97	7.933	1.0198144	2.05E-45	1.66E-45	3.91	4.08	3.92	7.78	8.29	7.73
OsR498G0409344100.01	4.446	24.42	2.4572628	0	0	4.21	4.97	4.16	24.11	25.79	23.36
OsR498G0409344400.01	0.336	1.886	2.7158769	9.93E-34	9.43E-34	0.33	0.34	0.34	1.9	1.82	1.94
OsR498G0409345100.01	0.296	1.323	2.1527472	1.34E-11	1.97E-11	0.52	0.14	0.23	1.1	1.18	1.69
OsR498G0409345800.01	3.386	7.203	1.0958298	8.97E-40	7.84E-40	3.79	2.76	3.61	6.52	7.35	7.74
OsR498G0409347700.01	1.273	7.386	2.549044	2.17E-214	6.10E-215	1.41	1.3	1.11	7.32	8.02	6.82
OsR498G0409347900.01	0.98	2.036	1.0534815	9.30E-08	1.56E-07	0.58	1.2	1.16	1.9	2.01	2.2
OsR498G0409349400.01	37.396	14.226	-1.382301	1.19E-128	5.02E-129	38.15	37.68	36.36	13.93	13	15.75

OsR498G0409350900.01	6.48	0.456	-3.82687	2.23E-50	1.71E-50	6.1	6.22	7.12	0.64	0.32	0.41
OsR498G0409354400.01	0.176	0.306	1.7113756	0.000336	0.0006733	0.02	0.32	0.19	0.29	0.37	0.26
OsR498G0409355100.01	1.103	2.89	1.373447	3.66E-18	4.55E-18	1.4	0.86	1.05	3.08	2.71	2.88
OsR498G0409363300.01	20.303	46.623	1.1903128	2.48E-170	8.45E-171	20.76	19.34	20.81	45.7	44.38	49.79
OsR498G0409363800.01	4.5	30.896	2.8034476	0	0	4.23	4.23	5.04	29.47	31.79	31.43
OsR498G0409364100.01	4.506	16.053	1.8411434	2.40E-145	9.20E-146	4.87	4.24	4.41	15.81	16.05	16.3
OsR498G0409364700.01	2.216	5.003	1.1206738	3.60E-41	3.08E-41	2.27	2.17	2.21	5.15	4.75	5.11
OsR498G0409367300.01	4.593	11.483	1.3637249	2.12E-77	1.26E-77	4.83	4.53	4.42	11.02	11.62	11.81
OsR498G0409368200.01	289.346	99.06	-1.547368	0	0	299.6	290.08	278.36	99.08	99.7	98.4
OsR498G0409368700.01	0.213	3.553	4.144335	2.66E-51	2.01E-51	0.18	0.15	0.31	3.4	3.77	3.49
OsR498G0409368900.01	1.523	4.873	1.6544407	1.14E-60	7.85E-61	1.5	1.56	1.51	4.47	4.88	5.27
OsR498G0409369300.01	3.843	9.143	1.253913	1.10E-55	7.96E-56	3.98	3.7	3.85	9.97	8.88	8.58
OsR498G0409371600.01	2.066	0.566	-1.948969	4.98E-13	7.02E-13	1.97	2.22	2.01	0.38	0.81	0.51
OsR498G0409376800.01	0.506	0.886	1.2821164	1.06E-05	1.95E-05	0.67	0.3	0.55	0.68	0.96	1.02
OsR498G0409378500.01	0.84	4.67	2.4743167	2.33E-101	1.16E-101	0.76	0.93	0.83	4.92	4.81	4.28
OsR498G0409380900.01	0.3	1.623	2.4169282	1.35E-29	1.37E-29	0.38	0.21	0.31	1.91	1.59	1.37
OsR498G0409388800.01	2.683	0.963	-1.477286	3.43E-08	5.65E-08	2.61	2.33	3.11	1.27	1.01	0.61
OsR498G0409389300.01	0.33	1.54	2.2322077	6.35E-15	8.53E-15	0.43	0.3	0.26	1.5	1.41	1.71
OsR498G0409389600.01	74.6	220.35	1.5527382	0	0	72.93	75.16	75.71	217.67	213.57	229.82
OsR498G0409392200.01	1.736	4.7	1.5299808	1.49E-33	1.42E-33	2.16	1.25	1.8	4.67	4.25	5.18
OsR498G0409394100.01	1.456	7.373	2.3401761	5.39E-106	2.60E-106	1.58	1.35	1.44	7.23	7.91	6.98
OsR498G0409395700.01	0.13	1.313	3.3436438	2.11E-07	3.59E-07	0	0	0.39	1.01	1	1.93
OsR498G0409395900.01	0.286	2.803	3.271027	3.96E-42	3.34E-42	0.34	0.26	0.26	2.62	2.83	2.96
OsR498G0409396200.01	0.016	0.446	4.8813006	1.33E-08	2.15E-08	0	0.05	0	0.22	0.62	0.5
OsR498G0409396500.01	1.046	3.543	1.7558547	5.92E-12	8.61E-12	0.92	1.57	0.65	3.88	2.69	4.06
OsR498G0409397800.01	0.26	1.303	2.336118	1.30E-09	2.04E-09	0	0	0.78	2.68	0.72	0.51
OsR498G0409399100.01	0.996	0.183	-2.440628	0.0007945	0.00164	1.31	0.19	1.49	0.18	0	0.37
OsR498G0409400300.01	4.113	11.806	1.5226508	5.26E-54	3.86E-54	3.44	4.25	4.65	12.27	12.07	11.08
OsR498G0409402700.01	12.983	33.196	1.3468158	6.41E-196	1.96E-196	13.49	12.81	12.65	34.23	32.81	32.55
OsR498G0409403100.01	0.026	2.253	6.4173535	6.51E-22	7.52E-22	0	0	0.08	2.31	2.23	2.22
OsR498G0409404300.01	22.35	59.356	1.4100614	0	0	20.48	23.65	22.92	59.58	58.68	59.81
OsR498G0409406200.01	0.073	10.443	7.144335	7.71E-34	7.30E-34	0	0	0.22	7.8	12.08	11.45
OsR498G0409409300.01	39.596	152.08	1.9291952	0	0	40.17	39.62	39	152.84	152.34	151.06
OsR498G0409415100.01	0.876	0.103	-3.069984	8.22E-19	1.01E-18	0.78	0.87	0.98	0.1	0.05	0.16
OsR498G0409417800.01	768.123	299.17	-1.359242	0	0	793.33	760.96	750.08	298.57	295.48	303.47
OsR498G0409417900.01	191.713	79.363	-1.265003	0	0	193.76	185.87	195.51	76.43	75.96	85.7
OsR498G0409418500.01	65.57	29.993	-1.13014	1.44E-78	8.48E-79	68.78	64.37	63.56	31.55	27.9	30.53
OsR498G0409422600.01	3.693	0.703	-2.401457	3.00E-16	3.90E-16	3.88	2.64	4.56	0.7	0.49	0.92
OsR498G0409423800.01	1581.25	337.88	-2.225981	0	0	1594	1581.2	1568.6	337.03	341.45	335.15
OsR498G0409426500.01	0.02	0.24	3.4979719	4.44E-06	8.01E-06	0.03	0	0.03	0.22	0.25	0.25
OsR498G0409429000.01	7.666	24.856	1.6981117	0	0	8.16	7.38	7.46	26.2	26.04	22.33
OsR498G0409429200.01	42.566	91.376	1.1023377	1.33E-121	5.80E-122	44.61	41.6	41.49	91.27	86.07	96.79
OsR498G0409431000.01	2.68	5.873	1.1482124	9.63E-32	9.44E-32	2.83	3.03	2.18	5.81	6.03	5.78
OsR498G0409431200.01	5.45	25.07	2.2103772	1.82E-227	4.89E-228	5.2	5.51	5.64	25.37	25.64	24.2
OsR498G0409432600.01	10.533	26.65	1.3282771	4.59E-82	2.64E-82	10.75	10.29	10.56	26.57	26.19	27.19
OsR498G0409432700.01	33.603	71.286	1.0854728	1.69E-231	4.46E-232	35.44	32.19	33.18	70.07	73.14	70.65
OsR498G0409433200.01	2.68	14.173	2.3785591	3.60E-92	1.91E-92	3.13	2.78	2.13	14.75	14	13.77
OsR498G0409438200.01	2.793	6.626	1.2294831	9.21E-15	1.24E-14	3.02	2.52	2.84	7.59	6.34	5.95
OsR498G0409438300.01	0.773	1.893	1.2889206	4.21E-07	7.25E-07	0.54	1.13	0.65	2.09	1.62	1.97
OsR498G0409445100.01	6.59	19.743	1.589697	5.85E-49	4.55E-49	6.5	6.59	6.68	17.91	18.43	22.89
OsR498G0409445400.01	0.013	0.19	3.6748497	0.0003128	0.0006259	0	0	0.04	0.13	0.26	0.18
OsR498G0409447000.01	0.71	8.416	3.5916128	6.63E-141	2.61E-141	0.54	0.91	0.68	9.13	7.77	8.35
OsR498G0409453400.01	1.093	0.163	-2.741797	7.78E-09	1.25E-08	0.93	1.14	1.21	0.28	0.07	0.14
OsR498G0409454200.01	3.836	7.496	1.0018907	2.32E-19	2.81E-19	3.34	4.27	3.9	6.69	8.61	7.19
OsR498G0409454600.01	2.196	6.323	1.4230279	2.08E-52	1.55E-52	2.12	2.16	2.31	5.61	6.69	6.67
OsR498G0409455200.01	0.113	0.343	1.6194198	1.31E-05	2.42E-05	0.21	0	0.13	0.49	0.29	0.25
OsR498G0409455900.01	21.553	66.816	1.6483658	2.35E-268	5.42E-269	19.34	21.45	23.87	66.82	66.13	67.5
OsR498G0409459400.01	13.056	41.16	1.5894871	1.46E-260	3.45E-261	13.76	12.9	12.51	40.51	42.8	40.17
OsR498G0409459600.01	17.503	37.866	1.1110225	2.53E-114	1.16E-114	17.89	17.07	17.55	39.7	36.48	37.42
OsR498G0409460300.01	5.596	26.006	2.2130354	5.60E-170	1.91E-170	5.64	5.57	5.58	25.76	25.33	26.93
OsR498G0409461000.01	23.383	93.696	1.9597052	0	0	23.63	23.79	22.73	93.53	96.52	91.04
OsR498G0409461600.01	18.61	220.81	3.563808	0	0	18.53	17.23	20.07	217.44	222.79	222.21
OsR498G0409461700.01	5.933	13.796	1.2180033	7.45E-48	5.87E-48	5.28	6.69	5.83	13.82	13.4	14.17
OsR498G0409462200.01	2.283	7.313	1.6769687	5.66E-85	3.18E-85	2.48	2.3	2.07	7.03	7.59	7.32
OsR498G0409463200.01	1.05	1.813	1.0445174	1.79E-11	2.64E-11	1.17	0.99	0.99	2.08	1.88	1.48
OsR498G0409463700.01	7.496	19.6	1.3815853	1.83E-117	8.21E-118	8.15	6.94	7.4	19.49	19.23	20.08
OsR498G0409465400.01	13.473	91.346	2.7694446	0	0	13.69	14.84	11.89	89.63	92.57	91.84
OsR498G0409465600.01	63.626	148.17	1.2188672	0	0	66.15	64.27	60.46	147.28	149.52	147.7
OsR498G0409471000.01	0.183	0.553	1.662466	8.72E-09	1.41E-08	0.21	0.16	0.18	0.64	0.38	0.64

OsR498G0409471300.01	9.826	22.82	1.2187483	3.11E-85	1.74E-85	11.07	9.6	8.81	22.13	21.65	24.68
OsR498G0409471500.01	4.18	9.216	1.1404199	1.48E-43	1.23E-43	4.86	4.01	3.67	9.54	8.79	9.32
OsR498G0409476600.01	1.713	6.223	1.8282775	5.04E-38	4.50E-38	1.25	1.85	2.04	5.84	5.46	7.37
OsR498G0409476700.01	1.423	3.6	1.3378803	3.51E-15	4.69E-15	1.3	1.67	1.3	3.28	3.77	3.75
OsR498G0409477200.01	3.486	7.533	1.1148251	1.54E-23	1.73E-23	3.42	3.17	3.87	7.61	7.01	7.98
OsR498G0409478300.01	4.456	10.206	1.1976041	2.87E-31	2.83E-31	4.81	4.3	4.26	11.61	10.1	8.91
OsR498G0409478900.01	11.093	69.366	2.6458778	0	0	11.29	11.76	10.23	71.84	70.14	66.12
OsR498G0409480000.01	0.436	3.12	2.7775526	5.56E-54	4.08E-54	0.53	0.51	0.27	3.26	2.91	3.19
OsR498G0409481100.01	0.23	0.023	-3.315425	2.58E-05	4.82E-05	0.14	0.36	0.19	0	0	0.07
OsR498G0409481300.01	0.43	0	-5.299852	2.59E-06	4.62E-06	0.24	0.72	0.33	0	0	0
OsR498G0409483700.01	0.086	1.576	4.2264129	1.21E-30	1.20E-30	0.04	0.11	0.11	1.78	1.54	1.41
OsR498G0409484000.01	3.6	10	1.473301	1.37E-145	5.23E-146	3.5	3.52	3.78	9.57	10.33	10.1
OsR498G0409485000.01	3.606	7.63	1.0574917	2.65E-51	2.01E-51	3.72	3.53	3.57	7.74	8.13	7.02
OsR498G0409485300.01	0.02	0.223	3.4979719	4.44E-06	8.01E-06	0	0	0.06	0.23	0.09	0.35
OsR498G0409485700.01	0.99	5.043	2.3556933	5.96E-68	3.82E-68	0.91	1.06	1	4.95	5.49	4.69
OsR498G0409486500.01	0.336	0.036	-3.02559	8.53E-06	1.56E-05	0.44	0.18	0.39	0.05	0	0.06
OsR498G0409486800.01	0.02	0.133	2.7817649	0.0008272	0.0017105	0.06	0	0	0.14	0.2	0.06
OsR498G0409490800.01	0.233	11.346	5.5800319	3.65E-149	1.38E-149	0.16	0.16	0.38	10.03	11.36	12.65
OsR498G0409491400.01	0.15	0.58	2.0118847	6.10E-10	9.45E-10	0.19	0.14	0.12	0.47	0.62	0.65
OsR498G0409491600.01	61.72	123.67	1.0030228	0	0	63.02	61.65	60.49	124.62	124.01	122.37
OsR498G0409493100.01	20.06	7.99	-1.340405	5.16E-74	3.15E-74	19.36	19.5	21.32	7.74	8.65	7.58
OsR498G0409494000.01	0.966	3.5	1.8068559	3.44E-20	4.11E-20	1.15	0.83	0.92	3.87	3.08	3.55
OsR498G0409495300.01	2.043	0.41	-2.30716	1.26E-19	1.53E-19	2.05	2.08	2	0.59	0.25	0.39
OsR498G0409495500.01	1.833	4.09	1.1537337	4.57E-16	5.97E-16	2.22	1.8	1.48	3.52	4.2	4.55
OsR498G0409495700.01	0.033	0.326	3.2474285	6.71E-07	1.17E-06	0	0.03	0.07	0.27	0.37	0.34
OsR498G0409499500.01	7.56	16.383	1.1285047	2.75E-46	2.21E-46	7.75	7.47	7.46	16.84	16.29	16.02
OsR498G0409505900.01	8.91	40.226	2.1674227	0	0	9.33	9.78	7.62	38.26	41.29	41.13
OsR498G0409510200.01	12.173	28.24	1.2150596	1.70E-93	8.92E-94	13.55	11.77	11.2	28.27	28.19	28.26
OsR498G0409513100.01	25.566	0.236	-6.97892	2.66E-256	6.39E-257	27.4	26.25	23.05	0.17	0.29	0.25
OsR498G0409516700.01	233.953	82.686	-1.498071	0	0	224.33	241.76	235.77	83.38	79.16	85.52
OsR498G0409516900.01	0.61	14.523	4.5648269	1.62E-236	4.22E-237	0.67	0.62	0.54	13.94	14.25	15.38
OsR498G0409518400.01	3.903	12.493	1.6748497	9.75E-76	5.87E-76	4.22	3.8	3.69	12.91	12.7	11.87
OsR498G0409518900.01	0.27	0.08	-1.713646	0.0003664	0.0007355	0.28	0.17	0.36	0.05	0.08	0.11
OsR498G0409519400.01	7.406	46.773	2.6568626	9.14E-280	2.05E-280	6.07	8.18	7.97	42.87	47.19	50.26
OsR498G0409520400.01	13.926	30.12	1.1136847	1.86E-179	6.11E-180	13.86	13.76	14.16	31.3	31.01	28.05
OsR498G0409520600.01	6.173	35.67	2.5294882	0	0	6.32	5.92	6.28	36.92	34.59	35.5
OsR498G0409521200.01	1.803	0.796	-1.184494	7.89E-18	9.89E-18	1.82	1.6	1.99	0.78	0.84	0.77
OsR498G0409522400.01	0.02	0.173	2.97441	0.0002639	0.0005255	0.03	0	0.03	0.39	0.13	0
OsR498G0409527400.01	2.823	9.333	1.7421191	7.50E-59	5.25E-59	3.03	2.49	2.95	9.76	9	9.24
OsR498G0409529300.01	1.583	3.743	1.2405841	6.35E-21	7.49E-21	1.81	1.48	1.46	3.66	3.4	4.17
OsR498G0409529600.01	1.066	0.486	-1.301224	5.42E-07	9.38E-07	1.07	0.89	1.24	0.3	0.43	0.73
OsR498G0409533900.01	1.183	0.216	-2.440628	1.34E-12	1.91E-12	1.65	0.88	1.02	0.13	0.34	0.18
OsR498G0409534300.01	2.763	1.113	-1.330031	7.02E-14	9.66E-14	2.98	2.9	2.41	0.94	0.96	1.44
OsR498G0409537200.01	0.02	0.216	3.4979719	4.44E-06	8.01E-06	0	0.03	0.03	0.22	0.2	0.23
OsR498G0409537800.01	0.556	0.146	-1.937024	0.0005227	0.0010616	0.29	1.05	0.33	0.21	0.08	0.15
OsR498G0409540000.01	26.13	66.606	1.3499957	4.71E-182	1.53E-182	25.67	27.08	25.64	65.44	63.74	70.64
OsR498G0409540700.01	7.466	2.706	-1.466163	6.77E-108	3.22E-108	7.1	7.55	7.75	2.79	2.34	2.99
OsR498G0409543100.01	4.17	11.933	1.5187305	1.45E-95	7.54E-96	3.92	4.55	4.04	11.49	12.11	12.2
OsR498G0409544100.01	1.563	3.856	1.273618	7.41E-19	9.08E-19	1.29	2.25	1.15	3.33	3.79	4.45
OsR498G0409545600.01	0.833	0.093	-3.113053	1.48E-19	1.80E-19	0.79	0.83	0.88	0.14	0.09	0.05
OsR498G0409550300.01	15.68	35.906	1.1939983	3.75E-177	1.24E-177	15.09	15.99	15.96	35.87	35.95	35.9
OsR498G0509556900.01	2.5	13.976	2.4754888	0	0	2.41	2.51	2.58	13.39	14.75	13.79
OsR498G0509558600.01	0	0.473	5.2374444	3.47E-06	6.22E-06	0	0	0	0.96	0.17	0.29
OsR498G0509559600.01	4.233	0.09	-5.569911	2.35E-101	1.17E-101	4.08	4.33	4.29	0.09	0.06	0.12
OsR498G0509563100.01	0.133	0.463	1.7817649	5.54E-08	9.19E-08	0.14	0.16	0.1	0.49	0.39	0.51
OsR498G0509563400.01	16.48	8.15	-1.007301	5.45E-117	2.45E-117	16.84	16.08	16.52	8.19	8.06	8.2
OsR498G0509564200.01	12.573	43.376	1.7781095	3.64E-193	1.12E-193	12.56	12.12	13.04	43.17	42.51	44.45
OsR498G0509564700.01	82.046	27.533	-1.560486	9.21E-188	2.91E-188	80.98	85.55	79.61	27.46	25.08	30.06
OsR498G0509565100.01	30.803	11.776	-1.387679	1.62E-55	1.18E-55	30.67	32.45	29.29	10.65	13.34	11.34
OsR498G0509566000.01	14.423	32.306	1.1627604	1.05E-84	5.93E-85	15.21	13.62	14.44	33.1	31.44	32.38
OsR498G0509568900.01	0.323	1.823	2.4937841	8.74E-72	5.42E-72	0.25	0.39	0.33	1.91	1.89	1.67
OsR498G0509569200.01	6.423	29.616	2.1923473	2.78E-227	7.45E-228	6.72	5.46	7.09	30.41	29	29.44
OsR498G0509570500.01	5.536	1.633	-1.767699	6.27E-27	6.66E-27	5.35	5.59	5.67	1.56	1.38	1.96
OsR498G0509572300.01	221.256	40.336	-2.335445	0	0	223.1	217.86	222.81	40.22	40.89	39.9
OsR498G0509574300.01	1.12	3.71	1.738597	3.53E-20	4.22E-20	1.21	1.04	1.11	4.07	3.34	3.72
OsR498G0509576200.01	0.026	0.236	3.144335	2.13E-06	3.77E-06	0	0.05	0.03	0.16	0.23	0.32
OsR498G0509577900.01	58.126	12.493	-2.243244	0	0	58.1	59.18	57.1	14.33	11.33	11.82
OsR498G0509578400.01	57.54	121.79	1.0856902	0	0	56.53	58.61	57.48	123.85	124.31	117.2
OsR498G0509582000.01	0.4	0.976	1.2889206	1.70E-12	2.44E-12	0.49	0.39	0.32	0.93	0.84	1.16

OsR498G0509583500.01	2.89	11.276	1.9633548	8.11E-107	3.89E-107	3.16	2.7	2.81	10.71	11.31	11.81
OsR498G0509584100.01	126.176	50.136	-1.340801	2.40E-236	6.26E-237	128.29	126.45	123.79	48.11	45.88	56.42
OsR498G0509585100.01	7.593	30.79	2.0041063	4.77E-246	1.20E-246	7.73	7.79	7.26	31.08	30.62	30.67
OsR498G0509586000.01	8.213	23.053	1.5109553	2.42E-63	1.63E-63	9.62	7.43	7.59	24.16	23.22	21.78
OsR498G0509586200.01	1.263	2.856	1.271027	5.38E-14	7.39E-14	1.26	1.16	1.37	3.16	2.6	2.81
OsR498G0509588100.01	0.253	0.746	1.5488801	9.17E-12	1.34E-11	0.27	0.36	0.13	0.68	0.68	0.88
OsR498G0509589100.01	98.76	42.456	-1.223147	1.45E-207	4.22E-208	94.53	102.19	99.56	42.53	40.98	43.86
OsR498G0509592800.01	1.32	2.846	1.1099976	6.68E-15	8.98E-15	1.19	1.09	1.68	3.18	2.58	2.78
OsR498G0509596200.01	9.483	20.643	1.1129708	1.92E-49	1.49E-49	8.08	10.39	9.98	21.55	19.39	20.99
OsR498G0509597100.01	307.746	135.09	-1.177537	0	0	309.44	305.99	307.81	134.07	141.37	129.82
OsR498G0509597500.01	4.236	1.223	-1.795182	3.06E-60	2.11E-60	4.27	4.16	4.28	1.1	1.02	1.55
OsR498G0509600000.01	0	0.236	4.5593725	0.0002368	0.0004696	0	0	0	0.23	0.06	0.42
OsR498G0509600600.01	0.993	3.19	1.7163418	2.46E-26	2.64E-26	0.97	1.09	0.92	3.01	3.13	3.43
OsR498G0509601600.01	28.97	58.103	1.0043095	4.94E-160	1.78E-160	30.68	29.26	26.97	58.48	60.68	55.15
OsR498G0509604700.01	0.053	0.996	4.2070707	6.84E-22	7.91E-22	0	0.06	0.1	0.63	1.16	1.2
OsR498G0509606600.01	9.186	3.653	-1.328753	6.57E-49	5.12E-49	9.78	8.82	8.96	3.48	4	3.48
OsR498G0509610500.01	10.376	21.6	1.0572782	1.49E-152	5.53E-153	10.08	10.68	10.37	21.33	22.68	20.79
OsR498G0509614800.01	0.126	0.656	2.3529216	5.18E-05	9.85E-05	0.15	0.15	0.08	0.9	0.45	0.62
OsR498G0509615200.01	7.273	19.856	1.4476621	8.12E-64	5.43E-64	7.74	6.26	7.82	20.16	18.58	20.83
OsR498G0509616600.01	0.14	0	-4.832945	6.10E-05	0.0001167	0.14	0.14	0.14	0	0	0
OsR498G0509618800.01	16.07	43.88	1.4410037	3.71E-199	1.12E-199	14.85	16.5	16.86	44.89	41.52	45.23
OsR498G0509621700.01	6.09	42.573	2.6706574	0	0	6.08	6.1	6.09	40.73	46.39	40.6
OsR498G0509623500.01	76.763	154.58	1.0103945	0	0	76.29	80.13	73.87	158.24	149.28	156.23
OsR498G0509624200.01	30.186	80.243	1.4091029	0	0	30.91	29.59	30.06	79.99	81.85	78.89
OsR498G0509625500.01	2.61	5.9	1.1726796	5.15E-14	7.07E-14	3.23	2.58	2.02	4.84	5.09	7.77
OsR498G0509627400.01	2.06	4.723	1.1475589	3.13E-17	3.98E-17	2.12	2.1	1.96	4.47	5.24	4.46
OsR498G0509627800.01	0.276	0.066	-2.02559	0.000819	0.0016919	0.17	0.33	0.33	0.12	0.08	0
OsR498G0509629300.01	0.006	0.14	3.9286063	3.97E-08	6.54E-08	0.01	0.01	0	0.19	0.12	0.11
OsR498G0509630100.01	0.376	0.096	-1.964189	1.73E-06	3.06E-06	0.35	0.32	0.46	0.05	0.1	0.14
OsR498G0509631100.01	31.616	14.853	-1.090712	9.42E-110	4.43E-110	31.9	31.82	31.13	14.23	14.94	15.39
OsR498G0509633500.01	9.186	2.136	-2.126568	1.40E-99	7.04E-100	9.51	8.35	9.7	2.08	2.25	2.08
OsR498G0509642200.01	4.07	1.71	-1.366272	4.72E-35	4.39E-35	3.92	3.91	4.38	1.73	1.61	1.79
OsR498G0509647700.01	0.07	0.266	1.933768	3.47E-05	6.52E-05	0.07	0.09	0.05	0.2	0.27	0.33
OsR498G0509648000.01	9.286	29.616	1.6705418	1.28E-204	3.78E-205	9.94	8.83	9.09	30.83	28.26	29.76
OsR498G0509648100.01	0.616	1.946	1.662466	7.89E-16	1.04E-15	0.69	0.48	0.68	2.27	1.79	1.78
OsR498G0509650600.01	2.296	4.843	1.075812	6.24E-11	9.37E-11	3.09	2.06	1.74	4.44	5.07	5.02
OsR498G0509652000.01	2.196	9.363	2.0739414	2.77E-54	2.03E-54	2.21	1.51	2.87	10.01	7.8	10.28
OsR498G0509652100.01	1.346	3.386	1.3159073	1.79E-10	2.73E-10	1.57	1.12	1.35	2.96	3.32	3.88
OsR498G0509653300.01	0.993	0.053	-4.251658	1.92E-30	1.92E-30	1.08	1.02	0.88	0.05	0.02	0.09
OsR498G0509654700.01	0.273	1.47	2.4452014	1.14E-35	1.05E-35	0.27	0.23	0.32	1.49	1.33	1.59
OsR498G0509655300.01	0.883	4.283	2.2766422	2.57E-37	2.31E-37	0.81	1.12	0.72	4.29	4.01	4.55
OsR498G0509655800.01	1.563	4.41	1.4954198	3.42E-26	3.67E-26	1.69	1.4	1.6	3.71	4.25	5.27
OsR498G0509658500.01	0.303	4.02	3.7362502	1.44E-63	9.66E-64	0.45	0.18	0.28	4.16	3.03	4.87
OsR498G0509662700.01	4.48	18.666	2.0418702	1.91E-203	5.65E-204	5.08	4.05	4.31	19.01	18.21	18.78
OsR498G0509666100.01	45.33	21.226	-1.084705	3.83E-88	2.10E-88	44.38	46.73	44.88	22.03	20.81	20.84
OsR498G0509666500.01	1.186	2.883	1.3213324	8.06E-17	1.03E-16	1.29	1.35	0.92	2.91	2.91	2.83
OsR498G0509668100.01	25.493	69.913	1.4538901	7.09E-169	2.44E-169	25.7	25.36	25.42	68.52	69.45	71.77
OsR498G0509671400.01	0.35	0.943	1.3788002	2.92E-07	5.00E-07	0.4	0.31	0.34	0.88	0.91	1.04
OsR498G0509672600.01	9.523	2.523	-1.918049	4.16E-133	1.71E-133	9.7	9.72	9.15	2.47	2.74	2.36
OsR498G0509676800.01	30.503	11.733	-1.377528	3.55E-101	1.77E-101	31.4	31.24	28.87	12.03	12.23	10.94
OsR498G0509681700.01	0.31	0.07	-1.900059	0.0007746	0.0015958	0.44	0.33	0.16	0.11	0.1	0
OsR498G0509688700.01	24.53	53.97	1.1400798	1.23E-254	2.97E-255	24.85	25.05	23.69	54.01	55.28	52.62
OsR498G0509689200.01	32.333	97.103	1.5818312	0	0	32.57	33.54	30.89	95.25	99.56	96.5
OsR498G0509694300.01	8.206	22.443	1.4954846	1.48E-126	6.32E-127	9.09	7.81	7.72	22.02	21.78	23.53
OsR498G0509696100.01	27.753	4.666	-2.578195	0	0	27.92	28.5	26.84	4.85	4.71	4.44
OsR498G0509696500.01	116.723	340.37	1.5293915	0	0	121.02	113.15	116	350.95	336.36	333.8
OsR498G0509696800.01	2.23	7	1.6488946	3.75E-40	3.25E-40	2.45	1.81	2.43	5.96	7.14	7.9
OsR498G0509698300.01	0.55	1.57	1.308829	5.54E-05	0.0001058	0.38	0.3	0.97	0.68	2.09	1.94
OsR498G0509699400.01	45.94	345.63	2.9070076	0	0	42.77	47.08	47.97	353.04	334.85	348.99
OsR498G0509700500.01	0.463	1.296	1.4769103	1.59E-07	2.69E-07	0.51	0.32	0.56	1.09	1.54	1.26
OsR498G0509702300.01	4.826	19.11	1.9517494	1.24E-153	4.57E-154	4.61	4.26	5.61	18.77	18.22	20.34
OsR498G0509702800.01	0.45	1.453	1.6922667	3.97E-12	5.74E-12	0.25	0.57	0.53	1.35	1.55	1.46
OsR498G0509703600.01	0.08	0.493	2.6066782	3.65E-06	6.54E-06	0	0.05	0.19	0.38	0.66	0.44
OsR498G0509708800.01	0.116	3.043	4.802229	6.07E-34	5.74E-34	0.07	0.14	0.14	3.16	3.27	2.7
OsR498G0509710100.01	23.063	56.516	1.2926872	3.25E-165	1.14E-165	20.87	23.31	25.01	59.33	55.4	54.82
OsR498G0509717400.01	1.533	3.77	1.2963381	1.33E-07	2.24E-07	1.43	1.75	1.42	3.74	3.52	4.05
OsR498G0509718400.01	0.276	5.533	4.2963381	1.20E-40	1.04E-40	0.09	0.37	0.37	5.49	4.83	6.28
OsR498G0509718700.01	0.1	0.75	2.8813006	0.000469	0.0009493	0	0	0.3	0.45	0.74	1.06
OsR498G0509720600.01	419.523	193.39	-1.114719	0	0	413.53	422.32	422.72	193.38	193.7	193.08

OsR498G0509722600.01	2.523	6.766	1.4226838	2.45E-53	1.81E-53	2.6	2.66	2.31	7.19	7.02	6.09
OsR498G0509722700.01	17.7	126	2.8810302	0	0	18.17	17.08	17.85	127.19	126.07	124.73
OsR498G0509725700.01	0.146	0.556	1.9123839	3.72E-08	6.13E-08	0.11	0.22	0.11	0.56	0.65	0.46
OsR498G0509726000.01	3.563	9.67	1.4707346	2.03E-114	9.33E-115	3.62	3.26	3.81	10.35	9.57	9.09
OsR498G0509726400.01	3.573	18.066	2.323788	7.01E-227	1.88E-227	3.42	3.61	3.69	17.95	18.4	17.85
OsR498G0509732900.01	3.516	7.606	1.1036205	5.75E-50	4.42E-50	3.48	3.16	3.91	7.52	7.75	7.55
OsR498G0509733400.01	4.08	18.89	2.2127088	5.80E-252	1.41E-252	3.79	4.47	3.98	18.6	18.69	19.38
OsR498G0509733700.01	3.983	21.026	2.4174582	0	0	4.16	4.23	3.56	21.6	21.11	20.37
OsR498G0509733900.01	0.14	0.463	1.7123671	3.19E-06	5.71E-06	0.03	0.13	0.26	0.39	0.47	0.53
OsR498G0509734200.01	0.39	0.866	1.1471108	8.35E-05	0.000161	0.27	0.39	0.51	0.88	0.82	0.9
OsR498G0509735900.01	3.216	11.096	1.7838452	1.18E-32	1.14E-32	3.52	2.82	3.31	9.24	11.95	12.1
OsR498G0509736900.01	0.273	1.493	2.8646218	1.09E-35	1.00E-35	0.34	0.24	0.24	1.54	1.59	1.35
OsR498G0509738400.01	1.993	0.62	-1.691101	1.33E-12	1.90E-12	2.19	1.97	1.82	0.65	0.55	0.66
OsR498G0509738700.01	14.443	32.753	1.1798247	2.39E-117	1.07E-117	14.61	13.89	14.83	32.95	30.64	34.67
OsR498G0509745600.01	1.756	4.406	1.3349234	1.49E-42	1.25E-42	1.62	2.07	1.58	4.88	4.41	3.93
OsR498G0509746000.01	0.156	0.456	1.5203783	3.91E-12	5.65E-12	0.14	0.17	0.16	0.42	0.6	0.35
OsR498G0509747600.01	10.183	4.47	-1.194884	2.49E-115	1.14E-115	9.85	10.37	10.33	4.36	4.48	4.57
OsR498G0509749400.01	2.62	11.603	2.1770011	2.33E-71	1.45E-71	2.1	3.49	2.27	11.71	11.56	11.54
OsR498G0509750100.01	4.66	12.323	1.4380662	2.01E-57	1.43E-57	4.84	4.26	4.88	13.29	11.9	11.78
OsR498G0509751300.01	0.44	0.136	-1.691853	6.64E-08	1.11E-07	0.46	0.54	0.32	0.18	0.14	0.09
OsR498G0509767300.01	10.756	23.973	1.1520749	2.94E-64	1.96E-64	11.48	10.03	10.76	23.97	23.7	24.25
OsR498G0509768100.01	15.856	97.913	2.624137	0	0	15.9	16.89	14.78	99.09	96.14	98.51
OsR498G0509769000.01	6.146	1.13	-2.442908	1.01E-58	7.05E-59	5.49	5.96	6.99	1.11	1.32	0.96
OsR498G0509773600.01	3.833	1.816	-1.06701	2.02E-19	2.46E-19	3.62	4.23	3.65	1.82	1.6	2.03
OsR498G0509775700.01	0.976	0.246	-1.99074	9.74E-14	1.35E-13	1.17	1	0.76	0.3	0.12	0.32
OsR498G0509776200.01	0.1	0	-4.72603	0.0001114	0.0002167	0.14	0.05	0.11	0	0	0
OsR498G0509778900.01	0.013	0.22	4.144335	1.55E-05	2.86E-05	0	0	0.04	0.22	0.33	0.11
OsR498G0509780300.01	7.826	16.393	1.1066677	1.17E-91	6.24E-92	8.3	6.91	8.27	17.61	15.74	15.83
OsR498G0509784900.01	21.163	59.113	1.4723277	2.52E-220	6.90E-221	19.92	20.31	23.26	61.34	57.12	58.88
OsR498G0509785200.01	3.096	13.19	2.090697	6.29E-132	2.61E-132	3.48	2.83	2.98	13.82	13.47	12.28
OsR498G0509785400.01	0.27	0.07	-1.973123	0.0001708	0.000336	0.24	0.15	0.42	0.09	0.06	0.06
OsR498G0509788100.01	33.56	119.62	1.8314903	0	0	33.19	32.02	35.47	118.07	110.25	130.55
OsR498G0509788400.01	4.66	1.29	-1.796649	3.63E-16	4.74E-16	4.38	5.24	4.36	1.34	1.25	1.28
OsR498G0509789100.01	0.663	2.823	2.0935542	1.85E-36	1.69E-36	0.78	0.54	0.67	2.62	2.91	2.94
OsR498G0509793900.01	51.593	1.233	-5.388782	0	0	51.41	50.69	52.68	0.96	1.36	1.38
OsR498G0509796100.01	0.15	0.033	-2.125126	3.89E-05	7.34E-05	0.12	0.13	0.2	0.04	0.04	0.02
OsR498G0509797500.01	28.88	66.726	1.2018334	0	0	29.54	29.02	28.08	67.58	68.19	64.41
OsR498G0509798800.01	0.303	0.043	-2.832945	0.0006839	0.0014027	0.17	0.44	0.3	0.07	0	0.06
OsR498G0509799000.01	27.69	60.806	1.1744363	0	0	28.12	26.76	28.19	60.18	63.55	58.69
OsR498G0509800400.01	21.31	92.463	2.1499363	0	0	21.37	21.6	20.96	93.3	90.54	93.55
OsR498G0509801900.01	162.41	75.47	-1.105471	0	0	162.29	165.37	159.57	76.08	78.46	71.87
OsR498G0509802100.01	5.266	1.23	-2.092888	1.61E-43	1.34E-43	5.41	5.31	5.08	1.11	1.52	1.06
OsR498G0509804600.01	1.026	3.853	1.9274667	1.59E-78	9.41E-79	0.93	1.05	1.1	4.05	4.07	3.44
OsR498G0509811000.01	0.683	0.02	-5.055337	9.69E-25	1.06E-24	0.55	0.87	0.63	0.04	0	0.02
OsR498G0509812900.01	0.226	1.13	2.2963381	2.01E-05	3.75E-05	0	0.57	0.11	1.01	1.23	1.15
OsR498G0509820600.01	10.476	36.543	1.8019927	0	0	10.78	10.47	10.18	36.02	36.22	37.39
OsR498G0509823300.01	72.636	274.28	1.8904537	0	0	68.47	72.96	76.48	269.03	278.03	275.77
OsR498G0509825800.01	0.353	0.17	-1.427688	0.0004179	0.0008427	0.25	0.35	0.46	0.14	0.22	0.15
OsR498G0509827300.01	0.113	0.676	2.5593725	8.67E-07	1.52E-06	0	0.17	0.17	0.78	0.57	0.68
OsR498G0509827700.01	0.003	0.106	3.6748497	0.0003128	0.0006259	0	0	0.01	0.08	0.23	0.01
OsR498G0509830800.01	236.45	92.526	-1.36922	0	0	234.01	240.57	234.77	95.45	91.76	90.37
OsR498G0509831100.01	475.62	71.016	-2.734877	0	0	466.72	486.99	473.15	69.42	72.47	71.16
OsR498G0509831500.01	0.213	0.726	1.7604802	2.26E-11	3.33E-11	0.33	0.09	0.22	0.81	0.78	0.59
OsR498G0509832300.01	1.09	0.526	-1.0745	8.62E-07	1.51E-06	1.07	1.1	1.1	0.65	0.62	0.31
OsR498G0509838800.01	7.673	1.963	-2.032347	7.27E-94	3.81E-94	8.27	7.24	7.51	1.9	2.07	1.92
OsR498G0509846900.01	1.126	3.11	1.4529504	1.22E-15	1.61E-15	0.5	1.2	1.68	3.22	3.35	2.76
OsR498G0509848400.01	0.973	2.53	1.4006747	7.28E-10	1.13E-09	0.89	1.1	0.93	2.36	2.82	2.41
OsR498G0509848500.01	4.43	10.666	1.2617578	1.35E-21	1.57E-21	4.58	4.44	4.27	9.57	9.88	12.55
OsR498G0509849600.01	0.713	0.026	-4.564749	7.67E-23	8.71E-23	0.83	0.55	0.76	0.04	0.02	0.02
OsR498G0509849900.01	1.913	0.72	-1.418932	3.04E-13	4.25E-13	1.77	2.01	1.96	0.61	0.76	0.79
OsR498G0509856700.01	1.943	7.996	2.0653836	1.70E-79	9.96E-80	2.01	1.89	1.93	7.82	7.42	8.75
OsR498G0509858900.01	1.13	2.966	1.3894475	1.07E-06	1.87E-06	1.02	0.9	1.47	3.65	2.2	3.05
OsR498G0509860600.01	4.866	1.703	-1.512856	1.79E-35	1.65E-35	5.24	4.81	4.55	2.18	1.5	1.43
OsR498G0509868200.01	0.426	1.923	2.1838633	4.98E-06	9.00E-06	0.48	0.48	0.32	1.86	1.54	2.37
OsR498G0509868500.01	5.173	13.936	1.4390514	1.23E-141	4.85E-142	4.99	5.33	5.2	14.45	13.61	13.75
OsR498G0509869000.01	21.536	6.37	-1.753823	1.71E-103	8.39E-104	23.71	20.93	19.97	6.49	6.86	5.76
OsR498G0509869200.01	0.45	15.806	5.1111585	0	0	0.5	0.52	0.33	15.16	15.78	16.48
OsR498G0509871400.01	1.336	31.703	4.5843631	0	0	1.44	1.19	1.38	32.89	30.29	31.93
OsR498G0509871700.01	0.253	10.633	5.3752894	4.45E-41	3.82E-41	0.38	0.19	0.19	9.57	9.91	12.42

OsR498G0509872600.01	5.516	11.646	1.0761486	8.93E-33	8.59E-33	5.39	5.57	5.59	11.94	10.83	12.17
OsR498G0509873100.01	2.346	5.26	1.1775018	6.37E-35	5.94E-35	2.29	2.4	2.35	5.44	5.18	5.16
OsR498G0509873600.01	0.826	3.353	2.021331	2.62E-54	1.92E-54	1.13	0.68	0.67	3.37	3.43	3.26
OsR498G0509875200.01	8.783	53.48	2.6248992	0	0	9.01	9.26	8.08	53.03	53.9	53.51
OsR498G0509901500.01	5.816	0.856	-2.766347	1.04E-60	7.13E-61	5.71	5.7	6.04	0.89	0.67	1.01
OsR498G0509916000.01	0.123	0.46	2.144335	8.17E-06	1.49E-05	0.1	0.17	0.1	0.49	0.41	0.48
OsR498G0509917000.01	0.426	21.693	5.6600348	0	0	0.33	0.51	0.44	22.96	21.76	20.36
OsR498G0509917400.01	1.693	6.963	2.038982	7.26E-65	4.80E-65	1.33	1.78	1.97	7.57	6.36	6.96
OsR498G0509918900.01	0.433	4.253	3.2901858	1.28E-85	7.14E-86	0.36	0.44	0.5	4.11	3.95	4.7
OsR498G0509920800.01	20.77	5.78	-1.877844	4.26E-166	1.49E-166	20.69	20.11	21.51	5.78	5.83	5.73
OsR498G0509935200.01	0.75	10.776	3.8455549	6.83E-235	1.79E-235	0.86	0.74	0.65	10.2	11.06	11.07
OsR498G0509943900.01	13.513	36.66	1.4418275	9.67E-135	3.94E-135	13.5	12.82	14.22	35.11	36.24	38.63
OsR498G0509953400.01	1.4	0.326	-2.109006	7.34E-12	1.07E-11	1.8	1.13	1.27	0.28	0.32	0.38
OsR498G0509955000.01	0.493	1.043	1.0358105	1.88E-05	3.49E-05	0.54	0.66	0.28	1.3	0.93	0.9
OsR498G0509955200.01	20.24	7.226	-1.72944	2.93E-148	1.11E-148	21.92	20.56	18.24	8.59	7.16	5.93
OsR498G0509956400.01	3.06	0.39	-2.979786	3.08E-08	5.07E-08	2.85	3.07	3.26	0.47	0.46	0.24
OsR498G0509960000.01	10.16	21.68	1.0904562	2.62E-55	1.90E-55	9.38	10.17	10.93	19.93	20.35	24.76
OsR498G0509967100.01	2.163	0	-7.347518	1.11E-19	1.34E-19	2.12	1.7	2.67	0	0	0
OsR498G0509968900.01	0.893	4.58	2.3517148	3.87E-32	3.77E-32	0.76	0.99	0.93	4.44	3.63	5.67
OsR498G0509970000.01	0.6	0.11	-2.375617	2.00E-11	2.96E-11	0.15	0.08	1.57	0.19	0.04	0.1
OsR498G0509971500.01	0.38	0	-5.273518	3.18E-06	5.68E-06	0.66	0.3	0.18	0	0	0
OsR498G0509978600.01	23.196	7.276	-1.789839	0	0	23.61	22.48	23.5	6.95	7.42	7.46
OsR498G0509982500.01	8.886	21.56	1.2786729	1.37E-118	6.13E-119	8.83	8.68	9.15	20.19	23.04	21.45
OsR498G0509990500.01	5.76	40.423	2.8193105	0	0	5.78	6.13	5.37	40.06	41.51	39.7
OsR498G0509991200.01	2.246	4.856	1.1200153	7.07E-24	7.89E-24	2.2	2.18	2.36	4.95	5.24	4.38
OsR498G0509996500.01	2.296	5.953	1.3714395	9.80E-23	1.12E-22	2.65	2.05	2.19	6.17	5.47	6.22
OsR498G0510001600.01	0	0.66	5.6297618	1.19E-07	2.01E-07	0	0	0	0.8	0.54	0.64
OsR498G0510002400.01	0.513	1.506	1.5481452	4.24E-11	6.33E-11	0.47	0.5	0.57	1.22	1.43	1.87
OsR498G0510013900.01	30.1	11.99	-1.559474	7.48E-215	2.10E-215	31.43	30.3	28.57	11.29	11.86	12.82
OsR498G0510015900.01	25.343	9.97	-1.320879	3.62E-184	1.16E-184	26.15	25.76	24.12	9.82	9.22	10.87
OsR498G0510017200.01	29.936	0.24	-7.191055	1.30E-292	2.80E-293	30.44	30.74	28.63	0.14	0.41	0.17
OsR498G0510026100.01	1.923	0.683	-1.491254	8.60E-06	1.57E-05	1.9	1.79	2.08	0.87	0.68	0.5
OsR498G0510044200.01	13.21	31.613	1.2056587	3.86E-123	1.67E-123	13.47	12.83	13.33	32.49	31.16	31.19
OsR498G0510046200.01	2.386	6.706	1.5252793	1.67E-34	1.57E-34	2.27	2.62	2.27	6.86	6.75	6.51
OsR498G0510053600.01	35.73	89.816	1.3029993	3.26E-226	8.78E-227	37.71	36.15	33.33	85.3	85.74	98.41
OsR498G0510059900.01	7.676	15.43	1.006294	6.29E-29	6.44E-29	7.95	7.18	7.9	15.69	14.75	15.85
OsR498G0510071600.01	8.183	23.726	1.5270985	4.92E-187	1.56E-187	8.9	7.61	8.04	24.07	23.67	23.44
OsR498G0510071700.01	0.65	1.696	1.4532441	1.84E-10	2.80E-10	0.53	0.74	0.68	1.65	1.91	1.53
OsR498G0510072400.01	0	0.133	4.4338416	0.0004331	0.0008746	0	0	0	0.11	0.15	0.14
OsR498G0510081500.01	16.703	42.466	1.3472055	0	0	17.32	16.9	15.89	42.64	43.46	41.3
OsR498G0510083100.01	70.62	27.256	-1.343795	0	0	66.66	74.21	70.99	25.89	26.58	29.3
OsR498G0510089500.01	0.413	1.426	1.7817649	4.35E-06	7.84E-06	0.36	0.26	0.62	1.12	0.95	2.21
OsR498G0510116600.01	0.266	3.283	3.6182358	1.30E-93	6.84E-94	0.23	0.25	0.32	3.35	3.22	3.28
OsR498G0510123000.01	4.463	10.133	1.1837953	1.41E-65	9.27E-66	4.33	4.6	4.46	9.95	10.53	9.92
OsR498G0510123300.01	1.12	3.126	1.4021491	3.37E-38	3.00E-38	1.2	1.34	0.82	3.05	3.6	2.73
OsR498G0510124100.01	8.68	2.036	-2.060566	5.84E-75	3.54E-75	8.13	9.2	8.71	1.63	2.24	2.24
OsR498G0510125200.01	1.7	3.36	1.0845929	1.85E-07	3.15E-07	1.73	1.65	1.72	3.33	3.35	3.4
OsR498G0510127200.01	0.546	1.373	1.5120667	4.35E-08	7.19E-08	0.58	0.75	0.31	1.32	1.56	1.24
OsR498G0510128800.01	0	0.453	5.6748497	7.66E-08	1.28E-07	0	0	0	0.29	0.44	0.63
OsR498G0510130300.01	0.383	1.263	2.144335	4.95E-13	6.97E-13	0.38	0.33	0.44	1.22	1.31	1.26
OsR498G0510133000.01	0.816	4.636	2.670575	3.89E-51	2.96E-51	0.55	0.79	1.11	4.83	4.85	4.23
OsR498G0510166100.01	5.42	14.07	1.1926467	1.78E-52	1.33E-52	5.18	5.13	5.95	14.37	13.26	14.58
OsR498G0510172500.01	8.81	22.723	1.3568013	1.59E-236	4.13E-237	9.08	8.17	9.18	22.62	23.42	22.13
OsR498G0510174900.01	0.203	0.566	1.7746404	1.04E-10	1.56E-10	0.29	0.16	0.16	0.54	0.57	0.59
OsR498G0510177300.01	0.41	2.383	2.5426937	1.06E-25	1.14E-25	0.51	0.38	0.34	2.36	2.92	1.87
OsR498G0510177900.01	0.436	0.083	-2.347518	2.97E-06	5.31E-06	0.34	0.52	0.45	0.11	0.07	0.07
OsR498G0510192800.01	0.77	2.976	2.4699374	4.63E-94	2.43E-94	0.61	0.86	0.84	3.01	3.05	2.87
OsR498G0510195400.01	0.02	0.073	1.9124768	0.0007097	0.0014579	0.02	0.01	0.03	0.08	0.07	0.07
OsR498G0510210400.01	8.14	2.026	-1.947305	1.47E-56	1.06E-56	8.45	8	7.97	2.36	1.43	2.29
OsR498G0510224000.01	21.113	48.13	1.1891936	4.66E-252	1.14E-252	20.8	21.27	21.27	49.16	49.39	45.84
OsR498G0510225500.01	3.43	8.436	1.2900058	1.23E-20	1.46E-20	3.34	3.06	3.89	8.52	7.16	9.63
OsR498G0510234100.01	0.25	0.83	1.7113756	1.22E-07	2.06E-07	0.18	0.32	0.25	0.6	1.02	0.87
OsR498G0510241100.01	0.72	2.23	1.7250505	8.26E-20	9.95E-20	0.91	0.62	0.63	2.43	2.26	2
OsR498G0510242400.01	1.776	0.516	-1.783617	3.29E-12	4.75E-12	1.54	2.12	1.67	0.27	0.54	0.74
OsR498G0510244700.01	2.09	4.32	1.0437026	7.72E-33	7.42E-33	1.85	2.36	2.06	4.17	4.85	3.94
OsR498G0510247500.01	5.99	29.523	2.3001674	8.37E-188	2.64E-188	6.48	5.41	6.08	29.02	30.17	29.38
OsR498G0510250400.01	8.8	22.966	1.3816184	5.87E-118	2.63E-118	9.73	7.86	8.81	23.58	22.66	22.66
OsR498G0510266000.01	1.7	17.566	3.3692325	7.50E-265	1.75E-265	1.98	1.52	1.6	17.68	18	17.02
OsR498G0510271100.01	0.43	0	-6.347518	3.18E-11	4.73E-11	0.39	0.45	0.45	0	0	0

OsR498G0510286200.01	0.346	0.853	1.2598122	0.0009737	0.0020275	0.38	0.32	0.34	0.87	0.91	0.78
OsR498G0510322300.01	5.57	47.82	3.1069691	0	0	4.99	6	5.72	48.28	47.74	47.44
OsR498G0510344200.01	253.15	72.523	-1.805736	0	0	256.09	247.53	255.83	71.75	70.87	74.95
OsR498G0510358500.01	2.006	4.133	1.0576931	1.33E-22	1.51E-22	1.95	2.27	1.8	4.63	4.13	3.64
OsR498G0510362200.01	0.14	1.043	2.9184536	3.09E-30	3.10E-30	0.15	0.13	0.14	0.97	1.27	0.89
OsR498G0510373100.01	0.706	1.8	1.33698	1.46E-17	1.84E-17	0.72	0.74	0.66	1.78	1.69	1.93
OsR498G0510378100.01	3.143	11.796	1.9116023	4.67E-85	2.62E-85	3.66	2.55	3.22	11.61	11.83	11.95
OsR498G0510384700.01	13.313	28.31	1.0851921	2.14E-166	7.45E-167	13.8	13.21	12.93	27.88	27.8	29.25
OsR498G0510392500.01	8.63	19.23	1.1614955	2.16E-105	1.05E-105	8.61	9.13	8.15	19.31	20.3	18.08
OsR498G0510399200.01	9.546	37.716	1.9815086	0	0	9.66	9.62	9.36	37.54	36.93	38.68
OsR498G0510406400.01	3.69	11.336	1.7515186	2.82E-73	1.73E-73	3.7	3.7	3.67	11.06	10.27	12.68
OsR498G0510408400.01	1.383	0.326	-2.087745	9.27E-13	1.31E-12	1.48	1.35	1.32	0.36	0.43	0.19
OsR498G0510408500.01	15.913	2.88	-2.464953	0	0	16.61	15.23	15.9	3.15	2.8	2.69
OsR498G0510414500.01	5.713	34.423	2.5874001	0	0	5.49	5.91	5.74	34.98	35.95	32.34
OsR498G0510426700.01	0.056	0.01	-2.681759	0.0008808	0.001826	0.02	0.09	0.06	0	0.03	0
OsR498G0510434500.01	7.413	2.043	-1.838919	1.43E-81	8.24E-82	7.27	7.47	7.5	2.27	1.82	2.04
OsR498G0510436100.01	27.386	63.39	1.2017403	3.97E-261	9.38E-262	26.72	28.11	27.33	63.67	63.54	62.96
OsR498G0510436600.01	6.92	23.683	1.7752331	4.31E-157	1.57E-157	7.1	7.47	6.19	24.92	23.02	23.11
OsR498G0510436900.01	0.19	0.626	1.7312751	0.0003588	0.0007199	0.57	0	0	0.76	0.56	0.56
OsR498G0510442200.01	0	0.316	4.97441	2.21E-05	4.12E-05	0	0	0	0.12	0.53	0.3
OsR498G0510446000.01	5.41	2.223	-1.287175	6.05E-13	8.54E-13	5.65	4.88	5.7	1.75	1.9	3.02
OsR498G0510448200.01	0	1.183	7.2411965	7.74E-19	9.50E-19	0	0	0	1.41	1.15	0.99
OsR498G0510449400.01	2.64	6.413	1.3265909	5.78E-58	4.08E-58	2.87	2.71	2.34	6.32	6.63	6.29
OsR498G0510455000.01	0.4	0.813	1.0236535	1.94E-07	3.29E-07	0.47	0.42	0.31	0.71	0.76	0.97
OsR498G0510456400.01	31.966	71.39	1.1562662	6.92E-113	3.21E-113	31.19	33.26	31.45	71.97	71.29	70.91
OsR498G0510468900.01	5.363	31.063	2.5210975	0	0	5.42	5.04	5.63	31.68	29.63	31.88
OsR498G0510477100.01	64.2	12.706	-2.280397	0	0	57.29	64.8	70.51	12.97	13.15	12
OsR498G0510479400.01	9.226	17.89	1.0050057	9.52E-104	4.66E-104	8.44	9.55	9.69	19.1	18.12	16.45
OsR498G0510486800.01	24.576	169.6	2.8050123	0	0	25.28	24.05	24.4	167.08	166.26	175.46
OsR498G0510489300.01	1.566	0.243	-2.695441	2.38E-12	3.42E-12	1.42	1.47	1.81	0.13	0.26	0.34
OsR498G0510489900.01	0.06	0.27	2.1760438	0.0002409	0.0004782	0	0.04	0.14	0.39	0.35	0.07
OsR498G0510492800.01	0.773	1.956	1.331962	1.68E-06	2.98E-06	0.65	0.65	1.02	2.08	1.67	2.12
OsR498G0510500000.01	1.083	38.946	5.1659275	2.85E-296	6.09E-297	1.03	1.39	0.83	40.59	40.15	36.1
OsR498G0510502800.01	3.56	1.173	-1.648688	9.27E-30	9.36E-30	3.22	3.61	3.85	1.27	1.15	1.1
OsR498G0510508400.01	0.533	1.81	1.8255466	1.05E-18	1.29E-18	0.43	0.61	0.56	1.39	2	2.04
OsR498G0510525000.01	0.706	7.826	3.4650451	1.06E-167	3.68E-168	0.49	0.88	0.75	7.8	8.31	7.37
OsR498G0510530600.01	8.06	18.793	1.220998	5.59E-97	2.87E-97	8.48	7.76	7.94	19.08	17.37	19.93
OsR498G0510532200.01	0.633	1.776	1.4889831	0.0001951	0.0003849	0.82	0.27	0.81	0.93	2.37	2.03
OsR498G0510532800.01	19.063	44.363	1.2743926	3.03E-226	8.14E-227	19.93	19.37	17.89	45.08	44.86	43.15
OsR498G0510544200.01	4.403	19.96	2.1530172	1.85E-257	4.42E-258	4.09	4.31	4.81	20.05	19.22	20.61
OsR498G0510549600.01	43.696	132.78	1.5996996	0	0	44.28	44.63	42.18	135.19	124.78	138.37
OsR498G0510554500.01	0.293	0.09	-1.73513	0.0001432	0.0002803	0.4	0.27	0.21	0.05	0.08	0.14
OsR498G0510555700.01	4.916	13.46	1.5157112	3.50E-52	2.63E-52	4.89	5.89	3.97	13.72	13.27	13.39
OsR498G0510566000.01	0.093	0.26	1.4769103	0.0005606	0.0011403	0.07	0.12	0.09	0.32	0.25	0.21
OsR498G0510567700.01	5.543	76.313	3.7793707	0	0	5.31	5.33	5.99	76.48	73.5	78.96
OsR498G0510577900.01	0.576	1.306	1.0780326	6.36E-06	1.16E-05	0.5	0.61	0.62	1.31	1.4	1.21
OsR498G0510578500.01	5.266	16.946	1.803462	2.23E-113	1.03E-113	6.94	4.33	4.53	24.74	3.45	22.65
OsR498G0510579100.01	71.45	8.716	-3.017527	0	0	71.97	70.19	72.19	0	26.15	0
OsR498G0510580000.01	1.556	0.22	-2.72603	1.03E-11	1.51E-11	1.27	1.52	1.88	0	0.26	0.4
OsR498G0510585900.01	6.02	14.786	1.2967667	3.20E-95	1.67E-95	6.91	5.6	5.55	15.04	14.95	14.37
OsR498G0510586600.01	16.006	97.85	2.6159975	0	0	14.92	16.88	16.22	97.53	92.68	103.34
OsR498G0510586700.01	36.013	91.836	1.3496929	1.10E-296	2.35E-297	34.44	36.63	36.97	93.6	90.1	91.81
OsR498G0510598100.01	30.71	84.536	1.4490639	0	0	30.54	31.34	30.25	85.11	84.88	83.62
OsR498G0510598100.01.	0	0.503	6.0512256	1.18E-09	1.84E-09	0	0	0	0.34	0.47	0.7
OsR498G0510601700.01	6.74	31.77	2.2685696	0	0	6.27	7.63	6.32	33.49	31.12	30.7
OsR498G0510602300.01	36.093	72.5	1.0050859	1.51E-166	5.24E-167	34.95	36.43	36.9	69.31	73.34	74.85
OsR498G0510613500.01	0.8	2.62	1.7037624	1.46E-38	1.29E-38	0.81	0.86	0.73	2.62	2.59	2.65
OsR498G0510615700.01	0.133	0.44	1.7292975	0.0005682	0.0011565	0.15	0.1	0.15	0.68	0.39	0.25
OsR498G0510617300.01	4.09	8.77	1.1337288	1.50E-91	8.02E-92	4.09	4.25	3.93	8.73	9.18	8.4
OsR498G0510626000.01	9.893	41.843	2.0788767	5.33E-243	1.35E-243	10.4	8.65	10.63	40.69	39.78	45.06
OsR498G0510637000.01	11.116	24.266	1.1072919	1.04E-90	5.59E-91	10.84	11.56	10.95	25.93	23.25	23.62
OsR498G0510637200.01	0.673	5.81	3.2161001	4.42E-103	2.18E-103	0.46	0.59	0.97	5.53	6	5.9
OsR498G0510638400.01	0.41	1.266	1.6280852	6.93E-12	1.01E-11	0.38	0.47	0.38	1.2	1.4	1.2
OsR498G0510641200.01	15.92	45.613	1.5243218	0	0	15.79	16.5	15.47	46.05	47.97	42.82
OsR498G0510658800.01	1.11	3.84	1.7346309	5.41E-25	5.92E-25	0.9	1.63	0.8	3.94	3.79	3.79
OsR498G0510660100.01	2.783	6.293	1.1740823	8.37E-16	1.10E-15	2.62	2.8	2.93	5.73	5.9	7.25
OsR498G0510662600.01	9.326	211.69	4.51201	0	0	8.76	9.75	9.47	206.5	208.16	220.41
OsR498G0510666200.01	3.853	7.973	1.0476359	4.82E-24	5.36E-24	3.92	3.75	3.89	8.78	6.89	8.25
OsR498G0510671000.01	2.586	7.423	1.519406	2.14E-14	2.91E-14	2.81	1.91	3.04	7.83	6.56	7.88

OsR498G0510673800.01	2.59	0.38	-2.75993	1.34E-114	6.12E-115	2.2	2.86	2.71	0.43	0.38	0.33
OsR498G0510674100.01	0.74	2.313	1.6403613	1.21E-33	1.15E-33	0.63	0.66	0.93	2.08	2.12	2.74
OsR498G0510674700.01	0.483	1.65	1.7741113	2.09E-18	2.58E-18	0.56	0.43	0.46	1.78	1.53	1.64
OsR498G0510676300.01	0.013	0.26	4.7292975	7.57E-08	1.26E-07	0	0	0.04	0.28	0.18	0.32
OsR498G0510677300.01	0.1	1.896	4.3280469	1.18E-41	1.01E-41	0.1	0.13	0.07	1.72	1.7	2.27
OsR498G0510677900.01	87.503	392.42	2.1481134	0	0	89.59	87.04	85.88	395.86	391.2	390.2
OsR498G0510679800.01	3.316	8.483	1.354441	2.02E-24	2.23E-24	3.29	3.53	3.13	8.38	8.54	8.53
OsR498G0510680000.01	1.236	4.516	1.873647	3.67E-24	4.07E-24	1.36	1.41	0.94	5.16	4.97	3.42
OsR498G0510680800.01	2.843	10.77	1.8981744	4.05E-64	2.70E-64	2.67	2.85	3.01	11.21	10.91	10.19
OsR498G0510685100.01	2.733	16.803	2.6153779	2.07E-144	7.99E-145	2.73	2.86	2.61	17.75	16.93	15.73
OsR498G0510689400.01	0.123	0.923	2.9491784	1.82E-18	2.25E-18	0.14	0.06	0.17	1.24	0.98	0.55
OsR498G0510689500.01	2.226	8.083	1.8577137	1.79E-27	1.88E-27	2.54	1.98	2.16	7.94	8.35	7.96
OsR498G0510694200.01	0.096	0.743	2.9378841	4.48E-08	7.41E-08	0	0.17	0.12	0.91	0.56	0.76
OsR498G0510697200.01	5.92	12.536	1.0803434	1.25E-20	1.48E-20	6.71	5.31	5.74	11.81	12.17	13.63
OsR498G0510698400.01	2.766	6.476	1.2272691	3.19E-52	2.39E-52	2.56	2.86	2.88	7.04	6.39	6
OsR498G0510699100.01	6.18	13.853	1.2223375	4.33E-46	3.48E-46	6.92	6.51	5.11	13.35	13.09	15.12
OsR498G0510702700.01	0.47	1.436	1.656234	3.79E-14	5.19E-14	0.61	0.33	0.47	1.58	1.52	1.21
OsR498G0510703100.01	1.37	6.316	2.2192475	1.13E-70	7.07E-71	1.75	1.11	1.25	6.52	6.56	5.87
OsR498G0510704100.01	11.383	50.1	2.130458	0	0	11.03	11.84	11.28	49.32	50.27	50.71
OsR498G0510710800.01	23.556	5.746	-2.041825	2.32E-214	6.52E-215	24.35	22.77	23.55	5.71	4.63	6.9
OsR498G0510717000.01	1.586	7.026	2.1469604	4.95E-33	4.75E-33	1.57	1.09	2.1	7.45	6.06	7.57
OsR498G0510737900.01	0.186	0.02	-3.113053	0.0001176	0.0002292	0.36	0.13	0.07	0.03	0.03	0
OsR498G0510739700.01	0.193	1.4	2.8692277	1.30E-20	1.55E-20	0.11	0.29	0.18	1.29	1.04	1.87
OsR498G0510740200.01	1.956	0.07	-4.76513	9.66E-45	7.87E-45	1.96	1.87	2.04	0.09	0.03	0.09
OsR498G0510740300.01	1.67	0.106	-4.079854	3.64E-30	3.65E-30	1.73	1.62	1.66	0.1	0.15	0.07
OsR498G0510755200.01	4.446	18.15	2.0785988	5.95E-214	1.68E-214	4.01	4.06	5.27	19.13	17.44	17.88
OsR498G0510755500.01	10.43	36.246	1.7966559	0	0	10.75	10.55	9.99	37	35.19	36.55
OsR498G0510757800.01	0.133	0.403	1.6182661	1.41E-07	2.39E-07	0.12	0.08	0.2	0.37	0.48	0.36
OsR498G0510759300.01	43.423	102.33	1.2295371	0	0	46.56	42.64	41.07	103.54	99.35	104.09
OsR498G0510761000.01	10.97	31.406	1.5252438	1.12E-93	5.90E-94	12.36	9.98	10.57	31.89	31.77	30.56
OsR498G0510763400.01	3.436	0.906	-1.926328	4.75E-37	4.29E-37	4.16	2.6	3.55	1.03	0.63	1.06
OsR498G0510765400.01	0.953	2.24	1.232722	4.85E-08	8.04E-08	0.68	1.06	1.12	2.3	2.45	1.97
OsR498G0510765500.01	4.76	13.78	1.5312802	2.84E-27	3.00E-27	5.45	3.3	5.53	13.96	13.21	14.17
OsR498G0510766100.01	4.17	8.373	1.0128395	1.47E-34	1.37E-34	4.47	3.87	4.17	9.23	8.34	7.55
OsR498G0510767500.01	4.053	0.843	-2.267665	4.98E-17	6.36E-17	3.08	5.33	3.75	0.97	0.77	0.79
OsR498G0510767800.01	31.313	12.48	-1.329677	9.06E-54	6.67E-54	34.46	25.46	34.02	12.04	11.37	14.03
OsR498G0510775200.01	12.36	30.123	1.3049659	3.18E-166	1.11E-166	11.58	12.59	12.91	30.14	30.78	29.45
OsR498G0510775500.01	4.693	15.543	1.7281331	2.68E-146	1.02E-146	5.4	4.88	3.8	15.64	14.4	16.59
OsR498G0510778200.01	0.456	1.036	1.1873737	6.97E-05	0.0001337	0.56	0.71	0.1	0.96	1.08	1.07
OsR498G0510778400.01	0.52	1.546	1.5826422	3.53E-11	5.26E-11	0.61	0.38	0.57	1.47	1.5	1.67
OsR498G0510779700.01	0.23	1	2.1566133	2.73E-15	3.63E-15	0.2	0.33	0.16	1.19	1.03	0.78
OsR498G0510783100.01	17.05	41.093	1.2613698	1.15E-235	3.01E-236	18.09	17.36	15.7	42.09	41.7	39.49
OsR498G0510783300.01	0.763	6.89	3.1731898	6.63E-37	6.01E-37	0.94	0.42	0.93	7.11	6.07	7.49
OsR498G0510790000.01	8.863	22.66	1.3470628	7.96E-104	3.89E-104	8.91	8.2	9.48	22.33	23.34	22.31
OsR498G0510790800.01	2.956	1.08	-1.470828	6.00E-14	8.25E-14	3.18	3.06	2.63	0.98	1.33	0.93
OsR498G0510792900.01	30.92	64.996	1.0736937	7.46E-170	2.55E-170	29.51	32.87	30.38	63.35	66.05	65.59
OsR498G0510794200.01	0.056	1.12	4.3201848	5.08E-15	6.80E-15	0.06	0	0.11	1.42	0.98	0.96
OsR498G0510794300.01	0.203	0.03	-2.832945	0.0006839	0.0014027	0.26	0.26	0.09	0	0	0.09
OsR498G0510796000.01	159.813	34.253	-2.222767	0	0	162.06	157.27	160.11	35.17	32.91	34.68
OsR498G0510797600.01	16.986	38.873	1.19255	5.44E-216	1.52E-216	16.85	16.5	17.61	39.8	38.53	38.29
OsR498G0510801400.01	44.68	91.41	1.0307263	1.00E-250	2.47E-251	44.46	43.87	45.71	89.21	90.23	94.79
OsR498G0510801700.01	12.376	37.233	1.5876813	1.94E-218	5.36E-219	12.37	12.24	12.52	37.01	34.94	39.75
OsR498G0510802100.01	0	0.44	6.0188041	1.75E-09	2.75E-09	0	0	0	0.36	0.47	0.49
OsR498G0510805500.01	0.033	0.356	3.4338416	7.99E-06	1.46E-05	0	0.05	0.05	0.43	0.34	0.3
OsR498G0510811600.01	0.136	1.513	3.5478768	1.83E-49	1.42E-49	0.15	0.12	0.14	1.78	1.48	1.28
OsR498G0510812600.01	0.323	0.043	-2.900059	2.72E-05	5.09E-05	0.4	0.13	0.44	0	0.04	0.09
OsR498G0510815700.01	11.43	30.39	1.3486917	2.18E-163	7.72E-164	10.94	11.61	11.74	30.36	28.29	32.52
OsR498G0510818600.01	0.856	1.97	1.189916	5.84E-12	8.48E-12	0.84	0.73	1	1.93	2.12	1.86
OsR498G0510822700.01	62.743	141.34	1.1728962	3.24E-303	6.80E-304	64.29	63.06	60.88	144.76	138.52	140.75
OsR498G0510822900.01	0.413	0.843	1.0876206	3.36E-06	6.02E-06	0.37	0.58	0.29	0.9	0.8	0.83
OsR498G0510825100.01	0.21	0.53	1.3643565	2.59E-06	4.61E-06	0.21	0.19	0.23	0.56	0.56	0.47
OsR498G0510825900.01	1.373	0.296	-2.214932	2.74E-24	3.03E-24	0.95	1.49	1.68	0.3	0.32	0.27
OsR498G0510826100.01	0.52	0.09	-2.559022	1.77E-17	2.24E-17	0.6	0.45	0.51	0.06	0.14	0.07
OsR498G0510829000.01	1.65	9.21	2.5502703	1.48E-133	6.08E-134	1.84	1.79	1.32	9.7	9.36	8.57
OsR498G0510836400.01	0.03	0.43	3.832391	1.29E-07	2.17E-07	0.05	0.04	0	0.44	0.26	0.59
OsR498G0510836900.01	0.25	0.67	1.5593725	1.08E-05	1.98E-05	0.19	0.19	0.37	0.68	0.59	0.74
OsR498G0510837700.01	0.39	0.163	-1.440628	0.0002392	0.0004746	0.38	0.41	0.38	0.26	0.08	0.15
OsR498G0510842500.01	3.216	10.49	1.718924	6.47E-174	2.17E-174	3.19	3.38	3.08	10.56	11	9.91
OsR498G0510842800.01	9.54	24.416	1.3440125	5.89E-193	1.82E-193	9.58	10.14	8.9	24.5	24.8	23.95

OsR498G0510843600.01	21.12	127.56	2.5933761	0	0	20.72	21.44	21.2	131.47	130.06	121.14
OsR498G0510845000.01	0.186	0.653	1.4556359	1.91E-11	2.82E-11	0.15	0.2	0.21	0.64	0.51	0.81
OsR498G0510848800.01	0.783	1.636	1.0636773	8.65E-06	1.58E-05	0.5	0.85	1	2.09	1.07	1.75
OsR498G0510849900.01	0.323	0.866	1.3939488	2.00E-08	3.27E-08	0.22	0.51	0.24	0.86	1.12	0.62
OsR498G0510850100.01	5.523	17.973	1.6797892	9.64E-169	3.31E-169	5.79	5.48	5.3	18.29	16.53	19.1
OsR498G0510850300.01	2.98	15.756	2.4169282	2.27E-140	8.97E-141	2.83	3.26	2.85	16.62	14.82	15.83
OsR498G0510852000.01	4.333	13.253	1.6124495	2.51E-42	2.12E-42	4.4	4.22	4.38	12.61	13.75	13.4
OsR498G0510860600.01	19.85	70.823	1.8335775	0	0	19.46	19.88	20.21	71.01	69.52	71.94
OsR498G0510863900.01	102.253	400.92	1.9664325	0	0	104.57	100.2	101.99	405.84	403.79	393.13
OsR498G0510866100.01	3.77	7.743	1.0436143	1.28E-49	9.86E-50	4.17	3.89	3.25	7.66	8.31	7.26
OsR498G0510866900.01	5.976	59.183	3.3352196	0	0	6.04	6.18	5.71	58.87	57.69	60.99
OsR498G0510868000.01	1.2	0.49	-1.257537	3.28E-07	5.63E-07	1.09	1.36	1.15	0.54	0.37	0.56
OsR498G0510876200.01	1.936	4.696	1.2801361	2.30E-28	2.38E-28	1.72	2.03	2.06	4.14	5.07	4.88
OsR498G0510880400.01	43.18	121.96	1.4982534	0	0	43.03	44.18	42.33	121	127.01	117.88
OsR498G0510881100.01	1.36	4.996	1.8889406	1.00E-42	8.39E-43	1.02	1.63	1.43	4.93	5.08	4.98
OsR498G0510886400.01	0.906	19.54	4.4305033	0	0	1.02	0.5	1.2	19.41	18.63	20.58
OsR498G0510888800.01	0.383	0	-5.72603	5.93E-08	9.87E-08	0.62	0.35	0.18	0	0	0
OsR498G0510897600.01	0.19	0.68	1.8369064	2.01E-05	3.73E-05	0.21	0.15	0.21	0.81	0.4	0.83
OsR498G0510897900.01	0.303	1.32	2.1228018	1.05E-16	1.35E-16	0.31	0.28	0.32	1.51	1.33	1.12
OsR498G0510898600.01	33.156	69.973	1.0504752	3.91E-281	8.71E-282	33.01	33.1	33.36	71.5	71.15	67.27
OsR498G0510898800.01	2.086	0.706	-1.625052	1.09E-17	1.37E-17	2.3	1.72	2.24	0.66	0.88	0.58
OsR498G0510901800.01	0.236	2.293	3.2314206	5.42E-44	4.46E-44	0.23	0.2	0.28	2.38	2.59	1.91
OsR498G0510902800.01	2.79	11.3	2.0217157	2.20E-125	9.48E-126	2.66	2.73	2.98	11.69	12.14	10.07
OsR498G0510903000.01	128.166	290.8	1.1789355	0	0	126.59	127.43	130.48	296.04	269.88	306.48
OsR498G0510911400.01	6.16	2.81	-1.136606	1.67E-64	1.11E-64	5.74	6.09	6.65	2.68	3	2.75
OsR498G0510912500.01	0.736	0.276	-1.422926	7.93E-09	1.28E-08	0.8	0.65	0.76	0.16	0.38	0.29
OsR498G0510915300.01	20.896	46.603	1.1335134	8.03E-206	2.35E-206	20.47	20.96	21.26	47.4	46.15	46.26
OsR498G0510918600.01	0.333	2.553	2.9378841	4.48E-08	7.41E-08	0.4	0.2	0.4	2.54	2.14	2.98
OsR498G0510922900.01	0.1	0.383	2.2963381	8.68E-05	0.0001676	0	0.06	0.24	0.35	0.54	0.26
OsR498G0510923200.01	19.51	6.38	-1.604141	1.77E-106	8.51E-107	21.36	18.06	19.11	6.01	7.26	5.87
OsR498G0510924000.01	21.496	48.49	1.1714904	1.29E-230	3.42E-231	21.97	22.66	19.86	49.06	49.05	47.36
OsR498G0510924200.01	1.51	3.163	1.0590724	1.46E-09	2.29E-09	1.55	1.39	1.59	2.63	2.66	4.2
OsR498G0510924800.01	0.316	0.646	1.0471663	0.0004324	0.0008727	0.36	0.23	0.36	0.44	0.82	0.68
OsR498G0510925700.01	15.29	7.606	-1.009573	2.70E-49	2.09E-49	16.15	14.52	15.2	8.02	6.11	8.69
OsR498G0510935200.01	5.473	21.16	1.9454839	4.24E-65	2.80E-65	4.19	6.03	6.2	19.31	23.79	20.38
OsR498G0510935600.01	0.053	0.883	4.0644646	5.89E-10	9.12E-10	0.07	0.09	0	0.81	0.8	1.04
OsR498G0510936200.01	0.306	1.663	2.4269222	1.64E-15	2.18E-15	0.34	0.29	0.29	2.29	1.28	1.42
OsR498G0510938100.01	2.306	4.59	1.0266399	4.77E-19	5.83E-19	2.45	2.4	2.07	4.68	4.68	4.41
OsR498G0510938900.01	2.83	14.98	2.3717199	1.37E-177	4.54E-178	2.91	2.52	3.06	16.09	14.14	14.71
OsR498G0510940500.01	14.51	31.793	1.1204623	1.49E-122	6.47E-123	14.54	14.32	14.67	30.86	32.61	31.91
OsR498G0510941100.01	0.053	9.006	7.7315221	2.02E-139	8.02E-140	0.12	0	0.04	9.48	8.91	8.63
OsR498G0510944900.01	80.076	448.61	2.4849154	0	0	75.37	83.08	81.78	462.14	432.47	451.21
OsR498G0510957400.01	17.67	4.843	-1.86949	1.56E-184	4.98E-185	18.05	17.88	17.08	4.84	3.98	5.71
OsR498G0510962400.01	5.98	2.723	-1.141243	1.25E-17	1.57E-17	5.5	5.85	6.59	2.42	3.02	2.73
OsR498G0510964700.01	22.096	9.003	-1.429893	0	0	22.17	22.57	21.55	8.86	9.83	8.32
OsR498G0510966100.01	4.516	0.783	-2.52809	3.66E-66	2.38E-66	4.81	4.43	4.31	0.78	0.89	0.68
OsR498G0510968600.01	77.61	37.723	-1.042564	0	0	81.16	76.6	75.07	37.67	36.3	39.2
OsR498G0510969800.01	3.62	1.106	-1.673605	5.62E-27	5.97E-27	3.1	3.31	4.45	1.3	0.83	1.19
OsR498G0510970900.01	0.123	0.46	2.1968024	5.55E-05	0.000106	0.11	0.11	0.15	0.54	0.49	0.35
OsR498G0510972600.01	24.243	8.6	-1.3439	4.88E-85	2.74E-85	24.3	22.65	25.78	9.22	8.09	8.49
OsR498G0510976100.01	53.31	116.8	1.1273487	0	0	53.36	50.34	56.23	112.53	115.72	122.14
OsR498G0510976700.01	17.503	43.783	1.3258129	1.89E-166	6.58E-167	18.72	17.67	16.12	42.3	44.53	44.52
OsR498G0510977100.01	7.926	23.833	1.5336553	4.63E-121	2.03E-121	8.67	7.08	8.03	22.29	24.98	24.23
OsR498G0510977300.01	0.1	0.696	2.637375	2.67E-18	3.31E-18	0.04	0.17	0.09	1.08	0.54	0.47
OsR498G0510978200.01	0.133	0	-4.347518	0.0007014	0.0014405	0.12	0.12	0.16	0	0	0
OsR498G0510978500.01	1.036	0.386	-1.408919	1.39E-05	2.57E-05	1.25	0.67	1.19	0.45	0.35	0.36
OsR498G0510979100.01	0.13	0.753	2.4937841	1.71E-13	2.38E-13	0.13	0.13	0.13	0.78	0.7	0.78
OsR498G0510984600.01	0.57	2.106	1.9147037	1.30E-29	1.32E-29	0.51	0.67	0.53	2.2	2.07	2.05
OsR498G0510986100.01	0.023	0.293	4.0618728	6.76E-09	1.08E-08	0.04	0	0.03	0.25	0.44	0.19
OsR498G0510987900.01	101.276	323.87	1.6739642	0	0	98.33	98.85	106.65	317.34	316.81	337.46
OsR498G0510991000.01	11.126	35.79	1.6787445	6.05E-168	2.09E-168	13.12	9.66	10.6	36.88	34.67	35.82
OsR498G0510994000.01	173.056	16.04	-3.42933	0	0	172.83	178.05	168.29	15.54	17.23	15.35
OsR498G0510996600.01	14.186	37.196	1.389739	0	0	13.95	14.09	14.52	38.51	37.32	35.76
OsR498G0510999400.01	0.4	0.056	-2.832945	1.94E-08	3.16E-08	0.43	0.37	0.4	0.11	0	0.06
OsR498G0511001100.01	0.19	0.516	1.3894475	3.42E-06	6.12E-06	0.23	0.18	0.16	0.62	0.5	0.43
OsR498G0511001400.01	0.023	0.343	3.7817649	2.33E-07	3.97E-07	0	0	0.07	0.29	0.33	0.41
OsR498G0511003600.01	0.033	0.41	3.5593725	0.0005724	0.0011661	0	0.1	0	0.51	0.41	0.31
OsR498G0511004000.01	2.623	6.466	1.3017465	1.56E-61	1.06E-61	2.4	2.78	2.69	7.02	6.48	5.9
OsR498G0511004700.01	0.066	0.35	2.832391	7.77E-11	1.17E-10	0.06	0.03	0.11	0.39	0.29	0.37

OsR498G0511006000.01	0.056	0.31	2.4073694	7.99E-09	1.29E-08	0.05	0.09	0.03	0.36	0.24	0.33
OsR498G0511006800.01	16.856	34.346	1.0179858	1.54E-76	9.21E-77	18.21	16.02	16.34	35.18	34.07	33.79
OsR498G0511007800.01	2.78	0.733	-1.924093	3.40E-36	3.11E-36	3.06	2.74	2.54	0.76	0.87	0.57
OsR498G0511009100.01	20.826	45.523	1.107302	4.80E-122	2.09E-122	21.48	21.04	19.96	46.41	46.27	43.89
OsR498G0511011900.01	0.04	0.22	2.3667274	0.0002249	0.000445	0.06	0	0.06	0.25	0.22	0.19
OsR498G0511016100.01	0.44	5.063	3.535804	1.54E-82	8.81E-83	0.37	0.51	0.44	4.96	4.8	5.43
OsR498G0511019800.01	0.29	1.733	2.5671081	4.63E-28	4.82E-28	0.34	0.25	0.28	1.49	1.81	1.9
OsR498G0511020400.01	3.956	8.826	1.1563563	1.11E-55	7.99E-56	4.75	2.54	4.58	8.94	8.05	9.49
OsR498G0511020500.01	6.716	17.493	1.3230965	4.25E-101	2.12E-101	6.11	8.05	5.99	16.61	18.36	17.51
OsR498G0511020800.01	0.723	2.03	1.489551	9.77E-27	1.04E-26	0.5	0.9	0.77	1.88	2.07	2.14
OsR498G0511021200.01	7.906	20.703	1.3753674	3.08E-177	1.02E-177	7.61	7.9	8.21	20.78	20.37	20.96
OsR498G0511021900.01	3.053	1.17	-1.381734	1.00E-05	1.84E-05	3.73	2.72	2.71	1.26	1.25	1
OsR498G0511022200.01	0.503	1.11	1.1387968	0.0001207	0.0002352	0.63	0.36	0.52	1.12	1.06	1.15
OsR498G0511025800.01	5.326	12.47	1.2286205	9.49E-52	7.16E-52	5.62	5.41	4.95	13.37	11.84	12.2
OsR498G0511029000.01	8.36	32.243	1.9457822	0	0	8.76	8.09	8.23	31.5	32.65	32.58
OsR498G0511029900.01	7.386	19.8	1.4204363	6.90E-72	4.27E-72	6.58	7.56	8.02	19.58	18.16	21.66
OsR498G0511031400.01	0.126	8.846	6.1160062	1.10E-247	2.74E-248	0.15	0.18	0.05	8.4	9.01	9.13
OsR498G0511032000.01	11.75	34.846	1.5639285	5.65E-305	1.18E-305	11.23	11.78	12.24	38.05	35.57	30.92
OsR498G0511033800.01	0.456	1.773	2.2485517	8.66E-36	7.99E-36	0.36	0.38	0.63	1.93	1.75	1.64
OsR498G0511035600.01	2.486	5.323	1.0958298	8.18E-21	9.66E-21	1.98	2.57	2.91	5.36	5.2	5.41
OsR498G0511037900.01	0.123	0	-4.72603	0.0001114	0.0002167	0.11	0.2	0.06	0	0	0
OsR498G0511044200.01	15.286	80.02	2.3851097	0	0	13.72	15.91	16.23	76.97	77.55	85.54
OsR498G0511044800.01	1.303	0.013	-6.595446	5.36E-24	5.97E-24	1.49	1.15	1.27	0.04	0	0
OsR498G0511046900.01	3.86	7.87	1.0303564	3.43E-33	3.28E-33	3.89	3.47	4.22	7.36	8.05	8.2
OsR498G0511049700.01	0.783	0.316	-1.406138	0.0007532	0.0015505	0.71	0.93	0.71	0.44	0.16	0.35
OsR498G0511052800.01	1.453	0.72	-1.083805	2.55E-09	4.02E-09	1.73	1.07	1.56	0.49	0.89	0.78
OsR498G0511056000.01	3.2	6.506	1.0548999	1.79E-22	2.04E-22	3.49	2.73	3.38	6.64	6.62	6.26
OsR498G0511056600.01	1.91	4.243	1.0803011	2.45E-15	3.25E-15	1.82	2.19	1.72	4.37	4.25	4.11
OsR498G0511061800.01	8.163	49.403	2.5979561	0	0	8.08	8.89	7.52	50.84	47.23	50.14
OsR498G0511068200.01	0.183	2.266	3.6182661	3.43E-21	4.03E-21	0.14	0	0.41	2.16	2.15	2.49
OsR498G0511072500.01	0.746	5.68	3.0447993	5.94E-65	3.92E-65	0.35	0.86	1.03	6	5.52	5.52
OsR498G0511073400.01	0.303	1.04	1.7931696	3.52E-13	4.93E-13	0.32	0.19	0.4	0.79	1.21	1.12
OsR498G0511074600.01	0.01	0.753	6.144335	1.41E-18	1.75E-18	0.03	0	0	0.94	0.68	0.64
OsR498G0511075200.01	20.01	7.603	-1.539456	2.19E-236	5.70E-237	19.86	19.75	20.42	7.53	8.31	6.97
OsR498G0511076000.01	9.933	36.57	1.8795665	3.56E-160	1.28E-160	9.72	10.36	9.72	35.63	37.18	36.9
OsR498G0511076600.01	0.283	0.74	1.4922583	4.49E-06	8.09E-06	0.31	0.24	0.3	0.62	0.86	0.74
OsR498G0511077000.01	6.2	32.18	2.3683457	5.13E-201	1.53E-201	5.35	7.02	6.23	31.53	30.69	34.32
OsR498G0511079200.01	0.603	1.953	1.6457872	2.66E-22	3.05E-22	0.55	0.54	0.72	1.94	1.92	2
OsR498G0511080900.01	2.633	5.793	1.1157658	1.50E-21	1.74E-21	2.51	2.71	2.68	6.49	5.56	5.33
OsR498G0511082600.01	0.4	1.3	1.633373	1.86E-06	3.29E-06	0.2	0.67	0.33	1.09	1.25	1.56
OsR498G0511082900.01	1.503	5.68	1.9168589	3.86E-69	2.45E-69	1.22	1.6	1.69	5.16	6.04	5.84
OsR498G0511083100.01	15.65	70.28	2.1437878	1.06E-299	2.25E-300	15.05	16.56	15.34	69.95	65.6	75.29
OsR498G0511086400.01	5.453	35.416	2.7007461	0	0	5.86	5.5	5	36.42	32.53	37.3
OsR498G0511087600.01	1.016	0.313	-1.589491	5.55E-07	9.62E-07	1.08	0.96	1.01	0.31	0.36	0.27
OsR498G0511090500.01	0.676	4.276	2.6789541	1.69E-53	1.25E-53	0.95	0.59	0.49	3.72	4.4	4.71
OsR498G0511092000.01	31.353	7.83	-2.02559	1.52E-288	3.31E-289	31.18	31.23	31.65	8.27	7.69	7.53
OsR498G0511092300.01	0.11	1.676	3.577751	3.77E-32	3.66E-32	0.17	0.08	0.08	1.81	1.58	1.64
OsR498G0511096000.01	55.243	229.42	2.0625948	0	0	56.57	53.51	55.65	225.89	217.24	245.13
OsR498G0511097100.01	13.433	71.403	2.4186466	0	0	12.4	13.93	13.97	70.01	68.41	75.79
OsR498G0511098100.01	0.266	0.816	1.6066782	0.0004702	0.0009519	0.56	0.08	0.16	0.55	0.7	1.2
OsR498G0511098700.01	9.34	22.736	1.2803707	4.91E-102	2.43E-102	9.83	9.15	9.04	22.2	21.68	24.33
OsR498G0511101500.01	7.126	3.383	-1.07603	4.32E-25	4.72E-25	6.96	6.96	7.46	3.43	3.15	3.57
OsR498G0511102000.01	576.7	222.4	-1.372154	0	0	576.27	583.03	570.8	220.03	224.09	223.07
OsR498G0511102100.01	1.14	4.433	1.9608642	2.38E-20	2.84E-20	1.3	1.63	0.49	4.36	5.09	3.85
OsR498G0511103500.01	0.09	0.206	1.3280469	0.0002418	0.0004799	0.07	0.12	0.08	0.13	0.28	0.21
OsR498G0511105200.01	32.563	76.273	1.2099478	1.23E-206	3.59E-207	32.2	31.16	34.33	73.87	73.27	81.68
OsR498G0511105800.01	1.286	2.796	1.1136563	3.58E-28	3.71E-28	1.43	1.29	1.14	2.79	2.91	2.69
OsR498G0511106300.01	9.68	31.86	1.7146314	4.17E-287	9.14E-288	10.22	9.97	8.85	34	32.88	28.7
OsR498G0511106500.01	2.653	6.556	1.3020973	6.52E-30	6.58E-30	2.84	2.88	2.24	6.78	6.36	6.53
OsR498G0511109100.01	0.08	0.4	2.2739702	1.37E-09	2.15E-09	0.04	0.11	0.09	0.44	0.36	0.4
OsR498G0511109800.01	1.846	5.67	1.6225663	9.29E-51	7.10E-51	1.59	1.9	2.05	5.53	5.51	5.97
OsR498G0511111400.01	6.553	16.903	1.365584	4.21E-87	2.33E-87	5.92	6.98	6.76	16.98	16.27	17.46
OsR498G0511113100.01	0.14	0.633	2.1723493	8.74E-11	1.32E-10	0.2	0.07	0.15	0.65	0.46	0.79
OsR498G0511114100.01	0.023	0.153	3.5593725	0.0005724	0.0011661	0	0	0.07	0.15	0.23	0.08
OsR498G0511115700.01	0.186	0.623	1.7292975	0.0005682	0.0011565	0.14	0.28	0.14	0.62	0.55	0.7
OsR498G0511118000.01	3.776	0.846	-2.155789	1.04E-24	1.15E-24	3.99	3.61	3.73	0.79	0.89	0.86
OsR498G0511119100.01	1.13	2.693	1.3439561	1.90E-24	2.10E-24	1.3	1.14	0.95	2.3	2.8	2.98
OsR498G0511119300.01	5.76	17.74	1.619842	1.79E-90	9.66E-91	5.65	5.97	5.66	17.32	16.2	19.7
OsR498G0511120900.01	11.146	39.703	1.8315427	0	0	11.61	10.65	11.18	39.49	37.47	42.15

OsR498G0511121700.01	0.066	0.723	3.832391	1.64E-13	2.29E-13	0.04	0.11	0.05	0.92	0.56	0.69
OsR498G0511122400.01	0.413	1.103	1.4173535	6.78E-13	9.58E-13	0.37	0.56	0.31	1.15	1.11	1.05
OsR498G0511123000.01	6.44	19.78	1.6021674	2.61E-111	1.22E-111	6.95	6.94	5.43	20.85	19.14	19.35
OsR498G0511129200.01	0.386	0.02	-4.347518	3.68E-06	6.60E-06	0.29	0.17	0.7	0.06	0	0
OsR498G0511140700.01	5.293	75.43	3.8184645	0	0	5.15	5.17	5.56	78.7	75.98	71.61
OsR498G0511143000.01	0.116	11.193	6.4901098	5.93E-170	2.03E-170	0.24	0.11	0	10.91	11.8	10.87
OsR498G0511143200.01	0.34	2.22	2.7520175	1.32E-23	1.48E-23	0.38	0.45	0.19	1.93	2.2	2.53
OsR498G0511153900.01	3.803	15.633	2.0728523	1.10E-144	4.22E-145	3.29	3.61	4.51	15.03	16.04	15.83
OsR498G0511155500.01	18.496	44.666	1.2514588	6.64E-295	1.42E-295	18.84	18.35	18.3	47.53	44.27	42.2
OsR498G0511158800.01	1.333	21.876	4.0365511	0	0	1.25	1.34	1.41	22.76	21.57	21.3
OsR498G0511160200.01	1.01	4.566	2.1749731	1.29E-36	1.17E-36	1.16	0.86	1.01	4.58	4.15	4.97
OsR498G0511161200.01	1.52	3.143	1.0090561	8.78E-13	1.24E-12	1.47	1.42	1.67	3.8	3.05	2.58
OsR498G0511165600.01	24.013	49.19	1.0344278	1.89E-266	4.38E-267	23.78	23.96	24.3	49.22	49.78	48.57
OsR498G0511166900.01	0.276	0.063	-2.095979	0.0004964	0.0010071	0.25	0.32	0.26	0.03	0.12	0.04
OsR498G0511172100.01	3.76	1.033	-1.862795	6.04E-30	6.09E-30	3.68	3.83	3.77	1.2	0.82	1.08
OsR498G0511173300.01	19.956	41.783	1.0566247	4.03E-139	1.60E-139	18.87	19.42	21.58	41.05	41.88	42.42
OsR498G0511173500.01	10.166	23.196	1.0322444	9.37E-107	4.50E-107	9.95	10.13	10.42	23.02	24.09	22.48
OsR498G0511173700.01	2.283	4.983	1.1269319	1.39E-15	1.84E-15	2.08	2.16	2.61	4.99	4.68	5.28
OsR498G0511179700.01	0.26	1.4	2.4538793	3.68E-21	4.32E-21	0.25	0.26	0.27	1.12	1.55	1.53
OsR498G0511181900.01	0.7	3.64	2.278744	9.66E-22	1.12E-21	0.71	0.71	0.68	3.74	4.27	2.91
OsR498G0511182600.01	0.883	0	-5.417907	9.99E-07	1.75E-06	1.14	0.88	0.63	0	0	0
OsR498G0511186500.01	14.163	1.073	-3.920987	0	0	15.33	13.29	13.87	1.09	1.3	0.83
OsR498G0511186800.01	44.616	1.173	-5.252866	6.45E-225	1.74E-225	47.01	45.94	40.9	1.53	0.41	1.58
OsR498G0511190900.01	7.05	16.17	1.1698221	1.87E-128	7.92E-129	7.68	7.07	6.4	16.4	16.24	15.87
OsR498G0511194100.01	10.256	45.426	2.1464123	0	0	10.18	9.84	10.75	45.67	46	44.61
OsR498G0511195200.01	24.69	227.84	3.1979962	0	0	24.96	25.1	24.01	229.43	234.7	219.4
OsR498G0511195400.01	0.616	1.773	1.512375	5.05E-23	5.72E-23	0.69	0.55	0.61	2.14	1.72	1.46
OsR498G0511195700.01	3.023	13.996	2.2522257	6.73E-207	1.96E-207	3.51	2.65	2.91	14.62	13.83	13.54
OsR498G0511196600.01	11.29	28.053	1.3178391	4.68E-242	1.19E-242	12.01	10.61	11.25	28.65	27.61	27.9
OsR498G0511203000.01	13.49	3.923	-1.411289	1.72E-75	1.03E-75	14.43	14.36	11.68	4.69	3.52	3.56
OsR498G0511207000.01	5.406	14.41	1.4148439	2.97E-173	9.99E-174	5.28	5.56	5.38	14.01	14.66	14.56
OsR498G0511211900.01	2.3	8.016	1.7469995	4.94E-76	2.96E-76	2.21	2.8	1.89	8.95	7.47	7.63
OsR498G0511218000.01	1.093	9.586	3.1358734	3.45E-60	2.38E-60	1.04	1.12	1.12	11.11	9.8	7.85
OsR498G0511218600.01	0.08	4.03	5.6698769	4.59E-74	2.80E-74	0.04	0.14	0.06	3.72	4.12	4.25
OsR498G0511218800.01	0.16	3.64	4.5357305	4.29E-127	1.83E-127	0.16	0.11	0.21	3.66	3.59	3.67
OsR498G0511221000.01	0.973	0.16	-2.610553	8.70E-16	1.14E-15	0.77	1.09	1.06	0.18	0.21	0.09
OsR498G0511221800.01	0.803	0.233	-1.786402	2.72E-07	4.65E-07	0.99	0.63	0.79	0.23	0.27	0.2
OsR498G0511222000.01	0.503	1.416	1.4785414	7.53E-22	8.72E-22	0.52	0.53	0.46	1.6	1.33	1.32
OsR498G0511222400.01	2.973	7.6	1.3349409	1.91E-19	2.31E-19	2.66	3.39	2.87	6.57	6.61	9.62
OsR498G0511224300.01	0.783	3.88	2.3067924	3.76E-36	3.44E-36	0.73	0.68	0.94	4.09	4.03	3.52
OsR498G0511224500.01	2.743	12.896	2.2383549	1.76E-77	1.04E-77	2.62	3.08	2.53	12.55	12.88	13.26
OsR498G0511224800.01	26.39	59.533	1.1615515	1.34E-186	4.25E-187	27.35	26.13	25.69	60.23	59.91	58.46
OsR498G0511225400.01	7.963	17.796	1.156888	1.08E-48	8.42E-49	8.67	7.56	7.66	16.5	17.43	19.46
OsR498G0511229400.01	0	0.216	4.6748497	0.0001298	0.0002536	0	0	0	0.15	0.45	0.05
OsR498G0511231300.01	2.03	19.573	3.2669086	0	0	1.97	2.01	2.11	20.08	17.86	20.78
OsR498G0511236100.01	2.56	7.74	1.6023039	1.26E-74	7.63E-75	2.42	2.5	2.76	7.99	7.76	7.47
OsR498G0511241500.01	30.196	64.583	1.1068482	1.32E-200	3.95E-201	30.72	31.11	28.76	64.63	63.34	65.78
OsR498G0511243500.01	53.043	110.79	1.0649306	4.48E-253	1.09E-253	51.79	50.56	56.78	115.54	106.99	109.84
OsR498G0511243700.01	0.366	1.036	1.5218978	0.0002209	0.0004369	0.33	0.28	0.49	0.82	1.27	1.02
OsR498G0511244300.01	5.333	12.586	1.2180501	5.03E-42	4.26E-42	6.49	4.66	4.85	12.7	12.97	12.09
OsR498G0511249100.01	0.453	2.89	2.6182661	1.70E-30	1.70E-30	0.46	0.5	0.4	2.87	3.15	2.65
OsR498G0511251000.01	6.31	17.56	1.5087707	1.64E-144	6.33E-145	6.37	6.36	6.2	17.23	18.55	16.9
OsR498G0511253300.01	5.943	36.04	2.5973686	7.52E-291	1.63E-291	5.36	5.9	6.57	36.44	32.36	39.32
OsR498G0511255300.01	0.066	0.966	3.8813006	4.45E-17	5.69E-17	0.04	0.08	0.08	0.88	0.84	1.18
OsR498G0511257600.01	0.216	1.22	2.5593725	6.33E-17	8.11E-17	0.26	0.1	0.29	1.4	1.23	1.03
OsR498G0511263000.01	5.14	13.166	1.3825798	1.45E-121	6.33E-122	5.03	4.97	5.42	13.09	13.35	13.06
OsR498G0511263100.01	9.49	19.506	1.0385403	5.30E-52	3.98E-52	8.89	9.96	9.62	20.03	19.68	18.81
OsR498G0511264400.01	0.35	3.24	3.1694259	5.11E-32	4.98E-32	0.19	0.54	0.32	3.12	3.81	2.79
OsR498G0511264900.01	2.333	7.42	1.6702882	6.45E-113	2.99E-113	2.16	2.28	2.56	7.8	7.28	7.18
OsR498G0511265200.01	1.87	6.446	1.8173888	1.68E-81	9.74E-82	1.65	1.92	2.04	7.37	5.9	6.07
OsR498G0511265600.01	0.403	1.033	1.3456658	8.70E-09	1.40E-08	0.37	0.26	0.58	1.06	0.9	1.14
OsR498G0511267100.01	0.92	8.646	3.2309893	1.09E-119	4.84E-120	0.99	0.94	0.83	8.12	8.72	9.1
OsR498G0511269200.01	0.186	0.026	-2.856454	0.0005987	0.0012218	0.16	0.24	0.16	0.08	0	0
OsR498G0511270600.01	0.083	0.913	3.4853719	2.83E-23	3.19E-23	0.05	0.1	0.1	0.91	0.96	0.87
OsR498G0511270900.01	5.75	36.316	2.6112684	0	0	5.64	6.11	5.5	35.48	35.63	37.84
OsR498G0511271700.01	0.016	0.793	5.4979719	1.58E-12	2.25E-12	0	0.05	0	0.82	0.51	1.05
OsR498G0511272500.01	0.666	1.83	1.8281892	2.12E-29	2.16E-29	0.33	0.92	0.75	1.63	1.91	1.95
OsR498G0511272700.01	0.203	0	-4.347518	0.0007014	0.0014405	0.25	0.24	0.12	0	0	0
OsR498G0511273000.01	0.086	0.336	1.97441	5.68E-05	0.0001084	0.12	0.09	0.05	0.35	0.42	0.24

OsR498G0511283000.01	0.253	3.18	3.6551314	2.59E-44	2.12E-44	0.31	0.27	0.18	3.04	3.33	3.17
OsR498G0511287500.01	0.6	1.77	1.637375	9.39E-32	9.20E-32	0.43	0.78	0.59	1.75	1.96	1.6
OsR498G0511288800.01	4.99	12.25	1.2272401	2.90E-26	3.12E-26	5.22	4.81	4.94	14.54	9.26	12.95
OsR498G0511289000.01	40.2	1.41	-4.79234	0	0	41.39	40.82	38.39	1.5	1.42	1.31
OsR498G0511289300.01	0.043	0.196	2.1575546	3.46E-06	6.21E-06	0.03	0.04	0.06	0.25	0.13	0.21
OsR498G0511289600.01	502.533	215.84	-1.218588	0	0	501.15	505.73	500.72	212.69	223.61	211.22
OsR498G0511291100.01	1.636	7.14	2.1406022	1.94E-45	1.57E-45	1.72	1.77	1.42	6.51	6.89	8.02
OsR498G0511292500.01	3.746	24.036	2.6796347	1.17E-289	2.55E-290	3.64	3.83	3.77	24.11	22.12	25.88
OsR498G0511293200.01	0.543	8.373	3.9473509	5.15E-121	2.26E-121	0.27	0.96	0.4	9.97	8.39	6.76
OsR498G0511294900.01	0.34	0	-4.485022	0.0003787	0.0007614	0.28	0.46	0.28	0	0	0
OsR498G0511296500.01	0.343	6.166	4.3558391	1.65E-173	5.55E-174	0.29	0.39	0.35	6.01	6.33	6.16
OsR498G0511302400.01	0.333	1.95	2.5499121	4.04E-44	3.32E-44	0.34	0.39	0.27	1.81	2.09	1.95
OsR498G0511302800.01	1.11	0.193	-2.52809	1.60E-06	2.83E-06	1.38	1.46	0.49	0	0.38	0.2
OsR498G0511303400.01	2.31	17.083	2.8862244	9.17E-188	2.89E-188	2.46	2.21	2.26	18.02	15.87	17.36
OsR498G0511303600.01	3.403	7.87	1.2071901	7.69E-32	7.53E-32	3.68	3.12	3.41	8.02	6.75	8.84
OsR498G0511305200.01	9.823	20.613	1.0762896	3.10E-96	1.60E-96	9.07	9.96	10.44	21.17	20.4	20.27
OsR498G0511307800.01	1.883	7.796	2.0486645	8.67E-34	8.22E-34	1.92	1.51	2.22	7.22	7.81	8.36
OsR498G0511310400.01	1.426	3.553	1.2831627	9.72E-14	1.34E-13	1.42	1.26	1.6	2.94	3.4	4.32
OsR498G0511311300.01	13.616	30.743	1.1734599	1.04E-99	5.26E-100	13.02	13.48	14.35	30	31.11	31.12
OsR498G0511312600.01	0.733	1.86	1.3425611	2.75E-08	4.51E-08	0.98	0.56	0.66	2.1	1.79	1.69
OsR498G0511313100.01	0.873	2.336	1.4218689	3.46E-17	4.41E-17	0.63	0.95	1.04	2.18	2.36	2.47
OsR498G0511315000.01	0.423	0.173	-1.454433	0.0004899	0.0009933	0.66	0.32	0.29	0.2	0.24	0.08
OsR498G0511318100.01	0.146	0.513	1.7817649	3.23E-19	3.93E-19	0.11	0.15	0.18	0.5	0.57	0.47
OsR498G0511318800.01	3.303	52.346	3.9837306	0	0	3.32	2.34	4.25	49.26	53.63	54.15
OsR498G0511318900.01	5.35	28.136	2.3960516	5.47E-160	1.97E-160	6.14	5.23	4.68	28.71	26.43	29.27
OsR498G0511319100.01	8.61	29.34	1.7635011	8.94E-252	2.18E-252	8.74	9.19	7.9	30.55	28.46	29.01
OsR498G0511319400.01	24.483	48.86	1.00264	5.65E-233	1.49E-233	25.06	26.06	22.33	47.78	50.32	48.48
OsR498G0511319700.01	1.046	16.443	3.9727696	0	0	1.06	1.17	0.91	15.95	16.64	16.74
OsR498G0511319900.01	0.153	0.013	-3.610553	0.0004877	0.0009887	0.19	0	0.27	0	0	0.04
OsR498G0511321100.01	0.41	4.746	3.5345691	2.43E-100	1.22E-100	0.45	0.31	0.47	4.94	4.67	4.63
OsR498G0511321900.01	5.856	31.54	2.4328118	2.25E-196	6.86E-197	5.48	6.04	6.05	30.46	30.63	33.53
OsR498G0511322100.01	6.496	46.036	2.8088239	2.00E-275	4.53E-276	6.04	8.14	5.31	45.87	46.37	45.87
OsR498G0511323500.01	2.646	12.32	2.1902793	1.52E-155	5.56E-156	2.85	2.32	2.77	12.78	12.88	11.3
OsR498G0511324200.01	2.676	6.21	1.2145685	2.99E-48	2.35E-48	2.74	2.79	2.5	6.25	6.28	6.1
OsR498G0511325000.01	29.763	76.69	1.3643371	0	0	29.49	29.94	29.86	75.52	79.99	74.56
OsR498G0511325400.01	0.596	1.563	1.3846939	5.54E-08	9.20E-08	0.38	0.8	0.61	1.65	1.69	1.35
OsR498G0511325600.01	0.436	1.22	1.4662631	7.53E-11	1.13E-10	0.38	0.34	0.59	1.16	1.29	1.21
OsR498G0511325800.01	1.31	5.153	1.9774124	6.84E-51	5.21E-51	1.41	1.11	1.41	5.61	4.54	5.31
OsR498G0511329100.01	8.27	17.01	1.0354818	3.77E-83	2.15E-83	7.62	8	9.19	17.57	16.61	16.85
OsR498G0511331900.01	5.693	2.253	-1.305698	8.16E-37	7.40E-37	6.31	5.31	5.46	2.11	2.83	1.82
OsR498G0511332700.01	2866.2	644.34	-2.15538	0	0	2828.3	2854	2916.2	629.68	604.98	698.35
OsR498G0511334500.01	7.523	15.683	1.1607526	6.23E-68	3.99E-68	7.27	7.18	8.12	16.68	14.93	15.44
OsR498G0511334800.01	1.32	10.036	2.9280209	1.72E-40	1.49E-40	2.05	1.53	0.38	10.25	10.19	9.67
OsR498G0511335100.01	10.256	37.626	1.8735962	0	0	9.35	10.54	10.88	38.16	35.12	39.6
OsR498G0511341800.01	9.22	28.523	1.6286817	1.16E-199	3.47E-200	9.42	8.91	9.33	27.46	27.91	30.2
OsR498G0511343400.01	6.06	18.32	1.5956069	3.00E-261	7.08E-262	6.52	6.21	5.45	19.12	18.03	17.81
OsR498G0511346000.01	0.103	0.853	3.3249072	4.95E-11	7.40E-11	0.21	0.05	0.05	1	0.79	0.77
OsR498G0511346700.01	0.59	0.046	-4.113053	4.44E-09	7.08E-09	0.89	0.26	0.62	0.14	0	0
OsR498G0511347700.01	8.98	46.473	2.3690622	1.50E-100	7.53E-101	6.8	10.11	10.03	44.88	48.27	46.27
OsR498G0511348700.01	18.04	41.07	1.1889917	8.44E-216	2.35E-216	16.82	18.08	19.22	40.77	42.46	39.98
OsR498G0511349200.01	6.24	32.183	2.3665349	1.00E-240	2.57E-241	6.48	6.37	5.87	34.48	31.38	30.69
OsR498G0511350900.01	17.443	88.266	2.3369339	0	0	16.65	19.62	16.06	89.64	81.72	93.44
OsR498G0511351500.01	3.39	8.95	1.3727687	1.52E-43	1.26E-43	2.84	3.93	3.4	9.5	8.92	8.43
OsR498G0511351800.01	2.726	36.643	3.7469995	0	0	2.79	2.58	2.81	35.88	38.95	35.1
OsR498G0511354100.01	0.09	4.146	5.4371167	2.92E-104	1.42E-104	0.14	0.04	0.09	4.19	3.3	4.95
OsR498G0511354700.01	4.36	19.643	2.1524083	4.15E-307	8.60E-308	4.27	4.34	4.47	20.58	20.08	18.27
OsR498G0511356300.01	4.636	1.14	-2.146197	2.68E-36	2.45E-36	4.96	4.65	4.3	0.55	1.5	1.37
OsR498G0511356600.01	5.296	2.006	-1.43191	2.61E-16	3.39E-16	5.31	5.52	5.06	2.21	2.37	1.44
OsR498G0511358900.01	2.956	7.85	1.4948633	1.22E-81	7.07E-82	3.13	2.49	3.25	7.11	8.25	8.19
OsR498G0511359300.01	21.906	1.033	-4.406947	1.76E-148	6.64E-149	21.31	21.99	22.42	1.33	0.87	0.9
OsR498G0511363600.01	1.413	2.956	1.0618728	7.35E-06	1.34E-05	1.33	0.97	1.94	2.85	3.01	3.01
OsR498G0511364900.01	18.35	2.676	-2.908694	6.64E-129	2.79E-129	20.2	16.55	18.3	1.89	2.5	3.64
OsR498G0511365400.01	1.313	3.35	1.3376649	2.20E-19	2.67E-19	1.24	1.76	0.94	3.69	3.23	3.13
OsR498G0511366100.01	5.596	13.213	1.210708	1.38E-29	1.40E-29	4.94	6.84	5.01	12.07	12.83	14.74
OsR498G0511368600.01	0.636	1.8	1.6011926	8.42E-10	1.31E-09	0.52	0.82	0.57	1.75	1.94	1.71
OsR498G0511373500.01	0.46	3.753	3.0333036	5.51E-31	5.47E-31	0.59	0.2	0.59	4.15	3.37	3.74
OsR498G0511374600.01	4.643	15.253	1.7113756	1.56E-80	9.06E-81	4.72	4.67	4.54	13.5	13.78	18.48
OsR498G0511376600.01	0.06	0.186	1.6118399	0.0007971	0.0016457	0.04	0.06	0.08	0.32	0.14	0.1
OsR498G0511380600.01	0.106	0.02	-2.440628	0.0007945	0.00164	0.16	0.06	0.1	0	0.04	0.02

OsR498G0511383100.01	44.46	9.59	-2.216303	3.89E-202	1.16E-202	43.87	42.31	47.2	8.02	9.26	11.49
OsR498G0511383800.01	89.603	18.923	-2.244227	0	0	90.98	88.63	89.2	18.71	19.32	18.74
OsR498G0511384200.01	0	1.026	7.9063293	2.30E-27	2.42E-27	0	0	0	0.99	1.01	1.08
OsR498G0511384800.01	68.576	26.903	-1.034749	8.22E-165	2.90E-165	70.74	66.24	68.75	26.35	29.54	24.82
OsR498G0511385100.01	0.186	2.133	3.4905735	5.73E-75	3.47E-75	0.14	0.26	0.16	2.22	2.05	2.13
OsR498G0511391200.01	1.196	5.306	2.1494967	1.60E-49	1.24E-49	1.28	1.19	1.12	5.95	4.98	4.99
OsR498G0511391600.01	5.14	137.61	4.7403766	0	0	5.82	4.31	5.29	134.43	126.95	151.44
OsR498G0511392000.01	0.506	1.58	1.6427885	7.94E-09	1.28E-08	0.55	0.43	0.54	2.01	1.21	1.52
OsR498G0511392300.01	0.923	0.413	-1.100358	4.66E-06	8.41E-06	0.84	0.94	0.99	0.52	0.26	0.46
OsR498G0511393500.01	0.72	2.303	1.6796667	6.73E-08	1.12E-07	0.66	0.94	0.56	2.29	2.46	2.16
OsR498G0511394200.01	0.01	0.206	4.2223375	8.50E-06	1.55E-05	0.03	0	0	0.17	0.22	0.23
OsR498G0511394400.01	0	0.63	5.7817649	2.57E-08	4.21E-08	0	0	0	0.54	0.87	0.48
OsR498G0511397100.01	0.68	3.276	2.2785633	2.22E-31	2.18E-31	0.63	0.83	0.58	3	3.41	3.42
OsR498G0511400300.01	1.503	0.04	-5.36544	5.49E-21	6.46E-21	1.2	1.3	2.01	0.06	0	0.06
OsR498G0511401000.01	690.67	308.78	-1.161657	0	0	714.39	696.99	660.63	303.74	305.1	317.5
OsR498G0511401700.01	4.186	10.523	1.3567813	4.71E-33	4.52E-33	4.66	3.64	4.26	11.2	10.07	10.3
OsR498G0511404300.01	0.08	0.27	1.8488791	8.72E-05	0.0001686	0.03	0.13	0.08	0.21	0.35	0.25
OsR498G0511406000.01	0.716	2.726	1.9396445	5.89E-10	9.11E-10	1.04	0.81	0.3	2.39	3.44	2.35
OsR498G0511409400.01	32.463	8.563	-1.92566	3.06E-197	9.28E-198	33.3	29.75	34.34	8.08	7.51	10.1
OsR498G0511410300.01	790.23	1581.5	1.0004304	0	0	794.98	807.2	768.51	1599.5	1612.7	1532.2
OsR498G0511410700.01	0	1.12	7.2097541	1.66E-18	2.05E-18	0	0	0	0.49	1.07	1.8
OsR498G0511410900.01	0.06	0.403	2.7469995	5.84E-08	9.70E-08	0.03	0.09	0.06	0.41	0.44	0.36
OsR498G0511411500.01	3.406	1.47	-1.214816	3.32E-17	4.22E-17	3.69	2.8	3.73	1.56	1.32	1.53
OsR498G0511411800.01	0.053	2.783	5.6512838	1.50E-49	1.16E-49	0	0.12	0.04	2.64	2.4	3.31
OsR498G0511413500.01	0.03	0.403	3.6182661	1.75E-11	2.57E-11	0.05	0.02	0.02	0.48	0.29	0.44
OsR498G0511413700.01	3.296	7.573	1.200205	9.28E-16	1.22E-15	3.53	2.69	3.67	7.09	7.78	7.85
OsR498G0511415200.01	0.096	0.946	3.2963381	1.37E-12	1.96E-12	0.14	0.05	0.1	0.89	0.7	1.25
OsR498G0511415500.01	0	0.226	4.2963381	0.0007942	0.0016388	0	0	0	0.07	0.13	0.48
OsR498G0511416300.01	2.21	0.993	-1.155651	3.08E-07	5.28E-07	1.95	1.88	2.8	0.72	0.89	1.37
OsR498G0511417200.01	0.113	0.436	1.9286063	9.10E-05	0.000176	0.13	0.04	0.17	0.46	0.46	0.39
OsR498G0511418600.01	33.75	3.246	-3.374168	0	0	33.28	34.73	33.24	3.3	3.79	2.65
OsR498G0511420500.01	0	0.106	4.2963381	0.0007942	0.0016388	0	0	0	0.07	0.14	0.11
OsR498G0511420700.01	0.403	1.83	2.1806155	1.39E-08	2.25E-08	0.11	0.8	0.3	2.35	1.57	1.57
OsR498G0511421500.01	1.683	0.286	-2.543438	1.23E-19	1.49E-19	2.12	1.41	1.52	0.31	0.19	0.36
OsR498G0511422000.01	11.683	32.843	1.4620846	3.46E-224	9.38E-225	11.92	11.73	11.4	32.83	33.49	32.21
OsR498G0511422100.01	1.246	3.313	1.4135216	9.58E-11	1.45E-10	1.07	1.81	0.86	3.02	3.45	3.47
OsR498G0511422400.01	0.17	0.816	2.5593725	6.24E-06	1.13E-05	0.17	0.17	0.17	0.81	0.97	0.67
OsR498G0511423600.01	1.916	0.413	-2.213217	1.39E-16	1.80E-16	2.11	1.91	1.73	0.36	0.32	0.56
OsR498G0511425600.01	3.2	1.253	-1.349941	9.03E-20	1.09E-19	3.5	2.98	3.12	1.6	0.84	1.32
OsR498G0511425700.01	0.043	0.153	1.832391	0.0002304	0.0004563	0.07	0.03	0.03	0.14	0.21	0.11
OsR498G0511426100.01	0.293	3.713	3.6221082	1.46E-74	8.89E-75	0.43	0.25	0.2	3.72	3.52	3.9
OsR498G0511426500.01	0.023	2.483	6.7945889	1.74E-27	1.83E-27	0	0.07	0	2.35	1.82	3.28
OsR498G0511428600.01	0.473	0.053	-3.02559	1.22E-12	1.74E-12	0.5	0.56	0.36	0.04	0.07	0.05
OsR498G0511430900.01	18.68	6.103	-1.611966	9.80E-82	5.65E-82	19.64	17.6	18.8	6.42	6.5	5.39
OsR498G0511433500.01	0.98	0.41	-1.258251	0.0003189	0.0006382	0.94	0.87	1.13	0.18	0.67	0.38
OsR498G0511434200.01	7.67	23.403	1.6091072	7.60E-167	2.64E-167	7.27	7.72	8.02	23.06	24.34	22.81
OsR498G0511434500.01	0.97	14.926	3.9443259	0	0	0.75	0.92	1.24	15.24	15.15	14.39
OsR498G0511435600.01	1.93	0.326	-2.49591	1.31E-38	1.16E-38	1.87	2.24	1.68	0.36	0.35	0.27
OsR498G0511436500.01	4.586	1.006	-2.186864	3.96E-109	1.88E-109	4.82	4.18	4.76	1.02	0.98	1.02
OsR498G0511436800.01	22.493	5.306	-2.086139	2.87E-166	1.00E-166	22.71	21.46	23.31	5.36	4.61	5.95
OsR498G0511441300.01	0.146	0.813	2.4662631	8.36E-08	1.40E-07	0.22	0.11	0.11	0.87	0.9	0.67
OsR498G0511443400.01	6.36	26.053	1.8696273	8.19E-194	2.52E-194	6.39	6.7	5.99	27.1	24.91	26.15
OsR498G0511444700.01	0.536	1.186	1.0868847	4.50E-05	8.52E-05	0.56	0.39	0.66	1.17	1.44	0.95
OsR498G0511448600.01	0.103	3.17	4.9098697	1.94E-71	1.21E-71	0.13	0.09	0.09	3.24	3.32	2.95
OsR498G0511449000.01	59.243	193.76	1.7098296	0	0	57.51	59.66	60.56	190.54	189.3	201.44
OsR498G0511460500.01	1.12	3.226	1.4524573	4.06E-12	5.88E-12	1.14	1.51	0.71	3.56	3.15	2.97
OsR498G0511462000.01	11.83	31.413	1.2191702	2.45E-139	9.73E-140	11.73	11.67	12.09	30.67	32.13	31.44
OsR498G0611468200.01	8935.02	3080.5	-1.534408	0	0	8777.3	9060.3	8967.5	3153.6	2986	3101.9
OsR498G0611469100.01	32.36	105.13	1.6985559	0	0	30.71	32.72	33.65	101.75	102.41	111.23
OsR498G0611470900.01	26.196	55.393	1.0832896	2.24E-303	4.70E-304	26.11	26.19	26.29	55.86	56.3	54.02
OsR498G0611471700.01	1.936	0.633	-1.610553	3.32E-06	5.94E-06	2.25	1.83	1.73	0.6	0.59	0.71
OsR498G0611472600.01	1.333	2.933	1.1391547	4.51E-08	7.46E-08	1.3	1.35	1.35	3.84	2.38	2.58
OsR498G0611474500.01	1.423	3.553	1.3766345	9.75E-24	1.09E-23	1.36	1.52	1.39	3.27	3.6	3.79
OsR498G0611477700.01	7.853	3.233	-1.287647	2.59E-51	1.96E-51	7.73	8.07	7.76	3.17	2.75	3.78
OsR498G0611478000.01	12.236	28.546	1.2491668	8.92E-165	3.14E-165	12.37	12.33	12.01	27.94	31.64	26.06
OsR498G0611482900.01	3.56	1.426	-1.34528	4.09E-21	4.81E-21	3.33	3.78	3.57	1.17	1.53	1.58
OsR498G0611486100.01	0.743	2.403	1.7224307	6.73E-13	9.51E-13	0.95	0.52	0.76	2.1	2.7	2.41
OsR498G0611487900.01	21.69	5.426	-1.921369	3.05E-292	6.59E-293	22.02	20.37	22.68	5.57	5.37	5.34
OsR498G0611488400.01	4.153	0.773	-2.426841	2.27E-34	2.13E-34	4.39	4.44	3.63	0.75	1.04	0.53

OsR498G0611490100.01	0.646	0.013	-5.549152	9.91E-13	1.41E-12	0.68	0.88	0.38	0	0	0.04
OsR498G0611490300.01	13.593	4.283	-1.659707	1.18E-84	6.64E-85	13.12	13.24	14.42	4.45	3.81	4.59
OsR498G0611490500.01	1.076	2.683	1.3148089	1.02E-25	1.11E-25	1.16	0.95	1.12	2.7	2.39	2.96
OsR498G0611495200.01	0.746	1.683	1.1779434	4.73E-09	7.55E-09	0.76	0.57	0.91	1.66	1.7	1.69
OsR498G0611496000.01	5.503	11.876	1.0988126	5.40E-101	2.70E-101	5.94	4.86	5.71	11.6	12.5	11.53
OsR498G0611496300.01	12.696	30.573	1.2687405	3.15E-164	1.11E-164	12.22	13.13	12.74	30.38	31.04	30.3
OsR498G0611497000.01	2.006	4.236	1.0800762	2.04E-42	1.71E-42	2.07	1.94	2.01	4.24	4.57	3.9
OsR498G0611498500.01	0.3	1.213	2.0204501	5.09E-20	6.11E-20	0.24	0.32	0.34	1.31	0.98	1.35
OsR498G0611498800.01	3.64	10.846	1.6420805	2.17E-93	1.15E-93	3.77	3.28	3.87	10.82	10.45	11.27
OsR498G0611505800.01	35.686	175.38	2.3007309	0	0	38.27	35.01	33.78	175.21	180.38	170.54
OsR498G0611506700.01	0.223	0.02	-3.02559	0.0002126	0.0004202	0.13	0.27	0.27	0.03	0.03	0
OsR498G0611507200.01	3.833	1.59	-1.270703	2.25E-19	2.73E-19	4.12	3.54	3.84	1.68	1.5	1.59
OsR498G0611507600.01	65.26	28.613	-1.196175	4.28E-165	1.51E-165	61.6	65.36	68.82	28.06	27.72	30.06
OsR498G0611508100.01	0	0.756	7.0522938	6.15E-17	7.89E-17	0	0	0	1.14	0.53	0.6
OsR498G0611508400.01	7.063	15.193	1.1046774	4.96E-36	4.55E-36	6.33	7.41	7.45	13.63	15.58	16.37
OsR498G0611509300.01	15.47	44.343	1.4985376	0	0	15.71	14.75	15.95	43.8	42.3	46.93
OsR498G0611510600.01	1.87	11.136	2.587473	6.59E-141	2.59E-141	2.07	1.72	1.82	11.68	10.27	11.46
OsR498G0611510800.01	11.07	54.493	2.2963381	0	0	10.19	11.89	11.13	52.92	53.56	57
OsR498G0611512600.01	1.43	3.43	1.271676	1.82E-17	2.30E-17	1.24	1.86	1.19	2.94	3.46	3.89
OsR498G0611513200.01	5.44	14.663	1.430386	1.75E-82	1.00E-82	5.16	5.4	5.76	14.55	14.61	14.83
OsR498G0611513900.01	2.073	8.47	2.0559398	6.67E-41	5.74E-41	2.42	1.63	2.17	8.31	7.08	10.02
OsR498G0611514600.01	0.326	23.216	6.1542402	4.49E-214	1.27E-214	0.27	0.43	0.28	21.74	21.36	26.55
OsR498G0611518800.01	0.316	17.65	5.8050767	1.77E-86	9.85E-87	0.38	0.22	0.35	17.13	19.64	16.18
OsR498G0611524600.01	0.19	0.026	-2.780478	6.15E-06	1.12E-05	0.15	0.25	0.17	0.02	0.02	0.04
OsR498G0611527900.01	6.236	13.84	1.1476027	6.11E-12	8.88E-12	5.93	6.7	6.08	12.91	15.09	13.52
OsR498G0611538400.01	0.59	0.296	-1.038101	0.000366	0.0007347	0.38	1.28	0.11	0.03	0.45	0.41
OsR498G0611538700.01	0.603	0.07	-3.206011	3.87E-13	5.43E-13	0.83	0	0.98	0.21	0	0
OsR498G0611547600.01	3.913	16.296	2.0112535	2.57E-239	6.63E-240	3.8	4.32	3.62	16.34	17.12	15.43
OsR498G0611548600.01	16.096	6.82	-1.253987	1.68E-121	7.37E-122	16.03	15.92	16.34	6.01	7.42	7.03
OsR498G0611551300.01	1.11	0.423	-1.382284	2.48E-08	4.07E-08	1.37	1.17	0.79	0.37	0.52	0.38
OsR498G0611553000.01	0.293	0.69	1.2963381	6.65E-06	1.21E-05	0.43	0.27	0.18	0.52	0.75	0.8
OsR498G0611554200.01	1.2	2.596	1.1580807	3.44E-26	3.70E-26	0.93	1.19	1.48	2.74	2.63	2.42
OsR498G0611556400.01	30.596	13.663	-1.169211	6.01E-215	1.68E-215	31.42	30.71	29.66	14	13.71	13.28
OsR498G0611556500.01	1.616	6.866	2.0760719	4.02E-107	1.92E-107	1.73	1.73	1.39	6.59	7.47	6.54
OsR498G0611556900.01	1.75	3.5	1.0018907	1.30E-07	2.19E-07	1.61	2	1.64	3.91	3.05	3.54
OsR498G0611557700.01	12.786	35.196	1.4862153	2.88E-235	7.54E-236	12.31	11.87	14.18	33.19	33.04	39.36
OsR498G0611558100.01	0.06	3.636	5.9819045	8.40E-47	6.68E-47	0.06	0.06	0.06	3.98	3.13	3.8
OsR498G0611560500.01	0.103	0.73	2.832391	2.92E-06	5.21E-06	0	0.23	0.08	0.75	0.52	0.92
OsR498G0611560700.01	0.753	0.156	-2.154873	8.51E-06	1.56E-05	0.47	1.06	0.73	0.3	0.17	0
OsR498G0611561500.01	1.58	5.543	1.8086315	2.25E-26	2.41E-26	1.7	1.74	1.3	5.72	5.28	5.63
OsR498G0611561800.01	21.436	58.076	1.4368493	0	0	21.98	22.31	20.02	56.06	59.5	58.67
OsR498G0611562600.01	0.61	1.223	1.0100339	9.27E-05	0.0001793	0.55	0.73	0.55	1.51	1.06	1.1
OsR498G0611562900.01	0.21	0.016	-3.832945	2.03E-10	3.09E-10	0.15	0.24	0.24	0.03	0	0.02
OsR498G0611565600.01	583.51	175.87	-1.732882	0	0	593.22	560.27	597.04	169.64	168.18	189.79
OsR498G0611573000.01	0.383	0.073	-2.305698	5.00E-06	9.05E-06	0.23	0.51	0.41	0.06	0.06	0.1
OsR498G0611576400.01	0.11	1.25	3.8224069	2.56E-16	3.33E-16	0.13	0.2	0	1.23	1.49	1.03
OsR498G0611578100.01	0	7.49	8.4338416	1.07E-36	9.74E-37	0	0	0	7.11	8.48	6.88
OsR498G0611579000.01	1.45	21.02	3.8565326	7.57E-142	2.97E-142	1.21	1.2	1.94	21.32	19.34	22.4
OsR498G0611582200.01	412.05	176.04	-1.225596	0	0	410.42	419.05	406.68	176.96	182.67	168.48
OsR498G0611582700.01	5.416	37.723	2.7997699	0	0	5.7	5.37	5.18	38.93	36.02	38.22
OsR498G0611583000.01	9.19	22.803	1.3130094	2.41E-170	8.22E-171	8.76	8.55	10.26	21.91	22.35	24.15
OsR498G0611583900.01	1.826	0.106	-3.932481	2.41E-14	3.29E-14	2.26	1.48	1.74	0.19	0	0.13
OsR498G0611584800.01	0.326	2.673	3.0618728	1.16E-44	9.48E-45	0.26	0.36	0.36	2.72	2.6	2.7
OsR498G0611585200.01	0.983	2.036	1.0703344	8.68E-09	1.40E-08	1.38	0.72	0.85	2.18	1.82	2.11
OsR498G0611587600.01	0.666	1.34	1.0157508	1.96E-08	3.20E-08	0.62	0.65	0.73	1.29	1.52	1.21
OsR498G0611587800.01	5.166	21.266	2.0429187	1.61E-148	6.08E-149	5.5	5.54	4.46	20.93	22.56	20.31
OsR498G0611588800.01	10.743	31.956	1.533605	5.59E-212	1.59E-212	10.56	10.67	11	31.07	31.76	33.04
OsR498G0611589100.01	45.66	101.89	1.1607832	0	0	43.33	47.8	45.85	104.84	99.29	101.55
OsR498G0611594000.01	1.78	7.61	2.1112082	1.48E-92	7.83E-93	2.14	1.72	1.48	8.42	6.99	7.42
OsR498G0611594100.01	2.573	6.753	1.3471623	3.95E-40	3.43E-40	3.13	2.47	2.12	6.83	7.35	6.08
OsR498G0611599500.01	1.13	2.566	1.1828091	5.05E-10	7.81E-10	0.92	1.37	1.1	3.08	2.32	2.3
OsR498G0611600400.01	6.273	18.763	1.6302333	2.14E-109	1.01E-109	5.89	6.39	6.54	20.38	16.52	19.39
OsR498G0611601000.01	0.186	0.07	-1.454433	0.0004899	0.0009933	0.24	0.18	0.14	0.08	0.08	0.05
OsR498G0611602200.01	10.486	23.93	1.1881892	1.52E-187	4.81E-188	11.01	10.71	9.74	24.03	24.58	23.18
OsR498G0611602500.01	0.51	0.103	-2.288624	0.0001056	0.0002051	0.64	0.57	0.32	0	0.12	0.19
OsR498G0611603000.01	0.32	1.486	2.2223375	7.75E-20	9.32E-20	0.45	0.18	0.33	1.63	1.3	1.53
OsR498G0611603400.01	12.5	25.046	1.0081666	5.48E-82	3.16E-82	11.91	12.39	13.2	24.71	25.56	24.87
OsR498G0611603800.01	334.93	16.79	-4.320161	0	0	331.48	342.31	331	15.37	16.68	18.32
OsR498G0611610100.01	6.746	19.816	1.5656697	5.99E-171	2.03E-171	6.74	6.77	6.73	19.62	20.33	19.5

OsR498G0611610500.01	0.11	0.793	2.7817649	1.37E-13	1.90E-13	0.06	0.09	0.18	0.86	0.98	0.54
OsR498G0611611000.01	2.93	9.233	1.6167941	6.44E-87	3.57E-87	2.84	3.09	2.86	8.84	9.27	9.59
OsR498G0611616000.01	5.206	12.273	1.1644765	3.07E-54	2.25E-54	5.57	5.54	4.51	11.52	12.44	12.86
OsR498G0611616200.01	1.133	4.08	1.9256743	2.79E-56	2.00E-56	0.97	1.11	1.32	4	4.35	3.89
OsR498G0611617300.01	0.01	0.28	5.0188041	2.36E-09	3.73E-09	0	0	0.03	0.28	0.35	0.21
OsR498G0611619500.01	2.123	12.233	2.4465958	4.09E-111	1.91E-111	2.33	1.89	2.15	11.08	12.29	13.33
OsR498G0611619900.01	2.03	0.33	-2.677224	3.12E-71	1.95E-71	1.99	2.05	2.05	0.32	0.26	0.41
OsR498G0611620900.01	2.033	4.47	1.0747605	1.06E-22	1.21E-22	1.57	2.52	2.01	4.68	4.7	4.03
OsR498G0611622100.01	0.08	0.876	3.4338416	7.99E-06	1.46E-05	0	0.12	0.12	0.83	0.83	0.97
OsR498G0611623700.01	0.01	0.343	5.0618728	1.33E-09	2.09E-09	0	0.03	0	0.36	0.39	0.28
OsR498G0611624700.01	0.576	6.063	3.3894475	5.44E-43	4.54E-43	0.33	0.74	0.66	4.5	5.61	8.08
OsR498G0611630600.01	0.223	0.616	1.7613008	0.0002973	0.0005939	0.31	0.06	0.3	0.84	0.38	0.63
OsR498G0611630700.01	7.24	2.903	-1.439521	5.34E-39	4.71E-39	6.98	6.74	8	2.82	3.49	2.4
OsR498G0611631000.01	0.17	0.896	2.6420588	7.08E-14	9.75E-14	0.17	0.07	0.27	0.98	1.1	0.61
OsR498G0611631200.01	7.326	14.756	1.0100339	1.10E-21	1.28E-21	6.29	8.52	7.17	15.52	15.71	13.04
OsR498G0611631300.01	11.213	25.443	1.1711339	1.65E-97	8.42E-98	11.96	11.53	10.15	25.67	26.2	24.46
OsR498G0611632100.01	1.053	2.29	1.1219671	3.84E-08	6.34E-08	1.25	1.09	0.82	2.29	2.46	2.12
OsR498G0611632500.01	46.973	176.91	1.9127456	0	0	48.07	45.3	47.55	178.41	179.47	172.84
OsR498G0611637600.01	85.41	41.473	-1.04013	0	0	85.76	87.09	83.38	42.17	43.65	38.6
OsR498G0611637700.01	37.516	17.093	-1.145577	5.20E-106	2.51E-106	37.68	39.74	35.13	19.12	14.69	17.47
OsR498G0611638700.01	2.36	8.08	1.8218963	1.87E-53	1.38E-53	2.76	2.2	2.12	8.13	7.97	8.14
OsR498G0611639500.01	10.6	2.96	-1.974606	2.50E-131	1.04E-131	11.19	10.48	10.13	3.08	2.61	3.19
OsR498G0611642400.01	0.13	0	-4.485022	0.0003787	0.0007614	0.14	0.21	0.04	0	0	0
OsR498G0611645500.01	4.643	12.543	1.3858089	9.61E-67	6.23E-67	4.29	5.05	4.59	12.88	10.68	14.07
OsR498G0611647900.01	13.35	35.396	1.4092142	2.72E-179	8.91E-180	13.34	13.53	13.18	37.47	34.26	34.46
OsR498G0611648200.01	0.233	9.46	5.3275568	2.83E-153	1.05E-153	0.35	0.17	0.18	9.1	9.12	10.16
OsR498G0611650800.01	5.17	16.63	1.6845518	1.87E-215	5.23E-216	4.87	5.45	5.19	17.75	16.15	15.99
OsR498G0611652400.01	0.02	5.823	8.1088363	2.22E-59	1.55E-59	0	0	0.06	5.73	4.97	6.77
OsR498G0611653900.01	4.613	13.71	1.5915912	2.84E-87	1.57E-87	4.28	4.1	5.46	13.36	14.57	13.2
OsR498G0611654200.01	78.576	27.31	-1.509684	0	0	74.63	78.65	82.45	27.35	25.65	28.93
OsR498G0611656300.01	161.123	18.24	-3.13997	0	0	157.26	166.5	159.61	16.1	18.67	19.95
OsR498G0611656900.01	152.713	22.43	-2.769353	0	0	151.8	152.78	153.56	22.82	20.48	23.99
OsR498G0611657500.01	3.95	10.81	1.4519434	2.59E-28	2.68E-28	4.38	4.02	3.45	10.84	10.72	10.87
OsR498G0611659400.01	0.22	0.906	1.6786714	2.99E-17	3.80E-17	0.24	0.24	0.18	0.68	0.95	1.09
OsR498G0611659600.01	2.09	9.11	2.1128919	2.34E-67	1.51E-67	2.36	1.48	2.43	8.6	9.73	9
OsR498G0611661100.01	2.066	4.173	1.0114569	4.47E-15	5.98E-15	1.77	2.21	2.22	3.99	4.78	3.75
OsR498G0611662800.01	9.09	23.286	1.3553226	1.98E-80	1.15E-80	8.42	9.09	9.76	21.94	22.74	25.18
OsR498G0611665000.01	108.02	12.86	-3.06847	0	0	109.51	106.43	108.12	12.75	13.46	12.37
OsR498G0611665800.01	1.336	0.416	-1.682702	3.92E-05	7.41E-05	1.18	1.56	1.27	0.57	0.19	0.49
OsR498G0611671900.01	0.113	0.383	1.7399447	0.0001282	0.0002502	0.17	0.14	0.03	0.5	0.2	0.45
OsR498G0611672200.01	0	0.386	5.6182661	1.33E-07	2.24E-07	0	0	0	0.5	0.36	0.3
OsR498G0611673700.01	61.776	14.62	-2.071998	0	0	63.52	60.9	60.91	15	14.69	14.17
OsR498G0611674100.01	2.823	1.013	-1.474897	1.75E-16	2.26E-16	2.91	2.63	2.93	1	0.82	1.22
OsR498G0611677500.01	0.456	6.203	3.6099985	1.63E-102	8.04E-103	0.45	0.46	0.46	6.07	6.17	6.37
OsR498G0611682700.01	0.98	2.24	1.1968024	2.24E-10	3.42E-10	0.95	1.05	0.94	2.33	2.43	1.96
OsR498G0611684100.01	7.963	28.383	1.83232	1.62E-235	4.24E-236	7.79	8.49	7.61	29.06	27.81	28.28
OsR498G0611684400.01	11.84	24.316	1.0249772	0	0	11.65	12.39	11.48	23.53	25.16	24.26
OsR498G0611686700.01	0.893	3	1.7371596	1.47E-19	1.78E-19	1.06	0.79	0.83	3.16	2.81	3.03
OsR498G0611687600.01	1.773	14.66	3.0421546	1.57E-183	5.05E-184	1.93	1.63	1.76	14.8	14.14	15.04
OsR498G0611687800.01	0.49	4.74	3.2784161	1.51E-144	5.81E-145	0.55	0.47	0.45	4.64	4.68	4.9
OsR498G0611697700.01	0.756	2.216	1.5475953	1.18E-10	1.78E-10	0.84	0.88	0.55	2.65	1.78	2.22
OsR498G0611698300.01	54	20.22	-1.428906	0	0	54.05	52.97	54.98	21.23	19.57	19.86
OsR498G0611699900.01	12.466	44.976	1.8933522	2.05E-221	5.61E-222	12.6	11.73	13.07	43.71	41.43	49.79
OsR498G0611707500.01	0.196	0.506	1.3804023	5.92E-05	0.0001131	0.14	0.12	0.33	0.55	0.35	0.62
OsR498G0611710100.01	0.963	6.74	2.8038756	8.79E-83	5.03E-83	0.98	0.82	1.09	6.63	6.45	7.14
OsR498G0611713700.01	11.956	35.42	1.522047	5.12E-222	1.40E-222	12.23	11.92	11.72	37.21	34.87	34.18
OsR498G0611714100.01	6.036	37.686	2.2066854	6.09E-265	1.42E-265	6.32	5.8	5.99	33.88	38.54	40.64
OsR498G0611716600.01	0.946	0.066	-3.847592	3.32E-22	3.82E-22	0.85	0.91	1.08	0.09	0.11	0
OsR498G0611717600.01	1.73	3.933	1.1216734	3.53E-31	3.49E-31	1.54	1.78	1.87	3.77	4.18	3.85
OsR498G0611719200.01	0.646	3.343	2.3677524	8.47E-47	6.74E-47	0.38	0.86	0.7	3.25	3.09	3.69
OsR498G0611724500.01	1.093	2.87	1.3540535	5.59E-17	7.16E-17	1.24	0.93	1.11	2.52	2.91	3.18
OsR498G0611725800.01	17.48	53.383	1.6137725	2.65E-91	1.42E-91	17.65	16.64	18.15	51.32	52.4	56.43
OsR498G0611728600.01	1.01	2.836	1.4902481	5.28E-13	7.43E-13	1.12	0.9	1.01	2.07	3.04	3.4
OsR498G0611733200.01	0.3	1.003	2.1916407	1.22E-12	1.73E-12	0.28	0.28	0.34	0.65	1.03	1.33
OsR498G0611734800.01	2.053	6.3	1.6221082	9.55E-24	1.07E-23	2.07	2.18	1.91	6.38	6.18	6.34
OsR498G0611737800.01	19.546	62.116	1.6656156	9.04E-120	4.00E-120	21.98	18.08	18.58	57.96	60.48	67.91
OsR498G0611738100.01	0.566	0.133	-2.02559	1.81E-06	3.21E-06	0.48	0.48	0.74	0.11	0.22	0.07
OsR498G0611738500.01	1.073	0.113	-3.247982	5.44E-17	6.96E-17	0.93	1.03	1.26	0.11	0.19	0.04
OsR498G0611739100.01	1.146	0.023	-5.235043	1.63E-10	2.48E-10	1.25	1.1	1.09	0	0.07	0

OsR498G0611741100.01	0.28	1.803	2.6748497	1.73E-07	2.93E-07	0.42	0.28	0.14	2.35	1.51	1.55
OsR498G0611741300.01	0.23	0.796	1.7938377	3.91E-07	6.74E-07	0.16	0.16	0.37	1.03	0.63	0.73
OsR498G0611742200.01	6.033	12.446	1.0227298	1.77E-59	1.23E-59	5.9	6.01	6.19	12.61	12.58	12.15
OsR498G0611742500.01	3.476	8.003	1.2019457	2.10E-43	1.74E-43	3.73	2.85	3.85	8.22	8.3	7.49
OsR498G0611744100.01	0.043	0.236	2.637375	2.97E-07	5.08E-07	0.02	0.06	0.05	0.24	0.24	0.23
OsR498G0611746400.01	1.463	0.03	-5.580179	1.98E-46	1.58E-46	1.43	1.44	1.52	0	0.07	0.02
OsR498G0611748600.01	0.1	0.646	2.690617	1.43E-08	2.32E-08	0.04	0.09	0.17	0.46	0.79	0.69
OsR498G0611757800.01	1.07	0.2	-2.485022	1.52E-24	1.67E-24	1.1	0.9	1.21	0.12	0.16	0.32
OsR498G0611758700.01	0.106	0.49	2.2223375	3.03E-06	5.41E-06	0	0.12	0.2	0.3	0.46	0.71
OsR498G0611759700.01	2.863	5.836	1.0282028	2.62E-34	2.46E-34	2.86	2.55	3.18	6.01	5.76	5.74
OsR498G0611761500.01	1.233	6.73	2.4480275	1.17E-69	7.39E-70	1.21	0.84	1.65	6.54	6.2	7.45
OsR498G0611763000.01	1.556	0	-6.932481	1.39E-15	1.83E-15	1.8	1.32	1.55	0	0	0
OsR498G0611763200.01	17.153	52.836	1.5998483	0	0	16.96	17.45	17.05	53.76	53.65	51.1
OsR498G0611777900.01	4.746	11.533	1.2700382	9.33E-43	7.81E-43	5.09	4.86	4.29	12.01	10.59	12
OsR498G0611780400.01	0.443	0.036	-3.610553	0.0004877	0.0009887	0.67	0.44	0.22	0.11	0	0
OsR498G0611782500.01	1.406	5.88	2.0607255	5.88E-137	2.37E-137	1.24	1.42	1.56	6.08	6.16	5.4
OsR498G0611782900.01	53.386	113.15	1.0474925	1.39E-272	3.18E-273	52.41	53.22	54.53	116.15	110.88	112.41
OsR498G0611788200.01	2.486	1.02	-1.288624	1.67E-08	2.72E-08	3.31	2.04	2.11	1.15	0.79	1.12
OsR498G0611788500.01	2.703	6.126	1.0547468	2.89E-18	3.59E-18	2.79	2.38	2.94	6.06	6.49	5.83
OsR498G0611789000.01	27.84	70.223	1.3319456	1.78E-276	4.00E-277	27.13	28.26	28.13	70.39	68.83	71.45
OsR498G0611790800.01	62.553	27.7	-1.177333	7.86E-199	2.36E-199	67.25	58.36	62.05	27.82	24.28	31
OsR498G0611790900.01	1.646	0.826	-1.001745	5.94E-12	8.63E-12	1.76	1.26	1.92	0.53	0.7	1.25
OsR498G0611792500.01	2.43	9.873	2.0306782	4.40E-79	2.59E-79	2.15	2.33	2.81	10.43	9.37	9.82
OsR498G0611793200.01	0.786	15.33	4.1418281	1.55E-244	3.93E-245	0.95	0.58	0.83	16.43	15.99	13.57
OsR498G0611794600.01	0.26	2.196	3.0848339	6.56E-18	8.21E-18	0.43	0.14	0.21	2.62	1.85	2.12
OsR498G0611795300.01	0.156	6.783	5.4541902	8.42E-85	4.74E-85	0.23	0.12	0.12	5.98	7.41	6.96
OsR498G0611795800.01	0.346	32.863	6.139177	0	0	0.48	0.27	0.29	34.23	32.93	31.43
OsR498G0611800600.01	0.6	1.463	1.2885185	8.06E-07	1.41E-06	0.88	0.39	0.53	1.09	1.84	1.46
OsR498G0611801500.01	1.79	0.31	-2.53255	5.13E-17	6.57E-17	1.75	1.68	1.94	0.29	0.34	0.3
OsR498G0611801700.01	3.373	1.346	-1.334139	2.53E-11	3.74E-11	3.22	3.01	3.89	1.17	1.04	1.83
OsR498G0611807100.01	3.466	27.55	3.0642205	0	0	3.17	3.7	3.53	28.95	27.19	26.51
OsR498G0611812900.01	9.113	27.806	1.6115809	5.82E-97	2.99E-97	8.52	10.29	8.53	28.09	27.11	28.22
OsR498G0611817500.01	2.57	1.05	-1.321046	3.08E-11	4.57E-11	3.11	1.67	2.93	1.07	0.93	1.15
OsR498G0611820900.01	18.47	50.443	1.4482514	4.28E-119	1.90E-119	18.87	18.39	18.15	50.07	50.28	50.98
OsR498G0611822700.01	2.376	6.13	1.3995792	2.59E-58	1.82E-58	2.61	2.36	2.16	5.63	6.13	6.63
OsR498G0611824500.01	0.54	0.23	-1.242401	2.69E-06	4.79E-06	0.72	0.58	0.32	0.24	0.22	0.23
OsR498G0611826900.01	0.033	1.906	5.8692277	1.31E-29	1.33E-29	0.05	0.05	0	2.29	1.71	1.72
OsR498G0611833300.01	1.983	5.21	1.3894475	4.91E-28	5.11E-28	2.1	1.9	1.95	4.09	5.57	5.97
OsR498G0611836900.01	13.75	32.606	1.2435966	6.55E-85	3.68E-85	13.22	13.62	14.41	31.5	32.81	33.51
OsR498G0611837500.01	0.533	0.196	-1.440628	0.0002392	0.0004746	0.48	0.52	0.6	0.12	0.19	0.28
OsR498G0611841300.01	0.723	0.286	-1.266598	0.0001558	0.0003056	0.72	0.77	0.68	0.46	0.21	0.19
OsR498G0611841700.01	39.066	78.676	1.006572	1.34E-132	5.53E-133	37.58	36.35	43.27	79.3	79.63	77.1
OsR498G0611846200.01	0.896	0.256	-1.805809	4.72E-09	7.53E-09	0.79	0.61	1.29	0.17	0.26	0.34
OsR498G0611848000.01	2.363	4.88	1.0333036	2.51E-16	3.26E-16	2.54	2	2.55	4.41	4.91	5.32
OsR498G0611849700.01	0.073	1.73	4.5593725	1.85E-12	2.65E-12	0.11	0.11	0	1.94	1.82	1.43
OsR498G0611849900.01	12.133	30.4	1.3357173	1.86E-134	7.61E-135	12.81	11.46	12.13	28.7	30.22	32.28
OsR498G0611850100.01	0.586	3.78	2.687128	1.31E-36	1.20E-36	0.74	0.68	0.34	3.91	3.27	4.16
OsR498G0611853300.01	1.106	10.433	3.2340319	1.35E-165	4.75E-166	1.17	1.09	1.06	10.46	9.88	10.96
OsR498G0611854400.01	2.766	6.06	1.1397714	1.99E-80	1.16E-80	2.99	2.65	2.66	5.68	6.38	6.12
OsR498G0611854800.01	0	4.333	9.3710147	2.41E-61	1.64E-61	0	0	0	4.82	3.46	4.72
OsR498G0611856400.01	0.603	10.22	4.0823751	3.58E-169	1.23E-169	0.47	0.66	0.68	10.05	9.98	10.63
OsR498G0611857200.01	3.333	124.59	5.1613582	0	0	3.22	3.46	3.32	122.06	124.41	127.31
OsR498G0611857400.01	0.72	32.05	5.4847869	0	0	0.76	0.67	0.73	30.3	32.62	33.23
OsR498G0611857600.01	1.736	69.04	5.3167841	0	0	2	1.35	1.86	69.42	66.12	71.58
OsR498G0611857800.01	0	0.233	5.5593725	2.32E-07	3.95E-07	0	0	0	0.38	0.17	0.15
OsR498G0611858400.01	13.37	3.916	-1.773672	2.52E-56	1.81E-56	12.93	12.29	14.89	3.15	4.17	4.43
OsR498G0611862200.01	0.29	1.61	2.4637948	1.39E-11	2.05E-11	0.47	0.13	0.27	1.12	1.96	1.75
OsR498G0611862900.01	2.746	1.113	-1.296444	2.24E-14	3.04E-14	3.13	2.71	2.4	1.17	0.76	1.41
OsR498G0611865000.01	1.556	5.103	1.7029633	2.96E-49	2.29E-49	1.38	1.84	1.45	5.19	5.76	4.36
OsR498G0611865600.01	0.126	2.533	4.3178178	1.05E-45	8.47E-46	0.15	0.04	0.19	2.37	2.18	3.05
OsR498G0611867100.01	0.92	0	-6.580179	8.73E-13	1.24E-12	0.71	1.05	1	0	0	0
OsR498G0611870100.01	19.376	65.676	1.5087464	6.47E-248	1.61E-248	19.51	21.12	17.5	60.49	67.78	68.76
OsR498G0611870800.01	1.756	3.576	1.0333036	2.18E-14	2.97E-14	1.71	1.93	1.63	3.39	3.02	4.32
OsR498G0611873300.01	0.29	0	-5.832945	1.96E-08	3.20E-08	0.25	0.31	0.31	0	0	0
OsR498G0611881900.01	0.94	2.583	1.4550358	1.27E-33	1.21E-33	1.13	0.75	0.94	2.56	2.66	2.53
OsR498G0611885200.01	8.896	27.806	1.6423704	2.09E-89	1.14E-89	9.76	7.77	9.16	25.97	26.98	30.47
OsR498G0611885800.01	2.343	1.013	-1.278781	5.80E-38	5.18E-38	2.11	2.59	2.33	1.09	0.87	1.08
OsR498G0611889100.01	0.33	0.776	1.2555104	3.82E-09	6.08E-09	0.44	0.1	0.45	0.83	1.07	0.43
OsR498G0611889400.01	280.296	65.99	-2.086661	0	0	266.79	298.77	275.33	67.21	68.82	61.94

OsR498G0611891900.01	0.183	0.043	-2.099013	8.32E-05	0.0001605	0.35	0.1	0.1	0.08	0.05	0
OsR498G0611893900.01	0.77	0.37	-1.044202	0.0009265	0.0019254	0.9	0.75	0.66	0.47	0.3	0.34
OsR498G0611899400.01	0.14	0	-4.969511	2.66E-05	4.97E-05	0	0	0.42	0	0	0
OsR498G0611899800.01	0.08	0.323	2.0126518	9.08E-06	1.66E-05	0.05	0.19	0	0.37	0.26	0.34
OsR498G0611900200.01	26.49	3.3	-3.006854	0	0	28.07	26.77	24.63	3.33	3.04	3.53
OsR498G0611902300.01	4.846	1.856	-1.380735	8.49E-38	7.59E-38	4.97	4.26	5.31	2.01	1.98	1.58
OsR498G0611905300.01	6.896	2.656	-1.37842	1.71E-45	1.39E-45	6.76	7.32	6.61	2.56	2.32	3.09
OsR498G0611907700.01	2.423	6.6	1.4474796	2.75E-26	2.95E-26	2.5	2.85	1.92	6.59	6.62	6.59
OsR498G0611908600.01	0.506	0.993	1.0762896	2.32E-05	4.34E-05	0.42	0.62	0.48	1.14	0.94	0.9
OsR498G0611909400.01	0.19	0.486	1.3529216	8.60E-05	0.0001661	0.17	0.2	0.2	0.59	0.53	0.34
OsR498G0611911600.01	5.54	13.273	1.333334	2.99E-102	1.48E-102	5.81	5.5	5.31	12.8	13.3	13.72
OsR498G0611912000.01	2.306	13.073	2.5163037	1.03E-96	5.32E-97	2.24	2.41	2.27	11.92	12.8	14.5
OsR498G0611912800.01	1.056	8.793	3.05533	5.85E-278	1.31E-278	0.88	1.13	1.16	9.3	8.96	8.12
OsR498G0611917700.01	0.19	0.03	-2.688555	0.0001499	0.0002937	0.18	0.18	0.21	0.06	0	0.03
OsR498G0611920000.01	1.476	10.36	2.8077962	7.73E-114	3.56E-114	1.27	1.51	1.65	9.71	10.43	10.94
OsR498G0611933700.01	8.846	36.5	2.0402397	0	0	9.67	8.78	8.09	35.74	37.68	36.08
OsR498G0611936300.01	0.726	0.156	-2.279347	1.68E-09	2.64E-09	0.81	0.66	0.71	0.25	0.22	0
OsR498G0611937400.01	0.136	0.916	2.7799913	3.43E-24	3.80E-24	0.09	0.08	0.24	0.91	1.14	0.7
OsR498G0611939200.01	0.103	0.92	3.144335	4.16E-11	6.21E-11	0.1	0.21	0	0.81	1.11	0.84
OsR498G0611941700.01	19.53	7.663	-1.349712	1.19E-100	5.94E-101	20.74	19.13	18.72	7.53	7.64	7.82
OsR498G0611945600.01	2.526	6.183	1.3179519	1.73E-32	1.67E-32	2.35	2.72	2.51	5.99	7.1	5.46
OsR498G0611947300.01	5.763	2.7	-1.092422	2.77E-12	3.98E-12	5.86	6.1	5.33	2.78	3.04	2.28
OsR498G0611952100.01	2.193	4.816	1.1007264	3.83E-16	4.99E-16	1.55	2.6	2.43	4.9	4.93	4.62
OsR498G0611957000.01	0.52	0.143	-1.873587	5.56E-07	9.63E-07	0.49	0.49	0.58	0.2	0.06	0.17
OsR498G0611959400.01	0.186	0.546	1.3749479	9.52E-06	1.75E-05	0.2	0.18	0.18	0.5	0.52	0.62
OsR498G0611960200.01	4.526	9.043	1.3741069	1.12E-22	1.28E-22	4.42	4.12	5.04	8.98	9.65	8.5
OsR498G0611962900.01	2.21	8.93	2.1079508	3.31E-58	2.33E-58	2.47	2.12	2.04	9.63	7.97	9.19
OsR498G0611963200.01	0.623	3.016	2.2428988	6.99E-21	8.25E-21	0.5	0.46	0.91	3.42	2.56	3.07
OsR498G0611963700.01	0.426	1.463	1.7695902	6.08E-07	1.05E-06	0.3	0.53	0.45	1.48	1.32	1.59
OsR498G0611965600.01	2.33	0.713	-1.722445	3.82E-19	4.67E-19	2.42	2.27	2.3	0.54	0.77	0.83
OsR498G0611970000.01	1.083	2.296	1.0813252	8.83E-09	1.42E-08	1.01	0.94	1.3	2.16	2.31	2.42
OsR498G0611970300.01	0	0.213	4.97441	2.21E-05	4.12E-05	0	0	0	0.32	0.24	0.08
OsR498G0611970800.01	19.436	47.45	1.2843807	1.44E-44	1.17E-44	18	18.75	21.56	47.51	45.52	49.32
OsR498G0611971500.01	0.236	0.61	1.3614331	8.27E-06	1.51E-05	0.22	0.27	0.22	0.64	0.72	0.47
OsR498G0611980100.01	0.203	0.713	1.8003806	3.15E-05	5.92E-05	0.22	0.12	0.27	0.93	0.41	0.8
OsR498G0611980400.01	0.303	3.256	3.3351573	8.39E-43	7.02E-43	0.34	0.18	0.39	3.05	3.43	3.29
OsR498G0611981300.01	1.013	2.65	1.396643	1.06E-22	1.21E-22	1.23	0.93	0.88	2.86	2.73	2.36
OsR498G0611981600.01	0.716	2.366	1.696876	1.70E-15	2.25E-15	0.45	0.83	0.87	2.33	2.29	2.48
OsR498G0611989000.01	284.333	137.86	-1.046147	0	0	276.45	289.47	287.08	139.65	132.19	141.74
OsR498G0611996700.01	22.866	50.1	1.1359396	0	0	23.17	22.96	22.47	52.4	49.66	48.24
OsR498G0611997000.01	0.69	0.053	-3.72603	6.62E-15	8.89E-15	0.51	0.83	0.73	0.04	0.12	0
OsR498G0612005300.01	0.043	1.57	5.1838633	2.42E-10	3.69E-10	0	0.13	0	1.77	1.13	1.81
OsR498G0612005800.01	0.163	0.756	2.1820054	4.78E-13	6.72E-13	0.23	0.14	0.12	0.79	0.75	0.73
OsR498G0612008200.01	8.043	1.203	-2.746935	6.68E-199	2.01E-199	7.59	8.3	8.24	1.23	1.17	1.21
OsR498G0612009500.01	1.62	3.5	1.0592989	6.38E-16	8.37E-16	1.45	1.67	1.74	3.57	3.29	3.64
OsR498G0612011200.01	0.466	1.013	1.1811921	9.67E-07	1.69E-06	0.27	0.51	0.62	0.94	1.34	0.76
OsR498G0612013800.01	5.263	12.483	1.2455254	8.27E-127	3.53E-127	4.91	5.29	5.59	12.71	12.24	12.5
OsR498G0612015400.01	7.686	3.716	-1.052258	6.96E-13	9.84E-13	8.99	6.32	7.75	3.3	3.12	4.73
OsR498G0612016600.01	0.03	0.47	4.0618728	1.76E-12	2.52E-12	0.03	0.03	0.03	0.47	0.38	0.56
OsR498G0612017700.01	0.01	0.173	3.97441	5.14E-05	9.78E-05	0.03	0	0	0.16	0.26	0.1
OsR498G0612018000.01	233.32	900.94	1.9521855	0	0	229.9	237.49	232.57	906.53	899.37	896.93
OsR498G0612018800.01	0.573	2.103	1.9458921	1.30E-10	1.97E-10	0.67	0.82	0.23	1.7	1.9	2.71
OsR498G0612024500.01	4.576	30.23	2.6951492	0	0	4.2	5.12	4.41	30.95	31.77	27.97
OsR498G0612025600.01	0.04	0.45	3.5593725	0.0005724	0.0011661	0.12	0	0	0.56	0.45	0.34
OsR498G0612026500.01	1.703	5.61	1.7307409	2.15E-44	1.76E-44	1.72	1.59	1.8	5.04	6.54	5.25
OsR498G0612027700.01	31.79	11.763	-1.444647	4.83E-241	1.24E-241	32.08	32.51	30.78	11.42	12.16	11.71
OsR498G0612031200.01	8.936	22.393	1.3391063	8.97E-53	6.68E-53	8.41	9.27	9.13	22.25	20.99	23.94
OsR498G0612033300.01	0.396	0.033	-3.417907	1.09E-05	2.00E-05	0.61	0.29	0.29	0	0.05	0.05
OsR498G0612037000.01	0.11	0.026	-2.273518	0.0004936	0.0010012	0.07	0.14	0.12	0.02	0.02	0.04
OsR498G0612038100.01	21.796	45.076	1.0484437	0	0	22.32	22.16	20.91	46.13	46.71	42.39
OsR498G0612043200.01	5.14	22.453	2.1260728	1.58E-212	4.49E-213	5.34	5.32	4.76	22.04	20.68	24.64
OsR498G0612051500.01	1.16	0.213	-2.438372	1.67E-07	2.82E-07	1.64	0.98	0.86	0.32	0.32	0
OsR498G0612054600.01	0.186	0	-4.832945	6.10E-05	0.0001167	0.28	0.12	0.16	0	0	0
OsR498G0612055300.01	0.02	0.18	3.2223375	4.64E-05	8.81E-05	0	0.03	0.03	0.11	0.2	0.23
OsR498G0612058000.01	2.3	0.09	-4.691413	1.62E-14	2.21E-14	2.07	2.13	2.7	0	0	0.27
OsR498G0612066600.01	11.343	5.396	-1.070947	4.82E-117	2.17E-117	12.04	11.17	10.82	5.42	5.58	5.19
OsR498G0612069900.01	7.603	26.306	1.7898399	4.00E-249	9.92E-250	7.53	7.82	7.46	27.43	26.73	24.76
OsR498G0612083600.01	0.623	0.166	-1.900059	0.0007746	0.0015958	0.43	0.34	1.1	0.25	0.08	0.17
OsR498G0612083900.01	0.403	1.283	1.6683068	1.07E-10	1.61E-10	0.36	0.25	0.6	1.28	1.07	1.5

OsR498G0612084600.01	13.51	28.453	1.0775272	1.02E-94	5.32E-95	11.99	14.3	14.24	27.98	27.92	29.46
OsR498G0612084700.01	4.543	9.24	1.0112467	3.80E-15	5.08E-15	4.99	3.66	4.98	9.61	9.14	8.97
OsR498G0612085000.01	1.82	4.916	1.4353837	1.64E-62	1.11E-62	1.7	1.93	1.83	5.17	4.65	4.93
OsR498G0612085100.01	0.273	2.256	3.4006747	1.10E-17	1.38E-17	0.25	0.25	0.32	2.24	1.92	2.61
OsR498G0612087300.01	2.69	0.463	-2.638033	3.18E-25	3.47E-25	2.56	2.78	2.73	0.29	0.7	0.4
OsR498G0612087700.01	3.98	1.586	-1.326422	1.29E-37	1.16E-37	4.32	4	3.62	1.62	1.51	1.63
OsR498G0612088200.01	0.29	0.806	1.4813699	2.13E-05	3.97E-05	0.37	0.23	0.27	0.71	1.02	0.69
OsR498G0612116600.01	0.563	0	-6.417907	1.13E-11	1.65E-11	0.93	0.44	0.32	0	0	0
OsR498G0612122900.01	1.343	3.133	1.2223375	1.60E-08	2.61E-08	1.23	1.58	1.22	3.65	2.87	2.88
OsR498G0612131000.01	0.046	0.363	2.97441	1.18E-05	2.17E-05	0.05	0.09	0	0.32	0.4	0.37
OsR498G0612134400.01	0.36	0.086	-2.02559	0.000819	0.0016919	0.27	0.32	0.49	0.05	0.16	0.05
OsR498G0612134800.01	3.003	10.536	1.7616398	2.71E-200	8.12E-201	3.15	2.93	2.93	10.95	10.31	10.35
OsR498G0612136200.01	0.266	0.563	1.0618728	0.000998	0.0020806	0.3	0.2	0.3	0.36	0.62	0.71
OsR498G0612137700.01	0.213	0	-4.485022	0.0003787	0.0007614	0.23	0.29	0.12	0	0	0
OsR498G0612146400.01	0.653	0.09	-2.755983	4.58E-13	6.44E-13	0.54	0.71	0.71	0.04	0.14	0.09
OsR498G0612148100.01	4.71	0.643	-2.879041	4.95E-53	3.68E-53	5.05	4.42	4.66	0.48	0.6	0.85
OsR498G0612159500.01	60.406	331.62	2.419872	0	0	59.68	62.59	58.95	352.53	325.52	316.82
OsR498G0612159700.01	0.65	1.54	1.2448078	3.62E-16	4.72E-16	0.62	0.58	0.75	1.87	1.51	1.24
OsR498G0612167300.01	7.756	18.98	1.2893062	2.93E-86	1.63E-86	7.36	7.84	8.07	16.73	19.41	20.8
OsR498G0612170100.01	0.93	22.156	4.6340491	4.85E-227	1.30E-227	1.05	1.13	0.61	22.13	21.52	22.82
OsR498G0612174700.01	1.05	0.463	-1.179624	1.71E-09	2.68E-09	0.98	1.05	1.12	0.39	0.52	0.48
OsR498G0612176400.01	0.026	0.173	2.6182661	2.64E-05	4.95E-05	0.04	0.04	0	0.23	0.08	0.21
OsR498G0612183500.01	0.62	0.146	-2.02559	0.0002922	0.0005834	0.65	0.87	0.34	0.25	0	0.19
OsR498G0612189500.01	0.55	4.846	3.0282768	5.58E-97	2.86E-97	0.56	0.63	0.46	5.17	4.89	4.48
OsR498G0612190900.01	6	12.68	1.0711423	4.50E-116	2.04E-116	5.89	5.91	6.2	12.48	12.89	12.67
OsR498G0612193200.01	0.903	2.333	1.4273769	1.46E-12	2.09E-12	0.97	0.78	0.96	2.58	2.05	2.37
OsR498G0612201000.01	4.71	10.45	1.1495811	3.65E-27	3.86E-27	5.06	4.5	4.57	9.82	10.63	10.9
OsR498G0612202200.01	0.286	1.033	1.857053	2.65E-07	4.53E-07	0.43	0.11	0.32	1.15	1.04	0.91
OsR498G0612204800.01	10.18	23.25	1.1660746	6.51E-71	4.07E-71	10.48	10.78	9.28	25.49	21.78	22.48
OsR498G0612218200.01	1.193	0.196	-2.59848	4.75E-19	5.80E-19	1.21	1.29	1.08	0.15	0.23	0.21
OsR498G0612226900.01	8.993	24.756	1.4700722	1.96E-178	6.44E-179	9.8	8.41	8.77	25.6	24.42	24.25
OsR498G0612227900.01	1.013	2.343	1.2188287	1.47E-09	2.29E-09	0.95	0.73	1.36	2.22	2.41	2.4
OsR498G0612230200.01	5.49	10.486	1.0314217	1.48E-48	1.16E-48	4.81	6.05	5.61	11.1	10.55	9.81
OsR498G0612238800.01	6.706	14.343	1.1672326	1.03E-95	5.36E-96	6.71	6.42	6.99	14.38	14.77	13.88
OsR498G0612251700.01	0.313	0.08	-1.900059	0.0007746	0.0015958	0.22	0.38	0.34	0.08	0.12	0.04
OsR498G0612253000.01	0.28	0.86	1.6099985	1.81E-08	2.96E-08	0.33	0.33	0.18	0.72	0.81	1.05
OsR498G0612254400.01	1.806	8.76	2.2730683	8.82E-32	8.64E-32	1.31	1.95	2.16	7.84	7.38	11.06
OsR498G0612259400.01	0.03	0.24	2.97441	0.0002639	0.0005255	0	0.09	0	0.18	0.22	0.32
OsR498G0612260000.01	0.1	3.076	4.8488791	9.57E-42	8.14E-42	0.05	0.05	0.2	2.6	2.96	3.67
OsR498G0612260200.01	0.296	0	-4.832945	6.10E-05	0.0001167	0.52	0.16	0.21	0	0	0
OsR498G0612261200.01	0.073	2.19	4.9051473	1.30E-29	1.31E-29	0.11	0.06	0.05	1.93	2.47	2.17
OsR498G0612264100.01	16.463	4.33	-1.927941	1.77E-73	1.09E-73	18.21	16.4	14.78	3.44	4.7	4.85
OsR498G0612272100.01	8.736	17.876	1.0321672	2.43E-83	1.39E-83	7.65	9.39	9.17	18.23	17.29	18.11
OsR498G0612272400.01	25.166	315.66	3.6468773	0	0	25.79	25.21	24.5	310.27	311.29	325.41
OsR498G0612274100.01	3.536	13.096	1.9021879	5.17E-49	4.02E-49	3.54	2.86	4.21	14.45	11.97	12.87
OsR498G0612274300.01	11.766	2.946	-1.999172	4.05E-53	3.01E-53	13.18	11.63	10.49	2.9	2.89	3.05
OsR498G0612275100.01	12.126	25.64	1.0777701	7.61E-118	3.41E-118	12.44	12.32	11.62	26.46	25.47	24.99
OsR498G0612275400.01	2.026	0.77	-1.241908	3.74E-10	5.75E-10	1.99	2.21	1.88	0.84	0.68	0.79
OsR498G0612275900.01	1344.62	165.72	-3.027702	0	0	1364.1	1308.2	1361.6	161.72	156.31	179.14
OsR498G0612281500.01	0.123	0.94	2.9516899	4.92E-12	7.14E-12	0	0.23	0.14	0.71	0.84	1.27
OsR498G0612282000.01	16.643	6.973	-1.272292	7.31E-43	6.11E-43	17.74	16.18	16.01	7.66	7	6.26
OsR498G0612290300.01	95.353	22.453	-2.086483	0	0	92.13	101.05	92.88	23.19	22.28	21.89
OsR498G0612291200.01	0.326	2.893	3.1397477	8.56E-56	6.18E-56	0.28	0.31	0.39	2.82	2.73	3.13
OsR498G0612294100.01	0.646	2.763	2.0928047	5.82E-20	6.99E-20	0.72	0.81	0.41	2.19	3.18	2.92
OsR498G0612294900.01	11.636	27.593	1.2375626	1.01E-127	4.28E-128	12.51	12.67	9.73	27.58	27.5	27.7
OsR498G0612303700.01	0.23	0.03	-3.02559	8.53E-06	1.56E-05	0.24	0.27	0.18	0.06	0	0.03
OsR498G0612304200.01	0.093	0.68	2.8850333	5.15E-11	7.71E-11	0.17	0	0.11	0.48	0.56	1
OsR498G0612306400.01	2.736	8.343	1.6061667	1.95E-23	2.19E-23	3.62	2.43	2.16	7.75	7.98	9.3
OsR498G0612309600.01	0.99	0.46	-1.222627	1.33E-06	2.34E-06	1.11	0.78	1.08	0.38	0.46	0.54
OsR498G0612313400.01	3.286	9.96	1.5994827	4.37E-96	2.26E-96	3.06	3.56	3.24	10.26	10.12	9.5
OsR498G0612314100.01	3.146	0.173	-4.195515	3.71E-29	3.79E-29	2.56	3.36	3.52	0.29	0.15	0.08
OsR498G0612315800.01	2.15	7.28	1.7542172	1.88E-107	8.99E-108	2.4	2.1	1.95	7.14	7.8	6.9
OsR498G0612317500.01	2.976	1.29	-1.06741	1.62E-10	2.46E-10	2.89	2.98	3.06	0.9	1.37	1.6
OsR498G0612318600.01	2.313	0.193	-3.61577	2.55E-56	1.83E-56	2.42	2.74	1.78	0.24	0.19	0.15
OsR498G0612323800.01	1.933	0.406	-2.244014	1.36E-16	1.76E-16	1.88	1.67	2.25	0.33	0.51	0.38
OsR498G0612327800.01	0.526	1.746	1.6634809	1.51E-14	2.04E-14	0.62	0.4	0.56	1.62	1.92	1.7
OsR498G0612331200.01	17.32	62.09	1.8426274	0	0	16.78	17.77	17.41	61.1	62.28	62.89
OsR498G0612334700.01	0.276	0.81	1.5593725	3.70E-12	5.35E-12	0.3	0.24	0.29	0.79	0.73	0.91
OsR498G0612340400.01	0.05	0.213	2.1119135	0.0003981	0.0008014	0	0.06	0.09	0.11	0.23	0.3

OsR498G0612350400.01	1.3	2.896	1.1398336	1.07E-12	1.52E-12	1.32	1.45	1.13	2.96	3.04	2.69
OsR498G0612358500.01	10.023	20.423	1.0195169	2.73E-183	8.79E-184	10.51	10	9.56	20.35	21.8	19.12
OsR498G0612359200.01	0.373	0.886	1.2584414	8.90E-16	1.17E-15	0.46	0.3	0.36	0.99	0.76	0.91
OsR498G0612364400.01	0.2	1.856	3.2223375	2.80E-35	2.60E-35	0.21	0.12	0.27	1.92	1.62	2.03
OsR498G0612365000.01	6.37	2.103	-1.600284	3.12E-45	2.53E-45	6.76	6.02	6.33	2.33	2.03	1.95
OsR498G0612369500.01	10.05	4.226	-1.251866	9.31E-22	1.08E-21	11.48	9.61	9.06	3.48	4.25	4.95
OsR498G0612375300.01	0.476	1.066	1.1394692	1.73E-08	2.82E-08	0.39	0.5	0.54	1.02	1.12	1.06
OsR498G0612378900.01	3.893	10.403	1.4023797	1.32E-41	1.13E-41	3.88	3.83	3.97	10.47	10.29	10.45
OsR498G0612383600.01	369.326	45.72	-2.966186	0	0	356.59	381.68	369.71	45.97	43.51	47.68
OsR498G0612388400.01	23.656	88.986	1.9111536	0	0	25.65	22.91	22.41	90.87	88.08	88.01
OsR498G0612404800.01	3.986	11.27	1.4912278	1.36E-58	9.52E-59	4.68	3.61	3.67	10.15	11.22	12.44
OsR498G0612408600.01	0.08	19.263	7.9039643	3.59E-256	8.64E-257	0	0.1	0.14	21.34	18.06	18.39
OsR498G0612412100.01	0.79	0.066	-3.610553	1.80E-06	3.19E-06	0.89	0.89	0.59	0	0	0.2
OsR498G0612412700.01	8.19	0.92	-3.281405	1.82E-168	6.27E-169	9.4	7.4	7.77	0.74	1.07	0.95
OsR498G0612416100.01	5.206	2.296	-1.185068	3.02E-10	4.63E-10	6.2	5.71	3.71	1.6	2.65	2.64
OsR498G0612416400.01	36.916	12.1	-1.924058	4.15E-228	1.11E-228	35.7	36.07	38.98	11.09	11.26	13.95
OsR498G0612423400.01	1.43	3.316	1.1175119	1.33E-10	2.02E-10	0.78	1.94	1.57	2.38	3.44	4.13
OsR498G0612426800.01	5.843	28.77	2.3173863	0	0	6.33	5.12	6.08	28.88	28.55	28.88
OsR498G0612442200.01	0.716	6.083	3.1006737	4.40E-184	1.41E-184	0.73	0.69	0.73	6.39	5.69	6.17
OsR498G0612448600.01	3.706	7.643	1.0479911	2.53E-23	2.86E-23	3.65	3.58	3.89	7.02	7.28	8.63
OsR498G0612449600.01	5.92	11.61	1.0372904	1.52E-49	1.18E-49	5.5	5.97	6.29	11.44	12.28	11.11
OsR498G0612466400.01	0.44	1.103	1.3239944	2.80E-06	4.99E-06	0.47	0.51	0.34	0.96	1.41	0.94
OsR498G0612478700.01	0.183	2.583	3.7911027	7.59E-33	7.29E-33	0.1	0.2	0.25	2.23	2.82	2.7
OsR498G0612482600.01	2.68	0.836	-1.676129	1.38E-13	1.92E-13	2.22	2.93	2.89	0.88	0.84	0.79
OsR498G0612483400.01	5.896	2.883	-1.073879	4.15E-32	4.03E-32	6	5.71	5.98	2.77	3.25	2.63
OsR498G0612488000.01	82.363	305.68	1.9246985	0	0	85.39	82.59	79.11	306.12	313.69	297.22
OsR498G0612489500.01	7.25	18.996	1.3978862	1.45E-140	5.73E-141	7.48	6.66	7.61	18.05	20.33	18.61
OsR498G0612492500.01	1.306	0.39	-1.822057	2.87E-20	3.42E-20	1.31	1.19	1.42	0.38	0.47	0.32
OsR498G0612499400.01	3.49	0.486	-2.844043	2.37E-43	1.97E-43	3.15	3.67	3.65	0.51	0.43	0.52
OsR498G0612504300.01	0.426	1.156	1.4250714	0.0002539	0.0005045	0.26	0.51	0.51	1.17	0.92	1.38
OsR498G0612507400.01	45.96	13.146	-1.844192	0	0	46.37	43.86	47.65	11.82	12.95	14.67
OsR498G0612515100.01	3.083	6.916	1.1675822	2.93E-20	3.50E-20	3.29	2.96	3	7.27	6.7	6.78
OsR498G0612516300.01	5.126	10.773	1.0801194	2.02E-32	1.95E-32	5.42	4.97	4.99	10.54	10.32	11.46
OsR498G0612517200.01	11.083	2.753	-2.061375	3.70E-100	1.86E-100	10.18	11.44	11.63	3.05	2.74	2.47
OsR498G0612522500.01	0.063	0.24	2.144335	9.15E-05	0.0001771	0.07	0.06	0.06	0.3	0.14	0.28
OsR498G0612527300.01	18.263	38.63	1.1268295	2.17E-87	1.20E-87	19.15	17.87	17.77	37.52	37.31	41.06
OsR498G0612533500.01	52.396	222	2.0844411	0	0	53.01	52.25	51.93	224.88	226.3	214.82
OsR498G0612536100.01	0.523	2.326	2.144335	6.35E-09	1.02E-08	0.45	0.45	0.67	2.09	2.3	2.59
OsR498G0612548600.01	0.513	0.09	-2.485022	7.91E-08	1.32E-07	0.74	0.45	0.35	0.14	0.1	0.03
OsR498G0612550400.01	7.363	3.763	-1.016312	1.21E-19	1.47E-19	6.56	9.04	6.49	3.78	3.52	3.99
OsR498G0612553000.01	4.53	1.34	-1.740511	9.75E-72	6.05E-72	4.4	4.39	4.8	1.4	1.41	1.21
OsR498G0612558900.01	60.14	214.83	1.8358479	0	0	63.06	61.43	55.93	222.5	209.92	212.08
OsR498G0612559600.01	0.366	3.546	3.2671917	4.92E-28	5.12E-28	0.37	0.38	0.35	3.52	3.98	3.14
OsR498G0612562800.01	3.09	10.076	1.7023304	2.67E-28	2.77E-28	2.62	3.18	3.47	9.91	9.3	11.02
OsR498G0612571700.01	0.136	0.89	2.6932282	1.72E-13	2.39E-13	0.1	0.1	0.21	0.77	1	0.9
OsR498G0612586600.01	1.9	13.45	2.8215354	3.95E-53	2.94E-53	1.99	0.99	2.72	14.91	12.92	12.52
OsR498G0612589000.01	186.586	458.65	1.3032355	0	0	181.07	189.45	189.24	453.75	458.27	463.94
OsR498G0612590400.01	0.55	1.323	1.2598122	0.0009737	0.0020275	0.41	0.62	0.62	0.91	1	2.06
OsR498G0612592000.01	0.353	0	-4.347518	0.0007014	0.0014405	0.11	0.42	0.53	0	0	0
OsR498G0612594200.01	1.323	0.503	-1.387047	2.01E-13	2.80E-13	1.32	1.13	1.52	0.52	0.54	0.45
OsR498G0612595200.01	7.89	23.57	1.5793215	2.22E-273	5.07E-274	7.69	7.81	8.17	24.38	23.28	23.05
OsR498G0612620400.01	0.303	0	-4.485022	0.0003787	0.0007614	0.25	0.33	0.33	0	0	0
OsR498G0612630800.01	15.863	2.063	-3.022307	6.49E-76	3.90E-76	14.92	16.15	16.52	2.14	1.4	2.65
OsR498G0612640400.01	1.54	5.463	1.8306745	4.03E-50	3.09E-50	1.54	1.5	1.58	5.67	5.35	5.37
OsR498G0612640900.01	0.65	1.51	1.2215028	9.51E-13	1.35E-12	0.75	0.6	0.6	1.44	1.76	1.33
OsR498G0612644700.01	4.113	11.556	1.493978	2.95E-138	1.18E-138	4.53	4	3.81	11.48	12.21	10.98
OsR498G0612647600.01	1.97	0.533	-1.885412	2.30E-11	3.41E-11	2.12	1.74	2.05	0.53	0.59	0.48
OsR498G0612651500.01	0.58	1.403	1.3117221	1.65E-12	2.37E-12	0.35	0.55	0.84	1.04	1.83	1.34
OsR498G0612656700.01	0.186	0.626	1.7507144	7.79E-11	1.17E-10	0.14	0	0.42	0.19	0.93	0.76
OsR498G0612663800.01	0.303	0.813	1.409347	5.05E-11	7.56E-11	0.36	0.3	0.25	0.83	0.89	0.72
OsR498G0612664400.01	1.716	0.526	-1.7084	3.37E-12	4.86E-12	1.73	1.63	1.79	0.49	0.71	0.38
OsR498G0612670100.01	0.246	0.023	-3.417907	1.09E-05	2.00E-05	0.25	0.14	0.35	0.03	0	0.04
OsR498G0612674500.01	26.43	70.773	1.0991311	2.90E-271	6.65E-272	26.49	27.71	25.09	68.71	70.31	73.3
OsR498G0612676500.01	0.08	10.746	7.0618728	7.07E-93	3.74E-93	0.08	0.16	0	10.6	11.57	10.07
OsR498G0612676800.01	0.306	1.253	2.0358105	5.46E-12	7.92E-12	0.32	0.24	0.36	1.24	1.2	1.32
OsR498G0612679600.01	0.17	0	-5.273518	3.18E-06	5.68E-06	0.16	0.19	0.16	0	0	0
OsR498G0612680000.01	6.49	1.686	-2.085273	3.57E-41	3.06E-41	6.18	6.61	6.68	1.96	1.49	1.61
OsR498G0612680600.01	1.553	0.123	-3.634399	5.96E-26	6.43E-26	1.71	1.47	1.48	0.04	0.18	0.15
OsR498G0612699600.01	6.71	1.706	-1.973123	8.30E-30	8.37E-30	6.22	7.43	6.48	1.75	1.88	1.49

OsR498G0612702400.01	0.063	0.633	3.2963381	2.59E-05	4.84E-05	0	0.19	0	0.66	0.47	0.77
OsR498G0612707100.01	1.07	0.296	-1.848712	1.54E-10	2.34E-10	1.02	0.9	1.29	0.2	0.34	0.35
OsR498G0612707400.01	0.36	1.02	1.5097164	8.60E-06	1.58E-05	0.27	0.38	0.43	1.1	0.99	0.97
OsR498G0612711400.01	4.63	0.803	-2.531943	5.06E-72	3.13E-72	3.97	4.67	5.25	0.92	0.65	0.84
OsR498G0612713500.01	3.756	1.576	-1.253618	5.55E-11	8.32E-11	3.31	4.14	3.82	1.54	1.41	1.78
OsR498G0612714500.01	0.206	0.066	-1.66302	0.0005716	0.0011637	0.2	0.29	0.13	0.02	0.09	0.09
OsR498G0612718200.01	1.006	4.22	2.0284048	1.71E-53	1.26E-53	1.18	1.04	0.8	4.31	4.24	4.11
OsR498G0612718500.01	0.133	0.743	2.4769103	5.78E-11	8.66E-11	0.1	0.13	0.17	0.65	0.81	0.77
OsR498G0612721800.01	0.563	0.226	-1.333601	0.0001258	0.0002455	0.89	0.36	0.44	0.27	0.2	0.21
OsR498G0612721900.01	0.193	0	-4.977924	2.52E-05	4.71E-05	0	0.58	0	0	0	0
OsR498G0612722200.01	0.89	0.413	-1.094303	1.93E-05	3.58E-05	1	0.77	0.9	0.3	0.6	0.34
OsR498G0612725200.01	0.02	1.093	5.6609105	7.08E-26	7.63E-26	0	0.06	0	1.04	1.01	1.23
OsR498G0612726400.01	0.01	0.733	6.2781907	3.81E-20	4.56E-20	0	0.03	0	0.58	0.71	0.91
OsR498G0612727000.01	2.836	9.556	1.7623125	2.00E-40	1.73E-40	2.91	2.3	3.3	9	9.68	9.99
OsR498G0612730000.01	22.966	55.913	1.2828941	2.91E-188	9.15E-189	22.44	22.52	23.94	58.1	53.69	55.95
OsR498G0612730500.01	4.47	14.16	1.7954403	1.31E-187	4.15E-188	4.23	4.53	4.65	13.9	14.53	14.05
OsR498G0612733300.01	3.143	10.56	1.7489316	1.92E-82	1.10E-82	3.44	2.99	3	9.93	11.26	10.49
OsR498G0612736800.01	0.183	4.626	4.6748497	3.91E-25	4.27E-25	0.27	0.14	0.14	5.21	3.2	5.47
OsR498G0612737600.01	0.033	0.53	5.331962	2.54E-11	3.76E-11	0.1	0	0	0.77	0.46	0.36
OsR498G0612741700.01	0.063	0.236	1.8591174	0.000621	0.0012687	0.04	0.04	0.11	0.17	0.24	0.3
OsR498G0612743000.01	29.026	5.893	-2.302856	1.17E-165	4.10E-166	29.26	27.68	30.14	5.99	4.93	6.76
OsR498G0612743600.01	0.686	0.313	-1.167609	8.92E-05	0.0001724	0.66	1.09	0.31	0.29	0.29	0.36
OsR498G0612746500.01	8.22	27.51	1.7444839	0	0	8.6	8.23	7.83	27	28.24	27.29
OsR498G0612749100.01	13.783	49.866	1.8540072	1.50E-214	4.21E-215	13.5	15.5	12.35	49.11	50.46	50.03
OsR498G0612749400.01	5.443	11.586	1.0846484	1.49E-53	1.10E-53	5.16	6.3	4.87	12.59	10.68	11.49
OsR498G0612751100.01	16.506	7.186	-1.196788	1.22E-100	6.11E-101	17.02	16.12	16.38	6.92	7.01	7.63
OsR498G0612752200.01	0.93	1.89	1.1657086	5.61E-09	8.97E-09	0.99	1.12	0.68	1.59	2.1	1.98
OsR498G0612756500.01	3.22	9.47	1.5567985	1.81E-165	6.35E-166	3.47	3.41	2.78	9.47	9.01	9.93
OsR498G0612764700.01	0.726	0.146	-2.495075	9.16E-12	1.34E-11	0.9	0.58	0.7	0.14	0.11	0.19
OsR498G0612766200.01	4.98	11.53	1.2032961	7.61E-35	7.10E-35	4.97	4.66	5.31	11.22	10.94	12.43
OsR498G0612767700.01	0.023	0.106	3.5593725	0.0005724	0.0011661	0	0.07	0	0	0.13	0.19
OsR498G0612773400.01	4.35	19.21	2.1416829	3.36E-247	8.41E-248	4.19	4.76	4.1	20.09	18.46	19.08
OsR498G0612777500.01	43.343	19.593	-1.144036	7.62E-156	2.79E-156	44.51	43.3	42.22	19.98	18.06	20.74
OsR498G0612784300.01	90.27	34.863	-1.373994	1.43E-197	4.33E-198	93.89	90.66	86.26	33.19	34.9	36.5
OsR498G0612791500.01	10.703	28.163	1.4060268	3.39E-150	1.27E-150	11.9	10.14	10.07	28.28	29.07	27.14
OsR498G0612792100.01	1.353	5.126	1.9246569	1.04E-46	8.26E-47	1.52	1.3	1.24	5.09	4.6	5.69
OsR498G0612792500.01	0.12	0.773	2.6524819	2.12E-06	3.77E-06	0.22	0.07	0.07	0.5	0.57	1.25
OsR498G0612792700.01	0.04	0.196	2.3894475	0.0009846	0.0020515	0.03	0	0.09	0.07	0.39	0.13
OsR498G0612796100.01	10.113	33.816	1.7339078	2.32E-95	1.21E-95	9.73	11.32	9.29	33.64	33.32	34.49
OsR498G0612796300.01	0.02	0.296	4.0188041	1.22E-08	1.97E-08	0	0.03	0.03	0.35	0.21	0.33
OsR498G0612796800.01	7.843	18.23	1.2166454	1.48E-114	6.77E-115	8.03	8.16	7.34	18.3	18.9	17.49
OsR498G0612801900.01	0.356	1.78	2.3249072	3.71E-08	6.12E-08	0.43	0.43	0.21	1.35	1.86	2.13
OsR498G0612802600.01	5.85	2.16	-1.440289	7.34E-34	6.95E-34	5.86	5.87	5.82	1.67	2.25	2.56
OsR498G0612804200.01	0	0.456	5.0073924	1.78E-05	3.31E-05	0	0	0	0.79	0.2	0.38
OsR498G0612805400.01	10.536	42.196	1.999974	0	0	9.9	11.15	10.56	40.31	43.74	42.54
OsR498G0612810100.01	0.333	0.033	-3.485022	2.44E-12	3.50E-12	0.31	0.42	0.27	0.04	0.04	0.02
OsR498G0612810500.01	0.013	0.19	3.97441	5.14E-05	9.78E-05	0.04	0	0	0.14	0.21	0.22
OsR498G0612812000.01	2.163	8.44	1.9514731	8.59E-127	3.67E-127	2.1	2.1	2.29	8.44	8.43	8.45
OsR498G0612814100.01	16.56	40.926	1.2922874	2.01E-178	6.63E-179	16.78	15.82	17.08	39.44	40.12	43.22
OsR498G0612829300.01	0	0.553	4.6748497	0.0001298	0.0002536	0	0	0	0.61	0.24	0.81
OsR498G0612830100.01	1.096	2.916	1.4147527	2.38E-14	3.25E-14	1.2	0.99	1.1	3.18	2.88	2.69
OsR498G0612830700.01	119.943	228.48	1.0232258	0	0	117.77	123.81	118.25	225.95	230.19	229.29
OsR498G0612834500.01	30.84	70.94	1.3103199	2.60E-307	5.38E-308	29.69	30.18	32.65	69.84	72.88	70.1
OsR498G0612834800.01	1.906	12.21	2.8503352	0	0	1.97	1.83	1.92	11.63	12.67	12.33
OsR498G0612836400.01	0.386	0.84	1.1212513	1.26E-08	2.04E-08	0.35	0.48	0.33	0.84	0.9	0.78
OsR498G0612836900.01	0.603	4.563	2.7557697	1.75E-18	2.16E-18	0.91	0.79	0.11	5.69	3.33	4.67
OsR498G0612846600.01	0.95	3.12	1.7074711	4.32E-12	6.25E-12	0.79	1.03	1.03	3.57	3.44	2.35
OsR498G0612846900.01	0.15	1.97	3.7251814	2.04E-36	1.86E-36	0.21	0.14	0.1	1.94	1.73	2.24
OsR498G0612847100.01	0.116	1.193	3.33698	6.86E-15	9.22E-15	0.1	0.1	0.15	1.28	1.03	1.27
OsR498G0612852100.01	0.683	4.256	2.6389957	4.39E-09	7.00E-09	1.25	0.38	0.42	3.76	4.49	4.52
OsR498G0612852500.01	0.496	1.983	1.8102922	1.49E-10	2.27E-10	0.18	0.52	0.79	1.2	2.3	2.45
OsR498G0612853600.01	0.086	0.253	1.5695004	9.29E-06	1.70E-05	0.06	0.12	0.08	0.21	0.25	0.3
OsR498G0612856100.01	1.68	5.806	1.7917599	3.20E-105	1.55E-105	1.71	1.8	1.53	5.7	6.56	5.16
OsR498G0612857000.01	0.196	0.54	1.5442656	1.18E-08	1.91E-08	0.17	0.29	0.13	0.55	0.68	0.39
OsR498G0612863100.01	92.12	266.53	1.5218656	0	0	95.06	90.17	91.13	266.16	263.64	269.79
OsR498G0612863300.01	0.106	1.53	3.8157122	4.04E-19	4.94E-19	0.05	0.11	0.16	1.59	1.37	1.63
OsR498G0612863700.01	0.023	0.353	3.8813006	7.15E-08	1.19E-07	0	0	0.07	0.42	0.35	0.29
OsR498G0612866900.01	0.696	2.57	1.8851426	8.29E-11	1.25E-10	0.84	0.5	0.75	2.36	2.51	2.84
OsR498G0612870300.01	5.383	0.526	-3.404635	2.83E-103	1.39E-103	5.55	5.28	5.32	0.4	0.61	0.57

OsR498G0612871300.01	0.16	1.986	3.6427885	1.91E-36	1.74E-36	0.27	0.14	0.07	1.86	1.95	2.15
OsR498G0612873900.01	103.31	38.19	-1.4209	0	0	104.41	103.28	102.24	38.42	39.29	36.86
OsR498G0612875900.01	1.403	0.363	-1.954249	3.82E-12	5.52E-12	1.51	0.95	1.75	0.49	0.22	0.38
OsR498G0612883100.01	4.876	2.233	-1.171096	2.78E-43	2.32E-43	5.25	5	4.38	2.46	2.2	2.04
OsR498G0612885700.01	0.66	4.486	2.7665304	2.51E-73	1.54E-73	1.05	0.49	0.44	3.83	5.17	4.46
OsR498G0612887100.01	8.35	22.326	1.4189515	1.22E-127	5.18E-128	8.61	8.99	7.45	21.23	23.95	21.8
OsR498G0612889800.01	59.333	12.19	-2.275162	0	0	60.35	60.71	56.94	11.74	11.96	12.87
OsR498G0612890200.01	3.28	8.966	1.4467147	1.03E-27	1.08E-27	3.34	3.94	2.56	9.19	9.52	8.19
OsR498G0612891500.01	24.626	83.386	1.7594115	0	0	24.65	24.58	24.65	88.29	81.86	80.01
OsR498G0612893400.01	5.2	15.976	1.6188941	1.09E-187	3.43E-188	5.26	5.24	5.1	16.65	15.67	15.61
OsR498G0612893900.01	6.64	15.126	1.1825885	8.01E-36	7.39E-36	6.87	7.05	6	14.69	13.68	17.01
OsR498G0612894200.01	18.173	8.026	-1.14185	7.09E-73	4.37E-73	18.71	18.24	17.57	7.96	7.88	8.24
OsR498G0612896000.01	0.066	0.343	2.3894475	0.0009846	0.0020515	0.13	0.07	0	0.57	0.13	0.33
OsR498G0612897600.01	0.37	0.893	1.2619865	3.64E-06	6.53E-06	0.37	0.37	0.37	0.64	1.12	0.92
OsR498G0612897800.01	11.723	38.2	1.7034848	3.70E-229	9.86E-230	11.68	12.13	11.36	36.85	38.2	39.55
OsR498G0612900400.01	1.65	0.133	-3.627626	4.03E-52	3.03E-52	1.68	1.56	1.71	0.11	0.11	0.18
OsR498G0612901100.01	3.106	0.626	-2.309798	5.25E-59	3.67E-59	3.33	2.72	3.27	0.78	0.5	0.6
OsR498G0612903300.01	65.353	13.626	-2.253248	0	0	67.97	62.48	65.61	12.72	13.28	14.88
OsR498G0612905200.01	0.896	0.37	-1.230313	0.0006493	0.0013291	1.42	1.01	0.26	0.49	0.17	0.45
OsR498G0612914100.01	9.67	0.623	-3.968402	9.63E-124	4.17E-124	10.76	7.57	10.68	0.57	0.37	0.93
OsR498G0612915100.01	3.24	8.313	1.342949	2.10E-42	1.77E-42	3.25	3.16	3.31	8.7	7.71	8.53
OsR498G0612915600.01	0.016	0.283	3.8813006	9.36E-05	0.0001812	0	0.05	0	0.37	0.1	0.38
OsR498G0612915900.01	0.046	0.446	3.2223375	4.64E-05	8.81E-05	0	0.14	0	0.56	0.49	0.29
OsR498G0612917100.01	631.326	126.52	-2.312775	0	0	632.29	616.61	645.08	124.37	123.97	131.22
OsR498G0612920700.01	2.866	0.913	-1.651666	7.26E-16	9.53E-16	3.31	2.71	2.58	0.7	0.92	1.12
OsR498G0612921000.01	1.43	0.92	-1.254781	2.28E-07	3.89E-07	1.44	1.08	1.77	0.68	1.71	0.37
OsR498G0612921100.01	0.166	0.08	-1.020519	0.0007339	0.0015099	0.19	0.11	0.2	0.12	0.09	0.03
OsR498G0612924300.01	0.1	0.366	1.9004094	2.10E-05	3.91E-05	0.09	0.03	0.18	0.2	0.4	0.5
OsR498G0612925000.01	0.03	0.17	2.5593725	0.0003356	0.0006725	0.03	0.03	0.03	0.14	0.28	0.09
OsR498G0612925400.01	0.086	0.35	1.999945	1.12E-07	1.89E-07	0.02	0.15	0.09	0.35	0.38	0.32
OsR498G0612928200.01	1378.61	537.74	-1.358364	0	0	1351.6	1388.4	1395.8	535.99	541.91	535.32
OsR498G0612928900.01	0.486	2.483	2.3521664	1.40E-98	7.11E-99	0.52	0.41	0.53	2.51	2.66	2.28
OsR498G0612933300.01	578.633	264.27	-1.131968	0	0	574.13	596.38	565.39	266.24	263.83	262.73
OsR498G0612934200.01	0.063	0.973	3.987234	3.31E-25	3.61E-25	0.03	0.03	0.13	0.72	0.94	1.26
OsR498G0612936500.01	0.946	2.633	1.4719096	1.77E-24	1.95E-24	1.15	0.82	0.87	2.62	2.99	2.29
OsR498G0612937000.01	0.99	0.353	-1.496481	6.36E-13	8.98E-13	1.02	0.97	0.98	0.31	0.33	0.42
OsR498G0612937400.01	4.136	46.34	3.5821296	0	0	3.69	4.14	4.58	46.89	45.14	46.99
OsR498G0612958800.01	4.08	8.493	1.0848009	2.73E-43	2.27E-43	4.02	3.93	4.29	9.23	8.35	7.9
OsR498G0612961000.01	3.273	0.27	-3.610553	7.21E-35	6.73E-35	3.35	3.55	2.92	0.11	0.11	0.59
OsR498G0612964900.01	2.196	8.033	1.8935421	2.91E-50	2.23E-50	2.37	2.18	2.04	8.27	7.65	8.18
OsR498G0612967000.01	0.486	0.983	1.4149825	1.84E-11	2.71E-11	0.87	0.32	0.27	0.96	0.89	1.1
OsR498G0612970100.01	6.183	31.38	2.3367801	9.09E-261	2.15E-261	6.14	6.68	5.73	29.36	32.45	32.33
OsR498G0612970500.01	3.856	7.603	1.050637	5.06E-81	2.94E-81	3.65	3.62	4.3	8.51	7.49	6.81
OsR498G0612972800.01	2.12	16.11	2.9118026	9.32E-193	2.88E-193	2.22	1.87	2.27	15.11	18.01	15.21
OsR498G0612975600.01	11.393	4.9	-1.226834	1.88E-86	1.04E-86	11.63	11.71	10.84	4.86	5.28	4.56
OsR498G0612982500.01	0.04	0.306	2.6472124	2.58E-07	4.41E-07	0.04	0.04	0.04	0.3	0.3	0.32
OsR498G0612991600.01	0.58	0.126	-2.1916	1.97E-05	3.66E-05	0.81	0.5	0.43	0.18	0.07	0.13
OsR498G0613005000.01	0.07	0.446	2.637375	0.0001939	0.0003825	0.07	0.07	0.07	0.63	0.49	0.22
OsR498G0613020200.01	3.043	0.916	-1.568732	6.82E-25	7.48E-25	3.24	3.01	2.88	1.04	0.91	0.8
OsR498G0613021200.01	41.576	19.01	-1.129852	2.40E-178	7.91E-179	41.09	42.04	41.6	18.08	19.35	19.6
OsR498G0613029200.01	0.306	6.43	4.3894475	2.41E-99	1.21E-99	0.31	0.26	0.35	6.66	6.17	6.46
OsR498G0613032100.01	481.68	186.73	-1.370161	0	0	486.12	465.9	493.02	189.09	178.67	192.43
OsR498G0613035300.01	0.093	0.733	3.2474285	4.29E-12	6.21E-12	0.05	0.12	0.11	0.47	0.96	0.77
OsR498G0613037000.01	1.16	2.98	1.3322874	6.87E-18	8.60E-18	1.23	1.3	0.95	2.83	3.05	3.06
OsR498G0613038900.01	7.443	0	-10.2616	3.08E-97	1.58E-97	7.82	7.59	6.92	0	0	0
OsR498G0613039900.01	0.013	0.19	3.8813006	9.36E-05	0.0001812	0.04	0	0	0.11	0.04	0.42
OsR498G0613040900.01	0.116	1.053	3.2070707	2.59E-18	3.21E-18	0.07	0.14	0.14	1.03	1.03	1.1
OsR498G0613042200.01	2.17	0.316	-2.772824	5.48E-31	5.43E-31	2.09	2.31	2.11	0.17	0.47	0.31
OsR498G0613046600.01	16.133	42	1.3852514	1.93E-177	6.39E-178	17.76	15.53	15.11	40.39	40.6	45.01
OsR498G0613049800.01	12.993	30.09	1.1971054	3.86E-157	1.40E-157	13.09	13.21	12.68	30.15	29.8	30.32
OsR498G0613052100.01	0.513	3.55	2.8029295	6.87E-97	3.53E-97	0.59	0.49	0.46	3.53	3.74	3.38
OsR498G0613052600.01	21.186	69.06	1.7130677	0	0	19.82	22.63	21.11	68.62	71.7	66.86
OsR498G0613053500.01	1.386	5.546	1.989802	1.14E-127	4.82E-128	1.42	1.51	1.23	5.36	6.06	5.22
OsR498G0613055600.01	201.356	455.9	1.1817234	0	0	200.5	204.23	199.34	461.75	450.95	454.99
OsR498G0613061000.01	1.43	7.516	2.3992152	7.02E-98	3.58E-98	1.51	1.4	1.38	7.03	7.35	8.17
OsR498G0613064000.01	8.206	3.12	-1.397559	6.67E-41	5.74E-41	8.24	8.39	7.99	3.08	2.99	3.29
OsR498G0613065200.01	0.376	4.67	3.7669679	5.01E-79	2.94E-79	0.25	0.47	0.41	4.69	4.24	5.08
OsR498G0613066600.01	0.25	1.146	2.2011824	2.32E-24	2.57E-24	0.29	0.25	0.21	1.13	1.27	1.04
OsR498G0613069100.01	0.32	2.17	2.7658233	1.75E-56	1.26E-56	0.31	0.26	0.39	2.1	2.3	2.11

OsR498G0613070000.01	0.87	2.156	1.3165969	4.14E-16	5.40E-16	1.16	0.57	0.88	2.18	2.14	2.15
OsR498G0613070300.01	9.76	28.356	1.536913	1.08E-88	5.91E-89	9.4	9.97	9.91	28.65	26.73	29.69
OsR498G0613074200.01	5.016	33.26	2.7614051	0	0	4.93	5.1	5.02	31.69	33.28	34.81
OsR498G0613074600.01	0.676	1.683	1.4162475	3.52E-12	5.09E-12	0.9	0.5	0.63	1.17	1.51	2.37
OsR498G0613074700.01	1.093	5.59	2.3555001	2.71E-30	2.72E-30	1.38	0.68	1.22	5.57	5.63	5.57
OsR498G0613076100.01	0.49	3.41	2.803298	1.16E-105	5.62E-106	0.32	0.69	0.46	3.53	3.44	3.26
OsR498G0613076600.01	0.736	2.056	1.4585318	1.61E-16	2.08E-16	0.79	0.71	0.71	2.24	1.8	2.13
OsR498G0613076800.01	4.386	1.106	-1.986419	1.27E-24	1.39E-24	4.49	4.69	3.98	1.06	1.05	1.21
OsR498G0613081000.01	7.76	16.153	1.0577312	1.81E-45	1.46E-45	7.89	7.05	8.34	16.69	16.17	15.6
OsR498G0613081200.01	20.076	42.32	1.0746677	2.34E-72	1.44E-72	19.91	18.81	21.51	41.94	41.37	43.65
OsR498G0613088000.01	27.316	8.883	-1.621441	4.27E-222	1.16E-222	26.04	28.63	27.28	8.55	8.77	9.33
OsR498G0613100100.01	28.32	12.123	-1.226527	3.65E-141	1.44E-141	26.82	28.81	29.33	12.16	12.26	11.95
OsR498G0613102200.01	0.77	3.42	2.1993763	2.39E-67	1.54E-67	0.77	0.68	0.86	3.54	3.49	3.23
OsR498G0613103300.01	11.296	37.096	1.7233751	1.14E-127	4.82E-128	11.24	12.03	10.62	35.92	38.69	36.68
OsR498G0613106800.01	0.04	0.843	4.3851764	3.80E-05	7.17E-05	0	0	0.12	0.77	0.85	0.91
OsR498G0613110600.01	38.103	95.856	1.354907	0	0	39.3	32.46	42.55	88.77	86.98	111.82
OsR498G0613111800.01	29.013	86.893	1.576133	0	0	30.74	27.37	28.93	88.65	84.56	87.47
OsR498G0613112000.01	1.383	5.21	1.9020591	1.10E-52	8.24E-53	1.47	1.4	1.28	5.02	5.93	4.68
OsR498G0613112200.01	44.72	109.41	1.291515	0	0	45	45.31	43.85	110.66	110.98	106.59
OsR498G0613117600.01	7.863	17.783	1.2098328	2.56E-103	1.26E-103	8.41	7.32	7.86	18.61	17.1	17.64
OsR498G0613118900.01	10.04	26.133	1.3799417	2.26E-183	7.30E-184	9.5	10.64	9.98	26.59	25.93	25.88
OsR498G0613119600.01	0.003	1.48	9.7372072	2.32E-19	2.82E-19	0.01	0	0	4.44	0	0
OsR498G0613121600.01	3.736	1.796	-1.054541	2.88E-20	3.44E-20	4	3.92	3.29	1.76	1.8	1.83
OsR498G0613123000.01	1.473	4.82	1.7148065	3.22E-37	2.90E-37	1.44	1.25	1.73	4.94	4.82	4.7
OsR498G0613124300.01	17	44.496	1.3967894	1.06E-246	2.66E-247	18.18	16.2	16.62	45.85	44.75	42.89
OsR498G0613126200.01	185.693	90.39	-1.036073	0	0	191.91	182.82	182.35	94.97	91.46	84.74
OsR498G0613133500.01	8.406	38.353	2.1881233	3.48E-213	9.87E-214	7.87	8.57	8.78	36.69	36.85	41.52
OsR498G0613134900.01	1.03	0.233	-2.163094	6.89E-07	1.20E-06	0.92	1.47	0.7	0.21	0.14	0.35
OsR498G0613137300.01	0.333	2.193	2.7134956	2.31E-69	1.46E-69	0.4	0.31	0.29	2.05	1.93	2.6
OsR498G0613138900.01	0.303	1.69	2.4524573	3.54E-38	3.15E-38	0.43	0.31	0.17	1.77	1.94	1.36
OsR498G0613145400.01	625.146	1631.8	1.3841401	0	0	630.57	633.62	611.25	1657	1660.6	1577.8
OsR498G0613145600.01	7.793	18.606	1.2945852	4.17E-161	1.49E-161	7.2	7.96	8.22	18.56	18.95	18.31
OsR498G0613146300.01	11.633	28.84	1.3172481	3.75E-284	8.30E-285	11.97	11.43	11.5	27.96	29.99	28.57
OsR498G0613146600.01	0.24	1.343	2.4807626	1.06E-18	1.31E-18	0.13	0.33	0.26	1.7	1.34	0.99
OsR498G0613148900.01	2.496	15.3	2.6155155	2.90E-170	9.91E-171	2.43	2.68	2.38	14.6	15.69	15.61
OsR498G0613149500.01	1.813	4.573	1.3620925	4.20E-32	4.09E-32	1.67	2.08	1.69	3.61	4.94	5.17
OsR498G0613151300.01	1.403	4.89	1.8318559	7.35E-64	4.91E-64	1.2	1.62	1.39	4.88	5.3	4.49
OsR498G0613151800.01	0.04	0.303	2.97441	0.0002639	0.0005255	0.06	0.06	0	0.28	0.28	0.35
OsR498G0613153600.01	0.256	3.446	3.7292975	1.55E-44	1.26E-44	0.29	0.29	0.19	3.8	3.12	3.42
OsR498G0613155600.01	0.73	15.633	4.4197205	5.26E-135	2.14E-135	0.39	0.78	1.02	14.97	16.81	15.12
OsR498G0613155900.01	0.38	2.116	2.4753941	1.78E-44	1.46E-44	0.56	0.33	0.25	2.48	2.25	1.62
OsR498G0613156100.01	0.026	0.246	3.6748497	7.57E-07	1.32E-06	0	0.04	0.04	0.23	0.28	0.23
OsR498G0613160200.01	5.373	10.906	1.0208455	1.39E-18	1.71E-18	5.75	4.73	5.64	11.09	9.73	11.9
OsR498G0613163800.01	3.243	31.34	2.1340495	0	0	3.35	3.18	3.2	29.14	33.27	31.61
OsR498G0613164400.01	12.683	179.3	3.8729128	0	0	12.31	12.41	13.33	183.85	183.03	171.01
OsR498G0613166500.01	1.303	26.823	4.3616564	0	0	1.28	1.27	1.36	27.04	27.61	25.82
OsR498G0613167600.01	7.736	33.346	2.0954984	0	0	7.71	7.6	7.9	33.01	32.65	34.38
OsR498G0613169700.01	0.413	0.06	-2.762556	8.51E-05	0.0001642	0.25	0.43	0.56	0.06	0.12	0
OsR498G0613173700.01	10.75	22.176	1.0221026	6.47E-115	2.95E-115	11.3	10.43	10.52	21.76	22.94	21.83
OsR498G0613174000.01	0.61	2.076	1.7629059	2.01E-08	3.29E-08	0.67	0.65	0.51	2.26	2.07	1.9
OsR498G0613178200.01	13.26	51.616	1.9752823	0	0	14.1	12.73	12.95	53.44	51.87	49.54
OsR498G0613183300.01	0.163	0.426	1.4006747	0.0002214	0.000438	0.14	0.28	0.07	0.31	0.56	0.41
OsR498G0613194600.01	5.846	2.56	-1.032576	6.20E-10	9.62E-10	5.16	6.36	6.02	2.59	2.83	2.26
OsR498G0613197700.01	10.85	22.38	1.0436111	2.60E-92	1.38E-92	11.35	10.72	10.48	21.89	22.28	22.97
OsR498G0613199600.01	5.423	2.046	-1.333122	1.92E-22	2.20E-22	5.85	5.27	5.15	2.15	2.16	1.83
OsR498G0613199900.01	0.26	0.07	-1.855665	9.25E-05	0.0001791	0.32	0.24	0.22	0.07	0.07	0.07
OsR498G0613200200.01	0	0.26	5.0618728	1.23E-05	2.27E-05	0	0	0	0.09	0.5	0.19
OsR498G0613200400.01	48.763	211.69	2.1207672	0	0	47.06	52.15	47.08	212.4	208.36	214.32
OsR498G0613200500.01	0.266	0.04	-3.115314	3.49E-06	6.25E-06	0.36	0.26	0.18	0.08	0	0.04
OsR498G0613200700.01	17.37	112.99	2.7004491	0	0	18.13	17.18	16.8	114.87	110.05	114.04
OsR498G0613202600.01	2.376	5.05	1.087304	1.31E-09	2.05E-09	2.75	1.82	2.56	4.77	5.32	5.06
OsR498G0613203000.01	0.126	0.373	1.5593725	8.67E-05	0.0001674	0.16	0.11	0.11	0.4	0.34	0.38
OsR498G0613203200.01	13.406	4.96	-1.443561	5.85E-117	2.63E-117	14.1	13.15	12.97	5.08	4.68	5.12
OsR498G0613204600.01	1.123	0.223	-2.33039	9.27E-08	1.55E-07	1.01	1	1.36	0.07	0.38	0.22
OsR498G0613207900.01	0.136	0.466	1.8762296	1.54E-08	2.50E-08	0.19	0.02	0.2	0.46	0.58	0.36
OsR498G0613208200.01	15.306	4.573	-1.740709	1.96E-51	1.48E-51	15.07	16.03	14.82	4.88	4.16	4.68
OsR498G0613211000.01	2.556	1.226	-1.088431	6.85E-15	9.21E-15	2.63	2.4	2.64	1.25	1.32	1.11
OsR498G0613211900.01	5.983	1.006	-2.585586	1.81E-59	1.26E-59	7.01	4.76	6.18	1.01	0.77	1.24
OsR498G0613213400.01	3.996	0.27	-3.903334	2.32E-32	2.25E-32	4.5	4.15	3.34	0.24	0.08	0.49

OsR498G0613214400.01	4.8	14.34	1.6113245	3.28E-117	1.47E-117	4.79	4.76	4.85	13.6	14.42	15
OsR498G0613215600.01	2.753	14.853	2.4526588	1.35E-273	3.08E-274	2.89	2.64	2.73	14.33	16.52	13.71
OsR498G0613217300.01	0.453	1.756	1.9412431	5.04E-06	9.11E-06	0.37	0.37	0.62	1.82	1.21	2.24
OsR498G0613225200.01	0.633	1.616	1.2167708	1.44E-11	2.12E-11	0.68	0.64	0.58	1.58	1.54	1.73
OsR498G0613225600.01	0.19	0.61	1.7292975	1.13E-08	1.82E-08	0.25	0.18	0.14	0.55	0.83	0.45
OsR498G0613226300.01	8.453	26.01	1.6147697	3.05E-216	8.50E-217	8.19	8.92	8.25	23.73	26.83	27.47
OsR498G0613226800.01	0.626	3.133	3.0776088	2.53E-87	1.40E-87	0.76	0.65	0.47	2.94	3.19	3.27
OsR498G0613227500.01	0.326	0	-5.72603	5.93E-08	9.87E-08	0.23	0.45	0.3	0	0	0
OsR498G0613228100.01	3.98	10.68	1.4189388	1.17E-61	7.93E-62	3.79	4.28	3.87	9.52	11.93	10.59
OsR498G0613229600.01	3.633	37.646	3.3726376	0	0	4.06	3.46	3.38	38.77	37.38	36.79
OsR498G0613231900.01	2.336	0.45	-2.39124	1.27E-10	1.93E-10	2.63	1.46	2.92	0.41	0.31	0.63
OsR498G0613233900.01	2.07	0.793	-1.385486	1.88E-11	2.78E-11	2.23	2.53	1.45	0.59	0.86	0.93
OsR498G0613235300.01	0.006	0.11	3.8813006	9.36E-05	0.0001812	0.02	0	0	0.07	0.15	0.11
OsR498G0613240400.01	0.356	0	-5.485022	5.65E-07	9.78E-07	0.3	0.37	0.4	0	0	0
OsR498G0613242700.01	0.013	1.103	6.331962	8.22E-21	9.70E-21	0	0	0.04	1.12	1.04	1.15
OsR498G0613243200.01	0.23	3.15	3.759187	7.99E-65	5.28E-65	0.21	0.15	0.33	3.29	3.42	2.74
OsR498G0613244800.01	2.97	8.326	1.4773124	2.50E-42	2.10E-42	2.79	3.2	2.92	7.56	8.93	8.49
OsR498G0613245900.01	3.306	8.046	1.2765579	1.27E-64	8.44E-65	3.29	3.08	3.55	7.38	8.08	8.68
OsR498G0613246200.01	15.95	47.11	1.5631605	0	0	15.92	17.11	14.82	48.2	47.91	45.22
OsR498G0613246700.01	4.65	0.456	-3.275685	2.62E-62	1.77E-62	4.62	4.83	4.5	0.87	0.28	0.22
OsR498G0613247300.01	4.86	1.663	-1.368862	1.59E-23	1.79E-23	5.24	4.81	4.53	2.04	1.41	1.54
OsR498G0613247600.01	0.333	3.976	3.4795284	2.81E-200	8.43E-201	0.4	0.22	0.38	4.31	3.95	3.67
OsR498G0613248700.01	5.14	1.036	-2.357972	1.09E-37	9.77E-38	6.14	5.16	4.12	1.21	0.92	0.98
OsR498G0613250900.01	0.91	4.363	2.3232422	6.70E-56	4.84E-56	0.76	1.01	0.96	4.28	3.75	5.06
OsR498G0613252100.01	0.793	0.2	-1.964189	0.0004779	0.0009679	1.14	0.52	0.72	0.3	0.2	0.1
OsR498G0613253600.01	0.936	2.693	1.6016833	2.09E-41	1.79E-41	0.85	1.11	0.85	3.15	2.64	2.29
OsR498G0613255600.01	5.76	26.41	2.1944584	2.72E-97	1.39E-97	6.11	5.98	5.19	23.31	27.13	28.79
OsR498G0613266700.01	0.09	0.013	-2.832945	0.0006839	0.0014027	0.15	0.06	0.06	0	0.04	0
OsR498G0613267000.01	3.956	1.163	-1.752685	2.24E-40	1.94E-40	4.15	4.15	3.57	1.27	1.2	1.02
OsR498G0613270100.01	1.553	3.913	1.3312979	2.94E-33	2.81E-33	1.66	1.51	1.49	4.11	3.37	4.26
OsR498G0613272200.01	2.066	4.783	1.3155411	8.61E-60	5.96E-60	1.75	2.24	2.21	4.12	4.77	5.46
OsR498G0613272300.01	5.073	15.09	1.5186194	4.28E-78	2.53E-78	5.43	5.17	4.62	15.24	14.73	15.3
OsR498G0613274200.01	0.126	0.443	1.8084	3.27E-09	5.18E-09	0.13	0.12	0.13	0.5	0.45	0.38
OsR498G0613275100.01	10.523	1.266	-3.02559	2.17E-177	7.18E-178	11.8	10.49	9.28	1.26	1.24	1.3
OsR498G0613276500.01	0.143	0.626	2.1348746	2.31E-10	3.53E-10	0.1	0.11	0.22	0.54	0.69	0.65
OsR498G0613278600.01	11.09	1.433	-2.955678	7.36E-162	2.62E-162	10.29	11.93	11.05	1.47	1.07	1.76
OsR498G0613279000.01	0	0.243	5.5593725	2.32E-07	3.95E-07	0	0	0	0.15	0.24	0.34
OsR498G0613281300.01	13.916	33.343	1.2573514	3.73E-240	9.59E-241	13.86	14.32	13.57	33.17	34.09	32.77
OsR498G0613282300.01	16.09	56.826	1.8188766	1.54E-306	3.20E-307	15.52	17.33	15.42	55.52	54.23	60.73
OsR498G0613282500.01	0.186	0.836	2.144335	8.17E-06	1.49E-05	0.14	0.21	0.21	0.97	0.48	1.06
OsR498G0613282700.01	42.026	104.4	1.3106552	0	0	42.44	40.49	43.15	105.01	102.06	106.13
OsR498G0613283500.01	0.466	0	-5.756773	4.35E-08	7.19E-08	0	1.4	0	0	0	0
OsR498G0613284700.01	0.673	1.433	1.0898872	1.81E-09	2.85E-09	0.41	0.77	0.84	1.53	1.47	1.3
OsR498G0613285800.01	30.243	102.18	1.8747666	0	0	30.01	29.67	31.05	103.62	103.23	99.7
OsR498G0613286600.01	0.036	1.253	5.075948	1.65E-25	1.79E-25	0.04	0	0.07	1.27	1.37	1.12
OsR498G0613287200.01	0.346	1.933	2.4827512	4.02E-24	4.46E-24	0.22	0.53	0.29	1.81	1.63	2.36
OsR498G0613287700.01	46.876	140.97	1.5926046	0	0	46.82	48.16	45.65	143.13	137.12	142.67
OsR498G0613287900.01	1.793	4.003	1.1527472	9.99E-08	1.68E-07	1.9	1.41	2.07	3.87	2.85	5.29
OsR498G0613289800.01	0.156	0.833	2.4524573	5.93E-07	1.03E-06	0.2	0.2	0.07	0.76	0.76	0.98
OsR498G0613292600.01	13.393	32.013	1.2568872	5.73E-241	1.47E-241	14.36	13.4	12.42	31.26	33.48	31.3
OsR498G0613293100.01	0.616	1.67	1.4356371	7.13E-15	9.60E-15	0.46	0.78	0.61	1.65	1.4	1.96
OsR498G0613296000.01	2.016	4.43	1.1364942	2.15E-18	2.66E-18	1.71	2.24	2.1	4.21	5.12	3.96
OsR498G0613296500.01	1.816	0.333	-2.496896	1.03E-18	1.27E-18	1.68	1.58	2.19	0.36	0.27	0.37
OsR498G0613298600.01	20.15	40.753	1.0087987	2.05E-140	8.07E-141	20.45	19.75	20.25	41.02	40.02	41.22
OsR498G0613300200.01	15.01	47.533	1.6023642	1.39E-131	5.80E-132	16.54	12.93	15.56	50.25	43.22	49.13
OsR498G0613301300.01	29.716	59.493	1.0186624	1.17E-217	3.25E-218	29.68	28.52	30.95	58.66	58.88	60.94
OsR498G0613302000.01	1.64	5.423	1.6753796	1.13E-15	1.49E-15	1.52	1.58	1.82	5.72	4.73	5.82
OsR498G0613302400.01	1.13	3.656	1.7435902	4.07E-29	4.16E-29	1.47	1.06	0.86	3.82	3.95	3.2
OsR498G0613303900.01	32.553	86.85	1.2762717	0	0	35.4	31.36	30.9	88.65	83.53	88.37
OsR498G0613305900.01	22.256	57.226	1.3508482	0	0	22.06	22.69	22.02	57.7	58.02	55.96
OsR498G0613306500.01	13.223	2.353	-2.492605	5.08E-107	2.43E-107	13.33	13.46	12.88	2.23	2.12	2.71
OsR498G0613308900.01	5.133	23.33	2.18353	5.49E-127	2.34E-127	5.75	5.31	4.34	23.31	23.15	23.53
OsR498G0613309200.01	0.553	2.206	2.0391802	1.28E-23	1.43E-23	0.84	0.35	0.47	2.63	2.23	1.76
OsR498G0613311100.01	6.17	20.646	1.7443252	3.07E-183	9.89E-184	6.14	6.39	5.98	20.7	19.66	21.58
OsR498G0613313900.01	0.85	3.6	2.04657	7.65E-71	4.78E-71	0.91	0.72	0.92	3.59	3.79	3.42
OsR498G0613317400.01	0.14	0.86	2.5916215	7.52E-30	7.59E-30	0.15	0.16	0.11	0.98	1.32	0.28
OsR498G0613322200.01	0.836	2.85	1.7816497	8.82E-52	6.65E-52	0.69	1.11	0.71	2.98	2.81	2.76
OsR498G0613325600.01	249.783	82.41	-1.601713	0	0	257.21	241.74	250.4	81.11	75.37	90.75
OsR498G0613327500.01	0.706	5.32	3.0139383	3.02E-75	1.82E-75	0.64	0.88	0.6	5.43	4.53	6

OsR498G0613331200.01	339.856	1477.3	2.1203893	0	0	343.92	337.79	337.86	1483.3	1473	1475.5
OsR498G0613332100.01	0.133	0.883	2.7292975	6.89E-10	1.07E-09	0.1	0.1	0.2	0.87	0.58	1.2
OsR498G0613332400.01	18.286	146.48	3.0001052	0	0	17.46	19.61	17.79	141.74	142.59	155.1
OsR498G0613341500.01	3.34	12.02	1.8469575	2.41E-234	6.32E-235	3.28	3.45	3.29	11.89	12.37	11.8
OsR498G0613342600.01	7.823	1.633	-2.259451	1.47E-208	4.25E-209	8.2	8.04	7.23	1.46	1.7	1.74
OsR498G0613346000.01	0.8	1.986	1.3154469	0.0007909	0.0016307	0.48	1.76	0.16	1.72	2.65	1.59
OsR498G0613346900.01	6.486	2.02	-1.639956	1.21E-33	1.15E-33	5.82	6.74	6.9	2.03	2.14	1.89
OsR498G0613347800.01	0.99	3.996	2.0058882	1.34E-59	9.32E-60	0.99	0.89	1.09	3.87	3.99	4.13
OsR498G0613351200.01	41.396	82.843	1.0009947	2.19E-210	6.27E-211	38.31	42.69	43.19	82.86	81.37	84.3
OsR498G0713354100.01	0.066	0.416	2.7113756	1.01E-07	1.69E-07	0	0.1	0.1	0.34	0.4	0.51
OsR498G0713359500.01	16.053	2.956	-2.441177	0	0	14.7	16.14	17.32	2.9	3.01	2.96
OsR498G0713364100.01	0.483	2.15	2.1595214	1.14E-23	1.28E-23	0.45	0.38	0.62	1.94	2	2.51
OsR498G0713366700.01	0	0.446	6.0188041	1.75E-09	2.75E-09	0	0	0	0.48	0.32	0.54
OsR498G0713370800.01	0.61	2.07	1.7301193	3.42E-39	3.01E-39	0.69	0.52	0.62	1.97	2.06	2.18
OsR498G0713371200.01	3.596	16.49	2.1987717	2.40E-201	7.14E-202	4	3.24	3.55	16.25	17.79	15.43
OsR498G0713372900.01	1.256	0.27	-2.216274	1.94E-14	2.64E-14	1.41	1.25	1.11	0.21	0.24	0.36
OsR498G0713373800.01	5.83	11.966	1.0383778	2.79E-33	2.66E-33	6.79	5.31	5.39	12.12	11.41	12.37
OsR498G0713375300.01	2.423	6.44	1.4207189	1.63E-45	1.32E-45	2.23	2.58	2.46	6.8	6.23	6.29
OsR498G0713375700.01	0.616	10.83	3.8933858	9.29E-103	4.58E-103	0.62	0.81	0.42	10.99	10.9	10.6
OsR498G0713376000.01	0.263	1.59	2.565826	9.81E-20	1.18E-19	0.31	0.22	0.26	1.63	1.64	1.5
OsR498G0713378400.01	88.336	28.893	-1.625411	0	0	87.02	91.95	86.04	27.68	28.91	30.09
OsR498G0713381900.01	2.513	5.583	1.149042	4.16E-44	3.42E-44	2.96	2.13	2.45	5.71	5.17	5.87
OsR498G0713382300.01	1.223	0.223	-2.471001	7.93E-37	7.20E-37	1.15	1.25	1.27	0.25	0.25	0.17
OsR498G0713382600.01	4.38	19.94	2.245557	1.51E-155	5.53E-156	4.5	3.7	4.94	19.23	19.67	20.92
OsR498G0713385000.01	13.603	30.143	1.1610903	2.77E-45	2.25E-45	15.38	12.72	12.71	30.42	27.01	33
OsR498G0713385200.01	5.363	15.97	1.5738809	1.19E-162	4.22E-163	5.64	5.39	5.06	15.74	15.54	16.63
OsR498G0713385500.01	13.936	39.5	1.4223089	3.56E-135	1.45E-135	13.77	15.14	12.9	39.68	38.98	39.84
OsR498G0713390000.01	0.726	3.963	2.4425588	7.83E-35	7.31E-35	0.54	0.82	0.82	3.5	4.06	4.33
OsR498G0713392100.01	0.66	0.176	-1.832945	1.10E-07	1.85E-07	0.76	0.56	0.66	0.26	0.15	0.12
OsR498G0713392900.01	0.456	0.06	-2.951589	4.72E-08	7.83E-08	0.36	0.54	0.47	0.04	0.07	0.07
OsR498G0713394500.01	0.69	0.153	-2.247982	2.33E-10	3.56E-10	0.74	0.69	0.64	0.29	0.06	0.11
OsR498G0713396500.01	29.836	69.603	1.2931131	1.44E-174	4.82E-175	27.86	30.78	30.87	71.36	64.45	73
OsR498G0713397700.01	2.266	4.863	1.0992164	1.56E-09	2.45E-09	2.47	1.96	2.37	4.24	4.94	5.41
OsR498G0713398700.01	0.273	0.6	1.1336086	0.0001026	0.000199	0.39	0.27	0.16	0.56	0.69	0.55
OsR498G0713401800.01	0.933	2.166	1.2088752	9.23E-10	1.44E-09	0.91	0.78	1.11	2.05	1.8	2.65
OsR498G0713405700.01	0.286	0	-5.549152	3.20E-07	5.48E-07	0.19	0.33	0.34	0	0	0
OsR498G0713407400.01	0.146	0.03	-2.195515	0.0008316	0.0017206	0.1	0.1	0.24	0.07	0	0.02
OsR498G0713410800.01	2.983	0.976	-1.61868	7.33E-16	9.63E-16	2.98	2.7	3.27	1.13	0.64	1.16
OsR498G0713412200.01	0.123	0.696	2.2641436	1.62E-15	2.14E-15	0.06	0.2	0.11	0.65	0.75	0.69
OsR498G0713412600.01	0.976	3.103	1.6618025	8.16E-16	1.07E-15	0.87	1.08	0.98	2.6	3.28	3.43
OsR498G0713413600.01	0.16	0.443	1.85634	0.0002018	0.0003984	0.18	0.26	0.04	0.42	0.48	0.43
OsR498G0713414200.01	0.356	2.41	2.7586813	1.26E-20	1.49E-20	0.3	0.41	0.36	2.78	2.37	2.08
OsR498G0713421700.01	36.37	12.19	-1.58181	6.16E-225	1.67E-225	37.12	35.97	36.02	14.48	10.96	11.13
OsR498G0713422500.01	0.46	0.01	-5.72603	3.53E-14	4.83E-14	0.43	0.53	0.42	0.03	0	0
OsR498G0713423300.01	0.133	4.656	5.144335	5.08E-35	4.72E-35	0.2	0.2	0	5.02	5.39	3.56
OsR498G0713425600.01	0.213	0.893	2.0829344	2.52E-09	3.98E-09	0.24	0.2	0.2	0.89	1	0.79
OsR498G0713426900.01	0.036	0.366	3.331962	3.21E-09	5.09E-09	0.05	0.03	0.03	0.38	0.4	0.32
OsR498G0713428100.01	1.506	9.87	3.1013347	2.35E-175	7.84E-176	1.61	1.41	1.5	10.18	10.75	8.68
OsR498G0713428300.01	0	0.74	5.766224	3.03E-08	4.97E-08	0	0	0	0.55	0.63	1.04
OsR498G0713428800.01	0.216	0.956	2.1353249	8.37E-22	9.70E-22	0.31	0.15	0.19	0.91	0.93	1.03
OsR498G0713428900.01	10.053	41.623	2.0361862	3.06E-143	1.19E-143	9.02	10.13	11.01	42.66	41.82	40.39
OsR498G0713429300.01	2.046	57.573	4.7693854	0	0	2.21	1.86	2.07	58.55	59.94	54.23
OsR498G0713429500.01	3.096	13.923	2.1694259	2.67E-251	6.55E-252	3.19	3.15	2.95	14.42	13.64	13.71
OsR498G0713429700.01	0.376	1.276	1.8042951	4.03E-20	4.82E-20	0.32	0.43	0.38	1.52	1.28	1.03
OsR498G0713430000.01	0.506	2.656	2.5810344	3.53E-24	3.92E-24	0.87	0.42	0.23	2.79	2.52	2.66
OsR498G0713432400.01	0.68	1.506	1.0349515	0.0001581	0.0003103	0.61	0.79	0.64	1.82	1.45	1.25
OsR498G0713432900.01	0	0.766	6.3667274	1.62E-11	2.39E-11	0	0	0	0.76	0.81	0.73
OsR498G0713434800.01	7.006	18.673	1.4165858	2.47E-173	8.32E-174	6.79	6.94	7.29	18.11	19.43	18.48
OsR498G0713435600.01	0.266	1.05	1.97441	8.24E-06	1.51E-05	0.16	0.16	0.48	0.7	1.09	1.36
OsR498G0713436800.01	8.8	70.32	2.9967778	0	0	9.34	8.28	8.78	66.03	71.79	73.14
OsR498G0713437900.01	4.5	14.083	1.6478205	7.57E-198	2.29E-198	4.17	4.63	4.7	14.37	15.05	12.83
OsR498G0713441000.01	5.45	11.683	1.1007872	2.07E-28	2.14E-28	5.51	5.98	4.86	11.32	11.76	11.97
OsR498G0713453500.01	0.266	1.9	2.9831671	4.23E-15	5.66E-15	0.41	0.15	0.24	1.96	1.78	1.96
OsR498G0713461300.01	1.3	4.173	1.457619	8.19E-71	5.12E-71	1.15	1.54	1.21	4.25	4.17	4.1
OsR498G0713461500.01	2.34	17.986	2.9884937	0	0	2.4	1.99	2.63	18.29	17.53	18.14
OsR498G0713461800.01	1.553	41.68	4.7449568	0	0	1.64	1.65	1.37	43.03	43.48	38.53
OsR498G0713462000.01	0.25	2.436	3.3101109	1.33E-77	7.87E-78	0.24	0.19	0.32	2.79	2.46	2.06
OsR498G0713462300.01	12.55	5.643	-1.151708	3.60E-75	2.17E-75	11.98	12.94	12.73	6.45	5.51	4.97
OsR498G0713462600.01	21.21	188.5	3.1519346	0	0	21.64	21.53	20.46	192.2	188.67	184.64

OsR498G0713463000.01	32.453	259.86	3.0010042	0	0	31.15	34.2	32.01	264.11	261.35	254.12
OsR498G0713463100.01	13.586	31.513	1.2129419	2.17E-205	6.36E-206	13.89	14.17	12.7	30.46	31.45	32.63
OsR498G0713463600.01	32.663	90.543	1.4696593	1.45E-201	4.32E-202	31.41	34.45	32.13	91.01	90.99	89.63
OsR498G0713463800.01	0.03	0.356	3.4338416	6.61E-08	1.10E-07	0.03	0.03	0.03	0.29	0.35	0.43
OsR498G0713464000.01	0.593	0.156	-1.89066	9.52E-07	1.67E-06	0.56	0.66	0.56	0.1	0.2	0.17
OsR498G0713464300.01	3.556	32.366	3.6587388	0	0	3.27	3.37	4.03	31.63	33.73	31.74
OsR498G0713465300.01	1.543	0.716	-1.115227	3.47E-09	5.51E-09	1.71	1.47	1.45	0.71	0.6	0.84
OsR498G0713465800.01	0.203	0.046	-2.195515	0.0008316	0.0017206	0.17	0.17	0.27	0.07	0.07	0
OsR498G0713470200.01	0.076	0	-4.832945	6.10E-05	0.0001167	0.05	0.11	0.07	0	0	0
OsR498G0713471500.01	3.863	10.003	1.3746505	1.49E-119	6.60E-120	3.78	3.77	4.04	9.55	10.67	9.79
OsR498G0713472300.01	0.153	1.566	3.3287595	1.48E-42	1.24E-42	0.17	0.12	0.17	1.39	1.66	1.65
OsR498G0713472500.01	5.936	2.936	-1.013849	5.54E-23	6.27E-23	6.5	5.34	5.97	3.4	2.31	3.1
OsR498G0713472800.01	20.53	82.503	1.8526868	0	0	20.16	22.68	18.75	83.15	84.49	79.87
OsR498G0713483300.01	0.086	3.74	5.3922625	1.47E-41	1.26E-41	0	0.13	0.13	3.97	3.11	4.14
OsR498G0713483800.01	0.59	2.303	2.0898872	2.36E-10	3.61E-10	0.67	0.52	0.58	1.93	2.69	2.29
OsR498G0713487700.01	63.483	131.03	1.020683	0	0	65.81	62.66	61.98	135.47	129.58	128.03
OsR498G0713488200.01	8.063	25.663	1.6671286	9.61E-66	6.29E-66	7.34	7.59	9.26	25.17	21.99	29.83
OsR498G0713488800.01	1.386	0.366	-1.918675	0.000277	0.0005521	1.12	2.24	0.8	0.47	0.31	0.32
OsR498G0713492600.01	0.076	1.723	4.5157832	8.04E-38	7.18E-38	0.03	0.1	0.1	1.82	1.52	1.83
OsR498G0713493200.01	2326.62	360.41	-2.684129	0	0	2331.3	2235.4	2413.2	353.16	350.49	377.58
OsR498G0713495500.01	0.16	0.636	1.97441	0.0003981	0.0008013	0.16	0.16	0.16	0.63	0.71	0.57
OsR498G0713496700.01	0.413	0	-5.347518	1.78E-06	3.15E-06	0.37	0.37	0.5	0	0	0
OsR498G0713496900.01	31.956	7.6	-2.088231	2.69E-266	6.25E-267	34.69	29.83	31.35	7.61	7.69	7.5
OsR498G0713499200.01	3.23	12.433	1.8495692	8.10E-67	5.25E-67	3.02	3.45	3.22	12.2	11.8	13.3
OsR498G0713510500.01	214.703	9.143	-4.584969	0	0	207.1	221.44	215.57	10.41	8	9.02
OsR498G0713512200.01	12.983	31.08	1.2489625	2.62E-141	1.03E-141	12.83	11.93	14.19	31.63	30.51	31.1
OsR498G0713515500.01	6716.25	1147.6	-2.551678	0	0	6544.6	6915.4	6688.8	1123.7	1123.9	1195.3
OsR498G0713516800.01	10.446	56.72	2.4391694	1.34E-252	3.27E-253	11.44	9.54	10.36	59.87	51.21	59.08
OsR498G0713518200.01	0.326	1.346	1.97441	6.00E-19	7.35E-19	0.27	0.29	0.42	1.72	1.05	1.27
OsR498G0713519100.01	16.31	85.716	2.3909011	0	0	16.65	16.29	15.99	81.34	84.43	91.38
OsR498G0713526100.01	0.046	0.63	3.7817649	3.45E-10	5.29E-10	0	0.09	0.05	0.54	0.71	0.64
OsR498G0713527800.01	0.59	3.17	2.5472996	2.23E-18	2.77E-18	0.87	0.49	0.41	3.02	3.56	2.93
OsR498G0713530800.01	0.046	0.443	3.1731898	2.24E-19	2.72E-19	0.01	0.12	0.01	0.49	0.46	0.38
OsR498G0713538700.01	0.086	1.073	3.6637091	4.91E-27	5.21E-27	0.08	0.15	0.03	1.19	1.01	1.02
OsR498G0713539500.01	7.426	15.92	1.0963501	4.62E-19	5.65E-19	6.86	8.01	7.41	14.28	14.8	18.68
OsR498G0713539800.01	0.043	0.12	1.4746934	0.0008597	0.0017809	0.01	0.08	0.04	0.14	0.13	0.09
OsR498G0713544300.01	1.73	0.296	-2.524818	1.06E-18	1.31E-18	1.96	1.24	1.99	0.48	0.29	0.12
OsR498G0713550300.01	0.816	1.913	1.0398829	3.58E-06	6.42E-06	0.26	1.18	1.01	1.56	2.32	1.86
OsR498G0713550700.01	2.48	10.78	2.1031436	1.18E-74	7.17E-75	2.61	2.13	2.7	10.94	10.45	10.95
OsR498G0713551200.01	0.123	0.713	2.4979719	7.77E-05	0.0001495	0	0.09	0.28	0.55	0.83	0.76
OsR498G0713554800.01	0.416	0.143	-1.511017	0.0001066	0.0002069	0.36	0.56	0.33	0.17	0.14	0.12
OsR498G0713560100.01	75.68	175.71	1.2116421	0	0	71.33	77.78	77.93	174.61	176.39	176.12
OsR498G0713561300.01	0.956	0.376	-1.417907	8.03E-06	1.47E-05	0.66	1.31	0.9	0.52	0.33	0.28
OsR498G0713564800.01	1.35	0	-5.02559	1.85E-05	3.43E-05	1.77	1.78	0.5	0	0	0
OsR498G0713565000.01	191.653	10.686	-4.205904	0	0	191.52	189.69	193.75	10.82	10.76	10.48
OsR498G0713568400.01	199.516	492.03	1.2998085	0	0	201.39	190.26	206.9	467.97	460.81	547.3
OsR498G0713568600.01	3.45	15.53	2.1692261	4.71E-89	2.57E-89	3.88	3.23	3.24	14.29	15.38	16.92
OsR498G0713569400.01	3.73	7.626	1.0294813	2.34E-22	2.68E-22	4.2	3.42	3.57	7.48	7.19	8.21
OsR498G0713570600.01	0.42	0.906	1.1015219	2.78E-05	5.21E-05	0.6	0.36	0.3	0.88	0.81	1.03
OsR498G0713571400.01	0.71	0.066	-3.485022	2.44E-12	3.50E-12	0.86	0.81	0.46	0.08	0.08	0.04
OsR498G0713572600.01	0.82	4.886	2.6194934	5.79E-44	4.77E-44	0.75	0.83	0.88	4.14	5.41	5.11
OsR498G0713572900.01	3.116	8.13	1.3828176	4.71E-86	2.63E-86	3.31	3.14	2.9	7.86	8.77	7.76
OsR498G0713574400.01	0.03	0.67	4.4338416	2.75E-16	3.58E-16	0.06	0	0.03	0.63	0.57	0.81
OsR498G0713575100.01	0.17	0.813	2.2510039	7.82E-09	1.26E-08	0.07	0.07	0.37	1.03	0.88	0.53
OsR498G0713575700.01	2.776	6.93	1.3014269	1.71E-41	1.46E-41	2.55	2.84	2.94	7.12	6.64	7.03
OsR498G0713577800.01	4.786	10.743	1.2048891	1.01E-122	4.40E-123	5.06	4.49	4.81	11.01	11.04	10.18
OsR498G0713578300.01	20.826	71.846	1.8088034	0	0	20.67	20.47	21.34	71.15	74.67	69.72
OsR498G0713583100.01	0.923	3.05	1.7206534	2.09E-19	2.54E-19	0.81	1.02	0.94	3.05	2.78	3.32
OsR498G0713584300.01	1.413	0.5	-1.491154	1.03E-20	1.22E-20	1.37	1.38	1.49	0.35	0.66	0.49
OsR498G0713585100.01	14.963	31.663	1.0789107	2.92E-129	1.23E-129	14.69	15.37	14.83	32.71	30.85	31.43
OsR498G0713585900.01	1.253	6.913	2.4966072	3.58E-44	2.94E-44	0.89	1.55	1.32	7.41	6.8	6.53
OsR498G0713586200.01	36.12	214.53	2.3293653	0	0	36.1	35.2	37.06	214.04	219.33	210.22
OsR498G0713590100.01	0.713	0.103	-2.780478	2.02E-14	2.75E-14	0.98	0.55	0.61	0.08	0.1	0.13
OsR498G0713590300.01	0.276	0.8	1.2963381	4.78E-05	9.08E-05	0.21	0.31	0.31	0.58	0.96	0.86
OsR498G0713590600.01	83.14	214.4	1.3613499	0	0	80.32	87.04	82.06	212.23	204.87	226.1
OsR498G0713594700.01	0.24	4.006	4.0618728	7.95E-34	7.53E-34	0.32	0.32	0.08	4.13	3.57	4.32
OsR498G0713594900.01	0.063	0.56	3.144335	2.13E-06	3.77E-06	0.13	0.06	0	0.43	0.8	0.45
OsR498G0713596000.01	12.44	34.243	1.4258581	1.67E-166	5.80E-167	11.47	12.81	13.04	33.68	35.77	33.28
OsR498G0713596800.01	8.323	30.456	1.87307	0	0	9.13	8.05	7.79	30.65	30.56	30.16

OsR498G0713598700.01	0.386	1.34	1.8762296	1.54E-08	2.50E-08	0.25	0.62	0.29	1.46	1.38	1.18
OsR498G0713599800.01	6.34	14.2	1.1422074	2.39E-40	2.07E-40	6.95	6.14	5.93	13.8	14.66	14.14
OsR498G0713600500.01	0.57	8.253	3.8482934	1.86E-167	6.43E-168	0.52	0.55	0.64	7.6	8.7	8.46
OsR498G0713603300.01	4.15	0.616	-2.748056	1.98E-47	1.57E-47	4.38	4.05	4.02	0.74	0.61	0.5
OsR498G0713605700.01	15.343	82.003	2.4194486	0	0	14.79	14.87	16.37	80.78	82.39	82.84
OsR498G0713607100.01	0.436	1.156	1.3223333	6.43E-05	0.0001231	0.51	0.43	0.37	0.95	1.36	1.16
OsR498G0713609400.01	2.656	0.196	-3.745482	4.79E-47	3.81E-47	2.29	2.3	3.38	0.21	0.24	0.14
OsR498G0713610300.01	0.04	0.36	3.0618728	0.0001482	0.0002902	0.06	0	0.06	0.25	0.25	0.58
OsR498G0713610900.01	0.543	5.14	3.2374444	3.08E-44	2.53E-44	0.26	0.52	0.85	5.04	5.34	5.04
OsR498G0713611400.01	3.33	8.336	1.3233051	2.58E-17	3.28E-17	3.12	3.2	3.67	9.39	7.79	7.83
OsR498G0713611800.01	2.35	6.67	1.4454529	1.49E-34	1.40E-34	2.4	1.91	2.74	6.23	6.77	7.01
OsR498G0713615700.01	0.29	1.186	2.0188041	3.40E-12	4.90E-12	0.29	0.18	0.4	1.07	1.06	1.43
OsR498G0713618700.01	0.21	0.016	-3.72603	5.42E-07	9.38E-07	0.22	0.17	0.24	0	0	0.05
OsR498G0713620600.01	451.026	217.69	-1.051944	0	0	454.33	445	453.75	216.98	213.89	222.19
OsR498G0713621100.01	0.55	1.893	1.7992019	2.11E-42	1.77E-42	0.5	0.49	0.66	2.05	1.85	1.78
OsR498G0713621600.01	15.54	41.57	1.4231367	1.15E-160	4.14E-161	14.52	15.74	16.36	41.22	40.16	43.33
OsR498G0713625500.01	1.046	2.1	1.0372977	5.24E-10	8.10E-10	1.03	1.22	0.89	1.98	2.24	2.08
OsR498G0713625700.01	168.346	1465.2	3.1203455	0	0	173.7	160.07	171.27	1490.9	1391.7	1512.9
OsR498G0713625900.01	597.063	5991.4	3.3253176	0	0	621.21	568.62	601.36	5983.6	5656.3	6334.4
OsR498G0713626200.01	2.686	17.62	2.685342	1.77E-265	4.12E-266	2.43	2.58	3.05	17.95	16.58	18.33
OsR498G0713626300.01	0.99	2.316	1.2556961	2.45E-20	2.92E-20	0.88	1.04	1.05	2.22	2.49	2.24
OsR498G0713627100.01	6.56	16.173	1.3017848	2.56E-120	1.13E-120	6.58	6.36	6.74	16.21	16.2	16.11
OsR498G0713627600.01	7.833	40.583	2.3503504	0	0	7.77	8.18	7.55	40.3	39.96	41.49
OsR498G0713631700.01	5.956	29.086	2.2853317	3.57E-305	7.44E-306	5.72	6.2	5.95	29.21	28.58	29.47
OsR498G0713631900.01	1.4	4.31	1.6329015	1.06E-39	9.26E-40	1.28	1.48	1.44	4.16	4.5	4.27
OsR498G0713633700.01	0.613	1.783	1.6208908	1.31E-19	1.59E-19	0.65	0.49	0.7	1.68	1.88	1.79
OsR498G0713634000.01	21.206	47.313	1.156632	8.35E-83	4.78E-83	21.7	19.78	22.14	47.6	46.67	47.67
OsR498G0713634400.01	6.99	16.406	1.2291121	8.96E-62	6.08E-62	7.07	7.79	6.11	17.19	16.38	15.65
OsR498G0713641800.01	6.76	31.316	2.2096264	1.01E-147	3.82E-148	6.56	6.51	7.21	30.7	30.57	32.68
OsR498G0713643500.01	97.263	37.053	-1.374956	4.34E-305	9.05E-306	95.19	100.74	95.86	35.81	36.44	38.91
OsR498G0713645200.01	11.56	5.453	-1.086831	3.55E-52	2.66E-52	11.33	12.11	11.24	5.05	6.39	4.92
OsR498G0713653700.01	6.466	20.91	1.7154245	2.14E-210	6.15E-211	6.14	6.68	6.58	21.38	21.24	20.11
OsR498G0713658300.01	182.18	58.326	-1.582193	0	0	188.09	179.36	179.09	56.36	58.73	59.89
OsR498G0713661400.01	8.463	33.996	2.0781642	1.38E-245	3.49E-246	8.11	9.07	8.21	33.24	33.19	35.56
OsR498G0713662100.01	1.876	0.463	-2.020501	4.48E-32	4.36E-32	1.62	1.84	2.17	0.43	0.5	0.46
OsR498G0713666500.01	2.123	6.053	1.5110094	8.24E-39	7.29E-39	1.96	2.23	2.18	5.89	6.37	5.9
OsR498G0713669500.01	28.006	192.67	2.7823403	0	0	27.73	28.77	27.52	190.96	199.38	187.67
OsR498G0713673300.01	0.486	6.79	3.7954398	2.61E-171	8.84E-172	0.39	0.51	0.56	5.71	6.44	8.22
OsR498G0713674800.01	7.99	21.403	1.4131517	7.93E-59	5.56E-59	8.47	7.85	7.65	23.54	20.07	20.6
OsR498G0713681500.01	1.766	3.973	1.125574	8.32E-19	1.02E-18	2.01	0.95	2.34	3.9	4.41	3.61
OsR498G0713682400.01	2.803	1.026	-1.004216	2.45E-09	3.87E-09	2.71	3.24	2.46	1.12	0.78	1.18
OsR498G0713684800.01	2.453	17.236	2.8144456	0	0	2.2	2.99	2.17	18.58	16.69	16.44
OsR498G0713686000.01	64.083	136.31	1.0829456	0	0	62.19	66.4	63.66	132.83	136.92	139.18
OsR498G0713690400.01	136.67	53.833	-1.398576	0	0	141.26	130.63	138.12	52.85	55.1	53.55
OsR498G0713690500.01	0.32	0.066	-2.195515	6.44E-05	0.0001235	0.32	0.21	0.43	0.03	0.17	0
OsR498G0713693700.01	1.16	0.21	-2.460976	5.00E-18	6.23E-18	1.12	1.22	1.14	0.2	0.14	0.29
OsR498G0713696600.01	0.813	2.37	1.5391946	7.19E-07	1.25E-06	0.51	0.81	1.12	3.09	1.88	2.14
OsR498G0713699500.01	2.716	5.626	1.0531531	2.30E-36	2.10E-36	2.92	2.55	2.68	5.69	6.15	5.04
OsR498G0713702000.01	1.04	3.886	1.8977887	1.91E-12	2.74E-12	1.62	0.86	0.64	3.89	3.35	4.42
OsR498G0713703300.01	14.11	31.31	1.1482686	8.04E-250	1.98E-250	14.46	14.29	13.58	32.8	31.85	29.28
OsR498G0713708100.01	0.793	3.093	1.9895698	2.30E-31	2.27E-31	0.6	0.66	1.12	2.86	3.41	3.01
OsR498G0713709500.01	0.243	1.32	2.4370369	1.58E-61	1.08E-61	0.19	0.24	0.3	1.25	1.42	1.29
OsR498G0713710700.01	0.446	1.29	1.4906597	3.03E-10	4.64E-10	0.35	0.63	0.36	1.64	1.19	1.04
OsR498G0713711700.01	5.283	28.626	2.4315115	4.89E-246	1.23E-246	5.5	5.58	4.77	28.36	27.02	30.5
OsR498G0713712300.01	0.376	0	-4.932481	3.36E-05	6.31E-05	0.3	0.3	0.53	0	0	0
OsR498G0713713900.01	105.216	231.11	1.1366451	0	0	106.35	106.63	102.67	232.67	237.13	223.54
OsR498G0713719900.01	0.856	0.033	-4.72603	1.01E-07	1.69E-07	0.5	1.77	0.3	0.1	0	0
OsR498G0713721600.01	88.363	19.193	-2.194371	0	0	84.96	90.91	89.22	18.69	20.43	18.46
OsR498G0713721900.01	3.013	0.27	-3.485022	2.44E-12	3.50E-12	2.97	2.47	3.6	0.32	0.16	0.33
OsR498G0713723400.01	0.36	0.043	-3.091679	2.20E-13	3.07E-13	0.29	0.45	0.34	0.08	0.02	0.03
OsR498G0713724300.01	0.643	3.413	2.4047605	1.33E-30	1.32E-30	0.71	0.84	0.38	3.31	3.29	3.64
OsR498G0713727400.01	22.733	84.09	1.8803573	4.29E-293	9.22E-294	22.38	23.82	22	76.16	87.75	88.36
OsR498G0713731400.01	4.18	0.413	-3.337759	7.99E-165	2.81E-165	4.26	4.22	4.06	0.5	0.36	0.38
OsR498G0713734100.01	9.846	21.293	1.1152554	2.77E-96	1.43E-96	9	10.84	9.7	21.92	21.79	20.17
OsR498G0713741900.01	4.793	2.623	-1.09567	7.53E-29	7.72E-29	4.58	4.58	5.22	2.36	2.31	3.2
OsR498G0713759600.01	3.933	1.843	-1.043512	9.40E-28	9.84E-28	3.61	4.5	3.69	1.97	2.04	1.52
OsR498G0713761600.01	2.863	0.176	-4.02559	3.65E-22	4.20E-22	2.79	3.3	2.5	0.26	0.09	0.18
OsR498G0713768000.01	3.83	11.833	1.6213433	4.58E-70	2.88E-70	3.64	3.84	4.01	11.69	12.55	11.26
OsR498G0713790800.01	12.326	30.01	1.2913025	1.96E-159	7.06E-160	13.05	12.08	11.85	31.58	28.93	29.52

OsR498G0713792500.01	1.31	4.29	1.6939935	2.96E-47	2.35E-47	1.15	1.29	1.49	4.47	4	4.4
OsR498G0713792900.01	1.353	6.35	2.1996078	2.29E-67	1.47E-67	1.29	1.35	1.42	6.58	6.48	5.99
OsR498G0713793200.01	0	0.103	5.1371034	7.28E-06	1.33E-05	0	0	0	0.15	0.08	0.08
OsR498G0713793300.01	120.956	293.81	1.2735289	0	0	116.75	125.57	120.55	289.39	294.62	297.41
OsR498G0713795500.01	0	1.56	4.5593725	0.0002368	0.0004696	0	0	0	1.56	1.16	1.96
OsR498G0713795700.01	12.456	73.586	2.5628104	0	0	12.22	12.19	12.96	72.48	75.66	72.62
OsR498G0713804500.01	0.303	0.626	1.0545803	0.0001163	0.0002265	0.29	0.28	0.34	0.83	0.48	0.57
OsR498G0713805700.01	3.213	15.436	2.2629795	7.67E-126	3.29E-126	3.37	2.74	3.53	14.32	14.88	17.11
OsR498G0713819100.01	2.653	1.156	-1.09774	1.69E-12	2.42E-12	2.12	3.56	2.28	0.69	1.19	1.59
OsR498G0713825000.01	9.456	50.76	2.4245454	1.53E-265	3.55E-266	10.46	8.73	9.18	50.4	50.79	51.09
OsR498G0713826600.01	11.623	52.293	2.1681817	0	0	11.39	11.17	12.31	51.82	50.03	55.03
OsR498G0713827300.01	0.706	1.64	1.2172665	6.19E-09	9.92E-09	0.85	0.53	0.74	1.41	1.64	1.87
OsR498G0713828700.01	1.313	5.106	1.9585091	6.27E-18	7.84E-18	1.57	1.01	1.36	4.86	5.41	5.05
OsR498G0713829700.01	8.023	19.386	1.2762943	6.11E-63	4.12E-63	7.72	8.31	8.04	18.62	19.14	20.4
OsR498G0713830500.01	0.06	0.273	2.2223375	0.0006354	0.0012994	0	0.09	0.09	0.34	0.17	0.31
OsR498G0713833100.01	53.37	145.55	1.4461901	0	0	50.27	52.65	57.19	143.83	141.61	151.22
OsR498G0713833600.01	15.973	49.686	1.6347177	5.12E-85	2.88E-85	14.83	13.75	19.34	49.22	50.48	49.36
OsR498G0713855000.01	14.73	1.246	-3.566353	4.10E-133	1.69E-133	15	15.79	13.4	1.41	1.21	1.12
OsR498G0713856600.01	2.626	5.343	1.0238786	1.46E-08	2.38E-08	3.13	2.47	2.28	5.19	5.25	5.59
OsR498G0713856800.01	0.063	0.513	3.1148912	2.14E-18	2.64E-18	0.07	0.05	0.07	0.51	0.38	0.65
OsR498G0713859300.01	4.076	1.493	-1.45388	7.71E-29	7.91E-29	3.75	4.47	4.01	1.63	1.37	1.48
OsR498G0713860700.01	5.66	1.54	-1.931808	8.83E-56	6.38E-56	5.28	5.63	6.07	1.65	1.43	1.54
OsR498G0713863300.01	0.186	0.94	2.3529216	1.05E-11	1.53E-11	0.19	0.15	0.22	1.26	0.71	0.85
OsR498G0713864300.01	0.273	3.05	3.4889831	6.08E-32	5.94E-32	0.35	0.12	0.35	3.18	2.94	3.03
OsR498G0713869100.01	149.74	69.94	-1.100071	6.54E-193	2.02E-193	150.36	145.94	152.92	76.86	61.29	71.67
OsR498G0713877600.01	13.813	31.726	1.2011808	6.64E-121	2.92E-121	13.12	14.03	14.29	33.1	32.86	29.22
OsR498G0713888200.01	0.323	2.02	2.6589081	4.55E-28	4.73E-28	0.35	0.24	0.38	1.97	2.03	2.06
OsR498G0713889300.01	18.98	80.94	2.0921945	0	0	20.28	17.52	19.14	82.53	77.61	82.68
OsR498G0713892000.01	0.246	1.056	2.0999408	4.67E-07	8.06E-07	0.2	0.27	0.27	0.65	1.24	1.28
OsR498G0713897300.01	27.633	4.57	-2.599644	4.33E-154	1.60E-154	25.53	28.63	28.74	5.38	3.52	4.81
OsR498G0713912700.01	0.963	2.656	1.4598368	1.01E-30	1.01E-30	0.88	0.89	1.12	2.7	2.81	2.46
OsR498G0713916200.01	0.506	0.123	-2.125126	1.27E-08	2.05E-08	0.31	0.73	0.48	0.11	0.05	0.21
OsR498G0713923500.01	0.32	1.683	2.24069	3.93E-23	4.44E-23	0.23	0.26	0.47	1.64	1.8	1.61
OsR498G0713935400.01	2.61	0.936	-1.479308	1.65E-10	2.51E-10	2.62	2.54	2.67	1.03	0.97	0.81
OsR498G0713938000.01	7.476	34.453	2.2033434	1.52E-277	3.41E-278	7.85	7.81	6.77	35.68	34.09	33.59
OsR498G0713940400.01	0.72	0.106	-2.734541	8.14E-15	1.10E-14	0.87	0.78	0.51	0.12	0.1	0.1
OsR498G0713942500.01	0.613	0.27	-1.269516	0.0003809	0.0007662	0.84	0.52	0.48	0.39	0.19	0.23
OsR498G0713946200.01	0.69	0.11	-2.65377	0.0002687	0.0005351	0.9	0.7	0.47	0.18	0	0.15
OsR498G0713948200.01	12.723	6.196	-1.037367	1.91E-16	2.48E-16	14.02	11.87	12.28	6.06	6.94	5.59
OsR498G0713968500.01	4.076	15.78	1.945651	1.02E-120	4.51E-121	3.68	4.18	4.37	15.42	15.79	16.13
OsR498G0713969200.01	0.166	0.953	2.8252665	1.61E-17	2.04E-17	0.26	0.06	0.18	0.87	1.1	0.89
OsR498G0713971700.01	6.063	12.73	1.0686004	6.18E-60	4.27E-60	6.09	5.72	6.38	12.35	12.79	13.05
OsR498G0713984000.01	4.416	9.956	1.1704751	1.06E-61	7.19E-62	4.27	4.89	4.09	10.28	10.84	8.75
OsR498G0713984400.01	10.116	79.906	2.9687634	0	0	8.44	10.91	11	78.24	80.35	81.13
OsR498G0713985600.01	2.716	7.46	1.4043903	7.84E-103	3.86E-103	2.77	2.61	2.77	7.36	7.57	7.45
OsR498G0713986700.01	0.27	0.726	1.4395327	4.47E-10	6.89E-10	0.21	0.35	0.25	0.74	0.84	0.6
OsR498G0713992100.01	6.646	0.513	-3.694274	2.18E-136	8.79E-137	6.03	6.87	7.04	0.45	0.44	0.65
OsR498G0713996900.01	0.183	0.046	-1.89958	0.0001136	0.0002211	0.11	0.29	0.15	0.07	0.04	0.03
OsR498G0713999000.01	18.206	120.74	2.7269767	0	0	18.1	18.25	18.27	121.41	122.17	118.65
OsR498G0713999800.01	8.403	28.216	1.8713834	4.41E-283	9.76E-284	8.74	7.7	8.77	27.82	29.06	27.77
OsR498G0714014500.01	56.893	126.31	1.1245295	0	0	55.55	58.01	57.12	130.69	127.19	121.04
OsR498G0714024700.01	0.576	1.296	1.1723493	2.01E-05	3.73E-05	0.72	0.3	0.71	1.23	1.29	1.37
OsR498G0714026200.01	1.046	2.666	1.3505584	5.25E-09	8.40E-09	1.21	1.13	0.8	2.35	3.44	2.21
OsR498G0714030900.01	10.27	25.056	1.2852201	4.68E-69	2.97E-69	10.82	10.85	9.14	25.39	23.59	26.19
OsR498G0714031400.01	0.04	0.26	2.8488791	3.66E-05	6.89E-05	0.05	0	0.07	0.35	0.18	0.25
OsR498G0714035300.01	7.64	16.813	1.0437875	1.42E-74	8.64E-75	8.05	7.96	6.91	16.34	16.93	17.17
OsR498G0714035800.01	12.216	24.096	1.0657843	4.18E-58	2.95E-58	13.37	11.76	11.52	24.11	23.38	24.8
OsR498G0714051300.01	18.38	37.88	1.0372733	3.51E-70	2.20E-70	19.08	18.35	17.71	37.9	36	39.74
OsR498G0714055800.01	12.186	34.923	1.4840072	9.68E-261	2.29E-261	12.84	12.31	11.41	34.43	35.41	34.93
OsR498G0714086300.01	0	0.21	4.2963381	0.0007942	0.0016388	0	0	0	0.19	0.25	0.19
OsR498G0714087400.01	4.66	9.706	1.0763234	7.93E-45	6.46E-45	5.74	4.32	3.92	9.43	9.64	10.05
OsR498G0714089600.01	0	0.96	5.8813006	8.71E-09	1.40E-08	0	0	0	1.15	0.95	0.78
OsR498G0714091600.01	0	0.186	4.97441	2.21E-05	4.12E-05	0	0	0	0.28	0.14	0.14
OsR498G0714096400.01	645.626	75.633	-3.068629	0	0	652.92	654.52	629.44	74.19	79.42	73.29
OsR498G0714096700.01	6.85	16.2	1.2394467	3.68E-39	3.24E-39	6.86	6.52	7.17	14.99	16.39	17.22
OsR498G0714097600.01	26.323	5.523	-2.255267	1.06E-220	2.90E-221	25.42	26.39	27.16	4.96	5.54	6.07
OsR498G0714098100.01	11.24	4.023	-1.482797	9.70E-47	7.73E-47	10.36	10.16	13.2	4.06	3.84	4.17
OsR498G0714100900.01	3.323	10.31	1.6336358	3.65E-47	2.89E-47	3.34	3.15	3.48	10.09	10.15	10.69
OsR498G0714112900.01	0.05	0.406	3.0740418	5.12E-09	8.18E-09	0.03	0.06	0.06	0.51	0.42	0.29

OsR498G0714129300.01	1.773	0.59	-1.586305	2.91E-06	5.19E-06	1.36	1.8	2.16	0.79	0.53	0.45
OsR498G0714131800.01	0.543	1.18	1.1249696	1.11E-06	1.94E-06	0.59	0.39	0.65	1.14	1.1	1.3
OsR498G0714136000.01	3.72	1.473	-1.371766	4.64E-48	3.65E-48	3.69	3.54	3.93	1.28	1.37	1.77
OsR498G0714154900.01	982.6	181.9	-2.445414	0	0	967.01	1018.4	962.36	174.88	178.87	191.95
OsR498G0714155200.01	2.25	17.183	2.9317824	0	0	2.15	2.46	2.14	17.64	17.41	16.5
OsR498G0714159000.01	3.043	0.093	-4.983224	3.02E-69	1.91E-69	3.29	2.85	2.99	0.04	0.15	0.09
OsR498G0714162100.01	12.683	35.226	1.5209746	7.92E-248	1.98E-248	12.31	13.16	12.58	35.56	35.47	34.65
OsR498G0714165900.01	2.103	5.873	1.4748389	3.44E-33	3.30E-33	2.47	1.92	1.92	6.69	5.75	5.18
OsR498G0714174100.01	6.493	2.25	-1.571123	4.42E-40	3.85E-40	7.14	5.16	7.18	1.99	2.45	2.31
OsR498G0714174300.01	0.023	3.603	7.6678969	5.14E-46	4.13E-46	0	0	0.07	3.93	3.95	2.93
OsR498G0714178700.01	11.57	0.786	-3.874968	4.17E-88	2.29E-88	12.18	11.26	11.27	0.89	0.81	0.66
OsR498G0714181800.01	1.566	0.37	-2.081182	3.89E-34	3.67E-34	1.45	1.63	1.62	0.3	0.44	0.37
OsR498G0714183600.01	19.926	47.39	1.2651723	0	0	18.79	19.79	21.2	49.62	45.63	46.92
OsR498G0714188400.01	14.47	44.726	1.5513447	3.61E-184	1.16E-184	16.13	12.69	14.59	46.33	45.12	42.73
OsR498G0714189600.01	0.136	0.676	2.3263953	5.79E-14	7.95E-14	0.13	0.13	0.15	0.75	0.82	0.46
OsR498G0714192100.01	4.913	20.776	2.0789733	3.14E-140	1.24E-140	4.46	4.91	5.37	21.32	19.15	21.86
OsR498G0714192300.01	0.76	1.61	1.0848339	1.04E-05	1.91E-05	0.78	0.77	0.73	1.77	1.71	1.35
OsR498G0714192500.01	2.176	16.77	2.9459712	1.21E-166	4.19E-167	2.42	2.45	1.66	16.75	17.1	16.46
OsR498G0714195500.01	416.013	963.07	1.206849	0	0	406.98	423.09	417.97	949.39	938.26	1001.6
OsR498G0714196800.01	2.91	15.263	2.4904251	2.98E-79	1.75E-79	2.64	2.76	3.33	15.07	14.91	15.81
OsR498G0714200800.01	13.06	6.28	-1.021621	7.08E-30	7.15E-30	13.18	12.34	13.66	6.33	6.54	5.97
OsR498G0714211000.01	0.76	0.423	-1.007074	6.93E-10	1.07E-09	0.79	0.78	0.71	0.45	0.24	0.58
OsR498G0714213200.01	0.463	1.553	1.7870398	3.07E-26	3.30E-26	0.54	0.37	0.48	1.62	1.53	1.51
OsR498G0714236700.01	0.16	1.306	3.0333036	3.97E-10	6.11E-10	0.16	0.08	0.24	1.4	1.24	1.28
OsR498G0714240000.01	2.5	5.236	1.0551113	1.49E-16	1.93E-16	2.23	2.42	2.85	5.22	5.56	4.93
OsR498G0714245400.01	2.193	4.453	1.0182642	4.44E-08	7.35E-08	1.39	2.84	2.35	4.28	3.71	5.37
OsR498G0714245600.01	10.97	1.566	-2.80809	4.02E-101	2.01E-101	11.03	11.18	10.7	1.67	1.21	1.82
OsR498G0714246500.01	0.763	0.09	-3.113053	1.12E-07	1.88E-07	0.41	1.07	0.81	0.13	0.07	0.07
OsR498G0714248200.01	193.003	83.46	-1.204556	0	0	195.77	194.58	188.66	83.36	81.92	85.1
OsR498G0714261400.01	5.903	1.223	-2.270897	7.28E-36	6.70E-36	6.28	5.9	5.53	1.19	1.19	1.29
OsR498G0714263100.01	361.076	114.24	-1.662609	0	0	358.04	363.63	361.56	111.07	109	122.65
OsR498G0714268000.01	7.393	17.53	1.2501963	2.78E-54	2.04E-54	7.39	7.17	7.62	17.15	17.49	17.95
OsR498G0714268500.01	12.293	27.36	1.1488631	7.69E-214	2.17E-214	12.56	11.78	12.54	27.31	27.67	27.1
OsR498G0714269700.01	0.496	1.976	1.9900068	1.50E-30	1.50E-30	0.52	0.45	0.52	1.47	2.36	2.1
OsR498G0714273300.01	0.47	0.18	-1.347518	0.0001051	0.0002039	0.54	0.45	0.42	0.05	0.38	0.11
OsR498G0714283900.01	35.53	8.133	-2.00073	1.45E-183	4.65E-184	34.52	35.84	36.23	8.53	7.46	8.41
OsR498G0714285400.01	5.683	1.31	-2.150396	1.06E-43	8.77E-44	6.11	5.49	5.45	1.41	1.45	1.07
OsR498G0714287500.01	0.24	0	-4.485022	0.0003787	0.0007614	0.13	0.39	0.2	0	0	0
OsR498G0714288800.01	1.256	9.093	2.8592742	7.50E-104	3.67E-104	1.55	1.32	0.9	9.54	9.13	8.61
OsR498G0714289000.01	0.536	1.993	1.832391	1.51E-20	1.80E-20	0.71	0.51	0.39	2.27	1.96	1.75
OsR498G0714294600.01	167.223	24.803	-2.75409	0	0	175.51	153.07	173.09	24.62	23.82	25.97
OsR498G0714294700.01	84.383	13.32	-2.667038	0	0	71.71	94.04	87.4	13.58	10.3	16.08
OsR498G0714295000.01	2.476	4.89	1.0214353	7.77E-15	1.05E-14	2.67	2.67	2.09	5.01	4.87	4.79
OsR498G0714302100.01	9.793	93.766	3.201754	0	0	8.9	9.66	10.82	91.81	94.81	94.68
OsR498G0714302300.01	30.823	71.423	1.1994423	2.96E-270	6.81E-271	31.77	30.29	30.41	70.09	70.09	74.09
OsR498G0714307700.01	0.356	0.073	-2.561643	7.24E-06	1.32E-05	0.48	0.44	0.15	0.07	0.04	0.11
OsR498G0714308700.01	10.28	23.17	1.1855718	9.83E-109	4.66E-109	10.69	10.27	9.88	22.71	24.17	22.63
OsR498G0714308900.01	0.97	4.793	2.2947605	3.41E-114	1.57E-114	0.94	0.87	1.1	5.33	4.81	4.24
OsR498G0714309200.01	4.543	10.546	1.2630449	2.68E-119	1.19E-119	4.3	4.98	4.35	10.7	10.73	10.21
OsR498G0714310200.01	0.013	0.486	5.3667274	1.45E-11	2.14E-11	0	0	0.04	0.69	0.31	0.46
OsR498G0714315500.01	0.383	0.083	-2.163094	0.0002982	0.0005957	0.38	0.59	0.18	0.05	0.14	0.06
OsR498G0714316600.01	0.793	0.136	-2.347518	6.24E-05	0.0001194	1.17	0.65	0.56	0.17	0.08	0.16
OsR498G0714319700.01	5.84	22.956	1.9302514	7.03E-240	1.81E-240	5.62	5.38	6.52	24.46	21.93	22.48
OsR498G0714320400.01	6.726	14.216	1.0613933	3.29E-32	3.19E-32	6.16	7.42	6.6	15.25	14.78	12.62
OsR498G0714327900.01	0.853	0.03	-4.744408	6.03E-20	7.24E-20	0.59	1.03	0.94	0.03	0.06	0
OsR498G0714331100.01	2.24	10.446	2.2216001	1.16E-59	8.03E-60	2.21	2.35	2.16	9.37	10.32	11.65
OsR498G0714335000.01	14.316	31.88	1.1408711	4.26E-129	1.79E-129	13.81	13.9	15.24	33.66	30.51	31.47
OsR498G0714335100.01	2.773	13.11	2.1901969	1.26E-94	6.57E-95	2.5	2.85	2.97	13.41	14.12	11.8
OsR498G0714337200.01	11.993	27.996	1.2272221	1.72E-103	8.45E-104	11.86	12.43	11.69	28.44	26.9	28.65
OsR498G0714345300.01	0.85	0.31	-1.479308	4.27E-06	7.70E-06	0.9	0.93	0.72	0.24	0.28	0.41
OsR498G0714350300.01	28.09	11.586	-1.301835	5.67E-129	2.39E-129	27.97	27.63	28.67	11.52	12.37	10.87
OsR498G0714354100.01	0.326	1.316	1.8577493	1.80E-08	2.93E-08	0.34	0.37	0.27	1.83	1.26	0.86
OsR498G0714357500.01	13.686	45.136	1.7047338	0	0	14.16	14.16	12.74	43.82	46.2	45.39
OsR498G0714359600.01	0.293	0.006	-4.113053	4.44E-09	7.08E-09	0.21	0.53	0.14	0.01	0.01	0
OsR498G0714374800.01	13.736	102.39	2.9020787	0	0	13.77	14.29	13.15	99.7	102.01	105.47
OsR498G0714384000.01	2.026	0.303	-3.093335	1.16E-20	1.37E-20	1.74	2.32	2.02	0.43	0.3	0.18
OsR498G0714391000.01	1.536	8.55	2.4721213	1.96E-109	9.23E-110	1.45	1.31	1.85	8.67	8.75	8.23
OsR498G0714394600.01	10.473	22.58	1.1267443	7.22E-88	3.97E-88	11.23	10.04	10.15	23.1	22.96	21.68
OsR498G0714401700.01	0.04	0.27	2.637375	0.0001939	0.0003825	0.04	0.04	0.04	0.34	0.21	0.26

OsR498G0714402400.01	210.23	531.64	1.3184755	0	0	213.54	207.51	209.64	514.69	512.6	567.64
OsR498G0714403500.01	3.813	11.146	1.5807914	7.63E-73	4.70E-73	3.76	3.99	3.69	12.13	10.96	10.35
OsR498G0714405700.01	11.896	24.14	1.0201283	3.08E-84	1.74E-84	12.78	11.97	10.94	23.92	23.42	25.08
OsR498G0714407100.01	0.11	0.6	2.4338416	5.63E-12	8.18E-12	0.07	0.07	0.19	0.72	0.53	0.55
OsR498G0714413800.01	0.186	0.046	-2.163094	0.0002982	0.0005957	0.13	0.2	0.23	0	0.02	0.12
OsR498G0714418200.01	0.373	0.953	1.354682	1.47E-11	2.17E-11	0.29	0.44	0.39	1.06	0.83	0.97
OsR498G0714418300.01	243.63	97.47	-1.31568	0	0	234.69	241.83	254.37	96.93	93.04	102.44
OsR498G0714419600.01	0.72	0.316	-1.095438	0.0005534	0.0011254	0.85	0.74	0.57	0.27	0.49	0.19
OsR498G0714419700.01	0.176	0.046	-1.868721	0.000589	0.0012016	0.15	0.27	0.11	0.1	0	0.04
OsR498G0714420700.01	2.973	6.51	1.1265435	1.84E-56	1.32E-56	3.18	3.02	2.72	6.59	6.38	6.56
OsR498G0714424300.01	0.606	1.7	1.4947528	3.75E-12	5.42E-12	0.64	0.84	0.34	1.51	1.67	1.92
OsR498G0714429900.01	0.07	0.353	2.3765084	1.68E-06	2.97E-06	0.06	0.12	0.03	0.27	0.46	0.33
OsR498G0714431500.01	5.333	0.523	-3.32771	8.40E-42	7.14E-42	5.56	5.76	4.68	0.72	0.27	0.58
OsR498G0714435900.01	0.03	0.486	3.9440363	1.84E-11	2.71E-11	0.03	0.06	0	0.46	0.34	0.66
OsR498G0714443100.01	0.596	0.203	-1.556922	7.91E-06	1.44E-05	0.47	0.67	0.65	0.23	0.12	0.26
OsR498G0714446100.01	6.786	1.296	-2.540651	1.87E-61	1.27E-61	6.95	6.36	7.05	1.24	1.65	1
OsR498G0714447600.01	2.406	7.71	1.681554	9.00E-98	4.60E-98	2.75	2.49	1.98	7.67	8.39	7.07
OsR498G0714454900.01	7.99	41.4	2.3760898	0	0	8.36	7.45	8.16	40.95	43.09	40.16
OsR498G0714458500.01	42.123	145.87	1.7946261	0	0	42.73	43.93	39.71	149.84	138.92	148.86
OsR498G0714459300.01	56.936	204.35	1.838479	0	0	53.37	57.31	60.13	205.14	215.02	192.9
OsR498G0714459700.01	4.876	22.276	2.1448342	4.89E-28	5.09E-28	5.68	4.19	4.76	21.12	21.2	24.51
OsR498G0714461100.01	0.2	0.933	2.2402594	2.09E-05	3.89E-05	0	0.44	0.16	0	0.39	2.41
OsR498G0714462100.01	0.18	1.143	2.4769103	1.18E-08	1.92E-08	0.48	0.06	0	1.47	0.91	1.05
OsR498G0714469600.01	1.1	0.083	-3.791125	1.12E-15	1.47E-15	1.22	1.06	1.02	0.05	0.05	0.15
OsR498G0714480600.01	0.006	1.583	7.5593725	3.42E-43	2.84E-43	0	0	0.02	1.53	1.49	1.73
OsR498G0714482300.01	0.51	2.46	2.2639166	3.69E-23	4.16E-23	0.43	0.38	0.72	2.25	2.44	2.69
OsR498G0714483700.01	13.806	4.196	-1.720129	6.57E-70	4.14E-70	13.39	12.43	15.6	4.23	3.65	4.71
OsR498G0714484800.01	6.466	3.173	-1.057494	2.15E-24	2.37E-24	6.57	6.55	6.28	2.58	3.41	3.53
OsR498G0714487700.01	2.703	6.313	1.2212795	6.44E-45	5.24E-45	2.39	3.08	2.64	6.08	6.19	6.67
OsR498G0714490900.01	1.533	0.233	-2.715906	1.38E-12	1.96E-12	1.63	1.81	1.16	0	0.38	0.32
OsR498G0714491100.01	1.476	0	-5.979786	3.80E-09	6.05E-09	2.01	1	1.42	0	0	0
OsR498G0714494500.01	1.383	7.03	2.365495	4.83E-60	3.33E-60	1.27	1.31	1.57	7.26	7.06	6.77
OsR498G0714494900.01	0	0.273	4.2963381	0.0007942	0.0016388	0	0	0	0.41	0.16	0.25
OsR498G0714495300.01	0.396	2.08	2.5061304	5.44E-20	6.53E-20	0.23	0.51	0.45	2.03	1.77	2.44
OsR498G0714495500.01	1.29	3.96	1.635751	1.27E-44	1.03E-44	1.19	1.17	1.51	3.38	3.97	4.53
OsR498G0714496300.01	0.063	1.763	4.8535556	1.50E-48	1.17E-48	0.1	0.03	0.06	2.06	1.77	1.46
OsR498G0714497100.01	0.593	4.88	3.0999408	1.35E-99	6.79E-100	0.51	0.64	0.63	4.48	4.84	5.32
OsR498G0714497700.01	0.536	0.253	-1.174453	0.0003757	0.0007549	0.41	0.88	0.32	0.33	0.16	0.27
OsR498G0714500100.01	1.06	0.523	-1.114857	3.38E-06	6.06E-06	0.83	1.14	1.21	0.56	0.43	0.58
OsR498G0714501100.01	0.746	4	2.4191948	4.16E-21	4.89E-21	0.83	0.91	0.5	3.73	3.87	4.4
OsR498G0714502100.01	0.4	3.263	3.1126261	7.40E-71	4.62E-71	0.26	0.4	0.54	3.37	3.17	3.25
OsR498G0714502600.01	0.06	0.206	1.7740894	0.0008111	0.0016748	0.03	0.05	0.1	0.18	0.19	0.25
OsR498G0714503300.01	0.006	0.19	4.4979719	7.90E-07	1.38E-06	0	0.02	0	0.15	0.22	0.2
OsR498G0714507100.01	0.13	0.663	2.3436438	1.27E-09	1.98E-09	0.1	0.16	0.13	0.64	0.63	0.72
OsR498G0714510800.01	0.71	0.076	-3.254409	2.19E-15	2.91E-15	0.63	0.85	0.65	0.11	0.06	0.06
OsR498G0714515000.01	8.033	19.02	1.2748676	4.28E-135	1.74E-135	8.16	8.22	7.72	17.46	20.99	18.61
OsR498G0714515300.01	1.34	5.683	2.0817629	9.16E-144	3.55E-144	1.24	1.42	1.36	5.61	6.09	5.35
OsR498G0714516900.01	2.626	1.026	-1.340577	2.35E-20	2.80E-20	2.82	2.38	2.68	1.13	0.89	1.06
OsR498G0714520000.01	0.35	0.73	1.0465597	6.00E-05	0.0001146	0.3	0.4	0.35	0.63	0.92	0.64
OsR498G0714521700.01	12.38	4.29	-1.523787	2.35E-98	1.19E-98	12.81	11.83	12.5	4.25	4.03	4.59
OsR498G0714524300.01	1037.05	55.303	-4.189838	0	0	942.12	1050.6	1118.4	57.96	52.07	55.88
OsR498G0714524600.01	11.653	0.973	-3.579191	1.66E-170	5.65E-171	8.69	13.69	12.58	1.08	1.17	0.67
OsR498G0714527900.01	958.263	260.22	-1.911588	0	0	957.9	959.29	957.6	259.93	263.11	257.63
OsR498G0714528300.01	4.666	1.403	-1.741797	6.83E-14	9.40E-14	5.4	4.15	4.45	1.69	0.69	1.83
OsR498G0714531000.01	54.57	214.53	1.9735987	0	0	49.53	58.87	55.31	210.66	213.29	219.64
OsR498G0714531200.01	2.256	6.576	1.5200595	5.85E-53	4.35E-53	2.01	2.19	2.57	6.18	6.81	6.74
OsR498G0714533900.01	9.07	3.73	-1.284283	5.15E-72	3.19E-72	9.77	9.11	8.33	3.69	3.92	3.58
OsR498G0714534100.01	3.433	8.543	1.257229	3.94E-46	3.16E-46	3.8	3.25	3.25	8.09	9.26	8.28
OsR498G0714537100.01	5.603	12.9	1.2017482	7.33E-26	7.91E-26	5.68	5.24	5.89	12.7	12.65	13.35
OsR498G0714537700.01	40.293	4.476	-3.171258	5.68E-204	1.67E-204	40.98	40.71	39.19	4.44	4.41	4.58
OsR498G0714541400.01	0.22	2.16	3.3118511	3.50E-69	2.22E-69	0.23	0.18	0.25	2.39	2.14	1.95
OsR498G0714549300.01	3.193	19.763	2.7028487	0	0	3.42	2.79	3.37	20.13	20.23	18.93
OsR498G0714549600.01	0.69	3.39	2.2963381	3.48E-09	5.53E-09	0.69	0.35	1.03	3.54	3.19	3.44
OsR498G0714549800.01	0.703	10.666	3.9239732	1.56E-69	9.84E-70	0.8	0.63	0.68	11.67	10.65	9.68
OsR498G0714550000.01	1.866	5.24	1.4924985	6.35E-55	4.62E-55	1.72	1.69	2.19	5.03	5.4	5.29
OsR498G0714559100.01	3003.98	1424.9	-1.078502	0	0	2930	3007.1	3074.9	1388.3	1379.3	1506.9
OsR498G0714565000.01	7.99	20.873	1.3856372	7.57E-180	2.48E-180	8.25	7.84	7.88	21.08	21.31	20.23
OsR498G0714565800.01	0.256	2.3	3.1630715	1.12E-28	1.15E-28	0.27	0.23	0.27	2.03	2.33	2.54
OsR498G0714567400.01	27.57	4.01	-2.812299	0	0	26.67	28.23	27.81	3.46	4.44	4.13

OsR498G0714568000.01	1489.8	55.45	-4.762541	0	0	1500.6	1472.1	1496.7	53.95	55.6	56.8
OsR498G0714569300.01	0.856	0.096	-4.02559	1.47E-08	2.39E-08	0.89	0.88	0.8	0.21	0	0.08
OsR498G0714569500.01	1.493	0.283	-2.38816	1.76E-06	3.11E-06	1.25	1.99	1.24	0.37	0.36	0.12
OsR498G0714570800.01	85.44	35.253	-1.277554	0	0	86.15	84.47	85.7	35.74	34.45	35.57
OsR498G0714572400.01	0.123	0	-4.832945	6.10E-05	0.0001167	0.1	0.14	0.13	0	0	0
OsR498G0714575800.01	0.153	13.353	6.4289149	1.08E-203	3.18E-204	0.09	0.14	0.23	13.1	13.45	13.51
OsR498G0714577600.01	0.736	0.183	-2.02559	6.04E-10	9.35E-10	0.94	0.69	0.58	0.23	0.06	0.26
OsR498G0714578700.01	1878.36	575.57	-1.708141	0	0	1865.3	1822.6	1947.2	562.95	545.51	618.26
OsR498G0714580500.01	5.46	15.69	1.5199213	1.65E-37	1.48E-37	4.85	5.67	5.86	14.16	16.22	16.69
OsR498G0714582200.01	0.113	1.673	3.8919478	1.57E-29	1.59E-29	0.23	0.07	0.04	1.61	1.6	1.81
OsR498G0714583500.01	0.543	1.32	1.2815193	2.47E-20	2.95E-20	0.53	0.48	0.62	1.54	1.14	1.28
OsR498G0714584500.01	4.053	8.35	1.0701689	1.30E-28	1.34E-28	3.81	3.98	4.37	8.56	8.16	8.33
OsR498G0714584800.01	0.106	0.813	2.97441	2.83E-12	4.07E-12	0.08	0.12	0.12	0.83	0.83	0.78
OsR498G0714585900.01	0.13	1.423	3.5333772	3.44E-33	3.29E-33	0.11	0.2	0.08	1.72	1.34	1.21
OsR498G0714587400.01	10.87	77.213	2.8277883	0	0	10.95	10.53	11.13	78.81	78.18	74.65
OsR498G0714587600.01	10.963	78.776	2.8375902	0	0	10.95	10.81	11.13	80.51	79.81	76.01
OsR498G0714588000.01	2.876	6.38	1.1507795	3.06E-30	3.07E-30	2.8	3.12	2.71	6.44	7.15	5.55
OsR498G0714589200.01	0.163	1.15	2.8073	1.35E-10	2.04E-10	0.19	0.12	0.18	1.5	0.72	1.23
OsR498G0714591300.01	1.366	4.193	1.6148676	5.59E-10	8.64E-10	0.61	2.05	1.44	4.48	3.52	4.58
OsR498G0714591800.01	15.133	51.226	1.7606031	0	0	16.23	14.92	14.25	51.63	50.91	51.14
OsR498G0714592800.01	0.22	0.726	1.7334019	2.66E-09	4.20E-09	0.23	0.23	0.2	0.86	0.74	0.58
OsR498G0714594600.01	4.25	13.433	1.6564838	1.42E-59	9.87E-60	4.19	3.49	5.07	13.74	12.38	14.18
OsR498G0714594900.01	0.56	0.063	-3.227224	4.72E-10	7.27E-10	0.54	0.6	0.54	0.05	0.07	0.07
OsR498G0714596000.01	0.996	2.16	1.1033964	6.63E-15	8.91E-15	1	0.99	1	2.46	2	2.02
OsR498G0714598000.01	18.073	73.04	1.9779979	0	0	19.14	17.87	17.21	71.91	72.74	74.47
OsR498G0714598700.01	0.363	6.81	4.9593031	7.44E-67	4.82E-67	0.34	0.3	0.45	5.66	7.84	6.93
OsR498G0714600800.01	2.95	10.31	1.7706415	3.11E-30	3.12E-30	3.61	2.14	3.1	9.4	10.51	11.02
OsR498G0714602700.01	4.136	12.886	1.6382279	2.05E-68	1.31E-68	4.39	4.49	3.53	12.1	12.91	13.65
OsR498G0714602900.01	0.093	1.223	3.6840682	1.28E-32	1.24E-32	0.05	0.02	0.21	1.32	1.06	1.29
OsR498G0714603200.01	1.556	4.276	1.4449821	1.14E-48	8.93E-49	1.55	1.46	1.66	4.34	4.12	4.37
OsR498G0714604900.01	29.26	60.77	1.0558617	3.21E-156	1.17E-156	29.29	29.32	29.17	62.55	61.38	58.38
OsR498G0714606800.01	2.833	10.88	1.9392535	3.28E-54	2.40E-54	3.1	3.07	2.33	11.12	9.85	11.67
OsR498G0714608200.01	1.363	0.436	-1.647078	3.03E-08	4.98E-08	1.9	1.22	0.97	0.55	0.45	0.31
OsR498G0714608600.01	3.51	8.62	1.3005959	1.30E-58	9.14E-59	3.33	3.53	3.67	8.85	8.47	8.54
OsR498G0714609700.01	0	0.146	5.0618728	1.23E-05	2.27E-05	0	0	0	0.19	0.12	0.13
OsR498G0714612500.01	1.18	7.603	2.6837006	6.31E-50	4.85E-50	0.64	1.41	1.49	7.61	6.82	8.38
OsR498G0714613300.01	0.333	11.103	5.2515682	1.68E-82	9.65E-83	0.67	0	0.33	10.99	10.72	11.6
OsR498G0714614400.01	4.776	25.883	2.4380662	2.74E-137	1.10E-137	4.8	5.07	4.46	23.83	28.22	25.6
OsR498G0714615300.01	7.8	276.02	5.1422725	0	0	7.78	6.82	8.8	277.67	257.65	292.75
OsR498G0714620200.01	6.723	0.553	-3.604529	2.11E-142	8.22E-143	6.54	6.91	6.72	0.47	0.6	0.59
OsR498G0714620600.01	4.21	18.613	2.1434925	5.78E-292	1.25E-292	4.52	3.91	4.2	18	18.07	19.77
OsR498G0714621000.01	5.373	29.946	2.4776611	0	0	4.38	5.72	6.02	29.96	30.55	29.33
OsR498G0714621700.01	0.073	0.866	3.5351249	2.83E-24	3.14E-24	0.11	0.04	0.07	0.98	0.92	0.7
OsR498G0714623900.01	2117.22	350.59	-2.607024	0	0	2090.7	2012.8	2248.1	331.42	335.32	385.02
OsR498G0714624100.01	13.373	42.33	1.6561996	4.16E-281	9.28E-282	13.26	13.04	13.82	43.05	43.35	40.59
OsR498G0714629300.01	1.223	0.526	-1.218235	7.69E-06	1.40E-05	1.2	1.32	1.15	0.45	0.62	0.51
OsR498G0714634800.01	9.576	46.783	2.2973454	0	0	9.61	10.09	9.03	46.24	47.07	47.04
OsR498G0714641300.01	93.84	1106.6	3.5606073	0	0	92.67	91.34	97.51	1082.9	1079	1158.1
OsR498G0714644200.01	0.47	1.526	1.7520175	2.20E-16	2.85E-16	0.47	0.54	0.4	1.89	1.5	1.19
OsR498G0714647100.01	4.573	12.176	1.4114738	1.83E-57	1.30E-57	4.84	3.95	4.93	11.07	12.34	13.12
OsR498G0714647700.01	0.026	0.336	3.5891198	3.12E-11	4.64E-11	0.02	0.04	0.02	0.16	0.47	0.38
OsR498G0714651300.01	8.586	26.123	1.6318038	1.22E-188	3.84E-189	8.06	8.71	8.99	24.68	24.37	29.32
OsR498G0714653900.01	32.266	101.4	1.6513408	0	0	31.31	34.62	30.87	103.87	97.56	102.77
OsR498G0714654200.01	0.873	4.656	2.4109614	3.03E-75	1.83E-75	0.83	0.81	0.98	4.43	5.1	4.44
OsR498G0714656600.01	0.64	5.836	3.2049763	4.18E-74	2.54E-74	0.83	0.67	0.42	5.95	5.55	6.01
OsR498G0714656800.01	1.19	0.02	-5.85848	6.39E-29	6.54E-29	1.36	1.03	1.18	0	0.03	0.03
OsR498G0714657900.01	12.266	28.176	1.219975	2.15E-186	6.84E-187	12.54	12.52	11.74	29.91	28.4	26.22
OsR498G0714661100.01	0.12	0.02	-2.52809	0.0004589	0.0009285	0.13	0.04	0.19	0	0.06	0
OsR498G0714667400.01	0.02	0.453	4.5989008	2.66E-18	3.29E-18	0.04	0.02	0	0.32	0.57	0.47
OsR498G0714670300.01	3.96	1.653	-1.229279	1.88E-16	2.43E-16	3.69	4.52	3.67	1.83	1.9	1.23
OsR498G0714675900.01	0.493	1.073	1.3518114	6.22E-08	1.04E-07	0.47	0.66	0.35	1.04	0.95	1.23
OsR498G0714676400.01	0.143	0.713	2.524607	2.07E-07	3.52E-07	0.16	0.16	0.11	0.88	0.57	0.69
OsR498G0714678000.01	0.143	0.01	-4.02559	4.20E-05	7.94E-05	0.11	0.13	0.19	0	0.03	0
OsR498G0714678200.01	0.073	0.263	1.7817649	0.0003631	0.0007287	0.11	0.08	0.03	0.31	0.18	0.3
OsR498G0714682600.01	0.156	6.756	5.4286551	3.40E-195	1.04E-195	0.15	0.17	0.15	6.68	6.46	7.13
OsR498G0714685300.01	0.05	4.666	6.5842043	6.36E-69	4.04E-69	0.1	0.05	0	5	4.26	4.74
OsR498G0714691200.01	0.566	0.18	-1.569911	4.85E-07	8.38E-07	0.59	0.51	0.6	0.14	0.22	0.18
OsR498G0714695200.01	0.843	3.73	2.1625396	2.41E-38	2.14E-38	0.85	0.95	0.73	3.98	3.62	3.59
OsR498G0714696400.01	0.233	0.96	2.0240407	1.28E-23	1.43E-23	0.17	0.25	0.28	0.94	1.03	0.91

OsR498G0714699300.01	2.483	5.486	1.1253967	7.56E-23	8.59E-23	2.39	2.46	2.6	5.47	6.02	4.97
OsR498G0714699700.01	11.993	24.146	1.0113365	4.25E-74	2.59E-74	12.73	10.98	12.27	23.27	25.46	23.71
OsR498G0714700800.01	0.13	0.633	2.2963381	3.48E-09	5.53E-09	0.13	0.1	0.16	0.41	0.75	0.74
OsR498G0714702700.01	4.62	10.556	1.2066614	1.52E-45	1.23E-45	4.41	4.57	4.88	12.15	10.57	8.95
OsR498G0714703200.01	0.043	0.466	3.3249072	2.74E-20	3.27E-20	0.04	0.03	0.06	0.54	0.43	0.43
OsR498G0714708100.01	1.543	0.43	-1.85848	1.02E-12	1.45E-12	1.92	1.17	1.54	0.44	0.24	0.61
OsR498G0714708800.01	41.246	13.92	-1.543247	3.48E-203	1.03E-203	41.29	39.22	43.23	13.05	13.85	14.86
OsR498G0714709000.01	0.056	0.433	2.9286063	9.53E-07	1.67E-06	0.04	0.09	0.04	0.33	0.46	0.51
OsR498G0714712900.01	31.156	63.79	1.0240724	0	0	32.38	30.96	30.13	63.37	65.8	62.2
OsR498G0714716900.01	0.16	1.683	3.3788002	7.84E-35	7.32E-35	0.11	0.11	0.26	1.7	1.63	1.72
OsR498G0714720100.01	0.746	2.623	2.0122781	5.41E-22	6.24E-22	0.49	0.98	0.77	3.19	2.6	2.08
OsR498G0714721100.01	7.633	2.08	-1.806666	2.65E-89	1.44E-89	7.42	7.81	7.67	1.95	2.17	2.12
OsR498G0714721300.01	0.41	1.423	1.8012611	3.06E-15	4.08E-15	0.58	0.39	0.26	1.66	1.59	1.02
OsR498G0714722100.01	1.933	8.97	2.2027353	2.43E-87	1.34E-87	2.11	1.69	2	8.74	9.47	8.7
OsR498G0714723300.01	1.373	15.596	3.5054422	8.99E-294	1.93E-294	1.18	1.53	1.41	16.04	15.61	15.14
OsR498G0714723800.01	16.106	33.216	1.0436528	1.31E-95	6.79E-96	15.1	16.44	16.78	33.08	32.96	33.61
OsR498G0714726900.01	12.28	5.666	-1.081624	1.21E-19	1.46E-19	12.31	11.68	12.85	5.24	5.82	5.94
OsR498G0714734900.01	13.75	6.616	-1.061659	1.90E-48	1.48E-48	13.84	13.59	13.82	6.49	6.52	6.84
OsR498G0714736800.01	11.066	31.026	1.4824186	2.19E-159	7.92E-160	10.58	10.69	11.93	30.94	28.9	33.24
OsR498G0714737000.01	42.523	140.72	1.7250178	0	0	42.37	42.01	43.19	138.78	133.67	149.7
OsR498G0714739200.01	74.68	18.633	-1.967651	3.85E-145	1.48E-145	77.23	72.61	74.2	19.64	17.04	19.22
OsR498G0714740700.01	1.99	0.883	-1.173147	9.61E-09	1.55E-08	2.33	1.94	1.7	0.93	0.56	1.16
OsR498G0714741100.01	1.846	5.853	1.6899566	1.55E-27	1.63E-27	1.72	1.93	1.89	5.56	6.39	5.61
OsR498G0714743600.01	0.673	17.643	4.7076234	0	0	0.63	0.76	0.63	17.53	17.92	17.48
OsR498G0714744000.01	5.976	24.623	2.0400632	1.44E-111	6.72E-112	5.91	6.22	5.8	24.65	20.95	28.27
OsR498G0714746100.01	10.166	30.34	1.5748335	2.28E-113	1.06E-113	10.53	8.96	11.01	27.78	29.1	34.14
OsR498G0714748200.01	3.48	15.643	2.1456476	1.54E-145	5.89E-146	3.59	2.85	4	16.32	14.89	15.72
OsR498G0714749100.01	3.706	11.61	1.6526491	3.26E-139	1.29E-139	3.41	3.64	4.07	11.55	11.9	11.38
OsR498G0714750200.01	50.973	108.23	1.0870454	0	0	50.78	49.91	52.23	109.9	110.38	104.42
OsR498G0714754000.01	7.106	15.17	1.0542107	1.18E-19	1.43E-19	6.41	7.13	7.78	15.96	13.57	15.98
OsR498G0714755000.01	0.34	0.11	-1.717468	2.52E-05	4.72E-05	0.51	0.29	0.22	0.07	0.08	0.18
OsR498G0714756300.01	6.913	2.196	-1.596981	1.03E-42	8.65E-43	7.64	6.3	6.8	2.36	1.91	2.32
OsR498G0714756800.01	3.69	26.533	2.8337888	9.92E-283	2.20E-283	3.53	4.03	3.51	25.72	26.04	27.84
OsR498G0714759000.01	1.1	0.256	-2.203927	4.50E-12	6.51E-12	1.19	1.19	0.92	0.4	0.11	0.26
OsR498G0714762700.01	0.566	0	-4.668292	0.000152	0.0002979	0.27	0.41	1.02	0	0	0
OsR498G0714763000.01	1.396	0.04	-5.165346	2.16E-18	2.67E-18	1.97	1.3	0.92	0.06	0	0.06
OsR498G0714763500.01	0.236	0.013	-4.02559	1.47E-08	2.39E-08	0.26	0.27	0.18	0	0	0.04
OsR498G0714765100.01	11.066	40.35	1.8866134	0	0	10.72	11.79	10.69	41.93	41.98	37.14
OsR498G0714765500.01	0.7	1.9	1.4338416	0.0008308	0.0017183	0.88	0.7	0.52	2.06	1.54	2.1
OsR498G0714766900.01	0.673	8.396	3.6352642	3.16E-134	1.30E-134	0.6	0.82	0.6	8.22	7.39	9.58
OsR498G0714768100.01	0.18	0.413	1.3106933	1.79E-07	3.03E-07	0.21	0.19	0.14	0.43	0.47	0.34
OsR498G0714768800.01	3.4	1.466	-1.193895	5.96E-24	6.64E-24	3.18	3.45	3.57	1.49	1.43	1.48
OsR498G0714770500.01	12.12	30.5	1.3230462	2.89E-170	9.85E-171	11.63	12.44	12.29	28.74	31.07	31.69
OsR498G0714770800.01	7.886	20.503	1.3686269	4.95E-82	2.85E-82	7.78	7.97	7.91	21.88	20.36	19.27
OsR498G0714771100.01	18.253	57.606	1.6566697	3.71E-123	1.61E-123	17.55	18.16	19.05	59.86	56.23	56.73
OsR498G0714772900.01	0.176	1.266	2.8517274	2.46E-28	2.55E-28	0.12	0.14	0.27	1.16	1.49	1.15
OsR498G0714773800.01	1.38	16.326	3.5675003	6.64E-138	2.66E-138	1.62	1.26	1.26	16.34	17.56	15.08
OsR498G0714775300.01	15.08	71.713	2.2400892	0	0	14.93	13.44	16.87	72.86	71.75	70.53
OsR498G0714775600.01	8.683	35.7	2.0307029	0	0	8.52	8.96	8.57	36.08	35.72	35.3
OsR498G0714777700.01	0.036	0.203	2.4769103	0.0005775	0.0011774	0	0	0.11	0.39	0.07	0.15
OsR498G0714778600.01	7.973	29.803	1.9020298	2.76E-105	1.34E-105	8.04	8.15	7.73	31.49	28.96	28.96
OsR498G0714783700.01	0.026	0.633	4.5891198	1.03E-12	1.47E-12	0	0	0.08	0.54	0.73	0.63
OsR498G0714785000.01	22.126	76.34	1.7820336	0	0	23.29	22.02	21.07	75.13	79.33	74.56
OsR498G0714789100.01	0.72	2.656	1.8813006	1.02E-14	1.37E-14	0.36	0.84	0.96	2.46	2.92	2.59
OsR498G0714790400.01	0.543	1.136	1.0610643	8.29E-11	1.25E-10	0.47	0.49	0.67	1.29	1.15	0.97
OsR498G0714797800.01	0.146	1.046	2.8488791	1.12E-08	1.82E-08	0.44	0	0	0.92	0.7	1.52
OsR498G0714798400.01	0.226	0.663	1.5593725	8.67E-05	0.0001674	0.24	0.1	0.34	0.99	0.42	0.58
OsR498G0714804800.01	86.216	35.716	-1.267979	0	0	89.47	84.46	84.72	34.95	35.98	36.22
OsR498G0714805900.01	2.426	7.543	1.6347543	1.92E-23	2.16E-23	2.61	2.67	2	7.35	6.91	8.37
OsR498G0714808300.01	1.653	9.493	2.5221614	1.18E-59	8.16E-60	1.73	2.19	1.04	8.8	9.56	10.12
OsR498G0714808700.01	2.743	9.3	1.7364665	2.01E-51	1.52E-51	2.78	1.85	3.6	10.02	8.66	9.22
OsR498G0714811700.01	2.37	6.49	1.3598771	1.14E-31	1.12E-31	2.01	2.22	2.88	5.94	5.43	8.1
OsR498G0714817700.01	1.52	0.03	-5.883571	3.81E-43	3.18E-43	1.42	1.67	1.47	0.03	0.03	0.03
OsR498G0714820100.01	0.31	8.716	4.8173888	2.34E-196	7.14E-197	0.28	0.25	0.4	9.65	8.53	7.97
OsR498G0714820500.01	12.403	69.236	2.4799839	0	0	11.36	12.61	13.24	69.89	69.2	68.62
OsR498G0714821100.01	0.026	5.88	7.8631532	8.85E-151	3.30E-151	0	0.03	0.05	5.98	5.76	5.9
OsR498G0714822200.01	0.44	0.99	1.4719096	3.01E-08	4.95E-08	0.51	0.62	0.19	0.89	1.03	1.05
OsR498G0714824300.01	1.186	8.263	2.8116805	1.48E-84	8.38E-85	1.08	1.12	1.36	8.67	8.62	7.5
OsR498G0714827200.01	2.88	0.64	-2.163736	3.78E-54	2.77E-54	2.71	3.06	2.87	0.77	0.69	0.46

OsR498G0714835900.01	30.02	60.906	1.0183298	4.61E-145	1.77E-145	28.49	29.63	31.94	61.34	60.78	60.6
OsR498G0714836200.01	0.02	0.463	4.7292975	7.57E-08	1.26E-07	0	0.06	0	0.6	0.31	0.48
OsR498G0714836300.01	0.116	0.453	1.9484147	2.86E-07	4.89E-07	0.2	0.05	0.1	0.37	0.63	0.36
OsR498G0714840200.01	1.5	3.9	1.4149825	2.36E-22	2.71E-22	1.68	1.29	1.53	3.83	4.28	3.59
OsR498G0714840300.01	16.63	57.173	1.7590723	1.91E-144	7.39E-145	14.39	18.46	17.04	53.24	55.76	62.52
OsR498G0714844300.01	0.176	0.026	-2.762556	8.51E-05	0.0001642	0.13	0.21	0.19	0.03	0.05	0
OsR498G0714845800.01	0	0.153	5.0618728	1.23E-05	2.27E-05	0	0	0	0.24	0.03	0.19
OsR498G0714846000.01	0.983	2.063	1.0695672	1.55E-07	2.62E-07	0.99	1.11	0.85	1.74	2.08	2.37
OsR498G0714848100.01	6.796	21.943	1.6908877	1.04E-189	3.26E-190	7.23	6.94	6.22	23.33	20.99	21.51
OsR498G0714848300.01	0.183	2.916	4.0739456	4.52E-52	3.40E-52	0.25	0.11	0.19	2.71	3.15	2.89
OsR498G0714849100.01	1.073	5.64	2.3910567	4.69E-79	2.76E-79	1.19	0.97	1.06	5.36	6.04	5.52
OsR498G0714852800.01	0.316	0.056	-2.440628	0.0007945	0.00164	0.12	0.59	0.24	0	0.17	0
OsR498G0714853900.01	55.363	138.79	1.3327018	0	0	53.94	57.31	54.84	140.52	140.58	135.27
OsR498G0714854200.01	0.88	0.213	-1.973123	2.32E-07	3.95E-07	1.16	0.88	0.6	0.24	0.15	0.25
OsR498G0714855100.01	0.24	1.04	2.0915935	8.73E-33	8.40E-33	0.28	0.23	0.21	1.1	0.97	1.05
OsR498G0714858600.01	61.196	28.58	-1.099204	2.59E-249	6.42E-250	59.66	63.76	60.17	27.9	28.61	29.23
OsR498G0714863800.01	0.61	2.443	1.9004094	1.51E-16	1.95E-16	0.94	0.39	0.5	1.96	2.41	2.96
OsR498G0714865400.01	4.293	2.363	-1.049838	1.68E-11	2.47E-11	3.61	4.88	4.39	2.45	2.78	1.86
OsR498G0714872500.01	0.09	0.296	1.7520175	0.0009565	0.0019907	0.15	0.04	0.08	0.26	0.4	0.23
OsR498G0714874300.01	0.15	0.446	1.5807865	2.36E-07	4.02E-07	0.18	0.13	0.14	0.56	0.38	0.4
OsR498G0714875900.01	0.326	3.453	3.4105091	1.17E-103	5.71E-104	0.35	0.32	0.31	3.33	3.35	3.68
OsR498G0714876300.01	0.79	26.32	5.0612487	0	0	0.6	0.8	0.97	26.74	28.05	24.17
OsR498G0714876800.01	1.233	2.65	1.0080333	3.41E-07	5.86E-07	1.09	0.9	1.71	2.38	2.92	2.65
OsR498G0714879900.01	6.733	23.97	1.8362984	6.93E-181	2.26E-181	6.68	6.87	6.65	25.03	23.64	23.24
OsR498G0714880400.01	9.99	3.173	-1.663157	8.17E-98	4.17E-98	9.92	9.4	10.65	3.32	3.12	3.08
OsR498G0714881300.01	0.826	1.68	1.0363377	4.73E-11	7.07E-11	0.96	0.69	0.83	1.71	1.8	1.53
OsR498G0714881900.01	0.823	3.66	2.144335	2.87E-24	3.18E-24	0.96	0.95	0.56	4.01	2.96	4.01
OsR498G0714882800.01	1.573	3.903	1.3272476	6.13E-25	6.71E-25	1.66	1.5	1.56	3.83	3.66	4.22
OsR498G0714887400.01	1.956	4.963	1.3561819	3.24E-28	3.36E-28	2.05	1.82	2	4.62	4.52	5.75
OsR498G0714888000.01	552.423	254.33	-1.125861	0	0	561.55	551.31	544.41	257.96	264.85	240.19
OsR498G0714888800.01	0.343	0.033	-3.536552	7.51E-13	1.06E-12	0.27	0.33	0.43	0.04	0.02	0.04
OsR498G0714890600.01	15.243	32.233	1.065344	3.49E-67	2.25E-67	15.43	16.08	14.22	33.66	29.95	33.09
OsR498G0714891600.01	0.176	1.176	2.7187911	1.47E-13	2.04E-13	0.32	0.12	0.09	1.37	0.93	1.23
OsR498G0714892500.01	5.7	13.42	1.2452639	1.43E-36	1.30E-36	5.74	6.05	5.31	13.9	13.93	12.43
OsR498G0714893500.01	0.133	0.716	2.5218978	2.92E-12	4.21E-12	0.11	0.19	0.1	0.63	0.74	0.78
OsR498G0714894200.01	0.766	4.113	2.4598368	3.60E-54	2.64E-54	0.82	0.84	0.64	4.97	3.9	3.47
OsR498G0714894900.01	3.703	11.13	1.5812793	2.63E-71	1.64E-71	3.77	3.65	3.69	10.6	11.04	11.75
OsR498G0714899700.01	0.486	1.353	1.4891664	4.65E-11	6.95E-11	0.45	0.28	0.73	1.47	1.61	0.98
OsR498G0714900500.01	39.566	103.29	1.3823804	0	0	39.86	40.42	38.42	100.86	104.9	104.11
OsR498G0714900900.01	4.07	12.08	1.5836829	3.72E-60	2.57E-60	4.62	4.22	3.37	12.69	11.84	11.71
OsR498G0714901400.01	0.203	0.453	1.1859141	0.0008569	0.0017748	0.16	0.13	0.32	0.55	0.4	0.41
OsR498G0714904000.01	0.803	0.21	-1.964189	6.83E-09	1.10E-08	0.84	0.77	0.8	0.31	0.17	0.15
OsR498G0714905900.01	1.803	0.233	-2.957572	7.80E-26	8.43E-26	1.54	1.56	2.31	0.18	0.33	0.19
OsR498G0714913200.01	1.816	0.733	-1.695441	0.0001192	0.0002323	2.2	1.61	1.64	1.39	0.26	0.55
OsR498G0714919600.01	0.323	0.66	1.0268774	0.0008219	0.001698	0.22	0.26	0.49	0.55	0.6	0.83
OsR498G0714925900.01	3.483	8.043	1.2068211	3.52E-59	2.46E-59	3.25	3.59	3.61	7.99	7.89	8.25
OsR498G0714931100.01	1.406	9.366	2.7309157	1.27E-68	8.07E-69	1.67	1.59	0.96	9.31	8.02	10.77
OsR498G0714931500.01	1.823	0.2	-2.815667	6.68E-15	8.98E-15	2.18	1.66	1.63	0.24	0.04	0.32
OsR498G0714931700.01	48.246	13.096	-1.793964	2.31E-131	9.63E-132	56.16	44.81	43.77	12.36	12.6	14.33
OsR498G0714932400.01	0.363	0.926	1.3529216	2.24E-06	3.99E-06	0.22	0.4	0.47	0.81	0.99	0.98
OsR498G0714934800.01	0.586	0.1	-3.007443	4.12E-15	5.50E-15	0.68	0.47	0.61	0.07	0.06	0.17
OsR498G0714938800.01	25.72	8.36	-1.634172	1.15E-178	3.79E-179	25.09	26.21	25.86	8.51	8.41	8.16
OsR498G0714939200.01	1.45	0.906	-1.02559	8.88E-06	1.63E-05	1.29	1.61	1.45	1.4	0.79	0.53
OsR498G0714939800.01	1270.36	544.56	-1.223725	0	0	1257.4	1260.9	1292.9	542.99	527.13	563.57
OsR498G0714940500.01	3.126	51.483	4.0860287	0	0	3.86	2.6	2.92	54.21	53.07	47.17
OsR498G0714941700.01	18.913	6.616	-1.55112	2.71E-144	1.05E-144	20.05	17.56	19.13	6.37	7.04	6.44
OsR498G0714941900.01	12.673	60.41	2.2517354	5.67E-304	1.19E-304	13.4	13.58	11.04	60.07	59.53	61.63
OsR498G0714942400.01	0.126	0.286	1.3765084	0.0006301	0.0012877	0.12	0.12	0.14	0.3	0.32	0.24
OsR498G0714944100.01	3.256	20.496	2.6637945	5.40E-125	2.33E-125	3.13	3.46	3.18	18.78	19.18	23.53
OsR498G0714947000.01	0.3	1.716	2.5104629	1.06E-05	1.96E-05	0.18	0.18	0.54	2.83	1.06	1.26
OsR498G0714949300.01	0.033	0.416	3.6748497	2.00E-09	3.14E-09	0.03	0	0.07	0.4	0.48	0.37
OsR498G0714950500.01	22.056	98.963	2.1299529	0	0	22.95	20.81	22.41	99.58	98.02	99.29
OsR498G0714950900.01	1.53	3	1.0484105	2.61E-11	3.87E-11	1.07	1.89	1.63	3.27	2.29	3.44
OsR498G0714951100.01	9.273	3.326	-1.481473	5.52E-44	4.54E-44	9.23	9	9.59	3.34	2.93	3.71
OsR498G0714952500.01	0.25	2.166	3.1314537	4.39E-40	3.82E-40	0.33	0.27	0.15	2.03	2.71	1.76
OsR498G0714955800.01	162.073	546.92	1.7542013	0	0	160.03	166.89	159.3	559.51	563.24	518.02
OsR498G0714959300.01	1.513	21.49	3.8157122	0	0	1.47	1.6	1.47	22.13	19.63	22.71
OsR498G0714959600.01	0.206	1.063	2.3631071	3.22E-27	3.41E-27	0.25	0.26	0.11	1.02	1.19	0.98
OsR498G0714961800.01	0.933	3.526	1.9130094	9.71E-47	7.74E-47	1.08	1	0.72	3.49	3.52	3.57

OsR498G0714963400.01	1.35	6.776	2.3746505	3.30E-101	1.65E-101	1.08	1.53	1.44	6.96	6.55	6.82
OsR498G0714964200.01	6.036	13.52	1.1649517	1.50E-43	1.24E-43	5.58	6.83	5.7	14.74	13.39	12.43
OsR498G0714966300.01	33.496	11.34	-1.572965	1.51E-150	5.65E-151	31.69	35.15	33.65	10.95	11.01	12.06
OsR498G0714967300.01	0.306	0.016	-4.273518	6.73E-06	1.22E-05	0.35	0.27	0.3	0.05	0	0
OsR498G0714968400.01	15.61	4.043	-1.949886	1.26E-294	2.70E-295	15.19	15.16	16.48	3.88	3.8	4.45
OsR498G0714973000.01	0.75	5.016	2.7421378	2.98E-61	2.04E-61	0.64	0.88	0.73	4.98	5.26	4.81
OsR498G0714973300.01	2.563	9.716	1.9119238	1.05E-109	4.93E-110	2.51	2.75	2.43	9.76	10.17	9.22
OsR498G0714974500.01	0.16	81.4	9.153188	0	0	0.19	0.17	0.12	80.59	77.07	86.54
OsR498G0714976300.01	534.023	175.03	-1.617195	0	0	534.33	515.68	552.06	170.96	166.5	187.62
OsR498G0714978600.01	7.683	84.843	3.4840978	0	0	7.38	7.9	7.77	85.38	85.51	83.64
OsR498G0714982400.01	14.436	5.963	-1.261629	3.54E-63	2.38E-63	14.58	14.04	14.69	6.33	6.01	5.55
OsR498G0714983100.01	2.15	17.206	2.9989712	2.44E-245	6.16E-246	2.1	2.33	2.02	15.86	17.45	18.31
OsR498G0714987100.01	2.006	0.753	-1.503637	4.42E-10	6.81E-10	1.93	2.11	1.98	0.91	0.44	0.91
OsR498G0714989100.01	4.183	0.39	-3.420636	2.08E-107	9.93E-108	4.63	4.38	3.54	0.26	0.53	0.38
OsR498G0714989400.01	4.18	0.4	-3.381105	2.44E-20	2.91E-20	3.47	4.69	4.38	0.39	0.56	0.25
OsR498G0714990300.01	0.64	2.09	1.7113756	7.16E-12	1.04E-11	0.59	0.64	0.69	2.23	2.17	1.87
OsR498G0714991100.01	0.326	4.723	3.8521542	6.69E-91	3.59E-91	0.23	0.46	0.29	5.32	4.16	4.69
OsR498G0714991900.01	0.2	0.633	1.4338416	2.76E-05	5.18E-05	0.16	0.26	0.18	0.92	0.38	0.6
OsR498G0714992200.01	1.98	6.376	1.6320677	9.99E-34	9.48E-34	2.18	2.03	1.73	6.1	6.82	6.21
OsR498G0714992400.01	57.99	136.79	1.2374918	0	0	56.14	58.89	58.94	136.87	139.78	133.73
OsR498G0714992800.01	0	0.793	5.4338416	7.08E-07	1.23E-06	0	0	0	0.75	0.75	0.88
OsR498G0714992900.01	15.676	46.5	1.5374486	2.55E-231	6.74E-232	15.84	15.25	15.94	47.48	44.82	47.2
OsR498G0714994000.01	0.343	1.443	2.0720208	2.01E-13	2.80E-13	0.25	0.57	0.21	1.72	0.96	1.65
OsR498G0714994900.01	1.843	4.323	1.3020593	4.92E-23	5.57E-23	1.51	2.04	1.98	4.26	4.24	4.47
OsR498G0714995400.01	0.166	1.126	2.7134956	1.24E-35	1.15E-35	0.14	0.12	0.24	1.26	0.87	1.25
OsR498G0714995900.01	0.066	0.466	2.8224069	2.37E-07	4.04E-07	0.08	0.08	0.04	0.58	0.46	0.36
OsR498G0714996300.01	1.32	3.58	1.4567107	2.03E-32	1.97E-32	1.38	1.18	1.4	3.65	3.53	3.56
OsR498G0714999400.01	2.13	6.103	1.5202247	2.17E-81	1.26E-81	2.33	2.15	1.91	6.07	6.49	5.75
OsR498G0715004000.01	10.636	21.743	1.0250662	4.34E-132	1.80E-132	11.28	10.55	10.08	23.3	21.14	20.79
OsR498G0715004900.01	1.236	0.403	-1.610553	1.91E-06	3.38E-06	0.75	1.85	1.11	0.48	0.36	0.37
OsR498G0715005400.01	0.343	0.836	1.2750694	3.12E-07	5.35E-07	0.38	0.25	0.4	0.81	0.69	1.01
OsR498G0715007300.01	0.983	0.463	-1.079029	2.15E-06	3.81E-06	1.32	0.99	0.64	0.47	0.6	0.32
OsR498G0715007500.01	108.89	16.923	-2.674902	0	0	108.14	110.39	108.14	17.22	17.43	16.12
OsR498G0715008400.01	2.423	5.36	1.019991	1.81E-33	1.73E-33	2.56	2.22	2.49	5.17	5.84	5.07
OsR498G0715009300.01	2.34	0.253	-3.273518	8.58E-71	5.37E-71	2.39	2.04	2.59	0.14	0.45	0.17
OsR498G0715011100.01	1.713	0.826	-1.052062	0.0006682	0.0013693	1.69	1.86	1.59	0.73	0.91	0.84
OsR498G0715011900.01	0.923	3.006	1.6555011	1.03E-47	8.11E-48	0.84	1	0.93	2.83	3.09	3.1
OsR498G0715013300.01	9.103	19.36	1.2096632	3.72E-186	1.18E-186	9.23	8.78	9.3	18.92	20.27	18.89
OsR498G0715013600.01	2.136	12.286	2.5278001	4.18E-185	1.33E-185	2.11	1.67	2.63	12.06	11.5	13.3
OsR498G0715015100.01	10.723	36.5	1.7664782	1.27E-268	2.94E-269	9.7	11.12	11.35	37.1	35.96	36.44
OsR498G0715020600.01	3.006	12.38	2.0574254	0	0	3.19	3.13	2.7	12.13	13.32	11.69
OsR498G0815025000.01	0.896	0.243	-1.888087	1.22E-05	2.25E-05	0.88	1.14	0.67	0.2	0.26	0.27
OsR498G0815026400.01	148.05	40.696	-1.864437	0	0	145.23	143.51	155.41	38.74	41.01	42.34
OsR498G0815026700.01	1.236	2.933	1.2435966	1.20E-06	2.11E-06	1.24	0.76	1.71	2.42	3.52	2.86
OsR498G0815026900.01	21.8	10.893	-1.002182	3.87E-36	3.55E-36	20.57	21.68	23.15	10.3	11.09	11.29
OsR498G0815027200.01	17.333	41.116	1.2391499	2.00E-255	4.81E-256	17.85	16.6	17.55	41.83	40.56	40.96
OsR498G0815028600.01	0.146	0.006	-3.932481	4.90E-08	8.12E-08	0.13	0.19	0.12	0.01	0	0.01
OsR498G0815028800.01	4.336	0.376	-3.537489	2.07E-61	1.41E-61	3.74	4.54	4.73	0.37	0.21	0.55
OsR498G0815033400.01	28.603	88.54	1.6263007	0	0	28.08	28.19	29.54	92.12	86.22	87.28
OsR498G0815033900.01	68.936	167.5	1.2960737	0	0	70.34	69.32	67.15	164.79	167.91	169.8
OsR498G0815035700.01	2.056	5.38	1.3941468	1.62E-103	7.97E-104	2.03	2.14	2	5.44	5.38	5.32
OsR498G0815037400.01	7.306	25.823	1.8214216	1.81E-215	5.06E-216	6.5	7.81	7.61	26.64	27.01	23.82
OsR498G0815038000.01	1.356	4.216	1.6350149	8.25E-42	7.01E-42	1.5	1.23	1.34	4.48	3.68	4.49
OsR498G0815040500.01	0.16	0.673	2.1068603	4.32E-08	7.13E-08	0.15	0.22	0.11	0.53	0.77	0.72
OsR498G0815041800.01	20.8	53.413	1.3634522	3.50E-285	7.74E-286	20.12	21.28	21	54.17	51.32	54.75
OsR498G0815042400.01	6.186	18.723	1.5988384	3.63E-148	1.37E-148	5.94	6.98	5.64	17.15	20.08	18.94
OsR498G0815042500.01	12.073	29.566	1.2923633	7.66E-132	3.18E-132	12.17	11.48	12.57	28.77	29.45	30.48
OsR498G0815044600.01	1.71	0.843	-1.02559	3.88E-05	7.33E-05	2.05	1.39	1.69	0.57	1.08	0.88
OsR498G0815045300.01	2.193	12.48	2.4786237	1.75E-228	4.66E-229	2.02	2.14	2.42	12.74	11.86	12.84
OsR498G0815049400.01	314.94	155.16	-1.021396	0	0	321.8	312.57	310.45	155.09	155.24	155.16
OsR498G0815054900.01	0.353	0.756	1.1011671	2.46E-06	4.38E-06	0.24	0.3	0.52	0.76	0.82	0.69
OsR498G0815055200.01	7.76	16.916	1.0770631	5.25E-62	3.56E-62	7.31	8.26	7.71	16.92	17.63	16.2
OsR498G0815055900.01	11.75	25.696	1.1288837	5.53E-187	1.75E-187	11.74	12.2	11.31	24.45	26.95	25.69
OsR498G0815056400.01	0.153	3.313	4.4118153	4.93E-16	6.44E-16	0	0.31	0.15	3.35	3.79	2.8
OsR498G0815056600.01	6.336	15.713	1.3164641	1.43E-157	5.19E-158	6.85	5.72	6.44	15.93	15.97	15.24
OsR498G0815058200.01	0.02	1.476	6.103693	2.08E-34	1.95E-34	0.03	0.03	0	1.25	1.44	1.74
OsR498G0815060600.01	0.626	1.343	1.1063216	1.05E-10	1.59E-10	0.88	0.5	0.5	1.48	1.39	1.16
OsR498G0815061000.01	5.37	15.526	1.5120485	3.16E-180	1.03E-180	5.42	5.33	5.36	15.69	15.89	15
OsR498G0815062700.01	1.226	8.263	2.7489749	1.23E-93	6.45E-94	0.87	1.69	1.12	7.55	8.85	8.39

OsR498G0815063200.01	4.573	15.476	1.7716827	1.84E-233	4.82E-234	4.49	4.55	4.68	15.84	16.24	14.35
OsR498G0815064300.01	33.323	14.44	-1.143771	1.50E-195	4.57E-196	31.49	33.96	34.52	14.23	14.99	14.1
OsR498G0815073900.01	0.6	0.106	-2.586305	2.72E-10	4.17E-10	0.47	0.61	0.72	0.11	0.06	0.15
OsR498G0815077300.01	35.123	169.33	2.2841191	0	0	36.85	34.78	33.74	161.1	161.77	185.13
OsR498G0815078600.01	2.083	8.173	1.9676314	2.36E-66	1.53E-66	1.96	2.12	2.17	7.97	8.38	8.17
OsR498G0815079400.01	13.74	55.28	2.0089173	5.38E-257	1.29E-257	15.62	12.62	12.98	52.03	53.29	60.52
OsR498G0815079600.01	16.203	79.266	2.2868622	0	0	14.59	15.96	18.06	78.19	79.16	80.45
OsR498G0815079800.01	16.143	33.813	1.066969	2.49E-101	1.24E-101	15.38	16.02	17.03	32.76	32.9	35.78
OsR498G0815080800.01	16.693	35.533	1.0543663	2.07E-99	1.04E-99	16.22	16	17.86	35.54	36.73	34.33
OsR498G0815081500.01	0.786	3.316	2.0366942	5.85E-32	5.71E-32	0.91	0.62	0.83	3.35	2.96	3.64
OsR498G0815084900.01	67.926	29.506	-1.182166	5.91E-187	1.87E-187	70.15	67.24	66.39	31.21	28.82	28.49
OsR498G0815087200.01	279.303	49.91	-2.489079	0	0	292.09	262.7	283.12	48.41	46.81	54.51
OsR498G0815087400.01	48.233	175.48	1.8879441	0	0	48.91	44.03	51.76	172.76	177.38	176.3
OsR498G0815090000.01	0.68	4.006	2.5504393	1.25E-46	9.94E-47	0.72	0.75	0.57	3.5	3.93	4.59
OsR498G0815103200.01	0	0.14	4.2963381	0.0007942	0.0016388	0	0	0	0.21	0.08	0.13
OsR498G0815103300.01	3.76	1.12	-1.756672	2.47E-52	1.85E-52	3.58	3.7	4	1.12	1.05	1.19
OsR498G0815104300.01	25.383	419.12	4.0446314	0	0	24.45	24.94	26.76	415.01	418.09	424.26
OsR498G0815105100.01	3.93	9.076	1.2148429	1.01E-24	1.11E-24	3.65	3.62	4.52	8.69	8.54	10
OsR498G0815105400.01	11.966	34.496	1.5138467	2.08E-172	7.00E-173	12.3	12.13	11.47	36.24	33.14	34.11
OsR498G0815107500.01	29.266	70.783	1.2710458	1.49E-91	7.95E-92	31.01	27.79	29	71.71	70.6	70.04
OsR498G0815110000.01	5.823	12.616	1.1047318	2.68E-24	2.96E-24	5.94	5.97	5.56	11.03	11.87	14.95
OsR498G0815110600.01	6.066	12.706	1.0754715	7.33E-41	6.31E-41	5.98	7.26	4.96	13.07	11.94	13.11
OsR498G0815111500.01	32.09	127.11	1.9803601	0	0	31.63	32.76	31.88	127.89	126.87	126.56
OsR498G0815112300.01	1.856	5.373	1.6468353	4.41E-22	5.08E-22	2.02	1.51	2.04	5.81	4.9	5.41
OsR498G0815112800.01	3.066	0.823	-1.900059	5.25E-17	6.72E-17	3	2.26	3.94	0.64	0.99	0.84
OsR498G0815115800.01	0.096	0.633	2.7399447	4.82E-12	6.99E-12	0.09	0.14	0.06	0.61	0.66	0.63
OsR498G0815116200.01	35.523	213.52	2.5837098	0	0	33.22	36.8	36.55	208.05	214.63	217.87
OsR498G0815116400.01	11.76	28.126	1.2296638	2.32E-231	6.15E-232	11.62	12.11	11.55	28.97	28.64	26.77
OsR498G0815116500.01	0.373	2.37	2.6598593	3.41E-13	4.79E-13	0.83	0.29	0	2.39	1.27	3.45
OsR498G0815117500.01	0.296	1.493	2.3311035	3.44E-28	3.57E-28	0.33	0.32	0.24	1.65	1.5	1.33
OsR498G0815117900.01	0.126	0.826	2.4931388	4.67E-22	5.39E-22	0.18	0.09	0.11	0.7	0.89	0.89
OsR498G0815123300.01	7.06	15.156	1.0866602	2.33E-67	1.50E-67	7.28	7.02	6.88	15.34	15.68	14.45
OsR498G0815124300.01	3.91	37.23	3.0677723	0	0	3.73	3.86	4.14	37.38	36.59	37.72
OsR498G0815126300.01	1.863	8.17	2.1156441	2.34E-79	1.37E-79	2.02	1.8	1.77	8.13	7.95	8.43
OsR498G0815126500.01	0.52	2.433	2.266933	9.12E-44	7.53E-44	0.58	0.53	0.45	2.28	2.16	2.86
OsR498G0815131100.01	11.653	27.173	1.2574871	2.12E-108	1.01E-108	12.51	11.89	10.56	28.04	25.95	27.53
OsR498G0815131800.01	2.426	7.09	1.5421827	5.72E-44	4.71E-44	2.24	2.76	2.28	7.2	7.82	6.25
OsR498G0815132200.01	0.436	2.753	2.7430844	3.24E-57	2.31E-57	0.39	0.58	0.34	2.78	2.9	2.58
OsR498G0815132700.01	0.023	0.52	4.5719914	1.00E-10	1.51E-10	0	0.07	0	0.26	0.72	0.58
OsR498G0815137300.01	0.583	10.96	4.2317978	6.61E-100	3.33E-100	0.59	0.51	0.65	11.66	10.27	10.95
OsR498G0815137500.01	0.16	1.803	3.4889831	6.92E-17	8.88E-17	0	0.34	0.14	2.28	1.27	1.86
OsR498G0815140400.01	5.466	17.62	1.6837242	7.14E-102	3.54E-102	5.55	5.41	5.44	17.46	17.79	17.61
OsR498G0815140600.01	21.986	10.696	-1.028546	4.79E-78	2.84E-78	20.84	23.39	21.73	10.75	10.64	10.7
OsR498G0815142800.01	5.35	0.01	-9.088985	1.57E-101	7.80E-102	5.28	5.37	5.4	0.03	0	0
OsR498G0815145300.01	0	0.183	5.144335	6.91E-06	1.26E-05	0	0	0	0.21	0.09	0.25
OsR498G0815148400.01	1.74	8.546	2.3212127	4.66E-133	1.92E-133	1.66	1.68	1.88	8.11	8.65	8.88
OsR498G0815152700.01	0.54	0.123	-2.125126	3.89E-05	7.34E-05	0.16	0.81	0.65	0.16	0.1	0.11
OsR498G0815153900.01	8.563	3.17	-1.435493	7.14E-57	5.10E-57	8.31	8.74	8.64	2.51	3.6	3.4
OsR498G0815163200.01	3.873	12.176	1.6541507	3.23E-112	1.50E-112	4.11	3.63	3.88	12.4	12.24	11.89
OsR498G0815167000.01	18.536	2.006	-3.109785	1.31E-154	4.83E-155	18.37	19.18	18.06	2.21	2.33	1.48
OsR498G0815185200.01	6.113	25.44	2.0442255	9.99E-216	2.79E-216	6.35	5.02	6.97	25.03	25.25	26.04
OsR498G0815191400.01	0.41	1.266	1.662466	3.85E-11	5.74E-11	0.42	0.28	0.53	0.95	1.71	1.14
OsR498G0815196000.01	1.31	2.313	1.0888093	3.39E-11	5.04E-11	1.45	1.39	1.09	2.22	2.1	2.62
OsR498G0815197100.01	2.723	33.026	3.4120543	0	0	2.53	2.79	2.85	32.83	30.39	35.86
OsR498G0815197900.01	1.99	6.883	1.772427	2.97E-49	2.30E-49	1.93	2.21	1.83	6.97	6.71	6.97
OsR498G0815199900.01	6.01	28.526	2.242024	4.24E-145	1.63E-145	5.41	7.42	5.2	26.18	29.72	29.68
OsR498G0815205300.01	0.296	0.073	-2.273518	0.0004936	0.0010012	0.22	0.22	0.45	0.11	0	0.11
OsR498G0815208700.01	0.886	2.713	1.6118399	4.45E-19	5.44E-19	0.78	0.88	1	3.1	2.4	2.64
OsR498G0815209400.01	2.863	1.086	-1.396029	1.94E-33	1.85E-33	3.03	2.82	2.74	1.07	1.15	1.04
OsR498G0815210500.01	14.936	30.783	1.0420751	3.42E-70	2.15E-70	14.63	15.29	14.89	29.67	28.13	34.55
OsR498G0815212200.01	3.886	12.806	1.7158249	1.32E-74	7.98E-75	3.7	3.93	4.03	12.67	12.62	13.13
OsR498G0815220500.01	0.146	0.72	2.2963381	0.0003791	0.0007624	0.11	0.11	0.22	0.64	0.75	0.77
OsR498G0815220700.01	0.036	0.64	4.0745466	2.58E-05	4.82E-05	0	0.11	0	0.72	0.78	0.42
OsR498G0815224900.01	10.153	55.383	2.442246	2.04E-286	4.48E-287	10.37	9.44	10.65	53.51	53.3	59.34
OsR498G0815226800.01	6.583	2.42	-1.44423	1.43E-28	1.47E-28	6.13	6.56	7.06	2.16	2.44	2.66
OsR498G0815228600.01	27.403	77.063	1.462491	0	0	26.06	26.16	29.99	77.93	73.41	79.85
OsR498G0815231500.01	2363.46	1172.4	-1.036737	0	0	2401.7	2363.7	2325	1176.4	1213.1	1127.7
OsR498G0815233600.01	25.336	52.44	1.0500293	0	0	26.08	25.86	24.07	53.95	52.1	51.27
OsR498G0815235000.01	21.6	57.786	1.419163	0	0	20.75	23.04	21.01	57.69	58.51	57.16

OsR498G0815235900.01	7.54	21.073	1.4929656	7.03E-123	3.05E-123	8.19	7.3	7.13	21.65	20.95	20.62
OsR498G0815236600.01	18.346	51.453	1.5056846	0	0	18.27	18.33	18.44	53.35	50.93	50.08
OsR498G0815236700.01	4.043	8.743	1.1123311	5.93E-32	5.79E-32	3.77	4.19	4.17	9.07	7.87	9.29
OsR498G0815239000.01	32.826	15.936	-1.020959	9.79E-74	5.98E-74	31.91	30.96	35.61	16.99	15.56	15.26
OsR498G0815241200.01	13.513	28.633	1.0834704	4.72E-86	2.63E-86	12.93	13.22	14.39	29.68	28.26	27.96
OsR498G0815243400.01	0.95	0.296	-1.710088	3.55E-17	4.52E-17	0.93	0.83	1.09	0.19	0.31	0.39
OsR498G0815249100.01	12.056	27.36	1.1832712	4.77E-170	1.63E-170	11.89	12.88	11.4	26.71	28.8	26.57
OsR498G0815249300.01	0.04	0.526	3.7292975	4.20E-07	7.23E-07	0.06	0.06	0	0.29	0.82	0.47
OsR498G0815251300.01	39.113	89.883	1.2021556	0	0	39.14	38.15	40.05	91.32	91.6	86.73
OsR498G0815254000.01	349.96	46.1	-2.925637	0	0	360.76	348.4	340.72	50.81	44.41	43.08
OsR498G0815255900.01	110.826	47.733	-1.239481	0	0	110.24	110.98	111.26	46.56	48.58	48.06
OsR498G0815257100.01	5.043	10.556	1.067957	1.20E-46	9.57E-47	4.66	5.45	5.02	11.12	10.01	10.54
OsR498G0815275500.01	1.12	0.036	-4.979786	2.92E-16	3.80E-16	1.41	1.06	0.89	0	0.05	0.06
OsR498G0815277000.01	0.403	1.253	1.6423618	5.14E-40	4.47E-40	0.35	0.42	0.44	1.3	1.23	1.23
OsR498G0815285000.01	7.896	16.143	1.0136098	2.14E-120	9.42E-121	7.78	8.01	7.9	15.76	16.61	16.06
OsR498G0815295300.01	2.19	14.836	2.7578787	1.95E-92	1.03E-92	1.97	1.81	2.79	14.89	13.36	16.26
OsR498G0815297600.01	9.323	18.833	1.0126349	4.06E-66	2.65E-66	9.63	8.9	9.44	19.17	17.21	20.12
OsR498G0815297700.01	0.946	5.223	2.4645747	5.80E-59	4.06E-59	0.9	0.82	1.12	4.12	6.31	5.24
OsR498G0815300300.01	0.376	0.836	1.1588345	3.28E-05	6.17E-05	0.31	0.41	0.41	0.9	0.93	0.68
OsR498G0815301600.01	27.996	174.5	2.6393718	0	0	26.81	27.69	29.49	173.41	178.51	171.58
OsR498G0815304200.01	2.166	14.03	2.6937217	2.03E-217	5.64E-218	2.18	2.12	2.2	14.34	13.12	14.63
OsR498G0815307600.01	1.713	8.723	2.3521398	2.74E-95	1.42E-95	1.48	1.73	1.93	8.71	7.96	9.5
OsR498G0815310000.01	28.15	5.283	-2.390313	2.22E-253	5.38E-254	27.65	28.51	28.29	6.02	4.24	5.59
OsR498G0815310900.01	3.103	0.273	-3.599412	2.08E-153	7.67E-154	3.16	3.01	3.14	0.15	0.36	0.31
OsR498G0815318300.01	23.65	9.266	-1.346946	3.63E-53	2.70E-53	25.89	22.38	22.68	8.9	9.07	9.83
OsR498G0815328600.01	209.276	87.473	-1.2727	0	0	214.16	206.83	206.84	87.49	86.74	88.19
OsR498G0815329300.01	1.16	0.25	-2.223529	3.24E-11	4.81E-11	0.99	1.33	1.16	0.26	0.22	0.27
OsR498G0815332900.01	14.516	6.606	-1.135036	2.48E-118	1.11E-118	14.76	14.56	14.23	6.54	6.6	6.68
OsR498G0815338100.01	0.363	0.02	-4.113053	2.28E-05	4.25E-05	0.45	0.32	0.32	0	0.06	0
OsR498G0815338900.01	1.196	0.02	-5.956327	2.60E-16	3.38E-16	0.83	1.29	1.47	0	0	0.06
OsR498G0815339300.01	0.26	1.74	2.7369106	4.17E-16	5.45E-16	0.39	0.17	0.22	1.86	2.07	1.29
OsR498G0815340100.01	1.743	0.316	-2.440628	1.34E-12	1.91E-12	1.84	1.17	2.22	0.25	0.44	0.26
OsR498G0815343700.01	0.016	0.263	3.97441	5.14E-05	9.78E-05	0.05	0	0	0.15	0.64	0
OsR498G0815344500.01	0.373	0.07	-2.383142	3.87E-07	6.66E-07	0.3	0.38	0.44	0.03	0.13	0.05
OsR498G0815345500.01	0.66	2.21	1.7420916	5.83E-25	6.39E-25	0.81	0.57	0.6	2.45	2.27	1.91
OsR498G0815345700.01	0.236	0.073	-1.72603	0.0006425	0.0013145	0.16	0.19	0.36	0.03	0.03	0.16
OsR498G0815346900.01	0.8	2.166	1.4378819	1.55E-28	1.60E-28	0.91	0.55	0.94	2.15	2.01	2.34
OsR498G0815347700.01	0.55	1.58	1.5173084	9.86E-14	1.36E-13	0.72	0.63	0.3	1.73	1.72	1.29
OsR498G0815349000.01	1.153	4.29	1.8943567	6.20E-34	5.86E-34	0.98	1.21	1.27	4.81	4.18	3.88
OsR498G0815351000.01	1.483	6.26	2.0799444	1.59E-80	9.27E-81	1.25	1.46	1.74	6.53	6.29	5.96
OsR498G0815352500.01	0.796	0.27	-1.588526	9.63E-07	1.69E-06	0.8	0.74	0.85	0.22	0.3	0.29
OsR498G0815353900.01	1.453	0.596	-1.277653	4.98E-09	7.95E-09	1.31	1.42	1.63	0.58	0.68	0.53
OsR498G0815361000.01	8.093	2.64	-1.623153	3.13E-38	2.79E-38	7.92	8.26	8.1	2.66	2.86	2.4
OsR498G0815365500.01	0.153	0.36	1.2642444	0.0009327	0.001939	0.11	0.17	0.18	0.27	0.45	0.36
OsR498G0815365700.01	0.07	0.273	1.9219425	0.0002409	0.000478	0.12	0.09	0	0.24	0.24	0.34
OsR498G0815366500.01	0.03	0.366	3.4979719	1.78E-10	2.71E-10	0.02	0.02	0.05	0.38	0.4	0.32
OsR498G0815366900.01	0.01	0.283	4.9286063	7.45E-09	1.20E-08	0	0	0.03	0.22	0.24	0.39
OsR498G0815367900.01	0.056	0.416	3.0787466	4.73E-09	7.54E-09	0	0.06	0.11	0.42	0.35	0.48
OsR498G0815372600.01	2.066	0.396	-2.370726	8.60E-51	6.57E-51	2.07	2.31	1.82	0.35	0.41	0.43
OsR498G0815372700.01	1.673	3.493	1.0613933	5.08E-17	6.50E-17	1.7	1.86	1.46	3.61	3.57	3.3
OsR498G0815379800.01	0.47	20.993	5.4465366	0	0	0.49	0.48	0.44	20.43	20.43	22.12
OsR498G0815380800.01	14075.5	3310.2	-2.087741	0	0	13967	13825	14435	3271.9	3214.5	3444.2
OsR498G0815381500.01	2.126	1.036	-1.085091	3.07E-10	4.71E-10	2.03	2.27	2.08	1.16	0.91	1.04
OsR498G0815382000.01	64.033	8.823	-2.940178	0	0	63.3	65.44	63.36	8.2	9.1	9.17
OsR498G0815382600.01	0.046	1.303	4.8073	1.01E-14	1.37E-14	0	0	0.14	1.23	1.7	0.98
OsR498G0815383300.01	0.116	0.463	2.0139383	1.34E-05	2.48E-05	0.18	0	0.17	0.4	0.46	0.53
OsR498G0815386000.01	4.53	18.973	2.2237481	0	0	4.86	4.31	4.42	19.5	19.68	17.74
OsR498G0815386500.01	0.346	0.996	1.6002447	1.83E-15	2.43E-15	0.42	0.28	0.34	0.97	0.98	1.04
OsR498G0815386700.01	41.093	9.103	-2.171612	0	0	41.16	40.94	41.18	9.04	9.14	9.13
OsR498G0815387500.01	1.94	0.296	-2.712972	2.23E-54	1.63E-54	2.01	1.78	2.03	0.26	0.2	0.43
OsR498G0815388600.01	1.296	3.003	1.2133766	6.57E-18	8.21E-18	1.44	1.31	1.14	2.31	3.49	3.21
OsR498G0815390700.01	0.476	1.086	1.2071834	2.40E-11	3.55E-11	0.54	0.45	0.44	1	1.2	1.06
OsR498G0815391100.01	12.766	58.693	2.1150075	0	0	12.37	13.55	12.38	62.41	60.89	52.78
OsR498G0815393500.01	0.116	0.463	1.97441	8.24E-06	1.51E-05	0	0.17	0.18	0.38	0.48	0.53
OsR498G0815394100.01	1.68	9.726	2.4880337	1.58E-60	1.09E-60	1	1.97	2.07	8.06	7.68	13.44
OsR498G0815394600.01	2.193	5.13	1.1524628	1.76E-26	1.88E-26	2.28	2.41	1.89	4.8	5.66	4.93
OsR498G0815397300.01	0.623	1.416	1.1874037	3.66E-14	5.00E-14	0.6	0.52	0.75	1.34	1.36	1.55
OsR498G0815400000.01	9.013	31.183	1.7863376	3.46E-142	1.35E-142	8.96	8.76	9.32	32.17	30.51	30.87
OsR498G0815402700.01	1.336	3.13	1.2492041	4.89E-16	6.39E-16	1.26	1.18	1.57	3.5	2.97	2.92

OsR498G0815409100.01	0.09	0.223	1.3599442	9.82E-05	0.0001902	0.08	0.13	0.06	0.27	0.33	0.07
OsR498G0815426900.01.	0	0.326	5.2253715	3.81E-06	6.83E-06	0	0	0	0.5	0.48	0
OsR498G0815443700.01	36.583	17.523	-1.26138	8.43E-167	2.92E-167	35.49	35.18	39.08	17.39	17.46	17.72
OsR498G0815464100.01	0.44	0.146	-1.610553	5.76E-06	1.04E-05	0.44	0.54	0.34	0.24	0.1	0.1
OsR498G0815464500.01	17.39	35.04	1.0155282	5.08E-242	1.30E-242	17.19	17.82	17.16	35.68	34.73	34.71
OsR498G0815465100.01	10.896	22.046	1.0507325	1.25E-86	6.94E-87	10.54	10.91	11.24	22	21.36	22.78
OsR498G0815465600.01	0.686	3.39	2.3298906	1.17E-23	1.31E-23	0.6	0.69	0.77	3.57	3.57	3.03
OsR498G0815469000.01	2.536	6.263	1.304278	4.41E-16	5.77E-16	2.97	2.82	1.82	4.05	6.48	8.26
OsR498G0815470100.01	6.436	1.143	-2.458695	2.88E-42	2.42E-42	6.5	6.88	5.93	1.46	0.79	1.18
OsR498G0815482300.01	0.336	1.093	1.6806788	7.98E-07	1.39E-06	0.27	0.53	0.21	1	0.99	1.29
OsR498G0815489000.01	1.183	0.28	-2.072896	1.24E-08	2.02E-08	1.15	1.37	1.03	0.11	0.33	0.4
OsR498G0815490400.01	2.45	5.306	1.1770391	1.05E-08	1.70E-08	2.35	2.19	2.81	5.12	6.03	4.77
OsR498G0815490900.01	5.646	11.523	1.0233196	4.23E-20	5.07E-20	5.99	5.79	5.16	12.72	10.67	11.18
OsR498G0815498100.01	12.27	44.963	1.8728077	1.39E-270	3.19E-271	13.57	10.73	12.51	45.89	44	45
OsR498G0815506900.01	4.493	1.913	-1.233078	1.28E-18	1.58E-18	4.14	4.6	4.74	2.05	1.75	1.94
OsR498G0815509300.01	1.146	0.476	-1.149071	0.0003924	0.0007899	0.82	0.97	1.65	0.39	0.47	0.57
OsR498G0815511900.01	0.14	0.803	2.4989462	2.03E-14	2.76E-14	0.1	0.26	0.06	0.68	0.84	0.89
OsR498G0815517200.01	3.013	0.47	-2.63069	8.77E-76	5.27E-76	3.19	2.68	3.17	0.5	0.48	0.43
OsR498G0815520600.01	685.573	220.32	-1.632943	0	0	691.57	683.99	681.16	226.88	218.17	215.92
OsR498G0815521100.01	33.116	16.283	-1.018777	7.68E-148	2.91E-148	32.11	33.94	33.3	17.11	16.23	15.51
OsR498G0815523900.01	101.056	210.04	1.0609073	0	0	103.94	101.87	97.36	210.84	217.18	202.1
OsR498G0815526000.01	0.203	0.683	1.6987755	4.41E-08	7.29E-08	0.16	0.29	0.16	0.56	0.91	0.58
OsR498G0815527100.01	0.493	0.116	-2.080038	9.19E-08	1.54E-07	0.36	0.46	0.66	0.19	0.05	0.11
OsR498G0815531900.01	1.72	0.903	-1.125525	1.37E-15	1.80E-15	2.15	1.72	1.29	0.94	0.87	0.9
OsR498G0815534900.01	495.69	33.486	-3.890377	0	0	492.18	495.28	499.61	32.6	34.97	32.89
OsR498G0815535500.01	26.296	2.3	-3.6012	0	0	25.46	26.38	27.05	2.11	2.43	2.36
OsR498G0815535700.01	11.326	0.843	-3.745314	0	0	10.73	12.28	10.97	0.75	0.97	0.81
OsR498G0815539300.01	10.873	32.426	1.6082821	8.32E-257	2.00E-257	10.63	10.52	11.47	32.32	31.98	32.98
OsR498G0815539600.01	0.973	0.15	-2.714889	2.10E-21	2.46E-21	1.25	0.61	1.06	0.11	0.2	0.14
OsR498G0815545200.01	0.526	2.553	2.2731581	5.84E-23	6.62E-23	0.45	0.55	0.58	2.28	2.79	2.59
OsR498G0815558700.01	6.683	17.03	1.2713485	1.16E-129	4.86E-130	6.52	7.4	6.13	16.88	17.14	17.07
OsR498G0815561600.01	0.57	3.126	2.4483411	8.94E-22	1.04E-21	0.64	0.63	0.44	2.48	2.96	3.94
OsR498G0815571800.01	0.006	0.11	4.144335	1.55E-05	2.86E-05	0	0	0.02	0.11	0.11	0.11
OsR498G0815584300.01	60.74	23.05	-1.384868	5.58E-201	1.66E-201	59.98	64.43	57.81	23.53	21.4	24.22
OsR498G0815593200.01	26.163	4.676	-2.48432	5.49E-143	2.14E-143	26.7	27.63	24.16	4.99	4.53	4.51
OsR498G0815593600.01	114.01	7.476	-3.930569	0	0	110.77	113.81	117.45	9.23	6.84	6.36
OsR498G0815595900.01	3.486	11.123	1.6882024	6.82E-51	5.19E-51	3.59	3.55	3.32	13.33	11.1	8.94
OsR498G0815597800.01	7.16	1.596	-2.254836	3.14E-105	1.52E-105	6.49	6.85	8.14	1.64	1.66	1.49
OsR498G0815603000.01	4.166	16.393	1.97441	1.33E-57	9.43E-58	3.76	4.28	4.46	15.77	16.15	17.26
OsR498G0815612100.01	11.996	37.296	1.6510975	1.25E-200	3.73E-201	12.54	11.98	11.47	37.98	36.77	37.14
OsR498G0815613500.01	0.043	0.31	2.7817649	0.0008272	0.0017105	0	0	0.13	0.07	0.39	0.47
OsR498G0815615900.01	22.07	10.636	-1.051512	2.57E-116	1.16E-116	21.97	21.88	22.36	9.72	11.22	10.97
OsR498G0815617800.01	0.396	0.063	-2.610553	2.10E-11	3.10E-11	0.4	0.45	0.34	0.05	0.07	0.07
OsR498G0815620900.01	3.416	1.07	-1.671693	9.83E-22	1.14E-21	3.59	3.09	3.57	1.24	0.99	0.98
OsR498G0815626400.01	6.266	28.296	2.1879424	8.72E-115	3.99E-115	5.46	6.67	6.67	26.36	28.5	30.03
OsR498G0815635100.01	0.786	0.283	-1.53685	7.15E-09	1.15E-08	0.78	0.66	0.92	0.21	0.42	0.22
OsR498G0815643600.01	8.38	2.726	-1.622094	1.10E-21	1.28E-21	8.55	7.4	9.19	2.45	3.12	2.61
OsR498G0815654600.01	4.896	15.266	1.4867201	5.28E-64	3.52E-64	4.48	4.88	5.33	14.61	15.78	15.41
OsR498G0815659600.01	9.696	37.573	1.9224722	0	0	9.62	10.2	9.27	37.56	38.8	36.36
OsR498G0815661100.01	1.013	2.606	1.3603011	1.52E-13	2.11E-13	1.02	1.05	0.97	2.41	2.76	2.65
OsR498G0815685200.01	0.783	1.673	1.1652057	2.23E-13	3.11E-13	0.62	0.87	0.86	1.97	1.86	1.19
OsR498G0815686000.01	3.53	12.276	1.8042975	7.75E-156	2.84E-156	3.64	3.69	3.26	12.62	12.18	12.03
OsR498G0815694100.01	0.243	0.463	1.0898872	0.0007283	0.001498	0.25	0.12	0.36	0.36	0.47	0.56
OsR498G0815698400.01	13.53	1.93	-2.72093	5.51E-109	2.61E-109	14.48	13.38	12.73	2.15	2.02	1.62
OsR498G0815699300.01	0.033	0.526	3.9130094	3.30E-11	4.91E-11	0.07	0.03	0	0.61	0.27	0.7
OsR498G0815705100.01	2.033	0.836	-1.422313	1.55E-20	1.84E-20	2	1.9	2.2	0.72	0.77	1.02
OsR498G0815730600.01	7.743	19.513	1.3440707	6.23E-101	3.11E-101	7.7	7.39	8.14	19.89	18.16	20.49
OsR498G0815732100.01	0.23	0.04	-2.537817	6.30E-05	0.0001207	0.24	0.27	0.18	0.06	0	0.06
OsR498G0815733400.01	8.51	22.04	1.3759497	6.61E-58	4.67E-58	8.61	8.86	8.06	21.03	21.68	23.41
OsR498G0815738100.01	1.856	4.046	1.1130152	3.00E-13	4.20E-13	1.46	1.91	2.2	3.25	4.32	4.57
OsR498G0815746700.01	0.193	5.673	4.8395511	4.66E-48	3.67E-48	0.08	0.33	0.17	5.79	5.5	5.73
OsR498G0815763300.01	3.333	9.283	1.4595187	4.05E-62	2.74E-62	3.41	3.48	3.11	9.11	10.1	8.64
OsR498G0815767600.01	0.45	0.12	-1.992423	2.94E-06	5.26E-06	0.2	0.5	0.65	0.09	0.07	0.2
OsR498G0815772600.01	0.14	0.426	1.9647063	7.40E-07	1.29E-06	0.17	0.07	0.18	0.52	0.47	0.29
OsR498G0815774200.01	12.07	24.56	1.0345855	1.06E-110	4.97E-111	12.34	11.92	11.95	24.88	26.23	22.57
OsR498G0815777800.01	95.223	37.78	-1.339773	0	0	91.13	95.48	99.06	36.5	35.46	41.38
OsR498G0815779000.01	1.313	8.406	2.6541822	2.88E-248	7.16E-249	1.54	1.13	1.27	8.19	8.63	8.4
OsR498G0815785200.01	15.19	57.023	1.9089569	0	0	14.99	15.37	15.21	56.55	57.36	57.16
OsR498G0815786500.01	2.543	10.56	2.0426947	6.89E-133	2.85E-133	2.25	2.84	2.54	10.47	10.58	10.63

OsR498G0815787600.01	5.693	2.61	-1.015409	1.07E-08	1.74E-08	6.81	4.25	6.02	2.84	2.7	2.29
OsR498G0815799800.01	0.546	3.643	2.7540199	2.06E-17	2.61E-17	0.64	0.58	0.42	3.57	3.39	3.97
OsR498G0815800700.01	0.09	1.066	3.552283	1.47E-40	1.27E-40	0.1	0.11	0.06	0.97	1.11	1.12
OsR498G0815802400.01	0.376	1.22	1.6524819	7.28E-07	1.27E-06	0.46	0.28	0.39	1.11	0.95	1.6
OsR498G0815804200.01	46.29	16.036	-1.618516	0	0	47.34	45.21	46.32	16.22	16.37	15.52
OsR498G0815805400.01	0.013	0.36	4.8813006	1.33E-08	2.15E-08	0	0.04	0	0.36	0.39	0.33
OsR498G0815812500.01	0	0.19	5.2963381	2.20E-06	3.91E-06	0	0	0	0.17	0.17	0.23
OsR498G0815813300.01	9.22	62.843	2.7381015	0	0	8.63	9.48	9.55	63.64	63.75	61.14
OsR498G0815815500.01	0.186	0	-4.932481	3.36E-05	6.31E-05	0.08	0.21	0.27	0	0	0
OsR498G0815825100.01	10.42	25.233	1.2735924	2.09E-49	1.62E-49	9.15	10.35	11.76	23.79	25.98	25.93
OsR498G0815825400.01	2.543	7.996	1.6361692	7.78E-49	6.06E-49	2.22	2.84	2.57	8.27	8.57	7.15
OsR498G0815832100.01	0.523	1.583	1.6024412	1.34E-22	1.52E-22	0.63	0.51	0.43	1.41	1.79	1.55
OsR498G0815837200.01	0.433	1.25	1.1076765	1.23E-10	1.87E-10	0.44	0.39	0.47	1.17	1.37	1.21
OsR498G0815837600.01	0.82	2.73	1.7941604	6.75E-49	5.26E-49	0.82	0.89	0.75	2.99	2.83	2.37
OsR498G0815845300.01	0.326	9.17	4.7874785	5.79E-173	1.95E-173	0.29	0.29	0.4	8.4	9.78	9.33
OsR498G0815850400.01	146.483	51.623	-1.504718	0	0	153.26	143.43	142.76	51.6	53.57	49.7
OsR498G0815855600.01	0.116	0.553	2.3894475	0.0009846	0.0020515	0.23	0.12	0	0.79	0.52	0.35
OsR498G0815858000.01	32.283	13.903	-1.28193	3.56E-109	1.68E-109	32.25	32.41	32.19	13.91	13.97	13.83
OsR498G0815866300.01	11.31	0.3	-5.226542	1.46E-200	4.38E-201	11.48	12.37	10.08	0.3	0.33	0.27
OsR498G0815873600.01	0.066	1.84	4.7817649	1.80E-14	2.45E-14	0	0	0.2	2.15	1.27	2.1
OsR498G0815877200.01	4.133	1.11	-1.920017	7.26E-48	5.72E-48	4.13	4.34	3.93	1.23	1.02	1.08
OsR498G0815884600.01	9.833	32.75	1.7141397	2.87E-206	8.39E-207	9.37	10.43	9.7	34.3	30.68	33.27
OsR498G0815886000.01	16.13	66.353	2.0398206	0	0	17.32	15.02	16.05	66.04	63.53	69.49
OsR498G0815886400.01	3.303	10.236	1.6286351	7.75E-32	7.58E-32	3.02	3.4	3.49	9.05	10.51	11.15
OsR498G0815887100.01	1.276	4.126	1.6543209	2.28E-14	3.10E-14	1.67	1.21	0.95	4.79	3.41	4.18
OsR498G0815887900.01	0.22	4.876	4.4566129	8.07E-100	4.06E-100	0.2	0.2	0.26	4.57	5.05	5.01
OsR498G0815908600.01	3.343	0.82	-2.033079	4.59E-39	4.05E-39	3.13	3.66	3.24	0.74	0.84	0.88
OsR498G0815911100.01	4.996	9.96	1.0508231	7.13E-42	6.05E-42	4.45	4.88	5.66	10.42	9.69	9.77
OsR498G0815912200.01	38.33	84.086	1.1258984	0	0	37.99	39.33	37.67	83.69	85.73	82.84
OsR498G0815920400.01	0.496	1.42	1.6876966	2.07E-13	2.89E-13	0.29	0.62	0.58	1.38	1.18	1.7
OsR498G0815926700.01	0.56	1.15	1.0041573	3.48E-07	5.97E-07	0.53	0.6	0.55	1.22	1.27	0.96
OsR498G0815932400.01	2.52	0.55	-2.020641	6.24E-33	5.99E-33	2.68	2.64	2.24	0.7	0.59	0.36
OsR498G0815937300.01	0.466	0.013	-5.195515	2.90E-10	4.44E-10	0.39	0.35	0.66	0	0	0.04
OsR498G0815941000.01	0.48	0	-5.608146	1.86E-07	3.16E-07	0.66	0.36	0.42	0	0	0
OsR498G0815946800.01	0	0.71	6.6468353	1.77E-13	2.46E-13	0	0	0	0.44	0.75	0.94
OsR498G0815961200.01	6.263	30.57	2.2826631	4.45E-130	1.86E-130	7.35	6.07	5.37	30.31	30.34	31.06
OsR498G0815962200.01	164.466	73.036	-1.17217	0	0	165.05	161.76	166.59	71.64	71.23	76.24
OsR498G0815970500.01	27.48	11.676	-1.241296	1.32E-111	6.16E-112	27.97	27.58	26.89	11.01	11.68	12.34
OsR498G0815974000.01	0.183	0	-4.72603	0.0001114	0.0002167	0.21	0.17	0.17	0	0	0
OsR498G0815974500.01	2.74	9.49	1.791415	1.04E-48	8.13E-49	2.94	2.8	2.48	9.15	9.7	9.62
OsR498G0815975800.01	2.553	10.186	1.9970587	8.64E-267	2.00E-267	2.83	2.36	2.47	10.09	10.16	10.31
OsR498G0815979300.01	4.543	51.146	3.4913193	0	0	4.74	4.52	4.37	51.71	47.73	54
OsR498G0815983000.01	0.04	2.54	5.9955565	1.56E-92	8.28E-93	0.02	0.04	0.06	2.17	2.77	2.68
OsR498G0815983900.01	1.16	0.313	-1.884992	3.34E-14	4.56E-14	1.06	1.4	1.02	0.32	0.37	0.25
OsR498G0815984500.01	0.356	1.026	1.550148	1.95E-07	3.30E-07	0.38	0.25	0.44	1.08	1.11	0.89
OsR498G0815988100.01	0.07	1.213	4.0618728	4.75E-16	6.21E-16	0.16	0.05	0	1.33	1	1.31
OsR498G0815992300.01	16.846	2.303	-2.886028	0	0	16	18.04	16.5	2.08	2.51	2.32
OsR498G0815993500.01	17.513	41.283	1.2492938	1.11E-234	2.90E-235	17.87	17.04	17.63	41.32	40.87	41.66
OsR498G0815995400.01	0	0.713	6.7023304	6.61E-14	9.09E-14	0	0	0	0.76	0.84	0.54
OsR498G0815998500.01	1.12	4.483	1.9958897	9.28E-44	7.67E-44	0.81	1.07	1.48	4.44	4.55	4.46
OsR498G0815999400.01	7.613	19.503	1.3554754	1.84E-82	1.06E-82	7.93	7.55	7.36	19.67	18.18	20.66
OsR498G0816006300.01	0.153	0	-4.610553	0.0002051	0.000405	0.18	0.14	0.14	0	0	0
OsR498G0816011100.01	20.83	41.826	1.0081942	2.54E-64	1.69E-64	21.78	21.29	19.42	43.16	36.08	46.24
OsR498G0816011700.01	1.726	4.483	1.165217	3.15E-11	4.69E-11	1.61	2.33	1.24	4.12	4.79	4.54
OsR498G0816026100.01	146.076	60.476	-1.279959	0	0	140.85	149.84	147.54	61.94	58.9	60.59
OsR498G0816026400.01	4.153	10.186	1.2940857	1.76E-101	8.75E-102	4.08	4.38	4	10.14	10.23	10.19
OsR498G0816026500.01	0.643	4.483	2.7971401	4.14E-132	1.72E-132	0.5	0.77	0.66	4.45	4.57	4.43
OsR498G0816028000.01	2.02	5.203	1.4492208	3.05E-28	3.16E-28	2.02	1.97	2.07	5.8	5.14	4.67
OsR498G0816030200.01	0.33	1.686	2.4736426	1.31E-21	1.53E-21	0.31	0.27	0.41	1.44	1.74	1.88
OsR498G0816031300.01	1.59	12.92	3.0258696	6.38E-248	1.59E-248	1.58	1.51	1.68	12.03	14.46	12.27
OsR498G0816031500.01	0.173	2.04	3.4901098	2.85E-36	2.60E-36	0.06	0.31	0.15	2.29	1.83	2
OsR498G0816033100.01	3.2	7.38	1.1625189	1.69E-48	1.32E-48	3.37	3.48	2.75	7.83	7.56	6.75
OsR498G0816038800.01	0.053	0.616	3.5129481	0.0007191	0.0014778	0	0.16	0	0.72	0.48	0.65
OsR498G0816040200.01	1.856	10.28	2.4676914	8.04E-32	7.87E-32	1.77	2.34	1.46	11.43	10.29	9.12
OsR498G0816041700.01	0.456	8.206	4.1675246	7.37E-95	3.84E-95	0.4	0.68	0.29	8.07	8.43	8.12
OsR498G0816044700.01	0.85	8.956	3.3958797	1.71E-129	7.18E-130	0.73	0.8	1.02	9.26	8.34	9.27
OsR498G0816045900.01	0	0.463	5.4960889	4.11E-07	7.08E-07	0	0	0	0	0.81	0.58
OsR498G0816047300.01	0	0.726	6.103693	6.09E-10	9.42E-10	0	0	0	0.68	0.8	0.7
OsR498G0816054900.01	26.396	115.57	2.1345263	0	0	26.67	25.93	26.59	120.42	118.32	107.97

OsR498G0816061900.01	34.04	75.933	1.1426863	6.55E-280	1.47E-280	31.77	35.62	34.73	70.67	76.21	80.92
OsR498G0816068700.01	0.106	4.91	5.6124838	8.38E-118	3.76E-118	0.13	0.06	0.13	4.47	5.07	5.19
OsR498G0816073400.01	49.06	112.77	1.1904183	0	0	48.38	49.22	49.58	112.83	111.79	113.7
OsR498G0816077100.01	16.756	34.313	1.0358328	1.32E-110	6.18E-111	16.24	16.57	17.46	33.62	34.65	34.67
OsR498G0816077400.01	3.14	1.53	-1.02559	3.56E-06	6.38E-06	3.03	3.6	2.79	1.69	2.01	0.89
OsR498G0816077700.01	0.076	15.746	7.6354754	4.16E-218	1.15E-218	0	0.09	0.14	14.15	14.04	19.05
OsR498G0816082900.01	0.77	0.386	-1.053071	0.000805	0.0016622	0.36	1.11	0.84	0.31	0.23	0.62
OsR498G0816086800.01	1.77	5.73	1.6975433	1.95E-73	1.19E-73	1.93	1.76	1.62	5.18	5.62	6.39
OsR498G0816088300.01	171.413	837.67	2.2874022	0	0	175.23	168.7	170.31	820.28	818.38	874.35
OsR498G0816088900.01	2.573	6.696	1.4078931	1.48E-36	1.35E-36	2.73	2.45	2.54	6.11	7.09	6.89
OsR498G0816104600.01	2.01	6.823	1.8259988	1.97E-53	1.46E-53	1.62	2.1	2.31	6.03	7.75	6.69
OsR498G0816110100.01	9.596	35.393	1.8732727	2.86E-303	5.99E-304	9.03	9.91	9.85	34.95	36.5	34.73
OsR498G0816110700.01	1.863	4.523	1.2781907	1.05E-14	1.42E-14	1.76	1.62	2.21	4.2	4.47	4.9
OsR498G0816112000.01	2.413	0.333	-2.853409	7.68E-36	7.07E-36	2.7	2.47	2.07	0.36	0.2	0.44
OsR498G0816116900.01	5.083	1.746	-1.546827	2.94E-13	4.11E-13	4.84	3.93	6.48	1.35	1.82	2.07
OsR498G0816118200.01	1.2	2.82	1.2310537	2.89E-08	4.75E-08	1.34	1.11	1.15	1.92	3.43	3.11
OsR498G0816118900.01	3.126	9.826	1.6338224	7.58E-55	5.52E-55	3.21	2.74	3.43	9.73	9.46	10.29
OsR498G0816119300.01	1.543	76.27	5.6290814	0	0	1.66	1.14	1.83	78.25	76.02	74.54
OsR498G0816121400.01	7.526	17.043	1.179501	9.54E-84	5.42E-84	7.63	7.64	7.31	16.64	17.61	16.88
OsR498G0816124800.01	6.6	2.076	-1.671159	6.50E-28	6.78E-28	4.79	7.12	7.89	2.39	1.58	2.26
OsR498G0816126000.01	39.303	18	-1.126458	0	0	40.04	39.66	38.21	18.13	18.8	17.07
OsR498G0816128800.01	0.65	0.206	-1.647078	6.05E-05	0.0001157	0.59	0.73	0.63	0.28	0.14	0.2
OsR498G0816133900.01	15.713	38.906	1.3000691	4.41E-185	1.41E-185	15.33	15.33	16.48	38.44	39.76	38.52
OsR498G0816135800.01	4.076	2.053	-1.010077	7.46E-16	9.79E-16	4.73	3.88	3.62	2.06	1.88	2.22
OsR498G0816137700.01	18.46	2.253	-3.036007	4.87E-96	2.52E-96	18.28	18.53	18.57	2.31	1.61	2.84
OsR498G0816148700.01	33.206	76.663	1.2041544	2.12E-254	5.13E-255	32.9	32.51	34.21	74.7	78.01	77.28
OsR498G0816149200.01	0.65	1.526	1.3982248	2.04E-20	2.43E-20	0.55	0.69	0.71	1.39	1.67	1.52
OsR498G0816149600.01	0.8	0.296	-1.389221	6.87E-07	1.20E-06	0.63	1.27	0.5	0.38	0.27	0.24
OsR498G0816150900.01	0.02	0.56	4.6609105	6.96E-25	7.63E-25	0.04	0.02	0	0.5	0.66	0.52
OsR498G0816151100.01	1.783	12.8	2.8421375	4.98E-135	2.03E-135	2.11	1.32	1.92	12.4	12.3	13.7
OsR498G0816151600.01	10.23	91.996	3.1636727	0	0	9.84	10.48	10.37	97.21	90.39	88.39
OsR498G0816153900.01	1.09	2.476	1.1754575	1.55E-13	2.16E-13	1.14	0.97	1.16	2.41	2.48	2.54
OsR498G0816159000.01	1401.83	162.22	-3.113775	0	0	1415.8	1380.4	1409.3	162.44	159.54	164.69
OsR498G0816162000.01	40.203	95.073	1.2376912	1.09E-182	3.51E-183	41.12	39.46	40.03	94.96	96.57	93.69
OsR498G0816166700.01	0.823	0.386	-1.055337	1.00E-05	1.84E-05	0.95	0.88	0.64	0.31	0.41	0.44
OsR498G0816167400.01	0.38	8.16	4.3964747	3.27E-151	1.22E-151	0.42	0.26	0.46	7.96	8.15	8.37
OsR498G0816168500.01	13.646	32.92	1.2853623	8.35E-131	3.48E-131	14.25	13.63	13.06	33.39	33.64	31.73
OsR498G0816170600.01	9.826	20.943	1.0895109	1.18E-55	8.54E-56	9.03	10.98	9.47	20.5	22.03	20.3
OsR498G0816171500.01	0.7	4.153	2.9970114	9.97E-129	4.20E-129	0.77	0.65	0.68	4.39	3.86	4.21
OsR498G0816173800.01	6.043	16.01	1.4079663	1.00E-62	6.76E-63	6.78	5.58	5.77	16.4	14.83	16.8
OsR498G0816180400.01	0.74	22.816	4.9487724	0	0	0.77	0.64	0.81	23.06	22.57	22.82
OsR498G0816189900.01	0.02	0.596	4.97441	4.19E-09	6.67E-09	0	0	0.06	0.72	0.33	0.74
OsR498G0816190600.01	23.43	53.746	1.1986902	7.75E-127	3.31E-127	24.15	22.4	23.74	50.85	51.76	58.63
OsR498G0816196900.01	0.21	0.74	1.8146295	6.61E-12	9.62E-12	0.21	0.23	0.19	0.9	0.76	0.56
OsR498G0816198200.01	0.426	0.183	-1.247982	1.33E-06	2.34E-06	0.48	0.39	0.41	0.17	0.15	0.23
OsR498G0816205800.01	0.293	3.283	3.4948803	9.29E-67	6.02E-67	0.26	0.35	0.27	2.68	3.5	3.67
OsR498G0816213300.01	24.083	7.816	-1.624128	1.53E-99	7.71E-100	23.74	24.25	24.26	7.9	8.03	7.52
OsR498G0816215800.01	0.006	0.086	3.6748497	0.0003128	0.0006259	0.02	0	0	0.14	0.04	0.08
OsR498G0816217100.01	4.34	11.04	1.3555575	5.71E-106	2.76E-106	4.13	4.48	4.41	12.43	10.91	9.78
OsR498G0816223900.01	23.32	5.846	-1.982111	4.85E-131	2.02E-131	24.83	22.21	22.92	5.29	5.96	6.29
OsR498G0816228700.01	0.816	5.356	2.7238654	4.57E-178	1.51E-178	0.75	0.97	0.73	4.98	5.66	5.43
OsR498G0816230500.01	6.346	12.733	1.0061188	2.91E-22	3.35E-22	6.53	5.66	6.85	12.14	12.81	13.25
OsR498G0816230800.01	0	0.616	5.5593725	2.32E-07	3.95E-07	0	0	0	0.59	0.81	0.45
OsR498G0816232300.01	0.133	0.64	2.2639166	4.30E-07	7.42E-07	0.13	0.09	0.18	0.65	0.56	0.71
OsR498G0816238400.01	0.18	2.643	3.8597674	1.17E-55	8.45E-56	0.12	0.24	0.18	2.51	2.56	2.86
OsR498G0816246400.01	0.2	1	2.3137201	1.32E-22	1.50E-22	0.29	0.16	0.15	0.87	1.01	1.12
OsR498G0816246800.01	0.176	0	-4.347518	0.0007014	0.0014405	0.05	0.05	0.43	0	0	0
OsR498G0816252800.01	1.673	4.816	1.5073778	1.86E-47	1.47E-47	1.66	1.54	1.82	5.12	4.8	4.53
OsR498G0816253000.01	0.81	2.636	1.7010336	6.01E-25	6.58E-25	0.88	0.91	0.64	2.01	2.84	3.06
OsR498G0816253600.01	0.156	0.94	2.4574928	2.98E-18	3.71E-18	0.18	0.14	0.15	0.8	0.99	1.03
OsR498G0816254600.01	0.3	1.223	1.984394	1.06E-16	1.37E-16	0.36	0.2	0.34	1.24	1.22	1.21
OsR498G0816256800.01	0.453	0.026	-4.154873	2.44E-09	3.85E-09	0.59	0.15	0.62	0.08	0	0
OsR498G0816257100.01	0.296	0.096	-1.610553	0.000882	0.0018289	0.23	0.26	0.4	0.03	0.19	0.07
OsR498G0816257600.01	0.523	1.326	1.3421417	1.94E-06	3.45E-06	0.46	0.45	0.66	1.58	0.98	1.42
OsR498G0816262400.01	1.87	4.156	1.1169491	2.04E-14	2.77E-14	1.52	2.17	1.92	3.78	4.05	4.64
OsR498G0816265000.01	50.846	20.603	-1.29178	7.52E-304	1.57E-304	52.94	50.55	49.05	19.77	20.74	21.3
OsR498G0816265500.01	3.233	6.636	1.1052574	3.87E-39	3.40E-39	2.66	3.42	3.62	6.37	7.41	6.13
OsR498G0816266200.01	0.026	0.563	3.0800948	0.0001352	0.0002645	0	0.04	0.04	0.27	0.27	1.15
OsR498G0816274900.01	179.49	45.08	-1.994106	0	0	179.49	176.14	182.84	45.83	42.87	46.54

OsR498G0816276200.01	5.24	0.35	-4.177027	8.78E-145	3.38E-145	4.95	4.57	6.2	0.11	0.55	0.39
OsR498G0816278300.01	1.493	0.263	-2.496896	1.03E-18	1.27E-18	1.4	1.35	1.73	0.21	0.36	0.22
OsR498G0816279900.01	11.153	4.783	-1.211457	7.66E-39	6.77E-39	11.54	11.39	10.53	5.09	4.55	4.71
OsR498G0816282000.01	0.596	0.203	-1.540163	0.0001233	0.0002404	0.72	0.31	0.76	0.22	0.17	0.22
OsR498G0816284600.01	1.84	0.163	-3.499521	4.87E-21	5.73E-21	1.22	2.37	1.93	0.22	0.05	0.22
OsR498G0816291500.01	6.793	17.12	1.3292856	7.36E-99	3.73E-99	7.07	7.34	5.97	16.42	17.36	17.58
OsR498G0816299200.01	0.823	0.16	-2.347518	3.22E-08	5.30E-08	0.75	1.08	0.64	0.19	0.14	0.15
OsR498G0816299500.01	0.566	2.926	2.3683242	9.87E-31	9.83E-31	0.56	0.43	0.71	2.54	2.99	3.25
OsR498G0816303400.01	8.01	25.636	1.6757898	1.55E-126	6.65E-127	8.26	7.67	8.1	24.56	25.52	26.83
OsR498G0816310400.01	20.76	46.3	1.158102	0	0	21.74	20.58	19.96	46.6	47.95	44.35
OsR498G0816314700.01	10.266	0.276	-5.178044	5.46E-252	1.33E-252	11.06	9.7	10.04	0.46	0.09	0.28
OsR498G0816319500.01	53.693	267.61	2.4180802	0	0	54.22	50.62	56.24	267.08	266.57	269.17
OsR498G0816320000.01	0.096	0.493	2.2545179	7.80E-06	1.42E-05	0.17	0.04	0.08	0.44	0.41	0.63
OsR498G0816320800.01	0	0.19	4.2963381	0.0007942	0.0016388	0	0	0	0.11	0.16	0.3
OsR498G0816322600.01	0.11	0.473	2.1204154	8.86E-10	1.38E-09	0.1	0.19	0.04	0.44	0.49	0.49
OsR498G0816322700.01	3.66	15.33	2.0707167	1.78E-159	6.43E-160	3.82	3.85	3.31	14.86	16.78	14.35
OsR498G0816325400.01	11.1	22.913	1.1331077	9.97E-71	6.24E-71	11.38	8.84	13.08	22.73	20.02	25.99
OsR498G0816328500.01	3.676	1.62	-1.191621	2.37E-09	3.74E-09	3.71	0.72	6.6	2.12	0	2.74
OsR498G0816328800.01	7.506	16.3	1.1046317	7.03E-40	6.13E-40	7.37	8.84	6.31	15.25	16.54	17.11
OsR498G0816336200.01	0.25	0.95	1.97441	8.24E-06	1.51E-05	0.15	0.24	0.36	0.92	1.13	0.8
OsR498G0816336400.01	0.503	1.206	1.3253171	6.30E-12	9.16E-12	0.48	0.44	0.59	1.12	1.43	1.07
OsR498G0816336600.01	30.14	98.006	1.7005633	0	0	28.31	30.15	31.96	93.31	101.01	99.7
OsR498G0816337900.01	2.623	6.373	1.2794642	5.70E-25	6.24E-25	2.44	2.87	2.56	6.78	5.73	6.61
OsR498G0816340500.01	1.96	4.88	1.290814	2.05E-23	2.31E-23	1.82	2.25	1.81	4.99	4.79	4.86
OsR498G0816343000.01	0.36	0	-4.485022	0.0003787	0.0007614	0.67	0	0.41	0	0	0
OsR498G0816347700.01	1.866	0.573	-1.449398	3.66E-09	5.82E-09	1.91	1.39	2.3	0.68	0.37	0.67
OsR498G0816352200.01	5.676	23.69	2.0679367	0	0	6.25	5.12	5.66	24.47	23.49	23.11
OsR498G0816352400.01	9.673	0.88	-3.483248	4.63E-157	1.69E-157	9.41	8.96	10.65	0.78	0.88	0.98
OsR498G0816355900.01	5.31	0.176	-5.120108	8.81E-98	4.50E-98	6.25	4.52	5.16	0.43	0.1	0
OsR498G0816362900.01	6.6	2.146	-1.635384	2.79E-25	3.04E-25	6.74	6.18	6.88	2.32	2.19	1.93
OsR498G0816363100.01	5.483	13.266	1.1908625	9.66E-155	3.55E-155	5.29	5.69	5.47	12.69	13.93	13.18
OsR498G0816366500.01	0.103	0.366	1.8674948	1.13E-06	1.99E-06	0.09	0.11	0.11	0.4	0.33	0.37
OsR498G0816366800.01	13.37	32.3	1.2896743	2.50E-150	9.36E-151	14.58	12.55	12.98	32.64	32.56	31.7
OsR498G0816367700.01	1.41	4.04	1.5102845	2.06E-58	1.45E-58	1.18	1.48	1.57	3.83	4	4.29
OsR498G0816368200.01	2.856	6.15	1.1711091	1.10E-15	1.44E-15	2.9	3.3	2.37	7.38	4.73	6.34
OsR498G0816372500.01	1.806	3.91	1.1136269	2.87E-31	2.83E-31	2.15	1.55	1.72	3.97	3.69	4.07
OsR498G0816373200.01	2.523	0.093	-4.748056	4.22E-32	4.11E-32	2.08	2.57	2.92	0	0.11	0.17
OsR498G0816373700.01	0.283	0.85	1.5844634	4.25E-06	7.66E-06	0.18	0.18	0.49	0.83	0.87	0.85
OsR498G0816380100.01	2.583	1.126	-1.185586	1.26E-19	1.53E-19	2.6	2.89	2.26	1.14	1.26	0.98
OsR498G0816380700.01	7.653	22.683	1.5632507	4.32E-166	1.51E-166	7.6	7.89	7.47	22.58	22.17	23.3
OsR498G0816389800.01	0.65	1.333	1.0278492	5.91E-06	1.07E-05	0.59	0.66	0.7	1.3	1.26	1.44
OsR498G0816390800.01	29.853	105.95	1.8360649	0	0	30.45	28.86	30.25	103.78	102.6	111.47
OsR498G0816391800.01	1.643	4.903	1.6629101	6.80E-31	6.75E-31	1.4	1.62	1.91	4.68	5.36	4.67
OsR498G0816397900.01	2.663	11.343	2.0905763	1.12E-154	4.12E-155	3.12	2.18	2.69	10.67	11.82	11.54
OsR498G0816398400.01	3.576	9.613	1.4343082	6.67E-52	5.03E-52	3.68	3.65	3.4	10.38	9.08	9.38
OsR498G0816401700.01	0.04	0.253	2.6748497	1.53E-05	2.83E-05	0	0.12	0	0.17	0.2	0.39
OsR498G0816418300.01	7.983	0.81	-3.362625	3.98E-73	2.45E-73	8	6.63	9.32	0.53	1.06	0.84
OsR498G0816420800.01	0.046	0.803	4.1241571	8.23E-17	1.06E-16	0.07	0	0.07	0.64	0.7	1.07
OsR498G0816422700.01	2.13	0.333	-2.75351	6.86E-59	4.80E-59	2.02	2.05	2.32	0.34	0.37	0.29
OsR498G0816425400.01	0.176	0.01	-3.932481	7.73E-05	0.0001487	0.25	0.14	0.14	0.03	0	0
OsR498G0816427800.01	1.326	3.75	1.5327045	5.19E-21	6.12E-21	1.63	1.2	1.15	3.44	3.75	4.06
OsR498G0816430400.01	0.316	1.036	1.6952559	3.75E-09	5.97E-09	0.39	0.28	0.28	0.8	1.35	0.96
OsR498G0816432100.01	0.843	0.153	-2.453167	2.59E-11	3.84E-11	1.05	0.52	0.96	0.11	0.24	0.11
OsR498G0816435000.01	0.823	0.173	-2.258251	2.25E-13	3.14E-13	0.66	0.92	0.89	0.23	0.13	0.16
OsR498G0816438400.01	0.02	1.603	6.6886555	8.49E-26	9.17E-26	0	0	0.06	1.5	1.64	1.67
OsR498G0816439800.01	0.68	7.486	3.4613245	2.81E-238	7.29E-239	0.78	0.66	0.6	7.37	7.33	7.76
OsR498G0816440900.01	5.366	25.21	2.2315563	0	0	5.51	5.09	5.5	25.67	23.34	26.62
OsR498G0816441200.01	0.33	0.026	-3.610553	7.25E-09	1.16E-08	0.28	0.38	0.33	0.08	0	0
OsR498G0816446900.01	0.013	0.26	4.4338416	1.91E-11	2.81E-11	0	0.04	0	0.19	0.25	0.34
OsR498G0816450400.01	0.466	3.973	3.0871101	3.93E-31	3.89E-31	0.7	0.49	0.21	3.97	4.16	3.79
OsR498G0816453100.01	100.37	20.29	-2.289771	0	0	95.24	103.8	102.07	19.98	19.39	21.5
OsR498G0816454300.01	0.006	0.126	4.0618728	2.82E-05	5.29E-05	0	0.02	0	0.13	0.18	0.07
OsR498G0816454500.01	12.526	27.166	1.1162878	3.85E-48	3.03E-48	13.71	11.03	12.84	28.11	26.92	26.47
OsR498G0816457100.01	0.243	0	-5.632624	1.48E-07	2.50E-07	0	0.73	0	0	0	0
OsR498G0816457300.01	4.776	11.413	1.2602901	2.66E-116	1.21E-116	4.97	4.66	4.7	11.04	12.44	10.76
OsR498G0816459900.01	0	0.113	4.4338416	0.0004331	0.0008746	0	0	0	0.09	0.09	0.16
OsR498G0816461800.01	0.043	0.263	2.5570697	3.25E-05	6.10E-05	0.05	0.05	0.03	0.13	0.24	0.42
OsR498G0816464400.01	1.406	4.503	1.6802972	4.33E-18	5.39E-18	1.53	1.17	1.52	4.58	4.3	4.63
OsR498G0816468300.01	1.22	3.463	1.4889831	4.95E-21	5.83E-21	1.19	1.19	1.28	3.14	3.62	3.63

OsR498G0816469100.01	18.676	46.363	1.3138929	0	0	18.66	18.5	18.87	47.39	48.52	43.18
OsR498G0816469400.01	0.22	0.966	2.1249696	2.97E-14	4.05E-14	0.24	0.21	0.21	0.83	1.27	0.8
OsR498G0816475800.01	23.62	52.193	1.1444718	8.09E-242	2.06E-242	24.17	24	22.69	51.38	53.36	51.84
OsR498G0816476700.01	1.186	0.543	-1.296892	1.03E-09	1.60E-09	1.56	1.19	0.81	0.64	0.37	0.62
OsR498G0816481400.01	2.093	6.01	1.2072987	1.57E-58	1.10E-58	2.03	1.92	2.33	6.09	5.99	5.95
OsR498G0816482400.01	1.226	0.163	-2.941198	3.71E-29	3.78E-29	1.47	1.04	1.17	0.22	0.11	0.16
OsR498G0816482800.01	9.76	3.26	-1.584624	8.60E-61	5.90E-61	10.37	9.85	9.06	2.59	3.39	3.8
OsR498G0816484000.01	0.103	0.856	3.2171551	4.32E-23	4.89E-23	0.04	0.16	0.11	0.75	1.11	0.71
OsR498G0816484600.01	0.386	0.866	1.1549822	7.39E-05	0.0001421	0.43	0.31	0.42	0.89	0.91	0.8
OsR498G0816484800.01	1.77	4.003	1.1677939	5.65E-15	7.57E-15	1.49	1.73	2.09	3.9	4.05	4.06
OsR498G0816489400.01	25.686	58.51	1.1895481	3.36E-181	1.09E-181	25.13	25.68	26.25	58.52	59.02	57.99
OsR498G0816490600.01	0.543	1.87	1.7817649	3.08E-14	4.20E-14	0.49	0.61	0.53	1.71	1.74	2.16
OsR498G0816492300.01	21.52	114.4	2.4094439	0	0	22.11	20.43	22.02	116	112.64	114.56
OsR498G0816498900.01	357.206	170.79	-1.049042	0	0	362.16	363.83	345.63	169.11	166.65	176.62
OsR498G0816499400.01	2.563	7.21	1.4950992	4.15E-32	4.04E-32	3.15	2.27	2.27	6.69	8.4	6.54
OsR498G0816499600.01	8.47	38.28	2.1743321	1.75E-292	3.77E-293	8.64	8.23	8.54	36.36	36.89	41.59
OsR498G0816499900.01	0.15	0.98	2.7023304	0.0002308	0.0004573	0.23	0.22	0	0.75	1.29	0.9
OsR498G0816502200.01	1.966	0.55	-2.125558	1.21E-06	2.13E-06	1.85	1.69	2.36	1.02	0.31	0.32
OsR498G0816509400.01	0.22	0.96	2.0898872	2.36E-10	3.61E-10	0.22	0.33	0.11	0.86	1.07	0.95
OsR498G0816510300.01	1.723	17.46	3.3394544	5.29E-168	1.83E-168	1.69	1.56	1.92	16.1	18.75	17.53
OsR498G0816514600.01	11.756	42.656	1.8580576	7.97E-204	2.35E-204	11.89	11.98	11.4	40.08	43.26	44.63
OsR498G0816515900.01	228.133	96.933	-1.204169	0	0	227.7	229.85	226.85	95.35	101	94.45
OsR498G0816519700.01	9.193	0.75	-3.610553	7.41E-108	3.53E-108	9.38	9.88	8.32	0.84	0.34	1.07
OsR498G0816520200.01	13.393	45.72	1.7625476	0	0	13.07	14.18	12.93	45.53	48.1	43.53
OsR498G0816520500.01	7.516	23.916	1.6703105	6.28E-252	1.53E-252	7.44	7.79	7.32	25.04	24.02	22.69
OsR498G0816522000.01	33.316	76.67	1.20396	5.81E-218	1.61E-218	32.86	33.12	33.97	78.25	78.18	73.58
OsR498G0816524700.01	7.123	2.873	-1.292926	2.01E-43	1.67E-43	7.19	7.49	6.69	2.69	2.87	3.06
OsR498G0816525700.01	0.32	0	-4.882576	4.55E-05	8.63E-05	0.2	0.55	0.21	0	0	0
OsR498G0816525900.01	0.03	0.753	4.563452	2.90E-07	4.96E-07	0	0	0.09	0.29	0.45	1.52
OsR498G0816530800.01	2.16	7.603	1.8128661	5.30E-21	6.25E-21	1.88	3.07	1.53	6.44	8.56	7.81
OsR498G0816530900.01	0.85	1.973	1.1625259	7.63E-09	1.23E-08	0.85	0.72	0.98	2.12	2.01	1.79
OsR498G0816531500.01	96.673	224.56	1.2505119	0	0	97.71	91.6	100.71	221.73	219.44	232.52
OsR498G0816534500.01	1.866	0.583	-1.647078	3.03E-08	4.98E-08	1.23	2.5	1.87	0.71	0.51	0.53
OsR498G0816541500.01	0.62	1.24	1.2114492	1.06E-11	1.55E-11	0.51	0.47	0.88	1.23	1.33	1.16
OsR498G0816541900.01	1.403	3.08	1.145174	1.76E-21	2.06E-21	1.56	1.23	1.42	3.35	3.08	2.81
OsR498G0816542500.01	8.98	27.026	1.5755964	1.08E-267	2.50E-268	8.94	9.52	8.48	26.79	26.98	27.31
OsR498G0816548900.01	0.833	1.866	1.1665512	4.42E-21	5.19E-21	0.76	1	0.74	2.05	1.73	1.82
OsR498G0816550300.01	0.873	0.076	-3.52809	2.38E-08	3.90E-08	1.39	0.46	0.77	0.23	0	0
OsR498G0816550500.01	0.956	0.073	-3.669446	9.88E-07	1.73E-06	1.27	1.03	0.57	0.11	0	0.11
OsR498G0816552000.01	0.096	0.29	1.6069563	0.0004584	0.0009273	0.03	0.06	0.2	0.36	0.25	0.26
OsR498G0816552100.01	2.59	8.923	1.7681252	1.35E-52	1.01E-52	2.41	2.76	2.6	8.92	8.65	9.2
OsR498G0816554800.01	3.74	14.786	2.0006893	4.13E-145	1.58E-145	3.64	3.68	3.9	15.42	14.8	14.14
OsR498G0816554900.01	3.56	13.883	1.9623946	2.72E-86	1.52E-86	4.05	3.26	3.37	13.19	13.53	14.93
OsR498G0816555100.01	0.333	0.906	1.3583249	3.65E-08	6.02E-08	0.41	0.38	0.21	0.77	1.13	0.82
OsR498G0816562300.01	3.566	9.82	1.4129064	1.25E-64	8.30E-65	3.81	3.1	3.79	10.87	8.59	10
OsR498G0816563700.01	0.076	0.276	1.9644368	2.61E-07	4.47E-07	0.09	0.05	0.09	0.38	0.22	0.23
OsR498G0816564000.01	3.536	9.266	1.3706493	7.18E-139	2.86E-139	3.56	3.46	3.59	9.64	9.64	8.52
OsR498G0816567100.01	0.86	0.413	-1.010326	0.0004553	0.0009208	0.63	1.1	0.85	0.28	0.45	0.51
OsR498G0816568800.01	0.393	0.173	-1.187861	0.0005417	0.001101	0.23	0.55	0.4	0.15	0.19	0.18
OsR498G0816569600.01	2.1	6.286	1.5900041	1.13E-55	8.15E-56	2	2.24	2.06	6.05	6.42	6.39
OsR498G0816570600.01	0.58	1.776	1.6221082	9.06E-13	1.29E-12	0.89	0.31	0.54	1.31	1.79	2.23
OsR498G0816573200.01	1.513	0.03	-5.80695	4.19E-67	2.71E-67	1.54	1.29	1.71	0.07	0.02	0
OsR498G0816578500.01	5.373	2.013	-1.417099	2.80E-16	3.64E-16	5.38	5.62	5.12	2.12	1.97	1.95
OsR498G0816581200.01	2.29	5.703	1.3215086	4.94E-20	5.92E-20	2.41	1.96	2.5	5.94	5.22	5.95
OsR498G0816584000.01	16.4	45.096	1.4607431	0	0	16.03	15.68	17.49	45.75	45.56	43.98
OsR498G0816584800.01	3.673	14.956	2.0246089	3.01E-103	1.48E-103	4.28	3.53	3.21	13.93	15.25	15.69
OsR498G0816588400.01	0.57	0.17	-1.703662	8.32E-06	1.52E-05	0.49	0.56	0.66	0.18	0.19	0.14
OsR498G0816588600.01	0.253	0.053	-2.347518	1.45E-07	2.45E-07	0.23	0.25	0.28	0.03	0.04	0.09
OsR498G0816589700.01	3.1	14.2	2.1944039	8.11E-72	5.02E-72	3.04	3.38	2.88	13.86	14.37	14.37
OsR498G0816590700.01	7.256	15.536	1.0902406	3.31E-59	2.31E-59	6.96	7.57	7.24	14.52	16.52	15.57
OsR498G0816591500.01	0	0.51	6.7714229	1.85E-14	2.52E-14	0	0	0	0.45	0.49	0.59
OsR498G0816593900.01	11.113	23.653	1.1300619	2.86E-125	1.23E-125	11.45	11.74	10.15	24.55	24.12	22.29
OsR498G0816595900.01	0.183	0.483	1.3894475	0.0003735	0.0007502	0.2	0.17	0.18	0.59	0.3	0.56
OsR498G0816597800.01	0.04	0.493	3.5593725	2.47E-06	4.39E-06	0.06	0	0.06	0.06	1.04	0.38
OsR498G0816598100.01	7.533	2.673	-1.533259	7.80E-80	4.56E-80	7.4	7.81	7.39	2.64	3.01	2.37
OsR498G0816600400.01	13.873	3.846	-1.85115	5.38E-123	2.34E-123	12.99	12.85	15.78	3.94	3.74	3.86
OsR498G0816600800.01	115.843	52.746	-1.135928	3.77E-299	8.01E-300	115.82	116.8	114.91	54.99	49.98	53.27
OsR498G0816601500.01	7.616	85.696	3.6095401	0	0	7.73	7.87	7.25	85.07	86.03	85.99
OsR498G0816601700.01	2.473	5.716	1.2019138	1.95E-20	2.32E-20	2.46	2.36	2.6	6.12	5.69	5.34

OsR498G0816602400.01	67.093	136.58	1.0150789	0	0	69.22	66.65	65.41	142.25	136.34	131.16
OsR498G0816602800.01	1.866	3.706	1.0509674	1.29E-10	1.95E-10	1.7	1.96	1.94	3.66	3.42	4.04
OsR498G0816603500.01	271.863	83.913	-1.696916	0	0	264.38	272.57	278.64	80.97	82.8	87.97
OsR498G0816604000.01	0.046	0.21	2.2598122	1.83E-06	3.24E-06	0.02	0.07	0.05	0.21	0.21	0.21
OsR498G0816605000.01	34.773	96.773	1.4728972	0	0	36.92	33.66	33.74	97.14	95.11	98.07
OsR498G0816605500.01	30.123	99.853	1.7279173	0	0	31.14	30.24	28.99	102.83	100.94	95.79
OsR498G0816606000.01	3.166	8.346	1.39233	1.54E-22	1.76E-22	3.34	3.54	2.62	7.87	8.73	8.44
OsR498G0816606100.01	16.88	88.413	2.3932067	0	0	16.22	17.34	17.08	90.85	83.09	91.3
OsR498G0816607900.01	0.556	1.463	1.3949856	1.25E-12	1.78E-12	0.44	0.74	0.49	1.3	1.62	1.47
OsR498G0816608100.01	0.483	2.883	2.5208983	4.39E-17	5.61E-17	0.62	0.44	0.39	3.13	2.47	3.05
OsR498G0816613300.01	3.383	0.99	-1.779167	4.54E-18	5.65E-18	3.34	4.64	2.17	0.69	1	1.28
OsR498G0816613600.01	2624.3	584.52	-2.168239	0	0	2664.5	2534	2674.4	572.38	556.63	624.55
OsR498G0816615600.01	1.146	0.13	-3.175337	5.89E-27	6.25E-27	1.14	1.18	1.12	0.13	0.13	0.13
OsR498G0816617200.01	20.53	127.52	2.7488956	0	0	22.9	19.51	19.18	129.48	128.73	124.36
OsR498G0816618000.01	0.16	0.713	2.144335	6.84E-08	1.14E-07	0.12	0.28	0.08	0.9	0.43	0.81
OsR498G0816631800.01	10.476	24.476	1.1946123	9.97E-184	3.20E-184	10.79	11.09	9.55	24.47	25.37	23.59
OsR498G0816632600.01	0.203	0	-5.02559	1.85E-05	3.43E-05	0.31	0.15	0.15	0	0	0
OsR498G0916647100.01	1.363	3.453	1.3685501	9.09E-20	1.10E-19	1.59	1.04	1.46	3.86	3.59	2.91
OsR498G0916648300.01	0.586	2.116	1.8199	7.72E-23	8.76E-23	0.58	0.59	0.59	2.08	2.04	2.23
OsR498G0916654600.01	3.433	11.86	1.041999	4.44E-30	4.47E-30	2.35	3.83	4.12	10.32	11.68	13.58
OsR498G0916657400.01	8.01	18.426	1.2241978	2.70E-95	1.40E-95	7.87	7.77	8.39	18.51	18.31	18.46
OsR498G0916666100.01	3.283	7.266	1.1321602	1.18E-25	1.28E-25	3.58	3.04	3.23	7.15	7.91	6.74
OsR498G0916670000.01	14.936	30.386	1.0214353	8.54E-80	4.99E-80	14.75	15.88	14.18	30.47	29.44	31.25
OsR498G0916674400.01	23.553	357.22	3.9223185	0	0	24.31	23.19	23.16	359.5	358.09	354.08
OsR498G0916674500.01	9.17	20.096	1.130848	9.55E-83	5.47E-83	8.52	9.61	9.38	20.35	19.18	20.76
OsR498G0916678000.01	0.373	0.05	-2.832945	2.58E-07	4.41E-07	0.52	0.38	0.22	0.06	0.06	0.03
OsR498G0916681000.01	0.846	3.083	1.8619352	2.87E-12	4.13E-12	0.85	1.1	0.59	2.81	2.96	3.48
OsR498G0916691400.01	32.316	80.786	1.3208763	0	0	33.59	32.96	30.4	79.6	83.36	79.4
OsR498G0916692600.01	65.54	363.63	2.5081416	0	0	67.92	64.36	64.34	363.3	347.25	380.34
OsR498G0916693000.01	2.283	5.703	1.3383456	2.86E-28	2.97E-28	2.4	2.13	2.32	4.92	6.12	6.07
OsR498G0916693600.01	2.513	1.32	-1.02559	0.0004328	0.0008736	2.65	2.09	2.8	0.7	1.08	2.18
OsR498G0916696500.01	2.163	5.01	1.2094068	1.57E-49	1.21E-49	2.33	2.17	1.99	5.42	4.96	4.65
OsR498G0916716100.01	2.62	0.163	-4.02559	2.23E-15	2.96E-15	2.59	3.07	2.2	0	0.12	0.37
OsR498G0916724700.01	0.183	0.55	1.5593725	0.0004212	0.0008495	0.2	0.2	0.15	0.35	0.69	0.61
OsR498G0916727400.01	0.546	1.333	1.2856116	2.13E-05	3.97E-05	0.67	0.73	0.24	1.72	1.12	1.16
OsR498G0916732700.01	0.186	0.006	-5.02559	2.95E-09	4.67E-09	0.16	0.24	0.16	0.02	0	0
OsR498G0916736800.01	0.303	2.66	3.1379087	2.51E-40	2.17E-40	0.29	0.29	0.33	2.76	2.75	2.47
OsR498G0916742300.01	0.436	1.253	1.5859093	8.17E-16	1.07E-15	0.41	0.59	0.31	1.46	1.24	1.06
OsR498G0916742900.01	5.51	1.876	-1.506216	6.79E-36	6.25E-36	6.26	5.35	4.92	1.93	1.99	1.71
OsR498G0916743300.01	1.066	6.84	2.6731924	1.62E-65	1.06E-65	0.77	1.04	1.39	6.82	6.71	6.99
OsR498G0916747900.01	1.523	5.073	1.7329526	6.50E-23	7.38E-23	1.32	1.69	1.56	5.2	4.75	5.27
OsR498G0916751800.01	7.973	3.816	-1.099368	1.12E-16	1.45E-16	9.38	7.19	7.35	4.67	3.57	3.21
OsR498G0916757600.01	2.34	4.82	1.0412848	4.71E-13	6.63E-13	3.24	1.89	1.89	4.79	4.13	5.54
OsR498G0916761500.01	6.096	14.013	1.2142616	2.73E-64	1.81E-64	6.28	6.86	5.15	13.49	14.49	14.06
OsR498G0916766100.01	3.4	9.923	1.5342503	8.43E-173	2.84E-173	3.05	3.53	3.62	10.04	10.74	8.99
OsR498G0916781000.01	44.596	215.55	2.2271246	0	0	45.23	43.41	45.15	215.53	209.55	221.58
OsR498G0916800400.01	0.016	0.266	3.9700048	8.80E-06	1.61E-05	0	0.05	0	0.26	0.37	0.17
OsR498G0916804200.01	0.37	0.903	1.2568097	2.46E-11	3.65E-11	0.39	0.36	0.36	0.88	0.75	1.08
OsR498G0916818100.01	0.133	0.01	-3.610553	0.0004877	0.0009887	0.13	0.07	0.2	0	0.03	0
OsR498G0916823600.01	0.713	0.27	-1.404102	4.89E-05	9.30E-05	0.7	0.74	0.7	0.28	0.24	0.29
OsR498G0916834900.01	0.236	0.596	1.340942	8.26E-08	1.38E-07	0.2	0.33	0.18	0.63	0.64	0.52
OsR498G0916837100.01	7.223	19.766	1.4416229	1.63E-65	1.07E-65	6.62	6.91	8.14	21.73	18.06	19.51
OsR498G0916838100.01	2.003	9.36	2.2093658	4.38E-83	2.50E-83	1.85	2.28	1.88	9.29	10.58	8.21
OsR498G0916838900.01	6.193	18.536	1.5822081	3.32E-135	1.35E-135	5.94	6.26	6.38	19.24	17.61	18.76
OsR498G0916851000.01	29.29	100.71	1.815985	0	0	31.53	27.92	28.42	102.52	96.89	102.71
OsR498G0916873200.01	2.126	5.6	1.3928333	6.75E-46	5.43E-46	1.98	2.22	2.18	5.41	5.59	5.8
OsR498G0916876400.01	0.13	0.306	1.2671917	0.0002569	0.0005107	0.14	0.17	0.08	0.35	0.34	0.23
OsR498G0916878800.01	12.753	90.723	2.8308238	0	0	12.41	13.52	12.33	90.25	94.41	87.51
OsR498G0916879300.01	16.2	6.796	-1.366134	3.85E-121	1.69E-121	15.69	16.09	16.82	6.96	6.65	6.78
OsR498G0916884000.01	150.04	464.4	1.6302308	0	0	139.87	156.99	153.26	450.34	462.91	479.96
OsR498G0916885600.01	2.983	0.39	-2.900059	5.75E-34	5.44E-34	2.83	4.08	2.04	0.5	0.48	0.19
OsR498G0916886400.01	80.546	195.72	1.2980283	0	0	84.41	77.53	79.7	192.43	192.77	201.97
OsR498G0916889000.01	0.136	0.04	-1.66302	0.0005716	0.0011637	0.09	0.13	0.19	0.07	0.04	0.01
OsR498G0916892200.01	1.436	0.606	-1.240603	1.20E-08	1.95E-08	1.5	1.35	1.46	0.74	0.51	0.57
OsR498G0916897000.01	8.753	0.05	-7.417907	8.08E-188	2.55E-188	8.99	8.55	8.72	0	0.06	0.09
OsR498G0916897600.01	10.18	4.593	-1.262431	4.84E-58	3.42E-58	10.04	10.94	9.56	4.55	4.43	4.8
OsR498G0916899900.01	1.106	2.993	1.4553	1.16E-18	1.43E-18	1.1	1.33	0.89	2.19	3.81	2.98
OsR498G0916906700.01	127.15	55.606	-1.194268	0	0	128.96	127.51	124.98	55.8	53.39	57.63
OsR498G0916907000.01	8.196	48.57	2.5729372	0	0	8.07	7.01	9.51	46.96	49.16	49.59

OsR498G0916926100.01	3.17	1.113	-1.508673	2.45E-19	2.98E-19	3.65	2.5	3.36	1.21	1.01	1.12
OsR498G0916929200.01	1.183	3.756	1.6676253	5.80E-22	6.70E-22	1.16	1.47	0.92	3.96	4.03	3.28
OsR498G0916942000.01	2.383	0.85	-1.480785	1.39E-13	1.92E-13	3.02	1.81	2.32	1.33	0.74	0.48
OsR498G0916953400.01	0.276	0.023	-3.610553	0.0004877	0.0009887	0.48	0.21	0.14	0	0	0.07
OsR498G0917003300.01	0.723	2.506	1.7511333	5.20E-22	6.00E-22	0.74	0.76	0.67	2.21	2.84	2.47
OsR498G0917003600.01	8.813	0.32	-4.78852	0	0	8.85	9.27	8.32	0.37	0.28	0.31
OsR498G0917004800.01	0.716	0.203	-2.113053	2.69E-15	3.58E-15	0.85	0.59	0.71	0.16	0.26	0.19
OsR498G0917010500.01	0.846	3.766	2.154116	2.23E-52	1.67E-52	0.91	0.74	0.89	3.72	3.62	3.96
OsR498G0917012300.01	12.103	28.26	1.2315323	7.28E-154	2.68E-154	13.09	11.76	11.46	29.4	27.62	27.76
OsR498G0917013200.01	2.263	7.006	1.6377725	1.88E-49	1.46E-49	1.95	2.64	2.2	6.29	7.29	7.44
OsR498G0917037300.01	12.81	5.313	-1.269374	1.14E-17	1.43E-17	13.52	11.41	13.5	5.27	6.23	4.44
OsR498G0917039600.01	2.75	204.37	6.2209809	0	0	2.21	2.61	3.43	207.63	200.92	204.55
OsR498G0917041300.01	0.14	0.833	2.5759367	8.82E-14	1.22E-13	0.15	0.15	0.12	0.61	0.81	1.08
OsR498G0917046000.01	0.486	1.38	1.5593725	1.08E-05	1.98E-05	0.56	0.35	0.55	1.24	1.47	1.43
OsR498G0917061100.01	0.156	3.886	4.6402925	1.92E-75	1.16E-75	0.07	0.18	0.22	4.21	3.7	3.75
OsR498G0917079600.01	3.07	12.656	2.0429692	9.67E-270	2.22E-270	2.72	3.32	3.17	13.49	12.26	12.22
OsR498G0917080900.01	1.386	2.986	1.1019234	2.95E-30	2.96E-30	1.23	1.53	1.4	2.92	3.01	3.03
OsR498G0917083400.01	18.533	42.826	1.2080677	1.62E-163	5.75E-164	19.25	17.1	19.25	45.09	41.05	42.34
OsR498G0917088600.01	6.653	31.426	2.2378341	0	0	6.77	6.95	6.24	31.31	33.18	29.79
OsR498G0917090600.01	7.726	17.3	1.1610605	8.10E-141	3.19E-141	7.14	8.18	7.86	17.41	16.44	18.05
OsR498G0917147300.01	0.433	0.993	1.2038918	4.45E-05	8.42E-05	0.45	0.4	0.45	1.13	1.09	0.76
OsR498G0917151200.01	3.15	38.476	3.6163572	0	0	3.24	3.11	3.1	39.42	37.19	38.82
OsR498G0917156200.01	0.07	0.996	3.7817649	8.22E-16	1.08E-15	0.04	0.04	0.13	0.85	1.01	1.13
OsR498G0917159900.01	1.093	3.193	1.5469887	1.17E-18	1.44E-18	1.1	1.21	0.97	3.32	3.1	3.16
OsR498G0917161200.01	0.006	0.07	3.5593725	0.0005724	0.0011661	0.02	0	0	0.12	0.05	0.04
OsR498G0917161500.01	27.29	57.923	1.0847477	4.67E-113	2.16E-113	27.8	27.13	26.94	54.29	58.69	60.79
OsR498G0917161700.01	36.693	75.556	1.0399627	0	0	37.83	36.01	36.24	77.93	76.08	72.66
OsR498G0917162200.01	0.343	1.873	2.4457157	4.99E-18	6.23E-18	0.24	0.42	0.37	2.2	2.1	1.32
OsR498G0917162400.01	0.066	0.603	3.4909855	3.13E-21	3.68E-21	0.1	0.05	0.05	0.49	0.62	0.7
OsR498G0917162500.01	6.18	20.81	1.7843935	5.28E-148	2.00E-148	5.84	5.94	6.76	21.77	20.48	20.18
OsR498G0917163000.01	1.63	6.07	1.9011488	4.45E-115	2.03E-115	1.69	1.62	1.58	5.69	6.71	5.81
OsR498G0917163400.01	13.196	36.293	1.4584435	2.47E-180	8.07E-181	13.6	12.99	13	36.24	37.02	35.62
OsR498G0917172500.01	0.69	0.023	-4.908233	1.67E-15	2.21E-15	0.78	0.59	0.7	0.03	0	0.04
OsR498G0917182100.01	5.326	43.21	3.0126267	0	0	5.35	5.91	4.72	42.3	44.31	43.02
OsR498G0917182700.01	0.193	0.866	1.7031079	1.82E-11	2.68E-11	0.17	0.15	0.26	0.72	0.94	0.94
OsR498G0917187600.01	2.27	1.003	-1.180868	1.55E-06	2.73E-06	2.44	1.87	2.5	0.61	1.22	1.18
OsR498G0917189500.01	1.266	0.45	-1.33818	5.97E-11	8.96E-11	1.28	1.11	1.41	0.52	0.32	0.51
OsR498G0917194500.01	0.333	0.043	-2.951589	4.72E-08	7.83E-08	0.37	0.34	0.29	0.1	0.03	0
OsR498G0917195700.01	2.033	0.753	-1.427688	6.06E-14	8.34E-14	2.32	1.84	1.94	1.03	0.7	0.53
OsR498G0917198400.01	0.04	0.416	3.2474285	6.71E-07	1.17E-06	0.04	0.04	0.04	0.47	0.3	0.48
OsR498G0917198600.01	21.25	3.766	-2.786748	1.03E-231	2.71E-232	20.97	20.96	21.82	4.84	3.02	3.44
OsR498G0917203100.01	5.94	1.833	-1.778367	6.68E-29	6.84E-29	5.87	6.22	5.73	2.17	2.02	1.31
OsR498G0917205200.01	0.013	0.113	2.97441	0.0002639	0.0005255	0	0.02	0.02	0.04	0.15	0.15
OsR498G0917207600.01	0.853	1.806	1.1387968	1.18E-07	1.98E-07	0.6	0.57	1.39	1.85	2.25	1.32
OsR498G0917209900.01	1.306	6.39	2.2921196	1.05E-85	5.88E-86	1.44	1.34	1.14	7.08	6.41	5.68
OsR498G0917211200.01	32.083	74.676	1.2163984	0	0	33.05	31.68	31.52	74.88	74.82	74.33
OsR498G0917211500.01	0.96	1.96	1.0306782	7.86E-09	1.26E-08	1.02	0.75	1.11	1.82	2.45	1.61
OsR498G0917216200.01	0.14	0.013	-4.485022	1.10E-06	1.93E-06	0.1	0.17	0.15	0.04	0	0
OsR498G0917225800.01	3.423	11.203	1.6980991	2.77E-109	1.31E-109	3.41	3.4	3.46	10.27	11.59	11.75
OsR498G0917227800.01	7.996	0.74	-3.428906	3.29E-225	8.88E-226	8.41	8.07	7.51	0.69	0.7	0.83
OsR498G0917236300.01	6.716	1.796	-1.909148	3.58E-70	2.25E-70	6.53	6.9	6.72	1.87	2.03	1.49
OsR498G0917237700.01	0.213	0.793	1.883703	1.99E-10	3.04E-10	0.19	0.16	0.29	0.95	0.57	0.86
OsR498G0917242100.01	22.22	1.496	-3.915186	0	0	23.23	21.34	22.09	1.82	1.41	1.26
OsR498G0917242800.01	1.583	3.603	1.1968024	1.40E-16	1.80E-16	1.46	1.48	1.81	4.18	3.55	3.08
OsR498G0917247600.01	9.186	40.07	2.19263	0	0	8.77	9.62	9.17	39.6	39.93	40.68
OsR498G0917251900.01	23.756	102.32	2.0964451	0	0	23.66	23.12	24.49	104.71	101.39	100.85
OsR498G0917253500.01	1.373	3.64	1.3883056	8.10E-28	8.47E-28	1.82	1.09	1.21	3.81	3.37	3.74
OsR498G0917255100.01	0.063	0.553	3.103693	9.95E-08	1.67E-07	0	0.19	0	0.47	0.56	0.63
OsR498G0917259300.01	2.266	0.96	-1.242549	7.48E-16	9.81E-16	2.12	2.04	2.64	0.75	1.2	0.93
OsR498G0917260400.01	0	0.203	5.5593725	2.32E-07	3.95E-07	0	0	0	0.18	0.25	0.18
OsR498G0917282000.01	3.226	14.94	2.1760438	2.79E-134	1.15E-134	2.89	3.48	3.31	16.67	13.81	14.34
OsR498G0917283500.01	2.253	17.293	2.8916358	2.55E-246	6.42E-247	2.29	2.1	2.37	16.7	16.26	18.92
OsR498G0917285100.01	1.56	12.486	3.0009708	2.89E-200	8.67E-201	1.65	1.54	1.49	12.36	13.02	12.08
OsR498G0917285900.01	0.353	1.39	1.97441	4.33E-16	5.65E-16	0.4	0.39	0.27	1.92	1.12	1.13
OsR498G0917289300.01	8.573	21.143	1.266246	5.69E-116	2.58E-116	8.63	8.57	8.52	20.73	21.05	21.65
OsR498G0917291500.01	1.633	4.83	1.4992515	2.29E-21	2.68E-21	1.35	1.54	2.01	4.66	5.14	4.69
OsR498G0917296000.01	0.383	0.01	-4.72603	1.01E-07	1.69E-07	0.67	0.18	0.3	0	0	0.03
OsR498G0917309800.01	1.243	4.333	1.7947916	1.87E-81	1.09E-81	1.04	1.22	1.47	4.45	4.58	3.97
OsR498G0917313200.01	0.7	2.16	1.6297618	2.13E-31	2.10E-31	0.74	0.68	0.68	2.16	2.27	2.05

OsR498G0917314300.01	2.673	8.976	1.7485675	3.37E-48	2.65E-48	3.08	2.49	2.45	8.41	9.37	9.15
OsR498G0917315900.01	0.376	0.12	-1.673288	1.29E-05	2.38E-05	0.34	0.48	0.31	0.05	0.14	0.17
OsR498G0917320700.01	0.326	1.036	1.7687429	3.46E-15	4.61E-15	0.46	0.14	0.38	0.9	0.99	1.22
OsR498G0917327200.01	1.53	3.776	1.3015747	1.66E-09	2.61E-09	1.51	1.5	1.58	3.66	3.41	4.26
OsR498G0917330300.01	0.19	0	-4.347518	0.0007014	0.0014405	0.06	0.28	0.23	0	0	0
OsR498G0917339500.01	0.48	1.526	1.6683068	3.43E-06	6.14E-06	0.68	0.08	0.68	1.73	1.07	1.78
OsR498G0917340000.01	6.336	3.07	-1.045656	6.92E-31	6.88E-31	6.94	5.77	6.3	3.38	2.93	2.9
OsR498G0917343800.01	1.453	0.17	-3.084484	4.77E-06	8.63E-06	0.88	1.91	1.57	0	0.51	0
OsR498G0917367500.01	16.373	47.22	1.5259328	0	0	17.22	15.83	16.07	48.2	47.44	46.02
OsR498G0917376000.01	1.173	4.34	1.8813006	3.20E-46	2.57E-46	1.17	1.13	1.22	4.27	4.21	4.54
OsR498G0917389500.01	1.113	19.163	4.0907151	2.50E-245	6.33E-246	0.95	1.29	1.1	18.74	18.37	20.38
OsR498G0917393900.01	1.246	0.286	-2.160016	1.58E-34	1.48E-34	1.26	1.42	1.06	0.32	0.27	0.27
OsR498G0917394900.01	4.536	9.77	1.1237389	1.44E-49	1.11E-49	4.74	4.74	4.13	10.31	9.84	9.16
OsR498G0917406000.01	0.963	6.173	2.6826166	2.98E-111	1.39E-111	1.12	0.93	0.84	6.39	6.19	5.94
OsR498G0917407400.01	93.786	201.53	1.110272	2.39E-291	5.16E-292	91.32	97.93	92.11	203.65	199.17	201.76
OsR498G0917410000.01	81.276	230.75	1.5059578	0	0	79.74	80.27	83.82	227.35	231.42	233.49
OsR498G0917411600.01	8.106	20	1.300758	4.53E-102	2.24E-102	6.96	8.27	9.09	18.7	20.33	20.97
OsR498G0917415600.01	1.83	0.893	-1.039665	7.87E-06	1.44E-05	1.85	1.8	1.84	0.72	0.9	1.06
OsR498G0917422300.01	0.92	2.29	1.3165159	1.50E-32	1.44E-32	0.81	0.85	1.1	2.39	2.35	2.13
OsR498G0917427900.01	1.68	8.496	2.3332033	3.46E-111	1.62E-111	1.67	1.74	1.63	8.52	8.08	8.89
OsR498G0917435800.01	0.056	0.22	1.97441	0.0001498	0.0002935	0.1	0.07	0	0.29	0.12	0.25
OsR498G0917436000.01	10.85	25.73	1.2296916	1.66E-98	8.43E-99	10.73	11.29	10.53	24.97	25.08	27.14
OsR498G0917437800.01	5.126	12.246	1.255649	3.42E-69	2.17E-69	5.19	5.21	4.98	11.36	12.07	13.31
OsR498G0917438000.01	28.483	260.95	3.1906407	0	0	30.23	24.04	31.18	260.51	241.08	281.27
OsR498G0917438600.01	1.44	0.09	-4.02559	2.23E-15	2.96E-15	1.36	1.61	1.35	0.13	0.07	0.07
OsR498G0917439900.01	0.446	0.973	1.1305292	3.29E-05	6.19E-05	0.54	0.46	0.34	0.82	1.45	0.65
OsR498G0917442200.01	7.93	40.163	2.3387041	2.58E-197	7.81E-198	7.71	8.46	7.62	40.24	39.59	40.66
OsR498G0917445700.01	2.156	0.666	-1.791125	2.73E-17	3.47E-17	2.11	1.72	2.64	0.69	0.63	0.68
OsR498G0917447400.01	0.646	0.026	-4.517443	6.27E-12	9.12E-12	0.39	0.56	0.99	0.04	0	0.04
OsR498G0917449000.01	30.04	6.556	-2.242133	2.63E-231	6.96E-232	32.78	28.77	28.57	5.53	6.54	7.6
OsR498G0917453500.01	31.976	122.62	1.9286803	0	0	30.95	31.38	33.6	122.67	120.43	124.77
OsR498G0917456300.01	1.323	2.93	1.1485844	1.60E-13	2.22E-13	1.2	1.4	1.37	3.42	2.52	2.85
OsR498G0917460800.01	1.41	0.36	-1.826281	6.75E-12	9.83E-12	1.44	1.01	1.78	0.54	0.25	0.29
OsR498G0917462400.01	0.58	2.27	1.97441	8.36E-22	9.70E-22	0.43	0.6	0.71	2.59	2.09	2.13
OsR498G0917463600.01	0.336	1.096	1.6695554	2.86E-07	4.89E-07	0.24	0.31	0.46	1.1	0.89	1.3
OsR498G0917465900.01	1.873	0.72	-1.394824	1.23E-09	1.91E-09	1.69	2.03	1.9	0.49	0.44	1.23
OsR498G0917467800.01	239.09	88.79	-1.434005	0	0	244.24	229.99	243.04	85.65	86.51	94.21
OsR498G0917467900.01	6.586	3.21	-1.133889	3.85E-40	3.35E-40	6.58	6.3	6.88	3.44	3.16	3.03
OsR498G0917469500.01	0.126	0.73	2.5149783	1.42E-10	2.16E-10	0.14	0.07	0.17	0.71	0.61	0.87
OsR498G0917472200.01	0.146	0.49	1.5593725	0.0004212	0.0008495	0.15	0.11	0.18	0.45	0.45	0.57
OsR498G0917472900.01	3.473	8.42	1.2759455	3.79E-20	4.53E-20	3.97	3.19	3.26	8.98	7.68	8.6
OsR498G0917475600.01	0.316	0.026	-3.549152	9.84E-11	1.49E-10	0.23	0.25	0.47	0.04	0	0.04
OsR498G0917476600.01	0.03	0.25	2.97441	0.0002639	0.0005255	0	0.09	0	0.23	0.14	0.38
OsR498G0917477200.01	7.873	41.246	2.3886034	0	0	7.72	7.47	8.43	42.6	38.46	42.68
OsR498G0917478800.01	0.29	1.733	2.5879416	1.74E-23	1.95E-23	0.21	0.21	0.45	1.79	1.54	1.87
OsR498G0917479700.01	0.243	0.006	-5.485022	3.05E-12	4.39E-12	0.22	0.18	0.33	0	0	0.02
OsR498G0917484800.01	0.816	4.106	2.3297142	4.39E-62	2.98E-62	0.68	0.73	1.04	3.91	4.15	4.26
OsR498G0917486500.01	2.62	0.523	-2.323271	2.69E-09	4.26E-09	2.54	3.33	1.99	0.78	0.52	0.27
OsR498G0917487700.01	3.47	1.57	-1.256916	2.23E-16	2.89E-16	3.99	3.1	3.32	1.37	1.69	1.65
OsR498G0917488500.01	22.183	75.14	1.7497644	0	0	22.19	22.42	21.94	74.03	71.2	80.19
OsR498G0917489000.01	0.813	8.91	3.4016911	1.67E-132	6.90E-133	0.78	0.89	0.77	9.55	8.67	8.51
OsR498G0917490000.01	14.25	1.89	-2.921603	2.60E-174	8.70E-175	14.8	14.78	13.17	1.55	2.09	2.03
OsR498G0917491900.01	1.666	0.376	-2.14559	2.03E-26	2.17E-26	1.7	1.35	1.95	0.48	0.27	0.38
OsR498G0917499400.01	2.416	10.876	2.1537337	6.84E-212	1.95E-212	2.51	2.33	2.41	10.63	11.32	10.68
OsR498G0917502000.01	4.69	10.336	1.0243149	6.27E-40	5.47E-40	4.61	4.53	4.93	10.94	10.39	9.68
OsR498G0917508100.01	0.106	1.083	3.3513794	6.98E-36	6.43E-36	0.04	0.12	0.16	1.12	0.8	1.33
OsR498G0917508700.01	2.706	16.916	2.740747	0	0	2.32	3.06	2.74	15.91	18.02	16.82
OsR498G0917512100.01	0.606	0.113	-2.404102	3.67E-05	6.92E-05	0.49	0.63	0.7	0.2	0.14	0
OsR498G0917515100.01	2.336	32.07	3.7800928	0	0	2.06	2.15	2.8	33.32	30.79	32.1
OsR498G0917518600.01	0.22	1.85	3.0771467	2.88E-18	3.58E-18	0.18	0.18	0.3	1.75	1.66	2.14
OsR498G0917518800.01	0.09	0.006	-3.485022	0.000899	0.0018658	0.17	0.05	0.05	0.02	0	0
OsR498G0917519700.01	121.456	33.693	-1.851718	0	0	121.09	116.73	126.55	34.28	30.8	36
OsR498G0917520500.01	297.286	39.173	-2.97118	0	0	283.78	305.06	303.02	38.18	38.02	41.32
OsR498G0917526600.01	0.633	1.396	1.1880054	4.85E-07	8.38E-07	0.8	0.36	0.74	1.28	1.24	1.67
OsR498G0917527500.01	4.546	21.71	2.2519572	0	0	4.7	4.41	4.53	22.05	21.89	21.19
OsR498G0917527700.01	54.896	125.7	1.2317317	0	0	58.12	54.37	52.2	126.35	128.09	122.65
OsR498G0917531100.01	4.633	2.056	-1.257154	5.27E-16	6.89E-16	5.25	4.87	3.78	1.69	2.39	2.09
OsR498G0917535200.01	4.613	38.25	3.063076	0	0	5.08	4.65	4.11	37.96	36.42	40.37
OsR498G0917535900.01	6.71	3.033	-1.252795	3.83E-66	2.50E-66	7.66	5.9	6.57	3.53	2.43	3.14

OsR498G0917537200.01	0.786	1.62	1.0397014	3.72E-06	6.67E-06	0.66	0.74	0.96	1.75	1.57	1.54
OsR498G0917537500.01	3.813	10.506	1.4473458	8.60E-76	5.17E-76	3.55	3.68	4.21	9.87	10.6	11.05
OsR498G0917538300.01	3.033	6.586	1.118887	3.79E-40	3.30E-40	2.95	3.17	2.98	6.64	6.77	6.35
OsR498G0917543500.01	11.976	25.17	1.0688183	3.66E-41	3.14E-41	13.84	11.07	11.02	26.01	23.51	25.99
OsR498G0917547300.01	0.216	0.033	-3.195515	6.51E-05	0.0001248	0.1	0.32	0.23	0	0.05	0.05
OsR498G0917550300.01	42.543	170.22	1.9910312	0	0	42.17	42.9	42.56	173.06	169.99	167.6
OsR498G0917550700.01	41.406	203.83	2.2953462	0	0	39.86	41.86	42.5	207.59	200.86	203.03
OsR498G0917551100.01	1.363	3.586	1.3316268	2.08E-17	2.64E-17	1.43	1.1	1.56	3.98	3.61	3.17
OsR498G0917551300.01	3.49	8.216	1.2320911	5.48E-57	3.91E-57	3.65	3.53	3.29	7.93	8.74	7.98
OsR498G0917555100.01	0.873	0.206	-2.117513	5.34E-11	8.00E-11	0.88	1.13	0.61	0.26	0.23	0.13
OsR498G0917557600.01	0.486	4.453	3.1861551	1.09E-56	7.83E-57	0.43	0.34	0.69	4.34	4.99	4.03
OsR498G0917566900.01	23.826	5.146	-2.21033	0	0	22.77	23.92	24.79	4.77	5.13	5.54
OsR498G0917567800.01	2.85	0.616	-2.216274	4.54E-27	4.81E-27	3.03	2.45	3.07	0.59	0.74	0.52
OsR498G0917573900.01	1.15	2.856	1.3571297	2.13E-26	2.28E-26	1.28	1.16	1.01	3.29	2.52	2.76
OsR498G0917580500.01	9.433	3.76	-1.284611	8.87E-82	5.11E-82	9.08	9.6	9.62	3.9	4.24	3.14
OsR498G0917581500.01	9.286	1.746	-2.394824	5.26E-59	3.68E-59	10.95	8.36	8.55	1.72	1.33	2.19
OsR498G0917587500.01	1.136	0.38	-1.578131	4.66E-05	8.83E-05	0.93	1.24	1.24	0.45	0.38	0.31
OsR498G0917588300.01	4.956	1.856	-1.414018	6.13E-26	6.61E-26	5.03	4.11	5.73	1.99	1.94	1.64
OsR498G0917590400.01	4.653	11.003	1.241514	1.09E-38	9.67E-39	5.14	4.05	4.77	10.94	11.49	10.58
OsR498G0917591100.01	30.843	10.433	-1.576283	8.24E-191	2.56E-191	30.42	31.36	30.75	9.62	10.87	10.81
OsR498G0917592600.01	8.783	36.816	2.0672781	0	0	9.31	9	8.04	36.9	36.87	36.68
OsR498G0917593800.01	13.486	84.25	2.6485441	0	0	13.7	14.03	12.73	83.91	82.82	86.02
OsR498G0917594200.01	2.633	14.403	2.3637135	2.97E-169	1.02E-169	2.35	3.16	2.39	14.19	14.25	14.77
OsR498G0917595200.01	8.583	3.51	-1.29197	1.65E-45	1.34E-45	9.23	9.09	7.43	3.62	2.98	3.93
OsR498G0917598500.01	6.463	17.176	1.4006747	1.14E-71	7.07E-72	6.27	6.56	6.56	17.62	16.62	17.29
OsR498G0917599400.01	0.106	0.233	1.1296882	0.000734	0.0015102	0.13	0.09	0.1	0.28	0.21	0.21
OsR498G0917601600.01	0.4	1.233	1.6182661	1.41E-07	2.39E-07	0.45	0.55	0.2	1.32	1.07	1.31
OsR498G0917604800.01	4.823	31.483	2.7046116	1.93E-306	4.02E-307	5.4	4.27	4.8	30.18	29.23	35.04
OsR498G0917605400.01	4.01	1.733	-1.210015	3.08E-13	4.31E-13	3.71	4.21	4.11	1.46	2.08	1.66
OsR498G0917606100.01	0.136	0.013	-3.273518	3.60E-05	6.79E-05	0.26	0.13	0.02	0	0	0.04
OsR498G0917608700.01	3.37	56.06	4.0669876	0	0	3.42	3.2	3.49	56.48	51.77	59.93
OsR498G0917615600.01	11.52	3.336	-1.788449	6.31E-41	5.42E-41	11.76	11.19	11.61	3.05	3.35	3.61
OsR498G0917615700.01	0.13	0.01	-3.932481	7.73E-05	0.0001487	0.18	0.13	0.08	0	0	0.03
OsR498G0917619800.01	0.12	1.056	3.1250473	2.86E-13	3.99E-13	0.11	0.2	0.05	1.22	1.08	0.87
OsR498G0917621300.01	11.706	4.256	-1.459271	2.64E-71	1.64E-71	12.78	11.34	11	4.8	3.75	4.22
OsR498G0917622100.01	94.733	27.336	-1.75852	0	0	100.68	88.99	94.53	26.2	26.75	29.06
OsR498G0917623600.01	1.97	0.086	-4.503637	7.47E-37	6.78E-37	2.09	2	1.82	0.04	0.18	0.04
OsR498G0917625800.01	3.913	9.79	1.3209337	5.75E-34	5.44E-34	4	3.65	4.09	9.27	9.03	11.07
OsR498G0917629000.01	0.15	0.05	-1.561279	0.0002318	0.0004592	0.17	0.15	0.13	0.07	0.07	0.01
OsR498G0917635400.01	150.023	369.54	1.2869539	0	0	148.86	156.26	144.95	377.09	369.66	361.86
OsR498G0917641400.01	0.32	0.883	1.4400735	1.65E-05	3.05E-05	0.18	0.23	0.55	0.63	0.96	1.06
OsR498G0917645800.01	4.66	1.586	-1.556465	5.69E-35	5.30E-35	4.33	5.25	4.4	1.56	1.37	1.83
OsR498G0917648500.01	53.316	287.19	2.4454595	0	0	53.65	52.74	53.56	278.34	277.5	305.72
OsR498G0917648800.01	0.206	1.333	2.6748497	3.58E-15	4.78E-15	0.13	0.36	0.13	1.18	1.43	1.39
OsR498G0917649000.01	10.3	29.32	1.5536785	3.44E-149	1.30E-149	9.84	10.31	10.75	28.37	28.23	31.36
OsR498G0917651200.01	1.47	3.506	1.2559803	9.65E-17	1.24E-16	1.34	1.21	1.86	3.52	3.46	3.54
OsR498G0917654500.01	5.716	1.42	-1.997576	4.78E-34	4.51E-34	5.5	5.08	6.57	1.36	1.54	1.36
OsR498G0917656700.01	9.923	4.01	-1.31144	9.70E-60	6.71E-60	10.6	9.17	10	3.83	4.27	3.93
OsR498G0917661300.01	1.79	0.343	-2.901621	8.07E-13	1.14E-12	2.14	1.67	1.56	0.23	0.57	0.23
OsR498G0917663600.01	1.186	5.383	2.1859141	2.08E-83	1.19E-83	1.04	1.55	0.97	6.06	4.65	5.44
OsR498G0917664100.01	1.44	6.133	2.0839623	1.11E-116	5.03E-117	1.71	1.23	1.38	6.41	6.2	5.79
OsR498G0917665300.01	0.223	14.063	5.9720528	0	0	0.14	0.21	0.32	12.87	14.61	14.71
OsR498G0917669600.01	1.02	1.603	1.0021017	1.78E-11	2.63E-11	1.04	0.87	1.15	1.46	1.64	1.71
OsR498G0917669900.01	3.643	0.286	-3.664629	1.46E-31	1.44E-31	3.52	3.9	3.51	0.43	0.43	0
OsR498G0917672200.01	22.32	61.52	1.4643715	0	0	23.71	21.88	21.37	60.92	63.35	60.29
OsR498G0917677100.01	58.5	192.26	1.6916083	0	0	58.25	57.34	59.91	195.17	186.49	195.13
OsR498G0917677600.01	1.176	15.316	3.7828607	2.16E-264	5.02E-265	1.49	1.09	0.95	15.97	14.45	15.53
OsR498G0917683100.01	0.22	0.736	1.7550969	1.86E-13	2.59E-13	0.19	0.18	0.29	0.8	0.78	0.63
OsR498G0917683500.01	2.976	11.11	1.8911037	1.95E-129	8.19E-130	3.01	3.1	2.82	11.54	11.47	10.32
OsR498G0917684000.01	0.753	4.87	2.70984	8.78E-95	4.57E-95	0.77	0.92	0.57	4.42	5.13	5.06
OsR498G0917684200.01	30.073	87.58	1.549369	0	0	29.35	30.3	30.57	89.57	87.09	86.08
OsR498G0917686300.01	3.993	1.766	-1.181385	1.16E-26	1.23E-26	3.96	4	4.02	1.9	1.77	1.63
OsR498G0917687600.01	1.92	4.27	1.1492842	7.15E-12	1.04E-11	1.55	2.01	2.2	4.58	3.64	4.59
OsR498G0917691400.01	0.246	0.603	1.2963381	2.74E-09	4.33E-09	0.22	0.23	0.29	0.57	0.69	0.55
OsR498G0917697100.01	1.766	0.433	-2.02559	7.44E-29	7.63E-29	1.88	1.59	1.83	0.37	0.51	0.42
OsR498G0917697300.01	3262.99	886.61	-1.881995	0	0	3253.3	3203	3332.7	868.39	828.54	962.91
OsR498G0917700000.01	7.866	3.226	-1.29088	1.36E-30	1.35E-30	8.17	7.45	7.98	2.93	3.23	3.52
OsR498G0917701900.01	0.023	0.606	4.7914916	1.04E-24	1.15E-24	0	0	0.07	0.38	0.58	0.86
OsR498G0917703700.01	1.99	16.476	3.0520002	0	0	2	2.11	1.86	18.01	15.85	15.57

OsR498G0917705000.01	0.363	4.96	3.5755282	7.96E-88	4.38E-88	0.45	0.41	0.23	4.6	5.73	4.55
OsR498G0917707100.01	1.89	8.78	2.2223375	1.40E-35	1.30E-35	2.26	1.98	1.43	8.71	9.4	8.23
OsR498G0917709100.01	0.843	6.376	2.9145986	1.13E-54	8.24E-55	1.06	0.53	0.94	6.47	6.65	6.01
OsR498G0917709600.01	0.926	3.003	1.696035	3.14E-30	3.16E-30	0.59	1.05	1.14	2.53	3.49	2.99
OsR498G0917713300.01	6.73	103.99	3.9445274	0	0	7.36	6.45	6.38	103.51	100.58	107.89
OsR498G0917716600.01	27.486	127.08	2.2531743	0	0	25.4	26.21	30.85	132.16	119.95	129.14
OsR498G0917724400.01	0.2	0.653	1.5850308	9.37E-11	1.41E-10	0.1	0.28	0.22	0.68	0.69	0.59
OsR498G0917724700.01	0.403	1.206	1.5773018	3.36E-07	5.76E-07	0.34	0.58	0.29	1.25	1.11	1.26
OsR498G0917725400.01	10.833	28.786	1.3948418	0	0	11.31	10.7	10.49	30.01	28.2	28.15
OsR498G0917730800.01	2.343	5.97	1.3113143	1.12E-34	1.04E-34	2.55	2.4	2.08	5.64	6.54	5.73
OsR498G0917732800.01	0.943	26.973	4.8163831	0	0	0.88	1.04	0.91	26.85	26.7	27.37
OsR498G0917733200.01	2.453	16.68	2.7654554	0	0	2.6	2.46	2.3	16.91	17.24	15.89
OsR498G0917734400.01	0.426	0.97	1.1808608	0.0001407	0.0002753	0.3	0.59	0.39	0.96	0.86	1.09
OsR498G0917734800.01	141.656	28.973	-2.27222	0	0	145.92	139.8	139.25	28.94	29.38	28.6
OsR498G0917736100.01	0.296	1.153	1.97441	3.94E-18	4.90E-18	0.31	0.2	0.38	1.42	1.11	0.93
OsR498G0917737100.01	0.133	4.976	5.2159959	2.92E-54	2.14E-54	0.13	0.2	0.07	5.08	3.76	6.09
OsR498G0917738200.01	10.61	62.813	2.5683972	0	0	9.4	11.52	10.91	64.98	60.99	62.47
OsR498G0917738400.01	3.546	13.456	1.9191161	2.92E-187	9.23E-188	3.76	3.55	3.33	12.85	14.52	13
OsR498G0917739300.01	1.316	2.673	1.0229085	5.24E-11	7.84E-11	1.39	1.24	1.32	2.43	2.88	2.71
OsR498G0917742100.01	0.513	1.426	1.4691746	5.03E-08	8.34E-08	0.61	0.33	0.6	1.53	1.56	1.19
OsR498G0917744900.01	33.263	85.86	1.3611892	6.78E-301	1.43E-301	34.6	33.65	31.54	85.16	86.69	85.73
OsR498G0917745400.01	157.33	326.63	1.0802012	0	0	155.99	159.8	156.2	324.82	327.52	327.54
OsR498G0917747400.01	0.093	1.503	4.0618728	1.00E-26	1.07E-26	0.12	0.04	0.12	1.58	1.42	1.51
OsR498G0917748700.01	1.89	4.746	1.3045586	1.89E-31	1.86E-31	1.53	2.01	2.13	4.21	4.98	5.05
OsR498G0917756100.01	6.976	37.65	2.4284924	3.02E-185	9.64E-186	7.82	5.98	7.13	38.48	33.19	41.28
OsR498G0917757000.01	0.456	3.16	2.7702692	7.19E-40	6.28E-40	0.57	0.31	0.49	3.2	2.81	3.47
OsR498G0917757400.01	1.69	13.5	3.0046562	2.72E-81	1.58E-81	1.82	1.56	1.69	12.49	13.44	14.57
OsR498G0917757600.01	23.066	46.166	1.009998	1.29E-76	7.71E-77	21.93	24.44	22.83	46.31	47.18	45.01
OsR498G0917757800.01	7.72	44.176	2.5186194	2.32E-218	6.40E-219	9.04	7.26	6.86	44.39	42.41	45.73
OsR498G0917759700.01	0.12	3.933	5.0114993	1.94E-69	1.22E-69	0.16	0.12	0.08	4.11	3.67	4.02
OsR498G0917760000.01	0.183	0.49	1.412983	5.62E-05	0.0001073	0.12	0.26	0.17	0.52	0.33	0.62
OsR498G0917762300.01	9.21	20.94	1.1847915	1.83E-179	5.98E-180	8.24	10.59	8.8	21.23	21.35	20.24
OsR498G0917762600.01	0.623	2.326	1.8082276	1.30E-19	1.57E-19	0.72	0.46	0.69	2.42	1.98	2.58
OsR498G0917763200.01	0.163	0.59	1.8305887	4.22E-07	7.28E-07	0.25	0.12	0.12	0.51	0.48	0.78
OsR498G0917764600.01	60.83	140.55	1.1787614	0	0	56.71	63.74	62.04	130.41	146.44	144.79
OsR498G0917775000.01	8.89	62.16	2.8078646	0	0	9.33	8.9	8.44	63.51	59.19	63.78
OsR498G0917777200.01	17.28	38.18	1.1286854	4.59E-159	1.66E-159	16.74	19.29	15.81	39.16	38.73	36.65
OsR498G0917779100.01	25.736	75.903	1.5661536	0	0	24.4	26.26	26.55	75.31	74.24	78.16
OsR498G0917779300.01	24.256	60.873	1.3221862	0	0	23.57	24.51	24.69	59.31	63.91	59.4
OsR498G0917780400.01	3.986	52.19	3.723711	0	0	4.14	3.77	4.05	52.2	56.1	48.27
OsR498G0917780800.01	0.01	0.16	3.97441	5.14E-05	9.78E-05	0	0.03	0	0.3	0.12	0.06
OsR498G0917782300.01	10.703	23.913	1.1623166	8.74E-69	5.56E-69	9.49	10.18	12.44	21.99	23.77	25.98
OsR498G0917787800.01	148.476	295.84	1.0039237	0	0	148.08	148.57	148.78	296.3	298.71	292.52
OsR498G0917788200.01	4.313	9.75	1.1595593	5.54E-44	4.56E-44	4.41	4.48	4.05	9.86	9.68	9.71
OsR498G0917791400.01	0.1	0.276	1.4584569	0.0001282	0.0002503	0.08	0	0.22	0.28	0.3	0.25
OsR498G0917791700.01	2.756	16.7	2.5394848	6.48E-231	1.72E-231	3.05	2.51	2.71	16.8	17.64	15.66
OsR498G0917792400.01	8.066	0.993	-3.083717	5.38E-172	1.82E-172	6.49	8.39	9.32	0.98	0.84	1.16
OsR498G0917792700.01	8.393	19.116	1.1723993	2.39E-82	1.37E-82	8.01	8.42	8.75	19.9	19.44	18.01
OsR498G0917793800.01	0.19	0.733	1.8438258	1.24E-10	1.88E-10	0.13	0.26	0.18	0.59	0.85	0.76
OsR498G0917794300.01	0.616	1.873	1.4598368	4.61E-12	6.68E-12	0.71	0.5	0.64	1.31	2.06	2.25
OsR498G0917794500.01	36.806	17.733	-1.054605	8.26E-102	4.09E-102	37.99	37.96	34.47	16.95	18.66	17.59
OsR498G0917795100.01	0.043	0.223	2.4598368	1.81E-05	3.37E-05	0.03	0.05	0.05	0.19	0.28	0.2
OsR498G0917796800.01	37.676	90.016	1.2563988	2.68E-262	6.30E-263	36.82	38.56	37.65	87.83	95.84	86.38
OsR498G0917797000.01	0.593	1.223	1.4822046	5.70E-08	9.46E-08	0.42	0.64	0.72	1.23	1.15	1.29
OsR498G0917800800.01	427.9	165.1	-1.38336	0	0	420.12	435.27	428.31	168.32	161.7	165.29
OsR498G0917802400.01	8.426	18.26	1.1216698	1.81E-110	8.49E-111	8.17	9.08	8.03	18.85	18.49	17.44
OsR498G0917802500.01	8.28	0.39	-4.419891	9.76E-222	2.66E-222	8.23	8.96	7.65	0.32	0.45	0.4
OsR498G0917803000.01	0.01	5.583	9.4881376	8.31E-129	3.50E-129	0	0	0.03	5.79	5.97	4.99
OsR498G0917803700.01	0.593	0.136	-2.09419	1.52E-11	2.23E-11	0.7	0.63	0.45	0.16	0.13	0.12
OsR498G0917804000.01	5.883	16.296	1.4695851	4.46E-106	2.15E-106	5.61	6.12	5.92	16.83	16.31	15.75
OsR498G0917804100.01	0.23	0.486	1.0584742	0.0008443	0.0017477	0.11	0.5	0.08	0.38	0.57	0.51
OsR498G0917804500.01	0.01	0.456	5.331962	2.54E-11	3.76E-11	0.03	0	0	0.83	0.13	0.41
OsR498G0917805300.01	0.02	0.7	4.9516899	3.26E-16	4.24E-16	0	0.03	0.03	0.9	0.59	0.61
OsR498G0917808800.01	0.246	0.02	-3.610553	0.0004877	0.0009887	0.37	0.06	0.31	0	0	0.06
OsR498G0917809200.01	11.726	1.45	-3.209799	6.73E-219	1.85E-219	11.4	12.62	11.16	1.32	1.29	1.74
OsR498G0917811200.01	13.543	30.59	1.1834334	2.06E-72	1.27E-72	13.35	13.48	13.8	29.77	32	30
OsR498G0917811300.01	20.093	50.173	1.3409069	2.01E-250	4.96E-251	19.97	21.11	19.2	51.62	48.02	50.88
OsR498G0917811500.01	0.04	0.25	2.7113756	0.0001114	0.0002167	0.08	0.04	0	0.15	0.37	0.23
OsR498G0917813300.01	4.96	20.656	2.0595919	2.85E-290	6.18E-291	4.84	5.03	5.01	20.41	20.95	20.61

OsR498G0917815700.01	1.02	9.87	3.3190135	8.81E-187	2.80E-187	1.13	0.87	1.06	9.88	10.13	9.6
OsR498G0917816000.01	16.43	33.22	1.0137198	7.56E-98	3.85E-98	16.77	15.67	16.85	33.29	33.81	32.56
OsR498G0917825500.01	3.64	9.273	1.3518592	5.27E-34	4.98E-34	3.36	3.57	3.99	10.5	8.23	9.09
OsR498G0917826500.01	0.066	0.316	2.1968024	5.55E-05	0.000106	0.1	0.03	0.07	0.4	0.17	0.38
OsR498G0917827000.01	2.29	5.18	1.3387277	3.85E-43	3.21E-43	2.3	2.36	2.21	5.25	5.03	5.26
OsR498G0917829600.01	0	0.57	5.9286063	5.09E-09	8.13E-09	0	0	0	0.82	0.49	0.4
OsR498G0917829800.01	5.956	13.69	1.2571663	3.85E-63	2.59E-63	6.09	6.33	5.45	13.38	12.47	15.22
OsR498G0917831400.01	36.1	8.803	-2.042716	5.90E-286	1.30E-286	37.38	33.99	36.93	9.09	8.57	8.75
OsR498G0917831800.01	5.263	2.29	-1.155101	1.66E-20	1.98E-20	5.07	5.46	5.26	1.87	2.22	2.78
OsR498G0917832900.01	0.153	0.54	1.804485	0.0001375	0.0002689	0.1	0.15	0.21	0.6	0.4	0.62
OsR498G0917833300.01	0.303	0.876	1.4827346	8.88E-05	0.0001716	0.12	0.14	0.65	0.93	0.89	0.81
OsR498G0917834500.01	0.643	0.193	-1.944972	1.20E-10	1.82E-10	0.55	0.69	0.69	0.18	0.23	0.17
OsR498G0917836900.01	14.416	4.883	-1.513111	4.78E-110	2.24E-110	14.41	13.66	15.18	4.95	4.28	5.42
OsR498G0917837400.01	0.253	0	-4.832945	6.10E-05	0.0001167	0.22	0.27	0.27	0	0	0
OsR498G0917844300.01	0.233	1.823	3.103693	1.15E-25	1.24E-25	0.16	0.36	0.18	1.98	1.87	1.62
OsR498G0917844500.01	9.143	21.28	1.2167129	2.05E-32	1.98E-32	10.18	7.35	9.9	22.31	18.37	23.16
OsR498G0917848400.01	1.193	0.356	-1.741797	7.64E-10	1.19E-09	1.4	1.36	0.82	0.27	0.41	0.39
OsR498G0917848800.01	0.236	0.496	1.0702934	0.0002157	0.0004266	0.31	0.2	0.2	0.48	0.54	0.47
OsR498G0917849600.01	17.496	279.58	3.9968543	0	0	17.07	17.38	18.04	279.88	267.29	291.58
OsR498G0917852100.01	140.94	32.016	-2.16989	0	0	144.44	142.98	135.4	31.5	32.32	32.23
OsR498G0917853100.01	5.61	11.493	1.0407252	5.42E-36	4.98E-36	5.7	5.1	6.03	11.59	10.51	12.38
OsR498G0917854200.01	1.813	4.163	1.1791545	2.18E-17	2.77E-17	2.34	1.53	1.57	4.86	3.39	4.24
OsR498G0917856000.01	3.91	9.13	1.1770755	7.36E-62	5.00E-62	4.23	3.56	3.94	10.07	8.27	9.05
OsR498G0917860100.01	12.343	28.616	1.2114013	3.72E-109	1.76E-109	12.46	11.86	12.71	27.01	29.42	29.42
OsR498G0917860900.01	9.123	3.19	-1.516244	4.15E-14	5.69E-14	10	7.67	9.7	4.38	2.96	2.23
OsR498G0917866300.01	1.59	0.536	-1.515916	2.97E-10	4.54E-10	1.21	1.59	1.97	0.61	0.45	0.55
OsR498G0917868800.01	0.21	0.02	-4.253639	7.88E-06	1.44E-05	0.29	0.14	0.2	0	0	0.06
OsR498G0917871200.01	0.29	0	-5.02559	1.85E-05	3.43E-05	0.16	0.38	0.33	0	0	0
OsR498G0917875600.01	0.58	0.206	-1.495075	0.0003264	0.0006536	0.63	0.58	0.53	0.19	0.19	0.24
OsR498G0917876200.01	0.77	7.763	3.3696273	3.09E-138	1.24E-138	0.77	0.77	0.77	6.75	8.05	8.49
OsR498G0917878700.01	0.516	3.32	2.6676253	1.45E-74	8.78E-75	0.69	0.4	0.46	3.69	3.46	2.81
OsR498G0917882200.01	2.693	5.743	1.0118847	7.43E-18	9.30E-18	2.44	3.32	2.32	6.7	4.73	5.8
OsR498G0917882300.01	0.103	1.096	3.4338416	3.49E-18	4.34E-18	0.08	0.12	0.11	0.7	1.37	1.22
OsR498G0917886400.01	10.146	21.19	1.0440228	4.75E-31	4.71E-31	9.63	10.25	10.56	21.11	21.02	21.44
OsR498G0917886700.01	3.943	2.156	-1.002131	1.25E-08	2.02E-08	3.8	4.18	3.85	2.82	1.83	1.82
OsR498G0917887000.01	8.68	18.166	1.0572068	5.52E-73	3.40E-73	9.05	9.61	7.38	19.05	18.16	17.29
OsR498G0917887200.01	3.34	23.656	2.8482689	4.23E-248	1.05E-248	3.58	2.96	3.48	24.22	24.03	22.72
OsR498G0917890000.01	0.273	0.913	1.7257308	2.28E-10	3.48E-10	0.24	0.27	0.31	0.66	1.15	0.93
OsR498G0917901700.01	0.423	1.39	1.7134956	6.68E-21	7.88E-21	0.38	0.37	0.52	1.38	1.4	1.39
OsR498G0917903500.01	3.28	0.086	-5.574027	2.54E-57	1.81E-57	3.19	3.67	2.98	0.13	0.06	0.07
OsR498G0917904600.01	1.06	0.456	-1.199783	7.63E-07	1.33E-06	0.97	1.11	1.1	0.48	0.58	0.31
OsR498G0917905800.01	9.76	0.643	-3.926812	9.68E-55	7.06E-55	9.72	11.4	8.16	0.79	0.45	0.69
OsR498G0917910100.01	1.393	10.21	2.8692277	7.90E-77	4.72E-77	0.85	1.17	2.16	9.7	9.53	11.4
OsR498G0917915800.01	0.013	0.29	4.5593725	4.39E-07	7.57E-07	0.04	0	0	0.36	0.25	0.26
OsR498G0917917300.01	0.193	0.66	2.4873888	5.89E-09	9.43E-09	0.35	0.14	0.09	0.68	0.35	0.95
OsR498G0917917600.01	0.073	0.84	3.5063783	6.31E-10	9.78E-10	0	0	0.22	0.67	1.07	0.78
OsR498G0917917800.01	12.996	29.656	1.2033591	2.79E-172	9.42E-173	13.16	12.1	13.73	29.36	30.54	29.07
OsR498G0917922900.01	1.306	8.836	2.7457093	4.68E-198	1.41E-198	1.4	1.14	1.38	8.75	8.87	8.89
OsR498G0917923700.01	0	0.2	4.5593725	0.0002368	0.0004696	0	0	0	0.05	0.4	0.15
OsR498G0917925200.01	0.803	0.173	-2.238584	5.60E-08	9.30E-08	0.62	1.08	0.71	0.14	0.14	0.24
OsR498G0917929400.01	4.8	50.85	3.4113713	0	0	5.03	5.17	4.2	51.82	52.05	48.68
OsR498G0917933900.01	0.506	1.933	2.0130904	2.59E-41	2.22E-41	0.58	0.45	0.49	2.24	1.79	1.77
OsR498G0917934300.01	1.356	0.336	-2.015809	1.71E-17	2.16E-17	1.56	1.43	1.08	0.27	0.35	0.39
OsR498G0917934600.01	0.266	1.643	2.650281	7.25E-40	6.33E-40	0.25	0.34	0.21	1.67	1.81	1.45
OsR498G0917934700.01	11.363	24.94	1.1815679	1.63E-39	1.43E-39	11.81	10.87	11.41	27.09	21.27	26.46
OsR498G0917935000.01	8.986	25.253	1.4896048	1.00E-160	3.59E-161	8.46	9.68	8.82	25.07	25.04	25.65
OsR498G0917939500.01	5.406	12.433	1.2013514	1.92E-90	1.03E-90	5.45	5.33	5.44	13.11	12.23	11.96
OsR498G0917940000.01	29.023	82.543	1.5185645	0	0	30.09	28.4	28.58	82.48	82.72	82.43
OsR498G0917940100.01	0.43	0.19	-1.172431	9.28E-08	1.56E-07	0.43	0.36	0.5	0.18	0.17	0.22
OsR498G0917941200.01	8.49	20.49	1.2724508	8.61E-65	5.69E-65	9.45	7.22	8.8	21.7	18.91	20.86
OsR498G0917941600.01	0.793	0.163	-2.294079	2.01E-08	3.29E-08	0.64	0.75	0.99	0.18	0.18	0.13
OsR498G0917941800.01	1.37	3.713	1.4370897	3.60E-45	2.92E-45	1.26	1.52	1.33	3.68	3.56	3.9
OsR498G0917942100.01	13.09	44.236	1.7542129	0	0	12.76	12.91	13.6	45.2	45.87	41.64
OsR498G0917942900.01	20.946	49.613	1.2377453	8.60E-101	4.30E-101	20.17	20.96	21.71	46.42	51.63	50.79
OsR498G0917945200.01	18.933	41.2	1.1225719	1.56E-246	3.93E-247	19.65	18.55	18.6	42.14	41.97	39.49
OsR498G0917947500.01	3.683	10.4	1.4949307	3.48E-71	2.17E-71	3.7	3.74	3.61	10.7	11.13	9.37
OsR498G0917947700.01	0.036	0.163	2.4338416	0.0001322	0.0002585	0.05	0.03	0.03	0.13	0.13	0.23
OsR498G0917948600.01	11.776	35.346	1.5840183	7.40E-117	3.34E-117	12.07	11.83	11.43	32.9	34.67	38.47
OsR498G0917948700.01	4.273	9.856	1.4521224	5.20E-83	2.97E-83	4.42	4.84	3.56	11.22	8.17	10.18

OsR498G0917948900.01	1.143	3.11	1.4529504	1.22E-15	1.61E-15	1.12	0.93	1.38	3.26	3.27	2.8
OsR498G0917952100.01	0.026	0.726	4.832391	5.71E-15	7.66E-15	0	0.04	0.04	0.59	0.67	0.92
OsR498G0917952300.01	0.083	1.623	4.2897654	5.29E-49	4.12E-49	0.14	0	0.11	1.36	1.77	1.74
OsR498G0917953300.01	0.053	3.163	5.884303	6.83E-114	3.15E-114	0.06	0.06	0.04	3.35	3.08	3.06
OsR498G0917953800.01	9.106	26.1	1.517716	1.40E-143	5.43E-144	9.78	8.93	8.61	27.02	23.18	28.1
OsR498G0917955500.01	0.433	1.676	1.9670304	1.32E-21	1.54E-21	0.61	0.32	0.37	1.49	2.07	1.47
OsR498G0917960700.01	1.416	2.923	1.049207	2.15E-43	1.79E-43	1.55	1.37	1.33	2.61	3.23	2.93
OsR498G0917960900.01	2.123	7.276	1.7416874	1.63E-44	1.34E-44	2.46	1.88	2.03	7.25	7.79	6.79
OsR498G0917961200.01	0.556	2.863	2.4913275	1.91E-43	1.59E-43	0.41	0.56	0.7	2.38	2.72	3.49
OsR498G0917961300.01	0.586	3.746	2.683067	2.18E-58	1.54E-58	0.59	0.51	0.66	3.96	3.64	3.64
OsR498G0917969800.01	0.653	3.446	2.4018312	4.07E-22	4.69E-22	0.95	0.47	0.54	2.96	3.67	3.71
OsR498G0917969900.01	5.656	31.096	2.4419434	3.82E-167	1.32E-167	4.76	6.06	6.15	29.24	30	34.05
OsR498G0917971200.01	10.756	68.573	2.6708134	0	0	11.62	9.97	10.68	68.31	63.71	73.7
OsR498G0917973100.01	0.103	6.083	6.0885714	1.30E-249	3.21E-250	0.08	0.16	0.07	5.75	6.39	6.11
OsR498G0917973700.01	1.17	15.016	3.3743543	0	0	1.22	1.23	1.06	15.75	14.79	14.51
OsR498G0917973900.01	2.826	5.653	1.0460329	1.59E-29	1.61E-29	3.08	2.67	2.73	5.8	6.05	5.11
OsR498G0917976000.01	0.01	0.303	5.103693	7.53E-10	1.17E-09	0	0	0.03	0.26	0.31	0.34
OsR498G0917976200.01	1.88	7.56	1.9844355	9.45E-97	4.86E-97	2.16	2.18	1.3	7.32	7.82	7.54
OsR498G0917976300.01	0.266	1.35	2.3454055	3.60E-12	5.20E-12	0.08	0.29	0.43	1.54	1.68	0.83
OsR498G0917977900.01	2.233	52.336	4.5491367	0	0	1.97	1.97	2.76	51.74	47.83	57.44
OsR498G0917978100.01	3.696	49.946	3.7498851	0	0	4.16	3.34	3.59	47.61	48.34	53.89
OsR498G0917978400.01	2.68	7.086	1.4575785	1.17E-52	8.72E-53	2.58	2.64	2.82	8.64	6.62	6
OsR498G0917980300.01	0	0.433	4.97441	2.21E-05	4.12E-05	0	0	0	0.4	0.32	0.58
OsR498G0917981400.01	8.56	56.356	2.7272824	0	0	8.51	8.73	8.44	57.61	56.32	55.14
OsR498G0917982000.01	0.03	0.35	3.5593725	2.76E-13	3.85E-13	0.02	0.02	0.05	0.4	0.24	0.41
OsR498G0917982900.01	21.413	76.29	1.8308372	0	0	21.54	20.81	21.89	74.85	70.97	83.05
OsR498G0917983600.01	8.083	40	2.2244923	0	0	8.52	7.45	8.28	41.91	39.02	39.07
OsR498G1017994000.01	92.26	20.486	-2.218831	0	0	88.82	91.93	96.03	18.15	20.41	22.9
OsR498G1017997800.01	15.18	108.28	2.8316153	0	0	14.8	14.96	15.78	104.13	105.52	115.19
OsR498G1018011400.01	0.156	2.303	3.9562626	1.44E-50	1.10E-50	0.23	0.09	0.15	2.03	2.36	2.52
OsR498G1018011600.01	4.516	9.63	1.0808609	2.33E-71	1.45E-71	4.71	4.71	4.13	9.89	9.74	9.26
OsR498G1018016300.01	0.183	0.963	2.3894475	0.0009846	0.0020515	0.55	0	0	1.08	1.08	0.73
OsR498G1018022900.01	0.22	0.72	1.864227	1.02E-07	1.72E-07	0.21	0.23	0.22	0.91	0.8	0.45
OsR498G1018029600.01	0.63	0	-7.111992	3.06E-17	3.90E-17	0.67	0.61	0.61	0	0	0
OsR498G1018031600.01	9.073	3.66	-1.295511	1.03E-58	7.22E-59	9.43	8.73	9.06	3.79	3.56	3.63
OsR498G1018034200.01	8.13	1.4	-2.40607	2.64E-118	1.18E-118	8.67	7.89	7.83	1.03	1.51	1.66
OsR498G1018036600.01	60.72	203.82	1.7490845	0	0	61.48	59.06	61.62	194.34	203.57	213.56
OsR498G1018037200.01	3.303	10.276	1.6323299	1.20E-98	6.08E-99	3.41	2.81	3.69	10.61	10.03	10.19
OsR498G1018037600.01	4.536	31.82	2.8106265	0	0	4.77	4.27	4.57	32.05	32.89	30.52
OsR498G1018039500.01	0.05	0.17	1.662466	0.00052	0.001056	0.05	0.05	0.05	0.07	0.17	0.27
OsR498G1018039900.01	0	0.956	5.2963381	2.20E-06	3.91E-06	0	0	0	0.71	0.71	1.45
OsR498G1018043000.01	4.053	12.993	1.631031	4.41E-80	2.57E-80	3.47	4.49	4.2	13.18	12.27	13.53
OsR498G1018044000.01	5.023	11.006	1.0598484	1.96E-47	1.55E-47	4.75	4.74	5.58	11.07	10.61	11.34
OsR498G1018056400.01	4.02	0.306	-3.813182	4.50E-61	3.08E-61	4.81	3.72	3.53	0.28	0.24	0.4
OsR498G1018061000.01	0.036	0.39	3.3568796	3.87E-15	5.17E-15	0.03	0.05	0.03	0.46	0.35	0.36
OsR498G1018061800.01	3.373	0.436	-2.979786	3.07E-79	1.80E-79	3.72	3.38	3.02	0.42	0.4	0.49
OsR498G1018062300.01	0.44	0.926	1.056204	7.57E-07	1.32E-06	0.62	0.37	0.33	1.01	1.15	0.62
OsR498G1018063500.01	0.236	1.84	2.9562626	1.63E-27	1.71E-27	0.25	0.25	0.21	1.73	1.55	2.24
OsR498G1018067200.01	2.053	4.796	1.225828	8.54E-31	8.50E-31	2.1	2.05	2.01	4.82	5.1	4.47
OsR498G1018069100.01	1.33	3.343	1.3305538	2.00E-05	3.71E-05	1.44	1.44	1.11	3.75	4.05	2.23
OsR498G1018071400.01	0.446	1.02	1.1910647	1.72E-11	2.54E-11	0.49	0.49	0.36	1.09	0.88	1.09
OsR498G1018073600.01	0.45	1.45	1.6748497	2.19E-12	3.15E-12	0.34	0.5	0.51	1.4	1.38	1.57
OsR498G1018080600.01	0.156	0.54	1.8109112	2.79E-06	4.98E-06	0.1	0.2	0.17	0.45	0.61	0.56
OsR498G1018081200.01	0.04	0.266	2.6748497	1.53E-05	2.83E-05	0	0.06	0.06	0.21	0.24	0.35
OsR498G1018082400.01	0.25	1.513	2.568591	7.20E-24	8.04E-24	0.26	0.32	0.17	1.39	1.42	1.73
OsR498G1018083600.01	0.046	0.27	2.6066782	3.65E-06	6.54E-06	0	0.11	0.03	0.34	0.23	0.24
OsR498G1018086000.01	0.083	0.343	2.0188041	3.53E-05	6.65E-05	0.16	0.03	0.06	0.28	0.4	0.35
OsR498G1018086400.01	0.08	0.703	3.0977924	3.55E-12	5.13E-12	0.07	0.1	0.07	0.51	0.65	0.95
OsR498G1018087000.01	0.156	0.463	1.5958983	9.68E-05	0.0001875	0.18	0.18	0.11	0.31	0.55	0.53
OsR498G1018090000.01	0.12	0.296	1.3529216	1.38E-05	2.55E-05	0.05	0.16	0.15	0.3	0.37	0.22
OsR498G1018102000.01	0.013	0.763	5.7557697	1.15E-14	1.56E-14	0.04	0	0	0.5	1.11	0.68
OsR498G1018103400.01	0.193	0.016	-3.485022	0.000899	0.0018658	0.16	0.05	0.37	0	0	0.05
OsR498G1018104800.01	4.05	41.833	3.3639325	0	0	4.11	4.08	3.96	42.93	43.11	39.46
OsR498G1018105500.01	0.246	1.956	2.9943095	7.35E-26	7.93E-26	0.33	0.16	0.25	2.04	2.07	1.76
OsR498G1018106100.01	10.776	77.806	2.8517524	0	0	9.55	10.73	12.05	79.22	80.08	74.12
OsR498G1018108500.01	9.423	22.096	1.2276749	2.80E-105	1.36E-105	9.08	10.55	8.64	23.24	20.13	22.92
OsR498G1018109100.01	8.383	2.763	-1.542798	1.53E-99	7.74E-100	8.24	8.05	8.86	2.78	2.68	2.83
OsR498G1018111400.01	0.62	2.156	1.7946228	2.50E-17	3.18E-17	0.42	0.91	0.53	2.04	2.29	2.14
OsR498G1018112500.01	5.306	15.47	1.5448063	0	0	5.45	4.95	5.52	15.53	15.59	15.29

OsR498G1018117600.01	0.13	1.4	3.4662631	4.11E-27	4.35E-27	0.13	0.13	0.13	1.42	1.29	1.49
OsR498G1018122500.01	0.593	0.193	-1.610553	1.00E-05	1.84E-05	0.53	0.62	0.63	0.1	0.27	0.21
OsR498G1018123300.01	1.106	3.94	1.8082371	1.14E-36	1.04E-36	1.26	0.89	1.17	4.13	3.42	4.27
OsR498G1018123500.01	11.596	25.79	1.1599318	8.88E-80	5.19E-80	11.38	12.1	11.31	26.4	26.15	24.82
OsR498G1018125100.01	0.473	1.136	1.2713917	2.20E-06	3.92E-06	0.37	0.56	0.49	1.7	0.94	0.77
OsR498G1018125300.01	8.083	50.973	2.6573315	0	0	7.91	8.18	8.16	49.78	53.67	49.47
OsR498G1018131600.01	1.65	0	-7.329371	1.76E-19	2.14E-19	1.39	1.31	2.25	0	0	0
OsR498G1018133600.01	0.106	0.506	2.2598122	1.83E-06	3.24E-06	0.12	0.16	0.04	0.48	0.44	0.6
OsR498G1018134400.01	2.216	6.933	1.4152875	9.35E-45	7.61E-45	2.23	2.19	2.23	6.33	7.58	6.89
OsR498G1018141300.01	0.026	0.266	3.4006747	1.01E-09	1.58E-09	0.02	0.04	0.02	0.22	0.26	0.32
OsR498G1018148000.01	2.15	4.41	1.1333539	1.59E-31	1.56E-31	2.16	1.86	2.43	4.83	4.56	3.84
OsR498G1018151700.01	0.78	1.77	1.2172665	2.70E-05	5.05E-05	0.67	0.91	0.76	1.78	1.55	1.98
OsR498G1018165700.01	0.186	0.483	1.4831629	1.88E-08	3.06E-08	0.23	0.18	0.15	0.49	0.48	0.48
OsR498G1018166100.01	0.31	0.823	1.4072733	2.91E-11	4.32E-11	0.26	0.24	0.43	0.82	0.87	0.78
OsR498G1018169000.01	1.286	3.126	1.2762004	2.52E-15	3.35E-15	1.57	1.11	1.18	3.11	3.54	2.73
OsR498G1018176900.01	0.326	1.366	2.0675194	1.13E-15	1.50E-15	0.3	0.42	0.26	1.59	1.23	1.28
OsR498G1018197800.01	0.396	0.813	1.1056545	9.94E-08	1.67E-07	0.43	0.24	0.52	1.3	0.56	0.58
OsR498G1018201600.01	8.59	38.07	2.1849124	1.70E-116	7.68E-117	9.46	7.99	8.32	38.96	38.49	36.76
OsR498G1018203800.01	6.343	27.373	2.0797049	1.27E-165	4.46E-166	6.25	5.85	6.93	26.95	27.08	28.09
OsR498G1018206100.01	0.733	0.223	-1.712432	4.32E-13	6.07E-13	0.69	0.76	0.75	0.33	0.14	0.2
OsR498G1018217100.01	67.576	28.866	-1.227411	0	0	58.42	71.57	72.74	29.38	29.21	28.01
OsR498G1018217600.01	0.03	0.13	2.3886045	0.0007001	0.0014368	0	0.09	0	0	0.08	0.31
OsR498G1018228800.01	1.153	5.376	2.2223375	7.72E-29	7.92E-29	1.16	0.93	1.37	6.04	4.97	5.12
OsR498G1018238100.01	1.53	0.26	-2.546422	1.65E-20	1.96E-20	1.54	1.28	1.77	0.24	0.3	0.24
OsR498G1018249900.01	0.746	6.673	3.2520488	9.51E-76	5.72E-76	0.64	0.82	0.78	6.07	6.58	7.37
OsR498G1018250900.01	27.736	121.51	2.1295734	0	0	28	26.02	29.19	119.8	117.34	127.4
OsR498G1018251100.01	0.093	0.556	2.6182661	2.64E-05	4.95E-05	0.07	0.14	0.07	0.46	0.53	0.68
OsR498G1018260500.01	0.48	0.093	-2.383142	3.87E-07	6.66E-07	0.46	0.42	0.56	0.07	0.1	0.11
OsR498G1018267400.01	0.053	0.256	2.2223375	0.0006354	0.0012994	0.04	0.04	0.08	0.18	0.31	0.28
OsR498G1018269200.01	0.81	1.873	1.1402188	1.04E-17	1.31E-17	0.82	0.94	0.67	2.04	1.72	1.86
OsR498G1018269500.01	3.033	1.216	-1.321983	5.89E-10	9.12E-10	3.46	2.72	2.92	1.4	0.95	1.3
OsR498G1018279000.01	2.316	9.53	1.9448419	1.78E-165	6.25E-166	2.27	2.42	2.26	9.48	10.21	8.9
OsR498G1018281600.01	88.343	26.29	-1.760354	0	0	88.56	88.77	87.7	26.74	26.35	25.78
OsR498G1018282200.01	3.066	8.193	1.4131344	2.44E-65	1.60E-65	2.84	3.27	3.09	8.2	8.09	8.29
OsR498G1018292300.01	5.096	10.436	1.0183533	4.99E-32	4.86E-32	4.59	5.13	5.57	10.36	10.05	10.9
OsR498G1018293300.01	0.58	3.326	2.5250581	1.13E-64	7.45E-65	0.65	0.47	0.62	2.97	3.3	3.71
OsR498G1018314600.01	124.213	44.62	-1.483426	0	0	128.47	120.88	123.29	44.28	45.79	43.79
OsR498G1018317800.01	4.523	10.146	1.1677733	6.89E-56	4.98E-56	4.97	4.29	4.31	10.14	10.46	9.84
OsR498G1018323000.01	0.52	0.14	-1.888087	1.22E-05	2.25E-05	0.51	0.39	0.66	0.15	0.19	0.08
OsR498G1018331900.01	36.926	89.466	1.285428	0	0	37.5	36.22	37.06	90.12	91.42	86.86
OsR498G1018338500.01	0.603	0.01	-5.85848	2.28E-15	3.02E-15	0.77	0.44	0.6	0.03	0	0
OsR498G1018339300.01	0.466	0.126	-1.913115	2.10E-05	3.91E-05	0.42	0.3	0.68	0.15	0.19	0.04
OsR498G1018362000.01	0.08	0.536	2.6748497	1.53E-05	2.83E-05	0	0.12	0.12	0.31	0.67	0.63
OsR498G1018374400.01	6.576	13.396	1.0248447	6.86E-43	5.73E-43	6.17	7.14	6.42	12.93	13.85	13.41
OsR498G1018375100.01	2.166	0.66	-1.68947	6.95E-46	5.59E-46	2.53	2.19	1.78	0.64	0.65	0.69
OsR498G1018378900.01	38.36	12.943	-1.566736	3.40E-172	1.15E-172	37.76	38.07	39.25	13.15	12.52	13.16
OsR498G1018385200.01	2.923	1.196	-1.183131	1.89E-07	3.21E-07	3.06	2.5	3.21	0.62	1.38	1.59
OsR498G1018387300.01	1.54	0.743	-1.271006	1.86E-35	1.72E-35	1.79	1.59	1.24	0.53	0.81	0.89
OsR498G1018393600.01	10.006	40.93	2.0323635	0	0	11.06	9.74	9.22	40.79	41.16	40.84
OsR498G1018420500.01	2	4.446	1.0633437	9.67E-17	1.24E-16	2.4	1.6	2	4.06	4.34	4.94
OsR498G1018426100.01	0.496	2.173	2.1241571	9.63E-10	1.50E-09	0.47	0.37	0.65	1.64	2.64	2.24
OsR498G1018427100.01	27.89	167.86	2.5882513	0	0	28.3	27.27	28.1	163.55	162.53	177.51
OsR498G1018431000.01	1.843	8.9	2.1884575	1.65E-88	9.02E-89	1.74	1.6	2.19	9.22	9.32	8.16
OsR498G1018432500.01	0.16	0.88	2.4338416	1.63E-10	2.48E-10	0.09	0.18	0.21	1.1	1.16	0.38
OsR498G1018439200.01	0.186	0.783	2.0930545	1.56E-18	1.93E-18	0.21	0.16	0.19	0.81	0.69	0.85
OsR498G1018440800.01	0.583	1.39	1.2374444	8.31E-05	0.0001603	0.35	0.77	0.63	1.58	1.03	1.56
OsR498G1018450400.01	1.693	4.8	1.5470679	1.07E-35	9.88E-36	1.64	1.93	1.51	4.65	5.16	4.59
OsR498G1018461300.01	11.976	2.283	-2.399483	5.62E-66	3.67E-66	14.43	10.34	11.16	2.68	1.55	2.62
OsR498G1018462100.01	275.103	109.65	-1.308913	0	0	273.2	287.55	264.56	116.73	102.63	109.58
OsR498G1018483900.01	9.226	20.373	1.1588119	5.19E-18	6.48E-18	9.5	9.39	8.79	18.78	19.77	22.57
OsR498G1018485600.01	0.103	0.963	3.2996454	2.62E-29	2.67E-29	0.13	0.1	0.08	1.01	1.04	0.84
OsR498G1018499800.01	7.393	21.82	1.5998073	3.86E-32	3.75E-32	8.41	6.34	7.43	21.51	18.88	25.07
OsR498G1018505600.01	8.15	22.946	1.4988635	1.41E-169	4.83E-170	7.85	8.68	7.92	21.99	23.19	23.66
OsR498G1018508100.01	0.816	2.176	1.357267	1.01E-09	1.57E-09	0.83	0.59	1.03	2.35	1.93	2.25
OsR498G1018520200.01	9.606	19.77	1.0396346	1.27E-48	9.95E-49	8.85	10.37	9.6	20.22	18.42	20.67
OsR498G1018534900.01	0.153	0.956	2.6346606	2.23E-27	2.35E-27	0.18	0.15	0.13	1.32	0.9	0.65
OsR498G1018535600.01	0.113	0.94	2.690617	1.43E-08	2.32E-08	0.16	0.14	0.04	0.75	1.11	0.96
OsR498G1018540900.01	7.906	2.093	-1.914559	1.24E-41	1.06E-41	6.98	8.15	8.59	2.6	1.96	1.72
OsR498G1018549300.01	22.816	10.913	-1.051101	3.85E-87	2.13E-87	23.65	23.01	21.79	10.7	11.3	10.74

OsR498G1018551500.01	12.96	27.026	1.0172723	1.02E-41	8.68E-42	10.97	13.72	14.19	27.9	25.51	27.67
OsR498G1018552900.01	1.01	8.35	3.0447993	5.94E-65	3.92E-65	1.02	1.14	0.87	8.02	7.99	9.04
OsR498G1018554900.01	0.086	4.776	5.7425943	2.76E-27	2.91E-27	0.09	0.17	0	5.13	4.48	4.72
OsR498G1018556800.01	1.356	3.743	1.4662631	6.61E-17	8.48E-17	1.38	1.47	1.22	3.32	4.14	3.77
OsR498G1018557000.01	4.493	9.333	1.0555634	7.26E-59	5.08E-59	4.71	4.33	4.44	9.29	9.86	8.85
OsR498G1018558500.01	0.046	0.296	2.7399447	7.12E-07	1.24E-06	0.03	0.11	0	0.29	0.36	0.24
OsR498G1018558800.01	52.173	19.646	-1.410683	0	0	50.02	53.35	53.15	19.33	19.61	20
OsR498G1018560400.01	0.233	0.913	2.1366814	2.05E-12	2.94E-12	0.19	0.21	0.3	0.93	0.75	1.06
OsR498G1018562700.01	1.24	0.366	-1.75351	1.98E-06	3.52E-06	1.2	1.4	1.12	0.55	0.2	0.35
OsR498G1018573900.01	4.763	1.713	-1.475317	1.71E-65	1.12E-65	4.43	5.1	4.76	1.76	1.72	1.66
OsR498G1018574700.01	0.23	0.076	-1.989064	8.07E-06	1.47E-05	0.21	0.17	0.31	0.05	0.16	0.02
OsR498G1018583800.01	9.83	35.523	1.8507786	2.13E-214	5.98E-215	10.22	9.59	9.68	35.85	35.35	35.37
OsR498G1018586200.01	50.68	14.99	-1.854214	0	0	53.39	49.26	49.39	16.01	14.39	14.57
OsR498G1018590600.01	0	0.216	5.144335	6.91E-06	1.26E-05	0	0	0	0.18	0.11	0.36
OsR498G1018595800.01	3.993	10.473	1.4029274	4.19E-87	2.32E-87	4.08	4.2	3.7	9.62	11.53	10.27
OsR498G1018620600.01	17.886	37.443	1.0556897	3.83E-125	1.65E-125	18.02	18.45	17.19	39.43	38.91	33.99
OsR498G1018621200.01	1.21	7.93	2.6616225	3.23E-57	2.30E-57	1.1	1.11	1.42	8.4	7.59	7.8
OsR498G1018621400.01	6.966	14.203	1.0088694	9.41E-32	9.22E-32	8.29	5.37	7.24	15.16	12.92	14.53
OsR498G1018622100.01	0.983	0.466	-1.003731	4.70E-10	7.25E-10	1.07	0.92	0.96	0.4	0.44	0.56
OsR498G1018622300.01	1.36	0.383	-1.808492	1.35E-12	1.93E-12	1.61	1.25	1.22	0.34	0.57	0.24
OsR498G1018622600.01	3.77	36.963	3.2959404	0	0	4.17	3.08	4.06	35.61	37.45	37.83
OsR498G1018623200.01	4.473	33.526	2.8418516	0	0	4.43	4.37	4.62	33.53	34.62	32.43
OsR498G1018623600.01	0.946	16.733	4.7043705	4.02E-211	1.15E-211	1.59	0.67	0.58	15.41	13.06	21.73
OsR498G1018624000.01	0.043	0.903	4.2720905	1.63E-14	2.21E-14	0.09	0.04	0	1.02	0.67	1.02
OsR498G1018625400.01	2.496	0.16	-3.977762	1.47E-76	8.77E-77	2.59	2.41	2.49	0.16	0.26	0.06
OsR498G1018626200.01	1.62	5.8	1.8434493	7.29E-84	4.13E-84	1.95	1.35	1.56	6.12	5.63	5.65
OsR498G1018626300.01	5.426	23.606	2.1165212	1.04E-301	2.19E-302	5.76	5.33	5.19	25.29	22.9	22.63
OsR498G1018626700.01	1.983	5.64	1.5076792	7.77E-67	5.03E-67	2.1	2.15	1.7	6.19	5.35	5.38
OsR498G1018631200.01	0.063	0.973	4.2598122	3.56E-10	5.47E-10	0.06	0.13	0	0.79	1.11	1.02
OsR498G1018635200.01	0.526	24.386	5.7280247	9.53E-266	2.21E-266	0.63	0.49	0.46	21.71	24.62	26.83
OsR498G1018638500.01	0.026	0.35	3.6748497	7.57E-07	1.32E-06	0	0.04	0.04	0.32	0.36	0.37
OsR498G1018638900.01	0.013	0.97	6.144335	1.41E-18	1.75E-18	0.04	0	0	1.04	0.84	1.03
OsR498G1018641400.01	14.57	4.18	-1.840599	6.61E-78	3.92E-78	14.95	14.39	14.37	3.56	4.81	4.17
OsR498G1018644000.01	0.513	8.643	4.1671666	0	0	0.54	0.46	0.54	8.92	9.21	7.8
OsR498G1018646800.01	0.653	1.503	1.3600636	1.35E-06	2.37E-06	0.81	0.52	0.63	1.56	1.32	1.63
OsR498G1018649200.01	1.086	3.08	1.5012207	7.92E-29	8.13E-29	0.78	1.03	1.45	2.97	3.19	3.08
OsR498G1018652000.01	4.51	0.43	-2.832945	2.31E-27	2.44E-27	4.54	4.81	4.18	0.68	0.2	0.41
OsR498G1018657900.01	6.35	13.556	1.0950609	4.98E-69	3.16E-69	6.32	5.82	6.91	14.4	12.27	14
OsR498G1018659800.01	6.323	15.94	1.3373336	1.34E-103	6.56E-104	6.46	6.16	6.35	15.21	16.28	16.33
OsR498G1018660600.01	1.42	0.5	-1.511017	0.0001066	0.0002069	1.12	2.23	0.91	0.4	0.59	0.51
OsR498G1018675600.01	0.38	0.056	-2.72603	1.08E-05	1.98E-05	0.48	0.31	0.35	0.04	0.09	0.04
OsR498G1018676700.01	3.74	1.033	-1.93743	9.83E-39	8.70E-39	4.13	3.62	3.47	0.77	1.15	1.18
OsR498G1018681600.01	6.99	16.663	1.2609327	2.84E-70	1.78E-70	7.47	6.53	6.97	16.37	16.56	17.06
OsR498G1018704900.01	0.95	2.316	1.2755795	2.89E-24	3.21E-24	0.81	0.89	1.15	2.33	2.31	2.31
OsR498G1018707200.01	27.06	124.59	2.2007988	0	0	25.04	27.93	28.21	121.19	122.32	130.25
OsR498G1018707600.01	0.836	1.71	1.0999408	6.13E-08	1.02E-07	0.78	0.73	1	1.88	1.43	1.82
OsR498G1018707800.01	1.486	4.356	1.5527222	5.33E-63	3.59E-63	1.31	1.5	1.65	4.12	4.31	4.64
OsR498G1018707900.01	7.316	22.76	1.6341623	1.52E-57	1.08E-57	7.36	5.83	8.76	21.15	20.95	26.18
OsR498G1018709100.01	15.353	7.4	-1.054595	5.10E-56	3.67E-56	15.69	14.43	15.94	6.93	7	8.27
OsR498G1018711500.01	0.113	0.36	1.70194	0.0008825	0.00183	0.09	0.15	0.1	0.38	0.25	0.45
OsR498G1018717300.01	0.05	0.213	2.2374444	0.000145	0.0002838	0	0.06	0.09	0.22	0.17	0.25
OsR498G1018717900.01	0.113	1.316	3.6037666	6.13E-21	7.23E-21	0.16	0.11	0.07	1.45	1.16	1.34
OsR498G1018718600.01	0.26	1.856	2.8415743	1.31E-32	1.27E-32	0.26	0.17	0.35	2.19	1.79	1.59
OsR498G1018720700.01	93.566	264.75	1.3419787	0	0	93.11	95.18	92.41	267.82	268.74	257.7
OsR498G1018723700.01	1.633	8.473	2.3720023	6.09E-72	3.77E-72	1.71	1.64	1.55	8.32	7.6	9.5
OsR498G1018725300.01	0.133	0.436	1.6748497	0.0008798	0.0018237	0.15	0.1	0.15	0.45	0.4	0.46
OsR498G1018728100.01	36.176	295.04	3.0300666	0	0	38.47	34.58	35.48	297.29	293.37	294.47
OsR498G1018732200.01	0.01	0.25	3.8813006	7.15E-08	1.19E-07	0.03	0	0	0.44	0.11	0.2
OsR498G1018736000.01	9.32	22.773	1.2983464	1.16E-141	4.56E-142	9.24	8.9	9.82	23.23	22.29	22.8
OsR498G1018736600.01	1.67	8.436	2.3389824	1.07E-53	7.91E-54	2.29	1.13	1.59	7.69	8.59	9.03
OsR498G1018737400.01	3.56	15.406	2.112713	5.04E-122	2.20E-122	3.14	3.33	4.21	15.41	15.14	15.67
OsR498G1018745900.01	0.013	0.226	3.97441	5.14E-05	9.78E-05	0.04	0	0	0.25	0.08	0.35
OsR498G1018746100.01	1.6	4.096	1.271862	5.19E-16	6.80E-16	1.57	1.46	1.77	4.4	3.92	3.97
OsR498G1018763200.01.	2.93	5.896	1.0053227	1.81E-17	2.30E-17	4.28	2.32	2.19	6.62	4.04	7.03
OsR498G1018769600.01	0.24	0.726	1.5956072	6.85E-05	0.0001314	0.56	0	0.16	1.45	0.3	0.43
OsR498G1018774500.01	0.84	0.393	-1.113053	0.0001003	0.0001945	0.84	0.85	0.83	0.32	0.37	0.49
OsR498G1018776400.01	0.526	0.206	-1.485478	3.84E-06	6.89E-06	0.4	0.65	0.53	0.23	0.22	0.17
OsR498G1018776600.01	0.226	0.06	-2.532116	6.62E-05	0.0001269	0.38	0.04	0.26	0.04	0.08	0.06
OsR498G1018783200.01	1.626	4.753	1.5449993	1.31E-59	9.08E-60	1.44	1.56	1.88	4.66	4.83	4.77

OsR498G1018785100.01	0.876	1.87	1.0898872	0.0001954	0.0003855	0.88	1.05	0.7	1.97	1.71	1.93
OsR498G1018793600.01	0.03	1.996	6.0041573	1.73E-47	1.37E-47	0.03	0.03	0.03	1.6	1.93	2.46
OsR498G1018794000.01	1.56	4.846	1.6204019	4.68E-28	4.87E-28	1.66	1.34	1.68	5.04	4.74	4.76
OsR498G1018794500.01	9.55	85.666	3.1438788	0	0	9.69	8.39	10.57	82.43	84.19	90.38
OsR498G1018797700.01	10.163	24.736	1.2847095	1.46E-79	8.52E-80	9.9	10.92	9.67	24.9	24.71	24.6
OsR498G1018797900.01	0.126	2.07	4.0133289	2.18E-56	1.57E-56	0.14	0.12	0.12	2.04	2.12	2.05
OsR498G1018798100.01	0.21	1.193	2.5027889	2.78E-22	3.19E-22	0.39	0.07	0.17	1.35	0.99	1.24
OsR498G1018798600.01	0.653	4.453	2.5666706	4.67E-164	1.65E-164	0.78	0.51	0.67	4.65	4.33	4.38
OsR498G1018799600.01	1.793	6.46	1.8186336	1.10E-35	1.02E-35	1.41	2.02	1.95	5.75	6.84	6.79
OsR498G1018800500.01	2.943	15.953	2.5520677	0	0	3.12	2.53	3.18	16.29	16.47	15.1
OsR498G1018801300.01	0.42	4.076	3.2730683	1.03E-45	8.33E-46	0.51	0.35	0.4	3.25	4.07	4.91
OsR498G1018803300.01	0.09	0.3	1.7190905	0.0008905	0.0018471	0.2	0.07	0	0.21	0.36	0.33
OsR498G1018810100.01	2.63	7.903	1.2539829	4.41E-45	3.58E-45	2.57	2.57	2.75	7.63	8.18	7.9
OsR498G1018810900.01	1.163	27.383	4.5534476	0	0	1.09	1.03	1.37	26.2	25.26	30.69
OsR498G1018811400.01	0.826	1.906	1.2038918	1.65E-08	2.68E-08	0.77	0.98	0.73	2.04	1.37	2.31
OsR498G1018816100.01	4.316	11.666	1.405928	5.13E-140	2.03E-140	4.46	4.21	4.28	11.05	11.96	11.99
OsR498G1018825400.01	2.623	1.256	-1.065854	2.94E-06	5.25E-06	2.54	2.59	2.74	1.34	0.98	1.45
OsR498G1018825800.01	14.916	6.166	-1.254635	1.24E-44	1.01E-44	14.01	15.07	15.67	6.69	5.06	6.75
OsR498G1018829400.01	11.866	25.836	1.1224143	2.01E-111	9.38E-112	11.28	11.99	12.33	26.29	25.38	25.84
OsR498G1018836900.01	0.323	2.103	2.7023304	1.50E-17	1.90E-17	0.37	0.18	0.42	2.25	2	2.06
OsR498G1018837500.01	2.426	9.86	2.0222792	3.28E-127	1.40E-127	2.48	2.05	2.75	10.63	9.27	9.68
OsR498G1018838300.01	11.206	27.15	1.2830178	8.09E-249	2.01E-249	12.3	11.59	9.73	28.11	27.76	25.58
OsR498G1018838600.01	1.33	2.993	1.2556961	4.68E-11	7.00E-11	1.67	0.72	1.6	3.58	2.86	2.54
OsR498G1018839400.01	0.54	2.47	2.2676183	1.15E-206	3.35E-207	0.47	0.57	0.58	2.46	2.56	2.39
OsR498G1018841600.01	6.516	2.19	-1.577316	1.15E-37	1.03E-37	5.94	7.01	6.6	2.56	2.08	1.93
OsR498G1018844300.01	0	0.733	4.6748497	0.0001298	0.0002536	0	0	0	0.84	1.01	0.35
OsR498G1018850800.01	5.13	12.726	1.3281618	6.68E-55	4.87E-55	3.56	5.96	5.87	18.73	10.1	9.35
OsR498G1018853400.01	1.02	7.06	2.6347445	8.20E-56	5.92E-56	0.57	2.22	0.27	7.5	6.99	6.69
OsR498G1018854200.01	1.95	5.1	1.3856054	6.53E-25	7.16E-25	2	1.57	2.28	4.65	4.59	6.06
OsR498G1018854900.01	17.46	7.84	-1.159755	4.13E-14	5.65E-14	15.19	19.08	18.11	6.74	7.3	9.48
OsR498G1018855700.01	7.67	16.44	1.0966382	4.86E-17	6.22E-17	7.88	6.74	8.39	15.51	15.44	18.37
OsR498G1018856000.01	2.556	0.87	-1.52809	6.15E-08	1.02E-07	2.94	2.48	2.25	0.3	0.74	1.57
OsR498G1018856300.01	0.473	10.093	4.4057215	0	0	0.42	0.41	0.59	10.54	10.47	9.27
OsR498G1018856700.01	3.873	12.59	1.7026933	1.01E-125	4.35E-126	4.49	3.7	3.43	13.12	12.75	11.9
OsR498G1018858200.01	34.276	3.103	-3.361436	0	0	34.62	34.78	33.43	2.76	3.67	2.88
OsR498G1018861400.01	5.39	39.41	2.8082279	0	0	5.93	5.17	5.07	40.58	38.55	39.1
OsR498G1018861800.01	0.08	2.333	4.8651809	3.07E-42	2.58E-42	0.12	0.04	0.08	2.11	2.72	2.17
OsR498G1018863100.01	0.106	1.04	3.2963381	1.37E-12	1.96E-12	0.17	0.15	0	0.82	0.87	1.43
OsR498G1018863400.01	13.92	6.346	-1.330486	4.86E-123	2.11E-123	14.79	13.57	13.4	6.17	6.72	6.15
OsR498G1018865600.01	15.146	84.246	2.4737926	0	0	13.55	16.9	14.99	83.98	81.74	87.02
OsR498G1018865700.01	2.57	1.06	-1.368277	1.45E-12	2.06E-12	2.74	2.38	2.59	1.09	1.19	0.9
OsR498G1018866600.01	0.873	3.003	1.7870398	5.07E-14	6.96E-14	0.81	1	0.81	2.81	2.92	3.28
OsR498G1018866800.01	13.93	31.05	1.1591954	8.30E-171	2.82E-171	14.29	13.9	13.6	31.37	31.46	30.32
OsR498G1018867600.01	23.486	55.616	1.2169619	7.58E-113	3.51E-113	24.24	23.75	22.47	53.28	51.56	62.01
OsR498G1018869700.01	15.696	37.306	1.2498455	8.82E-122	3.85E-122	16.43	15.28	15.38	38.3	34.42	39.2
OsR498G1018869900.01	0.166	0	-6.02559	2.21E-09	3.49E-09	0.11	0.14	0.25	0	0	0
OsR498G1018872900.01	3.083	6.996	1.1809094	7.49E-95	3.90E-95	2.77	3.1	3.38	6.89	6.83	7.27
OsR498G1018874400.01	0.73	3.773	2.3730088	1.35E-59	9.40E-60	0.74	0.49	0.96	3.69	3.91	3.72
OsR498G1018874800.01	10.043	29.646	1.5787215	4.15E-203	1.23E-203	9.88	10.5	9.75	30.11	29.71	29.12
OsR498G1018879300.01	0.366	0.093	-2.031006	4.76E-06	8.61E-06	0.31	0.38	0.41	0.11	0	0.17
OsR498G1018889900.01	0.07	0	-4.72603	0.0001114	0.0002167	0.05	0.11	0.05	0	0	0
OsR498G1018893600.01	1.276	0.086	-3.888087	5.68E-35	5.29E-35	1.44	1.19	1.2	0.09	0.07	0.1
OsR498G1018898300.01	0.143	0.87	2.5593725	3.48E-10	5.35E-10	0.13	0.26	0.04	0.91	0.86	0.84
OsR498G1018898500.01	1.53	4.323	1.4632965	3.78E-24	4.20E-24	1.33	1.45	1.81	3.78	4.39	4.8
OsR498G1018901500.01	1.743	5.416	1.6434379	7.21E-58	5.10E-58	1.85	1.76	1.62	6.22	5.18	4.85
OsR498G1018903500.01	0.706	0.19	-1.926054	2.19E-07	3.73E-07	0.72	0.57	0.83	0.15	0.15	0.27
OsR498G1018903900.01	1.68	4.906	1.540691	1.30E-62	8.80E-63	1.77	1.53	1.74	4.72	5.17	4.83
OsR498G1018914100.01	10.796	31.793	1.5294031	0	0	10.65	11.19	10.55	31.3	31.74	32.34
OsR498G1018928300.01	1.196	2.71	1.1828091	5.05E-10	7.81E-10	0.98	1.4	1.21	3.26	2.54	2.33
OsR498G1018931800.01	6.873	23.92	1.8475401	1.99E-135	8.07E-136	6.14	7.72	6.76	24.04	23.49	24.23
OsR498G1018934100.01	0.603	0.236	-1.355739	4.30E-07	7.41E-07	0.64	0.53	0.64	0.32	0.12	0.27
OsR498G1018936400.01	0.373	1.06	1.5139295	1.39E-10	2.10E-10	0.34	0.39	0.39	1.39	0.78	1.01
OsR498G1018940100.01	3.263	10.12	1.6405353	6.62E-74	4.04E-74	3.83	3.15	2.81	9.45	9.95	10.96
OsR498G1018940800.01	0.096	0.746	2.9352394	2.00E-19	2.43E-19	0.21	0.02	0.06	0.67	0.75	0.82
OsR498G1018946300.01	0.883	3.166	1.8635259	4.67E-36	4.28E-36	0.56	0.92	1.17	3.38	3.59	2.53
OsR498G1018952600.01	122	3.763	-5.04408	0	0	127.28	117.41	121.31	3.61	4.3	3.38
OsR498G1018954400.01	5.43	1.62	-1.74938	1.30E-30	1.30E-30	5.76	4.61	5.92	1.31	1.55	2
OsR498G1018955900.01	0.116	0.35	1.5593725	5.14E-05	9.79E-05	0.12	0.16	0.07	0.32	0.37	0.36
OsR498G1018957900.01	11.12	57.463	2.3678628	0	0	10.37	11.45	11.54	56.99	55.61	59.79

OsR498G1018962600.01	1044.89	194.85	-2.403162	0	0	1043.1	1066.3	1025.2	201.87	200.74	181.93
OsR498G1018962700.01	0.25	0.066	-2.02559	3.79E-05	7.15E-05	0.28	0.2	0.27	0.05	0.07	0.08
OsR498G1018963700.01	3.276	10.253	1.6589081	9.77E-67	6.34E-67	3.87	2.68	3.28	10.65	10.03	10.08
OsR498G1018966500.01	13.03	0.81	-3.971034	2.01E-208	5.82E-209	13.77	13.82	11.5	0.6	0.97	0.86
OsR498G1018969000.01	31.5	5.273	-2.592468	0	0	31.69	31.05	31.76	4.73	5.02	6.07
OsR498G1018975500.01	5.35	13.573	1.3449817	1.75E-114	8.03E-115	6.66	0.32	9.07	11.08	14.05	15.59
OsR498G1018978800.01	0.403	1.506	1.7711586	2.34E-19	2.84E-19	0.45	0.31	0.45	1.82	1.4	1.3
OsR498G1018979200.01	1.523	7.563	2.3058788	3.92E-133	1.61E-133	1.44	1.7	1.43	7.45	8.63	6.61
OsR498G1018979400.01	0.523	2.136	2.0506205	1.24E-49	9.53E-50	0.51	0.58	0.48	2.1	2.25	2.06
OsR498G1018979500.01	0.826	4.04	2.1482394	7.64E-23	8.67E-23	0.79	0.84	0.85	3.67	4.21	4.24
OsR498G1018982300.01	53.446	17.643	-1.596738	0	0	54.86	50.42	55.06	17.95	17.32	17.66
OsR498G1018985300.01	0.326	0.076	-2.72603	1.03E-07	1.73E-07	0.34	0.54	0.1	0.1	0.08	0.05
OsR498G1018985500.01	0.246	2	2.97441	2.22E-25	2.42E-25	0.19	0.36	0.19	1.81	1.97	2.22
OsR498G1018985700.01	5.293	10.92	1.0131944	4.66E-30	4.69E-30	4.96	5.45	5.47	11.07	10.85	10.84
OsR498G1018986200.01	4.433	0.283	-3.754694	1.78E-63	1.20E-63	4.09	3.98	5.23	0.39	0.24	0.22
OsR498G1018986700.01	60.983	22.2	-1.499189	0	0	62.57	61.2	59.18	21.42	21.39	23.79
OsR498G1018987100.01	1.8	7.44	2.0468841	2.06E-49	1.59E-49	1.78	2.01	1.61	7.82	7.67	6.83
OsR498G1018993600.01	0.023	0.313	3.6748497	0.0003128	0.0006259	0.07	0	0	0.58	0.21	0.15
OsR498G1018996000.01	5.05	2.476	-1.027416	6.57E-13	9.28E-13	5.29	4.46	5.4	2.7	2.08	2.65
OsR498G1019003500.01	0.103	0.34	1.758263	1.93E-07	3.28E-07	0.02	0.22	0.07	0.44	0.37	0.21
OsR498G1019004800.01	387.503	55.646	-2.805208	0	0	390.7	402.49	369.32	56.57	54.22	56.15
OsR498G1019005800.01	0.446	1.32	1.5593725	2.27E-06	4.04E-06	0.77	0.25	0.32	1.31	1.05	1.6
OsR498G1019006300.01	0.856	0.286	-1.585462	6.58E-15	8.84E-15	0.89	0.76	0.92	0.31	0.29	0.26
OsR498G1019008300.01	0	0.293	5.6182661	1.33E-07	2.24E-07	0	0	0	0.24	0.28	0.36
OsR498G1019008700.01	0.036	0.446	3.637375	3.58E-09	5.69E-09	0.11	0	0	0.49	0.49	0.36
OsR498G1019009100.01	0.776	2.92	1.9138295	3.43E-22	3.95E-22	0.89	0.87	0.57	3.01	2.94	2.81
OsR498G1019009300.01	0.97	3.656	1.7935654	2.37E-38	2.11E-38	0.98	1.02	0.91	3.65	4.25	3.07
OsR498G1019009500.01	2.393	10.033	2.0675194	1.38E-98	7.03E-99	1.72	2.79	2.67	10.54	10.4	9.16
OsR498G1019015500.01	10.893	28.34	1.3668824	9.57E-142	3.75E-142	12.29	10.58	9.81	28.62	28.5	27.9
OsR498G1019015800.01	0.716	1.656	1.2366832	2.13E-21	2.49E-21	0.69	0.68	0.78	1.59	1.56	1.82
OsR498G1019015900.01	5.43	13.06	1.2652597	9.22E-34	8.74E-34	6.31	6.05	3.93	12.06	14.41	12.71
OsR498G1019017900.01	11.603	32.086	1.3670557	8.13E-161	2.91E-161	11.8	12.71	10.3	33.28	29.91	33.07
OsR498G1019024000.01	191.763	91.95	-1.061609	0	0	200.04	184.77	190.48	92.37	88.14	95.34
OsR498G1019027100.01	11.446	23.996	1.0673307	1.43E-105	6.90E-106	10.71	11.74	11.89	24.59	24.16	23.24
OsR498G1019030000.01	0.32	4.053	3.6684942	2.91E-90	1.57E-90	0.44	0.27	0.25	3.78	3.84	4.54
OsR498G1019030200.01	0.616	7.993	3.6961707	4.06E-82	2.33E-82	0.66	0.59	0.6	9.61	7.25	7.12
OsR498G1019030600.01	0.283	0.036	-3.221987	1.15E-11	1.69E-11	0.25	0.23	0.37	0.02	0.02	0.07
OsR498G1019031400.01	22.943	9.24	-1.314553	8.20E-138	3.28E-138	22.96	22.88	22.99	8.57	8.73	10.42
OsR498G1019033100.01	3.02	0.233	-3.698015	7.87E-53	5.87E-53	2.82	2.58	3.66	0.1	0.28	0.32
OsR498G1019033400.01	0	0.213	5.4979719	4.04E-07	6.96E-07	0	0	0	0.22	0.25	0.17
OsR498G1019036300.01	544.433	117.02	-2.227017	0	0	527.58	573.99	531.73	117.13	114.59	119.34
OsR498G1019038500.01	2.803	11.973	2.1004351	5.36E-148	2.03E-148	2.88	2.66	2.87	11.37	12.5	12.05
OsR498G1019040200.01	0.96	0.426	-1.301714	1.27E-10	1.92E-10	0.94	0.79	1.15	0.41	0.38	0.49
OsR498G1019042200.01	1.626	0.283	-2.520355	7.57E-28	7.91E-28	1.29	1.67	1.92	0.33	0.23	0.29
OsR498G1019043400.01	2.153	0.606	-1.832945	2.38E-08	3.90E-08	1.58	1.38	3.5	0.72	0.45	0.65
OsR498G1019047400.01	0.98	3.88	1.9841793	5.58E-38	4.98E-38	0.69	0.87	1.38	3.77	4.05	3.82
OsR498G1019049200.01	105.793	257.05	1.2789952	0	0	104.09	108	105.29	257.39	251.58	262.18
OsR498G1019049300.01	5.326	12.513	1.2305512	8.91E-64	5.96E-64	5.19	5.25	5.54	12	13.32	12.22
OsR498G1019051400.01	4.363	12.946	1.5675997	1.04E-92	5.49E-93	4.51	4.49	4.09	13.56	13.1	12.18
OsR498G1019053100.01	3.02	6.353	1.077247	1.05E-27	1.11E-27	2.97	3.05	3.04	6.45	5.86	6.75
OsR498G1019054500.01	0.283	0.766	1.4250714	0.0002539	0.0005045	0.34	0.34	0.17	0.67	0.72	0.91
OsR498G1019056900.01	0.57	0.05	-3.52809	2.38E-08	3.90E-08	0.83	0.6	0.28	0	0.1	0.05
OsR498G1019057600.01	2.526	9.06	1.8338977	4.27E-90	2.31E-90	2.56	2.7	2.32	8.57	9.61	9
OsR498G1019058300.01	1.433	3.53	1.3116744	1.47E-20	1.74E-20	1.33	1.48	1.49	3.69	3.24	3.66
OsR498G1019058700.01	2.386	4.806	1.0091754	2.57E-14	3.50E-14	2.37	1.91	2.88	4.42	4.58	5.42
OsR498G1019060300.01	0.826	16.556	4.3371656	4.11E-242	1.05E-242	0.58	0.93	0.97	14.77	17.82	17.08
OsR498G1019064100.01	4.41	11.71	1.4366059	3.06E-44	2.51E-44	4.09	4.26	4.88	11.81	10.64	12.68
OsR498G1019064500.01	2.286	0.363	-2.633273	2.13E-39	1.87E-39	2.13	2.16	2.57	0.44	0.28	0.37
OsR498G1019065900.01	2.26	0.073	-4.956327	5.21E-16	6.82E-16	2.12	2	2.66	0	0	0.22
OsR498G1019066700.01	7.63	17.426	1.1861025	3.50E-60	2.41E-60	7.32	8.64	6.93	16.99	16.94	18.35
OsR498G1019069600.01	0.07	1.86	4.7817649	8.30E-21	9.81E-21	0.14	0	0.07	1.85	1.97	1.76
OsR498G1019070200.01	1.51	4.863	1.6995227	1.22E-93	6.42E-94	1.46	1.5	1.57	4.94	5	4.65
OsR498G1019071400.01	0.136	0.013	-3.195515	6.51E-05	0.0001248	0.07	0.16	0.18	0.02	0	0.02
OsR498G1019073200.01	0.506	6.313	3.4753082	3.36E-114	1.54E-114	0.6	0.53	0.39	5.81	7.05	6.08
OsR498G1019074000.01	6.153	1.23	-2.333489	4.45E-41	3.82E-41	6.35	5.69	6.42	1.06	1.12	1.51
OsR498G1019074400.01	0.806	2.04	1.3421417	1.94E-06	3.45E-06	0.94	0.7	0.78	1.9	1.81	2.41
OsR498G1019075200.01	3.146	24.176	2.3398184	1.50E-175	5.01E-176	3.32	3.03	3.09	26.89	23.58	22.06
OsR498G1019075300.01	0	4.933	9.1139613	2.45E-53	1.82E-53	0	0	0	5.37	5.25	4.18
OsR498G1019075700.01	0	1.993	7.5289988	3.79E-22	4.36E-22	0	0	0	3.01	1.47	1.5

OsR498G1019075900.01	0.06	6.726	6.7774647	7.79E-78	4.62E-78	0	0.06	0.12	7.11	6.13	6.94
OsR498G1019076300.01	0.62	14.41	4.5336422	1.32E-173	4.44E-174	0.51	0.73	0.62	14.12	14.61	14.5
OsR498G1019076500.01	0.103	2.26	4.4769103	8.60E-17	1.11E-16	0.21	0.1	0	2.41	2.65	1.72
OsR498G1019078700.01	5.32	0.186	-4.841507	7.94E-41	6.84E-41	5.03	5.18	5.75	0.09	0.09	0.38
OsR498G1019078900.01	8.286	29.946	1.8513383	1.20E-129	5.02E-130	8.44	8.9	7.52	29.16	29.88	30.8
OsR498G1019079100.01	0.366	5.9	4.0208844	7.22E-74	4.41E-74	0.42	0.21	0.47	6.23	5.54	5.93
OsR498G1019079300.01	0.243	0.73	1.6024412	0.0002771	0.0005524	0.33	0.2	0.2	0.64	0.83	0.72
OsR498G1019080300.01	1.443	0.2	-2.823546	5.17E-26	5.57E-26	1.53	1.42	1.38	0.16	0.19	0.25
OsR498G1019080500.01	1.53	30.406	4.3106933	0	0	1.79	1.4	1.4	29.61	28.13	33.48
OsR498G1019080700.01	0.09	3.353	5.2411965	1.87E-37	1.68E-37	0.07	0.07	0.13	3.37	2.9	3.79
OsR498G1019080900.01	49.416	169.4	1.7761159	0	0	49.08	48.15	51.02	165.96	166.23	176
OsR498G1019083200.01	380.553	145.62	-1.387296	0	0	400.63	372.14	368.89	141.39	141.41	154.06
OsR498G1019083900.01	20.593	7.64	-1.431865	3.31E-52	2.48E-52	18.96	19.39	23.43	7.48	7.69	7.75
OsR498G1019085000.01	3.363	9.686	1.5236193	1.58E-43	1.31E-43	3.08	3.6	3.41	9.86	8.73	10.47
OsR498G1019088600.01	23.723	82.72	1.7915084	0	0	25	22.27	23.9	80.61	79.49	88.06
OsR498G1019090300.01	0.166	0.51	1.6118399	0.0007971	0.0016457	0.06	0.33	0.11	0.38	0.54	0.61
OsR498G1019091500.01	1.773	5.806	1.7169912	6.77E-70	4.26E-70	1.84	1.6	1.88	5.72	5.72	5.98
OsR498G1019092300.01	2.383	1.063	-1.166946	5.64E-09	9.03E-09	2.16	2.47	2.52	0.79	1.3	1.1
OsR498G1019092700.01	3.593	0.133	-4.738308	7.34E-56	5.30E-56	4.19	3.07	3.52	0.13	0.04	0.23
OsR498G1019099800.01	0.1	0.873	3.103693	9.95E-08	1.67E-07	0.15	0	0.15	0.67	1.04	0.91
OsR498G1019100000.01	1.023	6.593	2.7125022	2.50E-65	1.65E-65	1.28	0.9	0.89	6.9	6.25	6.63
OsR498G1019100500.01	1.746	4.843	1.4514571	3.14E-22	3.61E-22	1.76	1.92	1.56	4.73	4.77	5.03
OsR498G1019102500.01	17.093	115.53	2.7423273	0	0	16.85	16.66	17.77	116.94	110.33	119.33
OsR498G1019102900.01	12.433	72.553	2.5329315	0	0	12.06	12	13.24	72.11	70.45	75.1
OsR498G1019111200.01	410.51	127.88	-1.684505	0	0	416.18	418.16	397.19	128.33	122.77	132.53
OsR498G1019115200.01	6.166	2.893	-1.177415	1.40E-45	1.13E-45	6.11	6.83	5.56	3.24	3.1	2.34
OsR498G1019115300.01	14.976	4.69	-1.699258	1.77E-175	5.89E-176	13.86	14.59	16.48	4.72	4.81	4.54
OsR498G1019118600.01	2.57	8.626	1.7214292	7.18E-83	4.11E-83	2.49	2.65	2.57	8.71	8.54	8.63
OsR498G1019122600.01	76.3	152.85	1.0054369	0	0	73.76	75.9	79.24	155.89	147	155.67
OsR498G1019123300.01	0.216	0.02	-3.72603	0.0002637	0.0005248	0.25	0.15	0.25	0	0.06	0
OsR498G1019124000.01	0.193	0.763	1.97441	4.66E-07	8.04E-07	0.22	0.09	0.27	0.87	0.61	0.81
OsR498G1019125100.01	6.293	24.853	1.9546571	1.35E-285	2.97E-286	6.25	6.18	6.45	24.76	24.12	25.68
OsR498G1019125300.01	10.753	63.3	2.492067	0	0	9.96	10.96	11.34	64.26	62.08	63.56
OsR498G1019125600.01	2.446	61.65	4.7412552	0	0	2.13	2.88	2.33	61.37	61.92	61.66
OsR498G1019125700.01	0.293	1.366	2.2331442	8.51E-18	1.07E-17	0.41	0.19	0.28	1.15	1.08	1.87
OsR498G1019131700.01	4.263	17.833	2.0575056	8.81E-232	2.33E-232	4.18	4.24	4.37	18.85	16.68	17.97
OsR498G1019134400.01	1.07	3.32	1.6339194	1.16E-23	1.30E-23	1.03	1.05	1.13	3.09	3.36	3.51
OsR498G1019136700.01	7.806	30.403	1.9648762	0	0	7.32	8.67	7.43	31.15	29.32	30.74
OsR498G1019139800.01	2.95	11.076	1.9111877	3.98E-143	1.55E-143	2.73	2.54	3.58	10.12	12.07	11.04
OsR498G1019140200.01	0.12	1.24	3.33698	6.86E-15	9.22E-15	0.05	0.21	0.1	1.23	1.17	1.32
OsR498G1019140600.01	21.54	107.04	2.2539644	0	0	21.95	21.52	21.15	107.71	109.24	104.18
OsR498G1019142000.01	1.103	2.37	1.1532839	6.72E-15	9.02E-15	1.16	1.13	1.02	2.24	2.42	2.45
OsR498G1019142900.01	0.04	0.286	2.8813006	0.000469	0.0009493	0	0.12	0	0.28	0.17	0.41
OsR498G1019144500.01	5.983	21.323	1.8362341	0	0	6.32	5.69	5.94	22.02	21.61	20.34
OsR498G1019148500.01	10.226	31.833	1.6466882	3.73E-293	8.02E-294	10.74	9.49	10.45	30.12	31.9	33.48
OsR498G1019155100.01	10.126	113.78	3.5006131	0	0	9.86	10.98	9.54	114.58	114.34	112.41
OsR498G1019156700.01	0.053	0.193	1.8994695	5.59E-05	0.0001066	0.09	0.07	0	0.12	0.24	0.22
OsR498G1019158000.01	2.743	11.353	2.036226	9.23E-98	4.71E-98	2.48	3.01	2.74	11.22	12.3	10.54
OsR498G1019160600.01	2.93	0.11	-4.742581	4.74E-62	3.21E-62	3.26	2.69	2.84	0.03	0.1	0.2
OsR498G1019162400.01	0.436	3.263	2.8990026	4.14E-67	2.67E-67	0.51	0.31	0.49	3.24	2.83	3.72
OsR498G1019166100.01	0.9	7.896	3.1233169	3.05E-142	1.19E-142	1.2	0.84	0.66	8.16	7.73	7.8
OsR498G1019170200.01	1.066	0	-7.134115	1.86E-17	2.36E-17	1.26	0.88	1.06	0	0	0
OsR498G1019170400.01	0.256	0	-5.02559	1.85E-05	3.43E-05	0.15	0.48	0.14	0	0	0
OsR498G1019172000.01	6.436	0.036	-7.600499	1.14E-126	4.86E-127	6.09	7.11	6.11	0.04	0.03	0.04
OsR498G1019174900.01	1.23	3.226	1.3529216	6.17E-15	8.28E-15	1.65	1.31	0.73	3.31	3.13	3.24
OsR498G1019181700.01	10.053	3.14	-1.680954	3.55E-99	1.79E-99	10.47	9.5	10.19	2.89	3.5	3.03
OsR498G1019182000.01	0.256	0	-4.374672	0.0006242	0.0012755	0.12	0.25	0.4	0	0	0
OsR498G1019189700.01	0.23	0.716	1.8574073	0.0001446	0.000283	0.07	0.31	0.31	0.99	0.34	0.82
OsR498G1019190000.01	0.306	0.66	1.0983	6.71E-06	1.22E-05	0.22	0.25	0.45	0.61	0.76	0.61
OsR498G1019190600.01	0.176	1.28	2.8687005	8.26E-29	8.49E-29	0.16	0.16	0.21	1.28	1.35	1.21
OsR498G1019191600.01	0.1	0.753	2.97441	5.43E-07	9.41E-07	0.08	0	0.22	0.44	1.13	0.69
OsR498G1019195100.01	1.786	5.836	1.7113756	6.52E-48	5.13E-48	1.57	2.06	1.73	5.45	5.91	6.15
OsR498G1019196100.01	0.793	0.316	-1.328153	4.37E-06	7.88E-06	0.58	0.8	1	0.44	0.09	0.42
OsR498G1019196500.01	30.526	164.22	2.4231265	0	0	30.02	31.44	30.12	167.99	165.94	158.72
OsR498G1019198300.01	0.023	0.27	3.615956	0.0004286	0.0008648	0	0	0.07	0.13	0.45	0.23
OsR498G1019198700.01	13.2	59.956	2.1820899	0	0	13.17	12.99	13.44	57.89	59.05	62.93
OsR498G1019201700.01	0.83	0.233	-1.843752	4.08E-08	6.74E-08	0.86	0.74	0.89	0.4	0.11	0.19
OsR498G1019202400.01	0.76	0.236	-1.762556	5.32E-08	8.83E-08	0.82	0.92	0.54	0.22	0.22	0.27
OsR498G1019203700.01	0.53	24.446	5.5299887	0	0	0.77	0.36	0.46	22.83	22.59	27.92

OsR498G1019205600.01	7.5	34.973	1.2316836	4.13E-99	2.09E-99	6.43	6.19	9.88	33.06	29.57	42.29
OsR498G1019205900.01	3.23	7.08	1.11386	2.80E-21	3.29E-21	2.61	3.29	3.79	7.18	6.58	7.48
OsR498G1019206100.01	0.52	1.28	1.3093942	2.39E-14	3.26E-14	0.55	0.58	0.43	1.33	1.15	1.36
OsR498G1019206800.01	0.53	6.323	3.5645902	8.65E-55	6.30E-55	0.35	0.62	0.62	6.11	5.2	7.66
OsR498G1019209200.01	19.35	100.92	2.2829058	7.89E-261	1.86E-261	17.23	20.2	20.62	99.82	97.62	105.32
OsR498G1019209600.01	14.776	35.61	1.2670175	7.05E-157	2.57E-157	15.52	13.99	14.82	34.86	35.09	36.88
OsR498G1019210100.01	13.543	29.036	1.0990258	1.82E-87	1.00E-87	12.19	14.17	14.27	27.76	30.45	28.9
OsR498G1019210300.01	2.466	5.6	1.1847698	1.26E-30	1.26E-30	2.45	2.54	2.41	6.06	5.59	5.15
OsR498G1019210800.01	1.896	4.65	1.2223375	6.06E-25	6.64E-25	1.85	1.65	2.19	4.88	4.31	4.76
OsR498G1019218100.01	0	0.173	4.7817649	7.17E-05	0.0001377	0	0	0	0.11	0.26	0.15
OsR498G1019219400.01	0.056	0.623	3.4853719	1.56E-12	2.23E-12	0.1	0.07	0	0.49	0.81	0.57
OsR498G1019222200.01	9.893	3.853	-1.362962	1.40E-70	8.80E-71	9.55	9.87	10.26	3.89	3.73	3.94
OsR498G1019225500.01	211.103	721.74	1.7743931	0	0	206.77	218.16	208.38	729.05	728.31	707.87
OsR498G1019226200.01	16.936	36.066	1.0896487	6.36E-214	1.79E-214	16.39	17.76	16.66	37.34	36.67	34.19
OsR498G1019226300.01	6.183	13.433	1.1131624	3.42E-78	2.02E-78	5.49	6.19	6.87	13.39	13.79	13.12
OsR498G1019227500.01	0.9	2.143	1.2449491	0.0001895	0.0003736	0.7	0.57	1.43	2.01	1.86	2.56
OsR498G1019231300.01	0.2	0.68	1.7469995	2.94E-05	5.51E-05	0.25	0.05	0.3	0.44	0.69	0.91
OsR498G1019231900.01	20.926	49.68	1.2643691	0	0	20.83	20.72	21.23	49.35	52.42	47.27
OsR498G1019234100.01	0.57	3.486	2.6091255	2.49E-52	1.87E-52	0.39	0.66	0.66	3.34	3.71	3.41
OsR498G1019235000.01	21.84	9.37	-1.220484	1.65E-129	6.93E-130	20.14	23.01	22.37	9.97	9.69	8.45
OsR498G1019235500.01	7.903	24.65	1.6892767	1.82E-216	5.06E-217	7.82	7.52	8.37	26.45	25.34	22.16
OsR498G1019235700.01	0.186	0.85	2.1760438	0.0002409	0.0004782	0.34	0.11	0.11	0.77	0.88	0.9
OsR498G1019237200.01	0.06	0.476	3.0898872	1.29E-10	1.95E-10	0.06	0.06	0.06	0.38	0.66	0.39
OsR498G1019246300.01	105.306	48.816	-1.111881	2.46E-301	5.18E-302	96.46	110.37	109.09	50.11	48.26	48.08
OsR498G1019249900.01	1.74	4.28	1.2988021	3.72E-18	4.63E-18	1.94	1.59	1.69	5.08	3.74	4.02
OsR498G1019250200.01	9.793	34.106	1.8197828	5.08E-212	1.45E-212	9.2	9.87	10.31	33.63	33.21	35.48
OsR498G1019250500.01	0.123	0.493	1.9967778	1.68E-08	2.73E-08	0.07	0.16	0.14	0.43	0.52	0.53
OsR498G1019250800.01	8.826	18.63	1.3748078	8.58E-127	3.66E-127	8.39	8.5	9.59	18.35	18.97	18.57
OsR498G1019253100.01	5.776	60.676	3.3949215	0	0	4.95	6.36	6.02	62.52	61.56	57.95
OsR498G1019254300.01	0.01	0.07	2.97441	0.0002639	0.0005255	0	0	0.03	0.08	0.05	0.08
OsR498G1019255200.01	57.066	17.973	-1.822585	0	0	58.82	58.24	54.14	17.85	17.75	18.32
OsR498G1019255800.01	3.1	16.91	2.4477399	0	0	3.09	3.13	3.08	16.72	17.12	16.89
OsR498G1019256000.01	25.406	225.63	3.1548901	0	0	26.95	24.69	24.58	228.55	227.93	220.4
OsR498G1019256300.01	0.936	0.1	-3.02559	6.46E-10	1.00E-09	0.83	1.21	0.77	0.06	0.14	0.1
OsR498G1019258300.01	2.306	0.426	-2.437016	1.28E-37	1.15E-37	2.28	2.56	2.08	0.33	0.43	0.52
OsR498G1019260300.01	6.306	120.15	4.2501929	0	0	6.44	5.9	6.58	117.36	114.22	128.88
OsR498G1019260600.01	12.246	26.16	1.1340666	2.05E-91	1.10E-91	13.69	10.63	12.42	25.21	26.35	26.92
OsR498G1019262500.01	15.373	32.87	1.0944657	4.83E-91	2.59E-91	14.66	16.13	15.33	33.42	30.04	35.15
OsR498G1019262800.01	4.843	47.293	3.2718949	0	0	4.33	5.32	4.88	46.19	47.68	48.01
OsR498G1019263400.01	2.14	5.3	1.3045586	3.71E-51	2.82E-51	2.16	2.24	2.02	5.2	5.86	4.84
OsR498G1019264900.01	0.31	0.823	1.3286385	0.0001639	0.000322	0.15	0.44	0.34	0.89	0.87	0.71
OsR498G1119277500.01	17.286	37.63	1.078723	1.81E-149	6.81E-150	17.65	17.27	16.94	38.32	37.28	37.29
OsR498G1119284300.01	35.243	12.82	-1.460233	0	0	34.54	35.42	35.77	14.12	12.01	12.33
OsR498G1119287100.01	3.276	7.79	1.2646038	7.55E-40	6.59E-40	3.29	3.23	3.31	8.09	7.6	7.68
OsR498G1119289000.01	1.866	8.47	2.1041902	1.49E-59	1.03E-59	1.66	2	1.94	7.77	9.67	7.97
OsR498G1119290000.01	4.396	11.38	1.3996301	4.26E-33	4.08E-33	4.1	3.83	5.26	11.33	12.48	10.33
OsR498G1119291800.01	0.793	10.103	3.6524819	1.19E-163	4.21E-164	0.81	0.58	0.99	10.16	10.38	9.77
OsR498G1119292200.01	56.17	22.25	-1.162447	8.96E-303	1.88E-303	56.28	56.03	56.2	23.98	22.05	20.72
OsR498G1119293000.01	36.04	334.6	3.2027149	0	0	34.01	38.42	35.69	339.58	341.67	322.54
OsR498G1119293100.01	1.99	3.54	1.0277966	3.37E-27	3.56E-27	2.2	1.76	2.01	3.55	3.92	3.15
OsR498G1119303000.01	0.26	0.793	1.6133113	3.35E-10	5.15E-10	0.25	0.29	0.24	0.67	0.97	0.74
OsR498G1119303200.01	0	0.236	4.97441	2.21E-05	4.12E-05	0	0	0	0.04	0.31	0.36
OsR498G1119303400.01	0.046	0.216	2.1808608	9.99E-09	1.61E-08	0.03	0.07	0.04	0.23	0.21	0.21
OsR498G1119304900.01	1.793	6.14	1.7553023	4.99E-42	4.23E-42	1.46	1.6	2.32	4.87	6.49	7.06
OsR498G1119309800.01	1.186	0.403	-1.585017	5.04E-06	9.12E-06	1.32	1.16	1.08	0.57	0.19	0.45
OsR498G1119313900.01	30.903	68.856	1.1056872	1.02E-126	4.37E-127	31.28	31.48	29.95	68.4	65.98	72.19
OsR498G1119317700.01	0.026	0.513	4.2223375	8.50E-06	1.55E-05	0	0	0.08	0.4	0.56	0.58
OsR498G1119318900.01	0.213	1.013	2.1762089	6.74E-12	9.81E-12	0.21	0.22	0.21	0.96	1.62	0.46
OsR498G1119321900.01	3.133	6.576	1.0545803	1.28E-16	1.65E-16	3.25	2.72	3.43	6.44	6.42	6.87
OsR498G1119322300.01	536.54	91.703	-2.53739	0	0	533.62	527.05	548.95	88.22	91.06	95.83
OsR498G1119323300.01	105.756	2.933	-5.006317	0	0	107.6	104.35	105.32	2.91	2.4	3.49
OsR498G1119323800.01	43.603	353.8	3.0361445	0	0	40.18	47.91	42.72	353.02	352.32	356.05
OsR498G1119323900.01	1.063	4.416	1.9808494	6.84E-24	7.63E-24	1.08	0.87	1.24	4.07	3.84	5.34
OsR498G1119325200.01	12.8	122.5	3.2558227	0	0	12.52	12.04	13.84	117.64	123.24	126.62
OsR498G1119327400.01	4.463	1.676	-1.418601	1.04E-18	1.28E-18	4.4	4.72	4.27	1.46	1.45	2.12
OsR498G1119329500.01	0.14	1.19	3.0163976	9.85E-13	1.40E-12	0.26	0.1	0.06	1.01	1.33	1.23
OsR498G1119330000.01	0.25	1.27	2.3132119	1.66E-12	2.37E-12	0.22	0.31	0.22	1.38	1.19	1.24
OsR498G1119330300.01	4.503	1.096	-2.081592	3.15E-65	2.07E-65	4.43	4.47	4.61	0.99	0.96	1.34
OsR498G1119330600.01	0.333	0.09	-1.895387	3.78E-05	7.14E-05	0.43	0.17	0.4	0.03	0.06	0.18

OsR498G1119331000.01	0.59	0.07	-3.172431	8.98E-13	1.27E-12	0.83	0.4	0.54	0.06	0.06	0.09
OsR498G1119333800.01	0.22	0.03	-2.932481	1.12E-06	1.96E-06	0.2	0.33	0.13	0	0.02	0.07
OsR498G1119336000.01	21.073	9.876	-1.096277	5.92E-48	4.66E-48	19.57	22.77	20.88	9.68	9.12	10.83
OsR498G1119348100.01	0.126	0.556	2.144335	9.15E-05	0.0001771	0.19	0.06	0.13	0.55	0.61	0.51
OsR498G1119348600.01	0.776	0.296	-1.361589	2.40E-08	3.93E-08	0.89	0.66	0.78	0.29	0.44	0.16
OsR498G1119350300.01	1.39	0.26	-2.561643	3.27E-14	4.47E-14	1.63	1.2	1.34	0.09	0.32	0.37
OsR498G1119352100.01	0.6	24.723	5.3617693	0	0	0.52	0.64	0.64	24.14	24.14	25.89
OsR498G1119354300.01	321.73	36.13	-3.156826	0	0	308.52	325.73	330.94	37.01	35.51	35.87
OsR498G1119354800.01	43.943	138.74	1.6517292	0	0	43.86	42.81	45.16	136.04	132.22	147.96
OsR498G1119355200.01	2.15	4.426	1.0424092	4.21E-19	5.14E-19	1.58	2.05	2.82	4.58	4.39	4.31
OsR498G1119356300.01	0.84	1.803	1.0977924	0.0002721	0.000542	0.99	0.81	0.72	1.67	1.22	2.52
OsR498G1119359200.01	7.703	15.62	1.0699788	1.38E-47	1.09E-47	8.24	8.02	6.85	15.33	15.48	16.05
OsR498G1119359600.01	19.376	134.11	2.8106082	0	0	20.71	19.02	18.4	136.7	136.44	129.19
OsR498G1119360200.01	0.126	2.196	4.129328	1.27E-45	1.02E-45	0.13	0.15	0.1	2.33	2.17	2.09
OsR498G1119360400.01	3.93	11.143	1.5111295	3.62E-83	2.06E-83	3.92	3.81	4.06	11.06	11.37	11
OsR498G1119361400.01	0.286	0.696	1.2963381	0.0003489	0.0006997	0.29	0.24	0.33	0.51	0.73	0.85
OsR498G1119361700.01	7.84	22.04	1.4735098	2.43E-104	1.18E-104	7.23	8.32	7.97	22.65	21.31	22.16
OsR498G1119362400.01	2.99	10.243	1.7735326	1.35E-109	6.37E-110	2.93	3	3.04	10.01	10.21	10.51
OsR498G1119363200.01	12.236	45.066	1.879589	1.45E-219	4.00E-220	12.38	10.3	14.03	45.28	42.6	47.32
OsR498G1119363500.01	0.053	0.833	3.9822782	4.02E-15	5.36E-15	0	0.08	0.08	0.85	1.17	0.48
OsR498G1119364600.01	0.383	1.55	2.0487885	3.83E-15	5.12E-15	0.45	0.38	0.32	1.36	1.86	1.43
OsR498G1119364700.01	1.73	4.573	1.4001059	1.19E-21	1.38E-21	1.81	1.36	2.02	4.2	4.27	5.25
OsR498G1119365200.01	0.156	0.386	1.2822092	7.64E-05	0.0001469	0.13	0.16	0.18	0.37	0.48	0.31
OsR498G1119366100.01	8.046	67.49	3.06603	0	0	8.05	7.82	8.27	63.08	66.27	73.12
OsR498G1119366400.01	0.293	1.12	2.0100339	2.37E-10	3.62E-10	0.21	0.29	0.38	0.77	1.13	1.46
OsR498G1119366500.01	0.433	4.663	3.4555366	2.74E-89	1.49E-89	0.47	0.49	0.34	5.11	4.52	4.36
OsR498G1119366700.01	0.016	0.213	3.7817649	0.0001711	0.0003365	0.05	0	0	0.09	0.41	0.14
OsR498G1119367200.01	0.01	0.086	3.3391063	0.0008819	0.0018283	0	0	0.03	0.1	0.14	0.02
OsR498G1119367400.01	0.406	0.963	1.243591	1.37E-15	1.80E-15	0.42	0.38	0.42	0.86	0.99	1.04
OsR498G1119370100.01	1.3	4.406	1.6987963	8.94E-47	7.12E-47	1.36	1.31	1.23	5.06	4.37	3.79
OsR498G1119370400.01	1.76	12.763	2.8257391	5.30E-230	1.41E-230	1.83	1.76	1.69	13.13	12.56	12.6
OsR498G1119371300.01	0.006	1.03	7.1676889	2.61E-34	2.45E-34	0	0.02	0	1	1.14	0.95
OsR498G1119371800.01	0.053	0.326	2.5593725	6.24E-06	1.13E-05	0.03	0.1	0.03	0.29	0.32	0.37
OsR498G1119374800.01	1.306	9.943	2.9136953	5.40E-207	1.57E-207	1.2	1.24	1.48	11.09	9.46	9.28
OsR498G1119375500.01	0.026	0.323	3.6709603	7.89E-07	1.38E-06	0	0.08	0	0.41	0.28	0.28
OsR498G1119377100.01	58.506	150.99	1.327034	0	0	58.81	58.12	58.59	148.51	156.31	148.14
OsR498G1119377400.01	2.046	0.52	-1.975892	9.55E-16	1.26E-15	2.32	1.58	2.24	0.42	0.41	0.73
OsR498G1119377600.01	4.28	21.063	2.3005286	4.76E-244	1.21E-244	4.47	4.15	4.22	21.28	20.64	21.27
OsR498G1119380000.01	0.816	10.496	3.6846972	4.48E-135	1.82E-135	0.76	1.08	0.61	11.47	9.04	10.98
OsR498G1119380200.01	12.2	31.57	1.3297602	1.31E-227	3.51E-228	12.33	13.33	10.94	30.25	33.45	31.01
OsR498G1119382000.01	9.396	3.963	-1.220053	4.12E-50	3.16E-50	9.04	10.25	8.9	4.66	4.2	3.03
OsR498G1119384000.01	0.856	1.856	1.0973124	2.25E-11	3.33E-11	0.88	0.74	0.95	1.57	1.9	2.1
OsR498G1119384300.01	20.22	42.576	1.0951045	1.41E-183	4.54E-184	19.08	22.22	19.36	41.01	43.92	42.8
OsR498G1119390000.01	7.243	22.003	1.6038911	1.01E-84	5.70E-85	7.84	7.4	6.49	22.77	23.15	20.09
OsR498G1119390600.01	3.603	9.686	1.4334372	5.57E-29	5.70E-29	3.92	3.39	3.5	10.98	9.1	8.98
OsR498G1119391800.01	1.583	3.636	1.1968024	1.10E-09	1.72E-09	1.11	1.99	1.65	3.56	3.28	4.07
OsR498G1119392100.01	5.016	11.243	1.0300738	1.62E-51	1.22E-51	5.34	4.65	5.06	11.25	12.62	9.86
OsR498G1119394100.01	5.453	24.663	1.8549807	5.04E-253	1.22E-253	5.76	5.71	4.89	25.23	26.12	22.64
OsR498G1119395800.01	39.063	94.896	1.2821681	0	0	38.5	39.09	39.6	94.21	96.53	93.95
OsR498G1119395900.01	2.053	5.266	1.3573732	7.19E-25	7.90E-25	2.01	1.78	2.37	5.08	5.17	5.55
OsR498G1119398200.01	0.513	51.13	6.6411665	0	0	0.62	0.55	0.37	51.17	48.39	53.83
OsR498G1119402100.01	3.083	19.31	2.6339851	0	0	3.39	2.51	3.35	18.94	19.29	19.7
OsR498G1119405700.01	0.033	0.56	4.1282153	1.26E-20	1.50E-20	0.02	0.08	0	0.5	0.58	0.6
OsR498G1119408100.01	1.27	0.09	-3.830047	7.37E-07	1.28E-06	0	0.89	2.92	0	0.27	0
OsR498G1119408600.01	3.55	8.953	1.3253958	3.99E-78	2.36E-78	3.65	3.41	3.59	9.25	8.73	8.88
OsR498G1119408900.01	7.033	18.763	1.4213769	6.47E-70	4.07E-70	6.14	6.73	8.23	19.7	18.45	18.14
OsR498G1119409200.01	0.236	8.713	5.2551807	9.86E-74	6.02E-74	0.31	0.24	0.16	8.46	8.91	8.77
OsR498G1119409400.01	0.06	1.843	4.9286063	5.77E-16	7.56E-16	0	0	0.18	2.54	1.6	1.39
OsR498G1119410000.01	0.153	2.06	3.802229	2.63E-30	2.64E-30	0.18	0.1	0.18	2.15	2.08	1.95
OsR498G1119411100.01	0.326	0.02	-4.383142	6.75E-11	1.01E-10	0.34	0.29	0.35	0.03	0.03	0
OsR498G1119414400.01	0.963	2.826	1.5870642	1.23E-21	1.43E-21	1.01	0.74	1.14	2.76	2.62	3.1
OsR498G1119417700.01	0.096	0.583	2.2374444	0.000145	0.0002838	0.09	0.11	0.09	1.04	0.5	0.21
OsR498G1119418100.01	2.9	0.22	-3.72603	3.19E-33	3.05E-33	2.86	3	2.84	0.16	0.16	0.34
OsR498G1119420800.01	0.336	0.073	-2.195515	0.0008316	0.0017206	0.51	0.22	0.28	0	0.11	0.11
OsR498G1119421800.01	21.546	7.87	-1.445174	1.07E-107	5.08E-108	22.18	20.99	21.47	7.63	7.84	8.14
OsR498G1119422800.01	9.023	37.226	2.0443635	4.33E-213	1.23E-213	9.04	8.34	9.69	34.65	36.46	40.57
OsR498G1119425200.01	0.04	1.286	4.8813006	4.05E-29	4.14E-29	0.03	0.03	0.06	1.28	1.33	1.25
OsR498G1119432200.01	0.543	3.36	2.6297618	5.58E-20	6.70E-20	0.33	0.73	0.57	3.26	3.32	3.5
OsR498G1119433200.01	3.893	11.526	1.5641496	2.16E-46	1.73E-46	4.26	3.76	3.66	11.06	11.52	12

OsR498G1119433900.01	11.693	2.053	-2.534604	6.86E-189	2.15E-189	11.16	11.77	12.15	2.38	1.7	2.08
OsR498G1119434100.01	5.673	2.553	-1.153346	1.76E-18	2.17E-18	5.85	5.98	5.19	2.53	2.38	2.75
OsR498G1119435300.01	16.946	44.353	1.3900914	4.60E-94	2.41E-94	16.7	16.33	17.81	45.17	42.89	45
OsR498G1119435900.01	3.84	11.13	1.5361027	9.36E-55	6.82E-55	4.05	3.99	3.48	11.39	10.77	11.23
OsR498G1119437300.01	0.063	3.376	5.7753099	8.78E-106	4.24E-106	0.05	0.09	0.05	3.31	3.23	3.59
OsR498G1119438300.01	0.153	0.636	2.0385403	1.22E-06	2.15E-06	0.3	0.08	0.08	0.62	0.7	0.59
OsR498G1119440800.01	2.233	127.22	5.8319961	0	0	2.35	2.06	2.29	130.41	134.09	117.16
OsR498G1119444400.01	4.44	27.15	2.6099373	8.24E-242	2.11E-242	4.84	4.89	3.59	27.53	24.45	29.47
OsR498G1119445300.01	0.046	0.893	4.0618728	6.76E-09	1.08E-08	0	0.07	0.07	1.22	0.63	0.83
OsR498G1119446100.01	0.39	0.95	1.2810713	1.76E-09	2.76E-09	0.33	0.33	0.51	1.02	0.92	0.91
OsR498G1119449200.01	61.41	21.573	-1.424661	7.50E-166	2.63E-166	55.53	61.62	67.08	18.63	22.45	23.64
OsR498G1119450600.01	3.48	11.33	1.680035	8.06E-127	3.44E-127	4.13	3.35	2.96	11.8	11.67	10.52
OsR498G1119451500.01	13.243	49.13	1.9054224	0	0	12.86	13.33	13.54	48.6	50.64	48.15
OsR498G1119461400.01	9.196	29.896	1.8215943	0	0	9.21	9.83	8.55	28.94	31.57	29.18
OsR498G1119465400.01	1.006	3.776	1.9110053	4.18E-92	2.23E-92	1.07	0.73	1.22	3.93	3.91	3.49
OsR498G1119465500.01	0.516	0.08	-2.662956	6.15E-13	8.69E-13	0.45	0.58	0.52	0.04	0.1	0.1
OsR498G1119465700.01	10.83	31.226	1.5054655	0	0	10.35	11.26	10.88	31.67	31.54	30.47
OsR498G1119475600.01	0.066	0.28	2.0695672	7.56E-07	1.32E-06	0.06	0.05	0.09	0.21	0.39	0.24
OsR498G1119479100.01.	28.136	66.793	1.2494411	9.52E-242	2.43E-242	25.75	30.73	27.93	65.93	70.05	64.4
OsR498G1119485200.01	1.333	4.093	1.6026999	1.58E-40	1.37E-40	1.3	1.24	1.46	4.15	4.03	4.1
OsR498G1119486400.01	16694.5	5338.4	-1.633915	0	0	16679	16603	16802	5293.6	5367.7	5353.9
OsR498G1119486600.01	10.87	4.22	-1.36558	1.69E-80	9.81E-81	9.72	10.97	11.92	4.57	3.99	4.1
OsR498G1119487000.01	5.073	14.903	1.5558631	1.11E-151	4.14E-152	4.94	5.11	5.17	14.7	15.89	14.12
OsR498G1119488200.01	0.09	0.44	2.3947418	1.31E-13	1.82E-13	0.08	0.13	0.06	0.36	0.47	0.49
OsR498G1119489300.01	1.823	0.09	-4.383286	2.44E-79	1.43E-79	1.65	1.68	2.14	0.06	0.05	0.16
OsR498G1119489500.01	0.543	0.033	-3.661344	3.99E-19	4.87E-19	0.57	0.42	0.64	0.06	0.01	0.03
OsR498G1119492700.01	0.446	0.046	-3.213217	1.73E-16	2.24E-16	0.47	0.41	0.46	0.06	0.05	0.03
OsR498G1119497200.01	8.636	22.85	1.3747838	7.31E-194	2.25E-194	8.84	8.37	8.7	22.81	23.75	21.99
OsR498G1119500400.01	0.25	0.06	-2.02559	0.0002922	0.0005834	0.25	0.22	0.28	0.15	0	0.03
OsR498G1119504300.01	0.18	1.88	3.3838009	4.86E-33	4.66E-33	0.2	0.17	0.17	2.27	1.58	1.79
OsR498G1119507600.01	2.93	9.403	1.626617	6.32E-125	2.72E-125	3.46	2.63	2.7	9.4	9.43	9.38
OsR498G1119518700.01	1.44	0.206	-2.79818	1.16E-14	1.57E-14	1.8	1.26	1.26	0.2	0.25	0.17
OsR498G1119519800.01	14.283	31.703	1.1271032	9.41E-126	4.04E-126	13.94	15.39	13.52	33.78	31.38	29.95
OsR498G1119522900.01	42.98	205.62	2.2775094	0	0	43.61	42.62	42.71	204.64	206.66	205.57
OsR498G1119523700.01	0.973	6.22	2.6782779	1.65E-94	8.61E-95	0.89	1.28	0.75	6.24	6.44	5.98
OsR498G1119524400.01	0.333	2.426	2.8597674	9.45E-23	1.08E-22	0.28	0.28	0.44	2.88	2	2.4
OsR498G1119525200.01	0	0.353	5.2963381	2.20E-06	3.91E-06	0	0	0	0.16	0.42	0.48
OsR498G1119526200.01	0.01	6.236	9.1862983	4.76E-109	2.25E-109	0	0	0.03	5.94	5.85	6.92
OsR498G1119529900.01	0.836	5.446	2.7244317	3.06E-90	1.65E-90	0.86	0.7	0.95	5.7	5.16	5.48
OsR498G1119530100.01	0.666	4.063	2.6312384	4.09E-58	2.88E-58	0.76	0.49	0.75	3.65	4.2	4.34
OsR498G1119531400.01	27.296	95.16	1.8002132	0	0	26.98	28.4	26.51	94.55	93.14	97.79
OsR498G1119535000.01	2.593	7.163	1.4913772	4.01E-114	1.84E-114	2.38	2.43	2.97	7.42	7.25	6.82
OsR498G1119536300.01	0.523	2.13	2.0374198	1.13E-26	1.20E-26	0.73	0.34	0.5	1.95	2.4	2.04
OsR498G1119545100.01	6.363	13.566	1.0946342	7.57E-116	3.43E-116	6.1	6.89	6.1	13.58	13.94	13.18
OsR498G1119545300.01	169.13	73.1	-1.209777	0	0	171.69	170.64	165.06	75.12	74.04	70.14
OsR498G1119545900.01	0.093	0.303	1.7663728	0.0008592	0.0017798	0.08	0.12	0.08	0.34	0.41	0.16
OsR498G1119550500.01	3.87	11.656	1.589385	1.74E-27	1.83E-27	3.47	3.24	4.9	12.67	11.1	11.2
OsR498G1119553700.01	8.3	56.883	2.8189498	0	0	7.88	8.06	8.96	56.3	55.81	58.54
OsR498G1119554100.01	1.346	2.69	1.0014782	1.09E-11	1.60E-11	1.53	0.99	1.52	2.39	2.87	2.81
OsR498G1119556300.01	0.43	1.456	1.758624	1.25E-06	2.21E-06	0.67	0.42	0.2	1.13	1.66	1.58
OsR498G1119559200.01	0.833	2.683	1.6873406	8.20E-33	7.88E-33	0.74	0.91	0.85	2.69	2.72	2.64
OsR498G1119563500.01	0.4	1.126	1.4922044	1.78E-08	2.89E-08	0.43	0.42	0.35	1.08	1.03	1.27
OsR498G1119569000.01	15.516	38.676	1.3205304	1.20E-279	2.68E-280	15.81	16.61	14.13	38.31	37.82	39.9
OsR498G1119569800.01	1.756	4.206	1.2577168	2.40E-22	2.76E-22	1.8	1.68	1.79	4.24	4.06	4.32
OsR498G1119570600.01	0.473	1.876	2.2638198	6.64E-20	7.99E-20	0.49	0.59	0.34	1.59	2.13	1.91
OsR498G1119570700.01	0.026	0.406	3.8618569	2.95E-09	4.68E-09	0.04	0.04	0	0.54	0.37	0.31
OsR498G1119571600.01	0.133	0.603	2.1838633	4.98E-06	9.00E-06	0.25	0.05	0.1	0.34	0.92	0.55
OsR498G1119571800.01	0.106	0.723	2.8224069	5.48E-13	7.73E-13	0	0.1	0.22	0.88	0.67	0.62
OsR498G1119575200.01	5.683	0.476	-3.586487	3.45E-110	1.62E-110	5.29	6.26	5.5	0.57	0.43	0.43
OsR498G1119584300.01	37.153	17.45	-1.08542	5.26E-259	1.25E-259	41.14	33.36	36.96	17.49	16.48	18.38
OsR498G1119585000.01	262.87	86.81	-1.598507	0	0	265.28	263.83	259.5	90.68	85.37	84.38
OsR498G1119592200.01	0.143	1.52	3.3838009	1.95E-17	2.47E-17	0.05	0.11	0.27	0.96	2.07	1.53
OsR498G1119592400.01	0.356	9.5	4.6759234	4.15E-161	1.48E-161	0.34	0.44	0.29	9.87	8.56	10.07
OsR498G1119592500.01	0.256	7.603	4.8857202	1.68E-149	6.33E-150	0.15	0.36	0.26	7.22	8.2	7.39
OsR498G1119596600.01	0.066	0.423	2.8157122	1.95E-08	3.18E-08	0.1	0.03	0.07	0.59	0.41	0.27
OsR498G1119596800.01	0.03	0.16	2.5593725	0.0003356	0.0006725	0.03	0.03	0.03	0.24	0.05	0.19
OsR498G1119597400.01	0.07	0.646	2.97441	5.43E-07	9.41E-07	0	0.08	0.13	0.42	0.71	0.81
OsR498G1119598200.01	1.423	5.013	1.7786934	6.51E-84	3.69E-84	1.53	1.43	1.31	5.32	4.72	5
OsR498G1119599000.01	50.213	19.483	-1.385213	0	0	51.15	49.75	49.74	20.09	19.76	18.6

OsR498G1119600000.01	0.863	2.4	1.4865219	5.35E-19	6.54E-19	1.18	0.82	0.59	2.54	2.81	1.85
OsR498G1119601400.01	0.193	0.703	1.8869471	6.52E-08	1.09E-07	0.24	0.17	0.17	0.88	0.49	0.74
OsR498G1119602000.01	0.01	0.15	3.5593725	0.0005724	0.0011661	0	0.03	0	0.15	0.2	0.1
OsR498G1119605600.01	0.226	24.69	6.7669679	7.38E-178	2.44E-178	0.19	0.39	0.1	22.26	23.96	27.85
OsR498G1119605800.01	4.123	12.656	1.5802598	1.34E-74	8.11E-75	4.48	3.87	4.02	12.73	12.76	12.48
OsR498G1119607600.01	21.603	0.72	-4.894346	0	0	22.21	21	21.6	0.62	0.77	0.77
OsR498G1119608900.01	0.67	2.166	1.6938362	1.73E-07	2.94E-07	0.67	0.86	0.48	2.06	2.23	2.21
OsR498G1119614900.01	0.416	1.053	1.3389824	8.70E-08	1.46E-07	0.47	0.56	0.22	1.19	0.94	1.03
OsR498G1119615200.01	1.496	0.766	-1.076937	8.56E-08	1.43E-07	1.99	1.34	1.16	0.58	1.07	0.65
OsR498G1119615600.01	8.473	27.696	1.7356209	0	0	8.15	9.09	8.18	28.29	27.7	27.1
OsR498G1119615700.01	0.686	1.576	1.2082242	3.28E-07	5.63E-07	0	0.45	1.61	2.06	2.46	0.21
OsR498G1119616200.01	8.153	18.553	1.1758326	7.62E-67	4.93E-67	8.83	7.44	8.19	18.6	18.8	18.26
OsR498G1119617700.01	63.396	12.276	-2.371825	0	0	66.07	63.04	61.08	10.52	11.12	15.19
OsR498G1119617900.01	2.55	1.056	-1.273518	6.43E-06	1.17E-05	2.93	2.61	2.11	1.38	0.88	0.91
OsR498G1119619100.01	11.116	47.406	2.0901729	0	0	10.79	10.23	12.33	46.71	49.36	46.15
OsR498G1119619400.01	322.246	129.99	-1.312912	0	0	324.35	318.46	323.93	125.6	127.51	136.86
OsR498G1119624600.01	4.506	10.876	1.119993	4.29E-27	4.54E-27	5.06	3.87	4.59	11.18	11.1	10.35
OsR498G1119632800.01	0.603	0.046	-3.72603	5.42E-07	9.38E-07	0.7	0.62	0.49	0	0.14	0
OsR498G1119644200.01	3.86	19.753	2.3111039	0	0	4.01	3.9	3.67	20.14	20.39	18.73
OsR498G1119646100.01	1.076	3.416	1.6602029	6.00E-53	4.47E-53	1.04	1.12	1.07	3.64	3.55	3.06
OsR498G1119648000.01	0.953	2.693	1.5111173	9.95E-33	9.58E-33	0.88	1.05	0.93	2.34	2.95	2.79
OsR498G1119648200.01	1.936	4.956	1.3502769	1.02E-14	1.37E-14	1.47	1.92	2.42	5.56	4.17	5.14
OsR498G1119648600.01	4.12	16.233	1.9881109	3.66E-56	2.64E-56	4.54	4.61	3.21	16.29	18.39	14.02
OsR498G1119649900.01	1.736	19.823	3.5019701	0	0	1.92	1.86	1.43	19.94	20.82	18.71
OsR498G1119656400.01	10.963	2.956	-1.893187	1.40E-213	3.96E-214	10.89	9.55	12.45	2.63	2.95	3.29
OsR498G1119661800.01	0.563	1.453	1.3378316	4.00E-11	5.97E-11	0.35	0.64	0.7	1.61	1.24	1.51
OsR498G1119662400.01	1.623	0.32	-2.40256	1.37E-26	1.46E-26	1.51	1.66	1.7	0.3	0.31	0.35
OsR498G1119662800.01	1.076	0.383	-1.544964	7.06E-05	0.0001355	1.5	0.87	0.86	0.32	0.37	0.46
OsR498G1119667000.01	3.243	8.61	1.5302813	1.05E-85	5.85E-86	3.55	3.41	2.77	8.54	8.02	9.27
OsR498G1119667700.01	0.516	0.143	-1.863245	0.0004418	0.0008929	0.58	0.56	0.41	0.06	0.12	0.25
OsR498G1119688900.01	1.953	6.316	1.6998062	1.18E-87	6.49E-88	2.04	1.98	1.84	6.19	6.59	6.17
OsR498G1119689200.01	0.193	0.82	2.0813252	6.98E-08	1.16E-07	0.31	0.18	0.09	0.78	0.74	0.94
OsR498G1119689900.01	3657.36	853.53	-2.087174	0	0	3631.7	3561.1	3779.3	831.4	826	903.19
OsR498G1119698200.01	0.206	0.456	1.1287381	8.71E-05	0.0001683	0.18	0.16	0.28	0.43	0.49	0.45
OsR498G1119706100.01	0.456	1.773	1.9648872	4.08E-17	5.20E-17	0.5	0.36	0.51	1.8	1.67	1.85
OsR498G1119707600.01	2.09	0.663	-1.660306	1.87E-11	2.75E-11	1.82	2.54	1.91	0.73	0.67	0.59
OsR498G1119714500.01	3.773	35.82	3.1661643	0	0	3.91	3.83	3.58	37.04	36.22	34.2
OsR498G1119717400.01	3.716	1.41	-1.507123	2.27E-33	2.16E-33	3.96	3.81	3.38	1.09	1.16	1.98
OsR498G1119726600.01	0.633	2.146	1.7988384	1.98E-09	3.12E-09	0.46	0.65	0.79	2.73	1.77	1.94
OsR498G1119728500.01	2.536	20.273	2.94475	1.38E-219	3.79E-220	2.74	2.64	2.23	21.74	19.9	19.18
OsR498G1119733000.01	4.53	0.956	-1.404833	2.54E-62	1.72E-62	4.33	4.25	5.01	0.87	1.07	0.93
OsR498G1119741400.01	1.516	7.503	2.2744549	3.08E-113	1.43E-113	1.52	1.46	1.57	7.47	7.61	7.43
OsR498G1119747200.01	8.153	22.656	1.5606612	4.63E-166	1.62E-166	7.34	8.31	8.81	23.09	23.82	21.06
OsR498G1119761700.01	16.07	44.76	1.4236632	2.39E-198	7.20E-199	16.59	15.87	15.75	47.2	44.01	43.07
OsR498G1119767300.01	0.883	2.82	1.6956264	1.52E-49	1.17E-49	0.8	1.04	0.81	2.65	2.74	3.07
OsR498G1119767600.01	1.123	3.003	1.4400735	5.82E-17	7.45E-17	0.99	1.17	1.21	2.76	2.37	3.88
OsR498G1119797800.01	0.416	0.1	-2.08972	6.82E-07	1.19E-06	0.49	0.49	0.27	0.19	0.08	0.03
OsR498G1119802100.01	0.8	0.283	-1.51314	0.0002051	0.0004052	0.93	0.74	0.73	0.47	0.06	0.32
OsR498G1119803900.01	1.246	0.193	-2.679012	9.66E-11	1.46E-10	1.21	1.66	0.87	0.18	0.31	0.09
OsR498G1119804300.01	0.05	0.243	2.2881487	6.05E-05	0.0001155	0.11	0	0.04	0.15	0.23	0.35
OsR498G1119808900.01	1.62	0.206	-3.115609	4.11E-13	5.78E-13	0.55	1.72	2.59	0.28	0.18	0.16
OsR498G1119820100.01	1.52	0.026	-5.789356	1.49E-40	1.29E-40	1.57	1.18	1.81	0.05	0	0.03
OsR498G1119823100.01	5.486	0.06	-6.426469	1.15E-120	5.06E-121	5.37	5.56	5.53	0	0.15	0.03
OsR498G1119833300.01	0.146	0	-4.832945	6.10E-05	0.0001167	0.07	0.22	0.15	0	0	0
OsR498G1119848800.01	0.323	1.83	2.4864495	1.76E-43	1.46E-43	0.31	0.33	0.33	1.91	1.84	1.74
OsR498G1119848900.01	0.083	0.386	2.2200577	0.0006452	0.0013202	0	0.25	0	0.55	0.26	0.35
OsR498G1119858800.01	0.25	1.73	2.8589387	7.24E-31	7.20E-31	0.23	0.17	0.35	1.79	2.26	1.14
OsR498G1119862900.01	6.26	15.786	1.3336118	4.60E-62	3.12E-62	7.13	5.58	6.07	15.01	15.35	17
OsR498G1119863900.01	2.256	11.253	2.302319	1.40E-210	4.01E-211	2.48	1.95	2.34	11.81	10.66	11.29
OsR498G1119877600.01	0.136	0.9	2.7142581	1.50E-51	1.14E-51	0.12	0.19	0.1	0.97	0.98	0.75
OsR498G1119883200.01	0	0.873	5.2223375	3.89E-06	7.00E-06	0	0	0	1.1	0.82	0.7
OsR498G1119883300.01	0.2	9.2	5.5181242	0	0	0.14	0.25	0.21	9.14	10.08	8.38
OsR498G1119884600.01	4.993	17.41	1.8094718	2.61E-118	1.17E-118	5.66	4.93	4.39	16.8	16.67	18.76
OsR498G1119892200.01	4.846	1.99	-1.321255	2.84E-38	2.52E-38	4.34	5.22	4.98	2.37	2.19	1.41
OsR498G1119901000.01	0.156	0.956	2.8674948	5.37E-10	8.30E-10	0.23	0.17	0.07	1.56	0.54	0.77
OsR498G1119911900.01	0.563	1.7	1.6246639	1.34E-10	2.02E-10	0.64	0.5	0.55	1.88	1.46	1.76
OsR498G1119966200.01	0.733	0.03	-4.580179	1.92E-12	2.75E-12	0.94	0.56	0.7	0	0	0.09
OsR498G1119993800.01	73.043	35.803	-1.015851	8.65E-267	2.00E-267	73.35	75.33	70.45	36.01	36.73	34.67
OsR498G1120008500.01	1.47	0.14	-3.383142	1.88E-09	2.96E-09	1.62	0.86	1.93	0.21	0	0.21

OsR498G1120014500.01	308.39	116.72	-1.405312	0	0	314.58	310.99	299.6	116.44	116.57	117.14
OsR498G1120016500.01	0.08	0.516	2.7292975	6.89E-10	1.07E-09	0.09	0.12	0.03	0.51	0.57	0.47
OsR498G1120018700.01	0.453	1.5	1.7292975	1.31E-15	1.73E-15	0.5	0.43	0.43	1.51	1.51	1.48
OsR498G1120018900.01	11.063	58.066	2.3872986	7.62E-273	1.74E-273	11.74	9.73	11.72	56.85	55.07	62.28
OsR498G1120019200.01	2.35	9.91	2.0748701	2.98E-50	2.28E-50	2.11	2.57	2.37	9.34	11.01	9.38
OsR498G1120020500.01	0.54	0.066	-3.141067	2.67E-06	4.76E-06	0.67	0.6	0.35	0.13	0.07	0
OsR498G1120027100.01	0.51	0	-6.310992	5.36E-11	8.02E-11	0.56	0.46	0.51	0	0	0
OsR498G1120029500.01	2.52	5.826	1.2060357	3.52E-12	5.08E-12	2.22	2.88	2.46	4.48	6.11	6.89
OsR498G1120030500.01	2.146	0.986	-1.125126	3.02E-09	4.78E-09	1.81	2.56	2.07	1.29	0.57	1.1
OsR498G1120030900.01	0.51	1.816	1.832391	4.15E-07	7.16E-07	0.48	0.67	0.38	1.21	1.48	2.76
OsR498G1120034700.01	0.826	0.27	-1.703186	2.55E-05	4.76E-05	1.4	0.17	0.91	0.43	0.08	0.3
OsR498G1120037500.01	0.826	0.27	-1.703186	2.55E-05	4.76E-05	1.4	0.17	0.91	0.43	0.08	0.3
OsR498G1120041800.01	3.42	1.603	-1.00383	3.63E-14	4.97E-14	3.35	3.35	3.56	1.61	1.89	1.31
OsR498G1120044700.01	9.25	45.21	2.4033272	0	0	10.64	8.24	8.87	47.17	43.63	44.83
OsR498G1120050700.01	1.02	0.39	-1.383142	4.69E-12	6.80E-12	1.09	0.93	1.04	0.34	0.36	0.47
OsR498G1120056000.01	4.68	1.273	-1.720869	6.46E-21	7.63E-21	4.53	6	3.51	0.53	2.06	1.23
OsR498G1120056800.01	0.956	0.06	-4.02559	4.20E-05	7.94E-05	1.08	0.9	0.89	0	0	0.18
OsR498G1120058300.01	0.736	3.71	2.3210001	1.20E-38	1.06E-38	0.88	0.8	0.53	3.77	4.03	3.33
OsR498G1120059500.01	2.25	4.873	1.1160711	2.26E-38	2.01E-38	2.62	2.11	2.02	5.13	4.76	4.73
OsR498G1120060300.01	3.356	13.446	1.9987256	3.41E-150	1.28E-150	3.64	3.01	3.42	14.27	13.54	12.53
OsR498G1120078800.01	0.31	0.62	1.0061188	4.04E-05	7.63E-05	0.27	0.43	0.23	0.58	0.6	0.68
OsR498G1120079200.01	21.816	47.853	1.1325132	9.20E-232	2.43E-232	21.49	21.47	22.49	48.32	46	49.24
OsR498G1120082000.01	9.06	0.333	-4.814425	7.28E-91	3.91E-91	10.59	7.41	9.18	0.34	0.27	0.39
OsR498G1120082200.01	161.726	64.13	-1.337946	0	0	161.94	167.32	155.92	64.18	64.11	64.1
OsR498G1120111800.01	1.063	3.923	1.8813006	5.96E-09	9.55E-09	1.28	1.28	0.63	3.9	3.26	4.61
OsR498G1120115000.01	9.766	2.77	-1.815667	2.64E-109	1.24E-109	10.26	9.45	9.59	2.83	2.7	2.78
OsR498G1120121900.01	20.57	41.483	1.0113726	2.25E-134	9.20E-135	20.95	21.45	19.31	39.99	41.95	42.51
OsR498G1120125900.01	20.39	46.076	1.1793252	1.91E-136	7.71E-137	19.76	20.84	20.57	49.26	45.27	43.7
OsR498G1120143800.01	0.013	0.1	3.0396452	7.01E-05	0.0001345	0.02	0	0.02	0.17	0.11	0.02
OsR498G1120145400.01	0.08	0.006	-3.341631	8.32E-06	1.52E-05	0.08	0.12	0.04	0	0.02	0
OsR498G1120145600.01	20.286	55.31	1.4907203	0	0	20.71	20.34	19.81	57.91	55.7	52.32
OsR498G1120146800.01	19.096	63.66	1.7362994	2.66E-287	5.82E-288	17.41	19.22	20.66	63.5	64.65	62.83
OsR498G1120147800.01	0.153	1.27	3.2720905	2.43E-12	3.49E-12	0.23	0.1	0.13	0.95	1.69	1.17
OsR498G1120153700.01	5.79	0.43	-3.761717	1.03E-117	4.63E-118	5.95	5.49	5.93	0.62	0.21	0.46
OsR498G1120154200.01	0.32	1.016	1.6710178	2.07E-09	3.26E-09	0.5	0.36	0.1	1	1.12	0.93
OsR498G1120157800.01	0.24	0.053	-2.191441	0.0009217	0.0019152	0.21	0.35	0.16	0	0.16	0
OsR498G1120160300.01	14.553	6.763	-1.105932	6.14E-23	6.96E-23	15.62	13.16	14.88	6.66	7.08	6.55
OsR498G1120160600.01	0.613	1.55	1.3486054	1.35E-17	1.71E-17	0.68	0.59	0.57	1.46	1.74	1.45
OsR498G1120167900.01	0.996	12.563	3.6552475	4.29E-144	1.66E-144	1.16	0.68	1.15	12.37	11.25	14.07
OsR498G1120171200.01	10.636	44.94	2.0820642	0	0	10.91	11.27	9.73	45.6	46.01	43.21
OsR498G1120177600.01	6.943	2.246	-1.629833	2.78E-55	2.02E-55	7.24	6.39	7.2	2.18	2.34	2.22
OsR498G1120196500.01	0.9	2.57	1.5459519	1.52E-09	2.38E-09	0.76	1.09	0.85	2.82	2.8	2.09
OsR498G1120210200.01	1.01	8.566	3.0829344	2.49E-118	1.11E-118	1.22	0.83	0.98	8.57	8.09	9.04
OsR498G1120213700.01	0.726	3.48	2.2612911	1.37E-16	1.77E-16	0.44	1.04	0.7	2.81	4.58	3.05
OsR498G1120216000.01	0.833	0.29	-1.521586	3.58E-05	6.75E-05	0.68	0.99	0.83	0.36	0.2	0.31
OsR498G1120222300.01	12.466	46.93	1.9066729	0	0	12.88	13	11.52	45.61	47.95	47.23
OsR498G1120222500.01	6.76	15.456	1.1915081	2.61E-28	2.70E-28	5.92	7.18	7.18	16.43	15.27	14.67
OsR498G1120235000.01	22.176	141.29	2.6673204	0	0	24.01	20.95	21.57	141.3	135.9	146.68
OsR498G1120235500.01	0.183	0.013	-3.610553	0.0004877	0.0009887	0.23	0.18	0.14	0.04	0	0
OsR498G1120235900.01	2.203	15.143	2.7500734	2.90E-273	6.62E-274	2.37	2.09	2.15	14.3	15.52	15.61
OsR498G1120277500.01	0.886	2.193	1.3894475	1.05E-09	1.65E-09	0.51	1.27	0.88	1.72	2.3	2.56
OsR498G1120278000.01	0.75	3.51	2.2714634	1.10E-71	6.83E-72	0.99	0.52	0.74	3.94	3.56	3.03
OsR498G1120278400.01	0.133	1.493	3.4853719	1.56E-12	2.23E-12	0.32	0.08	0	1.64	1.16	1.68
OsR498G1120279100.01	0.02	0.546	4.7292975	7.57E-08	1.26E-07	0	0	0.06	0.36	1.03	0.25
OsR498G1120279400.01	23.49	5.91	-1.989906	1.54E-306	3.20E-307	23.06	23.15	24.26	6.07	5.81	5.85
OsR498G1120285000.01	0.896	10.476	3.5411417	3.45E-166	1.21E-166	1	0.62	1.07	9.4	10.29	11.74
OsR498G1120287000.01	1.963	9.276	2.2366668	1.91E-134	7.83E-135	2.16	1.76	1.97	9.15	8.97	9.71
OsR498G1120287900.01	7.596	22.3	1.5532464	1.59E-104	7.76E-105	8.01	7.88	6.9	21.81	23.12	21.97
OsR498G1120292700.01	4.313	8.823	1.0240851	5.79E-39	5.11E-39	3.87	4.82	4.25	8.15	9.31	9.01
OsR498G1120293500.01	0.34	0	-5.417907	9.99E-07	1.75E-06	0.29	0.44	0.29	0	0	0
OsR498G1120295700.01	28.146	7.39	-2.005412	9.94E-302	2.09E-302	27.73	28	28.71	7.22	7.67	7.28
OsR498G1120296600.01	11.87	2.336	-2.51307	1.34E-236	3.49E-237	12.59	11.54	11.48	2.43	2.3	2.28
OsR498G1120297100.01	2.216	6.713	1.5984723	1.78E-44	1.45E-44	2.13	1.89	2.63	6.59	6.7	6.85
OsR498G1120297300.01	1.65	4.176	1.33698	3.03E-11	4.50E-11	1.58	1.8	1.57	3.84	4.44	4.25
OsR498G1120297500.01	0.213	0.016	-2.832945	0.0006839	0.0014027	0.13	0.04	0.47	0	0	0.05
OsR498G1120311700.01	4.283	9.013	1.3053677	1.42E-57	1.01E-57	4.42	4.31	4.12	8.82	9.55	8.67
OsR498G1120313000.01	0.54	2.486	2.2288312	1.63E-41	1.39E-41	0.69	0.49	0.44	2.56	2.32	2.58
OsR498G1120315000.01	4.143	14.263	1.7836545	2.73E-137	1.09E-137	4.55	4.39	3.49	14.17	13.85	14.77
OsR498G1120315800.01	6.88	15.463	1.1345152	7.27E-58	5.15E-58	6.71	7.61	6.32	16.24	15.37	14.78

OsR498G1120316900.01	5.006	11.21	1.1497282	1.48E-29	1.50E-29	5.23	5.44	4.35	11.66	12.44	9.53
OsR498G1120320200.01	15.356	34.716	1.0899714	1.83E-100	9.21E-101	16.29	15.63	14.15	36.02	33.89	34.24
OsR498G1120321000.01	3.293	18.316	2.468607	2.46E-218	6.80E-219	3.7	2.91	3.27	18.01	19.38	17.56
OsR498G1120321700.01	14.073	30.116	1.1060068	3.86E-153	1.43E-153	13.8	14.53	13.89	29.97	30.37	30.01
OsR498G1120322200.01	13.046	29.246	1.1715564	4.22E-108	2.01E-108	12.62	13.22	13.3	28.68	31.03	28.03
OsR498G1120324400.01	0.073	0.776	3.506905	3.89E-17	4.96E-17	0.06	0.06	0.1	0.84	0.59	0.9
OsR498G1120327500.01	2.9	6.483	1.1632668	2.27E-32	2.20E-32	2.94	2.81	2.95	6.76	7.05	5.64
OsR498G1120327700.01	0.043	0.256	2.5593725	4.55E-05	8.61E-05	0.09	0.04	0	0.27	0.22	0.28
OsR498G1120330300.01	0.43	1.633	1.6171609	6.05E-15	8.12E-15	0.42	0.38	0.49	1.61	1.55	1.74
OsR498G1120332600.01	2.94	6.753	1.2016154	5.29E-61	3.63E-61	3.15	2.89	2.78	6.86	6.8	6.6
OsR498G1120333200.01	1.53	4.016	1.4447299	1.62E-10	2.46E-10	1.58	1.76	1.25	2.96	4.66	4.43
OsR498G1120333600.01	14.633	35.396	1.2701065	2.01E-60	1.38E-60	13.51	13.62	16.77	33.14	30.79	42.26
OsR498G1120337200.01	1.476	0.533	-1.461689	2.59E-14	3.53E-14	1.53	1.65	1.25	0.73	0.51	0.36
OsR498G1120340200.01	0.306	0.706	1.5921624	1.63E-08	2.66E-08	0.44	0.24	0.24	0.66	0.68	0.78
OsR498G1120348100.01	4.066	0.273	-3.883571	7.56E-32	7.40E-32	3.55	3.78	4.87	0.49	0.16	0.17
OsR498G1120348600.01	0.766	1.993	1.3842858	3.03E-13	4.24E-13	0.86	0.72	0.72	2.01	2.1	1.87
OsR498G1120348700.01	0.013	0.196	3.6182661	1.37E-06	2.41E-06	0.02	0.02	0	0.16	0.14	0.29
OsR498G1120349100.01	0.416	0.966	1.1912213	6.38E-06	1.16E-05	0.54	0.41	0.3	0.68	0.77	1.45
OsR498G1120350200.01	1.103	0.186	-2.575787	1.44E-13	2.00E-13	1.1	0.96	1.25	0.2	0.16	0.2
OsR498G1120350400.01	15.346	6.923	-1.144803	1.08E-55	7.83E-56	14.75	15.73	15.56	7.16	7.03	6.58
OsR498G1120351600.01	0.36	0.166	-1.144889	0.0001249	0.0002437	0.33	0.39	0.36	0.29	0.05	0.16
OsR498G1120352700.01	0.056	0.22	2.0524125	8.30E-06	1.52E-05	0.04	0.09	0.04	0.19	0.26	0.21
OsR498G1120367700.01	1.543	0.45	-1.778152	5.59E-13	7.88E-13	1.47	1.52	1.64	0.47	0.39	0.49
OsR498G1120368900.01	2.566	0.613	-2.069156	1.96E-10	2.99E-10	2.29	2.86	2.55	0.86	0.29	0.69
OsR498G1120370300.01	0.27	0.103	-1.379227	0.0001468	0.0002875	0.2	0.26	0.35	0.12	0.12	0.07
OsR498G1120370800.01	23.513	50.343	1.0931475	1.81E-170	6.15E-171	24.27	23.23	23.04	52.24	49.37	49.42
OsR498G1120371700.01	2.44	21.996	3.1703302	3.68E-89	2.00E-89	2.54	2.13	2.65	20.17	22.94	22.88
OsR498G1120371900.01	1.77	14.596	3.0418729	1.47E-67	9.42E-68	1.93	1.58	1.8	13.79	13.4	16.6
OsR498G1120379500.01	5.963	18.66	1.6442614	2.72E-68	1.74E-68	5.38	6.21	6.3	17.06	19.43	19.49
OsR498G1120379700.01	1.82	6.05	1.756783	3.74E-44	3.07E-44	2.21	1.79	1.46	5.47	6.36	6.32
OsR498G1120381200.01	1.223	0.356	-1.779572	9.05E-20	1.09E-19	1.17	1.08	1.42	0.38	0.36	0.33
OsR498G1120383600.01	0.36	0.863	1.2899118	6.00E-08	9.98E-08	0.23	0.45	0.4	0.76	1.1	0.73
OsR498G1120384800.01	0.81	2.653	1.7824647	9.17E-35	8.57E-35	1.01	0.62	0.8	2.69	3.28	1.99
OsR498G1120391500.01	6.04	12.45	1.0407123	5.95E-101	2.97E-101	5.89	6.05	6.18	12.69	12.66	12
OsR498G1120393600.01	0.236	0.506	1.0868847	4.50E-05	8.52E-05	0.23	0.27	0.21	0.64	0.43	0.45
OsR498G1120394400.01	0.28	0.636	1.1968024	8.55E-05	0.0001651	0.37	0.25	0.22	0.75	0.6	0.56
OsR498G1120394800.01	0.163	0.43	1.4032533	3.92E-06	7.04E-06	0.13	0.2	0.16	0.44	0.46	0.39
OsR498G1120403400.01	1.933	4.666	1.2707844	5.52E-67	3.57E-67	1.65	1.77	2.38	4.47	5.06	4.47
OsR498G1120405900.01	0.37	2.096	2.5163037	2.11E-15	2.80E-15	0.13	0.52	0.46	2.02	2.09	2.18
OsR498G1120411100.01	0.81	1.963	1.2720905	3.84E-08	6.34E-08	0.56	1.11	0.76	1.78	1.67	2.44
OsR498G1120411600.01	22.73	81.413	1.8397087	0	0	24.64	22.41	21.14	79.39	80.17	84.68
OsR498G1120413700.01	0.116	0.513	2.144335	5.56E-11	8.33E-11	0.11	0.16	0.08	0.51	0.39	0.64
OsR498G1120417100.01	3.036	7.316	1.2838741	4.59E-27	4.86E-27	2.58	2.74	3.79	8.08	6.85	7.02
OsR498G1120418500.01	5.72	12.306	1.1027641	1.26E-97	6.44E-98	5.47	6.46	5.23	13.11	12.09	11.72
OsR498G1120421300.01	22.663	47.4	1.0654993	9.68E-89	5.29E-89	22.62	23.13	22.24	44.61	47.32	50.27
OsR498G1120425000.01	0	0.316	7.3494494	5.07E-20	6.08E-20	0	0	0	0.41	0.19	0.35
OsR498G1120427500.01	0.163	0.02	-3.195515	6.51E-05	0.0001248	0.08	0.11	0.3	0.03	0.03	0
OsR498G1120432000.01	0.853	0.416	-1.02559	6.74E-05	0.0001293	0.91	0.59	1.06	0.46	0.42	0.37
OsR498G1120433900.01	0.966	0.18	-2.417907	2.29E-07	3.90E-07	0.9	0.69	1.31	0.4	0.07	0.07
OsR498G1120436500.01	0.43	0	-6.451855	6.73E-12	9.80E-12	0.3	0.42	0.57	0	0	0
OsR498G1120440500.01	0.01	1	6.6326214	6.05E-25	6.63E-25	0.03	0	0	1.03	0.97	1
OsR498G1120441100.01	1.896	6.68	1.8128998	1.08E-67	6.91E-68	2.27	1.47	1.95	6.68	6.72	6.64
OsR498G1120444000.01	0.013	0.316	4.4338416	1.43E-06	2.52E-06	0	0	0.04	0.43	0.3	0.22
OsR498G1120446600.01	0.046	0.4	3.2070707	4.88E-10	7.53E-10	0.08	0.03	0.03	0.23	0.48	0.49
OsR498G1120452900.01	0.246	2.126	3.0972667	2.43E-68	1.55E-68	0.3	0.31	0.13	2.33	1.92	2.13
OsR498G1120454800.01	48.096	153.75	1.6755096	0	0	49.89	45.97	48.43	152.37	146.16	162.71
OsR498G1120466600.01	0.166	7.366	5.4662631	1.03E-43	8.55E-44	0.13	0.12	0.25	6.83	7.66	7.61
OsR498G1120466900.01	19.073	62.113	1.7121089	0	0	18.77	20.1	18.35	61.25	60.16	64.93
OsR498G1120467700.01	11.803	59.866	2.3224862	0	0	10.95	12.62	11.84	60.66	56.44	62.5
OsR498G1120471000.01	26.026	59.393	1.1898848	1.69E-250	4.16E-251	26.5	25.61	25.97	58.9	59.9	59.38
OsR498G1120474300.01	0.9	2.103	1.2374444	3.21E-14	4.38E-14	0.77	0.8	1.13	1.84	2.29	2.18
OsR498G1120474500.01	21.806	108.75	2.3164243	0	0	22.48	22.47	20.47	108.52	108.86	108.88
OsR498G1120475100.01	18.803	80.23	2.092259	0	0	19.91	19.09	17.41	78.78	79.72	82.19
OsR498G1120482500.01	16.07	41.333	1.3717019	2.95E-197	8.95E-198	16.94	16.1	15.17	41.63	41.64	40.73
OsR498G1120483200.01	2.68	10.186	1.9208517	1.91E-131	7.95E-132	2.62	2.56	2.86	10.65	10.15	9.76
OsR498G1120483500.01	0.023	1.483	5.8792038	3.81E-69	2.41E-69	0.05	0.02	0	1.37	1.51	1.57
OsR498G1120484000.01	0.383	0.16	-1.247982	0.0001094	0.0002125	0.23	0.53	0.39	0.16	0.2	0.12
OsR498G1120484100.01	0.306	0.06	-2.065118	8.38E-06	1.53E-05	0.3	0.38	0.24	0.04	0.1	0.04
OsR498G1120486300.01	0.87	0.416	-1.03819	2.63E-06	4.68E-06	0.9	0.86	0.85	0.39	0.28	0.58

OsR498G1120487000.01	40.51	129.35	1.6599687	0	0	41.98	40.37	39.18	129.96	131.64	126.45
OsR498G1120488400.01	17.203	54.05	1.651756	0	0	16.52	17.86	17.23	53.62	56.77	51.76
OsR498G1120488700.01	19.31	61.21	1.6444397	0	0	19.63	19.39	18.91	61.88	63.22	58.53
OsR498G1120491200.01	6.01	17.25	1.5129674	7.16E-107	3.43E-107	5.62	6.26	6.15	16.85	17.94	16.96
OsR498G1120505100.01	20.86	9.64	-1.127329	1.15E-69	7.25E-70	20.83	20.75	21	8.89	9.97	10.06
OsR498G1120508800.01	22.23	48.196	1.1282855	3.95E-139	1.57E-139	22.87	21.88	21.94	45.27	51.51	47.81
OsR498G1120511100.01	0.68	2.92	2.0999408	2.09E-12	3.00E-12	0.56	1.02	0.46	1.99	2.88	3.89
OsR498G1120525600.01	0.863	3.58	2.0678368	1.32E-34	1.24E-34	0.92	0.6	1.07	3.06	3.8	3.88
OsR498G1120526500.01	42.976	20.54	-1.08899	2.39E-109	1.13E-109	40.79	43.2	44.94	19.93	19.77	21.92
OsR498G1120557200.01	0.12	0.01	-3.273518	3.60E-05	6.79E-05	0.15	0.12	0.09	0	0	0.03
OsR498G1120561800.01	0.43	1.206	1.4769103	0.0005606	0.0011403	0.54	0.32	0.43	0.95	1.05	1.62
OsR498G1120562000.01	25.046	4.806	-2.383363	1.61E-108	7.63E-109	27.96	22.16	25.02	4.95	4.03	5.44
OsR498G1120562200.01	26.17	55.273	1.0771985	8.83E-154	3.26E-154	27.63	25.26	25.62	54.73	56.47	54.62
OsR498G1120563300.01	13.22	47.453	1.7401181	0	0	12.13	13.35	14.18	47.54	50.35	44.47
OsR498G1120566500.01	7.59	16.48	1.119264	1.82E-138	7.28E-139	7.69	7.55	7.53	16.36	17.27	15.81
OsR498G1120574700.01	1.823	0.586	-1.685515	2.23E-08	3.64E-08	2.15	1.67	1.65	0.4	0.67	0.69
OsR498G1120576800.01	4.053	2.023	-1.104817	2.57E-13	3.59E-13	4.06	3.66	4.44	1.71	2.57	1.79
OsR498G1120580000.01	0.146	0.696	2.2223375	0.0006354	0.0012994	0.22	0.22	0	0.66	0.98	0.45
OsR498G1120584100.01	0.673	1.436	1.0880667	2.56E-07	4.38E-07	0.57	0.79	0.66	1.59	1.16	1.56
OsR498G1120587100.01	6.453	24.47	1.9332219	6.24E-118	2.79E-118	5.8	6.16	7.4	23.42	23.43	26.56
OsR498G1120591500.01	6.18	0.413	-3.907149	5.77E-115	2.63E-115	6.81	5.51	6.22	0.3	0.46	0.48
OsR498G1120605300.01	5.05	17.48	1.734266	4.19E-166	1.46E-166	5.2	4.99	4.96	16.69	18.24	17.51
OsR498G1120617700.01	0.02	2.01	6.857053	3.59E-55	2.61E-55	0.03	0	0.03	2.08	1.74	2.21
OsR498G1120626700.01	3.883	9.546	1.3758514	1.17E-106	5.64E-107	4.16	3.76	3.73	8.55	10.69	9.4
OsR498G1120631700.01	0.383	0	-5.417907	9.99E-07	1.75E-06	0.33	0.49	0.33	0	0	0
OsR498G1120635000.01	18.526	48.873	1.4157063	0	0	18.32	19.08	18.18	49.11	49.77	47.74
OsR498G1120641000.01	8.923	26.18	1.5505486	6.81E-144	2.64E-144	8.78	8.46	9.53	25.27	27.36	25.91
OsR498G1120642100.01	22.21	45.213	1.0251174	2.59E-137	1.04E-137	20.22	23.52	22.89	45.4	46.56	43.68
OsR498G1120644200.01	2.556	5.633	1.1685385	4.54E-28	4.73E-28	2.26	3.01	2.4	5.31	5.66	5.93
OsR498G1120646200.01	2.99	1.546	-1.02559	2.76E-07	4.73E-07	3.24	2.77	2.96	1.47	1.84	1.33
OsR498G1120663600.01	1.123	2.476	1.1358734	1.46E-05	2.70E-05	1.25	1.06	1.06	3.04	1.81	2.58
OsR498G1120669100.01	0.31	0.623	1.0083573	6.64E-05	0.0001274	0.2	0.44	0.29	0.71	0.41	0.75
OsR498G1120669300.01	10.006	22.336	1.1688048	1.34E-79	7.81E-80	9.78	10.4	9.84	22.63	22.3	22.08
OsR498G1120671100.01	21.126	45.163	1.1040975	2.50E-174	8.36E-175	22.21	21.1	20.07	44.59	45.75	45.15
OsR498G1120671500.01	1.46	6.536	2.1829966	6.20E-26	6.68E-26	1.19	1.58	1.61	6.29	6.81	6.51
OsR498G1120680000.01	1.926	10.02	2.3739066	1.25E-143	4.84E-144	1.77	1.91	2.1	10.09	10.47	9.5
OsR498G1120681300.01	0.023	0.39	3.6748497	5.45E-12	7.90E-12	0.02	0.05	0	0.34	0.55	0.28
OsR498G1120682100.01	0.65	1.693	1.3830066	1.00E-22	1.14E-22	0.53	0.6	0.82	1.73	1.98	1.37
OsR498G1120682400.01	7.893	22.17	1.4851069	1.04E-144	4.00E-145	7.86	7.83	7.99	22.67	23.05	20.79
OsR498G1120683400.01	40.013	119.19	1.6236393	0	0	40.2	40.68	39.16	120.78	119.25	117.53
OsR498G1120685800.01	790.64	276.58	-1.518408	0	0	768.41	825.02	778.49	277.93	276.17	275.64
OsR498G1120688900.01	0.303	0.816	1.4250714	5.03E-07	8.69E-07	0.15	0.27	0.49	0.98	0.83	0.64
OsR498G1120689300.01	0.296	1.023	1.7318397	7.95E-08	1.33E-07	0.41	0.16	0.32	0.67	1.21	1.19
OsR498G1120691200.01	2.393	28.75	3.5867729	0	0	2.13	2.53	2.52	30.15	26.72	29.38
OsR498G1120691300.01	0.053	1.87	5.0618728	1.89E-17	2.40E-17	0.08	0	0.08	1.47	2.04	2.1
OsR498G1120693800.01	2.086	4.94	1.2420316	4.26E-41	3.65E-41	1.99	2.04	2.23	4.76	4.76	5.3
OsR498G1120695800.01	0	0.15	4.5593725	0.0002368	0.0004696	0	0	0	0.18	0.19	0.08
OsR498G1120696900.01	0.1	0.7	2.832391	7.77E-11	1.17E-10	0.14	0	0.16	0.71	0.65	0.74
OsR498G1120697100.01	0.056	0.69	3.8157122	1.92E-10	2.92E-10	0.05	0.05	0.07	0.7	0.92	0.45
OsR498G1120697500.01	0.04	0.25	2.7292975	8.81E-06	1.61E-05	0.03	0	0.09	0.22	0.3	0.23
OsR498G1120697700.01	0	0.313	5.2963381	2.20E-06	3.91E-06	0	0	0	0.23	0.28	0.43
OsR498G1120697900.01	0.2	1.493	3.0385403	1.57E-32	1.51E-32	0.21	0.2	0.19	1.25	1.54	1.69
OsR498G1120698300.01	0.633	1.883	1.48005	1.85E-17	2.35E-17	0.52	0.5	0.88	2	1.96	1.69
OsR498G1120698700.01	0.336	1.29	1.9072958	4.42E-14	6.06E-14	0.32	0.35	0.34	1.47	1.37	1.03
OsR498G1120700800.01	9.853	2.406	-2.081085	7.18E-49	5.59E-49	9.9	8.86	10.8	2.98	1.92	2.32
OsR498G1120703700.01	0.163	0.443	1.7817649	4.35E-06	7.84E-06	0.22	0.2	0.07	0.4	0.37	0.56
OsR498G1120704100.01	7.82	22.8	1.7663596	9.24E-165	3.26E-165	7.12	7.47	8.87	22.42	21.56	24.42
OsR498G1120704400.01	0.1	0.233	1.2639166	0.0005001	0.0010148	0.1	0.08	0.12	0.3	0.19	0.21
OsR498G1120707000.01	0.13	0.39	1.5324054	1.61E-05	2.99E-05	0.13	0.09	0.17	0.36	0.3	0.51
OsR498G1120707400.01	11.646	24.48	1.0717349	1.71E-51	1.30E-51	11.23	11.38	12.33	24.01	25.01	24.42
OsR498G1120710100.01	0.98	2.613	1.5720783	7.81E-89	4.26E-89	0.99	0.99	0.96	2.88	2.46	2.5
OsR498G1120714500.01	1.563	0.61	-1.3555	4.15E-15	5.55E-15	1.52	1.75	1.42	0.8	0.6	0.43
OsR498G1120723800.01	0.206	1.09	2.9827184	8.26E-14	1.14E-13	0.19	0.16	0.27	0.61	1.74	0.92
OsR498G1120726800.01	0.536	0.03	-4.273518	2.64E-14	3.61E-14	0.6	0.61	0.4	0.04	0.05	0
OsR498G1120729800.01	105.95	31.153	-1.808639	0	0	104.39	107.22	106.24	31.05	31.59	30.82
OsR498G1120730600.01	0.746	3.183	2.0822132	4.03E-29	4.11E-29	0.89	0.77	0.58	3.38	3.12	3.05
OsR498G1120733900.01	1.81	0.64	-1.496289	1.03E-28	1.06E-28	2.08	1.8	1.55	0.57	0.78	0.57
OsR498G1120740500.01	7.433	3.306	-1.136696	3.72E-37	3.36E-37	7.85	7.42	7.03	3.6	3.51	2.81
OsR498G1120741700.01	4.98	19.38	1.9481784	4.43E-263	1.04E-263	5.26	4.86	4.82	20.06	19.03	19.05

OsR498G1120745500.01	1.146	0.48	-1.259712	3.50E-15	4.67E-15	1.22	1.05	1.17	0.38	0.46	0.6
OsR498G1120747000.01	0.616	0.003	-7.699194	1.94E-12	2.78E-12	0.47	0.52	0.86	0.01	0	0
OsR498G1120747200.01	0.97	0.01	-6.669446	1.00E-42	8.37E-43	1.09	0.91	0.91	0.03	0	0
OsR498G1120747600.01	0.883	0.053	-4.323271	7.88E-28	8.24E-28	1.16	0.6	0.89	0.02	0.05	0.09
OsR498G1120756400.01	0.13	0.03	-2.227224	0.000178	0.0003504	0.15	0.12	0.12	0.05	0.02	0.02
OsR498G1120759300.01	2.3	0.836	-1.560104	7.72E-23	8.77E-23	2.35	2.5	2.05	0.9	1.11	0.5
OsR498G1120760100.01	6.22	24.763	1.9833152	0	0	6.35	6.1	6.21	24.51	24.55	25.23
OsR498G1120761400.01	0	0.443	5.2223375	3.89E-06	7.00E-06	0	0	0	0.21	0.76	0.36
OsR498G1120762900.01	0.096	0.796	3.0787466	4.73E-09	7.54E-09	0.06	0.06	0.17	0.72	0.99	0.68
OsR498G1120764000.01	0.016	0.666	5.3667274	1.45E-11	2.14E-11	0.05	0	0	0.61	0.66	0.73
OsR498G1120764600.01	0.036	0.963	4.7817649	4.23E-08	6.99E-08	0.11	0	0	1.23	0.61	1.05
OsR498G1120767700.01	534.82	238.74	-1.166292	0	0	528.6	539.61	536.25	238.3	242.56	235.36
OsR498G1120768100.01	0.44	6.633	3.9093149	6.92E-55	5.04E-55	0.39	0.62	0.31	7	6.74	6.16
OsR498G1120769100.01	0.28	1.456	2.3894475	1.92E-20	2.29E-20	0.22	0.31	0.31	1.41	1.23	1.73
OsR498G1120769500.01	221.363	1515.2	2.7734796	0	0	216.55	230.31	217.23	1483.6	1487.3	1574.9
OsR498G1120770000.01	4.693	11.456	1.2832622	7.43E-38	6.64E-38	4.49	4.68	4.91	12.49	10.63	11.25
OsR498G1120770600.01	0.11	2.423	4.4411096	1.98E-45	1.60E-45	0.22	0.07	0.04	2.06	2.2	3.01
OsR498G1120771100.01	0.903	6.453	2.8381197	9.68E-120	4.28E-120	0.76	0.93	1.02	6.29	6.57	6.5
OsR498G1120772300.01	0.223	0.056	-1.964189	0.0004779	0.0009679	0.21	0.14	0.32	0.06	0.11	0
OsR498G1120774600.01	37818.9	13400	-1.498205	0	0	37282	38011	38164	13238	13385	13577
OsR498G1120775000.01	6.11	0.95	-2.680093	1.17E-52	8.77E-53	6.45	5.69	6.19	0.79	1.36	0.7
OsR498G1120776600.01	77.016	214.49	1.5012872	0	0	77.67	77.99	75.39	214.26	208.06	221.16
OsR498G1120778500.01	10.51	4.793	-1.132361	4.96E-37	4.49E-37	10.78	10.74	10.01	4.99	4.96	4.43
OsR498G1220783400.01	5.436	12.45	1.1929085	6.72E-38	6.00E-38	5.16	4.9	6.25	13.11	10.61	13.63
OsR498G1220787900.01	10.473	24.363	1.1592782	8.51E-113	3.94E-113	9.5	10.7	11.22	24.08	24.23	24.78
OsR498G1220789200.01	19.746	53.306	1.3860681	0	0	20.91	19.92	18.41	54.67	53.63	51.62
OsR498G1220794300.01	23.263	9.766	-1.248341	3.23E-259	7.68E-260	23.31	23.27	23.21	10.55	9.45	9.3
OsR498G1220797200.01	1.246	7.076	2.4927979	2.89E-51	2.20E-51	1.41	1.5	0.83	6.23	7.34	7.66
OsR498G1220798000.01	3.173	11.83	1.8976528	1.76E-72	1.08E-72	3.52	2.55	3.45	12.75	11.02	11.72
OsR498G1220798200.01	1.303	38.35	4.8630929	0	0	1.57	1.13	1.21	38.54	37.59	38.92
OsR498G1220799800.01	85.56	1099.8	3.6744095	0	0	83.91	84.77	88	1112	1081.9	1105.5
OsR498G1220805900.01	0.03	0.193	2.7790143	6.53E-05	0.0001252	0.03	0.06	0	0.14	0.23	0.21
OsR498G1220808500.01	0.44	0.086	-2.52809	1.60E-06	2.83E-06	0.49	0.31	0.52	0.07	0.09	0.1
OsR498G1220812100.01	0.9	1.993	1.139996	2.56E-09	4.04E-09	0.88	0.91	0.91	1.53	1.92	2.53
OsR498G1220813400.01	0.053	0.5	3.2474285	4.29E-12	6.21E-12	0.03	0.05	0.08	0.48	0.4	0.62
OsR498G1220815000.01	2.323	5.643	1.2801412	8.52E-45	6.94E-45	2.44	2.31	2.22	6	5.13	5.8
OsR498G1220816200.01	0.05	0.316	2.637375	0.0001939	0.0003825	0	0.1	0.05	0.2	0.55	0.2
OsR498G1220816700.01	1.963	4.036	1.0288577	4.82E-09	7.70E-09	1.44	2.58	1.87	3.61	3.54	4.96
OsR498G1220818100.01	0.93	0.15	-2.634399	9.21E-11	1.39E-10	1.24	0.82	0.73	0.27	0.13	0.05
OsR498G1220819900.01	36.31	83.033	1.1915259	9.90E-170	3.39E-170	35.26	37.61	36.06	81.66	79.54	87.9
OsR498G1220827000.01	7.346	1.67	-2.14346	2.31E-101	1.15E-101	5.81	7.1	9.13	1.44	1.84	1.73
OsR498G1220833500.01	15.456	33.556	1.120952	8.58E-184	2.75E-184	15.59	14.95	15.83	33.56	34.11	33
OsR498G1220834300.01	1.423	0.146	-3.244759	1.06E-28	1.09E-28	1.15	1.45	1.67	0.17	0.15	0.12
OsR498G1220834900.01	0.51	0.243	-1.089586	0.0003386	0.0006786	0.47	0.55	0.51	0.26	0.18	0.29
OsR498G1220836000.01	0.053	0.23	2.0789854	0.0002536	0.0005038	0.03	0.13	0	0	0.69	0
OsR498G1220836100.01	0.41	1.36	1.7425943	3.52E-11	5.24E-11	0.31	0.46	0.46	1.71	1.26	1.11
OsR498G1220836800.01	0.1	0.836	3.3042511	5.03E-09	8.03E-09	0.08	0.07	0.15	0.86	1	0.65
OsR498G1220841100.01	7.85	65.11	3.0436877	0	0	7.23	7.83	8.49	63.94	66.04	65.35
OsR498G1220843100.01	27.756	146.14	2.3712211	0	0	28.84	27.62	26.81	144.49	151.5	142.44
OsR498G1220843600.01	407.343	38.84	-3.400522	0	0	407.01	411.94	403.08	38.93	36.87	40.72
OsR498G1220844800.01	158.22	14.24	-3.476295	0	0	157.05	149.58	168.03	13.58	13.36	15.78
OsR498G1220844900.01	0.703	0.166	-2.095979	1.87E-06	3.31E-06	0.81	0.55	0.75	0.15	0.1	0.25
OsR498G1220845200.01	385.396	124.67	-1.630385	0	0	357.29	403.39	395.51	126.38	114.5	133.14
OsR498G1220845600.01	1.266	0.076	-4.086406	1.17E-14	1.58E-14	1.22	0.6	1.98	0.1	0	0.13
OsR498G1220847000.01	0.17	0.6	1.861796	1.45E-11	2.13E-11	0.26	0.14	0.11	0.56	0.71	0.53
OsR498G1220848300.01	0.073	0	-4.485022	0.0003787	0.0007614	0.04	0.08	0.1	0	0	0
OsR498G1220854200.01	1.42	0.296	-2.296057	1.81E-27	1.90E-27	1.29	1.65	1.32	0.37	0.24	0.28
OsR498G1220856100.01	3.91	0.92	-1.987271	3.29E-36	3.01E-36	3.4	4.25	4.08	0.72	0.95	1.09
OsR498G1220858100.01	3.626	30.686	3.0797843	0	0	3.12	4.23	3.53	30.73	30.21	31.12
OsR498G1220859100.01	0.063	5.536	6.5073502	3.92E-108	1.86E-108	0.04	0.11	0.04	5.3	5.42	5.89
OsR498G1220859700.01	545.846	80.963	-2.750529	0	0	527.48	568.03	542.03	82.37	81.98	78.54
OsR498G1220861000.01	7.36	26.466	1.8441471	3.85E-54	2.83E-54	7.92	7.31	6.85	26.07	25.42	27.91
OsR498G1220861200.01	0.953	2.62	1.4586095	1.87E-17	2.37E-17	0.99	0.95	0.92	2.42	2.54	2.9
OsR498G1220861400.01	3.586	10.786	1.5885126	1.25E-83	7.09E-84	3.26	3.83	3.67	11.23	10.97	10.16
OsR498G1220862500.01	0.92	2.91	1.6597744	2.32E-11	3.43E-11	0.75	0.74	1.27	2.4	2.75	3.58
OsR498G1220865000.01	0.496	1.01	1.0209525	1.58E-06	2.79E-06	0.54	0.56	0.39	1.31	0.81	0.91
OsR498G1220865400.01	23.143	61.923	1.4308064	0	0	23.93	22.09	23.41	61.38	62.9	61.49
OsR498G1220866400.01	0.533	9.05	4.0791549	1.12E-186	3.56E-187	0.61	0.31	0.68	9.32	9.18	8.65
OsR498G1220867600.01	6.113	14.99	1.2800531	1.57E-89	8.50E-90	6.5	6.36	5.48	14.04	15.29	15.64

OsR498G1220869900.01	0.013	1.106	6.2900124	2.73E-20	3.26E-20	0.04	0	0	1.11	1.37	0.84
OsR498G1220870600.01	0.466	1.666	1.8357037	6.99E-12	1.02E-11	0.42	0.28	0.7	1.55	1.86	1.59
OsR498G1220870800.01	0.356	1.326	1.5931212	7.40E-09	1.19E-08	0.42	0.33	0.32	1.38	1.32	1.28
OsR498G1220872300.01	4.316	58.053	3.7465333	0	0	4.91	3.42	4.62	57.54	51.88	64.74
OsR498G1220873000.01	1.496	4.803	1.7150233	4.88E-24	5.43E-24	1.38	1.7	1.41	4.63	4.12	5.66
OsR498G1220873500.01	0.116	0.34	1.5593725	0.0002484	0.0004931	0.12	0.14	0.09	0.28	0.34	0.4
OsR498G1220874000.01	4.793	10.666	1.3224591	1.66E-30	1.66E-30	4.59	5.22	4.57	10.48	10.47	11.05
OsR498G1220875300.01	0.51	1.736	1.8069532	2.39E-25	2.60E-25	0.45	0.53	0.55	1.79	1.66	1.76
OsR498G1220878500.01	7.75	16.423	1.0643399	1.89E-114	8.66E-115	7.92	7.34	7.99	16.42	15.94	16.91
OsR498G1220879500.01	0.623	2.073	1.6936735	5.71E-13	8.05E-13	0.68	0.56	0.63	1.94	2.4	1.88
OsR498G1220880200.01	0.28	1.926	2.7864803	6.39E-07	1.11E-06	0.35	0.49	0	1.82	1.53	2.43
OsR498G1220880500.01	0.026	0.713	4.6418764	1.52E-24	1.67E-24	0.02	0.04	0.02	0.4	0.97	0.77
OsR498G1220885200.01	1.33	9.306	2.8182079	7.70E-187	2.44E-187	1.08	1.46	1.45	9.57	9.39	8.96
OsR498G1220885500.01	0.03	0.406	3.7113756	1.11E-09	1.74E-09	0	0.06	0.03	0.37	0.37	0.48
OsR498G1220885900.01	0.256	2.176	3.1416884	7.91E-17	1.02E-16	0.32	0.18	0.27	1.87	2.33	2.33
OsR498G1220890200.01	1.863	9.65	2.3664761	1.57E-166	5.47E-167	1.84	1.78	1.97	9.8	10.05	9.1
OsR498G1220890900.01	12.233	24.016	1.072674	9.21E-42	7.82E-42	14.46	11.53	10.71	24.18	25.51	22.36
OsR498G1220891200.01	23.686	52.16	1.2746578	3.38E-237	8.78E-238	23.11	23.2	24.75	60.17	50.74	45.57
OsR498G1220891400.01	0.363	2.53	2.7848119	1.13E-28	1.16E-28	0.18	0.43	0.48	2.81	2.25	2.53
OsR498G1220891600.01	31.6	75.643	1.2356363	0	0	31.08	31.74	31.98	76.05	78.3	72.58
OsR498G1220892600.01	7.273	1.233	-2.679691	1.04E-166	3.63E-167	7.16	7.3	7.36	1.46	1.08	1.16
OsR498G1220894600.01	0.783	2.506	1.6620354	6.75E-25	7.40E-25	0.8	0.97	0.58	2.43	2.62	2.47
OsR498G1220896200.01	0.276	0.013	-4.273518	6.73E-06	1.22E-05	0.31	0.43	0.09	0	0.04	0
OsR498G1220897000.01	1.463	0.506	-1.531481	6.35E-07	1.10E-06	1.19	2.15	1.05	0.48	0.48	0.56
OsR498G1220899400.01	3.173	7.173	1.1760438	3.53E-17	4.49E-17	2.99	3.29	3.24	8.26	6.7	6.56
OsR498G1220901700.01	9.85	35.406	1.8432144	0	0	9.93	10.32	9.3	36.52	36.85	32.85
OsR498G1220902400.01	3.776	9.316	1.3293128	1.40E-63	9.36E-64	3.34	4.06	3.93	9	8.9	10.05
OsR498G1220905200.01	0.053	0.363	2.7817649	0.0008272	0.0017105	0	0.16	0	0.23	0.31	0.55
OsR498G1220905600.01	2.253	15.77	2.8060942	6.37E-94	3.34E-94	1.7	2.89	2.17	16.02	14.7	16.59
OsR498G1220908900.01	0.37	0.05	-2.832945	4.82E-05	9.16E-05	0.32	0.47	0.32	0.1	0	0.05
OsR498G1220909100.01	1.453	6.096	2.0898872	7.36E-89	4.01E-89	1.19	1.5	1.67	6.39	5.76	6.14
OsR498G1220913400.01	0.566	1.893	1.7399447	7.05E-18	8.83E-18	0.47	0.58	0.65	1.92	1.44	2.32
OsR498G1220917200.01	1.17	3.403	1.545762	4.08E-17	5.20E-17	1.23	1.24	1.04	3.5	2.97	3.74
OsR498G1220917400.01	0.19	0.726	1.97441	4.66E-07	8.04E-07	0.08	0.22	0.27	0.77	0.84	0.57
OsR498G1220921600.01	0.876	4.196	2.2545179	3.08E-63	2.07E-63	0.78	1	0.85	4.46	4.11	4.02
OsR498G1220926300.01	3.193	0.59	-2.697207	2.04E-35	1.89E-35	3.8	2.81	2.97	0.69	0.63	0.45
OsR498G1220930000.01	128.506	44.97	-1.516023	0	0	128.61	129.81	127.1	46.61	46.02	42.28
OsR498G1220932600.01	6.393	18.413	1.521006	3.69E-151	1.38E-151	6.04	6.33	6.81	18.41	18.84	17.99
OsR498G1220937300.01	2	6.183	1.630107	7.52E-51	5.73E-51	2.26	1.97	1.77	5.79	6.1	6.66
OsR498G1220938900.01	8.586	66.113	2.9430767	0	0	7.96	9.37	8.43	65.61	64.45	68.28
OsR498G1220940200.01	0.033	0.413	3.6182661	1.37E-06	2.41E-06	0.05	0.05	0	0.35	0.69	0.2
OsR498G1220940400.01	0.07	0.226	2.5187305	1.48E-06	2.61E-06	0.04	0.09	0.08	0.28	0.19	0.21
OsR498G1220940900.01	0.986	3.736	1.9206387	7.29E-33	7.00E-33	1.37	0.73	0.86	3.52	3.96	3.73
OsR498G1220941200.01	0.033	0.183	2.7817649	0.0008272	0.0017105	0	0.04	0.06	0.16	0.19	0.2
OsR498G1220943800.01	0.08	0.3	1.8674948	0.0003847	0.0007738	0.11	0.1	0.03	0.24	0.24	0.42
OsR498G1220945800.01	12.886	49.42	1.9348245	4.71E-137	1.90E-137	13.86	11.98	12.82	47.11	50.22	50.93
OsR498G1220946600.01	0.01	1.113	6.6609105	2.26E-25	2.46E-25	0	0.03	0	1.06	1.09	1.19
OsR498G1220949700.01	15.873	7.15	-1.153728	5.18E-61	3.55E-61	16.28	14.79	16.55	6.27	6.72	8.46
OsR498G1220950900.01	3.306	1.216	-1.399818	1.16E-17	1.46E-17	3.23	4.1	2.59	1.07	1.6	0.98
OsR498G1220952600.01	0	0.49	5.2223375	3.89E-06	7.00E-06	0	0	0	0.48	0.65	0.34
OsR498G1220953200.01	2.3	8.81	1.9147037	1.34E-126	5.73E-127	2.26	2.54	2.1	8.57	8.9	8.96
OsR498G1220953900.01	59.03	24.81	-1.274529	0	0	60.85	57.91	58.33	24.04	25.96	24.43
OsR498G1220954200.01	3.803	1.853	-1.148872	7.04E-37	6.39E-37	3.84	4	3.57	2.23	1.58	1.75
OsR498G1220955300.01	0.05	10.376	7.6538901	1.22E-45	9.82E-46	0.15	0	0	10.87	9.32	10.94
OsR498G1220963600.01	18.2	8.85	-1.042827	1.83E-35	1.69E-35	17.47	18.03	19.1	8.67	7.94	9.94
OsR498G1220963800.01	29.653	6.063	-2.407471	0	0	30.1	30.22	28.64	5.12	6.78	6.29
OsR498G1220967900.01	0.77	2.223	1.526951	4.89E-08	8.11E-08	0.7	1	0.61	1.88	2.25	2.54
OsR498G1220969300.01	0.033	0.843	4.7292975	5.72E-14	7.86E-14	0	0.05	0.05	0.93	0.88	0.72
OsR498G1220972100.01	0.783	0.236	-1.651775	1.57E-07	2.65E-07	0.8	0.91	0.64	0.21	0.42	0.08
OsR498G1220972700.01	0.163	1.186	3.0275213	1.95E-29	1.98E-29	0.15	0.18	0.16	1.48	0.97	1.11
OsR498G1220973100.01	0.156	1.233	2.9463956	2.35E-18	2.92E-18	0.04	0.36	0.07	1.46	1.17	1.07
OsR498G1220981300.01	2.533	7.226	1.5047184	6.98E-56	5.04E-56	2.66	2.33	2.61	7.35	6.7	7.63
OsR498G1220983000.01	3.703	12.613	1.7643747	1.08E-48	8.41E-49	3.89	4.07	3.15	11.62	11.78	14.44
OsR498G1220984300.01	0.18	0.76	2.1003764	2.22E-05	4.14E-05	0.38	0	0.16	0.76	0.94	0.58
OsR498G1220985400.01	0.173	0.886	2.33698	2.02E-15	2.69E-15	0.15	0.1	0.27	0.86	0.88	0.92
OsR498G1220987300.01	0.246	0.696	1.4180166	4.48E-06	8.09E-06	0.33	0.22	0.19	0.83	0.65	0.61
OsR498G1220988600.01	1.283	0.683	-1.050536	7.32E-09	1.18E-08	1.42	1.3	1.13	0.52	0.72	0.81
OsR498G1220990300.01	3.22	6.563	1.0227438	1.70E-44	1.39E-44	3.11	3.21	3.34	6.43	6.73	6.53
OsR498G1220994200.01	0.036	0.933	4.6637091	1.05E-30	1.05E-30	0.03	0.06	0.02	0.92	1.01	0.87

OsR498G1220995600.01	0.62	4.006	2.7540199	4.36E-64	2.90E-64	0.56	0.62	0.68	3.84	4.04	4.14
OsR498G1221001800.01	0.06	0.2	1.7244317	7.64E-05	0.0001469	0.08	0.05	0.05	0.31	0.19	0.1
OsR498G1221002500.01	0.953	4.236	2.151603	1.15E-69	7.23E-70	0.87	0.88	1.11	4.35	4.42	3.94
OsR498G1221002800.01	5.85	12.686	1.1145606	1.65E-49	1.28E-49	6.11	4.99	6.45	12.55	13.28	12.23
OsR498G1221004000.01	0.26	0.6	1.1968024	7.47E-06	1.36E-05	0.24	0.26	0.28	0.66	0.59	0.55
OsR498G1221010700.01	0.423	2.503	2.5531135	7.14E-66	4.67E-66	0.39	0.42	0.46	2.67	2.49	2.35
OsR498G1221014100.01	8.376	57.656	2.7635718	0	0	8.7	8.06	8.37	59.95	55.93	57.09
OsR498G1221014600.01	0.136	1.13	3.0515914	1.59E-17	2.00E-17	0.19	0.07	0.15	1.3	1.04	1.05
OsR498G1221014700.01	0.05	0.686	3.7664981	5.29E-07	9.14E-07	0.15	0	0	0.76	0.62	0.68
OsR498G1221016000.01	1.093	6.496	2.4582257	1.33E-49	1.03E-49	1.27	0.86	1.15	6.6	6.2	6.69
OsR498G1221016100.01	4.59	11.596	1.3409036	2.49E-63	1.67E-63	4.59	4.47	4.71	11.02	11.26	12.51
OsR498G1221019400.01	97.74	301.93	1.6069683	0	0	98.15	98.1	96.97	307.07	298.32	300.39
OsR498G1221020200.01	0.31	0.156	-1.003564	0.0004048	0.0008154	0.33	0.34	0.26	0.24	0.11	0.12
OsR498G1221020700.01	32.14	1.903	-4.078009	0	0	31.93	31.6	32.89	1.83	1.85	2.03
OsR498G1221022200.01	2.523	11.606	2.202321	4.39E-179	1.44E-179	2.91	2.53	2.13	12.18	11.39	11.25
OsR498G1221022400.01	1.586	5.343	1.7513912	2.52E-30	2.53E-30	1.5	1.28	1.98	5.75	4.91	5.37
OsR498G1221022700.01	6.346	16.833	1.4070465	3.53E-114	1.62E-114	6.51	6.02	6.51	16.73	16.06	17.71
OsR498G1221022900.01	3.733	10.416	1.493111	1.40E-70	8.80E-71	3.44	3.68	4.08	10.75	10.16	10.34
OsR498G1221023300.01	1.813	4.143	1.3805927	3.50E-48	2.75E-48	1.91	1.8	1.73	3.79	4.4	4.24
OsR498G1221024900.01	0.5	3.313	2.6606558	2.06E-53	1.52E-53	0.55	0.43	0.52	3.14	3.11	3.69
OsR498G1221025700.01	5.05	15.133	1.5522038	2.70E-60	1.86E-60	4.47	6.13	4.55	13.33	16.62	15.45
OsR498G1221028300.01	0.173	0.026	-2.656649	0.0008663	0.0017948	0.16	0.17	0.19	0	0.08	0
OsR498G1221028600.01	0.476	0.086	-2.44136	0.0002402	0.0004766	0.42	0.6	0.41	0.07	0.19	0
OsR498G1221029800.01	0.08	0.35	2.0898872	0.0001502	0.0002945	0.08	0.08	0.08	0.36	0.32	0.37
OsR498G1221036900.01	0.523	1.876	1.8488791	4.17E-13	5.86E-13	0.67	0.66	0.24	1.89	1.79	1.95
OsR498G1221038000.01	2.51	6.193	1.3019846	2.59E-23	2.92E-23	2.08	2.55	2.9	6.43	5.54	6.61
OsR498G1221038300.01	12.613	28.236	1.1666506	2.36E-169	8.08E-170	12.79	12.47	12.58	28.34	30.33	26.04
OsR498G1221039500.01	7.356	18.093	1.301276	2.53E-75	1.52E-75	7.2	6.9	7.97	16.81	18.7	18.77
OsR498G1221040200.01	3.496	8.136	1.217967	2.51E-43	2.09E-43	3.73	3.6	3.16	7.61	8.55	8.25
OsR498G1221044200.01	0.15	3.306	4.4853719	4.44E-102	2.19E-102	0.18	0.09	0.18	3.11	3.41	3.4
OsR498G1221045200.01	33.23	71.026	1.0997306	1.56E-241	3.99E-242	33.51	31.61	34.57	70.99	70.3	71.79
OsR498G1221049100.01	0.15	0.573	1.8813006	5.96E-09	9.55E-09	0.25	0.09	0.11	0.6	0.46	0.66
OsR498G1221050600.01	6.273	21.21	1.7241929	0	0	6.71	6.35	5.76	21.81	21.99	19.83
OsR498G1221051500.01	85.303	29.133	-1.568301	0	0	84.55	86.75	84.61	30.83	27.61	28.96
OsR498G1221051700.01	4.546	9.213	1.0212308	2.11E-24	2.34E-24	5.19	3.81	4.64	9.93	10.02	7.69
OsR498G1221056000.01	0.036	0.71	4.7292975	7.57E-08	1.26E-07	0.11	0	0	0.84	0.64	0.65
OsR498G1221058200.01	1842.85	528.19	-1.804585	0	0	1756.3	1850	1922.3	512.79	519.23	552.55
OsR498G1221059300.01	2759.02	606.42	-2.189226	0	0	2702.9	2762.9	2811.3	599.39	580.64	639.23
OsR498G1221061300.01	0.6	1.833	1.5187305	4.51E-24	5.01E-24	0.64	0.6	0.56	1.86	1.84	1.8
OsR498G1221061600.01	906.386	3322.4	1.8732564	0	0	884.93	916.41	917.82	3322	3337.3	3308.1
OsR498G1221062400.01	0	0.203	5.5593725	2.32E-07	3.95E-07	0	0	0	0.2	0.25	0.16
OsR498G1221076000.01	1.95	3.823	1.0203038	2.04E-28	2.10E-28	1.92	1.98	1.95	4.04	3.56	3.87
OsR498G1221086600.01	0.03	0.23	2.9130094	2.08E-05	3.87E-05	0.03	0	0.06	0.24	0.21	0.24
OsR498G1221087800.01	6.91	1.116	-2.618764	3.86E-130	1.61E-130	6.69	7.09	6.95	0.89	1.31	1.15
OsR498G1221088200.01	2.496	22.463	3.1649319	4.96E-136	2.01E-136	2.74	2.2	2.55	20.14	19.37	27.88
OsR498G1221088800.01	0.38	1.416	1.8949755	4.90E-12	7.11E-12	0.66	0.28	0.2	1.63	1.31	1.31
OsR498G1221094300.01	7.76	0.033	-7.712091	6.32E-91	3.39E-91	7.67	7.64	7.97	0.05	0	0.05
OsR498G1221094600.01	0.7	8.936	3.7931696	0	0	0.69	0.68	0.73	8.7	9.39	8.72
OsR498G1221095700.01	0.626	1.236	1.0613933	5.08E-17	6.50E-17	0.64	0.55	0.69	1.38	1.3	1.03
OsR498G1221097200.01	2.703	0.5	-2.41088	7.55E-36	6.96E-36	2.87	2.57	2.67	0.36	0.46	0.68
OsR498G1221099600.01	5.263	3.996	-1.215387	1.03E-86	5.72E-87	5.59	4.93	5.27	4.4	4.2	3.39
OsR498G1221105700.01	4.613	0.656	-2.655877	6.42E-45	5.22E-45	4.91	4.39	4.54	0.45	0.48	1.04
OsR498G1221108800.01	0.27	0.753	1.4250714	0.0002539	0.0005045	0.34	0.33	0.14	0.54	0.77	0.95
OsR498G1221109700.01	27.063	71.536	1.413716	0	0	26.15	27.17	27.87	73.75	70.24	70.62
OsR498G1221129300.01	0.386	0.996	1.3658073	1.99E-12	2.85E-12	0.27	0.48	0.41	1.1	0.75	1.14
OsR498G1221137200.01	2.64	5.506	1.0616568	1.43E-35	1.32E-35	2.58	2.47	2.87	5.81	5.55	5.16
OsR498G1221142200.01	58.776	29.2	-1.008784	8.95E-263	2.09E-263	58.5	59.62	58.21	29.2	29.28	29.12
OsR498G1221142800.01	3.376	0.176	-4.09407	4.03E-34	3.80E-34	2.88	3.94	3.31	0.17	0.2	0.16
OsR498G1221146700.01	39.996	197.19	2.3016625	0	0	39.81	39.29	40.89	195.14	200.46	195.97
OsR498G1221146900.01	299.523	48.75	-2.606965	0	0	299.52	301.01	298.04	49.6	49.07	47.58
OsR498G1221157700.01	0.386	1.916	2.308829	3.21E-16	4.18E-16	0.51	0.3	0.35	1.52	1.86	2.37
OsR498G1221161500.01	1.183	0	-6.517443	2.42E-12	3.47E-12	1.35	1.18	1.02	0	0	0
OsR498G1221165600.01	0.323	0.72	1.1379087	2.18E-09	3.44E-09	0.31	0.36	0.3	0.69	0.67	0.8
OsR498G1221167400.01	1.373	3.766	1.4708358	8.75E-18	1.10E-17	1.32	1.62	1.18	3.6	3.78	3.92
OsR498G1221186200.01	0.266	0.986	1.8940678	1.02E-23	1.14E-23	0.29	0.28	0.23	0.97	1.15	0.84
OsR498G1221188800.01	0.78	1.746	1.1844955	1.80E-22	2.06E-22	0.86	0.82	0.66	1.91	1.59	1.74
OsR498G1221192400.01	0.196	0.023	-2.479766	0.0006253	0.0012776	0	0.04	0.55	0.07	0	0
OsR498G1221199100.01	0.823	1.75	1.0739456	1.97E-05	3.67E-05	0.77	0.82	0.88	1.15	1.32	2.78
OsR498G1221199400.01	0.033	0.306	3.1838633	3.18E-08	5.24E-08	0	0.05	0.05	0.4	0.22	0.3

OsR498G1221200700.01	51.443	117.5	1.1913125	0	0	52.35	52.16	49.82	119.24	118.62	114.65
OsR498G1221201000.01	7.506	16.843	1.1659726	1.11E-28	1.14E-28	7.6	7.19	7.73	17.76	16.73	16.04
OsR498G1221222000.01	0.23	0.89	1.9841251	4.15E-17	5.30E-17	0.2	0.33	0.16	0.96	0.85	0.86
OsR498G1221222200.01	22.01	82.433	1.9036333	0	0	21.98	22.81	21.24	81.71	84.88	80.71
OsR498G1221222700.01	0.373	0	-5.113053	1.02E-05	1.88E-05	0.2	0.66	0.26	0	0	0
OsR498G1221223500.01	0	0.51	6.832391	5.79E-15	7.77E-15	0	0	0	0.52	0.39	0.62
OsR498G1221227900.01	0	0.45	6.5593725	7.85E-13	1.11E-12	0	0	0	0.48	0.61	0.26
OsR498G1221234900.01	0.84	0.143	-2.590375	2.61E-22	2.99E-22	0.9	1.02	0.6	0.21	0.02	0.2
OsR498G1221249000.01	10.956	46.546	2.0917756	0	0	10.66	12.37	9.84	44.07	45.04	50.53
OsR498G1221249900.01	0.05	2.816	5.7469995	4.17E-40	3.63E-40	0.1	0.05	0	2.69	2.46	3.3
OsR498G1221257500.01	1.51	0.463	-1.709286	9.85E-24	1.10E-23	1.63	1.59	1.31	0.54	0.39	0.46
OsR498G1221260600.01	0.45	0.123	-1.887648	0.0005166	0.0010489	0.21	0.54	0.6	0.21	0.05	0.11
OsR498G1221260700.01	0.13	0.026	-2.209231	0.0004399	0.0008887	0.13	0.1	0.16	0.03	0.05	0
OsR498G1221261800.01	0.2	3.243	4.00121	2.61E-69	1.65E-69	0.24	0.21	0.15	3.46	2.86	3.41
OsR498G1221264600.01	8.106	20.523	1.3438829	2.90E-239	7.47E-240	8.44	8.01	7.87	21.5	20.87	19.2
OsR498G1221265400.01	0.113	0.65	2.5104629	1.06E-05	1.96E-05	0.14	0	0.2	0.53	0.6	0.82
OsR498G1221277200.01	0.126	0.556	1.9130094	3.07E-06	5.50E-06	0.15	0.12	0.11	0.65	0.61	0.41
OsR498G1221278200.01	0.693	1.973	1.5310437	4.71E-27	4.99E-27	0.67	0.67	0.74	2.15	1.99	1.78
OsR498G1221281600.01	12284	3812	-1.689535	0	0	12241	12202	12409	3697.4	3730.7	4008.1
OsR498G1221297100.01	15.1	104.45	2.8209366	0	0	15.76	15.19	14.35	104.46	105.01	103.87
OsR498G1221303800.01	114.196	537.04	2.2572912	0	0	114.46	115.88	112.25	550.92	542.96	517.25
OsR498G1221305400.01	0.376	1.556	2.1068603	4.32E-08	7.13E-08	0.5	0.47	0.16	2.07	1.36	1.24
OsR498G1221305700.01	1	3.44	1.7307409	5.11E-55	3.72E-55	1.11	1.01	0.88	3.66	3.55	3.11
OsR498G1221307200.01	0.81	2.5	1.6657244	9.96E-18	1.25E-17	0.67	0.9	0.86	2.55	2.36	2.59
OsR498G1221312700.01	1.196	2.71	1.189973	3.96E-29	4.04E-29	1.17	1.39	1.03	2.64	2.86	2.63
OsR498G1221314200.01	1.976	4.356	1.108711	1.24E-05	2.28E-05	2.17	2.46	1.3	3.67	4.67	4.73
OsR498G1221319200.01	0.006	0.063	3.5593725	0.0005724	0.0011661	0	0.02	0	0.06	0.02	0.11
OsR498G1221325200.01	111.066	31.196	-1.954	0	0	110.46	109.75	112.99	31.44	31.19	30.96
OsR498G1221325900.01	4.623	11.12	1.2808084	9.95E-65	6.58E-65	4	5.05	4.82	11.07	11.03	11.26
OsR498G1221328600.01	1.253	3.216	1.3590738	3.19E-10	4.89E-10	1.4	1.04	1.32	2.72	3.79	3.14
OsR498G1221332000.01	23.913	65.653	1.4599212	0	0	23.5	24.43	23.81	65.57	64.44	66.95
OsR498G1221340400.01	2.023	4.04	1.1010434	5.13E-12	7.44E-12	2.4	1.7	1.97	4.4	3.87	3.85
OsR498G1221346400.01	8.486	18.746	1.0766178	1.61E-81	9.31E-82	7.98	8.86	8.62	18.99	18.49	18.76
OsR498G1221348500.01	7616.55	2175.6	-1.807693	0	0	7696.7	7544.9	7608	2139.2	2116.8	2270.9
OsR498G1221349500.01	3333.75	1389.7	-1.26375	0	0	3291.1	3388	3322.2	1385.8	1380.7	1402.5
OsR498G1221350300.01	1464.66	465.62	-1.645957	0	0	1521.8	1432.5	1439.7	488.39	449.85	458.62
OsR498G1221351500.01	154.626	66.06	-1.21892	0	0	152.62	157.67	153.59	66.44	67.85	63.89
OsR498G1221362900.01	3.583	10.113	1.4923474	2.06E-52	1.54E-52	4.44	3.45	2.86	9.87	10.26	10.21
OsR498G1221368500.01	21.956	54.133	1.2997998	8.83E-95	4.60E-95	23.24	20.53	22.1	51.49	52.72	58.19
OsR498G1221369000.01	7.72	57.643	2.949053	0	0	7.47	8	7.69	55.42	56.59	60.92
OsR498G1221374800.01	3.976	10.276	1.368649	3.85E-64	2.56E-64	4.06	3.68	4.19	9.94	9.99	10.9
OsR498G1221375600.01	1.5	0.463	-1.715456	2.21E-07	3.76E-07	1.09	1.57	1.84	0.19	0.29	0.91
OsR498G1221377600.01	0.393	0	-5.02559	1.85E-05	3.43E-05	0.81	0.1	0.27	0	0	0
OsR498G1221394700.01	0.306	1.096	1.8224069	2.29E-08	3.75E-08	0.37	0.23	0.32	0.95	1.13	1.21
OsR498G1221402700.01	4.67	2.326	-1.007888	6.81E-08	1.14E-07	4.26	4.66	5.09	2.45	2.19	2.34
OsR498G1221404900.01	0.73	0.29	-1.330445	0.0003731	0.0007493	0.58	0.83	0.78	0.31	0.3	0.26
OsR498G1221412700.01	1619.65	633.04	-1.355054	0	0	1582.7	1638.2	1638	631.24	627.9	639.97
OsR498G1221413300.01	1855.05	309.46	-2.586083	0	0	1876.4	1804.1	1884.7	301.41	295.71	331.26
OsR498G1221435000.01	153.286	23.243	-2.740562	0	0	167.23	144.56	148.07	23.62	22.18	23.93
OsR498G1221439900.01	1.63	0.313	-2.312113	3.34E-22	3.84E-22	1.56	1.61	1.72	0.36	0.3	0.28
OsR498G1221448500.01	0.023	8.173	8.4582257	1.74E-72	1.07E-72	0	0.07	0	8.16	8.26	8.1
OsR498G1221451200.01	0.606	1.493	1.2982733	1.32E-22	1.51E-22	0.66	0.49	0.67	1.3	1.5	1.68
OsR498G1221453900.01	0.34	10.36	4.940873	7.35E-244	1.87E-244	0.39	0.27	0.36	9.97	9.87	11.24
OsR498G1221464500.01	12.84	2.53	-2.347518	3.18E-19	3.88E-19	13.11	13.51	11.9	2.52	2.23	2.84
OsR498G1221467600.01	0.536	3.67	2.7817649	1.15E-14	1.56E-14	0.83	0.52	0.26	3.65	4.06	3.3
OsR498G1221468700.01	0.836	3.97	2.244017	5.38E-44	4.43E-44	0.76	0.82	0.93	4.03	4.25	3.63
OsR498G1221468900.01	16.193	35.106	1.0983515	1.97E-135	7.99E-136	16.44	15.56	16.58	35.27	34.96	35.09
OsR498G1221469100.01	95.34	9.15	-3.415608	0	0	89.47	95.89	100.66	7.76	9.62	10.07
OsR498G1221471600.01	1.58	0.096	-4.051125	2.97E-50	2.27E-50	1.55	1.57	1.62	0.06	0.08	0.15
OsR498G1221472800.01	13.943	34.373	1.2991641	3.76E-87	2.08E-87	13.74	14.44	13.65	32.36	33.16	37.6
OsR498G1221488200.01	34.38	6.36	-2.436495	0	0	35.47	31.98	35.69	5.89	6.25	6.94
OsR498G1221500100.01	13.176	38.406	1.5565738	1.12E-302	2.35E-303	13.52	13.03	12.98	37.79	36.92	40.51
OsR498G1221501200.01	5.156	13.876	1.4447129	2.71E-117	1.22E-117	5.32	4.72	5.43	14.59	13.08	13.96
OsR498G1221505500.01	0.353	0.713	1.0217157	0.0004961	0.0010064	0.46	0.35	0.25	0.62	0.92	0.6
OsR498G1221506900.01	0.333	0.07	-2.394824	7.99E-06	1.46E-05	0.27	0.52	0.21	0.11	0.1	0
OsR498G1221518200.01	52.53	23.996	-1.14559	0	0	52.14	52.97	52.48	24.1	23.2	24.69
OsR498G1221540900.01	0.173	0.46	1.3473621	5.96E-07	1.03E-06	0.2	0.17	0.15	0.49	0.41	0.48
OsR498G1221541200.01	4.123	8.34	1.0284788	4.67E-33	4.48E-33	4.06	4.3	4.01	7.98	8.48	8.56
OsR498G1221562700.01	8.666	3.676	-1.315514	1.17E-90	6.29E-91	9.08	8.56	8.36	3.46	4.41	3.16

OsR498G1221574600.01	0.033	0.473	3.7817649	2.33E-07	3.97E-07	0	0.1	0	0.35	0.6	0.47
OsR498G1221579700.01	0.28	0.04	-2.832945	0.0006839	0.0014027	0.42	0.3	0.12	0	0	0.12
OsR498G1221589400.01	0.78	1.776	1.1561209	1.65E-17	2.09E-17	1.08	0.72	0.54	1.62	1.91	1.8
OsR498G1221589500.01	16.166	54.763	1.7517058	0	0	16.64	14.95	16.91	52.92	49.71	61.66
OsR498G1221591800.01	0.333	2.2	2.7198371	7.46E-19	9.15E-19	0.3	0.35	0.35	1.84	2.75	2.01
OsR498G1221602100.01	20.146	6.726	-1.477502	1.05E-123	4.54E-124	19.71	20.43	20.3	6.51	7.11	6.56
OsR498G1221602400.01	10.346	2.223	-2.21811	8.80E-199	2.65E-199	10.39	10.38	10.27	2.21	2.28	2.18
OsR498G1221605600.01	0.83	4.35	2.3901864	7.42E-86	4.14E-86	1.01	0.75	0.73	4.49	4.26	4.3
OsR498G1221620100.01	10.606	0.71	-3.902962	2.04E-242	5.18E-243	10.42	11.34	10.06	0.67	0.64	0.82
OsR498G1221623000.01	12.53	29.623	1.2198593	7.74E-90	4.19E-90	12.76	11.76	13.07	28.44	31.19	29.24
OsR498G1221633900.01	0.026	0.43	4.2223375	6.41E-10	9.93E-10	0.04	0.04	0	0.31	0.57	0.41
OsR498G1221644700.01	0.54	1.06	1.1296882	4.02E-06	7.23E-06	0.47	0.38	0.77	1.1	1.09	0.99
OsR498G1221645400.01	6.056	14.3	1.2055292	5.67E-32	5.53E-32	6.34	6.51	5.32	13.71	14.18	15.01
OsR498G1221647100.01	0.386	1.28	1.5187305	0.0003744	0.0007521	0.6	0.19	0.37	0.87	1.53	1.44
OsR498G1221649500.01	0.36	1.283	1.8204973	1.27E-15	1.68E-15	0.31	0.29	0.48	1.17	1.07	1.61
OsR498G1221651400.01	3.416	1.136	-1.705384	1.60E-44	1.31E-44	3.18	3.79	3.28	1.23	1.32	0.86
OsR498G1221661000.01	3.726	9.986	1.4979719	8.04E-37	7.30E-37	3.9	4	3.28	9.86	10.06	10.04
OsR498G1221661300.01	0.13	0.456	1.8488791	8.72E-05	0.0001686	0.17	0.13	0.09	0.83	0.33	0.21
OsR498G1221663000.01	3.156	10.65	1.7531442	2.09E-49	1.62E-49	2.97	3.3	3.2	11.09	10.97	9.89
OsR498G1221666900.01	0.16	0.84	2.3894475	7.16E-06	1.30E-05	0.16	0.16	0.16	0.55	1.01	0.96
OsR498G1221670300.01	7.01	23.073	1.7297342	4.24E-107	2.03E-107	7.6	6.78	6.65	24.4	22.83	21.99
OsR498G1221671000.01	0.163	0.536	1.9255004	1.75E-07	2.97E-07	0.14	0.15	0.2	0.55	0.41	0.65
OsR498G1221671600.01	1.923	5.946	1.633546	2.83E-36	2.58E-36	2.03	1.88	1.86	5.67	5.71	6.46
OsR498G1221672600.01	72.736	246.71	1.759638	0	0	72.03	74.07	72.11	238.76	256.47	244.9
OsR498G1221673100.01	2.98	10.916	1.8800002	1.74E-107	8.29E-108	3.22	2.76	2.96	11.13	11.55	10.07
OsR498G1221685100.01	15.37	41.683	1.4214886	1.61E-215	4.49E-216	14.95	16.59	14.57	38.9	43.92	42.23
OsR498G1221685300.01	0.013	0.593	5.5295619	9.17E-14	1.27E-13	0	0.04	0	0.37	0.59	0.82
OsR498G1221685900.01	1.606	4.106	1.3551175	1.79E-17	2.26E-17	1.75	1.28	1.79	3.93	3.81	4.58
OsR498G1221686500.01	3.77	24.693	2.7353639	0	0	4.15	3.57	3.59	24.8	25.6	23.68
OsR498G1221688500.01	6.863	21.526	1.6456462	8.15E-182	2.65E-182	6.36	7.57	6.66	22.81	21.17	20.6
OsR498G1221689600.01	2.823	9.563	1.7466846	8.59E-57	6.15E-57	2.93	2.4	3.14	9.35	7.85	11.49
OsR498G1221691900.01	10.633	38.223	1.8435625	4.30E-119	1.91E-119	12.13	9.56	10.21	35.92	36.04	42.71
OsR498G1221697600.01	0.713	1.756	1.29238	7.14E-12	1.04E-11	0.82	0.71	0.61	2.03	1.82	1.42
OsR498G1221698200.01	0.11	2.243	4.3529216	5.76E-47	4.58E-47	0.13	0.07	0.13	2.35	2.24	2.14
OsR498G1221701800.01	0.29	4.253	3.8619352	8.78E-47	6.99E-47	0.23	0.17	0.47	4.27	3.29	5.2
OsR498G1221703100.01	5.793	12.093	1.050587	1.56E-46	1.25E-46	5.44	5.83	6.11	12.33	12.04	11.91
OsR498G1221704200.01	0.156	0.566	1.8674948	3.63E-09	5.78E-09	0.19	0.14	0.14	0.57	0.73	0.4
OsR498G1221705000.01	31.043	62.876	1.0284233	5.20E-256	1.25E-256	32.29	30.98	29.86	63.55	59.38	65.7
OsR498G1221706400.01	0.08	0.763	3.2411965	1.87E-15	2.48E-15	0.09	0.06	0.09	1.09	0.65	0.55
OsR498G1221715900.01	2.613	0.856	-1.595326	6.13E-31	6.08E-31	2.86	2.34	2.64	0.93	0.75	0.89
OsR498G1221716600.01	23.666	55.923	1.2256241	6.61E-292	1.43E-292	23.95	24.04	23.01	57.38	55.94	54.45
OsR498G1221717700.01	100.7	1039.3	3.3655291	0	0	96.11	97.62	108.37	1034.2	992.06	1091.7
OsR498G1221720100.01	0.026	0.286	3.4338416	7.99E-06	1.46E-05	0	0	0.08	0.43	0.31	0.12
OsR498G1221720900.01	6.726	16.843	1.3366253	1.16E-35	1.07E-35	6.59	6.59	7	18.08	14.95	17.5
OsR498G1221722100.01	6.276	0.72	-3.129927	7.33E-32	7.16E-32	5.83	6.12	6.88	0.43	0.53	1.2
OsR498G1221723300.01	0.06	1.186	4.3083107	5.63E-28	5.87E-28	0.06	0	0.12	1.02	1.43	1.11
OsR498G1221724500.01	0.276	0.08	-1.851561	1.95E-05	3.62E-05	0.19	0.38	0.26	0.1	0.09	0.05
OsR498G1221725100.01	0.5	1.996	2.0041573	3.37E-22	3.87E-22	0.47	0.56	0.47	2.21	1.69	2.09
OsR498G1221725400.01	0.956	3.556	1.8909939	3.81E-34	3.59E-34	0.81	1.19	0.87	3.09	3.76	3.82
OsR498G1221726800.01	17.946	45.646	1.3445823	1.68E-54	1.23E-54	18.83	18.79	16.22	48.73	41.73	46.48
OsR498G1221728800.01	30.913	9.703	-1.672071	1.53E-177	5.06E-178	33.85	29.04	29.85	9.75	9.16	10.2
OsR498G1221729000.01	2.99	1.343	-1.157502	1.26E-06	2.22E-06	2.71	3.74	2.52	1.45	1.36	1.22
OsR498G1221757200.01	0.57	1.9	1.7244317	3.26E-11	4.85E-11	0.42	0.68	0.61	1.85	1.64	2.21
OsR498G1221758000.01	15.54	32.47	1.0569694	1.63E-141	6.39E-142	15.01	15.6	16.01	32.53	33.27	31.61
OsR498G1221758800.01	2.84	5.816	1.0317431	1.03E-10	1.56E-10	2.62	3.06	2.84	6.15	5.15	6.15
OsR498G1221760100.01	18.64	41.106	1.1433631	5.94E-120	2.63E-120	19.07	19.09	17.76	41.97	41.19	40.16
OsR498G1221763100.01	10.026	21.966	1.1591834	2.87E-89	1.56E-89	10.06	9.95	10.07	22.46	20.6	22.84
OsR498G1221764600.01	0	0.533	4.6748497	0.0001298	0.0002536	0	0	0	0.84	0	0.76
OsR498G1221771400.01	2.576	7.67	1.5717562	6.86E-28	7.16E-28	2.68	3.32	1.73	7.41	7.06	8.54
OsR498G1221774300.01	3.503	1.006	-1.79818	7.98E-17	1.03E-16	3.54	3.45	3.52	1.06	0.87	1.09
OsR498G1221788200.01	8.19	22.69	1.4670866	1.09E-198	3.28E-199	7.51	8.44	8.62	23.12	23.14	21.81
OsR498G1221790500.01	0.133	1.186	3.144335	4.16E-11	6.21E-11	0	0.13	0.27	1.11	1.11	1.34
OsR498G1221809000.01	6.613	32.066	2.278489	0	0	5.94	6.7	7.2	32.24	33.39	30.57
OsR498G1221837800.01	9.35	83.973	3.1654453	0	0	9.34	9.3	9.41	84.47	86.25	81.2
OsR498G1221839300.01	3.623	10.573	1.5282978	1.31E-47	1.03E-47	3.45	4.37	3.05	10.11	11.25	10.36
OsR498G1221852200.01	0.053	0.686	3.802229	2.63E-30	2.64E-30	0.09	0.04	0.03	0.8	0.64	0.62
OsR498G1221854600.01	0.743	0.253	-1.559725	5.60E-12	8.12E-12	0.88	0.7	0.65	0.23	0.27	0.26
OsR498G1221863500.01	88.36	21.63	-2.031867	0	0	89.77	87.58	87.73	20.71	21.44	22.74
OsR498G1221864900.01	0.28	2.87	3.3494494	3.35E-62	2.27E-62	0.31	0.31	0.22	3.33	2.74	2.54

OsR498G1221865900.01	1.26	6.246	2.2289251	5.74E-54	4.22E-54	1.53	1.1	1.15	5.17	8.31	5.26
OsR498G1221866100.01	1.47	18.53	3.6461051	0	0	1.39	1.38	1.64	18.47	18.84	18.28
OsR498G1221867600.01	25.46	51.376	1.0173401	8.50E-97	4.37E-97	24.24	25.69	26.45	52.43	46.2	55.5
OsR498G1221871700.01	0.48	1.246	1.4598368	3.03E-09	4.80E-09	0.3	0.51	0.63	1.28	1.5	0.96
OsR498G1221874400.01	18.343	5.733	-1.671633	2.08E-210	5.95E-211	17.73	18.45	18.85	5.71	5.47	6.02
OsR498G1221877200.01	22.603	45.653	1.0060762	3.22E-85	1.81E-85	22.33	22.61	22.87	45.29	46.46	45.21
[truncated: 44,833,849 more chars]
